# Professional, scholar, or knowledge worker? Identity construction of Chinese management researchers amid the research–practice gap

**DOI:** 10.1371/journal.pone.0306833

**Published:** 2024-08-29

**Authors:** Shubo Liu, Mengna Lv, Qiuli Huang, Yiduo Wang

**Affiliations:** 1 Busines School, Central University of Finance and Economics, Beijing, China; 2 Warwick Manufacturing Group, University of Warwick, Coventry, United Kingdom; School of Health Binawan: Universitas Binawan, INDONESIA

## Abstract

We move beyond discussing the desirability and feasibility of bridging the research–practice gap to introducing an identity perspective to explore how Chinese management researchers make sense of the research–practice gap and what kinds of career identities are constructed. We conducted a qualitative study among 34 Chinese management researchers working at or studying for a PhD at research-oriented business schools in China. The findings show that management researchers in typical Chinese higher education institutions prefer constructing a single identity (i.e., professional, scholar, or knowledge worker identity) rather than a hybrid identity such as "academic-practitioner" as studies of their Western counterparts suggest. Moreover, before seeking and emulating role models to construct their desired career identities, researchers in China studying management reflexively search for referent groups by identifying either with a narrow disciplinary group (US mainstream management researchers or traditional intellectuals) or a broad group of knowledge workers. Furthermore, this study delineates how researchers with varying career identity narratives adopt corresponding identity work strategies (i.e., redefinition, defense, and distance) suggesting that identity work strategies do not always lead to achieving or preserving positive identity.

## Introduction

The gap between academic research and practical application in business management is a long-standing issue that has significant implications for both academic and professional communities [1–3]. This phenomenon is particularly evident in China, where the academic evaluation system heavily emphasizes publication in high-impact journals and researchers often prioritize theoretical contributions over practical relevance [4]. For instance, a report from the National Natural Science Foundation of China revealed, "Many management research projects fail to align with China’s management practices largely due to the academic community’s insufficient interaction with the business sector … Researchers often lack focus and a deep understanding of the current state of China’s management practices, failing to produce influential perspectives…. The disconnection between research and practice has become a unique chronic problem in China" [5]. Additionally, news from Guangming Daily (2021), one of China’s official media outlets, indicated a growing disconnect between the knowledge taught in business school and the needs of management practices [6].

Notably, considerable academic controversy exists regarding the feasibility and desirability of bridging this gap, posing identity challenges for management researchers [1–3]. For example, some management researchers marginalize their peers who maintain close ties with the practical world [7], and others criticize those who prioritize rigor over relevance [8], resulting in what Gulati (2007) termed "brutal identity warfare" [9]. Considering the situations in which research is detached from practice, generating intense debates in academia, how do management researchers make sense of and construct their career identities?

The limited studies that have suggested that management researchers aspire to construct a hybrid identity to maintain a balance between the two selves as a scholar and practitioner [2, 3, 10] have primarily involved "academic-practitioners" acting as boundary spanners navigating both the academic and practical realms. However, there are also considerable management researchers based in business schools or universities where institutional norms focus on academic-specific tasks such as the pursuit of high-quality research, publication in top-tier academic journals, and participation in academia-oriented events. Their primary work responsibility is producing "scientific management knowledge" rather than "practical application," commonly observed in China [11]. Unfortunately, these "pure" researchers without practical application roles have received little consideration; thus, how they construct career identity amid the research–practice gap remains poorly understood. Therefore, this study’s central research question is as follows:

1. How does the research–practice gap affect Chinese management researchers’ career identity construction?
  We divided this broad question into three distinct questions:
2. What are the identity narratives of Chinese management researchers?
3. Why do Chinese management researchers construct these career identities?
4. How do Chinese management researchers implement identity work strategies to maintain these career identities?

Based on 34 in-depth interviews with Chinese management researchers, we identify three identity narratives categories: professionals, scholars, and knowledge workers. Different categories of Chinese management researchers identify with distinct referent groups (i.e., US mainstream management researchers, traditional intellectuals, and knowledge workers) and frame their identities in terms of referent group membership. Furthermore, we delineate three identity work strategies that correspond to the three identified identity narratives: redefinition, defense, and distance.

This study makes a threefold contribution to the literature. First, it reveals how Chinese management researchers who are not "academic-practitioners" construct their single rather than hybrid career identities and the identity work strategies that they adopt to maintain their desired identities. These findings contribute to the emerging literature on the career identity construction of management researchers amid the research–practice gap. Second, this study enriches the literature on the identity construction of professionals by including a new situation in which an ideal career identity has not yet been commonly identified and shared by members of professional communities. In doing so, we illustrate how Chinese management researchers self-categorize and identify with referent groups before they seek and emulate role models to construct their desired career identities. Third, unlike other studies illustrating that individuals employ identity work strategies for constructing positive identities, this study highlights how the identity narrative of knowledge workers challenges this prevailing perspective.

The remainder of this study proceeds as follows. We first summarize the relevant literature on identity and the identity work of professionals and subsequently review studies on the research–practice gap in the management field, focusing on the management field in China. Following this literature review, we introduce this study’s qualitative semi-structured interview method. Further, in the results section, we discuss three types of identity construction adopted by Chinese management researchers in navigating the research–practice gap; specifically elucidate the meanings of each identity narrative, underlying reasons for its construction, and corresponding identity work strategies. The final section presents the theoretical implications derived from the research findings.

## Theoretical background

### Identity and identity work of professionals

Emphasizing the social dimensions of identity, Tajfel and Turner (1979) contend that an individual’s identity or self-perception is rooted in their group memberships and derived from the social categories to which they perceive themselves as belonging [12]. Moreover, the degree of identification with a referent group is a pivotal factor influencing an individual’s willingness to incorporate a given social category into their self-definition [13].

The processes and practices employed to construct and manage identities can be understood as identity work [14, 15]. Sveningsson and Alvesson (2003, p. 1165) define identity work as "people being engaged in forming, repairing, maintaining, strengthening, or revising the constructions that are productive of a sense of coherence and distinctiveness" [16]. The concept of identity work emphasizes individual agency and recognizes the substantial influence exerted by social groups [14, 15, 17]. Furthermore, according to social identity theory, social identities are contingent upon social interactions; that is, individuals observe and categorize people into categories, aligning themselves with those that uphold and augment their self-esteem [18]. The motivation to identify with a specific category is partially rooted in the human drive to enhance individual social identity [18].

Professionals working in various fields have been the research object of identity studies. Moreover, studies have examined the process of identity work among professionals and the effects of socialization and role models. For example, Pratt et al. (2006) and Obling (2023) examine the dynamics of identity enactment and reconstruction in young medical residents and military career officers at later career stages, respectively [15, 19]. Both studies emphasize the importance of professional socialization. Consistent with the socialization literature, Shapiro et al. (1978) demonstrate that role modeling contributes to identity construction and influences professional identification processes [20]. Role models are part of a group’s collective identity, which is members’ shared sense of identity as a group [21]. Further research examines the function of role models in crafting "provisional selves" [14], development of "managerial identities" [13], and enactment of "collective identity" [21].

The aforementioned studies reveal that when experiencing identity challenges due to career transitions, professionals observe and emulate role models to construct their career identities [14, 15, 19], commonly identified by members of professional communities such as accountants, physicians, consultants, and investment bankers. However, how professionals construct their career identities within a professional community without consensus on a shared ideal identity has been scarcely explored. For example, we will demonstrate in the context of the research–practice gap, researchers within the management discipline have not reached a consensus on the meaning of the research, leading to an identity crisis regarding "who are we, really, and what are we here to do?" [9]. Unfortunately, limited studies have investigated how researchers construct their identities in academic communities where a commonly identified or recognized career identity is lacking or not clearly defined. Therefore, several management scholars have called for further research to explore the identity construction of management researchers amid the research–practice gap [1–3].

### Research on the research–practice gap in the management field

Various management researchers attempt to impact practice but often do not succeed, widely known as the "research–practice gap" [3, 22]. Studies tend to discuss the reasons and outcomes of the gap; the feasibility and desirability of bridging the gap; and when advisable, how to bridge this gap [22–25]. Specifically, Susman and Evered (1978) attribute this gap to increasingly sophisticated research methods and techniques, suggesting "As our research methods and techniques have become more sophisticated, they have also become increasingly less useful for solving the practical problems that members of organizations face" (p. 582) [25]. Other commentators opine that researchers are motivated to generate knowledge and publish research primarily evaluated by peers rather than appraised by practitioners and applied within organizational contexts [26]. Thus, the outcome is that practitioners often criticize the knowledge generated by researchers in the management field due to its limited or negligible practical applicability [8, 22, 24]. Therefore, some scholars recommend bridging the research–practice gap to generate practical and theoretical (or relevant and rigorous) insights [22]. However, other scholars argue that bridging this gap may be neither feasible nor desirable [23]. These debates give rise to "brutal identity warfare" [6]. Specifically, some self-appointed "serious academics" show disdain and marginalize their peers who speak with practitioners [7], and others who believe strongly in the value of relevance denigrate researchers who focus on rigor more than relevance [8].

Despite numerous studies on the research–practice gap, how management researchers make sense of and navigate the gap at the individual level is poorly understood. This knowledge gap is surprising because how to perceive and whether to bridge the research–practice gap occurs at the individual level [3]. Although Gulati (2007) argues that management researchers bifurcate into two tribes (i.e., the rigorous research and applicable findings tribe), and each tribal member forms their identity as either "serious scholars" or "management types" (p. 777) [9], this identity distinction is theoretical and has no robust empirical support. Notably, with the rigor-relevance debate simmering, management researchers have increasingly recognized the challenges associated with establishing "either-or" identities and have endeavored to address them [2, 3]. Therefore, the identities constructed by management researchers may not be as clear as Gulati (2007) suggests [9].

Regarding empirical research, a few studies suggest that attempting to bridge the research–practice gap would lead to researchers experiencing intense identity conflict due to their perceived incommensurability of dual work identities of scholar and practitioner [1–3]. However, management researchers who do not pursue practical relevance out of concern that it might compromise high-quality basic research exist [8]. Notably, a substantial number of management researchers are affiliated with business schools or universities, where institutional norms emphasize academic-focused duties such as conducting high-quality research, publishing in leading journals, and participating in academic events. This phenomenon is increasingly prominent in China (which we will delineate). However, how these management researchers who worked in environments where their primary obligation centers on generating management knowledge rather than practical application perceives the research–practice gap and shape their career identities remains underexplored.

### Research–practice gap in Chinese management studies

Management academia in China has introduced US academic standards, and thus the disconnection between research and practice. Numerous Chinese commentators believe that the research–practice gap is more prevalent and severe in China than in the Western context. For example, Zhang (2008) asserts, "Randomly selecting a certain number of papers from academic journal websites, summarizing their main conclusions or viewpoints, and then organizing discussions among researchers and business practitioners to assess the authenticity of these conclusions—perhaps the proportion of conclusions that withstand scrutiny may not exceed 50%!" (p. 338) [27]. Furthermore, Han and Xi (2010) state, "Our perspective differs slightly, suggesting that it may not be, perhaps, not more than 50% withstand scrutiny, but rather around 90% of models and assumptions are solemnly examined and proven to be common sense" (p. 11) [28].

According to some commentators, the research–practice gap in Chinese management studies is partially due to the "scientification of management." In the 1980s, China began importing Western management theories, especially from the US, coinciding with the peak of the US "scientification of management research." Influenced by this trend, researchers in China became fixated on constructing several theoretical models or conceptual knowledge, excessively emphasizing scientific rigor and normativity [29]. Thus, they overlooked the intimate connection among morality, ideals, emotions, and practice, leading to a decreasing emphasis on its relevance to practice [30].

Furthermore, the long-term adherence to a single empirical paradigm in academic training has led researchers to detach themselves from reality. Developing an awareness of issues arising from managerial practices becomes challenging due to this detachment. Researchers may become accustomed to overlooking the complexity of reality, equating management research with the manipulation of abstract conceptual relationships, resulting in a disconnect between research content and real-world practices [4, 29].

Other commentators attribute the research–practice gap in Chinese management studies to the market-driven academic evaluation system within business schools. With the advent of the globalization of higher education, Chinese business schools are actively seeking to participate in global competition and rankings. Since the quantity of papers published in top international journals significantly influences rankings, numerous business schools consider this a crucial component in assessing faculty performance. The desire to improve rankings has prompted government agencies and academic institutions to introduce policies encouraging or mandating faculty to increase their quantity of research and the rate at which they produce [31]. Therefore, under the pressure of performance assessment, an increasing number of Chinese management researchers have limited time to engage closely with managerial practices. Thus, academic research has become more about publishing papers than solving real-world problems, resulting in a significant gap between research and practice [32].

To address the issue of the research–practice gap in Chinese management studies, several Chinese management researchers are calling for changing the management research paradigm to better align with management practitioners’ perspective [4, 29], advocating for management researchers and practitioners to construct a common language [33] or suggesting managers as researchers [34]. However, numerous Chinese management researchers appear indifferent to these calls [28]. At present, Chinese management researchers primarily comprise faculty who have retreated to the "ivory tower" of universities after completing their doctoral education. The majority of these researchers have little practical experience, tending to select research topics solely from the literature rather than practical engagement; hence, these topics do not offer insights into the subjects of their research [4, 11, 29]. Consequently, their output may be mere intellectual exercises without real-world benefits [4, 11, 29]. Notwithstanding the escalating scrutiny and criticism within Chinese management academia concerning the research–practice gap, how Chinese management researchers understand and construct their career identities remains a crucial question worth further investigation.

## Materials and methods

### Research design and data collection

Consistent with other studies examining the identity construction of business school researchers, we used a qualitative semi-structured interview method [1, 35, 36]. Considering that our study explores how Chinese management researchers construct their career identities in the research–practice gap context, we conducted interviews with Chinese management researchers working or studying for a PhD at research-oriented business schools, which are typically in elite Chinese universities. To ensure a representative sample, we purposively selected interviewees at various career stages (i.e., complete, assistant, and associate professors and PhD candidates), who represented a range of management research fields (i.e., strategy, marketing, organizational behavior and human resource management (OBHRM), operations, as well as finance and accounting). We approached the interviewees through established social networks and snowball methods [37]. The first three authors of this paper, comprising one male associate professor and two female doctoral students, all had experience in interview methods, and conducted the interviews with each participant. The interviewers usually began the interview by asking the respondents to describe their academic background and impressions of management studies before discussing the factors that inspire and guide their research. These questions often led to conversations regarding their experiences in research, including selecting research questions, conducting empirical studies, and publishing practices (S1 File). To adhere to theoretical sampling principles, we terminated the interview when no new concepts, categories, or relationships emerged from further interview analyses, that is, when data reached saturation [38].

Between September 13, 2021 and July 22, 2022, we conducted interviews with 34 Chinese management researchers from 12 business schools in China. The team members had varying degrees of familiarity with the interviewees, ranging from professional colleagues to new acquaintances recruited via snowball sampling. Over half of the interviews were face-to-face, while the remaining interviews were via WeChat video, a necessary adaptation due to COVID-19 restrictions. The face-to-face interviews were conducted at various locations based on the participants’ preferences, including their offices or homes, providing a comfortable and convenient environment for open dialogue. The WeChat video interviews were conducted online, allowing for remote participation and accommodating a flexible approach to data collection that included participants who might not be able to attend in-person meetings. This hybrid approach to the setting of data collection ensured accessibility and participant comfort, facilitating a rich and diverse dataset.

Prior to the interview, each participant was provided with a participant information leaflet (S2 File) detailing how their data would be used, stored, and protected. Following this, participants signed a consent form (S3 File) indicating their informed consent regarding data collection and usage. These measures ensure that participants are fully aware of the confidentiality protocols in place and agree to the terms before participating in the study.

Each interview, lasting between 45 to 150 minutes, was audio-recorded with a professional voice recorder capable of automatic verbatim transcription. Since the interviews were conducted in Chinese, the transcripts are also in Chinese characters. Within 72 hours of each interview, the second and third authors independently reviewed and cross-checked the transcriptions to ensure accuracy and maintain the integrity of the original meaning. These transcriptions were then sent back to the interviewees for verification. The process resulted in over 650,000 Chinese characters of transcribed text (S4 File). To protect the confidentiality of our participants, all data were de-identified before analysis, with identifiable information replaced by pseudonyms or numerical identifiers. Each interviewee was denoted with distinct codes. Table 1 summarizes the profiles of the interviewees.

**Table 1:** Interviewees’ profiles.

Participant Code	Research Field	Gender	Age	Career Stage	Interview Type	Interview Duration (min)	Interview Text (Chinese characters)
J01	Strategy	Male	>45	Complete professor	Face-to-face interview	110	21623
J02	Marketing	Male	30∼45	Complete professor	WeChat video interview	60	16182
J03	OBHRM	Male	30∼45	Complete professor	Face-to-face interview	89	24542
J04	OBHRM	Male	>45	Complete professor	WeChat video interview	105	22056
J05	Operations	Male	30∼45	Complete professor	Face-to-face interview	115	28386
J06	Accounting	Male	30∼45	Complete professor	Face-to-face interview	53	18084
F01	Strategy	Male	30∼45	Assistant professor	Face-to-face interview	149	35120
F02	Marketing	Female	30∼45	Assistant professor	Face-to-face interview	81	22187
F03	OBHRM	Female	30∼45	Assistant professor	Face-to-face interview	88	19432
F04	OBHRM	Male	30∼45	Assistant professor	Face-to-face interview	61	14274
F05	OBHRM	Female	30∼45	Assistant professor	WeChat video interview	109	19761
F06	OBHRM	Male	<30	Assistant professor	WeChat video interview	127	26607
F07	operations	Male	>45	Assistant professor	Face-to-face interview	112	26528
F08	Finance and Accounting	Male	30∼45	Assistant professor	Face-to-face interview	79	22498
L01	Strategy	Female	30∼45	Associate professor	WeChat video interview	45	10255
L02	Strategy	Female	30∼45	Associate professor	WeChat video interview	86	20748
L03	Strategy	Female	30∼45	Associate professor	WeChat video interview	90	19913
L04	Strategy	Male	30∼45	Associate professor	Face-to-face interview	106	22454
L05	Marketing	Male	30∼45	Associate professor	WeChat video interview	80	19168
L06	OBHRM	Female	30∼45	Associate professor	WeChat video interview	55	2872
L07	OBHRM	Female	<30	Associate professor	WeChat video interview	84	17675
L08	Operations	Female	<30	Associate professor	WeChat video interview	84	20382
L09	Operations	Male	30∼45	Associate professor	WeChat video interview	89	16304
L10	Finance and Accounting	Female	30∼45	Associate professor	WeChat video interview	64	14146
L11	Finance and accounting	Female	<30	Associate professor	Face-to-face interview	72	17066
B01	Strategy	Female	<30	PhD candidate	Face-to-face interview	99	28446
B02	Marketing	Female	<30	PhD candidate	Face-to-face interview	120	18037
B03	Marketing	Female	<30	PhD candidate	Face-to-face interview	55	10694
B04	Marketing	Female	<30	PhD candidate	Face-to-face interview	67	11767
B05	OBHRM	Female	<30	PhD candidate	Face-to-face interview	64	12807
B06	OBHRM	Male	<30	PhD candidate	Face-to-face interview	96	18571
B07	Operations	Female	<30	PhD candidate	Face-to-face interview	82	24966
B08	Operations	Female	<30	PhD candidate	Face-to-face interview	62	12079
B09	Finance and accounting	Female	<30	PhD candidate	WeChat video interview	65	15189

### Ethics statement and data management

This study received ethical approval (S5 File) from the Research Ethics Committee of the Central University of Finance and Economics (approval number: AC-SB-CUFE-2021-0013). Prior to participation, each participant was provided with a participant information leaflet (S2 File) to be informed of the study’s purpose, procedures, assessments, potential risks, benefits, and outlined our intentions to publish findings in peer-reviewed journals or conference proceedings in the future before recruitment. Then, he/she gave written informed consent (S3 File) in line with the Declaration of Helsinki and its subsequent amendments or comparable ethical standards. Participants were also clearly informed that their involvement was entirely voluntary and that they could withdraw without providing a reason.

Moreover, all collected participant data was de-identified and pseudonymized by assigning each participant a code (e.g., J01) prior to analysis and securely encrypted for storage. The data were stored on isolated data platforms and backed up on multiple non-networked hard drives. Regular security updates and anti-virus checks were conducted to ensure data integrity and security. To oversee and manage the data, we utilized NVivo software. This software facilitated the organization, coding, and analysis of qualitative data, providing a secure environment for handling sensitive information. The software’s built-in encryption and access control features ensured that only authorized research team members could access the data. Additionally, the software allowed for comprehensive audit trails, enabling us to track any changes or access to the data, further enhancing the security and integrity of our data management process.

### Data analysis

We conducted an inductive data analysis, employing the grounded theory approach [39]. This involved iterative cycles of data examination and theory development facilitated by Nvivo12 software. This software facilitated the creation of a coding tree, allowing us to visualize the hierarchical structure of our coding scheme and track the relationships between different codes.

Our analysis comprised three major steps. First, during open coding, the first three authors independently reviewed the raw text of the interview transcripts line by line. They systematically identified and labeled discrete units of meaning, referred to as initial codes. These initial codes were descriptive in nature and stayed as close as possible to the words used by the interviewees. For instance, a quote like "Prof. X from Wharton School is my role model. His work really speaks to entrepreneurs and genuinely helps them. He has shown me that it’s possible to create research that’s top-notch and valued by the real-world folks. (F01)" was categorized under the code "encountering role models with traditional intellectual temperament." Another example "Calling what we do in management ’scientific research’—that’s a stretch. We’re basically word factories crafting texts. (B07)" was coded as "regarding her as an ordinary worker dealing with knowledge or words." Following this initial coding process, the first three authors convened to discuss respective coding schemes and identify areas of agreement and divergence. Through collaborative dialogue and constant comparison, they sought to reconcile any discrepancies and refine the coding scheme to ensure consistency and coherence. This iterative process of coding and recoding allowed us to discern patterns and connections within the data, gradually refining and consolidating our coding scheme. By the end of the open coding phase, we had repeatedly separated and combined the identified initial codes and generated a comprehensive set of eighteen first-order codes, each representing a distinct aspect of the data. These codes formed the foundation upon which subsequent stages of analysis were built, serving as the raw material from which more abstract conceptualizations would emerge during axial and selective coding.

During axial coding, which involved a deeper level of analysis to identify relationships among the initial codes and to group them into broader categories, we engaged in several iterative movements between data and theory to ensure that our coding captured the complexity and interconnectedness of the participants’ experiences. Specifically, each of the first three authors independently reviewed the first-order codes generated during open coding. Then, they conducted a series of collaborative meetings to discuss and collapse these eighteen first-order codes into nine broad second-order codes. For example, grouping the first-order codes "statements about redefining the boundaries of researchers’ job responsibilities," "statements about redefining what it means for research to influence practice," and "statements about redefining the profession" under a broader second-order category labeled "redefinition." These nine second-order codes represented more abstract concepts that captured the underlying themes within the data, setting the stage for the final phase of selective coding.

**Fig 1:**
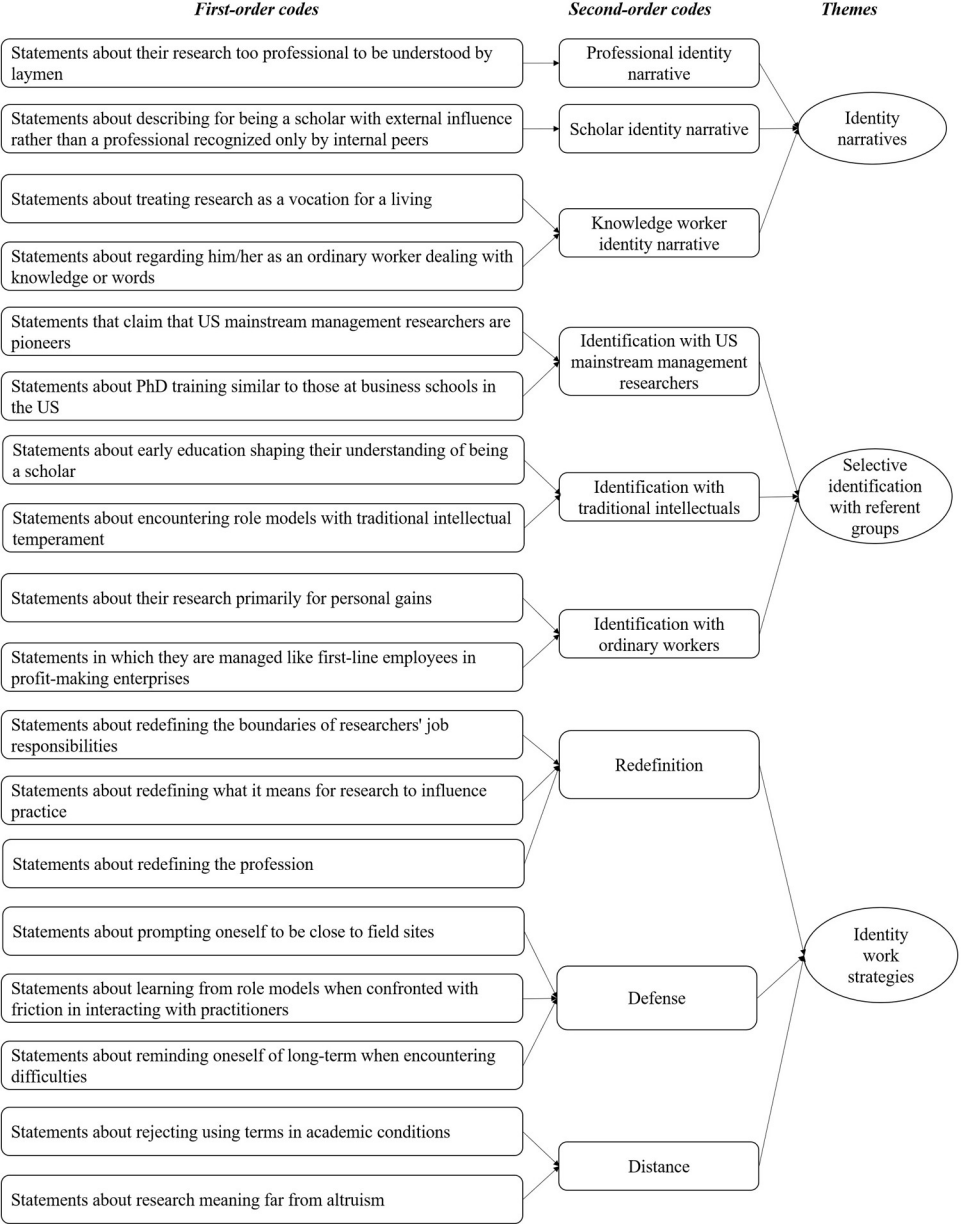
Data coding structure.

Finally, during selective coding, we focused on integrating and refining the second-order codes to develop overarching themes that represented the core findings of our study. Each of the first three authors independently reviewed the second-order codes identified during axial coding and proposed potential themes. They then conducted a series of collaborative meetings to discuss and harmonize these proposed themes. This process involved several iterative cycles of comparison and refinement to ensure that the themes accurately reflected the underlying patterns and relationships within the data. For instance, grouping the second-order codes including "redefinition," "defense," and "distance" into a theme labeled "identity work strategies." By the end of this phase, we had distilled the second-order codes into three aggregate dimensions: identity narratives, selective identification with referent groups, and identity work strategies. These themes reflect three types of identity narratives that Chinese management researchers construct, why they construct such identity narratives, and what identity work strategies they adopt to construct their identities. Table 2 details the specific connotations of the main themes and Fig 1 presents the data encoding structure.

**Table 2:** Specific connotations of the main themes.

Theme	Connotation
Identity narratives	This theme refers to how participants make sense of and articulate their identities in the research–practice gap context.
Selective identification with referent groups	This theme explores how participants selectively identify with certain groups or communities. It delves into the reasons behind these selective identifications and how they influence the participants’ identity narratives.
Identity work strategies	This theme examines participants’ actions to construct and maintain their identity narratives.

### Measures to ensure rigor

To ensure the rigor and credibility of our qualitative research, we implemented several measures throughout the study. First, during the data collection process, each interviewer maintained a reflexive journal after every interview. This practice involved continuously questioning and reflecting on their assumptions, emotions, and values to minimize the influence of personal biases, preconceptions, and assumptions on the data collection and interpretation [40]. Second, during the data analysis, we employed researcher triangulation. The first three authors independently conducted open coding, then met to discuss discrepancies and reached a consensus. This process was repeated for axial and selective coding, with each author working independently before meeting to discuss and agree on the final coding results. Third, after data analysis, we randomly fed back the analysis results to eight interviewees to solicit their opinions and feedback. The chosen participants generally provided affirmative responses, expressing agreement and validation of the research findings. This step is crucial as it aligns with the qualitative research practice of member checking, ensuring that the research outcomes are both researcher-conceived and participant-endorsed [41]. Last, we referred to the consolidated criteria for reporting qualitative studies (COREQ) checklist from Tong et al. (2007) to report crucial aspects of the research team, study methods, study context, findings, analysis, and interpretations [41] (S6 File). This measure enables readers to replicate our findings.

## Findings

Our analysis revealed three distinct identity narratives and respective identity work strategies among Chinese management researchers experiencing identity challenges in both their academic and practical realms in the research–practice gap context. Consistent with the self-categorization theory, our respondents categorized themselves into new referent groups and defined their identities in terms of group membership [42]. In particular, they reflexively identified either with a narrow disciplinary group (US mainstream management researchers or traditional intellectuals) or a broad group of knowledge workers. Three types of identity narratives emerged, namely, professional, scholar, and knowledge worker identities, and each had a unique strategy for identity work, namely redefinition, defense, and distance, respectively.

### Professional identity construction

#### Professional identity narrative

Approximately half of the respondents (13/34) believed that practitioners’ difficulty in understanding management research highlights their professional identity. Considering the exclusivity inherent in the profession, laymen (practitioners) not understanding their professional work (management research) was expected. Thus, the observed divide between research and practical application was not perceived as a salient issue warranting concerted attention. For example, an associate professor explained the rationale for practitioners disregarding her research content.

Initially, sharing my papers didn’t get much buzz, which was kind of disappointing. But then I figured it’s pretty much niche stuff and it’s cool if it’s not everyone’s cup of tea. Then I decided to quit the show-off game. (F02)

According to these respondents, professional identity was derived primarily from acknowledgment within academic circles and remained unaffected by lack of recognition among lay practitioners. Moreover, most interviewees mentioned top-tier journals’ authority, contending that articles featured in such publications undergo rigorous peer reviews and garner extensive peer acknowledgment. Therefore, the consensus among these researchers was that the higher the frequency of publications in top-tier journals, the greater the enhancement of their professional identity. J06 commented:

I got to say, top-tier journal articles are top-notch—rigorous, detailed, and pro. Land a paper there and peers see you’ve really nailed it, marking you as an ace researcher.

#### Identification with US mainstream management researchers

These respondents constructed a professional identity primarily because, during their academic socialization, they gradually considered US mainstream management researchers as a referent group and identification target. Specifically, the US educational model and management style profoundly influence the academic socialization of Chinese management researchers. Various PhD courses are now in leading business schools in China similar to top-tier business schools in North America. As articulated by one respondent who received his PhD from one of the top two universities in China, the doctoral education systems closely mirrored those of top-tier business schools in the US:

The PhD program at our university is pretty much cloned from the US one—identical curriculum, same teaching methods, and research methods. My PhD advisor said doing a PhD here is like doing it in the US universities. (F06)

A long-standing focus on US academic standards resulted in these respondents viewing US mainstream management researchers (who have published in top-tier US journals) as their referent group and identification target. As indicated by F08, "We obediently and unquestioningly look to the US academic standards and readily adopt their criteria as professional benchmarks during evaluations. Unconsciously, we perceive US mainstream management researchers as authoritative figures." Hence, they tended to consider US mainstream management researchers’ research as the most normative, legitimate, and professional. Some respondents mentioned that when they observed US professors showing little concern for the research–practice gap and engaging in exquisite but irrelevant studies, rationalized such studies as a reflection of professional identity. For example, a respondent who was a visiting researcher in the US stated the following.

We just cranked out three papers. Looked for research questions from literature, collected data from the Internet, clueless about where it came from. Turned that into articles, chatted about managerial implications, and submitted them. I know it’s a bit wild, but it’s the same abroad (in the US) and even more so. (J03)

Similarly, during the initial stages of his doctoral education in mainland China, another interviewee did not understand the practical relevance of mainstream management research. He experienced a sense of depression and anguish stemming from this concern. However, after one year of being a visiting researcher in the US and observing how referent group members conducted management research, he found solace regarding the disconnect between research and practice but he also gained a heightened sense of confidence in his professional identity, as he explained:

My year in the US totally transformed me; after I came back, I evolved into a professional. Noticed lots of big-name US profs were super focused on their niche, kind of ignoring the big picture and real-world effects. That kicked up my confidence and eased my worries about the gap between research and practice. After all, I recognized that numerous influential figures in the US academic landscape shared a similar professional orientation. (F06)

These narratives suggest that these respondents constructed their professional identity through academic socialization and appeared to have rid themselves of the troubles of the research–practice gap. Furthermore, they regarded US mainstream management researchers as role models, considering their approach to the relationship between research and practice as a professional standard. Consequently, they emulated these role models to construct a professional identity.

#### Identity work: Redefinition

When challenged by the accusation that their research cannot influence practitioners, potentially threatening their professional identity, the respondents who regarded themselves as professionals applied a "redefinition" identity work strategy. This implementation involved redefining the boundaries of researchers’ job responsibilities, what it means for research to influence practice, and the profession. By doing so, they intended to repair, maintain, and strengthen their preferred professional identity.

First, some respondents sought to redefine the roles of management researchers and practitioners. Some metaphorically depicted management researchers and practitioners as two distinct occupations, namely "craftsmen" responsible for forging weapons and "commanders" tasked with leading troops into battle. Accordingly, researchers were perceived to have a duty limited to crafting "weapons" for practitioners rather than replacing practitioners in directly engaging in the "battlefield." As stated by F05, "It seems that our research is akin to stocking the arsenal and practitioners choosing what they want to use." Thus, by redefining the scope of responsibilities for researchers and practitioners, these respondents strategically retreated from their work duties to a domain that did not jeopardize their professional status. Moreover, the publication of management research papers in top-tier journals in the US supported their adoption of this identity work tactic. A professor in the operation management field explained:

Most top US journal articles aren’t super practical, but they’re very delicately constructed. Their prevalence and dominance prove their value. It’s just how the game is played. (J05)

Second, some respondents redefined research’ influence on practice, arguing that assessing the impact of management research on practice should be based on the collective findings of multiple studies rather than relying on the outcomes of one or limited studies. This tactic was aimed to address the critique that solely individual management studies may not substantially affect practice. They posited that the value of an individual study was akin to grains of sand—each grain might seem inconsequential on its own, but collectively they were an indispensable component in building a towering structure. In other words, the accumulation of numerous seemingly meaningless studies synthesized the force propelling theoretical advancement, offering guidance for practical applications. An assistant professor in the OBHRM field stated the following.

Thinking one study can guide practice is just kidding ourselves. Real research is about big-picture stats, not one-shot tips for a company. Doing management research is not gathering or telling stories; instead, it is searching and discovering the universal laws underlying the management world, regardless contexts of US or China. Intentionally pursuing the practical value of a specific study seems incompatible with scientific principles. But yeah, when you stack up a bunch of studies, that’s when you get theories that can tackle most management issues. (L06)

Moreover, they perceived that the impact of research on practice did not necessarily imply that research must guide practice. By contrast, practice could guide research development; subsequently, research could synthesize the experiences of practice. As expressed by an associate professor in the marketing field:

Management is practical, but that doesn’t mean theory always leads practice. Often, theory plays catch-up, summarizing practice. (F02)

However, the associate professor did not elaborate on how to advance based on summarizing practical experiences, making their advocacy of "research summarizing practical experiences" seem more like a less scrutinized pretext for justifying the claim that research can influence practice.

Finally, redefining the profession is another tactic used by respondents who described themselves as professionals to legitimize the divide between management research and practice. The term "profession" originally referred to exclusive groups formed by individuals using specific abstract knowledge to address particular situations, characterized by an altruistic orientation [43]. However, these respondents redefined the profession by emphasizing exclusivity while undervaluing altruistic attributes, giving legitimacy to research disconnected from practice and then maintaining their desired professional identity. They argued that numerous management studies, although not directly contributing to societal well-being, held signaling value within the management academic community. Such studies proved to the discipline community that researchers possessed the skills in writing management research papers that nonprofessionals may not master, demonstrating their professional capability. An associate professor indicated:

Writing academic papers isn’t really about their realistic significance or practical implications to managers. It’s more symbolic, like a ticket into academia, like holding a luxury bag expected by the high society. (F07)

### Scholar identity construction

#### Scholar identity narrative

Our interviews revealed that almost one-third of the respondents (11/34) aspired to become scholars rather than professionals; they perceived a crucial distinction between these two identities, with the former emphasizing a sense of social responsibility and mission akin to traditional intellectuals. Moreover, they advocated for altruistic-oriented research with societal impact, transcending mere recognition within the academic community. One informant referred to his accountability to society: "I see myself working for the common good of society rather than working solely for the academic community" (L05). Hence, they explicitly resisted management research that did not impact practice. In their view, commendable management research should examine issues genuinely pertinent to practitioners and the public comprehensively and profoundly. Furthermore, the purpose should result in a tangible impact on practitioners and the public, guided by their concerns, rather than being constrained by artificially narrow disciplines or professional domains, as often observed in professionals. A professor engaged in interdisciplinary management research articulated:

For me, being a member of a specific discipline or profession isn’t crucial. I don’t see myself as an economist, linguist, sociologist, or management scientist. I view myself as a scholar, a thinker—that’s my aim. Those so-called professions? They don’t hold meaning for me. (J01)

Since they emphasized the impact of research on the external world, they perceived that practitioners and the public should assess management research’s value instead of being limited to internal evaluations from peers who are supposedly similar to "professionals." An associate professor claimed:

Ideas that matter, ones that even non-academics get. We’re not just penning stuff for our circle. We’re shooting for ideas that resonate beyond, even outside our turf. (J04)

#### Identification with traditional intellectuals

Early educational experiences, including family education and primary and secondary education, are crucial for helping the respondents hoping to become scholars identify traditional intellectuals as a referent group and form a scholar identity. Some demonstrated how early exposure to a family of scholars influenced their career choices and understanding of scholar identity. For example, L07 shared, "My father was a university teacher and my mother also worked in a university, so my personality was significantly influenced by the scholarly environment … Since childhood, I’ve aspired to be a scientist, and I desire to engage in research and become a scholar contributing to the betterment of society." Further, other interviewees mentioned the values instilled in primary and secondary schools. For instance, L10 shared an ancient poem by Fan Zhongyan (*范仲淹*, a role model of intellectuals in ancient China) that she learned in secondary school: "Worry before the people fear something will happen and be happy after the people are happy." Since then, she has been keenly interested in public welfare, frequently engaging in discussions with classmates and friends. Even today, as a researcher in the finance and accounting field, she continues to prioritize the interests of the public. She stated, "I believe that good research should primarily serve the welfare of society rather than merely aiming to maximize shareholder profits. If a study is solely focused on finding means for companies to make money, in my view, this is bad research since it does not maximize societal benefits. That is why I pay considerable attention to corporate social responsibility in my research."

Furthermore, during their academic socialization, the respondents who wanted to be scholars were inspired by role models. They observed management researchers who could effectively bridge research and practice, providing them the confidence to pursue management research that has both theoretical and practical significance. F01 noted:

Prof. X from Wharton School is my role model. His work really speaks to entrepreneurs and genuinely helps them. He has shown me that it’s possible to create research that’s top-notch and valued by the real-world folks.

#### Identity work: Defense

The strategy of defense refers to those respondents who want to be scholars taking measures to counter the research–practice gap, adhere to pursuing the public value and practical utility of management research, derive research insights from practical experiences, and apply them back to practice. We identified that this strategy relies on three tactics: prompting oneself to be close to field sites, learning from role models when confronted with friction in interacting with practitioners, and reminding oneself of long-term when encountering difficulties.

First, to conduct practically impactful management research, these respondents consistently motivated themselves to be immersed in field sites. Further, drawing on commonly employed ethnographic research methods from disciplines such as anthropology and sociology, they strived to integrate themselves into the real managerial world. As L07 said, "I’m eager to conduct research deeply rooted in Chinese practical contexts; hence prolonged close-range observation, experiential immersion, and interviews are necessary." For some, close engagement with field sites was not aimed at producing academic papers. Instead, this aim stemmed from a genuine desire to generate insights that could inspire management practitioners. Moreover, they were willing to invest additional time and effort in this pursuit. For example, an assistant professor indicated the following.

I aim for my research to truly impact management practice; hence I dive into understanding real-world happenings, even if they stray from my paper. I feel I must get it; without understanding, there’s no way to offer practical insights. (L02)

However, despite their intention to involve themselves in practical fields, communication with practitioners was occasionally hindered. Specifically, disparate expectations in communication between researchers and practitioners contributed to this challenge. Researchers often approached their research with specific research questions, aiming to summarize solutions through interaction with practitioners. These questions might pertain to problems already resolved by practitioners or issues no longer pertinent to them. Conversely, practitioners hoped that researchers could assist in overcoming the current challenges. Thus, such misalignment in expectations can lead to communication barriers. For instance, L02, an assistant professor who researched entrepreneurs, mentioned:

I chat with entrepreneurs who throw hard questions at me, wanting straightforward models: "You’re the expert having knowledge, tell us what to do." But often I’m stumped by their urgent problems … And some say, "Books and theories got it wrong. They preach ’focus’ in startups, but how can I do it when I’m struggling to pay salaries? My business is sinking. Why ’focus’?"

When confronted with friction in interacting with practitioners, certain respondents proactively sought guidance from their role models. For example, L02 stated the following:

Whenever I meet someone authoritative, I ask for advice to clear up my confusion. My mentor once told me, in corporate research, don’t act like you have all the answers. Make it clear to practitioners from the start that we’re here to learn, not solve all their problems.

Finally, research grounded in practice often demands a substantial investment of time and effort, with a long output cycle for research outcomes that do not match the short-term evaluation system prevalent in Chinese universities. For early career researchers in particular, who have not secured tenured positions, dismissal is a risk. When they experience difficulties that threaten the survival of their academic careers, the respondents who constructed their career identity as scholars often reminded themselves to adhere to a long-term perspective. One assistant professor articulated:

Research work is like a marathon, not a sprint. It’s about diving deep into what truly matters, not just churning out quick empty papers for ticking boxes. I keep telling myself: Don’t rush, don’t let the outside noise steer my course. Sure, it’s easy to know this but challenging to do. So I frequently remind myself to play the long game. (L10)

### Knowledge worker identity construction

#### Knowledge worker identity narrative

In contrast with the identity work strategies of redefining and defending that helped Chinese management researchers construct positive identities (professional and scholar identities), certain respondents described themselves as ordinary knowledge workers adopting distance as an identity work strategy to enact and sustain a sense of disillusionment. Within this narrative, they did not construct their career identity as a positive or powerful identity; instead, they downplayed or denied links with their research work. Furthermore, several participants repeatedly described conducting management research as "meaningless" and "bullshit" (interviews with F04; L11; B06–08), challenging achieving positive self-concepts through research work. They perceived that a sense of meaninglessness led these individuals to view academic work merely as a routine vocation for livelihood, positioning themselves as ordinary workers working with knowledge or words. A doctoral student expressed:

For me, writing papers is switching to work mode, treating research as a job without interest or passion. Just completing the task itself is sufficient. (B01)

#### Identification with ordinary workers

These respondents often did not explicitly embrace the identity of knowledge workers when embarking on the academic path. Notably, they once aspired to pursue the identity of a scholar, aiming to conduct research beneficial to managerial practice and society. An assistant professor articulates the following:

I used to perceive the academia as a sacred place, harboring grand aspirations. I aimed to achieve a magnificent ambition through academic work, aspiring to become a person of societal value by contributing to meaningful endeavors. (L09)

However, due to the increasingly strict requirements for normativity and rigor in academic papers, these respondents often felt confined and constrained by established standards, akin to "dancing delicately on stage with chains" (L09). This issue often leads to a weakening of their focus on practical significance. Consequently, they did not perceive the value their research could bring to society, and practitioners’ skepticism toward research conclusions further intensified their doubts and negations regarding the meaningfulness of their work. An assistant professor in the strategic field mentioned the following:

I often fret over whether my research really helps businesses—like does it boost profits or give them an edge? But the execs I chat with seem doubtful; they say a paper’s ideas aren’t game-changers for them. Leaves me wondering what mark my work’s really making. Still figuring out if it’s got value, to be honest. (L04)

These respondents did not perceive the altruistic value and public attributes of conducting management research, consequently missing the sense of accomplishment and professional passion. Their research seemed to primarily serve personal gains, including obtaining a doctoral degree, achieving tenure, securing professional promotions, or receiving performance bonuses. Consequently, they view themselves as no different from ordinary workers, exhibiting solid characteristics of a secular work identity. An assistant professor stated:

Right now I’m just doing research to snag tenure. It’s like ticking boxes for the boss—nothing special. No fame, no dazzle in it for me. (L11)

Often, they deliberately conceal their researcher identity or self-deprecate themselves as pedantic word processors. For example, a doctoral student in the field of logistics perceived her work as being a wordsmith:

Calling what we do in management ’scientific research’—that’s a stretch. We’re basically word factories crafting texts. (B07)

Another reason leading these respondents to identify with the identity of ordinary workers is corporate-style management. Currently, elite business schools in China actively adopt management systems from profit-making enterprises, such as implementing performance assessments to manage researchers and establishing numerous assessments or competitions, aimed at the speedy and efficient publication of papers. Such systems lead to researchers working overtime to write papers, reducing the time originally intended for investigation and surveys. Thus, the high-intensity workload leads to psychological diminishment and instrumentalization of the researchers’ self-identity. As an assistant professor expressed:

We have a major assessment every three years and a minor one every year. We’ve become paper machines buried in writing papers every day. Where is the time to get close to practice? Aren’t we just screws in the factory? (L04)

#### Identity work: Distance

Regarding constructing the identity of a knowledge worker, these respondents attempted to distance themselves from the academic community, avoiding defining themselves as members. Instead, they sought closeness with a broad community of ordinary workers, using the language and behaviors commonly associated with these demographics.

First, they often used terms common in other industries when referring to their academic work. For example, they described research projects as KPIs that must be completed, comparing themselves to screws in the machinery of their institutions’ performance tasks. Their aims for using this strategy were not only to demean research work but also to diminish and instrumentalize themselves, eliminating the distinctiveness of their work identity. For example, an assistant professor stated the following on having to continue conducting research projects.

Academia’s just a job to me now. You can’t stop for lack of mood; hit a KPI then onto the next. Even if it sucks, you do it. It’s like any job—you don’t quit over dislikes. Of course, you can resign, but there are always things you have to endure. (L11)

Evidently, management research had been relegated to routine piecework. Furthermore, the research meaning of the respondents who regarded themselves as knowledge workers was often far from altruistic. Since their management research typically had limited impact on practical applications, they struggled to experience a sense of achievement and significance from academic work. Instead, they portrayed academic work as similar to pursuing personal interests, especially material gains. For example, an associate professor specified:

Research isn’t useless, it pays the bills … Come bonus time, I see the point of my articles—it’s cashing them in for a bonus, which helps alleviate the emptiness inside. (F04)

Notably, some respondents viewed their identity as knowledge workers as a temporary compromise due to current circumstances. They hoped to maintain their initial intention of contributing to society and aspired to become genuine scholars. These respondents often did not have tenures and were experiencing career uncertainty and insecurity, prompting them to temporarily compromise by engaging in research that they might not fully endorse, such as mediocre studies without practical significance. Another assistant professor experiencing upcoming probationary assessments stated:

As a scholar, you can’t live only for yourself; it’s about serving society. But now, survival is my focus. Once I snag tenure, I’ll dive into work I believe really makes a difference. (L09)

For him, cynicism is a means of protecting oneself while challenging the idealized scholar identity of the management researcher.

## Discussion and conclusions

This study addresses how Chinese management researchers construct their varied career identities in response to the identity threat posed by the research–practice gap. Using a qualitative research method, we revealed that the research–practice gap does influence the construction of career identities among Chinese management researchers. However, individual researchers tend to develop distinct identity narratives. We identified three types of identity narratives: professional, scholar, and knowledge worker. Furthermore, we discovered that the primary reason for these varying identity narratives is the different referent groups chosen by the researchers, which, in turn, lead to differences in seeking out and emulating role models. Specifically, they reflexively identified either with a narrow disciplinary group (US mainstream management researchers or traditional intellectuals) or a broad group of knowledge workers. These choices shape their identity narratives. In addition, Chinese management researchers adopt different identity work strategies to construct and maintain their respective career identity narratives. Those identifying as professionals engage in redefinition, scholars employ defense, and knowledge workers utilize distance. These strategies help them navigate the tensions between academic rigor and practical relevance, illustrating the complex interplay between individual motivations, institutional expectations, and societal influences in the context of the research–practice gap.

Specifically, certain Chinese management researchers regard themselves as professionals because, during their academic socialization, they consider US mainstream management researchers as a reference and then their research as the epitome of normative and professional standards. When they observe members of this referent group showing limited concern for the research–practice gap and conducting exquisite studies irrelevant to practice, they rationalize such studies as a reflection of professional identity. To maintain and strengthen their ideal professional identity, they employ the "redefinition" identity work strategy, for example, redefining the boundaries of researchers’ job responsibilities, what it means for research to influence practice, and the profession.

Other Chinese management researchers tend to identify with traditional intellectuals since they were young due to their early lives. They are keen on the scholar identity and advocate that management research should influence practice for exerting external influence. During their academic socialization process, they are often fortunate to be inspired by role models capable of effectively bridging the gap between research and practice and exerting influence in both the theoretical and practical realms. Thus, the role models serve as exemplars for the integration of scholarly pursuits with real-world applications. To form and protect the scholar identity, they adopt the identity work strategy of defense, comprising three tactics, namely, prompting oneself to be close to field sites, learning from role models when confronted with friction in interacting with practitioners, and reminding oneself of long-term when facing difficulties.

There are also Chinese management researchers with an ordinary knowledge worker identity, and they believe that they work primarily for self-interest, similar to first-line employees in profit-making enterprises. To sustain their ordinary knowledge worker identity, they attempt to distance themselves from the academic community and its traditional calling, consciously refraining from identifying themselves as socially responsible scholars. Table 3 summarizes the main findings.

**Table 3:** Summary of three career identity narratives of Chinese management researchers in the research–practice gap.

Identity narratives	Professional identity	Scholar identity	Knowledge worker identity
Referent groups	US mainstream management researchers	Traditional intellectuals	Ordinary workers
Identity work strategies	Redefinition	Defense	Distance

This study contributes to the nascent literature on management researchers’ career identity construction in the research–practice gap context. Previous studies have demonstrated that management researchers often adopt hybrid identities to navigate the tension between scholarly pursuits and practical applications [3, 10]. However, our study reveals a different pattern among Chinese counterparts—they tend to develop single identities. This divergence likely stems from our focus on informants based in typical higher education institutions in China, whose primary roles involve producing high-quality research and publishing in esteemed journals rather than being "academic- practitioners" who engage in both theory and practice [1–3, 10].

Partly consistent with Gulati (2007) [9], our research identifies that some Chinese management researchers form their identities as scholars; however, different from his findings, the practitioner identity narrative does not exist in the Chinese context. As previously described, the participants in this study were not required to engage in managerial tasks directly; hence, they did not construct a practitioner identity.

Second, our study contributes to the understanding of identity construction among professionals. Previous research on professionals’ identities has primarily focused on situations where members of professional communities share a common ideal career. Under such circumstances, professionals observe and emulate role models to construct their career identities [14, 15, 19]. Our study examines the field of management academia, which is characterized by a lack of consensus on an ideal identity. It illustrates how Chinese management researchers select referent groups before seeking and emulating role models. Specifically, they reflexively identify either with a narrow community of discipline (such as US mainstream management researchers or traditional intellectuals) or with a broad community of workers (ordinary workers), strategically positioning themselves within these referent groups. This finding supports Empson’s (2013) observation that individuals resolve identity conflicts by finding a peer group with whom they identified [3].

Third, our study demonstrates that individuals do not always construct positive identities to enhance self-esteem. Previous research on identity work has generally assumed that the quest for positive meaning motivates identity construction and that individuals adopt identity work strategies to achieve or preserve positive identity [44]. However, Ahuja et al. (2019, p. 989) challenged this perspective, arguing that "identity work may not always lead to the accomplishment of a positive sense of self but can express a sense of disillusionment [45]". They found that apart from identity work strategies of idealizing and reframing that provide opportunities for self-affirmation, some junior architects adopt a cynical and self-deprecating emotion talk to distance themselves from preconfigured notions of an ideal career self. Similarly, our study shows that, while professional and scholar narratives enhance social identity and allows researchers to construct positive identities, the knowledge worker narrative expresses frustration and disillusionment among some Chinese management researchers when they feel disappointed in their research experiences. Furthermore, our study responds to Beech et al. (2016), who called for further insights into identity work that is not self-affirming [46].

Notably, our study reveals a nuanced divergence between informants’ professional identity construction and traditional definitions of professionalism, which historically underscores serving the public interest over seeking intra-group recognition [47]. Participants in our study actively redefined the profession, emphasizing exclusivity and redefining the job responsibilities of management researchers and practitioners and what it means for research to influence practice. They strive for professional legitimacy through a "redefining" identity work strategy. This approach highlights the potential of redefinition to foster a positive professional identity under conditions where career identity encounters challenges from peers [48]. Moreover, our findings offer a contemporary perspective on "professional retreat," traditionally viewed as delegating lower-skilled tasks to preserve work purity and prestige positions [43]. Contrary to the traditional view of striving for the most valued aspects of the profession, our analysis suggests that professional retreat may also involve relinquishing these aspects to maintain the legitimacy of professional identity. Furthermore, our findings resonate with other commentators’ criticism that increasing management researchers have become career-oriented, producing esoteric knowledge for a small community of peers [26].

Our study also reveals that approximately one-third of the participants who aspire to be scholars try to bridge the research–practice gap. However, others either justify the divide by using a redefinition strategy or maintain the divide by devaluing their career identity, feeling helpless about the divide. This finding aligns with existing literature, which indicates that numerous Chinese management researchers do not appear to be attempting to bridge the research–practice gap [28].

Finally, due to the provisional nature of identity work [44], management researchers may construct distinct identities at different academic career stages. Similar to the experiences of interviewees L04 and L10, although they yearned for the identity of scholars, the pressure of the "publish or perish" environment forced them to adopt the identity of knowledge workers temporarily. However, this study does not aim to capture the dynamic process of identity construction. Further research could employ a longitudinal method to track participants over an extended period, investigating how management researchers’ identity narratives and strategies evolve across different career phases.

## Supporting information

S1 File Interview schedules. (PDF)

S2 File Participant information leaflet. (PDF)

S3 File Consent form. (PDF)

S4 File Interview data. (DOCX)

S5 File Ethical approval. (PDF)

S6 File COREQ checklist. (PDF)
