# Supplementary material for: Professional, scholar, or knowledge worker? Identity construction of Chinese management researchers amid the research–practice gap
Source: PLoS One. 2024 Aug 29;19(8):e0306833. doi: 10.1371/journal.pone.0306833 (PMC11361602; doi:10.1371/journal.pone.0306833)
Supplement: S1 File — (PDF) [file pone.0306833.s001.pdf]

Interview Schedules

Study Title: Institutional Transformations and the Survival Dynamics of Management Scholars in Chinese Business Schools

Investigator(s): Shubo Liu; Mengna Lv; Qiuli Huang

Personal Learning and Growth Experience	· Why did you choose the academic path?<br>· What influences did you encounter before making this decision?<br>· Before pursuing a PhD, what were your perceptions of academic research? And what were your expectations?<br>· When you were pursuing a PhD, what major challenges did you face? And what were the sources of routine pressure?<br>· What does being a doctoral student mean to your relatives and friends? How does their perception differ from your own understanding?
Career Development Experience	· Could you briefly review your experience of seeking employment in academia?<br>· What were the interview process and content requirements? What questions were you asked during interviews?<br>· What do you feel are the criteria for academic recruitment (implicit and explicit)?<br>· What are the job responsibilities and challenges after joining academia? How do you handle them?<br>· After starting your job, did you experience any gap in your skills or knowledge? If yes, could you elaborate on the specific area of the gap and the reasons behind it?<br>· Could you choose research topics you're passionate about?<br>· Could you provide examples of instances where you encountered disparities between reality and your ideals in your professional or personal life? How do you typically address these differences?<br>· What aspects of your organization have "Chinese characteristics"? What interesting organizational phenomena exist?<br>· How diverse are your colleagues in your department?<br>· Do you have a role model around you?<br>· What do you enjoy most about your work? What activities give you the most fulfillment?

	• Do you encounter conflicts and contradictions in your work?<br>• How do you evaluate the significance of your research work?<br>• What do you think the “academic community” is like?<br>• Do you have autonomy and freedom to define your job content? Is this autonomy expanding or diminishing? Why?<br>• What are the main tasks currently? If you were to assign proportions to them, what would they be?<br>• Based on your publishing experience, what are the requirements for publishing articles in top-tier journals? In comparison to domestic and international journals, what are the advantages and disadvantages of publishing in each? How do you define “good management research”?
Reflection and Outlook	• If given another chance, would you still choose the academic path?<br>• If you could give yourself some growth advice from the past, what would it be?<br>• What are your requirements and expectations for your future career, or what kind of scholar do you aspire to be?<br>• If we could make some changes now, what aspects of business schools or management discipline in China do you think need the most adjustment, and how would you like them to change?
