# Supplementary material for: Professional, scholar, or knowledge worker? Identity construction of Chinese management researchers amid the research–practice gap
Source: PLoS One. 2024 Aug 29;19(8):e0306833. doi: 10.1371/journal.pone.0306833 (PMC11361602; doi:10.1371/journal.pone.0306833)
Supplement: S2 File — (PDF) [file pone.0306833.s002.pdf]

中央财经大学 商学院
Central University of Finance and Economics | Business School

Participant Information Leaflet

Study Title: Institutional Transformations and the Survival Dynamics of Management Scholars in Chinese Business Schools

Investigator(s): Shubo Liu; Mengna Lv; Qiuli Huang

Introduction

You are invited to take part in a research study. Before you decide, you need to understand why the research is being done and what it would involve for you. Please take the time to read the following information carefully. Talk to others about the study if you wish.

Please ask us if there is anything that is not clear or if you would like more information. Take time to decide whether or not you wish to take part.

Who is organising the study?

The investigators all come from the Business School of Central University of Finance and Economics. The research team consists of one associate professor (Shubo Liu) and two doctoral students (Mengna Lv; Qiuli Huang).

What is the study about?

The swift evolution of the Chinese economy and the rapid advancements in commerce have provided a foundation and ample opportunities for management research and education. Over the past three to four decades, the management discipline, crucial for cultivating urgently needed managerial talents, has experienced rapid growth. However, at present, most management scholars focus on the development of business organizations but overlook the distinctive characteristics of their own discipline field. Consequently, Chinese management scholars have also become a neglected group. To address this dilemma, we aim to conduct interviews to retrospectively and systematically understand the historical development of Chinese management discipline, and the perceptions, attitudes, and behaviors of management scholars within this context.

What would taking part involve?

We would like to conduct a semi-structured interview with you, focusing primarily on the following topics: "Personal academic background, perceptions of the current development status of management studies in China, perspectives on the existing issues within the Chinese management academic community, and understanding of one's own academic identity." Interview schedules are as follows:

Personal Learning and Growth Experience	· Why did you choose the academic path?<br>· What influences did you encounter before making this decision?<br>· Before pursuing a PhD, what were your perceptions of academic research? And what were your expectations?<br>· When you were pursuing a PhD, what major challenges did you face? And what were the sources of routine pressure?<br>· What does being a doctoral student mean to your relatives and friends? How does their perception differ from your own understanding?
Career Development Experience	· Could you briefly review your experience of seeking employment in academia?<br>· What were the interview process and content requirements? What questions were you asked during interviews?<br>· What do you feel are the criteria for academic recruitment (implicit and explicit)?<br>· What are the job responsibilities and challenges after joining academia? How do you handle them?<br>· After starting your job, did you experience any gap in your skills or knowledge? If yes, could you elaborate on the specific area of the gap and the reasons behind it?<br>· Could you choose research topics you're passionate about?<br>· Could you provide examples of instances where you encountered disparities between reality and your ideals in your professional or personal life? How do you typically address these differences?<br>· What aspects of your organization have "Chinese characteristics"? What interesting organizational phenomena exist?<br>· How diverse are your colleagues in your department?<br>· Do you have a role model around you?<br>· What do you enjoy most about your work? What activities give you the most fulfillment?<br>· Do you encounter conflicts and contradictions in your work?<br>· How do you evaluate the significance of your research work?<br>· What do you think the "academic community" is like?<br>· Do you have autonomy and freedom to define your job

	content? Is this autonomy expanding or diminishing? Why?<br>· What are the main tasks currently? If you were to assign proportions to them, what would they be?<br>· Based on your publishing experience, what are the requirements for publishing articles in top-tier journals? In comparison to domestic and international journals, what are the advantages and disadvantages of publishing in each? How do you define “good management research”?
Reflection and Outlook	· If given another chance, would you still choose the academic path?<br>· If you could give yourself some growth advice from the past, what would it be?<br>· What are your requirements and expectations for your future career, or what kind of scholar do you aspire to be?<br>· If we could make some changes now, what aspects of business schools or management discipline in China do you think need the most adjustment, and how would you like them to change?

The interview is expected to last for 1-2 hours, and you may extend or shorten it according to your preference.

You can choose the interview location at your convenience, such as your office, a meeting room, or a café.

We fully respect your willingness to answer questions. If there are any questions you prefer not to answer, we can skip them, and you also have the right to withdraw at any time.

If you are willing, we would like to record the interview for accuracy. Afterwards, we will transcribe the interview text for your review. Any information you do not wish to disclose can be omitted.

Do I have to take part?

No. Participation in this study is completely voluntary and choosing not to take part will not affect you in any way. You can also choose to withdraw your participation without giving a reason by contacting one of the research team. Further details about withdrawing from the study are provided later on in this document.

What are the possible benefits of taking part in this study?

Through conducting this research, we aim to explore the current ecological environment of Chinese business schools and the management academia,

understanding the academic experiences of each management scholar. Our interaction may bring the following benefits to you and the academic community:

- Our communication may help you to review your academic career trajectory from its inception to the present stage. It may assist you in summarizing the lessons learned along the way, providing inspiration for a smoother academic journey ahead.
- Understanding the doctoral experiences of the interviewees also aids in analyzing the challenges and pressures faced by management doctoral students. This analysis can provide suggestions for improving doctoral training in business schools, helping them better support students' academic development.
- Analyzing the challenges and sense of disparity felt by management researchers in their work can provide suggestions for improving the work environment and incentive mechanisms for university managers, enhancing researchers' job satisfaction and well-being.
- Analyzing the existence and characteristics of the management academic community can help understand the organizational culture and values within academic institutions, providing reference for building a more inclusive and innovative academic environment. Additionally, it can offer suggestions for academic system reforms and talent cultivation to university managers and policy makers, promoting the healthy development of the academic environment.

What are the possible disadvantages, side effects or risks, of taking part in this study?

Time Commitment:

The interview requires participants to invest a certain amount of time and energy, which may encroach upon their daily work and personal life. To mitigate this impact, we will arrange flexible interview times and locations as much as possible, allowing you to engage in an interview at your most convenient time and location, minimizing disruptions to your work and life.

Concerns about Personal and Professional Image:

Participation in interviews may affect the establishment and maintenance of personal and professional image, leading to concerns for participants. To alleviate these concerns, we will ensure that your statements are kept strictly confidential and used solely for academic research purposes, without disclosure to any other individuals or institutions. In addition, our interview guidelines have been pre-tested, ensuring a neutral questioning approach to safeguard against any potential emotional distress for you.

Expenses and payments

Any expenses you may incur during the interview process, such as travel expenses or meal costs, will be reimbursed to ensure that you do not incur any financial burden as a result of participating in the interview. Additionally, as a token of our appreciation for your acceptance of our interview, we would like to offer you three books: “The Slow Professor,” “From Discretion to Science,” and “Critique, Construction, and Reflection of Management Research: Towards a Journey of Self.”

Will my taking part be kept confidential?

Regarding the collection and use of your data, we will provide detailed explanations below. We will make every effort to ensure the security and confidentiality of your data and ensure that you have a clear understanding and informed consent of the data usage and processing.

- How your data will be collected:

We will collect your data through face-to-face interviews, and record it using professional recording pen (with your prior consent). The recorded interview will be automatically transcribed into text. Subsequently, we will meticulously review and check the text, then send it to you for verification.

- For what purpose their data will be processed:

Your data will only be used for this research project, solely for our understanding of the career development trajectory, work experiences of management scholars, as well as the development history, organizational management status, and institutional environment characteristics of the management discipline and units (business schools) involved.

- How your data will be de-identified:

To protect your identity information, you will be assigned a research identifier to replace your real identity. Identity information associated with this identifier will be stored separately, encrypted, and kept separate from research data.

- How your data will be stored securely during the data collection period; for the duration of the research project and beyond:

During data collection, as well as during and after the research project, we will strictly protect your data security through encrypted storage and access control measures.

- Minimize collection of personal private data:

We will adhere strictly to privacy protection principles, minimizing the collection of personal sensitive information such as race and religious beliefs. We are committed to safeguarding your privacy and will only gather necessary information to fulfill the research objectives.

- Data access permissions:

Only members of the research team will have access to your data, and we will strictly adhere to confidentiality agreements.

- Data transfer and sharing:

If it is necessary to transfer your data or share it with other organizations, we will seek your opinion in advance. If you agree, we will ensure that these organizations also provide equivalent data protection measures.

- Confidentiality limitations:

We will make every effort to protect the privacy of your data, but in certain circumstances, such as when you or others may be in danger, we have an obligation to report to the relevant authorities.

- Use of direct quotations:

If we intend to use direct quotations in research reports or publications, we will seek your consent in advance and ensure that the quoted content does not disclose your personal identity.

What will happen to the data collected about me?

As a publicly-funded organisation, the Central University of Finance and Economics has to ensure that it is in the public interest when we use personally-identifiable information from people who have agreed to take part in research. This means that when you agree to take part in a research study, such as this, we will use your data in the ways needed to conduct and analyse the research study.

We will be using information from you in order to undertake this study and will act as the data controller for this study. We are committed to protecting the rights of individuals in line with data protection legislation.

Research data will be **pseudonymised** as quickly as possible after data collection. This means all direct and indirect identifiers will be removed from the research data and will be replaced with a participant number. The key to identification will be stored separately and securely to the research data to safeguard your identity.

Data Sharing

The Central University of Finance and Economics has in place policies and procedures to keep your data safe.

This data may also be used for future research, including impact activities following review and approval by an independent Research Ethics Committee and subject to your consent at the outset of this research project.

What will happen if I don’t want to carry on being part of the study?

Your participation in the interview is entirely voluntary, and you may withdraw from the interview at any time without providing a reason. This will not result in any adverse consequences for you. If you wish to terminate the interview during the process, please inform us directly, and we will fully respect your decision. If, before our data analysis, you no longer wish for us to analyze your interview data, you can contact any member of our research team directly, and we will delete all your data immediately, ensuring it will not be used for future research.

It is important to note that if you want to withdraw from the interview after data analysis, we will be unable to retract your data, as it will have already been anonymized.

What will happen to the results of the study?

The results of this study will be thoroughly analyzed and interpreted by members of our research team. We plan to submit the research findings to reputable academic journals for publication and undergo peer review by experts in the field. Additionally, we may present our results at academic conferences or seminars to foster discussion and dissemination within the academic community.

Who has reviewed the study?

This study has been reviewed and given favourable opinion by the Business School of Central University of Finance and Economics’ Academic Committee.

Who should I contact if I want further information?

You can contact any member of our team using the following contact information:

Shubo Liu
Email: liushubo@cufe.edu.cn
Tel: 18801083908

Mengna Lv
Email: 2019211077@email.cufe.edu.cn
Tel: 19800367835

Qiuli Huang

Email: 2018211028@email.cufe.edu.cn
Tel: 17313092964

Please feel free to reach out to us with any questions or concerns you may have regarding the study. We are here to assist you.

Who should I contact if I wish to make a complaint?

Any complaint about the way you have been dealt with during the study or any possible harm you might have suffered will be addressed. Please address your complaint to the person below, who is a senior Business School of Central University of Finance and Economics entirely independent of this study:

Head of Research Governance
Central University of Finance and Economics
39 South College Road, Haidian District, Beijing, China
Email: liuxiaoyuan@cufe.edu.cn
Tel: 86-010-62288081

If you are not satisfied with our response or believe we are processing your personal data in a way that is not lawful you can complain to the Information Commissioner's Office (ICO).

Thank you for taking the time to read this Participant Information Leaflet
