# Supplementary material for: Professional, scholar, or knowledge worker? Identity construction of Chinese management researchers amid the research–practice gap
Source: PLoS One. 2024 Aug 29;19(8):e0306833. doi: 10.1371/journal.pone.0306833 (PMC11361602; doi:10.1371/journal.pone.0306833)
Supplement: S3 File — (PDF) [file pone.0306833.s003.pdf]

中央财经大学 Central University of Finance and Economics | 商学院 Business School

CONSENT FORM

Participant Identification Number for this study:

Title of Project: Institutional Transformations and the Survival Dynamics of Management Scholars in Chinese Business Schools

Name of Researchers: Shobo Liu, Mengna Lv, Qiuli Huang

1. I confirm that I have read and understand the ***Participant Information Leaflet* (version 18/8/2021)** for the above study. I have had the opportunity to consider the information, ask questions and have had these answered satisfactorily. ☐
2. I understand that my participation is voluntary and that I am **free to withdraw at any time** without giving any reason, without my medical, social care, education, or legal rights being affected. ☐
3. I understand that data collected during the study, may be looked at by the research team from Business School of Central University of Finance and Economics, and that no personal identifiable data will be collected or used and all the data collected is for this research project only, encrypted drive will be used. ☐
4. **I consent to audio record and use pseudonymized verbatim quotations.** ☐
5. I consent to being contacted and invited to engage in this research project, and the researchers are free to use the data I provided for their research project data analysis, presentation of research findings, as well as the thesis of this project and publicationss. ☐
6. I am happy for my data to be used in future research. ☐
7. I agree to take part in the above study. ☐

______________________	__________	__________
Name of Participant	Date	Signature
______________________	__________	__________
Name of Person Taking Consent	Date	Signature
