# Supplementary material for: Professional, scholar, or knowledge worker? Identity construction of Chinese management researchers amid the research–practice gap
Source: PLoS One. 2024 Aug 29;19(8):e0306833. doi: 10.1371/journal.pone.0306833 (PMC11361602; doi:10.1371/journal.pone.0306833)
Supplement: S4 File — (DOCX) [file pone.0306833.s004.docx]

# 受访者1

刘书博老师：那行，那我大概先问一些个问题，然后两位同学有一些细节性的问题。我们还是从个人的成长经历和教育经历说起吧。您本科是学新闻专业是在人民大学，对吧？后来去爱大读研究生，还有接着读博士，就转到管理学了。您当时学管理学是具体有一个方向就是人力资源管理，对吗？那当时转专业选择方面有什么原因啊？

受访者： 对，我大三的时候应该是第二学期，去丹麦哥本哈根大学交换，然后跑到的是对方的社会学系。

社会学系当时选了一门课是人力资源管理，然后那个课的老师让我感觉这个学科是我更感兴趣的一个方向，所以当时就决定硕士我想要换一个专业学习。

刘书博老师： 您觉得人力资源这方面有没有一些具体的兴趣点？

受访者：应该说是研究人本身吧。其实新闻学也是感兴趣的，也是研究人本身。我当时其实觉得自己的想法比较天真，就是感觉这个学科是研究员工如何能够更快乐的参与劳动。我当时是以这样的一种比较天真的想法去学这个学科的。

刘书博老师： 其实就是这个学科它的目的是可以帮助组织员工更快乐的去工作，所以您当时看到这一点就被它吸引，还有吗？

受访者： 还有可能是逃避。因为新闻传播学本身要跟人打交道的。我自己是去做过一段时间实习记者，我感觉自己不是那么外向，不是很能胜任新闻传播学的对口专业的这种工作方向。然后当时觉得自己是比较想继续去读研，可能会读博，想去做老师做研究的。然后感觉人力资源管理这个方向实践性更强。而且相对来说对于语言沟通的要求可能会相对来说要低一些。

刘书博老师： 明白。所以当时您对自己的这个性格和兴趣应该也有了解，然后寻找的是一个更适合自己的一个方向。

当时您说是在丹麦一个交流项目，对吗？

受访者： 对，就是一个国际交流项目。应该是人大和丹麦哥本哈根大学之间的那种国际交流，我只去了半年。

刘书博老师： ok，明白。那后来研究生是就当时申请是爱大，也没有想去这个哥本哈根呀，或者去到美国呀

受访者：因为考虑到语言还是英语，丹麦的话毕竟在那块儿生活你不会一点丹麦语比较艰难一点。

刘书博老师： 对，是的，那当时没有考虑美国，他们应该是全球的这个学术霸主。

受访者：可能算是我自己的个人偏见吧，因为我觉得在他们那边的治安不太放心。后来是去过开国际会议的时候去过一次，应该是2019年的时候，就是疫情之前去那个academy of management meeting去过一次波士顿。还好。但是我整体来讲还是对那边我感觉应该算是我个人的偏见

刘书博老师： 对，他们枪支合法嘛，欧洲会好一些

受访者：毕竟还是我是学新闻的嘛，就是社会新闻看的可能也···当然也是输入的会有一些偏激。但是我自己还是很清楚自己是风险回避型的性格，所以比较偏向于英国或者欧洲的环境吧。

刘书博老师： 哦，他们的环境偏向于更安全，是吗？

受访者： 一个是治安，还有就是人文环境。我自己能够感觉到我当时在丹麦的话，我也是比较喜欢欧洲的这个风格。后来我觉得也算是验证了我自己的一些想法。就包括欧洲这边会对定性的还有不同学术观点的研究会更包容，相对更多元。而美国那边会比较集中于定量的研究，然后会我觉得相对来说学术的这种观点比较同一性，就比较趋同。这只是我个人的想法，欧洲那边会更包容更多元一点。

刘书博老师： 所以很早的时候您就了解到有这样的差异

受访者： 其实我觉得就是我的个人感受后来是被验证了，就是读到博士的时候，我觉得是我当时的那个直觉是被验证了

刘书博老师： ok，ok。所以您我觉得还是很敏感吧，很敏锐，就在那个时候就···

受访者： 这是直觉。其实我也不知道为什么、从哪里产生这种感觉。就是我感觉是我可能本科或者是高中的时候可能···就是我也不知道是从阅读的书籍还是从哪边的感受。就是感觉美国更就是那种资本主义的特质更强烈，就是所有的导向都是，包括管理学里面的或者工业心理学，它的导向更多的在于盈利，就是如何使劳动力的生产能力最大化，利润最大化。就有这种很明显的导向。

但是欧洲相对来讲他们还是有一些就是相当于他们应该是左翼运动的影响，或者是这种社会公平的影响在里面。他们会相对关注对劳工的这种权益保障，会有更多的这种研究关注这些东西。因为我自己个人不是一个非常喜欢纯粹的这种所谓自由主义竞争，或者说纯粹的这种社会达尔文主义、优胜劣汰的人，我还是比较关注社会公平这一方面，包括我自己做的研究也是相对来说会关注到这些问题。

刘书博老师： 明白，明白，ok，那就是这种关注啊，或者说对于资本的敏感或者那种社会达尔文主义的敏感，那是新闻学的教育赋予您的这种思考的维度和视角吗？

受访者： 这个感觉是我觉得很难说是新闻学的视角。应该是我高中的时候或者是初中的时候就跟朋友就会聊这些问题，包括教育公平。啊，会关注到一些呃就是那种阶层分化，就是很小的时候就会讨论这些东西。来源我其实不是很确定，我不知道这种可能是天生的一些兴趣点，包括在那个时候其实也没有接触到什么政治经济学。政治经济学看过一点点在本科的时候，但是我觉得我了解的不够深入。就是天生的就是对这种东西会比较敏感，就感觉是不公平的东西，会触动痛点的那种感觉。也是有点情感化的这种表达。

刘书博老师： 那我觉得其实您表现出的这种敏锐性或者是这种视角啊，我觉得对于做学术还是挺关键的。尤其是做人文社科的，那当然做自然科学那个可能这个没必要。其实在管理学里面有这种理性主义与人文主义的碰撞，可能您在做研究的时候也会有这种碰撞的感觉。就是尤其是当这个我们之前探讨说定量和定性研究好像有这种分野非常大的隔阂的时候。其实也有一点这种理性主义和人文关怀，它有相对来说矛盾冲突的地方，但也有融合的地方。

受访者： 嗯

刘书博老师：您是从小在北京长大，对吧？

受访者： 我是在北京出生的，然后在北京长大的，但是祖籍在XX。

刘书博老师： Ok，ok，那能能方便问问您父母是从事什么专业工作吗？

受访者： 我父亲就是大学教师，母亲也是在大学工作，但是在实验室工作，比较偏向于教育辅助岗位，比较偏向行政一些。

刘书博老师： 那您父亲的专业是什么呀？

受访者： 农业类的。

刘书博老师： 农业类。ok，那应该就是自然科学的那一块儿。

受访者：自然科学

刘书博老师： ok，那您的性格会不会比较受到这种学者的影响？

受访者： 会，我觉得这个影响基本上就是为什么我从小的就是当科学家，就是想去做研究。

刘书博老师： ok，ok，因为我觉得您表现出来的精神气质就让我能够大概猜到这个方向，现在也验证了。好，那我就接着再进一步问，就是您到爱丁堡大学读这个研究生，当时对于这个新的专业感觉怎么样？因为他是商学院嘛，跟之前的新闻专业就不一样，能大概谈一下就是到商学院学习的感受吗？

受访者：其实我是一个社交没有那么广的人。但是我觉得在商学院里面有感受到的应该是他们会比较偏爱社交性比较强的人。因为我当时我对商学院的感触在研究生阶段其实不太好，可能是因为我自己的学习经历里面会受到其他国家学生的歧视，所以我当时是一个比较自闭的状态，对商学院的感触没有那么深，反而倒是博士期间感觉会更深一些，博士的时候，感觉就是爱大商学院的这个环境比较适合，是你可以去做一些学术上面的这种探讨。就是你感兴趣的话，你可以去跨学院进行，也给了很多这种资源。包括我当时是旁听了一些政治社会学学院的这种课程，就是好的统计学或者是那种定量定性研究方法都是在那边上课的。然后包括很多这种讲座，基本上你在网上能够查到，我就可以跨学院去学习。而且经常会有这种来自世界各国的知名学者来学校做演讲，就是爱丁堡大学是整个整体包括商学院是很适合，如果前期想要做研究的话是很适合去学习的一个环境。而且我对商学院的好感很大程度来自于我的导师，因为他真的是那种尽力的会创造一切条件帮助我的那种老师。就包括他教学的时候，我在硕士的时候是听他的课，他上课就是很有那种激情，对自己的研究很有激情，算是我的精神楷模吧。我觉得是他感染我，就是包括我当时做出来要读博的这个决定，我是跟他聊过的。我说我想要申请博士，因为我当时硕士是专业第一名，就是论文和课程都是第一名。然后我心想说我觉得我是不是有潜力去读博士？然后我的导师说他欢迎我来申请

刘书博老师： ok，ok。那这个专业第一名还是挺不容易的。因为像咱们留学生其实有一些课他是需要文字表达什么的，所以其实拿第一还是挺挺厉害的，

受访者：因为我是跨专业过来的，相当于完全没有基础，就是零基础开始学习。那硕士那一年我之所以说我对商学院没有什么感触，也因为我没有住在学校宿舍，我是在外面一个人住。还有就是我真的很多时间都花在看教材上，就是英文教材上来什么基础都没有，就是在啃那些教材。所以就是就埋头苦学吧，补这些知识，所以也挺苦的。

刘书博老师： 是的，是的，但这个也是学者必须要有的一种刻苦的一种素质。那您到博士阶段之后这个感觉怎么样？因为这个时候就开始涉及到比如说学科的一些特点，就会就接触到一些范式呀，认知论、方法论。然后就会有不同的研究设计呀，视角呀。那当时您有没有遇到一些觉得挺意外的情况呀。

受访者： 读博期间我觉得印象最深的就是我自己的研究计划几乎是非常严格的执行了。我自己写的博士研究计划的内容，基本上当时最开始就定的是我要做定量和定性研究。然后我导师的建议是让我再做一篇文献综述，就是文献综述也是一篇单独发表出来的文章。对，所以我的博士论文结构就是三篇期刊文章的形式，一篇文献综述，一篇定量文章，一篇定性。

然后我觉得最大的挑战就是自学能力，因为基本上就是以研究问题为导向去学习研究方法。而且我自己本身因为学的是文科，这种数据统计的基础是比较薄弱。所以就是上来我导师的建议就是尽量要做那种多层线性结构方程模型。那时候我就是一脸懵，因为我的老师自己是不会多层线性模型的，就他知道这个方法，但是他是让我自己去学的，并没有办法说去辅导我怎么样。我是旁听了隔壁学院的课，就是那个社会政治学院的统计学课程，然后自己去琢磨，不停的琢磨，然后认识。

然后我当时应该是遇到我的学姐，然后那个学姐是硕士期间是在北京大学就读的。她就是基础非常好，就是在硕士期间国内的这个北大教授，就他的导师已经在手把手的教那个怎么去用统计软件去做多层线性结构方程模型，然后怎么去做收集数据，因为他们北大是有很严很很严格的这种传帮带的。包括他导师是大牛，然后他的导师的朋友是荷兰某所高校的大牛。然后他们就会合作，包括出钱、出资源这种，文章发表很多就是很盛产的这种。呃，然后我当时就说我很震惊，有点羡慕吧，我觉得就是国内有资源，然后国外也有合作的，还是挺羡慕的。

刘书博老师： 那您说这个同学是北大光华的吗？

受访者： 应该是北大光华管理学院的硕士生。

刘书博老师： 明白，明白。英国是这样，英国就是导师没有特别细化的在指导，大概就是一个方向性。

受访者： 而且我比较特殊的一点是，我做的方向跟我导师做的方向并不一样。这一点其实是我如果是我给其他博士生提建议的话，我是不建议这样做的，最好是跟导师的方向一致。我做的是工作生活平衡。但我导师其实他做的就是员工的福利和老龄化。他对（我）这个课题有所涉猎，有所涉猎是指他讲课的时候可能讲过，但是他并不研究这个方向。所以说基本上还是我自学的多，然后我自己去提课题的研究方向、有什么样的理论建设。其实全部都是我在主动进行，他会给我指出一些可行的方向，或者是帮助我提供一些比如说最近他看到有些什么seminar或者是讲统计方法的讲座，他会帮助我去预定或者是告诉我说有这个机会。但是整体的把握，从确定研究题目，到研究方法的执行，再到这个写作基本上是必须得我自己去学很多东西，但是这个也是一个很好的，其实日后是很好的优势，但当时是非常痛苦。

刘书博老师： 嗯，您觉得假设如果是像国内那种完全的手把手教的培养模式，相比于您在博士期间导师的这种培养模式，那现在做一个评价的话，您觉得如果再来一遍您会选择哪一种？

受访者： 我会倾向于二者的结合，结合的点就是这些统计学的一些方法。如果那个课程可以教的更细致的话，就是不一定是手把手教学，但是最起码可以有更好的课程。因为我在英国听的那个课程是非常初级的，实操的时候你发现完全不够用。在统计学方法或者是在这种研究方法上面的这种课程，我会比较倾向于国内的传帮带的方式，甚至是说师兄师姐可以指导师弟师妹或者老师指导指导这个学生的程度。但是在定课题的方向的话，我比较偏向于学生的主动，学生充分发挥主观能动性吧。

刘书博老师： ok

受访者：我自己指导本科生研究项目或者研究论文时，我会比较偏向于先询问学生的兴趣点、感兴趣的知识点和感兴趣的研究对象。然后启发式的，希望是他们自己来选课题，而不是问我有什么课题需要做。因为我觉得是一个需要培养学生去进行研究探索的一个基本的能力。

刘书博老师： 是的。其实咱们国内就还是有点像分包似的，老师有什么然后直接给学生。那欧洲我觉得还是就挺尊重学生的选择的。像我的导师当时也差不多是就是很自由，他总是在问我你要做什么？然后他再帮我具体进一步的做判断，可能都是有这样的不同吧

受访者： 就是自由裁量权是比较大的。但是我觉得尤其是在研究生这个阶段，或许说这种老师指出方向比较重要，可能会比较有帮助。但是我觉得到博士这个阶段，尤其是培养学术型博士的话，培养这种独立研究的能力是非常重要，就是尤其是在确定选题方向，不能永远指望老师给你指方向。这一点非常非常重要。因为我在后来有接触到过学妹吧，相当于就是后面再去上爱丁堡大学去读的这些学生，就是有一些中国学生。然后她就问我选题应该要怎么确定的时候，我觉得我没法回答，因为我不知道他们的人生兴趣点在哪里。我也没有办法去指出方向。特别有意思的是，她问我的导师有没有项目。就是因为国内的话是项目团体制的。我知道有很多硕士和博士的项目都是依托于大的研究团体，可能导师会给你指出非常明确的研究方向。然后学生加以执行，我觉得这是好事。但是相对来说，如果要是也给学生一定的自由裁量权，选择自己确定自己喜欢的课题也很重要。因为这个发现问题的能力我觉得是需要培养的，不是说一直被指定一个题目，他就能自然而然的就知道以后要做什么。

刘书博老师： 对的，是的，是这样。ok，那后面您整一个博士的过程，后面的过程一直到毕业还算顺利吗？有没有遇到一些个挑战呀或者是意外呀？

受访者： 就是在做第二篇文章的时候，有几个现在回想起来是小的打击。第一次是应该是博士第一年的时候，当时上很多课就比较崩溃，但是还要写文献综述，那时候还冬天，人有点儿抑郁了。因为课业量很大，第一年上的博士课业量作业量还有考试量很大。当时我导师还希望我帮他去输入那个问卷儿，就几百分问卷儿一个一个填，那个录入到电脑里面。然后我当时是严睡眠严重不足的情况下，跟我的那个西班牙学姐，我导师当时带了另一个博士生，发生了一些冲突和争执，就是相当于有误解，然后她就跑去我导师面前去告状，说把一切错都归在我身上。然后就是那个时候我导对我产生误解，那是比较崩溃的一个，但是跟学术没有直接关系。后来也慢慢的就是长期了解一下之后，我导师发现不是那么回事儿，不是完全是我的错。

然后还有第一年就开始要投会议。出现了一个很奇怪的事情，那个会议刚开始给我发的邮件是中了。然后第二天，人家主办方说是被黑客黑了，说没有得到这个机会。当时我的情绪就很崩溃，因为当时是科研压力很大。本来就觉得我能够中一个学术会议是对自己的肯定，结果发现告诉你中了又没中这个状态。就比较崩溃了。

因为刚过去去适应博士的环境比较困难。我不知道别的英国的大学制度是不是一样？但是爱丁堡大学课业量都集中在第一年，其实压力有点大。

刘书博老师： 是的，我反正第一年是焦头烂额的。而且好多同学还有一些课不及格。当然还有比如说学哲学方法论的那些很抽象的哲学的东西啊

受访者：我当时是定量和定性都是学了一部分，因为我当时就知道我自己想两边都要做，所以也是挑战的感觉。里面的课程量比较大，然后到第二年的时候情绪比较崩溃的时候，就是做定量研究。我用的是英国的一个相当于是二手数据，是一个基于英国全境的零零后孩子的一个长期追踪调查。因为是二手数据库，不是针对我的研究问题设计的，所以就是要找到有理论意义又有实际意义的变量非常难找。而且最后做出来的结果跟我理论是相反的，就完全没法解释，就很崩溃。因为那种试的话真的是试了几百个模型，几千个模型都有，那就是相当于每天从早到晚几十天都在试那个模型。那个时候是情绪崩溃。我当时跟我导师说我要不然还是放弃吧，就是有点博士都有点不想读、读不下去的状态。那个时候是最崩溃的。

后面就相对顺利了。就再之后就是疫情期间，疫情应该是2020年3月份的时候我决定回来，就是我想休学，想回来。因为我觉得英国那边的环境是不对的状态。然后我导师就非常不理解，因为他是长期在英国，他当时的状态就跟其他的英国人，还有他们的政客和媒体宣传那样，觉得新冠不是个事儿。然后我导师就有点要决裂的状态，他说你回去了我就不辅导你了，你爱毕业不毕业的那个状态。但是我回来之后，过了大概两个月，我导师意识到这真的是英国政府有问题，然后他觉得新冠真的不是什么大号流感那种，然后关系就恢复了。现在我们也还经常是在交流，因为还有研究合作的这种活动在进行。

刘书博老师： ok，ok，那那就是在再进一步，我们这个看毕业之后找工作，那找工作的过程这个经历怎么样。当然您当时也到这个我们中财嘛，但是就像上次聊的，您会感觉到这种研究的视角是不一样的。也就是比如说他们可能更多站在资方像美国的那种叫效率主义至上，或者说如何去提升这个profitability，那可能像您就会关注比较多的是员工的权益啊或者work life balance。那除此之外还有其他的感觉吗？

受访者：我应该有试讲过四个学校。，都是在北京，因为我家在北京，我就觉得我应该先不出这个范围尝试。然后我的感觉就是他们会有几点倾向的。

一个是学术方向可能会比较倾向于用美国的标准进行评定。包括他们提供的期刊列表基本上都比较偏重于美国的期刊和美国的这种定量研究方向的考核。然后第二点就是对于学校，性别方面会非常非常倾向于男老师。还有就是可能会有萝卜坑的倾向，就是有的学校在招聘广告上列出来的很多就很模糊的这种，比如说多个学科方向都可以，就是在筛简历的过程中把我选中了，然后筛简历的人也没有跟我说任何事情。然后等到现场的时候我都已经讲完了，相当于准备了很长很长时间，然后讲完了之后那个现场老师说，我们招的根本不是你这个专业的老师，就是有这种陪跑现象，就是我是不太欣赏这种做法，当然也没有办法。

刘书博老师： ok。那当时您有了解英国那边的job market或者是有没有考虑留在英国

受访者：因为我当时已经回国了，我回去的可能性也不是很大。但是我的导师会其实是比较建议我去回到英国去就职的。因为我当时手上已经有两篇还算比较好的期刊，三星和四星的ABS的，他说如果要是去英国的话是一定能找到还不错的教职。但是在国内的话其实不太认定我那两篇文章，可能JVB还好一点。但是IJMR，因为我当时写的是一篇系统文献综述，在英国那边人家会认为这是一篇不错的文章。在国内我当时收到的评价有说，一个中国老师问跟我说，你这篇文章就是一般文献综述都是大牛级的人才能发的，然后你是怎么发上去。因为IJMR的影响因子非常高，他说你一个博士生怎么能发上去？

刘书博老师： 他真是少见多怪哈。

受访者： 反正我不知道是不是这样子，就是说好像国内确实对文献综述这种东西感觉看不上

刘书博老师： 没有呀。我不知道我同事怎么样。但是我觉得年轻一点的老师都不会觉得文献综述就不是一个正儿八经的那种，还挺难，有时候还我们会觉得文献这个东西review嘛，可能更难弄一些。

受访者： 所以反正面试给我的感觉就是，人家对于文献综述那篇非常看不上。我当时写的是系统文献综述，其实还是有比较严格的可复制性的描述的。但是就是这给我的感觉有很强烈的倾向性。我估计如果我要再说我的定性文章的时候，有一些学校会更觉得不想要这样的方向。因为出成果的时间周期会更长，他们会更倾向于录用能够快速有回报定量的学者

刘书博老师： 是的，是的，是的，我有同感，当时我遇到的是同样的问题。

受访者：这确实是一个挑战，这也可能是为什么我决定当时在博士期间是定量定性都去学都去做的。因为我考虑到我当时比较倾向于回国就业，但是我经常看青椒的相关的新闻，我猜到这种考核压力之下的话，校方会严重的倾向于这种回报见效比较快的定量研究

刘书博老师： 就短期效用最大化的。

受访者： 但是我觉得我自己的理想是做一些中国管理实践的理论化工作，因为太多的都是用沿用西方的东西，西方的东西并不完全是用中国的实践。但是你想做理论化的东西，我觉得定性的研究是少不了的，观察或者体验，或者是访谈这些东西是必要的。

刘书博老师： 对对，是这样。我不知道您现在这个工作进展怎么样，比如说academic work，那其实这种工作和生活的界限是非常不清晰的。或者说我们就认为生活就是工作。很多美国那边培养出来他们就是workholic，而且会非常骄傲于此，非常觉得因为自己是这样,所以我会比你更优秀,或者我更有价值感。

受访者： 工作生活平衡这个方向来讲的话，因为我当时去美国开会的时候，就是随机的跟一些学者坐下来聊天，然后他们知道我研究工作生活平衡。他说美国人根本没有，那是欧洲的东西，在美国只有工作，没有什么生活不生活。就是有些地方是连带薪产假都没有的这个状态，所以根本不要讲。但是在欧洲的情况下的话，对学者，尤其是对女性学者相对来说是比中国或者比美国都是更 友好。

刘书博老师： 而且像高校的师资制度、人事管理制度欧洲跟美国也不一样，在英国有一些学校有tenure，但是我看到多数学校还没有这个，它直接就是permanent contract，是长期雇佣的。

受访者： 我的导师跟我聊到他们的制度，就是也有产出的要求，但是产出不是那种高负荷劳动下才能达到结果。而且对于定性的学者会比较友好，会给你更长的时间。因为有些定性的文章的可能三五年都得不到发表，但是发表到顶刊上就是很惊艳的一个视角、很惊艳的一些学术观点。相对来说英国还有这个欧洲的学校对于这种的包容度是比较高的，就是你可能长期没有那么大量的产出，但是你的质量我是能接受。比如有两三年没有发表很好，没有发表文章的状态是可以的。包括学校一般不会轻易的就说开除你了。就是这种非升即走，没有达到多少多少的产出不会就立马走。它会有一个人会找你谈话，但是不会说硬性的把你开除。一般的话有些老师会主动的去寻找其他的更适合的学校，相对来说是对学者比较好，对这个高校的教师成长是比较有利的。

当然也有人会觉得说这是在养闲人。

但我觉得对于这种有创造性的工作，尤其是想要对这种高质量的研究有追求的话，（闲是很重要的）。要求学者一直不停的大量发顶刊文章，这是一般的学都做不到的，尤其是对于新进的学者，就early career时期的researcher来说这是不现实的，你总需要有一个雕琢的时间，不可能就像那些有名的教授一样，很多学生然后立马每年发几十篇的这种，这个根本就不现实。

刘书博老师： 是的，是的。但在国内的状况就是，我们那种大跃进的心态就是在历史当中出现过，现在在学术领域再一次的能感受到，就学术大跃进嘛，比学赶超之类的。

受访者： 但是我注意到有一些转向，说要以质量取胜。但是这个考核的评定还是很难。还有一些学校已经开始逐渐退出这种国际高校排名，它可能会有一个转变风向的作用。

我是比较希望，国内的管理学教育的发展能够给学者一定的喘息的喘息的机会，或者更应该说是去打磨的机会，包括这种做定性研究，或者做一些需要长期观察的research，你需要一些时间去积累，而不是说每年都要发顶刊，这种相当于是一种快速生产的工业化流程。很多学者就集中去做这种短时间的重复测量，得出来一个什么样的模型。但是从我的角度来说，这样子就会引发研究的同质化倾向。所有人都在做，用同样的方法，因为它是最短时间能见效的研究类型。但西方也有这种问题，欧洲也有这个趋势。但还是美国更甚一些，中国也是这样。

刘书博老师： 反正我现在感觉中国比美国还要严重一些，就美国可能还有一些学术自主性嘛，那我们因为有一些制度特点，导致我们在标准的落地方面特别的一致性，导致从上而下的控制。

受访者： 但是其实还有一个问题就在于国内的博士，包括海归就是供过于求。因为高校现在招聘的这种岗位非常有限，这些培养出来的大量的博士生，他如果在学术界的话其实是有困难。尤其是人文社科类的博士生项目，理工科、工程类的话他还可以转向业界，都有比较好的发展的机会。但是像人文社科类的话可能过饱和了，这也是为什么高校青年教师里面内卷非常严重。

刘书博老师：而且我们人本的这种精神似乎也不是特别强，就感觉我们是一种极端。

受访者： 如果从我的角度来看的话，也不能说中国没有人本精神。我也会看其他领域的，比如说温铁军，那他们是非常有人文情怀，也有这种立足于中国实践的这些理论感触的。但是少。

因为现行的评价体制比较倾向于理工科的那种，很理性，然后很量化，不太有你去自主发展变化的空间。其实这样子是不利于中国去打破西方的这种学术理论的垄断地位。因为你现行的体制之下，你要求的是数量，而非给打磨的空间的。但是质量性的，和这种有突破性的这种研究，你不给他雕琢的时间的话，就相当于不停的在巩固西方在管理学中的这种垄断地位。因为你想有突破性的东西的话，一定是需要时间积累，需要包容性。年轻学者的这种新的思维，新的想法的积累，也是需要时间的。

而且我觉得现在做理论构建的或者是诠释主义的，它是可以推动这种理论视角的产生。但是你像管理学，尤其是国内培养的博士，大家这个理论构建能力没有得到培养。大家一上来就是“传帮带“，非常紧密的在培养他的研究方法和使用工具的能力，但这种理论感就差的比较多。

我觉得我很同意的就是刘老师说的，就是有一种在做工具人的感觉。包括您之前说的贴标签生产的那种感觉，

刘书博老师： 就是学术贴牌生产。

受访者： 就是我们在做他们的代工厂的那种感觉，这是非常明显的有一种倾向。

我刚才说的那种手把手教这个模型的构造，就是怎么去用这种统计方法，但是你没有传达一种精神，就是可能老师也没有办法去传达那种精神——就是我们希望去做一些立足于我们自己本国实践的一些新的理论。就是说可以不是那种突破性，但是最起码是告诉西方人说，中国有些东西文化上或者是实践上跟你们有什么不同，但是它是合理的，就是这样一种理论性质的探讨也好，或者挑战也好，我国管理学还是缺乏这方面的东西。

更直白的话，我的感觉就是会快速的教会学生做论文，就是我告诉你怎么做，用什么软件，做什么模型，然后哪几个变量可以相互影响，你把这个全部生成了，然后就能够迅速的投出这个稿子，然后把英文写的好一点，然后我们就去发什么样的期刊。就是这种工业化流程的东西，非常非常直白的呈现给了学生。但是这个不能说不是好的事情，不能说不是一个好的教学方法，但是它是工具化的，这种教学是缺少一定的灵魂的感觉。

跟学生进行灵魂式的理论的探讨，有助于避免话语权完全被西方垄断，我觉得是一个追求的目标。因为世界上不只有美国，不只有欧洲，还有包括中国、印度呀，他们有很多不同的管理实践，需要我们去重视。

刘书博老师： 是的，是的，我特别赞同，是这样。所以我们现在从上到下倡导把研究做在中国大地上，要构建中国的理论。其实说的应该就是我们现在这个弊病，就学术太工具化了，没有思想。

刘书博老师：那范老师我大概就问到这里，那后面还有一些问题交给咱们同学们。你们有问题就可以简短的问一下。

吕梦娜： 好的，刘老师，我先来吧。

因为前面刘老师跟您聊的话，我对您这个读博的经历大概有一些了解，然后同时也会交流到关于这个学术研究方面的。那咱们都是在管理学这个大的领域研究嘛，然后您应该也会听到或者了解到我们管理学其实面临一些合法性的争议。我主要是想看看您对这些争议是怎么理解和看待的，然后包括对您这个实际的学术研究有什么影响。

第一个国际上最热的一个讨论就是管理学的实践性和理论性，或者说叫这个科学性之间关系。有的人可能会追求“为了学术而学术”那种追求真理式的那种研究。但是有的人会觉得这个管理学毕竟是一门应用学科嘛，就要追求这个实践的相关性。然后我想知道您对这两者的关系有什么样的看法，包括就是您在研究过程当中是怎么去做的？

受访者： 我其实也注意到有探讨说管理学有这种理论和实践脱节的问题。尤其是在我看到的西方的英文期刊的文章里面，尤其是在欧美国家人做的很多理论的概念，我觉得有点过于学术，或者是为了构建新的理论而强行去编制一个新的词汇，有这种感觉，就是过于理论而与实践有所脱节。我反而是觉得西方人的思维有点过于抽象，会对学术有过于理论化的一种发展。

而管理学本身是一个立足于实践的学科。我自己的感觉是应该要依托于实际实践来做研究。就是所做的研究应该是对实践有一定的指导意义，不能说完全能够适用于所有的范围，但是最起码在一定的范畴内应该是对实践有意义的，而不是说为了学术、为了理论而去强行的去创立一些新的概念、新的词汇，我是比较反对这样做的。

然后还有一点，我自己觉得中国人的思维反而没有那么抽象化。我自己的了解的话是中国人会比较实际、会比较不容易提取出来这种高度概括的理论化的思想或者是概念。但实际上中国有很多比较特殊的独特的现象，或者是跟西方国家的管理实践是不一样的这种现象，那么我们其实是应该依托于实践去做一些理论化的阐述，理论化的概括。

然后我个人的话是有这种想法，我现在是在研究工作生活平衡，所以我目前在做新中国的社会发展与工作生活对于中国人的工作生活平衡有怎样的影响。这个其实是有一些偏理论化的这种提取的。就是依托于中国实践，而不是完全采用西方已有的这些概念和理论。

吕梦娜： 了解。那那您方便就是稍微具体一下说，您是怎么开展这个在新中国情境下的这个工作生活平衡的这个研究吗？

受访者： 可能没有办法特别具体，因为这篇文章还没发出去投稿。但是也是使用访谈的方法采访，从二十多岁到八十多岁的学者、科学家，或者是做研究的研究人员和这个高校的老师。然后结合中国历史，就是实际上就是一些历史上的数据，还有一些被访者的个人经历，去看中国的社会变迁，对于中国人的这种工作和生活经历的影响。这个点出发是考虑到什么？因为工作生活平衡其实是美国最先提出来的。它是在1960年代提出来的，我自己感觉它是一个工业化完成以后，西方国家转向员工福利，对于这种工作生活平衡的追求之后提出来的一个概念。而中国其实是跟西方历史发展阶段是不一样的。中国是属于从极度贫穷落后的一个农业国家快速的发展，然后转向这种工业化的，现在应该是中等收入国家，这个经济社会历史变迁是有巨大的这种变化。所以我觉得本身这种宏观的背景对于人们的工作生活平衡是有巨大的影响的。而西方的目前的工作生活平衡的研究大多都着眼于企业层面或者是个人层面的这种工作生活经历，而对于社会变迁有很少有考量。这是中国可以提供的特殊的视角，包括历史也包括社会这种宏大叙事的问题，这是一个新的东西。

吕梦娜： 对，我大概了解了。那我能够看得出来，你确实是在类似于扎根实践的那种嘛，因为要通过访谈，就是深入的交流。然后同时你也希望提出自己的理论，就像你说的要做基于中国管理实践的一个理论化研究嘛。那在做这件事情的过程中，你有没有感觉到一种矛盾： 就比如说你要追求理论性和实践性的这个这个不太好平衡？或者说为了追求实现一些理论，可能要损失一些实践性或者怎么着？你有遇到过这种这个矛盾吗？

受访者：因为我这篇文章其实是比较偏理论的，我就是把实践的过程提取成理论，所以我对这个矛盾倒没有。但是写作本身会有顾此失彼的感觉，因为它有篇幅限制。就是说把实践抽象成理论的过程中，具体是采用哪个人的经历，不采用哪个人的经历，有这种挣扎。但是你说的这个损失理论或者是理论和实践的平衡，我倒没有这方面的问题。但是如果还是说发英文期刊，遇到主要的问题可能会是文化差异，你怎么去把中国的这种实践经验，用西方人能够理解的这种思维方式表达出来，就能不能让别人也理解你所说的这个问题、你的这种思路，这个是比较困难的。

吕梦娜： 了解，您这篇文章到时候是在英文期刊上发表的，对吧？

受访者： 还不知道，但是目标是英文期刊。

吕梦娜： 了解。刚刚我们提到这个就是理论与实践脱节的这个问题嘛，也算是一个合法性的一个问题。然后另外一个就是关于这个研究的本土化与国际化的问题。因为现在国内有一些学者说要做类似于中国式管理或者提出叫东方管理学，我不知道你有没有了解，就是好像一方面想要说我们要跟西方保持一定的距离然后做自己的研究但是另外研究者还是在跟着西方的这个路子。您对这个国际化和本土化这个有什么看法吗？

受访者： 我觉得都不矛盾。就是学术其实是一个有一定自由的工作。你既可以用西方的一些理论去解释本国遇到的一些社会现象或者一些问题，但是你又可以依托于本国的这种实践经验或者遇到的问题来构建新的理论。

我觉得具体的话就是合不合适的问题。就是你用西方的理论，有的时候中国的现象和其他国家现象并没有什么本质上的不同，就比如说社会阶层会分化，在任何一个社会里面都会遇到这个问题，就是说本质上它都是这个问题，那么其他国家的一些经验和理论是可以用于中国的这种现象和问题的解释和预测，那么这就不成为问题。但是如果要是西方的一些理论明显的不合理，就是用来解释这个问题不合理的话，那么它就成为一个我们需要做一些本土化理论研究的一个着手点，一个突破口。

同时我觉得国际化不能等同于采用西方的理论，也可以是中国理论走向世界，就是包括中国的一些社会实践可能会对其他国家产生影响。就比如说我记得之前有日本的这种管理模式，汽车制造业的管理模式其实也对美国呀，欧洲的一些管理学的这种理论产生了巨大影响。就是说中国的一些管理实践如果要是在比较好的情况下也是可以这样就是走向世界的。我是这么这么理解这个问题。

吕梦娜： 所以我听起来，这两个其实并不是什么特别矛盾的事情，就是得看具体的研究问题，到底是不是说就是国际都通用的样子，还是说就是我们本土的情况？可以这么理解吗？

受访者： 对，我觉得就是所谓的普适性和情境性。

这个理论有没有普世价值？或者说是这个理论可以在不同的情境下有什么样的这种变化？这个其实是更本质的问题。我觉得本土化和国际化也可以演变为这个问题的讨论，对于是普世的还是在一定情况下有所变化的讨论。

吕梦娜： 就结合您刚刚提到那个工作生活平衡那个研究，我听起来好像就是这个概念虽然说是西方提起来的，可能全世界会面临一些这样的问题，但是你也是扎根在我们中国的这个实践我们的情境之下。然后做的一个本土化的研究，但同时又想要把这个研究展示给国际上的学者，我们一起来探讨，就是一起来看，好像是这样一个感觉，是吧？

受访者：嗯嗯。

吕梦娜：谢谢你。然后还有一个问题，就是前面提到这个范式的这个争议嘛，我们管理学其实也会有一些。包括您可能也遇到过，可能这个定量研究的范式会一定程度上歧视定性范式。然后您做了一个很好的结合，就是你把两个范式都有学习。那你有没有在听说过其他的同事也好，或者学者也好，他们是怎么来处理这个矛盾的？

受访者：我其实现在就比较纠结的在于很多学者会相互排斥，就是好像形成了两大阵营，相互排斥。我有两个导师，一个是定量学者，基本上只做定量，然后另一个定性的基本只做定性。

我两边都做，然后向他们解释的时候会有困难，我向定性的学者去解释我定量做了什么，他基本上是没有办法理解。然后像这样的学者，因为我的导师相对来说接受度比较高，但是他也需要去从头去学习一些概念和一些想法，他其实对定性的理解会有一定的困难，因为他们长期都是只做一方面的研究。包括他们跟我沟通的时候也会说，相当于他们的academic community，他们这种学术学术圈子的人中间会有天堑或者鸿沟的这种状况。就是我只做这一边，我不会去做另一边，甚至有些人会出现我不屑于去做另一边的，我就是不去看那个研究的，都有一定的这种倾向性，那个是比较极端的状态。所以我觉得客观上是存在隔阂的，但不能说是完全没有可结合性，应该还是有一定的可结合性。

吕梦娜： 那您就是博士毕业论文之后，就现在做的研究是怎么来协调这个范式的？

受访者： 其实我想找到很好的结合点，从纯粹的学术发表的角度来说，我还没有能够完整的在一篇文章中把这种定位进行完美结合，可能我在系统文献综述里面有一定的结合。但是当时的那个期刊编辑会比较倾向于让我更偏重定性的描述，而不是定量的这种反馈。那么我自己比较倾向于，比如说如果我自己做那种社科项目，那么就是这个项目里面拆分一些细节，有一些部分可以用定量的这种方式去研究这个问题。然后有一部分可以使用定性的方法去研究同一个问题。就是从不同的角度，但是针对到学术发表的这种环节的话，其实难。最大的难度在于有两大阵营的这种学者，他们彼此之间都很难去理解另一方这种研究方法的意义，还有他的这种思维过程，然后他们会对这种混合研究方法的东西提出自己的质疑，这是最大的难点。

吕梦娜： 是的。那这样是不是好像在一篇文章当中好像不太应该呈现这两个范式，因为总有人喜欢就有人不喜欢似的。

受访者： 我自己的感觉是其实不应该这样，应该是灵活的依托问题，一切都应该是以问题为导向。包括我自己的理念，也是我遇到的这个问题我想去解决。如果需要研究学习新方法，或者是需要采取不同的方法，那我应该就是以问题为导向去选择我的研究方法，而不是说单纯的以我个人的偏好，我就只想做定性或者只想做定量，我自己是不太赞同这种截然分开的。但是在实际上的执行过程中确实是考虑客观情况。你就是去请reviewer都有困难，他们一般能请到的都是只做定量或者只做定性的学者多，这是一个现象。

吕梦娜： 了解。其实我觉得在你身上学到很多东西，我们现在所所说的矛盾，其实它并不一定是矛盾。像这个定定性和定量范式吧，本身就应该是通过研究问题去界定你用什么方法的。但是现在争执，争执多了之后就变成了好像为了范式之争而争议似的，就有这种感觉。

受访者： 就是有一种互相看彼此不顺眼的这种感觉，有点这种对抗性的。我其实从读博开始就感受到这种对抗性，但是我其实是不理解，因为我当时就是觉得应该一切以问题为导向，所以我这个想法其实是一个异类，就是如果在当时那个环境下可能是一个比较异类的想法。

吕梦娜： 那个时候跟你有这样的想法人还是少数，是吗？

受访者： 不太多。但是老一辈的就是我们的导师，他们很多都是只选择了一边去做，但是他们在带学生的时候是鼓励大家都做的。就是我们新一代的可能培养出来的这些博士生，一般都做。所以可能在长期的话会有变化。

吕梦娜： 您之前有考虑过为什么会出现这种范式之争吗？

受访者： 这个好像是跟他西方的这种实证主义的发展，因为我也听过一些研究方法，或者说这种哲学基础的这种课程，他们是有一个发展过程的。就是跟西方的学术史有关系，就是最开始是实证主义的，大家都严格的做实验，有点像这种生物学、物理学那种最开始流行这样的这个东西叫科学，然后后面的慢慢的这种人本主义的兴起，还有各种各样的这种社会因素，导致人们会转向一些观察法，体验，就是那种解解释主义、建构主义。

刘书博老师： 上个世纪60年代有一个叫linguistic turn（语言学）的转向。

受访者：就是大概会转向一些更偏向定性的学术哲学基础。

刘书博老师： 其实在欧洲60年代之前也像美国那种非常实证的非常量化自然科学，但是那个转向之后欧洲就跟美国不一样。

受访者： 那这也是我偏爱欧洲的一个原因吧，他们更多元一些，更包容一些。这个的根源还是在于历史，随着历史发展我觉得慢慢的可能会有新的变化。

就像我说的，欧洲这边老一辈的学者会推荐新的博士生两边都去学习一下。但是他们不是说强迫你去做这件事，有跟我同期的博士生也还是选择只做定性或者只做定量。但是我是接受度比较高，我两边我都学，而且我觉得我确实受益。因为我能感觉到定量和定性有不同的思维模式，但是不同的思维模式其实有很精彩的碰撞。你可以看一个研究问题的学术问题的这个角度更多了，而且你的想法思维就是会有两者结合，会有更精彩的一些发现，我是这么想的。

吕梦娜： 了解，了解。我们前面说到，我比较关心管理学合法性的一些问题，包括这个理论与实践脱节呀，包括这个本土化与国际化。还有刚刚说这个范式之争。那您你自己的体验当中还有其他的吗？

受访者：我觉得我没有什么需要补充的了。

吕梦娜：谢谢范老师。我感觉你的就是给我提供了一个不同的想法。就之前因为我看了一些文献嘛，就对这些问题可能有些提一个提炼出来。但是我感觉大家好像都是那种要么在这边要么站这边，但是你给我的感觉就是，其实他们并不是矛盾对抗冲突的，所以确实给了我新的启发。谢谢范老师，我今天的问题就先到这里。

黄秋莉： 好的。前面老师们和同学们都聊的比较全了。然后我想请问一下范老师，就是您现在工作和您的预期是不是一致呢？

受访者： 因为我刚入职，现在还属于没有把自己的身份从博士转换为一个独立研究者的这种感觉，所以我可能说的只能是代表我当下的心境。如果要是说理想和现实的差别话，就是在博士这个阶段的话，我是基本上实现了我的最初的计划。就包括怎么去做一个系统文献综述，定量和定性的研究几乎是按照我的计划严格执行了。在这个方面是没有太大的差值或者出入的。

 但是如果要说我入职之后，这种现实和理想的这种碰撞或冲突的确是有的。包括我之前跟刘老师交流提到的这种考核压力。我想做的一些研究可能会偏向定性或者是偏向需要一段时间去打磨或者需要一些积累才能去做的研究，可能不适合我们学校的这种考研考核的这种要求，因为它需要时间，它不能快速发表，这个是最我最大的顾虑。

然后包括我跟我导师进行探讨，我的英国导师就跟我说，希望我能够在完成考核要求的情况下，尽量多做一些自己喜欢的工作。就是额外去做一些自己喜欢的事情，这个东西就是你必须得妥协的，对我来说这也是一个解决的方案。

黄秋莉： 所以最大的一个就是考核压力方面的一个问题。

黄秋莉： 据您的观察，您身边有没有一些比较有意思的特立独行的学者？

受访者： 因为我现在是4月份刚入职，而且我们这学期没有太多的跟那个老师接触……

黄秋莉： 您对自己未来的一个职业生涯有什么样的一个期待？就是你希望成为一个什么样的一个学者呢？

受访者： 我希望再去做一些自己感兴趣也有价值的，就至少自己认为是有价值的。不是摆烂的研究，不是说只为了完成考核要求而去发表文章。

但是可能会迫于现实压力会有一些不那么精彩的文章，但我尽量会想去做一些自己认为有价值、有意义的文章。

然后同时我希望是保持国际交流，能够将中国的管理实践向外也进行一些传递和交流。包括与其他非西方学者的交流，我觉得也是必要的。

然后除此以外希望自己能在教学上面也能够做的比较好，学生能够真的学到点什么。

黄秋莉： 谢谢范老师，希望有机会能跟你进一步的交流。

刘书博老师： 范老师，那首经贸的考核要求怎么样？上次咱们聊也是非升即走了，是吧？

受访者： 对，六年的非升即走制度，具体的我还不是很确定，就是不知道他们三年会不会刷人。目前来看的话，六年内完成考核要求的可能性还是比较大的，但不知道中间会不会有没有改变。

刘书博老师： 什么要求呀？是UT DALLAS吗？

受访者： 两篇B2以上的文章，应该是ABS的3星期刊。如果要是能够发一篇3星或者A的期刊的话，可能就已经能够完成文章发表本身的工作。然后另外一个相对更难达成的是有国家级或者是省市级的这个项目。

刘书博老师： 对，这个就有些运气成分。

受访者： 对教学的评分也有要求，就是学生的评价不能太低，要求85分甚至90分以上，但是这个可能是有一定的这种回旋余地的。这个可能是跟其他的学校不太一样的地方，就是对教学的这个评分的要求。这个会跟我以前看到的一些211的学校是不太一样的，他们会更多的依托于项目和文章发表的质量来进行考核。

刘书博老师： 我觉得现在这个环境也可能是整体改变了吧。因为像我们学校在评职称的时候也会强调这个要高于多少分，之前是没有的。

受访者： 这方面可能对学生会比较有利的。

刘书博老师： 好，那我们这个今天就问到这里，也一个半小时了，挺长时间，特别感谢范老师，有机会我们线下交流。比如没有疫情的话，我们找个时间聚一聚，然后再详细的沟通一下。回头我们把文字稿发给您。好，那先这样。

# 受访者2

刘书博老师： 好的，那于老师那我先问啊，两位同学就不做自我介绍了，等一下你们在问问题的时候可以介绍一下自己。那我的第一个问题就是我看您之前学习的这个专业好像是数学，那后来是到心理学，然后也不是一直在大学的象牙塔的环境，可能也有工作过。所以我就想了解一下，您当时是什么时间进入学术志业的。就为什么选择读博士，然后又选择进到高校？

受访者： 呃，怎么说呢？因为好多人走的路其实最后都是误打误撞进来的，对吧？你比如说我原来学的数学，那从小是有数学家的梦想，但是后来走着走着这个路走不通了，那只能走其他的路，然后就走到现在这个样子。

我原来是青岛师专毕业的，我在上大学那时候呢是90年。那两年在师专学数学也是稀里糊涂。当时呢我们学校是青岛下面一个县级市吧，莱西。因为当时莱西相对来说比较不发达的一个县吧，那我们一些老师特别紧张啊，我们当时是青岛教育最落后的一个市。

尽管我当时92年师专毕业，但还是分到了莱西三中（我们当时还是分配啊）那个莱西三中也是我的高中母校。就回到原来学校去了。当然我当时的大学同学，其实很多人都到的是初中，就是我们莱西的几个同学到了高中。那在高中的时候，当然我个人可能还是年轻的时候吧，我们说总是向往自由。然后就跟学校领导啊经常互相之间看不顺眼，他也看我不顺眼，那我也看他不顺眼，就是总是要追求自己的自由嘛。但是因为我们莱西三中就是在一个乡镇上相对来说比较闭塞，然后这个学校的领导呢也比较土的管理风格。那就是这个矛盾越来越深。大概工作了两三年之后，我就想哎，怎么能离开莱西三中啊？怎么离开这个地方？所以我就准备考研。

但是考研选择学什么专业呢？因为其实内心还一直对数学非常有热情的，但是后来很现实的考虑是，如果说考数学呢这个路其实会更难走。那另外一条道路就是我既然当老师，那应该好好研究一下教育的规律啊。那教育呢我们面对的是学生，那就学生的心理很重要。我们就一直有个观念叫拾草打兔子——两不耽误。那既然当老师那我就考研考心理学吧，就是考不上也对我的这个工作可能也有帮助，对吧？就开始准备考心理学的研究生。

因为我师专毕业嘛，英语是其实很菜的，那个是弱项，然后专业课啊，心理学专业课也没有啊。在大学只学过一个叫什么很基础很基础的心理学。就是作为老师来说可能基本都要学的一门课就是心理学，然后其他的专业课基本就没接触过，但都要自学。那这个过程当中也是有很多朋友给我帮忙，就比如说联系山东师范大学有心理学专业的同学帮我借教材呀、借笔记啊等等。然后就准备开始考研道路，因为也没有说我一定要考上啊，毕竟还是在体制内工作嘛。所以也是每年没有认真准备，就是到考试前那一个月就说我要去准备考研了，所以连续考了四年。第四年终于考上了，读了这个山东师范大学心理学研究生，我们当时三年啊。

其实我在青岛师专的时候，当时对数学其实没什么太大兴趣。但还是看了大量的书，比如说我们那时候90年代初其实书还是比较少，还是读了大量的小说呀，或者说一些哲学的书啊都去读，读了大量的书。那然后到了山东师范大学读研究生之后呢，其实对一些学术上的一些观点呀，或者说这样一些思想还是非常喜欢的。所以说也是不停地去读这样一些相关的书。

研二的时候其实还是有过出国的这样一个准备，也去考了托福，然后好像当时发现成绩达到那个所谓的及格，就可以出去的。但后来到了研三的时候，就说哎呀先考博吧，因为我们当时其实考博士也是挺难的，就是在我们之前，山东师范大学心理学专业很少有学生考上博士，就是所谓的大家在私下里一说谁考上博士，那就是导师帮他联系了博士生导师。当时我的导师也不出名，所以说那我就我自己去考呗。我就自己跟北京的几个导师写email，然后跟他们联系，然后约来聊一聊，对吧？专门儿还跑了北京，见了三个导师，北大的，还有北师大的，还有中科院心理所的。

这个过程当中其实好像是没有刻意的安排，就是到了这个点儿好像就说大家都这样走，那我也走走试试呗。即使在研究生毕业的时候也是找过工作，当时也找了两个学校，一个是在北京联合大学，另外一个是山东经济学院。但是后来因为3月份考博了，这两个都不了了之了。

然后很幸运考博还考上了，到了中科院心理所开始读博士。那读博士的时候呢其实自己学术的基础还是挺弱的，也没有说我要去做什么。其实一直没有沉下心来去做一些事情。而且那时候我们感觉到我们的日子还是蛮快活的，每天下午都打打球啊，或者说出去聚会啊。因为我们当时心理所的这个体制跟其他高校体制不太一样嘛，我们其实那时候也没什么竞争，就是人也不多。我们那一级一共十四五个博士吧。然后在论文选题的时候，因为我导师他当时一直做复杂系统中的人的绩效，就是安全问题，工业组织当中的安全。然后那我就自然选择这样一个领域啊，其实原来也是对文化蛮感兴趣的，后来就说做安全文化吧。就开始做相关的安全文化的一个研究。其实做安全文化的研究还是蛮艰难的一个事情。因为像当时的文献，一个是我们检索不太好检索；另外一个就是安全文化的研究呢，大多数欧洲人在做这个东西，他的研究范式跟我们还不太一样；另外一个是美国人做的很少，国内几乎就没人做嘛。

然后我们就开始做这样一个领域，但是做了之后，就后来去齐鲁石化那儿，因为我当时做的叫参与观察，那就联系了一家单位，齐鲁石化。大概待了半年时间，在那儿还找了他们职工宿舍。然后在那儿待了半年的时间去做参与观察。其实当年我们那个时候研究方法特别不规范。一个是量化研究还是刚刚开始，大家都在自己摸索，然后质化研究很少人做。那个主要是靠自己去探索嘛，然后那时候中文文献几乎就没有，英文文献这对我来说还是蛮吃力的，就是因为它还是欧洲的人做的多，然后反正就在那儿也闲着嘛，就是参与观察，也不知道去观察啥，反正就在那儿待着呗。

之后自己的工作就是在那儿跟别人去聊呀，去看呀。那时候其实也没有现在这样一些工具。比如说我去录像、我去拍个照片啊等等都没有。那时候带的是访谈的工具，就是小录音机，大概做了六十多个人的访谈，然后再做了半年的观察。素材收集回来之后那就开始做了。

在开始写论文这个过程当中其实一直也没头绪，所以我做到6月份才做完啊，那这个过程当中，我也没有说我对学术有多么热爱，就是说那时候先做完论文，然后也对未来没什么规划。包括在04年春节的时候找工作也没有认真去找。找工作时在想是不是要去企业招博士的岗位？因为当时确实还是想去企业，就去接触一下实践吧。然后再联系了几家学校，当时也有中财啊，就是大概到了四、五月份的时候，中财让我来试讲，然后就说基本差不多还可以来试一下，那时候其实我博士论文还没做完呢，能不能毕业还不确定。然后那时候其实也没什么太大压力啊，就是也没感觉压力。到了6月份的时候，我们心理所的研究生部一个老师跟我们导师说，“你今年博士要不要毕业？你看你好几个学生都不毕业了”。因为我们当时毕业要求发英文文章。就是后来说英文文章发表不了也没关系，有投稿就行，但是国内要求四篇CSSCI。因为我也是工作比较拖拉，一直拖懒。一年级、二年级没发文章。到了三年级下半学期的时候才发了。就是发了四篇吧，数量凑够了中文的要求。但是还有英文的，直到后来研究生部的那个老师说，“诶，你要抓紧时间去完成这个英文的文章”。我想那先凑吧，先弄出去吧。所以其实当时也没有说给自己个清晰的规划，然后后来那个过程当中也应该说还是相对来说比较顺利吧。这个顺利的前提是当时环境比较宽松。

然后就开始中财当老师的这个经历啊，早期其实青椒的生活还是蛮辛苦的，就是这个生活的压力呀，工作上的压力啊，学术上的压力呀等等，而且我当时做的叫安全管理的这样一个研究，在商学院这个环境体系下其实是很不契合的。这个过程当中，一方面要上课，一方面还要做自己的研究。而且做的是工业企业里面的安全管理。尤其比如说我们当时做的高风险企业嘛，就说石油石化煤矿等等这样一些企业的。就是在06、07年的时候，我还去过煤矿，又去齐鲁石化待过差不多有半年的时间。

但后来还是觉得我的研究的主题跟商业的这个整体的方向不太一致。大概在2010年左右的时候就想是不是要调一下、放弃原来的那个领域。所以在2011年的时候、第二个国家自科申请下来之后帮助我能够完成职称，那我就不做了。就开始另外的方向，包括现在做的价值观呀、领导力呀，其实这些方向呢跟原来方向也差不多，只是换了个场域去做，但是主题还是在原来的主题——文化和领导。然后走着走着的过程当中，还是越来越喜欢。

其实你看我青岛师专毕业后去当老师，然后当时因为要脱离，其实当时也不是说脱离教师这个行业，而是说说要离开莱西三中。然后就考研最终离开了莱西三中。但是最后找工作的时候还是在教师这个职业里边儿找，最后读了博士之后还是当了老师。其实冥冥之中也是这样一个道路吧，然后呢后来就是越来越喜欢教师这个职业。而且啊我自己观点就是，你看，有人给你发钱，让你做自己喜欢做的事情，让你在课堂上言论自由。所以我觉得这应该是天底下最好的一个职业了。因为最重要的是，人有表达的欲望，这是每个人都有这样一个需要。所以你看，有人满足你的生理需要，然后同时呢让你衣食无忧，同时还满足你的一个表达欲望和需要。我觉得这不就是最好的一个职业嘛？

所以啊走到现在我就是对当教师觉得还是蛮庆幸，而且现在是大学老师，还可以做自己的研究满足自己的兴趣。大概是这样。

刘书博老师： 好的，于老师。这也是我第一次听您这么详细地回顾自己的求学道路，包括职业兴趣的形成过程。我这边挺清楚了，我可能还有一些补充的问题，比如说您当年读博士的一个学术环境，现在您觉得还是那样子吗？因为现在您也在管理咱们学院的博士工作，那您能不能谈一下就是现在对于学术人才的培养是不是有一些变化？

受访者：相对来说我们那时候博士还是少，然后整个国家的培养理念也是没有什么体系化的培养模式。这个体系化的培养模式就是许多东西成熟之后就开始体系化了。

我们中科院心理所应该是90年前后才开始招心理学的博士。许多时候还是一种叫手工作坊式的培养模式，也不是手工作坊式的培养模式，就不是一个体系化的、模式化的培养。然后就是老师只是说给你一个方向那你自己去弄吧，至于说怎么做、怎么来完成他就不管，他只管成果，有问题也可以去讨论。

然后我们心理所毕竟是科研机构嘛，就是我们都是有组会的，每周开会，然后在开会的时候就有同学主持，每个人有主题报告，然后大家一起来讨论，就这样一种方式。一般来说都是在一个屋子里边儿，人多的时候会分两个屋子。但是也是紧挨着，大家都是串来串去，就是相对来说上下级之间、同事之间关系比较密切，就是联系比较密切。因为大家一直在一起学习呀，一起开会，还有包括生活都是在一个楼上。这时候大家有更多的机会聊学术的东西，也会聊生活，就是大家无话不聊嘛。

然后我自己是这样写文章，就是说写了之后给老师看一看，然后老师给你改一改然后就投出去，是这样一个模式。其实那时候还比较宽松，但是后来导师的经验越来越多，这个模式越来越成熟。那现在我们这个博士的训练就是越来越程序化的、程式化的一种训练模式。当然也是因为现在博士扩招啊，这样博士之间的竞争就会越来越大，然后我们现在不是在说内卷，对吧？就是确实是这样，青椒的主要原因就在于这个内卷啊，就是如果没内卷的话，大家其实走上这个道路其实还是因为热爱学术。但后来为了发表的压力，为了生活工作的压力，那最后 就是追求一些短平快，或者说程式化的一些东西，对吧？这也没办法。

现在我们来说这个博士培养的目标，可能每个人也在调整啊。比如说我们现在许多人读博士，其实也不一定就是为了学术的吧，很多就是为了那个博士帽。像很多北大的博士去找街道办事处这样一个工作。其实啊你可以说这个浪费人才，但是你也不能说这些浪费人才，人家个人所爱。就是说对个别博士来说，一个博士帽满足自己曾经的一个梦想，就是说就业还是从现实的角度去考虑。

我们当年博士毕业其实要求挺高的啊，我们有四篇CSSCI，或者一篇英文的发表。如果说没有英文的SCI的发表，那你应该有四篇CSSCI再加一篇英文的投稿，完成投稿就可以。这个有点还蛮奇葩的。但是我也知道现在这个心理所那边儿好像也在降标准。因为不需要用这个标准去卡学生，他有就业的压力，本身他就要发文章，所以就导致从一定的条件来说，从管理机构的角度来说，他在降标准。但对于学生来说，他要拼命的发文章。

像我们我读博士的时候，其实国自科等等非常少的，大家更多的是跟着导师在某些领域去做。那现在可能许多博士都在做的是导师的课题，然后跟着导师去做，这个确实是成体系化的。

刘书博老师：明白，明白。那于老师那您在读博士的时候，是有田野调查人类学的这种，那您当时那种环境可能也没有特别体系化，或者有很多的这种标准的教科书式的东西可以系统的学习，那这个过程您是觉得怎么样？是最终也是有所发现、有所成果，还是说如果重新来一遍的话，会有一些不同的做法和思路。

受访者：如果能够重新来过肯定也要重新来过。那就是一些零碎的、片段化的记录，当然那时候访谈还是可以做。后来因为时间的问题，当然也因为知识产权的问题啊，其实博士论文的很多东西后来都没去弄，因为中科院心理所那时候他要求就是你博士毕业之后，你的论文发表都要署名中科院心理所，不能署你现在单位。

所以因为这样一些知识产权的要求，那个博士论文做完了也就放在那儿了。但是实际上当时做的访谈还是蛮规范的。访谈提纲、访谈转录等也是花了好多钱。之后，我们这个访谈文本的分析。之后还是有发表的东西。因为当时做了五六十个访谈，访谈每个人差不多一个小时左右，但后来就没有动力去做这个事情（发表）了。

然后参与观察呢，其实没系统的参与观察的方法，就是那时候边看书边去琢磨怎么来做这个参与观察。我们就把现场观察的一些行为片段啊，就是现象嘛，一些典型的现象，给整理出来。比如说今天看到什么样的一个现象，比如说有意思的现象，尤其是不符合自己预期的，或者说跟管理制度不一样的地方等等。那就把它给整理下来，然后形成这样一些情景再加行为吧。我记得好像是这样一个就一个情景再加行为的一些片段，大概最后会整理出来。就是当时用了一些标准，不过什么样的标准我都忘了。然后大概最后汇总了一百多个情景，然后对这些情景行为进行分类嘛，因为做研究就是分类，对这些行为分类之后啊归出大概五六个类吧，那最后在归类之后，再去做解释，解释过程的都忘了。

当时那篇文章就是参与观察那个研究，我投给《管理科学学报》了。《管理科学学报》让我改，最后我就是硬生生的因为不让我挂中央财经大学就扔在那儿了，想想那时候应该是改改就改出来了，但是还是没去改了。那个大概就是收取情景行为的片段，然后再进行分类。我们用的方法是访谈里面儿用的扎根方法。但是在观察的时候其实用的就是对这些行为片段进行分类。

我导师对我那个参与观察的研究不满意

刘书博老师： 为什么啊？

受访者： 不符合他的预期。其实他一直想做的是想通过这样一个研究能够去谈文化的，比如说这个柔性啊，谈这个刚性、柔性，他那时候原来一直在说文化的柔性和刚性的问题。后来，他又跟我一个师妹又重新做了一个分类，用了那个刚性柔性的概念，发表了，他发的是英文的。那就是相同的材料，他用的不同的分析框架嘛，是这样一个方式。

刘书博老师： 明白。所以那个时候他不满意。那没有说有比较，比如说咱们现在很多都是跟导师比较密切的。像我们前面跟王震老师也聊过，那他会比较精细的去让学生知道怎么样能够达到他满意的一个结果。那当时您导师也没有说有过这样的一些过程性的密切的指导啊，主要是靠你自己去探索嘛。

受访者： 对，自己去折腾吧。这个方法呀，甚至说联系被试啊，联系这个厂啊，联系这个企业都是自己去联系

刘书博老师： 明白，明白。好的，于老师。那我这边问题就暂且问到这里。

吕梦娜： 刘老师，那我先问吧。于老师您好。我是吕梦娜，之前因为上过您的课嘛，然后可能您对我还有点印象，今天很高兴能够访谈到您，因为您现在既是管理学领域比较资深的教授学者，另外呢也是咱们商学院的很关键职位的领导。所以说可能对我们这个课题，会提出非常宝贵的一些见解。我这方面主要是围绕学科相关的一些问题哈。

首先第一个就是您现在的这个研究领域OBHR。那您觉得这个学科或者说这个研究领域在中国的发展情况是什么样的呢？它有什么特色？

受访者： 这个学科像中财人力系是在2001年开始招生。当年我们只有一个老师，就XXX老师当时是硕士学位来学的，学的也不是人力资源管理学的，好像是政治学之类的吧。那然后另外一个就是XXX老师，这两个老师其实早早就都退（休）了。就是这两个老师撑起这个专业，当然你也可以说“系”吧。我是我们系的第四个人。前面还有一个XXX老师，XXX老师03年来的，然后他也是硕士，就是硕士毕业从外地调过来的。然后我是我们系的第四个老师，这就当年的一个情况。

因为当时我们国内一直是政治挂帅，人力资源管理现在它是专业性的一个职业，那在过去其实我们是很不受重视的，因为在中国这个名字原来叫做人事科，它原来其实更多的是就是简单的发发工资、发发福利呀等等这样一个工作，你的工资都是跟你的级别（挂钩的），就是固定好的，就没有太复杂的，比如说我们叫人格匹配的问题。

所以那时候邓小平提出多劳多得，就是按劳分配，对吧？这个概念是非常非常重要的，因为在此之前其实是没有所谓的按劳分配的一个概念的，只是说你是什么级别，那你应该是拿多少钱的工资。当然一说改革开放之后，我们说就是按劳分配，多劳多得，那这个按劳分配你怎么对劳动进行定价，这个问题就是非常复杂的一个技术问题了，就是怎么来给劳动进行定价？怎么来对人的贡献进行定价？这个内容肯定是跟人力资源管理相关的，对吧？那比如说这个工作设计呀，怎么来衡量它的价值呀，怎么来衡量他的劳动的价值，这都有涉及到，就是它是有规律的，要尊重科学。那然后就是人力资源管理这个专业，就开始慢慢的兴起，我们这个不管是OB还是HR，其实都是西方的舶来品，我们要肯定西方科学的价值，就是我们不是唯科学至上，但是你要肯定科学的价值理念或者说程序方法。然后国内的这个人力资源管理开始慢慢走向正轨。

早期其实我们那个教材尽管有中国人写的，但是中国人也是抄老外的教材嘛。那后来在慢慢发展过程当中，我们是从台湾、香港开始的这种本土化的运动。因为最早的本土化运动是在心理学这个领域里面，后来再拓展到管理学这个领域。就是本土化运动让我们反思，要从自己的视角来看待我们所面临的问题，来给出一下解决方案。现在有很多就是在提中国的理论体系，这都是一些非常重要的尝试。我们不是说他提的一定对，但是你只要去提，那就有未来的一个空间，因为西方的学说也是一种解释，我们不能否认它的合理性，但是也不能说它就是绝对正确的真理，对吧？我们经常说的一方水土养一方人，确实我们要发展适合自己的理论体系，当然也不是说发展我们自己的理论体系，我个人一直认为理论没有西方理论、中国理论之分，因为理论就是要解释一般的规律的。但是有的是什么呢？有所谓的中国的故事、中国的经验、中国的方法，这些是可以有的。但是你不能说我有中国的理论，你可以说是中国人提出来的理论，是基于中国现象提出来的理论。但不能就简单的说它是中国理论。因为理论总是要超越情境进行解释啊。所以我觉得这个未来发展还是有很大的空间的，我们从紧随西方到开始有些人有意识在自主的或者说独立的谈我们自己的现象，用中国的经验去建构一些理论。

吕梦娜： 明白，那我听起来就是我们从跟随西方然后到现在本土化，那您觉得就是做这种本土化研究的人多不多？或者说有没有一个群体特征？我可能会觉得资历比较深的老师才会做这方面研究，像年轻的学者可能还是会跟随西方的这条路上走

受访者：到底什么叫本土化的研究？其实现在也是有很多的分歧嘛。比如说前些年特别火的一些概念“关系”、“面子”等等。但是大家最终发现走这条道路是走不通的，为什么走不通呢？是因为“关系”、“面子”它只是现象，你不能把现象跟概念等同起来。在做本土化研究的时候呢，其实很容易走到一个误区里面，就是直接用中国人本土的概念，就是生活的概念进入了学术领域里面。而生活当中的概念呢每个人都有自己的定义。因为学术还是要去进行对话的，不仅是我们自己小圈子的对话，还要跟圈子外的人进行对话。

比如说北方人说的饭，跟南方人说的饭，其实是不同的概念。北方人说的饭，一般是说的吃馒头、吃面食，那南方人的饭是吃米饭。那这种情况下，大家对饭这个概念理解不一样。那么这时候你再基于这个概念发展理论，大家就很难有共同的理解啊，最后导致遍地生花，也可以说是各立山头，每个人都有自己的一套理论，都有自己的一套说法。所以无论是西方的研究还是中国的研究，尤其是中国人的研究要走的远，其实很重要一点是不要简单地只是把生活概念直接纳入到学术概念当中。因为学术毕竟高于生活，要对生活现象有一些高度的概括，形成一个概念。所以在这一点上费孝通那个差序格局的概念（就很好），他没有简单地用“关系”、“圈子”等等一些概念，而是用差序格局。你会发现差序格局里面儿没有任何一个词是我们生活当中的概念，或者说我们所谓的本土化的概念。所以啊，生活化的概念只能叫做研究的对象，不能成为学术的框架，是这样。

然后年轻人来做这个呢，怎么说呢？如果我们想走的远啊，不计较一年两年的得失也好，或者说这一年两年的时间的话，做本土化的研究，做这种质化的研究，我觉得还是很重要的。因为你厚积薄发嘛。如果说你就想要尽快出成果，相对而言呢，流水线可能更适合。这就是两种：一个是机械化大生产流水线，另外一个叫手工作坊。不代表谁更高级。因为中国的教育也好，中国的学问也好，其实是一种生命学问嘛，从这个角度来说，就是他把知识和人生是放在一起来看的，那西方就说知识就是力量，知识就是工具，对吧？这是知识和人的目的是分开的。但对中国来说，就像那篇文章马克思·韦伯写的《以学术为志业》，然后其实后来复旦大学还是上海交大有个老师做过一个演讲，谈的是要把自己的生命情感融入到自己的研究当中，还有近几年比较火的项飚说“把自己作为方法”等等，这其实都有一些相同的观点，就是不要把自己跟研究对象给对立起来，其实我们本人就是研究问题的一部分，就是从这个角度来说的话，做质化研究还是蛮好的一个东西。因为我们不是为了产出文章，我们就是去探索生命或者探索世界，因为我们本身就是世界的一部分。从这个角度来说，质化研究还是挺好的。如果说你有耐心去做，然后也不急于求成，对吧？

但是我们也不能简单地去批判流水线。因为我们借助于流水线才能让我们过上所谓的现在的生活、你想要过的生活。你想过更好的就是另外一种生活的话，就是这个手工作坊式的，其实也是蛮重要的。但是这个手工作坊呢，你们要真的是沉浸于其中啊，然后才能成为大师，如果就像我们说现在有很多手工作坊就是黑作坊，那可能就不是那个套路吧。

吕梦娜： 对对。然后刚刚您提到了研究嘛，我想知道您是怎么去定义一个好的管理学研究或者OBHR的研究？我相信肯定有很多很多标准吧，但是对您来说最重要的几个标准是什么呢？

受访者：XX老师谈了好的研究的标准，就是叫“三有”，有趣、有用、有光。

我原来上课的时候也跟同学讨论过这样一个话题啊，我一直觉得其实研究最重要的是有趣。因为有趣是你坚持下去的基本条件，好奇心是最好的老师，对吧？你首先对这个东西感兴趣，但什么叫有趣？不是说你跟别人唱反调就叫有趣，真正的有趣是让人着迷的，科学就是解谜的活动，从这个角度来说，你首先这个迷本身就是让你觉得着迷的一个东西，它里面儿有黑箱、有未知的一些因素，而且让你很着迷。那么科学就是你去解这个谜。

那有些迷你不感兴趣，那就不要去做嘛，因为值得我们去做的事情太多了。所以最重要的是你觉得有趣，这个有趣是真的充满好奇的，而不是为了有趣而有趣，为了跟别人唱反调就说这个东西有趣啊等等。是真正的内心充满着这种好奇。我始终认为，对一个学者本人而言，我们不要把自己当成工具，首先你要去探索真正有趣的、让自己充满热情的一些东西，这个有趣不是为了跟别人唱反调，而是说我宁肯饿肚子也愿意去探索的一个东西。就是让你达到废寝忘食的这种状态，这是要真正的有趣。

刘书博老师：于老师，我这边稍微插一下。如果“有趣”是您看作好研究的一个重要标准。那因为我们现在这种比较规范式的学术生产方式在有趣方面就会打折扣，您觉得会不会有这样的一些副作用啊？比如说如果是那种自主探索的、不是特别规范式的研究，可能会有更多有趣的东西。现在一旦规范之后，会不会就限制住人们去寻找有趣，而更多是想要去做有用或者是更高效的一些研究呢？

受访者： 肯定会影响的吧，就像钱学森之问，其实钱学森之问不是问大学，问的是社会，问的是政府，对吧？你在倡导什么样的东西，你在支持什么样的东西。就像我们现在做这种自主探索的研究，做自己真正感兴趣的研究真的越来越难。

尽管好像这两年在我们学术圈里面有人在说去做有趣的研究，但是这个形势越来越严峻。像现在《管理世界》、《经济管理》等等，一边在说要去响应做一些案例研究、做一些有趣的问题研究，但一方面它在努力做政策解读。这个留给学者的空间越来越少，这个就是一个矛盾的东西。但是真正有趣的研究，就说总是有识货的人嘛，你十年磨一剑，你做这种质化研究，做这种另类的研究，其实是需要勇气啊，这更需要的是耐心啊，更重要的是你为什么会说有趣的很重要，就是你不发表你也愿意去做。

刘书博老师： 是的，是的。Ok，好，我就插这个问题。

吕梦娜： 那于老师，您对什么是一个好的管理学研究或OBHR研究还有补充吗？您刚刚提到了“有趣”这一个点。

受访者： 我就是从“有趣”开始说，我不是说我们要摒弃有用或者说不管他的理论价值，而是说有趣是一个开始，是一个起点。因为只有从有趣的方向去走，我们才能走的更远。是要有激情在的，就是当你跟别人谈你的研究、谈你的观点的时候，你是两眼放光的。在这种情况下，我们说你这个话题是有趣的，至少对你自己而言是有趣的。

2:05:385月8日9点的会议

首先要愿意去探索，然后当你真正探索到把这个问题达到一种真知灼见的时候，不是只是玩儿的时候，那这时候它自然有它的价值，就是不管是它的应用的价值还是它的理论 的价值。我们说从艺术作品的角度来说，梵高的那些画也是这个样子，他就自己画自己想画的东西与他的激情所在，虽然当时大家不识货，但是总有人会慢慢的发现它的价值，人们越来越喜欢他的这些东西。我说的有趣，不是说我只是沉浸在有趣就可以。像刚才说的有他激情所在，就说你愿意为此而付出自己的这样一些生命情感的东西，这是真正的有趣。当你真正付出的时候， 他其实他的用不一定在当下，而可能在其他的地方。 然后“有光”，你可以去看一下王老师的解释，但是我其实是另外的解释。

刘书博老师： 是说要对社会有帮助，对社会的一些问题，它具有社会责任感。

受访者：我忘了他怎么说的。我觉得“有光”，其实做研究也好，或者是说写作也好，本质上都有一个共同的使命， 就是“看见”，是让你看见你生活当中忽视的东西，你本来就可以看见的 东西，就要让你看的更多，看得更远，这是一个。 另外一个就是“启蒙”这个概念。我特别喜欢两个概念，一个是“启蒙”，另外一个叫“洞见”。启蒙是enlightenment，它中间有个词是light-光，就是一个研究， 它最重要的是让人看到希望、看到光明。

 那我在做研究的时候，我特别强调的一点，就是你的研究不要让人读了之后绝望，你让每一个读者读了这篇文章之后能够证明生命能够看到，希望能够积极的投入到 自己的生命活动当中，你 这样的是好的研究。所以我特别讨厌那种研究，比如说外向的人更容易有绩效，你可以把这个机制揭示的更清晰，这个也可能做出好一点研究。但如果说你的自变量就是说外向会导致你的好的绩效、更好的位置、更多的财富， 那我说这样的研究是什么呢？这是一个叫做 “邪恶的研究”，因为它 会让很多人看不到希望，我们知道其实这个内向、外向可以说55开吧，但是他就会 打击了那些内向的人嘛。还有就是说这个银行行长的脸部的特征宽高比的这样研究。那你要去整容呢？还是说抱怨自己的爹妈把自己生的这个宽高比？这都是一些邪恶的研究。 当然也不是说你自变量和因变量就是这个，然后他就一定是邪恶的。如果说你就是做这样的研究，一定要揭示他的关键机制到底在哪儿。不要简单的用一个自变量和因变量把问题这个全部一带而过了，就看不到背后丰富的、复杂的规律。

所以就是我在上课的时候，我会特别喜欢说的一个研究，我们现在一直在说一个二代的概念。 就是关于二代的研究呢，在上个世纪三四十 年代吧，就应该就做了很多研究。就是这个叫父母的社会经济地位决定了孩子的社会经济地位吧。那么这个不就是我们现在一直说的二代的问题吗？就是他在做的时候他没有简单的说父母的社会经济地位会显著的预测或者说决定孩子的社会经济地位，而是探讨了 中间的这个传导的机制是什么。现在一直在说教育的问题，就是教育是一个关键的中介。这时候你可以说这是一个有良心的研究，让人看到希望，就是说其实 不在于父母的社会经济地位，而在于这个教育。那我有钱，我给一个好的优质教育，我没钱，我也依然可以寻求教育的机会。当然也是从国家的角度来说，你要提供公平的教育机会。那我觉得这样的研究是好的，也就让人看到希望。不管让个体还是 让这个组织，还是让这些政府看到努力的方向在哪儿， 那这个就是好的研究。这就是我说要让人看到光，所以说叫enlightenment，就是好的研究也一定是具有启蒙作用的，摆脱 束缚的，摆脱这个浅见邪见或者是说偏见的这样一些研究。真的是 你不仅仅是看到别人的问题，更多人要看到自己的问题等等，这是一些好的。

刘书博老师： 我就想到那个启蒙运动嘛，启蒙运动其实也是人性解放嘛，就是让人们看到之前那些愚昧的观念对自己自主性的影响。

受访者：另外一个概念是insight-洞见，或者叫顿悟，sight也是光的概念，这种光是内生的光。所以那我说一个好的研究，它一定是有趣、有用和有光。

吕梦娜： 就是于老师还是比较认同王震老师他提出的这个好的研究的三个特点，可以这么讲吧？

受访者：对。

吕梦娜： 我问题就到这儿，谢谢您。

黄秋莉： 第一个问题您之前在读博士的时候非常喜欢看书嘛。然后在访谈的时候也听您提到各种各样 的书籍。那您现在的一个研究idea的获取方式和研究的模式是一个什么样的状况呢？就是和读书的时候有没有什么样的差别呀？

受访者：哎呀，我觉得挺惭愧的，作为人家的导师，一直都没给学生一个帮助，就是我基本来说不太想招学生，不想误人子弟。因为我个人是发了好多文章，但是好多文章都是叫灌水的文章，没什么太大的价值，就是都不一定出自自己的兴趣，更不会说是给别人带来什么新的知识，当然也可能成为别人引用的注角，但是也是彼此互相利用。从这个角度来说，我其实这些年都不想做这种所谓的规范的研究，就是自己更多的是去做自己感兴趣的东西吧。但是因为在带学生，所以我现在的原则是学生做什么研究，我就做哪个领域。学生的研究问题就是我的研究领域，然后就是他做什么研究，我就去看什么东西。我个人还是在关注文化、领导等等这样一些现象。

其实现在我最感兴趣的就是人和人之间的影响，叫人际动力，这几年其实也有好几个学生，比如说有做信任的，有做这个权力的，还有做师徒关系，或者是上下级互动等等，都是在这个领域里面去讨论的。

从研究范式的角度来说，我还是让学生在做什么呢？问卷调查短平快地会做，然后实验也尽可能的去做，然后质化研究也尽可能去做。所以我通常来说这个博士的话，我会要让他至少做两个研究。一个是质化研究，一个是量化研究。

因为质化研究还是必要的，因为让你真正的对这个现象 有更深刻的理解。因为量化研究你只看到数字而没看到数字背后的人的丰富的想法呀。或者说一些行为背后的这个动机非常复杂，所以纯粹做量化研究的话，其实你会发现，哎呀，这最后就是统计工具，就是用统计工具换来换去，或者说 跑来跑去。我不反对，但是我觉得不能只停留在这个属性的基础上。

黄秋莉： 您提到这个问卷调查和实验法，可能就是在OB领域还是就是非常受认可的嘛。但是现在可能在营销这个领域的话，好像有些老师就觉得问卷调查和实验就不太好，就更倾向于这种二手数据，那您自己是一个什么样的看法呢？

受访者： 都可以。因为你说的这个应该只是个别老师，但是我觉得营销领域里面儿主流的还是做问卷呀、 做实验。因为你想在营销领域里面，其实现在大家特别喜欢用的是那个叫赛勒的这个助推理论，其实在营销里面用的特别多， 其实他还是用做实验来做的，也包括大量大数据这种可能好像比较多。然后社交媒体上面的这种文本呀这种，有这方面的趋势，因为这是也是工具化技术导致的这样一个结果嘛。

但是大数据永远代替不了这个实验设计，就是它可能会替代问卷调查，但是永远替代不了实验设计，因为大数据没有因果。但是我们说做研究其实最重要的是探讨因果关系。因为我们人自古以来就有这个原因的追求。然后为什么我们说迷信呀？迷信的原因就是他要找原因嘛，这是一个基本的需要。

黄秋莉： 明白了，于老师，那就是因为现在可能有您的这个工作呀，有包括这种研究呀，然后培养学生呀，也有这种行政上面的这种事物。那您自己觉得现在这个工作过程中比较具有挑战性的一些地方是什么呢？或者你觉得就比较的随心所欲，都还挺ok的那种？

受访者：没有，我就觉得你们老是延期毕业，对我很大的挑战。前天这个x老师拿过一个表，就是我们这个在读研究生，就是主要是那个博士，也有硕士的一个今年情况表嘛，2018级的博士，当年应该招了差不多20个吧，去年毕业了一个，今年毕业了四个的。然后在里面儿就大概有十几个延期，但是不光是2018的，2017的还有在延期的。这个从这个工作上来说，我觉得就是这个 压力还蛮大的，就是怎么能让大家尽快的毕业。

刘书博老师： 就现在毕业难是因为标准高导致的，还是说学生好像不努力导致的？因为学生那边听到的跟老师这边也有一些出入。有一些可能导师也不是特别管，甚至很多博导他自己已经远离这个研究的兴趣或者是工作。那会不会有类似这样的种种的原因导致最终压力到您那边去了，我就大概是提这一点。

受访者： 哎呀，是这样，因为我们两年其实一直在努力的加强过程管理，这个过程管理一个是增加环节，然后增加这个学生的参与，然后让年轻老师参与到我们学院的整体的博士培养当中。但是我们这里这里面儿还有一个是什么呢？就是老师和学生双方的原因吧，因为不管是预答辩的论文，还是这个最后外答辩的论文，就是总觉得这个论文有点儿太水。跟我们毕业那个时代相比，我们那时候在技术上、在方法上其实是特别原始的方法，但是我觉得我们那时候还是做了大量的工作。我们论文的要求没有说你一定要有多高明的方法，或者有什么真知灼见或者创新，你最起码工作量要够。而工作量又体现了什么呢？你认不认真待这个事情上。所以我们现在也蛮痛苦的一个事情。当然你说学院这个博士毕业的条件，过去是一个A，现在提高了两个A，这是没办法的办法，什么叫没办法的办法？就是你毕业论文如果说太水的话又没法儿管得住，那那就通过过程吧，那就提高毕业的门槛，你得把这个罪要受下来，然后你再谈毕业的问题。两方面都有，有老师的原因，也有同学的原因。现在确实同学下的功夫，不是所有同学，但是很多博士同学其实下的功夫还是不够的，读书的这个日子，其实太轻松了，要么就是你读书的话也只是自己瞎读。现在学生圈里面有个学术共同体的问题，就是大家经常一起来讨论。因为博士来说你真正有用的许多方法呀、许多问题其实不是跟在老师那儿得到，是同学之间互相切磋。所以我们今年有个研究里边儿我就特别提了一个叫什么呢？就是用切磋这个概念，我觉得这个切磋是非常重要的一个学习的方法，你跟同辈之间互相切磋问题，加强自己对这个问题的理解，或者说互相交换观点嘛。那博士生论坛，从原来冬夏两季，现在改成一年四季，我们给大家创造这样一个机会去讲自己的东西。你讲着讲着就能更清晰，但是你看来看去，只会越看越糊涂。所以我们说语言有它特有的这样一个内在的功能，就是你你在讲的过程当中，你对问题的理解就越来越深刻。所以我还是希望大家都去讲。但是我们x老师那边儿了解的，我们很多同学把我们的这个博士生论坛当成一种负担。好像还说学院给他增加了很多额外的要求。从学院的角度来说，给他搭这个场子请老师来做这个讲座，来做这个评委，学院是要花钱的，需要花时间去做这个事情的。但是我们有时候确实有些同学又在抱怨这个事情。但是我们还是还是要做的，我说这个意思就是我这个位置上就是非常难两难的一个事情，就是我一一边希望我们同学都是如期毕业，一边又担心我们的最后毕业的论文太水。所以只能在过程上多做一件事情吧。

吕梦娜： 您觉得就是一个不太水的或者比较合格的甚至比较优秀的博士论文应该是什么样子的呢？

受访者： 一个不太水的论文，我们首先工作量要够，文献的积累呀，或者说你的思考，然后你的这样一个数据的分析，等等，然后看起来还是做了大量的工作和心血在里面的。虽然我们现在也说要都创新，但是哪来那么多的创新贡献？你首先工作量要够。然后有那好的问题，你对某个问题进行了这个系统深入的探讨，并且提出自己的观点和理论框架。我们始终还有个目标是什么呢？是理论贡献的，你不是用理论去解决了一个问题，而是通过你解决这个问题，反过来对这个理论有所贡献，推动这个理论的发展。

吕梦娜：您怎么评价您带的学生的这个博士论文？

受访者：首先他们工作量 都够，这个是肯定的。但是还是有些目标是没有实现的， 但是没办法，还是有有遗憾。就是最基本的没有建筑一个理论。

吕梦娜： 但是我发现他们的质化研究做的都蛮扎实的，就是也是出现了理论的模型。

受访者： 我不是说那样一个理论模型，而是能够通过你的这个研究呢，对权力的本质有进一步的理解。因为我们经常说这个概念是一个我们认识世界的一个窗口，最好的研究是让让人对一个概念有了新的理解，而不是简单说你建了一个什么样的模型。你模型最重要的贡献，你还是要说你这个模型能够说明这个概念，就是大概是这样吧。我觉得就是他们做的工作还是够的，就是在有些地方还是有遗憾。

受访者：我就问一下，前两天让老师跟咱们同学一一沟通咱们有什么样的一个困难呀或者有什么样的诉求呀，你们提了吗？ 你们没提，没困难也没有诉求？

吕梦娜： 我们是那个老师拉了一个群，然后就是在群里面问了一下，然后大家就报了 一下平安而已。

受访者： 我知道。因为我就看到了我们在那个我们不管是MBA群里呀还是这个什么，就是大家最后提到大家都是没困难没诉求。

刘书博老师： 这个群里面问，那有一些敏感的问题，那怎么说呀？其实我觉得不应该在群里，他应该一对一的嘛，因为我们要求有一对一。

受访者： 实际上就是我觉得这是一个很好的机会，就很明显就说，这个新冠快三年了，我们大家都有很多困难，生活当中困难，工作当中，情绪上的一些困难、心理困难。然后我们也有很多诉求，大家好像就是都不说，总觉得说是不是就说也没用？因为这个我们现在就是生活不正常。你只有去表达你的困难，表达你的诉求，别人才会看得见。但是我觉得还是挺遗憾的，就是我们说上来的其实就没什么困难和诉求。

黄秋莉： 其实我们可能也是希望导师和学生的这种沟通应该是一个比较持续化的或者是常态化的吧。就是猛地这样的话我们也不敢说，也没啥说的。

刘书博老师：我们探讨过说，我们一块儿在做一个这样的研究，如果未来有研究产出，但是我又不是你们这个正式的导师组的成员。然后可能导师又不是特别就是好沟通，那这种情况，那我们署名的话是怎么署名？那比如说我现在学校评职称，然后像正高的话又必须只认第一作者，通讯他都不认。那如果我是第一，你们似乎作为通讯又没有什么于你们毕业有利的这样的帮助。所以制度层面，学院是不是能有一些更多样性的安排？所以这个问题可能也是请于老师给帮忙看看。

受访者： 梦娜，你是2021级的，你们有这现实的这个困难，为什么不提你们的诉求。比如说希望降低博士毕业的条件。

吕梦娜： 于老师，当时我刚上博一的时候，我提了三次意见，没有一次哪怕被口头提到的，所以我是心凉。这次收集那个意见本身应该是一对一的，但是也只是建了一个群。

受访者：你需要用正式的方式提，你总不能一说你碰到老师说：你给我降低标准。因为作为一个体制来说他肯定要有正式的程序。因为只有正式程序，才会把它纳入到议事当中。也不能说老师去微服私访等等，你去了解这些情况这个事儿。尤其是在这种正式的方式场合下征求大家意见，那你完全可以说：因为很多学校都降低了这个博士毕业的条件，那是不是说我们也降低一下这个标准？但是我们说在研究质量上要提高，但是在这个毕业的条件，在论文发表方面我们要降低等等。因为既然这是学院学生的想法，那肯定要去议论。这是你跟 体制打交道的一个很重要的方式，你不能跟他只是一个抱怨，你要去提你一个正式的申诉也好，诉求表达也好。所以我们特别地让卫老师去给老师们做了一个问卷，让老师去填学生的这样一个情况。而且让每个老师单独给卫老师说，但是还是没 几乎没有老师去说。

黄秋莉： 可以面向学生直接调查吗？就是可能你还要通过导师在网上反映问题，这个是不是……

刘书博老师： 就导师在中间，学生会觉得有一些意见直接跟导师讲，导师会有一些不满意的地方。有时候导师可能很重要，但是我觉得不是所有的导师都是那种春风化雨的或者是很关怀的，可能还是有很多那种导师，他不希望学生提出一些反的呀，或者是更多自己想法的这种意见，可能会有这样的一些担忧吧。

受访者：因为这本身也是一个制度化的考虑，也是通过这种方式让导师去了解一下学生的情况。后来也说让导师去跟学生解释这个防疫的政策，但是最起码首先同学要把自己的诉求都表达出来，我们可以理解国家的这样一个环境政策。但是我们同样也有诉求。因为可能确实又提过三次意见，都没有下文儿，但是你要反思一下，你怎么来提意见的，你跟谁提过意见？

吕梦娜： 之前就是类似于导员儿征求意见，就是刚刚上这个博士的时候，就是比较正式的这种渠道，包括填问卷什么的，我觉得是比较正式的，包括我们博士生开会的时候也会提到过这个。我没有说就是私下里就是必须怎么怎么着，但是好像也没有被解决。所以觉得是不是行政老师有这个KPI的这种感觉。

受访者：行。反正我们还是希望大家都如期毕业，同时也希望大家做出一个让人满意的毕业论文。

刘书博老师：于老师，我这边有一个关于中国大学管理和商学院管理特点的，因为您也是在做了这个中层领导挺长时间， 那整个过程您觉得是不是有一些中国特色的内容？因为我之前在英国，我觉得那边好像也是有一些政治方面的东西，但是我们中国这块儿，政治性又更强。就比如说我看新闻说这个南京大学、兰州大学等等，他们又开始带头说不参与国际学科排名了，那可能就是因为我们现在又有一些从上到下的一些想法。所以这方面在商学院的管理方面有没有体现？尤其是在这个商学院有没有一些中国特色的东西？

受访者：中国高校是知识分子比较扎堆的地方，当然现在这个知识分子到底什么叫知识分子？就是现在也是很难去说清楚。就每个人自己理解不一样，现在知识分子是不是古代那个士的概念，就是这个都是大家去讨论 的一个话题。但是总的来说，因为在中国就是我们高校也是体制内嘛，就是这个政府如何跟知识分子打交道这个问题其实一直是一个没有解决的问题，一直没有很好的一个跟知识分子打交道的方式。所以这也导致其实高校管理，真的是很难谈得上好的管理。而且有时候也在说教育吧，这个问题就是特别复杂。就是说要培养一个名校要经过好几代人的努力才可以。所以说我们很容易给我们的学校、为什么我们现在高等教育的不尽人意找一些借口。但实际上，我们也知道香港科技大学其实是九十年代初才刚刚成立，也就十几年的时间他可以成为世界名校。这个时间短，没有历史积累，只是一个借口。根本原因我们还是没有很好的去处理好怎么来跟这些知识分子打交道，或者说怎么来发挥这个知识分子作用等等。

其实你还有可以回到“钱学森之问”这个问题上，为什么中国培养不出大科学家。就是我们还是没有很好的学会跟这个知识分子打交道的方式。那这个对高校商学院来说其实也蛮有意思的一个事情，就是本来商学院是教别人管理的一个地方，教企业家和管理者如何做好管理，而且现在最流行的课程就是领导力，如何做领导。但是中国，我们不能说百分之百的这个商业也好，经济管理学院也好，但是绝大多数，商学院管理学院都是管理很差的一些地方，你一边在教别人管理，一边自己做的管理其实很差。当然你可以说这是知行不合一，就有这样一个解释，但是确实是中国这个商学院管的好的不多，管的差的很多。

刘书博老师： 那您觉得管的不好是商学院的特色呢？还是说其实所有的学院都差不多？

受访者： 所有的学校都差不多。

刘书博老师：那您脑海当中的那种有序的或者是比较好的这种学院的管理模式，它应该是有什么特点？

受访者：其实也是都在探索。就是西交利物浦，我还不是特别了解它的内部机构运作的模式，但他好像做的还是不错的。然后南方科技大学的社会学系，而且他有些就是人文学院，我觉得他那种模式也挺有意思的。就是更多的是你把这些人聚在一起，大家去做研究，或者说形成这个群落的模式，而不是用行政去做主导的这种模式。、就是不光商学院的各个就是大学里面的各个学院，他首先是一个学术问题，而不是一个行政机构。就是到底是教授治校还是教授只是治学？到底权力属于谁？这个问题就是太复杂，因为你理想的应该是教授治校，而不仅仅是治学，但是现在中国大学都是在教育部的管理下。那如果一个大学他是教授在治校，那他的这种专业身份也会发生内容的改变。就虽然他是一个学术的专业身份，但是到了这个岗位之后，他就需要去执行自上而下的那种行政指令，所以他即使是来自于一个教授背景，可能到了这个岗位上就会发生一些工作内容和理念的改变。

黄秋莉：还想问一下就是老师关于这个AACSB的一些看法。就是这个好像对我们学院来说是一个非常重要的事情，然后我想知道这个AACSB给我们学院带来了什么样的改变和影响？

受访者：就这个AACSB，它是叫什么美国精英商业俱乐部也好，或者说这个商科教育也好，这就跟我们说质量管理这个PC的循环一样，它是一个程序化的，是要规范，是要程序。从这个角度来说，因为中国的高等教育，一些商科教育其实发展还是比较晚。 那我们确实是需要有一些制度化、 程序化的东西，标准化的东西，因为你不能说我们中国的大学老师都是野蛮生长的，就是按照自己的方式去上课，按照自己的方式去做事情，从这个角度来说，我们确实是需要有这样一个 这个标准。但是这个标准它是一个外在的规范，那如何来起作用？

你首先要认同这个规范它才起作用，所以在这个过程当中，他最终的影响，不是认证过了之后我们有什么样的一个影响，现在比如说大家去上课呀都会把AACSB的标识挂在自己的PPT上，或者说在印的名片上，这其实是没有什么 意义的。就是他的真正的意义不在于这个认证之后给我们带来什么样的好处，或者说让你有什么样的一个标识。 而是在认证前为了完成认证，就 上下的努力也好，或者讨论也好等等做的那些工作，那些工作让我们重新认识了 管理教育。比如AACSB很重要的叫使命驱动的嘛，那这就是所有的行动都要围绕使命，所以对我们来说也是一样的，我们现在叫教育立 德树人，不忘初心，牢记使命，这是很重要的。 你只有回归原点，回归初心，我们才能知道我们到底为什么来做这个事情。其实它之所以有这么强的生命力， 它背后的逻辑其实就是它恰恰是符合一个现代化组织的一个管理模式。尽管我们叫做高等教育，这个大学是一个特殊的组织，但是它也是一般的组织，那作为组织者来说，它的基本的逻辑就是在这个过程当中，大家商科教育的目标和基本方法上我们达成了基本的一致。而且他给了一些基本的标准，比如说作为任何老师来说，你应该达到什么样的一个水平你才可以站上讲台，而且成绩你不能乱给，然后因为教学，你不能说我每年都在重复，就是一套课件给不同的学生去讲等等。那么这个情况下确实在高校里面儿是个司空见惯的事情，但是AACSB认证上说你 不能只拿着一套东西给不同的学生一届又一届的重复，应该持续改进。你要基于上一届目标的实现程度来反思自己的教学过程，然后做出一个改进。然后在下一届新的学生能够有新的方法，能更好的达成我们的教学的 目标，这套方法就把我们过去的那些简单的比如说不好的一些东西，它可以用程序化的方式 固定下来，建立一个基本的流程，我觉得这个对于商科来说是非常重要的，通过认证做的各种努力而形成这样一个统一的理解、达成共识吧，有了一个 基本的规范在。

对中国高等教育来说，我们毕竟还是中国，在文革是也是有断层的，那我们从80s开始办高等教育到现在也不过四十 几年，其实就是一直在摸索嘛，尤其是引进这样一些成熟的 体系理念也是非常有必要的。但它也解决不了所有的问题，还有很重要的问题它还是没有解决， 就是所有的规范始终没有解决的， 是我们这个人的认同的问题，因为你这个外在的认证还是一个外在约束，你怎么让老师们热爱教育、热爱教学， 热爱学生。这可能是这个认证没法解决的 一个问题。

黄秋莉： 那一个硬币有正反两面，AACSB可能确实是一个非常好的理念。我们商学院发展也有很重要的作用。那它在我们这个因为它是一个外来的规范。在我们这种商学院，这种包括这种中国传统文化呀，这种政治体制。这在我们商学院落地的过程中，它有没有带来一些负面的影响？

受访者： 但是什么叫负面的影响？就像你刚才说一个问题总有两个方面。

黄秋莉： 这个制度在执行的过程中有没有带来一些意想不到的结果呢？

受访者：任何规范的引进总会带来一些冲突， 这是肯定的，然后所有的成长都要付出代价的。从这个角度来说，认证过程 当中其实也面临很多矛盾。比如有些老师甚至说连PPT都没有，就一张嘴，然后就到教室里面去随便讲一讲，就这样就可以完成自己的工作。那你现在要课前做这么多工作，课后还要做那么多工作，而且那个认证要求老师对学生有充分的了解，这个于我们这个大学课堂来说其实是很大的挑战。那就可能会导致大家其实还是在形式化的完成任务，就没有真正的坐下来去讨论这个东西。我们用了大概六七年的时间过了认证，其实真正艰难的是如何在这个认证的道路上一直按照认证的理念叫做持续提高，这是最重要的。因为前面只是有这个身份，你怎么来把这个身份做出它的内涵呢？这可能是后期要继续努力的。因为前面我们还是有很多是材料写的好，就是做材料做出来的，而不是我们实打实的，材料只是我们工作的一个总结而已，当然说认证最重要的，他也强调不要光看材料，为什么他一定要有现场认证？就是在于而且查你的过程的记录，就是说我不是要你说的，是要看你去做的。

黄秋莉： 明白。然后“预聘-长聘”制度在我们国家的推行也是一个非常就是的话题，我看到很多学校都已经在这个制度了，您对这个制度有没有什么样的看法或者是了解呀？

受访者：那“预聘-长聘”这个制度就是国外的一种方式，我们是要取长补短也好，或者是学习别人的长处，然后我们改进我们的这样一个管理，这个始终是要有这样一个问题，就是不能唯我独尊，唯我独大。我们始终要有谦虚的。就是中国人的传统观念，一直是谦虚， 你要学习别人，学习别人的要看到别人的长处，也要学习别人的长处。那“预聘-长聘”确实也是一个比较好的方式。一方面在预聘期间，你是要有压力的，同时因为这种压力就是让你更多的投入，更多的这样一个追求产出，追求尽快的成长。那么从人才成长的角度来说，也是一个必要的措施。

但是因为我们也说这是一个筛选机制，对老师来说是找到自己合适的位置，或者说合适的地方。然后于学校来说找到自己想要的这个人才， 这个制度或方法本身没有好坏。如果说有好坏的是你在用的这个办法的时候，你怀的动机。这就是我们中国许多企业在找咨询公司来做的时候，他其实不一定是用咨询公司来给他解决问题，他其实叫借刀杀人嘛。那这个也是一样的，就是你的动机是啥？你是要去干嘛？要是真是从老师的角度来说，让他去更好的成长，还是说是追求成果，为了让学校发表更好看，为了得到更廉价的这个劳动力，然后我再去这样去做？这个动机不一样，最后导致的结果也是不一样的。另外一个是大环境的问题，就是这整个社会有没有都用这种体系，然后就是最后只看这个老师的成果，或者说只用老师这个人的一个成就来说话。但是我们知道其实许多学校在做的时候，人为干涉的因素很多。就是有的人是同样的条件，有的人可以，有的人不可以，那这种情况下预聘制他就只是一个杀人的工具，它不是一个让个体更好成长，促进组织良性竞争的 一个工具。所以说制度本身没有 好坏，有问题的是你使用工具的动机和方式方法。

黄秋莉： 我觉得老师说的很具有启发性，谢谢于老师。

刘书博老师： 谢谢，谢谢，那个时间也不早了，非常感谢，回头把那个记录发给您看。

受访者： 再见，再见。

# 受访者3

刘书博老师： 好的，行，那我就先问这个第一个问题，就是张老师我看您本科就出去，然后在什么时间就决定要走上学术道路？因为这个其实很多在英国的留学生可能在研究生读完就回来了，就想要找工作了。您是一直读到博士，所以我我想了解一下，当时您选择的这个学术方向有什么考虑？

受访者： 我当时其实是研究生毕业回国了可能小半年吧。然后我回国其实工作了一段时间，然后工作了可能四个月左右吧，然后后来就是我感觉到那个时候可能年纪也比较小吧，然后我觉得就是作为研究生回国也没什么竞争力。可能我工作的那个行业也不是我自己特别心仪的行业，所以我就后来又回去读博了。

刘书博老师：那你当时对于这个做学术有充分了解吗？比如说你相比于工作，可能您觉得自己做研究会更加擅长一些或者更喜欢一些。

受访者： 其实我对于学术体系就是特别像英国这个学术，其实我不是很了解。 但是我觉得我自己还是比较擅长于就是这种独立做一些事情吧，然后包括其实我在读本科和硕士的时候，我觉得可能就是学习对于我而言，其实就是相对会比较简单或者比较专注的一件事儿，我自己还是比较喜欢。

刘书博老师：

受访者：所以我就还是决定回去了。

刘书博老师：那当时在英国学习的......您进行这个研究生包括博士的这个学习，他是需要上专业课吗？然后再开始自己的这个独立的论文撰写。

受访者： 就读博期间，是吗？

刘书博老师：对对。

受访者：我们学校是没有强制的专业课，但是就是每周都会开那个workshop，就是不同类型的。然后如果跟你的研究方向比较契合，或者是一些研究方法课的话，就是要去自行选择的，没有强制的。

刘书博老师：明白，那个时候您的这个研究的议题是怎么样确定的？因为我们都是要做一个毕业论文嘛，这个题目是跟着老师吗？还是说自己有一个独立的方向？

受访者：我自己基本上就是三个方面吧。第一个方面是我的那个研究生就是硕士论文，就是做那个品牌管理方向的。然后第二个呢，就是我硕士的时候的导师是我读博的那个学校毕业的，就是他的博士也是在那读的。然后他的研究方向就也是品牌管理。所以就是当时他还帮我写了一份reference。所以当时就是我自己基于导师的方向，就是研究生导师的方向也是这个方向。还有第三个原因就是我自己可能对这个比较感兴趣，所以就还是确定了这个方向。

刘书博老师：那整个博士学习期间，您觉得这个经历有没有一些具有挑战性的，或者说遇到一些事情会跟您读博士之前想象的挺不一样的？就是有没有一些挑战或者意外类似的经历？在读博士期间。

受访者：其实我觉得最大挑战就是从研究生...因为英国硕士不是都一年半嘛。

受访者：所以对于学术的整体其实就没有特别多了解，所以基本去开始读博的时候算是一个空白状态吧。然后就是在前期还是需要挺长的一段时间摸索，包括其实像读博的时候才开始大量的阅读学术文章嘛，所以其实语言方面也是也算是一个小挑战。

刘书博老师：嗯。

受访者： 还有一个比较大的挑战，就是文章吧，可能就是到博二、博三的时候开始写文章的时候。当时也是反正就是感觉还是比较有挑战的一件事儿。

刘书博老师：那比如说就是可能写作本身得用外语，还是说写作有一些方式方法会对您造成挑战？

受访者：我觉得可能更多的是方式方法吧。

刘书博老师： 那比如说在学习过程中有没有专门的老师会带您去学习这个比如说研究方法呀，写作方式呀，还是说主要是靠自己在这个实践当中去点滴的摸索？

受访者： 我们那边就基本上都是靠自己，你用的方法可能导师都不太明白。基本上我感觉整个三年我的导师可能就是起到了一个辅助和一个讨论的作用。

刘书博老师：

受访者： 没有就是真的去教我怎么做这个

刘书博老师：那听起来跟我是比较像的，因为反正导师每周会要求我跟他见面聊一些具体的进展或者是泛泛的交流一些问题。

受访者：进展，对对对

刘书博老师：那您觉得这个过程...

受访者：咱们应该挺像的。

刘书博老师：对对，是的。那这整个过程其实就有人会说这是放养式的，然后导师也不是特别的...像我有一些同事就是每周都要开周会除了周会还有一些其他的这种例会什么的，然后会带着学生去...

受访者：其实我非常...

刘书博老师：您说。

受访者： 对，其实我的感受就是我觉得国内的这个整体的学术训练的体系，我觉得是比国外要完善的。

受访者：因为国外确实就是我觉得可能导师他真的是一个辅助作用，他不会说是去教你什么或者是去真的去指导你、催促你，更多的还是我觉得个人吧。

刘书博老师： 对对，所以那边应该我们都是一致的。然后最后毕业论文通过外审，然后答辩就可以拿到学位了，也没有说要必须发表一些期刊论文，这样对吧？

受访者： 对对，我们应该一样。

刘书博老师：然后您当前所在大学的博士毕业是需要再发表一些核心期刊论文吗？还是说也就一篇大的博士论文？

受访者： 好像现在我们学校应该是要求两篇c吧，两篇c起

刘书博老师：c刊还是c级那样？

受访者： c刊，就cssci

刘书博老师：ok，明白。当时英国那边您导师他有会经常提到这个发表论文的这种考核压力吗？

受访者：我的导师没有，但有的导师有，可能还是跟导师风格吧。

刘书博老师： ok，那所以当时您观察到的这个商学院或者是经管学院他们是有什么样的一些风格的不同呢？就整个英国那边的商学院。

受访者：我的感觉就是可能文章发的特别多，或者就是特别self-driven的那种老师，他就会他就会push学生去发文章。

受访者：对，对。但我导师就是比较佛系，他就是“你看你自己的情况”，然后他也不太让我去帮他做事情。我觉得可能也是因为我们没有奖学金吧，所以导师总觉得可能我们要给他干活的话，他自己可能觉得有点过意不去啊之类的。

刘书博老师：明白。那您博士毕业之后，有没有想着说留在英国那边工作，或者说当时都在看？

受访者： 我就都在看，对，我是都在看。

刘书博老师： 那博士毕业的时候就也是比较坚决的说要走走向学术的，像大学老师这样的工作岗位，还是说也在看这个行业呀，公司呀？

受访者： 我就是一直想进高校，所以就还是目标比较明确了。

刘书博老师：那您当时坚定的一个想法是...就为什么一定要进高校？你当时怎么想？

受访者：其实这个就是，第一是我比较可能喜欢比较简单的工作环境。

受访者： 然后就肯定还有高校的一些优点吧。其实当时的话，确实是对国内高校的工作内容包括考核其实我也不是很了解。我当时只是觉得高校的环境比较简单，然后可能时间比较自由。

受访者：对，觉得是这样的，就是其实这里会比较好一些，氛围比较好一些。

刘书博老师： 所以当时我会想，因为我会观察英国的那种高校的环境。所以我觉得中国似乎应该差不多，可能有这方面影响。我不知道您对于高校的这种期待是不是也受到英国那种大学环境和特点的影响？没有国内的一些前辈老师给您说说中国的一些特色不同吗？

受访者：我当时还真是不清楚，回国以后才体会到的。

刘书博老师： 那张老师您是哪儿人呀？

受访者： 我是山西人。

刘书博老师：山西的，所以当时回国找高校工作是聚焦在北京地区，还是说也全国各地都看了看？

受访者： 我就是基本上是聚焦在北京了。

刘书博老师： ok，那您当时找工作经历咋样啊？我不知道这个是不是看了好多学校也遇到了不同的这种面试方式。还是说就一下子......

受访者： 当时其实我是中国和英国都申了，英国也申学校了。

刘书博老师：嗯。

受访者：然后我在北京就是我记得我是申的首经贸和中国政法。因为其实是我在毕业的前一年我就申中国政法了，然后当时的话就是就收到了一些反馈。但是那年我没有答辩嘛，所以时间就没凑上。然后第二年的话我就又跟之前那个跟我联系的老师联系了一下。所以就是政法这边相对会比较顺一些，就是时间也比较快。然后后来那个英国也有一个学校就是我的博士导师在那边也有教职的。但是那个就是有点儿晚了，这边已经定了，所以我就直接来政法了。

刘书博老师： ok，明白。那整个面试的过程怎么样？还挺顺利嘛。

受访者：我自己感觉我自己比较紧张，但是流程还是比较顺。

刘书博老师： 那当时您选的是首经贸没有选对外经贸呀。

受访者： 没有，其实我就投了政法和首经贸。

刘书博老师： ok。那听起来还是蛮顺利的。

受访者：我觉得相对还是比较顺利的

刘书博老师：那您觉得就是比如说咱们做大学老师这份工作最有趣的、最有意义的，或者您觉得这种价值感最凸显的，对于您来说是有哪一些工作内容呢？

受访者： 我其实自己的感觉是我比较喜欢和学生互动，其实我的成就感大部分来自于学生吧。

刘书博老师： ok，我理解。那研究呢，你觉得研究这个价值感不够凸显？

受访者： 对，首先我觉得国内体制和国外还是还是不太一样吧，就是国内的研究怎么说...我觉得还是要在国内有一定的经验积累，可能会比较顺一些。但是我自己现在我还没有感觉到顺。

刘书博老师： 明白，那国内做研究的方式方法和内容跟英国那边会有一些区别嘛？

受访者： 我觉得中国可能更注重一些学术圈吧

受访者：就有自己的一个学术圈子。因为如果可能在国内读博的话，可能就是会有导师带嘛，所以很早的就进入这个学术圈。然后包括你的发展方向啊、整个你未来的方向、未来研究的发展我觉得可能都会比较清楚吧。

刘书博老师：是的，就像我刚才说的，像博士导师会在博一甚至是研究生阶段就有比较清晰的标准化的培养方式。好像英国真的是没有太标准。

受访者： 对对对，就是完全看导师风格，我感觉是。

刘书博老师： 是的，而且我觉得就是英国导师还是比较尊重学生的研究兴趣的。

受访者：对对对，确实是的

刘书博老师： 其实像我的经历就是他不会主动给我说你要做什么，他更多的是问我说你现在想做（什么）？

刘书博老师：您当时也是这样？

受访者：对对。是的，是的

刘书博老师：那您当时也是研究生阶段就开始有一个兴趣方向了？。

刘书博老师： 那现在呢，现在如果是做研究的话，您的这个方向还有变化吗？跟英国读书时期（相比）。

受访者： 方向变了

刘书博老师： 为什么变了？

受访者： 其实因为我当时做的是跨文化研究，然后包括像一些品牌研究，其实就在国内的话，这个话题首先就是比较偏社科嘛。然后其次是我的同事很多给我建议，就是觉得文化这个话题太大了，不太适合青年老师去研究。然后另外一个方面，就像品牌研究其实在国内...我觉得现在特别像市场营销方向不算是特别主流吧，它比较小众。

刘书博老师：嗯

受访者： 所以文章也不太好发，所以现在就转了。

刘书博老师： ok，ok，明白。行，那我这边问题大概就问到这里，我刚才围绕的一个中心还是您的求学经历，还有就研究兴趣的形成。那下面看一下秋莉或者梦娜，你们谁先问。

吕梦娜： 好的刘老师，那我先问吧，张老师。

受访者：嗯

吕梦娜：我想接刚刚刘老师这个话题，就是您不是后面转研究方向、研究领域了嘛，我想问一下您当前这个研究领域是什么？以及当时您是怎么转到这个领域的呢？

受访者： 我现在其实转的就是基本上两个方向吧，一个方向是创新创业。

受访者：然后转这个方向主要是因为我现在在中国政法的教研室，我们是创新创业和战略教研室，我们教研室的老师包括我们系主任，然后包括一些前辈老师他们是做这个方向的。所以我们在教研室、在科研会啊、各种会上可能会对这个方向聊的比较多，所以我就基本上是跟他们契合了一个方向。然后还有就是我原来一直做的市场营销嘛，就我这个方向。但之前的话可能更多的就是聚焦在平台，然后现在更多的就是聚焦在社交媒体了。因为这块儿其实我自己的兴趣是一直在市场营销，然后现在就是对社交媒体这块儿我觉得还是我的兴趣点，所以我还是在基于我的兴趣点在做一些事儿，做一些工作吧。

吕梦娜：了解。就主要是结合您的兴趣以及学院前辈老师给您的一些讨论。

受访者： 对，就是这样。

吕梦娜： 因为我们之前访谈也有了解过。有的老师转话题可能是因为申请基金课题方面的压力，就不知道这个没有影响到您是么？

受访者：也有压力其实。

吕梦娜： 这个申课题也会影响到您研究方向的转向吗？感觉您的这两个方向和课题要求好像还是比较契合的。

受访者： 对，现在的这个主题的选择，就是转到现在这个方向比较契合嘛，但我之前跨文化和那个品牌的话就不太好申了。

吕梦娜：了解，明白明白。这是接刚刚刘老师的话题稍微问了一下哈。然后我这边有几个关注的问题。因为刚刚了解到您找教职的时候首选的是北京的城市嘛，然后就是关注了首经贸和政法大学。那我想问一下，您当时聚焦这两个学校的原因是什么呢？

受访者：我记不太清了。可能是听我之前的师兄师姐有在首经贸工作，可能那边的考核相对会比较轻松一些。

受访者：然后政法是因为我前一年申的时候就有政法的消息嘛，所以第二年就还是延续了政法

吕梦娜： 您第一年当时去政法面试的时候，那个消息是也是从师兄师姐这边来的吗？

受访者： 那个不是，那个时候我就是从网站上看到的。

吕梦娜： 那感觉还挺有缘分的。就是感觉您并没有我们现在所说的海投。但是能够找到这样的一个学校，并且后面还在这个学校里面待下来了。

受访者：还有一个问题，是因为当时其实我是有朋友之前在政法上的学。

吕梦娜：

受访者： 对对，因为我们学校比较特殊，就是我们主要的学科是法学嘛。

受访者： 所以我们商学院就是算是政法比较小众的一个专业吧。然后当时政法就因为...我是学市场营销的嘛，所以当时政法正好缺市场营销口的老师。

吕梦娜：了解。您前面也提到就是说其实在英国就是您本硕博都在英国嘛，然后跟中国可能不太一样。但当时您选择高校的时候好像并没有非常深切地体会到这种不一样。那您现在也是在高校工作了有四年多的时间了吧？

吕梦娜：然后您觉得现在的现实和您当时的那种理想之间的一些差距是什么？因为刚刚您提到了导师方面的这个差异嘛，然后其他方面您还能说说吗？比如说就是管理方式，对于老师的管理也好，对于学生尤其是研究生、博士生的管理也好，您能从这几个方面来谈谈嘛？

受访者：对于老师的管理，因为我没有在英国工作过，所以我对那边在职老师的管理其实不是特别了解。我知道的可能唯一的区别就是，我有同学在英国工作以后呢，他们可能前两年他们的所有工作就是要偏向教学的。前两年是没有科研工作的。

受访者：对，可能也是为了让青年老师能够更快的适应那边的工作环境吧，我觉得。

受访者： 然后其实对于培养学生这边，我觉得差距还是还是挺大的。我觉得国内的培养体系更成熟吧，就更成体系吧。在国外的话基本上就是选择读博的话，我自己的感受是你全程要自己摸索。这其实就是很大的一个差别。

吕梦娜：那以您自身的这个体验来说，您更倾向于...假如现在你还有选择的话，您更倾向于国内这种有体系的还是国外这种自我探索、自我摸索的那种？

受访者： 其实如果不考虑环境啊或者文化环境各方面，单考虑读书这方面就是学习科研这方面的话。如果想要很快地去了解科研，能够很快的进入这个角色的话，其实我觉得国内帮助是要比国外大的。但是如果是站在比如说就是像你们读完博，肯定大部分读博的学生可能未来有一条路，可能就是选择高校就职嘛。

受访者：但是现在国内的高校就职的这个前景的问题就是...相对好一些的学校，他可能都会要求一些国外背景。

吕梦娜：是的。

受访者：所以说这其实就是一个很矛盾的状态啊。你在国外读可能在进校的时候会有一些优势，但是你进校以后你的后续的科研工作可能就没有优势了。所以我感觉就是选择联合培养可能是一个比较好的方法。

吕梦娜： 是是，这样是一个合适的（方式）。那我在网站上看您也是硕士生导师，也指导过学生做研究。那您觉得您在指导学生做研究的过程中和您当时接受英国导师的这个指导，有什么区别吗？

受访者： 我可能会给的工作或者给的压力多一些吧，会比我之前的高啊。

吕梦娜： 确实，英国的老师那都不算特别有压力，是吧，至少相比于国内，那您觉得您和国内的一般的老师相比的话，会比较那种push型的吗？

受访者： 跟国内导师比的话我肯定不行了，就没有那么(push)...。

吕梦娜：对，明白。所以您在培养自己的学生的时候还是跟国内的这个气质比较相符哈

受访者： 我跟国外还是比较相符的，我觉得跟国外比较相符。

还有一个问题是什么呢，就是首先我特别在指导研究生的时候，我会去了解一下他未来的那个职业发展的规划。如果他要是打算进高校的话，那我可能会push的更多一些。但如果他觉得可能研究生读完以后他就要去公司或者去企业工作，我可能对学术的要求就会降低一些。

吕梦娜： 明白，还是做到了差异化的。

受访者： 对对对，还是要看他们未来发展方向吧。

吕梦娜：嗯嗯明白。回到刚刚那个话题，就是中国和英国商学院的这个差异嘛。您提到了英国对于年轻的老师可能前两年会教学偏向一些嘛，那中国的话您观察是什么样的现象呢？

受访者： 中国肯定一上来就是要有科研考核嘛

吕梦娜： 对，好像我们是颠倒的哈，还有老师说不需要教学都行，只要是看科研。

受访者： 对，因为像在国内，特别是教学它是一个基本要求嘛。就是你的工作量是基于你的教学课时，但是在考核的过程中，他考核科研还是非常重要的一个部分，如果你科研考核过不了的话，现在不都是非升即走嘛，就都是这样的情况。

吕梦娜： 对对，您刚刚也提到非升即走，我想知道您现在是这样的一个阶段吗？

受访者：对，我们也是，我们是六年。

吕梦娜：我听说好像政法大学是18年开始的，正好是您来的那一年

受访者： 对对，正好被我赶上了。

吕梦娜： 那您现在是已经通过了第一个考核期了，您现在感觉怎么样？这个有没有考核的压力？还是...

受访者： 对，因为现在其实我们学校有一个项目考核，就他要一个省部级的东西嘛，就是主持省部级的东西，但是怎么说呢？我自己是有一个教育部的产学研，但是这个吧，就是我们如果评职称的话是不算的，但考核的好像应该还是算吧。所以就基本上考核的话没有太大问题。但是评职称的话，我们肯定还是要国资国社或者是北京市、教育部啊类似这样的项目。

吕梦娜： 那可以理解为就是这个非升即走，大概是不太有走的这种生存的危险，但是可能评职称还是需要再满足另外的考核要求。

受访者： 对。

吕梦娜：明白。您对这个非升即走制度是怎么看待的？因为毕竟您也是执行这个制度的老师嘛，你是怎么看待这个制度的？

受访者： 这个事情我自己觉得这个事情比较复杂了。因为怎么说呢，就是我自己的感受是你如果作为一个做学术的学者的话，你真的想要出好的成绩，或者你想要做一些你觉得自己真的觉得有价值研究的话。我觉得卡时间的话，肯定就是会让你量多质少，你肯定是这么一个...但是呢现在怎么说，就是因为确实现在人才太多了，可能站在这个高层领导的角度而言，可能就是他们更需要急于出成绩吧？这个就是看你站在哪个角度去思考吧。

吕梦娜：嗯

受访者： 但是站在老师的角度肯定是觉得这是一个相当于让你量产的一个过程嘛，肯定是。

吕梦娜：嗯对，所以其实就是得看在什么样的一个立场上，然后才能去评价这个制度。

受访者： 对对，就是你真的是要看到站在什么样的立场。当然作为老师，就我们自己下来，可能同事之间可能会抱怨抱怨吧，但是确实是现在就是这么一个现状，就是没有办法。

吕梦娜：嗯嗯

刘书博老师： 哎，张老师。那我这里插着问一个问题。就是您觉得就比如说科研这种量化考核的方式它会对于科研学术工作产生一些负面性的影响吗？当然我们知道它会提升产出效率，那有没有一些除此之外的其他的结果和效果或者是一些负面的...？

受访者： 我觉得肯定会就是你为了提高量，我觉得肯定是降质嘛。

刘书博老师：但不是它还看这个期刊的等级嘛，它似乎就会觉得期刊的这个级别就是质量的代表。那您说的这个质量可能是另外的一个意思，对吗？

受访者： 对，就是我觉得首先怎么说...我们现在可能还是在一个求生存的阶段吧，还不到享受学术的阶段吧

刘书博老师： ok，所以就是其实学术这种您说的享受是比如说是做自己觉得有兴趣的研究课题？

受访者： 对，有兴趣研究课题，然后可能就不会有时间啊各方面的压力。你可能真的就能沉下心来去认真的去花一定的时间去做这个事情吧。

刘书博老师：嗯明白，ok，那我就插这一个问题，那梦娜你继续。

吕梦娜：哎，好嘞。

吕梦娜： 张老师刚刚也提到就是对于这个制度，可能作为老师，尤其是青年老师同事们可能会私下里面会稍微抱怨一下嘛，是不是对这个制度就是没有办法？就是我们作为青年老师只能去被动的接受，而不能说是提出一些改进意见呀，让学校再比如说放宽一些什么的？

受访者： 我觉得老师可能就是怎么说……，因为就是一个大势吧。我觉得可能对于老师而言，我们可能会在一些比如说在一些学术会上会提一些，但是其实只是我们自己的建议吧，但能起到多大作用的话就是很难说了。

吕梦娜： 是是，我感觉其实现在学校或者学院的治理方面大多是形式上征求意见，然后实质上就是比较专断一点。

受访者： 所以读博的时候就了解了这些东西，看看这个未来的情况就怎么发展吧。

吕梦娜： 对对，因为之前包括访谈其他老师也会提到嘛，像我们稍微年轻一点的，就是90后会比较反叛精神强一点的嘛，就是说可能未来我们可能不会再忍受这种剥削，有可能哈哈哈哈。

受访者： 哈哈，那可以呀，就是你不忍受剥削，就是你离开嘛，

吕梦娜： 是是是

受访者： 就是你很难改变环境，只能改变自己了。

吕梦娜： 刚刚提到中英商学院的这个差异吧，包括导师，包括管理方式，然后您觉得其他方面有没有差异？比如说这个范式，因为我经常会听到像英国留学回来的老师都会觉得范式上面会有一些冲突。因为英国可能会比较偏向于定性的。那您有这方面的感觉吗？

受访者： 我还好，因为我一直是做的定量。

吕梦娜： 在英国也是？

受访者： 对，我一直都是做的定量。

吕梦娜：导师也是吗？

受访者：对，他也是，他是做那个数字营销和信息系统吧，他其实更偏数据。

吕梦娜： 您当时在英国的那个学院的话，大家是一个什么趋势？

受访者： 我周围好像都是定量的。

吕梦娜： 那还好，那好像没有遇到很大的争执哈。

然后还有一个小问题，就是关于这个课题基金方面嘛，我想知道您在申请课题基金方面有没有遇到一些蛮意外的或者是觉得挺具有中国特色的一些事情的呢？

受访者： 就是在国内读博士的同事们，他们肯定就是会有导师包括像师兄师姐会帮他们看本子嘛，就会给他们提意见，就是他们有他们的圈子，像我们就比较吃亏嘛。

吕梦娜： 所以还是回到那个问题，我们中国的学术界，这个圈子文化还是挺明显的。包括这个指导学生也好，包括申请课题也好，感觉似乎每一个比较重要的环节都离不开这个圈子。

受访者：对，是的。

吕梦娜：然后假如我们现在可以改变一些东西的话，您觉得我们国家的这个商学院啊或者是管理学研究方面，您觉得哪些是最需要做出调整的？

受访者： 站在哪个层面而言呢？

吕梦娜： 可以在学院和学校。

受访者： 我觉得还是要有一个很好的环境去培养青年老师吧，就是比如说有一些相似的研究方向的老师愿意去共享吧。因为我自己的感受是，像在我们学院，特别因为我是工商管理系嘛，我们系的话做市场营销的老师就特别少，所以我基本上就是很难跟别人聊这些事情。所以我觉得还是要能够更合理高效的安排师资吧。

吕梦娜： 明白。我们现在读博士阶段的话，就能感觉到大家做研究其实更多是单打独斗，就是其实合作还是蛮少的。

之前访谈过一个老师，他的一个观点跟你很像，就是希望学院层面能够招一些研究方向比较相近的老师，然后大家共同的一块儿去做一些研究。然后张老师我这边的问题就到这里，主要就是问了一下关于中美商学院方面的一些差异，特别是具有中国特色的一些东西。然后你也给了我很好的意见和观点。

黄秋莉： 第一个问题，您提到您当初工作了几个月之后又去读博了，然后您非常坚定的选择学校嘛，您会觉得学校比较自由。那您现在也工作了几年了，您现在觉得自由的这种状态实现了吗？你是否还是会觉得自己当初的这种选择是对的呢？

受访者： 对，其实我到目前为止，我觉得我的选择还是对的。虽然说我刚刚开始的时候想的会比较简单，就可能我觉得高校可能会更加轻松一点吧。但是到目前为止，我觉得80%的满足了我对工作的预期吧。

黄秋莉： 就主要体现在哪些方面呢？我觉得可能老师们一个最大好处就是时间可能比较自由，不用坐班儿什么的。

受访者： 就是站在很多很多方面考虑的。第一，工作环境确实是很简单。我自己周围有很多年轻老师嘛，就是我们之间相处啊什么的，我觉得很轻松。我其他在行业里面干的那些同事们，可能会有一些职场的问题。

其次，我觉得就是一直在跟学生们接触吧，可能就是我作为老师而言，其实也能从学生身上学到很多，我觉得其实是互相学习。 然后也能够接触到现在年轻人最新的一些东西吧，心态会比较好。然后特别是我觉得也很有成就感。

当然其实大家也都觉得大学老师社会地位会比较好一些，就是受人尊重吧，这也是一个精神层面的一种满足吧。

除了工作方面，就是在家庭方面，其实我觉得特别是对于女性而言，我觉得高校老师还是一个很理想的一个工作。包括未来子女的教育上，这些方面其实我觉得都是有好处的。

黄秋莉： 是。刚才老师说在学校工作还是有非常大的自主安排的自由度的。但是同时我也听到很多老师说，做学术有一个不好的地方，就是这个工作和生活的边界不太明晰。你现在这个工作和生活能平衡吗？

受访者： 那肯定就不太明晰嘛。但是其实我觉得更多可能就是正常上班之外的那些时间，你自己写一些东西吧。然后还有就是跟学生沟通吧。其实我觉得这些也不是说会让我感觉不能接受或者压力很大、很厌烦的这种事情。因为毕竟比如说以后你在行业工作，就现在你像那些大厂，然后包括像大家可能会比较看好像这种金融行业，哪个行业不需要加班啊。这我觉得都是一个比较正常的现象吧。

黄秋莉： 对，相对来说学校老师是已经是非常好了。

受访者： 对对，其实就是看相对的嘛，肯定是看相对的。

黄秋莉： 然后我对老师刚开始说的印象比较深刻。就是好像您接受我们的访谈有一个重要的原因，就是从国外回来的老师回到国内之后会有一些问题和困扰嘛。您能给我具体的介绍一下吗？就是您自己从国外回到国内以后遇到的一些问题啊或者是不太适应的地方吧。

受访者： 在教学方面，更多的就是一个备课的压力吧。刚开始回来工作嘛，毕竟还是会有一些刚工作的压力，这个反正我觉得挺正常的。

然后其实给我带来的最大的困惑就是了解国内的这个科研体系和评价的一些体系，其实我刚回国的时候，就是大家说的什么c刊啊什么，这个其实我都不知道到底是什么东西，就是花了一段时间去了解。然后还有像刚开始国内说的所谓的项目，我也不太清楚是什么，就是在这个前期的摸索阶段，其实就是花了比较多的时间。所以可能在刚刚进入这个行业的时候，还是需要一段时间去适应。

黄秋莉： 您这个适应的一些途径的话，我能想象的可能就是跟这个周围的老师们请教一下。那您自己当初是怎么适应过来的呢？

受访者： 其实我还是跟各位老师请教。

黄秋莉：不知道您身边有没有从美国回来的老师呀？

受访者：也有啊。

黄秋莉：那您会觉得从美国回来的老师和咱们从英国回来的老师，他们的工作方式之类的呀，会不会有这种方面的差异呀？就可能个人也有，但是他们因为受教育的不同嘛，我不知道这种国别有没有带来一些差异呢？

受访者： 我觉得这个就是很难说，因为毕竟有限的嘛，它是一个体，所以我不能用他的个体代表整个国家的一个教育文化。我自己感觉更多的可能就是人的个性的问题，其实并不是说这个教育体系带来的问题啊。

还有一个问题就是在国外待的时间的长短，可能待的时间长一些的可能会更受影响，时间短一些的可能就不会太受影响。

黄秋莉： 您提到“跨文化”在我们国内来说就会显得有点大。那咱们国内这个市场营销研究的这个主流大概是一个什么样子？

受访者： 我不能说主流，就是可能现在的方向，更多的比如说像跟AI和大数据结合，然后还有像前段时间可能提的比较多，就像元宇宙、拟人化这些。可能现在还有那个绿色营销，这些方向未来可能会比较好一些。

黄秋莉：就是可能要比较时髦一点哈？

受访者： 我觉得更多的可能是因为像我们之前做营销的话，我们更多的是分析人嘛，就我们更多的是偏向分析心理、分析消费者啊。但现在更多的可能就是用数据了，那就需要用技术和营销相结合吧。

黄秋莉： 那老师您现在一般研究的这种idea是怎么获得的呢？就是您除了和自己教研室有一些沟通和交流以外，这个社交媒体是您自己的一个兴趣驱动以外，那这个研究的具体的idea，您一般是怎么涌现出来的呢？

受访者： 其实还是看文章吧，还是会看一些好的期刊的那些文章。

黄秋莉： 我们通常也会说这个文章要具有一定的创造性嘛，可能就是每个老师都会跟我们这样强调，那您觉得一篇文章的这种创造性呀，您自己是怎么把它凸显出来呢？就是有一些具体的套路或者是技巧嘛？

受访者： 其实我自己感受就是像营销，肯定是行业跑在了研究的前面。

那肯定是行业中出现了什么样的全新的现象，然后我们再通过理论数据去分析这个现象出现的原因，然后同时可能给他未来一个预测吧。所以我觉得可能更多的是要追踪于最新的营销趋势吧，更多的是现象结合理论吧。

我还会看一些行业报告，有一些咨询公司会出一些是行业的一些发展方向、发展趋势的这些报告，我也会关注一下。

然后还有一个方向就是现在提的比较多，就是交叉学科嘛。就看看有一些什么新的学科可以应用在营销里，其实之前用的比较多的是心理学嘛，现在可能更多的就是像计算机科学呀，然后包括我看有一些老师就是在做眼动、研究脑电波，其实这些也有老师做了，但是这个对设备要求比较高，就是看学校有没有这些东西了。

黄秋莉： 听起来挺高端大气的那种感觉。

刘书博老师： 我这边插问一个小问题，就是您觉得英国为代表的这个欧洲，他们在市场营销领域的研究跟一些美式的期刊，那这两个区域他们对于就是市场营销这种研究是不是有各自的特点呀？或者说您觉得没有，还是趋于一致的？

受访者： 我其实没有太关注这方面的问题。

刘书博老师： ok，ok，行，那我就这个问这个问题。

黄秋莉： 那我们要是想把文章发到国外的期刊上面，需要具备哪些条件呢？

受访者： 我觉得第一就是你肯定是要多看一下那个期刊近几年发的文章吧，看他们在哪些领域吧。

然后还有就是文章的范式肯定比较重要，然后语言是一个基础嘛。然后如果投国外的文章的话，国外的期刊的话可能就是区域性可能要稍微注意一下，就是数据这些的。

黄秋莉： 好的，那我以后也好好的注意一下。那对于您自己来说，去判断一个好的研究有没有相应的标准呀？

受访者： 我的标准就是我读完我有没有读下去的动力和我觉得他有没有意思。

黄秋莉：就是可能自己一看就能晓得哈。

受访者： 对，我首先看题目，我觉得他就很吸引人，我就会很想读。

黄秋莉： 然后就是想问一下，您不是也有指导一些硕士同学，然后你有没有遇到过就是特别适合做学术的的学生呢？

受访者： 我自己是没有，因为我是去前年刚评硕导嘛，我去年刚带第一个学生。但是我教研室有在读研二的时候已经发了很好文章的学生。就很厉害。但是其实他导师也特别着重的培养和给他压力，让他去做这些，他自己也很感兴趣。

黄秋莉： 那您觉得如果我们要成为一个学者的话，就是成为一个科研人员的话，以您的经历来看，有没有需要具备一些特别的特质呀。或者是这种什么性格呀上面有没有什么要求呀？或者是你觉得比较合适的，也不能说是要求吧。

受访者： 我觉得第一就是有那个好奇心吧，就会经常问问题，就是会有问题会质疑，我觉得这个是需要具备的。

然后还有就是有专注力，可以沉下心来，可以自己去孤独的去做这些东西，就是还是比较枯燥的一些工作。

还是就是你要感兴趣吧，如果不感兴趣的话，还是挺苦的这个东西。

还有像未来要做科研的话，特别是做社科管理学的话，我们的薪资可能刚开始还会比较低。所以说，就是我觉得还是需要一定的可以让你去做科研的一些经济基础吧。

黄秋莉： 还是要有一些的经济基础，就是做学术挣不了大钱。

受访者： 对，还是比较苦，特别是像社科类的老师刚开始。

然后还有就是有一定的人生规划吧，就是毕竟它是一个连续输出的一个过程嘛，不断学习，连续输出。比如说人生的一些重大事件可能会耽误一段时间，所以就是在规划方面还是我觉得也需要去考虑一下。

黄秋莉： 明白了，就是特别对我们女孩子来说，可能确实也是要考虑一下的。

受访者： 对，特别像现在高校不都是“非升即走”了嘛，如就我感觉如果女孩儿真的要从事这个的话，就还是要有一定的准备吧。

黄秋莉： 就是要面对这样的一个考核，压力还是挺大的。

受访者： 我觉得特别是在读书期间，还是要多多跟导师沟通。就是我感觉国内特别好的就是可能有一个圈子能够帮你们快速去适应，就一定要利用这个优势。

黄秋莉：明白了。 那回顾您的这个学术道路的话，您觉得对您影响最大的人是什么样子的？

受访者： 我觉得可能还是更多的是家庭教育吧。

黄秋莉：您做老师跟您自己的这个家庭环境是不是也比较有关系呀？就是可能是周边有人做大学老师或者是做知识工作什么的。

受访者： 我还真没有，我父母都不是做教育行业的啊。我感觉更多的可能是一个开放的家庭环境吧。然后包括其实像我很小的时候不是大学就出国了嘛。所以可能更多的还是一个独立思考问题的一个能力的培养的。

黄秋莉： 明白，明白。那就是您在英国读书嘛，然后您觉得自己在读博的时候有一些什么样的收获？可能知识上的收获是一方面吧，您觉得读博那段经历那段时光怎么样？

受访者： 其实除了学习方面哈，然后我觉得有两方面就是我的收获比较大。第一方面就是享受孤独，或者是说独立做一些事情的能力吧，就是培养的比较好。然后还有一方面就是欧洲每个国家都离得很近嘛，所以基本上我就是去很多地方旅游了，这也是我觉得一个比较好的经历。

黄秋莉： 对，那有没有你自己会觉得有一些比较遗憾的地方？

受访者： 我觉得遗憾的地方就是我的导师对我push的不够狠。可能就比较散漫，所以就导致了回国工作以后的压力可能就是成倍。

黄秋莉： 主要是现在大家不是达标性的这种考核吧，而是要跟自己的同同辈竞争吧，可能这个也有一定的关系。

受访者： 对对，就是到了一个这样的一个环境里，这个环境肯定会给你产生一定的压力嘛。

黄秋莉： 那到现在您有没有一些学术榜样在引领着你前行呢？

受访者： 就是我周围的同事们都是榜样。像跟我比较关系好的这几个同事，他们其实基本上都是博士出国或者是博士联合培养的。所以他们对国内的整个学术体系是非常熟悉的。然后其实他们也非常优秀，就是我感觉就是他们在这个行业里面可能做的更如鱼得水吧。

黄秋莉： 就是你会不会觉得这种对学术体系的一个了解程度也会影响到一个学者的发展呀？

受访者： 肯定会影响。

黄秋莉： 对这种学术体系规则的一个了解嘛，您觉得也会影响到学术人员的职业发展。您能给我具体的介绍一下吗？他在我们的工作当中有哪些具体的体现吗？

受访者： 比如说像你们读博的时候，可能你们就会了解到国内的高校的考核制度啊。然后你们会发展自己的学术圈儿，然后就会有一些潜在的合作者。同时你们可能也会在上学期间去参加各种会议，然后会认识跟你们相关的一些学者呀，包括像国内一些相同领域做的很好的一些知名学者吧。然后跟导师做项目这些，其实你们读书期间对这些都已经有一定的了解了，但是像我们的话是从开始工作的时候才开始做这些东西，所以其实基本上就是浪费很多时间去做这些本来其实在学校时间就应该做的事。

黄秋莉： 我们可能心里就是知道了，也就会有一定的预期了，就会去着手准备了，我明白你的意思了。

然后我想请问一下张老师，您觉得就是你什么样的学者可以算得上是一个比较合格的学者？有没有一个标准或者是就是百花齐放的都可？

受访者： 我觉得这个可能是比较个人吧，我自己是比较喜欢那种书香派的学者。

黄秋莉： 书香派的是一个什么样的学者呀？可能您提到书香派的，我就会想起刘老师，他会读非常多的书嘛，那您见到的这种书香派的老师是什么样子的？

受访者： 可能就是我们印象里面稍微有一些古板，对于知识的了解对他是最重要的事情吧。然后还有一些价值观念吧，可能因为我也见到过有一些非学院派的一些学者吧，他可能更向于像企业家那种类型的。就是实践派，可能他们会在行业中就是可能更多的就是会在销售方面更加擅长吧。我自己比较偏向那个书香派的这种。

刘书博老师：就是scholarly这个形容词对应的吧？就我们会说到他比较学究气，是这样吗？

受访者： 对，稍微有一点学究气，我比较喜欢这种的。

刘书博老师： 那我这边补充再问一下，您觉得我们管理学这种专业性，比如说评价说他专业能力很强，那您觉得如果这么评价他，是需要他达到什么样的一些标准吗？

受访者： 我觉得就是应该非常了解自己的研究领域吧，我可能还是看研究方面吧。

刘书博老师： 发表？

受访者： 对，发表肯定是一部分吧。我自己的经历是我接触的在行业工作的人比较多，就是我从小到大接触的人。所以我能够明显的感觉到，高校的一部分学者和这些人他是有一个很大的差别的。所以我就是比较偏向于书香派的。

黄秋莉： 那张老师您对自己的一个未来的职业规划和期待是什么呢？

受访者： 就是通过考核，解决职称问题。

黄秋莉： 那等你评上正教授以后呢？

受访者： 我觉得可能就是要去做我自己感兴趣的事儿吧，回到我感兴趣的领域。

黄秋莉： 您是会继续深耕在这个社交媒体，还是会回到这种跨文化研究？或者可能又有变化什么的吗？

受访者： 我自己其实对跨文化比较感兴趣。但是未来我觉得就是把一些计算机技术和营销相结合，我觉得我自己也还比较感兴趣。就是像机器人工智能，包括像一些计算机技术，其实这些的壁垒还是比较高的，如果作为一个完全学社科的想要去对学习这方面，我觉得还是需要花费大量的精力的。我现在可能就是没有那个精力去重新去学习这些。

黄秋莉： 那我在您的那个网页介绍上，您还承担了这个学院的一些行政工作，这个会对您日常的科研啊什么的有影响吗？还是说是一个相互促进的一个过程？

受访者： 行政工作肯定会影响，因为在做科研的时候，你肯定是需要一个专门的时间去专注的做。但是行政工作就很碎片化，它就是其实很容易打断你做科研的这个过程。

黄秋莉： 那你以后会想要说“学而优则仕”吗？会想着说在行政方面更进一步吗？

受访者： 没有，我完全没有这个想法，我对做行政工作也没有兴趣。

黄秋莉： 明白了，可能现在就是一个经历。

受访者： 对，就是一个经历。可能跟每个人的职业规划不一样吧，我可能没有什么这个走仕途的野心和欲望。

黄秋莉： 那我想再问最后一个问题，我就听你聊天嘛，就有种岁月静好的感觉。那对你自己而言，就是在这种学术工作过程中有没有让你觉得比较难熬呀？让你觉得困难的时候呢？

受访者： 就是被拒的时候有点困难。

黄秋莉： 明白了，那我没有什么别的问题了，非常谢谢张老师。

刘书博老师：那我觉得时间也差不多了，这个最后还是特别感谢张老师，在这个休息的时间还接受我们的访谈。可能这两天疫情形势又吃紧了，等一段时间或者五一放假，我约大家一块儿来坐一坐。

受访者： 反正我们都在北京。

刘书博老师： 那我们后续把文字稿誊出来也发您，那今天我们先到这里。

# 受访者4

受访者：就按部就班的，其实像你说的就是他也一直在岸上，他就差不多也就混过去了。

其实我觉得很绝大多数老师还是有一些学术理想或者报复的。但是在这个制度下他的的确确面临着一个要被考核、被评价的竞争的压力。如果说我们想做一些比较长期的一些研究，特别是做比较偏质性的、人文的一些研究，可能更需要时间比较久。而且现在你看光看论文，他其实著作就是看你发的什么级别的期刊的论文。这个评价体系说是代表作啊，就我们学校目前的这个评价来说的话，有代表作评价，但实际上还是有很多这种数数的呀。

我之前看应星有一篇文章，应星就讲中国的“学父”，就是说这些老师把学术作为自己利益的一个工具。

我不知道在管理学领域怎么样，但是我觉得在教育学里面那种山头鼎立的局面还是很严重

刘书博老师：我们也是。

受访者：我看到有一些年轻老师要想申课题，就必须混到某个圈子里去，或者依附于某一个老师。那这样的话学术自主性就很明显的（削弱）。很多老师其实就是做只做十年学术，然后很多资历深的老师年轻的时候有一些代表性的东西，慢慢好像就成了一个权威，但是其学术产出有的时候反而成了这个学科发展的一些阻碍，后面的人就很难有发展空间。

刘书博老师： 对，是的，我们管理学也是这样子，虽然发展的历史不是很长，但是就是很多山头林立、学阀横行。我前几天还跟一个比较岁数比较大的、60后的一一个老师聊，然后他可能见的比较多，他个人总结了一下。他说相比于他见到的美国的那些学者，中国的学者呈现出的一个特色就是好像身体精神状况不是很够。大概就是到了一定年纪就干不动学术了，他就躺平了。然后我跟他说不是身体问题，可能是前面他是一种为稻粱谋的态度，然后那他已经拿到了这些资源，那干嘛还要继续的努力呢？那美国可能还是会兴趣为导向的以学术为业，所以他会持续很长时间嘛。所以我们经常看到一些到了50岁、甚至不到50岁的老师后面不再有真正的价值产出了。

我觉得你书上写的传统文化对于读书人的影响其实也挺重要。那您觉得除了这些文化，有没有比如说大学治理方式、管理模式会导致目前的一些问题？

受访者：我感觉我们从小到大社会化的过程中，沾染了很多不求真的东西吧。就是我觉得跟我们这个文化、政治等制度性的东西可能有关系。我们做学术，“吾爱吾师，吾更爱真理”，但我们那种学术热情，我觉得还是会很容易被浇灭。就比如说对大学来说的话，有些制度我觉得是非常不利于去培养学术人才的。他培养的只是服从于规则的人，就是在这个规则下最大程度的去取得利益的人，比如说这个保研，就特别的积分制嘛。最后大家刚上大一的时候好多同学就特别关心这个。

刘书博老师：我看我们中财各种评比，就要参加好多学生活动呀什么的，然后就这个分数体系就建立起来了。就现在有人会认为这是一种制度的进步，对于人的评价更加的细致。

那可能我们刚才讨论的似乎并不是一种进步，我们总是在强调制度建设，但制度建设的理念有没有副作用，好像很少有人去关注。尤其现在每年都要有培养方案的修正，教育部要求每年都要修正，那为什么每年都要修正？然后修正之后效果怎么样？也没有人过问，只不过是要求你每年修正，最后就变成了一种形式主义的工作。

受访者：我觉得我们在制度设计的时候，很多时候考虑的一个因素是积分嘛，按照这个工分制好像是很公平公正的。但是其实公平公正有很多种办法，而且这种公平公正呢很容易导向极端的精致利己主义、而且是精致的计算。很多同学其实他内心也是有点厌恶的。

比如说我们这边有些规定简直就是特别的不合理啊，比如说你只能加入学校的八大社团，这八大社团是加分的，但是其他小社团或者是一些兴趣类的社团就不行，那就把学生的兴趣都要限制啊。那一个学生以后做学术，他从里面脱颖而出保研了，然后后面做学术了。那整个人的这个天性啊、创造力都被压抑了。这个东西就让我们每个人都会束缚住的。

刘书博老师： 甚至我会觉得在分数机制筛选出来的特别会算的、特别乖巧的那种学生。他可能会放大他的那种理念，然后把他的这种理念给再传递给、强化式传递给自己的学生。

然后像我们现在的大学老师，他会进入到大学的管理体系，那我们选拔管理者的时候也是只看这种标准嘛。所以这个体系、这个制度建设就朝着单一的维度去发展。所以我比较担心的是这样的问题。就是我们会认为学而优则仕，那“优”的判断可能就是比较单维度、有问题的判断，进而他会复制这样的制度，甚至强化这样的制度。

受访者： 对，其实人被压抑了之后，比如说一个学生吧，如果在老师这儿他感受到的是老师是非常的压榨或者是剥削的，或者是对学术非常功利的态度的话。学生其实有的时候一方面可能自己对学术就没有什么憧憬了，然后另一方面如果他还想自己做学术的话，有可能以后对学生会办变本加厉，就是媳妇熬成婆了这种感觉

刘书博老师： 是的，那现在我感觉好像这个趋势越来越明显，像我们学院，就是有很多老师做学问等同于发论文，然后每年能发十几篇，然后他就搭建一个学术流水线，我们访谈的时候他也会坦然的说，生产就是垃圾，就是灌水。甚至我导师都会说这些都是没价值的。但是他要上岸啊，他可能就是通过这样的方式把工作做到极致，进而能够在评价标准当中突出自己。

像人文学科的价值不能够被明显的彰显出来，那似乎就被边缘化。那对于这种边缘化的趋势，我们是有办法去进行所谓的抵抗嘛。

受访者： 我觉得这个可能一方面是有个人的坚持吧，然后更广泛的来说，其实个人在这个方面力量是非常有限的。因为这个制度设计规则在这儿，你不按这个规则玩很容易就把自己玩死了。

我觉得国家在科研体系评价方面，内在是有张力和矛盾的。就是一方面是要破五维啊，是要防止评价成了一种数字游戏，其实没有真正有价值的学术成果产出。但是呢，另一方面在偏人文的学科里面，经常是被警惕的。我之前看到网上有一个规定上写的就是期刊发文都要优先省部级或者是国家级的课题。就是说人文学科方向有非常强烈的国家取向。因为课题都是提前拟好的，就跟选择题似的，列出了好多选题。

但是其实人文学科跟理工科不太一样，对于理工科来说，国家确实可以做顶层设计，就说哪些是我们国家目前比较需要前沿发展的领域，我们需要重点来投入。但是人文学科的想象力或者内在的创造力应该是来自于没有约束、就是思想自由嘛，本来就是一个关于思想的学问，还是保持一种批判的态度。但是这些东西，不一定是当政者喜欢的嘛。因为我本科学社会工作，然后这个社会学就是个讨人嫌的学科嘛哈哈，它不是说要去唱赞歌啊。所以做比较偏人文性或者有批判性的一些研究的话，很多时候国家还是希望把它统合到一个大的体系里面。但其实有些东西不用统合，不统合反而会让这个学科发展的非常好，让古今中外或者一切可以用的资源都可以生成真正的思想。要不然我们人文学科的发展还是会受到很多的这个限制。

就包括我之前也是有一个乌龙啊，就是我这本书本来印的就非常少，就印了几百本吧，然后后来就卖光了。我就看到有一个微信群里一个同事发给我的，就说有人在传这本书被封杀了，就哪都买不到了。当然这是一个乌龙啊，是因为那个出版社脱销了。但是就这么一个事情，大家真的有人信啊，你想就这样一本书如果说它都被封杀的话，那就很难想象什么样的东西才能够出版。

刘书博老师： 那就成样板戏了，那就样板的东西才能够出版呗。

受访者： 对呀，那我觉得我们思想就不可能有活力了，那我们生活在一个什么样的时代呢？这个时代就对做人文性或者批判性的学者就太不友好了。那只能是大家天天窝在家里水一水无关痛痒的东西就算了。

刘书博老师：对对，是的，是的。尤其是人文社科，要有一种桀骜不驯的精神气质，或者说所谓的自主性吧。那现在我们是感觉很多知识生产是需要自我审查的、批判性不足。尤其管理学，我们几乎都是站在效率至上这边、管理者资本家这一边。像前一段热议的系统对于人的压榨啊，我们管理学的视角就是说算法特别好，就有一种颂扬的，怎么去让算法发挥最大的对于人的管理。但是几乎没有中国的管理学者看被管理者、看劳动人民。那在欧洲这一块儿是有很多研究者都关注的，他们会有所谓的批判管理研究，但是中国管理学这一块儿是零。然后我前面也是参加一个管理学会议，我去提说能不能讲批判管理主义，那他们就说批判这个事情其实是敏感的，最好不要讲。我就感受到我们不能批判。虽然马克思很批判，然后我们又是把马克思看做是信仰，但是我们不能批判，这一点就很糟糕嘛。

受访者： 所以这个真的是有一点让人灰心的事情啊。但是像您这边其实也还好，就是有些东西也可以拿到国际上发表。像我们国内教育学也还好，但确实也有这方面的限制，比如说有些词啊，反正有一段时间就是“公民”都不太让提。那你说中华人民共和国公民，这宪法都要这么写吧，然后公民教育却不能这么提，那就有很多意识形态的考虑。其实我觉得是一种很不自信的表现吧，我们天天讲这几个自信，至少思想上要自信嘛，对吧？对自己的发展道路应该是保持一个开放性的态度，要不然最后就只能是自己说自己好，那就没什么意思了。

刘书博老师： 我就想到那个詹姆斯·斯科特，也是人类学家吧。他写的《国家的视角》讲的就是国家有一个宏大的、向好的、理性的计划。但是有时候挺自负的，自己想的跟现实还是有距离的。反倒是说我们去让一片森林繁荣，那你不要太改造它，你要关注它的生态系统发展，那生态系统土壤比较肥沃的时候，它自然就会繁荣。那现在我们似乎总是想这边切一块儿，那边嫁接一块儿，最后生长出来很多橘生淮北的感觉。

我最后还是加一个问题，就是程老师您平常学习写作这方面是有一些自己的方法吗？就是怎么样去能够让自己的写作变得非常的既优美、又具有学术的那种严谨啊

受访者：有时候我觉得自己写的东西比较空泛，或者是比较虚妄，或者是比较矫情，经常是对文字有这种感觉。那个博士论文还是经过很长一段时间的凝练修改，然后后面包括我导师也帮我整体上做了很多细致的修改。所以我感觉这个写作确实是一个需要长期去做的事情。

而且写作我觉得很重要一个是要言之有物吧，就是我感觉现在我读到很多东西都很学术文化。但是读了之后感觉他对我没有什么触动或者启发，就我没觉得他说出了什么东西，这个东西就比较不好。

如果是想做社会学、人类学的话，社会学、人类学这些经典的作品可能需要看一看。像我自己学这个，像费孝通的《乡土中国》现在已经进入到初高中的教材里了，就是他对于中国社会的洞察，还有对一些理论性东西的使用，我觉得是特别值得我们学习的，但这个东西又不是轻易能学到的。

还有一个我想最引导性的问题，就是我们自己的问题呀。还是要有自己真实的困惑、真实的问题、就和自己有关的东西。所以如果写东西的话，特别是毕业论文那种缘起的地方，其实很多同学有的时候会觉得不太重要。就觉得哎呀，那我就提出问题，我好好的论述文献，然后方法，然后结论发现。但其实对我来说的话，我会特别看重一个同学写的研究缘起，我觉得这个东西写好了后面才能成立。如果这个东西写不好，你不能让读者进入到你自己见过的问题域的话，其实后面很多东西有的时候就总感觉不太对。所以研究缘起是带着读者进入到你的那个独特的看问题的视角里去，如果读不进去，那后面就越读越读不进去。这是我的一个感受啊。

刘书博老师：您说这个就是缘起啊，其实我经常也跟同学们讲，就研究问题的引出也是你的研究动力和激情的一个来源吧。我觉得管理学最要命的问题除了范式的单一化和美式的霸权后皈依者狂热。还有就是我们的研究问题距离自己的生活太过遥远。学生在本科时候就研究董事会的决策、公司的治理模式、跨国企业在海外的投资决策等等。他们完全是从文献到文献，对于现实没有任何的感知。在这种情况下就比葫芦画瓢，然后做出来的东西呢，我觉得是两看相厌啊，老师看起来很头疼，他自己做起来很痛苦。最后就产出了非常多的精致的废话。现在我们本科写毕业论文百分之九十就是实证研究了，很恐怖的一个事情。但是现在我们这个范式导向让学生在本科阶段就只是完成一个比葫芦画瓢的工作。导致他对于研究的看法也非常的不正确，更不要谈研究的激情和兴趣了。所以我非常赞同程老师说的，我们在做研究肯定还是要带着一个激情、好奇心，然后才能够持续的把一些东西给挖掘出来。

吕梦娜： 程老师，刚刚我们说到这个研究缘起，我感觉特别有体会。因为我这个专业是企业管理，我一个没有去过企业的人去研究企业管理，就是这种论文，尤其是同学们写的论文，明显能感觉到隔了一层。就是这个问题，你再怎么引人入胜，感觉有点像假案例的感觉。但是做我们这个企业管理的，尤其是没有接触过实践的、没有进入过田野的人，就会觉得好像犹犹豫豫的不太能够接受你的这个观点。然后说到这个田野，我就很好奇，您之前去高中、初中做过这个田野嘛，去了很长一段时间，我就想知道在这个过程中你有没有遇到过到现在感触还比较深的事情？

受访者： 像你做的是这个企业方面的研究的话，要想做比较偏社会学、人类学，不做那么多这个假的、或者空的、远的这种研究的话，其实到企业里去就是一个比较好的办法。你就到一个企业里去嘛，比如说你研究某个类型的企业，那你就真的像人类学那种在里面以某种身份进去。我以前读过那个潘毅的，因为她是做劳工社会学，当然她去的那个企业可能都是制造业，都是流水线。但她主要是从人的角度去关心。这也是一种很好的方式吧，不一定都是研究董事会，都是研究那个很大的东西，可以从小的地方入手，大的东西一起考虑。

然后我自己做田野研究，比较深刻的事情，其实还是有挺多的。比如说我开始做探索的时候，先去这个农村的学校，所谓的这个成绩好的学生，大多数还是跟我这样一个外来的保持距离的。但是反而是那些不怎么学习的，平时就喜欢各种惹是生非，各种在学校里打闹、玩耍打游戏的、谈恋爱的这些同学，他反而会更主动的接近你，然后他们的也会做更多让你觉得有意思的事情。但是那些好学生是比较神圣的，他即使对你有好奇，他也不会有那种很热情的感觉。像我后来做访谈的话，我会感觉到人讲自己经历、诉说自己的这种愿望还是比我们想象的要大。就是我们有时候会觉得，哎呀，别人可能不好意思说，或者都不会愿意跟我说，然后怎么怎么样。但是其实每个人在合宜的情况下会愿意去讲自己的事情，也会有这种讲述的意愿。这些东西他不讲，它也会存在，但是它不是以有形的方式存在，他以一种不可见的方式存在，而我们很多时候还是希望能把这些不可见的变得可见。

就我们做社会学、教育学、管理学，很多时候你看做企业研究也是这个企业的经验嘛，企业的经验其实也是很多企业中的人的经验、人的生活、人的故事。所以最终还是要做偏质性的、偏人文的研究，还是要把这个故事讲清楚，把那个故事的复杂性体现出来。

吕梦娜： 对，我觉得其实大家能够有这种写作欲望，一个是本身有意愿，另一个我觉得您给出的这个激励也是蛮棒的。就是像我们做这个企业方面的研究，可能会做问卷吧，也会有一些“小恩小惠”吧。但是我觉得那个东西对他好像没有特别大的吸引力，但是您当时提到说，包括自己的自传可能会分享给大家，包括给导师的书。如果说我是这个被访的，我也会很愿意去分享。我觉得这是一个互惠的一件事情，我也能从这个过程当中学到很多。

那您当时提到了在田野的过程中，其实“家伙们”会跟您有更多的接触嘛。在这样的过程，你内心有没有什么变动？比如说因为您读过那个《学做工》那本书，可能大概能够预测说，这些同学未来会子承父业嘛。所以说您当时跟他们交流的过程中有没有一些内心的波动？

受访者： 刚才你说的意思是说我看到这些田野里的这些所谓的“家伙们”和这个威利斯笔下的“家伙们”有什么区别，是吗？

吕梦娜： 就是您观察到这些人的时候，包括您之前读的那本书嘛，然后您内心有什么感触？就是我自己代入的话，那看到他们虽然现在还很天真浪漫，然后生活很丰富多彩，但是可能会为他们后面的这个生活有一点触动，我是这么觉得哈，不知道您当时是怎么想的？

受访者： 因为当时其实去到那之后，中国的情景和英国的情景还不太一样，像有一些在中学的同学，他有时候还是很想要努力，但是又处于那种比如说某个学科特别差，比如说英语，我当时甚至还买过这个英语卷子去给他们，想要给他们补补课之类的。

但是像有些同学他就是完全放弃的，但这些同学确实也是没办法在学校里获得认可、获得意义，他确实是在那混日子，他也不一定就是为了抵制某些更宏大的所谓的阶级之类的。其实这些同学他们的未来也不一定就不好，因为他们有的家里做生意，有的以后也许会去学一个技术。像现在整个城镇化的过程之中，他们很多人还是可以在县城买房子，然后过上（好一点的生活），有人去工厂打工，但是也不一定就是这样。就是他们的未来也是比较多样的，但是它主要还是依赖于整个国家经济形势比较好。特别是近一两年吧，因为疫情加各种原因，反正我感觉各行各业的人，特别是在比较做不太稳定工作的，需要特别依赖这个国家经济的人，还过得挺艰难的。

吕梦娜： 了解。那“家伙们”比较能够映入你的眼帘嘛，那可能像“读书的料”会稍微默默无闻一些，但是您其实研究的是后者，您当时这个角度是怎么有意识的去聚焦过去的？

受访者：我开始就带着这样一个问题去的，所以我就是想要去关注这些孩子。但是其实这个田野做的也很失败嘛，后来就变成了这个成长叙事了。

吕梦娜： 了解，好的陈老师，因为时间的原因，咱们争取线下能够有更多交流。

黄秋莉：陈老师您好，就是我看到《读书的料》嘛，然后我就想起来我们以前同学的一个经历，就是我们也是从村里出来的，然后他高中非常的努力，可能就有这种您说的文化再生产的过程，他有自己一个先赋性的努力呀等等。但是他到大学之后，他就可能觉得自己的高中太苦了，然后就自己给自己放松了很长的一段时间。

然后就让我联想到刚刚您和刘老师提到的第一个问题，就是我们现在的这个学术场域中有很多“只做十年学术”的这样的一类学者吧。就是刚刚您可能和刘老师提到了很多这种社会层面、体系层面的原因，包括他从小接受的教育制度呀，或者是我们这种评价考核，还有这个“占据山头”。那就是想说据您的观察，还有没有别的原因，就导致了很多学者“只做十年学术”？

受访者：我觉得不光是做学术，整个社会都有一种这个功利化的一种文化的蔓延。其实我们传统文化里面，读书人或者说知识分子还是有一种就是道统的这个传统的。但是我觉得像在我们这样一个国家，经过那么多次的政治运动……

其实我觉得很多时候还是这个制度本身不够。就是大家不是每个人都想说什么、随便说什么都可以，这个制度本身就是这种在某些时期比较好，在某些时期就不好，他就有比较大的这个不确定性。然后每个人还是有一点“自我审查”，就自己给自己约束了。就慢慢的我觉得这个自我审查的东西、敏感的东西太多了，我们的语言就会越来越贫瘠，我们的这个想法也都会自我设限吧。而且这个东西有时候也不是说是从上面来的，就是慢慢的，人们自己就已经在心里结成了这种自我审查的机制。

然后学者的功利化，其实也跟各种各样的评价、绩效主义的这种管理有关。很多评价的情况下，他没有说去培养一个人的长久的热情。刚才刘老师说的特别好，就是说这个土壤建好了就行了，不是说还是要从下面拖着、从上面压着。有很多时候还是从上面来压着，总是来评价你，或者是催促你，来考核你、监督你。他不是说给你提供一个支持性的土壤，然后尽量少的去干扰你，甚至说这个土都空了，他还是要抓着你往上走，那这个后来长出来的就不可能。好。

黄秋莉：刚刚您提到可能以前的学者会好一点，因为我自己看的话，我就会觉得像钱理群老师呀，或者是像费孝通老师他们这种，都是有一种终身学习的志向在的。那您在现实生活中有没有看到一直做学术、一直对学术保持激情的这样的一些老师啊？

受访者： 那肯定是有的呀，还是有不少老师是我觉得挺有激情或者还是比较有学术坚守的吧。就还是想做真正的学术研究，不是说只是为了某种外在的东西去做，我觉得还是有不少的。但是的的确确就是这个分化也很严重。

刘书博老师： 就这个分化，我觉得其实我们应该把它写成英文的论文，我觉得西方学者似乎没有看到这个。但我不知道教育学领域怎么样？像我们有一个组织行为的这个organization study，还有human relations这两本，是我们管理学的顶刊。像human relations他有很多对于商学院老师的这个研究，但是他这个背景都是在欧洲，我看美国的也很少，主要是欧洲的学者在做，那似乎我们也可以去看一看中国的学者，然后描述一下，让世界认识认识。

黄秋莉： 对。其实我们自己在访谈的过程中，我就会发现好像跟那个布迪厄提到的一样，如果你有更多的资本的话，可能就会有利于你去抵抗住这种评价、考核或者是土壤带给你的影响。比如家里有钱呀，当然这可能只是一个方面。

受访者：当你就是不太需要通过学术来获得物质上的回馈，或者也不太需要通过学术一定要得到某些人的承认，就像某个阶段，或者有的大学是非升即走嘛，那如果我不需要这些，我就自己想做什么就做什么。我如果是一个有学术热情的人，即使我的学术研究的主题或者是我的研究范式不太得到承认，那我还是能在这里生活下去，就我还可以有这样一个学术职位，这是一个最基本的东西。如果这个东西能保障的话，就是说其实很重要的还是学术共同体内部的一个声誉的评价。我想国外很多学者他其实很在意自己的这个学术声誉。

当然这个外在的评价，外在的什么奖和荣誉啊也是一个方面，但是同行评价（对他而言更重要），别人觉得你的研究怎么样，或者别人觉得，哎呀，你这些年就没什么研究，或者说你的研究整个就变味了，或者是就那这个东西大家也都会在意你在意这个学术声誉，那这个就不是三年聘期或者是发某个期刊有多少钱。自己还想做出真正能被学界所承认的东西，这个东西我觉得是维护和真正建设学术共同体，而不是把学术共同体搞得乌烟瘴气的。特别是我们的很多期刊，如果它变成某个人的一个私人物品，或者是一个小圈子的一个自留地了，那对整个学术圈就是一个很大的破坏。因为你就迫使这个学者他必须要去讨好、巴结，或者是怎么样才能发表某个类型的文章。那这个学术共同体还有什么声誉可言呢？如果是这样一个状态的话，大家就会变得山头主义嘛，变得圈地文化，然后就完全没有把学术作为一个公共的事情，它的公共性就被忽略了。

黄秋莉： 就我自己的观察的话，我会觉得像欧洲回来的一些学者哈，或者是以前在企业工作过的很多学者，他们对学术的这种热爱好像就是会比较持久一点。

受访者： 有可能，但这个我不知道是不是有大量的访谈或者大量的数据的证实。但是怎么说呢？可能在不同的学术文化下成长，自己对学术的这个热情还是会有差别，他对学术的理解本身就会有差别，所以这个你说的这个有可能是有区别的。

黄秋莉： 就是影响他们对学术的一个理解，可能造成了他们对学术热情的不一样。

受访者： 我感觉不管在哪种学术文化背景下成长，可能很重要的还是你能感受到它的魅力，就是你能感受到你做的事情是一个有意义的事情，你能感受到它跟社会公益，跟整个社会甚至说跟整个国家的某些公共性的事业是相关的，就是这种感召吧。所以像韦伯说的这个学术作为志业，不管你在哪个地方，你能有这种感召，那可能你的学术热情就会比较久。

而且你在现实生活中你能感受到你的这些热情还是有同道中人，就不是说别人都是很功利化的，让你自己一个人这样，自己一个人真的还是挺难去长期的坚持的吧。最后就自己，只能变得非常压抑，因为你得不到任何人的认可，那这个过程就是憋着憋着。所以这样一个属于公共性的东西还是需要所有人一起来维护吧。

刘书博老师： 有一个非常大的意义体系，就是你做的事儿不是单独为自己，而且你可以链接到人类共同体的更宏大的意义。

黄秋莉： 然后第二个问题，就是我看到您提到“懂事”，我自己的感觉是我经常听到这个话，如果家长夸一个小孩儿比较好的话，就是说这个小孩儿好懂事儿。那除了“读书的料”在他获得学术成就的过程当中有这样一个懂事儿的因素，在我们现在大学当中有没有看到这种比较懂事的现象啊？或者“懂事”的内涵，我看您写的就是说能看到不合理的当中的一个合理性，那除了这一点之外，这个懂事的内涵有没有一个扩充呀或者是什么的？

受访者： 你说师生关系吗？

黄秋莉： 就懂事儿这个内涵来说吧。

受访者： 这个内涵我觉得它在不同的情境下是不一样的。比如老师有的也会说，哎呀，这个学生比较懂事。我说那个懂事还是在这个家庭生活中的。你刚才说的那个能看到不合理当中的合理，它主要讲的还是一种理解，接近同情式的理解，他能够去看到观念背后更多的东西。比如说我们观念不一样，然后我能够理解你观念形成的原因，就是你为什么会有这样的观念，而不是只是觉得我们不是同道中人，甚至就是敌人那种，或者就是完全不理解它的一种认知。

黄秋莉： 对。就像我们村里夸一个人懂事的话，有一个场景就是说如果你想买一个比较贵的东西，然后家长说哎呀没有那么多钱，然后你就说好吧。

受访者： 那这也是“懂事”，就是说更进一步的场景就是他根本就不会提。

黄秋莉： 当一个学生进入到大学以后，他这种懂事儿会不会就会体现出一种精致的利己导向？

受访者： 这两个还是完全在不同的情境下来说的，因为在家庭里面这种懂事还是一种利他导向的，但是从家庭的私领域进入到公共领域，那这种懂事他会有什么样的一个演变？一般来说的话，他在家庭中关心他人或者体谅他人，是在公领域道德的一个基础。我们中国人讲推己及人嘛，就是如果你对自己的家人不好，也很难说你就能对别人好。但是你确实对家人好，这是不是就意味着你能能把这种私领域的这个道德或者对大人的体谅和关心能够推广？那也不一定。因为有的人他是非常在意家人的利益，但是他到公共领域，他就是非常的就是家庭主义，他就对我好，我就什么都能干。

刘书博老师： 差序格局嘛，就中国人首先得满足身边亲人的，然后再扩展。所以可能亲人都满足不了他，更不要说扩展。

黄秋莉： 我感觉公共话语的懂事儿就是和私人当中完全是不太一样的，就是在私人领域能接受不合理（比如接受不去买一个自己非常喜欢但是买不起的东西），在工作领域它会不会变成一种对标准的非常清晰的认知，然后去服从这种标准？

受访者： 在公领域的话，很多时候你比如说有的在单位里面，有的时候大家也会说，哎呀，谁谁谁他就不懂事儿，或者他可能乱说话，对吧？那个懂事儿和家庭中的懂事儿指向是不同的。

黄秋莉： 好的，那我没别的问题了，谢谢程老师。

刘书博老师： 那今天大家线上交流了好多维度问题，我受益匪浅，也很开心。我认识的一个人，他其实不是典型的读书的料，他只是在高中好好读书，前面非常调皮，后来考上的是西安财经，但是他后来在工作就还是比较顺利的，也不是说顺利吧，就比较幸运吧，也比较能吃苦，现在就是在做一个企业的联合创始人和管理者。那他的这种心路似乎跟在学校当老师的这个寒门子弟的心路又有一些不同，包括像刘强东这样的。如果能够了解他们的这种内心世界，我觉得还是挺有意思的一件事情。所以我们看这个未来如果有机会的话，还是能够在线下大家一起交流。

受访者： 那太好了。

# 受访者5

受访者： 你是不是要先介绍一下这是干嘛的

刘书博老师： 我们研究主题呢有一个大的理论背景，就是专业社会学。那可能再细一点儿的就是管理学学科，还有商学院对应的一些管理呀，制度呀，它在中国的环境下有什么特点，可能映射的就是中国的高等教育制度。所以我们这一块儿可能对话的文献就是知识社会学、专业社会学，还有就是可能未来会看教育研究呀，这些期刊。可能也有Human Relations，因为涉及到我们作为大学老师的这种管理方式、身份建构呀，还有就是一些外来制度对于我们管理上的一些影响等等这些。

那我们问的问题大概三个维度。第一个是我来问的，就是对于知识工作者、大学老师的一个专业化过程。就是比如说求学，你的成长经历。还会涉及到比如说我们学术标准，我们学科的知识标准啊，我们做学术需要注意什么，还有就是商学院的一些日常管理，包括中国特色的管理，那我就先问。

王老师是我们非常优秀的，而且我觉得是我们现在80后群体啊非常具有代表性的、在管理学领域做的非常好的一位老师。所以我们就非常好奇王老师是不是很早就奠定了或者确定了你当时要做学术的职业发展方向？那有没有一些重要的影响人物或者是有一些比较具有启发性的经历会引导你走上这条道。

受访者：我觉得你要说是有没有规划，说实在的好像是没有什么规划。那我是从小呢基本上别人都觉得我特别适合当老师，就是从各个方面的反馈都觉得比如小时候是很安静的，然后呢不怎么调皮捣蛋，然后呢上学呢也是相对来说比较沉稳的这种，就没有什么特别大的离经叛道的感觉。

然后上大学我是本科学工商管理的，当时在武汉嘛。后来呢有一个读双学位的机会，就当时武汉七所211高校联合办学，当时很多同学就去选武大华科的那些专业。当时呢我是选了心理学，华中师范大学的心理学，其实叫做应用心理学，但那个时候它叫心理学，是在他们的心理学院，相当于同时修两个学位。那个时候我印象中应该是大三读的双学位。

然后我不记得是前后关系是什么了哈，应该是在大三的时候我们有一门课叫组织行为学，那个时候那个老师叫XX。因为我们学院的工商管理学就什么都包括的，组织行为学也是一门必修，在那之前也学过什么市场营销、战略啊，我可能没有特别大的兴趣。然后组织心理学这门课呢我觉得诶还挺有意思的。然后那那个老师讲的其实不是特别好，当时用的也是罗宾斯那本书，后来N多年之后，大概十几年之后，我翻译罗宾斯第16版的时候，觉得是一个特别好的阴差阳错嘛。当时没想那么多，然后那本书呢也是孙老师翻译的，当时呢也就正常的学。然后我觉得诶那里面那些东西我觉得挺感兴趣的，再加上我当时读心理学的双学位，然后就建立了一个联系，那门课我就考了99分还是98分，是我大学所有专业课考的最高的分。然后那门课学完之后紧接着就开始准备要保研了。

受访者： 保研当时呢其实也没想着说要保什么专业。当时呢也是想着在武汉。因为当时我数学不是特别好，上大学的时候，所以呢我觉得考研肯定我也不太能考得上。然后我就想着保研，那个时候其实成绩也没有那么的好。但是最后还有两个学期还是一个学期，然后呢我就把成绩好好地提了一下。然后呢就达到了保研的那个条件。然后我当时就也是一个无意中的机会，别人告诉我可以读人力资源。那个时候我才知道人民大学的劳动人事学院，其实在保研之前我是不知道这个学院的。然后他们就告诉我专业特别好巴拉巴拉，然后我就申请了。然后当时我们其实习惯性的是去华南理工读研究生，就相当于有点类似于一个保研基地。

那个时候我就面临了一个特别大的冲突，就如果我去华南理工的话，我就赶不上人大的那个。所以一个呢是非常的稳妥，他们相当于是撞车，也不是撞车。我们当时是这样，就是和现在还不一样。那个时候是就不管你是拿到什么学校的offer，你就拿到你就排队。我们学校当时是按照顺序取多少名，就哪怕你去了一个很烂的学校，但如果你是第一个，那你就占了一个坑。然后他们去华南理工的那些人呢是安排在人大之前面试。所以如果他们去了之后，我人大那个就不一定，我要去人大的话，我去老那个华南理工也不一定，但最后我还是选择去人大。

当时心理学的那边双学位也拿到了。就现在的华东师范大学的心理学院的副院长嘛是我导师。当时就说你看人家来修双学位的毕业论文都做的比你们本身学心理学专业的要好。当时就给了我挺大的自信的。然后读本科期间就跟着XX老师，然后也做过一些研究。当时最早是做人-组织匹配的，所以那个时候其实打下挺好的基础的，那个时候我觉得我特别喜欢是因为那个时候结构方程刚刚兴起。然后我就自己去学，也找不到相应的材料。然后后来到了人大读硕士2007年的时候老师讲课，那个时候我已经有论文发表了，所以就觉得比同龄人就过早地接触到这些东西。然后当时那个毕业论文也获得湖北省优秀本科毕业论文。

然后就到北京来了嘛，跟着孙老师读研究生，然后研究生两年。其实我觉得过的不是特别的好，因为08年的时候我想着要出国，但后来那个时候自己也没有特别的说一定要出国或者怎么样，所以左右摇摆，最后就没有申请。当时我想跟着孙老师读博士。然后呢其实也是因为孙老师想让我跟他读博士，所以不管怎么样吧，09年反正我就跟着孙老师读博士了。然后其实在读博士的第一年，我这个人呢是怎么讲就是准备工作做的特别多。就是那些东西我都会，但是我就是不写，所以实际上在博士的第二年之前我基本上没有什么成果，我们同龄人，就是我们班的其他同学都有好几篇文章，但我就没有。但是我就第三年的时候我就拼命的去写，然后呢最后毕业的时候有一篇《心理学报》、有一篇LQ、还有几篇在审的，然后就到中财来。我就这样的一个过程。

所以我觉得简而言之，我就觉得在整个的过程当中没有一个规划。但是给我的感觉是我从来没有想过做其他的事情。因为当时硕士毕业的时候，我们很多同学就去企业做HR嘛。然后我也不知道为什么，我就顺理成章地就要读博士。博士毕业的时候，我们博士班的班长，还有很多同学去企业、去国企。然后呢我也没有想过说去哪里，然后就顺理成章的也就到学校了。

然后到中财来也是一个意外。

刘书博老师： 对，之前你跟我说过

受访者： 对对，你大概知道就是也没想到要找工作的事，因为是10月份的时候，然后就很自然的，别人推荐说递一个简历，然后就到这边来

刘书博老师： 然后校长还给你拜年

受访者： 大概是样子。所以整个的过程我觉得可能···你说规划吧倒没有，但是我觉得还是有两个维度吧。

第一个就是我在整个的过程当中我很享受这个过程。就是有意愿做这个事情，不像现在的说，是被家里要求要去当老师或者做研究。

第二呢，我觉得从能力的角度来讲，我觉得我擅长这些。

所以基于这两个方面的原因，再加上整个的过程当中，包括学的组织行为学，跟着王老师做了一些论文；然后呢硕士到博士期间没有换导师，包括博士毕业的时候找工作也比较顺畅。所以整个的过程好像用我们人力资源管理的amo理论，有能力、有动机、有意愿。所以可能就自然而然的就这样。

刘书博老师： 天时地利人和，还是你本身包括性格比较安静，是一种学者型的性格。

受访者： 对，对对，应该从小就是样子

刘书博老师： 从小就是，而且可能会比较愿意坐下来对一些细节进行思考。

受访者：对，我特别喜欢做整理工作哈哈，就是这个比较擅长

刘书博老师： 猫头鹰似的那种学术。

受访者： 对，对对，就读博士的时候，一个问题如果想弄明白，可能一晚上都不睡觉那种。

刘书博老师： ok，那我觉得还是有天赋的。

受访者： 我觉得是，就是至少在某个点上是需要有你特别特别别人没有的优势的

刘书博老师： 是的。ok

黄秋莉： 我想问一下，王老师就是您身边您的家庭或者是熟识的人，有做老师的吗？

受访者： 没有。

黄秋莉： 就是有这种知识分子吗？

受访者： 没有。

黄秋莉： 那大家是怎么觉得您比较适合当老师呀？就是跟别人会讲题吗？

受访者： 你说是小时候，是吗？

黄秋莉： 就是您一直以来嘛，大家会给你一个反馈说觉得你比较适合当老师。是怎么个表现？

刘书博老师： 就是班长那感觉

受访者： 也不太像班长，因为班长比较外向，我小时候其实属于那种比较勤奋的。

刘书博老师： 你说你数学不是特别好

受访者： 对，不是特别好，所以我高中是文科嘛。

然后我觉得怎么样呢？就是因为我读了大学之后，包括硕士、博士，其实家里就已经没有任何办法给我反馈了，对吧？

那他小时候我觉得他们给我的反馈最多的就是我奶奶以前没去世的时候，经常和别人讲说。有一天就是放了学，还是夏天，然后找不着我了。然后整个的大家族都到处去找我，晚上后来才知道。我一放了学，我就跑到屋子里，里面还停电了，我点个蜡烛在那地方把作业做了，然后还不止一次这样。就是我回到家我要先把作业做了我再吃饭

黄秋莉： 这是一个什么样的···像我啊我小时候就是我先做完了之后我可以去玩儿别的东西

刘书博老师： 你是为了玩嘛

受访者：不不不，我没什么玩的。所以我小时候就很无聊，特别无聊。就包括现在也很无聊，我也不玩游戏，然后也不搞体育

黄秋莉： 整理，会喜欢做家务吗？

受访者： 也不做，我就是擅长，就是你看我电脑上的文件夹都排的整整齐齐的。比如每周我就要把上一周的东西整理好、分门别类。我家务倒不怎么做，但是像这种文案性的工作···所以我其实还特擅长做秘书，就工作井井有条。

刘书博老师： 那我觉得小元也这样，你觉得呢？

受访者： 是嘛？

刘书博老师： 他也很喜欢做文字整理啊。但是他在成绩方面就不行

受访者： 他细节可能抠的不够。

刘书博老师： 嗯。嗯。就是因为我们在同一个办公室嘛，他天天就是在处理那些比如党政的教改

受访者： 我也在弄那些。

刘书博老师： 对，就是那种工作，我个人我就觉得我受不了，因为太琐碎了。

受访者： 我比较擅长，所以可以这样说，我不能容忍不确定性，我比较喜欢规则感。

刘书博老师： 那当时你对那个学科知识觉得特别感兴趣，组织行为学这一块儿、人力资源这一块儿，为什么对它们特别感兴趣？

受访者： 我现在其实有时候也在想，因为那个时候我们同时开了工商的其他的课。我为什么非对组织行为学感兴趣？然后我刚才也讲了，我不太确定是我先读的双学位还是先修的组织行为学，或者是同时。但是我们心理学呢他刚开始修是修普通心理学，什么心理学研究方法、变态心理学，其实和组织行为学还没什么关系。但是我后来觉得他们好像都是研究人的问题。因为组织心理学当时有一章讲工作满意度，工作满意度有哪些测量方法啊，然后工作满意度有什么作用啊，好像具有那种一条一条的知识点的那种东西。然后呢又是和人打交知道的。我就觉得我好像挺感兴趣的，但是呢我这个人其实还不是特别喜欢和人打交道。所以我也有点搞不明白是为什么对这个感兴趣，但我觉得它可能就是很有条理，它是就是、不是就不是，对吧？它还有一套确定性，而且它有一套完整的研究方法。

刘书博老师： ok，ok。

受访者： 包括我现在给MBA讲组织行为学，其实我就会把这个课定位于，基于证据对人进行管理。

黄秋莉： 那工商管理给您的感觉呢？

受访者： 工商管理给我没什么感觉，因为当时就是个大杂烩嘛，和现在一样，就是你什么都得学。

我们当时没有人力资源管理专业。

刘书博老师： 我前两天访谈的是XX。咱们都认识，他跟你就很不一样。他就是一开始就确定要去行业的，但是各种机缘巧合他没去。

受访者：有这种。

刘书博老师： 然后他就觉得自己在充分认识行业之后会觉得自己原来更适合学术，他是那样的。所以王老师就是was born就天生适合

受访者： 对，因为我从来没有考虑过其他的。而且其实我也没有像别人一样，因为···我认识那个XX，你回头也可以访谈一下，我给你介绍一下。XX原来是问题少年，他追星，然后呢经常去给超女做后援团的团长，然后他的经历也更奇特了，他是男的。然后呢硕士毕业的时候没有论文出来，准备要去企业了，然后就是给我们杂志投了一篇文章。他的文章我当时做编辑就是无意当中就看到这篇还不错，就和他联系，结果他毕业之前就发出来了。发出来之后呢他就有资格申博士，然后他就申博士了。

后来我把他拉到我们编辑部做编辑。有一次我们年会一个晚上的青年学者交流，他第一次分享博士成长的历程。那我觉得他也是一种比较奇特的、误打误撞的去读博士了。

受访者： 像我这种，我觉得就是没有考虑其他的

刘书博老师： 因为学术工作的一些特点跟你是特别契合的嘛。

受访者： 对，应该是因为你需要去整理，你需要去抠细节。

刘书博老师： 没错，其实我就这点就不好，我对于细节的整理我就没有耐心，比如说我就烦躁

受访者： 我很有耐心

刘书博老师： 很羡慕。

受访者： 我现在博士生的训练就是细节导向的。就是比如说我们有一个复盘制度。就是每一个项目哪怕是做一个PPT或者是一篇文章。然后呢我都和学生约定好文档的命名方式。然后呢每次我修改，他给我第一版，然后我给他“正”再加上比如今天是0421

刘书博老师： 最后名字会很长是吧？

受访者： 就是要几十个，我待会儿可以给你看看，就是每一个项目都这么复盘的。比如第一篇文章我通常给博士生改十几遍，那就有二十多个文档。

刘书博老师： ok

受访者： 然后他们还要每天要写日记，每天一篇日记，每周一个周报，每周一次组会，每十天一个读书会。

刘书博老师： 日记是什么

受访者： 日记不需要给我，他们想干嘛干嘛，我就让他们每天记录自己在干嘛。我自己也有日记，记了很多年了。

刘书博老师： ok。那为什么要做日记？

受访者： 我就觉得我今天做了啥我要知道

刘书博老师： 所以你可以就是很细节的掌控自己的精力分配。

受访者： 对，我以前呢是每天一个word文档日记，现在不是了

刘书博老师： 那是什么时候开始的？

受访者： 现在都是就是很简单，就是1月1号、1月2号就干嘛了，这样的每天，你看是多少次了。我有55000字了，今天是4月21号，大概2018年开始的

刘书博老师：那个时候为什么想要开始？

受访者： 那个时候我记得是元旦的时候，然后当时有一个什么什么笔记，就是现在不是很多学生流行那个什么“印象笔记”类似于那种，然后我就在上面当时是把每天看的文献的阅读笔记也放上去

刘书博老师： 很好啊。

受访者： 但是后来那个东西就不放到我的日记里面了。我会在日记里面写上，今天2点~5点读了文献，然后呢具体的笔记见其他的文档。我现在就有点像流水账，但是有的时候比如今天特别不开心或今天特别开心，我有时候一写就会写很多。

受访者： 你想现在是4月20号我写了55000字，相当于平均一天多少字。

刘书博老师： 一个月1万呗，差不多一万多。

受访者： 那一天就得至少300字

刘书博老师： 对。ok，行，那我觉得我这个问题其实就得到答案也比较straightforward，就比较直观。而且我觉得其实你也挺幸运的，因为你找到了一个自己的天赋跟职业完全是对应的

受访者： 对，应该是没走什么弯路。

刘书博老师： 对，而且前面包括成绩在很早的时候就显现出来

受访者： 但是在我读大学之前并没有，就坦白来讲，虽然我也不能说很努力吧，反正我觉得我就比较乖，但是成绩还确实不是班级前多少名的那种啊。

刘书博老师：现在管理学会特别强调方法技术的复杂化。那会不会有时候对于数学能力要求会特别高？

受访者： 我觉得倒不是什么问题。就说你数学好不好和你学研究方法和量化分析和统计，我觉得是完全是两回事情。因为实际上我经常和学生讲，我们只是学他的应用和操作，你会用、会解读、再知道一些原理，你不需要去推导。对吧？就比如像那些专门ORM的，或者是那些杂志上面那些文章，其实我都不太读得懂，但是我就看他前面有一堆数学推理，后面你不得附一个syntax嘛，还会有一个叫example，我把那个搞明白就可以了。所以我觉得不是什么特别大的障碍。

刘书博老师： 那你比如说对于现在这种前沿文献，你也是每天都会、及时地更新阅读嘛

受访者： 这两年不行了。这两年不行了，因为我有一个习惯也是就是定期的把最新的文献整理成word，然后呢找学生给他们付劳务费，然后以前找咱们的本科生。就比如说每三个月系统地下十本期刊，然后也是有命名，比如AMJ2020年第一期，然后十篇文章，然后又统一一个命名。然后呢我不管看不看我都会放在那儿。

然后呢以前追的比较新，但是这两年我觉得自从我回国之后，我就没有每天去看新文献了，所以我觉得这个算是一个转变。就从19年回来之后，我觉得我就状态和以前不一样。

刘书博老师： 为啥？为啥会不一样呀？

受访者： 就是陷入到那种特别特别琐碎的事情里面。我觉得我效率最好的时候是16年，那时候我住在魏公村那一块儿，我就每天去舞蹈学院旁边有个咖啡馆，我每天从那从早坐到晚。然后就效率特别好。

刘书博老师： 你可以有一大块时间做一个事情

受访者： 我就是这种。所以我其实就是同一个时间点···，就虽然我可以同时做好几个事情，但是呢我必须倾向于这一段时间主攻事情。

刘书博老师： ok，明白

受访者： 我不是特别擅长在开会间隙在看一篇文章那种。

刘书博老师： 那学术工作可能会有一些传统的那种特点，第一是要专注，要缜密，还有就是它可能需要有自主性和自由。那这两个你觉得对于你价值大吗？就比如说你就想做自己感兴趣的研究，但是有时候是不是自己想做的跟期刊热点它不是很一致的？

受访者： 我没有碰到过这种冲突。

刘书博老师：一般你做的也正是期刊会想要看到的

受访者： 这其实就涉到选题的问题。我自己和我自己讲选题有一些标准，我和学生讲也有学生的标准。也可能是因为我比较乖的原因，我不会选一个离经叛道的题目或者小众的题目，或者说我能想到的其实都不是那么歪门的选题。所以就不会存在说我做的不是主流的。

刘书博老师： 明白，明白。ok，行，那我问题就问到这

黄秋莉： 老师，那您是怎么获取你idea的？就是怎么想到要做题目？我刚刚我听到的是您会定期的整理期刊文章

受访者： 我现在其实给学生讲有几个不同的来源，但是我现在最常用的或者让学生最常用的就是从文献当中来。这也是为什么我会经常给学生发这些打包的，比如我们半年更新一次，那我就会让学生分三次来阅读。第一次呢我就会让学生去看一下，比如集中三天的时间，把最近这一年的十本期刊，假如一共有150篇文章，对吧？因为我们都做成了word，非常的简单，就是我们还有那个结构预览，什么第几卷第几期。就是格式排的特别的好，我待会儿可以给你看一下。然后学生呢就看这个word，可能有200页，这三天什么都不干，就看200页的word

我们那个word上面有标题、有作者、有卷期、页码，还有摘要。然后呢学生就可以根据他的兴趣去广泛浏览。然后呢在第一遍的过程当中，你可以做任何的笔记和批注，然后第一遍完了之后呢，学生要达到一个什么样的效果，就是要把你感兴趣的、你发现最近这段时间很火的、频繁出现的腾到另外一个word上，这就是第一遍的工作。就是你要把标题和摘要，然后你可能还会排列重组。因为可能这个杂志发了一篇文章，那个杂志去年也发了一篇类似的文章。这就慢慢的把一个基于文件包的阅读变成了一个可能有那么几个大的选题了，对吧？有个有大的话题，这是第一步

黄秋莉： 我特别想知道怎么确定自己对哪个感兴趣啊

受访者： 这就是我们也有一系列标准啊

第一就是啊你首先自己要感兴趣，你发自内心的就有兴趣，比如像商业伦理，有些人对商业伦理学不感兴趣，那你再怎么你也不会感兴趣，对吧？比如我有朋友做绿色行为，还有朋友做什么工间休息，我就完全不感兴趣，这种文章我看都不会看的，对吧？这是第一，你要自己要发自内心要感兴趣。

第二呢，接下来就是要有用。因为有些话题我觉得它就是没有用的，它就是一个知识导向的，它不落地，他没有practical implication。

然后第三个就是我觉得要有点热度，就是如果说你看了这两百多篇，只有一篇讲这个，我可能也不会特别的选。什么叫有热度呢？就是有一些但是又不多、又不是那种大陆货。

最后一个就是要有基础，最好是和我们比如共用一些理论、共用一些方法的嘛。

那我觉得第一轮最主要的就是要有兴趣，就尤其是刚进来的博士对他来讲什么都是一空白的，所以时候他就先选有兴趣的，然后呢和我们以前做的有关系的，然后呢相对来说比较前沿，就是有热度的；还有一个就是有用的，这四个一嫁接你就可以从这两百多篇文章当中组合成20篇文章，对吧？20篇文章可能又划分为比如五个东西，但你可能有零散的嘛，这是第一波。

第二波呢就是对这20篇文章的比如有四个大的话题嘛，你分别对这些话进行评估，就你不能只看这几篇文章了，你要去顺藤摸瓜、摸关于东西的前面的那些文章，对吧？去做一个我们叫微综述。微综述就不是写成两三万字的综述，就是一个简单的比如几千字的，不见得是写了，就是画个图啊，前因后果进展到什么程度了。比如说学生如果觉得有兴趣，结果一挖发现东西前面其实已经研究挺透彻的了，已经有那么二三十篇文章了，我们可能也不会跟进。

就这四个我们叫report完了之后我们就可能去跟进其中的两个，这就是从主题到话题了。

然后最后一步就从话题到问题，就是你聚焦于这两个，那你就要去仔细的研读关于这两个主题的所有文献。然后呢去coding这些文献，然后去找到研究的问题。所以从主题到话题到问题，然后每一个阅读的方式也是不一样的

黄秋莉： 感觉是一个非常成体系的。

受访者： 对，我对博士生的培养就是非常有体系的。然后我从第一次课和他们讲学术的规范，比如citation怎么去标，然后APA是什么样子的、到如何读文献、到如何写文献综述、到如何选题、如何写response letter、如何怎么怎么样，就是有一套东西

黄秋莉： 这个是老师自己归纳的嘛

受访者： 我自己的，我自己2020级的博士开始记录，现在已经有六百多条了。就我对他们的指导

刘书博老师： 到时候可以出版

受访者： 所以这一次评优秀研究生指导老师我没有评上，我觉得我挺生气的

黄秋莉：谁评上了啊？

受访者： 研究生院的院长啊、科研处的处长、金融学院的院长啊

刘书博老师： 应该跟那个XX说一下。

受访者： 哎，算了，我反正我觉得我学生的培养效果还挺好的。这一套就经过了检验。你看我现在有五个（指导的）毕业了的硕士，五个硕士当中有四个拿到国家奖学金，有四个拿到A类论文，100%都拿到了科研立项，然后60%拿到优秀毕业生，还有40%拿到了校级优秀论文，所以其实我觉得挺不错。但是人家就是“我目前培养了37个博士，或者我从教20年来培养了一百多个硕士”，那就没得比了。

黄秋莉： 我们也挺羡慕就是老师带的这种博士生嘛，他们现在也是取得很好的成果

受访者： 但我觉得还是得匹配。因为你也知道我有一个学生，他就是被我劝退学了，我就觉得我从他身上看不到任何潜质

黄秋莉： 是吗？就是您会觉得什么样的学生适合做科研，就是什么是有潜质的呢。

受访者： 这个问题我觉得特别好，就是我自己也归纳过。博士生入学的时候，比如下周二我会面试两个到我们这儿来读研究生的。一个是川大的人力资源，一个是西交的工商管理。然后他们都给我写邮件。以前呢我是想着希望他们读博士，但是现在我对于这个东西没有特别大的要求了。如果是博士想读我的，我就会有一套严格的甄选的流程。我会和他们去做两次面试，我和他们介绍我们六个一，我刚才讲的六个一，你能不能做到，对吧？然后呢还有就是你能不能达到我的条件，我对学生的期待有8点

黄秋莉： 您能讲讲吗？

受访者：这些东西其实我可以给你的。我觉得我现在说可能不系统，我那个文档里面会更系统。因为我说我很喜欢整理嘛，我所有的这些东西其实全部都有文字性的东西，我都是用word。

你看我这边有一个文件夹叫“博士培养”，然后呢我这边有一个“师门传统和培养”，然后这边有一个“对毕业学生的总体期待”。新生进来之后呢我就会和他们讲一下，其实这些东西都是会留存的。这样我就省得每次···这也就是为什么说每次我给学生开组会，我都让他们录视频录音。就是因为有的时候新的学生进来，我就不需要再重头讲一遍了。所以我们现在每次组会都有录音录屏。

然后比如今天教学生如何去写引言，就会有一个专门的学生把我们今天讲的还有前期我给学生改的那种引言整理成···这就是一个百宝箱。我有一个秘笈，我待会儿给你看一下，就是总体期待是这六项，后来就变成八项了。然后六个一，这是学生写的日记、周报。就是每周要一个周报，每个周天的晚上24点学生要给我发周报。周报里面就是你这周都做了啥、下周要准备干啥、有什么问题，然后等等。然后这个是组会，我们也是按时间来排的，你看每次都这样，所以其实做的很完整的。然后项目复盘，你看这是XX（受访者的一位博士生同学）的一个文献综述，他完了（发表了）之后需要有一个流水记录，就是从哪点开始的，然后紧接着你看这是大纲，我给他反馈的大纲，然后综述第一版本都有反馈的，事实上对学生来讲，我的反馈速度是比较快的，然后这个是相当于是记录。然后这是他另外一篇文章。

所以我们每个文章都是有这样的一个东西，然后这个流水记录里面还要列出我每一版核心存在的问题和我的反馈。这样的话你回头就形成自己的百宝箱，然后我们每个学期要做对学期的一个梳理。我觉得很系统。这个东西也是从2020级博士才开始的，因为我想着我既然带博士了，以后肯定每年都会有新博士进来。那我要把东西重新再讲一遍也很烦，对吧？我就让学生整理。然后呢这样的话新来的博士进来我直接就是···

黄秋莉： 就是有积累的，就是每一次都是在以前的基础上进步的。

受访者：对，就是不断的不断的去更新和迭代。

黄秋莉： 您之前那个劝退的学生，他是···

受访者： 那个学生主要是我觉得他本身他读博的意愿很强，但这个意愿呢是他父母给他的，他从小就听他父母的，所以他父母让他干什么他就干什么，这是一方面。

另外呢就是他的能力确实不行，所以我觉得潜质很重要。他没有悟性，他不能举一反三，然后呢主动性也很差。什么叫主动性差呢？就是比如你让他去怎么样，我需要给他写outline，我给每个学生都会写outline。比如我们讨论完之后，比如我们引言部分分四段，第一段讲什么···他呢就是特别依赖于我给他这个东西，他就是特别擅长往里填空，但是他没有自己的想法。然后经常出现什么情况呢？他读了很多文献，确实读了很多文献也很用功，问他有什么想法，没想法，所以我就说他是典型的读文献的时候是用眼睛读的，不是用脑子读的，更不是用心读的。所以我对学生要求说要从点到线，从线到面，从面到网嘛，他就是点，这篇文章他读的非常熟，所以这篇文章他了解的很透彻，但是他从来不会去想这篇文章和那篇文章的···所以就没有融汇。然后他从来不会想到这几篇文章，比如谁和谁是打架的，谁和谁是一脉的，所以经常问他你最近读了这篇文章有什么想法，没有想法。所以后来他也意识到问题，他父母其实也知道他这方面可能没有天赋，所以我觉得确实是需要（潜质）的。所以我对于学生的专业没有什么要求，我觉得就是学习方法、学习习惯的问题。

你像XX我觉得就是我最好的学生，就是我刚刚给展现的，有很多是他的，就是他做的。包括现在今年有一个江西财经保研过来的直博生，就是属于那种能够举一反三的。

而像XX（被劝退的那位学生），第一篇文章不是要改十遍嘛，我改了这个地方，这个地方和那个地方是同样的问题，他就看不到那个地方的问题，他就只把这个地方改了。但是XX（受访者的另一位学生）呢，他就是不光把这个问题改了，他还给我写个周报里面，“老师一共有120条批注，这120条批注当中有15条是关于用词的，有十几条是关于逻辑的”。他会思考，他会整理，然后他会写成自己的百宝箱，我给你看一下我们的百宝箱

刘书博老师： 是非常实用的建议，就可以直接拿过来用

受访者：我随便给你找一个，这是关于如何选题的一个百宝箱。我就是给学生整理，比如如何确定选题方向，我刚刚讲的要有四个东西，这里后来又更新了五个。然后选题的途径怎么选？然后三种阅读方式等等。然后怎么去确定话题，怎么讲故事？然后我还有自己的电影理论和做菜理论等等吧。然后还有一个我给你看看，就是我想和你们分享一下我怎么带学生的。

刘书博老师：这就是我们专业化的过程嘛。

受访者： 就是每个东西我都会有一个文档。你看如何认识数据库，我就是相当于是给他们讲的第一次，然后每次其实我们都会录屏，然后后来的学生就可以看到了。然后呢还会做成一个文档，相当于就是不断地去建秘籍。然后这是我们的一个秘籍常用的，然后你看新来的学生我直接给他，我就不需要再去跟他讲了，然后如果他有更新他就更新再给下一级，对吧？就是这样的一个持续优化的过程。

刘书博老师： 那你也付出了很多精力

受访者： 巨多无比。

刘书博老师： 因为每个学生你给一百多条comments，都是非常特色的那种

受访者： 然后第二篇我就给80条，第三篇我就给60条。

黄秋莉： 这也是有标准的吗？

受访者： 不是不是，就是你会肉眼能感觉到少了，比如用词可能就不会出现这种问题了。但是刚开始第一篇文章真的是特痛苦。

我一定要给你们找到那个，你看这是XX（受访者前面提到的带过最优秀的学生）的，你看这是PPT阶段，刚开始是让他做一个PPT，然后每个PPT其实都需要复盘的。然后综述阶段，我刚才讲了这些。然后紧接着是修改阶段，然后这个是整个的时间线。

黄秋莉： 相当于XX（受访者前面提到的带过最优秀的学生）是您的第一届博士生？

受访者： 第一个博士，我第一年就阴差阳错招了两个博士，所以呢也让我有动力去做事情。就是前人栽树后人乘凉的，所以现在XX（前面提到的本科室江西财经大学的直博生）进来就没有走前面的流程，我就直接把这个东西打包给他。但是我也不是一次性打包给他，我是要看时机，就是他到了阶段，我就会给他这些东西。因为你一次性给他很多很多的东西，他不知道从哪开始。所以XX（前面提到的本科室江西财经大学的直博生）进来之后我就和XX（受访者前面提到的带过最优秀的学生）一样，第一年第一个学期啥都不干，就读文献、做笔记、做coding、写综述。每个学生第一篇文章肯定是综述文章，不管投不投都是这个样子的。

黄秋莉： 感觉您提到XX（受访者前面提到的带过最优秀的学生）会多一点，提到另外一个我觉得会少一点

受访者： 都挺棒的。但是XX（受访者前面提到的带过最优秀的学生）我觉得更有我的那种风格，就是井井有条，然后特别擅长整理。

黄秋莉： 那您在带学生的过程中觉得最开心的时候是什么时候？

受访者： 最开心的时候就是学生论文被录用的时候，我给你找一个···那个我觉得特别特别好的···我们这儿还有一个学生做PPT和做pre的一个···就是学生做PPT怎么做、然后学生去pre的时候···就是你看我会有提到，对于主题类的分享你应该怎么办，然后对于这种场景类怎么办，然后对于proposal类的。然后会有讲到，比如开始之前要干嘛、第一步要干嘛、第二步要干嘛。所以我觉得这应该算是专业化。就是有一个guideline，有一个指引手册，就相当于是现代化。就有点类似于说，学生进来之后拿到手册就可以从事科学研究了。第一步要怎么样？第二步怎么样？读文献要按照我的三部曲，然后选题要按照我的四部曲，然后一步一步的，就是有点类似于是流水线，SOP。这些其实就是SOP

刘书博老师： 就是现代化的、标准化、程序化、然后可以去衡量。但你在衡量这一块儿，整个过程你的判断还是主观判断，你可以把它就把变成量化的那种指标吗？

你这个就程序化和标准化很清晰了。那你判断学生到底···你比如说有一些指标，你就直接打勾就行了，那可能还没有办法做到

受访者： 没有办法做到，这个就相当于是一个投入，对吧？那学生每个人都看到这一个guideline了，但是他的产出一定是他给我发的文档，我得自己判断，对吧？我经常会说，“哎，你们还是没有学到怎么去写引言”。我把引言分成六种类型，就六种切入方法。然后他们写了之后，我还觉得，诶你这种切入方法不好，你还是要再回去看看。这就说明我虽然给了他们东西，但是他们还没学到位。

刘书博老师： 因为他可能还是需要导师在主观的判断

受访者：肯定。因为这个东西相当于什么呢，我给他们做这些guideline的时候尽可能的往里面加了一些活生生的例子。但例子毕竟是做过的例子，他接触到一个新的项目的时候，他还是不能够100%的应用。所以还是需要说这个东西给你只是一个基础性的东西。然后呢每篇文章都有自己独特的问题，对吧？那我就需要去批注了。所以我其实也特希望学生每做一个项目都有提升、有积累，这样的话下一个项目就会少。肯定我觉得总体趋势是越来越少，因为他越来越成熟，但是每个肯定也会有新问题。新问题我们就加到那个百宝库里面。

比如像我，如果做访谈的话我就会做记录。

黄秋莉： 我感觉我刚刚听到了很多，然后就是想问一下就是您就是有一个好的想法了，那后面的这种产出呀就是付诸实践的过程也是有一个清晰的步骤吗？就是你自己有一个比较好的想法了，然后你怎么把它产出一个比较好的论文呢？就是怎么变成一个论文的过程。

受访者： 这就是按照整个研究的流程了，有想法你就根据相关的文献把它细化，去体验出它潜在的这样的一个贡献点，然后讲故事，然后呢去收数据，然后去写、改，这样的一个过程。

黄秋莉：在写论文当中，各个老师也都在强调说理论意义和实践意义，就是说要体现出这篇文章的一个创造性。那创造性的话你觉得是怎么在自己的文章中体现出来呢？

受访者： 我们也有整理。我们有一个关于如何讲故事（的百宝箱），其实如何讲故事，就是如何做贡献。我们分成了六大类，比如其中有个叫制造冲突，比如有一个叫做解决冲突，然后解决冲突，我们又分为从方向上解决，还有一个是从程度上解决。

就是给我的感觉，好像是现在我自己是有一些标准化的东西在里面。我就拿着东西我就去复制，我是有感觉的。就是一旦你形成这个东西之后，我就想我今天要讲一个什么样的故事。因为我相当于把论文当中每一个部分都拆解，有一个一个的guideline。

这个时候我觉得也有点不太好，就是不会特别的具有那种原创的那种东西。

黄秋莉： 我感觉就是对理论有很大的帮助。那对实践的关照呢

受访者： 我现在我觉得我越来越多的讲到实践价值，包括政治···也不叫政治正确。就说比如研究压力，很多文章他讲故事就讲怎么样去减少压力对员工的负面影响，比如让员工去进行工作重塑啊等等。像这种文章我是非常不喜欢的。

然后我如果讲故事，我就会讲我怎么样从组织层面去减少这种压力语言的出现。而不是说我作为一个人力资源管理者，我作为一个研究者，然后我去研究员工在碰到这种压力的时候员工自己来去改变。当然是可以。但是他有点过于···就是组织要去挑选那些我们这样的员工，然后这种员工更能适应这种压力，我不喜欢那种文章，因为我觉得他没有人本。我现在特别在意这一点，尤其是我现在做关于压力的研究，我发现很多文章的立场是有问题的。

刘书博老师： 就站在资本家的立场

受访者： 他就假设这个压力是不能改变的。然后我就要找能适应这种压力的人。而不是想用现在组织的资源和组织的能动性，怎么样去减少压力源的出现。

黄秋莉： 所以就是您其实对自己的论文有一个标准的改变哈

受访者： 以前没有

刘书博老师： 我觉得是一种追求新的这种价值方向

受访者： 我觉得是比较明显的。现在很多很好的杂志上的文章，虽然我发不出来，但是我不太喜欢。因为我就觉得他可能就是说明了一个现象，它没有提供解决。

我现在也特讨厌那种研究，比如辱虐管理的积极作用，就是坏东西的好的一面。我现在特讨厌这种文章，我就在想so what？你是要鼓励辱虐管理吗还是怎么样

刘老师刚刚说的很对，我就是要突出人的重要性、人的价值。所以我给学生讲三个标准就是“有趣、有用、有光”。以前我们都是特别强调文章要“有趣”，然后“有光”insightful，我自己也有这样的一个标准，以前呢我们都强调有趣，现在我把有趣排到最后，我会把“有用”排在前面，然后“有光”能够启人心智、引人深思。然后有趣我把它排到最后。

黄秋莉： 那您现在会去更多的去深入实践吗？

受访者：我感觉我是基于我自己的观察，我还真的没有特别走进实践去解决实践问题。所以我觉得这还是有一个gap。就我现在意识到，我想这个文章真正去讲一个故事，然后呢能够给管理者实践启示；但是呢我在选题的时候呢又从文献当中来，还没有做到那个真正的从实践当中切入。

黄秋莉： 所以您就是因为我们可能限制就是我们的一个工作场域，主要是在学校嘛

受访者： 我觉得这是主观的问题，这不是客观的问题。

这是一个想不想的问题，不是有没有机会选择的问题。你刚刚的意思就是说因为我们在学校我们没有机会接触，但我们有很多机会接触啊，我不想而已。

黄秋莉： 您以后会往这个方面走吗？

受访者： 反正我觉得这就是目前我的问题——我没有真正的想去和实践接触

黄秋莉： 那您未来对自己整个的职业期待是什么样的

受访者： 整个的职业方面。哎呀，其实呢我觉得我有点晚了。因为本来我其实早些年的时候别人和我说可以加加油，然后申请个什么帽子啊之类的，然后我觉得其实我挺看重的。但是我现在都37了，基本上就没有戏了嘛。然后没戏之后呢我反而不太去想这些事情，我觉得我对我自己的认同还是来自于在研究上。但我现在我觉得过了那个年龄，我现在没有像很多年轻人一样aim到很high的那个程度，

刘书博老师： 他们aim high是指很高的帽子嘛

受访者： 不，不，不，他们有些“我就要做AMJ的文章，我除了AMJ的文章我就不发”，我不是，我也不知道为什么，我经常会觉得比我差很多的人，或者是刚入校的博士，他们一开始就要励志发什么AMJ，但是我好像从一开始做研究，我就没有这个想法。所以可能就和人的境界有关吧，我觉得

刘书博老师： 是不是因为他们“非升即走”的那种外部标准

受访者： 我觉得有这方面的压力。然后呢也有一些可能就是个人本身他是一个比较aggressive的人，我本身就比较保守，我投文章我永远是投比我的文章相近或者比我的文章质量要差的期刊

黄秋莉： 不喜欢不确定性

受访者： 对。还有一个我跟你说，如果是审稿人的意见很差，我就不敢了。但是我认识的很多人，他们就坚信只要审稿人没拒你，那么就有希望。我不这么想。拿到letter之后我自己会评估，如果太难我就放弃。所以我可能就是一个保守主义。

黄秋莉： 那您有没有一个学术榜样呀，你觉得就是长期以来的话

受访者： 我现在没了

黄秋莉： 以前有，是吗？

受访者： 我不追星啊，然后呢我也从来不想把任何人作为自己的榜样

刘书博老师： 或者说不是具体的人，你就希望自己能够做成什么样子。

受访者： 我觉得我最近这两年就想多做一些学术的服务。然后呢我觉得是我特别特别在意的

刘书博老师：为什么在意？

受访者： 我也说不好，首先第一你说他有权力吧，它确实也有权力；但是你说它到底能给你带来什么，我也说不上来它能带来什么。就比如像新的HRDQ，我每周要花一整天的时间在这个杂志上，我每周要处理三篇稿件的初审拒绝。因为既然是初审拒绝，一般来讲你就要让别人心服口服

刘书博老师： 你要认真。

受访者： 然后我后来想我做这个干嘛。首先因为这个杂志的规定，如果你作为主编团队，你在任期之内你是不能在杂志上发文章的哦，所以那我就在想我干嘛呢？那我觉得可能是好面子，我觉得是这样

黄秋莉： 就给您带来一种比较良好的感觉？

受访者： 对，然后至于说能够带来什么社会资源或者是学术资源，我觉得应该也是有的。

刘书博老师： 但是你说“好面子”可能是谦虚的，可能你会比较想要有reputation

受访者： 对，我想要reputation，我很在意这个东西，我很在意别人怎么看。我有一段时间我没有文章，我会觉得别人是不是觉得我最近老没有文章。

催我上进的最好的情感，就是羞耻感。我跟你说有段时间呃，就是很坦白的讲，大概在刚来中财的时候，我一直aim都不是特别high嘛。然后就刚开始就是发一些说好也不好、说差也不差的文章。然后呢我有段时间我就频繁的看到我的师弟，还有和我差不多水准的人，哎呦，人家这么悄无声息的就发出来很好的文章

刘书博老师： ok，

受访者： 我经常哭，你知道吗？

黄秋莉：我明白

受访者： 我以前订了很多杂志的那个email alert，就只要出现新的issue了，他就会给你发邮件。我后来就不定了，因为我老受刺激

刘书博老师： ok

受访者： 所以我现在不主动地去看这些新文章。但是我每次看到，比如昨天无意当中看到谁发了一篇文章，我就想，以前他还向我请教过很多问题呢。然后我昨天日记里面就把我自己好像反思了一遍。

黄秋莉： 可是就是在我们看来，您现在是年轻有为嘛

受访者： 那你要看和谁比啊，你们看是和你们比、或者和大多数人比，但是站在我的角度，我就会和比我更高的人去比。

刘书博老师： 是的，所以学术界我觉得这种内驱还是挺重要的

受访者： 内驱特别重要，因为你要有羞耻感。

刘书博老师： 对对。然后当然现在我们有很多外部的驱动，非升即走的，类似这样的。

受访者： 以前我没办法理解国外那个tenure，就是拿到tenure之后就不干活了。

确实当然有一部分人就不干活了，但是还是有很多人持之以恒的。你看如果简历里2018、2019、2020都没断，到了2021年没有文章了，他可能就觉得“哎那我在干啥呀？”对吧？他就会有这种内在的驱动力。

黄秋莉： 是。就是您提到您之前那个研究嘛，要有光，那这个是不是也跟您现在的一个研究方向就是道德和服务型领导有关系

受访者：我觉得没有。

黄秋莉： 那你当初是为什么选择这个？因为好像您的很多文章都做这个

受访者： 我最早是做商业伦理的。那商业论理其实我说不上喜欢，就说实话，真的我不觉得我喜欢他、也不讨厌他。我觉得原由就是我读博士的时候，在我第三年的时候，有一篇《心理学报》。那其实也不是我做道德领导，而是我们数据里面跑出来一个道德领导的文章。然后就发出了《心理学报》了，2012年。

然后呢2013年的时候，我开始申国家自科，当时已经到了中财来，然后当时我可以申我的博士论文，然后也可以申···。因为你申第一个项目你还是要有基础嘛，然后想想巴拉巴拉我就发现，诶，就像我选题一样，我申课题之所以100%的能够中，也是因为我自己有一个课题选题的一个标准。比如其中有一条就是要有前期的积累，然后我想了想，我积累可能无非就是我博士论文的领导成员交换差异化。还有另外一个原因，那是因为在我早一年之前，另外一个老师申了我的博士论文，那我就只能申道德领导嘛，然后就申到了道德领导了。申下来以后你得做嘛，对吧？那就持续不断地做，越做你肯定就越多积累嘛，那你就做起来了。你想想你在道德领导上已经发了五篇文章了，那个文献综述你都已经滚瓜烂熟了，几百篇文章你都看过了，对不对？你就不需要再去挖一个新的矿了，对吧？就持续不断的去挖它

黄秋莉： 还是一个持续进步的过程

受访者： 因为我选题的一个标准就是要有基础的，但是你始终要保持你自己的一个矿。就刚刚你看那个图，但是肯定要适当的延伸，你不能越做越死嘛。你像我最近这几年做关于工作家庭的，也是在那个基础上延伸。就像我做服务就是因为2017年申课题的时候，我又在拔拉我有哪些想做的，那个时候正好有一篇human relations的文章，然后还有一篇《管理评论》的文章，还有一篇什么的文章，好像还是一篇《心理学报》的文章。我想诶我好像这个有点积累，我就申了。

去年那个自科又和第二个自科有关系，因为第二个是服务导向人力资源，那我在申第三个的时候想，诶，那我申什么呢？那我继续往前走，要么申道德，要么是人力资源，那我就申了人力资源，就变成了人力资源归因。所以基本就是build on的过程

黄秋莉： 除了您就是有基础就可能是做的比较的深嘛。你觉得申课题还有一些别的经验吗？因为听好多老师都会反馈说申课题非常具有不确定性，就对他们来说很难嘛。但是对您来说就很简单

受访者： 我上次在学院也分享过申课题的经验，我觉得这是一个系统工程。几句话讲不清楚啊。我觉得有几点，首先你这个人别人应该是认识的。就至少在目前情形下，申面上项目首先你不能是默默无闻的人，那就意味着你前期基础要好，对吧？

第二，你选题要好。

第三你写的要好，什么叫写的好。我觉得这个真的是特别有技巧。你开篇怎么开篇，对吧？你起承转结怎么转。包括一些很表面化的工作，你行距、字体、字号、转页啊，这些都是要特别注意的，我就抠的特细。我给你举一个例子，比如这一行我会因为它可能在另外一个版本上会出现下一页只有一行，或者是这一页的最后一行是下一段的第一行，我就会有意识的空一行。而且我不是简单的空，我就会插一个分页符，因为我怕如果简单的空行到了另外一个电脑上就会乱

黄秋莉： 就不需要别人抠了，你自己已经抠的很细了

受访者：绝对是这样。

黄秋莉：百发百中，那有没有什么让你比较意外的地方嘛？

受访者： 我现在申的八个课题都中了。

刘书博老师： 就把王老师照片放在你桌子上，睡之前拜一拜哈哈

黄秋莉： 那您长期都是在OB这个领域嘛，那您觉得这个领域在国内有没有什么问题，整体来说

受访者： 我觉得问题肯定是有，包括我自己的问题，第一就是远离实践。我虽然没有太多的接触实践，但是我真的觉得这是一个很大的问题。就比如我举个很简单的例子，我最近做了三个文章，都是在网上收的数据，我自己都知道问题，对吧？我们就是看文献，然后看文献存在什么问题。我们就提出问题，然后我们就拍脑袋去想，然后我们就去收数据，然后我们都不知道这个数据来自于哪里、谁填的，对吧？然后我们就把它写成文章投出去，然后还提管理实践（启示），我觉得根本就不是一个正常的生态。但是国外也这样啊，而且国外比我们更严重，对吧？我们说我们不能数据驱动，不能得到数据之后然后再跑模型。我以前很天真的认为不应该这样，我去了国外之后发现老外更喜欢这样，不是也这样，而是他们更喜欢这样。他有个博士生在这边，几个教授讨论，你试试这个行不行、那个行不行。因为他们更擅长去讲故事，有些人为什么统计不会，但是他能发很好的文章，就是因为善于表达。即使一个很烂的故事、一个很烂的模型组合，他都能给你讲出花来，就他很擅长去story talking。所以我觉得就是脱离实践是一个特别大的问题。

第二，我觉得第二个问题就是越来越微观化，越来越在自己的一亩三分地里面，比如举个例子就是领导啊，今天他提个领导理论，明天他提个领导理论，有几十个领导理论，那在企业当中到底怎么用啊？你这篇文章确实证明了这个领导好，然后你的管理实践就是组织要招聘、要甄选、要培养这种领导。然后那篇文章又说那个好，那作为管理者怎么办呢？如果哪一天我看到了你们这十几篇文章，那我到底要选什么样的领导呢？

刘书博老师： 是的

受访者： 我觉得就是科学分工太细了，导致众说纷纭，就难以整合了。

刘书博老师： 对，是的。就针对你刚才提的第一个问题，有的学者会认为我不管现实，我只是在知识创造。那未来可能现实会用到我创造的知识工具。他们会以这种方式来表示自我慰藉或者理解。

对于第二个问题呢，那有的人会说现在那就是专业化的时代，你只有够窄才能够深，所以我们未来如果合作，每个人都是泛泛的都知道很多，可能每个人都不是专家。他们会这样说。那么对待这两种回应，你觉得还有其他的？

受访者： 你是说怎么回应这两种comments是嘛。我觉得他们说的不是没有道理。我觉得我不能同意，但是我肯定也不会不同意他们的话。因为确实每个人都是有分工的嘛，我学者我就是发论文啊，然后你实践者，你就是用。那我觉得在中国这种背景下，我们少了联系这两个群体的人。在国外有一波叫行为科学家，或者咨询顾问，他们会把学术研究转化为一些管理咨询的方法论，然后呢去供企业来用。但是在国内，我们的咨询公司他没办法承担这个工作，所以我觉得你分工可以，但是你分工中间得有人做这个分工。你不能说我管我的，他管他的。当然你站在自己的角度没错，但是你要站在整个的community的角度来讲的话···确实可以这波人就天天做基础研究，然后这波人呢你就管理实践。但是比如作为政府，需要有中间的那一拨人，但现在中国没有中间那一拨人。

刘书博老师： ok。

受访者： 所以如果没有（中间人）这种情况下，他们说的对不对呢，我觉得对，确实是站在自己的角度来讲。

但是要站在更高的层次，各扫门前雪对吗？我觉得对，只要规则够清楚、分工很明确，每个人扫自己门前的雪，整个街道就干净了。但现在问题就在于说，有几块儿区域是没有人扫的。所以呢你确实扫好你自己的了，但是整个街还是不干净

刘书博老师：是的，那再具体到咱们学校，加上您现在也在做学院领导工作，感受怎么样？如果让你来重新设计工作流程呀或者是组织架构呀，会不会有一些不同的想法？

受访者： 我觉得我没什么感受，反正我挺不喜欢的。我挺不喜欢我现在工作的，我觉得我不享受这个过程，就是从我个人来讲，我不想做这个过程。所以我不一定非要接下来做这个工作，我自己也没有想好到底是做还是不做，但是我没有那么想做。然后呢我现在其实尤其是最近这段时间，你也知道什么思政啊这样，或者是今天林老师又说研究生也要督导组了，马上下周也要成立，然后我就会觉得，就是规则总是很容易就改变了，没有持续性。就像今年那个评职称的规定，好多人给我反馈说，“哎，今年怎么突然就这样了？”

黄秋莉：那些老师们都向您反应什么呀？

刘书博老师： 我给你看个文件，这是我们新的大学章程。你看就他明显改的就很明显，比如说要加强党的从严治理全面制度，他就写到章程里。之前没有那么明晰

受访者： 就和咱们培养方案改的一样，也是一样。

我觉得吧修改是没有问题的，关键是你得持续一段时间。

刘书博老师： 对对，他好像改了之后也没有反馈机制，就每年改了之后效果怎么样，也没有人去追踪事情

受访者： 对，我觉得得看每个人的定位了，我还是觉得那句话，就是人要有不可替代性。就是在任何时候你要有自己的一个杀手锏，就是你只要有杀手锏，其他的东西你可以不管不问。所以我是觉得权利来自于依赖，对吧？依赖要么来自于你很重要，你不可替代，然后呢你稀缺，对吧？我经常告诉自己只要是比如在某一方面能做到这一点，从功利的角度我来讲，我可以不用管学校和学院这些事情。所以有时候其实挺羡慕有些老师的——就自己把自己的文章发好了，你学院再怎么样又怎么样呢？对吧？

但是得看你怎么看了，站在你自己的角度可以这样去做，但是要站在学院每个人都这么想，那肯定也不行。

刘书博老师： 那现在这个工作会比较累

受访者： 我觉得每天好像都在救火一样。

刘书博老师： 救火？

受访者： 嗯，这又来个文件，那又发了文件···

刘书博老师： 所以就是自上而下的那种

受访者： 你没有觉得OA系统更新的很频繁嘛哈哈，而且我自己也有个转变。以前我从来不上OA的，我现在每天我都会去刷好几遍OA系统

刘书博老师： 因为你不刷可能在信息层面就···

受访者： 对，我觉得是我慢慢的被制度化了。

刘书博老师： 哦~所以还是与我们这个大学治理模式直接相关的，就是教育部管着你

受访者： 我觉得他是一个超越了我们个人作为研究者所能够控制的东西。所以要按照我的性格，其实我就是很温和，就是我如果说不做这个事情，那我可能就什么都不做了，我全部工作全都辞掉，然后我就很享受我自己作为一个有价值的人，或者对学院来讲不可能辞掉我的人的工作方式。但我现在还没有一个决定到底是什么样子的，这是很真实的和你表达目前的想法。

刘书博老师： 那你不担心慢慢会被制度化成那种方式吗？你就可能慢慢的就···你会内心可能就熟悉了之后习惯，习惯之后就被转变了。

受访者： 唉，我觉得也有可能，我现在肯定不敢说我不会那样。因为你到了那个位置，你可能考虑的事情，就不是你现在没在那个位置上考虑的事情。

刘书博老师： 对。

受访者： 我为什么当时学院找我做这个事情的时候，我未置可否？首先我主观上没有特别大的欲望去做事情，但是呢我自己在做事情的时候呢，我还会去考虑说，如果很多智商很高的、情商很高的人建议我做这个事情，那肯定是有什么我不知道的、但是他们知道的。然后呢建议我做、或者做有什么好处的东西，这样我就也没有拒绝。

刘书博老师： ok，ok，你可以说人在江湖，身不由己

受访者： 身不由己（这个词）好像感觉有点被动。但我好像还不是特别的被动，就说你看有人研究做的很好，然后他研究做的比我好他都做这个事情，那肯定说明有什么

刘书博老师： ok，明白。

黄秋莉： 你发现有什么好处吗？

受访者： 到目前为止我一直没发现

黄秋莉： 然后想问一下王老师也去国外了嘛，您觉得国外的学术环境跟我们国内有什么样的差别？

受访者： 最大的差别就是他们非常非常的自由，就是他们一个学期都不一定开一次会。我在那个学院就是有什么事情发邮件就行了，对吧？你想想有多少事情是不需要讨论。我那个学期就一次

刘书博老师： 他们更没有党务会啊之类的。

受访者： 对呀，就是省了很多很多的事情

黄秋莉： 他们可以专心做自己的研究

受访者： 第一是他们可以专心做自己的研究。

第二就是我觉得有些老师其实只是把它作为一份工作。他们发的论文好与不好，好像并没有我们中国人那么那么的在意——就是说这个关系到我的饭碗，关系到我能不能留在北京，关系到我能不能孩子上学。

刘书博老师： 所以他们的work life balance，界限还是很明显

受访者：界限很明显，我觉得根本的原因还是因为他们压力小。

刘书博老师： 嗯。那美国听说好像标准也挺高的嘛

受访者： 对，但我觉得他们就是怎么说呢。可能是有这种情况，那些去了好学校、比较标准的那些人，他能够去handle压力。

然后现在还有就是非升即走制度下学校其实会招比他需要更多的人，就是有可能80%的可能性不会留下那些人，但是他就招进来了，那这是自然而然的，他就会有压力的。

刘书博老师： 所以他们那边就是大概有两个位置，那就招两个。然后后面你自己看能不能达标，但是我们呢就是有两个会招十个。

受访者： 对对，很多时候呢咱们不是人和制度比而是人和人比，像咱们学校就是啊，咱们学校评教授就是看你的竞争对手是谁。那国外呢不是啊，就是合同里面写好了，比如六年发几篇，然后做了多少学院服务，评教成绩可以了，就送外审，外审就是和标准比

刘书博老师： 然后过了就过了，所以我们就是锦标赛，就人为的搞很多比赛。

受访者： 水涨船高嘛。

而且他们的制度又变得比较慢，所以相当于他们有很大的缓冲期去做这个事情

刘书博老师： 对，我们的制度又总是在调。

黄秋莉： 除此之外呢

受访者：你说学术生态哈，我还觉得有一个特别不一样。他们不像我们国内有圈子的文化。他们是你发你的、我发我的。我觉得这一点我特别感同深受。就是在国内你比如像我，我也有圈子，对吧？然后呢每个人都有圈子，举个例子，你想申课题、你想发论文、你想报各种教学的奖项，你肯定要有network，你不可能没有的。对吧？然后我们有各种各样的学术群，什么OB群。各种各样的就是这种中国的信息如此密切，实在让人的压力特别大。OB群里面有500个人，每天都有人发谁发了一篇文章，谁发了没有，谁发进去了，就特别特别的难受，每天都是各种恭喜，然后各种祝贺，然后谁又获什么奖了，谁又发了什么JAP了，我现在都把它禁言了，就是这种搞得大家工作和家庭没有边界感了，我觉得那国外他可能没有一个类似于什么wechat的一个学术群，就是有什么论文就发进去

刘书博老师：对对。是这样

受访者： 国内圈子的文化，这种工作使得你不得不24小时都在工作状态当中，你比如我要是找谁问了个什么事情。我要在国外的话我就要发邮件，我可以选择我看不到或者是第二天再回，但是（国内）现在领导给你打电话，那我觉得这一点是特别特别要命的。就是在国内你做任何事情，哪怕是学术上的事情，报个奖、申个课题，都有人打招呼，都要network。然后在国外呢我觉得他们就是祝贺，知道了就祝贺你一下，他们不会有特别多的人际交往。

我觉得现在看年轻人啊，我们这一代人，我觉得压力最大。我们往上走要去迎合那些60后的资深的教授们，比如各种各样的年会，我们要喝酒。反正我在这个圈子里面基本上就是四场酒，第一场是主办方的那种所有人都参加的自助餐或者是桌餐，这种不喝酒的或者少量饮酒的。

第二场呢就是主办方的比如院长、副院长，或者是当地人比如去武汉开会，武汉肯定也有一些比较资深的人嘛，他会张罗那么一两桌，这是第二波。大概到了（晚上）10点左右了。

然后第三波、第四波，越往后越晚回酒店的，就是越在圈子的核心

刘书博老师： 你观察得特别好

受访者： 自助餐就拿餐票的那时候，然后拿餐票的时候或者提前你就会就会突然发现被拉到一个小群里面了。然后发个位置。

但在国外你看人家开AOM会议，那就是非常非常熟悉的合作者才在一起吃个饭。

我觉得这一点特别累。

刘书博老师： 所以我们的职业生态跟西方可能不太一样

受访者： 对于他们来说，这就是一个工作，下了班就下班了。

刘书博老师： 但是你了解日本人什么样吗，我读过一本小说叫《白色巨塔》，是医学界。

受访者： 我知道这个小说，但我没看过

刘书博老师： 你看看特别像，我觉得可能跟我们儒家东方文化有关系的、封建文化的

受访者： 我刚才还没说完。就是我觉得现在的年轻人90后，就比如像XXX（受访者的一位同事）啊，像他们这一年代就是我们基本上就没看到他们再去混圈子。但是我不排除他们自己有自己的圈子，但是我觉得好像他们已经不是特别在意这个事情了。所以我觉得我们80后，像我，我觉得我们圈子里面就玩的比较好的，基本上是从75~88年左右。90后的也已经成老师了，90后也有成副教授的了。我就发现他们没有特别的想要迎合我们80后或者70。

黄秋莉： 还有别的吗？

受访者： 我说了2点了，我觉得应该就是这些，我感受比较深的。

黄秋莉： 那您在那边待了多久呀？

受访者： 我联合培养待了一年，然后访学待了一年，相当于是两年。

黄秋莉： 那您在那边做研究的一个状态和在国内做研究的一个状态，有什么差异嘛

受访者： 我觉得不能直接这样去回答，因为你的目标不一样，因为在那边访学我不需要上课，我也不需要授课。所以相当于我想干嘛就干嘛，在这边的话，因为你必须要上课嘛，然后你还有社会服务，所以可能没有办法直接比较。

黄秋莉： 那您在两种状况下自己的感受怎么样？

受访者： 那边我就觉得时间都是属于我自己的。

刘书博老师： 对，他在那边也不是一个全职教师的工作内容。

黄秋莉： 可能在那边就像我们做博士研究生一样

受访者： 还不是。因为没有老师管我，所以非常的自由、更自由，一天24小时想干嘛干嘛

黄秋莉： 还有一个就是比较重要的问题，就是梦娜想问的，关于非升即走的制度嘛，您可能不是，但是咱们身边也有。那您是怎么评价这个制度？

受访者： 首先我觉得非升即走制度我自己个人并不反对。我觉得什么东西都得有规则，你在企业里面工作你达不到业绩，你不一样要被淘汰嘛，对吧？所以我觉得这个制度本身没什么问题。我觉得没有问题是基于这样几个前提的：第一，你标准要明确，你签订合同的这两方要有充分的沟通，就说你不能赖账，你也不能突然提高要求。我觉得这是非常重要的一点，不能说当时签订的时候是三篇文章然后现在改成五篇了。一定要人和制度比，而且制度在至少在一个人的周期当中应该是保持不变的，不能人和人比说。比如说要求3年之后你升不到副教授再走，我觉得这是有点问题了。因为在国外确实也是升不到副教授走，但是副教授是有一个制度标准的呀，你只要达到制度标准没有什么特别大的问题，就可以留下来

第二。我觉得问题在于这个制度背后的支撑。就一个制度你去实施的过程当中是需要有一些支撑点的。我觉得能够具备这些支撑点，制度运行才是没问题的。那什么支撑点呢？比如年轻老师给他三年的时间或者给他六年的时间，那在这六年的时间里面他的核心工作是什么，哪些事情是他要做的，哪些事情是可以不用他做的？不能说你必须要达到这些文章的同时，你又塞给他很多很多的本来可以不需要他来做的事情，我觉得这一点也很重要。假设我们有个极端的情况，你让他六年发多少篇文章，那同时你让他上很多很多的课，你让他去做很多很多的服务。那显然这个制度就不合理了，但你说这个制度本身有问题吗？没问题啊，对吧？但是制度实施过程当中是有问题的

黄秋莉： 那跟我们这个中国特色有什么关联吗？

受访者： 中国特色就是你工作没有严格的边界呀，没有严格的job description啊——哪个是你该做的、哪个是你不该做的。在国外，不是我做的，我可以say no。在国内不是，非升即走不是取决你的论文的，你论文达标了要跟人比呀，那你人家做的服务你没做，虽然你论文比他发的多，可能留下的是另外一个人。所以在国内非常棘手，你既要搞论文，又要搞论文之外的其他的东西。那很多时候就是因为时间上的问题。

但是我觉得非升即走这个制度制度本身没问题

黄秋莉： 它的合理性第一个是激励嘛

受访者： 第一个不是激励，第一个就是这个制度本身你要沟通好，制度要有持续性，要确实是一项制度，不能是人为的东西。

第二就是说制度实施过程当中，你更多的是应该让这些人能够留下来，而不是说留不留下来是你自己的造化。但是在这几年时间里面你要做这些，你要做这些，你要做这些···

黄秋莉： 就是达到这些标准，它就是一个好的制度。

受访者： 我觉得是

黄秋莉： 那它的一个好的地方就是达到这些之后，它能够达到的一个什么效果？

受访者： 我们现在的假设就是这些老师他是有内驱力的，虽然他现在是有外在力让他去做更好的东西，那如果说老师有内驱力，那我觉得就是可以有很多很多的论文、很多很多的产出、很多很多的科研，然后教学方面也不差。我觉得这是好的。

所以这个制度本身我觉得没问题。而且制度如果实施的好是可以有很好的效果的。但问题在于我们制度本身的沟通的问题，以及支撑制度在运行过程当中的一些问题。

黄秋莉： 还有一个问题就是关于发中文期刊和英文期刊，您觉得您在投这两类期刊的时候有什么不一样的地方？

受访者：我觉得中文期刊现在越来越难投，我现在不投中文了

刘书博老师：难投是为啥？就是拒稿率高吗？为啥难投啊？

受访者： 我觉得内卷的很厉害，就是期刊的数量少，投稿人又多，这不就内卷吗？然后国外的话杂志多，你有很多可选的余地。

黄秋莉： 可是我们学校层面是对英文期刊的认可度会更高一点。

受访者： 没有，现在中文变得越来越高了，现在都是讲中国故事啊，发中文文章啊。但是我觉得至少对我而言，我不在意。就像我刚刚就讲了嘛，如果你想在制度当中存活下去，你就要让你自己变得不可替代、重要和稀缺。

这话怎么说呢···就是可怜之人必有可恨之处。就是如果你在某个制度下不能够存活，我觉得最主要是找你自己的原因

黄秋莉： 明白，我好像没有别的问题了。

刘书博老师： 我觉得也差不多了，我觉得非常充分

# 受访者6

刘书博老师：我们就开始，然后我这边做一个录音，会把稿子腾出来之后发给魏教授去看。如果有不妥的地方再去修改，然后会做脱敏化处理。那我就先来问一些问题。首先第一个问题就是那魏教授当时经过不同学校的学习，从河南老家到陕西，然后又到上海。那当时就是怎么样确定自己的学术志向？为什么想要选择走学术这条道路？还是说有一些重要的人在影响，还是自己有一些不同的想法。

受访者：因为我原来上学的时候我不是高中考上来的，我当年是读的一个中等师范，那个时候没有上高中，就是上师范。上师范只有一个保送的机会，我好不容易有了个保送的机会，那个师范是培养小学教师的。但是我是有了这样一个机会，然后没有机会选择学校，那时候我就读了师范大学，在本省的师范大学，那是固定的定点的、专业也是固定的，就是教育管理，那时候教育管理刚刚开的一个专业

这是刚开始上大学，那读研究生是顺理成章的。那我稍微的转了一点（专业），就是从教育学转到心理学，但是其实也算是相关专业。因为本科学的教育和心理学不分家的，学完心理学之后想继续的往企业管理、往管理学方面靠。因为从就业呀各种角度来想，那就顺理成章的就是考到人力资源管理这一个方向。它也算心理学的一个非常紧密的一个相关专业。那么就考到了复旦的企业管理，企业管理主要的方向是人力资源和组织行为学，这还是在我原来的基础上，这是专业的背景啊。

那为什么会走上学术这条路呢？刚开始我是没打算去做学术的，因为在那个时候读了博士之后，大部分人是不做学术的，我也是没打算做学术的。当年我就觉得我一直在师范大学里面读书，就好不容易跳出来师范大学，就决定不做老师的。不做老师干嘛的？那就去企业里面吧。

那么我当时在博士上学期间，就跟着一些老师在外边给人家做咨询项目。当时也没有怎么找工作，就是我帮他们做过（咨询）的一个企业正好在招人，那我就先跟他们聊一下吧，然后他们说咱们都很熟悉了，已经帮他们做两个月了，他说找外边的一个顾问来跟我谈。他外边的顾问是长江商学院的副院长，是一个教授。他跟我谈完之后呢，他说他觉得我还是挺适合做研究的，他说长江（商学院）有这样一个职位，问我要不要考虑。

当时我就想了一段时间为什么要做研究呢？那个时候完全没有想清楚到底是做研究还是不做研究，因为最初想的是不做研究，要去企业去的。那么在那个时候我是在毕业之前发了两篇文章，其中一篇是《管理科学学报》。发这两篇文章之后，他们都问我要不要做研究，因为我当时做的那个毕业论文，我觉得做的还挺好。然后当时用比较规范的实证的方法来做，那个方法呢其实大家都不太懂，老师啊、学生啊都不太懂，我也是自己摸索的。当时是现在香港中文大学的罗胜强教授给我们开了这样一门课。当时找不到研究方法，然后讲了这种结构方程模型啊之类的东西，上的时候没怎么好好上，也没怎么学的懂。但是呢当我开始写毕业论文的时候，我当时学心理学的时候有这样的一些基础，我就觉得应该做实证。做实证的话用最前沿的方法，那就用他的方法。那个时候我就把他当时的教材、发的一些材料翻出来，似懂非懂的看了，知道了大致情况。然后有的时候碰到一些技术上难关，就发邮件问他，他给我一些指导，然后就做了东西，做了一个实证的东西。当时做的应该还算比较好。那么那些老师啊，复旦的老师和上交大的老师，他们觉得做的这个东西还挺好的，要不要留校啊？我说我原来没考虑过留校，不是要到企业去的吗，但那个胡老师说要不要留校啊。胡老师是我们专业的那个带头人，我后来想了一段时间，我想想也觉得挺好，我觉得自己做研究也还是有点感触的，我基本上有了留校的意愿。

但是呢刚开始走流程的时候发现留不了，并不是说你想留就能留的了的，那到最后没有留下来。那么同时交大的一个老师啊，他是我们同行，那个他说，“诶，要不要来我们这里来呀，我们这地方也挺缺你这样的人的”，然后我就去了跟他聊过一次，他也是在走流程，走下来也留不下。

但是呢他影响了我对职业的一个选择，就是原来根本就不想去学校的。经过这一趟折腾之后，我觉得去学校也行，所以说我更加的犹豫了是去学校还是企业。因为想有去学校的心，也有了去企业的心。在这种情况下，长江商学院教授说，“来长江吧”，我知道长江是商学院，我一想长江就介于高校和企业之间，那我就去了，当时我就想着反正是到了那个地方之后探索一下，还可以两个方向都可以走。然后我就去了。去了之后去刚开始还是做研究，研究做了几个月，跟着李XX老师，因为他是做行政的副院长，他说那边的那个人力资源经理辞职了没人干了，说我去到北京去帮一下人力资源的活儿。然后我就去代理去做他们人力资源经理，因为我原来学过这个东西。但是在北京做了几个月吧，大致有半年，就是年过了个年。

过了个年之后呢，我觉得我还是需要回上海，不能在老在那地方待着。因为刚刚开始我就没打算离开上海嘛。那个时候我就赶快招个人力资源经理，他说我就招个人把我替换下来我就可以走了，然后就拼命去找。很快面试了一些人之后，我当时那个时候主要的事情就帮他们面人，那个很多的当时的一些行政人员都是我招进去的。后来就给自己找了一个接手的，然后我就回到上海。

回到上海那就还是做研究。所以说接触了很多的企业的运营哈，虽然那个HR的东西是商学院的，其实是跟企业是一样的，企业的运营也做了很多的研究。后来又到中欧跟他一起去，他先去中欧，后来我觉得我们这个项目还要继续做，就跟他先后到了中欧。然后我们做了一些项目，这些项目呢基本上就是访谈企业家，或者做案例。那么在这个过程当中有几件事儿对我确实有影响。

第一件事儿是我把我原来的毕业论文的相关东西啊……，虽然我没有到高校，没有人要求我去发表。但是我就把它整理一下发表出来了，发表了很多文章哈，包括有三篇《管理科学学报》，然后还有一些《南开管理评论》，还有国际的SSCI。这是一个影响，影响什么呢？我觉得我能做研究啊，我觉得我做的可能比当时的那些去学校的人做的还好，因为发表来说比他们做的要好看一点。

这是第一个，应该是有了一些信心导致的。第二个呢，我刚才说你们项目的时候有一个好处哈，就是接触很多人，听人家讲人家的故事。我们在中欧的时候跟CCL合作，访谈了100位企业家。这100位基本上我全部都接触了，就全部访谈。我有一个感受，我觉得在企业里边做其实还挺不容易的。这是怎么不容易呢？首先一个就是不自由，让我了解了他们成长的轨迹等等，我觉得还是挺有价值。但是我自己就逐渐逐渐越加明显的感觉到我要回学校，因为自己感觉自己价值观当中最重要的一个东西是自由。就是不受约束的东西，但是到企业里边我就会发现那些企业里边的人呀，其实有很多的身不由己。跟学校里面的人有很明显的不同就是可能拿的薪水会高一些，但是所有拿薪水高的人都会更累、更忙。就是我看了这一百多号人之后我就会有一些自己的判断。

然后我想回学校时候呢就有了一些机会，我的一个合作者他回国做上海大学管理学院的院长。他就是说他这边需要一些人，然后需要跟我一起做，能够把商学院能够做的好一点哈。然后他就问我愿不愿意来。那个时候我已经有了想法，所以说我就顺理成章去了。当然他还招了一些别的人，这是我正式地跳到学校里边去，相当于自己想清楚了，我是这样走上这条道路的。所以说中间会有很多曲折的故事。

刘书博老师： 是，所以我听起来就是一开始很清晰地想要不在学校、想要去行业产业公司。然后呢由于机缘巧合呀，或者是有一些比如程序的一些阻碍呀，导致还是在学校。基于学校的平台接触了大量的企业，就了解了他们的真实面貌之后，然后会觉得自己更喜欢自由这样的一个价值。

那我就比较好奇的是像李XX老师给您的印象，或者说因为前面一直是在中国的大学，然后导师可能都是大陆的这样的背景。那李老师是新加坡的，那新加坡可能在管理学方面要领先于大陆。所以当时像李老师对您有什么样的影响吗？或者说当您跟他一块儿工作的时候，有哪一些是受到他的影响而产生的新的东西，或者改变了之前的东西？

受访者： 我觉得李老师是跟中欧和长江这样学校里面的教授是比较像的。那一类人都是这样、或者说都普遍具有这样特质，就是说这些人是既做研究又做教学又做社会……就是跟社会交流非常深入的这种服务或者是自己的研究吧。那这三个方面都同时并重的，不像我们现在商学院里边，年轻老师来了之后就必须90%的精力是在做研究、做学术、发文章。他们不是，我觉得他们可能也就1/3、1/3、1/3这种状态。那么我也跟李老师一起接触了很多的企业，然后他有很多的项目我都必须要去做的。那么在做的过程当中，我觉得确实我不太像那种纯做学术的气质和方式。但是这个是对自己后来是有影响的。

这种影响的话分两方面，一种方面就是说，并没有集中聚焦的做某种东西，而且心无旁骛的做下去。我觉得在学术学者的阶段啊，尤其早期阶段，心无旁骛的做一个点，然后把它挖深，这是非常重要的。但是我并没有那样去做的很深。因为我是觉得啊那些跟社会跟企业界深入交流可能是很重要的，所以说会花了不少的精力在跟社会接触，然后包括去外边做培训，所以说这三件事同时做的时候就会影响学术方面的深入。

但是反过来好的一个方面自己是相对来说比较综合一点，然后考虑问题会更加的跟企业界更像一点。

刘书博老师： 所以我听起来是长江，当然中欧那些老师我也接触过很多，他们多数是来自于美国那样的一个培养体系。现在应该主要几乎都是美国的培养体系。因为前面我们我们也做过访谈，就是在美国的体系当中似乎也有一些多样性。就比如说像哈佛大学，他们可能会对于这种奇思妙想呀，或者说对于创意的新的idea会更加的重视一些。那有一些学学校像哥伦比亚在研究上更专业、在管理学这一块儿更职业化。那我听起来如果中欧是1/3、1/3、1/3的话，那在哥伦比亚的标准看起来他不是那么专业。不知道您怎么看？就是这种多样性。

受访者： 中欧其实你也比较熟悉，中欧可能在他早期需要很多年纪较大一点的、教学经验比较丰富的人去开拓市场，所以说教学主导。那么后期呢稳住市场地位之后，他逐渐的走向全面。然后进来很多做学术的人，当然他希望做学术的人既能做学术，就是国际的发表，又能去影响企业界，但是实际上做不到的。你也知道当时像XX这样的人他们应该是两方面兼顾的不错的。当然还有一些人比如说那个XX教授他们是学术做的更强一点，但是教学方面弱一点，就是逐渐逐渐教师越来越全面。那么现在进了很多年轻人，年轻人肯定是市场会弱一点，但是他的学术会强一点，所以说到了后期呢，就各种各样的人都有了。都有的话，其实是全面的，我觉得中欧是这方面会强一些，因为还是比较理想的一种生态。

刘书博老师： 对。因为中欧现在像蔡教授就我当年的老板，他还是教学为主，然后像他一块儿进来的有一位美国的老师战略系的，然后他就教学可能太弱了，在当时那个环境下就没有办法留在中欧，那就去到清华了，他现在还在清华。所以我不知道你有没有这种感觉，就中欧他会觉得自己比北大清华要厉害

受访者： 对。他觉得教师的背景更强。

刘书博老师： 对。那现在可能又过了这么长时间，因为我是15年离开的，就七年了，不知道现在中欧那边有什么新的一些变化。我看那个网站上是招了不少新的老师啊，年轻的，而且女教授还挺多的，招的新老师。可能管理学就是慢慢的阴盛阳衰会很清晰了。因为现在博士就是这样的一个（局面），研究生更过分。像我们学院研究生就是60个人，50个是女生，十个男生。（男生）考试考不过女生。那不知道您所在学院有没有也类似？

受访者： 差不多，女生为主，男生可能是战略的稍微还好一点，其他的方向都是女生为主。

刘书博老师： 明白。那我这边问题大概就这样啊，主要是您前面学术志向的一个确定。那后面我让两位博士生来主要问一些其他维度的问题。

吕梦娜：我就是就着您刚刚提到的一些话题，然后想继续了解一下。因为您其实是在零几年就开始做博士论文了，您那个时候就已经说要做前沿的，比如说要做实证，我想了解一下您那个时候是怎么得到的信息呢？就是说零几年的时候就已经比较注重方法上的训练了吗？

受访者： 我们的方法应该是来自微观领域的研究，所以说这跟我的学科背景有关系。我的硕士是心理学，我硕士论文就是用自己摸索的因素分析啊那些东西来做。甚至再往前，我本科论文也是用一些统计的东西做的。

吕梦娜： 那这个方法主要是跟美国学习的吗？

受访者： 不是。我们跟心理学有关系，心理学这个专业基本上是这样

吕梦娜： 所以相当于是之前的心理学的教育背景。

那您后来工作之后要主动的把博士能给发表出来。我想了解一下，您那个时候是一个什么样的想法呢？为什么要一定要把它给发表出来呢？

受访者： 其实我在长江（商学院）期间也是在做研究，那个时候我觉得我刚开始发那些论文还挺顺利的。所以说我就投一下，他们就本上就很顺利就能接受，那我就觉得那我就继续发嘛。那我那篇博士论文里边儿有一些东西我摘摘摘就发了，就是基本上非常顺理成章就发出来了。

吕梦娜： 我就是很好奇，您当时想要发就是这个动作的动机是什么呢？

受访者： 因为博士毕业之前就发了几篇，接下去本来就是一个做研究的……，我本来就在那做研究，发表对于做研究应该也是正常的。那时候跟李教授还有另外一个教授，那个教授当时马里兰回来的。我们三个基本上会在一起经常来做，像做研究设计呀，发问卷调查呀，都是在做研究的东西。然后我的博士论文是我自己的，没有跟他们合作。

吕梦娜： 明白。您后来到上海大学管理学院的时候，因为我看上海大学管理学院是08年成立的嘛。我觉得您应该是属于那种元老级的人物了，您可能也深度的参与到了学院的建设过程，那我就想了解一下在那个建设的过程当中大概是一个怎么样的发展。就比如说有没有一些参照的对象呀？因为可能上海更加国际化一点，比如说有没有参照美国？还是说比较体制内的？像咱们985、211这种高校的建设？我不太了解。

受访者： 没有。他是上大的管理学院，他是有一些历史的，只不过是在那个时候呢引进了一个国外的院长，他当时回来做院长，因为他当时跟学校有很多很多的协议，当时是有计划把这个打造出来一个比较有影响力的管理学院。他其实也是一个比较全面的人。就是除了学术性的研究，还有社会影响力的研究，这两方面他都在做，管理能力也很强。所以说他来了之后第一个需要引进一些人，要打造学校的影响力哈。所以说他是着力想引进一些人，这些人当然有一些资深一点的，有一些年轻的。当然我们现在回过头来看，现在上海大学脱颖而出的这些人都是在那个时候先后进来的。

吕梦娜： 那引进的这些人才有什么特点吗？比如说都是国外的，还是说咱们国内的清华北大？

受访者： 没有国外的。基本上是国内的背景的。

吕梦娜： 明白

受访者：因为当时学校在薪水上不够有竞争力。就是说国外没有太引进过这样的人才。虽然做过这方面的努力，但是最后没有多少结果。

吕梦娜： 哦，了解。您之前还有在美国的访学经历嘛，然后再结合您在上海大学也好或者XX大学也好，就是中美商学院，您觉得有什么不一样的地方吗？或者是有鲜明的相同点也可以，不同的地方也可以

受访者： 其实中欧是挺像西方的商学院的。博士项目很少，本科生也没有，主要是培养这种商学教育啊这样的培训，所以说差别很大。

吕梦娜： 那您可以比如说从工作的氛围上面，或者学校的氛围上面，还有学校管理方式方面能稍微再谈一谈吗？

受访者： 我觉得可能现在大家越来越清晰的知道学校之间的差异了，比如说对老师的要求和期望。现在体制内的学校对老师的期望就是你发UTD，这是唯一的要求。在六年之内就要发几篇UTD，这是唯一的要求，其他的似乎都不重要。

但是呢中欧不会是这样要求的，中欧的list十几年下来就是FT的，引导性的就是FT发表，其他的也没有说太多。大家心目当中都有一个标准——觉得什么是可以尊敬的杂志，什么是一般的杂志，都是差不多的。也没有说你一定要发多少篇东西。但是他们要求你上课一定能够站得住讲台，就是第二年你就得上课，上课你就得站得住，站不住的话，你就得走人。最低的要求你得不要被学生赶下来。然后，他们会有一系列的措施来帮一个人怎么样教学。

吕梦娜： 那这个在国内这种就很少，是吗？

受访者： 国内教学不重要啊。所以说就是理念上差别很大哦。

吕梦娜： 所以说您刚才也提到了中欧和美国比较像，那美国也是这种，比如说对于期刊的发表并没有特别的执着于UTD之类的？

受访者： 没有太多硬性的要求，比如说我在美国的时候，我那个合作教授，我那时候他在XX做副院长，然后我回来的时候他去了XX做院长，然后前两年去了XX做院长。其实他就没有几篇UTD的文章啊，我记得也就是两篇吧。然后他就成了教授，发表不是那么多。不是说我们一定要发六篇到八篇等等，然后你要评这个评那个的。

我觉得如果说没有说你要评这个，没有说要评那个，大家就会发自内心的去做他觉得应该做的事儿。

那现在我们说这个东西可能有点不太好哈，就是你要评职称，你要评杰青，再一个你要申请项目等等哈。有了这么多的东西在前面摆着，就会让这些有希望拿到那些东西的人啊不淡定了。然后就去做那些东西去了。这事实上就偏离了市场。

国外都是没有东西的。然后他们就可以想更好的为市场服务，把我的学术的知名度、教学的知名度，然后还有为社会服务的这方面的影响力给打出来。然后东西其实是接地气的东西。事实上我们指挥棒在前面引导着做了很多东西是不见得接地气的。我们学院有一个教授是做管科的，他是做航空的，他教授被基金委给拿来做标杆，为什么呢？因为他做的研究就是直接能够转化成机场的调度啊什么东西的，软件呀，立刻就可以产生效果的。为什么做标杆呢？因为大家都做不到嘛。

吕梦娜： 是，所以现在能感觉到，我们一方面在引导，比如说考核呀，比如说项目，论文各方面的。但是另一方面我们的课题好像又在引导我们要做扎根实践的研究，就好像是有点脱节的，是不是？

刘书博老师： 我觉得有点矛盾吧

受访者： 研究与实践本来就脱节的。

刘书博老师：像我们学校今年新出了一个正教授的职称评比办法，就说你必须是四篇第一作者的a类文章，很清晰。然后你像在中欧，不管是第几作者，甚至不是通讯和第一。只要你有一个固定的研究团队，那他们都会觉得是非常认可的。而且他们主要还是看文章的内容。那我们现在好像国内这就是数数啊。虽然去五维，但是我感觉不到大家行为当中任何的变化，甚至我们学校还是逆天下变化而行，变得更加的这种量化，更加的清晰。

受访者： 这就是跟我们从小接受的教育是一样的。我们的教育从小学到大学都是相对比较，你看你排第几名？然后就现在我们在体制内也是相对比较，你看我们现在只有这么多的（名额），然后你排在这个，你就拿不到等等哈。为什么非得要跟别人比呢？

刘书博老师： 对，对对，那就是文无第一嘛，它不是一个对抗性的，我们其实把一些非对抗的东西一定要变成对抗性的，就像我们好多事情都是用战争的话语在表述，跟那种急行军似的。所以就好像我们在近现代充满了战争的那种紧迫感，或者是你死我活的这种丛林。

受访者： 就要让大家互相卷。

刘书博老师： 是的。

受访者： 我觉得是从上到下的一个理念，上面的理念让下面不得不去追着卷，我就觉得有一些商学院的院长……曾经XX老师有一段时间是这样能做到一点是，我不按你的套路走。我觉得清华也是有能力做到这一点的——我做我的，我不按你的套路去走，最后能够回归到本源，或者说一定程度上回归到本源。我觉得是如果能往这个方向、如果说有这样比较有智慧的院长往方面引领，我觉得这些学校是一定程度上能做到的，而且是应该做的。但是其他学校根本做不到。

刘书博老师： 因为清华北大这样的是有自己的文化资本和影响力，所以他们能够做到。

受访者： 他们不用评，他们不用看上面的脸色，就不评。你能把我怎么样？对吧？他不评的话就不受这些指挥棒、这些数量来制约了，他就可以做他觉得对的（事情）。

我觉得大家都知道什么是对的，只是说没有办法。

吕梦娜： 现在的情况好像就是北大清华会在评的方面好像做的更加激进一点。就比如说我们很自然想到“非升即走”嘛，似乎像清华他们老人也是用新办法来进行约束，不行就走了，就感觉好像会更加激进一点

受访者： 哎，可能还是没有办法哈。我觉得至少可以做到教授之后，可以适度的去不要……但其实可能也不行。他们还有很多的杰青啊等着他们长期地追求

吕梦娜： 是的。我们之前有访谈过了解到，就是像教授可能也是一刻也停不下来，因为有年轻的人发表太多就被卷起来了嘛，然后也有挺大紧迫感的。

刘书博老师：前两天我们学校中层干部述职嘛，然后我就听各个院长在讲述他们的绩效、工作成绩的时候，自然比如说我们林院长那要拿出来跟经济学院的可能就很杰青那个level的人去对比。所以他虽然到了教授、到了院长，他还是有一个区别心、对比欲嘛。他他还是会被裹挟着要进行新的卷法。他依然是卷。

受访者：这两次学科评比给搞得越来越就样子，越来越强化。

吕梦娜： 是的，魏老师刚刚接着那个“非升即走”的话题再聊一聊，您对这个现象有什么关注没有。就是您怎么看待它为什么会在我们高校这么快的、挺普遍的，就推广起来了。

受访者： 我觉得还是上面指挥棒吧，排名制来导致的。然后大家都想让自己的排名更高一点，学校的校长、学院的院长都想要排名来显示自己的政绩。那就没有办法呀，从而造成我们绩效考核里边的末位淘汰。

末位淘汰好不好呢？我们上课都在讲，当然是好的啊，它能够促进企业绩效。但是现在为什么大家都不弄了呢？现在绩效考核都要搞OKR了，都不搞末尾淘汰了呢？就是还是负面的东西多吧。我觉得就是会有一个阶段性的，但是企业可以不搞末位淘汰，他们还有自主权。然后我们学校没有自主权，学校就是受上面的影响。

吕梦娜： 对，是这样，您了解XX大学，像比如说经济与管理学院这个政策是什么时候开始的呢？或者说政策具体情况是怎么样？

受访者： 那我们2010年左右的时候就有了，但是那个时候执行的不是很严格。就是说没完成也可以留下来，但是近五年可能是严格执行。所以说今年、去年年底、今年年初。反正就是现在这个时候吧，就是第一波进来的那些非升即走签的严格合同的人已经在操作了，那些人就已经走了。

吕梦娜： 没达成要求吗？

受访者： 我们的要求很高，他们应该很多人达不到。但是达不到的话，我们觉得依然是我们行业当中非常受尊敬的学者，我们再引进的人似乎还达不到这种情况状态，但是这个东西你既然有制度了，还得走

吕梦娜： 您了解他们都走哪儿去了吗？

受访者：有去那个香港的学校在深圳校区的，还有去哈工大的，反正是往不那么有吸引力的地方或者稍微往下一点的学校走。哈工大的还给了个副教授，我们这边连讲师都留不下来

吕梦娜：那这个政策是不是每年都在变化呢？因为我听说有的学校好像一年一变，有点朝令夕改的感觉

受访者： 可能变得越来越严。

吕梦娜： 对，就比如说今年的要求，假如说是四篇，明年可能就五篇的那种的

受访者： 最上一层的这些学校可能不会再严了吧

吕梦娜： 为什么呢？

受访者：因为最上面这一层学校他们的竞争格局是差不多的。他们最早开始这个东西一步到位的，他们的标准已经达到顶峰了，跟国外是一样的。

但是再变的可能是二流的或者说再往下的，然后他们觉得原来自己下不了手，现在觉得市场上的人多了，他可以再狠一点。

吕梦娜：我后面还想问一下关于您研究方面啊。因为我看到您简历上面申请了很多的基金项目，然后就想请教一下您在申请课题的过程是怎么样的，比如说这之中有没有包括一些您感觉到比较意外的呀，或者挺具有中国特色的一些事情发生。

受访者： 我申请项目一直都不是很顺利的

吕梦娜： 但是感觉很多啊

受访者： 还是不多的，还是比较少。因为在我年龄的这些人当中，可能我是做的不好的，申请项目一直都不是很顺利。一个原因在于我的选题有点过于微观吧。就OB的主题可能不是那么地有市场；另外一个也是可能我们的体制……可能要讲很多的社会资本，我自己可能社会资本相对来说会弱一些。

吕梦娜： 了解，刚刚您提到选题方面过于微观，我们之前也了解过一些老师，特别也是做OB领域的，就感觉好像会对我们学科更加地不太友好一点。所以这方面您会做一些调整吗？比如说往宏观、中观靠一靠之类的。

受访者： 对，我一直在往这方面转，所以说这个研究也是不够深入。那么作为一个学者，尤其是在你们刚走上社会、刚走出学校的时候，研究在未来十年之内一定要聚焦，对吧？就是聚焦是非常重要的，但是我一直都不聚焦，所以一直在转，一直在转。这就导致我的研究不够深入，或者说没有在某一个领域树立一个品牌和标杆形象。那别人想到你的时候就会说他是做什么的啊，我就会觉得有点模糊，模糊的时候其实就不是很好。但是我一直在这样做，我就觉得还是跟原来在中欧、在体制之外学校的经历是有关系的。我觉得应该尽可能的做有用的、有价值的或者说企业感兴趣的东西。

然后我原来是根据我的受教育的背景我所能做的。但是可能并不是我最想做的，那我就逐渐逐渐往我最想做的在靠。原来是我是一直算是做领导力的，原来是做基层的、中层的领导，后来就到了CEO层面的领导，甚至是TMT的团队和创业团队，基本上会在讲他们这些人，研究对象会发生一点变化。那么到了高层团队之后呢，我对企业的理解会更多一点，那顺应的也会更容易转到一些偏战略的东西。比如说平台呀，比如说什么类似，就是社会上热的东西。

吕梦娜： 所以说我们在申请基金的时候就是会有一些比如说学科或者是主题的一些偏向。有一些主题或者学科相当于天生会更加的容易得到这个项目，是这样吗？

受访者： 我觉得在中国是有一些这样。

吕梦娜： 了解。您刚刚还提到了一个社会资本，就是这里面是不是会在评审的过程中有一些什么故事呢？您了解吗？

受访者：我们的自科是实名制的嘛，那肯定会有影响。

吕梦娜：就您刚刚也提到您在关注领导力嘛，我觉得您也是在OBHR大领域里面已经做了很多年了。然后我就想了解一下您觉得这个学科或者是这个研究领域在中国的发展情况是怎么样的。比如说它具有哪些特色呀，或者未来能有哪种趋势呀，您可以基于您的经验谈一谈。

受访者： OBHR领域呢其实越来越小众。我觉得原来其实做的人很多，现在做的人也很多，但是走到一定程度上就发现走不下去了。很多人比如说到了身边的一些人哈，到了教授啊，他要申请重点项目的时候就发现没有了，所有的重点项目都不是微观的，都是比较宏观的。而且就算他用比较微观的思路去申请宏观的东西，到答辩的时候人家评委问的问题自己就搞不懂，就没法交流，大家的思路就不一样。比如说到创新他就会说个体的创造力，跟人家说的根本就不在一个层面上。所以说呢就会导致大家在条件允许的情况下转一转、往宏观偏一偏。当然其实我了解不是很多啊，这几年一直在讲徐淑英老师在方法论普及方面的贡献。前两年有很多很多的争论，我没有仔细看这些争论的在说什么，但是总是觉得哈，问题导向和方法导向这两个东西是有争论的。

OB更多的是方法导向的多一点，问题导向有点太小，问题导向可能是少一些，或者说我们问题的话跟企业的运营的关联度小一点。还有比如说在中国越宏观的越受关注，比如说中国的经济学就比西方学校里面的经济学的地位要高，因为它非常宏观

吕梦娜： 对，我们好像比较追求一种类似于普世的那种价值感，但是OB这种微观的好像就不太吃香了。

受访者： 还有就是我觉得现在一些导向就会发现，市场经济发达的时候，企业管理类的学科可能还是相对繁荣一点，市场经济弱势一点的时候、计划经济强势一点的时候，我们企业都变成了弱势群体了，尤其近三年企业都成弱势群体了，研究企业的人的地位也在下降，不如研究政府的人、经济的人、行业的人。

吕梦娜： 明白，所以我们研究真的跟国家也好、时代也好，有很大的关联性。后面就是官网上提到您的研究嘛，我看到非常长的list，在很好的期刊上也有发表。然后就想了解一下，就以您经验来看，在好的期刊上或者说顶级期刊上发表文章需要具备哪些条件呢？

受访者： 好的期刊分中文和英文。英文的期刊基本上是属于理论导向，中文的期刊应该是实践导向。第一个筛选要按导向，在此基础上然后再要求你全面都做的很好。西方的期刊要理论导向的话，那你首先必须要对最顶级的那些文章的一些模式要非常熟悉，相应的背景，尤其是，什么是理论、什么是有理论价值的有理论贡献的研究要非常的清楚。然后了解了我知道怎么做了，知道他一篇文章为什么好，为什么能发表。这是第一步的。这个东西都不知道，那你标准都没有搞清楚，对吧？

知道了他为什么好之后相对来说就好做了。但是要真正做的时候就会发现其实做好的研究，资源是非常的重要的，我没有那么多的资源，没有那么多实证的东西，知道怎么好你也做不出来呀。 所以说有很多人有很多的资源，可以拿到很高端的数据、别人没有的数据，然后你这个东西不就是容易做了嘛。所以说这两个东西中间的怎么样？弥合这两个东西就是个人的能力嘛。能力相对来说比如说写作呀，逻辑呀等等，但是那两个头都得有。

吕梦娜： 明白，您刚刚提到英文的顶级期刊上主要是指美国的这些期刊，是吗？

受访者：对。

吕梦娜：因为我们之前还听说过，包括上课的时候也说好像有一个很重要的能力。除了你提到理论，包括数据就越fancy的数据就越能够发表。然后就导致大家就卷起来了，就好像比谁的数据更加难得那种。那您自身而言的话，您觉得中外文的这种顶级期刊上发表哈，有什么优劣势吗？就对您个人而言的话。

受访者：  我觉得选题很重要，如果你选择题目是国内比较看重的题目，那在国内是发的机会会大一些。如果说你的选题是国内所不在乎的一个主题，时候就尽最大努力去发国外。

吕梦娜： 是，确实。我们也能够发现好像是有这样的一个导向，先看国内的合不合适，然后再去国外再看一看。

受访者： 对，是这样。但是竞争力都很强，都是不同的人在做相同的事儿。

吕梦娜： 魏老师，我还有一个最后一个问题，是关于你作为导师这样一个身份角色的一些问题哈。就是您有没有在指导学生的过程当中，遇到特别适合做科研或者是适合走上学术道路的人呢？他们大概有什么样的特征呢？

受访者：首先因为我们这个东西考的大部分是智商，所以说智商相关的东西就是说学习好，什么学习呢？英语好，然后逻辑能力强。逻辑能力肯定是最重要的，因为写东西的逻辑能力一句一句的，是一个都不能多，一个都不能少。

然后读文献读的快，领悟的比较准确。

另外一个就是非智商的东西，怎么样跟导师合作的好等等，这个东西每个人是不一样的。

吕梦娜： 了解，那您反过来有没有遇到一些觉得不太适合做科研，就是不太建议他未来走上学术道路的这些人，他们大概是哪些共同点？

受访者： 我们现在都是硕博连读，所以说硕士一年多的时间给大家一个考察，不适合的时候就劝退，或者他自己退出。

吕梦娜： 你们学校劝退比例高吗？我们好像不太有，除非自己转会有。

受访者： 确实有的年份高，有的年份不高，但是不确定，然后跟这一两年学校学院的政策是有关系的。我这边还好，我基本上每年都会有。

吕梦娜：就不管学校政策如何哈，就您自己觉得有没有一些学生？比如说可能他们有一些共同的特质，但这些特质似乎不太适合做科研，不适合走向学术。有这种吗？

受访者： 有，逻辑能力不强等。我们反正是通过练习让他写一篇文章，看一看从头到尾的东西。在这个过程当中他总是不能切入正题，总是抓小放大呀，抓不住重点，看东西快慢呀等等。 但是我觉得非常重要的是想清楚了没有，有没有决心和信念。

吕梦娜： 明白。好的，谢谢魏老师，然后我这边的问题先问到这里，非常感谢您。

黄秋莉：魏老师，您好。就是之前刘老师提到您的时候就给我们介绍说您是一个非常优秀的学者，然后就一再跟我们强调说要借此机会向您好好的吸取一些经验嘛。所以可能就是有一些比较这种学术方面的问题想向您请教一下。

受访者： 可能有很多年轻老师更厉害。

黄秋莉： 没有。就是我觉得刘老师的评价是非常的中肯吧，他是第一次就是跟我们强调说非常优秀。然后我们也可以看到嘛，你基本上每年都有学术成果的产出，就感觉你好像就没有遇到过什么瓶颈期一样，就一直有不断的新点子冒出来，然后还能把新点子变成非常优秀的学术成果嘛。那可能我第一个问题就是想问一下，您是如何获得这些研究idea的呢？

受访者： 我这边倒是想做的东西比较多，因为我在不停的扩展自己原有的那个关注点。比如说我原来关注“心理契约”，关注做了一段时间，后来逐渐逐渐聚焦到领导力。领导力的东西，其实他有很多很多新的不同的领导。然后在领导力的角度上继续的开拓的时候，他总会有很多相关的东西，就看着看着就有相关的新的观点的产生。

第一个就是跟自己接触到的资源是有关系。比如说我做领导力的时候有咨询公司他们有很多的数据愿意分享给我，然后我就会想我怎么样把他的数据用起来。这个时候就会把自己原来的想法再开拓开拓，原来会在讲方面的一个东西，对吧？各种各样的领导风格。那现在就会讲，因为他们的数据会偏领导力培养的，那我就会往这方面这方面去偏一点，偏过来之后就会发现跟原来是有不同的。那么这里边当你看文献的时候，你要集中在一个点里面，其实有很多很多可以做的，只要你有相应的资源可以保证你去做调查。你想法总是会有很多的。我觉得是一直都有，除非你不看新的文献了。因为他的学术领域很相关的热点，他总是有层出不穷往外冒。

第二个跟方法是有关系。就是你原来用统计用回归用结构方程模型，但是现在你就会发现有很多新的模式出来，然后模式出来了之后，就发现你原来的一些想法一下子就可以再冒出来很多，在那基础上继续开发，对吧？然后就可以在你原来的东西上继续在做出来一些新的东西。有了一些方法出现，就会导致你原有的研究内容它会更加的热闹一点，就会出现更多的内容。

一个是方法引发的内容，一个是研究主题相关的一些topic它就会出现。

黄秋莉： 看起来好像是有一个理论、方法、实践三者之间不断的迭代循环，然后就促进了idea不断的就体现出来了

受访者： 提炼的挺好。

黄秋莉： 有一个好的idea就有好的研究计划了嘛，就对我自己而言，在付诸实践的过程当中还是比较困难的。那您能够保持这么快速的产出的话，您是怎么做到把自己的idea付出实践的呢？

受访者：  所以说很多大牛都是有团队，然后这些团队都是年轻的老师。那么他相应的来说他就可以做很多东西，对吧？因为这些年轻的老师本身的生产率都很高，但是我没有那么多这些资源，我有几个学生还是比较能干的。所以说一个学生会做一部分内容，就会把自己的这些东西给落地下去，就是要不停的培养能干的学生出来。

黄秋莉： 就个人的力量还是比较少的。

受访者： 一个人能做的效率不是那么高。

黄秋莉： 其实之前我和梦娜就是也交流过，然后她就觉得同时兼顾几个项目好困难呀。然后有的时候又看到一些老师同时进行三个项目呀，甚至十个项目的都有。那您在过程当中有没有一些什么好的就是方法呀？或者是您觉得困难吗？有这种经验分享一下吗？

受访者： 我原来有一次我在国外的时候听说xxx，我们领域的能同时开八十多个项目，就是他有一个团队的话就问题不大了。

黄秋莉： 可能作为一个学者，不仅仅是知识方面嘛。还有一个管理方面。

刘书博老师： 我这边补充一下，就是前面魏老师也说了学术能力的考核维度，就是学习能力方面，就有一些学生确实他阅读速度很快，而且他领悟的很快。所以老师带的学生多了之后会发现这种能力的参差。那确实是有一些天分表达出来，理解力、逻辑能力这些东西基础不行，然后经过锻炼有一些学生就会快速的提升，那有一些就会依然比较慢。所以其实博士阶段有时候也是在认识自己的这样的一个阶段。所以其实我觉得技巧是一方面，能力也是一方面，然后还有就是习惯吧。

受访者： 擅长做什么。

刘书博老师： 对对。你像有我见过有一个大牛，是一个老先生，八十多岁了，然后每天他都是很规律的一天到晚都在思考，都在写作。我跟他聊天，我就觉得八十多岁的脑子也非常的sharp，你说什么他立马就知道get到那个点。所以有时候我觉得人的智慧是有层次的。

黄秋莉： 有可能还是得接受自己的平凡。

刘书博老师：需要去加强学习的强度。人是在压力环境下才可以成长的，没有压力是不行的。

受访者： 就是知道自己能做什么，不能做什么。要完成一篇文章，它是需要很多东西，有的学生擅长数据分析吧，数据分析并不是说你把它拿进去算出来，有的时候需要一些清理，有的时候需要建模，有的需要构思。但有的同学他是擅长文献的迅速的理解，把握的重要的点，还有的他是适合写作等等，每个人都有自己的特点。但是我们原来就想一篇文章重要的不就是写作吗？但其实你就会发现写作阶段文章已经基本上完成了一大半。什么东西都想好了，构思已经放在那里，模型都放在那里了。那就再写，其实相对来说就不难了，如果是一个没有价值的模型，写的能力再强也不行。因此全过程走下来，你会觉得作为一个学生，构建模型他确实存在很多的不足，就需要有人指导。

黄秋莉： 明白。就是您发了很多成果，然后又是教授嘛，就对论文有一定的就是有评价能力嘛。你刚刚提到有价值的研究，那您心中的一个标准是什么呢？就是一看就是一个好的研究的这种感觉是什么？

受访者： 比较有价值的研究就是看了之后让你觉得有没有新的收获，作者肯定知道的。他写这篇文章当中他有没有创新点，他自己是知道的，我是真有创新点还是硬说我有创新点？反正文章看了之后你扫一遍就知道，这篇文章就是一个常规的做个练习的文章，就算别人他说别人没做过等等的也不过如此。但是有很多的文章确实是好的，文章不光写的好，你就看创新点那几个点，你看他写的你就知道，他这里边有很多第一次，有很多是突破以往的局限的。他的东西你看着就很开心，你就会知道有很多的新东西都是值得自己学的。还有很多文章是理论性的，前几天我在看这些文章，就是和你的主题我觉得挺像的。昨天和今天这几天一直都在看了，就是跟你是一样的身份怎么建构起来的。他就给你的一个理论的东西往这一放，你就发现我肯定想不出来，然后他怎么能想这么丰富？他的故事就是他的他的思维一层一层一层，那你看了之后有很多的收获，这就是好东西。但有些东西看了之后你觉得虽然没人做，他做了，但是模式上、主题上什么都没有。新意就是跟另外一篇文章是一样的，而且不会让你产生兴奋的，那就不是一个很好的文章，就是一般的文章，哪怕他发的再好。但是我看了这篇文章，一直觉得：“哦，原来都没有这样的。”他思路、他点子、模式就觉得让你一下子眼前一亮，那就好。

黄秋莉：就听起来可能要有一些新的洞见在里面，我听起来挺学术方面的评价的。那让我想起来就是之前有一个老师对陈春花老师的一个评价。就是陈春花老师嘛，他可能在企业界会颇受认可。但是就是可能有好的评价，也有一些负面的争议嘛。有的老师是从学术的方面来看陈春花老师就觉得nothing new，但是这些老板们又对他有高度的评价，似乎这里面是有一个差异和矛盾在里面的。那从您的角度来看的话有什么样的评价呢？

受访者： 我没有太仔细的读陈春花老师写的东西哈，虽然我也关注他的公众号，但是没有仔细的读。确实我觉得在实践的角度来说，就是跨界角度上来说，他是我们现在最成功的一个跨界学者哈。但是如果说从高端的发表的角度去讲，肯定他的想法在理论的层面上还是缺一些东西，因此有很多发表的这些学者可能会不太认可他的东西。但是我们不可以否认的话，他有很多的智慧，包括管理方面的一些系统性的思考。但是这些思考，也许他就不觉得要去在理论上做什么深化，那是两个层次。我们现在管理学，就是理论和实践是脱节的呀，是吧？

黄秋莉： 我现在看一些文献，是有这样一个批评哈。那您怎么去回应外界说的理论和实践脱节的这样一个批评吗？您会觉得这是一个问题吗？

受访者： 我觉得是一个问题。就是本来这是理论，这是实践，它本来就是脱节的。那如果有人能把两边的东西很好的平衡好，甚至把它关联起来的话，那人确实是我们两届都应该认可。那这样的人相对来说很少，要么有人在方面就会强一点，要么有人在方面强一点，单方面强的人有很多。但是怎么样把连接起来，我觉得比如说有些是可以的，比如说搞会计的、财务的、战略的，相对来说他跨的相对好一点。比如说中欧的那个xxx，后来去菜鸟。

刘书博老师： 对，他去阿里的那个菜鸟当部门老大吧，但是他后来又回中欧了，但是现在他参加一些论坛，我感觉他整个人的那个说话方式气质变了，也可能是自己刻意在改变。以前是一个台湾的柔柔弱弱的，就是那种受人欺负的感觉，现在就搞得有点硬了那种感觉。他也发那个也发了好多好的文章，在战略这一块儿而且我觉得他是有自己的想法。

受访者： 战略相对来说跟实践能够结合的点相对多一点。

刘书博老师： 对，因为战略也是本身就是挺抽象的、挺理论的。

受访者： 但陈春花老师做了很多是business的东西，business的东西脱节是非常严重的，不太能够做的两届都认。

刘书博老师：这里我就补充一个问题。就是您觉得对一个管理学领域的教授，我们评价他的专业性，那会有一些咱们中国特色的标准。然后这些标准是不是跟美国又不一样？因为刚才我们说到可能不同学校，中欧、美国的那些学校、中国大陆的学校，可能我们对于价值的评价标准也不一样。那您觉得评价管理学者的专业性应该怎么评价呢？

受访者： 我觉得中国非常多元，比世界上其他地方都要多元，尤其是对管理学者的评价更是多元的。我觉得可能源自一个东西就是大家都有很强的圈地意识。

根据自己的特长他圈定一个范围，然后他出于一个有点私心的角度，他觉着跟他范围像的都是好的，跟他不像的都是不好的，我觉得这种倾向是有的。我们经常看见大家互相吵，搞实证的说搞理论的不好，搞理论的，其实也不见得是搞理论的，不搞实证的就说搞实证的不好，其实我觉得这更多的是出自一个本位主义的思考，如果说能够客观一点，那好或者不好，其实它还是有一些标准的。那有的人做的既然它是两个脱节的东西，那就实践有实践的标准，理论有理论的标准。那你在实践上做的好，那实践上的人会给你一个客观的评价，理论上做的好理论会有一个理论的评价。

但是如果从一个学者的角度上来说，我觉得一个能够被给予很高评价的一个学者的话，他做的东西应该是有理有据，能够让人信服的。他的作品是大家觉得是有说服力的。那么为什么说这些学术上的学者，比如说发很多AMJ的这些学者他是有说服力的？因为他经过很多很多同行评审，东西他已经说服了很多很多刁钻的人了，所以说他是令人信服的。那么没有走过这样一个程序的，你可以想象哈，他没有经过很多质疑，那背后就算很多人认可，那肯定还是有很多人质疑的，很多人不承认的。因为他没有经过一个那个修改呀、磨练呀、更正、升华呀、拔高呀，对不对？所以我觉得无论是学术发表还是说他不停的在各个地方去讲他的东西，都要有很多的批判之后，他提炼之后才能够更高，我是觉得那样的东西会更加令人信服。当然有的人思维的深度高一点，有的人思维的深度低一点。那经历多的人相对来说他的思维的深度和广度会更多一点，会更深一点。

黄秋莉： 刚刚也提到实践和理论的一个脱节的问题嘛，就是可能老师还是站在一个比较逻辑的评价方面，就是它符合一个逻辑性的，就说明它还是能够得到认可的。那我们现在针对实践和理论脱节问题，我们国家不提倡要把研究做祖国的大地上嘛，那这种做在祖国大地上的研究多吗？还是说现在是一个趋势？

受访者： 相对来说不是很多。因为OB的东西更加接近于科学，我觉得它是有基本原理的，基本原理就像数学，情景影响不太大。就是说当我们把OB领域的一些东西应用到我们中国情景下的时候也是同样适用的，差别也不是那么大。

黄秋莉：然后再提到就是说研究嘛，大家您刚刚也提到说要做一个一看就是前人没有做过的。那我觉得评判标准就是就是创造力嘛，就是要表现出一些新的知识点。那在您自己做研究的过程中，这种creativity是怎么体现出来的呢？有没有经验可以分享一下？

受访者： 怎么样体现自己研究当中的创新性，对吧？有一篇文章说新意是有理论贡献，然后会讲各个层次，会说到加个变量、减个变量，那也是有一些新意。但是更大的就是有问题，就是问题本身是原来所解决不了的。提出一个有价值的，不是无病呻吟的那种新的问题，其实是最有价值。所以我们做的很多东西都在想，我们首先是根据实践上看到有这样的东西，比如说我们现在就在想领导者怎么样从中层领导shift到一个高层领导，这是一个过程的。你做的也是有这样的，就是怎样从一个一般的学者到一个更好的学者，叫身份建构，它是怎么样建构的，这中间有很多的东西。

我昨天看了一篇文献，我就想知道我怎么样用到东西，据我现在研究的情境，我能不能研究出来这一群中层的管理者，到两年之后怎么样转成高层管理者？这中间过程是怎么做出来的？我想用实证的方法。因为我们发现那一篇比较新的文献给我们梳理了之后，发现所有过去研究身份建构的东西全是质性研究，大部分都是访谈的，大部分都是通过个人经历来做出来的，没有实证的。我觉得你现在做的就是这样，身份建构怎么样做出来的。那么我就在想，如果我用实证的方法做出来，其实是有贡献的。

但是我短时间没有那么多的精力来读那么多的质性研究，我就觉得问题是我不知道的，他是怎么样能够从一个身份建构到另外一个身份的，但我不知道，就需要继续看文献吧。当然看完文献的结果，你会发现东西没有回答，或者说问题已经完美的回答过了，或者说问题只回答了一半儿。那么如果说问题，看完文献觉得回答了一半儿，那你做出来之后你就会有贡献，我们对问题本身的贡献，因为他问题没解决。

我觉得我们前面发的那篇文章也确实是有贡献的，怎么做呢？在一个团队的的人，就成长需要而言，有的低，有的高，会导致什么？如果都高的话，大家像一个人一样就会迅速的往前发展，对吧？都低的话也没问题。但是如果有人高有人低状况是什么样子的？我们就把polynomial的这种方法引入到高管团队的领域去，然后做了这样一个东西。目前为止我们没有看到在这个领域或者叫我们做的是小企业的创业团队领域用方法，那么我们用方法就可以回答很多方法之外所回答不了的问题。因为方法本身的背后它含了很多的问题，因此我们做了这个东西你就会发现确实就回答了很多的新问题。

黄秋莉： 方法还是能够带来很多新的知识创造。

受访者： 新的方法是代表了我们原来回答的问题只能用原来的方法回答，回答不了的问题原来是就在那儿扔着，现在有了方法就能回答新的问题。

黄秋莉： 感觉跟科技的进步有一点异曲同工之妙的感觉。您刚刚也提到，您对实践还是挺看重的，那您工作的一个场所主要还是在学校嘛，那您关注社会实践问题的一个主要方法或者途径是什么呢？

受访者： 一个方面是从教学，因为在教学的过程当中他们会提出各种各样的问题。

黄秋莉： 是MBA的吗？

受访者： 是，有很多的企业高管。然后我们就去思考那些问题。比如说两年前有一个学生说呀，做服装的很多线下店都死了，但是他就做线下店，他不会做线上的。但是他就蓬勃发展，一年赚快上亿了，然后利润率达到60%，很多人全国各地来学他们，我就觉得为什么会这样，为什么能成呢？为什么你能做成，人家线上线下都活不了，你为什么能做这么好？然后我就去研究问题。就是现实的知识是回答不了他的问题，我就去研究这样的一个问题，所以说会有一些案例去思考。

另外我们有一些项目要做，然后在项目对应的情况下，我们去做一些访谈。做一些企业访谈过程当中，它是有个聚焦性的东西，基本上跟刘老师做的很像，就是案例教学，案例的东西会来自于现实。

黄秋莉：明白，那就是听您说话，感觉您是一个非常善于思考也很喜欢思考的一个学者，然后也很坦然平和的这样一个状态。那您在写论文儿呀，做课题呀，在带学生过程当中有没有遇到一些比较困难呀，让您比较焦虑的事情呢？

受访者： 写论文一般都是这种不好的感觉，总是在克服各种各样的困难，写论文的过程当中都是这样。包括模型怎么打磨，就一遍一遍的去讨论吧，更多的讨论才能解决问题。当然是需要一些有效的讨论。

黄秋莉： 你自己在工作当中是怎么去进行这种有效的讨论？

受访者：一般情况下是跟我们团队的学生讨论，然后讨论各种各样的想法。然后学生去找一些文献来看一下现状，然后我们再讨论东西往哪儿做，能做成什么样子。另外一个去跟我们的同事讨论，跟别的老师进行讨论，还有一些是跟一些国外的合作者讨论。所以说不同的时候讨论的问题是不一样。

黄秋莉：就是您之前也提到嘛，你在企业就是有这种感觉，就是他们身不由己嘛。那您最后选择回到了学校就非常看重自由，那过去了这么多年了，您觉得您回到学校之后达到你心中那种预期吗？您会觉得您是自由的吗？

受访者： 还可以，因为我在外边还是待的时间挺长的，回到学校之后，基本上是相对自由的在做自己想做的东西。但是也不见得自由是自己内心的真实想法，定力其实很考验人，

黄秋莉： 这是什么意思？感觉有点思辨性在里面。

受访者： 比如说校内的要做的事儿你都可以自由的选择，你选择上什么课、不上什么课、什么时候上，对吧？选择你的研究的主题呀，选择你平时的生活的节奏，都是自由的。还有那些不自由的时候，就在于内心还有一些框框，还有一些欲望，然后你自己能不能完全驾驭自己的内心

黄秋莉： 就是在我们看来嘛，就是您的现在成果那么多，然后在XX大学也是担任教授嘛，这本身就是一个非常强的认可了。而且您在AMJ这种西方顶级期刊上面也发表了研究，这种来自西方的认可能是被我们认为是更高级的认可哈。您觉得这些认可会给你自己带来什么样的影响吗？

受访者： 发表的很多和发表的好，可能是同行会认可，但不是同行就没有人会知道，所以说更多的认可来自于社会。所以说学者怎么样能够被社会知道，这可能是也是重要的，怎么样被企业界知道。所以说有的教授上课上的好，就会有企业界的人找他，他就会影响很多的企业家，然后他们就可以改善人家的企业状况。对吧？但是一些实打实的价值就可以影响很多的学生，改变他的思想和命运，我觉得那个是最本质的改变。那现在的所谓的你说的认可，那就是评职称了可能会有点用，但其实受众非常少。我觉得这不是我们做学术的一个终极目标，因为学术的最终极目标是把我们的思想真正做出来有价值的思想，然后被非学者所认可。

黄秋莉： 明白了，要找达到这种跨界的影响。

受访者： 对，我们不是写给自己看的，不是写给同行看的。

黄秋莉： 那您自己对自己这种职业未来的一个期望，也就是能够成为这样的一个学者吗？

受访者： 对，希望能够做出来一些有价值的研究，真实有用的研究，能够让别人觉得有收获的东西，我觉得是重要的，而不是发出来一篇只有数字，然后没有什么价值。

黄秋莉： 明白了。

受访者： 当然有的时候发的虽然不一定高，但是他本身确实有思想、有价值，那也是对的，不一定非得用杂志来评价的。

黄秋莉： 感觉您现在就已经达到这种状态了，可以随心所欲不逾矩了。但可能很多年轻学者就做不到这一点。

受访者： 只不过说没有明确的约束你一定要干嘛。所以说只不过看起来自由一点，没有说一定要评职称，然后要发表怎么样，稍微好一点，但是要想达到一种好的被认可的状态还挺难的。

黄秋莉： 那您目前可能还是有一种压力或者说是焦虑吧，希望有更多的认可。

那您就是工作了比较多的时间了嘛，回过头来看学者的成长道路的话，您头几年做研究，然后做了几年的研究和您现在一个工作的状态，你觉得会有很大的差异吗？

受访者： 就是早期的研究和现在研究，是吧？早期的研究发表导向更强一点，现在可能不是那么强。

黄秋莉： 明白。好的，我觉得我已经没有什么问题了，谢谢魏老师。

刘书博老师： 那我这边稍微还有一些补充，就是关于商学院管理，您在XX经管感觉怎么样？因为管理方式肯定是咱们具有中国特色的。但是我发现就不同学校它的这种管理还是有细微差异，就比如说我们可能跟对外经贸又不一样，然后跟上财肯定也不一样。那能不能谈一谈上大和XX，商学院人事制度呀或者是这种管理，能感受到的有哪些显著的不同吗？

受访者： 他们差别不大。这两个学校比较有很多的关联性。比如说上大很多人毕业了之后去的XX，XX毕业的人再在上大。然后原来XX的院长又到上大做了很长时间的院长，相对来说还是差别不大，这两个学院的互相交流会比较多。

刘书博老师： 那您参加到学院的管理行政工作吗？因为像我们学院就是学而优则仕，他们都是学术做的成绩突出，然后就自然的走向了学院领导这样的岗位。那XX是否也是这样子？

受访者： 我基本上是只管我们系的，我们学院是管科导向。所以说学院管科的声音会更强一点，包括在学校里边的话语权。

刘书博老师： 那日常比如说开会呀，或者说有一些党的这种会议，他会对您的时间产生一种压力感吗？现在怎么样？

受访者： 会，事比较多。我还做支部书记，会挺多的。但是该开的都得开，对吧？现在线上，导致很多的会开起来并不是那么的费时间了。所以说我们事情该做就会做。然后我们党支部里边人多一点，大家互相就分掉了，就是很多事情做到什么程度就适可而止吧。然后学院里边的行政的事儿，我们系没有本科生，所以还是少了很多的事。学院里边的事儿基本上都是各个副院长在定的，行政的队伍会做很多，那我们还好。另外一个就是上课呀，面试啊、答辩呀，这种事情相对来说会比较多，那自己就适当的参与一点嘛，没有时间没有精力就少参与一点。

刘书博老师： 明白。最后还有一个小问题，就是您觉得在作为大学的老师，最喜欢做的、让你觉得最有价值的是什么样的工作内容？

受访者： 我觉得是做研究和教学这两件事儿，这两件事情同等重要。那做研究的话会让自己感觉到能产生很多的能磨练自己的思想，并且能够产生一些有价值的影响人的思想，那教学的话会直接的会影响一些人，让他们得到一些成长，所以这两件事基本上都是都很重要。

刘书博老师：那有没有一个现在对于您来说有一个这样的model在行业里，可以匿名说，也可以说出是谁，那就是他有哪些方面觉得可能特别好，特别具有这种示范效应？

受访者： 没有。因为一般来说我们了解别人不是那么多，除非是自己的老师，跟自己关联很大的人。

刘书博老师：那或者就抽象的说一个model他应该是什么样的人呢？

受访者： 我觉得我自己的想法有点儿像西方似的，就是不像中国的现在，很多大家认为做的好的，长江啊什么title的，都有很大的资源呀，可以做很多的事儿等等吧。可能很多人看着那个应该是，但是我不觉得是那样，我觉得是一个独立的学者应该会更好一点。就是能够做很多的有价值的企业管理方面的思考。原来我跟你讨论过，比如说像XX这种，拿自己的学术基础，去很多企业解决实践问题。当然他可能年龄大了哈，他可能只到了只到了咨询和教学，但是学术这方面没有，我觉得在学术方面还是应该有，就是学术的东西很重要，然后会跟企业的实践相关联，这两个东西都做好。然后再通过教学影响一些更多的企业家，我觉得这三种状态都比较好的情况下比较理想。当然这里边可能就缺了一个，然后我们常说的叫行政。但是我没有想到行政应该怎么样关联。因为行政会用很多的精力和资源去做另外的事儿，就不能把事情做了对其它事情有互相补充的作用，不过也说不准。

刘书博老师： 好的，那我们问题都问完了，时间也过得很快，感觉意犹未尽，但是已经快两个小时了，我觉得也是非常不好意思，我们问了那么多问题，但是魏教授都是非常非常有耐心的给我们回答。然后我觉得在学术价值观层面我们还是需要向魏教授看齐。当然在博士期间，我觉得你们应该尽可能有效地全方位的提升学术能力。所以今天两位同学也问了很多关于博士学术应该如何做的一些细节性的问题。好，那再次感谢，希望我们有机会线下能够当面请教魏教授。

# 受访者7

刘书博老师：您当时从什么时间开始形成了对于学术道路的选择？还是很多机缘巧合的因素，我就不知道是从本科呢还是从研究生还是博士阶段就开始决定要走上学术道路了。

受访者： 我觉得应该大概是在研究生二年级的时候吧。因为我本科是在天津财经。那么我觉得天财和中财有一点相似的地方，就是这种财经类的院校的学术氛围没有那么浓。尤其是在本科阶段啊，氛围比较浓的是实习、考证。

所以应该在本科时候还不是特别明白学术是什么，我当时去北大也是运气很好，我觉得是到北大之后我才开始慢慢了解真正的学者的生活是什么样子的。

然后其实我们刚去的时候，有让我们选直博还是硕博。当时我们都选的硕博，那大概是08年的，我是08年参加的夏令营。那个时候好像大家还不像现在愿意读博士的人非常多。那个时候想读博士的人没有很多，我当时的想法只是想上北大，但我并没有说要做学术。

我觉得是有一些去那儿之后有一些引导吧。我们当时是有一个项目是徐淑英来搞的IPHD。我一直觉得我蛮感谢他的，他弄这个项目确实我觉得影响非常大。当时我觉得有两方面的原因导致我最后做学术。一方面，（有的时候我也跟学生说）有点像认知失调。就是我们第一年的时候大家都非常投入，我觉得和那个氛围是非常相关的。就是我们上一届的师兄师姐都不愿意读博士，当时就有一个同学还是在老师的极力劝说下，然后转了博士。然后还有一个本来直博的师姐还拼了命要转硕。所以老师们当时很想发展博士项目，然后他们又都不愿意读，因为当时就业非常好。所以老师们也很着急。

然后赶上我们那一届负责我们的coordinator也是刚刚来光华，他08年来的，属于非常有热情的阶段。然后当时我们系好几个老师都是刚刚来，就是非常有热情，就可以热情到经常单独请我们吃饭，然后给我们讲学术的生活。我印象很深，凌晨三四点有的时候还会给我们发邮件，就是你能感觉到这种极大的passion在里边。

刘书博老师： 所以您说的是coordinator属于这个项目的行政老师吗？

受访者：不是。是专任教师。就是当时企业管理有三个方向嘛， marketing， strategy and OB的。然后呢每个方向会有一个coordinator来负责IPHD项目。然后当时我们那个老师也非常非常有激情，然后他现在也说我们那一届是他投入的心血非常多的一届，然后我们那一届也恰好是组织方向第一年开始招夏令营，所以老师非常有热情。然后我们那一届的同学投入非常非常多。然后在那样的一个环境下就是其实我都没有想要不要读博士，但是就是不想掉队，你需要跟上同学的节奏。就现在来看，我依然觉得就是我们那一届的培养应该是光华史上确实非常非常突出的一届，所以氛围非常重要。结果导致老师们用力过猛，我们那一届有十个博士加硕士。然后呢当时其实很多人都想读博士了，所以老师们当时遇到了一个很极端的情况，上一届是没有人要读博士，我们那一届突然发现名额不够了。最后我们班有两个同学是出国了。然后老师们也是极力争取我们那一届，最后留了四个人读博士。之前我们上一届都没有人读。所以那个时候我刚开始我其实是不太能分得清，就是我想读博士还是说我希望优秀。但是有一点是不矛盾的。我现在也认为光华对我们博士生那个时候的训练是非常好的，就是理论和方法的训练都非常好。所以虽然那个时候没想读博士，但是基本功还是打下了。所以在二年级末要不要决定硕转博的时候就是还是决定要转。那个时候就每天开始还担心自己会不会转不成，就转成了就会觉得很珍惜，能理解这种感觉吧。

然后我们同学那时候也都非常优秀，大家都非常努力。包括我那天我感觉压力非常大。我数了一下，我们那一届发过A的同学已经超过70%了，大部分同学都已经发过A了，搞得我压力也非常大。那个时候就是这种强烈的氛围导致我现在其实也说不清楚，是因为我想优秀还是因为我热爱学术，反正最后就转了博了。

其实它是一个过程啊，没有说某一个时刻我就下定决心就读博士，没有啊，就是大概我什么时候觉得我是非常认可职业道路的，就是在四年级找工作的时候，就是我一点动力去公司都没有。我是通过我的行为意识到说，我可能真的还对（学术）职业有很高的commitment。因为其实那个时候也有其他的选择，有选调，有一些研究院，有一些央企、国企，其实都是有的，还是在光华有很多机会。但是就是一点动力都没有去投（简历），然后我才会意识到说我可能真的对这个职业非常非常的commitment。

刘书博老师： ok，那个时候是不是因为在博士期间有不错的成绩出来，所以会有一些正向的激励。

受访者： 没有，我觉得我是属于动作比较慢，我不属于那种一上道就非常快的，然后我的第一篇文章是我导师带着我发的，是JWB，很好的期刊的， journal of world business，然后第二个期刊是我访学的老师带的，这是一个类似harvard business review的文章

刘书博老师： 很好的文章。

受访者： 然后我真正第一篇比较好的文章是《管理世界》，大概是五年级上才出来。所以其实是很晚了，而且那时候没有正向激励。因为你知道我们那届比较极端，就是我有一个同学太优秀了，就是那个XX，我不知道你是不是了解，毕业发了JAP，现在在中山大学。然后他一年级发《心理学报》，二年级发《管理世界》，就属于几年都遇不到的（类型）。

刘书博老师： 那他为什么做的如此好，如此突出啊？

受访者： 我觉得有几方面原因吧。

受访者： 第一个就是他没有犹豫，他从第一年进来的时候就非常的明确，就是要让这五年全力试一下，行就走学术，不行就不走学术，我觉得他是我们当中不犹豫最早的人。那你像我之前两年其实还在犹豫嘛，就是其实是过了qualify之后我才定下来，我都已经没什么指望了。其实在qualify之前我有时候都想过，如果说当时不是我们同学都想转，我会不会也不想转。但是XX没有，XX应该是从一进去就比较坚定。

然后还有一个我觉得XX是一个非常非常会选题的人，非常能抓住热点，我觉得这是他非常强的一个能力。

当然我觉得最主要的还是他极其勤奋，我觉得确实是属于一个现象级的人物。所以当时我没有什么正向激励呀，其实我那个时候按说毕业的时候package也不能算差，但是因为他太优秀了，导致所有的人都黯然失色。而且当时我们班有一个出国的同学，然后刚去美国就跟老师一起发了JAP。导致你像我们班一共才几个同学呀，就是还没毕业，大家就都开始发JAP了。我觉得没有什么正向激励。

刘书博老师： 明白明白。那其实也是一种鞭策式的激励，更多是来自于一种peer pressure那样。

受访者： 我觉得光华的peer pressure还是有一段时间让我觉得蛮大的。但是我觉得后来其实是非常正向的，有一段时间我觉得是负面的激励，因为你会觉得是不是自己不够优秀，但是后来你就会觉得这是一个非常正向的激励。因为你可以从他们身上学到很多，然后大家之间相互的交流是能得到特别正向的激励。其实我现在都特别怀念那个时候的氛围。

刘书博老师： 明白，所以您坚定走上学术道路应该是在博士四年级。然后那个时候当然也不是完全的正向的（激励），可能也是来自于环境、所谓的peer pressure，或者说大家都是在相互学习快速进步的一个氛围。所以就是让你···

受访者： 当时觉得氛围特别重要。

刘书博老师： 好，那在整个博士的学习过程中，有没有与之前想象的那个样子不一样的一些意外啊？或者说有一些觉得特别困难的，或者说经过了一些心里面的转变呀？或者是类似这样的一些艰难困阻的东西，就是在光华的学习期间有没有？

受访者： 我现在只能记得那个时候有感觉到很困难的时候，但是你说什么东西让我觉得特别困难，我又记不起来。很具体的事情，可能就是有些时候感觉到大家都很优秀，可能会有一些压力吧。但是我觉得我又想不起来很具体的事情了。

刘书博老师： 那光华整个对于博士的培养教育体系，应该是···就比如说会跟美国的藤校合作，像您之前是在哈佛有过交换。然后呢他们也会可能像徐淑英这样的一些学术做的非常棒的老师会到光华去专门开系列讲座呀，甚至开一门课。那这些是不是也是光华的一个特色？会让我们的同学们能够及时的受到这方面的一些训练。

受访者： 我觉得是，我觉得是，因为我觉得那个时候光华给我们的机会真的非常多。现在我觉得其实很多学校的训练都已经非常好了，但是在我读博士那会儿，也许是一种bias哈，但是我那个时候真的觉得光华的训练是最好的，没有之一。因为在那个时候，在二年级还是三年级时候，我们去参加了一次中国管理学年会，我就会觉得交流的时候明显的感觉，怎么说呢，有一种优越感吧，如果说不太谦虚的话，有这种感觉，而且我觉得包括去哈佛交流的时候，我也觉得光华给的基础训练是很好的。

但是有一点是光华比不上哈佛的。就是哈佛老师的眼界，就是人家选题就能确定是A···就是只要能把这个东西都做出来，这东西肯定是A。就是你去那儿当学生，你只要跟着做，你肯定能做出A。我觉得这个方面还是不一样的。

但是就说基础的训练啊，我觉得光华的训练确实很好

刘书博老师： ok，那个时候光华的训练要教什么、怎么样教啊，这些东西您知道是怎么样建立起来的吗？具体细节您知道吗？

受访者： 具体细节你指的是我们怎么培养的吗？

刘书博老师：对对。就是就比如说我们现在博士也要上课，那课程可能是我们老师要开会啊，要拍脑袋要决定，或者是中财的一些传统——之前上现在也上。那我不知道光华对于学生的学习培养是怎么样一个理念，他怎么样确定它的培养内容，还有就是标准是什么？

受访者： 光华的培养就是抄美国啊，课程体系几乎完全一样的

刘书博老师： 几乎完全和美国一样？

受访者： 几乎完全一样，几乎完全一样。我觉得跟哈佛没有太大的区别。他的课都是围绕怎么发paper来建立的，就是我们都不会去上我都觉得很奇怪，就是我们的博士生上很多奇奇怪怪的课程，我觉得我们那个时候不会上没有有利于写paper的课程，不会上这种课。

刘书博老师： ok，ok，那会不会有一些枯燥？可能也是我的偏见吧

受访者： 不会呀。因为你完全不会写paper，这就属于你开始建基本功的时候，而且我一直认为说在任何一个行业的基本功都是极其枯燥的。

刘书博老师： ok

受访者： 对吧？你肯定是要反复训练的，对吧？

刘书博老师： ok，明白。那所以过程一般是前两年上课教大家怎么去进行学术论文发表，然后是一个资格考试，然后过了之后后面的时间呢，是自由写论文了还是说在后面的时间也是有一些别的安排。

受访者： 就是和美国一样，完全就是。其实现在中财也是这个模式嘛，就是资格考试，然后是开题，答辩，就三关嘛。

刘书博老师： 对对。ok，那当时光华对于发表论文跟毕业有挂钩吗？

受访者： 光华这一点还蛮好的。当然我也不知道这个东西是利是弊哈，光华对发表没有要求很高，我觉得比中财要求还低，就是要求有两篇文章就可以，而且其中一篇可以用AOM的conference paper代替，就是你只要有一个publication就可以。

刘书博老师： 明白

受访者： 但是东西其实也后来老师们也会反思，就是有的时候你需要和大环境有一个契合。光华学生有一个特点就是，一上来老师的给大家的灌输就是如果不能发A你就不要去做。所以光华有一些同学可能就是毕业的时候还没有publication，有可能会有这样的危险，因为你知道A它周期也很长嘛。

不同学校它的values不一样，光华可能认为说我就是要发A。但是有些学校觉得我需要有一个证明，你说你有A的R&R，那发不出来怎么办呢？但是现在光华的老师压力也很大，我那次还跟我导师一起聊。他后来也觉得就是说，要为学生做一点，因为从老师们的角度来说，对于光华的老师来说，他们发一个B对他们都没有意义，他们还是要靠发A，但是学生有的时候毕业还是要有一个东西，像有的学校他就拼量。所以光华也不是孤岛嘛，他的学生也要找工作嘛。但是就光华本身他的values的话，他肯定就是···而且就是这两篇的（毕业发表）要求，也不是光华愿意，就是可能为了达到国家或者什么的一些基本要求吧。

刘书博老师： 对，对对。所以在您那个时候氛围是很好，那后面怎么样啊？还有就是前面怎么样？前面的，我刚才听您的说法是，大家好像能工作就不读博士啊，是有那样的一个心态。那后面会不会就转的更想读博士。

受访者： 近几年我觉得可能是经济不好啊，近几年想读博士的人非常多，近几年就非常多。我们之前那个老师们很头痛啊，劝他们读都不读，都去找工作去了。因为那几年光华找工作非常好啊，他们那个单位也好，前景也好，薪酬也高，就是老师根本劝不动。

刘书博老师： 对对，而且那个时候好像互联网行业呀什么蓬勃发展。

受访者： 那几年经济非常好，这几年就是大家都愿意做一个缓冲，所以这几年太卷了。我觉得我要到这几年可能都不一定能读，因为太厉害了，大家太卷了，我们那个时候没有这么卷。

刘书博老师： 是的，而且说今年好像就业形势是这几年最为严峻的一年，好像对于本科、研究生、博士都是这样子。而且你看现在我们那个时候找工作还没有那么普及的师资博士后嘛，那现在几乎都是这样。所以当时就找工作您是有其他选择吗？就是为什么选择到中财？或者说换现在换一个问题，就是您当时找工作的时候会考虑哪些因素？

受访者： 考虑哪些因素哈，我觉得我的那个思路不是特别正确。我当时还跟XX（受访者的一位同学）讨论过，然后他是完全不考虑，就是从高往下这样选。

我当时还是选地域，他是只要是好的综合性大学就行，然后我当时是考虑地域，就是我先考虑到上海因为我当时的男朋友，他要去上海，然后我就在上海找。其实我觉得这个事情还非常不好意思，其实那个时候上财基本上给了我offer，虽然就没有签，但是他其实已经答应是有一个offer。

然后后来就是因为一些原因，又要在北京找，那在北京找的时候，已经比较晚了。然后我试过北航，是过了他们的试讲。但是后来他们说要一个管工的还是一个什么学生，但是他们也没有明确跟我说不行，也没说性，然后事情就不了了之了。

然后我还试了北师，但是北师说他们要师资博后，然后那个时候就不想做师资博后。但是我现在非常后悔哈哈

刘书博老师： 为啥呀？

受访者： 你难道不想去考虑是小朋友的···

刘书博老师： 但是他给的是师资博士后嘛，还是有不稳定因素的。

受访者： 但是那个时候好像基本上都还可以留下来，我其实不是特别清楚，后来就没有再了解过哈。然后当时还试了中财

刘书博老师： 对外经贸？

受访者：我没有投外经贸，因为我导师跟外经贸他们关系很好。然后直接打电话问了一下他们系主任，他们系主任当时明确说我们现在有好几个女老师要怀孕，我们不要女的、而且不要光华的。你知道外经贸当时招了好多光华的，就是他们那年已经觉得overload了，所以说你不要投简历了。

刘书博老师： 那现在在对外经贸的光华的您那些师兄师姐他们怎么样？因为我听说对外经贸好像流失率很高。

受访者： 我们那一届有两个去了外经贸的，然后现在一个去了人大，一个去了北师大，都走了。

刘书博老师： 哦，他们走就是人往高处走。

受访者： 有一个同学应该是吧，去人大那个。还有一个肯定就是想小孩儿教育，然后去了北师大

刘书博老师： 好的，好的，所以当时考虑就是首先是中学校比较好，然后区域也要考虑一下？

受访者： 我先考虑的区域。我当时为了保险起见，我还试了天大和南开。然后南开因为我本科不是211，他们不要；然后天大是可以的，天大是当时有要，但是我觉得还是首选北京吧，然后最后就中彩要了，我特别的开心。

刘书博老师： ok，好的，行，我这边问题暂且就问到这里。

吕梦娜： 张老师，我之前上过您的那个人力资源管理的课程，不过当时因为是大课嘛，就上过一两节课。可能对您更多的了解是在就是给学生做点评，然后给我们做讲座分享的时候，所以印象还是特别深刻。然后今天访谈我觉得更多是一个跟您学习交流的过程吧，因为我也是人力系的嘛，就觉得可能会学到很多东西。那我后面就开始就是有一些小问题想问问你哈。

首先就是您在博士期间是到哈佛大学联合培养过。那对比一下中美这两国商学院的培养经历也好，或者是在氛围方面也好。您觉得这两个商学院有什么异同点吗？

受访者： 光华像美国的二流学校，就是光华还是以发表文章为导向，就是我怎么样去找寻规律，找到发A的技巧，然后我们能发个A。

然后呢我觉得哈佛最宝贵的一点就是我觉得他们的学生包括老师非常敢想，他们会做一些不太一样的研究。我给你们举个例子，就是当时我去的时候，我会觉得他们的研究都不是那么的传统，你想在那个时候他们包括MIT那个时候也有一些seminar我也去，他们那个时候就开始用机器人去做研究。然后他那时候会做一些就是就非传统的OB的研究。那个时候我印象很深的一件事情是我做光华的助教，社会心理学，就是辅导他们本科生做研究。然后其中有一个组就做了星座。然后我就跟他们说，我说东西没法儿概念，而且他是一个伪科学。这怎么去做呢？虽然我觉得很有意思，但是过了若干年之后，然后我就知道MIT的一个学生，然后就把它发了JPSP，就发了很好的期刊。就是我觉得像这种美国一流的学校，他们的宝贵之处，就在于他们的想象力以及他们能把东西落地。我觉得是非常难得的，就是光华也能发a，哈佛也能发a，但是你去看他们发的a是不一样的，这个你得承认。我刚去哈佛的时候，我就觉得他们的学生构成不一样，至少我去那个时候他们很多人是这样。就是我们的学就是我们的学生参加夏令营的时候就已经非常的清楚paper应该怎么读，然后怎么怎么样。然后我当时去的时候我们有一个师妹去那边读博士，刚一去说他们的学生怎么连什么是R&R都不知道。你就觉得那些基础知识非常的小白，但是人家毕业的时候就能就能发a，我们慢慢的就落后。当然一方面是他们那边有一个特点，就是他们很多学生是在咨询做过，包括你看host我那的那个老师，他们很清楚就想做学术，他们比我们的学生坚定，然后比我们有想象力，我觉得这个是特别难得的。我觉得啊我们的研究缺少想象力，都能发a，但是不一样，光华像美国二流商学院。

吕梦娜： 您说我们缺乏想象力，会不会是因为我们一开始就比较规范，会不会对想象力有一点的制约作用呢？

受访者： 有。还有一部分我觉得也是能力不够，就是他们想象力有一个地方在于说他们的老师是有能力去判断什么样的想象力的研究能落地，还有就是怎么样把这种有想象力的研究落地。然后我觉得我们不具备。我们可能就是能够模仿一些非常traditional的研究，但是这种引领风向的研究我们缺少想象力。

吕梦娜： 了解了。那就是您之前在光华然后求学嘛，现在又在中财也工作了很长时间。那如果是对比一下这两个商学院呢，因为前面你也提到了天津财经大学，就感觉可能还是有很大的不一样的。您觉得光华和我们这些财经类的院校商学院相比有哪些相同点或者不同点呢？

受访者： 我觉得光华的学术训练会更好、更严谨一点，就是给学生的基本功打的要比中财的好很多。

吕梦娜： 是的。其实我们自己因为也是学生嘛，也能够稍微感受到一些。就感觉好像尽管很强调基本功，但是似乎训练的过程又不是特别的严谨。

受访者： 这个确实是。我当时就非常感慨哈，我比如说一周布置四篇（文献），然后就轮到报告就报告，然后不轮到就不看。我觉得基本功就是没有量的积累是不可能有质的变化的。我们那个时候一周精读的paper就是课上要看的在20篇左右，然后你一周读5篇和20篇，那个差距是是很明显的。

吕梦娜：那您觉得除了学术氛围方面的差异之外，还有其他方面的差异吗？

受访者： 视野吧，我觉得视野也很重要。你像我们那个时候没有疫情啊，所以seminar非常多。然后有很多人去来光华讲，虽然一流的少，像特别顶级的少。但是比如说像美国二流学校这种一年发三四个a的老师是非常多的。所以至少你能有一个感觉，就是好的研究应该往哪个方向去走，这个还是蛮重要，视野不一样。

吕梦娜： 而视野可能也是我们这方面资源也有一些差异，是吗？

受访者： 对，觉得有一点关系。

吕梦娜： 您提到就是在参加中国管理学会的会议上的时候，你觉得其实光华其实是很好的，甚至是最好的。那您能够具体聊一聊是通过哪些方面能够感受到这种差异的吗？

受访者： 就是受过良好学术训练的人，他在讲东西的时候会非常有逻辑，非常清楚，比如说他不会出现一个不明不白的概念，然后他不会出现这种研究设计上很明显的错误，但是我们当时去听很多其他学校的人作报告，你会觉得明显觉得是有点低级的错误，就是这很明显不是学术。因为就好像你现在在看一个本科生论文，你也会感觉不一样，这个说不出来。但是你能感觉出来，严谨性、规范性。

但是呢我现在感觉不一样了。你看今年来我们系最后招了一个浙大的毕业生嘛。然后我现在会感觉这几年其他高校的进步非常非常大。像浙大、中山大学、人民大学、上交，我觉得这几个学校在培养上的进步非常非常大。

吕梦娜： 您知道原因可能什么？比如说我们都在学北大光华还是怎么着

受访者： 就是大家摸清套路了嘛，这个东西也没有那么难，是不是？

吕梦娜： 就是我觉得会更加的专业化，就是那种professional那种的培训训练。

受访者： 对，就是大家掌握套路了嘛。就是我觉得一流商学院很难，但是我觉得像这种二流就是这种paper machine的训练，我觉得我们是很有优势的。你像那个江浙那边，你像那个浙大他们那边就挨着阿里巴巴，是吧，然后各种资源，然后中山那边和香港那么近，所以他们其实想赶非常容易，对吧？你像那个长三角那块儿，资源也非常强。所以就是我觉得这几年已经没有什么差距了，甚至有些他们可能后期做的更好。但是在我们那个年代，确实还是我觉得光华有一些优势啊，就是不谦虚的说

吕梦娜： 是，确实是这样的。

刘书博老师： 这里我补充一下，就刚才张老师说优势像华东华南，我觉得其实华南优势还挺明显。因为他们挨着香港，香港有特别多的那个···比如港中文、港大、港科，他们的管理学，我觉得应该是paper machine的重镇吧。因为你看他们发的是很厉害，所以他们去到广州、中山、深圳也是这种便利性嘛。然后像东边儿就是中欧国际工商学院这样的，那其实因为他们政府有钱，或者说像中欧这样的学校特别有钱，所以其实就吸引到了很多美国的也不是一流的，就是二流大学的商学院的老师，所以他们就会把那种方法快速的带入到当地的学校学院。

受访者： 对，是的

吕梦娜： 那张老师，今年年前我记得是腊月28号吧，您给我们就是做了一个分享关于学生如何发表文章的。然后有一点我印象特别深刻。就是说你在北大读博的时候，可能会在枕边放一个小本子。就是专门用来记录想到的一些idea啊，或者想到的一些想法啊什么的。我就感觉您好像是达到了一种心流的状态，不过我觉得这种状态似乎是在比较放松的情况下才能够达到。但是像您也提到就是在光华读博压力也是蛮大的嘛，包括那个IPHD项目会希望您在顶级期刊上发表文章。所以我很好奇就是你那一段的读博经历是一个什么样的状态？

受访者： 我觉得首先我这个人本身还是比较简单，就是我对知识是有一些比较天然的热爱的。所以就是我会因为某一个想法就晚上想它，然后就很兴奋就睡不着。所以这是我很自然的一个反应。压力也确实是有，不过压力最大可能还是三年级或者什么时候，那后半程更多一点，我觉得那时候还好，干什么都有压力嘛，然后好像我觉得这两个也没有很冲突，就是压力是有的，但是也并不影响说压力大到我就没有办法去想东西，好像也没有那么夸张啊。因为我觉得我那时候的同学就是，虽然大家有这种压力，但是整个的氛围还是非常好的。我们当时就是所有的博士生大家都会在一个空间，所以就是大家一起吃饭，然后一起学习，关系还是很好的。那你说有没有一些暗暗的较劲儿，可能也会有，但是大家还是很open的，还是会一起交流啊什么的，就是压力和享受这种交流的快感，我觉得不矛盾、不冲突。

吕梦娜：其实我觉得您是谦虚了，就是缺乏正向激励这一块儿，可能跟我理解的还不太一样。因为我现在觉得是压力很大，然后又很缺乏正向激励的那么新的一个时期嘛。然后就觉得有的时候确实会压力大到可能没有办法像您那样还在不停的去思考。所以我就想知道你是怎么去解决这样的···或者说心态上面去怎么去调节的？

受访者： 我那时候确实没有正向激励呀。你想我跟我同学对比，然后他们都JAP已经出来了，《管理世界》出来了，我还毛都没有，这个东西确实是没有正向激励。但是我觉得是这样，你只要在做的过程当中就不会有那么大的压力

吕梦娜：就每天都在进步，都有这种暗示

受访者：对，就是你压力大是因为你什么都没做的时候，你做的时候就没有压力了，你做的时候还有什么压力呢？

刘书博老师： 还有一个建议啊，我这边再插一嘴啊，就是那个中欧有一位XX老师，那他的一个你给我的建议就是你一定要有很多的pipeline，那就是你同时进行的工作一定要足够。这样的话你就压力感会小一些，也就是说我们一定要勤奋。因为每天都要动起来，因为你的pipeline非常多嘛，然后你有合作伙伴，你必须得把他们推进。

受访者：这一点让我提醒，我觉得还有一点就是你周围一定要有能跟你一起进步的伙伴群体。我说光华虽然让我觉得有一些压力，但是压力归压力，但是他们也是战友啊，就是大家一起并肩作战，一起去解决很多问题。你不是一个人在走。我觉得这也非常重要。包括你有一些比较靠谱的合作者，然后大家相互激励，这个也可以大大的缓解压力。就是千万不要自己在那里搞，自己搞是很容易有压力的。

吕梦娜：从你指导学生做研究的这个经历来看，你有没有遇到觉得特别适合做学术或者是特别适合当大学老师的这样的学生，你觉得他们有什么大概共同的特征呢？

受访者：你说我带的学生吗？

吕梦娜：对。

受访者：然后我来中财带的第一届本科生，然后我当他们班主任，14级的学生18年毕业嘛，然后我觉得他就很好，然后后来他也很坚定做学术。后来就给他写推荐信，然后到这个光华，她现在是我导师的学生，所以现在也是我师妹。然后她当时在光华夏令营表现也非常好。

他们夏令营的时候的一位教过我的老师还打电话说你问她要不要来，就是她那个时候可能自己都不知道，但是我知道她那个时候的排名是第一的，然后前一段儿时间我也挺开心的，她拿了校长奖学金。然后她现在也跟我之前的导师在读，然后应该也是马上要找教职。我在光华的老师会说我们两个有一点像，就是我对学术还是有热情的，然后想问题会比较有逻辑，我觉得这个是优点。

然后后边还有个学生，其实我觉得我现在带的那个研究生，我觉得他很好，我觉得他有两点让我觉得是我没有的东西，我觉得他非常好。一个是他干活儿，缺点是有的时候比较糙嘛，但是他非常快，我交给我，就是我问他在不在，就永远很快，而且你交给他的任务很快就会给你一个版本，就是不管怎么样，就是很快就会给你一个反馈，我觉得这一点让我觉得非常好。然后还有就是性格非常好，然后他喜欢运动嘛，这个有不开心的，他说去打打球心情就好了，我觉得让我非常喜欢。

然后还有一个我今年带的一个本科生，我觉得他的好处是百折不挠，然后有什么困难行动力非常强。后来他要去浙大了嘛，然后他跟我浙大那边的一个师弟要一起读，我还特意跟我那师弟推荐了一下。我说这个学生真的很不错。

我觉得这几个学生吧，这几个学生是我目前教过，我觉得我比较得意的。然后也是我觉得他们身上有一些这个特质吧，第一个是爱思考，然后第二个是动作快，然后第三个就是心态非常好，非常乐观，非常积极。

吕梦娜： 了解，那如果相反来说，有没有遇到一些感觉不太适合做科研或者不太适合未来走上这个科研道路的那些学生。他们又有什么特点？

受访者： 我觉得爱纠结的人做科研会很痛苦，因为科研的周期非常长，然后那种犹犹豫豫的性格我觉得不行，就是一会儿这样，一会儿那样。我觉得这个做科研一定要非常的坚定，就是因为这个东西反馈周期很长，你不坚定的话就不太好。

吕梦娜： 那除了这个爱纠结和不太坚定之外，还有什么特点吗？

受访者： 还有这个情绪不稳定，我觉得这是我的一个缺点，我做科研状态和我的情绪状态非常相关。然后我在光华的老师对我的批评，他说你要是情绪再稳定一点，你的这个状态会更好。还有我觉得不喜欢思考的不太适合，就是别人做了一个什么a到b到c的模型，然后拿过来做一个a到b到c的模型，也不想，就是什么事情不思考，我觉得这种也不太行。

吕梦娜： 那如果遇到这类学生的话会有去引导吗？然后引导会有作用吗？

受访者： 我不引导，我有的时候我会告诉他你不适合做研究，因为我觉得这不是引导出来，这个有的时候是个性特征，有些人就真的不适合做学术。但是有些人不适合做学术，做其他的很好。我之前带过一个我们人力专业非常优秀的学生，然后其实他做学术也做的非常好，但是我后来明显感觉到他对这个外面的世界更感兴趣。就是他喜欢这种快节奏、高压的这种生活方式。然后他做学术也能做的很好，但是我明显感觉到他能从其他的工作中得到更多的快乐。然后后来他申请这个夏令营的时候，我们就一起商量。后来他说还是决定去申学硕的项目，然后他前一段时间跟我说他拿到了那个xxx（一家企业）的offer，我觉得非常好，我觉得那个就特别适合。 我从来不会引导学生做学术。但是如果他很适合会告诉他你可以去尝试，但是如果他不适合，我绝对不会让他去做的。

吕梦娜： 然后下面一个问题，就是因为您在外文还有中文的一些比较好的期刊上都发表过很多学术文章嘛。那我想知道如果以你们的经验来看的话，在好的期刊需要具备哪些条件呢？

受访者： 百折不挠。发文章真的这个很痛苦，然后会遇到各种各样的事情吧，就是一个宗旨，就是只要他没拒掉，你只要还有机会，你就要尽120%的努力去把他的这个东西全部搞定。

吕梦娜： 了解，您刚刚提到了百折不挠嘛，这个比较类似于个人的品质或者是特质方面的，那还有其他的条件吗？

受访者： 选题很重要，你那个选题一定是a的选题。如果是 a的选题，你后边数据可能有些不完美，然后你再写的不完美，然后这个能发b就不错了。但是如果你开始这个选题就有问题的话，这个后面就一点救都没有。

吕梦娜： 那作为研究者或者作为学者的话，我们在发表这种高水平期刊的文章上还需要具备哪些条件吗？除了选题和百折不挠之外。

受访者： 模仿，我觉得模仿非常重要。因为我们现在如果按照美国这个主要的标准，二流还没有达到，就是还达不到引领潮流的这个。我觉得如果想发顶刊就是模仿，你去琢磨他的文章，然后他要什么样的一个范式，我们要善于模仿。

吕梦娜： 了解，那您就是对比一下在中外文期刊发表上的这个经验的话，您觉得自身在这两种期刊上发表有什么优劣势吗？相对你而言。

受访者： 优劣势，你指的是什么呢？是说中英文有什么不一样？

吕梦娜： 就是您在中英文期刊上发表文章的时候，比如说你在中文期刊可能会更有优势一些，或者是在外文期刊那种优势一些。

受访者： 没有，我觉得都是一个充满随机性的过程。就是以前我们上学的时候有一个美国的老师来，就说他发在a的期刊上的这个文章不一定是他最得意的文章，他最得意的文章有可能发在一个b的上面。就是也有可能有一些异常顺利，有可能会一直不顺利。比如说我的那个《管理世界》发出来，然后就是8月份投稿，12月份接受，多顺利嘛。但是等到我再投《管理世界》的时候，就是两年半审了三四轮，然后最后一轮拒掉。所以这个是一个充满方差的一个过程。但是总的来说哈，当时那个美国来的那个老师就跟我们说，他的一个感受就是他说是一个鸟枪法，就是你肯定投的越多，最后你整体接受的会越多。所以还是要勤奋。

吕梦娜： 了解。那你是怎么去定义一个好的研究呢？

受访者： 好的研究哈，第一它是一个make sense的研究，就是它是一个合理的研究，然后第二个就是它能够给我们的生活有一些启发的研究，这是第二个层次。然后第三个是他能够对社会产生一些影响力的研究。

吕梦娜： 了解，您对这个社会影响力是能具体展开吗？因为现在可能有一些争议的，或者是说希望管理学者能够做一些跟管理实践挂钩或者是贴切的研究。我不知道您是不是指这个意思呢？

受访者： 因为我还没有做到，我不知道。但是也不一定说一定要指导管理实践。我其实并不觉得说我们做研究一定要直接要指导实践。

吕梦娜： 那我们做研究的意义是什么呢？如果说不是去指导实践的话，那我们现在做这个理论研究的意义是什么？是更多是贡献于理论方面的这个领域吗？

受访者： 这个看你怎么想，如果你是从个人的角度来说，它是为了满足你个人的一个求职的欲望。如果你把它看成一种职业，它也是一种自我价值的实现。

如果你往大的方向去看的话，就是我一直觉得是这样的一个关系，就是你去看那些实践中他们用的很多的这个东西，其实不一定是我们现在研究最潮的那些东西。你像现在很多企业在做okr，你也做人力的学生，但是okr他不是现在提出来的，很早就都提出来了。我之前有过一个感觉，就是我也是觉得我们做这个研究就一定要指导实践。但是我后来发现不是这样的，我们做的一个研究好像是往这个兵器库里面去填东西，然后实践自己去从里面选自己要用什么。

我有这个感觉，是因为有一个学者叫陈春花，然后他确实干企业的嘛。然后有很多人就听了他讲座，觉得这个非常有有insight，然后非常有思想什么的。然后我就特意去把他的那一套书全买来，我看了一下，我就发现从学术的角度就是nothing new。然后我就在想，为什么这些老板听了就会觉得他特别有sense，就会觉得他讲的非常好。后来我发现在于说他可以筛选出那些最有用的东西，就是他讲的那些东西nothing new，但是他能够把这个兵器库里最好的东西筛选出来。所以我后来就调整思路，就是说我们不一定说给自己那么宏伟的一个目标，是吧？我今天发了一个研究，然后可能马上就让一个企业的performance好了，我觉得不现实。我觉得就是在我们了解现实的基础上，我们去做一些我们认为有意义的这样的一些研究。然后如果他是金子，然后有一天就会被人发现。

但是在我们做研究的时候要知道外面的世界我是这么看的，

吕梦娜： 明白。那您是怎么关注到领导力这个研究领域的呢？我感觉发表的这个论文来看，似乎您一直很坚定的在这个领域里面深耕。所以我就想了解一下你是怎么发现这个领域的？以及这个领域现在有什么特点来吸引到你的呢？

受访者： 就不需要发现，你也是做OB的同学，OB的同学有人不做领导力吗？清华的那个姓名都改成叫领导力与组织行为学，是吧？它本身就是OB里边非常主流的一个或者在商学院非常主流的一个东西，你说哪个圈尤其是OB的人，一点儿领导力的研究都不做，我觉得这个也不太可能。就是你不做领导力的研究，反而是你不太那个什么。

但是你说深耕还是有一些这个其他的原因，一个是我导师，然后他就做这个，所以可能有一些路径的依赖。另外一个就是在早期还没有自己领域的时候，就是去看发什么。我记得我们在读二年级的时候，我们当时老师组织我们大家一起去review各个顶刊的文章的topic。然后我们当时就总结出了，至少我们那个年代，我不知道现在哈，我们那个年代最热的两个topic，一个是leadership，还有一个是creativity，现在其实这两个话题也是（最热的）。

那个时候我们是真的去找哈所有的ab期刊看，我们去总结。然后后来那个老师还带着几个师弟把那个东西整理成了《心理学报》，你们可以去看一下，当时我们就去摸规律嘛。

吕梦娜： 了解。然后我想了解一下您现在来看的话，你觉得OB这个大的领域这个学科在中国的发展情况如何？以及它具有哪些特色呢？

受访者： 哇，这个太大了，这个你应该让大牛回答，我要能引领方向的话……

吕梦娜： 在我们学生这边您还是有很高权威性的。

受访者： 这个我……但是现在肯定是有一个共识哈，就是OB的研究要有一些转变。我可以给你们说一个很具体的例子，就大家都有这样的一种感觉，你看我们今年去招学生的时候，我有一个师妹来这儿，我有两个师妹，一个师妹发了AMJ，是第二作者，还有个师妹是第一作者，拿了JAP的RNA，然后中财都没有给他们offer。然后要了浙大的一个学生，而且这个浙大学生，是大家一致认为他非常好。就是他做的东西非常酷炫，然后做平台创意这些有关数字化的一些东西。所以就是OB如果还是做那种非常纯传统的，我觉得会很难有出路。

那你说具体怎么转我也不知道，但是我可以跟你们分享，就是我现在做的这个东西。一个就是我现在做一些稍微有一点技术，就是比如说我现在在做一个东西叫做auto leadership。它其实跟“算法管理“有点像，但是它是放在leadership这个领域去研究的。以前是人来领导我们，我们以前制造机器的时候，我们有一个基本的机器人定律是说，我们机器要受人的控制嘛。但是现在其实很多时候我们是在受机器领导，那么这种新型领导力有什么不一样？就是我觉得可能要做一些稍微不一样的东西。

然后再一个我觉得我们要和这个大数据有一些结合，就是不能再做传统的这种心理学的研究，就是用实验、问卷。我现在其实都已经有一些尽量避免用这个问卷，或者说是实验，最好能有客观的二手数据来做。这个我觉得肯定是要变一下的，因为你这种问卷也好，实验也好，不够客观，而且也太卷了，也没什么特别新的东西。我觉得还有就是要学一些新的技术，还有就是和其他可以有一些交叉。我也没有特别多的感受哈，但是在做一些尝试，就是因为我老公是做金融的嘛，然后可能大家会一起做一些有关东西。就是我觉得可以做一些交叉。就是肯定要做一些稍微有点不一样的地方，因为传统的OB不好做了，而且也不够酷。

吕梦娜： 那我听起来好像是传统的OB和现在就是你说的比较酷炫一点的OB，他们主要的差距是在于研究方法上面，是吗？

受访者： 不一定，研究主题也不一样的。

吕梦娜： 对。这是我们发展的一个转变。

受访者：这不能叫发展转型，这是我的一个转型，你说OB怎么转型，这我真不不知道。

吕梦娜：那你现在看我们中国的这个发展情况来看……

受访者：可能你这太大了，这个我真不知道这个，我要知道我就去当领导了。

吕梦娜： 明白，明白。那我们就先跳过这个问题。因为现在您就是在家庭方面也有一些事情嘛，然后您当前如果就只看工作内容的话哈，您主要包括哪一些呢？如果说能够赋予一定的比例的话，比如说都是十分制的话，你会怎么去分配你的时间呢？

受访者：我们的工作就是教学、科研和社会服务呗。

吕梦娜： 那你具体有什么比例呢？

受访者： 我想最多的肯定还是科研。因为我现在这个阶段我觉得要占到六吧，然后教学我觉得能占三左右，然后剩下的这个服务是一，服务现在在往上走一些。当然我对学院的服务可能不像刘老师那么多了，但是我现在有意识的去了解一些实践，有一些结合，但是总的来说还是写，我的生活本来也比较单调。

吕梦娜： 那这个社会服务主要是不是说学校里面的一些行政工作？

受访者： 学校也有一些啦。因为我还在做我们系的这个副系主任嘛，然后偶尔有一些委员会的活动。 像今天我来学校就是有那个招生宣讲。但是这些活相对来说是就是领导分配任务去做的，就是我还没有去要自己initiate一些东西。

我不知道你们有没有感觉，其实别人分配你做任务其实是很简单的，因为不需要过脑，你就做就可以了。但比如说像我们系主任，我就觉得副系主任和系主任差别还蛮大的。就是他做系主任他可能就会要想一下我们这个学科的一些东西或者系里边的一些事情，那我就不需要想这些东西。我基本上就是配合就是完成任务，我觉得这个占用的时间会非常少。

吕梦娜： 了解，那您就是作为学者，作为科研工作者，您认为就是在整个学术职业发展当中都需要哪些能力呢？

受访者： 你说学者的能力，是吗？

吕梦娜： 对，就是需要具备哪些能力可能才适合走上这个学术道路。其实前面你也提到了一点嘛，因为现在你已经是大学老师了，可能比我们这种的博士生小白要理解的更多一些。

受访者： 我觉得第一个就是对生活的一个敏感，就是你们对生活中的很多事情是有感触的。我觉得这个非常的重要，就是那种比较敏感的人，其实这个有好有坏。可能生活中敏感有的时候会比较累，但是这个敏感对于我们寻找idea什么的还是有一些帮助的。我觉得首先是要对生活非常敏感。

然后第二个就是要能够接受这个学术当中最枯燥的两个部分。一个是这个反馈的周期非常的长，还有一个就是有巨大的不确定性，就这两个你要能够接受。你像刚才提到那个高丹雪嘛，然后他后来去业界了。他也发了很好的期刊，我说你怎么这么可惜，就不做了嘛。然后他就说他实在忍受不了这个反馈周期，让他现在做行业，他去看一个项目，就是你是好是坏很快我就能得到一个反馈，我这个看准了没看准了什么，这个市场的反应非常快，然后每天生活工作有一个比较清晰的界限。但是学术不是这样，学术就是生活和工作永远是混在一起的，除非你自己人为的划分，然后永远有不确定性，然后永远是巨大的漫长的周期。就是你能忍受学术最negative的这些，那我觉得这一点很重要。

然后再就是再就是一定要这个百折不挠，我觉得还是要非常非常坚韧吧。

吕梦娜： 就是学者还是那种得能够坐冷板凳。

受访者： 我其实不喜欢用冷板凳，这个怎么会是冷板凳呢？就是你的生活也可以很丰富嘛。

吕梦娜： 那您怎么理解这个丰富呢？因为刚刚你还提到说比较会简单一些，您这个丰富是指什么呢？

受访者： 我觉得这个丰富是你自己的一种感觉吧？你像我有的时候觉得看paper、读书，你会觉得你的生活会很丰富，这个就是你精神上的一种丰富吧。就是你说天天逛街你就丰富了嘛，你就开心快乐了吗？

吕梦娜： 那最能够让您体会到这个学者身份给你带来的这种正向感情是什么时候呢？

受访者： 有了孩子之后，就是老师这个工作确实很宽容，尤其是我生完孩子之后是赶上疫情了，然后周围很多朋友压力非常大。比如说他们有些人要兼顾家庭，可能就辞职了。然后还有就是经济不好，有些可能就面临着减薪或者裁员。然后我现在是对老师这个工作真的发自内心的充满感恩，就是我觉得他是我能想到的最能帮助女性来平衡工作、家庭的一个很好的一个职业。真的是这样，我觉得我最近两年半有了孩子之后，我对现在的工作真的是充满了感恩，这是实话。

吕梦娜： 对，明白。那我之后再有一个小问题，就是你可能或多或少也听闻过当前高校推行了这种“非升即走”的制度，或者说变相师资博士后吧。那您对这种制度是怎么看待的呢？如果你身边也有这样的朋友，或者是师弟师妹，也在接受这种考核的话，你也可以结合他们的经历谈。

受访者： 首先我是觉得这是一个非常不好的制度，但是现在都是这样，就没有选择，你说他们有什么选择吗？

吕梦娜： 是的，其实你觉得不好，很多人都会觉得不好，那为什么我们高校会在越来越普遍的去推行，不仅仅是211、985甚至这些普通的高校也会在推行这个。你觉得有什么原因呢？

受访者： 供大于求了嘛，现在不缺人才呀。你像现在你不来他也会来呀，就是学校有很多的选择。你像这个现在中财就我们学校想招一个人有那么多，是不是？

吕梦娜： 对对。好好谢谢老师，耽误你很长时间了。然后我就问到这里确实，能够跟你学到很多东西。好，谢谢。

刘书博老师： 我觉得梦娜刚刚提的问题挺好的也挺全面的。另外我补充一下，就是张老师刚才说的这种丰富和简单。那丰富就是如果你是一个追求智趣生活，就是比如看看书，你都会觉得天天看书很丰富，但有一些人会觉得很枯燥，所以这是两种不同的理解。所以这一点我觉得是主观的感觉。你像有的人天天逛街，他觉得特别丰富，但是有的人会觉得特别枯燥。

 另外就是“非升即走”，那我觉得除了这个供大于求，还有就是我们工业社会都是这样追求效率，效率至上。那这样的制度会短期效应最大化，所以它的效果是非常清晰的。所以其实我们学者也是受到这样工业思潮的影响。

（秋莉，你接着问，我们这个抓紧时间。）

受访者： 咱们争取半小时内结束因为我确实要回去看娃去了

黄秋莉： 好，那我就长话短说。那第一个就是您提到徐淑英老师来了之后嘛，您觉得他办那个项目特别好。我就想了解一下，就是徐淑英老师来到光华以后，就是在光华做了哪些事情？就是有没有给光华带来一些比较具体的改变？

受访者： 有，这个以前光华培养方案是一个样子，然后她来了之后就把美国这一套搬过来了，然后她来了之后然后就开始主导招了很多在国外的这个老师过来，就相当于把美国这一套就搬过来了。我觉得这个是非常直接的一个影响吧，就是我们现在整个的这个研究范式肯定和之前是不一样的，就以前大家都不知道定量研究是什么

黄秋莉： 可能从国外回来的一些老师，给你的第一感觉是他们比较具有激情嘛，就是可能半夜两三点还会给您发一个邮件之类的。除此之外他们还有一些别的不一样的特点吗？

受访者： 我觉得这不是国内国外的区别，国内有很多特别有激情的老师。

黄秋莉： 您觉得从国外回来的老师他们和以前光华的老师有一些不一样的地方吗？

受访者： 因为光华国际化特别早，我去的时候那些老师基本上都已经是从国外回来的。

黄秋莉： 都是从国外回来的。

受访者： 给我们上课的都是国外拿PhD的老师，好像我也不知道国内是什么样。但是我觉得至少光华和中财的培养不是特别一样。

黄秋莉：因为您提到您目前正在一个可能要评正教授的这一个阶段嘛。那对于您来说您会觉得比较有压力吗？

受访者： 会呀。

刘书博老师： 今年这个中财突然把那个标准提高了好多，他只算第一作者，以前是认通信作者，现在不认通信作者，不知道谁定的？那为什么改成这样？

受访者： 对呀，而且他以前说有没有项目都可以嘛，至少他没有明确说一定要有一个项目。今年是明确必须有一个国科。

刘书博老师： 所以我们前面提供的意见他们等于是无视了，就装模作样的要征求意见，征求完该怎样还是怎样。

受访者： 对呀，而且还要求有教学的项目，我这两天还在想怎么办。你有没有想试那个教学发展研究中心的一个项目。评教授还要有一个教改的项目，然后还要有别的，我那天看好几项。

刘书博老师： 我大概看了一下，因为我还没有特别着急，因为张老师马上要评了。所以我看了之后我就觉得首先我得先把文章给凑够，然后文章我必须是第一作者，然后这一点就我觉得特别不合理。因为有的学校人家改是反着改，就是之前只认第一作者现在改回到也加上通讯。咱们是越改越到了越回到这种不合理的境地了，也不知道怎么回事。是科研处他们改的吧？

受访者： 我也不知道。我今年倒也不评，我是明年评。但是我拿到之后我就觉得好焦虑，因为我现在还缺一个自科，还缺一个教改的项目。尤其是自科这个不确定性非常大嘛，然后所以就顿时有压力了。之前觉得好像还可以，现在就觉得压力很大，肯定有压力呀，这个这个毫无疑问。

黄秋莉： 是的，是的。就是刚刚您也提到您可能不太喜欢“坐冷板凳”，你觉得不存在坐所谓这个坐冷板凳嘛。那也有些人就会说可能评上教授了之后就是上岸了，那如果现在是处于副教授呢就处于一个未上岸的阶段，那您对这个上岸的内涵您是怎么样理解的？

受访者： 我觉得我内心还是对那个学术还是有一些热爱的，包括我现在很在意这个东西，可能就是觉得稍微达到这个要求可以松口气，但是并不表示说我之后就不去做了。因为现在职业生涯最多也就算到中期，那还有很长的路，我觉得和我后边会不会继续投入学术没有任何的关系，就是我还会继续投入，但是它会让我有心理压力。

黄秋莉： 是，就刚刚也提了你可能对学术这样一个追求。那您有没有想过自己未来的一个职业的规划或者是定位是一个什么样子的。

受访者：我没有想具体，但是我还是很想发一个A的。

黄秋莉： 您不是已经发了很好的那个文章了吗？

受访者： 我没有以第一作者发过A呀，然后我觉得就是不管做什么，这是我的一个职业嘛，我肯定是希望我至少能得到我这个职业标准的认可，我是需要别人肯定的一个人。

黄秋莉： 明白，那您就是觉得一个合格的学者是什么样子的吗？就是合格学者他有没有一个什么样的标准呀？

受访者： 这个标准不是我定的，学校“非升即走”，你能留下来说明你就合格呀。

黄秋莉： 就是要符合外在的规定。

受访者：我认为这是基本的嘛。你如果从职业的角度来说，这肯定是基本的职业要求。

黄秋莉： 那达到一个优秀的学者嘛，就是在现实生活当中有没有让您特别特别敬佩的学者？

受访者： 有，我很佩服徐淑英老师，我很佩服她，我觉得她给中国的学术界确实带来了很多的改变。当然有些人说也许是不好的改变。但是我认为是非常积极的正向的改变，就是改变了很多人对于研究范式的这样的一个认识。包括她现在其实也在自己反思，包括定量定性的一些关系呀什么的。其实不管什么工作吧，就是能够让生活更美好，能够帮助到更多的人，我觉得这个的标准是一样的，优秀的学者和优秀的其他职业的人标准是一样的，就是你能够帮助到别人，就是一个好的标准。

黄秋莉： 学者有一个社会责任哈，就是有所创造。那我就问最后一个问题哈，也不耽误您太多时间，就是因为您先前提到就是这个就光华而言，那可能就是离美国的这种标准还有一定的距离嘛。那我们现在也是国家层面提倡这个要创世界一流，争夺这个学术话语权，就你的这个视角来看，您觉得我们国家有什么样的一个出路吗？就是只学习吗？

受访者： 首先哈，我不喜欢说这个“争夺话语权”，我觉得这个话语权不是争夺来的。然后第二个问题太大了， 这个你开始问我这个商学院，然后学科，现在直接问我国家，我这我真没法儿答，这真没法答，这太大了。

黄秋莉： 那我就没有什么别的问题了。

刘书博老师： 好。那我们再次感谢张老师，特别宝贵的一次机会让我们能够这个深入了解到我们这个优秀的学者的成长之路。我觉得今天下午我受益匪浅，更多的是两位同学受益匪浅。我们后续还争取有更多机会能够跟张老师来进行交流，包括这个具体专业的学习，还有就是我们有一些生活经验的交流，张老师在生活上也做的特别好

受访者： 每个人的模式不一样，我现在每天会有各种新的问题。

刘书博老师： 我就特别佩服，我觉得学术虽然比较自由，但是其实压力也不小，它弥漫在我们的日常中，尤其是对于一个带孩子的母亲来说，可能要兼顾两头吧，都是比较操心费力的事情，所以这个过程还是挺不容易的。

受访者： 但是千万不要打退你们哈，我对两位女同学说，就是真的，如果你们想找一个能够兼顾家庭的工作，真的高校老师是一个特别好的选择。就是压力再大的话，我觉得外面工作压力会更大的。

刘书博老师： 对。而且张老师很多优秀的同学、校友，他们都是在这个做创业企业，而且张老师自己也在跟企业做很多的这种案例研究式的工作。所以有机会我们一块儿这个探索一个方向，一块儿有一些成果出来。

黄秋莉： 行，好，谢谢。好，谢谢书博，谢谢两位同学，好的，拜拜。

刘书博老师： 我们先这样，好，再见，周末愉快。

# 受访者8

刘书博老师：那我们用一个回溯式的视角看，黄老师一直在民族大学求学，那在什么时间确定进行学术事业的发展，当时有没有想过去行业到公司，为什么选择了读博士？为什么选择走学术道路，当时有什么想法？

受访者：应该是研究生期间确定了想要继续去做学问。原因可能是在读书的……其实我觉得从本科到研究生的这个转换还是挺重要的。然后这个过程中我觉得核心的、灵魂的一个关键人物就是自己的导师，因为自己导师的出现，然后让我对于整个人生的发展有些比较大的不一样的看法，因为以前本科到研究生那会儿，就会觉得说将来毕业之后找一份好的工作。可能当时很多研究生的目标是为了以一个更好的身份去实现更好的就业，找到更好的机会，去更大的平台。

然后遇到自己导师之后了，其实是他给你很多的一些灌输，可能他对我自己的有些潜力的看到，然后他也是对我很多的引导，就觉得可能做学术会实现一种宏伟的抱负。就是说无论是你在读研究生，还是说未来自己想去就业的时候，其实每个人以前的心中都有一种很宏伟的抱负。所以呢也是在跟导师做很多的项目研究过程中，导师让我知道了其实这种阳春白雪式的学问，或者说其实你去做一些研究是可以从更大程度上去实现你的这种所谓的宏伟抱负。其实这个所谓的宏伟抱负是咱们每个人都想说成为一个对这个社会有用的人吧，就是想做一些有价值的事情。我想这可能说起来很抽象，但其实是当时一个非常确切的、具象的想法，就是可能想成为一个有价值的人。那么在做学术的过程中，首先你也会发现到了研究生去读书的时候，当你读了更多书的时候，你会发现整个人会更多一些思考。然后呢在导师的引导下其实是特别关键，他让我认识到学术这条路的魅力。所以说从跟他读之后，其实就慢慢的产生了这样一种继续去读博士、去做学术这种想法。所以这个关键人物其实是导师的影响是很重要的。

刘书博老师： 嗯嗯，那就是有一个非常关键的导师启发，他也是志业的一个引导者。那刚才说研究生阶段，然后再到博士阶段，博士阶段可能就更加的系统化、更加的成果导向、要发论文了。那这个过程中有没有一些意外的或者是跟你之前想过的这种价值创造为中心的不是特别相符的一些事情。

受访者： 嗯嗯，那肯定有。就是其实不管是在读博期间也好，还是说现在毕业工作之后，其实继续做学问这条路的过程中肯定还是有产生一些迷茫，首先是在写博士毕业论文的阶段吧，其实我想每个人都是很迷茫的。因为做博士论文就是在一个汪洋大海中去寻找自我的这种感觉，所以说是非常的一个孤独的过程，在那个过程中其实每个人都会想过放弃。所以说其实第一个挑战我觉得就是博士毕业论文，因为毕竟做博士的这个学位论文跟你以前的论文都不太一样，它的意义、它的要求也不一样，思考是非常重要的。但我觉得也还是很得益于当时的导师，就说我在写论文的过程中他的战略和战术的指导。他就是让我一定要去读更多……因为我们是搞人文社科的，所以说他没有让我去做太多这些叫技术性的工作，他也不希望我在论文中去呈现这样一些……因为像我们经管类文章，可能有些博士论文就是拿一个数据模型，他可能就成了一整篇文章，最后呈现出来没有什么太多理论的意义。其实包括我导师，也包括在我读博士期间遇到的一些老师，就是不断地跟我们在强调要去跟理论对话。我一开始就一直有这样的一些灌输，要去跟理论对话，那理论是什么？理论在哪里？这个都是自己要去寻找的。所以说导师给了一个方向，但是呢导师没法带着你做，就全都得靠自己在汪洋大海中去探索，所以这个过程中其实人是非常的孤独，所以说也是非常的难做的一件事，艰难的一个时刻。包括师门里头也有50%的流失率吧，就是他们读不下来。其实就是在做博士论文的过程中没法做下去，因为他找不到……可能做着做着心里头就产生了一些比较困难的情绪。所以就是在这个探讨过程中，我觉得可能想过放弃，可能这个博士论文是我面对的第一个比较大的难题。

但是还好一点，就是导师包括别的老师从我博一开始就要求我做理论对话，所以说特别注重去看书读书，所以说在书本去找到真正的理论去跟这些人对上话，然后呢再去开始论文写作，慢慢才展开了。所以说我觉得第一个可能是在博士论文阶段，就会让我产生一些犹豫。

那么第二阶段呢其实是在毕业的过程中，院校对博士生有很多的一些要求吧，就是各种发论文的要求啊，各种方面的。可能我们这边还好一点，因为我们的要求可能也没那么高，我们那时候就是你能发出两篇C刊就可以了，然后导师当第一作者的就视为你是第一作者。所以说我们那时候对博士毕业的考评标准不算太高也不算太低吧。所以说在毕业要求硬条件上可能没有造成太大的阻碍。

然后现在工作之后呢其实是有一些。因为你会看到就是主流的很多期刊都还是以实证模型为主。我们之前导师的训练方式，不像别人是拿模型去训练你，他是拿这种理论来训练我的。所以说我们可能在理论研究上会比较擅长或深入，但是现在在发表的过程中，如果你不做实证模型又比较难（发表）。所以你就会陷入一个比较尴尬的局面。然后你做理论呢，如果一般的这种情况，就感觉有种社会上的默认，就说你不到一定的水平和学位是没法去承认你的理论研究的。

我今天正好给研究生讲课，我给他们上《现代学理论》这门课。然后今天正好讲的是科斯的一本书，就是《企业、市场与法律》。其实科斯人家提出这个交易费用、就是在写企业性质的文章的时候，人家才大学本科毕业生，那么年轻的一个人就写下了企业的性质。因为他当时跟着老师去考察企业，然后他就去思考，所以他写出了这个企业的性质这篇文章，在里头就已经提出交易费用的这个概念。但是呢长达五十多年的时间并没有人关注他，压根就是当成一篇毫不起眼的文章嘛。然后直到1991年，他都已经年纪很大了，那个时候才给他颁发诺贝尔经济学奖。然后给他颁发诺贝尔经济学奖，最主要核心是因为他在经济学中看到了交易费用理论的这个概念，这个就是在《企业、市场与法律》那篇文章里。他（科斯）就讲到“我提出了这样的理论，一个看似很简单的理论，不证自明的理论，但是不为学界所认可”，然后当时的学界大家都是去研究没有人性的消费者、没有组织的企业，他是这么认为的。然后他觉得在那个时候经济学家对整个人性的认识是苍白的。这都是他当时在文章里写的，这篇文章是他获得诺贝尔经济学奖之后写的，其实某种程度是对自己学术的一种反思和总结吧。

所以说他虽然很早提出了交易费用的理论，但是其实并不为学界所认可。而且因为那个时候的学界是什么呢？他说学界是去追求于很精致化的、用数理的模型去探讨。所以其实咱们看到就是时代其实在发展，但是呢很多内容并没有改变。

刘书博老师： 现在管理学好像也是依然是追求精致化，他会把精致化看作是专业化

受访者： 包括现在的经济学其实也一样，也都还是。所以为什么说他们都要强调用这一种实证的模型去做。其实我们不是要去批判实证模型，而是说做完实证背后要有用，就是要能产生价值，这才是最关键的一个作用。就是其实经济学应该是去阐述这个经济现象的，而不是去进入到自己的一个经济学的世界里，然后自我满足。然后他（科斯）说当时的这种分析其实都是一种空中楼阁，看着是匠心独具，最后他把它叫做“黑板经济学”。就好像是你教授觉得这一种假设、设定、条件的约束啊等等做的特别完美，但是现实世界并不是这样子的。所以你想这个放到现在来说也是。

所以像咱们这种做理论吧，在外在的这样一种评判下也没办法，因为作为青椒你必须评职称。所以说是有这样的一些困惑，就是外在的评价标准还是要让你 “带着镣铐在舞台上精致地跳舞”，但是你又想赤脚踩在田野上去探讨经济的本源。所以说你在这一过程中其实就会对你自己产生一些怀疑和挑战。

刘书博老师： 是的。我觉得你刚才谈的这个也是一个共同的问题。因为前面我们访谈过一些同行老师，也经历过这种所谓的分裂吧。然后有一些老师是皈依了，有一些老师是犬儒，有一些老师呢是上岸的心态，都有不同的这种心理建构。那刚才你回忆的XX大学好像是还挺具有特色的。因为现在我们看到以光华为代表的这些商学院，他们都是在精致化的路线上进行所谓的专业化。不会特别强调说价值导向的、理论建构的。那他们就是完全的工具主义的，非常的精细。所以XX大学这个特色是一直以来都有吗？

受访者：应该是，我觉得可能确实是不同的学校有不同的氛围。然后本身可能XX大学起家就是社会学和民族学嘛，有好多民族学和社会学的大师，XX大学有这么个辉煌的年代吧。所以我觉得这可能就奠定了这个学校的学风、校风、风格，可能就还是以理论研究为基础一些。所以我觉得可能跟这个学校有关系啊，包括你看像学校的教训就是美美与共，知行合一。就还是很强调社会调查跟理论之间的结合，所以我觉得可能是这个学校本身有的一些风格。然后呢我们XX大学的很多老师又是那样一个时代培养出来的人，所以他们就传承了那样一代的精神吧，所以我觉得可能有种精神的传承在里头。

刘书博老师： 嗯，ok，不同学校有不同的气质面貌。我们还计划访谈以光华为代表的这一派系，就很好奇北大为什么孕育出了光华，因为北大一开始也有一些“大家”嘛，但现在会特别的美国化。所以我们就回头再看一下他们，他们到底是怎么样的一个面貌。

那在发表论文的过程中有没有遇到一些挑战？你像你刚才说的，现在好多还是追求那种量化模型的或者是美式的那种实证主义研究。你在这方面投稿发表，有没有经历一些故事，让你会感受到好像是有一些阻力、是有一些不同？

受访者：这阻力其实对XX大学来说更大，因为在咱们这种政治氛围之下呢，每个学校有自己的特色，对吧？那XX大学的特色肯定是民族，毫无疑问的。所以说这也是为什么国家设立你的原因，就让你去研究民族地区，研究民族问题。但是呢你扯上民族，期刊会认为这属于这种政治问题，把民族都等同于政治。所以现在所有的主流期刊，就是像咱们经济学管理学的这种主流期刊，只要摊上民族，那么一般的就是直接拒搞，就是基本不会发。所以你只要去回看他们发的文章，没有一篇是带民族的。

然后因为我不可能去民族学的期刊去发文章，我们是搞经济管理的，所以我们只能往经济管理的期刊（去发表）。而且我们学校现在搞这种双一流建设啥的，最后还是得看主流期刊发表嘛。对吧？所以说我就得去主流期刊发文章。但是呢这种主流期刊呢只要看到你民族，肯定就是拒绝。所以说如果我做特色，那么我就发不了，我可能只能去一些就是很普通的期刊去发文章。

刘书博老师： ok。

受访者： 但如果我跟随主流那就没有特色。没有特色的话，做的文章永远是跟着别人走。所以说你没有特色，那么你自己在学术界里也就没有特色了。所以说其实对于XX大学来说有更大的一些限制，就在于我觉得可能大家还不够开放吧，或者说可能需要更长的时间让大家去接受。

其实民族也是一个学科，就是这种学术体系可以去研究的内容。因为他们（主流期刊）害怕，因为一些研究导致的政治失误，所以说主流（期刊）一般都不发。

还有很难的一点是在出书，现在只要我摊上民族的、历史边疆的等等（话题）。我们的书都属于重大选题，那么就需要报到国家部委去审批，任何一本书（都需要审批）。所以任何一本书，我们的出版周期都特别的长。我们好多的人基本上排个三年、五年，你想，全国有多少个搞民族（研究的），还有做这种边疆的、历史的都属于重大选题。所以这些东西都要归到上级政府部门去看。全国这么多的人写这么多的书，一本书又是几十万字的，猴年马月，所以我们只能无期限地等待。所以这可能是在这种政治环境下，已然有的一些更难的方式吧。

但是即便很难也得往前走，因为你不做这个问题就更没人做了，因为你作为XX大学的民族领域的研究学者，你不去做那么更没有人做。所以说其实大家只能继续往前做。但往前做就很难发到一些好的主流期刊上，这种基本挺难的。我们要么就是得换选题，要么就是……反正其实在生存和发展这两个方面自己去探讨了。

刘书博老师： ok。那个我昨天也是在规划处开会，然后了解到新一轮的学科评估，他们就改了一个要求，就是每个学校只能报40篇研究成果。那不像之前我们可以无限量的去报，所以中财在数量上总是第一嘛。那现在有这样的一个框架，可能我们在质量上就达不到。像对外经贸、人大，他们可能最基本的也是《经济研究》这样的（等级）。那可能这个问题对于XX大学更严重吧？因为你说的主流期刊他们会有一些政治考量，所以就会影响到我们学科评估的这个位置。

受访者：是是，所以可能很多人采取的措施是什么呢？就是只能先往后退，就是我只能先求生存，那么我可能只能先改变自己研究的一些方向，我先按着主流的东西去做，做完之后呢，可能像对个人来说我实现了职称的评比了，那么我再来专心致力于我真正觉得能做出学术特色的东西。

然后对整个学科来说可能也是（先往后退），因为现在XX大学像经管学院还没设立下博士点嘛，所以说我们现在都是挂在民族学底下，这个很大一个原因就是我们在主流期刊发表的文章量，因为博士点的建立跟学科评估是一样的，就是以前的标准就是你发了几篇a呀、b呀、c呀，就类似这样的一些标准。但是在那个评价体系之下，那XX大学的学者做民族学，民族学又发不了主流期刊，就是陷入了一个死循环吧。所以说那怎么办呢？你这个学科想发展，你总得先立住脚吧，那你只能够让大家先去往这方面发展。然后你先按照主流学科的要求去做，做完之后，等你将来真正有学科了，你自己再去发展你的特色。我估计可能只能采取迂回，就是曲线走路的方式吧。

刘书博老师： 嗯，明白

受访者：现在个人、学科和学院发展的态势应该也是这个方向，就是迂回

刘书博老师： 就是先被一致化，然后再去追求特色化。

受访者： 只能这么做，要不然的话你都走不出来

刘书博老师： 嗯，是的，ok，那你们现在有没有进行过AACSB认证等这些国际认证？

受访者： 呃，没有，目前XX大学这种各类认证目前都还是没有做的。

刘书博老师： 那你们有计划吗？现在不是商学院都在搞这个东西，你们有计划吗？

受访者： 目前来看还没有听说，包括学校层面。其实说这种认证是与国际去接轨，但是我觉得目前来说XX大学这些领导们可能都还没有去关注这一点吧。现在可能关注点还是怎么与国内主流接轨，还没到世界（接轨），还没有看。

而且其实现在来看这些认证，我估计再往后是不是还有这么大市场，那也不一定了啊。因为这样一种认证体系全是国外来主导的方式，那么现在我们去强调自己的学科体系的时候，是否一定要依照着人家的认证、被牵着鼻子走呢？就是你完完全全融入到别人认证的话，那你就会牺牲掉自己的东西。那就跟企业一样，你完完全全按照人家的体系去融入的话，你还是被别人牵着走，就是你很难形成自己的一个特色出来。

而且我觉得现在其实有个很显著的特点，就是现在商学院院长换届，好像以前是咱们觉得叫职业派的，就是海归归来的甚至是华裔外国人这种外籍担任院长的，好像正在改变，这种改变的方式可能也会对这一种体系带来不一样的方式。

刘书博老师： 嗯，对，因为之前像90年代刚开始发展商科的时候，大量的美国留学回来的像张维迎为代表的（院长），他们就担任重要的比如北大光华的院长，所以从张维迎开始，光华的发展就非常的国际化嘛，然后美国的很多标准啊，研究的范式呀，用人的标准啊都是直接拿过来，包括钱颖一。好像现在这些院长不再是海外的华人学者或者是外籍。

受访者： 嗯，你看人大的商学院院长都换了，我觉得这可能也是一定的导向吧。因为我觉得那个时候呢其实也是我们国家发展到那个阶段了，以前老觉得商科一定就是市场经济啊，一定都是这种按照海外的这种方式来发展，包括咱们的商学院。

所以为什么我觉得说北大清华发展着发展着特色去哪儿了呢。就是感觉吧。因为你按照人家认证体系走、就跟着人家标准走，那是一个标准化的东西，你的这套方式设置全是按照人家标准去做，包括里头也是很看论文嘛，填表也让你去填你在国外发了几篇论文，而且他们还有一个数量叫什么国际化师资人才数。

我觉得以后再看吧，我觉得这种认证体系……确实（我们）是需要去跟世界对话，但是不是一定要去用你（国外）的标准？因为显然其实我们参与他的标准，也并不一定意味着能给你带来什么。那我们就拿来看一下，那现在清华北大早就进入AACSB这种认证体系了，那哈佛每年有几个学生来他们学校交换呢？

刘书博老师： 对，是的。

受访者： 所以我觉得这是很重要的一个评价。因为我觉得现在可能很多（商学院）是为了认证而认证了，认证完其实一个好处是说好像拿到了一个通行证，我可以进入到国际的商学院里头去。但你现在回看效果呀，你能往人家那边去，但是几个人来你这了呢

刘书博老师： 所以它是不平等的嘛

受访者： 是。所以说我觉得完全按照人家这个标准体系，说白了我们没有话语体系的。

刘书博老师： 其实就是我们是学术的代工厂，就是富士康的感觉，别人让你生产什么你生产什么，他们还是同一个体系嘛，都是生产的一个体系，只不过人家决定你的标准，你只能是生产别人让你生产的，所以学术代工这个感觉很清晰了。

受访者：我觉得咱们国际化并不意味着要西方化，我觉得这是两个很大的差异。国际化道路是我们去拥抱世界，但并不一定要按照西方的方式，就是把所有东西都改掉。其实咱们一度就是这样的一个方法嘛，所有的商学院全改成了西式商学院的方式，但最后其实有很多时候反而显得不伦不类了，就是你的特色在哪？而且落地的时候，中国的本土的一些管理现象你解决不了了。你全部拿西方的这种理论，你反而解决不了自己本土的东西。所以我觉得咱们现在老说自己理论的缺失，可能我觉得也与咱们那个年代是有点关系吧？

刘书博老师： 对啊，我非常赞同。因为我们是在一个所谓文化的边缘地带，然后我们想要去融入中心地带。那中心地带他们是有很多自己的先天的优势的，我们没有办法完全变成他们。而且这个历史文化、社会政治经济背景都不一样。所以刚才你说到的，我们现在这个院长都开始有这个成分的改变。

那还有一点我的问题是，也是前两年吧，就中央、科技部他们联合倡导把研究做在中国大地上。然后呢像你导师的导师也是在积极的推动。那这块儿你觉得经过这两年的政策出台到现在，有没有一些现实层面的改变。当然这个院长的成分改变是我们可以观察的，除此之外还有嘛。

受访者：首先是这种学界观念的改变。其实我觉得XX大学的氛围就在于它的包容性。XX大学在做什么事情呢，XX大学其实从校领导到学院层面都在积极拥抱创中国学派，就是拥抱这个变化。我觉得其实是反而说可能XX大学在当些年就这样，我们就以认证为例吧，那么以前大家都去做国际认证，去走西方式的商学院的方式，XX大学没有往前走，它是落后的。那么现在我们来强调中国学派的时候呢，XX大学往这边转，反而少了一些包袱，所以它可能变化的会更加积极一点。然后呢其实按照我们这边院长的说法，这叫做弯道超车。

刘书博老师： ok

受访者： 所以说其实也是可以去这么理解就是，首先呢像经济学、管理学中国学派有个六神论坛，这个论坛创始学校就是XX大学，而且XX大学还承担了这个论坛的办公室。这个六神论坛其实把这包括社科院、北京大学、清华大学、还有全国各大高校的经济学院管理学院院长呀，就是开始去探索中国学派这种研究的人成立在一起，一直在做。从18年开始，然后每年会有春秋季论坛、年度论坛，反正XX大学这边都在做这个事情。所以说这个六神论坛的影响力也是越来越大。

像金碚现在就提出来一个叫域观经济学，它的核心点是什么呢？就是说现在我们传统的经济学理论里是微观和宏观的范式，对吧，他说微观宏观范式的经济学理论，其实也是基于像牛顿的粒子空间一样，就是一个无摩擦的世界，最重要的核心是把经济学不能解释的因素全给抽象掉了，像我们说的民族、文化、风俗等等，把这些就是经济学不能解释的东西抽象掉了。所以说在里头每个人都是经济人嘛，咱们经济学最核心的假设就是每个人都是经济人，每个人都要去实现利润的最大化，然后我们每一个人的核心目标就要去实现资源的最有效配置。所以金老师他的这一套学术提出来就是说，其实在宏观和微观以前是史观范式，就是说在古典经济学、新古典经济学之前是一种史观的范式。你看像马克思也好，包括亚当·斯密，其实他们在做研究的过程中，他们都是拿历史在进行研究，然后过渡到了微观。到了微观之后发现不能去解释这些社会现象的时候，开始有宏观经济学把政府给引进进来。那么他认为到现在其实是存在一个域观，就是说不同的地区、不同的国家都有自己的一个域，那么不同的域会表现出不一样的域观，这个域观就包括文化属性、地理属性等等都是不一样的。

他说中国最大的域观特征就是中国共产党，那么他举个例子说，西方国家的宏观经济学里是把政府当成是应对市场失灵的一个角色。他说你觉得党能等同于西方经济学的政府吗？他说那是不行的。因为西方经济学说的那个政府是很有限的，就是很难发挥作用的。但是中国共产党是一个超然存在体，他去引领，你看咱们经济社会各方面，其实都是在共产党的领导下去开展的。它跟西方经济学这种政府当出现市场失灵时去规制是不一样的，所以说如果用西方经济学这个范式来研究我们国内的这些经济现象，你就会发现解决不了一些问题的，那你就会把党给抽象掉。但它是一个客观存在的现象，比如说咱们这么多年，特别是改革开放40年取得这么大的成就，我相信每一个中国人都不会去否认。这中间有很大一个因素是党的领导让全国能够齐心去发展。包括说咱们一代一代的过程，不是一代去否定一代，而是说有一个五年规划的形式去实现经济还有社会发展的脉络，所以说不能忽视它的这个特征的。不能因为说西方经济学理论里没有你这个角色，所以我就要把你这个给去除掉。他说这是一种削足适履，所以他说就得回到域观的特征，就要结合文化、政治、民族等等，你要去研究他的这个经济现象，不能抽象掉他。所以他在域观里头就特别的批判说，现代经济学和管理学是穿着非常漂亮的高跟鞋在跳舞，但是呢他并没有把真正的现象给反映出来，就说过度去追求这一种最优最大。但他的域观经济学强调的是次优，就是其实不应该最优化、应该是次优。那么适应、适合才是最主要的一个价值取向，而不是去追求最大最优。

所以说整一个落地到XX大学，像包括这种中国学派的成立，校领导都特别支持。其实不仅仅是说经济学这一块，包括XX大学一个青年长江学者就说，他之前在做中国特色的宗教理论，特别像基督教等等，就是有地域特色的，跟国外不一样。但是他那个时候呢在做这种过程中就是因为比较另类，在早些年很另类。所以说主流期刊都不发他的（文章），他只能在小期刊上发表。但是他说他很感谢XX大学很包容他的这种做法，就认为（XX大学）会去接纳他，给他很多资源，很多方向，让他可以很长时间去做某一个方向的一些研究，然后甚至把他推为青年长江学者。然后后面进入到现在强调中国特色，你看现在咱们一直在强调这种中国化的宗教嘛，然后一下子他的研究就成为前沿了。所以前沿跟非前沿我觉得看的是时代。所以说是学校对人的支持。

我们XX大学今年正式开始评职称实行代表作制，选三篇文章做代表作，不再追求你的数量了，而且只是说三篇高水平文章，而且不限定是什么文章，只要是三篇高水平文章，然后有什么教学，有什么著作等，就是你达到一定的条件之后你就能去评职称。然后呢也不看你发了几篇a、几篇b，每个人选三篇代表作去进行评比，这也是一个导向吧。

刘书博老师：你刚才说不看什么文章，是指不看期刊的级别，对吧？

受访者： 对，不看期刊，学校就只看你选三篇文章送出去，由三个人去评分，那假如说一分、两分、三分，那么至少你要达到五分以上才能进入到答辩的环节。

刘书博老师： ok，所以他就是有点说让评价权回到评审人那里，而不是仅仅期刊那里。

受访者： 是的，是的。而且他也不按你期刊说，比如你发《管理世界》可以算几分几分那种，就是不看这个了。现在你就拿三篇代表作出来，可以是文章，可以是著作，可以是你给国家递交了一个什么咨询报告。就是强调你的个人代表成果，只要你能达到五分以上，那么你就能进入到现场的答辩环节。答辩环节可能就更看重的是你这种学派呀学科的探索，这是一种好的评价方式吧。

黄秋莉：黄老师我想问一下，这是一种达标赛还是一种锦标赛呢？

受访者： 就已经不是达标，以前可能我们可以叫达标，以前那就算分嘛，然后呢到最后我们看谁的分数高，假如说我发了一篇SSCI，然后1区、2区、3区，每个区对应几分，那我加起来就我有40分，那么你只有20分，那我40分就胜利了，对不对？因为那个时候就是拿这种分数算。那么现在呢就相当于你只要达到门槛都能来评，这个门槛也低，只要有三篇高水平的文章就可以去评价。然后还要有著作、还要有课题，就是他对你要求更多了。就是你不能仅仅是发文章就行了，我们要求要有文章，要有课题，要有著作，要有教学的一些成绩。

黄秋莉： 嗯，好的。

刘书博老师：相比你们来说，我觉得中财还是换汤不换药、新瓶装旧酒，虽然说去五唯，但是我们还是强调在A类期刊发表几篇，还是在评职称的文件上标的很清楚。而且他更进一步说你必须是第一作者，通讯也给去掉。那个考核方式呢也是换成分数，然后分数再换成钱。所以他科研奖励也是同样的一个奖励，只不过中间加了一层。

受访者： 那确实是换汤不换药。我们以前就只认第一作者，但是这一次是加上了通讯作者。

刘书博老师： 我们调的方向与之有点相反。

受访者：以前只认第一作者，现在是把通讯作者也认了，就是只要求三篇高水平文章。

刘书博老师： 那民大认为什么是高水平文章？

受访者：至少你得是核心期刊C刊以上吧，至少得是你不能来个没有核心期刊。就是相当于我只要有三篇以上的这个C刊，我觉得你自己就有底气去找三个代表做评论。反正这个方式也是刚改，所以不知道以后会是什么样一种结果啊，不知道能不能真的达到这种说“去五唯”或者说中国学派的这种特色能够体现出来啊。

刘书博老师：有人经常会说我们不看论文，那难道看这个个人的主观判断吗？然后他们又会说那在中国会存在这种关系啊，或者是会存在这种这个所谓的相互勾结啊这样的。

受访者： 现在这个送外省，他好像是利用的像类似于咱们教育部那种，就是一个第三方，他不是说内部自己去评，也不是学院自己评，而是对应的双一流高校的那个层次。现在包括我们的博士论文的评审都是这样。就是说他会把文章发给对应的一些双流的这些高校里头的这些老师们去看。就是这个不是你院内去把握，也不是学校他们通过第三方去弄。当然你也不能完全排除里面有这种可以寻租的空间啊，但其实没有一种机制是真的可以排除的。但是至少我觉得他能在更大的一定程度上去实现你的这个评价的合理性，毕竟找三个专家你也是匿名的方式嘛。你提供的文章给人家，人家那边是匿名的，那人家那边也不知道评的是谁，就跟咱们评论文差不多，所以可能有点作用吧。那你要说完全可以杜绝一些事情，那也也很难说，毕竟包括期刊也是，他们也是有这种关系的。只不过我们现在把期刊的权力收回到学校里或者是学术共同体，现在反正也是这种方式吧。但是其实说白了这种关系的事情，其实这种关系也不是只有你中国独有，你要去看国外，中国可能隐晦一点，那国外的那些就是更明目张胆一些吧，就是人家可能就是这么个形式，对吧？

刘书博老师： 是的。

受访者： 所以我觉得只要是人与人之间（都存在关系），就可能就是一种方式。我觉得可能实际上变的是什么呢？就是咱们的期刊太少了，所以导致大家这么难，我觉得未来我们是不是有很大空间，将来努力去创办期刊。

刘书博老师： 好的。行，黄老师，我这边问题先问到这里，他们两位同学也有一些问题。

黄秋莉： 黄老师，那我先问。就是听了您和刘老师的交流，我就学到了很多嘛，然后我自己列了一些问题，然后可能跨度比较大。第一个的话就是您在咱们央财也工作过很长时间嘛。您在MBA中心工作的时候，您的这个主要工作内容是什么呢？因为我在网络上也看到了您的很多发言。

受访者： 主要做行政方面的工作，然后是做MBA的招生市场这一块。

黄秋莉： 您在这边工作了多长时间呀？

受访者： 六年吧。15年~21年。

黄秋莉： 那还比较长的。那您觉得在这个行政工作当中，您觉得有哪些内容是比较具有挑战性的吗？或者是比较有意思的东西吗？

受访者： 你说这个行政工作，是吧？

黄秋莉： 对。

受访者：其实那个项目是一个比较市场化的一个项目。然后其实在我去之前中财他也没有去做市场拓展的，你去查2015年之前，其实经常因为中财不接受调剂嘛，所以经常招不满，就是总会缺二三十个人招不满。那招不满你肯定报的基数还不够大，那时候中财MBA有点像那种就是我坐等着大家来报名，就是他们也没去做，网站也不做美化，也不做公众号，也不去宣讲，就是他没有那个市场运营的思维。那时候我估计当时的领导也是看中了我以前在学校的时候是做团委宣传部的这个东西，然后可能也是看中我这个能力吧，所以想让我去那边做这一块。所以当时对我来说其实相当于有点像从零开始去做这个市场的工作。其实那时候在中财是一个很大的平台。其实我挺感谢中财给我那么大一个平台吧，因为给我很大的施展拳脚的机会，我可以去做很多的事情，所以对我的锻炼也挺大的啊，因为平台更大，给的锻炼机会也很大。所以说在那个时候让我去做这种市场工作，而且给了很大一个空间和平台，所以说我觉得这是比较具有挑战性的工作啊，毕竟是从零开始。

黄秋莉：是，因为现在也能看到您之前的很多这种网上的记录，就说明您真的是做了非常多的工作嘛。那您在这个长期的工作当中，您对这个MBA的这种发展历史啊，就是我们学院的这种发展历史啊，有了解吗？就是从最开始到现在他有什么样的变化呢？

受访者： 咱们也是在不断地向好的方向迈进，其实你从学费的层次来看就能看到了。你像2004年才招第一届学生，到现在咱们最新的学费已经二十万八一个学生，所以我们叫做进入了第一梯队或者第二梯队吧。我们第一梯队可能是清华北大人大，三十多万的这种水平，那么至少进入20万以上的话，目前估计也就是这个外经贸、中财，对吧？反正你能进入到这个梯队里头，那至少说明咱们这个整个发展水平是不断在提高的，所以说你的社会认可是不断的在提高的。所以咱们这一块的这个项目还是做的特别好的。

黄秋莉： 那它的这个国际化的发展是一个什么样样子的啊？

受访者：其实中财目前国际化，我只能说目前属于起步水平吧，我觉得应该只能用起步水平。因为也就是刚做完了这种国际认证，其实按理说做完认证应该是你真正去融入这样一个国际过程嘛，对不对？包括之前像刘教授带的这个xxx项目，那也是这种国际项目的一种探索。所以说我觉得整个应该算是在国际化的这样一个起步到起步之后的一个阶段吧。

中财的国际化，要相比别的学校的话，就对比像这种北大清华人大，他们还有这一种双学位的MBA项目，就是你在我这还能拿国外的一共两个学历，是吧？包括说国外学生来读书的人，咱们以前还是很艰难的招，但目前咱也没有这种说国外学生来咱们这边读。但你要去看到像清华北大这些学校的MBA项目里头，它里头还是每年有一定这个比例的外国学生在这里读MBA项目。

黄秋莉： 就是这个MBA就是有名还是没名，我自己的理解是不是就跟这个学校的名字比较相关呀？

受访者： 那肯定有很大关系，毕竟你的学校是它的主体，对吧？那你说你脱离了你的主体你肯定办不好。所以说学校肯定是他的一个非常重要的基础，大家也是冲着这个学校去的。但是你只是说你学校好与坏是一个原因，但是你能不能做得好就是另一个方面了。那你要这个学校特别好，但是你没有把这个项目做好的话，那是学校好不一定意味着你项目都能做好；那你项目好肯定有很大原因，也是因为你学校好，就是这两个是这么一个关系。

黄秋莉： 明白，就是还是要靠比如说这个老师啊都安排的比较好一点。就感觉跟做一个项目也没有多大的区别。

受访者： 是的，因为你项目毕竟是运营的吧。但也不乏有一些学校，你看他学校可能稍微也不大，对吧？但是他也做的很有特色啊，所以学校好是一个很重要的因素，但是你这个项目你要运营的好，那么你就是争光添彩的。

黄秋莉： 明白明白，咱们这个MBA在咱们这个商学院当中这个创收的地位是不是还挺重要的？

受访者： 应该不止学院的，应该是在全校，我觉得在全校的创收地位应该是属于头位吧，应该是第一位的作用。

黄秋莉： 这么厉害。

受访者： 对对。所以说整个学校对MBA项目都很支持，因为学校发展需要资金，这种MBA是一个很大的收入来源。

黄秋莉：所以就有很多资源方面的倾斜到咱们这个项目当中去。

受访者： 对对。

黄秋莉： 那您在咱们学院工作了六年，是什么原因让您又回到了中央民族大学去呢？是一个什么样的契机？

受访者： 契机就是去年是博士毕业嘛，然后我们这边有这个招聘啊，所以说基于个人的事业发展的这个考虑，所以选择了跳槽。

黄秋莉：所以就是您是毕业就去这个教学科研岗？

受访者： 对，其实也正好得益于整这个民大比较包容吧，因为现在很多自己学校培养的毕业生都不愿意留，防止所谓的近亲繁殖，其实现在我看有人在提这是个伪命题。我在人事处了解到他们、包括校领导那个层面，他们就是已经在想，要打造这一种学术传承的话，那你就光看你自己学校里头有多少人。像我们民大老一辈，这些校长、校领导，还有这些院长，相对老一辈这些人全都是民大自己培养的学生。所以说他们这种对自己学校的感情和对学术的这种传承是不一样的，所以现在这边也正在调整这种思路。像我本硕博都是民大的，所以他们能够接纳我。而且我回来之后，像我回到学院里头也并没有大家会觉得你就是民大怎么怎么样，反而大家都觉得你是民大培养的学生，反而大家觉得说我们自己培养学生留在学校，感觉这些我以前的老师，现在我的同事，他们也都觉得他很光荣的样子。所以我觉得就是说他们这种氛围吧，就是领导可能有这种决策的魅力，就说我现在愿意去招收我自己留校的学生。所以去年不止我一个，还有别的一些专业都是留了自己本校留下来的学生，包括像好多学院之前就是一直就有，也不是最近这段时间，民大就一直还是有这个传统，就是我要留本校的学生。

黄秋莉： 对。我也是看了很多学校的公道嘛，他们就感觉这个从国外回来的这个学校牌子越响越好，然后在国内的就知道清华北大的。这样听起来咱们大学，我觉得挺人性化的那种感觉。

受访者： 可能每个学校的风格确实都不太一样吧，就是大家的这种理念方式，还是有些不一样的差异吧。

黄秋莉： 对，那您现在应该也是上课没多久嘛，那您这个感觉怎么样？

受访者： 还行，就是还挺不错，就是毕竟新老师所有的课都是从零开始，所以说这个备课的压力会很大。但是能跟同学们分享还是挺开心的一个过程。这是一个教学相长，也是一个双向奔赴，所以我们入职以来这些教授给我们组织的这种培训都是让我们成长吧。但是压力确实大啊，就是比行政压力大多了，以前行政可能下班了就下班了，那现在感觉当老师之后就没有存在下班这个时间了啊，

黄秋莉： 就工作和生活还能平衡吗？

受访者： 这个得靠自己，可能刚刚毕业的人都很难。因为你太多压力了，这个有教学的压力，你自己有科研的压力啊，也有做课题和发文章的压力，对吧？所以各种压力都挺多的，其实刚刚毕业的年轻人，像我们还有这种考核的压力，因为我们现在是预聘制，所以说我们都得完成学院跟我们签的一些合同，就是三年之后都得去进行考核。

黄秋莉： 反正就刚刚听您的分享嘛，我觉得听您上课肯定是一个非常享受的事情。那您刚刚提到这个预聘制的这个三年考核，他这个标准是什么样的？就之前听您说好像有各种各样的门槛，就是想请您给我详细的介绍一下。

受访者： 现在就是预聘制吧，反正学校不管，是你学院自己去跟你自己所聘的老师签合同。然后放在你的合同书里，然后像我们学院给我们签的就是完成像abc，然后还有一个国家级课题，这是我们跟学院签的。然后不同的学院都不一样的，因为有的学院可能是比较松，你只要完成两篇c以后，你只要做个课题就可以。就是每个学院不一样的，根据院长他们自己设定的这个目标，他跟你签不一样的这个合同。

黄秋莉： 咱们民族大学这个预聘制是第几年了呀？

受访者： 从18年开始的吧。

黄秋莉： 18年，感觉我们中财好像也差不多是从那个时候。是3+3吗？

受访者：不，就三年就一聘。

黄秋莉： 那咱们民族大学第一届已经结束了，他们之前留下来的人多吗？

受访者： 可能是有一位老师还没有完成任务，而且这个老师还是清华北大来的老师。反正你没完成，那就看学校的人数，学院跟这个老师现在还没有具体给出方案了。但是校领导是非常强调这个预聘制的这个威力的啊。

黄秋莉： 就说好走了你就得走。

受访者： 或者他不一定会非得让你马上走，可能会给你延期，给你机会。实际上博士都不那么年轻，那你这个也能延期，但是也不会给你无限延吧。所以说可能也会看学校最后的方式。

黄秋莉： 对，那老师您今年一共进来的预聘有几位呀？

受访者：就两位。

黄秋莉： 对，那我觉得还是挺好的。因为看市场上嘛，像中山大学啊，武大啊，他们这个就可能招的人会更多一点。

受访者： 那种属于差额，然后我们还是人性化点，我们就1:1。其实说白了，这个我们不存在跟别人竞争，就你自己只要完成任务就可以。然后他们那种就是你不仅得完成任务，你还得比别人完成的更好。我觉得那种有一点点有点病态了啊，这种不应该这么去做。那大家毕业都通过博士的这样一个培训，而且训练出来这个年纪了，你还搞这种淘汰制？我觉得那种有点不太正常，我估计就以后可能都得改这样的方式，不够和谐。

黄秋莉： 那据您了解，因为您可能有这个求职的经验或者是关注嘛。那您关注到咱们这个市场上，现在像中山、武大这样的做法多吗？就差额的这种很人性化的。就感觉把是博士生当成一个他们的生产论文的工具这个样子，这种情况不知道多不多？

受访者： 就可能越是这种，我觉得可能敢这么做的也就只有现在咱们经常说的排前10，20这样的学校吧？我估计只有这种学校他们才有这样的底气去做这种事情啊，可能大部分学校还是不会有的。大部分学校其实也就是说可能会给你预聘，但是就是要求你得完成任务，但是不会说我给你搞个差额。

黄秋莉： 对，明白。那您怎么评价这个三年的预聘期呢？您觉得合理还是合不合理呀？就是就您自己来看的话。

受访者： 要从学校管理的角度，他肯定是想要……其实我觉得也是一种督促吧，因为人嘛，人人都是会有这种懒惰的本质，其实他正好是一个激励。然后其实最关键的是相应的这种未来的奖励到位才可以。反正我觉得可以实行预聘，这也是一个学校管理发展的需要，但是我觉得考核任务呀不能太重，然后要有落地性。反正我们现在前面进来的好多老师都已经超额完成，你看我们有一个老师已经是一个A，然后两篇B、五篇C，还有个国家自科。然后另一个老师是发了好几篇A，然后加国家自科。

黄秋莉： 那您会有压力吗？

受访者： 你要跟他比你就内卷起来了，所以还好，我们就自己完成自己任务就行了。

黄秋莉： 对，就感觉这个标准还挺合理的。那因为您自己也是局中人嘛，您觉得预聘制度有哪些可以改进的地方就是会更合您的心意一点儿呢？就是从您自己的角度来说。

受访者： 因为我们现在是教育编制，我不知道别的学校是不是那样啊？中山大学和武汉大学他们那种差额的方式，他们应该是以年薪制的形式吧。然后其实我们这边的收入不是年薪制，我们就是属于编制，其实我们都已经是相当于老师们都已经进入编制内了，只不过是给你叫做预聘。所以我们拿的是编制内老师职工的工资，也没有说像年薪制那么高。然后就是这种时间可能会给人带来点压力吧。我觉得其实考核量还是得稍微降一降，就是不要太重。包括说三年得完成国家级课题，因为国家课题，就是有点有像国自课和国社科，就是有点偶然性的因素在里头的，所以我觉得标准就可以有多处，但是不要太高。

黄秋莉： 这个具体的细节我其实很难理解，就是说这个基金他们给钱嘛肯定是好的。但是也有的老师说嘛，就是这个基金呀，对他来说他没有什么必要性，就是有不同的观点存在。您是怎么样的看法呢？

受访者： 对我们来说，我们肯定很需要。因为我们评价标准，预聘的一个任务就是我们必须得去申。对我来说，我们是需要的。

黄秋莉： 好的，我感觉我已经问的差不多了。谢谢老师。

吕梦娜： 方老师你好。然后我再补充几个小问题哈。因为在官网上很难看到您就是一个比较完整的一个简历。然后我听莉莉问的您应该是15年~21年是在中财工作，那您与此同时也在民大读博吗？

受访者：对，对对。

吕梦娜： 那这两个时间线是重合的，是吗？

受访者： 重合的。我是18年开始读的博士。

其实在做工作的时候，我其实也是一直在跟着老师做课题了。就是因为大家报博士嘛，你肯定还是有一个数据的。

吕梦娜： 意思就是说你是硕士毕业之后，然后来中财MBA中心工作，然后后来又同时在民大读博，是这样吗？

受访者： 对，而且本来中财也有规定，行政老师想攻读上一级学位必须工作满三年才可以。

吕梦娜： 哦。了解，怪不得我在知网上能看到您的文章，我还在想一边那边做行政工作，然后一边做学术工作，感觉好厉害。

受访者： 中财也支持嘛，就是你这个行政老师工作满三年，你可以去攻读上一级学位。

吕梦娜： 了解了解。那您其实提到说写博士毕业论文的过程中，就类似于在汪洋大海当中去自己去探索嘛，然后也会经历一种孤独感。包括您看到师门也有可能有50%的一个流失率。那我就很好奇你是怎么去解决这种比较孤独的这种心态呀，或者是怎么去做自我调节？因为那个时候你还有行政工作在嘛。

受访者： 对。你看我虽然是18年才开始读，但是我其实我因为刚从研究生，因为我一直就跟着一个导师做。所以我从15年包括工作那三年其实我也没闲着，我一直跟着老师在做课题。所以说一个好导师很关键，我一直跟着他做课题，然后其实该有的一些，就是像咱们写论文过程中除了与理论对话，你还有这种经验的或者说叫一种证据的检验，对吧？

所以说相当于我在之前就把好多的一些问卷啊、数据啊，就把那一块都已经给积累了，完成了很多东西。所以到我真正开始读的时候，我主要专攻的一个方向就是去跟理论去做对话了，所以就相当于有了一个很好的一个基础。然后在做理论对话过程中，其实最核心的还是你得去读文献，你再通过文献来帮助你找到这条路。我们那会读博士的时候，所有的老师都在跟我们强调大家要去读书。然后这个书本里头去思考，去找到一些理论的东西。所以我们从那个时候开始，就是老师们上课一遍遍给你灌输你要做理论对话。你这个不是写一篇经验性的文章，你一定要去跟前人的理论对话。然后你跟前人理论对话过程中你要去追溯到这个理论根源在哪儿，然后你要去跟他对话，包括现有的这些文献大家做了什么样的一些研究。然后你再去提出你自己的一些观点，所以说这个理论对话其实真的非常需要读书，这个读书是很重要的一个内容吧。所以那时候我在我在写论文的过程中，你看我在财大我是老师可以借30本书嘛，然后我在民大博士生又可以借30本书。所以我光借书借了60本，那再加上我自己去买了好多的书，就是至少有一百多本书。

就相当于我的车的后备箱就是我的书库了，我全部堆在了我的后备箱里。然后你写论文之前一定要把书给读到位了，你才可能去做。就是说你得把理论要做到位才能去动手，要不然的话你一旦瞎写起来的话，整个就是只会写一些废纸。所以说理论对话特别重要啊，所以那个时候就一直在读书吧。所以我觉得说博士生读书是特别重要的一个环节。而且咱们现在大家有点倾向于天天去读一些文献，就是别人写的文章这种东西，就不是说不需要，但是你不能只读它。你只读它，你相当于你只完成咱们的文献综述，你只完成了研究的很小的一个环节。那算博士论文的1/10吧，那9/10的环节是跟理论的对话，就是你得去读原始的理论。所以说你得去读那种专著啊，而且是这些“大家”的写的书，我觉得这些挺关键的。

吕梦娜： 确实读书是很重要的嘛，因为你们对话的这个原因，那你当时面对一百多本书有没有一种心理压力，还是觉得挺积极？

受访者： 读书你不能瞎读，你瞎读瞎找你会越读越迷茫，其实很简单的方式就是你做什么方向，假如说我想研究这个方向的博士论文，那你首先肯定你导师得在行，对不对？所以咱做的论文一定是导师能指导的东西嘛。所以我从导师写的文章引用的文献里头开始着手，然后往里揪。另一方面就是比较知名的这些大咖里头，他们用的发表的文章，他的背后引用的文献是啥。所以我是从他们的引用文献里头去寻找，顺藤摸瓜的这样一个路径。

吕梦娜： 了解了解，那您也多次提到您导师对您的一个指引作用嘛，然后也让您感受到了学术这条道路的一个魅力。

 那我想知道就是您读完博，包括现在在做老师，这种学术的魅力还体现在哪些方面呢？

受访者： 你说是老师还是说什么？

吕梦娜： 就是做学术这个职业。

受访者：其实就让你能够回到初心了。你去做一些对这个社会有点价值的。所以说其实这些年我们一直在做的很多的课题都是一些横向课题，都是国家一些部委托我们去做的一些东西，然后都是给政府的决策咨询起到一些作用的。这时候你会发现究通过你手中写的文章去做研究，你可以去改变一些事情。因为他最终会变成政府的政策决策的形式。但写政策报告和搞研究还是有不一样的学术套路，但是这背后是需要你有这种学术的理论基底去支撑的。所以其实这也是咱们国家一直强调嘛，所以大家搞研究一定要服务于社会，服务于现实，不要自己在那，就是好像在自己的这个学术里好像做的特别精深。

所以现在很多的这些搞模型的一些学者，你们可以看到就只搞实证模型的这些现象。就是做没有对现实解释意义的实证模型的话，他可能可以很快评上教授，但是他的发展路径就很限制了，因为他不知道怎么去服务于这个社会了。因为我觉得我们作为一个学者，你不是为了自己而活，你不是为了说你发表几篇文章就结束了。你要是为了发几篇文章而去当个学者，我觉得这个就太没有价值了，那我觉得最重要的还是得服务社会。

所以说像咱们再去看以前的科斯呀以及马克思呀，还有亚当斯密这些，咱们这种真正的权威写的文章，他们的文笔全都是基于服务社会现实的。没有人去在自己构建的一个好像很完美的世界里去追求那些精致的、那么漂亮的一套工具，而是说我要把我的理论去服务现实、服务社会，我觉得这是督促你去保持学术兴趣的一个很重要的一个方向。

吕梦娜： 那您刚才也提到了，就是一般是通过这种课题的方式，然后给国家提一些建议政策，然后能够实现做学者的一种社会价值。

 那在写这种论文，特别是你也提到了这个主流期刊论文哈，这种写作能够实现这个目的吗？

受访者： 有些可以吧？因为有些期刊你发表了，毕竟你能在主流期刊发表能证明你的学术影响力吧。而且你在主流期刊发表文章跟你给政府做决策，这是两个完全不同的写法，那这是不能混的。咱们做学术的阳春白雪式的这种方式跟你给政府写的这种报告是完全是不一样的，这是两套不一样的写法，不能混在一起。

所以你在期刊上发表还是更多的达到学术共鸣、学术对话的这种，跟别人一起对话；然后你要是做这种决策报告了，可能是说你去跟社会接触。当然你在期刊上发表文章，你这篇文章能经得起历史检验，那肯定也有自己作用。你像科斯这篇文章的话之后无人问津啊，50年之后才被人发现它的价值，那我们能不能经得起这样的等待？就是看你每个学者有没有这样的一些追求了。

吕梦娜： 对，明白，就是说到主流期刊，您前面提到好像民大似乎如果发表一些关于民族相关的话题就很难被接收嘛，那民大有没有自己去做一些期刊？比如说就专为我们民族话题做一些期刊？

受访者： 有。首先每个学校都有自己的学报嘛，学报它反正是C里头比较好的一个学术期刊。然后民大自己又做了一个民族教育研究，然后这也是C类的期刊。然后今年又刚刚创办一个期刊叫《中华民族共同体研究》，就专门研究中华民族共同体的，然后这个期刊也是由这个国家民委来牵头做的，这种也正在发展嘛。所以现在让大家写作发表文章要回归本土之后，其实也是给本土期刊创造了一个发展空间。我觉得其实高层肯定也会有意识到这个问题，咱们这个期刊太少，然后僧少粥多怎么解决？包括财大这两年也是新增了很多期刊，所以这些期刊将来都会发挥作用的，就现在可能都没有名气，但是若干年之后他发展起来，那他就能够在这个社会上具有重要的作用。

吕梦娜： 没错，没错。那后面您提到您现在是预聘制嘛，然后您提到一句话是现在校领导非常强调这个预聘制的威力。那我想知道你这句话是什么意思呢？

受访者： 就是校领导会跟我们一直强调预聘制下，你要是完不成任务，那将来我们就要按照我们合同的这个方式来走啊，

吕梦娜： 就是对这个事情还是蛮严肃的，对吗？

受访者： 对，挺严肃的。包括我们现在已经实行全员聘任制了，就是所有的这个教授啊，还有这些副教授啊，全部都要实行考核，他们都要三年一考核，然后如果你三年没有做相应的事，没有任何期刊的话，那你教授就要进行降档。包括我们博士生导师，还有这种硕士生导师啊，今年我们已经取消了一批硕士生导师资格了。就说我不跟你说虚的，我跟你玩真的，就是我们今年像取消了一大批。就是他要求你三年考核期内。因为你说老导师不发文章，因为咱们很多老师可能做上教授、副教授他就躺平了。就是其实每个学校都有嘛，就是这种老师他就不搞学术了， 那我就可能忙于自己的事情。但是你忙于这个事情，对整个学校对学生都不太好，你不做学术不做课题。所以民大这边包括教授、副教授都是这种三年一考核。如果你没完成任务，那我再给你一个延缓，延缓后你还没有完成，如果你是四级教授，你就往下降成三级教授。

那你要是副教授，那你就直接降成讲师，所以我们是一个全员聘任。然后导师和博导、硕导资格也是，如果你一定期限没有完成博导和硕导的任务的话，那么我就取消你的资格。

吕梦娜：像很多学校都是新人新办法、老人老办法，那就全部这样，我能感觉到是所有老师都动起来了那种感觉，

受访者： 所以现在像那种好多本来都五十多岁的那些老教授，都开始纷纷发文章，或者就去发这种社论。就是是人嘛，还是有这种偷懒的倾向的，我将来可能也会有这样的倾向，对吧？人嘛，很容易理解。所以说学校基本上还是给三年一考核，然后你要不完成任务就不行，我觉得还是跟校领导有关吧，校领导可能是有这样一个魄力去干这件事情。

吕梦娜： 那后面的考核会稍微小一点吗？

受访者： 那肯定，可能不同的级别不一样考核。像对副教授来说一般是三年两个C吧，然后对教授来说又有一个相应的标准，就是都不一样的，但是也不会太高了。

吕梦娜： 怪不得您会说就是希望能够先生存后发展嘛。是因为前面三年压力会更大一些，然后后面的稍微会小一点，就可以做更多自己感兴趣的研究了， 是吗？

受访者：对，至少你职称也评上去了，你可以做更多些事情吧。

吕梦娜： 其实我对您刚才说的那句话，就是带着镣铐在舞台上起舞，我感觉特别形象。

受访者： 其实这句话是那个金培老师写的文章啊，是咱们社科院的学部委员，因为咱们社科界没有院士嘛，所以他们自己聘的学部委员。他都70岁了，然后他依然在做这方面的探究，反正是一个很了不起的老人家吧，包括他现在提这个域经济学。

其实你再去看像什么科斯呀或者亚当斯密呀，像科斯说他提的这个东西好像是不证自明的，很简单的一个理论。但是将来如果经历了这个历史的检验之后，你可能就能成为一个重要的影响力。所以他也是在做（这样的事情），因为他本身是从美国留学回来的，人家接受了西方经济学那一套训练回来的一个学者，然后他又重新结合中国去反思。你们感兴趣可以在网上搜一下他写的文章他，也不用那些很晦涩的语言，都是一些特别的甚至让你感觉有点幽默的语言，就是他会去研究一些现象。其实这就是学者要回归到你经济学管理学研究的这样一个初心吧，因为你本来就是要去解决经济社会现象。

吕梦娜： 了解了。您还提到申请自科、社科可能会存在一些偶然性因素，我想知道你所说的偶然性因素是指什么呢？

受访者： 因为每年申请的人很多，然后像我们社科的本子你只只有7000字的论证。本来这种学术的东西带主观性，所以说肯定有偶然因素在里头。

吕梦娜： 就是说这一批评委人可能会更倾向于的观点，可能另外一批人可能就不太倾向于你的观点，是这个意思吗？

受访者： 对，有时候会有这样的一些东西。

吕梦娜： 了解。那我还有最后一个小问题哈，就是因为您也是做老师，可能也就一年或者还不到的时间。然后您对未来的这个职业规划，无论作为老师也好，还是作为学者也好，你有什么想法？

受访者： 职业规划方面，就是像我自己说的，因为我之前做对口资源也是这种本土的一些东西，所以说希望将来还是能实现自己的初心吧，就做一个对社会有价值的人。然后能用手上的笔来改变这个社会，推动这个社会的发展，我觉得这是一名学者应有的担当，

然后因为我们高校老师的一个方面是你要去育人，对吧？你要去培养更多的学生，让他们走向一个好的道路，就是自己也能够去做出一些有价值的研究吧。我觉得这是明确的职业规划，因为老师也就是遵循学术这条道路去发展的嘛。所以说还是在这条路上，就希望自己将来能够做出一些有价值的东西。然后我觉得我并不一定非得要去什么主流期刊去发表或者怎么样，就反而能出版一些专著，然后可以在将来得到一些更多的人的认可，或者说能够对整个社会阐释能发挥一点作用。

吕梦娜： 了解，我感觉黄老师从一开始听到现在，就是很明显能感受到那种传统知识分子那种社会责任。

受访者：学术的力量是很强大的，学术之国的传统，虽然不对外彰显，但是各种政策制定都有学者参与，来自于学者研究成果。

吕梦娜： 了解，谢谢老师。

刘书博老师：谢谢。

# 受访者9

刘书博老师： 巫老师，我们因为是做质性研究嘛，可能得做一个录音，到时候会把文字稿发给您，如果您觉得有什么不妥的地方就可以删除这个相关内容。

受访者： 嗯，好的，你现在做的是商学院的什么来着，是文化还是什么

刘书博老师：比如说咱们中国商学院的管理啊，治理模式呀，包括一些我们对于学科标准的看法呀，我们怎么样去管理学术发展呀等等这些。我觉得中国商学院跟美国有时候会更像一点，那英国可能也有自己的模样。所以我就觉得这个多样性还挺有趣的，就为什么会有这样的多样性？还有就是我们这个管理学学科，它的发展一开始学苏联嘛，后来又学美国，但是现在学的又感觉有点缺乏自己的东西。所以我就想了解一下这个学科它整个历史发展的过程，大概做的是这个。

受访者：所以你是做OB的吗？

刘书博老师： 我其实是做strategy，IB领域的，但是我对这个教育管理挺感兴趣的，然后现在……

受访者： 这个很好哎。

刘书博老师：对对。就教育管理其实在英国有一个领域叫critical university studies。它就看英国的大学比较市场化，跟咱们还不一样，他们就搞得大学特别像公司，然后KPI啊什么的。就导致他们出现研究大学管理的批判理论。所以我就觉得我们似乎也要有一些自己的东西，然后看一下能不能用他们的那个理论视角看中国的现象

受访者： 哦哦，就是英国有这个领域的研究的，专门以大学为背景的。

刘书博老师： 对对，而且他们发的文章主要是在Human Relations还有Organization Studies。大概是这两本

受访者： 哦哦，我还很少读过这方面的文章

刘书博老师： 对，然后可能美国他们做的多的就是知识专业社会学或知识社会学或专业社会学这样的。

受访者： 那这个也算是在商科里面的吗？

刘书博老师： 其实商科在英国很多，美国它不是有一本Academy of Management Learning & Education期刊，那上面挺多的。

受访者： 对，我就感觉这种可能是专门对应一些education相关的杂志。

刘书博老师： 是的，那美国管理学会他们有专门一个Academy of Management Learning & Education。然后中欧之前有两位教授在那上面就发过一篇，他们那个研究是关于EMBA的中国教育模式，那很早之前了，那个是13年的时候。所以我觉得这一块儿可以关注一下。

受访者： 对，而且很有意义我觉得

刘书博老师：是是，因为咱们都在这个场域嘛，所以切身感受，以后有机会我们可以合作，再看看怎么探索一些视角呀或者一些新的文章。

那您（现在）怎么样？因为中欧之后咱们就没有再交流过，我看到您去到XX大学嘛，那是大概哪一年？

受访者： 19年吧，19年还是18年末。

刘书博老师： Ok，那非常好啊，因为我觉得XX大学的选人标准很高的

受访者： 就是我感觉……，反正是公立大学……，我觉得哎呀也有自己的问题

刘书博老师： 我记得巫老师老家就是XX的。

受访者： 对，我就是XX的，所以才去XX大学。

刘书博老师： 那挺好，那挺好，那我们就正式问一些问题，大概这个时间我们就控制在45分钟左右吧，我们尽量快一点问。

首先我来主问，就是想要了解一下，您当时为什么选择要读博士？当时是已经确定要选择走上学术道路吗？

受访者：其实我觉得有性别的原因在里面，就是在我们国家，一般就会觉得女生可能没有必要读个博士，但是我当时研究生之后毕业，我是在商业银行里面工作。但是我发现就是商业银行里面工作的技术含量特别低，就是和学的一点都没有关系。然后可能更多的是这种social skills吧，就是陪人喝酒吃饭这种。然后我自己是不太擅长这种东西的，所以我就在想还是想做一个比如说大学老师这样，那做大学老师嘛就肯定要读博士，所以就并不是说因为热爱学习才读博士

刘书博老师： ok，那当时对大学老师这个工作的内容也是有所了解了吧。

受访者： 嗯，对，但是不全面。就像一般人现在都觉得老师肯定是以教书为主的嘛。我可能觉得就是教学科研都有，但是就是没有想到我们现在其实是非常偏科研的嘛。

刘书博老师： 对对，ok，那您研究生就是在英国吗？后来博士也在？

受访者： 对对。

刘书博老师： 哦，所以当时这个专业也是自己兴趣导向？

受访者： 对，因为我研究生学的就是finance嘛，然后博士学的还是finance

刘书博老师： OK，那博士当时在英国这个感受怎么样？就比如说有没有一些出乎意料的，是不是当时想象的那样？或者说感觉有什么挑战？

受访者： 我总体而言觉得挺好的，我不是非常喜欢美国的那种style，就是我觉得英国的style比较放养型的嘛，你是纯兴趣驱动的，也就是说导师就只是给你提供一个support，不会像美国，可能有这个奖学金啊什么，就是会要做很多和学术无关的事情嘛。当然有一些是对你有好处，但我觉得英国他没有任何时候说是你一定要去做那些事情。那么就是说我觉得第一个我没有被压迫的感觉。

第二个就是还是以自我的incentive去驱动学习。然后我感觉很大的一个不同，就是欧洲相对是一个很宽松、而且很supporting和encourage的环境。就是我想很多时候美国培训出来的PhD可能质量比较高，但是很多时候我觉得他们好像就失去了一种自信或者爱好。因为他们的压力是很大的，很intense的。四年的学习学出来就是感觉好像大家都觉得，“哎呀，我再也不想经历这一番事情了”。所以我想我自己在英国读书的经历，就是欧洲人的这种理念嘛，就是兴趣驱动。而且很人文的，如果你说压力大啊什么的，他就会说那你就先不用学习了，你先把你的mental health给调整好。我想这一些是非常重要的，因为它是一个sustainable的发展

刘书博老师： 对对对，是的，就比较可持续嘛。其实我觉得这跟那个社会环境有密切关系。因为美国他就是麦当劳的发源地嘛，所以学术也是那样子。

受访者： 对对，其实我们国家更多时候和美国是很像的，就是求高速的发展，所以很多时候肯定是会有一些牺牲的，特别是在这个精神方面

刘书博老师： 对，那当时在英国求学的时候就奠定了您对于学术标准的看法嘛，或者说在那个时候有没有一个让您了解到学术应该怎么做、什么样是一个好的学者。当时有没有思考这些问题

受访者：你想说的是什么样的标准？是指什么呢

刘书博老师：就比如说我们会说这个人是一个优秀的学者，那他很专业。就是如果我们觉得他在这个领域很专业，那有什么判断标准吗？

受访者： 这个我觉得有的呀，英国的老师我认为很大的一个特点，我觉得他们相对而言不是那么急功近利，然后做东西是非常的稳扎稳打，就是也和他们考核的压力相对而言不是很大有关吧，就是你很少看到他们做事情就是为了出一些东西，而很快的去做一些东西，或者有一些这种机会主义或者很功利的说，要不先投一下试一下。他们做的东西我认为还是很认真啊，相对而言我觉得这会导致学术欺诈更少一些。我当时就觉得，就是我导师自己也快50岁了嘛，但是就是做东西非常非常的认真，比如说对于数据的保护意识啊，然后每一个co-author应该要做些什么，哪些人应该是co-author，哪些人应该只是acknowledgement，我想就是他们都是有一个自我约束在的，不需要外部的一些约束。比如说像我们国内只认第一作者，因为他认为如果不只认一作的话，大家就会搭便车，那我想这就是学术的ethics的问题。如果大家都搭便车那就容易产生这个问题，但是欧洲从来不会说只认一作，他们认为你只有有contribution才会写你的名字，没有contribution根本就不会写你（挂你的名字）。我想这些东西对我来说，做学术的ethics是很好的。

然后很多时候他们虽然也看重美国的期刊，但是如果发不上，我觉得他们也不会觉得有什么。有的时候要说文化自信，可是我们国家也只看美国的期刊，而且他们有自己European的一些期刊，他们会觉得发不上美国的期刊，但能发European的期刊也很好。这个时候就不会觉得很焦虑啊或者什么的，因为这个东西你不能只拿美国的期刊当唯一。我想这个也是他们比较可持续发展的一个原因，就是即使发表到美国最top的期刊，他们仍然愿意一直做。

刘书博老师： 嗯，是的，是的，我很赞同。就是他们会讲究学术的操守，然后又有自己的学科自信。还有吗？

受访者： 嗯，其他的我觉得就没有什么了吧

刘书博老师： 嗯嗯，Ok。那您在整个博士学习过程中，从博一到最后毕业，整个英国学术教育给您带来的价值，您是怎么样感知到的？

受访者： 你是指学术价值吗？

刘书博老师：也可以谈谈其他的，学术价值是主要的价值，那除此之外呢

受访者：首先对我而言这是一个谋生的手段吧，就是因为我的目标是想做一个大学老师，那首先最功利的目标就是让我如愿以偿去做我想做的职业。

然后是问我从博一到毕业有什么价值是吧？

刘书博老师： 对，比如说让您在某方面得以成长，除了知识方面还有别的吗？

受访者： 我感觉差不多就是知识方面的成长吧。其他的成长，因为你不管干什么事情，你这四五年应该都有成长，跟别的（职业）不一样的主要就是学术知识的成长。

刘书博老师： 明白，明白，那您在当时毕业没有选择留在英国而是直接回国了，那你回国之后找工作这个经历怎么样？因为我知道你是到中欧做国际研究员嘛，那当时中欧是第一选项，还是说还有其他的一些选择？

受访者：我就联系了一个中欧，然后他说可以，我就去了。

刘书博老师：当时没有想去，比如说国立的那些大学商学院。

受访者：没有，因为我可能也是在英国待的时间比较长嘛，我就在想中欧至少听起来比较国际化，就是稍微有个过渡吧，因为我在国外会听别人说公立大学bureaucratic的事情比较多，就是不知道可不可以习惯或者说和领导的这种为人处事啊这些东西。就是想就是先去一个稍微国际化的地方，然后就是慢慢适应的一个过程吧。

刘书博老师： 嗯，嗯，不知道您当时在中欧工作的这些年，对于这个商学院有什么有什么感受。然后是不是有一些没有想象到的、有一些意外的、或者是挑战性的。

受访者： 就是我第一份工作的感受是吧？

刘书博老师： 对对。

受访者： 还是跟想象的不一样的，就是欧洲的生活和学习的style和国内差别很大，回来的第一个感觉就是这边人的工作效率都非常的高，特别的高，自己就觉得很累，有一种相形见绌的感觉，就是别人都很勤奋。

刘书博老师： 您当时是跟XX教授、XX教授这些中欧的教授比嘛。

受访者： 嗯，对，是的，就是跟他们比。我是跟着那个XX老师做的（研究）嘛，就是学术方面差不多，因为我本来就是相当于做一个postdoctoral工作嘛，那去那儿确实也基本上做的就是一个postdoctoral的工作。然后他们有更多就是和industry接触的经验嘛，所以他有时候会和你说一些想法，还是比较受益匪浅的。

但主要就是觉得工作的速度和节奏吧，就是会突然变得很快。

刘书博老师： ok，那你觉得学术能力，比如说XX教授他们有什么值得您学习的，比如说对于工具方法的掌握还有什么嘛

受访者： 呃，主要就是我刚说的，他们和业界接触比较多（因为那些学生主要都是业界的），所以更多的insight是来源于他们对于业界的一些了解，但是technical的方面他们是不讲的。

刘书博老师： 明白。ok，那中欧之后当时您找工作是以XX为主，还是说在上海为主。就当时又换了工作嘛，所以这段有什么经历可以分享一下？

受访者：因为去了中欧之后会发现，可能作为年轻的老师来说，就并不是特别适合在那里长待，因为他们对teaching的要求是很高的嘛。那我的经历我觉得有限，我没有办法teaching和研究兼顾。所以我想还是要去一个体制内的大学，然后我自己是XX人嘛，那我就肯定首选XX大学了，所以就问了一下XX大学，然后就去了。

刘书博老师： 对。那XX商学院对比中欧有什么特点和不同吗？

受访者： 基本上没有什么相同的吧。中欧更像是个teaching school嘛，然后XX大学就是标准的这种体制内大学吧。就是它看FT50嘛，因为UTD的那个我们finance和accounting的杂志非常少，好像就三本，FT50好像是五本还是六本，稍微多一些。就是说考核就只看research，然后其他啥也不看，就是这样。

但是科研的任何方面都是很supportive的，它可以提供你任何想要的support，但是我觉得考核只看这个科研、而且就只要top是不是稍微单一了一些呢，我想这个有一些矛盾，现在不都“去五唯”论嘛，要破五唯，要把文章写在祖国大地上，但是考核还是只看外国的期刊，就是觉得有一些矛盾吧。

刘书博老师： 那是不是也是因为制度有一个惯性，他们之前定的，然后“破五唯”这个就中央倡导之后还没来得及改。

受访者： 也改了，现在不允许唯论文奖励了。我觉得制度上改的是很快的，但是实际上并没有改。就是现在也会把其他的写进来，但是实际上还是只看文章。

刘书博老师： 嗯，就比如评职称可能还主要看这个是吧？

受访者： 只看这个。

刘书博老师： 那你跟我们有所不同。我们现在评职称都开始强调教学，比如说评正教授的话必须得课评前30%。然后有教改项目这个也提倡，就是会参考。

但是（发论文这块儿）我们也改了，就之前通讯作者也认，现在就只认第一作者。

受访者： 现在只认第一作者哦，但是因为你们本身排序是按contribution排序的，对吧？

刘书博老师： 嗯，对，我们是这么排序的。你们金融好像不是这么排的，你们好像是按字母顺序

受访者： 对，我们是按字母顺序排的。像我就永远只能争取做通讯作者，就很尴尬，我是一个junior，然后整天要做通讯作者。

刘书博老师： 哦哦，我们就第一作者更高。然后奖励也还发，但不是那么明确的说，他就把它中间过滤了。

受访者： 哎，对对，我们也是这样变成了算公分。

刘书博老师： 对对。科研公分是这样，你发一篇文章多少分，然后再用分儿换钱

受访者： 换钱对对

刘书博老师： 天下乌鸦一般黑

受访者：我们评这个职称还有一个问题就是，不管你发的文章有多好，如果你没有别人好，你还是上不去，就是变得很卷。

刘书博老师：对对

受访者：你们是符合了这些条件就一定能评上吗？

刘书博老师：不是

受访者：像我们比如说副教授每年最多上一个，就是最多上一个，那我们一下子一年招五个，根本排不过来了。

刘书博老师： 哦，那你们是商学院一个吗？

受访者： 不是，我们是管理学院嘛，管理学院一般会有两个名额一年。那我们管理学院就还有三四个系，就是说还得系之间的平衡。比如说今年是你们系上，那你肯定不能上两个，你肯定是上一个，就是有个平衡

刘书博老师： 你们评副高是权力下放到学院了，对吧？

受访者：也不是的，也要到大学层面

刘书博老师： 我去，那你们这样挑战就有点大。因为综合性大学好多这个理工类的发文章好快的。

受访者： 嗯，对，是这样子，所以就是很那个的，就是商学院强调要FT50，然后到了学校他们只看SSCI，这就很那个了。

刘书博老师： 哎呀，那这个是有点，那我们像财经大学可能就没有，就相对来说。

我们副高就是在学院评，然后正高的话，其实大管理这一类也是不会被自然科学给卷的特别（厉害）发那么多文章。

刘书博老师： 现在我们其实发文章大户是会计专业，然后还有管理工程，他们建个模就好多篇文章出来。

受访者： 哦，是这样子。那你们像副高就是满足了那些条件就是可以上的，还是说也是像我们这样是要PK的。

刘书博老师： 我们要PK，我们是在学院PK，我们学院就是所有老师到场大概五六十个，然后投票嘛。想要申请的就依次演讲，然后大家现场投票，最后就按票数

受访者： 哇，你们这么民主啊。哎呀，那我们跟你们差远了，我们可能在你们20年前的制度

刘书博老师：你们是小民主制度吧？就主要领导决策对吧

受访者： 对，主要是领导。

刘书博老师： 哦，那可能也好也不好吧。

受访者：是的，我们就会有人走的，因为就是不公平嘛，然后人家就会走。

刘书博老师： 诶，反正现在我就觉得本来大学是比较自主的、清闲的，但现在感觉比公司还要忙，有这种感觉。

受访者： 对呀，007，

刘书博老师： 嗯嗯，好吧，好吧。那现在巫老师您应该是已经申到国家自科基金了，对吧？

受访者：嗯

刘书博老师：那没问题，我觉得在你们学院你又是本地人，还是非常有希望成为未来的学院领导。那你们行政管理主要是老同志做还是把这些交给年轻老师呀。

受访者： 行政管理工作的话，我们的秘书，比如说我们会计系秘书很多，我们会计系就有四个秘书。然后行政的事情副系主任会做一些，还有教授和副教授会做一些。像我们这种最底层的就不用做。

刘书博老师： 那现在你们进人是预聘制吗？

受访者： 对。现在都是没有编制的那种，什么专职科研。

刘书博老师：你是三年、六年这种考核机制吗？

受访者：对对。

刘书博老师：您当时去的时候也是tenure吗？

受访者：也是这个。

刘书博老师：哦，好吧，那压力还挺大。要求高吗？就比如说转成“长聘”这个要求高吗？

受访者：要求挺高的，要求一直都在变，然后你只能base on之前上去的人，也很不一致。就是可能比如说三个人同时PK，是那个发表最差的人上，所以你也不知道这个是个什么样的运行机制。然后一般的话，像我们accounting或者finance如果你有两个比如说UTD，那就比较有希望申到副教授。但是你三年谁能发两个UTD呢？其他的任何他都不是很能保证，因为他们一直强调平衡，就是学科间的平衡，很多时候给不给你上是要看其他系有多少个副教授

刘书博老师： ok，ok，明白。行，巫老师，那我就问到这里，还有一些问题让两个同学来问，谢谢。

吕梦娜： 巫老师你好。那我接着刘老师的问题再补充几个小问题哈。您之前是在中欧工作过大概三四年的时间吗？

受访者： 是一年半到两年。

吕梦娜： 然后在XX现在工作了有三四年的时间了对嘛。

受访者：对对，四年

吕梦娜：那这两个学校都有考核嘛，因为刚刚听到说XX是有考核的，那中欧呢？

受访者： 中欧也有考核

吕梦娜： 他们有什么差异吗？或者各自是怎么考核的？

受访者：差异就是，中欧那个职位是不续聘的，就是三年之后不续聘，所以他有考核没考核都没有区别，反正三年都不能再续聘的，就说中欧的考核相对会松一点吧，就只是说那是你的目标，你尽量完成；然后如果不完成，好像也没什么关系。

那XX的就是你一定要完成，不完成就会走人

刘书博老师： 那XX大学有因为没完成走掉的同事吗？

受访者：  基本上像我们会计系都没有完成，都走了。

刘书博老师： 那听起来走的人很多，那大多数都是去了哪里啊？

受访者：像管理学院基本上就是上财，还有之前去哈工大（深圳），然后还有南方科技的，就是那段时间那些深圳的一些学校给钱很多的嘛。

刘书博老师： 那感觉去向还都不错呀，因为上财又回到一线城市，还是不一样。

受访者： 对对，反正那段时间走了挺多人的，还有人就又回了美国，然后近年的还有一些，比如说他是山东人，他就去了山东大学。 因为它是三年的考核嘛，就是说本来三年就不出什么也是很正常的，假设我投UTD，本来这个就是这么长的时间，所以我觉得工作也是好找的嘛，因为你肯定有一些什么，只是没有出来而已。

刘书博老师： 那三年就要决定走还是不走？好短，我以为六年。

受访者： 对，虽然它说是六年，但是三年要考核，它的考核就是你一定要出文章。然后如果不出的话，一般学校会降薪嘛，对吧？你们应该是降薪嘛，我们是直接走。

刘书博老师： ok，ok，明白。

吕梦娜： 那您是已经通过了第一个考核期了，那后面会不会压力能够稍微小一点？

受访者： 没有，因为它只是通过，但是它不能升职称哦。

吕梦娜： 了解，那您刚刚提到说那个考核每年都在变，这是个什么意思？那您当时不是有签订合同，就是按照那个合同来考核您吗？

受访者： 但是他每一年就是会变的，然后之前是做了三年非升即走嘛，但是我们没有任何人到了三年是完成的，然后要不就是走，要不就是再签三年。然后中国的contract都很那个嘛，就是简单嘛，就是领导的可解释余地是比较大的。

吕梦娜： 最终解释权在领导那里。了解。那就是您在XX大学工作了四年的时间，然后您工作感受怎么样？如果能用三个词语来概括一下的话，你会选择哪三个词语呢？

受访者： 工作的感受啊，整体就是领导很support，压力是比较大的，同事之间的氛围是很好的。

吕梦娜： 所以其实听起来还不错。那您在做研究的这个过程中，您是怎么去定义或者去追求一个好的研究的？在您看来什么是一个好的研究？

受访者： 好的研究就是我喜欢的研究。

吕梦娜： 听起来比较主观，有什么比较客观点的标准吗？

受访者： 没有什么客观的标准。好的研究就是我觉得对社会有益，就是说能倡导一些我认为比较正向的一些观点吧。因为我觉得社科类的研究谈不上什么科学性，就是不是那种science。很多时候其实你已经是有了一个自己的想法，然后你再去做你的结论。所以我认为你自己是怎么样看待这个社会、理解这个世界其实非常重要。我认为可能好的一些研究更多的就是说要为这个社会的福祉去服务，而不是只是说为了……比如我们是做金融的嘛，就是以前都是说怎么样为公司赚钱，这个我们叫做股东利益最大化。那我认为你研究来研究去都是研究怎么样让公司赚钱，在我看来这就不是一个好的研究，我自己不喜欢这种研究，它没有让社会最大化的人去享受到这个福利。所以我就是会做一些企业社会责任这方面的研究，这个是我喜欢的研究。而且我认为会比较有意义嘛，就是说你去研究怎么样去赚钱，那这些就是他们公司会去研究，银行也会研究。我相对而言评价标准比较主观。

吕梦娜： 了解，我感觉您是一个很有社会责任感的学者。那您在成为这样的一个学者或者在追求这样的研究过程中，有什么比如说role model之类的吗？或者之前有什么人对n您触动比较大？还是说就自己探索觉得这是对的。

受访者： 我想model肯定是非常多，比如社会上面的舆论导向啊，潜移默化的，包括你的领导、你的同事其实都会有潜移默化的影响。没有一个特别的role model。但我想我周围的人、事物以及我们每天在媒体上面听到了他们倡导的东西，一定都会潜移默化的去引导你去认为什么是重要的东西吧。

吕梦娜： 好的，好的，谢谢您，然后我这边先问到这里。

黄秋莉： 巫老师你好，刚刚听您和刘老师、梦娜讲了很多，我觉得我听到了很多非常有洞察力的见解吧。然后我这边列了一些问题，可能跨度比较大。那第一个问题就是说因为当初您的经历比较丰富嘛，您在商业银行也工作过，那你当初可能觉得这个是更偏向于社会技巧的，就特别喜欢当老师那种有所创造的感觉。那您工作了一段时间了，长期以来您觉得您的学术工作是不是能达到您当初的这种要求呢？就是能够进行自由的探索呀，做自己想做的事情等等。

受访者： 总体而言还算是达到的，因为这个是取决于每个人的特长和喜好。我自己还是比较喜欢做科研，然后也比较喜欢教书的，所以整体而言是比较达到的。

但只是说现在学校的考核机制是不是过于短视了？因为三年就要让你一定出成果，要不就走人。我想这个导向有问题，就是可能强迫别人做一些机会主义的事情。当然这个也不是我能改变的事情，但是本身做大学老师这个事情我自己是非常喜欢的。

黄秋莉： 对呀，因为我看您还评上了优秀的教学团队，就很厉害。

 那为了应对这种非常短期的考核，要求又比较严，您现在的一个应对措施是什么样子的呢？

受访者： 应对措施就是躺平，没有应对措施。

黄秋莉： 因为我感觉你会对自己的学术研究有一定taste，就是品味嘛，你可能会对自己的研究有要求，那您现在这个躺平是什么意思，您能展开给我讲讲吗？

受访者： 就是说我尽量无视学校的这个规定和要求，学校制定的不管它是短视还是长视，不管它有它的一些原因和一些处境啊。但是我自己觉得我不能被他牵着鼻子走。我不能因为我达不到它的要求，就强制自己去改变，让自己做一些我觉得没有意思但是可以很快出东西的文章。我一直都告诉自己不要这样做，因为可能对我来说我并不是一定需要在某一个大学，就是换一个学校对我而言都是一样的。因为我们这种研究其实在哪个学校都是一样，反正都是自己做自己找co-author。所以我希望自己不因为外界对我的评价而改变我自己的这个步伐。

但是这个事情很难，就是当你身处其中的时候就发现很难，因为人总是个社会性动物，总是希望能够和比较，跟别人一样嘛。所以应对的措施就是告诉自己不要随大流。

黄秋莉：哇，我刚刚听了您的话，我特别佩服您，因为我觉得随波逐流就是很简单嘛，但是像您这样做到能够坚守自己的内心就是非常的难，可能会付出一定的代价。那似乎对您来说就是离开也就是无所谓哈，因为哪个地方工作对您来说都是一样的。我自己想着这种内心的挫败感应该是比较强烈的。

 受访者：对，就是很难的事情。

黄秋莉：我不知道，就是之前已经离开的老师是个什么样的状态？他们有一些什么样的表现吗？您能给我介绍一下吗？

受访者： 对。其实你后来看看，其实有的时候他们走掉的老师过得也挺好的，就是去另外一个学校，有些学校的考核会更合理一些。很多时候我想你离开一个地方可能就是危机嘛，就是有“危”，一定也有“机”。就是说这个事情要看你自己怎么看待这个问题。

你讲的那个挫折感其实是非常非常重的，但是我想一个人，其实这个工作，它只是你看待这个世界的一个方式。它不仅仅反映在我对工作上面，我对于其它各方面的要求，可能我都是这个样子。就是说我希望自己做一个从内到外比较统一的人吧，所以那些挫败感不可避免的会来，然后就会焦虑。但是这个时候就会更加触发自己去思考你人生的一个价值观是什么样的。我想这些东西它不仅仅会影响你的工作，包括你对感情啊、生活啊，其实都会有影响。

黄秋莉： 对我自己这种心情也挺有启发的，特别谢谢巫老师。然后因为您之前提到您刚回国的时候嘛，可能有个不适应，就觉得中欧的工作速度和节奏非常快哈，那您现在转到南京大学了，这种学术环境，就周围的老师是不是还是如此的勤奋呢？

受访者： 还是如此的勤奋。但是我觉得比中欧会好一些。第一个是上海整体而言，作为一线城市肯定是工作压力更大嘛，然后也没有什么归属感。那我自己去二线城市，而且又是我的家乡，相对而言这个归属感是很强的。然后虽然大家还是勤奋，但是我觉得这个学校年轻人更多嘛，就是说大家奋斗的这个目标比较一致，像中欧的话都是稍微年纪大一些的人，就是感觉好像不是特别有话聊。

黄秋莉：明白，那您觉得周围的老师发表的效率高不高呀？

受访者： 挺高的呀，特别是男老师发表的效率很高。

黄秋莉： 他们怎么这么厉害？

受访者： 就是男老师工作时间很长的啊。

黄秋莉： 听起来挺自我驱动的。

受访者：哎，我也不知道他们到底是自我驱动还是迫于职称的驱动。反正就看看他们升到了副教授之后，是不是还是像刘老师这样如此的勤奋。那像刘老师这样就是自我驱动型，有的可能只是压力驱动性吧。

刘书博老师： 我还好，我完全是兴趣导向。

受访者： 对啊，对啊，我觉得刘老师就是非常兴趣导向。

刘书博老师： 是的，是的。

黄秋莉：刚刚听到巫老师说您现在的工作是007嘛，这个工作和生活能够平衡吗？

受访者： 以前不太能平衡，但是我后来发现这个不能平衡根本也产生不了什么。因为做研究还是比较看你的洞见，你（工作）时间长了其实没有什么意义。很多时候你去看那些我们自己觉得文章写的特别好的人，虽然就很主观性，可是他们是靠硬熬夜熬出来的吗？也不是。其实他写的东西已经在他心里了，他只是需要时间把它呈现出来。如果很多东西它不在你心里，其实我认为看再多的paper也都没有用，因为就是好的idea，并不是说你看别人的paper你看的越多就越好。到了一定的量之后，你看再多也不会有什么提高了。所以我想就是得告诉自己吧，就是很多时候啊，work life balance吧。可能对于别人来说不是这样，但对我来说就是加大时长没有什么用处。

刘书博老师： 是这样。我非常赞同巫老师的观点，因为我觉得学术中的洞见是最为重要的吧。有人说是天赋，那天赋表现出来就是一种洞见嘛，就是那个idea。但是我们现在被内卷到好像不得不把洞见转换成很多那种数量型的文章，所以我就觉得这事儿就是变质了。就做连连看。

黄秋莉： 巫老师，不知道您了不了解咱们商学院这个AACSB认证呀？

受访者： 不是特别了解，但是我知道这个。不过我们好像从来不讲这个认证。

黄秋莉： 就是您对这个感知不是很明显，是吗？

受访者： 你讲的是那个教育部评的吗？

黄秋莉： 是美国的那个认证。

受访者： 我们根本不看，领导不关心的啊。

黄秋莉： 那我们学校这个比较强一点

刘书博老师： 那AACSB在工作中没有体现吗？就比如说我们要填好多表呀，比如课程方面的。

受访者： 那你们这个评上了，你们学校认证的这个对你们学校的好处是什么呢？

刘书博老师： 就可以宣传了嘛，就它有点像一个符号价值。

受访者： 是的，但是宣传哦……

黄秋莉： 可以去国外交流。

受访者： 那因为在北京可能你们竞争比较激烈，我们XX大学在XX是没有学校去跟它竞争的。然后你要跟上海竞争吧，你也根本竞争不过它。就是你再多的努力你也竞争不过上海的大学，然后你在当地你根本不需要竞争，所以这些我们都不关心。

黄秋莉： 主要是学生去国外访学交流啊，国外的学校会比较认可。

刘书博老师： 但是你们也认证过了，也是AACSB认证的商学院，对吧？

受访者： 我不是很清楚，因为我们从来没写过，但应该是的吧。

黄秋莉： 是认证过了，我看官网上有。

刘书博老师： 那其实这个认证不要求咱们任课科研老师做什么，完全行政老师给做了吗？

受访者： 我们行政老师做的事情挺多的。

黄秋莉：我回到最开始啊，因为听到您和刘老师在讲的时候，刘老师说我们管理学科一开始学苏联，后来学美国，会比较缺乏自己的东西。然后我觉得您当时的语气是特别认同哈，然后您后来也讲到这个学科自信嘛，就是您能不能再给我仔细的讲讲，我觉得您的想法都特别的有这个洞察力啊。

受访者： 就是说学科的建设，是吧？

黄秋莉： 类似于这种学科自信吧，就是学科缺少自己的东西。

受访者： 我想就第一个是要多研究中国问题。我自己是觉得，虽然我是海归啊，然后对于中国的国情啊文化之类都不是很懂。但是我觉得我们应该还是首先倡导更多的研究中国问题。因为我发现accounting领域研究中国问题的比较少。因为研究中国领域发不到top期刊，就是每一个国家的杂志一定有自己的意识形态。就是我们研究中国问题他们根本不怎么接受，而且他们听不懂。所以就是为了迎合他们的期刊，我们都研究美国的问题，这个我觉得就很成问题啊。你不研究中国的问题，然后你又不在美国的国土上去研究美国的问题，这个就是纯为了发文章而再去做研究。

第二个，我觉得相对而言还是要有一些……虽然学术应该倡导自由，就是我们想做什么就做什么。但是我觉得每一个学校或者说我们每一个系，它要有自己最强的一个领域。也就是说我们现在招人的时候，也不管他到底做什么领域的，就是说院系要招人的时候想要做什么方向，比如说有中国特色的公司治理，那就要多招这方面的人，稍微有一点点像命题作文那样子，就是说更多的老师去研究这个非常重要的问题。像我们现在就是你做这个、我做那个，完全不成体系。因为很多研究就是自己收集数据，但有一些非常有意义的研究，你是承担不起这个代价的，你不可能去收集到那些数据。但是如果我们是一个系主攻这个，那我们可以花很大的时间去研究，去搜集这个数据，然后去研究这个最重要的问题。

这个我认为也是可以思考的一个方向，现在的话就过分的散了，每一个老师都在研究自己的问题，然后其实也不是什么重要的问题，就是好发文章的，就是非常非常的散。就像一个个砖头，这边丢一块砖头，那边丢一块砖头。我希望还是能尽力的去把砖头往上垒，然后能垒成一个建筑，不要最后垒完就是一地散的砖头。

黄秋莉： 我以前可能就是看到了一些问题，但是不知道有什么样更好的方法。那听您这么一说，我觉得豁然开朗的一种感觉。刚刚也提到公立大学的问题嘛，因为您就有这种在英国和在中欧都工作过或者学习过，有这种对比的视角，您可不可以跟我讲一讲就是您体悟到的、您看到的这种公立大学的问题呀？

受访者： 公立大学的问题就是官僚式的那一些共通的综合型的大学都存在的问题。刚才刘老师也讲到了，我们和所有的理科、工科、文科一起评，这个就主要看校长是哪个学科的，还有学校领导是哪个学科的，可能那个学科就评的公平公正一些，其他学科他也不了解，就是综合型大学就很难平衡，这是个问题。比如说我们现在的话那种information system招的人比较多，然后他们出文章很快，那就会有个问题，就是这个学科容易不平衡。那他们出的快，他们就升的快，然后招的人就多，可能其他出的慢的学科就会萎缩，然后包括资源都向他们那边倾斜。国外的话是accounting的就accounting的去评嘛，我不可能和information system的人去PK一个职位。然后只看这个数量，国外的话就是A大学的accounting和B大学的accounting横向比的，我们现在是A大学的accounting系和这个A大学的information system系这样子去PK。可能像财经大学会好很多，像我们这种综合性的大学就是这种问题。

刘书博老师： 其实有一个补充问题，就是就非专业的老师在管理专业的老师，就比如说行政老师拍脑袋定一些东西就会出现这种问题。其实我觉得我们的学术共同体啊，就是专业性没有变成一种自制，还是这种行政在管理。我觉得这个是导致所有问题的一个根结吧。

受访者： 对，这是一个问题。还有一个问题就是我们是一个relational base的一个社会。很多时候你觉得不合理是因为合理的方法可能动了一些人的奶酪。

刘书博老师： 对，就前两天那个政法大学刘教授写了一篇断交声明，哎呀，就把潜规则完全暴露出来了。这就是relational base的一个特点。

黄秋莉： 就是刚刚巫老师讲的这个，其实也想跟您共享一下我们访谈得到的信息，像中国政法大学的老师，他们说他们在评职称的时候有一个要求，就是要独立著作的一个书籍。他说这也是因为他们是这种法学的老师治校嘛。可能也跟这个比较有关系。

受访者： 对，是的。

黄秋莉： 然后我再问最后一个问题，因为您可能会站在劳工的这一方，就是为我们这种普通人发声的这种感觉哈。那您在一开始的时候也提到，就是这个研究商学院的这个场域，您觉得会特别的有意义。我就是想请问一下，您是在这个商学院场域中看到有哪些问题需要改变吗？可能您刚刚提到比较多的就是这个可能考核体系比较短视啊什么的，就想请问您有别的补充吗？

受访者： 就是商学院的这个管理有什么需要改进的，是吗？

黄秋莉：就是商学院组织啊或者是管理学呀这种都可以。

受访者： 我感觉第一个，我们学校的政策有一些朝令夕改，没有连续性。所以大家都不知道考核的标准到底是什么，然后经常是临时一个月说今年是这样子评副教授的。这个东西就让人非常的……就是这么重要的事情提前一个月就变了。当然他们是说在这个改革阶段啊，可是我想这个改革阶段也不能朝令夕改吧，就是政策要有个连续性，主要就是这个问题。

黄秋莉： 我今天也是看到一篇推文，就是那个社会学家项飙老师他写的就是我们中国可能有个问题，就是虽然是发展，我们不断的改革，但是好像是东一头西一头，就也没有说是有所进步。

受访者： 我想从管理学的角度来说，比如说像我们商学院的院长可能就是当四年，或者当个两届就是八年，那你想他只当四年，就是他怎么样能制定出来一个长久的计划呢？那就是这个我觉得也是有问题的吧。所以很多时候他就希望他招的人立刻在三年内能发文章嘛。因为他只做了四年，那他之后要考虑自己的这个业绩政绩的问题。

黄秋莉： 在英国的那边的这种院长呀，他们的这种时间会长一点吗？

受访者： 他们相对而言自己的ethics是比较高的。因为那个时候我是学生，我不是很确定。但是比如说像系主任啊，这些东西其实没有什么人愿意做的，因为他们这个制度的话没有什么特别多的权力和油水。像这种系主任行政事务比较多啊，也没有什么人愿意做，大家都想要去做科研。所以他们是自下而上的，就是说大家都想要去做好学术，然后都觉得不应该短视嘛，他们觉得只有穷的学校才会去做很短视的事情。

如果说资金充裕的学校，我愿意花十年甚至二十年去培养一个我认为有发展潜力的老师。他们就是这样子，所以不存在这个问题。但是我们这里更看重的是效率嘛，就是出成绩嘛。

黄秋莉： 明白了。就是您刚刚提到这个ethics嘛，然后刘老师也经常给我们介绍说他觉得英国就非常注重dignity的问题。

受访者，我没有问题了，谢谢您。

刘书博老师： 好的，好的，没有问题了，谢谢巫老师。那我们也超时了，今天晚上向您学了很多，那回头我们希望还是有更多机会继续向巫老师学习。太感谢了。

受访者： 你们结果到时候出来记得发给我看一看。

刘书博老师： 没问题，我们共同探索。我回头把一些我最近看到的、您可能感兴趣的文章也发过去，您可以看一下。

受访者： 非常感谢。

刘书博老师： 好的，我们以后多交流，感觉价值观是比较一致的。

好的，行，巫老师，那就不再耽误您时间了，那我们今天晚上就到这了，谢谢巫老师。

# 受访者10

刘书博老师： 嵇老师，我们这个研究主要关注的是商学院和管理学专业，就是抽象的是管理学专业，具体的是商学院，是这样子。然后在Human Relations这个期刊，在欧洲尤其是北欧，有一个Critical Management Studies，还有一个更细分的就是Critical University Studies，他们可能研究比较多的是新自由主义对于大学组织的影响。那这块儿我看国内好像还没有对于人事制度改革、对于现在这种市场化呀公司化呀、对于传统的科教文卫等的批判的。所以我们可能会关注类似这些方面的问题。那今天很高兴很荣幸能够听一听嵇老师对这方面的看法，那我们还有一位博士是黄秋莉，梦娜应该之前跟嵇老师有过交流了，秋莉要不先介绍一下自己？

黄秋莉：好的，嵇老师您好，我叫黄秋莉。然后我也是跟着刘老师做这个关于商学院和管理学的研究嘛。那我选择的一个细分领域可能是跟身份规制和职业自主性相关的话题。今天也是非常感谢您能给我们介绍您的一些看法。

受访者： 没有，没有，我觉得你们有勇气做这种定性研究还是很棒的，对你们博士来说，我们学院大部分都做定量的，其实我觉得博士做做质性研究挺好的

刘书博老师： 是的，我还是鼓励他们要做一做，坚持做下去。

受访者： 对，那主要是因为很难嘛，要不是你带着他们做可能也做不了

刘书博老师： 我们一块儿摸索吧。因为我觉得咱们其实有很多新的现象，可以把它给先表达出来，然后再去搞一些理论出来。行。那嵇老师我们就开始。

受访者： 嗯，好

刘书博老师： 好的，谢谢。那还是我来先问一些问题，我可能主要问的问题是围绕嵇老师在之前的学习经历，比如说当时您是在XX大学读研究生阶段就已经确定、或者在更早的本科学习阶段就确定要去追求学术事业吗？就是要去读博士吗？还是说那个时候还有其他的想法？我想看一下您作为大学老师，这个心路历程是什么？

受访者： 其实我本科阶段也没有想好，我是一个想法还比较多的人，然后也喜欢尝试。所以我本科既试过做学术，我当时本科的时候就跟着北大光华的一个老师做学术，还有包括跟着我们学校的XXX老师，当时他还在劳人院，主要是跟着他们两个做学术。

另外也尝试着去实习。既试了国企，也试了私企。当时国企是去的XX实习，然后私企是去的XX实习。实习完之后我就发现这两个都不太适合我，我发现企业可能不太适合我吧，尤其是国企。因为我当时在国企实习的时候，是我导师推荐我过去的，就把我推荐给了某个分中心的主任那里。但是呢后来干着干着活，我们中心的大主任缺一个懂英语的人，所以他就经常叫我去那儿干活，然后干着干着我当时没有觉得任何不对。但是后来有一天我导师突然跟我说在国企工作主要靠“悟”呀，然后我就问他是什么意思？他说他把我推荐去的那个分中心领导跟那个大领导其实有非常大的矛盾，我本来应该是给那个分中心的主任干活，结果跑去了给主任干活。他就说那个分中心的主任非常的不满，然后我就觉得很无语，再加上这是一个非常老派的国企，然后里面的人全部都是博士，北师大、清华、北大的博士都有，就是非常的谄媚，就一起去唱KTV。我记得特别清楚，有一个师兄对着那个主任唱《父亲》，我真的是···。再加上当时那个主任有的时候也会点我，然后就会说，“哎呀，你们90后真的不会伺候人呀”，然后说“我之前工作的时候，就会每天提前半个小时来给领导打扫卫生、打扫办公室、然后给他倒茶什么的”。这就坚定了我不想去企业的一个决心，就国企是pass掉了。

然后去私企，这是非常典型的“你只是螺丝钉”，因为他们分工非常的明确，我当时负责招聘，而且只负责某一个部门的招聘，就是我们那个团队帮他们招JAVA工程师。每天干的事情非常的重复。我就觉得在这样的单位工作也没有什么意思，所以我就彻底歇了想去企业的心。

然后就开始觉得我应该是比较适合学术。因为我说实话，我是比较偏内向的人，我觉得我在企业里面不是很适合，所以我最终决定了做学术。所以我觉得我选择学术这条路吧，或者说当老师这条路，最最最主要的原因，还是因为我觉得我适合，并不是说我有多么热爱什么的。当然另外一方面是因为我是比较喜欢当老师了，我比较喜欢跟学生沟通交流。

刘书博老师： 嗯，嗯，ok，ok，有时候我们战略领域有一个经典的说法，就是“什么是战略？要想想明白，就是你不是要想明白去做什么，可能更重要的是想明白不要做什么（自己不适合做）”。我之前跟您也差不多，就是在企业有过一些类似的这种经历，就觉得真的受不了。后来我决定了还是要进一步读博士然后做研究。

所以您之前做研究的过程怎么样啊？这个比如说您提到跟XXX老师，然后这整个过程就是我们在博士阶段受到的培训或者是教育啊，这个过程怎么样？有没有一些觉得比较意外的啊，或者也就是您想象的那样子。

受访者： 因为我不是在商院嘛，我本硕博都在劳人院，然后劳人院说实话不是非常的注重学术。但是我觉得我非常幸运的遇到了很好的指引我的老师。就我本硕阶段的XX老师，还有北大的XX老师，然后后来到博士阶段我就遇到了很好的合作者。其实主要是有这些合作者，包括带着我一起做科研的老师，我觉得他们是带着我真正走上学术道路。所以我说实话受的这个教育不是很规范。比如说在博士课程的各种方法呀，我觉得这方面我可能会弱一些。但是有这些人他们带着我教会了我怎么做学术，我觉得这是很重要的。包括他们教我怎么讨论选题，我们经常会头脑风暴啊，然后教我如何看文献、整理文献，然后教我如何去写一篇文章，我觉得这是很重要的。至于具体的学习技术方面，说实话我都是自学为主，因为现在也很发达嘛，各种资料都有，就是各种分析我还是以自学为主。

刘书博老师： 嗯，ok，那其实跟我了解到的北大光华就很不一样，他们就是非常注重这种规范式的技术式的学习，包括咱们现在中财

受访者： 对是的，但因为我不在北大嘛，我是在劳人院，而且我还是在劳关系。所以我其实并没有太多的那种技术方面的课程，这个是很少的。

刘书博老师： 那当时劳人院他们对于博士的培养特色是···，我听起来是比较导师引导或者是个性化比较凸显，不是像美国商学院的那种标准化的，对吧？劳人院听起来还是不同的学院。

受访者： 对，可能人管系标准化强一点，但我在劳关系嘛。劳关本来就是一个交叉学科，像我的师兄师姐有法律背景的，有经济学背景的，有管理学背景的，有社会学背景的，然后老师根本指导不了啊。所以我们全部都是靠自己。

刘书博老师： 哦，那这样我听起来劳人院会很有趣，因为大家的理论背景和思考问题的视角是比较多样性的、多元化的。

受访者： 对。

刘书博老师： 那当时您在选择专业的时候，就本科就开始到劳人院嘛，然后在那个时候为什么会选择这个专业呢？

受访者： 因为我记得特别清楚，我一进大学的时候，我们书记就跟我们说，劳人院三个专业代表三个不同的立场。人管专业代表的是企业立场，社保专业代表的是国家立场，劳关专业代表的是劳动者立场。然后我就是可能稍微有点“愤青”吧，我就是特别想要为劳动者做一点什么，然后其中本科阶段也参加了很多农民工的NGO啊，就是劳工组织啊之类的。

刘书博老师： 那这个我觉得特别好，因为我现在其实比较关注的就是批判理论嘛，我们商学院（尤其美式的商学院）可能更多站在管理者或者资本家的立场。这个批判理论就站在劳动人民或者是打工者的立场。那现在我看好像国内管理学似乎站在被管理者、打工者的这种研究不是特别多，更多还是说要提升管理的效率啊，然后让人更好的被管理，是这样的一个视角。但是您说您的背景应该是站在劳动者这个视角，所以您现在的研究也是在这个视角吗？

受访者： 两者都有吧，说实话，现在我有点偏向人管。我之所以有点偏向人管是因为非常现实的原因，就是像您说的，您很少看到这样的研究，是因为这样的研究很不好发。因为劳关没有像人管这样的体系，没有像人管这样标准的研究范式，它还是以理论研究为主，所以他很难发文章。而且在国外一直是一个没落的阶段，就美国labor relations这个专业也叫industrial relations，其实在上个世纪还是很火的，在五六十年代，在商学院的地位甚至是超过人管的。但是后来主要是因为工会密度的一个下降吧。因为站在劳动者的角度，工会是一个很重要的研究对象。因为我们都讲要把工人联合起来才能够和管理者对抗嘛，不然一个人的力量是不够的。所以这个学科就没落了。

而且也是因为这种工人的基础已经很薄弱了，因为说实话产业工人越来越少，现在越来越讲这种知识工人，然后他们也不需要一个工会来代表他们的利益。所以industrial relations这个专业在美国的学校里面被砍的越来越多，现在好像最好的只有康奈尔有了。

那你想他的学者少了，在商学院的地位下降了，那在这个学术研究就会越来越小众。所以我就发现如果我继续做劳关的研究的话，我在北京找工作只能去两个学校，一个是首经贸，还有一个是中国劳动关系学院。只有他们两个有劳动关系专业。所以我就转向了一点人管。但是我现在也还在做劳关，因为我毕竟学了九年的劳关，我也一直对这个领域很感兴趣，但是的确这个领域的文章太难发了。

刘书博老师： 嗯，对。但是咱们现在看到就是比如说这个零工经济、平台经济、平台企业。他们其实会有一些社会关注的这种对于员工的新形式的剥削。就像前一段有一个报道式的文章写的，就是困在系统里的快递小哥，好像社会其实还挺关注的，那我不知道你们那个领域有没有就对应···比如说社会学它会有系统的研究，这这样的一些新的现象呢？

受访者： 有。有。其实，我们劳关研究最多的···像零工经济出来之后，像这些小哥还有包括之前闹得很火的滴滴司机，他们跟所在公司之间究竟是不是劳动关系？如果他们不属于劳动关系，就不属于我们研究的范畴。你说滴滴司机跟滴滴公司是劳动关系吗？

刘书博老师： 滴滴公司会说他们是合伙人，他们是生意伙伴，类似这样大家一块儿创业吧，京东刘强东说都是兄弟嘛，是这样的。

受访者： 哈哈是呀，他们肯定否认是劳动关系，这样他们就不用负责。

刘书博老师： 对对。我不知道您有没有看过就去年吧有一个英国新拍的电影，就讲的亚马逊雇的快递员，然后就是像就有点像滴滴的那种司机，没有那种对于员工的关怀，更多是签一个非常松散的合同。然后呢那个电影就演的是一个非常悲惨的、做了快递员之后的一个中年男人，然后和他家庭的故事。我回头可以发您看一下。

我觉得其实这样的事情在中国好像也同样发生了，但是我们这个研究领域，尤其是我们看管理学研究，好像更多是站在公司的那一面。所以这一块儿我觉得好像没有太多研究。

受访者： 对，而且非常的敏感。像我当时记得特别清楚，我们本科做大创的时候，我们做的是一个农民工的NGO，然后当时找指导老师都找不到，他们都说你们这个太敏感了。像我们系有两个老师，那是长期被国安局给监控的，因为一遇到大罢工，全部都是他们去调停的。其实因为劳关比较敏感，它不像人管比较微观，它一旦上升到宏观的层面，就可能影响社会的稳定，所以它比较敏感。有的时候你写的一些东西吧，国内也不一定能发的了。

刘书博老师： 对对对。但是其实有时候就国内的这些现象，国外好多期刊也是欢迎的。

受访者： 对，所以就大家发国外嘛。比如说很多人研究工会，就是说中国的工会其实并不完全代表劳动者的利益嘛。

刘书博老师： 对，最近我看Human Relations那个期刊就发了一篇，他那个作者应该是来自于中国台湾地区的一个学者，他其实出了一本书，那个回头我也发您看一下，他其实讲的就是这个，就是中国的工会到底是扮演的什么角色，还挺有意思的。

受访者： 对，但是这样的说法在国内就很难发嘛。

刘书博老师： 对。那现在其实我们发外文期刊也可以没问题，对吧？因为好像在管理学领域我们还是外文期刊为主，他的重视程度还是比较高。

受访者： 嗯嗯，就是这个领域可能顶刊还是很难发吧，我是很少看到

刘书博老师： 您最近不是发了那个JAP嘛，非常非常恭喜嵇老师，这个也是杰出的成绩了。那这个文章发表也比较具有挑战性吧，这个过程怎么样呢？能不能简单聊一下这篇在整个发表的过程啊？

受访者： 啊，这个其实是我们众多尝试当中唯一成功的一篇了，就是我们其实当时想的很简单，就是我们不能够写大家写过的那些常见的概念变量，因为我们肯定是达不到那样的水平的，我们就想试一试写一些新的东西。然后我们就发明了一个新的词叫cyber leisure，leisure是大家研究烂了的一个东西嘛，但是因为现在电子技术的发展，所以我们就想说大家leisure出现了一种新的形式叫cyber。然后我们就是研究了这个新的leisure形式对员工工作的一个影响。然后其实过程总体而言还比较顺利，比我现在在R&R的JAP要顺利多了。

我现在有二轮被拒的，三轮被拒的，中间让我收两次数据都被拒的，这一篇已经算比较顺利的，中间没有让补收过数据。但是就是理解错了editor的意思吧，就是我们第一次拿到R&R的时候，就说我们中间一个关键的中介机制，他觉得有点问题，问我们为什么不考虑另一个变量？然后我们看他说的语气非常的软，我们以为他真的只是问一下我们，所以我们就解释了一下为什么没选另一个变量。结果到第二轮的时候，他就说我并不是在recommend你，我是要求你必须要考虑这个变量，而且他把我们的那个文章变成了high risk，他就说如果我们不能解决这个问题，他只能reject我们。所以我们最后就改了模型，其实本来不想改模型的，最后只能改了模型，还好，改了模型之后他就接受了。

我觉得最主要的一个原因也是因为他对我们提出的这个新概念很感兴趣，导致我们中间有很多地方他觉得有问题，比如说测量有问题，方法有问题，他都忍受了，他就觉得只是第一篇，那么只是跟大家介绍一下有这样的一个概念，那么之后可以再做深入的研究。

刘书博老师： ok。那听起来也是因为他感兴趣，所以在某一些标准方面，他不是按照研究比较多的领域的那样的研究去控制，所以可能在定义一个研究的题目的时候还是挺重要的。

ok，那这篇研究你发表之后的感受怎么样？当然除了刚才讲的那一条经验，还有别的一些经验吗？因为还有两位博士他们想要再学习一下，就在写作啊或者说跟顶刊的编辑，我们在投稿的时候是否也可以有一些主动的选择权啊这些

受访者：我觉得一方面是写作方面吧，说实话我们自己写还是挺难的。比如说像我们第一轮，说实话，只是我跟一个合作者，我们两个中国人投过去了，当时他就说我们的language不太行。然后到第二轮的时候，我们改完之后，他说我们的language变得更差了。所以到第二轮的时候我们又加了一个外国人进来，然后他就是并不需要做任何事情，只是帮我们改language，然后改完了之后，第三轮的时候editor非常的满意。所以我觉得我们管理学还是要讲究讲故事嘛，我觉得这个language还是非常非常重要的，所以如果能找一个native speaker能够加入进来还是有很大帮助的。

刘书博老师： 那这个作者是之前就认识的吧

受访者： 之前就认识，也算这个领域的吧。

然后另外一点就是reviewer的任何意见都要加倍的回复，就是一定要非常详细认真的回复。然后最好能收一些小问卷，比如说对这个测量有一点小意见，比如说原来量表是十个item，然后我们只用了五个，你就不能只说这篇文章他用了五个item，就算之前有文章用了五个item，你也一定要再做一个问卷，反正也就200个人，然后就收一下这五个item、十个item，然后做一下他们的correlation，然后跟reviewer说一下我们这五个item是可以的，就是一定要回复的让editor看见你的effort，effort我觉得是非常重要的

刘书博老师： 嗯嗯明白。

那还有一个追问的问题。就是您觉得现在有一些负面的声音，说现在学术变得越来越所谓的专业性的专业化。那可能让实践者有点接受不了，或者是不理解、看不懂。那就是您做了这篇论文之后，会不会觉得它对于这个现实有所贡献，或者帮助很多实践者、能够给他们带来价值，有没有这样的成就感呢？

受访者： 哎，实话实说，并没有，这是为什么我喜欢做劳关研究的一个原因，因为我觉得劳关的研究更有现实意义。说实话大部分人管研究我是说服不了自己有怎样的现实意义的，尤其是我自己都对我自己有的时候有些不满意。就比如说像MBA的课，我到现在一直都还没有勇气去上，就是因为我觉得我还没有那个水平。你想我是劳人院出来的，我在本科我就听XXX等人的课过来的。我就觉得他们那些才是真的能够给实践大佬上课的人，而不是像我这种没有什么实践经验的、就是完全只做学术的人。我总觉得在实践方面还是差很多。

刘书博老师： 可能也是您谦虚了

受访者：没有，没有。其实我在国外的时候，我看其实国外的研究型的学者和商学院讲课的老师是分开的。商学院虽然有很多业界大佬来讲课，但他们也不是教授嘛。真正的教授大部分也是只做学术研究，其实还没有中国的老师跟这个实践界连接的紧密。所以我就觉得好像也没有必要。不知道，很纠结。

刘书博老师：是的，好像美国好多商学院他们是有可以跟实践对话的学者，但是好像非常多的还是做学术型研究的，或者我们现在就说他们是专业性的学者。但是在中国情景下，我们有时候会想，那美国就一定是我们未来要变成的样子吗，还是说可以走出一条不同的道路。另外美国是全球的学霸，那现在我们会从上到下的提倡把研究做在中国大地上，然后关注中国的问题，其实也是在说我们自己要构建理论，要有学术话语权和独立性。那在这种大的从上到下的倡导的情况下，我们是否还是沿着光华他们走的那条路去走，还是说我们可以去做一些不同的事情，或者说这个专业性标准到底会不会变化？可能都还是挺有意思的可以探索的方向。

受访者： 是。反正从现在申请课题来看，对现实建议要求越来越高了，因为现在都是从问题出发了。就是你申请课题的时候都是从问题出发，而不是像原来从理论意义这种虚的东西来出发了。

刘书博老师： 哦。所以之前我们访谈也有一位老师说，这个课题是要接地气的，好像就是说要解决现实问题、身边的这些现实问题、重要的现实问题。而且我们好像课题还是比较重要的。

受访者： 是。我觉得肯定是一个大趋势吧，你看《管理世界》上大部分文章，我觉得还是非常有现实意义的。

刘书博老师： 对，对。那XXX老师是您的学长嘛

受访者： 其实也不是，因为他是人力系的嘛

刘书博老师： ok，对，XXX现在在康奈尔的劳人院，他去访学去了。

受访者： 对对，他去之前我还让他跟我们劳人院一个老师吃了个饭，康奈尔回来的。

刘书博老师： ok，但是我看像我认识的劳人院的老师好像都是在做实证研究，有没有做比如说社会学、人类学的这种案例质化研究啊，应该刚才你也说过有这个社会学的。

受访者： 人管的估计比较少，除了XXX是做案例研究的，年轻的老师基本上以实证为主

刘书博老师： 明白。那刚才您说是这个（劳关研究）越来越少，是因为美国的比如制造业工人整个规模在萎缩，然后工会在萎缩，导致这个学术标准可能也在萎缩。那中国的情况为什么也是这样子？

受访者： 先跟您区分一下啊，就是一个是人力系，一个是劳关系

人力系都是以实证为主的，我刚刚说的所有这些人基本上都是人力系的。劳关系的我没有提，因为劳关系的老师我估计您也都不认识。劳关是个非常小众的学术圈子。我刚读本科的时候，劳关都没有成立系。我当时本科拿的还是社会保障专业的学位，然后有括号劳动关系方向。在我读大三的时候才成立了劳关系。然后XX大学已经算是最早成立劳关系的学校之一了，所以劳关系的历史非常的短，所以学术发展就···本来也就没怎么发展起来，又遇上整个国外的学术界都在衰落，所以整个就没有怎么发展起来。

刘书博老师： 明白了，明白了。好的，好的。那我这边主要的问题就问完了，那后面就交给两位博士生，让这个秋莉和梦娜他们再问一问。

吕梦娜： 好的，嵇老师，那我先问吧啊，我很高兴能够跟嵇老师有更深入的一个交流。之前上过您的课嘛，然后就对您有一些些了解。我印象最深就是给我们上HLM课的时候，就那个方法课。然后您备课特别认真，就我感觉已经接触到就我上过课老师的天花板了，所以真的就是印象非常非常好。

然后后来就是我两次答辩，都是您作为评委老师，我觉得您提的意见真的是会让我反复去看的那种。就是刚开始听我会觉得有启发，然后后面可能会真的促进我去改一些东西，然后就很高兴。

刚刚听您跟刘老师又聊了这么一些，感觉这个形象真的是越发高大了。然后我准备了一些问题，就是我担心后面我可能就不像一个专业的访谈人员那样对您问一些课题相关的问题。确实是希望能跟您取取经，然后您也给我们一些建议啥的哈。我尽量有点条理。

受访者： 没事儿，没事儿

吕梦娜： 遇到自己系的老师，我有点激动哈哈

那我就先从您上大学的时候开始吧，因为我看您本科还有硕士都是双学位嘛，经济学和管理学的，那想请您比较一下，或者您评价一下，这两个学科有什么异同点？有没有觉得比较鲜明的异同点呢

受访者： 因为我是理科出生，然后我还是属于理科非常强的，属于在高中参加过全国奥林匹克竞赛的人，然后高考的时候因为填志愿填失误了，去了一个管理学，就是觉得管理学对于我这种理科背景的人来说，它不是一个非常科学性很强的一个东西。人家都说管理学也是一门艺术嘛，但是对于我来说我就想学一门理科。所以我当时又辅修了一个经济学。我就觉得经济学对我来说它更偏向于数学，就所有的东西都非常明确，有一个答案，这是我比较喜欢的。

然后管理学，我一开始就很疑惑，本来我文科学的也不好，然后整天背那些东西，然后学那些东西，我就总是会对自己产生怀疑。所以我就是在本科阶段我觉得我对经济学的喜欢还是要超过管理学的。所以我在硕士阶段我也还是经济学、管理学都修的。但是我在最终到博士阶段我选择了管理学，因为那时候也成熟了嘛，我觉得管理学还是非常有魅力的。所以我最终还是选择了管理学

吕梦娜： 那这个时候就不会对科学性过分的追求了吗？

受访者： 我还是用科学的方法来研究管理学呀，因为毕竟研究和之前本科阶段的学习不一样嘛。学习阶段基本上上课就是听老师侃大山，一节课听下来，听的时候我就觉得哇老师好帅，就是让我全神贯注，就是觉得非常非常有道理。但是听完之后我又觉得好像什么都没有学到。

但是研究阶段又不一样吧，你做管理学的研究其实也是非常科学的啊，还是用一套非常规范的范式来研究的

吕梦娜：是，但是就是您刚刚也提到了会用比较科学的方法，就像我们说实证这种的。那质性的研究您觉得它科学吗？

受访者： 我现在是越来越发现质性研究才是王道，因为我原来还是比较迷恋实证研究的。所以你看我其实到现在我都没有做过质性研究，但是我最近有在尝试做质性研究。其实我接受质性研究的训练是非常多的，因为我在劳关嘛，劳关所有的老师基本上都是做质性的，所以我基本上接受的是质性研究的训练。然后所有的老师都说质性研究很难。就是我记得有个老师说的特别清楚，在经济学家里面，做实证的永远只能是二流经济学家，然后一流和三流做的都是质性。所以你要真正做得好、你要成为一流，你必须做质性。但是质性是挑人的，你要是能力不够，你就只能变成三流。所以我就是总觉得自己能力不够很难做质性，当然也是要尝试。

吕梦娜：那您觉得质性和这个科学性冲突吗？矛盾吗？

受访者： 我觉得没有吧。没有冲突，因为现在一方面质性研究它也有非常完善的方法嘛，像扎根理论。另外一方面你做完了之后，我们现在都讲究也可以去验证你的理论。

吕梦娜： 嗯，嗯，是的，是的。那后面就是您在硕士期间是去美国留学还是访学还是怎么着了？

受访者： 是留学。

吕梦娜： 哦，是多长时间呢？

受访者： 嗯，就一年，我那个硕士学位就一年

吕梦娜： 那这一年的经历，包括您在中国也接受了很长时间的高等教育嘛，您觉得中美之间有什么比较鲜明的对比吗？

受访者：说实话，我在学习期间感受到的差异并不是特别的大，我主要是在找工作的时候发现了特别大的差异。就是中国和国外的商学院有很大差异。我当时先面的是国外的很多学校，因为我们有AOM会议嘛，你们到时候找工作也可以去AOM上找，强烈建议你可以去AOM上找。然后AOM上就是在开会之前你就可以投简历，一般每年都有两百多个商学院在上面，你就投简历嘛，然后去面试。然后我当时只是面着玩玩，所以我最后只接了一个二面，就是UCL的二面。然后我去UCL二面的时候，我就发现跟国内的面试差距太大了。

UCL的面试持续一天。早上的时候是博士陪我吃饭，然后我就问那两个博士，我说你们这边面试新老师都是博士来陪吗？然后他们说是的，他们就是会收到一个candidate的list，然后他们会选，然后你对哪个candidate感兴趣你就去陪他吃饭。然后上午的时候是做一个job talk，整个学院的老师都在。然后中午的时候是跟系主任吃饭，主要是跟系主任聊聊。然后下午的时候是参加博士的一个reading seminar。就是他们博士每周都会有个seminar，然后你去参加他们的seminar，跟所有的博士进行沟通交流。然后晚上的时候是跟同事吃饭，跟你们系里面其他的老师吃饭。因为国外一般系也很小，没有几个老师，然后就所有老师陪你一起吃过饭。然后我还问了那些博士，我说他们有没有发言权，在选老师的这个事情上。然后他们说他们也是可以提意见的，就是他们的老师也会问他们更喜欢哪个candidate，或者你觉得哪个candidate是有问题的，你也是可以提的。

我就觉得其实选老师就可以看出来一个学院他的一个建设的目标嘛。他既然需要所有的老师来吃饭面试，那就是他需要一个能够跟所有老师合作的人。另外他还让博士来面试，那就说明他是希望这个老师能够更好的教博士，能够跟博士有更好的沟通交流。所以我觉得这个是他们的一种理念，我觉得特别的好，但是像我们国内其实基本上就是老师面试，然后学院领导定。我就觉得就是跟国外的理念还是有挺大差异的。

吕梦娜： 对，咱们是不是跟公司有点儿一样？

受访者： 对对，是的。然后我问他们的博士培养是什么样的？他们就说，他们也有他们的苦了。像我们国内的博士说实话导师还是很负责任的。像你发文章呀、博士论文啊都是会很负责带你的。但是国外的不是这样，国外他们都是自己找导师合作，而且导师不会给你提供idea，就是你有什么idea了，然后你就去找你觉得可能跟你合作的导师，问他们感不感兴趣，我觉得这也各有利弊吧。

吕梦娜： 嗯，嗯，了解。

刘书博老师： 那嵇老师您在国内是什么体验？

受访者： 国内找工作的话，国内我的体验就是感觉主要还是一方面跟所有老师接触的比较少，就是我单方面的输出。

 就不像在国外的时候沟通交流特别多。在国内就是做一个job talk，然后会有人问问题。但其实大部分时候都没有人问问题，都很尴尬。然后或者就是有一些人问一些无关痛痒的问题，就是沟通交流比较少。

刘书博老师： 了解。那这里我补充一下我的经历，我没有在美国那边找过。但我在读博士的时候还在中欧打工，所以中欧的面试就是美式的那种。他有一个campus visit或者是一整天的interview，当然也不是说一群人在听他讲，当然有这个环节。然后会拜见不同的这个系的各个老师，都会有一个small talk，然后就是会有一个学生问他，就比较开饭的那样的环境。

然后我后来就开始在其他中国一线的高校，什么985、211呀找工作。然后给我印象最深刻的是南方的华南理工，它的面试完全是公司的面试。然后这个候选人就在外面排着队坐在一排，然后他们里面做一个会议室。然后每个人进去大概有20~30分钟的时间。

就这么长时间。然后就差不多很快的决定。那当然这个决定就是师资博士后嘛，会招挺多的人啊。然后他做决策的，我觉得整个周期还是挺短的，他就没有充分的想要去认识你，就是走一个流程的感觉。

然后还是像嵇老师您说的那种沟通交流就是单方面的，然后他们问问题可能也不是特别的能够问到点上。然后后来我又去了南京大学，然后那个体验就更糟糕，它是没有那么多候选人排队，但是老师就完全听不懂你在讲什么的那种感受。那么问的问题就非常带有挑衅的意味，甚至是这个贬低的意味啊。除了南京大学，还有四川电子科技大学也是同样的。可能跟我的研究范式也有关系，因为他们都是做实证的嘛。然后我给他们讲的是一个话语分析，就案例研究，所以他们会觉得你这个东西好像不是一个研究，就各种的批评指责什么的。好，我就补充一下这个。

吕梦娜： 那李老师我继续问哈。就是后面您选择当老师。就是做教职之后，你选择学校主要关注了哪些因素呢？

受访者： 我主要就是关注氛围。因为我也不是一个成就动机很强的人，然后就是想要待一个非常让我觉得舒服的一个环境。然后我其实也面了别的学习，不过我面的学校不是非常多，因为我选择非常有限，我只能在北京，所以面的学校很有限，但是中财给我的感觉特别好，面试的时候当然有可能是因为有我直系师兄在，所以感觉很亲切。然后有很多人跟我分析过这个北京的各个高校嘛，比如就跟我说对外经贸商学院氛围非常的可怕。

刘书博老师： 能不能再详细的说一说。我们前面也

 访谈过对外经贸。但是我们访谈的那位老师他就感觉有点敏感，但是咱们八卦起来是可以聊的。

受访者： 就是他们跟我说对外经贸有两个清华的女魔头，然后就是她们掌控着整个戏，然后还有内斗。所以就是你去了还必须得站队，我一听到站队我头都大了，

刘书博老师： 回到国企了。

受访者： 对，所以我就很烦这样。

刘书博老师： ok

吕梦娜： 所以你觉得中财的这个氛围是让你比较舒服的，这是你最看重的。那其他地方有考量吗？比如说考核。

受访者： 考核我觉得现在都半斤八两吧，我对比了一下各个学校的考核，说实话都差不多。因为你合同上的那个考核标准只是一小部分，你最终想评副教授、教授是跟你这一届卷的情况来的。

大家都很卷，合同

 上写什么其实也没有那么重要。

吕梦娜： 了解，就是我很好奇您现在也是签合同，但不是师资博士那种，您是几年的合同呀？

受访者： 三年，我们三年一签。

吕梦娜： 就是3+3，然后就是长聘的，了解，我感觉听您说好像合同的任务还相对比较简单的。

受访者：不简单，到现在还没完成了。

吕梦娜：我以为会比较简单。所以您说到副教授。

受访者： 只不过在找工作的时候我就并没有太考虑合同的要求。

吕梦娜： 了解了解，那因为之前听过我答辩，您也知道我现在比较关注非升即走这个问题嘛。我想知道在您看来您对这个制度是怎么评价的呢？

受访者：你知道屁股决定脑袋，我既然站在这个位置上，我自然不是非常喜欢这个制度的。我倒不是不喜欢“走”，而是不喜欢时间特别短。

因为我其实有几个朋友是在国外大学任教的，他们也是要非升即走的呀，没有任何一所大学一进去就是track。但是国外一般都是给的时间很长，一般都是八年。但是我们国内这个三年实在是太短了。就像我那篇JAP，从我开始想到这个选题到收数据，到写到发出来一共花了四年的时间。然后我现在为了应对我这个考核，我不得不把我当初JAP的文章把它投到别的期刊上。因为我没有办法接受这么长的时间，我完成不了我合同的任务。所以我就是觉得这个时间太短。我觉得如果放到八年，你如果八年完不成任务，然后你就要离开，我觉得这样我是可以接受的。但是三年我觉得太短了，尤其是对于国外发表来说，尤其是我们学校还不能是online的，还必须是published的，这个时间又拉长了很多。

吕梦娜： 明白，那您觉得我们现在高校推行这个非升即走，它主要关注的是它的 什么功能呢？

受访者：我觉得一方面是选拔吧，从人力的角度来说。因为你在招聘的时候你只能看到他博士期间的一个水平嘛。所以你其实很难预测他未来真正学术生涯的水平。所以我觉得这个合同其实给了学校一个更长的一个考核的期限。然后另外一方面其实也是一种督促机制吧，虽然说学者不应该被条条框框束缚，应该让他们任意的做研究，但也这个就是x理论和y理论。

吕梦娜： 是的。其实咱们学术界的很多研究者，包括青教也好，包括即将踏上工作岗位的博士生也好，其实对于非升即走的这个评价或者口碑都不太好嘛。但是为什么非升即走又能在中国高校这么顺畅的推行下来。因为现在就是很多双一流高校，或者说一些研究型高校大部分都实行了这个非升即走嘛，所以您觉得这个主要原因是什么？

受访者： 从我们经济学的角度来分析，就是这个劳动力市场的问题嘛，就是因为现在供大于求。但是我觉得这个现象也不会持续太久吧。我现在身边越来越多的人远离高校了。就是大概十年前高校并不受欢迎，这十年高校又非常受欢迎，但是之后十年又不一定了。像今年来应聘我们人力系的，其实有两个师妹，我还挺熟的，都是北大一个发了JAP，一个发了AMJ。然后现在他们俩这个水平都已经面不上我们中财了，就已经卷到这个地步了。然后他们俩现在都已经去面国企了，而且其实更prefer国企的offer。包括我还有个师妹，她是在北大经院读博，她拿到了两个offer，一个是北师大师资博后的offer，一个是中办的offer。然后她毫不犹豫的选择了中办。所以就是我觉得搞师资博后、搞非升即走以后，就是会留不住这些人的。我觉得再这么搞，未来也是没有前途的，就是面对剥削，我们这一代人总会觉醒的。我觉得

大家又不傻，我觉得现在就是这样。尤其是

你们这一代。像我们这一代，虽然我是90后，但我觉得我的思维还是80后，就是还能够忍受这些。我觉得年轻一代越来越不能忍受了。我今年还有一个我本科同班同学，他是去香港读的博士，博士时间延长，他今年才找工作，然后他拿到了两个offer，一个是我们劳人院的offer，还有一个是西交利物浦的offer，然后他竟然去了西交利物浦。就是要像我看来肯定选劳人院，就是人大怎么着，这个牌子在那儿，即使是师资博后，但是我觉得未来发展也好，他就不这么觉得，觉得工资又低，考核又那么严，去西交利物浦就已经可以享受生活，还可以有钱买房子。就是我觉得你们这一代人就不一定能够接受这么残酷的剥削了，就如果还这样搞的话。

吕梦娜： 对，您这么一说，对未来前景还是比较有希望的。

刘书博老师： 我再插一句啊。就我个人经历，我当时其实也拿到这个985学校，像同济大学，它一开始说给这个教职，但是后来又出尔反尔，就给到这个师资博士后。那当时我其实也是毫不犹豫的就选择换一个，就换到中财嘛。所以我觉得这个事儿，虽然我是80后，但是我比较90后，咱俩颠倒。

受访者： 对，就是像这种不愿意被剥削的人

越来越多，自然会逼着大学

改变。

刘书博老师： 对，对。而且我们前面访谈过清华大学的。那个老师说他后面三届的师弟师妹啊。都没有再去往学界。就博士毕业就离开学界了。然后他们老师还很生气。

受访者： 现在真的是一个趋势了，尤其清华北大的博士，因为他们在业界找工作非常好找，工资又高，就没有必要憋屈自己了。

吕梦娜： 对啊。然后您现在工作得多长时间呢？

受访者： 我工作快两年了。

吕梦娜： 对，就是您毕业之后就直接来中财这边工作了。那您之前实习的那两段经历，对您这个后面找教职有什么帮助吗？除了坚定您找教职的决心之外。

受访者：其实在硕士到博士期间，因为我硕士在国外念书回来考博，中间隔了一年嘛，就等博士。我中间工作过一年，在咨询公司，然后我觉得还是让我对企业有了更深的了解吧。尤其是做咨询，我觉得对我做学术还是比较有帮助的。

吕梦娜： 对，也很相关的。然后您定在高校的时候想法很多，然后尝试也很多，包括实习也很多。我就非常的羡慕这种时间把控力这么好的人。

刘书博老师： 您您研究生是在纽约、华盛顿？

受访者： 对，在DC。

刘书博老师： ok，ok，我看那个人民大学也有一个硕士。

吕梦娜： 对，是的，双硕士。

刘书博老师： 你就念完人大的又去到美国念，是这样吗？

受访者： 是，因为我本来已经保研了。但是大四的时候我们人大有一个项目，就那个张磊他不是跟耶鲁大学很熟，然后每年有20个学生可以去耶鲁交流。然后我去了一趟耶鲁之后我就想出国了，但是因为我已经保研了，而且我当年申请又来不及，然后我就后来又申了一个。

刘书博老师：好，那后来那个咨询工作是在哪个公司？方便说吗？

受访者： 在xxx，它是北京国资委下面的一个企业，属于国企咨询公司。

刘书博老师： ok，好的。

受访者： 然后主要我们是给国企做咨询，就是管理咨询，我主要做人力。

刘书博老师： ok，好的，了解，谢谢。

吕梦娜： 稽老师，就是因为我上课的时候有时候听老师们说在博士期间的那种科研的强度也好，跟当老师之后是不可相比的。就是当老师之后好像拿能拿出比较多的时间做科研的时间是比较少的，相对来说就可能会有这种时间方面的压力。那您会有吗？

受访者： 太有了，每天都在做挣扎，感觉时间不够用。不过其实我觉得央财已经好很多了，尤其是对于我这种其实学院事务参与不太多的老师。其实平时花在那种行政事务上时间是很少的，我觉得中财这方面特别的好，然后我们的课也并没有那么的多。我主要是因为身为女性的一个问题，就是家里要照顾孩子的问题。因为孩子太小，所以就是工作生活难以平衡。

吕梦娜： 明白。那感觉您对咱们学院的评价还是蛮高的，您觉得咱们学院有哪些地方是需要去提升改进的吗？

比如说非升即走这个时间太短了，但是他可能改不了。还有其他的吗？

受访者： 我最大的遗憾就是跟学院老师沟通交流特别的少。就是我看到其他同学在其他的学校就是感觉他们有很多的活动呀，比如说其他老师之间的一些沟通交流，当然有可能我是2020年入职，刚好遇上疫情，所以导致我导致入职了两年，大部分老师都不认识，然后老师们也不认识我。我就是这一点比较遗憾，就是老师之间的沟通交流比较少。

另一个，其实像我们现在人力这一块的研究，你也知道我们是越来越偏向大数据的。所以我也是非常想跟系里的老师请教请教，然后看有没有合作呀，但是就是缺少这样的机会吧。

吕梦娜： 了解。后面还有几个零散想问的，就是您作为老师也两年了嘛，然后您觉得会让您感觉到比较有幸福感或者自豪感，或者其他比较正面情绪的时候是什么时候呢？

受访者： 其实说实话，就是我最幸福的就是上课的时候，尤其是比如说像你一开始在访谈前跟我说的那段话，我听了之后我就觉得特别的幸福。就是文章发了当然会比较幸福，但是我觉得更幸福的就是比如说像你刚刚说的那些话，像比如说我去年指导的一个学生，然后之后他给我送了一个杯子，我觉得就这些事情让我觉得特别的幸福。

吕梦娜： 确实是。那有没有让你感觉到很困难，或者比较压抑比较难熬的时候？就是作为老师这份工作的话给您带来不太好的消极的情感体验的，有吗？

受访者：我觉得还好吧，如果我今年课题没升上的话，我估计会比较的伤心。然后还有就是比如说有老师说你要多参加学院的活动，要多给学院做贡献啊。但是我有的时候就找不到方法，就是你不知道应该怎么参与进去。所以那个时候可能会比较挫败，我觉得其他方面还好。

吕梦娜： 了解，这些其实不是你能控制的，都是强加给你的。那您作为学者就是研究者，而且是人力这一方面的嘛。然后就是我也稍微有一点了解，就是您觉得在这个过程中能够给您带来正向的体验以及负向体验的事情分别是什么呢？或者人力这个词儿去掉也行。就是你作为学者，作为研究者的话，在你做研究的过程当中。

受访者： 其实我做研究的动机并没有那么强，但是做研究还是一件我觉得还是比较开心的一件事情。我尤其是喜欢读文献，我也不知道为什么，就是读一读别人写的东西，然后尤其是发现一些东西就是突然打开了你的思路，让你眼前一亮，就是那种感觉让我非常的喜欢，我觉得这是我非常有幸福感的事情。 看来我是一个我是一个接受型，不是输出型的人。写东西并没有让我觉得那么幸福，还是看东西让我觉得比较幸福。

然后挫败感主要是我觉得我现在在学术当中，作为学者最大的挫败感就是其实自己有很多设想，就是没有办法实现。我觉得这是我现在最大的一个挫败。比如说我想做大数据方面的研究，但是我没有这样的数据来源。比如说我想到了一个model，然后我所有的数据不能支持我的model，我只能放弃。就是这个让我有比较大的挫败感。

吕梦娜： 就是说做研究的实行方面可能有一些现实的一些阻碍什么的。那您刚刚也提到研究，您是怎么定义一个好的研究的。特别是对我们管理学来说，怎么定义一个好的管理学研究？

受访者： 我现在认为一个好的管理学研究它必须要基于现实问题。就是真的是现实生活当中，我们现在这么快速发展的社会，真的出现了一些问题需要我们去理解、去思考、去解决。当然解决是很难的，我们主要还是在描述问题，然后能够用非常规范的方法能够把这个问题给回答了。这就是一个很好的研究。

吕梦娜： 其实你也提到了在课题方面会对现实问题更加关注嘛。然后对于我们博士生来说，你觉得有什么好的办法能够去接触到这些现实的问题？我还包括周围几个同学聊天可能会觉得还是从文献到文献那种就不落地。

受访者： 是的，是的。其实从文献当中找问题是很难的，比如说其实你有很多同学没读博士的已经工作的，你跟他们聊是最快的，我都是这样。我就是没事儿跟他们吃饭，然后就跟他们聊他们工作当中有什么好玩的事情，遇到什么问题，就是这样。

吕梦娜： 了解了解。那您对于未来的这个职业，老师也好，学者的这个身份也好，您有什么期待吗？

受访者： 我的期待吗？我的期待就是能够早日没有学术研究压力，做自己想做的研究。

吕梦娜： 会回到劳关方面的研究吗？

受访者：会是劳动者，其实现在也在做。

吕梦娜： 好的，老师太谢谢你了，感觉问了好多好多问题。

受访者：没有，没有，我们以后可以随时私下交流。

吕梦娜： 好呀好呀，太好了。老师，我先问到这里。

黄秋莉：好的，刘老师，那我现在问哈。稽老师您好，就是刚刚听您和刘老师和梦娜已经交流了很多嘛，我觉得已经很全面了。

我主要有以下几个补充性的提问了。第一个就是还是挺好奇的，就是因为您当初是理科生嘛，您会觉得这个管理学会偏文科，所以最后导致您的去向就是会喜欢经济学嘛。然后您提到申请博士的时候，您突然意识到这个管理学就非常有魅力嘛。我就很想了解一下您的这个转变原因是什么。是不是有发生什么特别的事情呀？或者是有什么样的经历吗？

受访者： 倒也没有特别的事，我觉得的确跟学科设置有关系。我们各个系最近都在讨论新的这个本科生方案。然后其实你们现在的这个培养方案真的是非常好，我觉得就是你们各个方面，比如说像你们还有计算机，像现在我们中财商学院的本科生，还有很多计算机的课，会学这个JAVA，学C++，学这个Python。就是我觉得这个是比较标准的比较好的商学院的基础课程。但是在我当时本科的时候并不是这样，我们那个时候大部分课程真的都是在“侃大山”，

所以才让我觉得管理学非常的非常的像一门艺术。但是直到我到博士阶段或者说到硕士阶段，我才接触到就是偏数理方面的一些管理学的基础课程，我才意识到管理学它其实也是一门科学。

黄秋莉：明白了，那之前听您提到这个质性研究，您觉得现在它是非常规范性了，那你觉得这种规范性就是它这种科学性的来源吗？因为我觉得这个质研究可能有的时候也挺像一门艺术的，但是又有很多学者会认为说质性研究就是很难嘛，我感觉当中有一定的悖论，也不知道我表达清楚没有。

受访者： 我理解，就是对于真正一流的这些研究学者来说，他们肯定不是用扎根理论做的。我最喜欢举的一个例子就是我当时上质性研究课的时候，那个老师跟我们举的例子就是毛泽东的例子，就是他去湖南考察农民运动，考察了100天，写了一个湖南农民那个运动考察报告。那当时有那么多人看到了这个农民运动，为什么只有毛泽东提出来了只有农村包围城市才能救中国？那毛泽东肯定不是扎根理论访谈出来的呀，他就是通过观察自己提炼。那这个其实就是个人的一个洞察力吧，我觉得这个是真正一流的质性研究学者，他所具备的一个素质。但是我们这些菜鸟不具备这个素质的时候，我们只能通过这种规范的研究方法，通过大量的访谈，然后一级一级编码，然后得出一个相对科学的结论。但是我觉得你如果真正的想要提炼一些理论和概念，我觉得不是靠这种方法得出来的。

黄秋莉：明白了，人的这种洞察力可能会比较重要一点。

那老师提到，感觉这个北大的教学和这个人大的教学方式可能是不太一样的嘛。就我刚刚听您提到的一点就是说这个北大教学可能会比较重视方法，那人大的话可能这方面就没有那么的注重这一块儿。那您自己也是有一个北大的指导老师和一个和人大的指导老师，那您在接触这两个学校的学者的时候，您觉得他们的这个这种学术研究方式有哪些方面的差异吗？

受访者：就像你说的两个的差别一个受偏方法嘛，像北大的我的那个合作者老师他是统计学专业出身，他的这个方法非常的强。

但是说实话我很多的选题，我还是喜欢跟人大的老师聊，因为这些老师他的一个特点就是偏实践。他们在外面会做很多的咨询，然后他们的思路非常的开阔，他们会提出很多问题。就是可能他们提出的问题也不知道怎么用学术的方法去解决。他们只是有这样的想法也没有什么理论的基础。但是我觉得这个是很好的一个开始嘛。因为好多问题是最重要的，就相当于他们更适合跟你头脑风暴，然后你获得你的一些灵感。然后你具体的实操，比如说有些方法不太懂，你可以去跟懂方法的老师再聊哇，

黄秋莉：那挺互补的，感觉这个就是非常完美的一个搭配了。

那您提到就是中财的氛围比较好嘛，那您有没有考虑过就是未来会一直留在中财工作吗？

受访者： 我看我们系的老师没有一个走的呀，大家都是终身奋斗在中财。

黄秋莉：就是挺有这种归属感的。但是这种非升即走下嘛，肯定是有淘汰的。就是像对外经贸的老师也说他们的流动率是很大的。然后我之前在看那个张维迎老师写的书，他提到这个非升即走，虽然就是看起来会让大家流动走，就是离开这个学校，但是他的一个解释就是说你离开了也能够找到下一家。那就您的个人经验来说，就是如果真的被淘汰掉了，在中国这种市场结构下能够找到下家吗？

受访者： 我没找过，不过我觉得应该能找到吧，就是找的差一点呗。

黄秋莉：您身边有这样的例子吗？或者是您看到过的例子。

受访者： 我在国内倒是没有，国外倒是有。我一个朋友，他是八年的合同嘛，然后他今年是第四年，然后他就已经预感到他完不成这个合同，所以他今年就换了个工作，其实换的也还挺好的

刘书博老师： 换了之后还是要再签一个八年吗？

受访者： 对，就是你签的是八年的合同，但是你达成他副教授的要求了，你随时可以升副教授。其实他大概再过两年就可以了，他的要求已经完成一大半了，就另一个学校要求没有那么高。

黄秋莉：明白，好的。我的问题可能跨度有点大。我下一个问题就是说因为您要评这个职称嘛，那您觉得当前的这个评价体系是否合理呢？

受访者： 我觉得稍微有一点点不太好的吧，就是我现在觉得我们国内的评价大部分还是一刀切。我其实觉得这样不太好，因为每个老师有每个老师的专长。比如说像我们系的林琳老师，今年她就评教授，她的专长其实并不在于发文章，她的专长在于教学。然后他的教学非常有特色，我们都知道，然后对学生非常的好，但是她就是评不上教授，因为她有个短板，就是她发文章的数量。但是像我就愁的是我在课程思政、课程建设方面啊就不太行。你如果把这个给我换成一篇文章可能会做的更好。就是我觉得我们现在的评价体系……当然全才是很好的，他可能考虑的是每个老师不能偏科，你必须既有科研又有教学，然后又有服务，但是我觉得应该稍微flexible一点。就是允许一种兑换机制吧。

刘书博老师： 我们现在有一个新的评职称的文件出来，我不知道嵇老师看到没？他对于这个评教授的要求，我觉得更严格了。他其实把第一作者就只看第一作者，甚至通讯它都没有写进去，所以这一点就对很多老师都特别不利，因为人家之前积累的突然一下子变了，而且他变得也毫无道理，对吧？

而且它虽然也区分了教学教研岗和教学科研岗。但是教研岗又规定一条，就是只能是有公共课的这几个学院老师才可以，那包括商学院就不行。所以就感觉这个不知道他那个规则政策是怎么样就出来了。

受访者： 对，而且之前好像也没征求过意见吧，就如果征求意见的话大家一定会很多意见。

刘书博老师： 对呀，或者说征求过意见，但是他就是直接就这样。包括我们前面访谈那个政法大学的，其实那个老师就说有时候这样的一个政策出台，就是学校人事处的老师拍脑袋定的。所以有时候我就觉得，哎呀，我们这个制度环境还真是挺特殊的，跟其他这个地方的大学挺不一样的。

受访者：我们行政属性特别的强。

黄秋莉：对，是了。那就是除了刚刚提到的要更加的灵活性一点。您觉得还有哪些可以更好的方面吗？

受访者： 其他的我觉得还好吧。

黄秋莉：好。就是因为之前有一个上海交大的图灵奖的学者在一个非常非常有名的论坛大会上就提到说啊，我们中国可能会强调这个客观的指标。那他的一个建议就是说可以建立一个值得信赖的委员会，我不知道国外是不是这个样子的。那您觉得这种评价方式怎么样呢？

受访者： 是的，国外是这样的，是有这个评职称的委员会。说实话这个对于我这样的内向者来说，我也不知道我是不是赞同这样的做法。因为我那个朋友他离开的一个原因就在这，你看他其实发表非常的好，但是你一旦到这个委员会上，他很多时候看的并不是你的指标了嘛，看的东西都很不确去。然后还需要你跟这些委员会的成员，你要去公关他们啊。然后在中国这个环境下肯定会是非常卷的，大家都要去公关，我觉得这样也不一定是一件好事吧。我觉得这个本身制度是没有问题的，但是我总觉得在中国这种环境下实施起来会是一个问题啊。

黄秋莉：就感觉这种客观和人的主观判断如何结合起来，在咱们中国真的好像挺难办到的。

受访者：所以我们中国其实很多制度都是设计的很好，但是落地的时候遇到很多问题。

刘书博老师： 其实谈到这一点，我就觉得KPI在中国有时候它是一个迫不得已的选项。因为如果要相信专业人，像西方他们会有一个学术共同体的操守，那可能我们这个土壤环境就跟他们不一样。所以又必须得回到对机械式的KPI。但是机械式KPI考核，它又不能够触发这个学术共同体的这种责任担当意识。好像有点这个恶性循环。

黄秋莉：就两位老师来看哈，张维迎老师的书里说，就是人才选拔制度嘛，你可能最重要的一条就是你要招进来最优秀的学者。那在当前的情况下，就是我们以这种KPI衡量的这种人才选拔制度是不是达到了他的这样一个目标呢？

受访者： 既然是KPI那肯定是有一定问题的。你比如说像这种论文我们早就说了“破五唯”，那现在也还是破不了，KPI肯定是有一定问题的。但是我觉得也是现在最优的一个选择吧。

黄秋莉：明白。那最后一个问题就是想问问您的这个学术榜样是什么样子的？

受访者： 我现在的一个合作者，我觉得他就是我的学术榜样。

首先他是一个非常有motivation的人，就是成就动机非常强的人。

其次他是一个非常work hard的人，他的工作强度我觉得得是我的三倍吧。我跟他共同做三个project，我就觉得已经占满了我所有的时间。而我只是他众多合作者当中的一个，他手上同时可以做十几个project，就是非常的work hard。

然后另外一点就是我觉得他非常的精益求精，我觉得做学术这一点非常的重要。我其实算是野路子出生，没有受过特别正规的博士训练，所以我其实很多这方面的想法都是他给我灌输的。就是比如说你在说数据的时候一定要非常非常严格。比如说如果有任何不符合规定的情况，比如说匿名，如果我们真的没有做到这一点，我们就一定不要这个问卷，宁愿不要，我们都不能要。还有比如说你在分析数据的时候，也是你要保证你的所有数据没有任何问题，然后你的所有的这个code都要保存好。然后比如说你在写的时候，你引用的每一句话你都必须真的能够在你后面引用的文章当中找到。我觉得精益求精这一点对学术来说非常的重要。

黄秋莉：明白了。好的，我觉得已经耽搁你挺长时间了，我这边没有问题了。嵇老师，刘老师。

刘书博老师： 好，特别感谢嵇老师那个这个尤其是在休息的时候，而且您还要这个照顾小孩儿的情况下，耽误您那么长时间。特别感谢。找时间请稽老师吃饭，我们线下再聚。

受访者： 好呀，好呀，祝你们这个项目顺利，早日看到你们的文章。

刘书博老师： 谢谢，谢谢您吉言，我们以后有机会一起合作探索。好的，那我们后续把这个访谈稿发给您，然后您看看有什么需要修改更正的地方。好，谢谢，谢谢稽老师。

# 受访者11

刘书博老师： 好的，那介绍完我们就正式开始吧，我们也不用非常正式，疫情原因也没有约您线下的时间，今天线上呢也不是非常结构化的问题。大概有一些主题。

第一个主题呢就是您当时选择读博士时是知道这是走向学术道路吗？还是说也不一定？

受访者： 对，当时是知道是要走向学术道路。我其实一开始读博是没想好的，因为我本科毕业之后呢其实是保送研究生的，然后当时去到了上海同济大学。当时其实还有一个学校也向我伸出了橄榄枝，就是西交大。那当时呢他的一个项目是硕博连读，我后来就没有去。其实当时我就没有想好要读博这条道路。因为我知道后面基本上读完博士可能就业的路径大概比较窄，就可能大部分的概率是去高校工作嘛，所以当时也没想好。

后来读完硕士一开始也是想找一份跟管理呀、咨询啊或者金融相关的工作，然后我也去做过一份实习，就是在实习过程当中，我慢慢就觉得可能这个不是我想要的。然后我就觉得好像实习的这个过程让我觉得每天虽然很辛苦、很累，但是好像没有太多的收获。我好像还是比较期待说每天我都能学到一点东西。这才坚定了我要读博的这样的想法。

然后当时其实同济也有机会留下来，就是从硕士转成硕博连读，也经过了面试啊、考核啊各方面，就是也有这个机会。但是后来想说还是趁这个机会去外面看看，去国外再看一看，所以当时就开始了申请的这个道路。

所以当时其实还是知道就是说读博士基本上是在高校就业这样子。

刘书博老师： Ok，那您本科是在哪里念的？方便知道嘛。

受访者： 我本科是在西安，是在陕西师范大学，是一所教育部直属的师范院校。

刘书博老师： 哦，哦，那我们其实本科都是在师范类，我是在东北师大。那你本科就是学管理学吗？

受访者： 对，对对，本科就是工商管理，然后细分的专业是市场营销，就是我们那个班是市场营销班。

刘书博老师： 哦。那您研究生是哪一年开始呢？

受访者：2010年开始的

刘书博老师：哦，2010年。那个时候我应该也在上海，我是2008年研究生毕业就到中欧国际工商学院。然后2010年开始读博。但是我在英国有一年的课程，所以其实课程上完之后我就又回到中欧，然后从2011到2014（2015）都在上海。

ok，那您当时申请为什么选择去荷兰的大学？

受访者：是荷兰的格罗宁根大学，就在北边那个小城市，北边那个城市就是格罗宁根市。当时其实也是身边正好有朋友想去到荷兰，然后相当于给我推荐了这个国家。我一看确实感觉不错，而且荷兰的这个教育水平各方面其实比较平均。不像说美国可能好的好、坏的坏那种。而且欧洲可能是我比较向往去到的一个地方，因为当时可能觉得各方面景色啊、生活啊各方面可能就比较适合，符合自己期待哈。所以就去到了那里。

所以也是身边有朋友正好有相同的这样一个求学的路径，正好一起开始了这个申请的过程。

刘书博老师： 明白，我那时候申请也是在上海，在中欧那边老师会极力的推荐说首先选择还是美国。

受访者： 嗯，对，对，我当时去到荷兰的时候，我跟我那个导师见面的时候，他也问了我这个问题，就说一般好像都会选择美国呀、或者是英国，他也问了我同样的问题，怎么会选择荷兰这个城市？

其实我感觉也都是机缘巧合，正好也是有朋友有这样的推荐，然后正好也是开始申请，然后后面的过程都比较顺利，所以就顺理成章的就去到了这个国家。

刘书博老师：所以您看到的就是格罗宁根大学有一个博士项目，他们在招生是这样子。

受访者：对，对对

刘书博老师：我记得是学生好像去了之后就有比较清晰的一个研究项目了，是这样吗？

受访者：我记得我个人的经历是我是写了自己的所谓的research proposal。然后我们那个学校相当于有个专门管理博士研究生的一个committee，就是委员会，有专门负责的人。当时其实我一开始也是像其他的同学一样会有这个套磁的过程，就是看到比较感兴趣的导师给他写邮件。然后后来可能这个导师看到我这个以后他比较感兴趣，他可能就直接跟这个committee的负责人就讲了，他是专门负责招生的嘛。所以就后来是这个负责人跟我联系说，“是不是对我们这个感兴趣”。那就开始组织一次面试，然后面试过程当中，有committee的这个负责人、还有对我感兴趣的导师、还有相当于管理系的系主任三个人，对我开展开了一个面试。然后后面就比较顺利录取了。

所以也是相当于我把自己的研究兴趣和研究计划先跟对方沟通了以后，然后对方也同样觉得比较感兴趣，才开始了这样一个过程的。

我记得当时也有一些同学可能直接就是老师提供了一个项目或者什么，然后他比较感兴趣。申请的就是跟我的路径可能不太一样。所以两种都有。

刘书博老师： 哦，明白，明白。那您去荷兰那边的博士训练过程，您觉得是之前都能够知道、还是说有比较多的意外呀？或者说一些让您觉得有没想到的地方。

受访者： 应该说还是有一些些的。虽然我去之前其实是比较懵的。我看现在很多学生他们申请博士各方面思路都比较清晰，可能也是因为现在信息也更容易获得哈。我那时候觉得我是比较懵的状态去到那里的。

然后我的那个学校可能也不代表全部荷兰的院校都这样哈。像我们从中国去到那边读博的话，他会先让你去学习一些所谓他们学硕的课程。他们的硕士分两种。一种就是读完一年大部分的人都去就业了，另外一种就是学硕，他可能会继续再读一年，然后很大一部分比例的人可能最后会走上读博的这条道路。是区分这两种硕士的。我觉得我们在那边没有任何的这种课程学习的基础，所以他会建议我们修大概40学分的学术课程。然后自己跟导师商量去选择这样的课程，然后这个课程可以在博士四年内修完，我当时是选择在第一年就全部修完，我的计划是觉得后面三年我可以专心focus在我的研究上面。所以是这样子的。这是我一个比较意外的点，就是我没想到过去还得上课。

然后另外一个就是——这可能也仅代表我跟我导师之间的互动——我导师是一个非常强调学生独立性的导师。我记得我刚开始做我的项目的时候，他就会跟我说这是你的项目，你要领导我们，就是你应该作为一个leader，然后我们主要是做support和guide。他还跟我说过一句话，我印象一直很深刻。他说你要做好fail的准备，就是他觉得有可能项目也会进行不下去，但是你要做好这种心理准备。所以整个过程就是他会非常强调独立自主。

而且荷兰这个博士项目会在第一年之后进行一个考核，也是由刚刚我提到那个专门负责博士生管理的committee共同决定的，就是到底go or not go这样一个决定。如果你考核合格了，他会把你留下来；考核不合格的话，可能就觉得你就得走，是这样子的。这个也是让我比较意外的一个点。

刘书博老师： 嗯，嗯，那情况跟我这边也是一样，我们也是第一年要考核，要上课，research master。

受访者： 对对

刘书博老师： 嗯，嗯，您接着说

受访者：就是这几个点是我比较意外的。就是独立自主这个哈，我印象中包括国内可能是这样一个模式，就是可能是你导师有个项目，很多博士生就会跟着一起做，然后师兄师姐师弟师妹之间会有很多的合作。但是我们即使是同一个老师的学生也是各做各的。包括我那时候相当于在荷兰做了一个survey来支撑我这个博士项目的，整个过程都需要我自己去做。当然也是因为当时我的导师可能在资源方面、network方面确实也没有。但他又觉得自己做一个survey对于博士生来说是一个比较好的训练。所以他非常鼓励我去做这件事情，尽管他没有任何资源支持帮助支持我。所以整个过程也有很多挑战性哈，也有崩溃的时候。但是回过头来看，这个就是一个让我比较意外的点，另外一个也是对我成长还蛮有帮助的。

刘书博老师： 嗯。嗯，是的，我听起来是跟英国的培养模式很像。因为我导师那个时候也类似这样子，包括我的一些师兄师弟师姐师妹，大家做的似乎也具有明显的差异性，也不是好像能够契相互契合在一块儿的。他们每个人都是有自己的独立空间。

那您在毕业之后当时是考虑直接回国嘛？

受访者： 对。因为我当时是走的国家的留学基金委奖学金出去的。所以按照这个政策规定、合同规定，我是必须毕业之后应该要回来的，至少我记得是两年还是几年，就是至少要服务几年，所以当时我其实也没有想过要留在当地，就是基本上是准备回国。

刘书博老师： 哦，我想问一下荷兰格罗宁根大学这个老师的人事制度，是按美国那套tenure还是说比较偏欧洲的那种，就有点像编制的那种？

受访者： 他们也是走tenure的。

刘书博老师： 那他们研究整体是什么样的风格，是比较偏美式还是偏欧式的？

受访者： 我觉得···我不知道您定义的欧式是什么样子的，反正给我的一个感觉是，至少我的导师给我的指导和各方面就非常强调理论方面的。

刘书博老师：那就是欧式的

受访者：对对对，就不像美国他们，美国很多时候他们数据可能来源比较多、数据比较多，所以他们可能很多时候是围绕数据开始的。我们通常···我记得我导师每次给我改论文基本上重点部分都是在introduction和理论部分，他实证部分看的很少，就是给的建议都非常少，他的大部分建议都集中在introduction和理论部分。

刘书博老师： 对，那这样就是还是比较偏欧式

受访者： 然后我每次去开会也是这样一个感受。我就觉得欧洲好像是基本上比较强调理论，包括理论贡献啊等等。然后美国是数据很多，所以他们有时候做的实证的研究还比较fancy

刘书博老师： 对，他们数据非常多，然后工具又很精细。反正这样我在英国念，然后我看那些老师做的研究，数据分析就很基础的那种，但是好像他们理论创新比较多。

受访者：嗯，是的，我记得我导师直接就跟我讲过，他说他自己的实证的技术不是特别扎实

刘书博老师： 嗯，嗯，

受访者：所以他基本上都是看introduction和理论部分。

刘书博老师： 那您找工作当时选择是XXX，当时为什么选择这个学校

受访者： 说起来又是机缘巧合。因为我本身是江苏人嘛，然后本来我的目标范围都锁定在江浙沪，就是这一片。然后当时确实也拿到了几个offer。但是后来是因为我当时一个师兄也是从格罗宁根回来的，我当时是正好问了他一个关于签证的问题，我是交了论文以后先回来的，因为论文到答辩还有几个月的时间，所以我就先回来了，我想趁这段时间可以先找找工作看看，然后我当时准备再次去到荷兰答辩之前，我就问了一下我这边的这个师兄，他是正好在XXX工作。我就问了一下关于签证啊各方面的事情，然后他就问到了我找工作找的怎么样。我当时就说我手里有这样几个offer，他也不知道是开玩笑还是什么，就说可以来我们学校试试。然后我当时觉得倒是也可以，就当来北京旅游了，我当时是抱着这种非常随意的一个心情和心态来的。然后我就投了一下简历，结果就安排面试了。然后经过两轮面试，还算比较顺利，就也给了我offer。当时就是权衡了一下，跟手中的另外两个offer权衡了一下，觉得可能这个平台会好一些些，所以就当时只身一人就来到了北京，是这样一个过程。

刘书博老师： 当时在北京也没有看其他学校嘛，就直接就到XXX了嘛。

受访者： 对，真的一点都没有，就看了XXX。

刘书博老师： ok，那您在入职之后应该也有几年了

受访者： 现在第四年，快结束了

刘书博老师： 那学院当时跟您签的是tenure这种合同嘛，还是什么？

受访者： 对。我们学校当时就是对海归的博士，只允许签这一种，就是tenure，就是非升即走嘛，然后是一个六年的合同期。

刘书博老师： 嗯，他要求怎么样呢？我听说相当高。

受访者： 对，它那个每一年都变，然后我们那一届的要求是要一个UTD24的A，这只是发表上面的。当然还有其他的，比如说项目啊、课题啊之类的也有相关的要求，还有教学。

刘书博老师： 好吧，那您觉得日常工作这个压力还主要来自于科研吧。因为我觉得还是相当美国范式的。

受访者： 对，对对。其实我们这一届对海归还是各有各的要求，就是每个学院它还是有一定的discretion，就是我们商学院的要求是这样子，但是其他学院可能要个国际A，但不一定是UTD的，就是这样一个情况。

刘书博老师： ok，那现在您工作的环境怎么样？尤其是跟母校格罗宁根大学相比，您觉得比较有突出差异性的地方是什么呢？

受访者： 我还没细想过这个问题。因为我在荷兰主要还是以学生的身份，所以可能没有感受过究竟在那边就职是什么样一个感觉。所以这方面我还真没有说同行比较过。

刘书博老师： 是的，是的。那您就作为老师的话，跟之前自己的预期有没有不同？

受访者： 这个倒还好。唯一一点可能是···因为我毕竟也是海归回来，可能在语言上面有相应的一定的优势，就是我会以为在教学过程当中，可能会有双语教学或者类似这种、甚至是全英文教学的课程。但是后来发现没有，我目前的教学都是中文教学为主。这点我比较意外吧，反正目前就没有碰上。

可能后期···因为我看到有一些老师有开设这方面的课程，但是学院倒是没有强迫你哪门课程一定是要双语或者全英文教学的，所以就是大部分都是中文。

刘书博老师： 嗯，嗯，那在研究方面呢，您觉得学院对于科研的这种管理是不是还挺合理的？或者说不是合不合理，就是我看现在整个中国对于科研这一块儿好像在说要去五唯。那我不知道这个声音对于我们老师有没有影响啊，或者说您怎么看发表论文？尤其是您从欧洲那边然后回到中国，有没有觉得研究范式或者做研究的方法或者是内容有一些区别。

受访者： 嗯，我觉得不一定是我现在这个工作单位给我的感受啊。就是回来以后我的感受是好像国内的发展···我不知道刘老师你有这样的感受吗，就是国内做学术的感觉好像还是偏向美式。

我觉得就是好像还是比较注重数据和方法。然后我觉得可能XX（受访者的一位同事）也有这样同样的困惑哈，就是因为他做的研究也是比较定性的。我记得他那时候present的时候我是很喜欢他的研究，我觉得他做的很扎实。但是他在求职过程当中可能也碰过很多壁，我感觉就是因为研究范式比较偏向美式的这样一个整体的状态导致的。这也是我回来自己个人的一个感受。

所以当时其实我回来以后，我的博士论文后续发表也不是特别顺利，其实也是跟XX（受访者的一位同事）遇到的同样的一个处境，就是我们都比较偏定性或者偏理论方面的一个创新，而不是说方法和数据，用了大量的大数据啊或者什么，就工具做的特别好，或者是模型做的特别好，不是这样子的。所以就是确实这是我回来以后很大的一个感受。但我感觉倒不是说我现在所在的单位是这样子，我感觉大概整体的氛围是这样的。

刘书博老师： 嗯，嗯。像我们商学院，其实我觉得有一些老师会比较open minded，或者说他们甚至会偏向于质性研究，因为他们总是表达出来，说管理学不能够用自然科学的范式去靠。但另外有一些老师就特别的···认为任何事情都应该科学化。所以我们学院我会明显的感觉有两派，我不知道您所在的管理学系或者学院是不是也有类似这种不同的观点呢？

受访者： 嗯，目前看倒是没有。我的感觉是我们学院老师有一派行政事务比较多，然后另外一派是可能相对来说比较学术。我记得我们学院里面好像规定是有行政职务的老师，相对来说各方面考核的要求可能会稍微低一点。因为考虑到他们这些行政事务比较繁琐啊繁忙之类的。所以我反而是看到有这样两个分类。而不是像您刚刚说的这个。

然后做学术的可能大部分还是偏美式。因为我感觉我身边来自欧洲的同事可能相对来说比较少。很多要么是国内清北人，要么还有一些是北美回来的。所以相对来说大家做学术的这个范式还比较统一，比较偏美式一点点。当然如果跟像欧洲回来的同事聊起来的话可能还有一点共鸣。

刘书博老师： ok，那还真是跟我们商学院不一样，我们就还是可以看出来有范式的争论或者是对抗。我观察到的是年轻的老师会越来越统一，就像您所说的研究这块儿越来越美式，有这样的趋势。所以我不知道未来这个趋势会不会越来越一致，还是说会反而转向多样性，您怎么看待这个？

受访者： 哎，我是觉得如果按照制度理论的话，这个趋同是会越来越明显。因为如果这一个时期一直是这样的一个制度的话，那可能只会越来越趋同。除非我们说从制度上、上层建筑上面有一些改变，可能后面会有一些改变，我觉得是这样啊。因为至少看到后面来的这些老师基本上也都是···，而且他们要求也是越来越高。既然有这样一个政策导向、一个合同这样的规定、有这样一个压力在，我觉得可能还是会越来越趋同。这是我自己个人看法啊。

刘书博老师： 对，我观察到是这样。再加上我们现在高校的人事制度变革嘛，因为原来中国高校还是事业单位的铁饭碗，现在都在搞这个非升即走。那大家为了生存，迫于这个压力，就要接受美国的标准嘛。因为这样其实是可以先生存下来，但是生存下来之后其实大家都被一致化，那我们的发展其实也是同质化的发展。

Ok，那范老师您做的研究是哪个方向呀？

受访者： 我博士的时候主要还是在国际化这块，然后是中小企业为主的，中小企业的国际化。然后我研究的侧重点还是在高管这块儿。

刘书博老师： 我之前跟着一个团队在做，就是SME internationalization。然后又decision making，就是高阶理论里面那个决策。

受访者： 是吧？嗯，这块我还挺熟悉的，因为我硕士就该开始做高管的东西了。

刘书博老师： 那您的研究方法应该是以问卷为主吧？

受访者： 对，博士期间是问卷为主。然后硕士阶段其实也接受过这种二手数据实证研究的训练。所以基本上两者都有接触过。

刘书博老师： ok，我之前跟那个教授叫john，他就在这个领域。然后前几年发了一系列文章，他组了一个跨国的（团队），包括英国，包括埃及，包括中东，包括中国，然后还有好多个国家，都是他的徒子徒孙。然后就在不同国家收集中小型企业国际化决策的数据。

受访者： 那很有意思。

刘书博老师： 嗯，对对。那您现在做研究的范式是以量化实证为主了嘛

受访者： 嗯，对。

刘书博老师： ok，好的。我这边问题大概就问到这里，我看剩下的时间就交由两位博士生让他们来问，谢谢范老师。

吕梦娜： 范老师您好，我这边的主要关注点是关于非升即走这一块儿的，然后正好您也正在处于这样的一个阶段嘛。您原先说荷兰的学校也是这种制度，那就跟咱们国内高校的非升即走相比，您觉得有什么异同点吗？

受访者： 我觉得这个倒是差不了太多。因为就像刚刚跟刘老师探讨了，我没有在那边就职过，所以没有细细的去研究说他们的tenure track。但是据我了解的是，基本上也是一个合同期。一开始可能也是非升即走，然后可能到了副教授这个level就所谓的终身教授了，就可能不会有走的可能性了。

吕梦娜： 您提到您签的是一个六年的合同期吗？

受访者： 对

吕梦娜：我听说好像现在是3+3，就是三年要考核一次。

受访者： 对，会有个中期考核。

吕梦娜：哦，就是您现在是已经通过了第一个中期考核，然后再第二个考核期里面，对吧？

受访者：对对。

吕梦娜：那您现在已经是工作了四年了嘛。然后您现在还面临tenure的压力吗，是不是前面已经过去了？

受访者： 有。还有。因为我们的要求还挺高的，要发一篇UTD其实还是比较难的，所以这个压力一直在。就是尽可能的争取有一些发表，但是UTD就是要看缘分了，只能这么说。

吕梦娜： 您提到还有一个考核内容是关于课题方面，我听说申请课题也蛮难的。您这个要求达到了嘛

受访者：这个要求是这样的，因为它有旧制度和新制度，现在在轮换阶段，如果按照旧制度呢它是要求省部级一项，如果按照新制度呢一定要是国家级的，所以现在也是在轮换期的，就是看到底到时候符合哪个了。

吕梦娜： 了解。那我们国家的很多高校都在越来越普遍的推行这个所谓的非升即走，您觉得站在老师的角度来看的话，为什么会越来越热衷于这个制度呢？

受访者： 我觉得可能一方面是想把这个产出量化，或者是想给一些motivation吧，就是特别是对一些青年教师，然后另外一个可能也是想强调说科学和教研是分不开的，就是两者之间可能是相辅相成的一个关系，所以也希望大家重视科研这一块儿。目前可能感觉有趋势在越来越强调科研。虽然也在往回掰，有些政策也开始强调说不要唯论文，包括越来越强调教学在最后考核里面的一个权重。

所以我认为就是这两个方面的原因：一个是给动力。一个是希望大家重视一下科研。

然后从国家层面来说，可能也是希望越来越多的科研成果能够帮助到国家的发展吧。

吕梦娜： 那您认为非升即走在多大程度上能够实现刚刚提到的这两个目标。一个是提供动力呀，然后一个是提高对科研的重视。因为我观察的是，感觉有点矫枉过正了。就是动力反而变成压力，然后科研确实是重视，但是好像压过了教学那种感觉。

受访者： 对对，确实，哎呀这个也不好说，因为现在每个学校的做法不一样，我觉得因为科研的产出毕竟是需要时间和过程的，可能也要看做的研究到底是像这种产出会比较快的呢、还是产出可能会比较慢，就是每个人的状况都不一样。包括怎么平衡教学和科研，因为我们很多学校哈，可能青年教师进来以后就开始教学了。但也有一些院校，据我了解的是，比如说中山大学，可能对青年教师的要求是前三年还是前几年不用教学，所以你可能会有更多的时间把你手头上的这些科研成果发表出来，我觉得这倒也是一个很好的政策。

所以就是现在也确实没有一个统一的比较好的做法。因为每个青年教师面临的状况也不一样，包括接受的博士训练也不一样，但是回来以后又要面临同样的tenure的压力，然后包括面对的也是比较偏美式的研究范式。我也确实能理解这种难度哈、这种挑战性，但是我也确实说不好怎样统一一个标准或者政策能够让它变得更好，我现在个人也没有太多的建议。但是我觉得确实一个是要给予青年教师一定的科研上的支持，然后另外可能也要给予一定的时间，就是需要一些自由度。可能现在规定的比较死，比如说我了解到有一些个案，就是正好在合同结束的最后一年，他有一篇文章在国际A上面R&R，甚至第二轮的R&R，但即使这样的情况，他可能还要面临走的一个情况。所以我觉得可能需要一些灵活度，会更人性化一点点，这是我个人的一些想法。

吕梦娜： 嗯嗯了解。那您觉得我国高校能够越来越多的去推行非升即走，是具备了哪些条件？就我刚刚听到您说，从欧洲回来之后发现国内学者会对实证量化的范式会更加的偏爱一点，因为这个出成果也快嘛。我觉得可能这个是给推行非升即走提供了一个条件，因为他比较好量化产出嘛。

受访者： 对对

吕梦娜： 那您觉得还有哪些其他的方面会给非升即走的落地与推广提供条件呢？可以从学校啊、学院啊等各个方面都可以谈一谈。

受访者：我刚刚在上一个问题的时候也提到了几点。一个是可能要根据青年教师遇到的这些情况、背景啊···包括我记得之前申请那个国家基金委留学金的时候，也是有区别化的，比如说你去到欧洲，他给的奖学金是这样一个数目，然后去到美国，奖学金是另外一个数目。所以同样的，可能对于高校来说也可以通过识别这样的一个教育背景啊、留学背景啊等等，来做相应的一个规定。

包括在政策上面我觉得也可以有更多的灵活性，然后就是给青年教师更多的支持。我觉得这些学术上的支持其实很多高校都是能做到的。

吕梦娜： 就是您能够感受到您学校给的一些支持对嘛

受访者：嗯。我们前一段时间学院里面还组织了一次···，就是老师可以申请，是所谓的学术新星计划，相当于一部分的经费支持。但不是给到老师，是说同时让一些优秀的学生来，就是老师可以先把自己的研究项目先列出来，然后相当于竞标的方式。如果有学生对这个项目感兴趣，并通过资格筛查，最后被选为学术新星的话，就可以跟老师一起合作。然后呢这个经费也是支持到学生做这方面的学术工作的。所以我觉得这种形式其实也可以帮助到一些青年老师，特别是一些比如说目前课题或者各方面的项目基金还不是特别多的（青年老师）。如果学院能有一些这种打双引号的“小恩小惠”、能够扶持到大家的话，其实也有一些帮助的。

吕梦娜： 了解了，那这个话题先稍微告一段落。下面我想了解的是您在欧洲您的导师比较会偏重于理论方面，然后您回国之后也做了一些实证方面的研究。那到目前为止，您是怎么定义一个好的学术研究呢？

受访者： 嗯，我觉得好的学术研究，就特别规范的研究可能还是要从你观察到的现象开始。就是它跟你之前读到的很多该研究领域里面的结论是不一样的，如果这是一个新的现象，又是比较有趣的，又是用已有的研究或已有的理论无法解释的，然后带着这个问题再去建立你的研究问题、去找各方面的假设、支撑等等，再找数据啊去量化它、去test这些假设，我觉得这是对我来说是一个比较规范也是比较理想的研究。

吕梦娜： 您刚刚提到了现象方面的一个驱动，那您会在研究当中追求研究与实践的一个联系是吗？

受访者： 嗯，对。

吕梦娜： 哦，了解，其实我还很好奇您博士做高管方面的一些研究嘛，我很想知道您的这个数据来源是怎么获得的

受访者：我当时其实就是在荷兰做了一个survey，然后我当时也没有任何的network。所以其实我们就是相当于从一个数据库里面找了一些符合中小企业定义的这样一些企业，我真的就是直接把问卷发过去让他们填。然后可能也是我比较幸运，可能荷兰人民也比较友好，我这个回收率还挺高的，就是至少能够帮助我完成这样一个研究。所以是这样获得我的一手数据的。

吕梦娜： 明白，那后面您有跟这些高管做比如说深入的交流比如访谈啊之类的吗？

受访者：这个比较遗憾哈，因为荷兰人有自己的语言，虽然他们英语普及率很高，但是可能你要真正做深入交谈的话，还是用他们当地的母语会比较好。但是我又没有学习过荷兰语，所以在survey的环节当中没有实地的访谈。但是有通过电话确认过，也是在荷兰同事的帮助下，就是做了一些预调研呐，然后还有包括确认的回收啊等等，只是在这些环节上面有一些电话的沟通。但是没有真正实地去做一些访谈，这也是比较遗憾的一个地方。

吕梦娜：了解了解。然后还有一个小问题，我想问一下范老师，就是您现在作为一个年轻的老师或者学者，您对自己未来的职业生涯有什么要求？或者是有什么期待吗？或者说你想成为一个什么样的老师？什么样的学者？

受访者：这个问题还挺好的。怎么说呢？我希望自己的教学和科研都做的很不错，然后我的教学和科研能够相辅相成，能够帮助到彼此。然后我经常说教师也是一份良心活嘛，就是因为你面对的是学生，所以无论是在传输知识的过程当中，还是教导他们一起做科研的这个过程当中，我觉得就是要做到对得起自己的良心，我觉得这就是挺好的一件事情，对我来说就真的能把自己的真实所学能够交给他们，我就觉得好，我就觉得挺荣幸的。然后我也是希望我的学生能够胜过我，就是我能把他们引入到让他们自己能够有能力去探索更大的世界。这对我来说就是一件特别值得骄傲的事情。

吕梦娜： 对，感觉您对于就是传统的传道授业解惑，包括教书育人还是非常的很热衷的，我能感觉的出来。还有一个就是您后面过两年就马上就要结束这个“非升即走”了嘛。就是之前的阶段和后面您有想过会有什么明显的调整吗？

受访者： 目前看我只能说我先把手头的做好，然后后面的就交给命运吧。就到底是需要去转换一个地方或者说自己想要主动去到一个新的环境还是怎么样，就是看后面的一个发展。目前我只是把手头的工作都做好，我觉得就是我现在想要做的事情。因为谁都不不好说未来会发生什么。

吕梦娜： 归零的时代。对，好，谢谢范老师，我的问题先问到这。

刘书博老师： 我这边接着梦娜

插入一个问题，就是您觉得咱们这个专业性，就比如说个人特别具有职业化的这种表达，那您觉得professional，或者说学者、大学老师、管理学老师，这个专业度是什么构成的？这个问题可能有点抽象，或者换一个具体的问法，就您佩服的那些同行啊，或者role model有什么特点？

受访者： 我首先说教学部分吧，因为就是我自己的经历是刚入职就开始教学了，但是其实我当时觉得是非常欠缺的。因为我从来没有在实业界做过，虽然我没有遇到类似的问题，但是我记得一个非常资深的这个同事跟我聊过，他就是聊他的这个教学经历哈，他说他第一次给MBA上课的时候就有人挑战，那个学生就问：“某某老师你创过业吗？你就来给我们讲这些东西。”但是好在是这位老师确实创过业，这个挑战对他来说还好。但作为我们青年教师，其实我们经都没有经过，或者说在实业界干过，或者说自己有过创业的经历，我们就开始教这些管理学的知识，所以我自己感受其实是蛮欠缺的。所以对我来说我比较敬佩在教学方面做的比较好的老师，就是他确实有过实战经验，或者说他平时有各种各样的机会能够接触到各种各样的企业家，或者自己会去主动找一些机会，比如说去调研啊，或者是去了解各种各样的管理呀各方面。我觉得这个对我来说就是在教学方面的role model。

那科研方面就不用说了，就科研做的好的人实在是太多了。当然除了说发表特别强的，我特别佩服的就是他能够始终保持着，比如说我现在可能也做不到哈，每天都有这样的习惯，比如说能够每天都能看一些论文。每天保持着这样一个自己的活跃度和在这个领域里面的知识的这种活跃性、前瞻性各方面。我觉得这也是我比较佩服的，就是能够把学术融入到自己的这个生活习惯中的这些人，我觉得在科研方面是我的role model。

刘书博老师： ok，好的，谢谢。

黄秋莉： 范老师您好，根据您前面的介绍我觉得我学习到了很多，然后我自己也是列了一些的问题，可能跨度比较大。那我第一个问题就是想问您，因为您当初也是有好几个offer在手嘛，那您最后选择了对外经贸，您当时的说法是孤身一人来到北京，那您现在会觉得自己当初这个选择是一个正确的选择吗？

受访者： 目前看还不错。

因为虽然当时是只身一人哈，但现在也认识了很多新的朋友啊，新的同事，包括也是在北京组建自己的家庭。所以我觉得这个现在来看还是一个不错的决定。

黄秋莉：那在学术工作方面，就对外经贸而言，您觉得这个平台能够达到您当初的一个预期吗？

受访者： 因为我在博士阶段训练哈，就是欧洲训练可能跟刘老师的经历是比较相似的，就是我们是比较习惯于个人作业。所以我觉得这个科研方面有没有什么获得？我觉得最终可能还是要靠自己个人在这方面的努力嘛。所以我觉得这方面目前对我来说没有什么不好的，就是可能我本身也没有太多的期待，说平台会给我什么什么东西，所以可能目前我还没有特别不满的地方。

黄秋莉：之前听您说咱们学校的这个考核要求是每年都在变，我想了解一下它是个什么样的变化。您觉得是越来越高吗？还是说就是一些细节方面的变化呀？

受访者： 据我了解越来越高，我们后面的要求比我们要更高一些。 当然他是提供几个option，就是他会在面试的时候会给你提供几个offer。就是不同的薪酬匹配不同的要求，然后你可以自己去选，是这样一个情况。

黄秋莉：据您了解，大家是一个什么样的选择，会倾向于高的还是说是会倾向于这种压力比较小一点的？

受访者： 其实也是看你这个选项组的设计。比如说你这个选项里面都提到了必须要达到A，那可能大家的心态就会说反正都要达到A，就是都要有A的要求，那我就冲一冲，可能就会冲最高的那个。然后可能也有一些同事哈，他可能也会权衡一下自己这个未来的发表潜力，然后包括各方面的压力，那可能他会选择自己比较匹配的。但我觉得大多数人的心态可能也跟这个offer相关。就是如果你的offer是这样，就像我刚刚举的那个例子是这样一个安排的话，我觉得大部分人心态会觉得那反正都要有A，

我就冲一下最高的那个。如果确实差异很大，那可能我觉得大家就会更理性一点，

黄秋莉： 就是是一个性化定制的吗？

受访者： 不是个性化定制的，比如说就有三个，然后大家就从这三个里面选，但不是个性化定制。

黄秋莉： 就是我们学校也有这种师资博士后嘛，就是经过对他们的访谈，老师告诉我说我们学校的考核方式主要还是以发表论文为主嘛。那在咱们对外经贸的话，考核方式当中有这种倾向吗？

受访者： 我觉得确实权重最高的也还是发表。然后可能教授课程要达到多少多少学分，然后你的这个课题可能有一项两项的要求，是这样子的状况。但我觉得大部分还是看你的发表什么样的。

黄秋莉： 论文发表情况，您觉得大概能占到一个多大的比例呀？

受访者：我觉得可能80%吧。

黄秋莉： 这是这个差距比较大哈。那您觉得这种考核是合理的吗？因为之前听您介绍说您觉得这种考核是有一定的目的性在里面的，要激励科研呀等等，那您觉得这种考核方式对您个人来说是合理的吗？

受访者： 我觉得对科研的考核本身这件事情没有觉得不合理哈，但只是说规定一定要是UT，这种要求可能相对来说会偏高一些些。但我不知道中财是什么样的情况，咱们对青年教师的考核我不知道发表一定要达到什么样的要求。

刘书博老师： 我们没有那么高，我们UT太高了，我们可能就FT。

受访者： 所以我觉得相对来说是略略高了一些些。因为我觉得大家选择一个高校工作，他可能考虑的不是单纯说这个平台，他可能还会考虑未来这个学校的一些配套。所以就可能可能也要权衡一下学校本身就有没有相关的这种匹配，能够要求大家要达到这样的一个水平。我觉得规定的细节方面可能要做一些调整。

黄秋莉：对，就像您说的要一些灵活性嘛。那对这种评价方式来说，是我们要把论文发到期刊上去这样才算数嘛。然后之前也听别的老师说他们评副教授的时候是要把自己的学术成果发到国际上去进行同行评审。然后另外一个图灵奖的一个学者，他提到我们国家建立一个值得信赖的委员会是一个更好的一个评价方式。那您觉得除了这种在期刊上发表文章以外有更好的评价方式吗？还是您觉得这就是最好的。

受访者： 我只能说建立委员会的这个方式，怎么说呢？肯定也会有问题在，因为它相当于是唯一的一个权力机构了。那可想而知就是可能会造成权力滥用了，所以我觉得这也不是最好的方式，可能需要一些结合。包括你刚刚提到委员会，包括同行评审啊等等，我觉得可能需要多个方式去结合，来相互克服各自的缺点，也许是一条路吧，我也不太确定。

黄秋莉： 对，那咱们荷兰那边儿这种学术委员会比较强吗？因为我听有些欧洲的老师就说他们那边儿同行评审的这个力度就会大于说你在哪个期刊上发的论文。

受访者： 这个我还真不了解，但是我记得他们对publication肯定也是看的比较重的，我听到的好像跟国内的也是差不多，他们也是有规定说要达到怎样的发表才能继续留下来。

但是确实我突然想到一点就有可能会映射到你刚刚提到那个问题。我记得我当时有一个华人朋友，他在荷兰工作了好多年了，也是在那边就组建了自己家庭定居在那里了。然后他当时其实也是面临跟另外一个荷兰同事一起竞争，就是基本上条件相当，甚至他条件略好一点点。他们一起申请副教授这样一个职位，但是最终结果是这个荷兰同事留下来了，当中具体发生了什么我们可能都不得而知，但是你从这个结果来看，可能就有你刚刚说的那个问题啊。就是可能有这样一个委员会，他们起的作用可能会更大一些，因为可能考虑到是不是本地人这样一个情况，可能有一点这种歧视在里面。

黄秋莉： 之前有一个老师介绍说鹿特丹有一个商学院就是非常的走美国化的这种道路嘛，在荷兰有没有这种趋势呀？还是说就是欧洲的这种传统？

受访者： 我觉得我们学校可能还好。鹿特丹的话，我觉得因为这个商学院比较有名，所以他可能吸引到的各方面的人才啊，甚至就是包括组建的这些学术委员会啊各方面，他们都可能有来自比较多的北美的一些学者。有可能有有这样的趋势。但是我觉得我们学校还好。

黄秋莉：所以发展的很好才能走美国化道路？

受访者： 倒也不是发展不太好，可能就是我们院肯定没有鹿特丹商学院这么厉害哈。相对来说鹿特丹在商学院里面可能在全荷兰里面排名第一的。但是我们现在很多的老师，他们的发表各方面都很好。比如说有一个叫XXX，他就是的一个副主编，他做的非常好的，他做那个文化维度的研究，他们之间有很强的合作。所以他是管理系里面一个比较杰出的人才。所以也有一些这样的学者在，我觉得我们整个管理系也很不错。

黄秋莉：对对。那我们对外经贸要求很高啊，要发UT这种，那他为了吸引人才有哪些措施呢？

受访者： 目前看主要还是在报酬上面做区分。

黄秋莉： 咱们学校还是很有钱。

受访者： 我觉得应该也差不多吧，不是这样的吗？

刘书博老师： 因为我们之前有个同事的老婆之前就在您单位那边，后来去到英国了嘛。所以我们会聊这个，还是对外经贸的收入比中财高。

受访者： 对对，因为反正咱们是兄弟院校，经常放在一起讲，所以我总觉得是差不多的。

刘书博老师： 没有没有，我们是比较比较保守，然后很多事情就还是慢。而且我们人事制度改革，比如说预聘制啊可能也才开始没多久。

受访者：是只针对海归吗？还是说对国内的青年教师也是这样的？

刘书博老师： 现在好像都是了啊，从去年开始 就 统一了，对外经贸呢？

受访者：我们现在还是区分国内岗和所谓的海归岗。然后会针对比如说清北人，觉得国内比较优秀的这些院校毕业的博士生，他也会提供海归岗的这个offer，但是他们可以选到底是海归岗还是国内岗。但是海归回来的人只能选择海归岗。

刘书博老师： 那我们之前是这样，

我进来中财那会儿海归可以选择编制或者是合同制，然后我是最后一批可以选择的，然后我后面海归来就只能选预聘制了。然后去年开始国内国外都只能选预聘制，有这样的一个变化。

受访者： 我们是反过来是国内可以选，然后海归不能选。

刘书博老师：我后来有一些同事海归他们就选了那个合同制。然后我当年选我也是听我们那个老院长的建议，他说就直接选编制。

受访者： 我们国内院校毕业就是北大毕业的，他当时能够选嘛，他当时也咨询了他北大的这个博士生导师，他导师直接跟他说选国内的，都是这么建议的，哎呀，我们是没得选。

刘书博老师： 对对，那还是这个老人家的建议，还是一定要重点听一听。

黄秋莉： 没有我觉得很好奇为什么海归有这样的待遇。因为我从就是北大清华他们能够选择来看，似乎预聘长聘制是一个很好的条件或者是什么样子的。那我就不明白为什么对于海归来说还有这样一个限制，就不让你选，我觉得挺奇怪。

受访者： 我不知道，我不是政策制定者。我只是从个人猜测，他可能对海归有更多的期待，就是逼着你一定要有更多的产出。

刘书博老师： 但其实我觉得很奇怪的一点，荷兰博士期间不需要一定要发表多少多少SCI或者之类的。

受访者：荷兰的要求是你这个博士论文是要达到能够发表的水平，当然这个是由倒是来评判的。但是它没有硬性的说你一定要发表，有这样那样的发表你才能毕业，这不是毕业的要求。

刘书博老师： 那英国跟荷兰，回来以后就很吃亏。

受访者：对，这也是我们回来比较吃亏的地方。

黄秋莉： 我觉得是作为人才引进来的，结果制度上面要求去做一些事情。然后他视为差一点的，就是所谓的国内的一般的人才的话，还可以有好的选择。我觉得还挺那个啥的。

受访者： 可能他会对你有更多的期待，然后就希望你有更多的产出。

刘书博老师： 这个问题，我自己的一个经历：就是我作为海归来了之后，那我很多同事老师啊甚至不是管理者啊，他们就会说你海归回来，你就要发英文啊，你为什么还要发表中文的，那他们的一个直观的反应，就不假思索的这种说法，就是你海归就要发英文啊。

我听了之后我就觉得这个对人挺不尊重的，凭什么我回来就要按照你的这个要求来。所以我觉得有时候中国这个环境他有点压制或者说有点单一的标准。包括要求博士生必须发那么几本，我们学校学院都定的很死，就觉得没有必要这样子。然后欧洲那边我就觉得会比较人本一些，而不是说标准为本，还是以人为本嘛，所以这点我其实感受还是挺强烈的。所以回来之后我就一直很愤怒嘛，因为老是受到这种我觉得很无脑的一些要求或者评价，所以我就觉得国内国外的环境还是有清晰的差异。

受访者： 我记得我荷兰导师也是，他说确实他也实践过，就自己带的学生，他比如说强调先发表。因为也知道未来找工作可能很重要哈，他也实践过。但是他会发现最后实践的结果就是他的博士会拖得很长。因为肯定不是说随随便便发表一个就好了，也是想往了好的发表，所以这个周期可能就会很长。好的期刊一般周期可能也会很长，所以就会耽误到整个毕业的时间和流程，所以他后面也觉得不需要这样去做，他说如果能够看到有潜力能够达到发表要求就可以了。所以我觉得这是也是确实比较人性化的一点。

黄秋莉： 是，范老师，我们学院的这个非升即走是第几届了呀？您了解吗？

受访者： 我记得我上面应该是有两届左右。

黄秋莉： 如果没记错的话，像先前提到xxx老师嘛，她是离开对外经贸了，那咱们这个非升即走的这个过程当中，这种离开的人多吗？因为中山大学、武汉大学离开的人就很多。

受访者： 流动率是很大的。其实我听到的不只是我们学校，我觉得人大海归的流动率也很大，就是很多也可能是意识到自己可能达不到要求就主动跳了。然后其实很多后面达到要求的，他可能也会跳，反正流动性还挺大的。

黄秋莉： 反正感觉像中山呀，这个武大是被迫的就流动走了，因为可能就一个岗位，它招了七八个人这个样子。那咱学校老师不是这样哈？

受访者：对外经贸倒不是。

刘书博老师： 就是你说的那个叫博士后，跟那个“非升即走”是一样的。

就中大和武大他们是博士后的那种进来，然后就更加临时工的那种感觉。

受访者： 师资博士后也不是说一定会留下来？

刘书博老师： 我觉得像中山大学，可能百分之90以上都是要走掉的。所以他们那个名气现在非常的恶劣，已经臭名远扬了，所以现在博士毕业都不会去。

受访者： 那这个真的跟以前不一样，以前师资博士后不就相当于一个流动站，然后基本上是留下来的。

刘书博老师： 不是，他们就完全变了，就变成有点像大厂、公司的那种感觉，他就尽量的用人嘛，然后不断的流动。

黄秋莉： 那个武大校长说他们要“瘦身强体”，就是要更换自己的血液，让更优秀的人来，要把部分人就是换掉，就像换血一样。

刘书博老师： 就是像一个老妖婆或者老树妖的那种，他吸取年轻人的这个精华，然后维持自己长命，特别不道德。

受访者： 天呐，那确实很像大厂的一些做法，今天我也是学到很多。

刘书博老师： 对。所以现在我们看到有一些学校就比较公司化、市场化，那可能就短平快的。

受访者： 我们学校现在好像没有师资博士后，我没听到有多少，有听到说招博士后的，但是好像师资博士后还真没听说有。

我也正好了解一下私人的问题，就是中财是一定要先走师资博士后吗？

刘书博老师： 我们现在也没有规定死，但是像我们今年的状况是如果有五位新人进来，四个是师资博士后，只一个是给到讲师。

黄秋莉：之前我认识的前两年进来的两位老师也都是师资博士后。

受访者： 那咱们这个师资博士后是一个岗位就是一个吗？还是招很多个？

刘书博老师： 我们还好，我们学校就没有那么坏，师资博士来还是想让这个人留下，但是给这个当事人压力感比较大，因为有很多不确定性吧。

受访者： 确实。

黄秋莉：之前听到说，您现在的一个主要做法还是做自己想做的事情嘛。可能您在欧洲接受的训练也是说要发好的研究，那现在面对这个考核要求，也是要求您发一个比较高的这种。那您的做法就是要冲顶刊吗？

受访者： 其实这也是我个人现在比较矛盾的地方，就是因为从理性的选择来讲，冲顶刊你也知道概率是很小的。所以这时候你到底是接着冲顶刊还是怎么做？因为我也看到我上一届的有这样的情况，他一直在努力冲，但是现在已经在最后一年，然而他还在rnr的阶段，就是他有希望，但是也有不确定性。但是现在就是面临要走，而且可能在很多地方都碰壁，因为毕竟任何的产出都没有，对吧？所以这也会让我开始思考，说这个strategy到底是一直冲顶刊，还是选一个更简单的途径，这也是我目前比较矛盾的地方，你们有建议吗？

刘书博老师：  我觉得如果我让我发顶刊，我是百分之百不去发了，我觉得我肯定发不出来。像北大光华他们写了一本书叫《博雅光华》，然后如何在顶刊讲述中国故事。我看了之后我发现一个共性啊，就是他们都不是独立的啊，就是一定要抱着大牛的腿才能发出来。有时候我会想这个顶刊它就是一种很隐晦的权力的游戏嘛。你在那个圈子里面我觉得有机会，我不在那个圈子里， 我肯定没机会的。

受访者： 对，我跟很多人探讨过，我们都意识到有这样一个问题，所以你就会觉得希望其实很渺小。对，所以就开始反思，我到底还是这样坚持呢？还是说先把一些东西先产出来？

刘书博老师： 对，我觉得先产出自己的特色。

受访者： 对对对对，是的，是的。确实我也是在思考

 这个问题。

刘书博老师： 是的，而且我没有经历过范老师您这样一直有一个考核压力在未来等着。我想象一下，也就是如果总是有这种压力感的话，那会影响我在研究方面的选择。

受访者： 动作会变形。

黄秋莉： 是的，这个动作会变形，我觉得好形象啊，好有画面感。

受访者： 是的，真的是这样。

刘书博老师： 而且我们中国的整个大学，我们把它比作市场吧，那跟美国又不一样。美国他们有很大的流动市场，比如说在哈佛评不上那完全没有任何问题，完全可以到其他很好的学校，还有很多。

那我们这边就跟那边的土壤都不一样，整个市场结构也不一样。所以他又把那一套直接迁移过来。

受访者： 我也想起来荷兰院校，就是老师有论文在更好，但院校不过分追求。因为他有一部分是专门做teaching的老师。因为这些人teaching真的做的非常的好。就是我觉得其实这两类人才我们都需要，但是现在高校几乎没有这样的操作。我甚至了解到比较差的院校，也在追求科研发展。

刘书博老师： 甚至贵州财经这样的学校，他们考核标准比我们还要高，还要严苛。

黄秋莉： 他们还有职称级别退步的压力。

受访者： 因为我觉得每个人擅长的东西不一样，有些人他真的上课就是做的非常好，为什么不让他focus在这方面呢？我觉得这也是我比较困惑的地方，让人能够自由选择才好啊。

刘书博老师： 就我们中国的这个环境就是特别爱标准化，然后除了标准化之外，还特别爱把标准单一化。

受访者： 让我困惑的还有一点是，比如说其实青年教师面临很大的非升即走的压力。但是他们确实他们的任务其实是其实是非常重的。没错，反而是教授级别的人越来越不爱教课，越来越少教课，就是他们没有科研的压力，但是他们教课又不多，对，就是压力全在去年这种事情上。我觉得这也是反过来的。

刘书博老师： 对对，是这样。

受访者： 本身我觉得他们的experience应该是更rich，然后同时他们又没有科研的压力，所以他们其实应该要更多的teaching才对。我个人的一家之言哈，但是反而是让没有多少teaching experience的人一直在做，同时还有很大的科研压力，所以我觉得可能两方面都做不好。

刘书博老师： 所以这个也跟美国不一样嘛，因为美国人家就说让青年老师把时间腾出来专门做研究。我觉得 这个才是对的。 我们就是学了西方的那种标准，然后土壤又不改变，制度也不改变。所以就导致（学术职业吸引力下降），我们前面访谈清华大学的老师，然后他们说近三届博士毕业都选择不在学校了，就直接不再做学术了，因为觉得这个毫无希望。

受访者： 天呐。我也蛮佩服这些人的勇气，好酷，

黄秋莉： 感觉去企业界也挺挣钱的。听他们说清华博士去美团，然后年薪60万什么的。

受访者： 是的，我记得我当时选择读博的时候，我导师就跟我说过，就是想要挣钱的，就肯定不要待在高校。

刘书博老师： 对，但是现在待在高校校的人他们还是想挣钱。所以就把学术生产、学术探索变成一种工业生产，就让他最大化的变现。

受访者： 真的是这样。我身边也有朋友，他当然还没有到那个阶段，但是他说自己也是在努力，就是后面升到了教授之后，他就准备创业去了， 就准备去搞搞副业了。

黄秋莉： 发文章赚不了钱吗？

受访者： 然后确实也有很多同事，他现在就在外面讲课嘛，就可能比现在学校里面更挣钱。

刘书博老师： 对，确实有 很多这样的事情，在外面讲一天课能够赚一学期的这个课时费。

黄秋莉： 对呀。那我觉得我们学校现在还挺奇怪的，就是拿钱让你去发文章。但是就是好像发文章赚钱的性价比还没有出去讲课的性价比高的样子。

受访者：确实是这样。

黄秋莉：那范老师，刚刚听到您说这个就是让年轻老师去教课又科研嘛。然后让我想起了我们之前访谈的一位老师，他说年轻老师不仅仅面临这方面的压力，还有一个压力就是要做行政工作，就是也是老老师不愿意去做，然后就让年轻老师去干。

受访者： 我们确实也是这样，第一年进来的时候确实有很多各种各样乱七八糟的活儿。

黄秋莉： 我们年轻老师进来后要做党支部书记之类的。

受访者： 但是我们没有正式的title啊，我们就是有一些琐碎的事情会找到你去做，

黄秋莉： 没有行政人员去干吗？

受访者： 可能不一定是行政事务，就是比如说要开会的一些东西呀，反正很琐碎。包括一些要写什么材料啊什么之类的，可能都会组织。青年老师在干，因为资深的老师可能都不会搭理你嘛。

黄秋莉： 这个也不是科研的内容啊。不过我看我们对外经贸的官网上，学术成就还蛮丰富的，就《管理世界》呀，《经济研究》，还有AMJ呀，都还挺多的。感觉咱们对外经贸的同事们都好优秀呀，

受访者： 是。我也是很佩服他们。

确实也有一些同事各方面都很强，然后发表都非常好。

黄秋莉： 有些人就比较适合。有没有那种数量也很夸张的这种情况呀？因为我只能看到他们发了很顶级的，那具体老师发了多少篇我可能就看不到。

受访者： 据我了解，好像还没有说数量上特别夸张的，倒还好。

黄秋莉：看来咱们对质量要求挺高的。您在对外经贸也工作了五年了嘛，那您一路走来，这个学院层面有没有发生一些什么样的变化呀？

受访者： 学院层面吗？你是指各个方面吗？

黄秋莉： 各个方面，就是您觉得印象比较深的就可以。

受访者： 印象比较深的就是我们院领导换了。

黄秋莉：院领导换了，那是不是方向也不太一样了？

受访者： 目前就是新的领导的要求其实也是之前的院领导定下来的，现在还是维持。确实我也能理解啊，就是新的领导上来，他不可能说我一下就推翻前面。尽管知道可能大家压力很大，就比如说私下里面沟通也知道大家的压力都很大，但是从他的这个出发点，包括维持学院的发展什么之类的，他可能也无法一下子就推翻过去的这个做法。所以目前还是维持这种政策。管理上的变动，可能目前还好吧。

黄秋莉： 学术氛围是有没有竞争越来越激烈了呀。

受访者： 还好。但是我印象中我们之前的领导他比较强调学术这块儿啊。

刘书博老师： 你们学院文化咋样呀？那个大家各自为战呢？还是说就竞争比较强？

受访者： 因为我不在这个圈子里面哈，所以我的感触不太深。但是我略有耳闻吧，就是现在管理模式比较以党政这块为主，就是好像院长得听书记的， 大概是这样一个状况。然后肯定也应该有所谓的这种小圈子，但是因为我不在那里面，我离权力中心很远，里面很多奥妙我不太知道。

黄秋莉： 好的，范老师，我觉得我已经耽搁您很长时间了，我没有什么问题了，谢谢范老师。

受访者： 不会，我很高兴能帮上忙。

刘书博老师： 好，那谢谢范老师，我们今天那个访谈也超时了，特别不好意思。

受访者： 谢谢。没关系，没关系，希望能够帮助到大家。

刘书博老师： 有机会我们线下再聚，那我们今天先到这里，拜拜。

# 受访者12

刘书博老师：顾老师，我们应该有很多共同的话题。因为我研究生阶段在华威，然后后来又去到爱丁堡，我看您之前也是在英国很长时间了。

受访者： 对，我一开始去的UCL，然后换了一个专业，读了第二个硕士，到了布里斯托，然后再到LSE的。

刘书博老师： 哦，那也挺长时间了应该，而且在伦敦的时间可能是挺久了。

受访者： 对，我记得我当时刚去旅行的第一站就是爱丁堡，我就觉得那个城市特别漂亮，比伦敦好哈哈。

刘书博老师：对，伦敦我也住过一段时间，我那时候是在东区，那边就挺挺多黑人的，感觉很不一样

受访者： 那个传说中是比较乱一点，但是已经算是比较中心的了。

刘书博老师： 对，一眨眼好多年过去了，您是哪一年回国的

受访者： 我是13年回来的，然后14年入职的。

刘书博老师： 哦，那我差不多，我是15年入职的，然后14年大概就交了论文。我们差不多同一时间

受访者： 对对基本上同时，那以后欢迎有空的话到我们学校这边转一圈。

刘书博老师： 好呀，好呀。

受访者： 或者我们可以在外面约一下也都行

刘书博老师： 好呀，那太好了，也是缘分。

那今天晚上您看到咱们会议室还有两位，我们一块儿在做有关中国商学院、管理学的课题。那前面我们主要是访谈了中财商学院的一些老师同事。现在呢也希望能够更广泛地了解一下我们中国其他商学院的同事们。然后会有一些相关的问题，大概是分成三个方面。

第一个就是想要请教一下您在求学阶段对于这个专业怎么形成认识的。然后是工作之后，一方面是我们做研究，另一方面是在学校管理的方面。大概就这三个大的主题。那下面就是还是按照常规先请梦娜和秋莉简单地做一个自我介绍，也让顾老师认识大家。

吕梦娜：好的刘老师。顾老师您好，我是吕梦娜，现在是中财博士一年级，跟刘老师一样对咱们中国的商学院还有管理学特别感兴趣。今天很高兴能够访谈到您，特别是对您求学的经历、以及从事学术职业的状态特别好奇、很感兴趣。希望今晚能够跟您多多学习，多多交流。

受访者： 好的，梦娜，互相交流。

黄秋莉： 顾老师，晚上好，我是黄秋莉，然后我已经跟随刘老师做商学院相关的研究有一段时间了。今天晚上非常感谢您抽出很晚的时间来跟我们进行一个短时间内的交流。然后看了您在学院上面个人信息的介绍，我也是跟您一样，在本科的时候是学习的哲学专业，然后今天希望跟您有一些深入的交流，再次感谢您。

受访者： 好的，好的，我也很高兴能认识两位同学。今天晚上我本着知无不言、言无不尽的原则，你们有什么问题的话就提出来，我这边就回答。我希望就是我这边的发言能够对你们的这个研究有一些帮助吧。如果你们觉得我答的不是很好，或者是你们还想了解一些什么东西的话，你们就直言不讳的问我就好了。

刘书博老师：好，谢谢，谢谢顾老师。

受访者：嗯嗯好。

刘书博老师：好，那我先来问吧。我问完之后两位同学可能有一些补充问题。

那我首先就想要了解一下，我看到您本科学的是哲学，后来转到可能比较宏观一些的、跟工业管理相关的专业，然后现在是在工商管理这个大的方向。那当时您在学业选择的时候，是怎么做考虑的呢。就是决策时有什么思考点，为什么会选择哲学？然后后来为什么要转专业？

受访者： 因为我高考的时候嘛，那个时候的专业我没有办法选择。其实我当时第一专业选的是法学，但是由于这个法学分数不够，所以后来就是要调剂。那调剂呢当时我是武汉大学本科，武汉大学招生组就联系我，给我推荐一下专业。然后当时就告诉我哲学是他们学校比较强的，有个什么基地班、人才培养班，然后告诉我这个专业的一个发展去向，包括什么公务员啊之类的、体制内啊都非常的有帮助。而且我是1999年高考的，那个年代就是包括我的父母啊等等对于工作还没有太好的一个认知。那我父母觉得体制内还挺好的，所以他们也比较支持吧，所以就选···也不算是选啊，那是调剂到这个专业的。

然后其实我一开始刚进去的时候是比较失望，因为毕竟不是自己想学的嘛。1997年中国提出了依法治国，当时法学大热门，所以我们大家都愿意报这个专业，但是我是调剂的。那进去之后呢我也本着既来之则安之的一个原则吧，反正就沉下心来学习，成绩还行，所以呢后来也培养出了对于这个专业的一点兴趣。所以呢我当时就决定读研究生还是读哲学专业。

当时就是选择出国，还是选择了哲学专业，没有转专业。那我去读这个哲学的研究生，也就是我第一个研究生，读完了以后呢，其实我走过了一段弯路，因为我当时读的过程其实已经挺困难的了，我记得当时在学课程的时候有很多的课程，就是有很多的文章我是读不懂的，比如说一些原著，一句话有大概五六行、六七行，每个单词我都认识，但连起来我就不知道是什么意思。读的很痛苦，但是成绩还行。所以当时我也是有机会跟我的导师聊了一下读博士的事情，我也顺利留下了，我当时是顺利留在UCL可以读这个哲学博士，然后读了差不多半年时间吧。我当时记得在跟高年级的博士生见面的时候，高年级所有的博士生当中只有一个是中国人，他已经30多岁了、快40了。然后他当时是社科院的一个副教授，在那儿读这个哲学的博士，然后他就看到我特别年轻，20出头，他说“小伙子你还挺有勇气的”，然后他就跟我分享了他自己的经历，他说他已经是第七年了，还没有毕业。但是按我们那边的规矩，第八年不毕业就等于是fail掉了。所以他当时就觉得我挺有勇气的。但我当时觉得这条路可能就特别特别难。所以我走过的那段弯路是我当时就退学了，退学了我就临时又申了二专业。因为我特别想读博士，我们那个年代嘛就想要读博士。所以我就选择第二个专业，那选择第二个专业，为什么选工商管理呢？

第一个是因为我本科专业的限制，我是哲学专业，所以我统计数学也没有学的很好。所以我要转一个什么经济金融也不太可能。那我只能在偏社科里面转，那社科能选的专业就那么几个。法学我是没法转，因为我没有四年的法学背景，我再去读法学非常困难，所以这个我就否了。剩下的专业当中我觉得商科包括工商管理可能是一个比较实用性的一个学科吧，就等于是以后工作可能无论是业界还是学界都可能会稍微好一点。所以最后呢其实是一个比较实际的一个导向，我选择了工商管理。所以我第二个硕士读的是管理。

那后来到了博士阶段呢，我其实也是读管理，我当时读第二个硕士的时候，我对于人力资源、组织行为比较感兴趣，所以我申博士的时候就是人力资源方向的，包括我跟我导师、我当时去面试跟他们聊的内容。所以最后我拿的叫做劳动关系，其实就是人力资源方向的博士。

刘书博老师： 好。那您刚才提到就是在英国，工商管理它需要的学科背景不一定跟金融啊经济一样是要求有高数、统计，好像这个在中国就有点不一样啊。中国像咱们做工商管理，反正在中央财经大学最基础的课程都是要学习这相关的，微积分呀、统计呀这类课程。

而且我不知道您在学劳动关系的专业时，它的研究范式是不是跟国内的有一些不同？因为我在英国那边读的是战略，那边老师几乎百分之七八十的样子都是在做社会建构主义、就是质性研究。然后可能少部分是在做实证研究。您那个时候在读LSE的博士时，那当时的商学院或是经管系，是不是也是这样的状态呢？

受访者： 呃，我那个系就叫做industrial relations，就是劳动关系系，但底下呢会有什么人力资源啊等等。我刚进去的时候LSE做了一次合并，就是把战略、运筹、information system、还有我们那个系就是劳动关系、人力资源都给合并成为了一个叫做管理系department of management。就合并了这个系，这个系其实就是要对标其他的商学院的。因为那个年代不是商学院就火了嘛，就对标商学院，所以把这几个系都合并在一起了。

而且刚刚合并的时候呢，我们这边儿还是传统的英国科研模式，就跟您是一样的，就是质性研究、定性的东西做的非常好，所以我们那儿的老教授都是定性研究的。但是呢我们合并的那个新的管理系，他其实对标的是LBS，对标的是美国那儿的商学院，所以我们当时第一届系主任请的是牛津商学院的系主任，把他们的系主任好像是挖过来作为我们的第一任系主任，就等于是要对标美国。

后来又大量引进了很多美国的老师，就美国毕业的老师。所以后面呢我们就整个的管理系做了一个非常大的改革和调整，包括成立了OB，就是组织行为学。原来英国都没有这个东西的，后来就成立了OB系。所以就开始完全走美国商学院的那条路了。一度呢我们那些老师都不是很适应，包括我的老师，其实我老师后来退休了他也无所谓了，还有一些老教授也都退了，他们做定性的。而且当时LSE曾经发生过一个什么事儿呢？就是英国老师和美国老师似乎在公开场合有一些争执，就是当时有一个美国过来的PhD，他就是当老师的faculty，他说过一句话，他当英国老师的面说,“没有定量研究的研究怎么能叫科研呢？”就是特别冒犯，用特别轻蔑的语气说了那么一句话，后来双方都挺不开心的。所以那也是一个转型过程吧，后来我知道现在就是完全是美国的那套模式，等于是我们走过这样的一条路。

刘书博老师：那LSE还是在挺早的时候就开始转向美国，跟那LBS看齐了。那我们爱丁堡大学商学院现在似乎还是在英式的那条路上，那似乎我们看到在英国还是有这样的多样性。

受访者： 对，而且我觉得欧洲那边很多都是做定性的吧。

刘书博老师：对，尤其到北欧那几个国家就几乎更加是质性的研究。那这样的话，其实您受到这一套系统性的（学术训练）也就是对标美式的（学术训练），那回到国内之后应该也会比较顺利吧。因为中国现在的商学院就是在美式嘛，以光华为例。

受访者： 对

刘书博老师：OK,我当时的经历可能跟您就不一样。我当时找工作的时候跟国内的一些老师在交流，就感受到可能有这种范式的所谓的对立或者是有一些偏见吧。

Ok，好，那下一个问题就是您刚才说可能哲学那个领域有点晦涩，那管理学这个方面，您在接受这个博士学术训练的时候有没有一些困难呀，挑战呀，或者说有一些跟之前想象的完全不一样的地方。

受访者： 呃，困难和挑战，是吧？

刘书博老师： 对。

受访者： 呃，首先就是我从哲学转过来之后，我觉得所有的文章都没有那么难读。反正当时我就觉得还挺开心的能转到这个专业。然后困难和挑战主要是文字方面的吧，就是英语写作方面的、英语听说写字方面的还是没有那么好。我觉得这个是涉及到是在国外求学的过程了，可能跟专业还不太一样。

那专业的话因为我是转专业嘛，所以我的背景就决定了我当时刚转的时候还是挺困难的。我博士读了五年半，为什么读五年半呢？就是因为我的导师认为我没有很好的一个基础。所以我的第一年、第二年就等于是选修了很多很多的课，我几乎把他们本科生的那些该学的课都学了一遍，就等于是帮我打基础的那个阶段。然后逐渐的也慢慢上手了吧。所以那个一开始的过程当中还是挺焦虑、挺困难的。因为觉得说我自己好像浪费了很多时间，但后来想想也不算是浪费时间。其实也是一件好事，就把你自己的基础给打牢一点了。

这是我当时面临的困难吧。

刘书博老师： ok，那其实听起来从哲学转到管理学就有点降维打击，在语言方面好像稍微好一点。

那当时您博士毕业有没有想要留在英国啊，或者说那边有没有机会，还是说您当时就非常决然的要回到国内来工作。

受访者：首先呢，我当时如果想留在英国的话，我是没有办法去学术圈的。因为我当时的论文发表不是很好，我没有什么论文发表，所以我想做学术的话是比较困难的。但是要留在英国工作，就没有什么（困难），就是我当时有找到工作，一个是汇丰银行做HR。还有一个是人力资源方面的咨询，这两个我是可以的。我当时在读博士期间也比较迷惘吧，就是对于未来我也不知道我要不要做学术。所以我当时也尝试了一些intern，我在consulting firm做了一些intern，其实那边是可以留下的。但后来为什么回来？就是因为可能年纪大了，然后父母都在国内嘛，他们也希望我回来，而且我觉得我在那儿待的时间太久了，所以这是第一个原因。第二个原因是因为中国发展速度太快了，我觉得我在国外那几年好像错过了很多机会。所以因为这两个原因吧，最后就回来了。

刘书博老师： 嗯，嗯，所以听起来我们还是有挺多共同的想法，因为我当时也是类似这样子。那个时候您回国，就选择去高校做学术这样的一个专业性的工作嘛，还是说当时也有考虑说到行业、到企业

受访者： 行业也可以去。当时我可以去咨询公司，咨询公司的话是确定是可以去的。因为我有同学在咨询，然后我这边也有咨询的intern，就等于我当时也去尝试过这个。但后来我的想法是做学术也没有什么不好，然后就觉得说如果去了咨询，学术那条路就一定肯定就会断了，但如果你做了学术以后，你还是可以做咨询，就比如说你在职也可以做，所以就想自己能够把路走的宽一点的话，最后考虑了很久还是选择做学术吧。

刘书博老师：那现在再回过头看，您觉得这个选择怎么样？如果再选一次的话，是不是还会选择进入高校做学术？

受访者： 那再选一次可能不会选了吧，因为我觉得国内的高校确实跟国外的差别挺大的，跟我想象中的不太一样，然后自己的学术能力也没有那么的强。还有就是收入方面吧，收入方面也是跟业界差别比较大一点。等等几个方面吧。

刘书博老师： 提到说学术能力，那您怎么定义学术能力这样一个概念呢？就什么是高的学术能力。

受访者： 其实我在英国的时候我对学术能力的认知是发顶级期刊的论文，就是我觉得质量更重要，不是数量。但是回国之后呢，我发现起码得先有数量再去讲质量。所以国内你看很多老师的履历，拿出来非常吓人，在我看来非常吓人。就是很年轻的，可能跟我同龄的，一看都已经发了一百多篇文章了。就觉得我的学术能力跟别人相比的话差很多，就是这种感觉。

刘书博老师： 国内现在有挺多paper machine，而且我知道尤其像香港、新加坡地区好像更加的追求效率，就是他们产出会更加的impressive一些

受访者： 他们就是publication导向的，毕业一定得有这个。

刘书博老师： ok，而且国内现在商学院的博士培养也是在硬性规定必须得发。像我们学校博士就是两篇a，不知道您学校的博士毕业需要发几篇核心？

受访者： 我们好像是要是两篇还是三篇c刊就行了，我们没有到a。中财的a是什么概念啊。

刘书博老师： a应该就是南大核心，然后我们又挑出几本管理学的，就核心中的核心吧。也不是说所有的南大核心都算a，是我们自己又订立了一个大概七八本的样子

受访者： 我们这边对于a刊的定义是什么呢？我们分abc，中文a刊只有《管理世界》， b刊好像只有《南开管理评论》。其他的我不太清楚了。就是中文的期刊我知道这个，所以在我们学校听起来a就是特别可怕的一个东西，就很难发

刘书博老师： 是是，ok，我再回问一下，您博士毕业那个时候也是一个博士论文，然后通过答辩就可以毕业，对吧？

受访者： 对啊，我们当时就是博士论文毕业就行了，然后博士论文就是好像是外审吧，有外部的内部的。就直接通过答辩，没有任何的publication的要求。

刘书博老师： 对对。那您了解LSE的老师，比如说从年轻老师开始做起，有没有跟国内现在对于年轻老师要求的这种显著性的差异？是不是那边当时也是在数量为主？

受访者： 没有啊，完全没有数量。

我还要补充一点，就是我读博士期间不是有过那个定性定量冲突吗？我的导师是一个老英国人，然后他做的就是定性，后来我们系呢就给我配了一个二导。那个二导就是一个很年轻的美国毕业的PhD，他是一个美国人，然后他是负责定量的部分。我那个二导他没什么文章，他跟我说就这些文章根本就没有用，就是他发了都没用，所以他只发那么几本杂志的。

刘书博老师： 他说那些文章是什么？是UT达拉斯的那些期刊嘛。

受访者：LSE有个自己的list，FT50和UTD都在那个list里面，之外还有那么几本吧。就是他们只发那个list上的，而且要求也没有那么高。他们也是3年6年（考核）跟国内一样的这种制度了，你有就行了，然后我那个导师跟我说，他一点压力都没有，他想发AMJ，但是发不出来也无所谓，用的是GAP就行，在我们领域也是很好的期刊，虽然不是UTD但是FT50。然后这本期刊在那个北大被认为是a，就跟那个UTD是一个档次的。那我的导师只要发这个就行了，他说如果这些都发不了的话，英国还有那么两三本的industrial relations的那种journal，不是什么FT50。他说如果其他的（期刊）过不了，他在那上面发两篇也够了。而且那个对于他们来说很容易发。

刘书博老师：那这么听起来其实年轻老师生存下来压力也不是很大

受访者： 我觉得也是有压力，但是不像国内的这个制度一样，就是他们那儿确实是有走的。我刚刚跟您说的就是有个美国老师特别轻蔑的说定量才是研究的那个老师，他是美国一个特别大牛的professor的弟子，他来了以后就目中无人，但是他三年一篇文章都没发出来，所以他就没留下，他还是走了。

我那个二导的话，他有发，但是比较少。虽然比较少，也无所谓，也还是能留

刘书博老师： 明白，明白。所以那个生态还是跟国内有所不同。因为现在我了解到的像985的一些学校，好像对于年轻人的压榨还是挺大的。尤其是改成师资博士后以后，然后现在都在做这样的事情，招很多人，然后定的标准又特别高，然后最后可能有百分之一二留下来。

受访者： 就是特别不人性化的，真的是把人当牲口。

刘书博老师： 对对。而且我觉得公司这么搞，那他还有一个外部市场嘛，但是高校这么搞起来就其实挺糟糕的。把自己看做市场，然后其实高校就这么多坑，它又不像公司世界，市场还比较灵活一些。就搞得年轻人非常的被动。

受访者： 我觉得很多国外的东西到了中国之后就变质了，就比如说非升即走。那非升即走在国外，也不是说盲目的——只有一个坑，却招十个人，就不可能出现这种情况。但是在中国很多高校就可以这么搞，然后XX大学现在也这么搞，从2018年开始，就是2018年以后入职的老师全都是非升即走。

那现在呢还没有到六年嘛，他们是三年的中期评估，然后六年是整个的考评。然后我今天下午还遇到特别年轻的老师，他就是非常沮丧，那个老师也是国外回来的，好像是澳洲回来的，他原来是学分子生物学，回国之后就先去了业界，然后在业界觉得没有高校好，就又跑来我们学校了。然后他现在做大数据产业经济那方面的。

他现在就特别沮丧，因为我们学校评副教授的要求是五篇c刊或者以上加上一个国家级或省部级的项目。他好像论文已经够了，但是那个项目就申不上。我也是这样，我觉得项目我怎么改、怎么写都申不到。那个项目方面是很难解决的，所以他现在就是想说要不要提前找找后路啊什么的。所以今天我跟他聊，他跟我说了这个

刘书博老师： 那有点太死板了，非得要一个项目嘛，发论文不就行了吗？因为项目其实也是在促进论文

受访者： 我们这边就是非得有项目。所以从2018年开始实行这个政策之后，每年都有很多的名额就浪费了。比如说我们商学院有两个名额，然后今年没有达标的，那两个副教授的名额全部浪费。就我们现在是这样的一个状态。

刘书博老师：我觉得这个政策有点不合理

受访者：非常不合理

刘书博老师：那像这种政策是学校自己定的吗？

受访者： 这个政策就是学校拍板，就是那些领导拍脑袋定出来的。我记得当时2015-2017那几年的时候呢，我们学校其实科研还挺差的。然后学校科研处就找我们年轻老师开会，说第一个要申项目；第二个要发高一点的论文，特别是国际论文；第三个是要写教材。

当时我们年轻老师就表示说，我们能力方面可能没有那么强，不能够完成这样的一些要求等等，就跟他们沟通嘛。后来科研处就干脆开始实行这个政策，实行这个政策之后呢效果非常明显。就是你看什么国社项目发榜的时候，我们学校的那个总数都能够跟985夹在一起。所以他们就觉得“实行这样一个政策不是挺好的嘛，你看学校不是有这么好的一个成绩嘛”，所以他们在心里面对于这个事情特别坦然。

然后我现在就遇到了问题，因为我现在不管发多少论文，只要没有项目就评不上副教授。我去年下半年我就找人事处副处长去聊过一次，我就表达了我这边的一个想法。我说不管怎么说，我们老师希望以不同的方式来为这个学校做贡献。我们老师在做贡献的同时也希望能够跟学校一起成长，反正我说的已经非常的明确了。虽然这个话已经很委婉了，但已经非常明确了，但那个副处长就明确地跟我说，他说人事处也有这样的一个改革的想法，因为国家不是在破五唯嘛，但是他说有很多的阻力，包括人事处这边有想法之后就跟科研处沟通，科研处那边不想放弃得之不易的美好成果。所以就等于是还在扯皮吧

刘书博老师： 那它其实就变成比较指标导向的那种所谓的官僚主义心态吧，就是追求他的政绩嘛。

受访者： 对，是，而且我就觉得那个科研处的处长挺年轻的，他70后，但是他因为这个就是自己前途大好吧，因为他做科研处长实行了这样的一个政策之后，就有了一个非常好的、明确的改观。当然他不知道背后有多少人在说这个事情，他肯定也听不到，他也不愿意去听这个事情

刘书博老师： 他是什么背景啊？他是教学科研老师，然后做科研处处长呢，还是说就是纯行政做上去的。

受访者： 不是行政，他就是科研出生。他是XX学科那边的

刘书博老师： 明白，明白

受访者： 对，我们学校跟你们学校不一样的一点是，我们校领导都是XX学科那边的人。而XX学和这个经管类的学科差别也很大，你们学校肯定校领导很多都是那个经管类的人嘛。虽然经济和管理也有差距，但还是有很多相通的一些地方，包括论文的一些逻辑都非常的相似。

那我们这边有一些非常不合理的规定。比如说我们评博导，还要求有独著，就是不仅得发论文，还要有独著，只有一个作者的。在我们管理学领域实在是无法想象的事情。但是这个对于XX学，很多老师都独著，所以就是一刀切啊，XX学的老师定的规矩，所有的学科都得遵守

刘书博老师： 那这个真的挺霸道的，他不考虑学科的差异性。

受访者： 是，我觉得像人大那种稍微综合一点的学校，他们都已经把科研考核标准的制定权下放到学院了。像人大很多经管类的，你看他们的a刊，他们每个学院都有自己的一个list。

像我了解到上海交大都下放到学院，而且上海交大安泰学院每个系都有自己的一个名单，最后系的名单是汇总的，所以非常清晰。而且我觉得他们定出来的标准都非常跟国际接轨。我也看过首经贸的list，首经贸那边可能就稍微宽一点。不像上海交大他们那边，a就是a，a减就是a减，就那么几本杂志，首经贸稍微宽一点，但我觉得首经贸那个list也非常的符合经管类学科的逻辑。

刘书博老师： 嗯，嗯，是的。那你们也是因为XX学科太强势了，导致大家都得被他同质化标准

受访者： 对，这是我们学校最大的问题。

刘书博老师：好，顾老师，那我的问题就暂且问到这里，后面的时间就交给我们两位博士生，她们有一些不同维度的问题。

吕梦娜： 好的刘老师，顾老师您好，那我先问吧。

我其实很感兴趣的是您简历上有些当时在英国的时候是读了三个专业的硕士，包括您说也一定要读博士。就在那种还比较年轻的时候，是怎么有这样的一个心理的素质、心理的状态去做这个事情？而且我好奇有没有经济上的负担？

受访者： 呃，首先其实不是三个硕士，那个我所谓的第三个硕士是怎么回事儿呢？就是LSE读博士都有一个upgrade的过程。就是你作为一个学生，你要upgrade以后你才能够到一个所谓叫做PhD status，然后就是PhD的一个状态。所以我是通过了那个upgrade的环节之后，我们学校呢就会给我一个叫做master in research吧、就是那种研究型硕士的一个学位。就以防万一你如果不能毕业的话，你还能拿一个硕士走，就是这个意思啊。所以我不是读了三个硕士，这也是LSE的一个机制

然后问题是我当时有有有没有经济的压力是吧？首先呢这个经济压力我是没有的，我当时刚申请到博士的时候我是没有全奖的，那我当时跟我的父母也沟通了，我父母就是表示愿意支持。但是我到博士第三年我就申请到全奖了，就是前两年是免学费，就是把我的学费给免了，等于生活费什么的是我自理的。然后到了第三年我又申请了一些其他的奖学金，然后就有了全奖。这个是我读博士期间的一个花费的情况啊。

吕梦娜： 了解。那在那个过程当中，因为我们随着年龄在不断的增加嘛，会不会怀疑自己走的这条路不值得花费这么长的时间，有没有这种动摇？

受访者： 一直都很动摇

因为我当时就是非常明显的感觉到，我已经跟国内的同龄人脱节了。就是比如说我回国后跟我同龄人在聊的时候，他们在业界工作聊的东西、他们的人生阶段和我都完全不一样，都聊不出什么东西来。然后特别是我快30岁的时候，那个时候我爸妈也都快要退休了，然后我们家就等于是要青黄不接，就等于快没人挣钱的那种感觉。所以这个经济压力是在后期有的，而不是在前期，（经济压力）慢慢的有而且越来越大。

吕梦娜： 了解，那个时候您是怎么做心理建设的，然后支持您读下来的呢？

受访者：因为我也没有选择了，那个时候我已经读到中间了，怎么能quit

吕梦娜： 可以说前面的这个沉没成本有点大吗？

受访者： 对，所以现在有很多人问，如果让我再选会读博士吗？我都觉得很犹豫，就是我会觉得说我要是不读博士，我人生的路走的也不一定不好。

吕梦娜： 了解，那您接受完博士教育之后，您回国求职的时候有考虑过哪些因素？就是在选教职的时候有考虑过哪些因素。特别是对于城市的选择呀，还有学校的选择，主要考虑了哪些呢？

受访者： 我首先选的是城市。那为什么在政XX学校？就是因为北京的985不是北京人我是进不去的，然后什么理工什么北航，我当然了解了，我也跟他们接触了。然后那边的情况是他们的评价标准更加偏向于理工科，然后我觉得我这个学科进去之后，我发论文是发不赢他们的，所以那两个也没考虑。然后中财和外经贸我试都没试，因为我看到他们的招聘标准是要有已经发表的论文。所以我当时能选的就是211这些。

 但外地985我也联系了，有一些是有机会的，比如说厦门大学，南开大学，包括武汉大学都是有机会的，但我是首选城市北京。

吕梦娜： 对于北京有什么偏爱吗？

受访者： 因为我觉得北京是首都嘛，那资源肯定是更好。

而且我自己在伦敦也待了那么多年，我觉得大城市还是有资源方面的优势的，所以就来了。

吕梦娜： 那关于学校，就刚刚您说的是从985后面排除，然后后面选择了政XX，是吗？

受访者： 对，我先锁定城市，然后再去城市当中去选我能去的学校。

吕梦娜： 就是说那个时候XX大学其实是没有论文发表要求的，对吗？

受访者： 我刚进来的时候，评副教授的标准是一本专著或者是四篇c刊。但是我为什么那个时候没有评呢？是因为这待解决的人太多了，得排队。

吕梦娜： 所以说等那些人解决了之后，可能轮到您的时候条件就又上升了，对吗？

受访者：对，反正还没轮到我，我们学校的那个（政策）就改了。

吕梦娜： 那这个改的时候就是2018年，“非升即走”也是那个时候开始改的吗？

受访者： 对，2017年出的政策，2018年开始正式实行。

吕梦娜：了解。刚刚刘老师也问到说学术能力的界定问题，您在国内可能会受到一些冲击嘛，然后现在您也从事这个学术职业大概七八年了，那您觉得在哪些方面可以有所提升？或者说您觉得哪些方面如果提升的话，会让您觉得更加的开心，然后更加的适应呢？

受访者： 什么叫做提升？你说的是制度提升吗？还是我个人能力的提升？

吕梦娜： 制度方面，因为这肯定是制度环境已经发生了一些不太好的方面，因为大家都能感受得到嘛，然后想知道您是怎么看待的。比如说哪些制度或者是可以有所改善。

受访者： 就是纯粹是我个人的一个想法，是我个人感受。我觉得像XX大学、XX大学那么搞是非常不道德的一种行为。就是明明只有一个坑，非要招十个人，然后给他们期望，最后让他们厮杀的非常严重，然后把一批人给裁了，我觉得这是一个非常不合理的一件事情。

当然我不是说所有的“非升即走”都不合理，我觉得这种是不合理的。然后第二个，我们学校的“非升即走”也是不合理的。我们学校的“非升即走”的要求没有那么高，但是其实那个薪水是给的非常低的，就是我们的“非升即走”是没有年薪制的，他们来了以后就十几万吧，就这么一点钱，然后还给他们“非升即走”的一个压力。所以我觉得没有与物质相匹配的“非升即走”也是不道德的，这是第二点。

然后第三点，我觉得这种灌水论文的评价机制，我是不太适应的。我不知道刘老师有没有这个感觉，我觉得英国整个国家还是一个比较relax的一个国家，就是好像大家的节奏都没有那么快，所以大家都过得比较好，我是觉得美国回来的就……

刘书博老师： 对，顾老师说的感受我特别赞同。我是觉得他们欧洲还是比较人本，那美国可能会比较工业。就工业化之后，可能大家会从对于人的关注转向了对于效率的提升。我们本来发明工业标准是为了人，结果人反倒是被工业的标准给挟持了。我觉得美国可能更那样一些，那中国甚至要比美国更加那样，美国可能还有这个work life balance，那我们这边就完全没有了。

受访者： 我觉得英国那块是非常的relax的，然后美国是要比英国稍微的拼一点。但是中国这边确实太卷了，就是太卷了。然后我觉得这个评价标准我不能适应，因为在我读博士期间接受的教育都告诉我们说要发好的期刊，而不是盲目的追求数量。但现在国内我身边的老师都告诉我说你得先有数量，然后再去谈质量，所以这个和我的认知不太一样。

吕梦娜： 了解，那刚刚您也提到就是并不是所有的“非升即走”都是不好的嘛，那您觉得就是“非升即走”好的地方或者合理性的地方体现在哪里呢？

受访者： 我觉得“非升即走”好的地方是首先你要有一定的配套吧？就是无论是从薪水方面还是各个方面，你得有一定的经济物质方面的保，这是第一点。

第二点是你的制度上得有配套吧，我觉得有些学校他们实行的比较好的点是在于，比如说给年轻的老师更多做学问的时间，你不要搞那么多的课去上，你不要搞那么多的行政活动或者是工作啊等等，你得有制度的一个配套，这是第二点。

第三点是我觉得你得有个合理的评价机制吧，就是你不能规定死了说我得要1个项目和5篇论文，我觉得这种评价就是有点太机械了。而且你怎么能确定每个人的长处都是一样的呢？所以我觉得这个也是不太合理的。所以如果一个学校就像我们学校一样，既没有物质保证，又让老师有很多的课要上，然后还得有一个定死的压力，然后去实行“非升即走”，我觉得这就是一种耍流氓的行为。

吕梦娜： 了解。那您觉得“非升即走”，如果说他能把这些配套的措施都有所提升的话，那它本质上还算是一个好的，是吗？因为比如有人会说它可以起到一定的激励作用，让大家能够活动起来，就是不要躺平。

受访者： 我不觉得“非升即走”一定是个坏事，因为各行各业都会有竞争的问题。而且我觉得很多你像欧美的学校在实行“非升即走”的时候，他们其实也有很多非常人性化的东西在里面。就比如说XX（英国某大学）他们那边对于老师的“非升即走”是会有一种人性化的措施。比如说你怀孕了什么的，他们会有一些放宽的，他们并不是说一刀切了。

吕梦娜： 我们这边好像是没有听说过。

受访者： 比如对于女老师和女博士，因为我们本来是四年要毕业，但是女博士可以放宽到八年，就是因为考虑到你要生孩子。跟我一起入职的有一个女博士，她就是生孩子，而且生两个，她是八年毕业的，所以就是很人性化。我觉得他们那边的“非升即走”也很人性化，对于老师也有一个延期，就是不会给你规定那么死。

吕梦娜： 行。然后下一个问题就是，您在英国可以说是见证了这种范式的一种冲突嘛，就是您刚刚举的那个美国人和英国人的例子。那对自身来说有没有一定的挣扎呢？对于定性和定量这两种方式。

受访者： 我倒是没有，而且我现在是广泛的学习。因为我当时毕业论文是既有定量的，也有定性的。当然后来我意识到了就是你既有定量又有定性的，那这两个做的都不深。后来我回国之后，我现在主要做的还是定量方面的东西比较多一点，但是我还是特别愿意去学定性的一些东西啊。x老师也在我们这儿，然后她是研究定性的，而且当时她来招聘的时候，那个我们系主任去不了，然后他让我代他去。然后我当时知道她研究定性的，我就特别想把她招进来，所以我当时也是跟系主任说了很多。然后我说我们系没有专门研究定性的老师，我说招进来的话就等于给我们学院的学术增添了一些新的东西。她现在给我们学生讲定性的研究方法，我觉得也是非常好的啊。所以我现在是两个方面都想尝试，我没有说我不想做哪个。

吕梦娜： 了解。您提到科研处可能会有一些比如说制定规则的人，但是他们后面其实并不被这个制度所约束的嘛。然后他们就是希望能够提升这个学校的科研的数量或者说科研的能力，但是人事处这边因为开始“破五唯”，好像也是希望能够做出一些探索。就是我听起来好像这两个处，科研处和人事处之间并不平衡？是竞争的部门吗？我不知道您了不了解。

受访者： 我觉得学校里面的部门都有自己的利益，我为什么这么说呢？

就是科研处有自己的利益，因为最后年终总结的时候，科研处一定是拿数据来说话的，所以他们有自己的利益。而人事处也有自己的坚持，因为现在教育部的导向是“破五唯”，他们也要考虑到这个问题，那教务处也有自己的一个利益。那教务处利益是什么呢？其实有很多跟教务处有关系的东西，比如说什么青年老师大赛、教学方面的项目、教改项目什么的，这些现在都不在我们的科研考核范围当中。如果哪一天这些东西都能够算到科研考核里面去，那教务处的重要性是不是就更高了？所以我觉得他们都是自己有一个小算盘要打，倒不是一个竞争的关系。

我觉得他们各有利益吧，所以保证自己的利益前提之下会有更多的诉求。然后我们这儿还有一个老师，他也是国外回来的，他也是没有申请到项目，然后他也是副教授没解决。他去年就去找那个科研处的老师去聊这个事情，然后他就跟科研处的老师说，他说都已经“破五唯”，科研处为什么还要再坚持这个项目呢？然后那个老师说我们这儿不会变的。科研处老师就直接跟他说：“我们这儿不会变的”。后来我们那个老师就很生气，就提了一句说难道你们不怕我们这儿有老师告到教育部去吗？然后那个老师就很有挑衅的说了一句，说“你们去告吧，我们不怕。”反正我觉得就沟通特别的不好啊。

刘书博老师： 我是觉得这样子挺典型的，因为学校老师挺弱势的，这个行政部门挺强势。

吕梦娜： 好的，顾老师，谢谢您，我这边的问题到这里了。

黄秋莉：顾老师，您好，我想问一下您现在工作的主要内容包括哪些呢？

受访者： 我现在主要是三块儿，一块儿是自己的科研，第二块儿是教学，第三块儿是这个行政。我现在是这个工商管理系的副主任。为什么我现在有行政职务呢？是因为我们学院的老教授们都不想做事了，然后他们就把年轻人都提上来做这个行政，就等于我们这边很多的像什么副主任啊之类的都是很年轻的老师，所以基本上就这三块儿我都得去弄。

黄秋莉： 那您自己对这个行政工作是一个什么样的态度呢？感觉好像是不得不做的这么一个状态。

受访者： 我是觉得是一种不得不做的状态，其实我内心是不想做的。就是我自己为什么觉得国内和我的想象不太一样呢？就是我自己觉得做科研老师是更加自由一点，然后更加随心一点，就是我可以有更多的时间去做我自己的那个科研，而不是说被很多的琐事牵绊着。

那我不做行政的话也不行。因为学院的领导他觉得他器重你才希望你去做，那你不做怎么在这个学院待下去呢？所以也是考虑到这些方面的种种因素，我不得不去做这个事情。

黄秋莉： 那您在做这个事情的过程当中是不是会对别的比如说科研啊、教学啊带来一些影响呢？

受访者： 当然，就占了很多的时间。所以我就觉得，唉，我现在也在做减法吧，就有很多事情我就尽量不做了啊，然后教学的话我也不会去教那么多的课了。就是我现在就是满足最基本的一个工作量就可以了，我就不会再去多上课了，那更多的时间我想放在科研上。因为时间是一定的，而且随着年纪增长，家里面的事只会越来越多，那肯定要占用你的时间。

黄秋莉： 然后以前我觉得就是做老师嘛，可能更多的时候就是做自己的研究。但是看就是可能听您介绍以后嘛，我们会面临很多方面的，比如是来自领导方面的隐性要求呀，就还有像这种考核方面的这种指标的硬性要求。那除此之外，您有没有觉得在自己做学术工作过程中有一些来自别的方面的要求吗？就可能我自己想的一个，就像社会上可能会觉得这个老师要传道授业解惑呀，或者是这个国家呀，也希望我们知识分子发挥一定的作用。那您自己对学术工作是一个什么样的理解呢？

受访者： 我想这个问题有点大。你说的这个问题，我忽然想到了一点，就是为什么我项目申请不到？因为我之前做的很多的东西吧，它都是所谓的有一点阴暗面的一些东西，比如说“组织的阴暗面”等等这些问题。那拿这种题目去申请科研项目那一定是申请不到的。那我们国内这边很多的科研项目都是非常接地气的一些问题，特别现在国家都在倡导说要把论文写在祖国的大地上，要把论文跟那个实实践相结合。所以我们学院申请到的老师无外乎就是什么就业、养老这些特别实际的一些问题。

刘书博老师： 顾老师您说的我听起来也没有说不接地气呀，那您可能是比较社会这种负面的问题去研究。

受访者： 其实我当时研究的是什么呢？我研究的是人力资源管理，就是中国的企业会有自己的文化，那很多的文化它是比较有中国特色的，比如说关系。那关系对人力资源管理又有什么样的一个影响呢？可能更多的是一种负面的一种影响。那这种是所谓的我们组织阴暗面的一个研究，这是没有办法去申请。

刘书博老师： 似乎我们应该立一个比较正向正能量的，这样子会比较讨喜。

受访者： 对，你看我刚刚有回答你问题吗？

黄秋莉： 就感觉是中国学术界的一种隐性的标准吧。然后想接着“非升即走”的问题。先前您也提到，就是您会觉得这种对数量的这种过分的强调会导致一些灌水的行为。那您身边的老师也会告诉您说您需要先有数量，然后再有质量。那您自己在面对现在这种考核的时候，您具体的做法是什么样子呢？

受访者： 我原来是不追求数量的。我现在是都追求，就是我既要有好的又要有普通的，我现在就是两手抓的策略。

黄秋莉： 我就觉得好像当您养成一个学术方面的taste以后，就再做一些您所谓的普通的学术研究的时候，我会觉得心里还会有一点不太认可的感觉。

受访者： 我倒没有什么不认可。就是我回国那么多年了，我自己一直都是希望能够更加接地气一点吧。因为我现在生活在中国，所以我要更加跟中国的实际相结合，得接地气。然后我现在做过的尝试：

 第一个，我要追求数量，那我就不得不发一些普通的，我不再追求都是好的那些期刊。第二个，我要开始发中文的东西。因为之前我是觉得我自己没有能力发中文的，因为我也不太会写，我也不知道这边的模式是怎样。那我现在也开始做这个方面的工作，就是发中文的期刊，所以我就是想说都能够试一试吧。

黄秋莉： 也是一个多方面尝试的一个过程。

那您对自己未来职业的发展的话有什么样的期待呢？假如等您评上教授了，就是一切的职称都评上了之后，您会怎么样对自己的职业进行规划呢？

受访者： 其实我还是想首先评上教授，这个是肯定不会变的一个追求。

其实我还是想好好的做一些学术的，就是我希望我成为教授之后，我还是能够做学术，就像我之前导师一样，但是实际上我觉得不太可能就是完全不再做一些其他的社会事务，因为我觉得这个经济方面的压力会让我去做一些其他的事情。

黄秋莉： 然后我想再回到先前那个问题，就是您对自己写的论文，您对他的这个质量呀什么的有什么要求吗？就是您会渴望发挥一些社会影响力吗？

受访者： 我当然是希望啊。我现在还有转变，就是我现在也在做一些接地气的一些东西，就比如说我们这边有老师研究就业，然后我现在也开始研究就业这个领域的东西。就是我跟那个老师说我想做一些就业方面的一些东西，那他非常欢迎，因为我就可以跟他一起做，成为他团队的成员。就等于是我现在也想做一些能够为我们国家的发展提供一些不管是参考啊还是建议呀各个方面的一些科研。

黄秋莉： 然后我想问一下，就是您作为咱们系的这个副主任嘛。就是有没有可能为其他的教职工谋取一些福利呀？就是能不能做到这种地步？

受访者： 我是想的，但我没有这个能力。我们后天周四上午，人事处会有领导来我们学院，跟我们学院的老师座谈。然后这次按我个人的理解应该是为一些改革铺路的，因为人事处在推这个人事改革的时候，就不说是所有人都认同，那起码得有绝大多数的院系还有部门都得认同或者是赞同。那科研处那边不认同，那这就可能会给让我们发出一点声音，那我们系主任当时就跟我说，让我去代表系里面说话。

 然后我的第一反应是，就是我们学校现在能不能用一个A刊来代替一个所谓的科研项目。但是我们知道A刊也挺难的，就是我说的《管理世界》。那我自己是已经有A刊了，但我发的是英文，我发的那个英文被认定为A刊，那我自己是有A刊了。但是我考虑到我们这儿有一些年轻老师不一定会有A刊，而且他们直接面临“非升即走”。

 如果学校真的说我们可以用一篇A刊来代一个科研项目，他们最后可能还是不够啊。所以我当时还跟我们系主任沟通，说我能不能就是提这样的一些想法，就是比如说两个B也能换一个科研项目之类的。那我们那个系主任，反正他也没有说反对，也没有说支持吧。他说你可以表达你自己的意见，但是最后都是那个科研处那边来定这些事情。我有这样的想法，但是我没有这个能力呀，因为我人微言轻的，我也只能就是提一些意见。

黄秋莉：咱们也只能发出自己的一个声音吧。不过“星星之火，可以燎原。”刘老师经常这样跟我们说。

受访者： 我希望你们的科研真的能够对这个中国的科研体系的改革能够起到一定促进和推动的作用。

刘书博老师： 对，我们这股气一直在憋着。

受访者： 是的，希望有好的结果。

刘书博老师： 是的，是的。

黄秋莉： 然后顾老师，老师要评职称，学校都要求我们去申请国家课题呀，然后发论文。这个是不是跟咱们整个学校的一个评估是有关系的？我就一直不太理解，我自己比较理想的想法就是一个学校嘛，可能教书育人、打造自己的特色会更重要一些，那为什么从整个学校的层面来看，它会非常强调老师申请科研基金呢？

受访者： 是的，有教育部的那个评估，还有各种评估，因为我之前也填过一些表，那个里面就有我记得我上一次填的是硕士点的评估。各种评估，太多了，这也是国内特别让人烦的一件事情。你看有学科评估，教学评估又分成本科评估、硕士评估，这是教学方面的，还有什么硕士点评估、博士点评估。很多评估，我们每年都在写各种材料，然后这个材料就是包括项目和论文。基本上我看到比较能够让老师发挥作用就这两个，一个是项目，一个是论文，你有的话你就把它列上去。所以为什么每个高校都开始要求，就是因为这个原因。那如果哪天教育部说我不认，我不看这个了，那学校肯定不会要你有这个的。

黄秋莉： 我感觉老师可能还是成为了一个要为学校的目的去服务的这种感觉，

受访者： 对，老师就是工具。

刘书博老师： 这是我们中国高校治理的一个特色嘛。然后指标化治理，然后以评促改呀，各种评比、各种比赛、各种排名啊。其实现在国外像英美也特别明显，像英国也有一个类似的评估系统，由国家在主导。他们也有一个教育部吧，然后也搞得非常KPI导向。所以就想到那本书《指标的暴政》，其实说的就是这个事情。

黄秋莉： 顾老师，目前咱们也不是提倡要建设双一流学校和学科。您对这个事情了解吗？然后什么样的看法吗？就是想了解一下您的看法。您觉得我们为什么要去做这个事情，然后做这个事情是合理的吗？我就有的时候会有这种困惑。

受访者： 从我个人的角度来看，我觉得是不合理的。你看最早的什么211，最后搞成了什么地区平衡，有一个什么新疆大学、云南大学不都是211。

然后后来的985，其实211还没那么伤害，985是最伤害的，985伤害的是谁呀？就是你们xx学校和我们xx学校这种211。就是跟人家比起来就是天壤之别了，当年只有211的时候大家都是211，后来有了所谓的985之后，像我们这种211就受到了非常大的影响。所以我觉得211的学校这种专业化的学校不会喜欢985这个东西。

双一流，其实我觉得这个也跟985差不多吧，双一流可能就是意识到了985的问题，985就那么三十多所，意识到985一下子把这个学校差距拉开了，然后就搞了一个所谓的双一流。但是双一流我也不知道这么搞下去对整个的教育是什么样的帮助，我也不太明白。

黄秋莉： 好的。然后就是想请教一下顾老师，因为我们可能以后也会走上学术道路嘛，就想着您工作了很长时间，就想您以这种过来人谈谈自己的经验也好，就是给我们的建议也好。

受访者： 我觉得第一点是，我觉得做科研还是得有一些本心的，就是你得有自己的一个信念，你得真正的对这个事情感兴趣。所以我觉得如果你没有兴趣的话就不要勉强，因为这条路不是很好走，如果你连兴趣都没有，那你之后会走的很辛苦啊，所以这是第一点。

第二点，就是既然我们在这样的一个体制之下，那如果你想要这条路走的更顺遂的话，还是得要符合这个体制下的一个游戏规则。所以就是该有的都还有都得有。我发现像我们这儿毕业过来的国内的一些年轻老师，他们的论文发的都挺好的，就是数量方面，我们不说质量。因为刚刚毕业质量很好的话，那还是比较少，但是数量方面能保证。所以我觉得起码他们是遵守着游戏规则，在玩这个游戏的，这是第二点。

第三点，我觉得作为科研工作者，你得有你自己的内心的一方净土吧。我觉得这方净土可能是留给你自己的一个科研兴趣的，或者是你自己喜欢的一个科研方向的，或者是你喜欢的一些研究内容的。我觉得要留出这么一个空空地来。那你找到一个你感兴趣的点去做研究，并且持之以恒，我觉得未来在这个方面肯定是会有成就的。我就提这3点吧。

黄秋莉： 好的，谢谢顾老师。然后我想问一下，就是顾老师有没有在身边发现受到这种考核体系伤害的老师的现象。想听一听他们的故事，可能也是对我未来这个学术道路的一个警醒。

受访者： 我们系除了我以外还有两个英国回来老师，有一个是拉夫堡的，还有一个是哪个学校我忘记了，然后这两个老师都是女老师。然后拉夫堡回来的那个女老师她读的是数学专业。然后她从小学就在英国，小学、初中、高中都在英国读，然后读完博士回来的。然后这个老师在入职的第五年就辞职了，就走了。因为她已经完全不能够适应国内的这种体制了，所以她就走了。

另外一个女老师，她还没走，但是我觉得她非常非常痛苦，因为她刚进来的时候是一个很漂亮、很阳光的一个女孩儿，她后来到了什么程度呢？就是脱发，掉很多头发，然后一度身体素质下降到了什么程度呢？就是脸上长了很多的油什么的，还去打激光把它打掉，反正她的压力特别特别大。

然后我就问他，我说你为什么不能像另外那个女老师一样就走呢？然后她说是因为她的父母必须要让他在这个工作去工作。这个女老师也是大概初中左右就去英国了吧，也很早，所以她现在完全不适应。而这个女老师她比较惨的是她还面临着“非升即走”，就是她是2018年之后进来的，然后我就劝她，我说你放松一点吧，我说这个工作不是人生的全部，你的身体才是最重要的。然后她说对反正父母不让她走，然后就是如果“非升即走”留不下的话，那就顺利顺理成章的走了。这两个是跟我离得比较近，然后我觉得他们受到了国内一个科研体制的一个摧残，我觉得他们的人生不应该过得那么那么艰难。

黄秋莉： 确实是，都是很优秀的人啊。然后顾老师身边有没有那种能够适应游戏规则的同时，就像老师刚刚说的，然后也能够做到保持心中的那种净土。就是他不仅仅能够生存还能够发展的那种人，有吗？

受访者： 我觉得这种人还挺多的。我们有一个那个北大光华毕业的一个博士，然后她当时来我们这儿应聘，我们是没有要她的。然后后来她选择来我们这儿做那个博后。我觉得她的适应能力非常强，也是个女老师，她适应能力非常强，而且她的学术功底也都非常扎实。所以我觉得你们读博士期间你们想做学术的话，就学术功底一定要打扎实，我觉得她就挺好的，而且她是一个非常乐观的一个女孩儿。

黄秋莉： 好的，好的，谢谢顾老师跟我们分享了那么多经验，然后我暂时没有问题了，顾老师，刘老师。

刘书博老师： 好的。那我觉得时间也差不多了。今天晚上我觉得聊的也很开心，因为顾老师也真的是非常敞开胸怀的跟我们分享他的经历和看法，尤其是对两位博士生来说给了非常好的意见。我也希望在未来我们有机会可以线下再深入的交流。

受访者： 好的，那刘老师，咱们今晚的就仅限于学术交流，就是不涉及到其他的一些言论啊。

刘书博老师： 明白。我们都会进行这个脱敏，然后就我们做质性研究，会特别注意这个事情，来保护我们这个被访者的隐私。所以到时候我们会整理出来一个文字稿，然后发给您看，您可以把一些内容给删除。好的，顾老师，那我看时间也不早了，再次感谢您的时间。

受访者： 好的，那我们今天晚上就先到此为止，我们后面再约时间，有机会再见。

# 受访者13

刘书博老师：李老师您好，您也看到线上还有两位我们中财的在读博士，吕梦娜和黄秋莉。我们都是基于兴趣一块儿做这个研究，大家都还是一个比较传统的这种学术探索。所以梦娜和秋莉要不你们也简单介绍一下自己。李老师我之前跟你们介绍过了，是清华优秀的博士生，然后现在是在西北大学任教，也是在做案例研究，包括我看到您还在ASQ上、《管理世界》上发表过文章，非常具有研究经验。所以我也希望带着博士一块儿来向李老师学习，那两位博士先简单的介绍一下自己。

吕梦娜： 好的，刘老师。李老师您好，我是吕梦娜。我也是对中国的管理学还有商学院比较感兴趣，然后正好刘老师对这方面很有研究，所以就跟着刘老师在一起做这个课题。然后今天很高兴能够访谈到您。因为您是很年轻的学者嘛，而且也是做定性和案例这一方面的，我就很感兴趣。想今天跟您多多交流，然后多多学习。

黄秋莉： 李老师您好，就是上次还是在2021年的时候在央财听您汇报了定性研究的主要流派。那今天也是再次感谢您的时间，让我有机会跟您有进一步的交流。

刘书博老师： 好的，李老师，那我们正式开始。大概也是按照昨天发给您的那些问题。第一块儿呢是我主要来问。那第一块儿主要就是想了解一下您求学的经历，就是为什么选择学术道路？当时是有什么样的想法？您去清华读博士具体是什么原因和动机呢？

受访者： 其实我读博士感觉就是非常巧合吧，我也没有说对研究有多么大的兴趣。我是先读了研究生，再读的博士。我本科本来是想着直接毕业的，结果就直接保研了嘛，想着哎呀这个东西就是有点鸡肋哈，想着到底上不上。后来我家里父母说，既然保了就上吧。然后我就读了研究生，研究生的时候我就遇到了研究生导师，他当年正好是去清华做了访问学者，他觉得清华是个非常好的平台，挺好的一个学校。然后觉得我个人可能在研究方面还有一些悟性，他觉得挺适合的，然后推荐我去考一下。我当时的心态就是一心想着要毕业，其实也没有想着考。所以当时我导师都说我去试一下，我就去试了，我当时考博其实就报了清华一所学校，后来竟然就被录取了。所以就是非常巧合地我就去读了博士。

刘书博老师： 您研究生是也是管理学专业吗

受访者： 对，我一直都是管理学专业。

刘书博老师： ok，ok。

受访者： 对。然后去清华的时候其实还是发现有一些差异的。尤其是西部高校跟这个北京靠东边的学校相比，它在氛围上、教育方式上、以及接触到的人和事儿可能都会有一些差异。我觉得清华那边可能会更加接近前沿一些。所以我从西安去北京的时候，当时就觉得，哎呀，我确实有点画地为牢了，觉得在西安接触的教育就已经非常好了。但是到了北京之后，就觉得老师授课的方式以及所能接触到的信息，确实是在北京更新地更快一点，北京老师们的认识也比西安的老师们更加广阔一些。

之后我又去康奈尔访学了一年，因为当时我已经在清华待了三年了，觉得清华已经可能是非常好了，在我的认知里清华是一个非常好的学校。但是到了国外之后发现真是山外有山。就是觉得康奈尔其实可能比清华更好一些。这种好倒不是体现在一些硬件上，而是一些教育的理念。我还记得我感触最深的事件是，我刚开始到康纳尔特别不适应，因为咱们国内教学大多时候是老师在说、学生几乎就是一个旁听的角色。但是到了康奈尔之后，我发现老师他好像一直在引导，他也没有讲太多，他只是在大家说完之后去做一些评论性的东西。当然我在清华去给MBA做助教或者是我们博士上课的时候，老师会让我们大家先pre paper，然后再去讲。国外他们也是这种形式，但是在国内实践出来的就是两套完全不同的东西。也有可能那些管理学很多奠基性的知识都是国外的学者根据国外的情境开发出来的，所以他们讲起来就特别得心应手，就是让你感到循循善诱的那种感觉。所以我刚开始不太适应，我觉得我可能不太适合这种方式。但是几个月之后我发现我还挺适应的，这种方式挺好的。以至于我后来回到国内我还去上了我们学校的博士生选修课，我又去听了一下国内的，会发现为什么全是老师在说，怎么没有让学生说。我当时其实内心里都没有意识到我自己变了，就是我去国外会觉得为什么都是学生在说，我回来居然立刻就感觉到为什么都是老师在说，这种差异还是挺明显的。

刘书博老师： 对，那这一块儿是教学理念的明显差异。那研究方面呢。

受访者： 研究方面我感觉更多的是交流吧。就是国内这种交流的环境会稍微差一些，而且像导师跟学生之间会有非常明显的界限，就是有一种上下级的感觉。但是在国外做研究会有更多的人愿意跟你交流，不管是老师还是学生啊，他们会组织很多的seminar，可能是康奈尔的财力支持会大一些，每一个老师只要愿意组织seminar，然后有学生或者是老师上去讲他的研究的话，学校就会给经费的支持，这种支持一般是给你午餐的支持，就是大家去开午餐会嘛。一个人在上面讲，然后有人去组织。然后我觉得在这个过程中其实大家也都没有耽误时间，只是一个用吃午饭的时间去交流。

我没有太多地在seminar上讲，但是我在听，我就会觉得他们很交叉。也许听你seminar的人是来自于别的学院的，我也不知道为什么康奈尔的金融、组织、商学院是分开的，所以有时候会有其他学院的人来，然后他在说的时候我会想为什么我从来没有想过这个事情。这是来自别的专业、别的视角的一个新提法。就是也许他不能给你贡献，但是你在做下一个研究设计的时候，你会把这个考虑进去，我觉得这个是挺好的。

还有一个比较好的研究方面的是跟我的外导。其实那个ASQ的书评嘛，它其实是一个短论，也不是那种非常正式的研究文章，是在外导的指导下一起把它搞完的，在这个过程中其实我是学到了非常多。做博士嘛，大家都会觉得博士是一个非常严谨的职业，但是我以前其实不太能理解我们在课本上或者是在一些什么奇闻轶事上讲国外的那些学者非常的较真，像德国的学者，就是较真到会像地毯式的搜索，就一点一点的过去，非要把这个掉在地毯上的针给找着了，就是这种精神。在国外我觉得我的导师会一个词一个词地问我为什么用这个词而不是那个词，为什么这个词上加了引号，我当时觉得特别的费解。但是就是这么走下来一圈，后来再去反思的话，就会觉得他们确实是严谨的，在每一个地方都抠的非常的死。我觉得这个对我的研究来讲是···我后来也反思我在写文章，为什么他们会觉得不严谨，尤其是做定性嘛，定性这种主要是靠你的逻辑去撑起来，需要给它足够的证据，足够的例子去说服他说我这个东西是对的。所以我之前就是投了一篇定性的英文文章被拒了。就是他们会提一些观点说中间逻辑链条不严谨，或者是没有呈现出他们想看到的东西。就是我跟着外导走过这一遍我就知道他们到底在care什么东西。如果是之前的话，我可能压根都get不到他到底说的是什么事情。

刘书博老师： 明白。所以这一点就是国外的交流氛围会更加不一样吧，它不是那种自上而下的或者是家长式的、界限清晰的，可能更多是大家平等一些，而且是跨学科更加频繁的这样一种交流。

受访者： 是的。

刘书博老师： 然后你说这个也让我想到，因为我博士是在英国念的，他们其实也有很多这样的交流。也是各个社会学科，有时候生物学的也过来，考古学的也过来，会有比较多不同的学科视角。所以我那个时候看起来就感觉我们商学院似乎更加专业一些，或者说更加聚焦，或者个体特性更强一些。那国外的似乎是会更加crossover，就是他的界限感也不是那么强，有时候老师在讨论研究方法范式的时候，也挺开放的。那国内，尤其是我找工作的过程中，我会看到很多问题都比较来自于实证研究的视角和问题。比如说我做的是话语分析，他会问我reliability、validity这方面，还有我的变量到底是什么。所以就是让我感受到好像学术的这种专业认识是不一样的，我不知道您有没有类似的感觉。

受访者： 有的，有的。我觉得可能您当时是在英国吧，我觉得欧洲做定性研究的人还会多一些，但是美国还是相对少一些，但是确实是比国内好一些。因为做定性研究我其实也是面临了很多的挣扎，最终让我下定决心就是彻彻底底的放弃定量研究的，也是在康奈尔的那一年。

因为在国内的时候，我慢慢觉得清华基本上以做定量研究为主，所以他上的方法类课程基本上都是定量研究。定性研究就是一个老师在一个方法课里可能就只开了一节课，就大概介绍一下让大家知道有这个事儿就行了。但是我接触之后觉得我挺喜欢这个事儿的，我就会慢慢自己去探索，我去旁听了社会学院的课。然后市场营销系有一个老师是做阐释主义方向的，我也去听了他的课。所以这些都是我一点一点自学、慢慢达到的一个状态。但是在这个过程当中其实面临很多的挑战，我觉得我可能是清华经管学院第一个全部做定性研究毕业的学生，所以当时毕业论文写作也是面临了很多很多的问题。刚开始我想要做这个方法的时候，那个时候应该是博二下学期了。我们前一年半都在上课，下学期的时候我在想我写什么东西。那个时候其实还是以定量为主，但是我已经在尝试去上一些课。然后当我博三的时候，就是有这个意向说我想做一些定性研究的时候就去问身边的人，反正老师的态度都是不鼓励做定性研究的，他也会跟你讲说，对于年轻学者来讲，这个定性研究是非常不友好的，发表文章周期长啊，然后这个那个，就是反正讲了很多。所以我其实在出国之前是定性和定量都做的、两个方法都在做。

到了国外之后，其实康奈尔也没有太多做定性研究的学者，因为美国嘛还是定量为主。但是有一个老师，他是劳人院好像，那个老师是做的。所以我就看到了他在做这个，然后我查到了他的名字，我就去问他我能不能去旁听他的课，他说他在组织一个seminar。所以我后来在没有疫情之前，我是每一次都去参加他的seminar的，然后那个seminar上我也讲了一次我的文章。我在听的过程当中，虽然他没有讲说定性研究应该怎么样去做，但是你听他去评论每一个人的idea或者是proposal甚至是已经成型的文章，会觉得定性研究如果你希望这篇文章发表的话，那你在哪些方面至少要满足评论者的要求。那个时候真正的觉得这个事情是可以实现的。就是感觉在他那里听到了一些方法论的东西，就是能够知道作为一个学者应该怎么样去入手，然后一步步应该怎么来，就是能够看到整个轮廓应该是什么样子的，以及他个人也发表了非常顶尖的文章了。当时我在的时候他发了一篇ASQ，然后后来他又发了一篇ASQ，然后他就走了。也有可能是美国的这个环境确实不行，他就去了英国牛津。我当时还挺伤心的，我就觉得我们就这么一个老师还走了。

刘书博老师： 嗯，明白。所以其实那个时候也还是有一个所谓的role model，他对你有比较具体应该怎么做的一些指导。所以这样我觉得在一个环境中这样的一个role model还是挺重要的。

受访者： 对对。你就是能看到他把这条路走通了，而且走的非常好。而且我主要觉得更多的是在具体实践上，他能让你知道你走完这整个流程，就是我看到他走完，我就自己会评估我自己能不能做到，然后我觉得我可以，所以我就觉得这个事情它确确实实可行了。

刘书博老师： 嗯，明白。那其实清华我觉得也还好啊，因为还是允许你做定性研究作为博士论文，像有一些学校可能都不会觉得定性研究是能够达到博士毕业要求的，他不说你做的好坏，但他会评判你只要是非量化实证的，那就不是一篇博士论文，会有这样的一些看法。我不知道您在跟这些老师打交道过程中有没有类似的这种看法的人。

受访者： 那倒也没有，我觉得清华虽然就是可能做定性的人少，但是他在包容性上面还是很强的。也有可能是我导师他指导我的理念非常的好，就是我有两位导师嘛，我的大导师是XXX老师，是一个在国外MIT毕业的博士，所以他本身思想就非常的拥抱国际，而且他MIT毕业的时候写的就是定性的文章，所以他懂，他不反对。我觉得我能够做定性研究的一个非常重要的点，可能还就是一方面是XXX老师非常支持，然后XXX老师也懂一些。然后XXX老师是我觉得清华那个年龄阶段唯一做定性研究的老师。

刘书博老师： 他是多大岁数？他应该是60后吧。

受访者： 对对60后。反正我觉得XXX老师的思想非常开放。然后他对我的教育我之前一直没太理解，在出国之前我是不懂的，就觉得为什么XXX老师看起来像是一个放养的状态。但是后来我出了国，我慢慢理解XXX老师他到底是怎么样的一种教育理念，所以他之前对我所有的指导我大概能get老师当时想让我知道什么事情。

然后另外一个我觉得就可能是我的第二个导师。我的大导师是属于那种不开组会类型的，但是第二导师就是每周都要给我们开组会，所以我是每周都参加第二导师的组会。然后第二导师之前的经历里面也是会跟企业（联系地比较紧密），我觉得清华所有的老师可能跟企业的联系会比较紧密，这其实是做定性研究非常必不可少的一个前提。就是他会带我们去企业参观调研，我们会去给MBA们做助教，做助教就会接触到一些这样的事情。然后我当时反正听了那些故事、参观那些企业之后，确实发现这些东西是我在文献当中读不到的。我觉得也许那个时候可能是觉得这个事情比较有趣，没有人把它做出来，所以我想去做这个事儿。这有可能是我的自驱力吧。

刘书博老师： 嗯。

受访者： 就是这样从博士慢慢地去跟老师调研、去企业座谈啊什么的。然后就见识到这些事情慢慢的积累到一定程度，我就觉得可以去完成一篇博士论文了。所以我觉得有一点水到渠成的那种感觉。

刘书博老师：明白。Ok，那现在您正在做的也是这方面的一个案例性的研究，对吗？就在新的工作岗位上

受访者： 对对。我还是以做定性研究为主。因为之前博士快毕业的时候就积累了很多的访谈嘛，那些访谈有些我还没有整理好，所以我入职这半年基本上一直都在整理那些访谈，然后把它设计成文章。

刘书博老师： 嗯，我先跳一步问一个问题。就是您在找工作的时候，那不同的经管学院呀、商学院的应聘者啊，他们学校的老师啊，对于您的研究有没有一些评价或者看法？因为我知道会有人希望说那你以后需要转做实证的啊，有没有这样的一些经历？就他们会理解你的这个研究吗？

受访者： 他们其实没有这么明说，就是说你以后要转呀或者要做什么事情。因为我当时在面试的时候，我其实有讲我做的定量研究，因为那时候我正好在跟国外一个学者在合作一篇定量研究，所以我是讲了一个定性、讲了一个定量。所以他们可能觉得我两个方法都ok。

刘书博老师： ok，明白。

受访者： 对对，所以没有这个。但是我当时之所以选择西北大学，一方面可能就是这边的系主任，他一直在强调，因为他本人就是一直做定性研究的嘛，他非常鼓励全院的人去做定性研究。所以我觉得在这个环境下也许就是对我来说更好一点。

刘书博老师： 是的，是的，有时候我会觉得关键岗位的一个关键人物还挺重要的。就像这个role model一样啊，可能真的对于这个职业的发展有时候起到非常关键的作用。就像之前我在找工作的时候，我会遇到非常多的那种范式的歧视的老师。但是我到中财，那个老院长就非常鼓励，所以就让我很快下决心似的来到中财。因为我觉得工作的价值是被人看到的，而不是被人所歧视的，这个事情很重要。

受访者： 对对，是的。我觉得之前就是很长一段时间，我当时也其实在担忧，我毕业的时候还在问我的两位导师，我说老师我这以后毕业了，我能用定性方法站得住脚吗，就这个事情太难了，就是做定性研究，你写英文期刊它是一个套路，写中文它又是一个套路。

刘书博老师： 对，是的

受访者： 对，然后就很艰难，我当时其实自己也不太确定。

刘书博老师： 嗯，嗯。ok。那我听说清华近三届毕业的博士大多没有找学术工作，而是去到了比如说企业呀或者其他行业。我不知道您听说过没？

受访者： 我们这一级我觉得确实去高校的同学数量可能有点下降了。之前有一段时间一直是考公务员的多。但是在我读的后两三年，我感觉突然这个找教职的压力一下就上升了，就从2020年疫情的那一年开始不太好找。我们组的我看就是找教职的人还行，我们这一级是11个人，目前毕业的已经有五个人在做教职，其他的可能六个就不做了吧，大概是一半一半。

刘书博老师： 那疫情开始也就是说那个时候有更多的回流，比如说海外的博士回到国内找工作而导致这个job market比较的困难，这是一方面因素。

受访者： 对，我去年有听到就是可能男生跟女生选择还不太一样，就是女生如果可以的话也许就去做这个教职了，但是男生···我去年还跟一个同学交流，他说他可能不做教职了，教职挣的钱太少了，养不了家，所以他就不想做了。然后说北京这个工资，好的才能一年18万，这都算好的，这能干啥，在北京这么大。我们这一届有一个去美团的（起薪）就是六十万、七八十万我忘记了。反正就是工资差的太大了，男生可能接受不了，他们就转了。

刘书博老师： 对对，那有没有可能说通过做教职，然后可以有一个比较好的生活？因为现在我听很多学校发表论文有一个所谓的奖励，发一篇给多少钱。那通过这种方式能够让这个职业变得比较富裕吗？您觉得呢？

受访者： 我感觉好像不太行，就是发文章的不确定性太大了。尤其我觉得很多人不想找教职，有可能是这两年发表文章也越来越难了吧。就是之前我记得我博一博二的时候，发表中文的文章可能还相对容易一些。这两年就是发表中文也很难了，英文也很难，反正就是都很难。然后我有一个师姐，应该是已经发表《管理世界》了，结果她说她不找教职。然后还有一个信管专业的，他已经发了那个领域的国际顶刊了，是那种纯研究的国际顶刊，他说也不做教职，我问他为什么，他说这个过程太痛苦了，不想干了，就是这样子。

刘书博老师： 对，我表示理解，确实挺痛苦的。发顶刊可能一个周期得两年，反反复复的。哎呀，反正有一些人还是挺能发的，然后每年可以发，也不说顶刊吧，像核心的都可以，我看我一同事发八九十篇，他可能就是生产线建设的比较好。但是我会觉得其实对于多数人来说，这是不太可能通过发表文章发家致富的，或者说达到一个行业收入水平的，可能更多还是希望有更多自由的空间和时间吧。但其实现在看起来，如果想要达到学现要求的双一流建设标准，对于老师的要求···，那他的这种自主性其实完全也就投入到工作当中了，我觉得工作密度也并不小，所以整体来说性价比也不高。

受访者： 对对，确实是这样，我那个即将要离开学术界的男同学也是这么讲，天天累的跟狗似的也挣不到钱。还有一个朋友就是他已经去做一所学校的博后了，但是他觉得可能留下来有点困难，干的活儿多钱少，图啥呢？走了。

刘书博老师：而且现在好多学校，尤其是985的，一线的，甚至像武汉这样的二线985他们都在搞师资博士后。其实让工作本身也变得非常企业性质，就是临时合同工的那个感觉，也就更导致这个还不如直接到企业去。

受访者： 对对，其实有时候我在想就是这种像产论文一样的感觉其实不太好。你看人家国外做一篇文章出来，那都是就是有兴趣；而且整个过程就像做创新一样，就是企业创新一样，它是一个过程，经历了一个完整的过程。但是就是中国这种跟风的现象还是挺明显的。

刘书博老师： 对，我就觉得我们太工业化了，我们就把标准拿来直接就生产，缺乏一种探索创作的。

受访者：嗯，嗯，是的，是的。

刘书博老师：行，那我这边问题今天就问到这里，剩下的时间我交给两位博士生，他们有一些不同视角的问题。

吕梦娜： 要不我先问吧李老师，我想问一下就是您在找教职的时候，当时是出于一个什么样的考虑？因为刚刚聊到您可能去清华读博的时候，似乎也不是特别的兴趣驱动哈，因为有一些机缘巧合在里面，那等您做教职的时候又是出于一个什么样的考虑呢？

受访者： 其实我读了博士之后，我的目标就相对明确了，就是进学校嘛，就已经读了博士了我觉得不进学校就有点亏。所以当时其实我是断掉了一切的后路，坚决的选择做教师。其实我是一个需要自由的人，我不太喜欢那种做班的生活，就挺辛苦的。觉得如果去做教职的话，对于一个女性来讲，可以有自己的时间去看看书，做做自己喜欢的事儿。其实我当初设想教师可能会给我带来这样一种生活。所以当时我的两位导师其实也是给我提供了一些帮助，他们说我除了找教师之外，可以去一些比较好的院所呀，就是大型的国企、央企啊这种，他们都有给我介绍。我当时其实都是坚决的拒绝了，我都觉得我要拒绝一切诱惑，坚决的走这条路。

吕梦娜： 嗯，很坚定啊，那就是自由方面的考虑会更多一些，那您在具体找学校的时候会比较关注哪些地方呢？因为刚刚您提到了系主任如果能够支持您做定性的研究，会让您更安心一些嘛，那还有其他方面的考虑吗？

受访者： 嗯，就是还是自由的事情。我当时其实只是想在两个城市之间选择，要么北京要么西安。因为西安离我家近，然后我在这儿读的本科嘛，所以对这个城市熟悉一些。后来就是没有选北京，因为也有可能跟我的经历有关吧，我觉得并不是所有的人都会这么想。因为我博士期间其实是出了一次车祸，然后出了那次车祸之后，我就会觉得我为什么要这么拼呢，我就觉得自己开心就可以了。然后到了国外过了一年的生活之后更加坚定了这种决心。因为我觉得像国外那些老师，其实是有非常明确的工作跟生活的界限的。我的导师他周五下午就跑去玩儿了，他一定是去玩儿去了，所以你不要找他，你找不见他的，包括那边的行政人员也是。我记得很清楚的是有一次周五的下午，我在听一个讲座，听完之后我去问他问题，我问完之后教室里只剩下我俩了。他当时跟我讲，这会儿外面的夕阳可美了，赶紧出去看吧。我当时心想今天这个论文还没写完呢，看什么夕阳啊哈哈。但是就是在这种环境中生活的久了，我就会觉得我是希望能够把我的生活跟这个工作的界限给分开，但是在北京我觉得这种可能绝对实现不了的。然后我当时是在想说，做研究在哪儿不是做，现在这个交通这么发达，去哪儿调研也都可以。所以我当时想那要不就选西安吧。

吕梦娜： 嗯，嗯，那西安有很多高校嘛，就是您做出去西北大学的决定是有什么其他的考虑吗？

受访者： 其实也没有太多其他考虑，我觉得我这个人比较随缘，我就是找工作也比较随缘。我当时其实有想了一下要不要回去我那个西安电子科技大学，但是后来想哎呀，就是怎么说呢，可能那边的老师过于熟悉，然后去了就感觉有点尴尬。所以后来正好有一个西北大学的老师他天天给我打电话说，这边可好了、怎么怎么地的。而且当时我在想西电可能是一个更偏理工科的院校，然后其实我一直上学都是在理工科的学校，清华也是一个理工科的学校，然后就想要不去文科学校感受一下。西北大学这个经管学院也还可以，所以就来了。

吕梦娜：嗯，嗯，了解了。那您到了学校之后，就是当前会面临像“非升即走”这种考核要求吗？因为我不太了解您是正常的编制还是说当前的这种“非升即走”？

受访者：没有“非升即走”，但是我感觉这学校目前正在改革，我不知道这个是什么个路子啊。西北大学应该是那种进来的时候给的编制，但是又给你签一个三年的合同，满足合同里面的要求。然后那个合同满足的要求吧，其实可低了，你可能一年就能做到了。

刘书博老师： 听起来应该不是“非升即走”，听起来应该就是编制的，还是传统的。

受访者： 对，它（学校）说进来的时候就是事业编，但是它又签了一个三年的合同，所以我也不知道这到底是个什么操作。

刘书博老师： 我来中财也是这样的，那只要是事业编，那个合同就是一个附加的、不是主要的，所以我觉得你们学校应该还没有像武大、中山那么激进的搞人事改革变动。就是西北大学我觉得还是很忠诚的、忠厚的。

受访者： 对对，确实，我感觉还是挺忠厚的

吕梦娜：所以其实您遇到的这个学校也很好，我听起来。然后下面我想换一个问题哈，就是关于您当前的研究方向，因为我看到您简历上写的主要是关于科技成果转化还有技术创业的，我听这个字面意思，会觉得跟其实践啊、企业关联还是比较大的。然后就很好奇，您是怎么关注到这个方向的？

受访者： 这个也是一个偶然的机会吧，就是我记得当时我是博二还是博三的时候，我一个师兄他要毕业，他在做啥来着我忘记了，反正也是跟创业有点关系。然后他就去访谈的时候，我导师就说我跟着他去学习一下，我就去了。然后那个时候可能是我第一次参加正式的访谈，那个师兄他其实当时做的也不是纯定性的研究，他那个访谈也是为了佐证一些事情。访谈就是跟我们现在讲的定性研究来对比的话也是非常的不规范。但是从我跟他去访谈了一次，我会觉得我还挺喜欢这个事儿的。

当时我记得有一个人他应该是博士毕业之后去干那个事情，所以我就关注到了，就是他提到了他们企业面临的一些困境，就是说怎么样从学校把这个知识产权给转移出去嘛。他当时其实当时最大的困难在于国有资产流失的问题，可能这两年会好一点，因为制度变了嘛。但是那个时候就觉得这是一个非常大的问题。后来我就沿着那个方向开始去关注了，不管去哪里参观调研，或者我碰到合适的人，我就会留心这个事儿，所以后面就跟他走了吧。

吕梦娜：所以其实也是比较实践驱动的研究，可以这么说嘛？

受访者： 嗯，对。

吕梦娜： 那您在做这个研究方向的过程中一直是很坚定的要沿着这个领域深耕下去，还是说中间可能也会有一些动摇，就是放弃啊之类的念头。

受访者： 其实我感觉做定性研究，没有说最开始你的方向就非常明确的是什么，你让我两年前说现在做的方向是什么，那我可能也不知道是什么，但你回头总结，可能把它总结到这个方向。就是我感觉质性研究本身就是一个探索的过程，就是一开始你可能只是看到了一个现象，然后慢慢的才会深入吧。

吕梦娜： 嗯，嗯，了解。其实我觉得您这样基于实践、与实践相结合做研究是很好的，然后现在不知道您有没有听说过，管理学有一些研究可能会被质，比如说和实践脱节，我相信您是不会遇到这种问题的，但是您身边有没有这样的一些人或者是听到过这些事情。

受访者： 哎呀，我遇到这种问题可太多了，其实这种问题倒不是来自学术界，学界当然不会关心这个问题。更多关心这个问题的是来自于我的访谈对象。我经常去会去访谈一些创业者，但是创业者···就是我之前还跟一个做定性研究的老师交流过，我说我这个编码最后编完都是问题，他说你怎么会编完都是问题呢，我说创业者抛给了我很多问题啊，因为他们做的是大企业，那大企业讲给你听的一定是他们走过的历程、他们怎么做的，所以提炼的更多的是策略性的一些东西，但是我看到的我满篇的全是问题，我最后提炼出来的是问题，就是很少有解决对策的。通常我坐在那里他会跟我说，“你就给我一个模型，你告诉我输入什么就可以输出什么结果，你是学管理，你还是管理学的博士，那你告诉我应该怎么做”。就是经常会遇到这种他们迫切的需要解决那个事情的，所以我有时候就会做不到。然后很多人也会讲，“你那书上写的是错的，书上说让我创业讲聚焦啊，我聚焦我这个技术，可是我员工都没钱了，我发不起工资了，公司都要死掉了，我还去做什么呀？那我肯定是什么来钱快我就先赚那个钱，这个时候我肯定是分散的，不是聚焦的”，就是他会去挑战你，所以这种问题还挺多的。

吕梦娜： 哦，我听起来感觉还是蛮有压力的

刘书博老师： 好，就梦娜最后一个问题，你说到这个脱节呀，或者叫gap。那其实我也是同样遇到非常多这种问题。其有点像是在国内啊，只要是做企业，他们必然会向我表达你们做管理学研究的，那怎么不做人该做的研究哈哈。好像他们不理解，就比如说参加过很多培训的那些年轻一点的管理者，就还有很锐气的表达，他们就对管理学者非常批判式的，所以国内会有这样的一个现象。可能国外我没接触过，不知道是不是同样，但国内是有这样的特色的。

受访者： 国外其实也有，因为我也遇到了这个问题。所以我碰到我觉得比较权威的人，我都会去向他请教是怎么样解决这个问题的，或者看书的时候我会稍微留意一下，就是他们会怎么样去解释这个问题。我当时觉得就是国外也有这个问题。我跟我外导交流，我问他，我说我去访谈国内的人啊，他们会问我问题，我要去帮他解决问题，但是其实更多的时候我是去向企业学习的，我应该怎么样去平衡这个呢？他说你去企业调研的时候，你可以不用说你是去去帮他们解决问题，你一开始就要跟他们说明白你是来学习的，不是来解决问题的。所以他（外导）也会有这个困惑，他说很多事情确实没有办法解决，但是我记得他当时跟我讲的是做研究是做研究，做企业是做企业，这两个是分开的。

刘书博老师： 对对。而且现在我们一些学者会把这种学术隔离状态进行合理性解释，他说这个科学性研究就不是关注现实嘛。但是我会觉得自然科学可以这么来，那社会科学不可能不关注现实，这个本质是不一样的。那还有一些学者在合理化解释之后，他就维持了自己的“学者的骄傲”，那这一点是更加招企业界人反感的，比如说他们会讲有一些学者就在那儿讲一些空话，然后把一些现象描述的天花乱坠，但其实就是那么一个意思，然后又要表现出高高在上的那种姿态。所以我很为这些同事感到烦恼，就碰到一些企业家，他们好像对我带有这种偏见和防备心。

受访者：  其实我对这个的体会也特别的深，其实这个是我现在做经济研究面临的一个非常大的问题。就是我没有办法在理论贡献和实践意义这两个之间达成和解，有时候我写出来的文章我发现这个理论贡献很强，但实践意义就会很小，就感觉一个东西你抽象到一定高度，它实践意义必然会小的那种感觉一样。所以我现在困惑的是我怎么样把它抽象到一个合理的高度，既有理论又有实践意义。我可能也是昨天才意识到这个事情，昨天刘老师咱们聊微信的时候，我那会儿其实正在参加一个线上的那种企业分享，他们分享很多实践中的一些东西。我从毕业到现在，去年没封城之前出去调研了一次，然后今年还没有开始调研。但是在这个中间歇的这一个多月的时间，我就会莫名的感觉有一些恐慌。这种恐慌是来自于做定性研究，怕你做的研究跟不上实践，你怕你做的研究不能指导实践的这种恐慌。因为我参加了很多这种企业的横向课题，我能够感受到有时候你报告里面写了什么点，他们会觉得非常的开心。有时候写了哪些点，他们会觉得你就写了个这就完了，就是会有感觉他们想要什么东西。所以我也是在努力，希望我未来的研究中能够做到一些对理论和实践确实有意义的一些事情。所以我会不断的督促自己去听企业正在发生什么事情，技术正在发生什么样的变革。这些事情其实都跟研究没有关系，但是我觉得我得去了解，你不了解的话就是确实没有办法去贡献实践意义。

刘书博老师： 明白。那您说这一点又让我想到这个评价标准的不同，茶余饭后我跟同事聊一个，一个学术做的很出色的年轻老师，我们就聊到陈春花。陈春花非常被企业家所接受，然后她似乎也能理解企业家的那些管理者的心态。我问过企业家，他们会觉得她写的东西就很好用。然后同时这个年轻的出色的学者，然后就评价陈春花，就说她的学术做的非常糟糕啊。所以有时候我会觉得似乎是完全不一样的标准。那如果我们用学术的标准把这个论文拿给实践者看，他们会觉得这是什么东西？然后如果我们用学术标准再看企业，他们想看的那些实践的读物，似乎又会觉得这个学术方面又很糟糕。这个是不是就是没有办法啊，让双方可以契合的？

受访者： 对，我现在其实也没有找到特别适合的，我刚开始说我现在面临的一个困境就是怎么样实现这个理论和实践的平衡。

刘书博老师： 尤其做量化的学者，那他们的认为好的学术产品应该就是像这种自然科学研究的那种实验室严谨的这种文章。那这样的一个文章因为太严谨了，可能对于现实的那种动态啊、整体啊就没有办法呈现出来。所以我觉得咱们现在管理学的学术标准啊，似乎也是像您刚才说的首先是严谨，那这个是很重要。那除了严谨之外还有别的吗？您觉得什么是好的管理学这个专业的好的作品？

受访者： 管理学专业好的作品？反正我一般会给他打五颗星的那种文章要么是我觉得我从来没有思考过的一个视角，这篇文章是提供了一种新的视角，那这个视角一定是有理论贡献的，但是我觉得理论贡献高到一定程度一定是有实践意义的。举个例子，我之前看了一篇ASQ的文章，我们一般讲企业惰性嘛，企业惰性一听起来就可能是大企业面临的问题，但是那个文章他就写了小企业也面临企业惰性。然后解释为什么会有企业惰性。然后他用的是认同理论，说这个技术类的创业者对于创新的认同度太高了，技术承诺太高、创新承诺太高，所以导致他就会在很早的时候就产生路径依赖，这并不利于企业成长。我当时觉得这个东西就非常对，因为我看到的现象确实就是这样的，但是我当时就想我为什么没有想到它可以归结到这里。这是我看到的就是他既有非常强的理论贡献，你可能没有想到过说小企业也会有惰性，这是他的理论贡献；那实践贡献是告诉企业说你不要对技术有那么强执着的追求。这是一种我觉得比较好的文章。另外一种就是我感觉他可能有点像研究报告类型的，就是我看到他对整个过程描述的特别清晰。比如说在我的领域当中，我能看到他会描述科技成果转化的这个过程，然后画了非常清晰的图。然后每一个步骤说企业会发生什么，然后列出来，但是它又不是那么太像研究报告，他会有一些理论的东西去解释，在我眼中是这两种。

黄秋莉：我想结合您在学术工作中的个人感受问一些问题。刚刚听到李老师说自己跟实践会有比较多的接触嘛，然后在这个过程当中会意识到很多实践的问题，那我觉得这是一个探索性的过程，看起来李老师也会觉得这个过程是一个非常让人享受的过程。那我想问除此之外在学术工作过程中让您觉得比较有趣或者是能激发您热情的，还有别的地方吗？

受访者： 别的地方能激发我热情的？

黄秋莉： 对，就会有加强您对学术工作认同的一些地方。就是觉得做老师比较好呀，跟一般是企业会不一样的地方

受访者：做老师的好处动力呀？其实我刚入职，还没有怎么带过课，我带的课可能都是线上完成，所以我没有特别真切的感受。但是我觉得教师是一个自由的职业，也许可能未来我在代课的过程中我能感受到来自于学生的尊重。但是我能感受到的是去年有几个同学来找我指导创业大赛，然后我给了他们一些建议之后他们会觉得他们会觉得非常的实用。因为我确实是看过了少说也有100家甚至是200家左右的创业企业。我知道他们面临的困难以及甚至从投资人的视角来看，说他们真正的在意什么东西。那些学生可能觉得我提的建议相比于其他老师来讲可能更加的落地一些，这种反馈让我感觉还挺好的。另外一个就是我去年遇到的事情，我刚来西安去参加了这边的一个校友会，然后那个聚会有很多企业、政府，当时好像只有我一个是学校的老师，其他人可能都是企业多一些。然后那个校友会上大家交流的时候就是会感觉到他们对老师这个职业……怎么说呢？具体也说不上来，我就没有想到合适的形容词来形容。就是我感觉到老师是一个中立者，你去了你不会妨碍任何人的利益，但是你又可以跟他们任何一方去合作。因为企业跟企业的之间的交涉，他一定会涉及到一些利益的交换，要么去卖产品的，要么去找客户的。就是我感觉校友会他们都是这个目的，当时就有一个人讲，你这个身份特别的好，就是觉得你不论做什么就是一个中立的角色。所以我觉得我还没想到说这个事情对于我做老师来说有什么好和不好，但是我觉得这个是一个我觉得不同的点吧。

刘书博老师：对，李老师说这个也是所谓的这个传统的知识分子提供给社会的一个价值，因为我们不站在任何一方去给他们做背书，我们就是冷静的旁观者。比如说看到社会层面有什么共同的问题，那我们批判啊，我们提供建议，而且这种批判和建议也不是为了自己，更多是一种对于社会的责任感。所以我会认为知识分子啊，我们之前经常会说公共知识分子，后来在中国可能被用烂了，但是其实这个知识分子它就具有公共属性，它是具有这种社会责任感，是需要守护社会的健康。就是说这个医生、老师、律师这三个职业是受到社会尊重的，为什么？其实他可以把这个事情做好，那这样的话汇集到整个社会那也会做的不好，所以我们需要这三个职业具有职业道德感。其他职业，传统来说就是农工商嘛，那为什么把商排在最后？传统社会就认为商是利己的，是个人利益最大化，所以就缺乏一种所谓的公共道德。

当然后来亚当斯密论证这个事情，市场也能达成公共受益。但是传统来说，老师这个职业是传道授业解惑的，而且更多是批判性的，是有这样的属性。所以李老师您说的那一点我还是认同，就是我们是一个中立者，进而是可以做出更多的贡献。

受访者： 对，是的，就是刘老师说的这个意思，我可能一下没想明白，但是您这么一说就感觉瞬间提上来了。

刘书博老师： 我也是看有一些文章这么说的，我之前也是探讨呀，就之前不是特别理解怎么国家、人民和家长对老师的期待这么高？为什么职业道德要求这么高？我一开始还不是很理解，后来我看到那个文章之后我就觉得还是挺有道理。就你这个事儿你做不好，可能没有人会发现你做不好，但是可能影响的是好多学生未来的命运，你做的好，可能也同样不会有人看到，但是他可能就是无声之中让很多其他人受益。所以如果老师都把良心活做好的话，那可能这个社会就会更好一些，然后我是现在是这么理解的。

受访者： 是的，是的。谢谢刘老师。

黄秋莉：一个比较优秀的学者需要发挥自己的公共属性吧。那李老师对自己未来职业的发展有什么样的期待吗？或者是定位什么的？

受访者： 从功利性的视角来讲，我肯定是希望能够快速的评到副教授、教授这样的职称。就是总感觉这个事情怎么说呢？就是在一种有压力的环境下做事情跟在没有压力的环境下做事情，它的动力是不一样的。我现在做一些事情都是为了说我怎么样能够快速的把职称先品上，我不再为我的生计去忧愁的时候，我可能就是更想去做自己想做的事情吧。我也不太确定未来我想做什么，但是我是希望在这种没有压力的环境下，真正的去做一些不为温饱而去做做的那些事情。我以前特别讨厌追风口，就有点类似于投资人追风口，中国学术界做研究也是，今年国家倡导数字化转型、数字经济，大家一窝蜂都去做数字经济，但是总有一些是冷的研究，像刘老师现在做的这个事情，我觉得就是一个非常有意义的事情。但是你为了生计考虑的话，做这件事情显然是不那么恰当的，但是这却又是你自己喜欢的。所以我觉得我未来的目标就是赶紧把这个生计的问题先解决了之后做一些自己想做的研究吧。

黄秋莉： 我不太能理解为什么说是我们要评到一个副教授或者是教授的话，就能够解决这个生计问题？因为之前对一个老师访谈的时候，他就说北大之前有很多老讲师，他们一辈子也都是在做自己喜欢的事情。我其实就不太明白这个转折点为什么会发生？也就是说为什么获得副教授或教授之后就能够解决我们的一个生计问题呢？

受访者： 因为一般到副教授他会有长聘机制，就是他就没有理由再能把你从这个体制内给你踢出去了，你这个时候就会觉得好了，就算你此生啥也不干，然后你就可以躺在这个上面了。这是一种来自于你不会被威胁的一种生计的考虑，倒不是说就是钱多钱少的这种。

黄秋莉： 明白，就是感觉我们现在还是面临一个生存的压力嘛。那您觉得这种对您具体的研究状态或者是心态有什么样的影响吗？

受访者： 其实对我影响不是太大。因为虽然我可能是希望尽快的解决这个事情，但是我觉得我一定能非常快的解决这个事儿。因为我在博士的时候，就是有时候你生命中总会有一些贵人出现，我博一的时候就有幸见到了一位老师，他当时就讲其实你在博士五年之间做的事情就会奠定了未来你职业生涯发展的前五年。所以我从那个时候开始我就知道我现在所做的所有的努力，那都是为了我工作之后减少负担。所以我在博士期间其实是积攒了很多的东西，包括博士论文写完之后，其实我博士论文全都是新写的，一个字儿一篇文章都没有发表，所以我现在可以把那些文章全部整理整理去发表。然后现在目前在做的这个事情，也正好是我自己喜欢的，我喜欢技术类创业这个领域啊，也是在解决一些事情，所以我觉得我现在这个状态是挺好的。但是如果能够解决那个长聘的话，我觉得就是好上加好吧。

黄秋莉： 是没有后顾之忧了。那感觉听起来就是我们这个学校氛围也比较好，然后李老师现在这个科研也比较顺利。那有没有还有一些让你觉得不太满意的地方呢？

受访者： 工作过程中我觉得不太满意的地方，更多的还是来自于来自于落差吧。能够达到清华那种条件的中国的高校非常的少，刘老师肯定也会有这种感触，就是在高校里边作为一个老师，你是为这个学校最能够创造价值的，你需要去给学生授课，然后你要搞研究，提高学校的声誉，这都是老师应该做的事情，所以他理应是这个组织里边最重要的角色。但是你在这个组织中却没有得到跟他匹配的尊重或者是什么也好，我讲的可能更多的是学校的教师体系跟这个行政体系之间的一个平衡。就是我也听其他好多同学在讲，可能很多学校都是这种行政高于老师，就会觉得我们老师更多的时候是一个被命令的这样一个角色，而不是就是被服务的这样一个角色。

黄秋莉： 看起来好像是发生了一些小的故事，是吗？

受访者： 那倒也没有，只是个人感受。

刘书博老师： 对，中国大学其实也是完全被管的嘛，教育部管的嘛，所以也是一套自上而下的管理体系和行政体系。我觉得中国大学也不可能完全国际化，就是我们我们还是有这个意识形态的作用。那西方我知道英国就非常商业化了，非常公司化，所以他们那边其实行政也开始越来越像职业经理人在管理老师，然后他们老师就从professionals变成了academic work啊，也会存在这种问题。然后下周我还要去审教材，审这个意识形态问题，还是一天审完三十多本教材，然后让老师去审，我觉得还是挺有中国特色的。

受访者： 是的，但是我感觉在清华会好很多，也许是清华老师个人的声望然摆在那里。刚才那个秋莉问我有没有什么小故事，确实是没有，只是说我在清华待了这么多年，然后突然来到这个学校里边，你会发现怎么这个做事的方式不太一样。

刘书博老师： 那李老师有没有想着说“学而优则仕”？比如说我们学校有双肩挑的这种老师，既做专业工作，又做行政管理工作。

受访者： 目前还没有这个想法，我现在这个心态主要还是学者的一个心态。因为我看到我导师走过了，两位导师都走过。一旦就是进了那个方向上，就会被一些行政类的事物缠住了，没有办法做自己想做的事情，反正就是行政嘛，我觉得那个事情也就是一种机械性的工作。

刘书博老师： 对，就完全不同于专业性的工作。

受访者： 反正我现在还是希望先把学术做好，先把自己喜欢的事情做好。

黄秋莉： 我想接着刘老师刚刚说的这个意识形态的问题。我的同学他是新疆财经大学的老师嘛，他就跟我讲他们学校审教材的要求特别严，他们可能会一字一句的花非常多的时间去做这个事情。那我其实想问问刘老师，对这个事情除了觉得这是政治意识形态在起作用以外，您对这件事情的态度是怎么样的？您会很排斥这个事情吗？

刘书博老师：我就是觉得这件事有点粗粗鲁，就突然发一个消息，就告诉我们教学委员会的老师要去审，他没有任何商量的余地啊。或者说这就是一则命令，然后这个命令似乎还蕴含着说你要审那你就负有责任，那你这个教材假设说未来如果有问题，那谁审的谁负责，我觉得那他就会蕴含有这样的一些潜台词。所以这个时候你就会意识到你的工作量其实不小，而且这个工作感觉跟我之前认识的那种专业工作的责任又不一样，这更多是一种对权力的维护啊，是这种责任，而不是说对真理的维护。

黄秋莉： 明白，就被迫的感觉。

刘书博老师： 被迫的，而且是毋庸置疑的让你去保护他的权力，是去守卫权力而不是守卫正义。

黄秋莉： 好的，好的，明白。 那我还想问问李老师哈，李老师当老师还没有很长的时间嘛，但是您有没有一个初步的感觉，就觉得当下的这种学术生态啊或者是制度环境啊，对于咱们个体的发展有哪些好的方面或者是不好的方面，就对学术生态的一个感知是什么？

受访者：学术生态是哪方面的？

黄秋莉：比如说考核评价体系，非升即走等等。那您好像没有面临这个方面的压力，那您同学呀是一个什么样的感觉呢？

受访者： 就是非升即走，大家更多的是排斥吧，但是可能没有激励也不行，因为我能看到的啊，我身边的同学对这个非升即走肯定都是全是抱怨。但是我也能看到西北大学他确实也面临这样一种困境，就是你不搞这个考核的话，那老师就会失去动力。因为老师这个职业比较自由嘛，你没有办法去限制说你今天必须干什么，然后你就要坐在办公室里，这样我就知道你干了什么，没有干什么。所以我进来之后，我也是最近一段时间才注意到，就是虽然经管学院企业大学经管学院有这么多的老师，但是像你刚才讲的，说老一辈的那些老师为什么在他的教师的岗位上待了那么多年，一直做个讲师也没有后顾之忧呢？因为他确实不需要有后顾之忧，就这个讲师的工资也是随着你这个教龄的增长是一直在涨的。所以他的工资收入其实也还可以，他讲讲课就完全ok了，我不想做研究，那我就不做了呗。但是学校可能对他们的要求就是除了教书之外，我还是需要你做一些研究的。但那些老师就会觉得我为什么要做研究？我不想啊。我就拿着这些上课的钱，然后我出去自己做一家公司或者我去做咨询，我活的也挺好，就是挣的钱也挺多。那我还挂着这个大学的这个名号，所以会有有一批老师就会选择躺平，然后用空下来的这种工作的时间去开展做副业还是什么的。所以我觉得学校确实会有这方面的困难。但是说非升即走这个制度要实施到什么程度可能还有待商榷吧。

黄秋莉： 好的，就是咱们西北就是咱们学院的这种改革是不是也是这种趋向？就是想要用更多的考核体系来规范我们老师的行为，不知道李老师了不了解这个改革的内容是什么。

受访者： 具体的我还不是很清楚，因为我刚进来也还没有太久，但是我自己能看到的就是他可能会面临这方面的一些问题。

吕梦娜： 李老师，我刚刚想接莉莉稍微的问一句，就是您听到了很多同学对“非升即走”的一些抱怨，那您有没有考虑过，就是这个制度在中国高校其实也已经断断续续的存在好几年了嘛，然后刚刚说西北大学其实也有这个倾向了，您有没有考虑过它这个制度存在的合法性是什么？或者说尽管这么多抱怨，它为什么会这么长时间还存在？

受访者： 我觉得非升即走这个制度，就是刚毕业的学生去的话就是非升即走。一般实行非升即走的这种学校，它的工资水平会比那些不实行这个制度的要高一些。所以这个制度本身它会有一些能够激励到部分人去干这个事儿，但是干了之后那些人可能没有想到我拿了你的钱，原来我需要满足这么多的要求，然后换来的是一个长周期的不稳定，所以可能后期激起了反弹吧。但是确实它在最初的时候我觉得是能够激励到一部分人的。

黄秋莉：好的，李老师。我觉得林老师发的文章挺多的，那能不能给我介绍一下就是发文章的经验是什么？就是对我来说我觉得挺难的，那感觉对李老师来说还是挺简单的，有没有什么诀窍呀？

受访者： 其实我那些发出来的文章也没有什么诀窍和经验。因为像这种文章怎么说呢？就是发出来那些文章就是发出来的，我也不知道为什么被接收了。但是我能够从我被拒绝的文章里边谈一谈体会吧。

像我之前发《科研管理》《科学研究》的这些文章，就是中文的嘛，我当时投了一次就中了，然后中了之后，对我个人来讲其实我就不想再投了。因为我觉得这个事情没挑战了，我想投到其它期刊。但是我一直在投《管理世界》，《管理世界》是一个挺难投的一个期刊，就是我两篇文章在第三轮被拒了，我觉得这个事情还挺可惜的。但是我觉得我是那种别人接收了我就不想投了，他要是不接受，我就想一直投的那种人啊。我觉得就是我一直在反思，因为《管理世界》一直拒我，我就在想这个期刊到底要什么东西？我总结下来的啊，第一点就是说你的主题一定要跟这个期刊 是契合的。 我后面听了一次他们的主编说，我们就解决中国当前最急需的一些重大的课题，卡脖子技术呀，重大工程啊这种类型的。所以就是不是这种风向的你就不要投，因为他一定会给你拒掉的，在第一轮的就拒了90%的文章。

 然后第二点要迎合中国学界对文章的一些认知吧。就是我刚才其实提到了说发国内文章和国际文章它是不一样的套路，尤其是在做定性研究，这个上面体现的更加的明显，就是中国定性研究可能起步比较晚，所以大家还都在探索的阶段。有些东西你的严谨性和你的理论贡献就可能上的太高，就是你跟国际期刊对齐的话，然后你到国内，审稿人可能get不到你这个文章的价值在哪里，然后他会用国内的标准去评判，然后国内的那些标准可能就是跟你这个文章不匹配嘛，所以他还是会把你拒掉的，所以我觉得可能这是第二个。

第三个点我觉得可能是跟我个人有关的一些事情吧，就是我一直在反思为什么就是投了两篇文章都是在三审被拒掉。我觉得第三个点就是混圈子。我觉得定性研究就是一个圈子，就是这个圈子还挺小的，就你得进入那个圈儿之后你才能够慢慢的建立自己的合法性，然后你会被接收。就是我感觉国内国际上发文章他是不那么在乎你个人之前的学术的经历是什么样子，但是国内他就挺在乎这个。对于我来说就是从0到1的过程。我之所以一直执着的说非要投一篇管理世界，就是因为我觉得《管理世界》它代表了你在国内的定性研究圈子里你有一席之位。因为管《管理世界》在定性研究方面的文章非常的权威嘛，基本上是一个top one的这样一个角色。但是难就难在这个从0到1的过程，我的两位导师都不是那个定性研究圈子里的，不是国内圈子里的，所以相当于我要单枪匹马，我自己冲出一条路来，这个挺难的。所以很多人会讲说你要去抱大腿，但是我觉得我现在还没有想好，我再试一次吧，再冲一下试试吧。

黄秋莉： 可以的！那李老师现在有没有升基金呀，就是学院有升基金这方面的要求吗？

受访者： 有啊，每个学校应该都是一样的吧，就是他会要求你升国家级课题呀，省部级的呀什么之类的。

黄秋莉： 您觉得这个要求合理吗？

受访者： 我觉得这个要求还是合理的，但是我感觉升基金的这个过程有点卷，就是卷到你需要把你的假设都给写出来。我觉得你做一项研究计划其实没有必要那么细，你还没有去做了，然后这个事情你就把它已经写出来了。所以我感觉他这样的一种筛选的机制，可能到最后筛选出来的都是那些已经做完了的，然后做好了的，其实不需要资金支持了。但是看起来那个本子非常的perfect，是这样的一个状态。所以它会造成我感觉像那种二八效应，就是穷者越穷，富者越富，就是你越大佬你越能拿到课题，然后你越是小渣渣你越拿不到。所以我感觉这种政策的导向会导致需要支持的没有得到支持，不需要支持的得到了更多的支持这种状态。

黄秋莉： 所以您觉得它的合理性在于它能够给我们提供资金支持。

受访者： 对，我觉得资金支持是必要的，因为要培养学生嘛，就是要带学生去调研啊，我觉得有一些课题的知识还是必要的。

黄秋莉： 那我觉得这应该是学者自发驱动的一个事情。但是从学院层面发出这种号召的话，是出于一个什么样的理由呢？

受访者： 我觉得在学科评估以及评价这个学校的排名的时候，一定要考核这些指标的，所以他才会鼓励你去申请。

黄秋莉：明白了。那老师在学术工作过程中有没有感受到一些同质化的要求呀？可能像先前提到的，有些老师会让你转向去做一些定量研究等等。那您有没有遇到过类似的或者是不仅仅是限于这种研究范式方面的同质化的要求？

受访者： 你讲的是我现在单位的要求吗？

黄秋莉： 可以这么说吧，但是也不仅仅局限于单位，就是广泛来讲，您觉得自己有同质化的倾向吗？

受访者：我倒是没有。就是结合我博士的经历，我的导师都非常的开明，就是他鼓励你去探索自己喜欢做的事情。然后单位的话目前我也没有感受到他强迫我去做什么。可能是因为成立了那个26期刊的案例联盟，所以这个环境稍微好了一点，所以我没有感受到这种压迫。

黄秋莉：范式方面感觉是变得越来越好了哈。

受访者我觉得很多时候是自己选择的问题。就是我在进入这所学校的时候，我就知道他这个氛围是支持我做想要做的事情的，如果他不支持的话我可能不会来。所以我觉得也许有些老师会遇到这个问题，是他可能一开始没有想到后面会有这些问题存在。

黄秋莉：刘老师，我暂时没什么问题了。

刘书博老师： 好，好好，那我这边也没有了，那我觉得今天那个也学到了非常多。再次感谢李老师的时间，我们以后有机会还是要多交流。因为咱们做这个质性研究的其实像您说的是一个小圈子，那以后我们互通有无啊。您刚才说的发表论文确实需要圈子啊，这个国际上也有，国内也有。然后因为中国有很多有趣的现象值得被研究，那研究出来之后还是值得我们去进一步探索，找到渠道把它发表出来。不管这个期刊到底是顶级的还是核心的啊，我们到时候再找机会再多多的交流合作。

受访者： 好啊，没问题。

刘书博老师： 李老师，那我觉得今天咱们时间就差不多了啊，再次感谢啊。

# 受访者14

刘书博老师：我们前面在中财做了差不多三十个访谈，还是有财经特色的，工具主义比较明显，我觉得跟美国的那种短平快的比较对洽。但是我不知道政法大学，可能更人文一些，出来了像罗教授这样的著名学者，可能土壤环境还是有显著的差异的。

我看另外两个同学上线了，那要不让两个同学先介绍一下自己。

吕梦娜： 邵老师您好，我是吕梦娜，我在跟着刘老师一起做商学院还有管理学方面的一些研究。今天下午我非常期待跟您探讨一下有关的问题。

刘书博老师： 那个梦娜的大导就是做CSR的对吧？张老师，

吕梦娜： 对，是的，是的。所以非常期待后面的交流。

刘书博老师： 对，但是张老师是非常实证研究的、非常实用的，还是比较美式的。那邵老师做的就是非常欧式的。所以回头我们可以一块儿再探讨一下企业社会责任这个话题。

黄秋莉： 您好，我叫黄秋莉。之前也是因为您跟刘老师的方向可能比较一致嘛，刘老师也跟我们介绍过了，然后在organization studies上看到了您的文章，今天下午能够跟您有这种深入的交流，觉得非常的开心。

刘书博老师：好，那我们就步入正题。邵老师，我们第一个议题就是想要了解一下您早期的职业梦想是不是就是想要做大学老师，然后这种做学术的志向有没有受到其他人的影响？因为我们想知道您在选择读博这条路的时候是怎么回事？

受访者：我原来的志向是想挣钱，所以没有想过做老师。然后我结婚特别早，我23岁就结婚了，然后我老公想要读博。本来我们两个应该是同一年申请，我就觉得同一年申请难度比较大。就是那时候他申博我申硕嘛。哪怕我再读一个硕士，因为我那时候已经在北大读硕了，后来就说那我出去交换一年，正好那时候随手申过一个欧盟的奖学金，并且真的下来了。下来以后，我们俩就在认真的想，觉得他先申，申完了，我再申，这样也方便一点，就比较有针对性嘛。而且那时候我也不太想考GRE，比较懒。然后我就先去了瑞典隆德，在隆德有两个影响。一个是我到了瑞典以后，我觉得欧洲比美国好太多，就是我那时候就跟我老公讲，“你别去美国，你去了美国我也不去，然后也不要去北欧···”

刘书博老师： 为什么？你为什么觉得欧洲要好太多？

受访者： 就是整个社会制度好一些。而且（那时）我特别疯狂地想生孩子。我就是觉得，在美国那个环境下读完博，然后挣那么点儿钱，苦哈哈的，在某种程度上讲，我也不是一个能吃苦的人。我觉得在欧洲待着挺好的，然后整个氛围也蛮好的，又有福利制度嘛，那时候觉得养个孩子也没有问题。

那个时候我跟我老公说也不要申北欧，没有太阳我也受不了。然后南欧也没有什么好学校，而且我也觉得英国不算欧洲。所以其实当时可以去的地方不是很多，我对地儿特别挑。

那个时候我老公是学密码学的，突然他就发现鲁汶大学有一个特别特别牛的密码学小组，现在国际标准都是他们定的，然后一些教科书里的人在他们组待着，所以他就申了这个组。申了以后，我当时觉得鲁汶这个地儿也不错，离布鲁塞尔是20km嘛，就是从海淀到朝阳的距离。

我那个时候是学国际关系的，那个时候我就想着去布鲁塞尔找个工作算了，所以其实当时的计划是他去读博，我去那边找个工作。后来就发现很困难，因为那边要荷兰语和法语双语，而且布鲁塞尔的绝大多数国际组织都是要求会一门起码是英语加一门欧盟语言。我当时找工作实际上卡在了语言上，然后就觉得还是在大学里待着吧，而且在鲁汶城里觉得真的很舒服，在那边读个博觉得也行。

我那个时候就不想读国际关系的博了，因为对这个专业没有爱，而且也觉得不好找工作，我也不想去外交部。所以就觉得转个商科还行，正好那时候看到我老板在招人。而且我那个时候在隆德，硕士两年的课，其实有一半的课是在上方法论的课。不仅是学方法，更多的是在上哲学课。

刘书博老师： 对。这个课程设计跟我当年研究生还挺像的，我在华威商学院嘛，然后几乎好多内容都是在搞哲学。

受访者： 就是三论，本体论、认识论、方法论。我觉得你一旦学过这些东西以后，你就没有办法做量化研究了

刘书博老师： 就觉得他们那个比较窄了，就是其中一个小块儿。

受访者： 对，就是觉得他们实在太浅了，就是智力上毫无挑战，然后整个的世界观也极为狭窄。所以我那个时候就觉得要读博不想读这种纯量化的博，那当时碰到我老板就觉得很合适。我老板也是看我的简历，她也觉得很合适，因为她想做跨国企业的，而且做的就是非常偏政治学、偏哲学。而且像他们看到隆德的简历都会觉得，我是Alvesson教出来的。所以就是一拍即合嘛，然后觉得也挺合适的。所以我刚开始读博是这样的一件事儿，就是各种机缘巧合凑在一起。

然后一开始读了就还挺开心的，反正觉得生活状态也挺好的。欧洲是先要读硕再读博嘛，第一年还是有一点点课。特别对于我来讲也有必要，就是学OB、OT、HRM这些课，我觉得挺好玩儿的，但是我也的确发现我对OB和HRM就属于那种让我听听可以，但让我做这种研究，我觉得太微观了，我的确不感兴趣。

然后OT，我们当时是给了15篇经典文献，让写comments，就是读书报告这种

刘书博老师： 就是organization theory这一块儿，对吧？组织理论

受访者： 对，就是什么制度理论、批判理论，这些都读。所以我自从开始读博，首先所有的课我都修的非常好，像写这个OT的comments，我们当时也有考试性质的吧。然后基本上除了我以外，这些新的博士生都会有一两篇的feedback不合格，要求回去重读。像我的话，我老板说反正就明显的每一篇质量很好，所以我开始读博就挺顺的。

然后组里的也都觉得蛮好，我老板其实一直对我很好。然后正好也是机缘巧合碰到了CSR video这个事情，我老板觉得这个可以干。然后就开始做这个事情。所以我其实是博一上课，然后博二的一开始，从9月份就开始写初稿、做初步分析。然后就投了，并且中了。然后就觉得我读博的头一年半极顺利。而且在读博第一年以后，我当时觉得我根本就用不了四年就能毕业。我觉得我可以生孩子了。所以我相当于是投第一篇文章的时候就怀孕了。然后怀孕也没什么事情，就接着干活儿呗，然后跟我老板说了一声。我怀孕七个多月的时候去开会，就碰到了Michelle Greenwood，就是JBE现在的主编。然后他们就正好把我安排在Michelle后面present。几个chair就明显的看到我的研究逻辑和Michelle的那篇文章有非常强的一致性，其实我们是一个思考方式。

所以就是我们俩连着pre之后Michelle来找我，他虽然跟我老板不认识，但是互相就是神交已久吧，只能说是，就是他一听我是谁的学生，就知道大概我是搞什么的。所以在会议期间他就来找我聊了一会儿，也聊的很好。然后他就跟我说不能光做这种传统的linguistic或者是discourse analysis，必须要做multimodal，因为他自己也在做。其实我在头半年之前就跟我老板有过这方面的讨论，然后我老板觉得还是做省事儿的吧，做这个multimodal太费劲了，方法还找不到。但是Michelle这么说了以后，我就回去又找我老板说，“不行，你看我还是得做multimodal”。然后我老板说“那既然Michelle都这么说了，就这么做呗”，然后我后来就邀请他做我的committee。所以头两年就是一直还挺顺的，然后在我博三九月份就生孩子。然后我的人生就急转直下了。

先是各种休产假，然后回来上班儿，说是回来上班儿，但实际上心也不是很在那儿，现在想想当时那几个月应该没干什么活儿。然后坚持到孩子七个月送了托儿所，就是比利时这种西欧的福利制度，然后就是噩梦的一年，孩子基本上每2~3周发一次高烧，我们两个也是，因为我们对欧洲的病毒没有抵抗力。作为一个成年人大概在那一年里发了十来次高烧，所以那一年基本上也是废的，所以我博士读了很久。然后大概孩子快一岁半以后不生病了，我又真正开始接着去做研究，这就是2017年嘛。

就是总的来讲还相对顺利，然后每次开会基本上都能套到一两个大佬，因为大家都觉得multimodal有足够的合法性，就是所有做discourse的人都天然的会对multimodal感兴趣，所以就是觉得混discourse那个圈子挺舒服的。

刘书博老师： 所以在那边它是有一个圈子哈，就是你很自然的就可以融入到那个圈子

受访者： 对，因为我老板就是做CDA的呀。然后做质性研究、做critical的一个小的联盟。然后我们差不多每半年见一次。但实际上就是我老板和他的徒子徒孙们、还有他们的朋友们。

刘书博老师： ok，所以也有对应的发表的期刊和途径，对吧？

受访者： 没觉得是什么。像我老板基本上所有的学生毕业的时候都是一篇Organization studies、JMS、human relation的。

刘书博老师： Ok，都是欧式的，应该都是英国的。

受访者： 对。

刘书博老师：所以好像欧洲也不是特别看那个美国的UTD达拉斯

受访者： 也不是，我在2017年年底的时候投过AMJ。我老板其实表示无所谓，但她知道我特别想要一篇AMJ。而且他们也觉得合适，但是后来审了我四个多月吧，还是被毙掉了，我觉得人家说的还是有道理，当时那篇文章的positioning有问题，之后就投了JMS。JMS我觉得比较冤，怎么说呢， reviewer说的对，但其实reviewer应该是三个，有两个给了revision，一个没有给，然后editor拖了我六个多月，就属于那种“不催没结果，一催就悲剧”的这种。

刘书博老师： ok，那Organization studies这篇呢，整个过程顺利吗？

受访者： Organization studies这篇就非常非常流畅，那个主编我认得，就是之前开会的时候也见到过，所以他知道我在做什么，然后他自己也是做multimodal的。一般情况下主编不亲自上手审嘛，但是因为那个是multimodal的东西，所以他就直接上手审了。就是OS那篇基本上没有悬念，就始终都特别顺。

刘书博老师： ok，我看现在Organization studies、 human relation好像一大半、70%都是在做质性研究，发表的也是质性的。

受访者： 其实没有，我觉得Organization studies还是属于什么都收一点儿吧。Organization science可能比AMJ还过分。对，像AMJ特别是这几年，我觉得主编Tima Bansal还是比较open的（尽管她做的质性研究更多是艾森哈特那一套的）。

我们那篇文章最大的硬伤在于，他们当时说我们前面的theoretical part太强，它不是一个empirical studies，它更多的是对theoretical construction的一个illustration。

刘书博老师： Ok，明白

受访者： 这个是Tima Bansal是说的，我觉得她说的非常对。就是相当于我们前面实际上更多的提供了一个看待这个问题的视角，后面只是举例证明说这个视角是valid。我觉得当时的那篇我是服的，我并不觉得她是因为带了范式的偏见，我觉得的确是我的问题。

刘书博老师： ok

受访者： 当然有一点点范式的偏见，就是他们觉得这个数据量太小，这个显然是AMJ的taste的问题。

刘书博老师： 对，像您觉得范式的偏见是什么时候发现的？

受访者： 这个范式的偏见我觉得一直就有，就是很明显，但是在我们组是一个很奇怪的氛围。就是在其他组，总会有人告诉你说你先做量化，等你拿到tenure你再开始做你真的想做的事情。连我老板都是这个套路。但是在我们组没有这个问题，就是因为我老板足够强势，我老板相当于是组里的大老板嘛。所以他勒令所有进组的博士生，不管做什么范式，都必须要学三论，就是社会科学这一套都要学。

在我们组，positivist是一个特别明显的贬义词。我们组只有做量化的跟老板闹说要做质性的，从来没有反过来的。我们有一个小老板是做量化，做纯美式的这种。然后他就说他的博士生一天到晚跟他说，“做定量的人就特别stupid，就是抬不起头来，我不想做定量，我要做质性”。

刘书博老师： 所以那个氛围真的跟美国的是完全颠倒。

受访者： 对，因为我老板是那种智商很高，然后对笨人不屑一顾，然后毫不掩饰自己的这种特别极度的judgemental，对笨人没有耐心。然后她会在听别的博士生seminar的时候听到一半，然后问一堆问题，出门就走了。就是她不太给人留面子的那一种，就挺任性的一个老太太

刘书博老师：跟我导师也挺像的，我导师可能有点耐心，但是对量化的研究特别不屑。因为我导师博士论文是做实证，做经济学的。然后做完之后她再也不做实证研究了。

受访者：跟我老板差不多。我老板读博的时候是心理学背景，做纯定量的东西，发了AMJ，然后觉得也就这样吧，没有任何智力上的挑战，还是做一点有意思的事情。

就是这种范式的偏见，其实我们也一直都知道，那我们就不是很在乎，觉得他们就是特别傻呗

刘书博老师： 那真正在乎是你在回国找工作那会儿吗

受访者： 我也不是很在乎，我一直都觉得他们傻呗。

刘书博老师： 但也确实见识到了很多这种偏见嘛？

受访者： 我觉得还好。就是总的来讲我觉得还好。因为在欧洲也有会有人说嘛，但是我们就把他们直接打一个“傻子”标签，就这样了。

一个特别好的鹿特丹的商学院是纯美国的那种范式，我老板就反正也不喜欢，虽然她没有特别明确的说，但是她一天到晚的意思就是这种感觉。

刘书博老师： 对。我们前面的访谈也跟你分享一下，就是美式的，他们会觉得像你不做这个统计学和量化的实证的就是不够专业。所以他们其实也会有自己的一套说法。

受访者： 我知道，我觉得就是无所谓吧，我真的就是觉得无所谓。因为我自己接触到的青年学者，总的来讲，不管是真心或是假意，起码都会跟我表示说质性太难了，我们做不了，反正让我觉得挺不舒服的。

刘书博老师： 是，我也是经常这么听到他们这么说。

受访者： 不管是怎么说吧，我只在读博的时候碰到一个国内哪个学校的博士生，然后很牛哄哄的跟我说啊，那你们好幸福，我们不做量化都毕不了业。我就直接说“你们什么学校啊，你们是民办大学吧。”他说我们是正经985，我说什么是985？然后他说“你哪儿毕业的，你连985都不知道”。我说“我是北大的，那可能我们不是985吧”。除此之外，从来没有人说这件事儿。就是反正我作为一个critical scholar，我怼人是可以的，对这些人我是从来不留情面的。就除非特别大的大佬，站在我面前，那我还是要稍微的收敛一点，就是可能会憋死，所以我也不跟这些大佬打招呼，但是总的来讲，年轻学者我就上去怼。

刘书博老师： 怼的好，我之前面试985南京大学嘛，我做presentation，然后他们就没听懂嘛，就说你研究怎么没有变量啊，没有自变量，没有因变量什么的，然后你的reliability怎么搞？其实他们就完全是按照那一套实证研究嘛。

受访者： 然后这种学校我也碰到过。我面过两次政法，第一次面政法的时候就有一个老师，不知道哪儿来一个老头儿，现在都不知道是谁。我就记得是个老头，他问我说那你做量化吗？我说不做。问以后考虑做吗？我说不考虑。我后来听我们系主任说的，说第一次他们那老头儿就坚决反对。

那个老头儿肯定是觉得我态度不好。那个副系主任就一直在替我找补，说她怀孕了，她情绪不稳定。但那个老头儿就坚决不要我。当时我也无所谓，我一直就觉得非要逼我改行，那这个就是不合适，我没有必要去委曲求全。

刘书博老师： 所以邵老师您还是比较勇敢啊，很坚定。像很多老师会觉得那我就学一学量化吧。像我在英国的一个老同学，那他博士期间就做质化，现在就在学量化。当然他也不搞批判，怎么说呢？他就是为了发表。

受访者： 因为我其实没有什么事业上太大的野心，我就是想要好文章。而且我知道有我老板和Michelle 在我后面，我发好文章只是一个时间的问题。

就是我这篇 Organization studies能出来，说实话我自己是非常有把握的。我当时觉得哪个学校捡到我，哪个学校捡到宝了，你们自己不愿意捡，那跟我没关系。我自己其实是比较自负的，这跟我老板长久以来的这种培养是密不可分的。而且因为我对工作这件事儿吧，我只是想做我喜欢做的事情。我并不是说我一定要做到副教授、正教授，我觉得这跟北大的气质也很有关系。像当年在北大就有这种一辈子做讲师的，就只是因为喜欢这件事。所以也有好多人跟我聊天说我是一个特别特别典型的这种曾经的浪漫主义的北大人，不是现在北大光华的这种人。

刘书博老师： 我正想说北大光华很不一样，好像不是很“北大”。

受访者：对，所以我一直知道我自己在正路上，只不过可能有些人不理解。就比如我刚一回国的时候，我去见过xxx，就是光华的，他发了很多AMJ，发了science，是芝加哥社会学系的博士。我忘了具体名字，反正我当时就约她见了一下，然后就是跟她聊了一下，她就问我做什么的，我就说我做什么的。但这个事儿我觉得她看的很透，听完我做什么以后，她问我说你们家家庭条件怎么样？我说反正养得起我，我在北京有车有房。然后她说那你就继续做你这个东西吧，真的挺好的。她说，要是你家里是农村出来的，那我肯定不能这么坑你，我就觉得也很坦诚。她说送你一句话就叫follow your heart。我觉得就聊的挺好的，我一直没有怀疑过是我有问题，我一直觉得是他们的问题。

刘书博老师： 可能这个系统现在就变成一种把学术那种传统的模样改成了一种工业化或者是商业化的体制了吧。

受访者：对，反正你像咱们就是都想要paper嘛，也很正常，大家都是想要paper。像我找工作也没有觉得特别困难，毕竟客观、坦白来讲，我觉得我的第一学历帮了我很大的忙，因为很多学校是认这个的。因为我是北大的本硕，所以我没有觉得在找工作这件事儿上有特别大的压力，然后我老板是xxx（某欧洲顶级期刊）的editor，Michelle 是xxx（某欧洲顶级期刊）的editor，我觉得光是这两个editor，再加上我们组的教授是鲁汶大学的校长，就是这些后台摆在一块儿，就已经足够，我哪怕没有publication也足够我当时拿到offer了。所以我没有在这个market上挣扎太久，我一直就是觉得还行吧。像那次就是因为那个老头儿不同意招我嘛，后来我也觉得完全无所谓。然后是我们系主任很想叫我进来，然后他就在第二轮面试的时候又悄悄把我塞了进来。然后那个老头儿也不知道，或者是换了一拨人面视，我也不知道怎么回事儿。反正就是既然系主任这样叮嘱我说“别太刚，他要问你，你就缓一缓，进来以后没人管你”。然后就像这种事情，我就觉得没有必要刚了吧。我当时就是已经沟通的比较好了，我知道我有足够的学术自由，这些问我的老头儿都只是院里的领导，他又不是直接管我的人，所以我就可以缓一缓。第二次就把我招进来了嘛，招进来了以后也的确没人管我呀，就挺好的呀，还挺愉快的。反正我的体验特别不一样，就是我觉得就是第一学历帮了我，然后老板们比较给力也帮了我。

刘书博老师： 那邵老师您觉得，像咱们这个学科中一个优秀的学者应该是什么样子的？或者比如说有一些这个偏见，说做案例研究的就是次一级的或者不够专业、不够精细的。那您对好的学者，您觉得怎么判定？

受访者： 我觉得的确是有这个问题，因为我认识的国内的个别做质性研究的的确不太行。像有很多做质性研究，只是因为他不会做量化。就是他们发的那些东西我也没眼看，所以我是挺理解国内为什么有这样的偏见，然后我觉得就是慢慢儿来呗。因为真的跟我聊的，特别是年轻老师，我去面过几个学校，不管要不要，我就是跟年轻老师都聊的特别好。然后就会有年轻老师表示说，好羡慕你的生活状态，或者好羡慕你的研究状态，可能是商业互吹，但我宁愿相信他们是真心话，因为我也觉得我做的东西比他们做的东西有意思太多了。

刘书博老师： 确实是啊，如果做过量化实证，就觉得那东西简直是消磨人的生命，没有任何趣味性。

受访者： 而且我觉得我自己做的东西我真的很感兴趣。然后就是真觉得这种生活状态也挺好的。而且像跟我聊的有一个就是老师，虽然她做量化，但她导师是做质性的，就是总会有appreciate，虽然看不太懂，但是比较appreciate质性的这种人跟我聊天儿。我觉得政法这边整个氛围也挺好的，就是教研室的人都表示不知道你在干什么，但是就是好像还行。

刘书博老师： 那现在学生他们要判断，比如说他比较单维度啊，就是大牛的话就是发文章呀或者基金呀这方面的结果非常好，那么他就会认为是很优秀的大牛。那像您做判断主要还是看他写的这个文章的内容，对吧？

受访者： 但有些人比如说xxx，我是特别特别服的，我做那种量化的话我也做不到人家那份儿上， 就是我觉得真的挺棒的。 就是我觉得还是看治学的态度，就比如我跟xxx老师也聊的蛮好的，他在西北工业大学，他是xxx的一个副editor，所以我认识他是通过Michelle认识的。你可以想象因为Michelle的关系，所以虽然他看不上我做的东西，但他对我还是比较客气的。

刘书博老师： 他看不上是因为范式吗？还是别的什么原因？

受访者： 对啊，因为范式啊。

但他起码因为Michelle的原因，他知道就是有大老师站我这边儿的，就是我不是很productive，但是我做的东西也起码是real thing。 所以我跟他也聊的挺好的，就是我们俩互相范式上不理解，但是我觉得还是基本的尊重，我也很尊重他，就是因为我觉得他治学真的挺严谨的，然后也很勤奋，就这种我都挺尊重的。我觉得范式上的差异，其实是小的差异，真正我不能接受的是那些你不知道他在干什么,可能就是写本专著，然后跟你说专著比publication和article有用，专著才能体现学术功底，或者是不发SCI，发SSCI，就是这些奇怪的人见多了以后，我觉得范式的争执都是小争执。所以我觉得像我跟我们教研室的那个北大光华毕业的姑娘，其实在范式上也完全聊不到一起，但是就求同存异呗。

刘书博老师： 对，我觉得这个方法论是多样的，但是可能那个好的治学的态度应该是一致。

 受访者：对对，是这样的，我现在也并不会觉得所有做实证的都是因为傻，但是我觉得很大程度是因为无知了，就是没有受到良好的哲学教育。

刘书博老师：ok，好。我看时间还就剩五分钟，要不让两个学生有什么问题赶快问一下。

吕梦娜： 邵老师，我就接着刚刚刘老师那个问题再稍微追加一点哈。就刚刚也提到了，就是您事业上可能不会追求要当副教授或者教授这种，只是想写好文章，包括治学的态度严谨。然后我再进一步想问一下，就是您作为老师或者作为学者的话，您对自己的这个职业有什么定位或者有什么期许吗？

受访者：我其实有很明确的这个role model，就是我老板和Michelle，作为FT50杂志的editor至今也是个副教授。当然我老板其实要更aggressive一点，她在美国读的博，整个人更像美国人一点。刘老师，你看我老板面相你就知道他是一个很aggressive的人，你能想象是吧？我觉得就是像他们这样做一些自己感兴趣的研究，然后你在小领域内大家都认识你就可以了，这是做研究方面。我在研究上就希望我能做到她们俩那个样子，发一些好文章，就是我还是很想要好期刊的。我对AMJ执念不强了，但我很想要AMR。但是最近的AMR的editor不是特别的friendly，就是感觉更偏positivist一点，实际上最近AMJ 其实有很多这种。但是我还是很想要好的期刊的，但我并不期待在国内有多强的学术地位。然后我觉得职称还是得拿啊，要不然很麻烦。

吕梦娜：这是您作为学者方面的一些期待，您作为老师呢？因为我听我政法大学的同学说，您对PPT都很讲究，就感觉你是一个很严谨的人。

受访者：我觉得我特别擅长开学术会议，我基本上每个学术会议都能套到大佬。这么说吧，一个就是因为我是中国人，然后我做质性研究，我做critical，这些条件在那个圈子里就像团宠一样。就是那个小圈子里大家都对我特别好，因为他们觉得我是他们散播到中国的火种。就是因为这，我觉得他们的确就是会给我很多资源上的倾斜。所以我觉得开会还是很重要的，就是你让人觉得你起码是能扶起来的吧。

但是带学生方面，刘老师有勇气带，我完全没有勇气带，我不知道怎么去带，因为我带学生我不确定我能给他们什么，或者说我觉得我现在没有能力带学生发一个很好的期刊，但是我又不愿意浪费我非常非常有限的精力去发一些不那么好的期刊。所以这个事情只能说是我觉悟没到，能力也没到。所以我自己是在刻意的回避带学生的，我只是没事儿对组里的硕士生、博士生，对人家指指点点，但是我自己是坚决不带的。就是因为我觉得我精力非常非常有限，你现在让我去发一些我都没听说过的期刊，我也不太想发。

刘书博老师：其实还是说明政法的这个环境挺好，挺包容的。像有一些学校，比如对外经贸可能就没这么nice。

受访者：我们就是六年要出五篇C刊嘛，一篇A可以顶三篇C。我觉得我再过一两年再出一篇FT50应该问题不大。而且我觉得像政法也不会真的开除我，历史上还没有真的开过人啊。当然我们最早的一波考核期也就才三年。但是就政法这个level，我直接一点说，我觉得这就是一个低就的好处。就算我再也不发文章，我好歹是全院唯一一个发过FT50的人。他们就真的因为我数量不够敢开了我吗？就是很坦诚的说我就是这么一个态度，你看不惯我你开了我呗。所以就是因为无欲则刚，我对事业没有太多追求，所以他们也不能把我怎么样。我觉得在事业上我真的状态非常非常的好，一个是政法这个学校氛围比较好，我们的小环境非常的好。而且不得不说有一篇FT50，你的底气会硬很多。像我们学院没有人会怼我的我学术能力不行，或者说我做的不是研究。所以我也不想往好学校跳，跳过去了，真有人能发UTD，他说你做的不是研究，那你咋办？我就在这个地方窝着挺好的，就是什么时候我憋出来，我再说这件事，所以大概我就是这么一个状态。我觉得我这个状态吧，就是刘老师问我，我会觉得你访谈我得不出啥结论，就是你将来如果要发问卷什么的，你可能问不出来什么具有普适性的参考价值。而且我觉得特别重要的一个事实就是，就像北大那个老师问我的，就是我家里条件也不错，我老公是做区块链的，没有人指着我挣钱，所以这个我觉得必须要承认。就是像我跟您之间我觉得一个非常大的区别，其实是性别角色的问题，就是我没有养家的压力呀。我们现在可能出于这个养家的压力，必须要向这个主流范式屈服，但我没有压力呀。

刘书博老师： 是，所以现在很多人都是身不由己的，做那些我觉得毫无意义的研究。

受访者：是的，所以我对他们与其说是敌对，或者是鄙视，其实更确切的描述是我对他们抱以同情。

 一个是我觉得就没有受到这种良好的教育，你不知道这个世界有多么丰富多彩；一个是哪怕知道了，大部分人也没有这个勇气，但是更多的不是能力，更多的我觉得是投胎问题，反正是一些其实不是他本人的问题。

刘书博老师： 对，那现在的学术环境可能跟那种传统的已经不太一样了。

受访者：为什么我老板和Michelle能活成我的model的这种样子，我老板的老公是做咨询的，Michelle的老公是大夫，我们小圈子里会说，就是每一个critical scholar都需要一个rich husband。

刘书博老师： 我觉得卡尔·马克思不是也一样吗？他是有一个rich partner恩格斯。

受访者：所以你看这个圈子里，为什么女性会稍微多一点？我觉得这是个很重要的因素。所以这个事情很多时候它不是能力问题，它是一个就是有没有条件的问题。只是因为我运气很好，所以这个体制现在并没有打压到我，没有迫害到我。

刘书博老师： 好的，邵老师，那不耽误你更多时间了，赶快赶班车，我们有机会线下再找机会聚。好，那我们今天就到这儿。

# 受访者15

受访者：我觉得管理实证研究是从经济学过来的，然后管理学呢实际上现在也用了经济学的思路，很多的管理学者都是经济学的背景，比如说战略里面的国民经济，波特他那个思路，哈佛学派原来就搞经济的，然后搞产业经济学，就搞小一点，然后呢就搞管理学。其实我一直有个观点，经济学跟管理学，这是两个学科，就像是物理跟化学的区别。很多人理解不了，就老是叫经管系，这就叫错了。某种程度上来讲，是把两个完全不同的学科弄到一块儿了。

经济学其实有一个最重要的假设，认为个体都是随机的。你看我们在做实证研究的时候，其实有一个很重要的假设，就叫做样本要来自于总样本，对吧？要有共同的期望值，而且呢更严谨的共同的方差，对吧？这个齐方差的要求是很严谨的，但是管理学本质上不是这个东西，其实管理学一直追求的是什么呢？就是与众不同，就是要把别人打败，就是跟别人不一样，所以管理学的初衷根本就不是说跟别人一样，而是说我跟别人不一样，其实有很多的这个数据呢也很不一样。

虽然大家按照这个约定俗成的规范来做，但是其实我个人呢很不认同的，为什么呢？他们都把所有的上市公司看成一样的，然后找出一个因素怎么影响另外一个因素，其实我觉得这个某种程度上完全是错的，严格的讲就是一个错误的路线。你比如说，纳斯达克前后上市了将近七八千家的公司，现存的大概几千家吧，但是纳斯达克前50%的市值，大概只有五六家企业创造的。所以说管理学里面其实它本质上是一个什么呢？是个创新，他就是与众不同，就是说你要能成功就得与别人不一样，那么其实我们要做的也是这样。所以说本质上你用实证研究出来的东西，基本上可以这么说就是错的，对不对？这逻辑上不是这样。

但是大家现在不太管这个，因为它逻辑上有它的数理逻辑的严谨性，但是其实严格地讲也不严谨，因为刚才我说的，企业不是同一样本的，本质上假设就完全不存在，这个完全是一厢情愿。昨天博士生开题答辩，他们研究一个问题，就是说年报的文本分析跟这个股价之间的关系。我说这个东西你是能研究出来一点点关系，但是呢这个关系实际上它只是一个非常非常微弱的，在现实中根本就不重要的、人们不care的东西。比如说我跟他们举个例子，我估计他没听懂，我说比如说吃鸡蛋，早上一天一个鸡蛋，那肯定对人有好处，我研究出来早上一天一个鸡蛋的人肯定会长得高一点、长得壮一点。但是呢可能这个影响只是影响我身高的1mm，甚至零点几毫米，但是大家就说啊这就不得了，实际上可能我们直觉上都能判断出来，可能跟你的生活习惯、跟你的锻炼很有关系。但是你看那个论文，从现实中其实我们会发现锻炼可能会影响我们几公分甚至十几公分，但是你那个鸡蛋就影响1mm。

有可能这个很琐碎的一个问题会做出很漂亮的一个论文。但是呢大家现在都这么做，所以后来我就说你也得研究强度问题，就是说文本做好了到底对股价有多少影响？一个（百分）点、两个（百分）点就没有用。

其实这个问题啊我个人认为我想明白了，但是呢实际上现在大家都不做这个东西，就是说因为它不出成果，就是说他要有一种理论上的自洽性。

其实我们商学院很多时候做的一个什么事儿呢？是对于人的甄别划分。比如说商学院要培养一个人才，其实我们对人才是用的一些代理变量来判断的。比如说我给你出一些很难的（问题），就比如说数理分析，你能把这些稀奇古怪的东西搞明白，这本身就是能证明你是ok的，对不对？就好像我们要选拔一个人才的时候，我们会弄一个游戏，这个游戏实际上本身没有意义，但是它是一个代理变量。所以我后来开玩笑说，你们商学院的学生来了，就光下围棋也能选出好学生，而且选出来的结果也不错。比如说我们商学院就是说，谁下围棋下的最好，下的最好的学生，我们给他最好的工作，没有错的。

刘书博老师：就不用围棋那么复杂，五子棋也行哈哈

受访者：对，五子棋也行，什么都行，或者就口算都可以。但是呢这里面有个问题，就是说你不能太low了，对吧？还是要搞的复杂一点，实际上商学院的功能我认为从现状的功能来讲，它某种程度是一个分层的功能。就是一个筛子，这个筛子是什么无所谓

刘书博老师：所以那个《精英的傲慢》那本书他说了，我们是个分类机器，

受访者：对，就是分类机器。那么实际上这里面就延伸到另外一个问题，就是对于一个社会来讲，其实分类比发展人才更重要，因为这个社会是需要分类的。

刘书博老师：嗯

受访者：其实分类本身就是管理学的一个基本部分

刘书博老师：对

受访者：很多人就说那这个就好像没有意义了——你应该培养我。其实不是，其实管理学一个最基本的功能就是要分类，因为分类是人类思维的最基础的一个功能，就是你干什么事儿首先要分类你才能去想问题

刘书博老师：是的

受访者：我们管理也是首先要分类。比如说我们管理学里面最古老的命题就是分工和协同，实际上你要说到协同首先要分工，你不分工协同个啥嘛，对吧？所以说分工这个问题就是个分类，这个东西其实是更重要的问题。所以这么说的话，商学院的功能是有的，就是分类。在中国来讲，更需要分类。因为中国是一个人口很多的国家，就是说其实这个人类社会呀，为什么东方跟西方有差距，其实在几千年以前就是因为一些地理环境的原因，包括人种的演化，它导致两种社会的演变模式，所以现在不能用“进化”，其实那个《进化论》的翻译是错的，应该叫演变，《天演论》，叫“物竞天择，适者生存”

刘书博老师：他没有说是进的高级

受访者：对，你看中国人的思维就反映出来了，我们就叫它《进化论》，就等于在发展，其实错了，这个很多人已经提出来了，它是演变，适者生存，适者生存是什么意思呢？不是说变强，所以我们老觉得老虎就是生物链的顶端，我们老是这么说，其实不是老虎。从自然界的角度来讲，老虎的适应能力可能比老鼠差多了

刘书博老师：对，而且最强的可能是蟑螂哈哈哈。

受访者：对，实际上我们人类跟老鼠比，我们是成功的嘛，不一定，老鼠已经存在大概有一两亿年了，它在那个时候就是拒绝进化，它就在那个层次演变。你说人类就能生存2亿年嘛，我觉得不敢说。所以我讲这个是什么意思呢？就是东方的社会跟西方不一样，西方呢强调发展，就是不断地把人变得更加strong，更加有攻击性，因为他们更快更强。东方呢就是分配资源，就是东方实际上一直在协调资源。中国就是要去分配资源、协同设计，我这是有数据支持的。中国在三千多年以前，人口密度一直就比西方要高，大概要高5~10倍左右。汉朝的时候，长安大概就有100万人，同时期的罗马，包括再早一点的雅典没有那么多人，那时候他们一个城有个一两万人，就属于是大城了。但是我们就是这么多人。你想啊，如果说这个城里有100万人，那个城里只有几万人，那那边大家的生存方式是什么呢，就是找食物嘛，因为他人少；然后呢这边呢就是协同，就是说我们要求的是稳定的生产方式。你比如说中国这个地理环境，就说农业啊，农业实际上要有一个稳定的生产周期，那时候基本上要大半年才能把庄稼种了收起来，那这个大半年之间实际上就要求要稳定的社会环境。所以很多时候中国的文化都是这么来的，农业文明，所以这个时候谁强并不重要，还是怎么样有序。这是我觉得中国文化很博大的一个特点

刘书博老师：这就是农业文明的特点。

受访者：对。

刘书博老师：我觉得我们在农业社会总是被北方的游牧民族给侵略，或者甚至是打败，可能也是跟这个···特别因为他们游牧民族就是侵略性的，

受访者：游牧也是一种生存方式。游牧人过得没有我们好。嗯，但是为什么我们不去发展呢？按道理来讲，我们生产力更好，人更多，为什么不这么干呢？其实这里面就用了经济学中的一个原理，就是交易成本嘛，就类似科斯的那个，他用这个解释一个组织的边界，就是解释组织行为，就是说我到底把这个东西放在市场，还是放在我企业内部，那取决于什么，就是你的交易成本跟你的组织成本之间的比较，对不对？实际上中国的这种生产方式，包括边界也是这么规定，比如说这个农耕民族一定不会到那个苦寒之地

刘书博老师：北纬40度再往上就不行了

受访者：生产效率就是水热光都不够，它不合算，这是一个。第二个呢，就是说为什么我们不搞强大的国防？因为北方民族很简单嘛，人又少，我们就用人堆也（打得过），但是实际上这是不合算的。为什么呢？有一个数据，你看宋朝的时候，一开始他们也挺能打，包括汉朝也有马。但是后来呢实际上这里面涉及到一个问题，就是马作为一种很重要的军事武器，那时候就相当于现在的核武器，这个马的战斗力非常强。其实在春秋战国的时候，游牧民族不行的，游牧民族是被汉族压制的，那时候游牧民族跟汉族散在一块，因为那时候的马是没有被驯化的，没有发明那个马鞍，所以不能用来打仗。

其实一开始马是不能够用（打仗）的，只能吃，这个时候就没有意义了；然后呢，马就可以骑，但是骑的是光马，骑到马上跑过去以后，然后大家都跳下来打仗。这个时候呢马的威力已经出来了——移动性很强，但是马的杀伤力还有限。其实是汉朝的时候出现马墩了，这个是可以考证的，马鞍跟马墩是人类历史上一个极其重要的···，实际上这个发明影响了整个世界2000年的历史。因为一直到成吉思汗，一直到工业时代就是火拼。但是骑马可以在马上斩杀

刘书博老师：这就是骑兵

受访者：非常厉害，因为第一个，移动速度是步兵的n多倍。第二个呢，他居高临下，因为那时候打仗就靠力气，他借助马的力气，一下就可以给你斩杀穿透，敌人根本扛不住，因为他有马墩了，他就可以站得住，就可以站在马山活动。还有一个，马本身是粮食，你看蒙古人征战欧洲的时候，他一个人带三四匹马，就是换着马骑，在路上把马给宰了，就吃马肉，所以说，这个简直就是一股泥石流啊。

但是回到原来的问题，汉人为什么不这么干？因为不合算，那时候养一匹马要是用农耕的方式来生产的话，可以养6~10个人，因为马在吃草啊，他在那里瞎吃，他吃的又多，对吧？这里其实就要算一比账了，养一匹马不如养六个人到八个人，我就用生产的东西跟你换，我就是买这个。所以说我们中国一直用纳贡的方式去换取与北方民族的和平。

所以北方人呢一般是这样：一到冬天了，他们要打谷草，就从北方跑到南方来抢一顿。我们做的事儿呢，不是抵抗他们，而是我们把自己养肥了，你抢一次也搞不死我，对吧？或者六个人，死了三个人，还剩三个人呢，比我原来只养一个人还赚了。但是这个就养成一种特殊的文化，这个其实是文化本身的一个本质，就是“适者生存”，“适者”所带来的影响，其实中国很多的文化都是因为这个原因。

刘书博老师：胡老师说的很深刻，就是我们现在很多现代社会的现象就来自于那个文化，那个基因，或者农业文明的基因，我们是要追求演化而不是进化。要适应，只要活着就行。有点像追求一种蟑螂似的存在，而不是恐龙的那种高贵的存在。

受访者：中国的管理思想讲究次序，就特别讲究次序，次序比去发展个人能力更重要

黄秋莉：嫡长子继承制

受访者：对对，这个是其中的一种表现，但是不管怎么样，首先次序说明你干什么事儿你都得名正言顺，所以这是一个。第二个呢，我们要解决实际问题，就是对资源的珍惜，或者说对生存的追求，这时候个性就变得不重要了。

刘书博老师：其实我觉得像日本、韩国他们也是受到东方这个影响的

受访者：但是他们在这个夹层中间，他们实际上是一种杂交文化，这就像文化意识在演变，包括中国现在也在演变，他们是属于一种杂交文化，所以他们找到了自己的一个缝隙。但是真正这个世界上几大文化主体，一个就是欧美这一块儿，第二个呢就是东方这一块。其实印度现在都有点变了，因为印度被英国人占领的时间太长了，我觉得他们已经找不到自己的那个方向了，他有点懵。现在呢我认为这个阿拉伯文明是有点退化的，他一直拒绝改变，但是他们呢还是比较坚守自己。

其实现在世界上两大有活力的文化，我觉得就是欧美文化跟东亚文化。但是这里面就有问题了，很多时候人家说东亚文化好像低一点，好像就是强调次序，好像一种“守”，一种被动，一种保守，一种隐忍的文化。这种文化在18世纪~20世纪的末期是被大家唾弃了。

我不认可这个东西，为什么呢？因为这段时间是人类扩张最快的时候，基本上那时候大概从十几二十亿人扩张到将近80亿人。所以说在这种环境下那人就雄心勃勃，自己是天之骄子啊。很自然的，以守为本的、以收敛为主的文化，就是劣势文化，就好像变成一个次等文化。其实我们再看啊，现在人类已经到80亿人了，你现在想人类还可能再变成300亿人嘛，不可能。所以说我为什么说中国文化它有它的价值呢？因为现在整个全人类其实又开始进入“守”的状态，你看包括现在“双碳”，这些东西就是人类自己在收敛了。

而且我们看到各个大的国家人口都在下降，中国去年是第一次人口下降，实际上人发展的程度太久，自己也在收敛。所以说中国文化的价值其实在未来的几百年，我觉得甚至是永久的，有它极大的合理性，就是说你怎么去守。实际上我不太赞同这个西方文化，西方文化一直是攻击性的

刘书博老师：攻城伐寨式的

受访者：对

刘书博老师：发展作为核心，而且是人类中心论

受访者：对，但是呢那个时候整个的欧洲只有两三亿人，全世界那么大地方他当然是没完没了的，他觉得世界太大了，随便到哪儿都有发展。然后呢中国精英也接受了西方文化，因为精英就那么几万人，他觉得全国几亿人那够他去弄的。所以说我对精英说句老实话，我有时候不是很赞同他们的思维，他们过于的自我了，应该某种程度上来讲是一种偏自私的意思。现在的企业家很多人都觉得受约束了···

刘书博老师：他们其实就属于西方那种攻城伐寨式的，凸显自我，凌驾于他人之上

受访者：对没错，那就是抢嘛。但是这个问题就是当整个社会都在膨胀的时候，世界在膨胀的时候，是ok的；但是现在全人类处在水深火热之中，你抢别人，别人往哪去呢？

刘书博老师：所以这个就解释为什么美国特朗普时期民粹主义在盛行，反而希拉里那种代表精英的就没被选上

受访者：对，我觉得民主党也有一点弄糊涂了。他那个美国的问题啊，我认为他有一些根本的问题，就是说原来呢是美国一个国家吃全世界，全世界有的吃。但是呢，中国确实阻击了他，中国等于把中低层的产业截胡了。那中国人为什么这样？就是中国人的生命力在这里面起作用了，这个又是一个话题了。但截胡了以后呢，他就在国外的那个空间受到了挤压，就是实际上美国就这样。然后他一受到挤压呢，其实美国内部又是一个分层的，原来如果说世界足够大，美国内部的那些底层人还是可以在一块混的，哪怕在门口站个岗，也可以一年拿几十万，没问题。

但是如果底下截胡了以后呢，他发现他们国内的人就不行了，就反弹了，就内卷了。现在很多的产品，中国人制造一半甚至更多，全给他截胡了。然后呢这个中国还跃跃欲试的，还要再往上掏他的这个经济基础，所以他们就很打压华为嘛。原来像手机啊、电脑啊欧美人随便生产一台东西，就能养起十个二十个看门的，他现在养不起了，那怎么办？那就内卷。所以说我一直有个观点，西方文化和东方文化应该互相吸取对方的精华，当然东方文化也有问题啊，但是呢我觉得东方文化的这个意义啊就出来了，就是说包括我们比如说次序啊，包括我们这个整体观啊，包括个人跟集体的关系呀等等，实际上呢它有它的合理的地方。

所以我觉得商学院它的作用其实就是内部在分层。但是这个分层我们要知道他在干什么，我觉得就有更有利于来做这个事儿，因为这个分层就马上推行标准化了，就像说考试，考会计或考什么大家都没意见，但是你要是考围棋肯定就反了天了。

东方文化强调对权威的尊重，你看那个孔夫子就是一直这么说的，这里面还有个分寸问题哈，这个中国文化确实后来也有点走偏了。其实我觉得要回到《论语》的那个时代，这里面就讲我见到君应该怎么办、见到大臣应该怎么办，他就讲我一定要小心、要尊重他，但是呢骨子里其实我对你还是有判断的。就是你要做的不好，你要是不仁，我就对你不义。尤其到了孟子的时候他就更牛了，孟子说了，你君王治理国家我给你提意见，你不听就拉倒，反正这个事情归你管，但是呢你要到村子里面，我是长老，你得听我的。所以孟子其实就把孔子的那一些比较理性的、或者说有一点偏积极的东西啊，他发挥的比较多，所以说孔孟你要结合起来看，这是最好的。但是呢基本上中国人儒家思想（或者东方思想）对权威的最地道的一个态度是什么呢？叫做尊重而不盲从。

刘书博老师：对对是这样，现在有时候他会变成一种权力的发声筒，就把孔孟变味变成权力的话语，但是孔孟他们其实讲的是你对我不仁，我对你不义，不是说你对我不仁，我还是盲从，还是下跪什么的。

受访者：对，你看一直到唐太宗的时候，水能载舟亦能覆舟。后来为什么走偏了呢？这里面有法家的思想，所以说儒家的法家化实际上是走的过头了，从董仲舒开始，趴在地下咣咣咣磕头，那样的就有点过头了，实际上这个是从汉朝以后呢就走偏了。

实际上中国呢严格的讲叫威权，不能叫集权，但是走到集权这个程度我认为有点过了。所以说这个东西有点敏感了，就是说走到什么程度。但是这个社会一定要有一个层面是可以公开讨论的，就是要有一个组织，就比如说我可以议论君王，但是你不要到处议论，所以说我认为在帖子上议论是不好的

刘书博老师：是不是议会制度的那种。

受访者：对，就是那种我们可以在一个屋子里，在一个场合里面，比如说我们在学校里面就师生之间可以随便谈，但是你们说出去我认为就不好，就说到你要在帖子上发那就不一样。这一点和西方还不一样，西方是“我怎么想的我在哪儿都可以说”，这个我不认可，因为这个就把次序打乱了，这个分寸你可以再掌握，比如也可以说到了教授级别才可以谈，副教授都不可以谈等等。反正你只要有一个度就可以。其实我们中国现在的政府也是按照这个逻辑来的，但是执行起来就不一样了。所以我们中国现在在管理理论这一块存在着一个重大的问题，就是有一点冲突，这个冲突我觉得是东方跟西方的冲突，就是说我们采取不同的管理理念

刘书博老师：其实意识形态是不一样的

受访者：对，这个就是意识形态，然后在内部呢也有冲突，内部呢是极端法家跟儒家的冲突。实际上我是最喜欢孔子的，我每次看完论语我都觉得挺舒服，我觉得孔子是很可爱、很有思想的，那么多年以前他就用很多的实例来解释管理思想，他说话很生动。你比如说它里面提到次序啊、威权啊等等。你看现在新加坡就做的比较好。新加坡下面的人该遵守遵守，但是呢也有一个讨论的地方啊。还有一个权变理论，孔子从来是什么都可以变。还有很多的思想，比如说民生思想，民生思想就是维持这么多人，但后来到法家就是军大于民，这个是错的。但是后来的君王一直按照韩非子的那个书来走的，这个书一直没有被认为是一个正统的东西，它就是变成了儒家的黑货。有的时候君王偷偷的看，如果让大臣看到了，就说我是随便翻一翻，这个都是很丑闻的，就跟看一些暗黑的书一样。像那个军大于民，这个东西是不能说的。但是实际上呢后来确实很多人这么干了，这也是后来东方思想的局限性，就走着走着也出问题了。所以说我觉得商学院其实如果能把自己中国的这些东西给···

刘书博老师：对，我们这块儿空白的，好像那个工业革命之前那一块儿完全没有。像我现在教《管理思想史》，几乎从工业革命开始，前面就很薄的一点点介绍，没有挖掘

受访者：而且在挖掘的时候它里面有个坑，这个坑呢就是法家。极端法家思想是东方文化的一个坑，这个坑不仅是让西方人用来埋葬东方思想，也是我们东方人的一个坑，就是我们走着走着掉进去。

所以我现在有时候也不愿意说，有的时候你一说，人家就说你原来是极端法家思想，这个东西很暗黑的。最后你就变得你无言，可是你很难把他们界限给拉开。实际上呢我不是那个意思，我的意思是说你得找到东方管理思想里面的精华，现在还仍然有价值的东西、对我们有启发的东西。

我跟你说我现在是迈不过这个坑的。所以说呢，这个坑儿要给他揪出来，不仅要跟西方的精神区分开来，别（让西方人）把我（我们东方思想）往坑里扔。第二个内部的人，也不要用这个坑来陷害我，只有跨过这个坑，你才能重新发掘思想。

我认为现在整个人类社会又回到了这个“守”的、收敛的阶段。

刘书博老师：所以《精英的傲慢》最后一段他会讲到一个关键词叫谦虚，谦逊，我觉得农业文明就讲究这个，人不要太张扬，一定要得尊重自然，尊重身边的这些

受访者：这个都是谦逊，就是他要有次序，这么多人他就要分配资源，当然要谦虚了，因为谦虚才有个好的姿态，你才能把这事儿干下去，如果大家都抢那就不行了。所以说我们在讲契约关系的时候，在西方的话，任何两个个体之间的契约是没有缝隙的，就所有的权利义务责任是严丝合缝的，中间没有灰色的地带。但是在中国来讲，两个人之间的关系一定有一个灰色的地方，这个灰色的地方是什么呢？就是我跨过我要做的这个东西，然后呢对方也跨过我这边，就是说互相之间有一个缓冲地带。我给你举个例子，如果说你只有十个人，大家互相两两签合同， 10×9÷2，是吧？我们都ok，人少可以。但现在想要100个人两两签合同，你要签多少份合约关系？所以这个时候最好的办法什么呢？我们每个人就是模糊一点，这个东西好像是我的，我往后再退一步，这个东西好像是你的，你再往后退一步，ok。这个中间大量的东西就变成了一种简约的、弹性的模式。实际上讲契约这个事儿，中国跟西方其实都讲究，有的人说中国人不讲契约，其实错了，中国人老讲信，对不对？但是一讲到这儿中国人就词穷，因为你要讲信，他能举出大量的不讲信的。但其实是误解这个契约的，或者要讲的这个理论呢，实际上东西方都是很关注的。但是呢西方讲的是狭义的契约，就是权利义务都规定的特别清，中国实际上是一种叫做弹性契约，你说心理契约也可以啊。心理契约这个词是可以用的，但是心理契约基本上还是在西方语境下的。

刘书博老师：我们是讲究说仗义呀，义气呀，是不是就等于是契约？

受访者：叫做非对称契约或者叫不对称性。这个不对称契约是什么呢？第一，我们这个契约可以在不同的人之间定，比如说西方一定要订契约，两个一定都叫法人，国王跟平民在契约关系上是对等的。但中国不是这样，中国人定契约所有的都是不对称的。所以说一个富人跟一个穷人，要尽的责任是不一样的。你比如说我跟我的领导之间实际上是有契约的，但是这个契约就是你要做的事儿跟我要做的事儿是不一样的。比如说我举一个例子，柳传志这个事儿，柳传志我后来是有点否定他的，他按照契约来讲是没有问题的，但是中国人不是这么认为的，中国人就是说你有多大的能力、就要尽多大的责任。不要说你当时跟我签了契约，即便我签了字也不算。为什么呢？因为我们不对等。你不能利用你的权利优势来签这种协议。说白了你再怎么弄，你再合法的手段，我就认为不对，因为我们根本就不是那种对等的情况。我们的契约是什么呢？就是说你能力比我强，你可以过得比我好，但是你不能太好，就是这个意思，对吧？不能太欺负人。就是这个逻辑叫非对称契约。

然后呢我们做的事儿按照老百姓一般的观点，你可以拿1000万，但是你拿500万，ok。然后呢老百姓觉得你能拿1000万，但是我容忍你拿到2000万，这事儿就很简单了，你看中国的事儿就很好办了，这就是中国文化，就像你说的谦虚忍让隐忍，都是这样。你不能说合同上没写我就干，那不对，中国人不认这个，所以你在做事儿的时候就有这个中国文化，一直到现在都是这样。包括父子之间的关系，你有钱了你就要多花，你没钱了你就可以少花一点，都叫好父亲。但是你有钱不花那就不行。但是呢国外就不是，反正你只要18岁，我都可以给你赶出去的。

还有一个呢中国人定的是长期不对称契约，外国的契约都是有年限的契约，但中国可能是长期的。中国的家庭就是个契约关系，但是这个契约呢是拉了一辈子的，就你小的时候，我养你，那等我老了，你养我。你看我们在讲一个小孩儿为什么要赡养父母的时候，其实我们讲的就是乌鸦反哺，反哺最坚实的理由是以前他养过你，我给你做过事儿，所以说这才是中国最大的一个···。但是如果说那个父亲早期抛弃了这个孩子，你发现中国人就容忍的多，这就是中国人的契约，他会把这个契约拉长到几十年，尽管没有写。

还有一个中国人的契约呢，可能是跨期的，也可能是跨个体的，比如说这个父亲养了孩子了，孩子可以不养我，但是你可以再养你的孩子，也是算一种回报。比如说我给你帮忙了，你去帮别的人，中国人认为也是符合中国文化的。所以说我对中国人的管理是比较认可的。包括中国的君王跟老百姓之间，实际上也是有契约的，你会发现一旦国王不好的话，百姓是会把君主给推翻的，甚至君主一旦因为恶行被推翻的时候，下场是极其残酷的，这就是说他不是一种对等的关系，比如说姓朱的全给杀的一干二净

刘书博老师：所以说我们是有点说连带的、传递的，我们就是一个共同体的

受访者：非对称契约，还是有双方的，冤有头债有主的，但是这个主主到哪儿去了也是搞不清楚。

刘书博老师：那西方呢他们就强调个人的权利啊，非常清晰的

受访者：个体对等，但是这种对等的交易成本极高。所以说我一直觉得在中国，管理叫简约化，就是不要过度的制定这种契约关系，谦虚后退一步，忍让。但国外不行，不能让一分钱，因为这是我的权利，你比如说国外觉得政府给汽油涨价了，他们就要去上街零元购，就要抢别人的。这个我就不赞同，为什么呢？我觉得我们中国人的观点，那政府做的不好，我们可以再看看，再看看，但是你做到一定程度，那你政府要知道你做要有代价的。

刘书博老师：对，我们北方尤其是这种感觉，但上海可能很西方的，我之前的房东给我算水电费都精确到几分钱，他们会觉得应该是这样，但是我会感觉你有这个必要吗？我们之间比如说，你do me a favor，那我以后还你个favor，这种所谓的仗义啊就没有

受访者：不舒服是吧，我也不喜欢。

刘书博老师：所以上海就没有这个，他们就完全西化的那种，他们会觉得我分的清，他们会很欣赏这种西方的。那像我是在北方长挺大的，像胡老师也是，我们就觉得这个很恶心。

受访者：其实我觉得东方人就应该这样（大气仗义）。

刘书博老师：尤其是像松下幸之助，他对于员工就是一种家长的那种，那西方人不理解，他说你就一纸合同，你都给他工钱了，你干嘛还要关怀呀？那松下幸之助在经济危机时期，还是坚持不裁退一个员工，他就是富有这种责任感

受访者：所以你就知道日本的企业家，包括政治家，他一出事儿他就先鞠躬，他其实有时候真不管这事儿

刘书博老师：咱们现在一出事儿就裁员

受访者：所以中国现在进入了一个混乱期。日本人因为人少，他自己就是不知不觉中搞明白了，或者是协调了。但中国太大了，所以导致了文化上的管理思想的困难。比如说如果我们按照这一套思路来讲的话，中国的很多管理背后是有深刻的道理的，比如说刚才刘老师说的这个谦虚，对吧。或者说这种非对称的自我承担责任，这个本身实际上是提高管理效率，在东方的语境下或者情景下，提高管理效率很重要的东西。其实我一直想写这方面的案例，就是我们要从这个角度来深刻的揭示。现在呢我们用的那一套体系呢，实际上用的西方的，比如说实证研究，并不是真正的臣服于他的那种思想，我们只不过把它拿来当做一个工具。实际上呢你写那些实证论文，本质上不是说那个东西多么有用或者多么符合中国的行径。搞这些东西就是门票啊，要有一个门票，这个门票就是说你要花代价进去，就跟那个包为什么要那么贵，这个包也是个门票，这个包并不是代表着它真有什么价值，而是说你能买得起这个包，就能说明问题

刘书博老师：就跟当时中国科举考试一样，四书五经到底真的能够帮助治理国家吗？可能有一定的作用，但是后来科举之后就把它变成八股文的那种，它就是一个门票。

受访者：这个里面有一个问题，西方科学是寻因的分析方式，就是研究因素之间的因果关系，这个东西是人类的一个基本的思维模式。他们把这个问题讲清楚了。但是中国呢讲不清楚，你比如说我们的八股文，我们一直说中国的科举制度是让中国社会稳定了一千多年的最重要的制度。“揽天下英才为我所用”，其实那些人根本就不算什么

刘书博老师：他揽得可能是服从性最高的那批人

受访者：一个是服从性。第二个，他揽出来了，实际上降低了交易成本。其实理论上来讲，你随便选几个长的差不多的、口齿伶俐的，照样能解决问题，但是这个交易成本就很大了，这个人凭什么做宰相呢？那底下人是要造反的，一旦你干的稍微有一点点问题，那么所有的人都要他···这个合法性就有个问题。实际上在中国，合法性既是一个很有用的一个东西，也是中国管理理论上的一个bug啊。就是合法性这一块，老是解释不通，实际上没什么问题，这个合法性其实无所谓的，但是呢没有这个东西就解决不了问题。所以大家胡编乱造一些东西，所以说我有时候也不说，我看到很多好像不（合理的事情）我也顺从，为什么呢？他就解决合法性的问题，合法性是什么呢？是所有人都认为ok的东西，那就合法了，是吧？这个不在于我们怎么想，而在于大家怎么认为的，对不对？所以说有的时候我们不能太另类了，这也是中国人的一个思路。但是外国人不是，因为他们是靠科学的，他就用寻因理论。

刘书博老师：而且像您刚才说的，他们就是不断开拓进取的、发现的，所以他们会想要突破之前的、创造新的。所以我们会发现其实实证范式一开始也不是主流，就是美国人搞出来的嘛，也是一个标准，它构建的标准。中国呢会觉得这个标准重要，构建不重要。

受访者：对对，你说的对，但是西方的那个东西呢，在理工这一块还是有合理的。因为理工就是寻因的，理工不存在合法性的问题，理工存在逻辑性的问题，所以说呢西方思想确实在理工这一块儿有他的绝对合理性，我认为是这样。但是呢你要知道，人的问题跟自然的问题不是一个问题

刘书博老师：您刚才说管理啊，它本身就是创新嘛

受访者：对，他就是不一样。

刘书博老师：那我们虽然搞管理，但是我们其实把管理学变成了一样。这就是一个很有趣个悖论，是矛盾点。

受访者：实际上呢，我认为现在的中国的商学院，是用中国人的思维去理解外国的那个···实际上国外商学院是他们那种科学思想的延伸，就是说自然而然的，实际上我认为国外的商业思想不是诞生在商学院，其实国外的真正的人文思想是来自于科学革命。其实资本主义的思想来自于工业革命带来的生产力，说白了，民主思想的基础就是工业革命，因为工业革命的条件下，每个人都是有剩余价值的。所以尊重人就有他的科学的合理性。为什么在农业社会不行呢？为什么农业社会的民主没有基础呢？因为农业社会生产力低下，基本上一个人只能生产我基本生活品的100%或者还差一点，就说我基本上我一个人辛勤劳动，我吃的勉勉强强。这个时候就有个问题了，那社会上有能力的人，怎么看待这些人？他就要打压这些人，这样就是本来你是95%的满足，我再打压你一下，有个70%就行了，我留25%。那你想，我本来就是勉勉强强，我怎么能把东西给你呢。那么这个时候威权就有他的合理性，就是我给你抢过来，所以说这个时候搞民主就不行了，我干嘛还给你呀，我自己还没吃饱呢，你最好再给我一点。你要是再往下压的狠了，社会就反抗了，就爆发了，就把你搞掉了，就是每一个个体都会权衡反抗的风险跟我饿点肚子的痛苦。但是呢在工业社会，每一个人只要给他生产资料，他就能大概生产150%到200%，所以这个时候大家想这是很合算的一个事儿，我打压你干嘛呢？打压你，你躺平了不干了，我一分钱没捞着。这个时候我给你生产资料，然后你拿120，你生活还变成中产阶级了，稍微好一点，然后你把剩余价值再给我。

但是呢实际上现在又有问题了，现在因为技术的发展又反过来了，又到了另外一个时代了，另外一个时代什么呢？实际上因为科技越来越高，越来越复杂，实际上你会发现50%的人又到了生产不了自己要的东西了。因为你要驾驭一台复杂的机器，其实你要驾驭好了，你一个人可以生产100个人的生产资料、生活资料，但问题是你就干不了，有的人一辈子你培训他，他也生产不了他自己需要的东西，因为他操作不了那个东西。所以这个时候实际上好像又回到农业时代。其实《人类简史》里面说过这个问题，他说以后1/3的人到1/2的人根本就没用，所以后来就奶头乐嘛。因为社会如果要培训你，让你能够操纵生产资料，他可能要亏1万块钱，但是呢，我要是养着你，让你处在一个最基本的生活方式，5000块钱够了。

所以这个这个就回到原来的话题，他这个其实是技术在推动人类思想的。所以这个民主思想一点都不奇怪，民主思想的基础就是工业社会，工业社会里面基本大多数人都能创造超过自己生活资料的东西。所以你要想把经济搞上去，最好的办法就是让每个人都自由，因为每个人的账很好算，他不干没得吃，有的干自己拿一部分，剩余价值给资本家给社会，就这么简单。

刘书博老师：所以就《国富论》为什么会成为经典著作，也是跟当时的社会环境有关嘛。资产阶级的形成需要有一个理论背书，然后他就写了这个

受访者： 是，所以未来的社会啊跟过去不一样了，你就发现东方管理思想就起作用了，因为原来我们在过去就要分层，当然基于的目的是不一样的。然后到了工业时代，我们不需要分层，因为每个人都是自由的，强调人权，强调平等。然后呢到了后科技时代，你发现又有这个问题。你会发现很多的时候，我觉得就比如说在管理这一块儿，我就觉得非对称契约就变得非常重要。对称契约实际上对人是不公平的，你一个三本毕业的一个学生，你跟他对称契约，他就饿死了，他或者饿不死了，他没有这么有用啊，

刘书博老师： 那这个时候除了权力伦理还有关怀伦理，我比你强，但是我有义务来关怀弱小的。

受访者： 对，这个关怀伦理在儒家里面也有一个思想，我觉得特别好。仁政实际上体现的民生思想，对人的基本的尊重，你都要有这个仁政这个东西，实际上不是基于国外的非对称契约。

所以说我觉得作为政府来讲，作为管理层来讲，他要照顾每一个人啊，给他们最基本的这个尊严，然后要给他这种机会，要给他一个hope，然后让他去能稳定的工作。这个做法就需要东方的这种管理思想，你比如说我们内部管理那种落后员工的那种KPI就是错的，你不能这么干，对吧？

刘书博老师： 嗯，KPI有点就像把他辞退了的一个合法性工具。

受访者： 对，对，但是它在微观上有它的合理的地方。但是整体来讲，如果一个民族都是这么干……所以我们现在学的那些东西啊，我认为这个商学院学的没有思想，他拿西方的这个工具来进行管理，其实这个工具都不能用，其实这工具只解决了一个问题，你们毕业以后是到国家机关还是到地方机关还是到一般的地方工作。但是这个问题解决以后，你们再拿这个工具发现没用，不好用。

刘书博老师： 我们商学院说的不好听了，我们就是个分类工具。

受访者： 对，对，我们本身就是工具。

黄秋莉： 别的学院也是在发挥这种功能吗？

受访者： 理工科是这样，但是这个就是有一些科学性的东西。比如说像哲学、文学、社会学，那这个我觉得基本上就不是这样。

刘书博老师： 社会学它本身跟管理学最大的不同，我觉得一个就是关怀弱势群体，社会学就是关注那些弱势的。

管理学院我们先天关注的是什么？上市公司老板，CEO他们的健康状况，就搞这些，我们天然不站在劳动人民那边。

受访者： 那你们要是真的能够围绕这个东方的思想建立一个自己体系就很不错。

刘书博老师： 那我觉得有些事是时代在召唤。

受访者： 对，我就觉得我们至少需要搞清楚哪些事儿是ok的，哪些事是我们反对的，我觉得至少我们从思维上来讲应该很清晰，你看现在我们搞不清楚，现在你看这个商学院，咱们动不动就投票，其实我反对这种做法。什么人投票呢？就应该像以前的长老啊，就是说你学院里面有几个……其实东方的很多问题长老就能够解决。但是西方还是有它合理的地方——不是终身制的。我们说非对称也不要那么完全回到古代，因为社会毕竟在变了，对不？所以了，我们可以在这一段时间用你，你不行了，我们再给你换了，你可以换的勤一点，但是一般的这个学院就是长老决定，你看中国过去的那个农村啊～

刘书博老师： 白鹿园里头那个族长一样。

受访者： 现在（这种思潮的）一个坑是什么呢？这个坑儿就是法家的集权思想的根，最后有些人就会批判说你看这些长老都腐化了，最后你看他们就老是维护自己，这些就不是孔子所说的那个非对称契约，非对称契约是说契约也有，就是说你搞了五年都没搞好，那你就得被换掉，那你不能老在那里干。所以这一点来讲我们就要回到这个最早的思想源头，实际上在轴心时代大家的差距没那么大，只不过是后来大家走极端了，就走成这样了。我们所说的应该是就比如说，这个长老是为了学院搞好，对吧？学院明显搞得不好了，或者不说学院了搞得不好了，就说一个公司一个组织，那个组织明显不好了，那你就该下了，但是你这个时候还说你不要管我，为什么让我下？这个就不好。

所以就是你搞不好就要下台，就是那样，这就是当时的孔子的思想，也不能说不是我的责任我就不管，那不是这样，就像老百姓服从你的时候，也不是说你说的对我就听，说的不对就不听，但是最后你把这事儿搞坏了，那你就得下，就是这么回事儿啊。

刘书博老师： 咱们现在组织就一股脑的把西方那一套标准复制过来，就完全缺乏对于人的这种考虑。不避讳地说，就学院不是要搞AACSB嘛，那他完全就是那套标准嘛。

受访者： 但是虽然表面上用的是西方的，但是我们在做事的时候又是东方的。

刘书博老师： 对，就两张皮。

受访者： 因为这个环境里有更强大的底层的力量，所以说商学院是个被撕裂的，我们这些人文学科就是个被撕裂的一个地方。所以说在这样的环境，为什么说我们骨子里要坚持，如果我真变成一个西方人，我也没有纠结的，我就按照西方的方式来生活就可以了。

刘书博老师： 就像上海人那样，水费都要精确地算到几分。

受访者： 所以上海人（内心）也没有（挣扎），因为上海人他实际上在中国就是有点像西方人，他们呢的扩张空间很大，他可以搞别人的东西，他那样做其实没毛病的。但是我们大多数人其实不是那样，我们实际上处在一个东方的情景下，所以实际上我们现在很纠结，其实我们把西方的东西拿过来以后，其实我们也没好好用，很多人就不东不西。

刘书博老师： 那有时候还被权力利用。

受访者： 对对。

刘书博老师： 这个就很糟糕，他会挑选一套对我这个权力有好处，对我有用的东西，如果对我权力有影响，（那就不用）。

受访者： 这个就是典型的东方文化嘛，对吧？但是他这里面就是被有些人利用了，就是说西方的好就拿西方的玩儿，东方的好就拿东方的玩，这个就又是个坑了，儒家不是这个意思。

刘书博老师： 就现在就中华田园女权就这个意思，人家西方女权是责任、义务于一身，咱们就是只要便宜的事儿，只想有权力，责任我都不要，最后就变成田园感了。

受访者： 所以要把这个理清楚我觉得反正有难度啊，就中国到底会怎么样？但是我有个感觉啊，我还是偏乐观的。嗯，因为我一直觉得就是环境决定这个思想。就是这个事儿，也许有人有一天把它说出来了，大家发现，哦原来是这么回事儿啊。反正现在你看商学院真的是所有的手段都是西方化，但是大家做事变成的是什么呢？就是没有人来真正承担责任。然后私下面小动作有时候还挺多，所有好的有时候就私下没小动作而已，对吧？咱们商学院还算好一些，但是有的地方那个下面的小动作就特别多，这个其实反而更不好。

刘书博老师： 这对共同体就是一种撕裂嘛。

受访者： 对，实际上整个中国的思想都处在一个撕裂当中。因为严格的讲，现在中国的生存环境变了。我看过历史，中国在几千年里其实一直是处在一种就是人多，人的生存受到资源约束的一个时代，现在颠倒过来了。在资源约束下大家一直想的问题就是解决资源分配的问题，然后怎么样生活下去的问题。

但是实际上……邓小平这个人我对他的评价是非常高的，因为他解决了一个问题，就是中国近40年来，中国从资源约束真的变了。这个改变我认为在某种程度上来讲是得益于邓小平的这个突破，因为邓小平他是走的另外一个叫实用主义，但是邓小平的实用主义他有一些非常好的支撑。

黄秋莉： 就是比如说黑猫、白猫，抓到老鼠的就是好猫。

受访者： 对，但是它有一个支撑，你们不能丢了，丢了之后它那个理论就会被人攻击。一个就是民生理论，就儒家仁政的这个理论就是民生，你看所有的社会主义都有一个目标——民生，这个一直到现在啊。我还是对中国比较乐观的，就是说只要涉及到民生的，那大家就不敢说什么了，对吧？这个很厉害。法家就是有一段时间把民生给丢了，这个民生是中国实用主义的一个非常好的一个东西，你只要是偏离民生了实用主义就有问题，这是一个。

第二个，它是一种自我改造，就是改革。其实改革就是自我改造，就是说我不固执，啥都可以改。其实很多人没意识到邓老的伟大，邓老他第一个是自己从最高层让给外姓的人啊，你看过去中国几千年都没有，他这样就是一个象征性的东西，意味着什么东西都可以改，改朝换代都可以，就是原来是邓姓的以后是什么都可以。这个一直到毛泽东时代都没有，毛泽东毕竟是终身制啊，毛泽东没孩子，所有的问题没办法验证了，但是如果毛岸英在的话也没准就传给毛岸英了，所以这个问题在毛泽东手上是没有解决的。邓小平是他自己是好好的，然后他还让位给别人了，这个就是改革，如果没有这个事件来做备注的话，那是很难有说服力的。

还有一个了就是包容开放。第一个就是说你有好东西我可以学，这个姿态就很好啊。第二个我不理解的也可以先试试。孔子是他遇到不知道的事儿就不说，我也不反对你，对吧？孔子有一个最典型的叫“近鬼神而远之”，这不是说他讨厌或者不相信鬼神，而是说我不知道，我没研究过，对吧？我也搞不懂，反正感觉就像这个不明觉厉，是吧？那我就不说你，你爱怎么搞，是你的事儿，鬼神的事儿就鬼神去解决。其实孔子不厌恶鬼神的，人家祖宗崇拜本来就有神嘛，对吧。

所以这个三个支柱就把实用主义提到一个新的层次。

刘书博老师： 那个是美国那个教育家杜威先提出来的吧。

受访者： 对，他们当时是出发点没有这个高，他把这三个支撑我觉得是秒杀。实际上他的精髓还是儒家的这个观点，是新儒家思想。

刘书博老师： 其实可以对应到西方的那个叫经验理性，不需要先有一个宏大的理论性的方向，我就先做，做的过程中不断的发现问题，再解决问题，摆正路线，就是摸着石头过河，这就是英美的经验理性。

欧陆了，像法国就很理想，他们总是搞大革命，因为他觉得我现在是跟理想很远的，所以我要颠覆，这个叫构建理性。邓小平应该是经验理性。

受访者： 所以美国人的方向会调的很快。其实实用也是一种演变的思维，你演变是没有方向的，演变是承认未来是不可知的，所以现在民主派的有点走偏了，是走到欧洲的那个。

刘书博老师： 就有一个政治正确的。

受访者： 对，有很宏大的愿景。所以美国现在是处在这个分离期，我没想到他们有那么自信，我觉得他好多这个理论现在有点懵了啊。

实际上中国人，因为他原来在这种资源约束的环境下面有一种隐忍和勤劳的生存能力，就是在这个新的儒家思想的领导下，然后再加上西方的技术扩散，中国一下就起来了。说句不好听的话，就是一个生物突然走到一个没天敌的一个地方了，哇，一下子就话了。

刘书博老师： 我们就是外来物种，就黄河大鲤鱼跑到美国的大湖里。

受访者： 所以尽管是美国、欧洲压着我们，但是中间有很大的一块是没有天敌的。现在中国了就在一个理论构建时期。

但是我觉得东方的这个新儒家的思想还是要有一种隐忍和谦虚呀，因为说句老实话，我们扩张的有点快， 我们现在其实动了别人的蛋糕了。

刘书博老师： 对对，我们其实内部也有问题，就是那种精英的傲慢非常明显。比如刚才说那些企业家，什么都要干，无所不能的样子，没有边界。

受访者： 中国实际上有一个很重要的思想状况，就是实际上在中国各种思想都有，如鱼龙混杂。因为中国权变，他要不断地去调整自己的看法，然后不断地去为自己的留后路啊了。我还有个说法，就是什么话都不说满了，什么理论其实都是存在的，只不过说主流或者是非主流，所以说中国整个的管理思想啊，其实也不是说铁板一块。

主流思想是儒家、法家，但是在这个下面每一种理论都有反其道而行之的。你比如说包括现在中国很多人就拿了一些支流的那个东西啊来反驳，比如说狭义的信用中国人也有啊，为了一个承诺，但是这个绝对不是中国的主流，但是呢这个东西一直也都有，但是你拿了一个事件来说就虽然没有说服力啊，包括中国也有不宽容的，也有讲究造反的，你比如说这个《西游记》里面，对吧？那个孙悟空啊见谁都打，所以这些东西他也也一直在存在，但是这个东西你要分清什么是主什么是次。

所以我们在研究的时候一个容易来的这个误区是大家拿一些乱七八糟的边缘的理论来侵蚀我们来对抗我们，所以中国什么理论都有。我再举一个例子，你比如说孝这个观点，其实在中国还存在着大量的实用主义的不孝的这个观点，。你比如说在中国的农村普遍有一种观点，就是人过90就该死，老人有一句老话叫老而不死为之妖为之贼啊，就是说你到了90岁了你还不死，你又不能干活了，你就要吃粮食，这个就是就是贼。这个在我们那个农村里面，你如果到了90岁，如果孩子不是特别孝顺，人家是可以原谅的，包括老人死了以后，如果说超过70岁以上，中国人叫白喜事，不消耗资源了嘛。

刘书博老师： 刚才说那个90岁就喜事儿有点像KPI考核，像35岁在互联网公司可能会被辞退。

受访者： 对，这个也符合中国人一些支流的想法，就是说你没用了，那你就得要被辞掉。

刘书博老师： 用后即弃。

受访者： 对，你在里面可能就要甄别他。

刘书博老师：  咱们现在好像对于西方的就是正确的，工具性的就没有错，就是理性的。

受访者： 还有一个现象，我看这些议论比较多，比如中国人不说真话，

不说真话实际上是个现象，因为中国人他确实不能说真话，你只有在对称契约的情况下说真话才有意义，你都不对称契约了，跟你说啥真话？比如说老子跟儿子，我刚才举的那个例子那就是不对称，如果老子过得很苦，你跟他说啥真话呢？你说了也没有用啊，你责任该承担承担，不是说我没钱了我就可以不养孩子了，你不能这样，所以说你说真话没有意义。

但是其实在这里面，我刚才说的一个例子也有一个陷阱，这个陷阱其实在中国主流思想里面中国人说假话可以，但是一定要善意的假话，不能损害他人。我可以这么说，基本上中国人确实是生活在假话之中，比如在外面我过得再差，回家去跟父母也说挺好的，对吧？但是问题是什么呢？就是说我们主流其实有一个潜在的一个认识，就是你要善意的，你说这个话的时候肯定是对对方没有伤害。但是因为我们都这么干，所以中国人就被贴了一个标签了，就是不管是怎么样都说假话，而且因为是假话，所以那大部分还是恶意的，因为你干嘛要骗人呢？其实不是，其实真话很多时候很伤人的，对吧？

刘书博老师： 对，所以我们得做好这个管理思想的梳理啊，就不能什么都放在主流里。

受访者： 要把主流的要分出来，你要梳理出来就是我认为这个中国的管理思想应该是什么？什么是正确的姿态这种。

中国这个情景大的还没有变嘛，但是也要去包容西方的理性的思维啊。然后要正确地认识中国现在的这个情景的变化。因为中国现在资源确实也在开放了，我说跟过去不一样了，现在中国人90%多的人能吃到肉。

实际上我有个观点很多人不同意，我认为咱们这个几十年是中国几千年历史上最好的时候，真的是最好的，因为中国在盛唐时候，在康乾盛世，中国人基本上一般人是吃不饱的，你要知道吃不饱肚子那是啥感觉？ 所以很多老惹招待你就是让你多吃点，这个对于他们来讲是已经到了骨髓里面了。

刘书博老师： 所以这个我觉得就很有研究或者是理论构建的意义。我们我们处在一个之前都没有经历过的历史阶段，然后我们现在的管理，对于适应这个历史阶段生产力水平发展的这个管理理论它没有出来。我们现在中国人还是在用西方的啊，要么就是传统的那些七零八碎的，没有一套这种系统性的或者是适用于当下，然后能够让外国人读得懂的那种。那现在他们有一些做传统管理研究的，一说就玄学，什么阴阳五行啊那种。

受访者： 那都是表面的关系，那都是浮在外面的。你要说到阴阳五行，实际上我也思考过，这个管理学里面中国有一个很重要的一个做法，就是进行形象化管理。中国人经常被说是没逻辑，其实是在说形式逻辑，我们叫形象逻辑。我们形象逻辑是什么呢？实际上是来自于古老的这个人类的一种认识，比如说这个我们会认为太阳每天这样升起来，所以我们对太阳就有信任感，对吧？

比如说这个水能流动，能滋养食物啊，而且它又很谦虚，他总是往下走，所以中国人就特别喜欢水，对吧？上善若水，再脏的谁，你弄一弄就干净了，就还可以用。所以说这个中国人你会发现他的所有的思维最后都变成了一个形象的符号之间的关系。

黄秋莉：嗯，其实我们的文字也都是象形文字啊。

受访者： 嗯，实际上中国所有的管理制度都是建立在形象的基础上，你比如说我们总是用家文化来比喻公司、比喻国家，为什么呢？就是家多好理解啊，我们就会完全可以根据家去对应那个系统啊。

刘书博老师： metaphor，叫隐喻。

受访者： 对，那个翻译为隐喻或者就是象征主义，是吧？你看包括我们把老板叫老大，老大就家长嘛，就是实际上这个东西它是有合理性的。我再给你举个例子，你看西方公共关系管理里面有一个品牌视觉识别，其实我们视觉识别这一块做的最发达，因为什么呢？因为在管理里面你要是管理几个人，你可以跟每个人好好的聊，但是你要管理一大堆人视觉识是最管用的。就形象的东西，一个logo，一个形象，一个仪式比什么都容易统一人心，实际上这就是高效管理的一种做法，视觉形象的传播是最容易的，所以说他是有合理性的。

所以你说的阴阳就这个东西，这里面的辩证、演变啊，这种环境太复杂了，但是你用那个鱼一表现你就能感受出来，真的，你就能感受出来那种神秘，然后它那种渐变隐变的变化，它是动态的，然后又均衡。阴阳鱼是中国最好的一个形象符号。

刘书博老师： 真是，它都成韩国国旗了。

受访者： 韩国它也是汉文化圈嘛，所以说这些东西没有问题，但是他们没有讲到这个东西的精髓的地方啊，所以说都是表面的东西啊，就有一点为了形象而形象了。其实我觉得我们这一代人应该对管理的思想（有一个总结），其中比如说有一个主题就是说形象这个元素在中国管理中的作用。

刘书博老师： 这是一个框儿，我们把这个也可以放进去。

受访者： 就中国人在管理中经常用一些什么形象，我觉得中国用这一块儿是为了节约管理成本，就是当你的人很多的时候，你要把管理成本降下来，能够达到一致的行动啊。当然这个东西有它不利的地方，不利的地方就它还是比较粗浅，因为你形象完了以后，就很多内在的要素就丢了。

刘书博老师： 会被解读，解读过程中会异化。

受访者： 对。

刘书博老师：那咱们会聊的非常好，你也会关心历史、人文啊。但是现在我们就会发现，像刚毕业的博士，90后的，甚至80后，像我这一批的，他培养出完全是不知道这一块儿，然后他们成长起来就会变成那种用完全的细化的一种工具进行管理，那面对这种情况该怎么应对？尤其是我们作为大学老师，一步步的受到这种工业化塑料感的那种管理，会对我们的身份建构或者工作意义产生一些什么样的影响呢？

受访者： 呃，我觉得也没什么，我实际上我就说演变这个概念，我个人的观点是存在的就有它的合理性。就是说他们现在这些博士生、本科生、研究生啊，他们这样做实际上是延续了过去的中国的这种生存性的，就是大家找饭吃的这种生存主义，然后找一个社会的位置，我要去挣钱，对吧？我觉得这个没毛病，因为中国现在有改善，但是没有完全解决啊，比如说房子就是一个问题啊。

但是我觉得这是两个吧，就是说一方面我承认这种合理性，但另外一方面我觉得已经对这个要求不太迫切的，以及我们对这个有意识的就要有一种责任感。你就是要唤醒一些人来去做这些事，就是我们来挖掘这个。我觉得就是要互相理解。比如说我们假设我们有一点点觉醒的意识啊，也不叫觉醒，就是有点思考的人，就是我们可以多做一点，然后我们可以把这个事儿想明白了，去多影响一些人啊，引起人的思考，然后同时我们要理解对方。

刘书博老师： 我们是很开放、包容、理解对方。但是如果对方他让这个组织变成非常的单一的标准来评价老师的价值，比如说拿AACSB举例，他们就会把老师进行分类。

受访者： 那就没办法，你看我就是一个典型的例子啊。我也有一些想法，你也发现了，哎，我有很多想法，但是为什么后来我不干了呢？就是我发现跟我的理念不一样嘛。不一样的情况下了，我就压缩我的需求啊。

我就没有那么需要那个东西，那这个时候我就不干了，你那时候还没来了，我是零几年开始在商学院做了，但是我发现理念不一样，那我就退了，但我退了我不会愤懑，我不会去……我是很乐观的，我觉得这个社会还是挺好的，我觉得这是个挺好的一个事。

刘书博老师： 但是您就会乐观，看到社会好的一面。

受访者： 对，对对。你要化解自己，就是有的人他发现这个不一样，他可能会指责别人或者干什么，我觉得没有必要，你要调整自己。但是你们要有人愿意跟我聊这些东西，我也很乐意去分享，他不跟我聊我其实也挺好。

刘书博老师： 那您会觉得他们都占据了这种学术的资源，就甚至会排挤不同的像不一样的。跟他们标准不一样的都很难有一个学术的渠道和平台去表达自己，会不会有这样的一个影响呢？

受访者：这个怎么说呢？我觉得还是分角色来考虑这个问题，就是说如果你是一个院长，将来你干到院长了，你应该搭这个平台，这是你的责任。但如果你要干到教育部长，你应该肩负起更大的则热门。

所以我对教育部的评价就不是很高，因为他在这一块儿没有主动做这些事儿。我认为一个真正的中国的那个儒家的话，他就应该去搭这个平台，让有那些想法的人有一个阵地和场合去讲这些东西。

如果说你是一个老师，那你就跟同学们聊聊，但如果这个影响了同学们考公务员，还有同学们对这个很在意，那就少聊一点。所以现在我跟同学们都是这样，你不主动找我聊我也不说。你要找我你愿意聊，我很乐意分享。

我一直认为，实际上我不是一个精英的思路，甚至我认为社会是一直在不断地演化，这个社会是适合就行，不需要进化。这个社会进化呀，实际上在这里面我有一个大的思考，实际上这个人类学家讲这个人的这个本质的东西，其实人类的本质不是在于个人，不是在于个体，甚至不在于幸福。其实你看有人就写的那个叫《自私的基因》，其实人类社会就是基因的延续。其实就是从上帝的角度来讲，人是nothing，是nobody。你是没有什么的，对吧？你其实没有意义，其实你的意义就是在于传承这个基因。但是这个说法看起来很low啊，或者说很悲观，其实也不是。我认为这个《自私的基因》他讲到的是人类生存的第一原则。

第一原则就是对于一个宇宙，对于一个人来讲就是生存就行了，你到底是老鼠还是恐龙其实无多大所谓，生物都是不断的在改变自己的这个基因组啊在谋求生存。但是人为什么成为人呢？出现了第二原则，这个第二原则就像那个我们在这个公司制度里面的代理制度。

实际上你会发现啊，比如说蜜蜂会去供养这个蜂王，它实际上没有想法的，它就是死了也好怎么着也好，反正他有得吃了，他就把那个喂养蜂王看作是一种本能啊，这个全是基因在控制。实际上这就是一种生存的方式。但是所以你会发现群体智慧嘛，每个蜜蜂都很傻，但是整个蜂群很聪明，这是一种方式，（受到第一原则的支配）。

其实人类是走的另外一种方式，它等于说是第一原则交出了一些控制权，这样代理权就出现了，实际上就是第一原则让渡了部分控制权给主人。公司代理制也是一样，所有人让出一部分权力给代理人，你就发现公司实际上它活了，它就变成法人了，这个就第二原则。

其实所有的民主啊、自由啊都建立在第二原则的基础上。对于蜜蜂来讲，自由有什么意思啊，根本就没有价值，它也感受不到，它也不会这么干。但是对于到了第二原则的时候，就出现了这个人的各种各样的想法。其实人文主义完全是诠释第二主义的。但是这里面有一个问题，第一原则跟第二原则到底有什么关系？你会发现第一原则跟第二原则在有些情景下是有冲突的，所以就涉及到另外一个东西了，《思考快与慢》里面有讲类似的这个。

人性这种，反正就是很深沉的东西是意识不到的。《思考快与慢》那本书，我建议你们看看，他就讲这两个思维系统，那个快系统实际上就是出于本能，那个慢系统更理智啊，不过实际上那个快系统你想为什么一直存在到现在？所以说这个东西是嵌入到你的里面，就是说那一套慢系统就是以前的这个第一原则埋下来的一些手脚，会控制你，但是它是一种很松散的一种控制。比如说我举个例子，你比如说对友谊的追求，你喜欢跟人在一块，其实本质上来讲，你要是从第一原则来讲的话，那对我有利我就合作，那这个确实第一原则埋下来的，所以你脱离不了这个东西。

你看那个中国其实有人意识到这个问题，你看王阳明起的那个叫致良知，你就跟着本性走，你良知就会告诉你，但是王佳明一直没解释一个问题，你良知为什么就在呢？为什么每个人的良知都一样呢？为什么这个良知就是ok的呢？实际上王阳明没解释这个问题啊。

其实对于第一原则来讲，很多事情都可有可无的。所以对于人来讲，我觉得舒适或者说你能自洽是一个很重要的一个东西。你能自己把这个想明白了就很好。但是有一点就像那个王阳明，他说这个本质的东西一定是好的东西，一定是让你感到舒服的东西，所以说你也不用纠结，这都是大自然已经安排好了，就比如说有友谊啊，比如说有人要谈恋爱，比如说母爱、父爱，对吧？比如说对父母的孝敬等等，这个都是你的本能，你不用去想他为什么有这个东西，合理不合理，你有了这个东西你就舒服。为什么这样呢？有本书就说这个都是变成了神经细胞嵌入到你的脑子中的，所以他有一个观点，他认为人的道德，也就是这个底层的东西都是来自于你的基因，来自于你的生物构造，不是你后面被塑造的，就是说还得教育。

就是说生态还是需要多样性的，你打压他就完了，最后就把这这些基因的人都给搞没了，就完了。

黄秋莉： 其实我就是从管理学来讲哈，我觉得现在这种共性会大于个性，那好像很多个性就会慢慢的被打压掉嘛，那感觉这个生态系统的多样性也快没有了。

受访者： 有还是有的。你看啊，我刚才说的那个就是第一原则在第二原则背，但是你比如说包容也是第一性的原则。因为你知道演变的一个基础，你们要看过进化论，知道就是达尔文的演变的基础就是突变，没有突变就没有演变，因为这个突变就是尝试的过程。

黄秋莉： 可是突变它有一个前提，它是有多样性的。

受访者： 所以他需要有多样性，这就是我们的宽容，对自己不认识的东西的包容和接纳，这本身就是我们第一原则给我们的一个指示。

就是实际上我很包容，就是遇到个不懂的东西马上就要打压，实际上这个是不对的。或者说第一原则有时候给你的东西它两方面都有，一方面他要让你有继承性，你可能对不认识的东西有一定的打压。但是另外一方面其实他又让你有包容，就是我们对不认识的东西宽容、对陌生的东西的友好。

为什么这个第一原则最后让渡给第二原则呢？实际上某种程度上来讲他有很多问题在，你用单一的东西解决不了，就是说你要完全靠一个原则（是不行的）。所以说对于机器人能够进化我是我认为是没有基础的，因为机器人它怎么复制，他这个包容性我就不知道怎么弄，所以说没有突变，没有开放性。没有一定的开放性，这个系统就死掉了。其实中国意志包容性是很强的，游牧民族是不断地进来。

黄秋莉： 我是觉得咱们这个管理学的标准会比较固化。

刘书博老师： 因为刚才说到就是中国人特别擅长学习标准，我们一直在复制。在中国来说我们的文化“以文化人”而不是培育（culture）。

受访者： 对，对对。

刘书博老师： 所以我们这边可能在学术这个人造生态里面会比较固化。

受访者： 这个我觉得你们就要打破，就是你想明白了，你该打破就打不破。这个事情我一直觉得，你就自己掌握分寸。比如说你要做学术，就一直会挑战这个原来的固化，一直挑战到你觉得忍受不了了，如果你能忍受你就一直挑战下去。

刘书博老师： 我觉得能暂时能忍受。

受访者： 能忍受，甚至你对这个挑战你觉得很舒服，那你就挑战，只要没有做那一种很坏的事啊，我觉得都可以。就是说我们把这个尺度要拉的大一点，就是不要觉得……我是觉得像书博，你是因为这里面还有一个表达写的需求什么的，我就不太喜欢写。你要愿意写，你就不断的拿出来跟人去交流。你就讲自己，因为我觉得只要能说得通的东西，慢慢的就会有人去认可。

刘书博老师： 星星之火，可以燎原。

受访者： 对。

刘书博老师： 是，我还是喜欢表达和写作的。

受访者：对对，你喜欢写，我是喜欢想一想就算了。

刘书博老师： 我们一块儿来表达。

受访者： 其实这个东西啊，我觉得现在的人其实生存压力没那么大，其实我们很多的时候还是会去追求生存的，我觉得很多属于一种惯性，实际上我认为你们这一代人像包括房子都不是个事儿。

刘书博老师： 你们都能活下来，即使是不同的方式。

受访者： 就是说我觉得我有想法，我自己就肯定自己了啊。但是了你有的时候你就会觉得我一定要有那个东西，那你应该去思考那个东西是不是对我也没什么意思啊。你比如说当个官，你在官场上迎来送往，想想也挺辛苦的，对吧？但是如果你实在是放不下了，你也不要勉强自己啊，真的是这样。但是应该去做到自己最大的极限，就得投身到那里面。但是如果你想明白了，我跟你说每个人的生活都挺好啊，其实我当时都想了，我退休了，那我找一个三线城市，我有吃有喝也挺好，对不对？而且人这个东西他也不可能永久存在，你过得好活100岁，你过得不好也可能也能活90岁，对吧？没有什么就是不可以的。

刘书博老师： 毛主席说的天若有情天亦老，人间正道是沧桑。

受访者：所以说这个第二原则是服从第一原则的，就像人家说树不可能长到天上，所以说人类呀，如果说你老是纠结于第二原则本身就是个悲剧。为什么呢？因为你不可能长生不老，因为你长生不老就彻底违背了第一原则，那你违背第一原则就不可能让你存在，所以说你追求的越多，痛苦就越多。

刘书博老师： 对，那黄老师说的就很通透，就有时候我们就一定要发个amg，才算能够出口气，或者是达到一个职业要求。其实不发完全可以，你发了也没人记得住你啊。

受访者： 有的人放不下这一点，我倒是觉得也别勉强，这里面就是让我们追求公平，让我们在社会上受人尊重，这本身就是一个我们骨子里的一个东西，就是我们一旦发现自己掉队了，或者是跟人不一样了，我们本身就有恐惧，这也是人性。所以你把这个想明白了，你就看看其实每个人都有点不一样，要不然这个社会怎么会这个演化嘛，就大家都铁打一片了，这样就不太行。

刘书博老师： 就蚂蚁的那种，感觉对人还是要有个性。

受访者： 你这么想，你随着它也是一种对社会的认可，但是如果你不随着他，你这种新的存在其实也是一种对人类的贡献，你把这个想明白了，也许你就释怀了。自我认可，释怀了。但是你会发现哦，真的你去吃也没得吃住也没得住，你肯定就释怀不了了，对吧？所以你这个分寸自己找，我觉得不纠结，不纠结。

刘书博老师： 我就经常那么说，这个做案例的质性的，也不是说不能生存，就是还有一大片管理学在做这个。

受访者： 当然，你们在挖掘这些事情，那诠释一些中国的成功案例。比如说柳传志那个案例，我就觉得其实……当然我不知道这样会不会刺激一些人啊，我觉得是能解释的通的，就是社会对他的这种反弹我是觉得有合理性的。他这是一个非对称性，你不能这样。中国这个问题是你有这么大的能力，你就要有这个责任，你就自己来想，不要别人来规定你的责任，对吧？

刘书博老师： 那我们现在有一些人想明白，就是总是拿西方的标准来思考那个东方的事情，他内心也很自利。

受访者： 所以我做事，我也就有跟人讲，你不要跟人对等，就咱们自己做事的不要也不要对等，就比如说别人对你差一点，你马上要反过去，不能这样。就是说你要多做一点，就是说你先做一点，我先做一点都无所谓，对吧？你就做了。那比如说他哪一天对我不恭敬了，或者刺激我了你再做多一点，对不对？如果说他一直要打压我，那我再去反击这个事儿，对不对？但是不要因为这个一点点的不对称就想着怎么怎么样。

当然我们做不到这一点，但是我们可以多忍让几次嘛，就是我可以三次、五次、十次，对吧？

黄秋莉： 就跟那个房子一样，你占了我的地方我还往后退一点。

受访者： 对，就那个宰相故事。你说我们俩争就别争了，我往后多退一点，什么原因不说，我往后先退几尺，但最后那一家也退几尺，一下子就让出来了，就是这个道理。这是中国人的这个管理啊，因为中国现在还是一个比较内卷的一个环境，其实我觉得在生存层面啊好的多了，但是现在在社会身份了，你比如说受人尊重，每个人都想得到尊重，但这个东西总量是有限的。

刘书博老师： 人总是要追求稀缺的。

受访者： 对，你得到了尊重了，别人就少了关注了，所以这个确实也是稀缺的，但是怎么想呢？这个事情自己要开导开导自己，但是总的来讲，我觉得比过去好多了。

刘书博老师： 我觉得就是稀缺导致的那个人的差别，就像小孩儿补习比之前都更严重。

受访者： 对。

刘书博老师： 就是和《精英的傲慢》那书说的其实是一个道理，一个原因就是我们好像就觉得自己努力就能拿到成功，成功之后就好像有了道德上的优势。现在我们就是这种，他就会让人们忘却成功者背后的运气呀，别人的帮助关怀呀，所以咱们现在就有时候就变得精致利己。

受访者： 对啊，所以这个事情啊他可能就是下围棋上来的，比如说啊，这个不一定有合理性，但是你不要想这个事儿，就是有的时候就自己释怀一下。

黄秋莉： 我觉着可能有些知识分子会想着要去对社会施加一些影响，不仅仅是要把自己开解就好。

刘书博老师： 可能胡老师说的，不是说就不关注社会，他的意思就不要比拼那些主流标准，我们做非主流的也可以贡献于社会。

受访者： 就是你尽量做，其实我是相信王阳明的那个致良知啊，原来王阳明那个东西我看了很久，我也一直有点困惑，这个困惑什么呢？就是说有的人说我求诸于内心啊，但有的人就走偏了，他就走不出去，所以当时王阳明那个就自己在家里想，你能想明白吗？对吧？有可能会，就是说我们内心深处的东西，如果说你真的不去干扰他，你就能想到，确实是这样。比如说跟朋友在一起聊天，或者跟家人在一块儿，那种幸福感就是油然而生的，就是发自内心，不用去想。就是说你不要去想为什么我跟那个人在一块儿就开心了？不要想这些问题，因为这是第一原则，他已经变成了基因了。所以说你体验这些，你会发现啊，你感觉舒服的都是好的东西，所以说你找找这些东西，像这个这些方面我们得到的满足是越来越多，包括甚至最简单的，你喝一口好茶，吃一个好的食物，其实这个带来的愉悦都是发自人的内心的。但是后面的就是很多的这个外在的东西让我们变得感受不到的，实际上这个社会已经给我们提供了很多的东西，对吧？

刘书博老师：没错，那谢谢胡老师今天的介绍。

# 受访者16

刘书博老师：我们就希望能够了解真实的一些情况，像我是社会学背景、人类学背景，就想研究熟悉的现象、身边的事情。因为好像现在有一些管理研究，像战略就有点太远了，像面板数据那种通过上市公司的年报进行数据挖掘，就在办公室里。我这个范式有点像在田野里面，跟真实的从业者对话，然后看一看大家是怎么样进行利益构建。

受访者：很厉害啊，这个。原来我记得北大之前搞了一个讲座，你还记得不？就台湾那个老师叶启政老师过来讲，我是全程听下来的。那是2015、2016年左右在北大社会学系。

刘书博老师：那个老先生叶启政，还有黄光国也是台湾的，他们台湾那边学者会有一些回溯反思式的，他们说美式的这套东西好像缺乏一种特殊的文化的考虑。

受访者：对。当时我听完之后启发挺大的，因为他从事科研的时间比较早，大概可能是七几年就开始做研究了

刘书博老师：对，他的背景是学统计的

受访者：我记得好像是学统计的，后来又转专业又怎么着的。

刘书博老师：嗯，叶启政写了《实证的迷思》这本书，他是看为什么现在美国的那个范式成为一个很标准性的，它是怎么发展，这个标准怎么来的

受访者：当时我记得印象特别深的一个，就是第一次听说中层理论，因为我学管理的嘛，那个东西就没听过，然后我在想这是什么东西啊？他就讲这个中层理论怎么来的，然后讲这个前因后果，我才知道，原来有很多范式。管理学有些范式就是从社会学借鉴过来的，包括一些方法啊都是。当时就觉得有些东西就是追根溯源的话，他就追了很多根、溯了很多源。他说，最早的时候，其实美国的社会学领域也是有一个发展过程，比较好的是德国

刘书博老师：对对，法国，德国，欧陆那边，美国后来去拜师学艺。但是他回去之后呢，他就开始搞自己的方法，他就做大做强了。是这样一个发展。

受访者：对对，你看我这么多年过去了，我还印象很深，当时就觉得讲了很多东西我没听过，就社会学的觉得很新奇。然后呢，那老先生讲课吧，他特点就是跟聊天儿一样，他就聊天儿，然后给你讲这些故事，所以印象特别深。还挺厉害的，我记得他应该那时候应该有70岁了。

刘书博老师：对，是的，

受访者：然后连着讲了三天还是四天。你想70岁的这样一个老学者站着讲了三四天，好家伙，我自己想想我都没劲儿

刘书博老师：这个是精力值很高的。

嗯，行。那我们就开始言归正传吧。我们这个也是想首先了解一下陈老师。

受访者：我一开始是在西安邮电大学读大学，我那时候学的专业叫理财学，理财学现在很多年轻的人可能不一定知道，就后来改名为财务管理。现在财务管理这个专业还有。最早那时候我就想为什么叫理财学，我就研究这个原因是什么。因为感兴趣嘛，我学这个的嘛。我们那专业前无古人后无来者，就开了一年。为什么就开一年呢？就是培养方案是97年做出来的，我98年考到那儿去的，然后98年那个教育部就出了新目录，就整个儿又规范了一下子，然后那个专业就没了，就合并了，所以一共就上了一届，我们就两个班，后来我就想为什么叫“理财学”，后来发现它就是财务管理或者说是会计吧，差不太多，我们那时候是在经济管理系，那时候没有学院，小学校，一共就四个系。

刘书博老师：哦，那后来怎么样选择开始读到学术这个方向。

受访者：其实当年我读大学的时候对这个读研读博没有认识，是我家里边有亲戚。上大学那会儿有时候想吃点儿好的就去人家里蹭饭。这个亲戚家里就读书的人比较多，老是跟我讲说，你要读研，你要继续念，别着急工作，不断地给我灌输灌输后来我也思考，确实当年因为考大学的时候呢，从一本调到二本，西安邮电是二本，其实心里面总觉得有一些失落。

然后我们亲戚就聊嘛，说你能考这学校来还可以，既然你有这个基础，你还是继续念。后来就老给我洗脑，做思想工作是吧哈哈

然后我考的硕士，我是考到西安交通大学，博士是中国人民大学。

刘书博老师：那博士学的专业换了吗？

受访者：呃，现在来说的话，其实不叫换专业，但是专业划分就会把它划成换专业，我之前是理财学，它是工商管理专业。后来我的硕士是管理科学与工程，就跨了一级学科了，他们就叫做换专业。然后我读博士又回到了工商管理，我又跨专业，跨到了企业管理，研究方向是物流供应链。

刘书博老师：ok，那博士毕业呢

受访者：毕业就来了这里呀，到咱们学校了

刘书博老师：就没有中间到行业？

受访者：行业有，我是硕士毕业去行业干了两年多，我其实那时候并不是非常想念博士。

刘书博老师：联通还是移动

受访者：中国电信

刘书博老师：就是没猜到电信哈哈

受访者：移动也猜对了，我本科毕业的时候找了中国移动，后来因为考上了研就没去，其实我已经拿到移动的录用函了，相当于去报道就行了。

刘书博老师：所以研究生毕业就去到电信了，电信在哪儿？

受访者：在广东那边，就中国电信有三个研究院，其实广东有一个我们叫广州院，然后上海院、北京院三个，我是在广州院。我做市场研究，就是咱们现在不是有大数据营销嘛，现在这专业还挺火的。其实我那时候做的就是这个，我很早就做这个，我们那时候叫精准营销，就是用数据库的数据去做分析，结合着调研的数据。那时候很多人问我是在干嘛的，是不是搞技术的呀，反正是要跟计算机、跟数据库打交道，确实是，包括提数据什么的，要用数据库，也会跟着去筹建数据库，但最后分析它是得有场景吧，就会面向各种场景了，市场气氛呐，套餐设计呀

刘书博老师：那为什么后来做了两年工作又还是继续读博了？

受访者：离家太远。我两年两个春节都没回家，回不来。

第二个就是经济上不行（工资太少）。

第三个就时间上不行，那时候放假是初一才放假，其实我们想回去过的是除夕，就是（那时候再）回去就没啥意思了——折腾几天再回来上班，就觉得算了吧，就在那儿过年吧。但是实际上心里边儿还是很凄凉的，因为一个人在那儿过年，所以这也是一个比较重大的原因

还有一个更重要的是过年的时候。广东的习俗跟北方差别非常大，他们不太看春晚，虽然说春晚也不一定非常好看，但就说我们会坐那儿包个饺子。他们就是会去逛花市，然后去外面溜达。我觉得就没意思，他们觉得很好，我记得我那个领导当时请我去他家过年嘛，他说你就来我家过年得了，他为了让我过的比较爽一点，专程包饺子，他就没包过饺子，我在那儿包了一次饺子。

后来想想离家也是很远，父母年龄大了，将来也得考虑往回走嘛。

刘书博老师：那也没想着在公司之间换，而是就直接换到学术的道路上？

受访者：我那工作用现在的眼光看是挺好的。我当时跳槽的时候，很多人觉得我胆子挺大的，就是不计后果，有点儿这个意思。

当时换的话，就是要找到一个同样的单位其实非常难，所以通过上学的方式比较方便，因为我工作那时候在国企嘛，就接触了一大堆东西，包括什么户口啊，什么很多东西。现在我们可能不太讲这个了，那个年代它会限制很多。我记得我办离职的时候走那个转单，我走了好多天才转完，就很多东西。然后那时候我在国企干，我就知道这个，所以当时想单位调动的话就非常难。所以说真的，你要想做出改变，上学是最便利的一个途径。

刘书博老师：所以当时就想通过读博士做出改变，有没有很清晰地说去学校做学术

受访者：没有。我就是想做出改变，然后就上了学，当时想的就是哪个学校的博士相对学制短，学校也还不错（就去哪里）。我斟酌来斟酌去，最后觉得中国人民大学是最合适的。

刘书博老师：那时候还是要考试吧？

受访者：要考试，我当时考试也是复习了好长时间。因为我当时是跨专业，别人考三门，我考五门，我要多考两门，但虽然那个不算成绩，但是你不能不及格。

刘书博老师：去了人大之后学了多少年？

受访者：三年，就三年制嘛

刘书博老师：三年就很快嘛。

受访者：那我因为我工作过之后，到了读博士的时候就很努力了，因为走了一圈儿年龄就大嘛，所以就很努力，使劲儿学，那时候投入比较大

刘书博老师：那时候博士毕业要求论文发表吗

受访者：嗯有发表，我们可能比现在的中财也不低应该。

黄秋莉：那你觉得难吗？

受访者：呃，还是有压力的，压力肯定有啊。反正导师带着，使劲儿使劲儿科研呗。就是你得投入，不投入肯定是不可以的，这个做研究嘛，不管你以后是不是走学术道路，至少你这段经历里头你做了这件事情就得投入嘛。

刘书博老师：对，那感觉怎么样？这事儿有意思吗？就是相比于工作

受访者：我后来其实我考上博的时候呢，我发现我这人有一特点，有批判思维，就是看问题往往会想的深一点，就会比一般人多想一层，就是具有批判思维是吧（用咱们学术的话来说）。这样的话，工作就会心情不太好，因为我会多想一层后面的。这种其实是适合科研的，具有批判思维的人。后来我发现我还是做学术比较合适

刘书博老师：我记得当时你跟我说，你当时的工作就是算各种短信的模型，然后让公司挣更多钱。

受访者：对，就那种套餐

刘书博老师：就是这工作似乎只是想挣钱，就缺乏一种···

受访者：对。我当时不工作了主要原因是因为离家远哈。此外还有一个原因就是说，工作当时觉得不是很有成就感。就是给他设计一个套餐，我把你这个用户弄多一点，然后多挣点儿钱工资，为公司多赢点儿利润，多卖点儿。

我就会思考这个东西有多大意义。别人不会想的——我把这个客户拉下来了，我奖金上去了就完了呀，我很高兴了呀。但我就会想这是有多大意义，这是批判思维嘛，就是它会为社会创造什么价值，我就会有这个想法。后来我发现有这种想法的人就可能做研究比较合适。

刘书博老师：做那种流程性的或者是公司里头事务性、标准化的那种工作，就感觉缺乏意义感哈

受访者：是这样的，这可能也是我后来走学术当老师的一个重要的方面。

刘书博老师：跟我那时候也挺像，我那时候也工作过一段时间，我就觉得太令人窒息了，天天按那种标准流程去做事情，让我个体完全消失，然后就完全没有自我发挥的余地，所以后来我就觉得还是读书会更自由一些。

受访者：对，其实对自己的认识是有一个过程的嘛，以前我也认识不到这个，就是后来干了之后，你发现自己会觉得不是那么的喜欢。然后做了学术之后呢还觉得还可以。

刘书博老师：那您到中财就属于是学术界的第一个工作了？

受访者：对，那是2010年。

刘书博老师：哇哦，十几年了。

受访者：其实我要是一口气念完的话，我应该2007年就可以博士毕业了。当时有这个条件，就不想念，因为也没想清楚嘛，觉得那时候年轻嘛，觉得世界很美好，我要出去看看。尝试之后才知道自己到底适合什么

刘书博老师：那刚来中财那个时候感觉怎么样？那个时候跟现在应该有不一样的地方

受访者：那肯定啊，现在中财越变越好啊，这个是很明显的。我最开始来的时候大概可能就这么一间房子，然后像这么大的柜子，没有这么多，小柜子比这还小，一半儿大的柜子，一年一个老师有一个柜子，然后一个学院就这么一个办公室。然后因为行政的老师有一些资料存放，他们也会坐班嘛，他们几个人很拥挤地挤在一个办公室里面。这是咱们学校的当年的办公条件，因为学校小嘛。那时候沙河校区刚启动，楼还没盖几栋呢，图书馆什么都没有呢，以前那边儿就启用了一个主教，西区还没启动，图书馆最早是在大学生活动中心。就是最开始的时候我们办公条件其实是比较受限的，没有面积嘛，盖不了房子。

现在你看看我们这多好啊，沙河校区那图书馆多棒啊，是不是

刘书博老师：对。那管理方面有变化吗？那个时候有像现在这样在教学科研方面有这么详细的管理方法制度吗

受访者：各种制度也都是有的，现在就是更加精细化了。以前学院人少，学校的人数相对来说可能也少嘛，制度都有，就是可能符合那个阶段，现在可能进入新阶段之后，各种制度更加地规范了。

刘书博老师：它有没有一些方向性的变化？在科研管理方面，就比如说，我说的可能不对啊，就比如说，可能咱们现在看的是UTD达拉斯的那种发表，那个时候呢

受访者：我来的时候是那样。并不是不看啊。就那时候大部分老师是够不着达拉斯，现在呢我们很多老师是够到了。那个时候也有期刊目录，里边也有UTD达拉斯那些期刊的list。但是不是按这个列的，因为达拉斯他也是后来才有的嘛，他也不是说早就有，咱们定list比那达拉斯那个list还早。我们说的制度嘛，对吧？那国外他也在不断的变化。

我那个时候可能发中文期刊的老师比较多，英文期刊相对来说（少），这也跟阶段有关系对吧？那个海归比较少，所以发英文的少。现在来看的话呢，基本上就发英文的比较多了。我觉得这是一个很大的变化，那现在就是海归的同事越来越多

刘书博老师：那现在是不是中央又有一些引导，说做中国问题研究啊，然后就是去“五维”啊什么的。这个事儿你怎么看？

受访者：这个政策肯定是好的，出发点都是对的呀，就是落地执行的时候可能有些困难。涉及到各种各样的实际问题，因为去“五维”了之后还得立嘛，对吧？还得立一个导向，这个就得去研究了，怎么立一个好的新导向。

然后就是去五唯并不代表不可以有五唯啊，可以有，就是说这个东西就看怎么去执行，还是挺值得思考的一个问题。你就说这个我们作为科研人员肯定是要发表的，不发表你没成果，肯定不行啊。但是就说你到底发表在什么期刊上、或者什么层次上对吧，不能说那个发表到达拉斯一定是特别好的文章，咱们也不能这么说，是吧？可能有些成果发到的期刊不一定好，但他的学术贡献很大，这个东西他就很难去甄别，学术本来就非常专业，他需要···

我记得咱们那个搞战略管理的学者，搞资源观的比较早的一个学者，那他比较有影响的作品就发表在journal of management上面。现在这期刊是挺好的了，但是在当年的话它是个新期刊，也不是特别好的一个期刊，那它就放在那儿，但是它的影响很大，对吧？按那时候，这也不是达拉斯，但学术界里边确实有各种各样的故事。

刘书博老师：嗯，有时候专业发展的过程也挺有意思，其实我就想看一下管理学在中国也是一个专业的发展，那现在我们会感觉，这个专业发展会比较有中国特色。但是可能供应链这一块儿不是很明显吧？因为我觉得像这一块儿更多是比较管理工程的，自然科学研究的，比较硬的。

受访者：供应链可能在咱们学院是一个专业或者是一个方向，其实它很大。按教职委来说的话，就本科来说的话，咱们工商管理教职委下面管很多个专业，包括市场营销啊、会计都是。咱们还有一个专门的教职委叫物流管理工程教职委，它和工程管理教职委是并列的。他下面管的四个专业，然后供应链管理是其中一个，还有物流管理。他这个领域里边的人就特别多，而且这四个专业涉及到的院系也很多，就是有在商学院的，有在机械学院的，有在工学院的，有在管理学院的，有在管理工程学院的；然后拿的学位有工学的学位，还有管理学学位。所以这么说来的话，供应链的学科呢其实是比较交叉的。另外如果说做研究的话，它背后的研究范式也很多。

其实现在我们会发现咱们学校从事供应链管理研究的一些老师，用的研究范式也都不完全一样，它本来就是一个交叉性很强的一个领域。

刘书博老师：对，你们有很多建模是吗？

受访者：也有建模的方法，就是最早从事运筹学的一些学者，就会进到这个地方，然后有这样的范式；然后也有做实证研究的；当然也可以用你的这个方法——质化研究。因为供应链研究相当于是交叉嘛，它会借鉴很多其他学科的方法。

刘书博老师：那我们中国的这个学科会有一个学习对象吗？比如说美国他们这边发展的很早，或者说欧洲一些国家，还是说我们就非常中国主导的这个学科

受访者：这个学科跟其他的管理学学科（比如战略管理）还不太一样，战略管理可能就是以美国为主导。这个供应链比较交叉，就做实证范式的有一批人，这批人美国有一大批做的挺好的、会有一些很好的成果。然后呢在欧洲这一边，他会用质化的方法或者案例的方法，他们做的也很好。而且美国对于学术的考核制度和欧洲也不太一样。比如说list吧，欧洲会认ABS，美国会认UTD。然后呢导致欧洲他们可能不一定发了UTD的文章，但是他有很多成果其实是在一般的期刊上，但他的成果影响很大。

所以你要说谁引领吧。反正现在咱们国内就是都在学，也学欧洲、也学美国。然后再一个就是，用量化方法的学者，这个方面中国人还挺厉害的，或者是说华人挺厉害的。就是在美国的一些好大学里面，有很多华人学者做的很出名、做的很好。这个就是咱们中国人嘛，从小爱学习，不怕数学。其实他们很多外国人、美国人很怕数学，或者就是不感兴趣，当然有几个很好的、特别强的，但绝大多数都不是很擅长

刘书博老师：其实中国人擅长数学的也是少部分。但是我们这个方面教育力度会比较大

受访者：可能有关系，量化这块儿我们很厉害的。

刘书博老师：而且我觉得像中国人咱们能够出去做到大学老师的，肯定是学习习惯特别好，很自律。另外一方面就是我们的英语比不上他们，所以做质性研究就吃亏嘛

受访者：做定量化可能也可以适当的弥补一下语言上的不足。

刘书博老师：对，是这样。

受访者：现在应该好多了，现在因为有这个polish，以前都没有这个东西，以前就靠自己写，然后写完之后你如果英语好，那就不好。现在可能这方面不是那么重要了。

刘书博老师：还是不行，因为polish不是那种专业的学术语言，他在表达上语法上没问题，但是那个感觉人家一看就看出来，他们还是对于native speaker有一种先天的认知偏爱，所以其实做质性研究还是挺不沾光。

受访者：我也有同样的感觉，就是看论文的时候，比如说看到是中国人写的论文，这论文就很好懂；美国人写的论文也还好，因为我们学英语；英国也还好；就是特别是其他国家的那些学者也是非native，我这种国家写的英语就很难懂，但是也能整明白，就是说费劲，可能跟他的文化表达习惯有关系。

刘书博老师：ok，所以现在我们中国学者在世界上来说也是可以发声的嘛，就比如说做量化这个方法的，在管理学领域这一块儿也是可以表达自己的研究的

受访者：那是肯定的。现在咱们华人在世界管理学界的影响越来越大了。我就举个例子啊，前年开始，张燕老师当时竞选战略管理协会的president。这个事件在咱们管理学圈儿内来看呢，我觉得是很重要的一件事情。因为之前战略管理这个领域里面应该是没有过华人，相当于以前都是西方的主导，但他是一个世界性的组织，现在就是有华人在里面成为一个领导，是吧？那这种影响就很大，他自然而然就会···你看后边我记得至少应该有两次了，他就会和那个管理学季刊搞一些学术交流活动。这个舞台就可以有中国人了，就是他可以召集，然后让中国人上去讲，当然也是英语，但是可以讲了，就等于有了一个发声的舞台了。像这样的事情，以前可能真的是想都不敢想。

但是实际他们那一拨人，包括比较早的像徐淑英老师，他们那一批也是华人嘛，然后再往后就是张燕啊，然后还有一大批呢，我就不举例子了。他们其实在国外做研究还是挺辛苦的，他们已经慢慢成名了嘛，成名之后，然后我们很多的华人去国外访学也好、去交流也好，其实他们都给创造了条件。

刘书博老师：ok，其实他们搭建了一个渠道，可以让更多的中国学者到美国去学习，而且可以发表更多世界性的期刊。

受访者：对，特别是他们很多人研究的就是中国问题，然后把中国的一些问题、取得的一些成绩可以展现给他们。

刘书博老师：那具体到咱们商学院，应该也是有一些中国特色，就比如说我们整个学校来说还是要一定是要讲政治嘛，那肯定是就是两条线管理嘛。这个对于供应链这一块儿的研究选题啊或者是教育啊有没有影响。

受访者：那肯定的呀。因为你看供应链这个事儿，你像我们研究供应链比较有重要影响的一个事件，就是2017年的时候，国务院发了一个文件，我们就把它叫做《84号文》。它里边就明确说要去发展这样一个供应链，不管是学科呀还是行业的全部要发展、要促进这个事儿。而且这个事件是国务院发的文，就是鼓励院校去开设供应链管理的相关课程和专业。

就我自己认知啊，当然别人也这么说。这个应该是中国有史以来第一次以国务院的高度针对一个领域或者一个专业去发文，以前从来没有过，这个比较罕见，所以说这个事儿呢对这个专业的影响就特别大。

刘书博老师：这个是为什么？为什么这么重大的一个？

受访者：那是2017年的时间，你现在回头看看供应链是不是很重大？我们现在遇到了各种各样的卡脖子的问题，其实我通过这个文件就能感觉到高层包括很多智囊早就认识到这个问题了，那文件酝酿不是一天两天的，对吧？

刘书博老师：卡脖子不是说芯片这方面吗

受访者：是芯片啊，但是实际上他是个供应链和产业链的问题，要在产业链和供应链的搭建上要有一个很好的设计，这就是比较宏观的。就是当成供应链战略嘛，其实美国他们也很早就意识到这个问题了，当时应该是2012年还是哪一年，奥巴马当总统的时候，他那时候就把供应链搞成国家战略了，美国的国家战略，其实不光是我们，他们也都很重视的。

你看导致的结果就是说2017年发了文，2018年我们国家就有供应链管理专业。你就可以看到说我们国家的战略，对这个专业发展影响非常的强

刘书博老师：对，嗯，我们完全是跟着国家的宏大战略布局在走。

受访者：这个也是世界发展的一个···也不光是我们国家，全世界都在重视。就制造业回流这件事情，其实很早就发生了，各国都很重视，制造业，你看美国啊，日本呐，英国也有，全都认识到这个问题重要了，其实制造业回流这个事儿就跟国家供应链有关系。

刘书博老师：嗯，ok，是的，那这样其实未来我们这个专业大有可为

受访者：我是特别看好这个专业

刘书博老师：那现在有什么眼前可见的一些挑战和困难吗

受访者：哎呀，现在最大的挑战就是分流的时候，学生不太了解这个专业，然后选这个专业的人很少，就比较尴尬。其实这个专业挺好的。其实国家未来的发展方向、国家的站位也好，包括这个专业所能学到的技能也好。

刘书博老师：那学生为啥不选？

受访者：有一个原因就是学生觉的这个专业难。

其实那些所谓的平台企业的背后，中枢神经就是供应链，就是说供应链是背后真正在发挥作用的中枢神经。这个东西确确实实不是很直观，就是大一的学生和大二的学生没有这个认知，他们平常见不到

刘书博老师：他们是不是误解为送快递的那些

受访者：应该也不会这么局限。现在的孩子们也挺聪明的，应该不会这么局限。他可能觉得供应链有各种研究方法，但其实这个也不是全部。

黄秋莉：还有个就业的问题，大家不是很清楚

受访者：供应链管理的就业其实还挺好，就是在我看来觉得挺好的，有很多用人单位的招聘就招供应链管理的岗位，也有很多公司专门设立了供应链管理的部门。

吕梦娜：那有可能是学生对这一套不理解吗，就是在跟您交流之前我也不了解这些东西。

受访者：对，因为这个你要是给学生很早的去讲吧，他也确实不是太能有感觉，因为之前没接触过，他不像做市场营销，你到超市就被营销过，你就知道它是什么。但供应链这个东西比较无形，不太能看得见，其实它很重要。

刘书博老师：这是一个挑战和问题，还有吗？

受访者：另外一个就是说，现在咱们学校的供应链管理是非常好的，社会的影响还是挺不错的。2021年6月份软科排名全国排第一。

吕梦娜：那这个排名主要是根据什么来排的？

受访者：呃，软科那套排名指标体系我就不是很清晰了，反正它影响力还挺大的。然后排在第二位的是山东大学。这个压力就来自这儿，这个后面还有西南财经大学，还有对外经济贸易大学，他们都有供应链管理专业。那现在要维持这样一个良好的发展的话，其实是压力也非常大。我了解到的啊，我不知道是不是准确啊，山东大学也是在大力搞专业建设，好像整个山东大学管理学院就四个专业，然后供应链管理是其中一个，我说这话什么意思呢？就是说他投入的资源，如果按照平均分的话，山东大学管理学院就会把1/4的资源投到供应链管理专业。那你想想我们这个压力有多大呀？山东大学985大学，对吧？他那个资源各方面的就不说了。跟咱们同一类的，西南财经大学、对外经济贸易大学这都很强。

黄秋莉：后有追兵

受访者：后有追兵，你说对了。然后前边儿是同学们不怎么选，后边儿有追兵，所以就我们这个建设压力挺大

刘书博老师：那这个是学科建设方面，那在不同的商学院、管理学院、经管学院，有不同的文化生态吗？这个有没有感觉。就比如说像对外经贸，比如说武汉大学，比如中山大学，他们会很快的，然后比较市场化的那种模式去发展，那中财可能会比较保守一些

受访者：咱们比较靠谱。我们这个发展应该是一步一个脚印，这是我的感觉，就这么多年过来，我们的发展包括学生就业，我们都是实打实的一步一步的走的。我们一直都是这个风格，好像我从来到这儿一直都是这种感触，不夸大，也着急，但是呢不会急功近利，是咱们的特点。

我就觉得中山大学那个不太好，原来不是招一个博士后闹得沸沸扬扬的？我们没有干这种事情

刘书博老师：对，我们也有师资博士后，但是不会像他们那样

受访者：我们很靠谱啊

刘书博老师：我们就是来了就是想让他生存下来。

受访者：对。要给成长的一个空间嘛，然后去发展嘛。

刘书博老师：所以人大应该也会靠谱。

受访者：人大我的感触也是跟咱们差不多。人大，号称是我党的第一个红色大学嘛，我们中财也是的

刘书博老师：北大清华他们好像就不是

受访者：清华北大那没在那待过啊，因为他们历史更久远，北大是军事大学堂嘛。清华是留美预备学校嘛，对吧？他们跟海外交流的比较多嘛，他本身就是那样一个学校。北大光华的国际化程度更高。

刘书博老师：光华几乎是美国的一个商学院。

受访者：这我不能这么讲。但是我觉得好像他们国际化程度很高，他们很早就发了UTD达拉斯这些，然后很快就有博士培养的项目，他们也有资源，师资也可以，很多学校也做不到，他们那个水平也高

刘书博老师：清华是留美预备学校啊

受访者：对，他历史就这么来的，

刘书博老师：光华是国际化程度很高，其实他们都国际化程度很高

受访者：清华本来就是当年有一笔庚子赔款美国返给中国，然后就办学成立了清华，他就这么来的，他一直都很有钱的。

吕梦娜：老师我看您那个简历上写，其实您本硕博都是跟管理学相关的学位，您觉得我们管理学的研究也好，或者做学术科研也好，有什么问题吗？

受访者：呃，对于研究工具的学习不太够。就比如说，你看我的简历看上去都是在学管理的，其实我花了很多时间去学计算机、学统计、学数学，就这些基础性的工具，简历里是看不出来的。但是我可能花了很多时间去学。这个就是你（现在）不学，将来也要学，因为你要写论文，你要用。

吕梦娜：嗯嗯，就论文也是这个导向，你如果不写这个数据什么的也没办法发表，是吧？

受访者：也不是没办法发，没数据也能发表，你像刘老师做质化也可以，对吧？就是说你要是按照那种定量化的范式、数据化的范式去做研究的话，那个东西你得懂啊，如果你都不会用这些工具，你怎么做研究啊？但是你学懂那些工具其实是需要投入很多时间精力去学的。

吕梦娜：对对。

受访者：现在就是，学生们又想做研究又想不费劲。就是你看学供应链吧，我觉得挺好，学生觉得难。好东西都是难的，这是我对这个的一个认识啊。

吕梦娜：您前面提到工作那两年的时间，有说到这个社会价值，就感觉考虑的还是比较深远的。那现在做了研究之后，特别是当了十多年老师之后，你觉得通过做研究能够体现出你的社会价值嘛，或者是能够实现你所谓的社会价值嘛

受访者：还是有很多时候有成就感的。特别是指导的学生毕业了，然后他走上工作岗位后干的还不错，我知道这个消息之后就是很有成就感。

吕梦娜：嗯嗯，主要是在指导学生这一块儿

受访者：因为学校嘛就培养人嘛，就这一个点来说的话，就超过很多了。

吕梦娜：嗯，嗯，了解。那做研究方面呢，就比如说跟社会关切的重大问题呀，因为现在管理学有一个被人诟病的地方，是研究跟实践有点脱节。如果是脱节的情况之下，没办法去实现你的社会价值。

受访者：这个问题是个老问题。我当年也有这样的困惑，后来我跟书博好像也聊过这个问题。马奇是说书斋式的这种研究，他也是管理学这个领域嘛，他说做研究需要有这样一批人。就比如说写的论文可能不能立刻变成生产力，但是需要有人去总结、去提炼、去结构化，有点儿基础研究的这个意思啊。这个理论呢你说有用吗？可能还真不一定有用，但是呢他会把这个理论体系搭建起来，然后慢慢的去充实这个理论体系，慢慢可能间接地就会产生一些效果，就需要有这样一批人做这件事情。

当然也需要做管理咨询的，那可能它算不上一个学术研究，但是它也是有贡献的，分不同的角色。然后你可能到了企业当中，你要是做企业的实战的话，那你可能没有用到一些方法，但是你可能做的也很成功。总是要有一些人去总结、去提炼，他会有不同的角色分工，我是这么看这个问题的。现在我也是这么理解，以前其实我对这个问题的理解，还是有一个阶段的。也曾经有过说管理学发论文好像也不能给企业带来效益，它有什么价值呢？那我现在至少反正我用这种思考理解能把我自己说服。

黄秋莉：但是老师，我们高校目前好像有一种要建成国家智库的感觉，那对咱们这种书斋式的研究不会造成一定的影响吗？

受访者：你这个问题问的特别好，这个问题我也思考过，我也有这样的疑惑。咱们国家大学和产业界之间的产学研结合，其实跟西方特别跟美国比，我们是落后的，至少我的认识是这样的。就美国这一块儿，相对会有很多人去做。有的人就是纯研究，也不产学研，就不断地发文章，然后可能他做的好，可能拿了诺奖。这样的人很多，对吧？

然后企业呢就是搞运营嘛、要生产嘛，然后就搞经营，对不对？其实不管哪个领域，从科研到生产力的转化，中间是有一大块儿工作要做的，这块儿其实咱们国家是有些薄弱的。包括你说的智库其实也类似。

黄秋莉：但是他们西方的感觉是有专门的研究院所，高校好像承担的这个职能会少一点，我觉得是这样

受访者：也不完全。我说的是一般意义上的哈，如果从管理这个角度来说的话，或者说从社会科学的角度来说的话，美国有很多的研究机构，它可能也是放在大学里头，也可能是独立的。它们就会长期的跟踪，如果说现在美国有一些报告要咨询的话，找它们就好了。因为它们就做这个方面的研究，它们就是产学研这一块儿的研究，然后它们又会有一系列的这样的机构存在，咱们国家呢就像这样的制度化的机构还没有。所以说你说构建智库呢，我猜可能高层是有这样的布局，就是说我们也要发展这样的，这个其实蛮重要的。

黄秋莉：嗯。但是我自己是这么一个感觉，我就觉得学术人员去反哺产业确实非常的重要。但是如果有这么一个导向的话，我们学术人员可能也会去跟着这个导向做，那我就觉得会对高校的纯粹性造成一定的影响，我觉得和他们之间是有一定的冲突的。

受访者：不会不会，特别是我们这个领域不会有冲突的。

刘书博老师：你看那篇公众号文章写的，是吧？那篇文章一个观点就是大学就是大学、智库不是大学，大学就是象牙塔那种理论导向的、就是很纯粹的。但是我觉得管理学这个学科就不是这样。

受访者：分领域，你知道吗？比如说我原来看电视，中央电视台的一个大科学装置，那是研究高能物理的，他的意思就是说这个高能物理，只要你来申请那个设备的使用，然后符合一定的手续，它是无国界的。这个因为它是纯理论的，那个研究出来的一些规律能不能变成生产力，谁也不知道，这就是跟学科有关系，他那个领域就可以这样做。我们管理学领域如果你去做这个，你就脱离实践了，我们就是要从实践当中来，然后提炼出来一些东西，然后或者你再经过深入思考，然后指导实践，就这么一个过程。但是呢会有一部分人就是更多的去发学术论文，然后有一部分人可能是做了产学研，然后有一部分人可能就是做实践，那有些能力强的人可能什么都干。

刘书博老师：那现在其实我们管理学这一块儿是有多元性的工作，但似乎我们会发现，那些我们认为高级的还是做纯理论的、学术性的研究，

受访者：你说的高级指的是说···

刘书博老师：就比如说咱们现在看UTD达拉斯的那些文章更多跟实践距离会远一些。

受访者：那肯定，达拉斯的大部分文章都不能管用，就做的很精美、很漂亮，但那个东西是需要的，这就是分工

刘书博老师：这个分工是有等级的嘛？那高级的就是那些纯理论的嘛

受访者：因为它比较难嘛

黄秋莉：对，我就是想说，我们确实需要跟实践结合，但是当国家有这么一个导向的时候，就比如说我们要去申课题、做基金、追热点，它是有一定的合理性，但是这种现象是否会有负面的影响？会不会侵蚀到学术本来的一些价值。

受访者：这个就是你做学术研究的一个很重要的方面。其实我现在也在思考这个问题，不是很成熟哈。就是因为这个时代的发展，我们科技领域不可能脱离时代，我们一定要跟着时代的步伐，用你的话就是“跟风”嘛，我们就要跟着时代步伐的，必须要与时俱进。

那还有一个就是说，回过头来看，我们这里面有一些东西它是不变的、规律性的，但是可能我们的规律和自然规律稍有不同、不太一样，但是这个规律性东西它很重要。你比如说马奇那个组织理论，他构建了一套东西，当然再往前还有西蒙的管理行为，再往前还有巴纳德的《经理人员的职能》。

其实现在我们很多管理学的理论，追根溯源还是实践。也就是说我们在跟着时代嘛，但是有一些东西是不变的，他是有根的，就那个东西是长期沉淀下来的，但是有些东西可能随着时代慢慢就被遗忘。但经典要反复读，反复看，然后你会发现里面其实有很多东西很重要。这就是我跟你说的，跟肯定要跟的，因为会有新东西进来，但是有些东西它会长期留下来，这是我的认识哈

吕梦娜：当前这种比较有思想性或者说比较扎实的理论研究好像不是很多了哈

受访者：这是一个很奇特的现象，我没想明白。我们现在的理论都是70年代的理论，我也很奇怪，就是为什么会有这样一个现象。而且其实不光是管理学领域，好像社会学领域也是这样，好像大部分是七八十年代那时候出现的，就百家争鸣，然后再后来就是修修补补，就是这种开创性的研究好像就少了，背后的原因我也没太搞明白。

吕梦娜：所以可能也是基于这样的一个原因，我就会觉得我们现在为了发表而发表的论文好像还有点儿称不上“基础性的研究”。

刘书博老师：像这种中观一些、宏观一些的理论比较费时，我们现在做的都是那种很细小的嘛，它会发表的比较快。那因为我们的考核制度在那儿，像你花了好几年就搞一个大一点宏观理论，但是这样就不利于生存，是不是？

受访者： 可能全世界都有这个问题。这肯定是个影响因素，全世界都有这个问题。美国的也是，现在都是，比如说做管理学研究的都是做实证研究，实证研究的特点就是不能原创，实际上本来就是这样子的，它就是要修修补补、验证一些理论。但是它做的可以很精美，然后让审稿人挑不出毛病来就能发表了。因为所有人都要吃饭，是吧？你这个不发表就吃不了饭了，所以这确实是个问题，我觉得至少是一个很重要的影响因素。

刘书博老师： 指标化嘛，可能传统一些的也不是看这个KPI，但现在我们都是KPI管理。

受访者： 对，你看七八十年代，在学校里可能比较松散的管理，就是我有兴趣了就做，没兴趣也不要求，可能就讲讲课。

刘书博老师： 所以他们就不缺吃穿。

受访者： 现在就不行了，现在生存压力比较大。特别是美国的制度，tennure-track这个制度，那年富力强的六七年就完全拼命的去发论文，做那些精细化的、很精美的研究。然后其实你创造力就那些年，就是40岁左右之前，你过了之后就很难搞。

刘书博老师： 对，而且它有一个副作用，就是大家都会锁死到那条线上，就路径依赖嘛，他不会想要说，我再考虑探究一些新的突破性的东西。

受访者： 是这样的，其实我一直在说，我还挺佩服你的，你敢于做现在这种方法。

刘书博老师： 因为在英国他们不是这样，他们管理学就跟咱们那个标准是颠倒过来，他们管理学大概70%是质性研究，是不是很奇怪？

他们质性研究也能发，也不少，像我导师一年也一篇JBE，只不过咱们可能觉得那个慢，但他们其实也很快，只不过他们做的很琐碎，也是那么一点。

 就比如最近我读了一篇，就讲这个高校里面对于年轻学者的霸凌啊，就欺负年轻人，然后他还是霸凌里面一个旁观者的视角，就这一点描述的比较细的，但是它可以把它深入下去。所以也不是说他就很整体的在写，可以切割出来是这样，只不过范式不一样。

受访者： 对，在我认知里面，我也不太懂你这个范示，我就觉得像你这样的方式写文章可难写了。

黄秋莉： 为什么会有这种感觉？像蓓蓓学姐她就说，其实她也觉得不是很慢呀，她也能出成果，但是很多老师会有同样的困惑，就说，哎呀，你这个是个难题，需要花很大的功夫，好像很多人都会有这种看法？

刘书博老师： 还是就是质性研究，我们可以探索探索，这是一片蓝海。

受访者： 我就是没学过，我说的也比较直白，就是没学过。当年我学实证相关工具的时候周围也没有人会，不会就觉得好难，自己看也没人带嘛。

刘书博老师： 但是像人大他们有一些岁数大点的也做案例的

受访者： 案例我可以。那案例还分两大派呢，你是建构主义案例，还有这种实证主义。

刘书博老师： 艾森哈特的。

受访者： 对呀，艾森哈特那套东西我可以，但你这套我知道你存在，但是我不知道你是什么样的一个神存在，你知道吗？是那种感觉，你很神秘。方法不会，不知道怎么去操作。然后它是怎么样一个规范？我们也没弄过。

刘书博老师： 我们不是很规范。就有点主观诠释。马克思那一套阶级剥削也是他自个儿构建出来的，也是基于现象提出那个词，然后解释一下，其实是一样。

受访者： 就是有老师带着做的研究，你就知道他怎么做了，没人带的话就不会。

刘书博老师： 徐淑英他们就带着做，好像就少一个质性这方面的大佬。叶启政是社会学的，但是管理学似乎没有。

黄秋莉： 毛基业老师他现在不是？

刘书博老师： 他是加拿大那边就是哈特那一套的，现在可能学术界做的都是艾森哈特的徒子徒孙。然后像欧式的，我还没有见过一个大牛过来带。

受访者： 从学术界来讲，你可能真的是有大机会。

刘书博老师： 我就奇怪，就是你看像实证的有徐淑英、张燕呀，然后还有艾森哈特那一套下来的，但是欧式的好像对于中国也不感兴趣，也不想来啊。

受访者： 反正我周围认识的人用你这种方法做研究的可能真的是不会。因为没接触过嘛，就是没用过。然后你说投入那么多时间去又发表不出来，或者写完之后被批的一塌糊涂，然后批完之后怎么改也不知道，就心里边儿对这个范式的把握就没有过。

我还请教过是刘老师，我说那个sense-making是什么意思呀？我是花了很长时间去理解这个概念。你给我讲过，讲完之后我又看还是不明白。

黄秋莉： 为什么咱们对这些不太理解呢？老师您先前说咱们管理学应该是一个人文社会科学，我就觉得现在太硬了。

受访者： 对呀，就是有艺术性、有科学性吗？我们比较偏科学性。

黄秋莉： 就感觉他不像是个人文社会科学。

吕梦娜： 那刘老师的偏人文。

刘书博老师： 好像就是一个光谱，最硬的就是自然科学的那个像物理啊、数学啊，最软的就是文学、艺术、宗教，可能我会偏这个。

受访者： 哎，像很多现在实证的方法都偏自然之类的。

吕梦娜： 为什么要偏量化？是因为考虑到专业性的问题嘛？就是之前比如说有人会说管理学就跟故事会似的，您怎么看这件事情？

刘书博老师： 你们就看《实证的迷思》就行了。

受访者： 有一个发展过程，就像我上学到现在为止，包括自己做研究，就周围基本上都是这样的人。就是我遇到一个与众不同的就是刘老师。这是我遇到的，就是他的方法我根本不懂。

刘书博老师： 所以我刚回国找工作，我就遇到了非常大的不理解。他们会问你论文怎么没有自变量、因变量，我也不知道怎么回答，就是说你的信度、效度怎么呈现，但我们不是那个语言体系，我是做话语分析嘛，就根本就没有这些，就不是一个话语体系。

黄秋莉： 像我们现在做的东西，别的同学就会说那你以后找个量表测一下其中的哪个变量，然后再继续做你的实证部分吧，就是大家都必须得有实证的东西才行。

刘书博老师： 包括应该有很多导师也会说，那你做博士论文必须得做实证。

吕梦娜： 对，我老师之前给一个学姐点评博士论文，学姐做了三个研究，第一个就是质性研究，后面两个是实证，在他印象里，他觉得学姐只做了两个研究，就是后面两个是，第一个完全不算是研究。

刘书博老师： 这就是科学主义的一种表现。

受访者： 这可能就跟你说的那个《实证的迷思》那个有关系，那个发展阶段可能是阶段性的。

刘书博老师： 其实管理学思想发展也是这样。在那个二战之后，统计学思想就进入到了企业管理嘛。那个有一个《蓝血十杰》，就是讲哪个学校统计学的几个毕业生，研究生毕业之后先在美国军队担任这个总指挥的那种辅助的参谋。他们通过统计的运筹的方法让美军的管理效率大幅提升，后来他们就帮助美军赢得了二战。后来这批年轻人在和平时期又去到了这个IBM呀，福特呀，通用汽车呀，就把那一套同样的管理模式，就是用统计进行管理的方式带到了商业世界，进而就进入到了研究领域。现在我们说的量化的管理、管理科学都是那个时候开始的。

包括那个时候美国的这一套是通过麦肯锡这样的咨询公司给普及到欧洲。所以它有点像传教士的那种把这个福音传到全球。那咱们中国现在《新闻联播》的那种上层话语也会强调科学管理，讲要科学，但似乎就没有说要关注人本，关注艺术，关注手艺。

受访者：对，发展中国家嘛，首先得经济建设嘛，这个东西太重要了，经济建设为了先吃饱饭。我是这么看这个问题。

刘书博老师： 所以就是效率主义。

受访者： 但现在已经开始慢慢的在讲追求质量了，我们原来是追求数量。

刘书博老师： 现在要质量了。所以那个时候还有四个现代化，现在是不是也还提这个了？

受访者： 现在就少了。

刘书博老师： 但是现代化，其实西方他们从韦伯那个时候开始就对现代化进行系统的反思，就比如说对于人的主体的压迫呀，或者工作意义方面的影响，刘擎那本书就是讲的这个。那我们对于管理学现代化的副作用还没有太多的讨论。

受访者： 至少从我的成长经历来看的话，中国以前是真穷啊，那时候真的是吃不好、吃不饱，我是没挨过饿，但是比我大一点的是挨过饿的、吃不饱饭，那真穷。现在这个社会主义新农村，你去看看，反正我回老家的时候，不能说家家有车，反正已经堵车了，因为现在很多人都在城里上班、回家住嘛，就是他回来的时候都要经过那条马路，就晚上下班那个时候就会堵车。

这个发展肯定会带来一些负面的，但是你要说你是愿意下雨的时候脚踩泥巴，然后你去医院看病，没有车，你要走路去，还是现在忍受堵车，然后十几分钟能到，你看哪个好，肯定是现在好啊。就是发展肯定是有负面影响，比如污染这些肯定也都有，对吧？

黄秋莉： 我们是想要有钱，又想要有蓝天。

受访者： 所有人都是这样的，西方也是一样的。

当年是真的是穷，现在而且以前那个农村这个人的寿命普遍是比较短的，就是那个岁数很长的人不太多。但现在为啥生病的多了呀，就因为生命变长了。这就跟生活质量改观有很大的关系。

刘书博老师： 而且心脑血管疾病发病率特别高，就是因为吃的太好了。

受访者： 富贵病嘛，肥胖症之类的。

黄秋莉： 然后我想问问老师，就是因为老师在这儿工作12年了嘛，然后就想说以前是在这么一个小的办公室里面大家一起工作，那除了这种工作环境的变化，你会不会有更多别的感觉？

受访者： 科研上的压力就会越来越大啊。以前我刚来的时候，有很多新方法、新工具，然后觉得自己还可以写很多好的文章啊，现在发现我已经是被狠狠拍在沙滩上了。因为后边儿的青年才俊太厉害了，这个很明显。现在年轻人都是学了新方法、新工具，而且发的文章的档次越来越高，我们以前哪有什么UTD、FT？没有，这些说法都是后来有的。

黄秋莉： 嗯，会对你造成压力吗？

受访者： 当然会啦，那同事人家发那么多论文，如果我这一个都发不出来，你觉得好意思吗？

吕梦娜： 主要是自己的一种心理上的上进嘛，不是说外面给你制度上的压力吧?

受访者： 职业压力嘛，因为你会有一种害怕被淘汰的一种意识。

刘书博老师： 对，我们从事这个职业总是希望能够处在职业的发展前沿嘛，是有这种紧迫感的。

黄秋莉： 那老师的这种压力就是看着人家发的比较多，那自己也会想要发更多的文章吗？

受访者：不光是这个，有些老师会说各种个人原因，比如我现在发不了我发个一般的，能完成工作也行。你还要带学生，学生他要毕业，然后现在学生水平很高，然后要求学生毕业也要达到很高的水平。这个时候如果老师有一桶水，才能舀出来一碗水，如果你觉得你那一桶水见底儿了，舀不出来的时候这就很可怕，因为带学生你不能误人子弟啊，对吧？这是最基本的。所以你还是得不断的充电、不断的充电，这时候就发现有压力了。

刘书博老师： 陈老师是有责任心的，有些老师可能就是“我就没责任”。

黄秋莉：还有呢？

受访者： 主要这两个方面吧，其实我们学校那个考核的压力还可以，我们学校比较人性化。

刘书博老师： 像北大清华他们就是老人也新办法。

受访者： 那压力太大。

刘书博老师： 不行就走了，那就是生存压力，所以我觉得他们特别的残忍。

受访者： 咱们学校这方面目前来说还是可以的，咱以后不知道会不会压力变大。

黄秋莉： 什么样的情况下会有恶化的可能啊？

受访者： 因为有些学校已经变了，北大清华那他会有制度的变化，等制度全变过来，那老人肯定是受不了啊，上了年纪之后没受过严谨训练，眼睛也花了，身体也不行了，熬夜也熬不动了，你让他出成果，他怎么出的来呀？他是有阶段性的贡献嘛，就是每一代人有每一代人的贡献嘛，他们之前也是做过很多贡献的。

吕梦娜： 我觉得很多老老师都非常的厉害，放到那个时代，我觉得那比我们优秀多了。

受访者： 时代在发展嘛，那你是比较好，你有历史观，你都会考虑这个，有些人是没有的。

吕梦娜： 而且我是觉得现在的年龄比较大的老师，放在现在我觉得也是很让人佩服，就是他们可能不会做实证研究，不会写那种漂亮的论文，但他们思想性我觉得还是很在线。所以说如果这样的话，还以新人的办法来要求他们，会不会有点过分了？太残忍。

刘书博老师： 北大清华就这样

受访者： 对，有些他就一刀切了呗。

刘书博老师： 很国际化的那种很激进的学校，可能就是这样。

吕梦娜： 老师，我还想问一下，就是您那篇文章也写到了，就是供应链管理专业也是对专业人才的培养嘛，就是无论学生也好还是老师，其实都属于专业人士，有这种专业的身份在的。那您觉得以供应链管理这个专业来说，他的专业性主要体现在哪些地方？或者老师学生的这种专业身份能体现在哪里呢？

受访者： 我没太理解你的问题，他本来就是专业。

吕梦娜：比如说我就撇开说专业的这个谈法，就是老师他们出来在社会上他们就是专业人士嘛，就是这种身份，那你觉得他们的这个专业性体现在哪里？

受访者：就是不可替代性吗

吕梦娜：比如您刚刚提到了很多工具是量化的这种方法，您觉得这个是能够反映专业的一个方式吗？

受访者： 不是这种工具，我给你举个例子吧，比如说在生产企业里头，生产线会安排生产，然后产品会出来，现在我们都是以流水线的形式在走的。然后这里面就会涉及到说要怎么让这个生产线比较顺畅的运行，那这里边就会有产品质量问题，有产品出现，然后有原料的供应，特别是复杂的产品，比如汽车，汽车如果你拆到一个个小的零件的话，得有一万多个零件，那最后这个一万多个零件要组合到一起，形成一个车，前面会有很多很多的供应。

那你得按照一定的节奏来，这就是个工程啊，在运营管理里面，这里面怎么定多少货？什么时间到哪儿？然后怎么组装？这里边你看上去很简单，其实你不能出任何问题，出问题的话就确定是次品啊，质量就不达标，对吧？你要把这个东西管好，当然比如说这个零件设计好之后，可能这个零件本身它是有技术的，但是你把这个零件组装起来它也是技术，这个组装就跟管理有关系。当然设备有一些原因了，但是可能管理这套系统它本身就是很难啊，他就是个工程啊。

吕梦娜： 我想到了精益生产啊。

受访者： 就是类似这样。就是不能说他是供应链吧，他至少是供应链的一个部分，以它为例子吧，你说的不可替代性啊，那你要是长时间在这个生产线上去管生产线的话，你会发现你确确实实有不可替代性，别人来根本管不了，那零件根本都不认识，认识完之后你怎么样把它协调好、沟通好、对接好，这里面还是需要积累的， 积累完之后，就是随便来一个人短期内是干不了这个工作啊， 他很专业啊。

采购里边也有类似的，就是比如说你有很多个供应商，因为采购这个东西就是供需关系嘛，这里边有些信息他是不公开的，就企业的比较机密性的，你比如说你去问你的供应商，他就不愿意跟你讲的，那你这些信息你要长期的管理，把供应商管理好，然后让他按照你的方式给你供货，这里边需要有很多的方法和办法啊，就是还是要靠脑子这一块，全方位的。要再涉及到说卡脖子的问题，就看国际贸易的这里头会有一些汇率的问题，这都有关系，国际物流的问题它是一个整体很复杂的。不是说现在我学供应链的，我就什么都能干，没人敢这么讲。他是一个很大的工程，你可能在里边某一个环节发挥了关键作用，供应链只要一个环节出问题，这个链就被卡住了。我们为什么叫卡脖子呢？对吧？它是一个很长的链，你卡了一个关键部分，整条链就不灵了。我们说卡脖子卡技术嘛，其实现在这个出口的各种政策他也在卡呀，其实技术本身应该说是无国界的，他现在主要是那些国家他拿的这个东西给你制定一些政策，不让那些公司出口。你出口他就罚他们，这些就很复杂嘛，我觉得要是具体的例子，就刚才说的那个工业工程那个我觉得比较形象。

吕梦娜： 确实有一定专业性。

黄秋莉： 对。老师，我想问一下，你平时除了上课呀做科研，平时的工作状态大概是一个什么样的情况？

受访者： 比较忙，系里边嘛反正也有一些活儿嘛。确实挺忙的，因为你要写文章，你看论文，这个是没有边界，因为学海无涯，就看不过来。

黄秋莉：工作和生活的边界比较模糊的感觉。

受访者： 我家里人会强迫说安排一个时间，我看一下没安排，他们让我去哪就去哪，我就会时不时会有那么一天左右的时间，会被带出去。

黄秋莉： 似乎是不太情愿的样子，哈哈。

受访者： 当司机开车，然后那我肯定干不了啥了，那就休息了，这样也挺好的，要不然我自己可能就老是坐在那看。

刘书博老师： 这样对你也挺好的，我觉得是需要给脑子放松放松的

受访者： 主要是这个对长期老做研究也受不了。

刘书博老师： 对，精神会出问题，要调节一下。

受访者： 需要放空一点儿，是吧？

刘书博老师： 对，有时候我们是在休息，但似乎大脑也在继续工作，是在进行信息重塑。

受访者： 这个年轻的时候多做点儿，精力比较比较旺盛的时候多做，多写一点、多学一点。

黄秋莉： 那你平时忙什么呢？主要忙的是什么样的工作内容？

受访者： 就很多啊，这个科研啊，上课、备课、指导论文，这不都是你能见到的呀。看上去好像不太多，但是它量多，比如说学生有好几个啊，今天指导这个，明天指导那个，是吧？有时候叫到一起还好一点，我东一个西一个，你会发现你一个礼拜你才几天嘛，如果每天都找你一下，其实你时间被切割的就没多少，然后我们还要开会，对吧？然后还有写论文还有讨论，那论文你不能只一篇，对吧？你们现在可以有一两篇，我们现在都好多个同时在进行的，然后这个改那个修，反正就是各种各样的情况，然后那个被拒，然后重新投这个，重新排版等等一系列的问题。

黄秋莉： 果然人生只有越来越艰难。

刘书博老师： 人生是越来越适应新的艰难。

受访者： 读博士的时候呢，你会觉得你挺忙的，压力挺大的，其实你这都是小毛毛雨了，

黄秋莉： 对呀，现在看来工作了以后更难。

受访者： 从事学术研究的话，那你这个状态你要有一个，当然不能还像你以前那种大块儿的时间去做研究，那是不现实的，但是你这种状态还得有。

刘书博老师： 我记得我上研究生那会儿，我那个老师当时还是助理教授，意大利人，他就跟我说过，你们现在还就一篇，每年我们都要写类似你们这样的一篇两篇的，现在他都是教授了，成果特别好。所以我当时我听到的感觉是，“我去你神经病，每年都要来这么一遭”，后来自己当老师后也一样的。

吕梦娜： 那你现在做研究、写文章主要是因为啥动机？

受访者： 原因就考核呀，然后有评职称啊，晋升啊，各种各样的因素，包括带学生啊。然后合作者他有要求啊，你不能说你这儿不要求，他还有要求的呀，所以还要合作继续写呀，你就不能停下来。

吕梦娜：这主要都是一些外部的原因。

受访者：  内部也有啊，你自己也有追求啊，你也想别被淘汰呀，然后能够持续的有成果呀。

吕梦娜：感觉上了一个跑步机上一直在奔跑。

受访者： 当然你强行停下来也能停下来，就说我都放弃啥都不要了，我就停下来躺平，躺平可能不行啊，这个词可能不太恰当，就是说你可能不追求那么多，你就一个最低要求，好像也允许，然后我们现在的制度还可以。我们现在的制度还比较人性化，就是比如说身体出问题了，这时候你需要需要休息了，需要调整了，你住院了，对吧？我干不动了，那我们有这样的制度说你不会淘汰， 但是有些学校不行吧，

刘书博老师： 有一些学校我听说化工还是哪，就是你比如说升到教授了，如果不达标，你就退回到副教授。我听了不是不聘，就是换成低一级的职称了。

比如说我要去中欧，我现在我可能又得从助理教授开始。

受访者：那谁去那儿啊？

刘书博老师： 给钱多呀，他一年都能给美国的那个薪资水平了。那你说姜峰去英国他也是副教授啊。

黄秋莉： 那老师对自己的这种学术期望、学术志向是一个什么样的期待呢？您是要做出什么样的学术成果？你对自己多年以后的那种状态有什么畅想吗？

受访者： 这个可能取决于很多因素，你自己期望有时候你做不到啊，比如说你想写一篇大作，然后成为经典，那当然好了，这个东西就很难的。咱们这个，尤其是现在这个阶段，你想想这个七八十年代管理丛林那么多理论出来的这么多年，这么多年都没有新理论，然后你指望着说某个老师能够有那样的理论出来，可能性很小。所以这个影响因素很多。

黄秋莉： 那您对自己的一个定位是什么样的？

受访者： 继续努力，看能不能按照现有的这种路径上再往上爬坡。

黄秋莉： 那什么样的对你来说是进步呢？

受访者： 目前来说的话，就是看能不能发更好的期刊了。你可能会想说有更好的评价标准，当然也许换个维度看更好，但目前还是想说发个好的期刊，要不你怎么证明你的水平高了呢？进步了呢？比如说我现在发一个SCI，我再发一个SCI，我现在真没有什么成就感，说实话你让我发个SCI我也没什么感觉，虽然对我来说也很重要，但是说就是那种成就感是没有的，当然发了挺高兴的，你也会有绩效什么的，但是你要是比如说我发一个FT，那感觉还是不一样，那还可能很好，如果再发个UTD，那感觉更好，至少这个阶段还是这样。就是我还没有说像人家发到手软，然后就已经不追求了，然后追求别的去了，还没达到这种境界。

在学术这条路上，其实是有很多外部也好，或者公认的一些比较阶段性的一个转折。就比如说一开始可能助理教授到副教授到教授，完了后面的发论文就是SCI，然后再往上。我只能说我还是跟随着那一批人。

就是有些人不是图发论文，我就不发，我就写一本书，然后流芳百世，这样的人有啊，我做不到，我还得随大众来。

黄秋莉： 我总是听到大家说就是努力好几年，然后就像我室友嘛，她就说我先好好学习几年，等我评上教授我就要出去玩儿，然后再也不这么辛苦了，她会这么说。那您身边的人如果是上岸了或者不叫上岸了，就评上教授了，他们会有一些什么样的选择呢？

受访者： 那个话就是大家说说而已，其实你会发现，比如说你在读博士，我读了博找工作，我再也不这么累了，你会发现你停不下来，然后你评了职称之后，你说我再也不干，其实你只要到那个阶段你就停不下来了，你还得继续。

黄秋莉： 你身边没有别的选择吗？大家都在这个世界里面不断向前狂奔？

受访者： 我举个例子嘛，刚才我其实讲过这个话，就比如说你想停下来，你周围的人他希望你不要停下来呀，那你停下来的话你会影响到别人。比如说你带了学生，你停下来学生怎么办呀？然后你停下来，你的合作者怎么办？然后你就很难停下来，这就像上了发条一样。

刘书博老师： 你身边都是这样的人？

受访者： 除非你强制停下来，你强制停下来也是可以的。

刘书博老师： 嗯，嗯，总是要有新的价值方向嘛。

受访者： 对，除非是说你发生一个重大变化，你说我就不做了，我甚至说我辞职不干了，我去开公司去了。不能说没有，但就是比较强制嘛，对吧？“我就是不干了”这样的是少数。大多数还是继续，就是路径依赖，大多数还在原来的道路上继续前进。

刘书博老师： 你的价值感就是来自于你最擅长的那个事情，原来的那个道路，你要再开辟新道路，似乎价值感不会一下子出来，所以这就是路径依赖。所以有很多老师会说，哎呀，这个发表游戏我评上上岸了，我就再也不玩了，但是他不玩了，他价值感就会嗖的下去了，所以他还是要寻找价值的。

受访者： 是的，大部分人还是会继续的。

刘书博老师： 包括就会有一些老师说质性这个，包括当时找工作有一个电子科大的那个老教授，他当时听完我的就说你怎么这么年轻就做质性研究？这研究难道不是像我这样有人生阅历的岁数大的人才做的？我就说你之前都不做，那人很容易就是在一条路径上一直走，到那个岁数和人生阅历也不可能一下就转换到另一个赛道嘛。当时我就是这种回答，但人家就没要。

受访者： 其实对于这样的认知，我也是经历了很长时间我才有认知的，就是没有认知，没接触过。写这样一篇全是文字的东西，你说它很有价值，但是我判断不出来。

刘书博老师： 对，有的老师看完之后就说老太太的裹脚布，又臭又长，全是字儿。

受访者：其实就是不懂嘛，如果你懂的话，你就能发现他的这个闪光点在哪儿。我现在比较熟悉实证研究，我在看实证研究的我就能抓到他的闪光点，我就知道这个文章这里写的好，那里写的不好，但发表了可能也不一定好，但是有的可能发表的期刊不好，但是写的好，我是能有自己的判断的。但是你要让我看这种质性的，我就觉得特别的吃力，然后也看不懂。

刘书博老师： 所以那个老师他说岁数到了才能做，但实际情况还是会路径依赖。那就还是回到刚才初始的问题，那上岸之后怎么样寻找价值？可能会存在路径依赖那样的情况。

受访者： 因为时刻会面临很现实的问题，比如说你们俩现在读博士你们要毕业，这是一个你们当前面临的最大的一个最直接的一个现实问题，而且要尽可能按期毕业，延期也尽量少延，对吧？肯定是有这样的想法。然后就是说如果延期的话，我争取多积累一点，为将来多打点基础，会有这样的想法。那这时候你会发现说你选一个风险小的，标准化大家都认可的，你就会觉得很安全嘛。你要搞一个有风险的，比如说质性研究它就存在不同的价值判断，统计那玩意儿它量化呀，你就看看指标就好了，咱俩指标一写，行就行，不行就是不行，他也不会说指标不行，对吧？他要觉得指标不行，你去找统计学家去，也没人会去找，是吧？这就会比较简单。

刘书博老师： 所以你们现在可能更凸显的是生存价值高一些，那如果上岸之后它不再是生存价值，它可能就是事业的影响力呀，或者是本身的职业价值会更高一些，他不再有生存威胁了。这就是个矛盾，因为上岸以后他也会路径依赖。

受访者： 对对，这就是个矛盾，其实我们倡导多元化，学术越多元越好。因为路径依赖，比如说我现在做什么已经路径依赖了，我觉得质性这个很神秘，对这个很感兴趣，但是我切换成本很高。

刘书博老师： 对。那我们就讨论到了这种学科发展的当下的一个制度特色，就它不是百花齐放的多样性的范式。大家因为要追求这种生存呀或者工作效率，就会退守到一个效率主义的那样的一个路径上。

受访者： 存在这个情况。全世界都是这样，现在你都得生存吧，你说特别是美国tenure-track这种制度，一开始我还不太明白是啥意思，现在我明白之后发现其实这个制度确实有点儿问题。

刘书博老师： 所以就香港、新加坡就学美国那个，包括咱们中国现在预聘制也是，但是欧洲他们就不是，英国就是咱们的事业单位的制度，就进去了就进去了，就是终身制了。

受访者： 姜峰就可以干到老。

刘书博老师：对呀，当然也考核。但是人家不是说威胁生存的那种，他会说你达不到这个科研要求，那我给你转到那个教学为重的，他有个分流，但是你们都还能够很好的生存，所以它本身这个制度会比较多样性的。

黄秋莉： 我看我们学校是不是也有表面上的分流，比如教学教研型教授和教学型教授。

刘书博老师： 教学型教授那个是公共课老师啊，然后我们其实刚才说还是有分层嘛，一般我们会认为高级的还是教研。

黄秋莉： 教学科研，那我们学院没有这种区分。

刘书博老师： 我们有呀。我前两天发给你们看，那个评上正高的那个，他有一个人叫师文杰啊，他就是教研教学型，他是公共课的教英语的老师。还有我们书记何秀超，他就完全研究员嘛，研究型、教学都有。但是我们其实最看重的还是教研的这种，因为我们觉得这个难度最高。

受访者： 数量比较多，大部分都是教学科研。

黄秋莉： 嗯，那您现在的工作和您最开始在电信的工作会有很大的差别吗？现在这种工作状态是比较满意的吗？就是学术的这种状况，会觉得和以前那种比较程序化的、比较枯燥的、不能发挥自己批判性的那种状态有很大的区别吗？

受访者： 区别那肯定有的呀。就是大学里的老师有个特点，就是你要在该到的时间点，必须要及时赶到，你要克服一切困难赶到。比如说你要监考，那必须不能出任何问题，然后这个过程要很严格的执行，这种强度很大，压力也很大。还有就是上课，因为学生在等着你，对吧？你不管什么，除非你真的是来不了，你要尽可能要来。就是除了这些之外了，我们会有一些这个时间上的弹性。就比如说你家里有事情，那正好，你现在不需要去监考或者不需要上课，你就有弹性，你可以安排家里的一些事情。其实对于个体来说的话这个其实挺重要的。

以前在公司工作的时候呢这个就做不到，你不管你什么原因你可以请假，你打卡，每天就去刷卡，然后上班刷下班刷，然后有时候你干完工作了也就在那儿你不能走，你走了也没意义，上午干完了有半个小时，但是你下午还要继续，对吧？你就不能走，就相当于把人拴在那里了。这个是一个很明显的一个差别。

刘书博老师： 我们自主性会强一些。

受访者： 嗯，对，我以前就特别强的欲望，希望放假休息的那种，就是我会想着什么时候我能连着歇三天啊，我啥也不干就歇三天，就这种期待特别强烈，现在就不是很强烈。

因为工作是程序性的嘛，有时候做项目，你就会持续很长一段时间，你就不能休息，不是说你就像一些行政里面盖章那种的，不是那样的，我们做的也是多少有点儿研究性的。有时候也是要加班加点，就有很长一段时间不能休息。有时候这个完了之后下一个马上就来了，甚至说这个还没结束下一个已经来了，然后就可能持续一两个月，你都没时间休息，就特别有压力，特别崩溃，就希望我能连着歇几天，我啥也不干。

然后另外就是有事儿的时候希望请假什么的，那时候那个请假程序就很麻烦了，你要是跟领导去说，你没有什么理由，你说我累了，我就想躺着睡觉睡半天，我心情不好，那这是不可以的，是吧？但是大学老师只要你没课，你可以在那躺半天，然后回想回想人生，思考思考问题，就给你这个时间去思考。

黄秋莉： 确实在行业里工作这种自由时间很少。

受访者： 你把工作得做到嘛，因为拿工资了。（自由度）这个差别是最大的。

黄秋莉： 所以大家都觉得大学老师是一个非常好的工作。

刘书博老师： 嗯，嗯。

受访者： 哎呀，这个只能说看适合不适合。

刘书博老师： 其实还是陈老师说的，批判性思维其实它是一种独立性的思想，我觉得那种把很多不是这种的思想状态，他会把大学工作变成工厂式的工作。

受访者： 嗯，对，其实在大学工作，你想想其实压力也挺大的，你比如监考啊，上课这些其实投入都很大。就是你们现在不上课哎体会不到，就是压力挺大的，特别是年轻老师刚开始的时候，但我现在到这岁数了，我上课因为讲了很多年了嘛，很熟悉了，就好很多。刚开始的时候压力是很大的，一个课给你，你以前觉得是学懂了，但是你要把别人讲懂，这是两个层次。

吕梦娜： 对对

受访者：这个是不一样的，刚开始你又要发文章，然后你工资又低，然后住房也有局限，方方面面也都还得起步会。

刘书博老师： 我是觉得大学老师最有意思的就是他有个体发挥的空间和自由。

受访者：你是几年级了？

黄秋莉： 我是硕博连读的，这是我第四年了。

吕梦娜： 我是硕博连读，我是第三年。

受访者： 硕博连读五年可能短了点，大多数人都短了点，一般六年差不多能出来，五年可能除非你特别的用功。

黄秋莉： 您这么说，让我心情变好了很多，让我自我怀疑没有那么强了。

受访者： 是这样的。我师兄那边有一个学生也是硕博连读嘛，他是六年，也是很赶非常赶，他也是非常努力，非常赶六年才出来的。正常五年是出不来的，你上完课再开始起步科研，然后在发表写论文时间上肯定是不太够。

黄秋莉： 对，特别是对我这种跨专业的，确实还挺难的。

受访者： 本专业也不太够，这个博士还是很专业，专业还是很强的。

黄秋莉： 但我还是会觉得好像是自己不太行才导致我自己要延期了。

受访者： 学海无涯就是积累了，如果你要从事科学研究的话，时间的积累是很重要，特别是在年轻的时候的积累。做研究它有个特点，就是一分耕耘一分收获，你没有去耕耘就没有收获。你想很快的去出成果，可能还真不太行。学术研究好像就有这个，我理解是有这个特点。

黄秋莉： 我听哪个人说，他说学术是最公平的。

受访者： 比较公平。因为你投入时间了，他就会有产出，你要是没投入想很快的产出，可能真不太容易。

刘书博老师： 这个也是我走学术道路一个原因，对于我来说就是它是相对来说还是公平的。嗯，有一些工作就不是这样。

受访者： 就是对你来说多一年就多水平积累的，积累的东西多，然后水平就会更高一点。那将来因为你长期持续嘛，你这个起点就高，别人虽然已经毕业了，但是他起点没在那儿，对吧？他后面还要补。

吕梦娜： 你这么说我心里好像多了一份安慰。我认识一个年轻老师，他就是顺利毕业了。然后他就跟我说现在压力特别大，因为他顺利毕业当时可能就是刚刚达到学校的要求，并没有太多积累，他说如果可以重新选择，他宁愿主动去延期，就是前面多点儿积累，后面可能还好过一点儿，你要长期的来看。

刘书博老师： 要长期主义。还有一点就是也不要完全是达标的心态，还是要开拓一个能够让自己有动力去做的那个赛道吧。

像我那个之前在中欧的老板，他们都是公司化，他前面就是特别精致、优秀，就是康奈尔、哈佛、剑桥一路走下来，然后全是全优。但是他现在已经枯竭了，就再也不想去学习，他好奇心已经完全没有，完全就那个时候把他那种完全给压抑，那种学习模式就是应试似的、达标似的。那后来他那个心灵就枯竭了，咱们都探讨过，你前面可能产很多，但是属于揠苗助长，或者是化肥用太多了，后面这个土地就板结了，就生产不出来东西。

黄秋莉： 对。其实我有一个同学还跟我讲过他自己这样的一个看法。他就说现在好像大家都在为了发论文而发论文，他好像没有学到知识，只是学会了怎么去发论文。

刘书博老师： 好多人现在都不看书。

黄秋莉： 对，他说他没有觉得学到了知识。

吕梦娜： 他就知道了很多技能。

黄秋莉： 对，他是这么说的。

受访者： 这个我是这样认为的，你是在读博士啊，我倒觉得这是正常的。你是不是觉得不正常？

黄秋莉： 我是觉得他会觉得自己有种知识匮乏感。那我们本来就是博士研究生嘛，我觉得作为一个学术人员，你不应该觉得自己没有学到知识，他觉得自己学到的是怎么去工作的饭碗。

受访者： 我对这个问题的看法啊，就是我倒觉得你说的那个情况是对的，就是可能跟你的看法有点儿稍微有一点不同。

为什么这么说呢？就是你当你念大学的时候，你是要学知识的，有时候你可能不需要知道知识是怎么来的，你只需要知道它是什么就够了。当然你如果能知道怎么来的更好，知其然知其所以然更好，但是你可能因为专业嘛很多课，是吧，你可能没有那么多精力，那你会发现你学到很多知识，一个新的课，一个新的方法，一个新的技巧也学到很多。等你读到研究生的时候，特别是当硕士研究生除外啊，特别是这个读到博士研究生的时候，你这时候已经不是学知识了， 你是知识生产者，你是要给别人创造知识的。所以你这时候你还抱着一个学习知识的态度的话，那就坏了。就是像到了你这个阶段之后，教材是基本上是不看了，你是创造出来给别人看的，你需要看的是什么呢？你需要看那些经典，因为他会给你开拓思路，帮助你加深思考。

黄秋莉： 但可能现在的问题是我们也没有时间看经典。

受访者： 就是你不要贪大求全，你知道吧？就是你把这个"做题"都看的差不多了，就你需要小步迭代，你先有一个文章，你就把这个文章有关的东西你把它整明白了。然后你会再写第二个，然后你不断不断的，你这个就是画圆嘛，开始这个小圆，但你这个圆你画的很好以后以后图片变大，等你积累多了之后，你慢慢慢慢你就能知道的，越来越能融会贯通了。

而且你开始画那个圆很重要，一定要找一个质量比较高的人，要跟着一个水平高的老师指导这样来，然后或者跟着一个比较高的合作者，然后能够带着你把这个先做好，这很重要。再一个就是哪怕你够不着，你也有人拽着你在高水平往那儿攀登了。

你要觉得你很舒服，然后你什么都会，在那个低层的徘徊，你觉得你很好，其实你距离那个高的还差的很远。

黄秋莉： 嗯，所以就是痛苦是正常的，说明你在往上走。

受访者： 对的，对的，如果你觉得舒服了就不正常了，有点不爽，实际上你是在进步，这样的话对自己的状况有一个比较好的认知了。

就是不能说崩溃吧，对吧？咱们说崩溃的时候可能有点儿太那个病态了，就是你会觉得你特别的不想弄了，都特别想这个发泄大喊，然后把电脑砸掉，然后写不动了，然后回过头来调整一下，回来接着写。然后你再调整回来之后，你一定能涅槃重生了。

我记得我有个学生写了个论文儿，我说你整理整理吧，他就按我的要求整理，整理完了之后他就跟我说老师我都不想写了。嗯，我说没事儿，我告诉你怎么一点点弄，要不你就先歇两天。确实是很崩溃的，但是他一轮一轮下来之后最后发表出来了，那个文章虽然是个问题文章，但是他的水平就上来了。

黄秋莉： 是，老师这么说我觉得好多了。老师您带学生就是那种就是感觉特别好那种，对待学生的方式很理想的那种。

受访者： 怎么会这样的认识？

吕梦娜： 就感觉有良师益友那种感觉，就是你会说让他不行的话歇两天，或者一直让他修改，你也帮他去改，我就觉得特别好的那种状态，可遇而不可求吧。

受访者： 反正我努力这样做吧，有时候也也着急呀，因为也不是我的学生，是我师兄的学生，也不能批评他，是吧？要关注他的情绪吧。

黄秋莉： 所以做老师其实是个很高很难的工作。

受访者： 其实这个说实话，就有时候比如说那个论文该怎么写，有时候我也没有完全想清楚，他也是在不断的迭代，特别是修改意见来了之后你怎么改，我也不知道怎么改是最好的，对吧？要考虑方方面面，最后一起讨论。其实就是在指导学生，我也在学习，教学相长。

其实合作者就是其实就是要创造知识，其实说白了我们就是创造知识生产知识的。不要有你说的那种我要学到很多知识，而是要创造知识，看完你的东西让别人觉得你论文挺好，挺挺受启发的。这个是我们要做的事情，如果你能出去出一本书，让别人看了之后花钱买，然后花钱买之后还放到床头上去看，那你这贡献就大了。

刘书博老师： 嗯，行，陈老师跟我们聊了这么久，两个小时了，谢谢陈老师。

# 受访者17

刘书博老师：顾老师，那我们首先想了解一下，因为当初选择本科就是管理学院

受访者：本科是经济学，国际贸易

刘书博老师：国际贸易，那研究生才转到管理学

受访者：对

刘书博老师：那当时就是有什么想法，就是转专业......

受访者：因为当时是在法国读的研究生嘛，所以法国XX高校没有可供太多选择的范围，就只能在管理的那几个门类里选。

刘书博老师：嗯，嗯

受访者：然后最后选来选去，觉得可能还是市场营销比较合适一点，因为之前也上过一些什么跨国的、国际商务之类的

刘书博老师：ok

受访者：就是那种方向的，然后所以觉得跟市场营销可能对接比较方便，比较容易啊

刘书博老师：那学了之后觉得有意思吗？那当然这个问题也不好回答，这整个学科就是......

受访者：觉得这个东西就是从学科本身的知识和做研究的两个角度考虑吧，就是学科本身的东西是挺有意思的。

刘书博老师：嗯

受访者：就是市场营销的一些实务，一些这种案例什么的，确实就是不同国家的，然后对比对比、分析分析，觉得还挺有意思的。

刘书博老师：嗯

受访者：但是如果你说从研究的角度去看营销的东西，就尤其营销现在研究的东西，我是觉得就没有那么有意思了

刘书博老师： 现在营销研究什么？

受访者： 营销研究就是几个方面嘛，一个是CB就是做实验，然后挖一些消费者心理，特别深层次、特别细节的一些东西

刘书博老师：然后脑电波

受访者：对对对，也有往那个神经营销学方向走的嘛。但是基本上就是借鉴心理学的那些理论去研究这种消费者层面的。然后还有一些研究，这个营销的分法挺奇怪的，他们把这个营销一方面分成CB，然后一方面就是营销模型

刘书博老师：CB就是consumer behavior ？

受访者：对的，然后营销模型那方面呢其实不是按研究主题分的，是按方法分的，这两个分类我觉得是不对等的。

刘书博老师：嗯

受访者：然后营销模型的建模，那一方面它就是用一些计量模型去拟合一些这种现状。

刘书博老师：嗯

受访者：那些就是有CB的题目，也有战略的，偏战略层面的，也有一些就是纯数学模型的，所以就是分这两个方向吧。

刘书博老师： 嗯嗯，那这种分法是谁分的？是美国那边的学术标准，还是...

受访者： 我觉得可能是按照美国那边标准分的，但是我觉得在国内可能相对来说改进了一些，因为这里头其实有一部分，如果你按主题分，其实应该是分成CB和战略；然后按方法分实验和建模和计量；三类方法嘛，它既不是按主题分，又不是按方法分，它就是按研究的人数，按相对人数分，估计是这样

刘书博老师：哦，那这个是比较主观性的分法

受访者：对对

刘书博老师：它不是非常合理的那个

受访者：是的

刘书博老师：好吧，那现在您是研究在哪一块儿？

受访者：要是按营销这个来分的话，那肯定是营销模型这一块儿

刘书博老师：ok，那这一块儿研究现状怎么样？

受访者：研究现状就是国内基本上不太多吧，应该说是不太多，国内反正我知道就是北大的、人大的都偏做CB，偏做那个实验的方法。然后有一小部分人是做营销模型的，然后这里头就是可能还分，就比如说有的人他可能能从那个企业拿到一些内部数据，什么京东啊这种数据，然后去做一些这种线上的消费者行为的分析什么的；还有一些人就像我这种可能也拿不到特别好的数据，然后基本上是从战略层面去做，就是拿企业的数据去做

刘书博老师： 嗯，嗯，嗯，就是企业怎么做决策？

受访者： 对，就尤其是这种营销的决策或者说营销决策产生的影响之类的。

刘书博老师： ok，ok，那当时就是读了研究生，在法国，然后博士是在人大

受访者：对

刘书博老师：那为什么选择继续读博士走学术？就那个时候有没有想法说我去行业...

受访者：就是也去行业干过一段时间，但是呢觉得其实意思也不大，因为行业里头、企业里头做也是做一些日常化的事情，可能是一个周期

刘书博老师：routine的那种

受访者： 对，特别对，就一个周期可能就一个轮回这种的，就是说你第一个周期可能是比较新鲜的，然后觉得第二个周期可能慢慢就没有什么...

刘书博老师：就变成程序化的那种重复工作

受访者：对，所以觉得要不还是回来读个书吧

刘书博老师：ok，嗯，所以您当时会觉得读书会经常会有新的，然后学到新的

受访者：对，至少是还是在探索世界吧，或者说就是能够了解一些新的知识什么的

刘书博老师：嗯，嗯，然后博士就是营销这个，那博士期间有没有觉得压力挺大的？不知道人大这个博士生的培养是什么特色？

受访者：还行其实，可能我觉得现在的博士生就是你们可能压力比较大，对吧？但是我们当时人大好像只要求发两个C刊就可以了

刘书博老师：然后北大核心和南大核心都可以

受访者：呃，不是，人大自己有一个自己的list。然后那上面其实基本上北大核心、南大核心都有，但是也不是完全都包括了，同时还加了一些人大自己觉得挺合适的期刊，但是又是两个都不是的。所以就是说只要在那个列表里头发那些期刊就可以了。所以相对来说毕业肯定是比较容易的，除非就是说你想要有更好的成绩，或者是想要再做点自己的东西，可能会有一些自己的那种压力。

刘书博老师： 那现在还是这个标准吗？

受访者： 好像中间调过一次，就是往高了调了一次，但是后来又好像又降回来

刘书博老师： 又降回来了

受访者： 对，现在不都是有点取消这个论文标准

刘书博老师： 对对，那这么看起来我们标准比人大还高

受访者： 而且现在人大好像说不用两篇C刊，一个C刊、一个C扩都可以哦，就是它对那个发文章并不是有很高的要求

刘书博老师： 嗯，那是比较合理的

受访者： 对。咱们学校应该是逆风而行

刘书博老师： 咱们是学校这样要求，还是学院这样要求

黄秋莉：学院（要求更高），学校只要发一个就可以

刘书博老师： 那咱们学院就是大逆不道哈哈哈

黄秋莉：老师可能是为了我们就业着想

受访者：不，也有人说咱们学院是试点

刘书博老师： 哦，我们是试点，就是我们合适了，就全校铺开了

受访者： 可能是这个意思吧

刘书博老师： 那就是逆这个潮流了，（其他）人现在都是在去五唯，咱们学校是在加嘛，这是何苦

吕梦娜： 我之前好像听林院长给我们上课的时候说过这个事儿，好像是之前有个学生的论文被查出来，好像不太合格，所以他又增加了一篇

刘书博老师：所以还是为学生好，是这样说的

那您觉得博士的这个训练是跟当时选择学术是一致的吗？你一开始觉得在公司还是routine，学术会探索发现、不断学习，那真正进入学术圈儿之后会不会有一些出入？

受访者：这个就不好说，就是在学习的过程中或者说在自己探索的过程中依然是符合预期的。但是呢一旦涉及到发文章或者说投稿，就到这种后续的事情其实就已经完成了探寻知识的过程了，现在后面的步骤就是说要把你的知识告诉大家。

刘书博老师：嗯

受访者：但是这个步骤就不太···其实和公司里也一样，就是说还是要受很多东西约束，不是说你要表达的东西，真的都是你特别想要去说的东西。你在写东西的时候还是要包装包装啊，然后可能就是要看看有没有一些奇技淫巧在里面。

当然不可能每个工作都是真的能特别理想，就是说这个工作（教职）反正大部分是好的，但是还是依然有一部分没有那么吸引人。

刘书博老师：ok，像咱们都在国外，尤其是在欧洲待过，那会不会觉得欧洲的那种学校文化氛围跟咱们是不一样的？

受访者：肯定是不一样的。最大的感受是国内的学校有很多的制度约束，对吧？其他的就先不说，就是财务制度，这个就是特别从中央到下面的很强的约束。

然后除了财务制度，比如说像是老师的一些方方面面的，包括对学生的这种制度，这种约束其实也特别强，然后还有就是从思想上面的，就是要求必须得一致，或者说行为上的一致，这种东西好像就是差异比较大。

刘书博老师：是的，因为我觉得像欧陆，法国我不了解，但是我会听说法国比英国更加的理想化、理想主义，那它背后就是对于个体的这种充分的自由的表达。英国反正我在那边感受到就是对老师是相比这边来说要更信任。像我当时在读博的时候让我出去开会，回到亚洲开AOM年会，然后报销，他们几乎都不审，就我直接填个数，他们就直接给我报了，咱们现在这个发票贴的特别整齐。

受访者：然后他有的时候他还要问你这是为什么发生这笔费用？

刘书博老师：对，有时候你还得必须订酒店，要不然他觉得你去没地方住，你不是在骗我吗，我就住公园我自己躺着不行嘛哈哈哈。

受访者：而且他有时候那种要求就是很莫名奇妙的，比如说你报销一本书，他非要让你写这本书和你的研究到底有什么关系？

刘书博老师：这个让我想起来，就我跟姜峰聊，把他给气坏了，他买了一本小说，然后那个财务就给打回来了，他说小说不是做学术，他们就给你判断了。

受访者：那天我在学院报了一本书，就是讲那个印象派艺术的一些书。然后院长就说这个书和你的研究有什么关系，他不理解，我说算了，那就别报了。

刘书博老师：其实有关系啊，像案例是纪实的，尤其是做话语分析的就很重要呀，但是他们非专业人士在评判我们的专业选择。对这一点就很气。

受访者：我主要觉得就是说老师的这种研究科研，不一定灵感完全是从专业里来的。我看一些那种发散性的东西，其实不一定就是说跟研究有直接的关系，但是有可能有间接的关系。就说你不能限制老师去探索这个世界，然后包括就有的东西可能跟研究没关系，但跟教学有关系啊

刘书博老师：所以你看我们这个例子，就每当提起我都义愤填膺，虽然不是我的书被打回来，就很气呀

黄秋莉：那如果您的书被打回来了怎么办？

刘书博老师：那我就找他吵架去

黄秋莉：您自己不应该会买很多书吗？

刘书博老师：那我都忘记报了，或者说我就在网上下载，买的时候还是比较学术相关的那种。

受访者：财务是这样的，他要求你写一句话，承诺说我的这本书和我的课题或者说我的教学研究是有关的

吕梦娜：那每写一次这句话不觉得很难受吗？

刘书博老师：所以有时候我们作为老师，尤其是在对比过不一样的欧洲那种，他们就充分的信任你，这边就是一种制度性的不信任。

受访者：包括教学也是，欧洲的教学其实就很随意嘛。比如说今天天气好，大家都出去去草坪上，或者说我们今天就不上课啦，然后晚上一起去看一个电影，大家回去写写读后感，或者是交流讨论，就可以了。但是国内就一定说你必须得在课堂里完成，如果你迟到了或者早退了，或者是组织学生出去了也不行，或者说如果你要有这个计划，你就要很多很多的审批手段

刘书博老师：对，是的，

吕梦娜：所以是劝退，是吧？

受访者：就算了呗，老老实实上课呗。

刘书博老师： 所以我们就很明显的感受到一个标准的存在，它对于多样性、探索性的不是很鼓励的。

在15年我们就来到中财这个学院，整个过程怎么样？你感觉就有没有一些之前设想的与现实中是有比较大出入的。就比如说刚才财务的这个事情

受访者：财务这个东西其实各个学校都差不多

刘书博老师：对，中国特色

受访者：对，所以这个东西其实还好。

刘书博老师：嗯，嗯，那我们再聚焦一下，就是像大的管理学，有没有就是觉得在工作之后会发现，诶竟然是这样的。

受访者：就是说落差比较大的这个，我可能最强的感受是这个基础设施，咱学校基础设施确实是不太行的，然后它和理想中的或者说我们见到的商学院的那种感觉是不太一样的

刘书博老师：对，我们是属于乡村企业家

受访者：就是特别古朴的感觉。我觉得这个确实是和商学院这个概念有一点冲突吧，然后包括和其他学校的商学院的这种感觉也是不太一样。然后包括感觉咱们学院的这个氛围其实是好的，就是相对来说比较团结一些，或者说比较民主一些，不像其他的商学院特别的···其他的商学院好像还是有点资本主义的那种感觉

刘书博老师：剥削压迫

受访者：对

刘书博老师：资本主义就是非常市场化的那种

受访者：对，比较市场化。

刘书博老师：嗯，嗯，就很自私，各自都在充分的想要把它变现

受访者：对，然后老师之间可能也没有那么多的嘻嘻哈哈呀，可能都是自己在干自己的事儿，或者出去讲课挣钱。但是咱们学院其实相对来说这个氛围还是好一些

刘书博老师：我们学院氛围还是好，整体来说还是传统一些

受访者：对，是的

刘书博老师：我同意就是咱们虽然没有那么快的发展，但是相比于上财和对外经贸，我们还是保留了传统的一些好的东西，像对外经贸就非常市场化的

受访者：我就是在学校里长大的嘛，就是我爸我妈都是学校老师。所以有的时候我能从咱们学院身上看到90年代（的身影），就是我小时候看到的那些学院之间的人际关系的那种感觉。

黄秋莉：那您是觉得喜欢吗？

受访者：就是觉得挺好的。

刘书博老师：就是中财我们有好的也有不好的，我会觉得是好的，这个发展慢，但是它没有外部的那种比学赶超（虽然短期看着很快，但是它有副作用）。

黄秋莉：那就感觉跟我们校区一样，它一直也没有变化，那么多年间长了

受访者：这个可能是受限了，也不是说学校不想做，只不过是不让做，因为那几栋平房不是说是借来的嘛，原来卷烟厂的资产，说不让去盖高也不让翻修也不让它外观变化。

刘书博老师：顾老师父亲是马克思这块儿，就我们会有时候还要坚守一些马克思对于资本主义的批判，就资本主义它是有副作用的，比如说像北京的发展也是高楼大厦都起来了，但是他的背后是好多古建筑就被拆走了，是那种文化的这种这种失去。所以有时候我们现在都在比学赶超的追求市场化价值、经济价值，但是学校本身它不是市场嘛，那如果都是市场逻辑，那学校原本的逻辑就会被排解

受访者：是的。

刘书博老师：所以你父亲是怎么评价这个事儿

受访者：我没有问过他这个问题，

刘书博老师：那他研究的主要是哪一块儿呢？

受访者：他研究经济思想史，尤其是马克思的经济思想史。

刘书博老师：中国现在应该是跟资本主义的那些市场机制都不一样，清晰的不一样

受访者：必须得是不一样

刘书博老师：我们会跟新加坡是比较一样的嘛

受访者：不，新加坡好像就是家天下的那种感觉。

刘书博老师：哦，对，他都是家庭成员

受访者：都是那个李光耀李氏后代。然后就是一代一代传下去，然后包括他的很多大企业好像都是那个家族控制的

刘书博老师：但是他整个也是为全体人民的那种，他是有强烈的关怀伦理在

受访者：这个就不知道。没有深度去了解。

刘书博老师：那像现在迹象也比较清楚，比如有四六级会考井冈山呀，然后也不组织圣诞节啊，然后会倡导说文化自信和理论自信嘛，那会不会说之前像那个朱镕基时那个时候就是非常新自由主义的那种，那慢慢的这套不再是那种新自由主义，我们会有自己的一个很清晰的、不同于美国的那种发展模式

受访者：我觉得应该是在探索吧。因为就是说经济发展它可能每一个阶段适用的理论都不一样，可能之前中国经济是比较落后，需要快速崛起，或者说快速发展，你就得带动所有人的积极性。然后就像这种自由主义可能是比较适合的，但是如果这个自由主义放任他去发展，肯定会带来很多的负面作用。所以这个时候可能就不能让他放任发展，就得用一些这种思想的去引导大家。

刘书博老师：ok，所以上层还是看的很清楚的

受访者：我觉得应该是有考虑的

刘书博老师：就像你父亲他是专门要建议政府去看到这些嘛

受访者：我不知道他在建议什么，我也不太了解他研究的东西到底是什么？但是他好像更多的研究是历史的东西，就是说马克思的思想是从哪儿来的？然后在中国是怎么传播开的。

刘书博老师： ok，嗯，那有点像史料性的研究。那你的研究能跟那一块儿结合吗？就比如说营销在中国···

受访者： 我之前博士论文写的就是品牌的思想史，但是为什么结合很难呢？因为我觉得品牌发展就没几年，那管理学在中国的发展其实相对来说是比较短暂的，而且这种思想性的东西不一定是中国的。就是如果要局限在中国的这个角度去研究管理思想的发展，其实我觉得沉淀的还不太够，要是从国际的、全球的管理学科的发展上来说，可能相对来说更值得研究一点。但是这个东西可能就和中国的现状又会有点脱离。

刘书博老师：Ok，那你现在做的研究是什么

受访者：我一开始做的就是偏营销战略的，然后呢就慢慢的又做一些战略的研究，然后后来又做一些公司金融的研究。最近研究比较多的可能是把（心理学的理论结合一下），因为营销比较关注心理学层面的一些理论嘛。然后这个心理学（我）之前讲过一门课，就是消费者行为分析里头用了很多这种非理性决策的这种理论。然后主要是把那个理论套用在企业决策里头，看看企业在决策过程中有没有一些这种非理性的特征。

刘书博老师：就行为经济学。

受访者：对，有点儿像行为金融

刘书博老师：这一块儿挺前沿的。

受访者：嗯，但是其实你从公司金融的角度上来说，他们其实做了也也有一阵子

刘书博老师：ok，但是这个在营销领域做的不是很多吧

受访者：营销领域基本上没有人做这个。但是其实如果从战略层面或者从企业层面去研究营销的问题，那肯定和企业的其他战略包括公司治理什么的就是分不开了。所以如果从企业层面去切入，其实我觉得这些问题都是一个大的问题。

刘书博老师： ok，那还回到刚才说的咱们学院是比较像90年代的那种氛围，那有没有其他学院不是这种氛围，像您刚才说的就是比较个体化，就是企业的那种感觉，大家都在一个学术市场上去竞争

受访者：就是相对来说可能更市场化一些吧。就像人大，人大可能比咱们市场化一些，但是它又不是站在市场上最前沿的

刘书博老师：最前沿的是谁呀？

受访者：最前沿我觉得应该是北大吧，北大清华应该都比较前沿，就都比较偏市场化，因为人大那个学校的性质是官校，就是党办的学校嘛，然后整个的阵地都比较偏“左”一点，所以它的那个商学院肯定也不会太夸张。

刘书博老师：嗯，这么说是像清华就预聘制，北大也是预聘制，然后包括那些年长的老师他们也要走预聘制，这个预聘就完全是一套体质，也不是“新人新办法，老人老办法”，那挺残酷的。

受访者：对

刘书博老师：因为有一些上了岁数的老师···

受访者：还得让他回来跟年轻人竞争，其实不太可能争得过

刘书博老师：对呀对呀，那就很残酷嘛。那为什么北大清华要做成这样，就是为了排名？

受访者：我觉得可能是竞争吧，因为他们得想办法，让这些上了年纪的、或者已经评了教授的老师不能躺平，必须还得动起来，还得贡献管理新知

刘书博老师：但是这个游戏规则，其实都是美国那边定义的。像我们战略，几乎就是美国人（定义），工商管理都是，OBHR尤其是这样的，而且都是在向物理学方向发展

受访者：就是理科的那种

刘书博老师：理科对对，管理工程就很吃香。他们是没有这种像文化的影响。但我觉得像尤其战略人力资源和营销都是受到社会文化的影响，那这个对像那些上了岁数的老师就很不友好，他们那一套严谨的、实证的、模型的，他们都没有经过比如说在海外的那种培训。

受访者：所以就是那些老师可能会找自己的路，比如说做案例，清华不是有个老师叫李飞，应该是营销这个领域的，然后他就找了个自己的路，就做定性的分析，然后做案例什么的，他不去搞那个量化的

刘书博老师：那个我还没听说过，回头看看。那他做案例的研究也是发到世界期刊嘛

受访者：不，就国内的一些期刊

刘书博老师：哦哦，然后好像有一个北航的欧阳桃花，也是做案例申请到了重大课题。

那现在比如说我经常听说像中山大学、武汉大学，他们会有这个比较大规模的师资博士后。然后应该这个也算是市场化的一个产物

受访者：其实咱们学校不是也有几个？就是咱们学院里也有几个师资博后

刘书博老师：但咱们还是比较友好的，就来了之后大概率是可以留下来的。像中大应该大概率是留不下来

受访者：就也是得靠竞争才能留下来嘛

刘书博老师：他们好像每年招十几、二十几个师资博后，然后只有一两个名额最终能留下，还是挺残忍的。

受访者：那就是说留下的人不仅成果得好，而且关系得好。就是这三年不仅得把自己的成果弄好，还得跟院里的这些系主任啊人际关系弄的特别好。

刘书博老师：对对，所以当时我找工作的时候有两个offer就是华南理工和同济大学，他们就给我师资博士后这种，我就没去，因为我在面试的时候就感觉不好。就是一个大房间，所有老师在里面。外面排了好多人一个个的进去，就是流程式的，它也不是特别想了解你

受访者：就跟那个企业招聘的感觉似的。

刘书博老师：对，就企业化。所以现在就商学院可能企业化的还挺激进，但我不知道其他学科怎么样

受访者：其他学科就不能说是企业化，就是说它很在乎利用竞争去激励这些老师去出成果。然后这样一个结果就是老师会特别的短视，对吧？就是说什么东西是现在研究的热点我就去做，然后什么文章好发我就去做，然后什么样的方法用的特别的多或者说特别容易接受我就用那个方法去做。但是呢自己想要研究的那个东西沉淀的就不够。

刘书博老师：是这样

受访者：就比如说那个金融学院，我可能比较了解吧，他们就会有很多的这种对年轻老师的这种激励

刘书博老师：他们是怎么激励的？也是发钱吗？也是像咱们这种发一篇给多少钱？他们发的力度怎么样？

受访者：其实跟咱们差不多，但是呢他们会有一些奇奇怪怪的制度，这个制度一上之后你就会觉得钻不了空子，就比如说那个A类期刊咱们是2万，他们也差不多。但是呢比如说你发现A特别好发，或者说有一些A的期刊就是很容易发出来，然后就会有人使劲儿去发那个期刊。这样的话呢，他一年可能能发十篇，那就20万就拿到手了，对吧？但是呢他们就设了一个限制，说如果你只靠A去拿那个奖励的话，你的上限就是15万，如果你想突破上限拿的更多的话，就是你的这个奖励里头必须有一篇AA类期刊，然后AA的上限呢是30万， 然后如果你想再拿更多的话呢，就得有AAA。

刘书博老师：那这种在咱们商学院的现实中还没有出现

受访者：因为咱们可能没有他们那么容易发表，他们可能特别喜欢算，所以有的老师就特别会算那个漏洞，然后每年就拿了好多好多钱

刘书博老师：那也挺厉害的

受访者：所以他们院长估计就在想，哎呀，这个不行，再这么发下去，我要赔了

刘书博老师：那你可以跟他一块儿写，然后你们双学院拿（奖励），double

受访者：对，这个空子可以钻一下哈哈哈

刘书博老师：所以他们在制度建设方面还是比我们要先走一步

受访者：嗯，因为他们可能发现的问题更多一些

刘书博老师：但是也反映他们更能发，为啥更能发呢？是因为年轻人多嘛

受访者：因为首先第一个期刊多，像这个国内的期刊，经济类的肯定比管理类的多，然后管理类的期刊也发经济类的期刊，但是经济类期刊不会发管理类的文章。所以这样一看，这种经济金融的版面肯定会比管理类的多很多，然后还有国外的期刊也会比较多

刘书博老师：明白，那他们的AA期刊是他们自己定的嘛

受访者：他们不认学校的那个AA期刊列表，中文是和学校的AA期刊列表吻合的，然后英文他们有一个自己的list，是按那个走

刘书博老师：好像这种就是发论文给物质奖励的国外好像没有吧。英国好像没有，你了解吗？好像是一个中国特色

受访者：可能是，反正我没听说国外的哪个老师靠奖励活着呢

刘书博老师：所以这个也是挺中国特色的。北大清华他们也有吧？

受访者：肯定有。但是他们是这样，就是说年薪制的就不发了，就如果北大清华他们都是年薪制的老师的话，就没有这个问题。因为年薪制就是打包成合同，然后一年给多少钱，发论文就是合同规定的嘛，除非超额发了论文之后再给奖励。

刘书博老师：是的，我听说那个咱们有一次开会，不是院长说我们都赤字了，都破产了，那还有钱发这个科研奖励？

受访者：不知道

黄秋莉：咱不是有1亿在这儿嘛

刘书博老师：1亿被拿走了嘛

黄秋莉：被拿走了啊，我以前以为我们学校好有钱，现在完了哈哈哈

刘书博老师：我们上次开会说了已经赤字了。就奖励科研的这些钱是那儿来的？

受访者：财政拨款吧

刘书博老师：财政部发的？

受访者：对吧，应该是教育部拨款拨下来的，不是每年每个学校按等级拨多少钱那种嘛

刘书博老师：就专门说让学校奖励科研？

受访者：那没说，那是学校自己这个盘子规划的，或者不是说每个学院要有那个绩效考核，然后他可能想不出别的东西，就用这种科研考核吧。

刘书博老师：那我能够想象就是如果极端情况的话，一个学院内部的贫富分化就非常的明显

受访者：对

刘书博老师：但是好像中央说过禁止物质跟科研直接挂钩，但是我们也可以通过这种积分换算的直接挂钩。

黄秋莉：比如说上面不让唯论文，那我们就加了让我们发《人民日报》这种或者是去得到国家课题，这就不是唯一的了

刘书博老师：但是那个更难，好机灵啊

受访者：所以其实以前大学里头老师没人愿意发论文，就是像我爸那时候刚当老师的时候发了好多论文，我说你咋那么容易发呢？然后我爸说没事儿干就写嘛，然后那个期刊也缺文章，然后有人投稿，他们特别高兴，现在完全反过来了

吕梦娜：供求市场发生了变化

受访者：所以那时候其实不以这个（论文）考核。

刘书博老师：我觉得就是朱镕基之后高校大合并，从那时候开始就新自由主义改革，包括工人下岗潮的那个时候，就市场特别的凸显，然后那个时候就开始看重这个（论文），那论文这个其实也不是会一直存在的，它就是人为构建的标准。

受访者：但问题就是，你畅想另外一个标准取代论文

刘书博老师：好好上课教学，不是上层一直开会强调大学就是要教书育人嘛

受访者：但是教书育人的衡量标准其实就不太好去统一起来。

刘书博老师：对，它衡量标准不是那种工业化KPI的清晰的标准。

受访者：对

黄秋莉：但不是有那个师德师风奖吗？听说奖金还挺高，这个怎么评呀？我听他们闲聊的时候说，你可以去申那个呀，就可能是开玩笑说，那个老师说那能有多少钱？另外的老师说，那钱可多了，我想着应该挺多的吧。

吕梦娜：老师，您现在的工作主要分为几个大块儿

受访者：这个大块儿啊就是教学、科研，然后还有一些学院的乱七八糟的行政事务

吕梦娜：大概是个怎么分配的时间。

受访者：以前可能教学的时间会多一些，因为要备课，有一些新的课，所以花的时间可能多一些啊，然后现在可能教学准备的时间会少一些。如果按一周算的话，可能30%在教学上，然后70%在科研上，行政事情可能也就20%，就这个是阶段性的，就有一阵可能特别忙，然后有一阵子就不忙了。

吕梦娜：那您现在做研究的主要出发点是什么呢？

受访者： 出发点现在是比较功利的，就是说什么好发做什么

吕梦娜：为啥？因为一开始不是说还是想要探索一下、满足自己的一些好奇心嘛

受访者：但是问题是有压力嘛，评职称的压力呀

吕梦娜：您现在不是已经是副教授了嘛

受访者：还得评上教授

吕梦娜：这个对您很重要吗？

受访者：我觉得还是重要的吧。

吕梦娜：那如果评上了之后呢

受访者：就评上之后，可能很多人都会说我评上教授之后就要做一点有质量的文章，或者说按我的兴趣做一点东西。就是这个是很多人都在说的一句话

吕梦娜：那您认可嘛

受访者：认可。但是我不知道他们会不会这么做，我争取这么做

吕梦娜：您刚刚也提到就是其实研究的前半部分还是能够比较自由探索，到后面可能到投稿、发文章这种就会被约束一些。那您觉得这个投稿啊或者发文章会约束你的选题呀或者是研究吗

受访者：对，就是有的时候写完一篇文章，自己觉得这篇文章很好，或者是我觉得这个文章的贡献很大，但是呢从编辑的角度，他可能就觉得这个题目和期刊的导向不符合，或者说你这个题目和现在的这种政策呀或者热点不契合

吕梦娜：热点？

受访者：对

吕梦娜：所以现在期刊都是热点导向的嘛

受访者：中国期刊是这样的

吕梦娜：哦哦哦，那您觉得这个合理吗？

受访者：那肯定是不合理的，但是你也得理解期刊也是有期刊的原因的，因为咱们中国的期刊不是独立生存的，他也依托一个单位嘛，大部分好的期刊都是社科院的，社科院呢又是和中央的战略导向比较相近的，他也得听中央的指挥，对吧？所以你也得理解这个期刊，他这么做并不是说这帮编辑真的就觉得应该热点导向。

吕梦娜：所以这么说的话，现在做研究得先了解一下中央的倡导导向是什么。

刘书博老师：这个非常重要，我们申请课题也是。

受访者：所以说相对来说，如果是一个学校办的期刊可能稍微好一点，但是如果是社科院呀或者说那种机构，像《管理世界》是发改委这种机构办的，他就会导向很明确。

吕梦娜：那您现在发文章对期刊有什么偏好吗？比如说中文啊，英文啊之类的

受访者：这个偏好可能得来自于选题，就是如果说我找这个选题可能正好和热点比较契合，那肯定是想要发中文的。然后如果说不是热点问题，就是一个很普遍的一个现象的问题，那可能就干脆一开始写就写成英文。

黄秋莉：那我想问一下，您这个研究问题一般是怎么涌现出来的？

受访者： 有很多涌现的方式。就是说这个如果从公司金融角度上来说，最简单的涌现方式就是，比如说你出去开会，看一个人研究了一个什么变量，诶，那这个变量是不是可以和我这个一起做一做呢？结合一下。但是其实有时候这种方式了，就是我们上课的时候会说这种方式不好，对吧？但是这也不是说完全不可取，就是说这个因为你自己的这种研究方向是比较稳定、比较固定的，然后你看到一个新的变量，你觉得是可以结合的，那这背后肯定有它结合的这种理由在里面。然后在这个里头就看个人能力了，就有的人能挖出很多的东西来，有的人可能就是做一个简单的这种关系就结束了。所以就这个肯定是一个很快速的产生idea的一个方法，还有就是一些热点事件呗，就是线下的一些这种突发的一些事件呀，或者说一些这种大家都关注的事情，现在不是这种双减呀之类的。

吕梦娜： 嗯，那你也会到企业里面去看一下吗？

受访者：不看，哪有时间？

刘书博老师： 看起来我们的问题会从这种大家比较关心的事情中过来，那您觉得我们做的研究能够反哺到我们现实吗？

受访者： 我觉得有一些。肯定说指导现实这个东西谁都说不清楚，因为就是说你做出来的这个研究，因为实证这个东西说实话它不会有特别超前的想法，因为他都是用过去的数据去验证你的一个东西。如果你的这个idea是一个很超前的东西，他过去的数据根本就验证不了。所以他只能去证明一些以前的事情是对的，或者说大家这么做是对的，对吧？所以你不能说是对现实有指导性的意义，但是它可以验证就是反应现实，或者说告诉大家说这么做是合理的或者是不合理的。

黄秋莉：那您会觉得您做的这个研究，这种实践意义呀或者是理论意义呀，这个好写吗？

受访者： 就是你在写文章的时候，写是好些，但是问题是你写出来之后有没有人信。这个还是就是说你要挖出那个理论的贡献，我觉得相对来说是比较容易的，就是好好读文献一定能挖出来的，但是问题是你要把那个写的特别好，然后让大家觉得我这个真的是一个贡献，这个就很难了。

吕梦娜： 嗯，所以这个好像还是从技巧方面的，就是你多用功，然后就是看文献能出来的。

受访者： 就是一个是技巧啊。然后还有一个就是说你对这个理论的一个认识啊，然后还有就包括就是你刚开始写的时候，你可能对这篇文章可能产生理论贡献认识是比较少的，但是你通过交流，通过反复打磨，然后可能你就能够慢慢得把那个聚焦出来。

吕梦娜： 哦，了解。那刚刚您也提到就是实证研究可能整体上可能不会对这个理论不会有太大的预测那种嘛，就是这可能是这个方法本身的一个问题。那您现在做研究的话，会不会觉得有点没有什么太大的意义？

受访者： 也不是没意义，至少养活了我自己。

刘书博老师： 这就是现实意义。

受访者： 就是你说研究本身，也不能说实证研究它本身就没有意义，我们之前说到他可以验证现实嘛。但是如果你期望实证研究真的能产生太多的理论发展，我觉得就是可能就不太现实。

吕梦娜： 你有没有想过就是跳出实证然后去用别的方法做研究？

受访者： 等评上教授可以探索一下。

吕梦娜： 那您现在工作的这个意义感主要是来自于哪些地方？

受访者： 工作的意义方面，其实我觉得有很大一部分快乐其实从课堂上来的。如果有同学说他觉得任课老师讲的课讲的好，或者是觉得通过上课获得了东西，这个确实还是挺让我感到快乐，这种快乐我觉得是大于发文章的这种快乐。

吕梦娜： 对，我们也发现其实很多老师都很上心，就是即便现在的状况可能是重科研、轻教学，但是老师们的这种责任意识还是挺强的。

刘书博老师： 就反正我发了一篇学术性的文章发到同事群里，似乎大家也不关心文章发现了什么，然后发给同学们，大家更不关心，发给实践者，他们表示看不懂。

受访者： 就是给做实践的人看，他们就会说，“嗯，这是什么？有什么意义吗？这我们都知道。”

吕梦娜：哈哈，你笑啥？

黄秋莉：我是觉得说老师的这个经历很有意思。

刘书博老师：我就发出来之后谁都不管，然后我觉得好失落呀，后来我就

不再show off了。

受访者： 我就发了一篇文章出来之后就是感觉是失落。

刘书博老师： 对，发完了之后你就觉得，哎呀，好空虚，就跟大家考完试的那个感觉一样。

受访者： 对，就是这种感觉。

刘书博老师： 他没有这种持续的这种，就是社会性的承认，所以我就特别爱写公众号文章，还有一些人反馈留言，或者说觉得有帮助。

受访者： 我觉得知网是不是每篇文章底下也应该开一个留言区。

刘书博老师： 所以现在学术就变得很专业性。

受访者： 在里面自娱自乐。

刘书博老师： 嗯，所以我非常认同顾老师刚才说的那个价值感，它是教育教学和与人沟通。如果只是发文的话，似乎就是有那种失落感，当然要除了发钱的时候。

受访者： 然后之后你就会知道，啊，我们写这个文章的意义在于换成奖金，这个还是能够抚平心里的那种空虚的感觉的。

刘书博老师： 但是整个写的过程还是挺痛苦的，我反正觉得挺痛苦，尤其我做一些案例还是有一些故事性和现实性，你像做实证，那就是那个标准化的，不断数据。我之前做过一次啊，我之后就伤了好久。

受访者： 我觉得写实证论文可能最可以发挥创造性的就是引言部分。

刘书博老师： 讨论也可以升华。

受访者： 但实证这边，讨论基本上也比较格式化，就是把前面摘一摘。

黄秋莉： 郭老师会跟别人讨论起你的文章吗？像蓓蓓姐她竟然回去跟她老妈介绍她自己写的文章，然后还问她的妈妈你听懂了没有？

受访者： 我也跟我妈讲过，对，是要这样的，就是说你要让一个不懂的人能够听懂你的研究意义在什么地方，这才是挺重要的。

黄秋莉：那老师您会跟别人在非正式场合交流您的文章吗？

受访者： 我在家里跟我老公经常交流啊。

刘书博老师：你们俩都有专业的。

 受访者： 对呀，但是我们俩经常就吵起来了。这种学术探讨反正最后都是他洗碗收场。

吕梦娜： 都是一个研究领域的吗？

受访者： 不是，他做那个金融的。

吕梦娜： 哦，那除此之外呢？

受访者： 就是你说非专业人士的话，闲聊的时候基本不太会讲这个东西，讲了之后我估计没人再找我玩儿了。

那其实是有很多割裂，就是自己的研究其实跟现实还是有所割裂的，就是其实也跟别人讲过一两次，你就发现人家对这个东西不感兴趣嘛，然后你就不能老讲。

黄秋莉：那你会想起你以前做的研究，然后会觉得我怎么那么有才？会有这种欣赏自己写的东西的感觉吗？

受访者： 没有，就是会觉得，哎呀那时候实证做的太差了，居然还能发出来。现在实证做的比那时候好，为什么还发不出来啊？然后就感叹说现在这个竞争太激烈了，然后这个期刊导向太明显了。

刘书博老师： 那现在像发顶刊的那种实证方法就是特别精致，是吧？是叫精致的平庸。因为我英国导师他就在跟他同事聊的时候，他也会发现就像现在AMJ、AMR都是并没有什么真实的问题，所以他那次吃饭就抱怨。

受访者： 是，我也觉得现在研究有点太重实证，但是你话说回来，就是说现在其实大家实证做的也都很不错了。

刘书博老师： 就是竞争更加激烈？

受访者： 对。所以你说期刊拿什么去评价说一篇文章好或者不好，他在初审的时候就只能看主题了，就说主题跟我们符合不符合。

刘书博老师： 我们可以合作，考虑一下把论文做在祖国大地上的那种，然后对应中央的号召，理论自信、文化自信，然后我党的这种伟大光明形象类似的，因为中国共产党其实在做品牌宣传的时候特别厉害。

受访者： 我觉得中宣部应该有很多品牌大师，我那天看中宣部的就是不是有很多网络流行歌曲，就是那种特别红的那种网红歌，然后被中宣部改词，然后改成那种宣传性的词，我觉得这简直是太天才了。中宣部真的每个人都是营销大师。

吕梦娜： 之前有一次春晚的野狼disco之前有一年特别火的，后来也改编了。

刘书博老师： 对。现在其实期刊如果看到这种研究会，我觉得会有一些捷径或者是开绿灯。

受访者： 对，他们喜欢这种东西，就公司治理还喜欢党组织参与治理。

刘书博老师： 对，中国特色的。

受访者： 对对。

刘书博老师： 所以我们可以一块儿经常探讨一下金融、营销，像我这个话语分析、案例研究都挺合适的。

吕梦娜： 老师您刚刚说现在就是实证的研究做已经很不错了，主要体现在哪些方面呢？

受访者： 就是那一套规范动作大家都能完成，就是大家越来越规范了。比如说像是以前那个管理这边也不怎么看内生性嘛，但是现在管理也越来越重视内生性，然后内生性的处理方法那一套，然后一做一套，然后你眼前就是那么多表。就是像以前其实管理的文章我觉得很简单，就是可能一个就是基本回归调节，再加上一些中介什么的，可能三四张表就完了。但是现在你一篇文章没个十张表，你好意思投吗？嗯

吕梦娜： 对啊，不过好像我们OA还好一点儿。

受访者： 对，你们还好一些。

吕梦娜： 哦，那其实主要还是计量占太大篇幅。

受访者：对。你们还是主要用那个结构方程模型吗？

吕梦娜： 对。所以在这种情况下就开始比主题了，就可能是唯一一个可以比较的东西了。

受访者： 对，还有就是比你这个合作者里有没有大佬啊之类的。就至少初审，初审的时候你这些东西是可以比的。

吕梦娜： 嗯。这个主要针对中文去看吧？

刘书博老师： 我觉得国外他们也看，而且国外的其实是更加隐晦的权利关系。

受访者： 其实他们说国外如果你的这种合作者里头有一个是国外的学校的话，就会比较容易过那个初审。

刘书博老师： 哦，是的，像我那个导师他是做非洲的案例，他跟他非洲兄弟们几乎投一篇中一篇，每年出一篇，因为那个领域就是他们把持着。

受访者： 发论文小团体那种。

刘书博老师： 对对对，发review啊什么都是他们，相互的。所以我们看怎么结合非洲，中国支援非洲兄弟这个肯定可以的，那现在没什么数据。所以其实这个说起来我们学术有时候也是有背景的，权利关系啊什么的。你们还有问题吗？

黄秋莉： 就想问一下老师，您觉得目前的学术生态跟您父亲那个时候是不太一样的嘛。那您会觉得您对当下这种环境会有一些这种反思性的批判吗？

受访者： 就是反正怎么说呢？就是有人的地方就有江湖嘛。不可能说真的存在一个完全纯粹的一个环境，所以说学术圈其实和别的圈没有什么太大的区别。

刘书博老师： 嗯，嗯。

黄秋莉： 哎，我早上看到那个姚老师发的朋友圈嘛，就说疫情之下我们对未来的信心是整体上不太好。那您会对咱们未来学术的发展充满信心吗？

受访者： 嗯，我觉得也不存在什么信心吧，我觉得再差也不会比现在更差了。

黄秋莉： 感觉再差也不会比现在更差？我们已经到谷底了吗？这种状况会持续很久吗？

受访者： 应该还会持续一段时间。

黄秋莉： 你有看到会改变的迹象吗？

受访者： 改变的一种可能性首先就是说取消这种博士生必须发文章的这样一个比较强制性的东西。就是说如果博士生不用发文章就可以毕业的话，那博士生可能就是有的就志不在此。那我可能就不想着说要发文章了，这样的话了其实老师的压力可能也会小一些。因为有的老师他其实也不想指导博士生发文章，对吧？他也不需要文章，但是博士生必须要发文章，他还得想着怎么给博士生解决这个文章。

刘书博老师： 而且你们都在发，那期刊资源就更少了。

受访者： 所以这个可能是一个契机吧，就是说通过这个去改变，但是他只能去改变那种由博士生推着老师让老师帮发文章的那些，那些份额可能会降低。但是本来就卷起来的那些人，自发内卷的，那些人可能不会受到影响。

刘书博老师： 自发内卷，对，他就像一个苦行僧一样的那种。

受访者： 就是从中得到了乐趣之类的。或者说我就是为了自己啊，然后为了自己的好前程，那个今天不是最后一天嘛，好多人都在发那个年终总结。然后看好多人写发了两篇中文、两篇英国，还有一篇2A，然后申请了多少课题。我觉得看这种人朋友圈真是压力太大了。

刘书博老师： 谁呀？那么烦人。

受访者： 对呀，金融那边儿的人。

刘书博老师： 给他评论一下，你说你的梦真好哇。

黄秋莉： 竟然大家会这么外显自己。

受访者： 很多人，看起来他们挺有成就感。

刘书博老师： 我就把他屏蔽。

受访者： 他就是从这上面获得了成就，然后并且愿意分享出来，跟大家同乐。

吕梦娜： 就他一个人快乐了，难道不是应该把自己的缺点显现一下，他们却把自己的优点都展露无疑。

刘书博老师：所以他就是个老好学生的那种感觉，仿佛得到了小红花和奖状一样。

吕梦娜： 刚刚老师说到博士生就是可以不考核嘛，我可能陷入一个指标的诅咒，就是怎么去评价一个博士生呢？

受访者： 毕业博士论文啊。

刘书博老师： 对呀，哦，我们英国就这样。

黄秋莉： 其实我觉得老师是能够判断博士你的论文是不是达到了一定的标准，对不对？

受访者： 太能判断了，而且有的时候你会发现有的学生发了很多的好文章，但是博士论文依然写的一团糟啊。

吕梦娜： 对，所以那是不是能反映出来其实是不太相信老师的决定，博士的去留？

受访者： 对，其实是没有，我觉得发文章这个只是一个过程管理啊。

吕梦娜： 对，就过程管理嘛。那其实是不是相当于不是特别信任导师的能够把学生评估好才有这么多的过程？

受访者： 这个政策制定的初衷我也不太明白是什么意思，但是我觉得可能有一部分考虑是觉得想要老师和学生之间更多的互动。

刘书博老师： 还有就是让你们未来好找工作。

黄秋莉： 对，反正林老师每次都是这个意思，发两篇都不够的感觉。

受访者： 但是其实就是说博士生在找工作的时候有个误区。文章好的，但是未必你拿着这些成果能去找到一个好的单位，就不是学校那种单位，就是说去一个国企或者什么银行，人家觉得你这个没有用。就是高校工作才会觉得你这个有意义。我有一个那个博士生今年毕业啊，然后他发了两个A一个B，然后还有一个2A是接收了的。这个成果我觉得还可以了，然后拿出去找工作，去各个银行总行啊，什么券商啊，人家就觉得不行，然后你这没有工作经历，也不需要你天天在那儿写文章。

吕梦娜： 这就应该去高校工作。

受访者： 就是说这个成果好的博士生他可能就觉得太苦了。就这样做下去，太苦了，也不是很想继续往下做了，就是真的没后劲儿了。

刘书博老师： 这个揠苗助长嘛，把他的好奇心都耗尽。

受访者： 他没有发现做研究有什么乐趣，就只看到了做研究的这种苦的地方。如果能表现出对做研究的热衷，我估计也可以再考虑考虑。

刘书博老师： 那除非就是拿鞭子抽自己，然后年末了发一个朋友圈期待获得别人的赞。我觉得这样的话一点乐趣了，所以就挺异化的。

受访者： 就是现在写论文已经失去了写论文的最终的那种意义。

刘书博老师： 就像你父亲那个时候还是有的，自己自发的那种内在价值。对，我们现在非常外在价值，而且赤裸裸的外在价值、经济价值。

受访者： 就是我觉得是这样，就是说一个什么新的行业，就是刚进去的那些人，就是总是能在里面发现这种做事情真实的意义。但是后面进去的就是开始卷了。

刘书博老师： 就是塑料化。

受访者： 对，然后卷完了之后就已经失去了他最开始的那个初衷了。

刘书博老师： 然后他也即将灭亡，就行将就木了。

刘书博老师： 就刚才我们说这个博士，我就觉得特别的具有代表性，发的多了，他自己就对于这个专业就退出来了，他不要做学术。其实就退守了嘛，那之前让他写那么多的意义何在？这就不是培养了，这是在劝退。通过这写文章不是培养而是劝退。

黄秋莉： 是的，我室友她就是想早点儿走，再也不想写了，再也不想这么辛苦了。

受访者： 哎呀，所以说这个博士读完了之后，可能如果还能留下来的人就……

刘书博老师： 真的是变态。那我们就剩下了好多变态在给我们，就跟变态一块儿卷，我们也卷，那这个职业是真的退守了。这么一想真的好绝望啊。

黄秋莉： 那这么看感觉也不太会好了，感觉这个世界会好吗？

刘书博老师： 今天是2021年的最后一天，我们讨论出了一个令人绝望的方向。

 但刚才顾老师说的，我就觉得那个很对啊，刚开始的时候，就大家都是真实的价值追求，走着走着就变成塑料感的那种假的、人工的那种东西。

吕梦娜： 对呀，那最近不是说娱乐圈儿倒退十年怎么样，倒退20年怎么样，然后人家就底下评论就说倒退那可太好了，就是那时候可能是乐坛比较丰富多彩的一个时代。顾老师刚刚说就是有的地方就有江湖嘛，所以说学术有圈子，您的这个结论也好，观点也好，是从哪里得出来的呢？是从小的时候，在高校的熏陶中感受到，还是自己工作之后啊？小时候其实还是没有太大感觉，

受访者： 这个世界会好吗？就是你就看着而已，就是你只有旁观者嘛，嗯，但是你长大之后，就是你参与进来之后，你就会发现就是还是一个江湖。

吕梦娜： 对。我我之前读就是我硕博连读嘛，在本科毕业还没有什么感觉，就感觉大学是一个很纯洁的象牙塔，就是那种觉得很神圣那么一个地方。

但来了之后就发现包括写论文的过程中，就觉得一点儿一点儿都变得抑郁。但是好像遇到刘老师之后我又开心了，就解放出来/

刘书博老师： 我有点儿自我安慰的那种逗逼感，我不是特别卷，我有时候就自个儿去玩儿去。但我真的觉得现在有的人挺卷的，而且那些卷王又要产生影响，把自己的标准普及开那种。

受访者： 我很好奇你在采访窦超的时候，他怎么说的。

刘书博老师： 我们可以跟顾老师分享一下我们的发现。他想法其实很清晰认为这个就是一个工作，那生活方面我还是个正常人，但是这个东西就好像我要去麦当劳打工一样。

受访者： 那种就跟公司上班儿一样。

刘书博老师： 对，他就已经非常这个低标准了，他就把学术看作是类似工作、类似麦当劳的那种标准，然后他会比较积极的说要升级打怪。就他会想要特别迅速的完成这样的一个过程，我觉得是因为他把这个工作看的非常不堪，所以他想尽量压缩这个过程，想要赶快上岸。上岸之后他就可以不去玩这一套东西，所以他表现出来就是非常急迫的比学赶超，就一年发8、9篇的那种。

受访者： 我特别不喜欢上岸这个词。

刘书博老师： 他就是上岸、然后“老板”。嗯，对呀，我也不喜欢这种，就他会把那种学术当作一种工作，这种如果是没有追求一些独立的或者探索的，那直接到公司工作不就行了。但是他们就带着公司那一套进来，就把它变成公司的那个样子了。

受访者： 那他们为什么当时不去公司呢？

刘书博老师： 这一点他是怎么说的？哦，他老婆是在医院当医生啊，然后他这样时间上更自由。

黄秋莉： 嗯，还是觉得大学是个好的工作环境。

刘书博老师： 非常的自由，是的，就比较现实，非常现实主义。那有的老师会比较理想主义，就像那个林语堂把不同的国民性格分成四个维度，现实主义、理想主义，还有幽默感和敏感度。那其实我觉得有的人是比较像中国的这个传统的国民性格，就是他现实成分能够占一半以上，理想的只有10%以下，然后也不幽默，然后非常敏感，他是比较传统的。中国人的这种非常现实主义，现实的同时又很敏感，敏感的表现就是对于制度特别的敏感，能够捕捉到现实层面的要求，然后他快速的就达到那个标准。然后林语堂说法国就是很理想主义，很不现实，所以他们老是闹革命嘛，就为了理想，英国是大概比较均衡。所以我觉得可能就是不同人的看法也不一样。

受访者： 对，确实。

黄秋莉： 老师不喜欢上岸这个词是为什么呀？

受访者： 就感觉好像我们现在都没上啊，感觉不知道怎么说吧，包括什么像考研也说上岸，对吧？然后找工作也在上岸，但是其实对应的就是不在岸上的都是在学习的过程。

刘书博老师： 就是我们还没资格，就感觉我们还是在岸下面。

受访者： 觉得会把那种学习和深造或者说拔高自己的一个过程看得很痛苦。

刘书博老师： 就是传统的那种苦海，学海无涯苦作舟，然后什么都要挣扎。然后头悬梁锥刺骨，学习就变成非常痛苦的一个过程，要流血的这样一种感觉。

受访者： 而且就是感觉上岸了之后就可以立马躺平。

刘书博老师： 对对，我们会问他一个问题，就是你上岸之后要干嘛？他其实没想过，他没想那么远，他反正就是不断的升级打怪。

受访者： 嗯，那他可能会上岸之后还有更多的怪要打，不是说评完教授就完了。

刘书博老师： 继续的要上岸。

受访者： 对，人家还有各种学者各种帽子。

刘书博老师： 其实他就是跟院长比较像。

吕梦娜： 对啊，原来教授之后还有各种帽子。

刘书博老师： 所以有时候会说年轻人做领导不合适，如果是现实的年轻人，他会先把领导这个平台看作是自己上岸的一个推手。但是领导需要是成全别人，所以它会有一个倾向性，所以有时候现实主义对于组织的发展具有约束性。

受访者：尤其是像是一个中层领导，其实他是处于不上不下这个位置，他还是可以借这个去再上一层。所以这个时候其实就是看他的态度到底是怎么样的，就是格局怎么样，他在这个位置上是给自己揽资源呢？还是说真的是为了整个组织发展。

刘书博老师： 嗯，那现在就很清晰，尤其老领导退了，新领导刚一届吧。

受访者： 一两年了吧，两年多了，好像明年换届了。

刘书博老师： 哦。

黄秋莉： 顾老师，那就是现在管理学可能会面临一些批评嘛，其实老师自己可能也有很深的感触，比如说我们管理好像离实践太远了，那老师您对这个事情是怎么看待的？您会觉得这是一个问题吗？还是觉得我们管理学可能跟实践也应该有一定的距离？

受访者： 怎么说呢？就是说这个事情肯定是不太正常的，如果说研究真的和实践有很大距离的话，那研究它干嘛？

黄秋莉： 那现在是有距离的吗？

受访者： 有距离的啊，而且这个距离是时间走在前面，我们走在后面。这个距离造成的原因其实有很多很多的原因，方法层面的原因就是一些实证主义嘛，然后还有这种制度的原因在里头，还有就是说比如这个学校和企业之间也没有那么多的沟通。

其实有很多的原因，但是你说真的是要把这个实践和理论给捆在一起，或者说结合起来，这个其实也不是一朝一夕可以去改变的事情，那他和这个学科的这种特征也是有关系的。就它是一个实践性的学科，就是说他不一定真的是理论去引导实践，有的时候可能这种学科就是理论在实践后面去总结它。

黄秋莉： 那我们管理学呢？

受访者： 我觉得管理就是这样的。

黄秋莉： 学科需要总结管理经验。

受访者： 对，总结管理经验，但是你也不能就局限在总结。就还是得有一些这种突破。但是你怎么去找到这个突破其实也是挺难的。其实我觉得可以借鉴经济学，经济学它其实也是一个实践的学科，但是经济研究出来的东西确实可以去有一些这种建言献策的这个作用在里头，但是为什么管理就不行？对呀，就是这个里头原因到底是什么？

刘书博老师： 你怎么看？这我也不知道为什么。

受访者： 我觉得可能的一个原因就是，管理是一个分散化并且是特特质化的一个东西。就说不可能有一条理论是适用于所有的起源，但是对于国家来说，我可以去找很多的经济学家去研究政策，然后我在这些政策中去采纳它。但是对于企业，其实我们企业是不可能做这些事。

刘书博老师： 对，所以有些学科是宏观一些的，有些是更偏向于中观微观一些。

受访者： 对，所以有的时候我就是觉得学管理的真的是不太适合读博士，或者说这个学科不应该培养太多的博士。

刘书博老师： 对，MBA就行。我理解是像社会科学，包括管理学、经济学、社会学、心理学、人类学什么的应该有光谱啊，一个比较偏心理或文化的、人性共性的。那管理是偏文化影响会多一点，经济会偏人性影响会多一点，所以一个更朴实，一个更特殊，所以管理它是往这边走啊，如果我们像经济学一样总是在找普适性的，那可能对于实践的指导就没有像经济学对于实践那么强。对，我是这么理解。

受访者： 其实你说经济学的很多理论，你所谓朴实，但是它其实也是个体的，它就是针对美国那个体制提出自由主义，对吧？其实也是一个体的性的。

刘书博老师： 所以也会受到文化价值观的影响。但是美国又会说我们是去价值观判断的，没有道德什么的，去伦理道德，就它会认为人都是有一个基础假设，但这个假设就是把人看作是一个像原子的，就自然科学化。所以有时候我们还是需要针对中国自己的这个文化去研究啊会更好一些。前天评正高，然后有一个人就评上了。然后评委对他的评价都挺好的，他就是做中国养老问题就心理啊什么的，就很针对中国特殊的问题，然后做的一个项目。然后未来对于这样的研究会越来越多吧，就还是来自于现实的真的问题，然后围绕这个问题我们再进行研究，然后再发表一些文章。所以我觉得未来如果按照这样的一个方式去走会好一些，包括像未来在评正高，这么卷都是2A的文章那么多，那就要看内容，包括它产生的社会影响。

吕梦娜： 嗯，对于老师您来说您做的研究会落后于现实吧？

受访者： 可能就是落后于现实，对。

黄秋莉： 落后于现实，那对您来说会是一个困扰吗？您会有对现实产生影响的这种欲望吗？

受访者： 就是对研究本身来说也不是困扰，因为这确实是一个现状。第二个问题，其实慢慢你也能看清楚研究它本身的这种价值在什么地方，所以它可能没有我们想象的那么有用处，或者说那么有实践上的价值。但是对理论上可能是有贡献的，反正就是说研究这个事情吧，他不可能说特别的现实化，如果说现实化肯定就做个调研，可能你搞个咨询会更有意义一些。所以研究它肯定在一定程度上也是会剥离现实，就是去一个更纯粹的一个世界。这也不能叫失望，就说这个是现状吧。

黄秋莉： 我会想说您会对现实产生影响的这种迫切的愿望吗？

受访者： 现在可能也不太有了，就是因为你认清了这个现状之后就不会有非分之想了。

吕梦娜： 我明白了，那您对扎根中国的研究现在的倡导，你是怎么看待的？你觉得这样的研究应该是什么样子？

受访者：说实话，就是说管理学的学者应该都是做这样的研究，就是因为如果你脱离中国这个环境你再去做管理学的研究，其实是没有没有意义的。对，肯定必须得做这样的研究。但是确实有一部分人他可能是就是拿着这样的帽子，举着这样的旗，但是做的是一个跟这个不太相关的东西。

刘书博老师： 习近平号召我们把文章做在祖国大地，然后下面第二句就开始讲这个自己的事情。

受访者： 就是这个东西吧，应该是我们要做的事情。但是怎么说呢？就反正肯定是我想做的事情。但是也会有一些人会钻这个空子，利用这个事情去就是戴帽子。

吕梦娜： 对啊，那具体怎么做呢？

受访者： 真正的具体的中国的研究，那首先你得思考的是中国的问题啊。

刘书博老师： 刚才说的，研究真的问题，比如养老在边远地区对中国是很重要的问题。

受访者： 尤其是在中国，就是很重要的问题。

吕梦娜： 嗯，那这种问题怎么去发现？

受访者： 你就观察嘛，靠你对这个国家的这种了解和热爱。

吕梦娜： 那前面您提到就是说可能不太会去企业，那怎么去观察？

受访者： 这个就是市场上也有很多的这种现象，然后包括你从这个新闻里头，然后包括和人聊天儿，你也可以获得一些信息的。

吕梦娜： 对这种东西还是都有一些敏感度的。那就是评教授也是看论文吗？

受访者： 咱们主要是论文。

吕梦娜：您觉得这个合理吗？

刘书博老师： 那他只能看这个，而且我们其实看对象是一方面，另外是谁在看？就是投票的主体是那些专家评委嘛，他们内心是有不同的标准的啊。你比如说如果卷王那些就是看中了，然后岁数大一些的，传统一些的，他们就不是那么看，他们可能会看你的社会影响力，对吧？所以这个问题也不能说一个标准答案。

受访者： 反正就是多方面努力肯定是必须的。

刘书博老师： 对，那我们研究一些真问题合作看一看。 到时候可以建议教育部什么的，对吧？

受访者： 没有问题。

刘书博老师： 我暂时也没问题了，那今天下午也挺长时间了。

# 受访者18

黄秋莉： 我就想了解一下老师现在的一个工作状态，就是您可能有一个短期的目标嘛……

受访者： 如果没有那些其他的行政事务和相关指标的话，我觉得我的工作还挺好的，哈哈。

黄秋莉： 这个问题问得太直接了，要不还是从您本科到香港读博的这个学习经历吧。

刘书博老师： 为什么要选择读博，就是这个学术动机是什么？

受访者： 学术动机是吧？就是也没什么学术追求，一步步走嘛。就是之前是也去业界工作过，研究生的时候是做金融统计嘛，然后去了一家投行实习，就觉得很累，不想早起，加上我本科保研之后，我去了一家公司也是做了三个月的实习，在中国人寿，我也觉得每天上班很痛苦，高峰期的地铁……我就觉得做学术可能比较自由吧。

刘书博老师： 就是自由？

受访者： 对，不用早起，作息灵活，可以睡觉。

刘书博老师： 还有吗？没有觉得学术本身会有一些地方让你觉得有趣？

受访者： 说实话，我觉得在本科和研究生阶段受到的学术训练并没有很多。我觉得在博士阶段受到训练，学到让我觉得可能会对学术感兴趣的东西。我觉得相当于从博士阶段是一个新的开始，我当时会觉得很浪费时间，我觉得我本科和研究生阶段什么都没学到。

刘书博老师： 那博士阶段，能不能说具体一些，为什么这个阶段是让你觉得很有趣？

受访者： 因为那个时候才会有大量的专业文献的阅读，包括英文文献，然后可能还有一些学术水平比较高的老师。对，说实话，我在中财的时候当时没有碰到特别让我觉得收获特别大的一些很好的指导。

刘书博老师： 那博士阶段，你觉得香港那边老师的差异性怎么体现出来的？然后让你觉得他是优秀的？

受访者： 我觉得还是专业性吧。

刘书博老师： 专业性是怎么表现出来的呢？

受访者： 就是首先他肯定是在学术的前沿，包括他至少在……比如说那边老师吧一个人一间办公室，然后他（教师）每天都会在那个办公室，然后带学生以及上课的时候跟学生的交流都是比较多的，这种regular、比较有规范、比较有制度化的。

黄秋莉： 您跟您的导师是一个什么样的关系？就是平时是怎么互动的呢？

受访者： 我比较复杂，换了三个导师。三个导师可能正好三个不同的风格吧，我觉得都还有收获，就包括现在我跟导师都会有合作。

受访者： 第一个导师是富萍萍老师，就是也是刘军的老师。

刘书博老师： 就是人大那边的。

受访者： 对，就我觉得导师对博士的影响特别大，所以包括我现在去带学生，我觉得很多时候我都会去copy、社会学习嘛，我都会去按照我导师教育的方式去带，所以我觉得这个影响真的很大。

刘书博老师： 刚才说他们那边比较regular、比较制度化，但是制度化当中还是有很个性化的东西。

受访者：是是，对。

刘书博老师： 他们是一个比较综合或者平衡性的状态。

受访者： 我觉得还是一个平等性，就是说自由平等吧，就是有制度化的同时他不会去拘束你的个性，然后让你自由发展，包括你选课、去上课、去做这个东西还是很自由的。就我跟第一个导师break的原因之一就是因为我觉得他束缚了我的研究自由。

刘书博老师： 就是像中财培养出来的学生都会比较乖一些，那你到那边反而是想要突破这种束缚？

受访者： 我可能比较有研究想法，其实中财还是给了我不少东西的。研究生阶段我就开始做残疾相关的研究嘛，就读统计的时候我导师正好是跟残联有一些合作项目，那我去分析了全国所有的残疾人数据，然后我到了香港之后了我会继续跟这边的导师去申请残联的课题，然后也会想继续做这方面的研究。那第一个导师就相当于会觉得说我没有去做他的研究，就是跟着这边的老师研究他会不开心或者说会不让我做，那我这个时候我就会比较逆反，或者我就很不爽，然后我就跟他说我就想做这方面的东西。

刘书博老师： 他让你做他比较熟悉的那一方面。

受访者： 他会做一些中国人价值观，中国式管理的一些东西了，因为她是一个传统的、是大陆的老师过去的，因为在香港有香港local的、有老外，还有大把大陆的老师嘛。

刘书博老师： 所以富萍萍在那种环境下她能够很好的生存吗？我理解因为她是大陆式的那种研究，但香港可能是比较走那种前沿美式的那种研究。

受访者： 她是在那个纽约，就是在美国拿了博士学位，再回到这边，她也发过ASQ，也做过一些很好的研究，但她整体的研究风格我不是很喜欢，她偏关系型的那种，她原来是新华社的记者，89的时候出去的，然后就是可能跟个人经历有关，那我觉得她骨子里还是非常传统的，中国老式的。

刘书博老师： 就是那种封建式文化的家长一样？

受访者： 对，会有一些distance，比如很明显的就是，我们喊富萍萍就是要喊富老师，然后喊另外一个老师，你要喊kenny你不能喊老师，你就不能加任何老师的东西，这是两种完全不同的风格。但是香港的学生和外国的学生就可以喊她萍萍，我们就不能喊，就觉得挺怪的。

刘书博老师： 那第二个呢？

受访者： 第二个是特别有名的一个老师，就相当于只跟了他半年吧，半年后他生病了嘛，然后那半年时间没有人管我。然后我就被放逐了大半年吧，我就开始自己做自己的吧，做自己有兴趣的东西，后来在第三年换了一个新导师，他当时是管学生的副院长，现在是系主任，所以我前段时间才请他过来。

吕梦娜： 那边换导师很容易是吗？

受访者： 其实也不容易，也是需要很多程序，但是你可以双向选择，对比国内肯定是要容易。就学生不喜欢老师可以提换老师的申请，所以当时富老师还没退休，第三个导师是香港中文大学的校友嘛，就是以讲席教授的方式请回来，就给了他很多方便，然后他当时没有学生嘛，正好他来了，我就转到他那边去了。

刘书博老师： 那你一开始对残疾人感兴趣是出于什么？就感觉残疾人离我们好像挺远的。

受访者： 就没有呀，就是比如说我有这个家里人，我有亲戚是这个视力障碍，那我在中财的时候，我在大一的时候我就去做了志愿活动，就是有个盲人相关的一些视力障碍的，所以其实相当于我在本科阶段还是参与了一些这样的活动的。

刘书博老师： 所以你会觉得这个事情本身是有价值的？

受访者： 对呀，还是有认同的。

刘书博老师： 那现在你做这个研究，从研究这个角度来看，怎么样能帮助到这些残障人士？

受访者： 因为我觉得我有时候是更加喜欢、更加偏向于就是基于现实的嘛，比较接地气的、就是扎根的，然后我会跟一些NGO合作，比如说我自己做博士论文的时候也是，我从香港回到了北京大概待了一两个月吧，然后去找别人给我推荐，就是我有这个残障的朋友，然后了就加入了他们的社群，长期在他们的群里面，有各种不同的社群活动，跟他们线下见面，跟他们一起吃饭，然后去访谈他们的一些领袖，就这个社群的一些意见领袖，然后就我现在也会跟NGO一起合作一些项目，就是他们会提出一些问题。那我们在跟他们合作的过程中可能觉得能够直接联系到现在的整个残障管理领域的或者是这些他们相关的领域，比如就业、财政和公益的一些问题了。

那我觉得就大概是通过这样的渠道，现在我想探索一些新的嘛，之前去那个社会企业创新论坛什么的，那个就是去讲如何做一个高效的学习，怎么样才能去贡献到我们这个社会创新、怎么样贡献到这个公益和平等的领域。就除了这个，我们作为学者吧，更加重要的一个肯定就是所谓的把握现象，就把现在表面上的这个故事抽象化成理论嘛，来更好的去解释去传播嘛，就这样从这个特殊到一般的过程。

那比如说我们写这个案例、做这个咨询研究也是很重要的过程嘛，所以比如说我现在做一些残障企业的就业企业案例的一些东西。那我想写案例，然后也可以去传播，第一帮企业宣传，第二帮大众更多的知道这个有这样的就业的人群，还有提高大众对企业的接受度和认可度嘛，然后第三个可能也是让更多人知道可以有这样的创业或者招聘的方式，大概就是在做类似活动吧。

就从我们企业社会责任角度来说，或者从我们高校研究者来讲，然后还有NGO，那还有一些政府部门，比如说残联，或者其他一些东西。比如说跟残联的人有一些沟通，因为他们毕竟是在推这个事情的一个很重要的主体，然后还有就是企业，包括有福利企业、还有一些真正的盈利企业、社会企业，他们就是去招一些残疾人，还有一些包括国企、外资嘛，世界500强他们作为专门有一个叫做diversity的部门吧，就多元化和包容就业的一个部门，所以也会跟他们有一些沟通，还有咨询公司什么的，反正就是基本上我觉得我现在已经做的还比较多。

刘书博老师： 那我看起来，像主流的像林嵩做的那个创新创业，或者是这个公司治理，或者是财务金融系，他们这种是比较显学的，就大家研究了之后可以知道怎么样赚更多钱、提高组织效率。但是你研究这个是这个方面的吗？

受访者： 因为我觉得在公司好像不是吧，我们现在所做的社会企业或者是做一种新型的企业，我们在算所谓的经济价值之外，我们还看社会价值，那整个企业创造的是整体价值，应该是经济价值和社会价值。所以除了他们的一些所谓的经济报酬、经济收入之外，我们还看对这个community对这个society的影响，所以我们现在来看就会看出我们是对全人类，对这个社会，对这个世界有贡献。

刘书博老师： 所以你这块儿应该是在商业伦理这块。

受访者： 我觉得还是有CSR。

刘书博老师： 其实说白了中国这个阶段我们做CSR更多还是没有主动性，它更多是一种应付，或者说还在以生存发展为主，那你在研究的时候会不会就觉得自己研究这一块儿它不是特别的主流？可能在发达国家他们会比较重视这个公平正义

受访者： 是，就比如说现在我自己做这个领域吧算是跟CSR有交叉吧。但我可能更多的是跟员工相关的研究嘛，就包括多元化就业，这个员工的福利，员工的这块CSR。但现在我感觉从我自己做残障这一块特别小的领域来说，就相当于国内商学院应该没有什么人在做。

然后还有就是我通过我写自科本子的经历，我第一年我申了一个创新创业相关的课题，我就写了残疾人创业，然后了有评委说做的很好、也有基础，就给了给予资助的意见；还有一个直接写这是一个冷门的方向，就直接建议不予资助，我就觉得他就已经给我定性了，这个领域是一个冷门。

刘书博老师： 就从题目来定性你这个研究。

受访者： 所以你看，评选自科的人算是这个专业方向的专家吧，或者是一些有一定指导性的、代表性的人，他们的观点就这样。所以我之后就不再申那个课题了。

刘书博老师： 你之后是怎么做的？

受访者： 就从人力资源管理申嘛。

刘书博老师： 我刚刚听你说，就是你做这个是比较社会互动参与式，那我们现在知道像发顶刊它必须是那种统计学的模型式的，就是说它其实是在做一种类似于经济学物理学的那种范式，所以这个是不是有一个不同或者是差异？

受访者： 我觉得不矛盾的，就说你自己去看怎么样去平衡。因为首先我觉得在解决问题的时候，你可能一方面是满足他们的需求，那可能是一个双赢的过程吧，就比如说我也有时候会找他们帮忙给我收数据，那可能我觉得这是两条腿走路吧，两个目的，那最后可以一起做一件事情。

刘书博老师：ok，就你在做这个事情过程中同时找到两个目的？

受访者： 第一个就是能够去满足他们那边的要求，就比如说获取一些能够向社会公众传播的知识，然后写一些比较科普性的或者是那种向社会大众传播的报告，还有一部分是我们做学术研究的需要，比如说我们需要去收集数据，需要建模什么的。

但其实有很多时候，你说的有道理，可能是有一些矛盾，比如说我们在建模之前，他们已经有基于社会实验的这个经验，比如说向残联或者向更高一级的政府机关去写什么样的建议或者什么样的政策建议报告的时候，他们知道该写什么了，那我这边的数据可能能够再给他多一点支撑这样子。

刘书博老师： ok，那有没有觉得像顶刊上的关于残障领域的研究，它有没有直接的或者是真实的价值？特别是对于这个实践者来说。

受访者： 以AMJ最近发的两篇文章为例吧，就是今年刚接收的一篇嘛，就讲立陶宛超市雇佣了残疾人，然后会给企业带来reputation，帮助他们有更好的经营，大致是这样吧。

刘书博老师： ok，那听起来就很……

受访者： 就是非常的common sense，就是你不做这个研究也能知道。

刘书博老师： 就你的回答来说，其实我理解就是似乎并没有看到非常significance的一面，更多地你会觉得参与这种实践互动或者直接的政策建议式的研究会对他们来说更有帮助。

受访者： 对，这样更能改变大家的现状呀，你学术文章写出来谁看呀？

刘书博老师： ok，所以咱们是有共识，就是学术可能还是有点自说自话的。

受访者： 对，就是学术圈的game，我就觉得其实是没什么用的。

刘书博老师： 那你在香港那边读书，你有没有了解到其他学科？他们是不是跟管理学很像，学术也是自说自话那种？比如说那社会学，是不是他们的研究和实践就会近一点？

受访者： 对呀，比如说社会学，什么社会工作呀，还有其他的他们也会去做，去NGO或者去做田野，甚至他们这些会去做评估，他们就是非常的沉浸于他所研究的这个对象和研究的环境中，我觉得我还蛮欣赏的吧，或者蛮羡慕他们那个生态的，但是他们也会有自己别的圈子，但我感觉不会像管理学的圈子这么的……我也不是特别了解，就山头林立或者是这么的……比如说，内地的管理界我不是很熟，因为我在香港上学……

刘书博老师： 香港怎么样，就先说香港。

受访者： 香港没有这么……就是那个什么……因为大家就是特别执着的盯着顶刊，可能就是太那个导向了。因为你看很多老师他也不参加社会实践，就是每天都在办公室嘛。

刘书博老师：他就有点像实验室的那种自然科学的那种感觉。

受访者： 对，对。

刘书博老师： 那香港有没有讨论说，这些老师应该走出到管理世界去了解真实的需求，他们有没有这种呼声也好或者反思也好？

受访者： 我觉得香港主要是那个tenure-track，就是那个压力太大了。因为他的考核里面只有发文章，就只有发文章，可能会有加一点对这个学校承担的行政工作，或者社会服务方面也会做一个评估，但我觉得最重要的还是看文章，你有文章就能留下来。

刘书博老师： 假设你留在香港做大学老师，你会觉得你就是只发文章，还是说你想更加接近实践，就是你刚才说的你要帮助解决那些现实的问题嘛。

受访者： 我觉得这可能是两种选择，因为有的时候……确实可能在不同的情况下选择会不同，比如在香港，因为我对香港认可没那么强，然后我也不是很喜欢香港人。

刘书博老师： 好像你跟姜峰不太一样。

受访者： 就我在香港的时候我也没有接触什么香港人，我会有接触香港的人，但那边的社会企业我就不会有很大的动力去了解他们、去探访他们、去花时间接触他们，因为我觉得我没有很好地社会融入进去，我感觉我不算自己人，然后香港的人的社会行为也没有这么强，还是比较功利主义的，其实我现在都怀疑香港的什么社会企业的一些东西。但是后来回大陆之后了，我回北京之后，我觉得因为大家比较熟吧，有一些感情方面所谓的亲近感。

我会觉得commitment和engagement很重要，就是你社群研究的这个环境和社群的这个土壤很重要，那我因为在那边没有这么强的所谓的社群支持和commitment，那我觉得我的最优选择就是只做好自己的研究就好了，就过这种专注研究、待在办公室就好，我觉得这种生活也挺好的。回到北京，因为我也没有自己的办公室，我也没办法做好自己的研究，我就只能往外跑，我就只能就是说把意义放在别处。所以我觉得这是环境和人共同作用的结果，所以没有绝对的答案，就在不同地方可能就会做不同的选择，就是这样。

刘书博老师： 那咱们再说说你对于中国大陆管理学的一些认识和看法吧，或者是一些总结提炼，就我们之前也总在一块儿聊嘛，从自科申请来看，能感觉到那种江湖的存在，对吧？其实有时候你会发现，诶，内蒙古工业大学的老师拿到了一个很重要的课题，最近XX竟然拿了俩社科，因为还有另外一个老师，也是他的人嘛。然后我就觉得，我认识他们，然后他们文章什么的我也看，（我就会觉得）他们凭什么，但是你再看他那个圈子，你就会明白，原来是这样，他们一系列的人就是等着在排队了，所以香港会不会这样？

受访者： 我觉得，香港所谓最重要的是那个……（类似中国课题基金的项目），这个是非常global review，因为他们是写英文的嘛，他会送给这个专业中最专业的人评，所以竞争是很激烈，但是评的时候是匿名的嘛。

刘书博老师： 咱们是单向匿名的，对吧？

受访者： 我觉得应该评审人他们肯定是知道的。我看过自科的项目本就是凭题目就能知道是谁在申请，所以这是有问题的。所以香港那边他们评论真的是靠自己的本子写的好，然后就能中，我觉得那边可能会更公平一些，然后确实也没有这么多圈子的概念。

因为我也没有听说过相关的事儿，另外，我觉得我认识的，比如说我的同学在香港一所大学就留下来了，他申请的课题也能中，那韩国的老师也能中，因为他们那边，可能主要是本土培养的博士留在那的也少，所以就不会说有这种所谓的圈子近亲繁殖，去香港的老师大都是从北美或者从别的地方毕业的，然后其实也diversity的嘛，有韩国人、英国人，就是还有白人老外就还挺多的。我就觉得可能还是跟人员组成，还有这个评审机制有关系，所以中国大陆和香港那边还是不同的特征的，他们那边圈子文化没那么明显。

刘书博老师：ok，那你觉得在咱们商学院，首先办公室这个咱们都很不满。

受访者： 对，就是没有尊严，所谓的工作平台和工作空间，这是一个很重要的尊严。

刘书博老师： 就是感觉好像领导者会觉得，你们反正也不来嘛，那我就不给你，给你也是资源浪费嘛。

受访者： 就是你来了还会问说，你怎么来了？你有课吗？我没课就不可以来了吗？这样其实特别阻碍博士的培养，阻碍博士跟老师之间的交流。

刘书博老师： 所以似乎我们学院也没有一个好的生态，我会觉得象牙塔就是一种大家时常会一起交流的这种氛围，但是我们学院的这种氛围似乎就有点参差，也有老师是非常愿意交流的，但是整体制度层面好像不支持。

受访者： 是呀。

刘书博老师： 那还有啥？你觉得除了办公室以外，还有啥你觉得比较特别的，尤其是相比于香港那边来说。

受访者： 就是对处在学术生涯上升期的年轻老师的保护呀，现在就像是故意欺负年轻人一样，就给你安排很多活，按理说这个东西都是在国外或在其他地方都应该是已经评上教职或者教授，他们去承担更多的行政职务。然后我们这边恰恰相反，我们是正好你需要写文章的时候给你安排很多活，而且不认可或者不感激你的这个工作，觉得是理所应当，会觉得就无所谓的那种样子。

刘书博老师： 那就很工具化，就把你仅仅看作是工具。

受访者： 我就觉得这边就是不太尊重人，这个现象还是挺那什么的，包括不尊重老师，我觉得现在领导也不尊重学生，尤其对博士生感觉很不好。

吕梦娜： 是的，说的我都要哭了，哈哈。

刘书博老师： 所以我们还是能够看清问题，有些人他就不觉得是有问题。

受访者： 就商学院里面缺少这种所谓的人文氛围，就没有去把人摆在最重要的位置，没有去treasure我们，这个community最重要的是people吧，就博士生很重要，那我们老师也很重要，然后他也没有去开展一些真正能够增强我们互动质量的活动，很多活动都很形式化。

刘书博老师：对，我们应该都有同感，然后这个硬件它也不支持，然后有一些制度呢其实也不是特别以人为本。那制度的话，我不知道你们博士生怎么感觉。反正就是他这个东西不合理，然后他只是在外部借鉴，然后拼接组合成了一块儿东西，然后一股脑地给你，然后出了问题呢，他似乎也没有想要去改变这个事情，所以就有一些我们现在学习西方的标准，但是学完之后长出来的东西似乎没有他们那边同样有效。

受访者：那个咱们学院对博士有什么支持政策吗？

黄秋莉：他所谓的支持政策可能就是我们学校学院举办的一些卓越论坛，然后包括老师的office hour也算吧，就是这种也算

吕梦娜：但是其实这些活动像您刚刚提到的一样，就是比较流于形式，很少那种真正去讨论，像上次Kenny给我们的讲座我觉得这已经是首屈一指的了，他能让我们去提问、去探讨，但现在很多论坛都是主讲人讲完问我们有没有问题，大家默不作声，就当没有问题，然后就解散。

受访者：那个不是学院举办的，那个相当于是我个人无偿奉献的时间好吧

吕梦娜：哦哦哦~就是感觉那个已经算是在我目前接触到的讲座里面，比较高水准的了

受访者：而且他没有所谓的就是那种常规化的设置，我发现每次都是比如说因为教师正好有这个资源才设置，而不会想着比如说，我们请Kenny每年都来设置这个课、做个培训，这个有可能需要我们由下而上的发起。那其实对我本人而言，其实我做了一次这个讲座，我已经累了，我没有这么大的动力说老去干这个事情，而且我觉得我从这个事情上没有任何人来对我说过一句感谢，就觉得是应该的，而且我本身就是一个利他动机，做这个事情对我一点好处都没有，所以我有时候我不是encourage做这件事，而是discourage做这个事情

刘书博老师： 对，就像我做那个MBA中心的工作，做的多了反而他们会觉得你产生了一定的威胁，对于他的一种利益格局来说，所以其实我觉得我们这个商学院有时候理性的表达要弱于政治性的表达，他会觉得你能力都太凸显了，那可能未来会影响他的……

受访者：是不是对有能力的人反而就不会……？

刘书博老师： 对对对对，所以一个人越是有想法有能力，有时候领导就会觉得难以驾驭，或者说会产生对于自己安全感的影响，所以领导会说那我最好还是找听话的，完全按照规则

受访者： 我觉得他们是不是缺少战略性的思路啊，因为大部分组织可能都是不确定性规避的，它是通过控制人去减少不确定性，而不是说去增加正规的制度或者是做一些制度上的改进，制度优化、组织优化来去减少这种对不确定性抗拒的能力，只是减少人带给我的不确定性。

刘书博老师：还是一种权术的，对于人的控制。其实说白了，跟前面说的，我们把人工具化是一脉相承的

受访者：是是是

刘书博老师：他就把人看作工具，所以他就要控制人。所以就是一种权谋术，他不是说我要制定一个以人为中心的制度

受访者：所以就是一个组织还有组织领导的人文理念和人文思路很重要，就整个组织文化就是你说的这个导向了。

刘书博老师：对对，所以这个时候我们就看到这背后是缺乏一种人文的素养，其实也是在标准层面学的很快，但是理念层面没有学，我不知道为什么，可能学院领导就是觉得这个事儿没必要

受访者：就比如说你学了tenure-track这个事儿，但没有学习这背后的支持。

刘书博老师： 对对对，它就变成一种，我用这个东西让自己利益最大化，然后压榨别人，这个生态就被破坏了，所以现在那个中山大学就招不着人了。

黄秋莉：老师的长期规划是什么样子的。就可能对自己的学者身份，就是有一个未来的畅想，您有什么榜样或者是……

受访者：计划就是还是好好做研究呗，但是好好做研究的过程中需要去排除学院的一些干扰。行政的干扰，就是一些杂事儿吧，我觉得可能还需要更专注一些，我觉得可能现在就是很容易让人不专注。因为我自己是比较喜欢做一个简单纯粹能够去专心做一个事情，我觉得我也不是能够去同时做很多事情的人，要承认人的能力是有限的，那你选择就很重要呀。那你可能就是要做一些取舍，但有时候我们个人做的取舍还不行，外部还有些东西必须强加给你的

黄秋莉：但是我看您好像就做了很多的事情，您去西藏支教了，然后在党支部也贡献了很多，然后现在还要出去访学，就感觉是一个很变动的一个状况

受访者：那是因为我之前都是为了去访学做的trade-off

黄秋莉：是吗？为什么是这么个意思？

受访者：因为我当时觉得得有一年时间要出去了，那我觉得我得对学院做一些弥补吧，我就去西藏教书了，西藏教书那两个月基本上不能做科研

黄秋莉：对呀，就是，其实是对自己有很大的影响的

受访者：但我觉得我是一个openness experience比较高的人，我觉得对西藏还挺向往的，我也想去体验一下那样的生活，然后我在我人生的规划中，你问的这个学者规划就在人生规划里面嘛，我觉得可能就是学术并不是我生活的全部嘛

黄秋莉：那就是想要经历更多的东西嘛

受访者：对呀，对呀，那作为一个完整的人来说，我觉得工作不是我生活的全部，还有其他的生活，那我工作能做到让我自己觉得ok满意就可以了，那我也不能说用要工作的一些东西来破坏我对生活的美的享受和发现，所以我觉得多经历些东西事情对我还是挺重要的，所以基本上我每年我会给自己定一些我希望我自己回忆过去的时候每年都会有一个里程碑式的事件，当然就今年的人生经历来说，我觉得今年去了西藏也算我的人生的一个milestone，也算我一个很好的经历、回忆了。对，所以明年再去访学我也觉得是很好的回忆

刘书博老师：所以你这种对于人生的看法是来自于自己还是受到其他比如说Kenny的影响，就是你怎么形成这种观点的

受访者： 这个好复杂

刘书博老师：Kenny是这样的人嘛，感觉他就是很扎下来做···

受访者：对，Kenny很professional，他是有点很职业

刘书博老师：就有点工作狂，能这样说吗？

受访者：可能是他有一些自己的原则吧

刘书博老师：我听起来就觉得他就是，而且我看到香港中文的那个日本人，他就是做研究做到把自己腰椎都做手术了

受访者：我们那个时候有很多博士生就是因为天天在办公室从早到晚，然后就是腰都有问题

刘书博老师：他就拄着双拐给我们上课

受访者：那个周围是竞争性很强的一个环境，所以我就觉得当时的博士生比这里任何的老师都要认真勤奋，我都是属于不勤奋的，因为我老不上办公室

刘书博老师：所以我们会以为你应该是那种非常工作狂的，但是你反而是有一些生活上的这种情趣式的追求

受访者：我觉得可能会跟周围的人有关系，受到家庭还有成长环境和人的影响。那比如说周围的人就说你不应该这么工作狂。还有是看到的事情多了，看到世界多了之后，我觉得就有一些事情就很没有意义，就可能会跳脱出来，就我们会有一个所谓第三者的视角，或者需要时不时的比如说不要想自己，就是你可能站在更高的一个位置，比如说看下来，把自己看成一个别人，看自己走过的这几年，或者你之前的人生到底是在往怎么样的一个方向走，最后想得到什么东西。我觉得可能涉及到一个终极的人生价值观的问题，我是觉得可能我们的研究、研究生涯的安排、长期的计划都是由我们的人生观、世界观、价值观的引导的。所以你说的这个问题就是，我的世界观、价值观可能是一个长期形成的吧，受到家庭还有教育的影响，还有周围的社会环境的影响

黄秋莉：就讲到周围的社会环境，我感觉我们中财好像竞争也挺激烈的，听说XX老师去年发了八九篇吧，是这个样子的，那您面对这种环境的话，给你的感受是什么样子的？

吕梦娜：有没有同辈压力？

受访者：因为我不认可这种……就是可能评价标准不一样，而且没有必要跟别人比吧，我就是自己比较，因为我觉得一旦人有自己的一些对世界、对自己的所谓比较坚定的看法之后，每个人都有自己的生活，就按照自己的plan、自己的目标走就好了，别人做什么跟你有什么关系呢？

黄秋莉： 您不是也应该面临这种评副教授的压力嘛，您觉得您对评副教授的可控力度大吗？

受访者：还行吧

吕梦娜：老师您现在是已经拿到tenure了嘛。

受访者：没有啊，2020年评上副教授，还需要再三年，我是3+3，第六年才参加考核，是有一些压力，所以我才明年去写文章去了

黄秋莉：是在国内写不好嘛哈哈

吕梦娜：行政压力太大了

受访者：就我走不了还得继续做党支部的事儿。

吕梦娜：老师您就一直是在学术这里。那除了实习之外有考虑过去工作吗？

受访者： 一直在考虑，

吕梦娜： 一直在考虑？现在也在考虑吗？

受访者：现在这个成本太高了，加上工作当时我也会有纠结，但最后还是我自己的初心，比如说工作太累了，对我的健康损害程度太高了

吕梦娜：所以说还是得关注自己的身体健康、心理健康。

受访者：对对对，就说实话，我其实不太喜欢那种非常高竞争的环境

吕梦娜：那香港压力那么大，你是怎么规避的？

受访者：躺平呀

吕梦娜：这个怎么能够做到？其实我前两年在硕士的时候嘛，我就觉得没办法视而不见，就感觉处处都是压力

受访者：你在读硕士期间的竞争压力很大嘛

吕梦娜：很大很大呀，就是这种环境令人窒息

受访者：有这么夸张啊，你们现在内卷的好厉害

吕梦娜：所以现在我也不卷了，我也躺平了

受访者：我觉得就是高竞争性的环境对人是有损害的

吕梦娜：对，是有损害的，所以当我跳出这个环境之后，我反而觉得人生还是很美好的，就不要留在这里卷

受访者：就高竞争的环境，有时候会让你偏离你自己个人的目标。就因为太高度竞争了，你就会更加关注你同辈的人在干什么，大家可能会形成一些非理性的标准

吕梦娜：然后我看您近几年发的文章，好像基本上就是这三年期间都是以英文为主，这个是因为啥呀？

受访者：因为发不了中文的呗

吕梦娜：中文比较难嘛

受访者：因为在那边受到的教育都是从英文文献开始学，然后那边课全都是英文的，写也是英文的，然后参加英文会议什么的，基本上跟学术相关的英文都会比较熟悉，但写中文的话反而要把英文翻译成中文的，我有的时候都不知道中文词该怎么说，所以得有一个适应化的过程

吕梦娜：所以说就学术论文这一块儿，您对语言的驾驭其实英语要比中文好。

受访者：对，我感觉我都不怎么会写中文的学术论文，但（写论文的）套路还是一致的，只是说语言的偏好。还有就是中文的发表我感觉还不如英文的容易发，因为中文的发表也讲圈子嘛

吕梦娜：对对对，英文就好一些。但之前听说就是那些比较类似于顶刊的文章哈，好像也得需要大牛来带一会儿，那您有这样的资源吗？

受访者：有吧。

吕梦娜：是导师这块儿还是同学、同事或者其他的老师

受访者：导师加上自己领域内的大牛，自己认识的，开会认识的

吕梦娜：怎么认识的

受访者：就聊，因为每年我都去pre paper，然后就他们也可能会认识我，然后我有idea我就想跟那人合作，然后就跟他去说，写邮件

吕梦娜：感觉听起来容易，但是好像做起来还是……

受访者：对，可能他需要认可你的，尤其是你需要有一些东西让他觉得他的投入和时间是有价值的，人家还是比较从business的角度来考虑的吧

吕梦娜：所以基本渠道就是通过参加会议然后宣读自己的文章

受访者：然后其实还有一些介绍啦，比如说你们共同的connections，你有共同认识的人，然后做一些介绍，这样子

吕梦娜：所以这么听起来好像是，对您来说，无论说是境外的还是国外的一些学术资源要比大陆的更丰富一些

受访者：对，对我个人来说是的。对你们来说可能（不一样），就是每个人情况不一样吧，可能在大陆读博士的找到国外的大牛也很多，他们也有出去交换什么的，就看导师，可能是导师多大程度上愿意去分享他的资源给你，或者其他人愿意去帮助你。然后还有你的行为和东西能不能得到别人认可。

吕梦娜： 对，那还有没有个原因是因为，在残障人士这个领域哈，好像国内不是特别认可，像您说自科的基金评委认为比较冷门，所以在国内发表可能会……

刘书博老师：如果是社会工作就很认可，但是在管理学似乎就是边缘化。

受访者：对，是，社保嘛，法学比较多的就是人权，但在国外还挺火的

刘书博老师：国外这方面不是那么边缘，是吧？

受访者：现在是主流价值观，而且从女性的平权运动已经到残障平权运动了

刘书博老师：所以你看就中国其实我们是说关怀人民、人民至上，但是在管理学这一块儿，咱们就变成了一种资本主义反动派的那种感觉

受访者： 我就感觉国外对people相关的，就是你会发现发表的残障这篇文章感觉质量并没那么高，但是人家主题非常正确，然后就很重要，他们会觉得这个主题很重要，所以其实国外很多期刊有专门的残疾专刊、残障文章专刊，没见到国内有吧？

刘书博老师：没有，就我们管理学还是非常坚定地站在资本家的那个立场

受访者：对，还有一些女老师就会来给我建议说，你做这个领域就比如说大家不会这么认可你的，或者说觉得这么冷门、这么奇怪，然后还说做leadership呀更popular更热门，所以我就很……

刘书博老师：你看我们这个热门就是什么数字经济呀、平台战略领导力呀，创业呀

受访者：有些人就是什么热门做什么，但我觉得有些东西是值得去很久去坚持或者去专注的，那它就是有价值的东西嘛。那我们作为一个研究者，我们必须有自己所谓的研究者的identity，就你是研究什么的研究者，你到底研究兴趣或者研究领域是什么？你得有自己的研究领域。但有些人就是什么热门做什么，感觉什么都做，那他没有自己所谓的研究领域。

刘书博老师：是的，所以你是在追求这个本质价值，而不是外在工具价值。所以这个时候我们工具价值的主题又呈现出来了，对人就是工具性，对于研究依然是工具。

受访者：是的，是非常一以贯之的，所以价值观还是有道理。

吕梦娜：老师刚刚提到说“意义在别处”哈，就是您觉得是做研究也好，还是做老师也好，意义在哪里？在别处是指哪个别处？

受访者： 我觉得就是人和人之间的关系很重要，那我比较珍视就是我们人和人之间的爱嘛，就是这种感情，我觉得就是这个吧。还有可能就是跟自己的关系、跟家人的关系。因为你研究本身也是所谓的那种关系，比如说你要去处理好这个群体还有之间的关系，你跟世界的关系、你跟社会的关系。

吕梦娜：那研究本身会给您带来什么嘛

受访者：研究本身提升我自己跟我自己的关系呀，就是带来成就感，然后还有改善这个群体跟其他群体的关系、跟社会的关系，提高他们的welfare之类的。

吕梦娜：我刚刚听到您提到“identity”，包括您网页介绍上也有写到您的研究也跟身份认同相关。您会对自己有什么定位吗

受访者：我就研究多元化呀，就diversity research。

吕梦娜：因为现在的话，比如说就是对于老师，特别是刚刚进高校的老师都可能自称为“青椒”，有点儿自嘲的感觉嘛，还有什么“科研民工”这种的，就是您自己之前有想过吗？特别是前三年

受访者：我觉得做老师挺幸福的

吕梦娜：挺幸福的，为什么呀，体现在哪里。

受访者：我也在网上看到很多，说“青椒”很苦之类的

吕梦娜：对，您觉得前三年过得还可以是嘛

受访者：还行吧，除了学术上没什么进展。

吕梦娜：不能这么说呀，看您近四年发了很多文章，就这叫没进展嘛，是我们的理解不一样嘛哈哈

受访者：没有达到我自己想发的东西，我自己真正想做的、想发的都没写，都是在跟别人合作。所以你说前三年，我觉得做教师这个职业我还是喜欢的，然后我还觉得还是有给我带来幸福感的，对你说的“青椒”很痛苦什么的，我觉得没有。

吕梦娜：具体是哪些（幸福感）？

受访者：就自由呀，这很重要，可以做自己想做的事情

吕梦娜：但是你不也说行政方面给你很多工作嘛

受访者：对呀，这不矛盾呀，就是这整个职业是自由的，但你可能在有些地方会有一些（不自由），那就别的工作也会有这样的事情，就相对而言还是比较自由的。但理论上应该更自由。

吕梦娜：了解

刘书博老师：你看我们要买那个XX（扶贫产品）就很不自由，就是必须得买

受访者：就我觉得教师这个职业还是挺好的。

吕梦娜：那除了自由之外还有其他的吗？

受访者：就是可以实现自己的价值呀。就你写的东西都是署上你自己名字的，都是你的呀，别人拿不走，就是你换了学校之后也还是你的

刘书博老师：因为你到公司了就是给人打工了，你其实签署了一个卖身契。然后你干成的事儿都是上司的功劳，只不过经验是自己的，但是这个经验可以随着这个产业的快速迭代，快速地失去价值，但是学术它就是专业性的，就是知识密集的专业

受访者：就是你积累的都是自己的，而且可能越老越吃香嘛。

刘书博老师：所以其实我研究的一个兴趣点就是这种知识密集的专业性工作，我就觉得这个很有意思，医生、律师、咨询师和老师

受访者：就是这个职业给你的自由，成就感、意义感很强

刘书博老师：你可以定义一些事情。可以解释，可以帮别人看世界，但是你要做公司那种就挺难的，更多是操作流程

吕梦娜：您刚刚也提到研究嘛，特别是如果是要迎合期刊的一个要求，这种学术论文，可能感觉意义也不是很大，就比如说一个线性关系，大家都make sense那种的，就这样的研究，现在在您做的研究中的比例还大嘛

受访者：那尽量避免吧，但是你有些东西是学术范式的，那你为了你的职业生涯得写。

吕梦娜：但是如果说拿到tenure之后，是不是就可以不顾这些东西

受访者：对，拿到之后就不写了，应该是评上教授之后吧，所以你要找没有评上教授的，以后不要找有这个动机的人合作。

# 受访者19

12月1日13点的会议

刘书博老师：我们就是一个比较非正式的（访谈），不像纪委谈话，我们都是同行嘛，那咱们就大概聊一下。

首先我们希望了解高老师为什么选择要做学术。一般我们会觉得做学术读到博士的女生比较少，但是像您这种颜值又很高，那当时的心路历程是什么样的，可以从本科开始讲。

受访者：我本科就是中财的金融学院，因为我大三的时候金融学院有那种“卓越人才培养项目”，其实就是跟着导师做论文的，然后本科生基本上大三开始写（论文），就开始接触科研，那个时候觉得很痛苦，因为很长时间做不出结果，就结果一直不显著。我印象特别深刻，就是我搞了一个学期都没有好的结果，但后来发现确实在实证过程中犯了一点小错误，因为我们那个专业可能要做很多实证，就是跟数据打交道，然后在一些排序上出了点儿问题，后来修改了之后结果还比较顺利，就终于有了一个结果之后，我就觉得做论文这个过程还是能有一些收获的。

然后到保研的时候，我当时也是在专硕和博士之间纠结，就是我既报的专硕也报了博士，因为当时也没想好，刚刚本科毕业嘛，其实也不知道自己未来要做什么，我当时两种项目都报了，但是读专硕可能去不了北大，所以最后选择直博的项目。然后我读博士的那个时候也没觉得一定要做学术，因为我想就是读完博士即使不做学术···

刘书博老师：所以你是本科直接就···

受访者：对对，我是直博，北大经济学院，当时也没有想就一定要做学术。我当时想的是就是读完博士哪怕不当老师的话，也应该能找到一个工作，就是这种想法。

后来到我博二的时候，有那种助教的课，就是要给下一届上习题课，然后给下一级上习题课之后，我就觉得对这个上课的过程挺喜欢的，然后对于写论文这个过程吧，没有特别的排斥，因为我觉得就是很少有人是真的喜欢写论文，但是真的有哈。就是我是觉得大多数人是把它当成一个工作，然后我不是特别讨厌写论文，然后我对上课这个过程还有一点儿感兴趣，我就觉得可以考虑把当老师作为一个职业选择，然后从二年级之后我就开始逐渐的，比如说是不是要考虑开会，然后之后要多准备一些论文，找工作呀之类的，所以基本上是从博二开始确定要走这个学术的道路。

到了博士四年级的时候出去访问过一段时间，然后在国外我看到确实有很多学者非常热衷于学术、热衷于在一个领域里把这个问题搞明白，就让我非常的崇拜，但其实我可能还是不太能做到

刘书博老师：是去了哪里

受访者：我去的是南加大，去了半年，然后那半年我倒是没有去那边做什么研究，主要是上课，然后听研讨会、感受那边的学术氛围，我觉得那边确实有很多学者让我肃然起敬，就是他们对于学术的热情很高涨，而且对于研究问题那种持之以恒的态度，让人非常的向往。

到后来，就是四年级回来之后，正好赶上疫情，一方面是一直待在家，也不知道能干啥，另一方面是马上就开始找工作了，就开始纠结焦虑。那个时候就在想要不要考虑其他的工作，就出现了一点儿思想上的动摇，因为一方面是觉得这个教职也不一定能找到，因为很卷嘛，现在也很卷，越来越卷了，你们未来会更卷的。然后那个时候同学们也都开始找工作了，就开始焦虑要不要跟大家一起投简历、考公务员什么的。那段时间其实也投了几个简历，但我就发现当我不是特别想找那样的工作的时候，在找工作的过程中就没有特别投入，就投了一些简历，然后面试也没有特别上心，所以就觉得没必要浪费时间。基本上到五年级的9、10、11月份就基本上确定只找教师这个工作，到后来差不多今年的4、5月份就确定了这个工作，基本上就这个流程。

刘书博老师：那面试除了中财还有其他的地方吗？

受访者：我面试的教职还有首经贸，然后南京大学，就是也面试了一些京外的学校

刘书博老师：都是经济学嘛

受访者：金融学。其实我专业是金融学，然后还有对外经贸这样的学校

刘书博老师：那面试过程感觉怎么样？有没有一些觉得有趣的、或者是让你觉得意外的一些见闻、经历，还是觉得大家都是中规中矩的

受访者：我感觉还都是中规中矩的，就是介绍一下自己，然后讲论文或者是试讲，然后他们再提一些问题，然后回去等结果。

刘书博老师：那当时是都给你offer了

受访者：对，当时首经贸、对外经贸和中财，还有南京大学那边。但是南京大学我没有继续推进，就是他们只面试过我一次，我没有再继续参加。

刘书博老师：为啥

受访者：因为当时就觉得还是在北京，对于去别的地方没有特别强的意向

刘书博老师：为啥？

受访者：一方面是觉得自己一直在这儿上学，另一方面就是觉得如果是老师朋友都在这边的话，好像这边资源会更丰富一些，去南京大学那边就自己，什么都没有。然后对外经贸其实我没有录到金融学院，我是录到了别的学院，他们应该叫统计学院。

刘书博老师：首经贸呢

受访者：首经贸是金融学院

刘书博老师：首经贸是给到编制还是什么？

受访者：其实这个我没有再继续谈了，因为他们催的比较早，他们好像1月份就要求类似于签三方之类的，但当时我还在等这边的结果，所以那边就只能放弃了

刘书博老师：因为咱们这边给你的是师资博士后。那当时你希望的应该是想直接就到讲师还是？

受访者：当时就这个师资博后，也问过好多这边的老师包括我的导师，他们确实对于这个就是持一定的质疑态度，就说这个是不是风险比较大呀，或者是你可能读完一个博后还要再重新走tenure的路，所以他们当时也一直提醒我要考虑这一点。然后当时也问了这边的老师，包括我们怎么考核呀，或者是我们这样的身份到底应该怎么搞。一方面我觉得这边好像也没怎么想明白，就是无论我怎么问，他们也好像没有一个明确的说法。然后我当时觉得另一方面，这里（中财）是我本科学校嘛，可能还是有很多的感情，我是觉得中财不至于说坑骗我，就是这不至于说我来了之后会因为这个师资博后或者是编制的问题吃亏吧，就我还是对中财抱着这样一个信任，盲目的信任，所以说还是来了这边

刘书博老师：高老师看的出是很善良的，愿意相信组织

黄秋莉：这好像央财是第一选择，其实还有点儿疑惑，昨天我室友说咋不去对外经贸、首经贸呀，那边好像钱给的挺多的

刘书博老师：首经贸不行，对外经贸是要好一些。

受访者：但对外经贸的话，好像国内岗跟国外岗是分开的，然后如果是国内岗想走那个国外的制度的话，要单独申请了，当然申请可能也能过，但是考核就会更严，我也担心自己过不了考核

刘书博老师：对于中财，我也是跟高老师一个感觉，他还是比较正统一些，他比较保守，但因为保守而没有沾染太多市场上的那种公司斗争，为了金钱而谋利的这样。所以高老师愿意相信中财。其实愿意相信人的人，他对于这种谎言呀或者是骗局有更强的敏锐感，这个是心理学的一个研究。如果一个人总是很谨慎，反而容易被骗，因为他总是不愿意去相信别人，所以他总是认知比较短、比较有限，所以愿意相信人呢，他可能就可以更加好的判断出来。当然这个跑题了哈哈

受访者：愿意来中财也是考虑到专业吧，就我去那边的话也是去金融统计这个方向。

黄秋莉：对呀，我当时也想着你是经济学的、金融学的，感觉商学院应该不是最对口的学院吧

受访者：对对，金融学院没卷进去。金融学院今年招的人都特别厉害，没有卷进去。

刘书博老师：好，那我们听起来是，似乎你也没有太多的心理抗争，就觉得其实做学术老师对于自己来说不是特别的难受，而且自己喜欢教学，也不是特别抗拒写论文。

受访者：嗯，因为我是觉得打工人在哪都是要打工的，就是都是很痛苦。

刘书博老师：对，是这样，那你来了学校之后时间也不是很久，感觉怎么样？

受访者：感觉这个职业比我想象的管理要严格一些

刘书博老师：你觉得怎么严格？是来自于学院的管理严格还是···

受访者：我感觉是整个大环境，比如说今天开那个会，然后私自旅游这种

刘书博老师：对，我们今天开会说了有一个老师去西藏还是哪儿开会，然后他开完会去布达拉宫，自己花钱买门票进去看，结果就被通报批评，因为会认为借由开会来去旅游就是错误的，类似这种，但这个事情是挺过分的一个案例。那你除了这个会议上通报，还有什么其他的感受？

受访者：其他的感受···。我觉得咱们学院可能整体上保守。当然也可能是中财的风格

黄秋莉：是比较吗？就是跟北大这么一比呀？

受访者：对，比北大是保守多了。

黄秋莉：啥叫保守？啥叫开放？

受访者：就比如说我们办很多活动的话，都不敢办了

吕梦娜：是什么活动？学术活动还是学生活动？

受访者：学生的活动主要是比较多，可能因为以前我在北大也是个学生，我不知道北大老师是不是会管的非常严，然后学生的话那边是很开放，像每一个班可能会有班费，大家可以出去班级活动什么的，就是为了沟通感情。但是在咱们这儿我感觉一切都要跟着规章走

吕梦娜：当时在本科的时候没有觉得班级有什么凝聚力或者是班风之类的

受访者：就那个时候，我没有意识到就外面的世界是不一样的，我那个时候只知道中财的样子，以为这就是正常的，当我出去之后会发现，可能有一些活动上选择更多样或者是更开放一些。我本科的时候基本上学校不会组织什么活动，我们都是班里自己组织，班里自己组织也是自己搞，就是跟学校或者学院没什么关系。

刘书博老师：不知道你本科是在哪个学院？

受访者：就是金融学

刘书博老师：是在沙河？

受访者：对，我在沙河待了三年，最后一年在这边

刘书博老师：然后接着就在去北大读博士？

受访者：对，读了五年

刘书博老师：那就你其实一直是在象牙塔的环境。

受访者：对对，你看我这种单纯的，也是有点儿傻哈哈

刘书博老师：你不能说傻，其实我们有时候做专业化发展，需要这种夯实下来定下来的这种、就不能来回的摆来摆去

受访者：有的时候我在想，我刚刚工作嘛，其实我（之前）没有工作过，可能是因为我没有工作过，所以有点不适应。

刘书博老师：ok。那你觉得北大在培养你成为学者的这个过程当中，这个经历你感觉怎么样？是完全的一种制度化的，或者说是按照每年不同课程表的要求一点点的推进，还是说你是比较有自主性的，还是说导师他会对你产生更大的影响？

受访者：我感觉整体是比较自主性，因为我导师对学生管理不是很高压的那种，就给学生充分的自由发展空间，这是导师的特点。然后学院和学校特点都是他们为我们提供充分的资源，然后就是各个东西都可以拿得到，就是想不想要去拿是你自己的事情，但是他们会提供好一切东西。

刘书博老师：Ok，那就是再说一下具体学术的研究的内容，你会觉得现在的学术标准是不是合理的，或者说，我们在做学术这个行业，有一些标准也是有点固化的。就比如说我们要做研究，然后要发表论文，然后我们要做一些想要探索的问题，必须要用某一些范式。在金融统计、在经济金融这一块儿，我不是特别了解，我这边可能是工商管理会比较多，所以你们一般就是用统计学的方法进行研究。

受访者：对，我觉得是存在这样一个问题，就可能在做实证的时候总要处理一些包括内生性、异方差、相关性这样的问题。但是从我目前的水平来看，我也不知道这是不是一个弊端或者怎么样，只不过我们在做这些问题的时候都要处理这些现象。

刘书博老师：ok，那你当时博士毕业的时候有没有想着因为学金融这个专业似乎很容易到金融机构，比如说到投行，到基金公司

受访者：当时也考虑过，一方面是觉得这样的话可能加班会比较严重，当然我们现在加班也很严重

刘书博老师：就没有一个尽头的，是这个感觉嘛

受访者：对。

刘书博老师：但是你不加班其实也行嘛，就完全自主嘛。

受访者：对

刘书博老师：那什么样的动力让你在一个可以自主、可以轻松的情况下还要加班加点的工作

受访者：可能是tenure那个考核吧，因为我们要评上六年“3+3”的考核或者是八年之类的，总要度过这个阶段

刘书博老师：对你的要求是啥？

受访者：我现在只签了一个合同，就是三年的，三年是要求有论文和课题，至少有一个国家级的课题，然后还至少有一篇2A的论文。

黄秋莉：是两个叠加吗？还是或者

受访者：叠加，然后还要至少有一篇英文

黄秋莉：这已经有三个了，是吗？

受访者：对，论文是至少三篇，其中有一篇是2A的，然后至少有一个国家级课题。然后下一个三年，我觉得跟这个相比应该不会说更简单，应该是更难，所以就现在可能也是为了这整个六年在考虑吧，我不知道等我拿到tenure之后是不是就更换一种生活方式。

刘书博老师：你感觉压力大吗？

受访者：我感觉压力还是挺大的

刘书博老师：一个2A就相当于一个FT的，我不知道你们那个领域是···

受访者：其实我们也不是特别了解，但是基本上就是看了一下那个列表嘛，还是有挑战性。

刘书博老师：那现在你完成的怎么样？就在投了嘛，在修改嘛

受访者：对对，然后也在写，而且包括这个课题，因为课题我就觉得我无法保证在三年内一定能申上，我也是不知道那该怎么办。所以也很纠结

刘书博老师：课题这个反正我是非常悲观失望，因为我是做那个社科比较多的，跟自科完全的又不一样，因为我们是社会学的。那你们像申请青年大概中的比率是1/4，应该是这样，那虽然是这样，但是受访者也没中过

受访者：对，所以我就觉得如果我三年之后完不成这个任务的话，我该怎么办？我也会纠结这个，所以可能这几年还是不敢松懈了。

刘书博老师：那你每天日程安排都是自己定的很满这样子嘛，是什么样一个工作的方式方法。

受访者：对，其实我没啥日程，我就是早上来了开始写论文，然后下午没事儿就写论文，然后周三就是开一天的会，因为我现在就接任了党支书嘛，所以周三就开会，然后周四、周五上课，然后周六有的时候如果没有事儿的话，我也会写论文，然后周日就休息了。

刘书博老师：那你现在写作文全都是你自己写还是说会有合作

受访者：会有合作，我之前的导师，然后我的同学

刘书博老师：所以其实也是有一个这样的合作团体，导师也会给你一些方向性的指导。

受访者：对对，然后团体的话我觉得倒说不上，只是因为大家都需要写论文，然后就一起写。

刘书博老师：那你们比如说选择一个论文题目是怎么样选择？

受访者：就主要是看大家常做的内容，就可能比如说像我同学，他们也写了几年了，可能有一些自己常做的领域，我们就基于这个。

刘书博老师：导师呢，他的影响大不大？就刚才你说你一个同学他是一个异常值，因为导师其实带着他们发，那如果要发2A的，我觉得似乎这个研究的方向和题目是比较重要的。那如果只是咱们同龄人在基于各自的积累去想一个题目，能不能够让2A期刊买单，我在想这个问题。

受访者： 对，所以可能跟年长一点的老师会有合作，就希望他们能带给我一个2A的题目。然后像跟同学们，可能大家就是期盼能发一个差不多的就可以了，就没有过高的预期。

刘书博老师：ok，那平常除了写论文、教学和学院服务的工作，还会比如说到一些机构呀，或者到一些公司里面去看一看吗，你们这个学科似乎也没必要去，是吧？是比较理论化的

受访者：去公司看一看是指？

刘书博老师：就比如像我在教学的时候必须要案例教学，我不能只给学生讲理论或者是研究方法，我必须要讲具体的案例故事、市场上最新的商业动态和商业模型，从而让他们理解理论，而且管理学、工商管理这一块儿非常来自于实践的总结，我们的理论几乎都是从经验当中提炼出来。但是经济学呢可能就是构建一个模型，基于一些数学公式，所以似乎学科的差异性还挺大

受访者：其实我平常不太会去到公司，但可能会去一些资管方面的私募呀，或者是公募，就看一看他们在这个领域接收的是什么样的一种思维方式，然后我们做的东西他们到底认不认可。会做一些这样的交流吧。

刘书博老师：那他们认可吗？

受访者：他们对于研究还是认可的，但是对于你的结果倒未必百分之百相信。

刘书博老师：所以像现在我们做研究是不是主要目标是要获得tenure

受访者：对对，是的。

刘书博老师：那获得之后呢？就你获得tenure之后呢？

受访者：我没有想过我获得tenure之后会怎么做，就我还是会继续在学术道路上继续前进吗？还是说我就累了？我不知道。因为我没有想过，毕竟拿tenure这件事情不是说百分之百的把握，可能还是在纠结怎么拿上，没有想过我就拿了之后的···

刘书博老师：生活呢？

受访者：生活的话就是我有男朋友，但是我暂时还没有结婚或者生孩子的打算。

刘书博老师：也是学术界吗？

受访者：不是，是码农。然后我休闲娱乐的话就是看综艺，嗑cp，然后出去玩一玩，逛街什么的

黄秋莉：老师跟学生时代也没有太大的差别哈

受访者：毕竟我们现在工作也是写论文嘛，跟博士其实也没啥区别。

吕梦娜：就感觉是我们的学姐那种感觉

老师你就是一直学着读上来的吗？中间有实习或者工作过吗？

受访者：实习过，其实我大四的时候去私募待了半年差不多，一方面我是想感受那个工作氛围，就想知道自己到底适不适合做那个工作，然后感受之后就发现金融界确实就是灯红酒绿

刘书博老师：夜夜笙歌

受访者：对

吕梦娜：所以你不喜欢吗？

受访者：我觉得喜不喜欢那个氛围取决于你的身份，作为一个新人，我肯定是不喜欢那种氛围，所以可能对那种职业的考虑，就下降了对它的期待，然后到博士的时候基本上就没实习

吕梦娜：就这样读下来，其实年龄还是很小的，刚刚你也说过，就是可能会担心后面tenure如果不是特别顺利的话，那这样的话有没有焦虑感，就是还不如趁年轻的时候去工作，如果后面可能还要再涉及其他的考虑

受访者：我当时想过，就如果我真的被学校开除了，就是如果真的我第一个考核期没有过，我就再出去找工作

吕梦娜：还找教职嘛

受访者：就找别的吧，那个时候（年龄）也没有特别大，所以如果我真的被学校开除了，那我要么就去考公务员，要么我就去做那个资管

黄秋莉：反正就对自己挺有信心的，去哪里也不是事儿

受访者：就是我觉得如果我一直努力，就即使我被学校开除了，那也是因为我们学校要求太高了，我达不到他的要求，但我没有说我这三年就白瞎了，就我一直在努力嘛，那我觉得不至于说到时候被学校开除之后我就没有地方可以去了，我觉得我应该能找到一个工作，就还是要努力吧

刘书博老师：那你有没有对tenure这个制度有想法，比如为什么现在我们都在用师资博士后这个制度

黄秋莉：对呀，有没有不是师资博士后的地方？

受访者：其实首经贸就不是

刘书博老师：北京的这些好像比较少

受访者：然后其实对外经贸也不是，中财是。然后理工类院校、综合类院校，可能都是，因为他们本来招聘的名额就很少，他们基本上一个学校只有一个经管类学院，就不会像咱们说每一个学院其实你都可能可以考虑一下。

黄秋莉：这是什么意思？没有名额但是学校还招人，这是什么意思？那个名额就是编制的名额是吗

受访者：你说的是理工类院校吗？

黄秋莉：就是所有的。不是讲了有名额没名额嘛

吕梦娜：没名额就只能师资博后嘛

受访者：我觉得咱们是没名额的原因，但是有些理工类院校我觉得是他确实不需要这么多老师，因为他就一个经管类学院嘛，又包括所有的专业

吕梦娜：那三年之后就一定会有名额？

受访者：咱们好像没有名额了

吕梦娜：这个是指编制的名额嘛

受访者：对，我们再也没有编制了。

刘书博老师：我们已经是在超编的状况下，所以就是新人新办法，老人老办法，就是之前进编的，那你们不会变，但是再进来的都不是编制的而是合同制，但是可能签两个合同之后，也就是六年之后它就变成长聘了，就类似于编制了，因为劳动法也是这么规定的，所以有这样的一个区别。

受访者： 所以就是我们此生都不会再有编制，不是说留下了就是有编制的身份。

刘书博老师： 但是过了前两个考核期，那就等于是有编制，就是长期合同。

黄秋莉： 就感觉老师你也不害怕高压力，也愿意挑战自己。

受访者： 因为我也没办法，我是觉得就是当我没有办法改变这个规则的时候，那我就只能接受它。

黄秋莉：老师您当初为什么没有选择有编制的首经贸？除了跟自己的专业也不是特别契合之外，还有别的原因吗？

受访者： 就可能如果我真的去了首经贸的话，我会觉得这个学校的平台会有点儿低，我可能会有这种纠结，所以我当时主要考虑的就是央财和对外经贸，这两个对我来讲其实说实话，就是我觉得无论选哪一个以后都有可能会觉得是不是之前选另一个会得到更好的结果。

刘书博老师： 我觉得中财是一个正确的选择，对外经贸不行。你当时找工作的时候有听说过中山大学？

受访者： 中山大学在我们学生群体里面都已经臭了。

刘书博老师： 对外经贸好多人都说，就是感觉它非常的市场化。包括当时我去面试华南理工，他面试的方式就是公司的那种，应聘的人排了一长队，然后好多老师围一圈儿，然后每个应聘者去讲个十多分钟，那些老师似乎也没什么问题，也不太懂的样子，就是走个过程，然后就发offer，意思就是我们把你先圈进来，像养蛊一样，你们这些毒虫中最毒的那个留下，然后就相互卷嘛，反正对于学校是好的，但是对于个人来说是不负责的，对吧？学校他可以每年招20个，然后最终可能就只有一到两个能留，那其实这就很不道德。中山大学就是这样，所以在学生圈现在已经臭名远扬了。

受访者： 我毕业那年中大在清北金融专业没有招到任何一个人。

刘书博老师： 挺好的，我觉得就是对他的一记耳光，我们有时候虽然个人力量有限，但是大家还是可以团结起来发出自己的声音。

受访者： 对，但是就是我如果没有办法改变的话，就只能接受。

黄秋莉： 那老师当初求职的时候，可能就是把平台大小是放在第一位？

受访者： 对，可能是有考虑这个。

黄秋莉： 现在老师是同时进行几个项目呀？

受访者： 写论文的话，常规的一般是两篇左右，就有一篇要结束了，然后开始下一篇这种状态，如果多的话就可能还会有一篇，最多可能就是三篇，其实我写的比较慢。

吕梦娜： 但是我听着就感觉是有好多想法，好像就是只要结束就可以进行下一个，不需要空闲。

受访者： 是因为我已经逐渐接受了，学术它不是一个需要灵感，当然可能也需要灵感哈，但它更多的是一个工作，就工作的话就是要一直进行，对，就可能是这种“卷”（竞争）的思想已经深入骨髓，就一直在“卷”，就是你不能说你现在不想写，然后你就可以不写，这种工作就是要一直去做。

吕梦娜： 但问题是可能在我看来有些想法还太low了，就是感觉可能有偶尔的想法，但是也很垃圾，就是不能写出来那种，那你会对自己的想法进行筛选吗？还是说会认为我想的都是比较好的，就是都能够实现，能发表出来那种想法？

受访者： 那不是，其实我觉得你可以多问问老师，就是这个什么值得做。

吕梦娜： 但你之前不是说导师不是很管吗？但是好像也给帮助的样子。

受访者： 我们导师的“不管”是这样的，就是他不会主动找你，但如果你找他的话，他会很愿意帮助你，是这种。

黄秋莉： 那挺好的。我看老师发了一篇很高级的文章，那能讲讲那篇文章的发表过程吗？

受访者： Financial Management那篇也没有很高级，只是一篇2A，我们有个同学还发了篇3A，其实3A很难发的，但是今年金融学院招的那个人发了两篇3A。对，然后其实我觉得一方面是选题吧，但是选题也是老师们定的，就我只是做实证，然后写稿子之类的。

黄秋莉： 就感觉挺顺利，我看可能博二就开始出去参加学术会议了。

受访者： 那篇文章，其实我从大三开始写，然后到差不多到博二的时候才发表出来。那篇文章是跟咱们央财金融学院老师写的，当初开展了一个培养项目，就是说可以跟老师写论文，然后我是从那个时候就开始写了，大三有半个学期基本上都在处理那个实证数据。

黄秋莉：老师的同学们去当老师的多嘛？

受访者： 其实挺少的，我们这一年有20个毕业生，就是包括保险、经济思想史什么的所有专业一起算，其中只有四个选择当老师，然后其中有一个还是选择去首经贸躺平，就是他是有编制的，然后就躺平了那种。所以说实话算起来就只有三个人还处于一种“work”的状态，因为大家都觉得太卷了，感觉又累、 又没意义。

刘书博老师： 似乎是这样的，那大家有没有做出一些有意义的、有趣的研究，就这个你们领域有没有这样的研究？

受访者： 感觉没有，我们这个领域其实就是跟随西方那一套，然后就是跑数据、做实证或者就是做模型。

吕梦娜： 那大三学期那半年，就是您刚刚也提到这个数据不显著嘛，就是当时大部分时间都在做这个数据吗？感觉难受吗？

受访者： 特别难受，就一直不显著呀，然后不知道怎么办，也不知道为什么。

黄秋莉： 那最后跑出了显著结果，你是会觉得其实是我的推导是对的，只是当时数据有点儿小问题，还是觉得最后的结果可能只是一个勉强的结果？

受访者： 我当时是觉得就是挺顺利的，当然我现在觉得是运气，就是我现在终于意识到当时是运气了。当时结果正常了之后就很开心嘛，然后觉得好像做文章也没有那么难。但我现在也终于意识到了，那时是运气，就真的是命运中所有的礼物都已经在暗中标好了价格，就我现在觉得那是运气。

吕梦娜： 那其实你想当老师主要是因为享受教学这个过程，就是从开始做助教开始？

受访者： 因为我觉得没有特别讨厌教学，没有特别讨厌写论文，然后本身当老师就挺自由的嘛，因为除了上课就是写论文，就觉得比较自由。

吕梦娜： 那现在的话你觉得这个自由实现了吗？因为刚刚说到管制比较严嘛。

受访者： 就这个自由是有一点折扣吧，可能跟自己原来想的不太一样。

吕梦娜： 那这个限制主要是时间上的吗？比如说要开会？

受访者： 一方面是时间方面的问题，另一方面是它好像限制了我的想象力，就比如说我可能要做很多事情的时候都会想，这对于一个高校老师来说是不是不允许的，就会有这种意识。

吕梦娜： 所以高校老师这个身份是不是感觉很有光环？就是不能对不起相应的规则？

受访者： 不是不是，就是比如说像今天我们通报了，你不能在微博上胡说八道，然后也不能去旅游什么的，就当我做一些其他的事情我也会想，就是虽然规章制度上没有写，但我在想这个是不是高校老师所不允许做的，像职场潜规则这种，就是你要去遵循一些不成文的规定。

黄秋莉： 这种是今天开会后给你带来的冲击吗？

受访者： 是一直以来的会给我造成的冲击，我不知道刘老师怎么看这个事儿？

刘书博老师： 我每次开完会就很生气，就气哄哄的走出了会场，第二天我就俩眼圈就黑了，因为气得没睡好，哈哈。

刘书博老师： 我们现在初步的研究结论就是中国的学术，理工科的老师，就是那种自然科学实验室的科学家们还好，他们研究客观事实、自然界，那跟我们要研究的社会科学不一样，社会科学是一个主观创造的世界，虽然也是客观存在，但是它是主观累积之后的形成的客观。那这种主观的客观是会受到理论尤其是我们学者构建的理论的影响的，就像马克思，好多国家就根据他的革命理论产生了革命暴力冲突，所以其实权力非常清楚，我们学者研究的这种东西、构建的理论对权力是有很大影响的，就会让人们去思考这个世界到底合不合理，格局现状是否需要改变。那改变之后，就像清朝末年看到了日本的那个君主立宪制，那慈禧就开始杀人了，因为这种思潮进来之后可能会（危机当前的统治）。所以反过来我们是被控制的，也就是我们没有独立的学术地位。

我们缺乏两种独立，一种是我们是跟着西方的标准，另外一种是我们必须听上面的话，我们的理论、我们的发言要被层层审查，所以就是没有学术共同体的概念。又因为没有独立性导致我们其实没有办法发展出来自己的标准，所以它是有一点恶性循环的这个感觉。所以就导致了我们现在这个局面，标准很铁打一片，我们就像被锁死了，所以我们只能在这个有限的标准范围内使劲的精打细琢，那就没意义了，这不产生新价值。就像当年欧洲他们为什么能百花齐放，那种新的后现代理论层出不穷的？几乎所有的社会学理论都是欧洲的，就是因为他们是一个独立自由的状况，所以能构建出来新的赛道。

所以就我们访谈了那么多，发现了我们的不独立是两种自由的失去，就是自己学术标准的定义权和我们国内的这种学术身份是依附于权力的，所以我们上课的时候要注意言行雅正，不能够说一些过分的话，甚至说我们现在写的这个研究成果也只能发表在外文期刊，那其实也很无奈，怎么办呢？

受访者： 刘老师有没有担心就如果这篇文章发表出去会影响你在国内的工作？

刘书博老师： 不会，我不会写的就那么直白，就去说权力导致了什么什么，我会用一个非常不那么直接的表达，就比如说行政逻辑，类似这种行政和官僚制度导致了我们学术逻辑不是那么单纯，那我们看到的文献有这么说的，是没问题，那就不会直接攻击权力，因为我们一定要懂政治。

对，其实我想写的就是把这些具体的生态土壤给它挖出来，层层的展示出来。老外他们很多时候不理解，他们可以理解的是我们这个政治权力格局，但是他们不理解这个政治权力格局逻辑下形成的生态，这个土壤是长什么样，我就是把这个土壤给他展示出来。就比如说我们中层的领导干部在学校当院长的，他的护照平常就被收了，老外会觉得“我去，你们是奴隶社会吗？你自己护照都被收走啦？”所以很多时候他们读到这样的社会学研究就会觉得还挺有知识贡献的。我们这个知识贡献不在于说要改变，似乎也改变不了什么，只是说在一个具体的管理学商学院的环境下，让他们知道我们有什么独特的现象，以及现象是怎么样形成当下的情况，这背后的逻辑是什么，就把这个说清楚就行了。

而且其实就有好多东西可以写，还有一点就是我们现在学术生态缺乏独立性，非常像清朝末年，然后我们沦为了半殖民地，半殖民地社会就是我们虽然也有一定的主权，但其实话语权、定义权都不是我们自己说的算。而且有非常多的洋买办和掮客，他们会帮助那些帝国主义国家实现他们在中国的意图，他们从中起到了很好的桥梁纽带作用，或者说叫“木马”的作用。就像我们工商管理有一个人叫徐淑英，她是在美国拿到教职的一个比较有资历的华人学者，然后当年就是她把美国的那一套范式引入到大陆，然后成立了“中国管理研究国际学会”的这样一个协会，你可能不知道，但是这个在管理学界几乎就相当于美国最大的管理学年会，然后就通过这样的方式快速的让中国的学术土壤的标准趋同美国，我们虽然有很多中国特色的研究的一些具体的事项、方法、标准、范式，但是他们的范式就高高凌驾于这个本土的范式之上，成了最高级的。就特别像当年的洋买办，然后或者叫布谷鸟，布谷鸟就是很坏的，它把自己的蛋下到别人的鸟巢里，然后让别的鸟去孵化它的蛋，就有点这种感觉。美国让你们中国人来做我们让你们做的研究，你们做出来对我们是好的，那不就是布谷鸟嘛。然后这个被寄生的鸟还非常开心，非常投入。所以这就涉及到我们现在这个政治学研究叫后殖民主义时代，那当然这个东西需要我们进一步去探索，这个后殖民主义在管理学的具体表达是什么样的，我觉得现在这个问题还挺严重的。

我们这个学术原本是比较自由的、自主的、有思想趣味性的，然后现在就变成非常内卷、非常不自主的这样一个状况。所以高老师描述的那种状况听起来其实就变成了一种叫“学术生产线上的组装工人”，就那个感觉。学术它本来是比较有思想性的、比较技术性的，那有时候我们会走向极端。

你们还有啥问题可以问问。

吕梦娜： 老师刚刚说说了学术独立性的问题，那在科研方面比如选题什么的，你觉得这个自主权还在吗？

受访者： 还是有的。自主性还在，只不过有一些限制。

吕梦娜： 对，就比如说让你几年发多少这种限制吗？

受访者： 还不是，就有一些题目可能你觉得在中国没法做。

吕梦娜： 所以这个其实还是跟中国的国情有一点关系，是吗？

受访者： 对，就是自主性是有的，只不过这个范围好像窄了那么一点，但其他的都可以。

吕梦娜： 那比如说让你发2A或者让申请国家级课题等等，这些不会再进一步限制你吗？

受访者： 就这不会限制选题。

吕梦娜：因为我之前听张老师说，像申请国家级课题可能要往那个方向去靠，就是你不能想做什么就做什么。

受访者： 这个就是可能会有影响，可能张老师觉得比如他想做那个可能不好申，就是另一个可能会好申，那另一个好申的那个就是我想做的，感觉我就很随欲而安。

吕梦娜： 那你做研究是为了什么？

受访者： tenure呗！

黄秋莉： 那老师您觉得咱们那个考核期刊列表怎么样？

受访者： 我觉得那个期刊列表是挺不合理的，他们都说问题特别多，但因为我对于这些期刊也不是说每一个都了解，就可能有一些它的等级不太对或者怎么样的，是有这个问题的，但是这个修改的话也不是我们这种级别的人可以修改的。他们学术委员会应该是可以修改，应该都会修订，但是修订的阻力好像特别大。我们北大老师也说过，就是如果你今年要改的话，但是老师们其实都是根据这个参考来的，他可能有几篇文章已经在这个过程中了，或者马上就要发了，那你如果突然把这个踢掉，那他就会不同意或者怎么样，所以修订的阻力还是挺大的。

刘书博老师： 是学术委员会，你说的是学院的还是学校的？

黄秋莉： 那我们依据的是学院的还是学校的？不就是只有学校那一份吗？

刘书博老师： 两份，学院有一份学校有一份。

黄秋莉： 我们只有有一份呀。

刘书博老师：我不知道你们的是什么情况，那学校那个就是各个学院提供自己的列表，然后汇集。

黄秋莉： 那我们学院跟学校还不一样，这是为啥呢？

刘书博老师： 就是我们学院会觉得学校的有一些猫腻嘛，然后我们学院要比学校标准高，就评职称也是按这个更高的标准来。

黄秋莉： 那为什么其实是老师自己（学术委员会）定的这种期刊列表，它还没有体现出一种专业性，还存在这种等级不对的问题呢？

刘书博老师： 因为我们是想让这个标准更严格，但是学校标准会松一点，所以我们需要自己内部有一个。

黄秋莉： 我明白是两份会不太一样。对，但是我不明白的是我听到有些老师会觉得这个期刊列表不太对，明明这个期刊比那个期刊会好一点，但是期刊上的排列又不是这样子的？

刘书博老师： 那应该就是有一些有发言权的、又话语权的老师，他经常在那个期刊上发，他就把他推上去了。所以学术其实说白了，它带着很多政治、利益关系，不会完全的像自然界的那种客观状态。

黄秋莉： 对，我还挺疑惑的，因为在我看来学术委员会应该是一个很自主的团体，大家会平等协商决定。

刘书博老师： 你想如果一个本质上就是缺乏独立性的这样一个职业，它就不会形成对自己的这种自治，他就不是自治的存在，因为他自治不了。因为自治不了，它也不是自治的，所以它不会自我要求很高。

黄秋莉： 我明白，咱们那个学术委员会的成员是什么样的构成啊？

受访者： 这个我就不知道了。

刘书博老师： 学院不知道，学院应该是崔新建老师、林嵩、王瑞华……

黄秋莉： 是怎么进行决策的呀？

刘书博老师： 投票。

黄秋莉： 投票，看起来挺……那像高老师比如觉得有些不太合理地方，会进行反馈吗？

受访者： 没有渠道，就是从来也没有人问过我对这个我有没有意见，就是没有渠道，他好像并不care我们是什么想法，一直都这样子。

刘书博老师： 这就是权力的任性。

黄秋莉： 咱们那个考核看起来挺严的，老师您自己也觉得完成存在困难，那您会觉得这个合理嘛，就是对老师有这么多的要求合理吗？

受访者： 我感觉是这样，因为现在就是他制定了这个规则，其实我只能接受嘛，然后如果我没有完成的话，那我就只能再找别的工作。

吕梦娜： 老师在您签合同之前已经知道我要完成这个规则了吗？

受访者： 直到我看到合同的那一刻，我才知道。

吕梦娜： 就是已经签完了，进来了之后在合同上才知道？

受访者： 基本上是这样，但是之前其实也问过，大概知道是什么情况，就是我觉得就先这样，不行再说吧，不行我就去考公务员吧。

吕梦娜： 因为像我们这一级，我是一九级的，然后一开始就说发一篇嘛，后来过了一年还是两年之后，然后学院说变成两篇A，我就会有一种被欺骗的感觉。

受访者： 那这个时候你们可以应该去找学院。

吕梦娜： 但是也没有，就大家都很菜……

受访者： 感觉大部分是说新人新办法，老人老办法，应该从改了那一年开始，然后那一年入学的人才会改。

吕梦娜： 我们就觉得好像已经上了一辆车，然后你要是下去就得摔下去那种感觉。

刘书博老师： 所以中国很多事情它的改革比较快，而且中国的传统也是它不是一个经验式的建设，它都是一想到一个完美的目标，然后就大面积的彻底性的改变，他很接受俄国的甚至就是欧陆的革命传统，就构建理性嘛，他会觉得我们设计一个花园园林，我设计的方方面面都很好了，他是这种非常自信的改革方式。但是改革实践之后会发现有很多问题，然后再掉过头再改。有时候你看这个英美，他们这种经验理性就不是这样子，他们是温和的、渐进的。

英国虽然跟美国是很同宗同源的文化，但是英国到现在他们的大学也没有搞美国那种，搞得像中国这样普遍。他们还是那种像我们传统的这个编制体制，英国还是这样，所以姜老师去英国他就直接就是，他不用再评什么，再参加这样的考核，包括我英国很多博士同学，毕业了就成为真正的老师。

受访者： 我以为英国也是，我以为就是外国的都是这样。

刘书博老师： 不是，德国也不是。中国其实一开始像欧洲那边，后来北大清华在2005年的时候开始引进这个制度，然后就这两年所有的211都在跟进，像湖南大学，像贵州，像武汉大学，贵州财经这种学校都很激进的推，这就很有中国特色。

黄秋莉： 咱们那个学院好像每周三都要开会。

受访者： 基本上都是，周三就是开会日。

黄秋莉： 这老师们平时上课、写论文，这开会干嘛呢？

受访者： 通报这种旅游，然后就是上微博上胡说八道的情况，然后给大家一点儿教育警示吧。

刘书博老师： 就像管教小孩一样，你们看一下教育部网站的内容，很能够显示出我们中国对于学术的管理模式。那你再看看英国教育部，他不会说张榜公布警示故事，然后这也是中国特色，所以这一点可以拿个二手数据，就是中国教育部或者说我们开会的警示很中国特色，就是也彰显出我们没有独立性，我们就是被管着这小孩儿。

黄秋莉： 要开会，还要开一整天，有那么多值值得说的事情吗？

刘书博老师： 本来你看制度结构上、组织结构也是不一样，就是书记和院长两条线，这个国外是不会明白的，那这一点和整个生态是环环相扣的。

黄秋莉： 我们学院一些什么大小事物会共同商讨吗？记得有一次我做学生助理的时候有统计大家去哪里玩儿这种事情。受访者： 不是我们

刘书博老师： 再问两个问题吧，时间有限。

受访者： 我没有很多经验，不太清楚有没有这种征集意见。

刘书博老师： 我觉得管控的是比较严格，就学生要干嘛干嘛，都是有辅导员这条线在管理。

黄秋莉： 就是学院会征集大家的意见嘛，关于学院事务这种决策？

刘书博老师： 有限的征集，几乎不征集，非常有限，就是各种委员会可能会讨论。

吕梦娜： 高老师你有想过做大学老师这份工作的意义感来自于哪里？

受访者： 我其实做之前没想过，因为我觉得这就是一个工作嘛，就干饭！然后我做了之后我发现，因为要当班主任，包括要带学生什么的，就是会发现有一群感觉需要帮助的人，你可以给他们一些建议或者是说告诉他们你是怎么经历一些阶段的，我觉得这还是很有意义的，这也是我之前没有想到过的。

吕梦娜： 那之前你有想过的什么吗？

受访者： 我现在就没想过，我觉得就是拿钱。

刘书博老师： 有没有觉得这个大学老师在社会上身份地位很好，尤其是咱们山东老乡还是有一个很高的评价？

受访者： 我其实并不太在意这个，因为我觉得这个东西它既不能让我吃得饱睡得好，也完全没什么用，学术上不能给你带来一些什么意义。我的意思就是这个职业社会地位高对我来说意义并不是很大，那学术上的那种成就感，我觉得远远比不上它给你带来的折磨，但是带学生我觉得还是挺快乐的，就是感觉到能帮助别人。

黄秋莉： 如果没有考核压力的话，你觉得你的工作状态和现在有什么差别吗？

受访者： 我可能就不那么辛苦了？我不知道。

吕梦娜： 刚刚提到教学生带来快乐，那这个性质或者说这个工作更像接近辅导员之类的，会不会有这种感觉？

受访者： 讲课或者是学生在发展上有一些疑问嘛，像班主任那种。

刘书博老师： 就还是一种在智慧上给予帮助，而不是说具体解决了一个什么打包、帮他们打扫宿舍之类的。

吕梦娜： 明白明白。

刘书博老师： 其实这个大学它是一个事业属性、社会事业，它更多是贡献于社会整体的，就像科研一样，我做这个科研不是说要赚很多钱，我是让他泛化到社会进而推动社会进步，要解决一个整体人类的问题，但现在似乎就是教学这个社会事业性属性很强的一个工作内容就变得没有那么重要。

受访者： 因为很难量化成果嘛。

吕梦娜： 所以不考核教学方面？

受访者： 也考核。

刘书博老师： 考核就是我们课评不能低于80分，就这个也很难低于80分。

吕梦娜： 课评是学生评吗？我昨天看到一篇文章说是逆向选择，就是学生和老师之间会互相博弈，就是老师可能会担心学生给自己打分，那他就可能会降低上课难度或者降低作业难度等等，然后学生给打个好分。

受访者： 对，我也想过这个问题。

刘书博老师： 就不应该让学生评分作为我们这个教学的一个评论，我觉得这个是非常简单粗暴的、不作为的行政和管理，因为学生有时候他不知道什么是好和坏的这个标准，还是得让专业的人来评。

但是这个就很难评论，就是说这个东西很难量化、很难被测量。但好测量的就是发文章，那其实这个事儿我觉得你们读那本《指标陷阱》，那个讲的很透彻，你们可以把那本书的内容借鉴过来。说白了就是如果我们相信kpi，那可能会导致工作变质，人们只是在关注这些容易被量化的事情上，那些更重要更有意义但不能够很好量化的就不再关注，就导致这个工作就变味了。

黄秋莉： 那我再问一个问题，就是老师在学术道路上觉得有哪些人哪些事儿给你带来一个很深的影响？

受访者： 导师吧，我觉得导师比较重要。

黄秋莉： 他给你带来的是一个什么样的感觉？为什么会觉得他如此的重要？他是给你带来一些哪些思想上的冲击，行为上的示范吗？

受访者： 应该是说他在我感到迷茫的时候给了我很多帮助，就是你有想不明白的问题或者是纠结的点，可以去问，包括学术上的或者是道路选择。

黄秋莉： 道路选择上具体是什么意思？

受访者： 就比如说你要不要做兼职，然后要不要直博还是说编制这种，倒不如说他最后真的带给了我什么，是这个过程让我觉得受到了很大的帮助，就非常感恩这种状态。

刘书博老师： 好，那就到这里，谢谢。

# 受访者20

今天中午吃饭和朋友聊天，我觉得要讲行政和党的意识形态，还是新疆那边比较突出

从行政来讲：

之前他们有个书记，是从检察院过去的，别的也不太会讲，但是官话说的特别漂亮，比如“刀刃要向内，深刻进行自我剖析”[破涕为笑]刀刃向内，真是要笑死我了！还有县长也去当过他们学院的领导

他们学院内部总是开会，朋友形容是“炒菜式开会”，这个领导说一遍，另外一个领导又说一遍，还有一个领导又会说一遍，就像把一道菜炒了一遍又一遍，让人毫无食欲。

他们的领导被形容为“妈妈式领导”，话特别多，说的特别琐碎[破涕为笑]

他们的政策总是变动的，一个新领导上任，就会安排一些新的事情，就说“你们几个领导组织安排一下，把这件事儿推进下去哈”。

教务处特别爱整事儿，课件检查、课时审核、上课督导等等一系列管控措施。特别夸张的是，他们每周会把教务处完成事项列一个list，在全校通报，以显得自己干了很多事。

这当然与教务处的领导想表现自己、获得晋升有关。

教务处抓教学抓的特别严格，设立了专门的教学督导，每个教室安排上监控，那个教学督导就在监控里监视每个老师的上课情况。曾经有一个老师A出现了教学事故，就是把该给民族学生的考试卷子给汉族学生用了，按理说这其中送卷子的、监考老师和老师A都有责任，但因为老师A刚来没多久，所以就让他承担了全部责任。事故的后果就是当月工资全部没有了。

他们除了辅导员，每个班还专门设置专职教师作为班主任。在这个过程中，辅导员会不断对专职教师发号施令，比如“老师，几点几点学生们一定要到哪个地方”。

作为班主任的专职教师有各种麻烦事儿，比如一段时间内要去学生宿舍楼值班等。
 行政老师态度不好，比如让填报一个东西不给人说怎么做，但提交一个东西给他，他又说不对，让人反复修改。

同时，这些老师欺软怕硬，如果你怼他一次，下次办事倒也恭敬。

他们老师会被安排去“驻村”“值班”等干各种杂事，有些老师刚入职还需要先做一年教学秘书，然后才能开始授课

清华大学等学校的老师去讲座，他们老师也被拉人头，必须去听。当时有个清华大学的老师去讲“雨课堂”，人家学院已经用了很久了，但这位清华老师还在讲一些“雨课堂”的概念等，老师都不愿意听，一些实在忍不了的就走了，还有些人都睡着了。但是大部分人都没走，因为领导在那里，他们老师不好意思走。

党的意识形态来讲：

他们学校内部每隔一段时间会进行全面自查，抓所谓的“双面人”（大概意思是民族分裂分子那种）

他们曾经安排所有老师对所有的教材进行全面审查，一个字一个字地查，查里面是否包含一些“不好”的言论

不局限于党员，老师们要求“自我批评和批评他人”，搞得特别认真严肃，其中一个比较搞笑的例子是有的老师特别实在，自我批评的时候会写“自己思想有所偏差”、“对党不忠诚”等。

那其实这种自我批评要求手写，还会保存下来，到时候如果想升职啥的，还会审查这些东西

维族老师不被允许用维语上课

老师有各种扶贫的任务，比如过年的时候被要求“买扶贫地的鸡”

老师还会和南疆的一些家庭结成“帮扶对子”，被要求在过节的时候去南疆家庭待上几天

组织目标：

新疆比较特殊，特别听上面的话，教育部发了文件要破五唯嘛，老师评职称不能全看论文，然后我们那边的学校立马就改了，以前评副教授要求先达到三篇南核才能评，现在改成了只要有一篇高水平论文就行了。但是评的门槛是低了，现在也是要大家一起竞争，看谁发的论文更多更好。

虽然上面说是要破五唯，但最终还是各个方面还是要看科研情况，你看我们学校从大专升为学院，再到大学，都要看你学校整个科研情况。我们学院现在没有博士点，发展就很受限，比如外面有什么课题，那在我们学院和别的有博士点的学院一比，人家肯定就会觉得有博士点的学院好一些，是不是？你是不是也会这样认为，对吧？如果想要申博士点，你学院得有科研吧，论文、基金数量都得够，不然你又没有论文、又没有课题，你怎么带学生？教学方面也看，会看你二级学科、一级学科的情况，但占的比例很少，基本上不看。

之前又一次我们学院申请博士点，把旅游学院、经济学院的论文一起凑了20篇论文，数量凑够了，不过质量也就一般般吧。当时由我们书记牵头，带着院长和系主任去见各个高校的专家，意思就是说新疆条件艰苦，能做出成果来已经很不容易，希望到时候评博士点的时候，各个专家能投一票。后来也没过，把我们书记给气的，哈哈哈。

其他方面：

有一个MBA主任，还是复旦大学毕业的，心眼特别小。他自己跟人聊起他自己的一件事儿：有一个老师B在平时没有跟他打招呼，他心里就记下来了，后来老师B评职称要投票，他就给人家投了反对票，当时他从材料角落里找了一个概念要老师B充分阐述这个概念的内涵和应用，老师B没答上来，然后他又找了一个角落里的概念，让老师B回答～我当时就觉得这老师也好意思跟别人讲他干的这种事儿！

补充微博投稿内容：<https://m.weibo.cn/status/4686099140839058?wm=3333_2001&from=10AA193010&sourcetype=weixin>

各种硬性指标的增加，诸如几年非升即走、拿多少省部级、国级项目、拿多少经费、发多少篇SCI等等在我眼里其实不算“卷”，当你攒够了评职称的项目、论文，发现还需要懂得如何让别人投你票时，一下子就觉得卷了起来

为啥在各种评聘中实行积分制的高校不多，不敢想太多，也不想想太多，只能强装笑容、硬着头皮走下去。

不同高校情况也不一样，可能您那里985更公平公正公开一些吧，青椒第一关就是评副高，评副高在我们这儿除了校外专家评审和基本资格线，基本就是学院（不是学校层级）学术委员会几个委员投票的事情[允悲]

资格线其实不算高，相信您能体会我想表达什么。其实天天做科研、讲课、写论文已经几乎没精力考虑其他事情了，但是又不得不去考虑，小到朋友圈点赞大到一起开会时的言行举止，都生怕细枝末节得罪了哪一位大佬[笑cry]

看您的微博里，大家都是在担忧论文、基金如何达到非升即走硬指标，我已经达标了，而且拿了好几个项目，才发现这仅仅是起步。很赞同您的观点，博士/博后阶段是很多人人生中最后能够以100%精力做科研的时代了

现在我已经不考虑过程的公平性，只追求结果了。换而言之，论资排辈或者是让位各种关系户啥的都不会引起我太大的波澜，只求最终能在35岁之前能把之前很多想法尽快做出高水平的成果出来清华大学等学校的老师去讲座，他们老师也被拉人头，必须去听。当时有个清华大学的老师去讲“雨课堂”，人家学院已经用了很久了，但这位清华老师还在讲一些“雨课堂”的概念等，老师都不愿意听，一些实在忍不了的就走了，还有些人都睡着了。但是大部分人都没走，因为领导在那里，他们老师不好意思走。

如果是硕士学位的话，在学校不太好混，比如值班等事情就不会安排博士学位的老师去干

有一些老师想要离开学校/新疆去别的地方发展，但是人家学校根本就不放人走，会把人事档案给扣下来。之前两口子老师去别的学校工作了，但是人事档案就是拿不走，现在也没拿走。

有些人想要做官，但还是有很多人喜欢教师这份工作，做行政的话杂事儿太多，做老师的话比较自由。自己性格也不喜欢巴结别人。做学术这份工作的话，只要长期持之以恒，还是能升职成功。

虽说比较外在来看比较自由，没有固定的上班时间，但朋友也提到，做老师工作和生活边界模糊，脑子里始终有一根工作的弦。

当然编制是前提，有编制的话工作就比较稳定，要辞退你要走人事处等各种程序，需要明确的理由。但是在企业的话，人家想辞退你就能辞退你。如果没有编制的话，做老师就不太好，你要说工资的话，肯定没有人家企业里挣得多。

朋友就觉得，与其在那边干各种杂事，还不如出来读博。他们学校大概有1000名老师，现在有100多个人在外面读博。

# 受访者21

黄秋莉： 我先问刘老师一个问题，其实是上次的话我们谈了还是挺多的，可能对老师自己的这种研究心得，您自己觉得在这种非专业性的影响的话......

受访者： 我觉得在中财可能非专业性会影响的比较多。

黄秋莉： 嗯，在中财是这样，那在别的地方呢？

受访者： 在中欧可能这个就不是那么清晰。

黄秋莉：哦哦，你是之前在中欧有工作过。

受访者：所以你得把问题给清晰化，我的经历它分成大概有不同阶段，我对于学术界的认识也是由这些不同阶段共同塑造的。第一个阶段就是我在读本科的时候在东北师大，然后我对于东北师大那些老师，对于他们的工作方式，对于他们的一些认知观念，我大概有了解。然后又去到英国一个教学型的大学叫胡佛汉顿大学，然后又去了英国很好的一个研究型的大学，但是它很新，是一九六几年成立的，发展的非常快。那博士研究生毕业之后就去了中欧国际工商学院，也是一个很新的，90年代成立的，然后现在在中国来说已经是数一数二的商学院了。但是它只是排名指标非常好看，我对于他的认识是它比较像咨询公司，比较像培训公司。然后就继续读博士，我工作两年之后去到英国，接着在爱丁堡大学，它是一个百年的、非常代表英国教育的一种大的综合型的研究大学，所以我经历的这些不同的高等教育的机构还是挺多样性的。我研究生就在商学院，在管理学领域，在英国那边，那现在呢我又在中国，然后又跟美国教育背景的教授和欧洲教育背景的教授都有过接触，所以我觉得对于管理学的全貌我还是可以有一些认识的，所以你们的问题可以再细化一些。然后这个商学院的研究、管理学的发展的研究我其实一直都比较感兴趣，只不过知识社会学它就是抽象的抽象，它不是那种简单的研究社会行为的一个社会学研究。

黄秋莉：嗯嗯对，它是研究文化的

受访者：社会学它是研究社会文化的，那它具体研究对象就是人的文化影响的行为，那我研究的还不是具体的行为，它更加抽象为一种教育活动，或者说观念世界的一种构建和流动，所以知识社会学它更多的是一个抽象的层级。所以这个研究我觉得要做好其实挺有挑战的，也是为什么咱们现在少有人做，因为大家要真正做好是需要花一些时间努力，包括你需要有一些个人的经验视角。那我觉得我是具备这种经验视角的，所以我一直想做。包括你们想参考的一个范例，就是我刚才说的那个斯坦福大学的周雪光老师，他的博士论文写的就是斯坦佛大学的这种创业教育制度，他研究的就是那个大学的教育理念，他们的学科制度是怎么样成立起来，所以其实你们可以看一下他是怎么做的。他是一个非常标准的社会学研究，还有一个，我觉得你们要重点参考的就是阿伯特，他有一本书叫《专业系统》，那本书翻译的不是很好，但是他那个书很重要，因为它是开辟了这一块儿——有专业知识的社会学这样一本书。所以咱们现在是这个社会学的分支领域，就专业知识社会学是在六七十年代才有的，那它也比较新，然后我们对于专业像研究咨询顾问、研究会计师事务所，研究法律律师事务所，这些都是有成熟的研究成果的，尤其是研究像麦肯锡这样的咨询公司，因为咨询行业也是比较新，所以我们看到一些研究成果的特点都是研究的是一个年龄不是很大，大概二三十年，然后呢他又形成了一个蔚然成风的行业，包括它有自己的行业标准、知识标准，有自己的专业属性，一般我们看到发表的成果都是这样的研究对象。那再回到商学院，我觉得中国的商学院和管理学就具备了这种特征，因为我们也才改革开放三十多年嘛，商学院之前是没有的，管理学也没有。所以我们现在也是一个蔚然成风的，包括中欧MBA已经进入了全球的前十名，而且我们发表了特别多的顶刊文章，所以我们在说这个事情的重要意义是毋庸置疑的，审稿人肯定会认可这个事情，这个也就是说：我们选题的合理性，还有我们研究的意义性已经有了基础。所以我们这个事儿只要能够做出来，它是有非常多的发表渠道，只不过是我们具体要把它写的比较精致。就像刚才那个窦老师说的：那种顶刊文章比较规范和细致，那现在我们这个工作在访谈层面还是有点少，我觉得顶刊像发到FT的最起码要近100个访谈量。

刘梦娜：是，我记得之前还看过一个，说是要求100个

受访者：对对，他那个数据量很够，所以我们其实要做这个难度不大，是能够访谈到一百多位

黄秋莉：嗯嗯

受访者：所以访谈量可能是我们现阶段最大的挑战，但是现在我们知道现象提炼，还有就是我觉得我们的研究问题设计都比较清晰，关键就是做访谈，然后对于数据有一些规范性的处理，是这样。

行，那我就回顾一下我对于管理学的看法，其实我那个看法很浓缩的写到那篇《管理学的尴尬》那篇文章里了，而且那篇文章我是大概一气呵成的，大概写了一个小时就直接就写出来，所以其实那篇文章的经历就是我在家下午没事儿，也不是说计划好久要写，就是闲着无聊，然后就想到这题我就开始写，然后很多内容就直接就出来了，写完之后我就发到公众号。公众号发出来，立刻被赵老师——赵向阳老师给看见了，他就发到那个管理50人论坛，然后那里头有一个上海交大的前院长叫王方华，然后他们他就给了很高的评价，然后他们内部就开始往各个群转，然后大家看到之后都比较认可，就反响比较强烈，所以很快的积累了大概三四万的阅读量。所以这个事情其实也是从一个侧面反映了我说的事情，我说的这些东西它是它是valid，就确实是这样。那管理学现在确实发展成了一个比较异化的状况，包括我在中欧工作的时候，当时的老师也是老板吧，他就说他是剑桥的博士，他就说现在翻开那个顶刊的期刊论文，看到的都是那种复杂的数量模型，大量的数据的表格，没有管理者会看这些东西的，但是大家又都是这么做，所以其实在08年的时候就已经是这样子，但是那个时候相比现在来说，我觉得现在会好一些。现在比如说AMJ，它每期都还是有案例的，它那个时候好像似乎连案例都没有，全是实证，然后AMJ在管理学季刊还有一个对于商学院教育反思，其实我们看到一直以来都是有海外的学者在对这一块儿进行反思，但是这个过程它不是一蹴而就的，它需要经过各方声音，大家共同的努力，它是一个缓慢演进的生态改变的过程，所以我们现在正处在这样的一个转变的过程中，所以你们可以再有一些关于我经历、关于我的认知看法的细节问题，我们再讨论一下。

黄秋莉： 老师，我有一个问题，就是我自己在看的时候，大家都在说其实商学院是一种趋同的发展哈，那我们国内也是因为我们刚开始学这种建设，包括学科的建设，包括这种老师的培养都是采用外国的模式。那我们这种特殊性还是体现在这种文化的土壤层面，我觉得现在在多个地方比较的话......

受访者： 我在英国读书那会儿，我觉得他们英国的管理学跟中国很不一样，跟美国也不一样，他们的研究范式多数是质性研究，这个研究的一个特点就是它会关注现象，它不是要追求那种普世的规律、测试一个假设，就是比较案例研究的那种范式，那案例研究其实说白了就是更本土化讲故事。

黄秋莉： 嗯对

受访者： 就像刚才窦老师说的，他会觉得理科生的思维就是：对于故事就会天然的觉得不靠谱，他要追求理性的规律，所以你看他其实他那个认知在哲学上讲就是认知论，存在论，它就是实证主义认知论存在。但是英国那边是比较偏社会建构主义，英国人他们会更加强调文化的这种差异性，美国更多的是标准化。其实我觉得历史塑造了他们不同的认知，因为美国向来就是把英国建构起来的这种文化标准、市场标准、法律经济制度标准直接移植过去，他们做的就是拿着最优秀的成果直接去用。所以他们美国的历史在我们看来就是拿来主义，他们有点像深圳，所以他们对于制度这一块儿并没有太多的反思，那其实我们理论就是在构建一种新的制度，所以美国的研究就像他们的泰勒主义一样，他们是把这个效率给最大化，他们并没有产生新的所谓的工业革命的标准。那他们的管理研究也是这样子，就是泰勒主义，英国管理学是这样说的。我天然接触的就是他们会有很多思辨，会有很多对于文化的反思，他比较像人类学、社会学，更多是这样子。

黄秋莉： 所以我们中国现在就是从一开始就学美国

受访者： 中国是比较偏美国的，我们似乎有点日本人的心态，就是我比不上你，我很佩服你，但是口头上又特别骄傲，我们要中华民族......这种，但是实际做的时候呢，我们在专业标准上又特别的听话地、服从地看向美国。我们会在专业评判的时候不假思索地把美国的这一套标准当作我们的标准，因为他会觉得美国就是老大，这个时候学者的那种桀骜不驯完全没有了。这就很荒诞，就是我们在话语当中强调中国特色，包括要做中国大地研究，但是回到评审标准的时候又在跟着美国走

黄秋莉： 这个老师之前有讲过嘛，我们的知识标准和管理标准就是完全是用美国的标准

受访者： 所以我就觉得这一块儿呈现出了后殖民主义的特色，包括今天上午给你们看的那篇《知识分子的消亡》，消亡说的就是这个事情，因为知识分子自己不思考了，他就觉得文科的东西没有理科那种有价值，这是一个好像原发性的在高中就已经非常根深蒂固的，就觉得真正有价值的就是数量化的、科学的、自然科学的，而不去思辨这个观念的世界。进而一步步地永远不思辨整个过程，所以导致我们不会对观念的问题有问题。

黄秋莉： 对，您刚刚就讲到了一个量化的问题嘛，但是我感觉那篇文章里面也讲到了很多其他的东西，他讲知识分子为什么消亡，他还讲了美国这种社会的变迁，就是以前知识分子会有很多聚集的地方，就是那种小酒馆呀，小茶馆呀，但是现在被高楼大厦给取代了，他们没有生存空间，他们被收编到大学里面去了，就导致他们不出现在民间了。就前几天我看到一个新闻，还讲了一个工人翻译了维特根斯坦的哲学，但是这么一个非常特殊的情况，可能在以前就比较...

受访者： 所以你看收编的好处是给他们一个专注的职业平台，让他们可以拿到稳定的薪水去做专业的事情，但是一旦被收编，这种管理主义的思潮，它就要被管理了，它就失去了自由，就像动物园的动物一样，它的野性就没有了。知识分子没有野性，知识分子没有独立性，就像老虎失去了野性，它就变成群居动物了，那群居动物它就没有知识分子原本的价值提供了

黄秋莉： 对，我们看到的是这么一个现象嘛，但是老师自己可能作为这种被规训的猫可能有一种感觉，知道自己是如何被规训的吗？

受访者： 其实我觉得我在英国不是特别被规训，因为我走来走去我会对比，我会有一个超脱的旁观者视角，而不是说一开始就在一个环境，那你有时候会像井底之蛙，觉得天就是这样子。我不在那样的一个环境下，我会反过来看之前的环境

黄秋莉：所以我会觉得你有一个多重的视角对比

受访者：对，我觉得美国跟英国就是不一样，虽然他们都是同一个文化，但是美国就像中国的深圳，深圳特点就是只搞钱嘛，没文化嘛，文化荒漠嘛。跟美国也一样，我做的事情虽然也有很多优秀的头脑和思想，但是整体的氛围是更加地被商业化。其实为什么他们选择实证主义？就是这个实证主义与他们的标准化是很契合的。实证就是科学主义嘛，说白了就是泰勒的科学主义。所以泰勒的科学主义可以让官僚制度产生最大的效应，就是效率提升嘛，但是其实学术有时候不是在追求发展效率这一个价值，它只是一个工具价值，除了工具理性之外，还有其他的价值理性。

就比如说我们不能够评价说爱因斯坦的相对论用效率主义来评价，它是一种颠覆性的，那我们说他应该是多元化的价值感，但是美国它很凸显效率主义。你看美国的商业发展，在二次工业革命之后，美国在效率层面是老大，欧洲就不是，欧洲很多时候对于新的思潮、新的管理手段，它的接受总是跟着美国，而且麦肯锡在这种扩散上起了非常重要的作用。可以说麦肯锡代表的就是美国的这种意识形态的霸权。它不仅是商业的，他还把表现在人的管理理论、认知管理学标准都是他们说了算，包括我们现在积极地融入到AACSB这种世界商学院认证体系，说白了也是认可到他们那一套评价系统里。所以美国人特别擅长把这个事情标准化。

黄秋莉： 对，您可不可以跟我讲一下您翻译的那个书，麦肯锡是如何在推向世界的过程中去推行他自己的标准？我感觉他推行这个管理标准对人的关系其实是跟美国的商学院在全国推广这种标准是有一致的地方。我想了解麦肯锡他自己在全球推广的时候，做了一些什么事情

受访者：他就是把美国最大通用汽车的或者通用电器的改革方法带到了欧洲的公司，让他们去用同样的方法进行所谓的多元化改革，所谓的组织部门重组的改革等等

黄秋莉：对对，他相当于是对这个地方进行了一个再造。但是好像国外的商学院对我们国家没有进行这样，反倒是我们自己去主动学习的。

受访者：我们其实也不是（完全自己去主动学习的），也还有麦肯锡这种掮客，还有就是徐淑英这样的人，包括我们学院请的那些香港的主编之类的，他们就像麦肯锡一样，他们也不是虔诚的传教士，他也有利益的关系。就像麦肯锡一样，麦肯锡总是说他追求价值，要带来企业长期的基业常青，但是他收的费用那么高，全球最高吧。

黄秋莉： 对呀

受访者：所以你会发现学术有时候也是一样的，这些学者，他们就像被利益吸引，被吸到中国。我们中国的学科发展晚，所以心态上似乎想要比学赶超，想要快速地弯道超车、变道超车。国家有这种指令，所以底下的人就会形成一种筛选机制，或者是劣币驱逐良币。发展的最快的就会被选拔出来，那什么发展的快呢？就是用那种显性标准，看是否达标了。我们快速地通过这种外部的麦肯锡式的方式，直接花笔钱把外部的东西买过来复制粘贴，也不管这个东西能不能够生根发芽，是不是适合中国土壤，我就先把这些kpi给达到了。这个问题就涉及的比较广，就会涉及到高校对于学术治理的问题了。那我们治理学术教育甚至中国足球难道就用kpi的这种方式吗？我们先说足球，他不能够只是口头觉得自个儿厉害，他不能像教育大学一样——我们自个儿说的双一流就是双一流，他还是要比赛嘛，所以足球从来不敢说自己双一流，但是他的这种发展模式跟教育是一模一样的，对吧？也是国家提出一个倡导，所以这些管理者总想走捷径，弯道超车，找来各种外部教练，花很多钱，在kpi上做文章，但他不考虑土壤环境的改变，，他没有耐心做长期的工作。结果就是走了弯路又走弯路，觉得不尊重这个事情的本质规律。

黄秋莉： 对对对，就回到刚才那个问题——“把老师都收编到大学里面去了”，这实际上也是知识分子为什么会消亡的一个原因吗？就讲到知识分子这种思辨性变弱了，您刚刚提到了一个是这种管理主义吗？

受访者： 可能这就是一个全球的大趋势。

黄秋莉：对，对对对对

受访者：工业革命就是用一套科学的标准和程序，然后让人们的组织更加高效，这就是工业革命带来的。就像那个现代化嘛，所以现代化也会产生一些副作用，现代化的副作用最标准的体现就是让人不去主动思考，所以鲍曼的《现代化与大屠杀》那本书讲的很清楚。我觉得你们在写文章时候可以去参考鲍曼的一些讨论。为什么现代化会引发一些灾难，因为人不思考了，人不思考，主要是人觉得科学技术可以代替人的思考

黄秋莉：对，这是一种伪科学主义

受访者： 对，科学主义倾向，鲍曼说的更加细腻，我们只是提炼出来一个一句话的概括，鲍曼说：这种不思考会让人们在一个组织环境中变得非常的狭隘，非常的像技术工人一样的工具化，缺乏主体性，缺乏对于世界的关注，所以它是一种道德上的懒惰吧，或者是有一个词叫“道德无视”。就像理科生不关注社会价值，觉得你们这个都是讲故事，这种是欺骗别人的，他会有一种偏见，他会觉得数字才是真正科学的，才是真正可以相信的。其实他刚才那个说法反映出来就是一种不思考，他就觉得你不要跟我说，就是知识分子也变成那种科学嘛，好像没办法去反驳这种科学的。

科学它不考虑价值。但是人是有价值观的，科学是没有价值观的，科学只是一种价值观，他没有对错，我们不能够用科学来审视人文的价值观。

黄秋莉： 我明白，我好像看过一个观点，他的意思就是说这种学术的世界造成了生活世界的一个异化，学术的一些标准其实已经导致我们的底层生活发生了变化。

受访者：因为学术现在就是科学主义嘛，而传统的学术是什么都考虑的，形而上学的，包括亚里士多德会考虑人的生活的方方面面，以及人性。包括休谟，他会考虑人性到底有什么特点，但我们现在用科学来审视休谟的人性论，他就不科学。

黄秋莉：对呀

受访者：他就是想当然的一个东西，它是哲学性的，对吧？所以就是我们现在哲学退化成了一种自然哲学，只是在这个圈子里面。我们现在的经济学是道德无设的经济学，不考虑对错，不考虑是非判断，只考虑基于理性人所构建出来的非常数学化的经济学大厦。

黄秋莉： 其实我觉得我们自己的文化本身是有这种思辨在的

受访者： 我们传统的农业社会，在工业革命之前都是会有思辨，大学教育就是精英教育，就是思辨教育。就是帮人去思考，而且思考的是社会的人的东西。现在我们思考的更多就是桌子椅子技术呀这些，

黄秋莉：就感觉西方的科学，现在我们国内表现出一种唯科学的倾向，对我们本土的思辨性具有一定影响。

受访者： 唯科学只是现代化的一个表达，现代化还有很多其他的表达方式，跟科学很接近的一个现代化的表现是量化指标，可以清晰度量kpi；第二个是标准程序，你做一个汉堡必须这么做；然后第三个就是对于标准的不断修进、改进，叫反思性的，这个是韦伯的观点，韦伯讨论我们陷入理性的牢笼的一个原因在于我们缺乏反思。缺乏反思就可能受到科学主义的影响，我们缺乏反思，所以会导致现代化出现了种种弊病，其实都是人的问题。

黄秋莉： 我们会认可普遍化标准的一个重要原因，会不会也在于它本身就会不断地改进？我看了一篇讲AACSB等认证的文章，这个认证除了包含一种工业化、标准化以外，还包括一种不断改善的含义。

受访者： 对，比如徐淑英他们这些人也说我们要不断的吸纳意见，改进自己，但是这里面有一个很隐晦的点，就是他们总是在非常自主的在说要改，但是这个权利关系是一种不对等的权力关系，对吧？

黄秋莉： 我自己的理解是我们是在改进，但是我们也是在被他人确定的那个框架内进行改进，所以导致我们跳脱不出去，但是我们还觉得自己在不断的改进。

受访者： 对，他们其实就有点像《水浒》当的那个招安嘛。

黄秋莉： 很奇怪的一个点在于也是徐淑英老师他们那批人首先提出来要做中国本土研究。

受访者： 他要维护自己的合法性，对吧？所以面对那些质疑他提出来做中国本土研究也是要维护自己统治者的合法性，说白了是这样。对，所以有时候我们看他表面上坦诚布公的或者非常开明宗义的，但这个不影响他的霸权地位，他还是一个霸权。

黄秋莉： 接着微信公众号推文“知识分子的消亡”，它里面提到以前有一些还是守着知识分子原则，为社会思考、为民众思考的人，但是后来国家开始给他们也是发基金呀，就导致原先那一批知识分子被清退了，然后剩下来的都是为国家辩护学院派知识分子。

受访者： 所以这个时候就还是学者没有独立性嘛。

黄秋莉： 对呀，就是讲消亡了嘛，那在我们国家好像更是这个样子的。

受访者： 对，我们更是这样子，我们知识分子的独立性首先受到行政的影响，第二受到海外意识形态霸权标准的影响，所以是双重不独立。

黄秋莉：关于行政的影响，您自己对这个有很深刻的感悟吗？就我可能就会很肤浅地认为行政就是有很多行政人员之类的。

受访者： 不是，行政的意思是有层级化的管理，就是官僚制度。包括泰勒的工厂也是官僚制度。像真正的学院应该是自制的，是雅典城邦那种选举样式的，但是我们学校没有选举，大领导就不是你选出来的，而是从上到下任命的，这种时候可能我们会觉得这还不是那种真正的理性组织，这就是一种权利组织，从上到下的去安排你、考核你、审视你而不是说大家平等的，像麦肯锡他们就是民主的。当然麦肯锡那个一开始的制度就是任人为贤嘛，大家共同投票选这个老板，那中国大学不可能让你投票。

黄秋莉： 对，首先我们这个组织就没有自己的学术权力组织嘛，学术委员会也受制于行政化，导致教师职业自主性受到压制，就是我们连自己的组织都没有，就自己表达自己观点都找不到一个出口。有一句话叫我们老师处于一种决策的失语境地，我们好像只能够去接收领导的决策，然后你即使有反对意见，你也没办法表达出来。

受访者： 你的表达和你的思考是在一个限制范围半径内的，你会比较清晰的感受到这个边界在哪里。

黄秋莉： 我有一个疑惑，就是每个老师都说我自己的选择是很自主的。

受访者： 那你觉得井底之蛙会觉得自己不自由吗？他觉得世界就是这样子，但是我们跳出来看就能发现它不是自由的。
 黄秋莉： 对，我就想去看这个井口到底是什么井口。就比如说我们是井底之蛙，但是我就想说到底是什么限制了我们的一个视野呢？

受访者：那就刚才讨论的嘛，就是学校这种从上而下的治理模式，权利限定了你的思考范围，比如说你要讨论经济经济学，你必须要考虑说我们现在是倡导的是社会主义优越性，然后你就不能像美国那样的去讨论，你就不能讨论说要限制政府权力，这个对我们来说是很清晰的呀，我们在中国生活都能够感受到权力对于学术的限制，

黄秋莉： 就有些话可以讲，有些话不能说。

受访者： 这个都属于常识了，而且我们每个高校都有马院，这个也是大家都可以看得见的一个权力的直观表达呀。而且真正的学术如果总是被权力所控制的话，那这个学术的发展我觉得是不真诚的，所以真善美的真就没有了。

黄秋莉： 对，但是我感觉这种情况应该是不会改变的。

受访者： 对，这个就不在我们讨论的范围了。因为我们不去探讨政治制度的问题，我们只探讨专业标准、专业制度的问题。我们可以说我们特色是有这种行政的，所以就用这个词就行，就叫行政逻辑嘛，像我们看这那个范文就说了市场逻辑吸收了行政逻辑，那这个表达就已经很充分了。

黄秋莉： 市场逻辑如何通过组织得以表达呢？我能知道有通过个体得到表达。

受访者： 组织表达特别明显，我们发一篇论文奖励多少钱这不就是市场逻辑吗？我们用这个把学术变成了一种货物，然后对它进行估价，

黄秋莉： 这就是学术资本化了嘛？

受访者： 你可以这么说呀，然后我们学院再加一层码，学校有奖励，学院的奖励更大，还有的学院就是你发了一篇顶刊每年都给你多少多少钱，对吧？那他这个就是对思想进行了一个估价，这不就是市场估价吗？

黄秋莉： 是，就感觉现在我们除了这种专业逻辑，我们在这个组织能看到他的一个行政逻辑，能看到它的一个市场逻辑，就是对我们个人也有很大的影响。除了市场逻辑、行政逻辑、专业逻辑，还有那种西方中心化这种殖民主义，从组织层面来说，还有别的吗？

受访者： 基本上就这几个。

黄秋莉： 我自己想到一个就是这种人和人之间的关系嘛，这种社会网络方面的困扰算一个吗？

受访者： 学术团队这种各个国家都有，只要是人他都有社会网络。

黄秋莉： 但是相对于专业逻辑来说，这种人情世故方面的属于异质性的东西吗？

受访者： 它比较像专业逻辑，但是你可以进一步说中国的专业逻辑，比较偏向于网络关系的这种，就中国的专业逻辑会更加强调关系，而不是学术标准。

黄秋莉： 我自己的一个体会就是我们国家和别的国家相比较，这种熟人社会、关系还是比较明显的。

受访者： 可以这么说。

黄秋莉： 那这个算什么呢？这个算文化？比如学阀、基金评审当中的人情关系等。

受访者： 我觉得不需要把它单独再拿出来，这种关系面向属于我们国家专业逻辑的一种特征，属于它的一种性质，就是内嵌于我们专业领域的关系嘛。

黄秋莉： 就相当于是我们专业逻辑的一个面向，是专业逻辑的一个特色。

受访者：对，为什么我们专业中比如可能评基金的时候会有各种问题，可能就是因为有这个圈子文化加持后的影响。

黄秋莉： 这可不可以把它算作非专业性对专业性的一个腐蚀呢？

受访者： 你再看一下阿伯特，看他在讨论的时候有没有这个视角。我觉得这个不成一个单独的视角，它是嵌入到专业逻辑里面的。因为各个国家都一样，那我们在研究的时候还是应该竖一个视角，这个视角得有意义，如果我们研究的东西，大家其实都大同小异，那它就没啥意义，而且我们研究说中国关系的特色，那这个有太多研究了，我们单独拎出来也没有什么新的东西，但是如果结合起来就有新东西。

黄秋莉： 市场逻辑和专业逻辑相结合。

受访者： 市场逻辑方面，比如论文方面虽然是专业性的评级，比如有3A期刊、2A期刊，然后我们一定要把它对应到一个市场化的评级。就论文本身是一种学术专业产物，我们一定要把它变成金钱的、经济的。它是本质性的，我们一定要外在性的。所以我们又造出来一个市场，让市场逻辑运转。然后市场就是个人利益最大化、追求私利，而学术专业它追求的应该是公益的，所以现在中国自己构建出来一种追求市场逻辑的东西，所以这个就是我们看到的中国特色。而且我们不会反思说把学术领域市场化会有什么问题，我们总是会说这会激励我们这个成绩快速提升，很多学校的管理者都是这样的一个解释。

黄秋莉： 我感觉这还是一种唯科学。

受访者： 这个不是唯科学，这是什么呢？这个就是叫工具理性。你得看一下工具理性跟价值理性的区别是什么。工具理性是指追求的是外部价值，比如世俗的经济价值，我们做这个研究可能很无聊，但是我们也要做，这是因为追求产品带来了这个外部价值、经济价值。但我们做学术本身是不是在追求它能赚多少钱？还是说我个人对这个事情有兴趣，自己觉得对社会公众有义务？这就是异化了，就腐蚀变质了。

黄秋莉： 那老师发现身边的老师是处于一个什么样的状态呢？

受访者： 就不思考嘛，普遍就不思考。也不是说他就跟笨蛋一样，他的思考往往很浅，就依然是没有深入思考，就像他会解释，那现在就是这样，这个行业标准就是这样的。就像刚才问窦老师，我说你评上教授之后要怎么样？他说他不会思考那么远，这就是一种我刚才说的没深度的思考，就他的这个事业的价值导向不清晰。

黄秋莉： 感觉学者在进行外部归因，就是他觉得自己之所以是现在这么一个人，是因为外部环境塑造了我，就是会从结构性的视角来解释自己？

受访者： 也不是，你看他也有自己的选择，有主动性的选择，他说我这样可以更自由一些。好的，梦娜有啥问题？

吕梦娜：之前您不是发过一个链接吗？叫什么狗屁工作。里面萨特提到一句话，说是人一旦闲下来就会思考人生的意义。然后我就在想会不会是我们现在的制度把老师或者还有我们博士生给压的，就是我们就要为了短暂的眼前的目标，然后就不断的去干什么，然后根本没有时间去思考。

包括结合像什么996这种社畜，他们可能心里也会觉得每天就为了生存，或者说为了挣钱在努力，根本没有去想我人生到底有什么更大的追求。然后了，对于我们做科研或者学者来说，也是因为短期的这些小的这些压力，然后促使我们没有精力去关注更长远的一些事情。

受访者： 我觉得你说的这是一方面的外部原因，内部的原因，我觉得重要的还是我们的教育出了问题。就是我们教育体系从来不强调学生应该对于观念世界进行自主性的批判和思考，我们从小就开始达标，去应试去要求达到相应的标准，但是我们并不知道，也没有思考为什么要达到这个标准。即使自己给出了原因，那都很简单，为了考试、为了考过、为了升学，所以我们其实缺乏一种生命的教育。所以其实这个我觉得中国最大的问题就是出在教育体系上，包括我们看到的各种创新不行，包括这个商业伦理水平败坏，包括中国足球呀，有时候我觉得包括中国的治理模式，它都是很多观念层面出了问题。那观念层面最负有责任的就是学者，那学者现在自身也变得不会思考，不愿意思考，不能思考。

吕梦娜：我比较了解同学们的一些想法，可以这么讲，就感觉大家好像对于思辨这个东西并不是特别的擅长。那就是您看您周围的同事、老师们，他们对这种方面的训练是怎么样，就是因为我现在接触不到，我就不知道老师们到底是一个什么样的训练过程，是什么样的老师。

受访者： 就我接触的人来说，像在英国，像我导师、博导他就非常思辨，他总会在一个我忽视的问题面前反复的思考或者是反问，我当时还觉得挺奇怪的，但是现在想想这就是他那种思辨的表达，在中国的同事就是非常理所应当的一种状态，大家不会去深入思考，就没有那种思辨的感觉，大家觉得就是这样。思辨这东西首先难以琢磨，所以我们会觉得这种虚的东西是没有价值，是不值得过问的，那些实实在在的才是有价值，才是应该去关注的。

吕梦娜： 但是很神奇的是，比如说什么答辩之类的，就是类似于这种场合哈，老师们如果做评委的话，他可能就会给出意见，说你这个缺少什么思辨还是缺少批判什么，就他们也会经常提，您对这个怎么看？

受访者： 那我觉得他们是别的问题问不出来了吧。

吕梦娜： 就是会给我一种很割裂的感觉，就好像是老师们受到的教育是那种非常有思辨性，然后批判性那种，我觉得是学术应该有的样子，但是我们所接受的教育了又没有这样，然后导致的问题就是到答辩的时候，老师经常会说我们这方面能力或者是水平达不到，所以我就不知道到底是一个什么样子。

受访者： 我觉得思辨能力就是哲学教育培养出来的，我们又没有这方面的专门的课程或者是对它的重视，那还要求思辨，或者说我们都强调理论贡献，但是其实本质上并没有理论贡献。我觉得他们似乎是对概念已经缺乏一种正确的认知了，对于比如说理论贡献，他都不知道什么是真实的理论贡献，他只是学着别人说而已。

吕梦娜： 然后我今天上午就听了一个讲座嘛，就是南开大学夏军老师的讲座，然后我就忽然发现有个很大不同，因为这个老师是国外的教授嘛，他在讲座之前给分享了三篇文章，然后我以为就跟我们之前上课的时候那种似的，老师只把文章丢给你，然后你自己去看，然后后面讲的内容可能跟文章无关，但是今天我非常惊讶的是他几乎三个小时的时间内一直在围绕他给我们分享的三篇文章，就讲的非常透，我今天感觉就是挺挺震惊的，我觉得应该是这样，就是老师们不要想当然的以为把文章给我们之后我们就能够消化吸收，其实并不是，我们理解的和老师理解的其实是有一个很大的一个差距的。但是我们现在的老师们好像就觉得就只要发给你的，你只要看了，你好像接受的就跟我理解的是一样，但其实是有很大的差距的。然后今天老师给大家分享的时候，我才发现原来就是对同样的一句话，我们的理解竟然是这么的不一样，就还挺震惊的。

受访者： 对，所以就还是我们生活在观念的世界里，那我们要认识世界的观念，还不能够仅仅通过咱们这一套教育的理念，对吧？好多观念在历史中都有不同的定义，历史是一个任人打扮的小姑娘，甚至有很多权力的意图，还有一个学科的不同标准，所以我们有时候学习管理的时候学习理论思想的发展就非常重要，但是我们好多老师会觉得这东西很虚嘛，这个包括他自己也没有接受过这种（理念层面的）教育，上来就是方法论的训练，所以这个问题还挺严重的，也导致我们在管理人的时候就也是想当然，教学的时候也是想当然的，他对于这种真实性是缺乏感知的。

吕梦娜： 在访谈之前我可能会觉得部分老师特别是倾向于量化的老师会对研究本身没有什么兴趣，然后会觉得研究很枯燥那种，但是这几次访谈好像也没有想的那么严重，就感觉大家还是对学术有一点热情，或者说老师您觉得大家说实话了吗？

受访者： 我觉得今天像窦老师说的，他不会对研究有咱们说的那种兴趣，他更多是说我这题目有自主权可以选择，但是他其实有个定义，就是最后90%我这个写都是垃圾。那受访者说的就是他会觉得这事儿有他内在的兴趣，他觉得这事儿本身是有趣的，对，所以也是有不同的。

吕梦娜：之前张宏宇老师给我们上课的时候就跟我们讲做的研究一定要有兴趣，然后包括我们研究动机是怎么样，但是说实话我觉得老师开头讲的和他中间给我们描述的他自己的写作经历和投稿经历，我并没有觉得像他说的那样感兴趣，我觉得是一个非常压抑非常痛苦的一个过程，但是却硬要说这个研究就是有趣的。

受访者： 你这点非常好，你抓住了这个细微的，我觉得这是一个发现点，就是老师在构建一种所谓的研究趣味性，就他在给自己做所谓的思想工作，或者他在做这种自我合理化解释。但是我们看到的是现实和他话语的这种割裂，这是一个我们可以把它提炼出来的发现。包括我翻译的那一篇“废话的胜利”嘛，我觉得实事求是的讲，这个研究它就是没意思，但是我们又总是在说，在教导别人和安慰自己说这个是有趣的，有意义有价值的，所以这就是一个自我洗脑，这个就是sense making，或者说他对于身份构建的一个途径，他觉得自己要有身份感，那这个工作必须得有意义，最起码有趣，虽然没意义，但是还是有趣的。

吕梦娜： 所以这个跟组织管理组织行为学当中的认知失调有点像。

受访者：我们不用“认知失调”这个词，因为这个比较偏心理学，我们还是用sense making或者说就是构建，但其实一个意思，就是其实咱们都知道这讲的就是一个意思。

吕梦娜： 明白，就是好像在他改变不了行为的时候，他就要改变自己的观念，就是我既然选择这条路，然后也没法改变，我不能说我不喜欢这个工作，我必须要说这个工作有趣。

受访者： 对对，就像一个女的爱上渣男，那她还是觉得渣男还有一些好的地方，这是一个意思。

吕梦娜： 对，对这件事进行合理化。

受访者： 那我们就是要具体去看合理化的过程是怎么样的？如何合理化的，这就是我们的一个发现。

吕梦娜： 明白，明白。

受访者： 那你可以再回头看一下我翻译那一篇“废话的胜利”，其实很明确的，我们现在可以打保票的说多数的研究，我可以说90%的研究都是垃圾，都是无意义且无趣的研究，就像富士康工人来交付他的一个工作业绩，他做这个东西完全没有乐趣可言，但是然后我们学者竟然沦为了这种同样的境况，我们需要解释自己的研究还是有趣的，其实就很具有冲突性。就是学者一直在说他是非常自主的，非常追求事情本质价值的，但是我们的访谈以及我们的观察发现事实似乎不是这样的。

吕梦娜： 老师，我昨天初步看了一个social identification相关的理论，核心表达的观点就是如果说这个社会身份遭到了破坏的话，他可能要么就离职走了，就离开了这个所谓的圈子，或者就是努力去改变，但是现在的话我在想我们这个作为老师的话，好像看到这个身份在一点点的被破坏，我们好像都在目睹了他在被市场侵蚀，被行政侵蚀等等，但好像并没有做出有益的举措去改变，他也没有离职，然后我们现在做的就是看他是怎么做的吗？我觉得这好像是一个理论贡献。

受访者： 对，可以，我觉得是可以解释的，这个逻辑是没问题的。

吕梦娜： 那我再深挖一下这个理论，

受访者： 行，我们今天先聊到这儿，录音笔快没电了。

吕梦娜：好嘞，好，好好。

# 受访者22

刘书博老师：大概聊的主题呢就是···因为你本科就是清华，那本科就考上清华是万里挑一

受访者：然而并没有卵用，哈哈哈

刘书博老师：清华它是非常具有引领代表性的一个商学院或者经管学院，所以我们特别想知道你在清华的学习成长过程，你对于中国这个学科有什么感受？也是比较个人发展历史的···

受访者：这么说我感觉题目太大了

刘书博老师：对，我就大概介绍一下，然后我们会有一些细节的问题。

受访者：行，没问题

刘书博老师：第一个细节问题就是你当时为什么选择这个会计专业？是服从调剂还是说自己···

受访者：那倒也不是，因为我最开始我进清华的时候学的是金融，后来我大二的时候遇到我后来的导师嘛，遇到了老板

刘书博老师：所以你本科报志愿是金融？

受访者：对，然后和导师···怎么讲哈哈。我那时候比较年轻

刘书博老师：眉来眼去？

受访者：比较年轻就被忽悠到了哈哈，也不是吧。听我导师讲那一堆东西之后我就发现，可能会计这个学科跟我过去想的那些东西完全不一样。其实说白了是我自己过去书读的少，不了解它，那真正了解之后发现这个学科里面其实很多东西我感兴趣。

刘书博老师：你感兴趣你能举例嘛，就为什么会感兴趣？那不都是数字吗

受访者：其实早期的话我是学文科出身的，后来为什么去了清华不去北大呢？其实我这个人虽然是个学文科的，但我其实理工科这种思维比较强。对，所以我本来其实可能多多少少有点理工科那种偏好，所以就去了清华，那都是经管，你知道咱们那个工商管理学科讲的不好听点，其实说白了就是万金油专业。大家都讲对吧，我们在讲忽悠，讲理论，讲故事，对不对？我发现会计虽然也是管理学科，但至少相对跟数字打交道比较多，对吧？感觉就是比较靠谱。什么东西你都得讲讲数据，对吧？用数字说话。所以这是我最开始的一个动力，我觉得这个东西，就好像一个人，第一的感觉就是觉得这个东西舒服。

刘书博老师：那你为什么高中没有选成理科？

受访者：这就是田忌赛马的故事哈哈哈

刘书博老师：那就是数学特别牛

受访者：对，我原来的老师跟我讲，你要是学理科最多也就去个华科，你要是学文科，可能最差也能去个华科，我想想还不如赌一把哈哈哈。可能就是天生某些科目比较擅长，有些科目就不太擅长。

刘书博老师：可能数学特别好，但是化学或者物理···

受访者：对对对对，理综就不行了

刘书博老师：但是又像你刚才说的，你又特别是理科生的这种，觉得这种数字的比较靠谱。

受访者：对对对对，所以这是最原始的动力嘛，这是一方面。然后呢，碰到我导师之后，我导师人也比较有魅力，然后他又跟我讲这个学科的这些事，我发现跟我想的完全不一样，我就觉得这个东西比较有意思

刘书博老师：那么能具体讲讲为什么被他启发到

受访者：其实最开始的话大家都觉得会计就是个算账的东西，对吧？那东西太没意思了，虽然说有数字，但后来发现这个会计跟金融其实完全就是一块的，那会计更多的就是一种工具，对吧？市场上这么多财务造假，包括这些坑蒙拐骗，你会发现这个东西它不单纯就是为了造假而造假，他背后跟这些上市公司高管掏空上市公司、跟大家发家致富、跟咱们中国资本市场这些年的风云变化等，关系太密切了，对不对？所以你就会发现这些人动不动身家百亿千亿，对不对？动不动携巨款潜逃，那会计在里面又起了一个很大的帮凶作用，但很悲哀的是，其实大部分人都不了解这个东西，所以你不觉得这个东西其实魅力就很大吗，它其实说的不好听点儿就是个武器，就是个工具

刘书博老师：它可以向善，也可以向恶。

受访者：对，你要是用的好，像欧美国家（倒不是说人家天然就好），那因为人家资本市场两三百年，制度已经很完善，所以的话会计在里面就起了一个良性作用。但是在中国的话，因为咱们资本市场1990年开始建立，到今年才30年，所以会计就成为一个相当于一个帮凶，所以这就是为什么一两年就能挣这么多钱，因为发现这个（会计）东西威力太大

刘书博老师：所以当时那个老师也是说这个事情有意义。我们一定要让他向善，而不是向恶。

受访者：对，因为咱们学这个学科，就是想看清这个世界到底是啥样的，对不对？我就发现原来通过会计，资本这个家伙都这么来玩这些游戏，我当时就觉得年轻嘛，哇，那原来世界是这么一回事，不是说简单的动动嘴皮子、说几个故事那么简单，你就发现原来这个东西就像刚才刘老师讲的，你可以把它用的向善，但你也可以把它用的向恶。

那为什么可以这么玩？怎么来避免这个问题？我们要讲道德，君子生财有道，对吧？

刘书博老师：对，所以当时本科你是金融专业。然后你那个时候对于会计这个领域感兴趣

受访者：对，所以我（本科）三年级的时候就转（专业）了，就跟着我导师了

刘书博老师：所以后来也一直很坚定跟着导师在···

受访者：对对对，其实这个学科一直到今天我在上班，我还是感兴趣的

刘书博老师：那还是挺幸运的，因为其实像本科阶段有很多（学生）甚至到研究生阶段都不是很清晰自己未来的道路。那你是就知天命比较早。

受访者：其实也不是知天命，其实就是“你不知道自己喜欢什么东西，但至少知道自己不喜欢什么东西”。把这个东西排除掉，好像感觉也没几条路可以走了哈哈

刘书博老师：但是你还是觉得这个对于你吸引力非常大

受访者：大方向可能有，但就是具体怎么做其实也不敢说自己有了。

刘书博老师：ok，那所以在这个学习过程中有没有遇到···就从被老师启发之后就一直会对它充满热爱，还是说你在这个学习成长过程中会有一些不是那么如意开心的，或者说看到这个学习它是有困难的

受访者：肯定还是有，我觉得一个最大的障碍其实就是后来做选择嘛，你也知道这个读博士，包括读硕士，工作，其实是三条完全不一样的路，对不对？

刘书博老师：对，那你就直接硕博连读了。所以你选择就三个

受访者：要么本科就直接工作了；要么读个硕士再工作；要么就是读博士，三条路

刘书博老师：那就是直接博士了，就本科直接到博士

受访者：一条路走到黑了

刘书博老师：那当时怎么想的

受访者：其实最开始想法很简单，我也没想过做老师。最开始清华有做官的传统，那时候大家博士毕业后基本上就去部委做官了，但政策赶不上变化，十八大之后，这些官员都要基层工作经验，我后来毕业的时候我想想，这喝酒喝不过

刘书博老师：哈哈那你和小利还是可以

受访者：哈哈我们当年八个人本科直博，大家都是这个想法，大家全部去做老师，都在高校

黄秋莉：都是喝不了酒吗？

刘书博老师：他能喝，他谦虚

受访者：在地方的话，这个喝酒的能力不是在学校

刘书博老师：在地方，那不是一个量级

受访者：对对对对对

黄秋莉：怎么感觉很有点儿草率

受访者：这个东西都是命，对，这个说不清的

刘书博老师：而且你本科学习成绩应该也一直不错

受访者：对，成绩还可以，读死书

刘书博老师：谦虚了，我也读死书，肯定考不上清华呀

受访者：殊途同归呀

刘书博老师：那博士阶段这个过程怎么样？你觉得有没有一些非常僵硬的呀···

受访者：其实我感觉好像咱们国内整个管理学科都一样，到今天为止，包括我们会计这样，就是像我们现在在高校做科研，科研的东西跟这个行业、这个市场上实际发生的东西不完全是一样的，这就是实证研究一个很大的问题。

刘书博老师：那这个时候是否跟你当时看到的那样——我们学习还是要让世界改变，变得好起来

受访者：其实我觉得自己的力量还是太渺小了，所以我有时候经常包括现在开玩笑，我总觉得我有点人格分裂吧，尤其给MBA上课的时候，那我们讲的就是真实世界发生的事情，写论文的时候强迫自己切入到另一个状况。然后我也相信别的世界又是这种模式，所以你会感觉这种变化其实太大了，所以做科研的状态跟讲课的状态，就是彻底的两个境界

刘书博老师：讲课还是在讲真实世界？

受访者：对，尤其咱们跟MBA讲课的话，那不讲虚的

刘书博老师：对对

受访者：我觉得这可能就是管理学科整个发展都经历一个很大的问题，我感觉非常明显。

刘书博老师：对。那会计我理解还不像战略。因为你们会计像现实也是很基于事实数字的，那研究也是事实数字的

受访者：其实也不是，我们会计就没有真的东西。我们会计只有假账，没有真账这个说法，所以在会计世界里没有真实这么一说。

刘书博老师：那西方是也这样。

受访者：对，这就是这个学科的本质

刘书博老师：我去

受访者：我们只有假的，知道什么是假的，但我们没有一个真的东西，所以只要不是假的都是真的，那选择有成千上万。

刘书博老师：那像你一直在学界里面，在象牙塔里面学会计，也能够了解外部的那种···嘛

受访者：所以就要出去

刘书博老师：那你出去过吗？

受访者：经常出去，讲讲课，因为我导师他自己已经很资深，他在外面混了很多年，所以我还经常会去听他的课

刘书博老师：ok，所以你可以有跟外部真实世界对接学习的一个机会

受访者：对，就必须要跟外面接触，要不然就是完全理论化了的东西，你说功利点的，你教课都镇不住场子，就像MBA课。说远一点的，就是我刚才讲的——这不是这个世界的全貌，只凭书本上的东西你看不清这个世界到底是咋样的

刘书博老师：那我还是有点不理解，因为我知道像做管理我们经常说它是科学、艺术、手艺，那会计怎么也有手艺感和艺术的，它不就是科学吗

受访者：会计就不是科学。对，我当年大一的时候学会计，我老板教课，他问我们会计是啥？我当时觉得这个东西条条框框，那么多数字，这是科学。等我学了四年下来，我发现这个东西完全就是···因为同样一个公司他的账最简单，净利润大家都知道是啥，我可以说他今年亏了100万，我也可以说赚了100万，都是对的，因为我们会计没有一个绝对标准答案。所以讲的不好听点，天马行空。

那我们会计准则其实主观操作性都特别强，你可以把一个企业做成赚钱，也可以做成不赚钱，这不就是你想怎么玩就怎么玩

刘书博老师：那这个时候像你知道怎么做艺术的会计，那会不会有一些比如说我们会看市场上有很多对于你能力的需求，那你还甘之如饴的天天在办公室埋头搞数据？

受访者：所以这就是老师讲的，只有傻子才去做老师

刘书博老师：那为什么你愿意做傻子呢？

受访者：其实这就是跟人的偏好有关，有的人胆子大，他愿意冒这个险、发这个财。像我就属于风险厌恶型，就不喜欢冒这种风险，也许没那么多钱，但至少日子也过得下去，所以资本市场不就是风险和收益成正比嘛

刘书博老师：是的，而且咱们学生你看毕业都觉得投行是最好的，去了基金证券就感觉特别骄傲，是吧？然后立马毕业之后对于老师就是一种俯视的···

受访者：你从金钱的角度看肯定，对吧？

刘书博老师：对。所以我们在一个世俗的、市场化的世界和社会，有没有感觉这种冲击。就是尤其给MBA上课，那他们会觉得老师你还没我赚的多，你还给我讲这些

受访者：怎么讲？我在读博士的时候这种落差感还特别大。但是都做老师的，这是我导师讲的，“不会赚钱，书读的多也没用”，这个知识就是要转化成这个财富，我觉得这也是这个商学院老师的一个特点，就是自己出去找嘛

刘书博老师：所以说我们还是就像你刚才说的，还得人格分裂

受访者：对

刘书博老师：就标准还是很不一样的，但是也很清晰嘛，就发表研究这个是自成一体的。

受访者：对，就是象牙塔这套模式。这个就说还是要有学术底线。另一方面，在外面，也得知道世界怎么玩，自己也得怎么着。

刘书博老师：对，所以其实你像你们专业我觉得专业性更清晰，它还不像战略，因为战略谁都可以说、谁都可以讲、谁都可以觉得自个儿有战略思维。但会计就不行

受访者：因为会计的实操性比较强、专业性比较强。

刘书博老师：对，专业性比较强，知识密度也比较大。

受访者：对对对。

刘书博老师：是的，而且像商业所谓的时尚概念，都不是来自于会计学，而是来自于人力资源呀或者是组织呀、战略呀

受访者：对对对

刘书博老师：有好多咨询师，有咨询公司的，他们就可以搞这个战略。你们不存在？就是有没有一些咨询公司从你们这个专业会计老师···

受访者：其实会计老师做咨询的倒并不是很多，更多的像独立董事，还有这些专业的培训

刘书博老师：就培训和独立董事

受访者：对，这两块比较多

刘书博老师：ok，所以其实在商学院，我觉得金融会计是更加有一个渠道获取市场资源、经济资源

受访者：这是客观事实

刘书博老师：所以你们就很有话语权嘛，而且我们发现国内商学院很多院长都是会计背景。

受访者：对，因为客观来讲，我一直觉得，这倒不是其他学科不行，就说因为商学院毕竟跟这个市场打交道也比较现实了，金融会计的话，它相对来说能从市场上获得更多的资源，所以这两个学科就相对来说能带来钱，那自然就相对而言话语权可能较强。

刘书博老师：对。是这样。那现在就是我们再回到这个象牙塔内部的一个学术研究，是不是我们完全没有中国特色的研究标准。

受访者：其实我觉得这些年好像这个趋势慢慢扭转过来了，大家开始讲有中国特色的故事了，对吧？像实证研究十几年前的话，基本上就是国外做了个什么东西，照搬国外的东西跑一遍，然后把国外理论拿进来，狗屁不通，那这些年呢我们做研究，我们也讲接地气了。

刘书博老师：Ok

受访者：但这其实又出现了一个分裂，就说我们发中文的论文跟发英文的论文，大家讲故事又是完全不一样的，对吧？在国内，你现在要是生搬硬套，那大家就不会买账，因为我们要接地气，但是在国外，核心的目的是要讲且让外国人听得懂。

刘书博老师：对，所以会计这个领域还是以美国为老大是吧

受访者：对，严格意义上还是以美国为老大。但其实会计这个学科也比较特别，因为它有点像政治制度，每个国家特点不一样

刘书博老师：它有点像政治经济学

受访者：所以你看全世界就说你愿意这么玩，那肯定就是美国老大，但有的国家不玩这个，比如像日本，日本的会计就从来不搞这些paper。所以你会发现我们做学术研究，日本人从来不搞这套

刘书博老师：对，日本，然后像北欧···

受访者：欧洲国家好像也不是有那么样的感觉

刘书博老师：英国比较接近美国，但是北欧就有点不一样

受访者：对，相对来说，欧洲的氛围还没有中国的强

刘书博老师：所以中国还是比欧洲更像美国

受访者： 对，所以其实这就是，嘴上说一套，行动很诚实，美国人的任何东西我们都在向他学习

刘书博老师：对，这一点我看也是，战略领域尤其是这样。而且有没有说会计像香港、新加坡那些地方，因为这两个地区似乎在影响大陆

受访者：对对对，格外强

刘书博老师：比我们还强？

受访者：其实国外发这些文章，说白了也是要做圈子，对吧，那我们现在找co-author基本上主要也就是通过香港、新加坡

刘书博老师：对，然后他们再找到美国

受访者：对，但我觉得国内相当于现在形成了一个默契，像我们会计这个圈子，顶刊大家都要搞，但是呢搞个一两篇呢，基本上大家就不搞了，相当于这就是一个叫什么sample···我们没有譬如说你五篇一定比我三篇厉害，你有一个就行啦，对，点到为止，说明你有这个水平，相当于现在就形成了这么一个潜规则

刘书博老师：那这个潜规则我觉得挺合理的

受访者：是，合理，其实也很正常嘛。因为你看国外的期刊，美国的期刊，人家更多的关系是自己国家的，就是你中国的期刊，谁没事发个印度的东西，那也不太现实，对不对？

刘书博老师：是的，那就是会计标准其实也深受文化社会的影响

受访者： 对对对，每个国家其实都不一样，哪怕我们跟香港其实都有差别

刘书博老师：为什么呢？因为立法不一样嘛

受访者：其实立法、执法、司法整个体系完全不一样。

刘书博老师：ok，因为我理解像战略这种，它特别受到文化的影响。会计似乎会比较标准知识

受访者：就像我刚才讲的，因为会计没有一个绝对的真实的东西，所以每个国家只要差一点，最后结果就完全不一样

刘书博老师：而且执行的过程也非常不一样

受访者：对对对对，其实跟你们战略差不多，大管理都是这样子的。

刘书博老师：对，那就挺像的，而且尤其是这个新加坡、香港他们就有点像洋买办的，然后把他们的一个逻辑标准···而且不仅在这个市场领域，在学术领域也是这样。

受访者：对对对

刘书博老师：那他们现在香港如果发一篇两篇还不行？

受访者：对，他们是拼数量

刘书博老师：他们还得拼数

受访者：对

刘书博老师：那咱们还是幸运一点

受访者：可能相对幸运一点点，但不知道未来的趋势是怎么样

刘书博老师：未来不是很明确

受访者：对对对

刘书博老师：那你不觉得说中央他们现在要把研究做在中国大地上，这个会牵引过来吗？还是说你不看好它

受访者：其实我觉得方向挺好的，思想也挺好，但关键就是落地这一块，政策执行该怎么做，否则就会沦落成那种境界——大家都知道是不好，但好像没有更好的办法

刘书博老师：就有点像制度惯性

受访者：对对对对

刘书博老师：组织惯性

受访者：对。否则你怎么来评判一个老师谁的水平高、谁的水平低。好像没有一个替代的方法。这个就比较麻烦。

刘书博老师：对，是的。那现在呢就是其实学科之间、专业之间，不好相互之间评比，只能是这个专业内部的。

受访者：对对对

刘书博老师：而且有时候咱们做的论文似乎那种思想性价值也不能够立刻凸显出来。

受访者：对对对

刘书博老师：那怎么评？那就很难评了

受访者：是，所以就只能看数量了

刘书博老师：ok，那现在也有一个趋势说要重教学、轻科研，你觉得这个···

受访者：其实我觉得大趋势是好的，我个人也很赞同作为老师本来就应该教书育人，这应该是核心的价值，咱们过去也是这么做。就是咱们中了美国人的魔道

刘书博老师：你有感觉这样一个变化吗？就是我们越来越轻教学、重科研，你上清华大一那时候是不是这样？

受访者：我觉得清华和北大是最早的重科研轻教学，因为他们最早引入tenure-track，你想想一个人有了tenure-track之后，他怎么能够把这个心思花在教学上

刘书博老师：对呀，他要生存饭碗。

受访者：对，先得要生存下来才行。

刘书博老师：对，所以你去的时候应该还没有吧？

受访者：有啊，清华不是向来就有这个说法嘛，一流的学生，二流的专业，三流的老师。

刘书博老师：所以你是2008年入学清华的，那确实，2005年（重科研轻教学）应该就开始了。所以那个时候你去了之后，老师们都已经开始重科研轻教学了。

受访者：对，我觉得这个非常明显，这当然不是说老师不好，就说能够感觉的出来，所以大家教完课基本上很难找得到人

刘书博老师：那你从毕业之前就一直是这样，有没有一些···

受访者：我觉得好像这个趋势越来越明显

刘书博老师：越来越明显？

受访者：对，所以在清华基本上就都是靠自己嘛

刘书博老师：ok，但是清华应该对于老师的收入水平是有所保障的，清华老师应该收入还是挺突出的

受访者：压力跟收入是成正比的

刘书博老师：ok，能赶上新加坡、香港吗？

受访者：赶不上，那新加坡、香港要求更高

刘书博老师：对，那个更变态，那个顶刊···

受访者：这个东西倒没啥说的，对吧，这叫干多少活拿多少钱，对不对？我跟老大也这么说，我说你一年给我开一个亿，我一个月发顶刊我也做得到

刘书博老师：一个月做得到？

受访者：钱到位了，有什么做不到？哈哈

刘书博老师：那咋做呀？贷款做吗

受访者：我很想说真到了这个份上，豁出去了

刘书博老师：你带一批箱子到哈佛找合作者哈哈

受访者：这倒不是这么赤裸裸，可能就是真到这个份上也没啥说的。

刘书博老师：那就比如说有一些老师，那他是否有一些先天的优势资源可以让他在这个发表上有···

受访者：那肯定，你不觉得干咱们这一行的其实也讲圈子吗

刘书博老师：也是圈子

受访者：对，其实圈子文化很明显，在国外也一样。

刘书博老师：对，就是师门传承的

受访者：在国内我觉得更明显，我不知道管理学科怎么样，会计学科这个小圈子主义特别神奇。

刘书博老师：那国内是一个圈，他跟国外还不是一个圈子？

受访者：还不是一个圈子，

刘书博老师：对。所以似乎我们国内的圈子会把持着国内的核心期刊。

受访者：对

刘书博老师：那国内的圈子，比如说想发顶刊，那怎么办？

受访者：强强联合嘛

刘书博老师：互相联合

受访者：对，互相资源输送嘛

刘书博老师：交换一下

受访者：对呀

刘书博老师：那你看其实像我这样一直在国外念，我就很傻逼了

受访者：那不一样，我觉得高校老师有个好处，至少他包容性还挺强。对吧？你想就是专门专注于科研这条路确实很难，但你就是对这个东西没那么感兴趣，至少也不会有生存问题

刘书博老师：那清华就不行。

受访者：那清华因为有个tenure-track在，你至少在前期必须要活下来才行。

刘书博老师：不是tenure在中国所有高校都有嘛，我不知道清华是所有学院都这样，还是···

受访者：所有学院

刘书博老师：那这个tenure似乎也不是像美国那样，它都是有tenure这个制度，它是一个全国性的，中国就似乎只是清华北大

受访者：那现在不是慢慢推广嘛，咱们现在不也是参与进来了嘛

刘书博老师：对，我们就是新人新办法

受访者：对，咱们新人新办法，咱们是2019年开始搞的

刘书博老师：ok，那你觉得这个制度变化怎么样？是好呢？还是说利大于弊呀，还是···

受访者：呀，这个东西真难讲，反正我们不是受害者，我站着说话不腰疼

刘书博老师：对，我们不在这个权杖之下，我们应该不是这个新人新办法

受访者：你要是单纯的就说像过去，管理学讲这种工业生产，泰勒这种思想，从效率的角度来讲，当然tenure-track就是好的，对吧？但是好像从生活的质量来讲，tenure肯定还是不太好，所以我也经常跟他们讲，你到高校里做老师你追求个啥？本身说来也不是图个钱。你就是tenure了给你加了点工资，你到外面去做啥拿不到？

刘书博老师：对呀对呀，压力还那么大

受访者：其实更多的就是在高校里感觉生活过的比较好，如果连这个东西都没有了，你不觉得这种选择的性价比就不是那么高了嘛，但是这是我个人的想法。

刘书博老师：对，所以我跟他们也说，他们未来也会面临的这个tenure，好像现在就越来越普及了，一线城市的985、211几乎都是这样。

受访者：对对对，但是这个tenure也得看高和低吧。有的学校也其实要求不那么高

刘书博老师：也是有，他也具体看发几篇、发到哪儿

受访者：对对对

刘书博老师：那现在好的研究和不好的研究是不是像泰勒那种他会对于学术质量有所影响。比如说发文章，如果是泰勒制，似乎它更多是标准化的生产。

受访者：对呀

刘书博老师：那学术能够以这种标准化生产，那似乎是不是对他质量···

受访者：那肯定。所以99.9%都是垃圾，是我老板讲的，我自己写的东西我基本上觉得都是垃圾，我说的很直白哈，所以我很羡慕他们发顶刊的。但是另一方面我知道他们发的也都是垃圾，但这并不说明我不羡慕他，我也很羡慕他，有机会我也想发

刘书博老师：对，就顶刊还是有好的，但是似乎···

受访者：也不能说顶刊都是好的，只能说顶刊好的概率相对高那么一点点

刘书博老师：那他们的好表现在似乎是有一些思想创新？

受访者：其实倒也不是，其实也是圈子嘛，你像你在美国读的书，你发的期刊不就是顶刊吗？我们在中国读的书，我们发的期刊···只是我们人为地把人家这个标准定的比我们高而已。它其实就是个圈子，如果你在美国读博士你也能发

刘书博老师：对，我是觉得这个还是有很多人为构建的···

受访者：对，但是不得不承认，顶刊相对而言我觉得研究质量还是高，不能说思想好，他要求高

刘书博老师：严谨

受访者：严谨，对

刘书博老师：详实

受访者：详实，对，我觉得这个规范性还是比国内的要好，这个还是得承认。

刘书博老师：对，没错，那就是真正像理论创新，似乎没有，有知识层面的一些跟进。

受访者：对，所以就是我们今天开玩笑讲的，感觉自己很牛逼，但会计领域你能说出几个人属于这种很有思想的？说不出，几乎没有

刘书博老师：那比如说在学术这个圈子里面，发展的这个标准就是数量。

受访者：对，其实现在基本上说白了，就是数量，质量其实也得有数量，对吧

刘书博老师：对。ok，好，那你有什么职业上的、学术上的一个定位，或者对未来的一个期许嘛

受访者： 这个东西不就是打怪升级，多多益善嘛。

刘书博老师： 多多益善，就按照标准尽量的去做的更好。

受访者： 对呀，因为就是其实前期对吧。咱们也一样，虽然咱们没有tenure-track，但还是有职称压力在，对不对？说到底就还是得升级打怪。

刘书博老师： 对，还得把职称这个怪给打掉。

受访者： 对，所以就像你们主任一样，对吧。得上岸了才摆脱低级趣味，那这个时候就可以追求人的高级趣味。

刘书博老师： 我就想知道咱们前面一直是这种标准化的工作，就打怪了，那一旦上岸了就是评上了教授，那这个再往后的阶段你有什么想法？

受访者： 还真没有想这么远，先打怪过去再说。

刘书博老师： 那你觉得有没有身边的一些老师他们在上岸之后有不同的活法？

受访者： 挺多的呀，你像我老板，我一个大老板，他现在就觉得学术都是bullshit，但同时他又觉得这个东西对年轻人来说是必须要达到的，他也跟我这么讲。

刘书博老师： 好分裂，就是你们知道是bullshit但一定还要去做这个bullshit。

受访者： 对，所以他就跟我们说要做老师必须要能分裂，我现在自己的体会也就是觉得“我好分裂”。

刘书博老师： 那就学者特别分裂，其他行业会好一些？

受访者：其他行业我觉得也分裂吧，也分裂。

刘书博老师：对，所以是说咱们特别分裂，一方面他能够把这个学术贬的那么低，然后又觉得你必须得（这样做），没有别的办法。

受访者： 我们这种分裂其实严格意义上还算是一种君子的行为，对不对？只是说这种想问题的角度不一样，那别的行业那是人格分裂、道德分裂，对吧？一边道貌岸然，一边阴险狡诈，那个更恶心。

刘书博老师： 那现在有没有觉得就比如说市场化的一些东西会影响我们学界的一些，比如说我们可能一开始安贫乐道也可以。

受访者： 我觉得咱们商学院没有人能做到“安贫乐道”，理工科学校可能可以。

刘书博老师： 为啥？

受访者： 因为理工科研究自然科学，他们有的时候就必须要沉下心做这些东西，对不对？商学院本身就是跟金钱打交道的，对吧？就是我跟我的学生讲的，我说你们来学商学院，你们的目的就是来挣钱的，你不是冲着挣钱的目的你也不会上商学院，对吧？既然是想挣钱，你就会觉得世俗观念、城市观念很重的，这波人怎么能做到各安平乐道？

刘书博老师： 对，商学院嘛，就是教人做生意的，对不对？教人怎么管理做企业。但是似乎我们的研究又不是特别能够帮到他们，是不是又有一个分裂的感觉？

受访者： 对，所以我们的研究没几个人看。

刘书博老师： 假设咱们不去管发表，那就是为了帮他们赚钱而去做研究，这样可以不？

受访者： 我觉得这样也不好，这样也不好。

刘书博老师：为什么？

受访者： 首先第一个就是作为老师你对市场接触不多，首先你做不到，对吧？第二个你与其帮别人赚钱，为什么自己不挣钱？所以没这个动力，对吧？没这个本事，没这个动力，那怎么去做这个事情？

刘书博老师： 所以还是要做理论研究。或者说我们就是成立一个小咨询公司，然后帮他们去解决一个他们具体的会计制度问题什么的呢？

受访者： 对呀，那你成立公司最后的目的还是你自己挣钱。

刘书博老师： ok，所以就很矛盾。那你有没有觉得现在生活和工作这个边界不是很明确，就大家总是生活的时候也在工作？

受访者： 对，我觉得这就是老师的特点，就是一方面咱们时间确实多，，但另一方面就是咱们随时随地也可能来事情。

刘书博老师： 你觉得清华的管理跟中财的管理是一样的吗？

受访者： 管理还是差蛮多的，差蛮多的。

刘书博老师： 那有什么具体的差别吗？

受访者： 就中财和清华，我觉得最明显就是行政性事务上清华真的少，可能在这里待过你觉得好像也没多少，但是如果仔细算起来还是挺多的。

刘书博老师： 还有就是我们学校管理似乎跟国外也很大不同？

受访者： 我觉得咱们整体好像“比上不足，比下有余”，大概就这样。

刘书博老师： 对，咱们学院还是相对比较规范。

受访者： 我觉得咱们就是很正规的、很传统的这种中国式的大学。你在爱丁堡待过，他们应该几乎没什么行政。

刘书博老师：是的，就没什么行政。

受访者： 对呀，我原来在清华的时候老师都抱怨说行政事物好多，等我来到这里，我发现那行政事物还算多吗？

刘书博老师： 我昨天还忘了上课，本来3点15就要上课了，但是3点我还在家，而且那课是交大附的课，还要给他们上这种课，所以你看我们这个时间完全的就不是说很被尊重，就我们有时候要做一些很荒诞的事情。

刘书博老师： 行，那你们这边有什么问题吗？

黄秋莉： 老师，您能不能给我具体讲讲行政事物？

受访者： 比如说监考、各种“文山会海”、要写很多材料、各种会议、各种培训……

黄秋莉： 那不参加不行吗？

受访者： 当老大亲自给你打电话的时候你不参加不行啊。

刘书博老师：可以呀，反正不是给我打，哈哈。

受访者：就是拒绝会带来一种内心的压力，你可以拒绝一次两次，但你总是拒绝其实也不太好。

黄秋莉： 确实，人和人之间的关系总还是要讲究一点。

黄秋莉： 那您当初其实首先排除去政界了，那业界呢？您是觉得风险太高了吗？

受访者： 其实也不是，我其实到最后一刻我才下的决心去学校的。我开始也拿了一堆外面的offer，外面钱很多，但后来没办法，因为我太太是做医生的，如果两个人都在外面折腾的话，这个家就基本上废掉了。所以考虑到家庭分工。

刘书博老师： 是什么外科医生？

受访者：北医三院。

刘书博老师：那么好的医院我怎么早不知道，我找你去。

受访者：所以这就是说比较现实的原因，差不多就这个状态。

黄秋莉： 那您觉得在学校就是因为这种时间安排比较自由，还有没有考虑别的因素呀，就你觉得在高校工作有哪些幸福来源？其实我也是想着为我以后的这种工作了解了解。

受访者： 在学校工作的好处大家不都看到吗？我觉得这个时间非常自由，就这时间你可以做任何事情。全世界还有什么工作比得上在大学做老师好？

黄秋莉： 以前也是这么觉得好自由，但我现在自己读书，我就觉得人家在外面好歹是996，我觉得咱们这是007呀。

受访者： 没有，就是说自己要学会调节，对吧？你想想其实讲的不好听点，我们讲的在大学八年抗战你不就实现时间自由了吗，对不对？三年的副高，五年的正高。你说在哪个行业能有这么好的这个？而且完全是无风险，对吧？非常稳定。

刘书博老师： 但是这种专业知识密集性的这种就有点像医生和律师，就越往后越好。

受访者： 对，我们是自主性越往后越强。至于钱这种东西，那就看自己的本事了。

黄秋莉： 那您现在是除了在学校的工作以外，有没有去社会上去做一些社会服务之类。

受访者：那为什么不做？

黄秋莉：那您觉得自己这个教学、科研和校外这种服务的话这种时间大概是怎么个比例？

受访者： 这个纯粹的比例也不好说，但我还没有到那种senior，这几年我很junior，但我感觉看到别人这个状态，就说那些做的比较好的老师状态，你会发现如果说你协调的好，这几块之间你慢慢就可以形成一个很好的良性循环，各块之间相互促进，基本上就不会就说会有捉襟见肘的感觉，就基本上不会出现这种状况，它会形成一个非常好的融合的状态。

刘书博老师： 对，是一个健康的互相促进的状态。

受访者： 对，但那种状态可能就得摸索，对吧？但至少就说如果你认真观察就能够发现，还是很多人能做到。

黄秋莉： 除了这种时间上的因素，研究上的自主是不是您考虑的一个重要方向？

受访者： 其实研究上，我说实话都谈不上自主，但就是说我不知道你们其他学科怎么样，但是像我们会计的风格，那就研究现实问题嘛。就是所谓的热点嘛，对不对？我觉得就是说咱们研究不能拍脑袋决定要做什么研究，你研究现现实中正在发生的这些事情。那我觉得，研究本来就这么无聊了，还不如研究点对社会有价值的东西、有意思的东西。那谁想看这玩意儿，对不对？我觉得这个这也不能叫自主吧，只能说自己要研究什么话题还是有选择权的。

黄秋莉： 那您是会从这种现象驱动来进行你的这种研究的选题。

受访者： 对。

黄秋莉： 那研究的过程中想着要除了考虑这种热点呀，还有没有别的需要考虑的因素？

受访者： 别的考量倒没有，其实我觉得我做博士期间，其实在科研成果上还是遇到挺多挫折，但至少有一点好，我在博士期间练就了那种自我造血的能力，因为其实我知道很多人相当于就是过去跟着老板混，“一人得道，鸡犬升天”那种感觉，一旦自己玩的时候很多东西就玩不转了，我觉得我那几年就是这个好，就是可能当时我搞那些东西，有些地方受了挫折，但是这个造血能力自己有了，所以就不怕，就手上有门手艺了，你也许不太可能发顶刊，但是活下来就是绰绰有余，那有了这个东西那就没有啥可怕的了。

黄秋莉： 自己能力在博士期间得到了锻炼。

受访者： 对呀。

刘书博老师： 而且会计学院似乎整体来说比商学院都要做的好一些？

受访者： 对，他们那个激励制度我觉得可能比咱们这里更强一点，但他们压力比咱们大多了。对，所以我也觉得这没啥问题。

黄秋莉： 看来您是非常喜欢在学校这种工作的氛围哈。

受访者： 可能我也不敢说我喜欢，我只能说据我自己过去体验的东西，（进入学术界）这个选择可能（不是十全十美），说实话，我经常自己也觉得很烦。那（进入学术界）跟其他的比起来，可能就说我如果在其他的环境可能更烦？

黄秋莉： 您现在是觉得咱们的这种工作状态还有改进的地方吗？除了“文山会海”让你觉得比较繁琐以外。

受访者： 其实我觉得做老师尤其科研遇到挫折的时候会出现这种负面情绪，有时候经常很烦。

黄秋莉：我还以为老师们就是会非常的快乐。

受访者： 这种东西就看你看不看得开，你要是能看开，你完全无所谓，对不对？你要是看不开的时候就会很烦，比如说你一天收十份拒稿的时候，就让你觉得比较烦。

刘书博老师：我也是，就感觉想把电脑砸了，哈哈。

受访者： 这种东西就看你自己有没有追求，其实你要是没追求，其实完全没压力。因为已经评上副高了，所以我们没有生存压力，更多的就是发展方面的压力，这属于自己给自己的一种压力，对吧？但我就觉得这个至少是个好事，可以促进你更快的发展。退1万步来讲，你觉得自己不干了，总是有碗饭吃。但就说你想前进的时候，可能确实会感受到比较大的压力。

黄秋莉： 自己的上进心带来了的压力。

受访者： 对，有时候很烦、很焦虑，想死的心情也是会有的。

刘书博老师： 就我会经常收到拒信，然后拒信内容看了之后我都想骂人。

黄秋莉： 科研当中的挫折很折磨人，就跟我们写不出论文是一样的那种感觉。

受访者： 包括生活中，因为中国是个人情社会嘛，有人的地方其实都有江湖，这个也会带来一些烦恼，只是我觉得在大学里面相对外面肯定已经算是好多了，相对纯粹一些。

黄秋莉： 因为您觉得这个我们生活在这个学校嘛，在这个学校工作你会觉得面临一些人情关系的困扰，那这种困扰会不会影响到您自己做研究？

受访者： 当然也会，而且有时候还挺严重，这个东西好像不太好说出来，那你将来干这个你就能知道，这个叫只可意会不可言传，你慢慢就能体现出来。但归根到底还是那句话，就是看你自己怎么想的，你要看得开。你看我一位师姐就看得很开，毫无压力，有时候我超羡慕她。

黄秋莉： 您自己觉得自己还是比较分裂了，就可能还是有自己的一个原则性的东西。

受访者： 对，这个就得自己看。

黄秋莉： 那您在这个学术界有没有见到过一些不太好的现象？

受访者： 非常多，所以我一直说学校是相对好一些，但肯定有不合理的地方，咱们每个学科都差不多，对各种龌龊的都有很正常。

黄秋莉： 可能我就没有工作过，就对这种感触不是很深。

受访者： 这很正常，我读博士的时候其实我也没什么感觉，我现在工作才三四年，其实好多东西也见到不少。

刘书博老师： 行，梦娜有没有问题？

吕梦娜：有一个问题我不知道是我没听清楚，还是老师太谦虚，就您评价自己写的文章90%以上都是垃圾嘛

刘书博老师： 我们私下内这么说的，到时你不能这么宣传。

吕梦娜： 我主要很好奇，就是您在写这样的文章的过程中您是一个怎么样的心路历程。我就是之前可能会有一些不太喜欢的研究，但是又不得不做，那种感觉我就是心里很难受很难受，我不知道该怎么去处理这种情绪，我想知道您写这样的论文当时是怎么想的。

受访者： 其实我感觉有些东西让我写出来我也觉得价值不大，我倒是很少遇到说写自己不喜欢的东西，我写的东西都是我自己选的，这个问题倒还不大，我的问题主要是觉得有些东西意义不大，更多的就是觉得从实践的角度来讲可能跟现实社会不是那么相符，我只能这么说，就是很书本化、学院化的那一套东西。

吕梦娜： 就是这个研究您本身是感兴趣的，对吧？

受访者： 对，这个大方向基本上都是我自己选择的。我觉得过去我在清华读书就有这个特点，清华的话像我们导师基本上都是放养的状态，他不会给你方向、也不会管的那么严，都是自生自灭，所以我经常骂有些博士，我说你得亏在这，你要是去清华那你绝对就挂掉了。

刘书博老师： 所以清华导师是不太管学生的。

吕梦娜： 老师您刚刚也提到了，说是工作的时候可能会有一些不太愉快的事情，比如说一些不太好的事情，您之前有想过放弃吗？比如说放弃读博或者放弃当大学老师，有这种念头没有？

受访者： 放弃读博我倒是从来没想过，但是放弃做大学老师倒是动摇过好几次。但其实当时放弃做老师倒不是因为这些不好，就是觉得外面赚钱赚的更多，这个很现实。

吕梦娜： 那有没有内心的什么信念又把您拉回到做老师这块，就是让您不去放弃了

受访者： 其实我觉得反正我当年读博下来可能相对来说还算比较顺吧，至少毕业上这些东西都没有太大的问题，其实到最后说白了就说哪条路可能对自己将来发展更好，其实都还不错，只是说哪个更好而已。所以可能更多的纠结是在这里。读博倒是一直没有纠结，但最后可能就说看想选择哪一种生活方式。

吕梦娜： 我之前听同学说您讲课特别有激情，我就想说你对于大学老师这种身份会给您带来一些什么样的主观感受呢？

受访者： 你看周围教授，你就会发现大学老师这个身份挺好，你就说不管你是跟当官的还是做生意在一起，也许你赚的没他多，权力没他大，但你大学教授这个身份其实很超脱的，对不对？那就说人家怎么着，其实都会多多少少给你一份薄面，所以我觉得有这么一点好处。

刘书博老师： 对。

吕梦娜： 因为昨天我正好看一篇文章，就是说现在大学老师好像都变成了“计件工”，您怎么看？

受访者： 年轻的时候肯定都得经历过这个状态，我觉得这个很正常。

吕梦娜： 你觉得这个年轻的时候很正常？

受访者： 就像小元老师一样，等你自己过了这个阶段之后你会发现就很开阔了，选择就很多了，就是说这个计件工这种状态，我觉得哪行哪业都一样，你做金融你也得从金融民工开始做，你去机关里面做政府职员，你也得从端茶倒水开始，我倒是觉得这个问题没有啥，都得要这么来。

吕梦娜： 但是现在其实有很多年轻的老师可能就是刚入职的这几年，尤其是当面临非升即走这种压力的时候，就可能真的太心累了。但取得了阶段性的一个成果之后，比如评上副教授之后，可能就选择躺平，您对此怎么看待？

受访者： 我觉得挺好的，就关键是自己想清楚自己想要什么东西就行了，对吧？你想清楚你要什么东西就行了，比如说你想要一个很舒服的生活状态，但你的内心又割舍不下那些什么荣誉呀、职称，那这不是自己跟自己过意不去嘛，对不对？就说你想清楚自己要什么就无所谓了。

吕梦娜： 您之前提到那个打怪升级嘛，这个怪可能是职称，然后就是这个无论副教授还是教授，您觉得更多是外界给您的这种推力让您去争取还是内心的想法说我就是想要，您更多基于什么样的考虑？

受访者： 我说句实话，其实主要是外面的，其实对自己来讲，你说评个副教授、教授他工资也没加几毛钱，对不对？但关键就是副教授、教授好像就观感不太一样，这说白了其实还是外界给的，但是你活在这个世界里面，你逃不掉这些东西，对吧？

吕梦娜：那您现在属于上岸这种状态了是不是？

受访者： 显然不是，评上教授才是上岸的这种状态。

刘书博老师： 小元老师就是教授。

吕梦娜： 那您是不是还得再打怪升级？

受访者： 我现在属于跟着书博老师谋求发展，哈哈哈哈。

刘书博老师： 我得到时候跟着老师多发几篇才可以，我这个研究范式速度就不行。

受访者： 刘老师做的是最有学问的东西。

刘书博老师： 就是做的慢呗，哈哈。

受访者： 这才是精华，对不对？我们这是快餐，精华才是最有营养的。说实话，我觉得做案例挺好的。

黄秋莉： 您自己觉得会计研究是不是有主流研究和非主流研究的区分？

受访者： 你所说的主流和非主流是什么意思？

黄秋莉：比如可能会觉得实证是主流，然后案例研究的话就会比较边缘一点。那在会计研究领域关于什么研究视角、研究话题有没有这种区分？

受访者： 其实就是话题本身的，也不能叫主流或不主流，可能更多就是看有没有在这个热度上，也就是是不是所谓的“热点”，对吧？

黄秋莉： 为什么咱们会有这种对热点的追逐？

受访者： 其实是因为现象驱动。现象驱动的情况下，一方面就是看看这个世界真实发生的事，另外就是大家已经做了啥，比如说我们会计领域内的政治关联，十几年前大家已经开始做了，并且做了成千上万个了，你现在再做就是炒冷饭，就不是热度了。

黄秋莉： 是不是研究前沿的这种区分。

受访者： 对，其实你说方法这个东西我倒是不觉得存在主流或不主流的区分，其实我个人觉得，实证都是我们这种小年轻做的，现在会计里面真正的大牛他们就是做案例。我们做案例研究也不是不可以，但我们达不到那个深度，这是个很现实的东西。大牛做案例研究有这个关系和资源，知道这个背后真的发生了什么事情。

黄秋莉： 所以那可不可以理解为就是说所有你能看到的现象都是可以研究的东西？

受访者： 我觉得是这样的，我觉得其实大家研究的东西都差不多，但我为什么说顶刊的研究质量确实还是高，你会发现那些人确实还是会讲故事，你发现现实中发生的一些什么新玩意，他们速度就很快，很快就弄出来，其实仔细思考一下，他们做的方法也就那么回事，但人家能够套个很漂亮的外套，这不得不承认，还确实是本事，这就是一个手艺。

刘书博老师： 他必须得经过前期的那种苦功夫，就跟做医生临床，你在前面就得反复的去扎针什么的，不然你当不好的医生。所以其实像博士早期，我觉得文献阅读写作是必须得有，不管是读paper还是读书，都得有一个学习的密度。

受访者： 这个过程虽然枯燥，但还是必须要经历。

刘书博老师： 必须经历，因为没有这个刻苦的过程东西出不来，有时候他刻苦读过之后，他那种理解似乎就接近一点。

受访者： 而且学术科研本来就是八股文嘛，读多了才能知道怎么写。其实咱们管理学的又不需要那么多天赋，只要足够努力。

刘书博老师： 所以其实我觉得读博士读的好的都是像郭靖那种，他不是说有多聪明，越聪明越做不下来，就会跳来跳去的，很灵活的。但是郭靖他能就扎进去，他很憨直，所以他能练出来降龙十八掌，关键坐得住，耐得下心思。 所以我觉得做学术不是说最聪明的能够做最好。尤其咱们社科又不像自然科学，那个就得天赋

受访者： 你看我们会计学科现在最牛的这三个人，一个是浙江工业大学出来的，一个是浙江财经大学的，还有一个是石河子大学出来的，本身都不是最好的学校出来的，那这三个人是我们国内会计领域做的最好的，顶刊发的最多。你会发现这个东西真的跟那个没多大关系。反正我是服气，我达不到他那个境界。

刘书博老师： 对，我感觉就是磨、磨、磨……时间到了它自然就有。所以这个东西必须还是得经过时间磨练。

吕梦娜： 可是咱们现在不是三年一评、五年一评，这似乎也没有给那么多时间去沉淀积累。

受访者： 这种东西吧，一方面就是看自己努力，另一方面确实就是机遇也很重要，运气很重要。当然你还太年轻了，不能跟你说这话……

刘书博老师： 就像这个考到清华这个完全是智力，考上清华遇到一个好老师，这就是运气。老师会指导，但是一定自己的基础还得有，两个结合才能产生结果。

受访者： 所以你们（学生）这样挺好的，把刘老师那一套都学会，这样就可以自立门户。

刘书博老师： 我们也是另辟蹊径的走一走，其实现在实证研究也挺红海的，现在大家都是就精细的概念在做。

受访者： 所以绝大部分都在瞎折腾这玩意儿。管他有的没的，就是只要数据显著然后就码字。所以有时候我想可能似乎做案例能够更接近现象，我跟你说句实话，就咱们先不讲功利性的东西，我是真正的觉得做案例是最有水平的。

刘书博老师： 反正工商管理战略在英国他们70%都是案例，我们国内正好颠倒过来。

受访者：其实我就觉得咱们中国人这点不太好，但日本人就很务实，讲这些垃圾玩意有啥用？你看包括清华也是一样，那早期那些老师都能给MBA教课，那现在讲课都必须要从外面找人，你说现在这班老师发论文是不是一个个都很牛，但你知道他们给MBA讲课能讲出啥，都不知道这个世界是啥东西，所以经常被轰下台，出了好几次这个事了。所以现在清华都是业界请人，幸好清华的校友资源多。

刘书博老师： 那也挺尴尬的。

受访者：是很尴尬，但没办法。

刘书博老师： 那现在清华有对这事儿有动作吗？还是就一直这样采用雇佣军的方式？

受访者： 雇佣军的方式嘛，基本上国外也这样，北美商学院也是这样。清华的副院长都被轰下台了。

刘书博老师： 反正我知道中欧他们就是要求老师必须得讲，要不行就淘汰就走。研究再好，讲课不行也得走。

受访者： 对，因为中欧的定位跟清华可能还是有点差异。

刘书博老师： ok，清华原来是这样。

受访者： 所以你会发现基本上现在讲课雇佣军的比例是最大的。

刘书博老师： 对，明白，所以最后就把清华本校老师都逼成专业的研究员了。

受访者： 对，我看基本上就是这个态势。咱们不也这个趋势吗？

刘书博老师：咱们有吗？我们不能让它发生。

受访者：刘老师讲课这么受欢迎，肯定不能肯定被学生赶出去！哈哈哈

刘书博老师： 那别的我觉得也没啥，我觉得今天了解挺充分，正好一个小时。

# 受访者23

吕梦娜：那咱们就从刚刚跟张老师聊的时候，您当时还说我们到时候访谈您的时候可以聊聊的那个问题说起吧

黄秋莉：AACSB，就是它可能对我们商学院有一些影响，但是对我们实质的个人，就是老师个人好像没有多大影响，好像就是停留在一个表面，但好像也有一些影响。受访者提到了一个对他思考层面的一个冲击吧，老师可能会有一些自己更多的想法。

受访者：其实就是他有一些明确的标准，比如说你们上课的话，必须要把这个教学目标先列出来，然后教学进度、教学效果，所以它的这个标准我们现在看起来就是KPI，就是它已经把这个事儿变成了一种科学主义，泰勒的那种感觉，所以教育它也是把它看作是工厂的标准化、流水线生产，一定是可以测量的，它的背后的一个意识形态就是我要测量你的工作，不管是那种生产还是教育，我都要测量，因为测量可以标准化，可以去保证质量，可以去管理，所以它是一个很强的管理主义的这种

吕梦娜：但其实测量也并不能保证质量，从我们现在来看

受访者：对，所以呢我们现实中就看到两张皮嘛，我们只是把它拿过来变成我们吸引客户的一个符号。但是它不产生真实的改变作用，所以就是老师在填那个表格的时候，可能看都不看、想都不想，就随便填，只是为了应付他们那个认证，认证有一个体系要求老师每年都要填，只是在应对这个官僚的要求。

吕梦娜：所以说其实并不会对您作为老师产生任何触动，就是那个表。

受访者：那个表现在它不会让老师更关注于教学或者标准化教学，是这样，所以它就变成两张皮嘛，我们又多了一份无意义的工作，学校学院会觉得自己又离“双一流”靠近了一些，但实际上不会产生正向的真实改变。所以这个叫decoupling，脱耦、分开，两套运转的体系。

中国我们现在所谓的这种学术的发展，AACSB是一个代表，会发现大家是花一部分钱，然后从西方认证的权威去买来这个标签贴上去，但是这个标签认证并不等于说我们就世界水平了，还是自说自话的，还是我们原有特点的，就这样。那跟学术发表也一样，我们发了非常多的文章，即使是发到世界顶刊，但是就意味着我们的学术水平就提升了吗?也没有。所以就是同样的事情。

黄秋莉：就刚刚老师也讲到这种脱耦策略嘛，那这种是不是就是老师当初经常提到的这个异化的概念？

受访者：对

黄秋莉：因为异化，好像我第一次明确地听到，是赵向阳老师说我们这个研究可能会聚焦到这一块儿。那对于老师来说，我感觉以前老师是关注这个学术麦当劳化，然后这个学术殖民主义，我记得老师之前也提到过，但这个异化是后面才涌现出来的，我可能就想了解一下，老师大概是怎么关注到、聚焦到我们商学院的这个研究范围，然后最后又怎么聚焦到这个异化，这种心路历程或者是所见思想？

受访者：我其实也没有清晰的一步步像你说的那种，异化是卡尔·马克思提的一个关键的学术理论概念，麦当劳化呢，有一本书就是《麦当劳化的美国》，作者是一个社会学家，你们找一下，那本书背后他也不是新提的概念，他其实就是讲的是马克思·韦伯的“铁的牢笼”，就是官僚制度产生的一种负面的结果。所以麦当劳化对应的就是韦伯的理论，异化对应的是马克思的理论，但是马克思和韦伯他们俩理论的共同点就是发现了异化和麦当劳化，就是人对于他的真实价值的追求就已经不在了，我们变成了一个自成体系的自运转的一个东西，这就是异化或者叫麦当劳化。

黄秋莉：您的意思是麦当劳化是这种在铁笼···？

受访者：麦当劳化的意思就是我们用工业化的效率至上的这种观念，然后来推动生产的发展。

黄秋莉：其实我感觉它的本意和管理主义是一个意思

受访者：也不是一个意思，就是他们是非常相关的。麦当劳化意思就是我们把万事万物都变成麦当劳。这个不就是异化吗？

黄秋莉：那韦伯“理性的牢笼”

受访者：“理性的牢笼”它的表达之一就是麦当劳化，理解吗？

黄秋莉：我不理解，为什么“理性的牢笼”···

受访者：“理性的牢笼”就是我们建造了一个麦当劳生产的系统，这个是基于理性，我们要量化，要泰勒的那种量化，要程序化、标准化，这个不是就是基于理性吗？

黄秋莉：我还以为是官僚的那个

受访者：官僚也是基于理性的，其实麦当劳化也是官僚的，只不过它是特别强调标准化的、科学主义的官僚，官僚也是我们大家一块儿来做一个组织合作，那我们是基于效率最大化去设计出来让效率最大化的步骤。那这个就是麦当劳化，是不是？对吧？所以这些不是分割的概念，他们相互之间描述的都是同样的事情

黄秋莉：不同的面相可能是

受访者：它只是这个现象，其中重点呈现了一个，就是我们要把这个很抽象的这个事情给他给具体化表达。所以我们发明了异化呀，官僚呀，官僚制度，麦当劳化呀，科学主义，管理主义，其实说的都是同样的事情。

吕梦娜：对，最近就是感觉自从知道异化这个词儿之后，就感觉好多地方其实都能看到这些异化的现象，比如脱口秀，之前老师发了一个犬儒主义链接，里面有个视频嘛，讲到了脱口秀。我才知道，我现在所看到的脱口秀其实已经是异化的脱口秀了，就是现在有点装疯卖傻那种感觉，其实根本触碰不到现实，或者不是脱口秀刚产生时想要达到的那种效果。

受访者：所以工业化它有时候就带来异化，工业化把它指标化了之后，那我们怎么异化呢？就是大家只看这些指标。可以容易被量化的那些dimensions。所以我们现在看到脱口秀它其实没有什么创新思想，他只是就在硬凹那种效力，拼凑出来一种相当于工业生产的麦当劳的肉鸡的那种，你吃起来它是鸡，但是它又没有土鸡的那种真实的味道，因为它一个月就成熟了，它非常快的，就像我们生产论文一样，那都不想看第二眼，因为它太工业化了。还包括食品添加剂，那口味全是什么味精呀、糖精呀，它不是真实的，它就是工业化的异化，这就是异化。我们爱情也是。似乎被消费主义挟持之后，表达爱完全是说要买礼物呀，或者说要打点儿呀

吕梦娜：我今天还看说DR，就那个说男士一生只能买一个钻戒，其实花500块钱就可以把消费记录给清掉，我就感觉特别的可笑

受访者：所以你看这种就是异化呀，它本质已经不在。

吕梦娜：对对

受访者：变成了这种可以去消费的，可以去量化的。但是量化这个，所以我特别让你们读的就那本书《指标的陷阱》，那个其实讲的就是异化，就是只要我们要衡量、要程序、要标准，那必然会带来异化，不再关注过程的本质的价值，而是只关注标准。这个跟我们现在说的这个学术是一样的。

吕梦娜：那次就是莉莉发的那段话，我还摘出来了，说是“我从不打印自己的论文，觉得浪费资源”，然后您说您也觉得是，这个我倒挺惊讶，我觉得您现在做的研究···

受访者：我会有发表的论文，都没给你们看的论文，我也有那种生产麦当劳的那种论文，就为了跟同行评比嘛，因为他们都在弄这个，我不弄显得我就不行了

吕梦娜：就被卷起来了，是吧？

受访者：对呀，所以这就是大家为什么被异化，当然有一些人只剩下那些论文，有一些人还有保留自己真的东西，就没有被异化。就我去生产那个肉鸡是为了生存，但是我养土鸡自己吃。是这样

吕梦娜：对，明白，那这样的文章现阶段来说比例还大吗？

受访者：不大，我最近反正我觉得副教授评上我就放松了，因为社会对于教授、副教授也没有特别强烈的分别感，反正带着教授，只不过同行他们会比较在意，但是我其实对同行的在意我不在意，因为他们我觉得挺弱智的，就完全被制度给挟持，小锅儿叮当响的那种，所以我觉得我可以更专注于想做一些、积累一些自己觉得有价值的东西。

吕梦娜：就可以做到自由探索了现在

受访者：对对

吕梦娜：ok

黄秋莉：这种教授和副教授，对这种职称是有一定的迷恋嘛？是为了面子吗？

受访者：对，为了面子，老外也是为了面子，就它是一种职业的等级嘛，它代表的就是你在这个专业领域达到了一个什么水平，但是其实我觉得在中国这个水平也比较混乱，有很多教授，你知道就是那种没水平的教授很多，所以我会觉得更多是要做出真东西产生的影响力，影响力是在外部。

吕梦娜：而且我昨天想查受访者的个人信息嘛，因为我不知道他是哪个系的，我发现战略系里的老师，您的阅读量是最高的，五万多阅读量呢。我觉得特别棒，看的我也很激动。

受访者：所以就是真学者他追求的还是影响力。那影响力可以是制度化的，就是title嘛，但是我们会发现很多教授我就觉得很可怜的

吕梦娜：咱们院长才八千多

受访者：对呀，而且他很不开心的那种，斤斤计较的那种，所以我觉得人生就已经被异化到自己难以察觉了，这就是挺悲剧的一个现象。

吕梦娜：对，是。

受访者：所以有时候人，像刚才那个小女孩儿，也是被异化，她会觉得不努力怎么就···一天一定要打卡要完成。她就觉得这个事儿就永远应该是这样，她就不快乐。

吕梦娜：她这个感受到跟我上研一的感觉差不多，我觉得可能确实是环境是有原因的，之前我一直觉得是我自己的原因，但现在越来越多的同学说了之后发现，可能是这个大环境是有一定原因的。

受访者：所以就是人现在就像肉鸡场的鸡一样暗无天日地过了一辈子，他并没有看到真实的那种野外的草原呀，或者那种自由的自主的生活状况，他的一生就匆忙的结束了，非常被制度化，那就是这样，但是肉鸡生下破壳而出之后就看着这个工厂的环境，他就觉得世界就是这样子。

吕梦娜：其实我们做这个访谈的时候，我就特别想访谈一下我本科的指导老师，因为她现在也在指导我嘛，就是我觉得她就是完全被制度化的一位老师，因为她刚来的时候“青椒”嘛，“非升即走”的制度，然后她现在好像是第二个合同期，反正压力特别大，我就觉得就是完完全全就是我们想要的那种样板的感觉。

受访者：有这样的，我也遇到过，尤其上海那边很多这种，我跟他们接触，我就觉得就是契科夫的《套中人》，那个文学作品嘛，他就是佝偻着身体，然后整天焦虑溢于言表，然后探讨的问题缺乏任何趣味性，你完全的你就能够知道他在想什么，他每天那种勤奋的状况，所以他是一个你不用管理、他已经自我管理得非常好的人了，而且这种管理是，就是咱们看到这种，就福柯说的那种自管理，权力的高级形态，它是以知识标准的形式在进行管理，而不是说你要是不这么做，我就要抽你的那种，对吧？所以我觉得真正的学者应该是自由的，就是自主自由。

吕梦娜：对，我其实今天问张老师这个评审意见的问题，也是在我本科老师身上想到的，我就觉得她特别特别的“乖”，就是她自己也跟我说，就是对评审人的意见一定要几乎是百分之百的遵从，然后去不断去朝评审人的方向去修改，甚至有一些词语，因为那个是投的外文的期刊嘛，然后她就会跟我讨论说这个语气有没有问题？就是会到这种程度，就特别特别的乖

受访者：那就是老好学生嘛。

吕梦娜：对

受访者：那张老师他还是有自己的思想，就我跟他交流接触，我会觉得他是有自己的想法，他会能够分清楚那些评审的不好的、虚假的、不真诚的。但如果像你刚才说的那个，她就是就有点像对待圣经一样的。

吕梦娜：她也知道有些评审人可能是不好，但是又没有办法，就是你指出他的问题对你于事无补的情况下，她可能就不指出这个问题来了。

受访者：那我觉得她可能有点懦弱吧

吕梦娜：可能就是被制度压的吧，现在还没出来

受访者：不管懦弱是为什么，她表达出来是懦弱

吕梦娜：对，是有点儿的

黄秋莉：其实我感觉老师对于现在的制度哈，可能是我觉得是有一种超越性在里面的，就比如说有些会议可能就不想参加就不去参加了，就是我想知道您在生活中有没有遇到除了会议之外比较繁琐的事情，感觉没有多大意思，就这种类似的事情有哪些？

受访者：除了会议就是比如说让我参加一些论文答辩呀或者是指导呀或者是跟学术不相关的，那我就不参加，我就说有事情

吕梦娜：就可以，是吗？

受访者：可以。那他也不能怎么样呀

黄秋莉：这种事情是比较多吗？

受访者：就是挺频繁的，就比如刚才这个让我去给XXX老师，好像哪个教育部审查，然后谈他在做委员会委员表现得怎么样？那我就直接拒绝了。这个事儿我为什么要去谈？我凭什么要谈，是吧？

黄秋莉：就是我们在科研工作当中哈，就不仅仅是科研工作，其实我们会面临很多这种组织上的非专业性的要求。

受访者：是的，因为组织里每个人都有自己的想法，那尤其是中国的这种学术机构，那它应该是有很多跟学术不相关的事情，包括什么思想政治教育，这些就相当于边界不明晰。我觉得在中国就是学术跟政治没有分开，学术总是被政治给把持着，所以我们有时候就没有办法去形成一个独立的学术共同体，而且政治把持学术的时候就会出现一个现象，那些特别谄媚于政治权利的学者，他可能会走的更快一点，他会把这个标准进行（复制），所以导致这个学术生态就不是特别好。

吕梦娜：对，其实刚刚老师提到教育学的一些期刊，他们会写一些文章，就看着真的很不爽，他就想表达那个意思，他就是不敢说出来，就一定要用一些好的词儿给他不断的修饰，就看着很压抑，就像你就能不能把这事儿给捅出来说出来？！但他好像就不敢这种感觉。

受访者：对，是的，那没办法，因为我们有很严格的审核制度，有一些话题的研究也不能够发表，其实这是一种大家心中有数的不成文的，这就是文化，大家约定俗成的一种认知嘛，而且我们生活在一个观念的世界，很多观念其实是学者应该去构建和维护的。那如果我们学者在这一块儿失守了，集体失语或者被制度化，那他没有一个独立性或者自由性，那这个世界就完全是权力说了算，甚至它可以篡改历史了，对吧？所以我们对于世界的认识不是说只是自然界，我们人的世界很多是观念层次上的东西，所以我们对于很多价值的判断，对于这个事情的定义、对错判断等都是来自于观念世界。所以中国有很多事情我会觉得观念就是错的，就是不合理的，但是它们又在自身体系在表达这个权力的合法性，就形成了很多学科，这些学科在中国就很有特色，对吧？

甚至这些特色都渗透到了各个学科，所以我们会有这个东西（一本管理学科相关教材上写着“马克思理论研究和建设重点教材”），这个管理学精品课程为什么要镶嵌这个东西？它只是一个理论、一个哲学的一个派系，然后他已经泛化到了所有的学科专业，那这个我觉得肯定是一种权力。

吕梦娜：我还想到刘老师您那天发的那个模型图，研究“闯”那个，您是怎么看的？

受访者：我觉得本来这个研究他不应该用这种实证的假设模型，他硬要把这个套上去，结果就变成一种不伦不类的东西，就大家拍脑袋想的这个东西。

吕梦娜：我看这个我会觉得大家对很多政策性、指导性的东西理解太浅了

受访者：你说这个把中国研究做在中国大地上？

吕梦娜：对

受访者：他其实还是没有去做到中国大地。

吕梦娜：对，他只是借用了中国已有的一些概念，甚至像刚刚老师提到这本书，我都觉得我们提倡的比如说马克思哲学或者怎么怎么样，然后他就很生硬的进入到这个，就感觉非常的不合理，可能人家背后想的或许不是这样，但是被执行出来之后就是这么两张皮给硬凑到一块儿去了，就比较违和。

受访者：所以我觉得这是在中国的一种权力结构下的一个特色，因为本身是从上到下，尤其是下面要去迎合上面的单一的权利，因为集权的有资源，你迎合他就给你，你必须要赞同他、支持他，不然的话你没有发展资源，所以就导致了各个层面都是在做这种示好。但是他可能也不是真正的去这样做，就是意识形态个人与上层并不是完全统一的，他虽然不认可，但是又要去表达，那这个时候做的就有点很粗糙了，就他对这个事儿不认可，我还要表达出来，做出来的东西可能就不伦不类。

黄秋莉：老师在国外待了很多年，然后又回到国内，会遇到很多老师的嘛，自己有国外的老师，然后再回到国内，那国内外老师在气质上您感觉有什么差异吗？

受访者：我觉得国外老师会更独立洒脱一点吧，我不知道，可能美国也比较制度化，那我会觉得美国之外的这个香港、新加坡全是美国体系的这个，他们会比较跟中国大陆一致的。那像其他澳洲欧洲会不一样，这个挺明显。

吕梦娜：您之前提到过贵族精神吧。

受访者：他们就是不会以我自己的标准赋予他合法性，我不要求我这么做是完全要得到别人的认可，我会强调我自己认可的价值，而且是非常勇敢地、自信地表达出来，这是一种贵族的精神气质，而且这种价值往往是利他和社会性，它不是一种世俗化的价值或者工具化的价值，就咱们中国的学者可能这种世俗化的现象会更明显，我们学者就贵族感很差。贵族像《白鹿原》里头那种乡绅，他其实做很多事情不是考虑自己要获利多少，他是为了整体的和谐安定，为了帮助有一些贫困的老百姓或者同乡邻里，所以他做这个事儿的时候他给你的那种感觉是他不是精打细算的，所以贵族其实在这个传统的封建社会，它就是一种社会的道德标兵，是那种散发着热量和光辉的那种感觉。

但是你看中国学者就是有点猥琐，斤斤计较的那种“小锅儿叮当响”，天天跟你说房市、找户口、找对象的，类似这种。那这还是好的，他就可能很世俗，但是还有一些就很猥琐，还有一些很变态，但那种贵族的那种精神，就像《无问西东》里面那种，他会描述刻画一些当时清华北大的那种学者，他们就很淡定，就很儒雅的或者是光明正大的那种，人家也很勇敢的来呈现自己的观点，现在学者有时候就会觉得他的素质好像比较低下。

吕梦娜：您会以大学老师这种身份而觉得骄傲自豪或者是有一种很好很正面的一种感觉吗？

受访者：我会有呀。

吕梦娜：会有是吗？因为我的感觉就是好像有些老师就像一个打工人、有种自轻自贱的感觉。

黄秋莉：我感觉老师都还挺骄傲的呀。

受访者：像xx这种，他是骄傲，但是他不是说为工作本身好像价值感很高，他是非常追求这个外部价值，他把学术本身的价值贬的很低。对，所以他不是骄傲于这个事情本身的价值，他只是说骄傲，你看我把它做了这么标准化工作，然后我知道“茴香”的“茴”有好多种写法，我就比你知道的多。他是这种很异化的骄傲。而且他的骄傲来自于自己比你厉害，他很简单，他的话语也很直白简单，“你厉不厉害”，“这个垃不垃圾”，那就很简单，所以他的话语就是他的世界观的一个表达。

吕梦娜：那您就这位同事也好，或者是其他同行老师也好，就可能根本不会把自己大学老师这个身份看得很高，或者是觉得就是一个普通的打工者这种感觉，您自身肯定不会，那您看同行有特别多吗？

受访者：特别多。

吕梦娜：是吗？他们有什么样的表现呢？

受访者：就给人一种“我是教授”这种感觉，我觉得年轻的会比较多一点，就他会自我管理的那种，他当然也有一种所谓的好学生的那种骄傲感，但他还是好学生的骄傲感。他的骄傲来自于他达到了既有标准，他在标准层面做的比他的同行要好，还是外部价值，他没有说这个事的内部价值。

吕梦娜：因为特别是像非升即走这种，他们也有kpi的嘛，就跟公司的话语体系就通了，就会觉得我其实也是个社畜，比如打工者会有这种感觉。

受访者：对，是的，就很清晰，是这样。所以这种学者我觉得特别无聊无趣，他没有一种意外的惊喜的那种，或者是好奇心饱满的。他们只是在自己的那个狭窄的范围内去进行那种好学生的那种感觉，而不是说他到一个陌生领域，他不是说特别充满好奇的想要去专注进来或者了解，你能感觉到他是会偏到他那个地方去了。对，就真的“专家”，就只是这一块儿。

但是我觉得学者跟专家还是有所区别，学者应该是有专业领域，但是他不会因为专业就封闭掉自己的心理，他会总是对这种知识的观念感兴趣，他会对于这个人力资源研究感兴趣，同时他也关注魏晋南北朝，他也关注印度的这个宗族制改革、种姓，他也关注这个物理的最新的研究，所以我觉得这是一种学者的那种本质、这个精神气质。

但现在我看到更多就是（只关注）一点儿，然后很工具性的，它很细节的那种，那什么meta分析，他会很工具层面、技术层面而没有思想层面。

吕梦娜：对，是这样，老师刚刚就讲到了几种不同形态的学者那种感觉。

受访者：我觉得中国本土培养起来的学者会更多像刚才我说的那种特别强调专业性和工具性、技术性。

我有的时候觉得可能他们本意不是这样，但是这种制度之下，特别是前几年的这种指标制度之下，会把他们逼得只能去看这儿，就像麦当劳一样，我生产肉鸡，我不是要求鸡的这个羽毛多样，我也不要求鸡它能呈现出来什么别的多样性，我就是让你长肉块，仅此而已。所以学术也被变成麦当劳的工厂和学术的麦当劳化，我们的价值已经非常单一了，对于价值评定的标准也非常一致了，那就是这样异化。

吕梦娜：您怎么看待导师跟同学生的这种“老板与下属”的关系？

受访者：那也是异化，也是一种市场化，市场化话语已经渗透进了学术界，而且大家会觉得称老板，老师也觉得没事儿，还有的老师还挺骄傲的，因为他觉得他自己还变成了老板，他有了市场价值。所以我们在价值评判的时候觉得这个事儿没错，我认可市场上的那些东西，所以这就是一种市场逻辑进入到了学术领域，进而排挤了学术原有的个逻辑，产生了腐化和变质。

尤其是我们中国人不擅长于反思，不擅长于批判，还有一点就是我们发展的太快，我们总是在比学赶超的大跃进，所以很多副作用我们有时候没来得及去反思总结可能需要改变。就像我们足球队一样，总是在换教练，他从来不思考到底背后有什么结构性的问题，是吧？总是急不可耐的想要出成果。

黄秋莉：讲党委的管理，就是这种党的意识形态主要是对这个学校场域有影响，那对咱个人的这种……

受访者：你个人就在场域里。

黄秋莉：是，但是咱感觉还是有很大的自主空间，还是说自主空间比较小？

受访者：我觉得还是挺有空间，你不要直接对着说他这个就是完全错误的，因为我们的专业不是政治学我们还是管理学嘛，我们除了这种政治权力之外，还有很多别的事情可以去探讨。即使政治学那我们还是一个弱势，我们不是说要批判我们这个权力，可能更多批判的还是西方的那种知识霸权，所以我不觉得它直接影响了我们思考的范围，当然也确实是有影响，但不至于说完全窒息，咱们知识霸权在咱们管理学还是提到挺多的，刚刚张老师也说这种投稿呀，特别是这种圈子呀，还有这种理论范式呀，其实都是很主要的方面，就把咱们的思想束缚在这一块儿了。

黄秋莉：对，是这样。如果是我们以中国的这种样本去向国外投稿的话，他们会对咱提出质疑吗？

受访者：会，就是“why China”，为什么你们只是选中国，当然你选择中国他们不一定会否定，就是他会要求你论证中国为什么它具有理论贡献，但其实我觉得这个影响不大。关键还是张老师刚才说的那个有一些隐性的标准，就比如说这个研究怎么样去进行一些数据的搜集和分析，哪种题目怎么样选研究视角、怎么设计，这些研究视角就是前沿性的研究中哪一些他们认为是有价值的。哪一些他认为价值不够，也就是价值判断。

黄秋莉：我们现在是不是觉得学术界会有一种倾向，就是说西方的就是规范的，西方的就是好的。

受访者：那我感觉他们确实是比较规范，确实是有这个方面的原因。

黄秋莉：但是像光华就觉得实证主义就是科学的，然后我们不要去做那种故事会呀之类的。

受访者：这也是一种知识霸权的体现。那就是一种哲学观方面非常的单一，他的研究范式也很单一，所以他就认可的这种自然科学的这种。

# 受访者24

刘书博老师：所以我们其实想要了解你为什么选择走学术，然后这个学术的过程中有没有觉得现实的跟理想的是有偏差的，尤其是中国咱们这个学术的制度环境类似的一些维度，所以你当时做选择的时候为什么想要到学术界？

受访者：这个问题我觉得还真是可以聊一下，因为当时确实面临这个问题，其实我毕业的时候也没有完全想好到底是去业界还是去学术界，当时找工作就是两个方面都找了，在银行也找到了，然后最后还是选择来学术界。因为一方面确实对这个感兴趣，这肯定是前提条件。第二点呢就是有一些家庭因素在里边，就是这个山东人的教育呢就是从小可能比较爱学习吧，然后就从大学的时候呢，就可能要觉得对这方面（学术）比较感兴趣，就立志要做这个东西，但是呢中间也是有摇摆的，毕竟中国的高等院校的待遇和金融行业的待遇实际上差别是挺大的。但幸运的是就是进入商学院，因为商学院的待遇总体而言要是要高于其他的学院的，所以，最后其实也不能把这个职业选择比较地这么细，最后还是看自己想做什么东西，所以就选择了来高校，当时在那个银行里做过一段实习，做的也是偏研究性的，是工行的博士流动站，偏做研究的，然后感觉如果都做研究的，还不如正儿八经的去做研究，这样更好一点

刘书博老师：跟你性格也有关系，因为你之前也提过说那种长袖善舞的，你不喜欢那一套

受访者：对对，这个是很重要的原因，你提醒了我这一点，就是在银行的环境是特别社交，这方面很重要，但是现在呢可能这种场合、这种机会变少了，所以最后呢个人的原因就是还是不太善于这方面吧

刘书博老师：那你的兴趣呢就是什么时候开始你觉得自己喜欢这个事情，教育可能一开始就是一种达标呀，那你的兴趣大概是从什么时候开始的

受访者：对对对，其实我觉得是在···我记得印象很深刻，就是大学二年级的时候，其实我们的那个当时是系主任吧，在山东大学。我有一个想法，然后关于保险业的一个资产结构的问题，就跟他讨论了，然后最后他就指导我写论文，其实从那一点是我主动地来找他，然后他觉得也比较有意思，然后就开始写。后来想想，就现在来看，其实当时本科生的想法确实有一些幼稚的地方或者不成熟的地方，但是老师愿意去带你去做，我觉得确实这位老师对我的影响还是挺大的，包括后来保送研究生的过程中也给了很大的帮助。

刘书博老师：研究生也是在山大？

受访者：对对，当时保送的时候，我们名额还是有限的，就是有五个人嘛。当时我实际上要考南开大学，南开大学的精算专业。后来呢保送了就留在山大了，读了金融学的专业

刘书博老师：所以那你数学这方面还是挺好的

受访者：对，但是数学我觉得还好吧，就是在山东的学生里也不算特别优秀的

刘书博老师：但不算是吃力的。

受访者：对对对对对对

刘书博老师：觉得做这个也不是说很苦。

受访者：对，我觉得就是如果很苦的话我就觉得无聊嘛。保送完研究生以后，我大四的时候，就跟着我的硕导，然后一起从整理数据开始这一套流程进来了，我理解的是在这个教育体系下，就是中国的教育可能是一个导师来带领你、指导着你，真的和你去融入到这个研究团队里一起做事情，要不然很难在这个学术圈子里存活下来。

然后从摸数据开始到后来的写论文，到了研究生三年级的时候，实际上又到了我们决策的时候，就是要继续读书还是工作。那时候呢就是比较幸运的是有一篇文章出来了，就是在《经济研究》上。所以我觉得既然有这么好的机会，我就再去努力一把，就来到了人民大学。

刘书博老师：那人民大学也是考试还是？

受访者：考试。其实当年博士的考试竞争很激烈，这个在当年的时候我们有一半是在职的学生，有一半是全日制的，所以在职的学生怎么进来的我们也知道，可能那方面的因素比较多，所以读博也是一个比较幸运的事情。

刘书博老师：那你读博那个过程怎么样的感觉？就也是比较幸运的，遇到一些好的老师？还是说会有一些小失望，小挫折？

受访者：坦白来讲，当年其实我对人民大学期望挺高的，但是呢进来以后，就从学生的构成来看，其实一半是在职的学生，一半是全日制。我们就明白他们可能不完全是学术化的培养，所以在课程设置上还是有些失望。但是人民大学的有一个好处就是，像我们这种院校一样，他的院系重叠度非常高，如果你不想在这个学院听课的话，你可以找到合适的资源去听课，所以我觉得人民大学提供的是一个好的平台，就我们有很多的交流的机会、上课的机会，这个是比其他学校、比那个山东大学交流方面要好一点。但是从我们学生的就业来看，我们那一级有四十多位同学，可能去高校的只有三位了

刘书博老师：那其他的呢？

受访者：有一半人根本就是有工作的，还有一半的话就是去金融行业

刘书博老师：也是在金融机构做研究？

受访者：这个其实分化也比较大，有的是做投行，有的百分之我觉得60%是在做研究相关的

刘书博老师：那现在就是你们那一届发展的怎么样？现在有联系吗

受访者：我们还是每年基本要见一到两次。怎么讲呢？就是我们聚到一起，聊聊大家最近的进展嘛，就是这个过程中发现确实在业界可能他们的待遇比较高，但是呢比较忙，就赚了钱没时间花，真是这个状态。包括我爱人她不是在银行吗？整天加班，但待遇，坦白来讲，没有我们高。

刘书博老师：就性价比来看

受访者：要差一些，但是有些行业比如说基金，像投行这些，他们收入是我们的3~4倍吧

刘书博老师：但是他们不是风险系数也比较高，就不是那么稳定嘛？

受访者：他们不稳定的概念是有的时候200万，有的时候150万，其实他们有一个···

刘书博老师：就是肯定是有收入的，只不过有的时候少一些

受访者：对对对，他们的底薪就比我们要高，所以就是确实行业差距还是挺大的

刘书博老师：但是他们那种职业可能是不是也是早期比较快的，然后是到了40岁以后就开始往下，有没有这种趋势

受访者：它不见得往下，就是平缓，那平缓也是比较高的层面

刘书博老师：我们是这种渐渐渐渐，到60岁了

受访者：就崩了哈哈，但问题是大部分人的薪酬并没有这么高，像银行的薪酬的话可能和我们差不多吧

刘书博老师：所以这个金融行业它也是有369等的。

受访者：对，而且在同一个公司，不同的岗位的差别也挺大的

刘书博老师：所以其实你跟这些校友聊还是觉得自己当年选择没错

受访者：我觉得其实挺开心的，他们也觉得确实高校现在挺不错的，但是行行出状元，也就有的人在某个行业中确实做的非常非常不错，就是他们也挺开心，但是有的人呢就感觉我们的学的专业和现在的工作其实差别也挺大的

刘书博老师：ok，那咱们差不多同时期来的，然后这个发展也算正常，有没有说一些不如意的地方，就觉得可能如果之前我不这么做，现在会好一些。类似这种？

受访者：我想想看，其实我现在倒是没有太大的这个感觉，主要是我们这个系不是有特殊性嘛，所以这一点我觉得不具有代表性，就是说我们系现在没有本科生，但是其他系，就像战略系，我们也参与挺多。就如果从我们个人的发展来看，其实教学这块儿是很重要的。那如果你这个专业本科生不存在的话，你的教学，你的本科的教学实际上是受到一些限制的，像现在一些精品课程、教学名师或者是这个团队的培养，其实受到约束的，这一点是客观的一个问题。

刘书博老师：但是我们不是还有一个说法，就是科研是专业性的主要代表。

受访者：现在我感觉这个现象在扭转，它是真是在发生悄悄的变化，那这个变化呢是一点一点来的，不可能一下子扭转过来。我觉得科研对于高校来讲是重要的，这是在国内外我觉得都是非常肯定的一件事情。但是呢可能中国之前的这种高校的教育太过注重科研了，而把我们的本职工作——教学，激励也没有，是吧？没有什么制度的保障。所以现在的改变就是，我看到的是，教育的这种教学工作的重要性在提高。表现在你申请一些比如说人才项目，申请一些奖励的时候，包括我们教学上面的奖励都是在提高的，这一点是我们能够看到的，但现在我感觉有个问题就是你会发现哈，当年做学术比较牛的人形成了一个学霸性质的一个团队，他们仍然···

刘书博老师：能保持着这个标准

受访者：对，对对，把持着这个资源，移植过来

刘书博老师：所以他同样移植过来。

受访者：对呀，对呀

刘书博老师：把名字改一下

受访者：对，是这样的，是这样的

刘书博老师：但其实可能他对于教学也并不认可的，但是呢他就有可能是教学方面的各种项目的承担者

受访者： 对，就是这个教学和科研的一个差别是，教学的质量我们很难去评判

刘书博老师：对，隐性的那种，长时间的

受访者：对对，像你现在举办的一些什么青教赛或者是比赛，它完全是一种表演性质的，真的教学效果怎么样，我们是不好评判的。

刘书博老师：没错

受访者：所以导向是好的，但是导向怎么去落地，就真正让老师就自己自发地去提高教学质量，而不是为了一个头衔、项目那种，所以我觉得现在的这个激励逻辑没有变，就是还是科研的那一套用到了教学

刘书博老师：还是用评科研的那个，以评促改或者促教，但是这种科研跟教学又是不一样性质的东西

受访者：是是是

刘书博老师：他就拿着那个性质评比来评这个

受访者：对，只能造成比科研的评价问题还多的问题。

刘书博老师：对对。

受访者：但是没有办法，我们现在年轻人想去做的话，实际上也比较难，比较难，有的时候教学说是简单，但是也不简单。

刘书博老师：对，它有很多细节，还需要有点耐性。也需要积累。而且这种耐性和积累又很难像科研一样把它显性化。

受访者：对，对，是的，是的。

刘书博老师：那科研呢。科研现在你这方面应该是相当不错的，就是从研究生开始一直到现在，有没有说你看到这个制度呀或者是学校管理治理，对于科研似乎也有一些需要思考、反思、改进的地方。

受访者：科研方面，我想想看

刘书博老师：你应该更多是在经济学领域而不是管理学。

受访者：对，就是经济金融嘛，这个学科，我现在对这个思考不多，但是我觉得其实本土的学生和海外的学生他们在竞争的维度上是不在一个层次上的。

刘书博老师：为什么？

受访者：就仅仅是经济金融就特别明显，就是我们这个科研里也是有圈子的，像我这种人很难去发一个就是顶刊，国外的顶刊哈，只能在国内混一混，然后国外的话我们就得一点一点自己去摸索，但是这个摸索的过程是很痛苦的，目前来看就是本土选手怎么去国外的好的期刊来做呢，就是有国内的大牛然后跟国外的大牛一起合作，那这种东西对我们来讲是有天花板的

刘书博老师：所以其实还是有一些隐性的标准。

受访者：对，对对

刘书博老师：这个时候如果没有关系是很难去做的。

受访者：对

刘书博老师：那这个能不能说咱们像你在耕耘的这个学科其实也是被海外的话语权给把持着

受访者：比较明显

刘书博老师：比较明显？

受访者：对，比较明显。

刘书博老师：那我知道管理学几乎90%是美国把持着，你们金融经济也是？

受访者：不见得90%是美国的，但是90%的是欧美的。这个把持是怎么讲呢？我们看各个期刊的editor来看基本都是欧美的。但是呢目前来看中国人在海外的发文量还是比较大的，特别是经济和金融领域，这个圈子就是中国人正在渗透。那渗透的前提是，确实基本都是国内的大牛在渗透。这个成长，唉呀，就对于本土选手来讲，这个可能受到限制更多吧。

刘书博老师：所以其实我们想要成为世界影响，跟海外比还是很有先天的这种弱势。

受访者：对，就是你刚才讲的这个话语权的问题。实际上有人统计过，很多国外的idea发表出来，就是有些研究是我们在国内已经发表出来的，然后在国外又发了。

刘书博老师：那这这一块儿像你们领域的学术创新，就我们管理学经常会批评说不关注管理现状，有一些本土的东西只是在自说自话，那经济金融是不是也有类似的问题？

受访者：我到了商学院以后，我也确实在时常反思管理学和经济金融学的差别哈，我觉得管理学确实存在这个问题，那我对管理学的定义就是我们探究的是个案，更多一点个案的智慧，那经济学方面探究的是一般的规律，所以他对于这个方面的质疑声要小一点，弱一点

刘书博老师：对，是这样，你们比较宏观嘛

受访者：对，比较宏观，然后找一个就是规律性的东西来。但是现在我们学科有一个发展的批评声就是太过于量化了，就是现在的数据，做那么多数据，有的时候呢实际上就从统计上就能看出来，看得出来，故意量化了。

刘书博老师：ok，好，那我就问这些

黄秋莉：老师，我就是结合您今天讲了自己的经历，您说您大二的时候是基于自己对保险感兴趣嘛？就说是一种自己的驱动。

受访者：是的，内向驱动，

黄秋莉：那您现在已经成长很久了，现在也做的非常好，那您对比您现在的这种学术研究心态呀，或者是状态和您最初的那种状态有什么样的不同？

受访者：其实当时也不知道自己是一个什么样的状态，现在也记不清了，但是我觉得当年主要是从文献里来到文献里去，现在的更多的是实际上我们两条腿在走路。一个就是理论方面的，这个是基础。再一个呢就是要观察现实问题，我觉得我们的研究就是要向研究现实问题这个导向去走，就是研究的问题要有意义，要关注现实中的问题，之前呢可能就是从文献来到文献里去，但是现在我包括我知道的那些学生也是，就是还是从文献里来到文献里去比较多一点，但是我们现在这个方向就是要改一改，就关注现实问题。

刘书博老师： 怎么关注现实问题，是看新闻还是说···

受访者： 就是看一些新闻报道，包括一些比如说国家提的政策，提出政策以后那企业就得有反应，那国家政策是实实在在有些制度保证的，比如说减排，那它对于企业的行为有什么样的影响，那这个我觉得现在是我们关注的热点话题

黄秋莉： 是因为一种现象驱动是不是？就是咱们做学术的一种···

受访者： 问题导向嘛

黄秋莉： 是信念嘛，就是咱们做学术的话就想着要去解决现实问题。

受访者： 对对对对，而且从文献里来到文献里去你很难去做出好的研究来

黄秋莉：但是我看一些就是相关的新闻哈，其实对咱们管理学就是一味的追热点，就是这种现象也会有一定的质疑。那老师您是如何看待这个？

受访者：对，有些确实过于追热点，就是这个研究，它是这样的，就是一个现象出来以后，如果要做实证研究的话，可能等一段时间来做比较好，有的呢研究确实追的比较快，他们是想通过这样来在这个领域形成一个先导优势，所以他们积极地追热点，但是我觉得关注热点话题是必要的，如果你就是一个现象，比如说我们的疫情刚刚爆发一个月，然后就去做，实际上太仓促了，可能就做不出好的研究来

黄秋莉： 也就是说，您觉着就是追热点其实也是一种比较好的解决现实问题的一种方式哈。

受访者： 对对对对，从我的这个角度来讲是这样的，

吕梦娜： 老师，因为我之前听说过，就是关于疫情，刚才您提到，因为特别是对我们组织行为这种微观管理，以前有老师就其实是持一种批判态度，他觉得疫情刚爆发，你去调查，那是对人的一种二次创伤，就是提醒他你很痛苦或者是你正处在水深火热之中

受访者： 是是是，发疫情财嘛？是吧

吕梦娜： 对，对对，就有这种感觉，您怎么看待

受访者： 其实怎么看待这个事情呢，我给你举个例子哈，就是我们的有些同行，就当时在武汉，他们做经济学，但是他们当时是采用调查问卷的形式做这个经济问题，这个问题其实挺重要的，因为当时他们是关注了中小企业的发展情况，当然他不是爆发了一个月什么的时候，他是疫情结束之后做的，然后他这个是追热点，但是呢他们是有一些比如说政策导向，提供出了一个运行的情况，包括他们的企业的需求，那湖北省或者是国家是要根据这个来制定政策的，所以这种就是有意义的，所以我觉得这种追热点的问题还是得看，就分问题来看

吕梦娜： 那这个它是自发的，想要去给国家提政策，还是说国家有这个需求，然后要求这个研究人员来做

受访者： 我觉得两方面。那个同行他是有这个动机的，因为他一直关心着民营企业嘛，但是具体过程我不太清楚，是不是湖北省让去做政策决策的内参，或者什么的，这个就不太清楚了

吕梦娜： 明白

黄秋莉： 就是老师您刚刚提到在高校工作，其实你也不后悔嘛，就是觉得自己选择的非常对，但是我刚刚听到，您觉得一方面就是收入比较可观。另外一个就是符合···就是上下时间班比较随意哈。那除此之外您觉得在现在从事这种学术工作还能给您带来其他的幸福来源是什么

受访者：我觉得是可能对比过后我们可能更加幸福，因为教学嘛，教学这个事情是一个互动的过程，我觉得是有意思的。再一个呢就是做研究的话就真真正正是关心你有兴趣的问题，然后你再去做，在这个过程中你也培养了学生，所以总体来看就是做了自己有兴趣的事情，感觉有意义的事情，起码从自己的角度来讲，那比如说别的在金融机构做的事情就是乌七八糟的，像一些材料的整理，他们也在做，就博士毕业也在做这种东西，就本科生就能干的工作，所以他们有的时候也抱怨，但是呢他们的好处就是下班以后我就可以关机或者什么，就是进入了生活状态，但是我们就是可能时时刻刻地想这些事儿，但是习惯了也就好了，刚开始两年还是不太习惯这种状态。

黄秋莉： 慧丽还说，哎呀，我觉得张老师可努力了，我都想不明白他哪来的那么多精力

受访者： 不努力不行，真是不行

黄秋莉：你觉得您现在就是文章发了那么多。还有什么？

受访者： 我的文章不多

黄秋莉：质量也挺高的呀。

受访者：那你就学生看着老师，可能发了很多，但是我们pk的是同行之间的pk，然后我其实如果不做的话，那天天干什么呢？

黄秋莉： 可以玩儿呀

受访者： 没有什么事情。但这个休闲娱乐也是有必要的，特别进入了这个生活以后哈，就是有各方面的事情，其实我觉得还是要挤出时间来，来做自己感兴趣的研究

黄秋莉： 一个是您的兴趣嘛，另外一个您说有很大的压力，是因为跟同辈的压力嘛

受访者： 压力是吧？比如说我们评职称的压力

黄秋莉：这是一个最重要的压力嘛

受访者： 最重要的压力，但是我觉得如果没有评职称这个压力，早一年晚一年都无所谓的话，其实也得去做

刘书博老师：为啥？

受访者：就感觉还是确实有兴趣在这里，而且你将来，唉呀，将来可能这也跟个人有关，就是感觉如果你评上教授以后，评教授我们早晚是要评上的，就是如果评上教授以后那你做什么呢？对不对？

刘书博老师：就是他是一个长期的···

受访者：长期的。对，对对，这个受我导师影响挺大的

刘书博老师： 怎么讲？

受访者：就我导师的心态是比较平和的，就是要就长期主义嘛。有些事情你长期来看都不是事情，所以做研究可能是一辈子的事情，包括现在我们毕业了以后，他还定期的组织这种讨论会，我看到有四五十岁的大师兄、大师姐过来也是在这里，尽管他们大部分在业界，但是这个学习真是一辈子的事情、做学问真是一辈子的事情。

刘书博老师：所以这个事情就是还是本质性的一个对于你的吸引呗

受访者：对对

刘书博老师：做这事儿确实能发现一些···

受访者：有意思的事情，自己感觉有意思的事情

刘书博老师：那你能再进一步说为啥有意思？

黄秋莉： 我就觉得还挺枯燥的

吕梦娜： 我特别是想问您，您也是最近几年开始是从这个现象中去找研究，就不是从文献到文献了。那您之前从文献到文献的过程中是怎么发掘兴趣的？我很想了解一下

受访者：文献到文献实际上就是享受那种做出来的感觉，真是这个问题有多大的意义，其实并不多

吕梦娜： 是因为模型很精致，然后能够推演出来吗

受访者：对，当时我其实第一篇做的是完全从理论的角度来讲，就看它的资产负债表对存款准备金率，对于保险公司的这种比如说它的资产配置的影响。就是这么简单一个问题，我从资产负债表这个角度，而且是理论的，当时是理论的，现在都做实证的。

觉得有意思这个问题，一方面就是我们在同行和同行之间的交流中觉得这个是一个好的研究，有些研究呢一看就是好的研究。但是有些研究确实是大部分是垃圾的研究。就是在这个比较的过程中，我们可能真的学到一些东西

刘书博老师：就是可不可以说它就是对于人好奇心的一个满足？

受访者：对对对。有的时候你就看到别人做出来了，其实这个问题我也可以做出来，为什么我没有做到？

刘书博老师：还是比较偏学术本质性的乐趣。

受访者：对，对

刘书博老师：探索和发现？

受访者：是这样的，如果你真是到政策层面，其实政策层面可能跟我们不是一个路子，他们就是有的时候就拍脑袋出来的。

刘书博老师： 对对。就很泛的那种，但我们就是一定要精准。有依据可循的那种。

受访者：对对，对对，

刘书博老师：但是我想问一下，就是你们这个经济学跟马哲就完全不一样。他们完全是文字的那种，那他们的合法性是什么？就像这种普世规律的寻找，那他们不要那种实证研究吗？

受访者：他们不要，他们做的都是这种逻辑的，还有对政策的解读，所以这个学科在中国现在看来，就这几年发展还是很快

刘书博老师：我觉得他更多是一种权力的辅助性的学术。

受访者：是的，就是政策的解读嘛

刘书博老师：所以我觉得那种学术本质性的乐趣是不是就少很多。更多是帮助权力在运转了

受访者：这个没跟他们交流过，我也不太看他们的paper

刘书博老师：包括我们现在了解的马克思主义哲学，马克思主义经济学，那其实它也是一种有点像哲学的···

受访者：有点哲学。对。对对

刘书博老师：就是你不能说它错了或者怎么样，它就是这种想法。

受访者：对，是的，所以他们更多的是哲学层面的东西

刘书博老师：所以我们可以把它们看作更偏哲学的。

受访者：是这样的，

刘书博老师：那你感兴趣的还是关注于真实现象的真实

受访者：对，金融呀，企业行为这方面

刘书博老师：那你会想要让研究更有社会影响力吗？就产生政策层面，甚至说对于大家观念认识层面的改变。

受访者：这个是我们追求的一个重要目标，但是很难去实现。

刘书博老师：那现在还主要是学术领域的对话

受访者：对，学术领域的对话多一点。

刘书博老师：ok

吕梦娜：您之前有想过就是把您的一些好的想法观点给更多的，比如说普通人，了解吗

受访者：现在在往这个方面努力，实际上这个是两条路子，一条路子呢是就是观点性的论文，不管是你在报纸还是在一些其他杂志上发表，都是一些观点性的，我觉得做这个观点性的论文的话实际上和实证的论文差别挺大的，他的要求并不低，你就怎么把这个观点表达地非常系统，用文字的方式表达非常系统，这个很难，现在在往这方面去努力吧

吕梦娜： 您前面说，就是相比之前同学，他们进业界嘛，觉得挺开心的，那您刚开始进高校的时候没有面临比如说“非升即走”这种制度性的一些压力

受访者：现在没有，我们那一批没有

吕梦娜：哦~还比较早的

刘书博老师：就直接进编了

受访者：但是我现在还是不太清楚编制和合同的区别在哪里。

刘书博老师：你说现在新人的合同呀

受访者：对

刘书博老师：新人他是要有一个考核，理论上讲他考核可以不过，不过就不续合同，但是我们就没有这个考核。

受访者：我们只是完成一定的工作量。

刘书博老师：对对，其实不完成也没事儿。不完成只不过说你收入层面受到影响，但是他们是有生存压力的。

受访者：所以就你看这种问题，就是我们感兴趣的问题就是编制的问题，编制的作用在哪里。这是一个非常中国化的问题，我那个合作者做的就是编制它的作用在哪里，发现就是编制提高了人们的幸福感。

吕梦娜：所以对您来说，做大学老师这条道路的选择，其实没有太多的不好的一些因素，比如说压力什么的，就刚刚您提到的评职称可能算是一个

刘书博老师：评到学阀

受访者：学阀的问题在于我们没有进入学阀集团，所以我没有这个问题，听说进入了学阀集团，他们过得很开心的。

刘书博老师：对，我们会成为利益的维护者。

受访者：对对对，这就是学阀的一个很典型的特征，就是在学界非常有影响力，我的导师在学界非常有影响力，但他不是学阀，他就自己一个人干

刘书博老师：学阀就是说它变成一种···

受访者：利益的交换，资源的对接

刘书博老师：它不产生真实的价值。

受访者：可以这么说

刘书博老师：有点垄断的意思就是

受访者：垄断学术资源，期刊资源的垄断，也是数据资源的垄断

刘书博老师：能说他是学术共同体吗？

受访者：他不是学术共同体。我们的学术共同体可以就我们几个高校的，可以组成一个学术共同体，这是学术联盟。但那个（学阀）是比较虚化的，他们的真正的是师徒的关系，这种师徒的关系在里边

刘书博老师：为什么中国有这个学阀呀？国外好像没那么明显，是吧？也有，但不像中国这么的嚣张

受访者：对，因为中国我觉得它是一个权力意识比较强，而且这种期刊或者是其他方面都是以权力为代表的。谁能干预了权力，谁就能垄断资源。所以在中国就是比较特别的···，但国外也有这种情况。学术的圈子，国内外好像都存在这个问题，

刘书博老师： 那你熟悉的这个学科领域在中国有什么特色吗，还是说你了解到国外的那个标准跟中国都是一样的，除了学阀这个。

受访者：国内的这种研究的话，怎么讲呢？

刘书博老师：它会有权力的干涉吗？就是除了学阀权力，包括政治权力···？

受访者：权力方面我觉得没有太明显，没有太明显

刘书博老师：就不说学术，就说咱们学校的管理，是不是咱学校管理就比较···比如说咱们了解到的，它是双线条的，一个书记、一个院长，似乎我们很多都是在···比如说在申请课题的时候都是学校给定义一个或者教育部定义一个，然后我们应该是朝着它定义的方向去。国外是不是也是这样？

受访者：国外这个我就不太清楚了，但我了解的，跟他们交流的时候好像这种现象比较弱，不太有这种问题，中国的就是给你几个选题，然后做重点选题嘛，是吧，这个重点选题可能来源于政策的需求。所以我们就会看到我们的科研是服务于什么的。所以这是整套的逻辑吧

刘书博老师：所以这一方面来说它也不是充分、完全自由的。

受访者：对，特别是申课题方面，我申课题申了这么多次，我一次也没中，我就感觉因素有很多在里边，其实这个评价体系，你说的是盲审，实际上是半盲审，他知道咱们是谁。所以这种在有些权力的配置下可能就会影响这种公平性

吕梦娜： 您刚刚提到说申课题这个问题，而且是国家定的，那会出现，就是可能您想要研究的、感兴趣的东西其实跟国家定的是不一样的吗

受访者：那这种情况是比较常见的

吕梦娜：那您会有意的就是靠近、调整下自己的研究方向，然后跟政策去···

受访者：这个是需要做一些调整的

吕梦娜：那这个申请对您很重要吗？对老师们很重要吗？

受访者：一个是象征意义。再一个就是确实有资金支持嘛

刘书博老师：而且它是相关联性的，比如说你评博导必须的。

受访者：对，对对，我觉得这个东西就···

吕梦娜： 就算是有利益牵扯吗

受访者：对

刘书博老师：合理还是不合理？

受访者：我觉得这个···就不管我是不是申请得上，我觉得都是不太合理的，因为这实际上是相当于一个条件或者一项荣誉来适应另外一项荣誉。你学术能力如何？实际上我们就是要简化这种评价体系，因为你评价越多了，指标越多了就越麻烦。对于这种事情一定要简化，我们既然有学术期刊的···

刘书博老师：所以中央说“去五唯”嘛，那现在我们似乎还是在具体层面还是“唯”，比如说你必须得有这个课题才能够评什么别的，还是有这样的一些限制。

受访者：是的，是的，这个很难打破，只能是慢慢改变

刘书博老师：我觉得这个可能就跟我们具体的这个学校学院的制度相关，有的学校可能不这样

受访者：这个好像现在学校里差别比较大，有的学校就比如说你要“破五唯”，之前不是给你奖励嘛，我直接不给你奖励了，就一刀切，现在就是分化比较严重，有的就一刀切，一刀切的问题其实也挺多的

刘书博老师：缺乏激励了。

受访者：对对对，其实这个事情我觉得得慢慢去改变。“破五唯”你破了以后怎么立？不破不立，你破了以后你怎么立。就是没有政策出口，没有政策出口的话那下面都是要执行的，对不对？执行的话那没有把手，他还是要按照这个来，只是形式有所变化。

吕梦娜：那关于“破五唯”，就是之前姜峰老师也提到过，有种“挣工分”的感觉，那么现在的话这种现象会有一些松动吗？

刘书博老师：现在有所松动，但是在这个组织感觉还是换汤不换药。

吕梦娜：老师那您工作的话，大概分哪几个方面？刚刚提到了教学、提到了做科研，还有其他的吗？

受访者：就参加学术会议，就参加会议，其他的都没有了。

吕梦娜：像刚刚您提到这种例行公事的会议多吗？在学院里面？

受访者：我是不多的。当然有行政工作的老师可能就比较多

吕梦娜：所以那就刚刚您提到这三块，大概您觉得时间分配是怎么样

受访者：教学算上备课的话40%，科研50%，剩余的10%

吕梦娜：了解。您刚才说其实本科是不带学生的

受访者：我们没有本科这个专业。但是有本科指导的学生，现在我们不是都是有导师制嘛

吕梦娜：所以上课的话也会给别的专业或者别的系的同学来上课。

受访者：对，基本就是一些全院的课嘛，就是那种会计学或者是金融学的课程

吕梦娜：了解。那您感觉教学的过程中怎么样？

（教学）

刘书博老师： 坦白来讲，从硕士到博士都没有一个正儿八经的课去教我们怎么去教课，是从站上讲台的时候你会发现，其实脑袋里是一片空白，所以这个过程是我们入职以后的一个学习的过程，所以就会发现像我这个前几年教学非常差，然后后来你就是去参加一些培训，包括了你一些自己的一些经验就是慢慢提高这个教学质量，所以刚开始几年确实挺有压力的，又要有科研还要学习怎么去教课。

但是之前也说教学其实是一个算是底线要求，就是不会给你很多明确的这个指示，你不达标又会如何如何，是不会这样子，教学就有一个底线，但是那个线比较容易嘛。但是我们面临着入职三年之后要评职称，这个是有一个就三年之后的事情在等着你。

吕梦娜： 就是评职称也是要看教学这一块儿的？

受访者：主要是看科研。

吕梦娜：那您想提升这个教学的质量还是您自发的想要去做好这件事。

受访者： 其实学校里也有一些制度保障，比如说提供一些培训项目、交流等等。

刘书博老师： 还是对于自己这方面有所期许，有些老师同事他们不期许，光利老师就是对自己特别要求高的那种，不允许自己有任何方面的疏忽，但有的就不在乎这个事儿。

黄秋莉： 我们上您的课都觉得你特可爱。

受访者： 这个有点儿，是吧？我很努力的就换回个可爱。

黄秋莉： 哈哈，大家肯定是因为很喜欢才会说出这样的话。

就刚刚老师提到您对未来有期许嘛，有没有想过就是40岁之后，60岁之后您成为在学术界成为一个什么样的学者？

受访者： 我现在对自己的定义就是很努力的人。但是天分来讲，其实我觉得做学术有的时候是需要天分，我觉得我这方面其实并没有那么好，所以将来我觉得我不会成为这个行业的领导者，但是可能就是有些成果在里边，然后做好教学就可以了。

黄秋莉： 有一种不忘初心、砥砺前行的感觉。

受访者： 对，就这个初心这句话确实要深刻理解一下，要是忘了初心的话，你就会觉得做这个事情比较无聊，

刘书博老师： 我们会看到有一些人就永远是做无聊的事情。

受访者： 但他们自己可能觉得很有意思。比如说我在社会上做一些什么东西我觉得很有意思。

（学术职业的个性化）

刘书博老师： 所以在这学校里，我们老师的发展还是要个性化一点。

受访者：对对，要个性化。

刘书博老师：所以那有没有感受到一些统一化的要求？

受访者：这个方面是有的，比如说我感觉现在对教学方面的要求有点过多了，就是设的条条框框过多了，其实教学它是一个很个性化的东西，每个学生都不同，如果你教一样的东西进去，其实效果并不好。

（AACSB）

刘书博老师： 就像咱们AACSB那个他总是要填一样的那种东西，进度也是一样的，这其实就是一种工业化思维、标准化思维。认证AACSB其实就是在让你去服从他们统一的标准。

说白了就是工业化思维，但是像我们会觉得大学教育他不是应试教育，应该以个性化为主，而不是标准化为主，对吧？

黄秋莉： 我想请问一下，就是我们学院做AACSB评估认证的时候我也参与了一些工作，我想说这个对咱学院应该是一个非常好的事儿哈？老师们怎么看这个事儿呢？我也不知道他到底在干什么，我只知道咱们学院有这么一件事情，但是老师您可能就知道比较多，您给我讲一讲。

受访者： 我确实也理解不多，AACSB它是一个对商学院的认证，就你有这个东西之后好像是大家就认为你这个学院的质量高了。就是你现在看每个学院就感觉认证这件事是一件特别大的事情，就是我们大家对这个事情是认可的，就感觉认证后就进入到一个核心圈子里了。

刘书博老师： 然后有老师会认为这个认证相当于给学院找到了一个院士，就相当于这个东西分量是一个院士来了的影响一样大。

受访者： 好像如果进入这个组织的话，学生出去交流的话更方便，比如说互换，然后对方说你是这个标准，那我们标准一致，那还可以做。所以他可能是一个圈子、一个平台，进来以后我们可以对话。因为很多老师名片上面都会印上获得认证的信息。

刘书博老师： 认证这个概念，你们可以查一下，最早是iso9001这种工业生产的质量认证。但是把这个概念移植到教育行业，那这个适不适合？就是我们是不是生产加工产品那样的标准化产品，还是说我们应该有一些个性化的东西？

黄秋莉： 每个国家都有自己的特色。

刘书博老师： 所以你们就可以用后殖民主义理论去思考一下，现在这种霸权它就是通过知识标准和组织管理标准来实现，他对商学院具有同化的作用。

黄秋莉： 我就想说对咱老师个人呢？因为我们那个时候收集了很多材料，好像还课程之类的，对老师个人有什么影响吗？就是这对于你有什么要求吗？

受访者： 我觉得他对我的要求可能实质上没有，但是他对我的这个教学方式具有理念层面的影响，我觉得AACSB认证让我看到了教学的另外一个方面就是要标准化，标准化是要求这样做的，在这个过程中可能会对我们这个教学形成一些这个思考层面的冲击，

黄秋莉： 会感觉自己像一个小螺丝钉一样，他让怎么转就怎么转？

受访者： 那倒不至于，我们怎么转他们也不管，他们只是让我们填表，但是填表的过程可能我们在想，这个目标到底实不实现，怎么去实现，需不需要做这些改变等等？

刘书博老师： 说到这个认证，第二个它就是一种window dressing，就是打点门面或者是给你打个商标那种，然后让你的品牌价值、符号意义更高，但其实在落地层面没有真实的改变我们。

黄秋莉： 我记得我们还确定了学院新的愿景使命，所以其实没有实质性影响，对吗？

刘书博老师： 没有太大实质性影响。但是他会要求对老师进行分类评级，不过现在也没有那么强烈的去执行。

就把老师分成几类，一类是比如说你这两年没有发表论文，那你就要分成那一类，就有点像二等公民似的，是这个分级。

受访者：除了教学的标准化，他方方面面都有，实际上他们对这个组织任课老师的结构它还是有影响的，有些老师就是提前退休了。

刘书博老师：对，所以他是一个很强管理主义色彩的一个标准。

黄秋莉： 有一些老师还被迫提前退休了？

受访者： 因为那些像我们不同年代培养老师，他岁数大一点可能就做不了研究了，而且他们硕士毕业就来工作了。所以他也没有办法在这个标准下生存。所以这种制度一经落地，他们几乎就要走，他们受到的教育跟今天相比是比较的差的。

黄秋莉： 这些老师成长于不一样的时代。
另外，AACSB其实是给我们带来了更多交流的机会是吧？

受访者： 但是疫情来了，潜在机会是有的，但是疫情来了。

黄秋莉： 老师您在提到人大给您的平台的时候提到了交流会比山大更多。对，这个交流是指国外的还是说学校内的呢？

受访者： 各种方面就是，你看之前山东大学生是一个综合院校呀，对吧？那经济金融可能就在这个学院，但是来到人大以后，它有各个学院都是涉及到这一点，所以会有更多同行交流的机会。另外其实学院资金还是挺多的，学校资金挺多，它可以邀请一些海外的人过来讲课，短期的比如说两周什么的，其实那个我觉得还挺有收获的，但现在好像越来越少。

吕梦娜： 我其实听您说到很多同行交流，您说同行交流是什么意思？

受访者： 你是说学生时代还是现在？

吕梦娜： 无论学生时代还是现在都可以。

受访者： 现在的同行交流主要是参加学术会议。

黄秋莉： 就是还是跟自己同一个专业的同一个领域中的？

受访者： 有时也不见得是同一个领域，反正是有的做你相关的研究了，你就进入这里边看一看是怎样的。

吕梦娜： 会有跟比如说咱们学院或者可能跟你不是一个专业的，然后这种同事之间的交流怎么样？

受访者： 挺多的。特别商学院这个交叉性的机会还是很多的，因为我们的专业可能差别比较大，但是真正的交叉起来比较难，我们有兴趣去做，感觉这个不同领域来谈一个话题，我们可能有一些新的想法会出来，但是真正的到他怎么去做的时候，我们可能就还是在各自领域里面去用自己的方法去做，但这个交流是非常好的。

吕梦娜： 因为我听说就是有一些年轻的老师可能压力比较大的情况之下他就会担心，比如说我交流把我自己的idea早期给透露出去了， 这种就你有没有听说过？

受访者： 这个担忧是有的，这个担忧是有的，但是可能聊到共同话题的时候，有 这个共同点的话就一起做，所以我觉得商学院整个同事氛围还是比较不错的。

刘书博老师： 你有没有了解过商学院不是特别好的一个样貌？

受访者：那是有的。

刘书博老师：就是什么有些什么不好的样子？

受访者：比如说为了争领导岗位，那就是相互接黑底。

刘书博老师：ok，为什么要抢那个领导岗位？

受访者： 怎么讲呢？这个还是对权力的欲望吧。

刘书博老师： 不过学院的leader和政府机构那种局长还是完全不一样，我们还是学术专业的一种标准嘛，有些学者学术水平高，他可以不鸟院长。

受访者： 但是也有一些人就是存在一种想去当院长这个倾向。

刘书博老师：因为啥？因为热爱权力？

受访者： 热爱权利其实这个是有的，因为院长这个东西这个职位在出去交流的时候还是挺有什么的，挺有圈子的，你比如说院长不管是山大还是央财的院长，那多少是一个院长。所以在中国文化下这种职位可以带来一些资源？

黄秋莉： 就是比如说去参加会议的时候听你说是个院长会主动给名片？

受访者： 这是很小的一部分，但是你像比如说在这个省里评这个省的优秀人才项目，如果是一个院长的话，你的分量就会大一些，你可以融入的资源多一点。

刘书博老师： 这就是中国文化的一种对于这个职位的一种优待，大家都很会来事儿，就自动的把资源放上去。

受访者： 是的是的，你不去争都会有，所以在各行各业都这样，单位的一把手都是挺让人（歆羡的）

刘书博老师： 你应该不太感兴趣这种吧。

受访者： 我现在还没怎么感兴趣。

刘书博老师： 张老师很严谨，未来可能，哈哈

受访者： 未来当我有想法的时候可能就过了那个年纪。

刘书博老师： 刚刚你说博士毕业的时候不是考虑去业界，后来为什么没去银行或者金融圈？

受访者： 主要是我喝不了酒，这是真的，我一滴酒不沾，所以他们经常喝酒的话我就没办法。

刘书博老师： 你说喝不了酒是身体原因还是说有其他原因？

受访者： 真是身体的原因，我喝完会特别难受，身体上会起很多红点，这算是酒精过敏，去医院检查了，也没有大问题，反正医生就说你别喝酒就行了。其实这个问题在学术界也是这样，就大家还是有这个酒文化，这几年可能好一些了，之前酒文化是非常深的，有一种观点就是说这个酒量越大学术做得越好，从观察来看就是这样的，因为可能通过酒形成一种人际关系嘛，所以更容易出成果。很庆幸这几年酒文化越来越弱，

黄秋莉： 以后希望能流行起喝茶。

受访者： 喝茶可能没有那个氛围，酒和茶还是有很大的区别的，酒会让人在一种放松警惕的状况下展示自己真实的一面。

刘书博老师： 酒是一种交流的催化剂。

受访者： 喝完酒之后大家更容易坦诚相待。

刘书博老师： 对，喝茶可能是越喝越清醒，然后两人还可能开始争吵，哈哈哈

（学阀性团队）

黄秋莉： 老师您前面提到了学阀性团队，您能更深入地给我们讲讲吗？

受访者： 学阀不一定就是指院长这类当官的人，学阀肯定是有比较强的学术能力，因为整个团队是基于师徒带领大家一起生产学术作品，出论文的速度会非常快。如果没有学术能力的话，整个团队也凝聚不起来。

刘书博老师： 这能不能说是一种工业化生产模式？

受访者：只能说是一种小作坊。

黄秋莉： 这种现象是一直都有的吗？自古至今一直都有吗？

受访者： 我不知道是不是从古至今，其实你看到现在这种发paper的现象其实也没有多少年，多少年之前大家都不去做研究的。

刘书博老师： 你可以把学阀性团队看成是一种对于资源的垄断，或者是资源的把持，它不是对外透明、可以充分竞争的状态。

受访者：对，学阀性团队就是一个对资源的把持。那古代也有呀，那只不过当时的学术资源不是期刊，但还有别的资源。

刘书博老师： 你认为做研究是体力劳动还是脑力劳动？

受访者： 现在是越来越脑力劳动，体力劳动的话，因为一些基础性的工作可以找助教，可以找自己指导的学生去处理就可以了。

刘书博老师：你对于有一些老师认为学术活动是一种体力劳动的看法，你怎么看？就有一些同行，他们会觉得学术就是体力。

受访者： 做学术，体力是必须要付出的，但更多地必须要有脑力的，体力是一个基本条件，所以要坚持锻炼。

刘书博老师： 但是你会觉得学术工作更多是脑力劳动吧？

受访者： 对，更多是脑力劳动。

吕蒙娜： 刚才您提到指导学生，现在博士生导师和博士之间存在的一种关系就是一种老板和学生的关系，就是学生称呼导师叫老板，您自己有这么称呼过自己的导师吗？您怎么看待这种现象？

受访者： 我自己不称呼我的导师为老板，这种现象在工科里比较比较明显，

因为工科里都是给你工资的，然后可能还接外面的活儿，就有的导师本身在外面就成立了一个公司，本身就是老板，所以这是称呼是从那个演化而来的，我们管理学科好像还不太明显。

吕蒙娜： 其实我也有听说浙大的管理学院，他们好像还比较倾向于称呼老师为老板，但其实他们也没有太多的项目，就是自己做科研，但是也会这么称呼。

刘书博老师： 就你带学生会不会特别的标准化？

受访者：现在我还没带几个学生，所以标准化起来也很难。

刘书博老师： 你想标准化吗？

受访者： 我不知道标准化能带来什么。

刘书博老师： 标准化是指就是麦当劳化，效率会大大提升。

受访者： 那不会的，那不会，这种标准化就是相当于我给一个主题，几个人分工，比如说你去做数据，你去写什么，那这个对于写文章不太行，因为你这个文章是前前后后反复修改的一个过程，几个人分开写那样不会形成好的研究， 还是一个人一个主题的去做，因为学术工作还是个性比较强。

（理论和实证）

黄秋莉： 老师，您之前谈到这个管理学是不是有偏理论的研究？在您这个职业过程中，有没有一个从理论转向量化的一个过程？还是说您一直做的都是量化的这种研究？

受访者： 我觉得反正经济金融的理论都比较简单，之前我们学过很多理论，我觉得大理论都基本做完了，就是现在我们学科里分为两派，有一派就是专门做理论的，他们理论就是什么呢？比如说我用博弈论的模型去做，我用这个求均衡的方法去做，这种求解求均衡我也做不了，而且就那个感觉确实兴趣不是很大，因为他那个主要是你在一个领域中持续的去做，持续地研究这个问题。比如说有一天你想放送某个理论假定，这样就又出来新的东西了，他们那个对现实的观察比较少一点，所以我现在主要是做实证这方面的研究。

黄秋莉： 这种实证的方法您是自己学习的吗？实证应该也是一个从国外引进来的这么研究范式，应该流行没有多少年的感觉。

受访者： 我感觉没有，在这个经济领域，实证本身就多一点，年数久一点，但也没有多少年，确实没有多少年。就现在对实证的批评有很多，比如说有人认为你拿着两个变量就是做实证，但是你会发现好多研究它肯定不是这样简单去拿两个变量去做的，就你看今年的那个诺奖的获得者，他们就是实证，因果推断那就是实证主义，所以这方面好的研究，我觉得不管你去做理论还是做实证，其实好的研究都是不易得的，都是需要一些规范性的东西在里面。

就实证主义来说，你像怎么去选变量，怎么去做研究设计，它都是要有比较强的合理性在里面，不是说你拿两个变量来进行调一调，就不是拍脑袋的就能行的。

（好的研究）

刘书博老师： 那你认为什么是好的研究？

受访者： 好的研究，这主要是通过对比，好的研究，你是觉得读完以后确实感觉挺让人佩服的，然后也恍然大悟的感觉。好的研究是一看能看出来的，差的研究有很多，

黄秋莉： 您现在觉得咱们这个学术生态差的多吗？

受访者： 挺多的哈。

吕梦娜： 能冒昧的问一下您对自己目前做的研究是一个怎样的评价？

受访者： 我觉得总体上一般的研究比较多，同时存在一些差的研究。

吕梦娜：为什么？是因为比如说当硕士生的时候自己的能力还没有达到现在这种程度，还是说因为什么别原因呢？

受访者： 我觉得做研究真的是一是要靠天分，第二是靠你在这个行业里面你自己的积累，就是有些问题，比如说几年前同样的问题我再去做，那我现在的角度肯定和之前的不一样，这个东西我觉得跟这个道长修行是一样的，就是这个你看顶尖的学生他们做的研究就和我们的就道行不在一个层面上，所以就是得慢慢积累。

吕蒙娜： 所以其实这样的话对您来说学术是一个不断成长的过程。

受访者： 对，

刘书博老师： 对，这是一个成长，成长过程就是你喝酒喝多了，你能品出来酒的好坏是一个道理。

（“土博士”与“洋博士”）

黄秋莉： 张老师，咱好像有土博士、洋博士的区分，这种区分现在还严重吗？

受访者： 这种现象的话我觉得现在依然很严重，之前是形式上的严重，现在是本质上的严重。
黄秋莉：怎么讲？

受访者： 因为现在好多海外的学生回来以后你会发现，你跟他竞争的时候

不在一个层面上。

吕梦娜： 但是也有比如说欧洲回来的学生没有发很多论文，他们可能觉得还竞争不过国内的。

受访者：但是怎么讲呢？现在之前是好的学生不回来，现在学的好的学生有的回来之前他们就有两篇或者一篇的文章在手里，是要发出来的。

吕梦娜： 这种更多说的是美国留学回来的吧？

受访者： 英国和美国的培养体系确实存在不同，我觉得目前来看，从我们这个领域来看美国的学生实力更强一点。

（与海外学者合作）

吕梦娜： 也因为我们照着美国来培养嘛，所以说人家比我们更有那个先天优势。似乎发英文的文章可能会相比于中文更有些难度？

受访者：好的期刊会更能，一般的期刊也都没什么的。

吕梦娜：那你要发好期刊会就是比如说找大牛合作一下或者是怎么样？

受访者： 可以，但是大牛不是说你来我就跟你合作了，大牛一天有多少好的idea等着他们，现在都是给他供稿，真是这样，只是人家不想跟你合作而已。

真的，如果有能力跟“大家”合作，这是肯定要做的，因为之前我跟他们就不能说大牛，反正也是国外的有好多发表的那种学生去合作。我觉得其实在这个合作中能学到好多东西。

吕梦娜： 所以跟大牛合作更多是想提升自己，而不是其他的原因？

受访者： 当然也希望直接能发表了，因为你看这个圈子这是一个很明显的现象，你去过看一下目前中国人发的国外的顶刊，基本都是一作的很少，基本都是由国外大牛带着发的，这是一个公开的现象。

吕梦娜： 你认为供稿这种合理吗？

受访者： 当然不合理，但是我们没有办法。

吕梦娜： 那我们为什么要可着所谓的顶刊去发呢？

受访者： 因为我们学科建的晚呀，我们的学科都是别人的东西，是一个西学东渐的过程，我们是晚辈，像我们那个西方经济学翻译过来的时候人家都用了多少年了，然后我们中国人才知道什么叫供给、什么叫需求、供给需求，包括这个阶级剥削、异化这些概念都不是中国人说的，都是老外说的。

刘书博老师： 我们认识自己世界，认识中国社会的观念，很多都是外部学说构建起来的。现在管理更是那样，几乎百分之百，

受访者：你们管理学不是强调要做中国的管理吗？这就是因为正经从学术来说，这个学科没有中国人构建的东西。

不过我想说就企业的经营来说，中国的企业经营和国外的企业经营管理方面有什么本质的区别吗？

刘书博老师： 要说没有也真没有，要说有也有，就是说我们完全可以构建一套新的概念，自自成其说的这种，但是老外可能会觉得你这个已经晚了，我们这个学说可以完全解释你们是这样，这个也是这样，是吧？所以其实还是后发的劣势嘛。然后这种霸权现在已经成为一种完全的学术标准的形式在被广泛复制了，然后我们就很难去弄。

受访者： 但现在在经济和金融领域有一个有利的方面，就是中国从那么一个贫弱的国家在短短几十年中迅速成长起来了，所以国外的研究是特别感兴趣的这方面的，即关注所谓的新兴经济体。因为现在西方人解释不了中国的这种现象，因为之前西方经济学都是市场的东西在里面，没有政府的东西在，现在有个市场这么差的一个国家突然涨起来了，我（指西方）怎么去解释的？所以国外是有这个需求的，想去看看怎么去研究它。

刘书博老师： 那也需要一套比较宏大的理论，但是现在的方法都是用国外的方法，用的都是国外的理论框架，那所以就很难弄嘛。你像我们现在很难出来一个像哈耶克或者凯恩斯（那样的学者）。

所以我们的源头上还是同一的，哈耶克、凯恩斯很难出现！而且我们现在学术这么制度化，就是我们还是论文导向，谁也不会说我写一个皇皇巨著去系统的解释一下为什么有这种宏大的社会差异。

受访者：对，我导师就写了一本非常厚的书，叫《制度金融》，写了好几年，其实我觉得他的创新性很大，但是也没有掀起什么波澜。

刘书博老师：他知道为啥吗？他有说吗？

受访者：他也不知道，他是解释中国的问题嘛，中国的制度经济学，所以老外也不认识，他只有汉语版，他就只发了一本书。

刘书博老师：那你给他弄成英文的投出去多好。

受访者： 这事儿我到现在毕业几年我都没读完，这个太厚了。对，当年他的状态就是真去国家图书馆里去复印资料的。

吕梦娜： 老师您除了发论文，还有其他比如著作等成果之类的吗？您会写书吗？

受访者： 我觉得很有必要去写书，

刘书博老师： 但是你也不写呀。忙吗？

受访者： 不是忙，是能力不行。现在我去做实证我觉得容易，但是写有观点的文章对我来讲很难，你如果把这个观点放到一本书里，再去详细阐述的话，我觉得太难了。

刘书博老师： 所以就是学术制度化。就制度它是以期刊发表为导向。

受访者： 如果现在说你必须要写一本书，那我也能写出来，但是写的质量可能就是凑起来，但是如果没有制度压我的话，那我就等等看吧，等什么时候想写了、就有东西写了再写吧，没有东西写你也不要想写。

黄秋莉： 老师，您讲之前和国外学者合作能学到很多东西，能讲讲具体您觉得学到哪些方面吗？

受访者： 第一个方面就是研究视角，就是这些人他有一些好的发表之后，他了解什么样的研究视角在国外可能更加有吸引力。第二个就是研究方法，（国外这些学者的）研究方法更加规范一点，就是包括数据怎么呈现、怎么去展示、怎么解释等，当然包括语言方面。

吕梦娜：您觉得视角更重要一些还是方法更重要？

受访者： 第一个是这种视角，这个是考验我们智力这个，包括研究设计，怎么去把这个故事讲出来就完全不一样，这个东西还有基本功，然后就是积累。

黄秋莉：那您说咱土博士和洋博士不在同一个竞争层次，也是讲这种视角、规范性之类的吗？

受访者： 研究视角、规范性以及学术圈，国外的学生在国外建立了比较好的学术圈子。

黄秋莉： 那您是就国际层面竞争而言吗？

受访者： 国内也是需要，就即使学校里不去给你奖励，但是如果你发一篇顶刊的话，在我们同行里面我们就会知道你做的特别好，你是牛人。

吕梦娜： 那您觉得就是以您目前发的文章来看，你觉得好的文章是发在更好的期刊上吗？是一个成正比例的吗？

刘书博老师： 对，目前是，70%~90都是这样。

吕梦娜： 那因为你做的研究一般是自己感兴趣想要去探索的嘛，有没有那种情况？就是你写完这篇文章之后，评审人给你的意见可能跟你的想法不太一样？

受访者： 很多情况都是这样的呀。

黄秋莉： 那这样的话你会尊重评审人意见还是会遵从自己内心的想法？

受访者： 60%是要屈服的。但是屈服的不多呀。其实审稿人他也不是有明确的（指令），就会说我认为这个应该这样做，他不会让你这样做的，好的审稿人才会这样，一般的审稿人就是怼你，乌七八糟的怼你一顿，那只好屈服，只好认同，然后就想办法去改。就是其实也不是遵从他的意见，他不会给你提出一个明确的修改方向的。

吕梦娜： 你改完之后还满意吗？

受访者： 这个也很难讲，大部分的情况是满意的，因为审稿人他不会给你明确地说怎么去写，他只能是去挑毛病，那挑毛病就会激励你再去做思考，你需要按照更加严格的标准去做。

吕梦娜： 我听说答复信这一块儿，可能有人写的就非常非常长，甚至超过了自己的正文部分，你有这种经历吗？

受访者： 我一般不会有自己的正文那么长，你如果写这么长的话，审稿人也会烦的。就是审稿人的意见要好好对待，不管他的建议中肯不中肯的都要好好对待，然后要想尽办法去解决，如果解决不了的话只能去跟他去辩论，有十个意见你可能改了八个六个，一般都没问题的，有几个你跟他讨论的话没问题。

也会遇到态度极其恶劣的，就是会出现怼人这种情况，反正今年评审课题的时候遇到了一个，态度特别差的审稿人，他给我特别负面的意见，我估计是一个同行。

今年审的好像跟我们那年不太一样，就是你是20级吧，他把它放到21里

去评，就有一些是这样跨学科的今年是20年还是21年做的改变，那同行就怼的很，他怼的理由关键很…… 怼你没有什么，但是说你什么研究基础上文献没做好

刘书博老师： 就语焉不详，是吗？

受访者： 一般就是这种语焉不详的，没有确切理由的话，一般都是同行，和他做同样的问题了，他把你先摁住。

刘书博老师： 我我一直都是收到这样的意见，就是大概他不告诉你具体哪不好，就一句很概括的，研究设计不合理、思路不明确、研究主题不恰当之类的。

吕梦娜： 听完这样评论什么感受？

刘书博老师： 我就把电脑砸了！

但他其实好好对待也没什么，他给评论说明很正常，但是你要接受不了，你就拒了，但他还要把你恶心一把。

黄秋莉： 目前说是要把论文写在祖国土地上，您如何看这个号召？

受访者：  我们始终是没有在天空上写，就是在大地上写。

刘书博老师： 号召性的东西可能有20%能落实就不错了。

黄秋莉： 那这种本土管理学研究老师您觉得咱一直都是在做这种本土管理学研究，那对于咱们这种号召……

受访者： 我们是做本土管理学研究吗？不太清楚。

刘书博老师： 他号召的意思就是他其实是不满意我们现在只是按照他们那种学科的评价标准，他希望我们能构建出来像你导师的那种新的体系。

受访者： 就我们现在经济增长起来以后，我们将来成为世界的第一大国以后，我们在这方面的话语权怎么去做？我们现在需要思考了。我们始终跟着别人的话语权我们肯定当不了。

刘书博老师： 对，所以就是其实咱们刚才聊也聊出来一个矛盾点，我们的经历都是被制度化的期刊导着走，但这个标准是西方构建的，那我们现在的精力和动机也没有真正的有像写书这种去构建一个自己本土的理论体系，似乎还是没有。

受访者： 就是怎么讲呢？就是知识这个东西你很难评价他是好还是不好。你不像足球是吧？文无第一，武无第二，就是这个什么是好的不太好明确判断，所以你看知识它特别容易自成一体，形成一种自我的、内生的合理性的标准。所以我觉得教育学里面提到的还得相信人、给人充分的自由才可以。

刘书博老师：是的，所以如果太标准的话，知识背后是有权利要表达自己，复制自己，那我们怎么去克服这个事儿这是一个问题。

刘书博老师： 我们再问一个问题，差不多就可以结束了。

吕梦娜： 老师，我想问一下，您对自己的研究整体上是满意的吗？因为我听说其实有很多老师，包括学生，博士生对自己写过的文章不想再看第二眼。

受访者： 大部分文章就不想再去看第二次，但有的文章还是要看，可能我刚才说的那个好的研究，还是可以再看看。特别是看在这个问题上，你如果再写新文章的话还是要看大。但像那些一般情况的，我就不会再去看，因为自己研究的什么东西自己也了解了，

吕梦娜： 不会觉得自己写的很好，再去欣赏一下吗？

受访者： 不会，不至于像那种文物宝贝一样孤芳自赏，不会的，但是说实话，有的时候比如说这个类似于随笔、日记那种情况，我有的时候还会反复看几遍，就是觉得还不错，还会让你想起一些事情，但研究的话你怎么去研究的和你做的东西基本都了解，没有必要再去回忆了。

黄秋莉： 所以是因为了如指掌，所以不再去回忆了。

刘书博老师：确实这个事儿（论文）有时候特别制度化，就很标准化，完全缺乏一种自己随意、生动发挥的空间，你像写日记有优美的文字，有描绘很好的场景，论文表述一定是那种金刚直男的那种表述方式。

访谈感受

刘书博老师：

张老师是个比较追求学术价值的人，不是那么社会化，他更多是追求一种学术的真标准，而不仅仅是一种达标的标准，比如他多次强调学术成果的价值。

他有一些比较轴的地方，对于学术管理标准有异议，也不会强调现实的合理性，对其做解释。

有的老师对现状的合理性解释就很凸显，只要看到不好，就首先是给不好进行辩解。

黄秋莉：

张老师是一个非常友善的人，我能感受到他愿意和我们交流，并不排斥我们的访谈，就像谈话结束时他说的那样“能有这样的谈话挺好的，平时也没有机会说这些”。

他在交谈中的表现与和我们上课不太相同，上课期间略显不自信、准备不充足、知识掌握不全面，但在访谈过程中，我发现张老师是有自己的“初心”和“原则”的老师，我觉得我们是一类人，知道自己天分不够，因此也就踏踏实实、勤勤恳恳、注重积累。

他是一个能够按照自己方式去达到标准的人，既能遵循自己做学术的原则，不太会去做一些偷奸耍滑、投机取巧的事情，如“灌水”等；但同时也在努力向学术标准靠拢，知道标准是什么，也知道如何达到标准，除了个人努力，也不排斥通过合理的方式、在不触犯学术原则的情况下去达标，比如和国外学者的合作。

我们谈论了很多问题，从他本科、硕士、博士以及现在的研究经历，到现在的学术工作。

# 受访者25

刘书博： 那你们先来吧。彭老师经历很丰富，就刚才说的他在这个实践界也就工作很久，然后又到学界，所以他的这种对比的视角能够帮助我们就跳出来看管理学。那你们开始问吧

黄秋莉：您本科是学什么的呀？

受访者： 我本科学过两个专业，一个是马克思思想政治教育专业，另外一个文学中文

黄秋莉： 为什么会有两个专业？

受访者： 因为当时我的本专业是思政，文学是我那个二学位。但是我其实思政呢就上过一门课，其他课我不上，因为我们那会儿上学1993年-1997年，大学管的很松，根本没人管，我就上过一门课，其他的就没听课。所以我其实大学四年呢看文学书、参加文学活动更多一些。那么说怎么进入这个管理学的？首先讲讲这个故事很好玩。第一，因为毕业之后拿学位两个···

黄秋莉： 大概是什么时候（毕业）？

受访者： 我1997年大学毕业，本来按照本专业分的话已经分到去当中学政治老师了

黄秋莉： 那个时候还包分配工作？

受访者： 那会儿师范包分配，现在也还包分配，像当时我就不想那个，因为这个中学老师，第一，这个活儿的话一眼看到老。这是第一个，另外一个呢政治学的东西、思想政治教育的课不太好教，因为有些东西其实自己也未必真认可，或者真吃的透，所以不太好教，所以我的这个工作就没干了，我就大学毕业去那个学校，就去了一个月，分到一中去了一个月，我就给学长留了个辞职信，我就不辞而别，我就下海了。下海你想到企业找工作···

黄秋莉： 那时候下海是这个社会氛围好吗？1997年的时候

受访者： 1997年那会儿其实还好，当时我去上海找工作，为什么去上海，那个比较有趣的，是因为我在大学的时候看书看报纸看到过，就是有一句话，反正好像是《中国社会报》上面一句话，中国这个政策的话，一般会从两个城市出发决定的，一个是上海，一个是北京，所以我想···因为当时我们上学时候是1993年嘛，1992年，你知道这个邓小平南巡讲话开发浦东，那正是九几年那会儿上海开发正热的时候，所以我就先去了上海。

去上海你要找工作，那么你这个学政治教育的或学文学的，那么别人看你的话，你找工作在企业里面，你说你学营销的，人家可能让你去做营销；那我又不是学技术的，就是学财务会计的，那也找不到会计。那就是在这个企业里边儿刚去的时候找工作，你可能别什么都乱找，所以可能主要找人事工作，那会儿叫人事还不叫人力资源。因为人力资源的工作是一个后面才来的一个概念。那会儿所以我先去了一个公司，一个上海一家外企做人事工作。人事呢，我那会儿去更多做一些绩效或一些企业文化建设这一块东西，那实际资本也不知道啥是企业文化建设。

那会儿有了（工作）之后，就去外面买一些书，我这个商学管理学教育是我自己这个边工作边自学的，那么这个当时呢其实有两套书就比较有意思，一个是MBA教育刚开始，官方的教育部的那个是97年第一届招生，我97年刚毕业那会儿正是MBA推广正热的时候，所以不知道是什么出版社的，我也记不住了，出来红色的一套就是哈佛那个MBA教科书，我把那套书就买了之后就是学了一遍、看了一遍，那个从核心课看了一遍，这是一方面。

另外呢还有一个公司现在肯定不太出名了，叫派力培训，它出来一套书，我把他的相关的书买了一些，像企业文化呀、商务礼仪呀，这些相关的东西，因为以前我们学这个思政的老师不可能学这些相关的东西。所以我在这工作呢完全就是自己边工作边自学，学完之后在上海工作三年，主要做人事工作，那这个招聘、培训、绩效都做，所以我在上海三年从1997年待到2000年，当然上海其实那个时候发展很好，但是我在那儿的话，因为什么人都不认识，就一个人，没找到特别好的发展机会，觉得也没有什么成长的感觉，实际上是刚刚进入社会嘛，没什么成长，也没什么劲，觉得没什么意思了，觉得这个上海城市文化跟自己也不是特别匹配，因为上海这个文化就是说他很理性很实际。那像我这种还有豪放气派气质的人在上海待的就觉得很委屈，加上上海到了秋冬两季，梅雨，这个雨下得人心发霉。所以我呢比较好玩，我在2000年6月份领完一个工资，我就买了火车票，从上海坐火车买了个硬座坐到乌鲁木齐，我在新疆玩了一两个月

吕梦娜： 为什么选择乌鲁木齐？

受访者： 那既然在上海在全国最发亮地方待着不开心、不愉快，那就到最原始、最落后的地方去看看嘛

黄秋莉： 你也是一个非常浪漫的人。

受访者：对，那是年轻的时候，很多人都有这个过程。所以当时我就从上海买火车票，那一会儿从上海到乌鲁木齐要72小时，我到乌鲁木齐那边石河子呀、克拉玛依、吐鲁番、楼兰等地方都去过，玩了两个月。其实那会儿钱也不多，那会钱更便宜，所以我的话就是玩了将近两个月最后发现就只有不到300块钱了，我就买了张火车票到北京西站，北京西站下车，我下火车就是56块钱，到北京照样是什么人不认识

黄秋莉： 还剩两百多块钱吗

受访者： 也只有56块钱了，你买张火车票路上还要吃点东西嘛，到北京下火车就是56块钱，那到北京那时候来讲的话，上海的人力资源工作也不太愿意做了，觉得没什么意思，所以当时呢在北京的一家公司叫群顾问，一家做医药行业培训咨询的。那个公司虽然说现在看好像不是特别有名，但那是97年成立的公司，是全国第一批咨询公司，他当时在培训行业、咨询行业算比较早的，那第一批咨询公司那老板也还算在当时是比较强的人，我在那儿帮做培训工作和咨询工作。

我大概可以说分三块儿吧，40%的工作是做企业培训，做内训和公开课，就像培训班那种；再有30%的话是做这个咨询工作，然后做咨询项目顾问，做那个书、调查些东西；还有30%做什么呢？做一个杂志，就是他们有个内刊，内刊他没有刊号，但是整个是像那个医药行业这些，我当时开始是做那个责任编辑，后面是执行主编

吕梦娜：实际上做的是很专业的工作

受访者：对，对对

吕梦娜：但您当时没有那个学位其实，没有管理学位

受访者： 没有，他就是因为你学文学的嘛，因为我在大学期间我做过学生报纸那个社长总编辑，因为这个学文科所以文字还不错，我也一直保持练习，所以他让我做这个工作，我做了三块儿，三块儿做了几年，那应该说我是那几块儿的话就成长的比较快。你想当时接触的那些公司的那个咨询顾问和培训老师都是中国第一批外企的职业经理

黄秋莉： 外请的？

受访者： 大部分外请的嘛，那些人就是在本职工作之外，在外面人愿意挣一份钱，那他第一批能够成长的，当时像外企的总监呐、营销总经理、人力资源总监、财务总监等，可以说代表中国整个当时职业文化第一批，所以通过那几年跟他们做培训这一块儿，因为我经常去做培训助理嘛，像我们做培训助理，大部分的培训助理把那个培训会场那件事儿弄完了就出去玩。

黄秋莉： 那您就听一听？

受访者：当时的话，教材不像现在，而是要印纸质的培训教材ppt，一般我会要求多印一份儿，因为去的话就是那边一个课老师讲过，还是说其他老师也讲过，我都认真听下来，培训老师讲，刚发现课堂里边儿那个培训学习最认真的就是小彭，就是我当时等于在那儿工作，从2000年到2003年，那三年时间来讲，对于我来讲是完成了一个商科的教育，他们那个时候因为培训的话，就要掌握一些东西、就要看相关的书。我当时做了一个比较好玩的事儿，一个就是经常到企业培训管理，那为了学这个课，我是把整个北京市当时所有的新华书店、王府井书店所有的员工培训、企业培训所有的书买下来看，我大概应该买了一百多本，整个都看完了。同时把当时很多报纸，包括《北京青年报》，还有个《前程无忧》51job有个报纸，他们所有关于这个培训的所有的文章剪下来，剪下来我就读，读完之后我是做成ppt，我一个人其实做了一套企业管理培训的从课程开发呀、管理系统、讲师培训整个这一套，我做了一百五十多页的一个ppt，因为这一块儿呢等于是算在培训管理领域，其实那几年的话就变得有点儿小专家这种味道。

那么同时我们做培训实际上不仅仅是做培训助理。培训都是在周末做。那么周一到周五干什么呢？做销售、卖课程，我呢就是2000年去， 8月份就入职了，我去入职半年，我干了一个好玩儿的事儿，就是我半年成为公司年度销售冠军，所以当时我们公司的那个老总他就说小彭你那个没干过、你没学过营销，居然那个销售能够最好，你就是销售天才，当时跟别人说怎么干成的，我说很简单，第一不怕，就当时打主要打电话销售，我管他是谁，董事长也好，总经理也好，总监也好，只要这个人我想找他，不管什么时候我直接打过去，我们那个同事经常先打座机，我不管，就打手机，手机找不到的情况下我再打座机问问试试。因为你要找的是重要人物，首先有什么最快速方式找到，这是第一位的，所以我当时比较生猛，因为什么都没学过，不会买卖，没人教过我们做营销，所以我半年就成为销售冠军，那公司2001年培训业务是700万，你想每个顾客才卖三千多块钱，那总共卖700万那是很大的，我一个人完成280万，当时他们觉得很诧异，想我怎么完成的？那个公司老板呢给我的钱比较少，那会儿才三千多块钱，当时北京工资那会儿其实算是不错，因为北京市那时候平均工资大概只有两千来块钱，就是月工资，我那时候拿到3500~4000其实还不错。到2002年的时候我每个月工资有五千多，只是说因为我个人成长速度比公司成长的更快，老板给我的这个发展也太那个，所以我就到了2003年我离职了。

这个过程中呢发生好玩的事儿，因为那个MBA想着自个儿上课培训就差不多了，但是觉得当年在本科是二本学校，那样的话觉得一个本科学历在企业想长期发展肯定是不够的。那怎么办？我当时就准备想在再拿个硕士学位，这是做业务很好的情况下我2001年又去中科院心理所，我去读的心理学硕士，我读了半年多，我跟所长吵架，我退学了，我觉得这个没得意思，就是2002年退学了嘛，但2002年考虑还得上一个，所以我2003年考虑好之后就考MBA，为什么考这个？第一毕竟在企业做管理，那么你是其他的一些学科你跨专业考有门槛，另外因为MBA不考政治，因为政治你考研是很难的一件事儿。再说，我们在企业工作很多年，再去读的别的东西心里会很抵触，那所以MBA是不考政治的，他就是面试之前会有个考试，但是那个政治考试只是一个辅助，就是完全一个任务，根本不算数，所以当时我就考MBA，但是考MBA其实当时很难，像我是文科生，因为MBA一般是考理工科嘛，要考数学，我大学是没学过数学、微积分、线性代数等，所以我是自学的这个微积分、线性代数、概率论，自学这个考过了，当时我考那个管理学的话，2004年1月份，这几门的话是基本过线，英语考了个60出头，管理学考的很好，80分，全国第二名全国，那五万多人我考了全国第二

吕梦娜：那时候是全国统一招生吗？

受访者：统一考试，但不是统一招生，统一考试很难，最高的、比我高一个人考81分，就是考80分很困难，从那个考试来看，那我应该有一定很好的基础。

考完之后2004年上学，其实我在2003年非典期间我还辞职了，我到另外一个国有投资公司做项目顾问，做了人力资源那一段儿，我们不是现在这几年都提工匠精神嘛，当时在国资委下面一个公司想引进德国双元制教育，职业教育双元，双元制职业教育就是那些学生跟正常普通大学不一样，就叫职业教育，职业教育训练就是两天半时间在学校，三天半时间在工厂，想引进这个德国双元制，德国工程工人技术那么好，很多是双元制教育成果，我们想把这个引进来的。当时那个项目书也是我写的，但后面因为国资委相关官员做事儿，因为不担当，所以当时是老是做调研、老是做论证，但是就是不往前推进，那就没意思，我就是上学9月份上学，我到年底我就辞职了，就不干了，就安心上了几个月学。

2004年寒假放假，放假了没回家，我就写了两篇文章，一篇文章呢发了个《科研管理》，《科研管理》好像在管理学排前五的期刊吧，那是我一个假期写的一个作业；另外一篇文章呢就送一个专业期刊，就是当时中华英才网办的一个杂志，我去了之后投稿给他，他把我作为作者，他问我你在做什么工作，我说我现在在上学，准备找工作吧，他说你没有兴趣来聊聊，所以我去就聊，聊完之后就让我去做记者，做编辑，就是从作者变成了一个投稿人，再变成编辑和副主编。这么我在那儿去的时候，刚开始是在中华英才网做这个杂志编辑，做一年多之后，因为老板呢确实觉得我还是有很多能力，仅仅做这个有点儿浪费了，又让我去负责一个就是评奖的研究项目，可能你没准儿你们都听说过，叫中国大学生最佳雇主调查，这是我当时做的事儿，就是做这个项目，写这个报告书，还有一个工作叫人才研究计划，还有一个工作，这个当时2006年中华英才网做的一个央视的节目叫《赢在中国》，一个很重要的节目，那个专业这块儿是我写的，就写这个研究筛选方案，还有海选方案，我也参加他们那个从108到36这个沙盘模拟做这个专家评委，我参加过，做这个事儿的过程中，同时我们老板把我就调到人力资源部做副总监，做了不到两年时间，因为在中华英才网的工作就是他那个培训体系是我建立的，绩效管理体系是我建立的，做这么一个工作之后，2007年我辞职我就不想干了，我就辞职读博士，读全职读博士了

吕梦娜： 那您MBA是读了多长时间？

受访者：MBA的话，我是在职读了四年，其实我早就上完课了，内容也写完了，只是没答辩，答辩的时候是08年，等于我考上博士了才答辩，答辩的时候08年才入职，读博士是全职读的，全职读了四年，在北京科技大学

黄秋莉： 就直接是自己考的管理学的一个博士？

受访者：对。

黄秋莉： 基于您对自己的一个追求，是吧？

受访者： 主要是在企业干的话已经觉得没劲儿了，跟你们讲，我当时这个职业生涯呀，最早的话我其实有机会去从政的，像我们班做学思政的人，大部分人从政。我们同学现在有做这个副厅长级别的，也有很多一堆处长，但是我觉得我这人的话不爱受约束，也不爱说假话吧，所以来讲的话不适合那个，在上海最早去的时候呢，我并不是说要去打工去的，上海当时工作呢就是我们当时在大学那会儿，93年~97年那会儿最火的企业是你们山东青岛的一个企业，海尔，海尔很火，我们那时候下海想自个儿想以后当企业家的，在上海工作三年之后发现，这个企业家不是想当就能当的，因为你办公司什么人都能恶心你，你还不能跟他太较劲，较劲就弄死你，那个很痛苦，那后面儿我在北京那个群顾问做这个咨询顾问的时候接触了很多的中国第一批外企职业经理，我当时还不敢到北京来，就说做个职业经理完了，后面跟那边职业经理交往发现到了40岁的时候，那职业经理也很痛，因为大部分企业都不要了，到了40多岁，你看到企业之后，你会发现没有40多、50多的人

刘书博：他是这样的一个曲线嘛

受访者： 他就到天花板马上就断崖，那我想这个东西干就没什么劲了嘛，所以我当时琢磨，其实到2002年，我从中科院心理所退学出来的时候我就想好了，我说这个东西我做记者嘛没法弄，因为在中国很多东西就写不了，也发不了。

那我说这个弄来弄去这块儿看来是没什么路可走的，所以我这是无路可走做学问的，就是你把前面都做了一系列减法之后、否定之后，就华山只有一条道了，安心做这个就完了

黄秋莉： 感觉您是因为觉得做企业或者是做记者有很多您自己的抱负没办法施展的

受访者： 控制不了

黄秋莉：对，您就觉得太受约束

受访者： 你努力未必有结果

黄秋莉：所以您觉得这个最开始对学术界就是一种自由的向往

受访者： 一个就是对自由的向往。再另外其实还有也源于在企业做咨询那个阶段，做咨询很多就是给企业做这个解决方案嘛，做解决方案好像的话你做完之后企业也当时鼓掌啦，也打款了，好像满意度还可以，但是你提供的这个解决方案、这个思路是不是真正解决问题了，科学依据在哪儿？其实不是特别有信心的，我呢读书其实从知识来讲，我确实都能够提供一个确定东西给他，因为一般咨询都比较浅嘛，所以就读书、做研究这一块儿，就是越读越远

黄秋莉： 就可以看到您对这个学术界也是像我们一样有一种非常浪漫的一种想法

受访者： 也不是，因为我在北京工作过三四年，2000年到2008年就是像上次聊这个彭剑锋老师，那只是一个，还有北京的人大、北师大、北大、清华很多一批知名教授我们都交往过，就是在大学也有很多不堪，也有很多新闻刊出来，我想明白之后我才进来，我对从要读博士到大学做这份职业——教师这个职业——并没有什么太多浪漫的想法，只能说这个工作相对于其他职业，相对于读书人来说还是一个相对合适的职业，他是半自由身，你要说在企业你的时间···

我曾在中华英才网工作的时候有一个最痛苦经历，上班有点下班无点儿，你人力资源总监嘛，9:00你得到公司，但是下班儿什么时候你可是不知道，像我们当时那个美国公司老板他们开午餐会，我们这12点，开完一两点。我2:00回去睡觉，9:00就赶来上班，就是这么赶。一个季度一回那也没什么意思，所以当时我是2007年辞职的时候，我们老板很生气，因为他觉得我当时32岁的时候做外企的人力资源总监已经很高了，并且他觉得是他给我机会成长了，觉得我是抛弃他，他就很不爽，他说按你现在这么一个仕途的话，你再工作十年肯定能够成为一个知名企业副总裁的，我说副总裁又怎么样呢？还不是做这些招聘、培训、绩效这些事儿，我说我在读博士，我说我再干十年，我就去一个一般211学校，我做个副教授

吕梦娜： 那你看来副教授比副总裁未来空间要大嘛

受访者： 那肯定大多了

吕梦娜： 您当时觉得空间大在哪里？

受访者：因为40、45岁左右是一个职业生涯的天花板，你很多时候到这儿你走不上去你就萎缩了，但是大学里面的副教授你还可以往上走，越老越值钱，空间在这儿呢。另外读书，你永远可以做自己最喜欢的东西，但你在企业工作，那你不能说你做人力资源工作天天创新吧，今天弄一个新表，下个月又弄一个新表，那公司员工不得疯了。

你做管理还得有一个稳定性的呀，你不能老创新呀。但是做学术就可以，就是上个月写一篇文章，下个月就可以写篇新文章。但是你在企业工作，尤其是人力资源工作，你不能说今天创新一把，明天又创新一把，那人家就得折腾死了。我当时有个手下，当时是我挺狠的，因为我做事是一个比较强势的一个总监，他给我协助做这个培训管理工作，那姑娘她一天跟全公司12个分公司嘛、那些人力资源专员发三个邮件，调查问卷上午10:00发了一个版本，下午2:00又发一个版本，晚上7:00又发一个版本，我一看到第三个的时候我直接就疯了，我就打电话我给大家，我说你现在开始停止跟所有人发邮件，他发邮件要发，一定要经过我转发，不转发的话他的邮件都可以不执行，你一天发三个版本让下边人怎么干，这就是你做企业工作，不能那么整天搞创新，你想成熟了才能干，你不能说一个培训调查，一天发三个版本，你让人家怎么办？

所以就是做这个企业管理工作，对我这种比较喜新厌旧的人或者喜新不厌旧的人就很压抑，长久就会很压抑。再一个，因为看过了太多企业里边儿那种肮脏的一面，像老总、副总盯着公司好看的良家妇女或女孩儿，我有一次喝酒的时候，我说你们要想那些事儿也不是个事儿，你们两厢情愿，另外去找小姐，我说你整天盯着这个良家妇女这种骚扰干什么呀？他们打赌谁长得不错，我们就赌谁能把他拿下奖励

吕梦娜： 那跟那个日本大屠杀那种···

受访者： 一个德性

黄秋莉： 就是您人品···

受访者： 不能说人品过关，就是看不惯这些东西，所以就说觉得在这儿干没什么意思。因为我当时也还在业界，我做人力资源那会儿的话还明显有点认可度，同时，那会儿2006年的时候我年薪已经到二十多万，那会儿北京市的平均工资才3000块钱，那也可以的，再加上觉得钱、名、权对我来讲不是那么吸引我的东西···

黄秋莉：精神的追求

受访者： 对，看书这个玩儿这个东西自由，想看书看书，想写东西就写点小东西，这是最觉得有动力的东西，所以我就是40岁做博士做学问的

黄秋莉： 现在我们就开始讲这个到您博士阶段了哈，您博士期间的话因为您是进入到您心心念念的这个学术界了，跟您想的一样吗？达到您的目标了吗？

受访者：做学问呢，用一个最好玩儿的法儿讲呢，成功的学者、做好的学者常常失败，基本上伴随着失败，多数是每天都跟失败打交道。

黄秋莉： 怎么有种做实验的感觉呢？

受访者： 因为一个句子写不成呀。

黄秋莉： 对对，是的，我觉得是

受访者：因为我博士期间做研究，我就想诚心做学问了，但是我这个人呢就是干什么都是批判眼光，做管理学研究，我并不把自己当成一个管理学者，我更多是把自己当成一个社会研究学者，我还说过嘛，我在企业培训工作期间，我就把管理学所有知识、相关经典书都读完了。

那到MBA期间的话，我把社会学相关的东西读了，其实我到了博士期间，学的是企业管理专业博士，但是我做的是用组织理论组织社会学做的研究

黄秋莉： 我其实很好奇你是怎么接触到这个流派的？

受访者： 看书吧，你看吧，用已经过世了一个学者邓正来（邓正来当年很有名的一个知名学者）的话来说，看书就是看这个行业前五名的，你首先挑出哪些是好学者，跟着他的读书节奏跟几年你就知道这个脉络往哪儿去了，我当时读这个书的话就是说像零几年我读一个是当时社会学入学的时候最开始入门儿，我就知道这个老大是费孝通，当时跟着他，把他周边的包括潘光旦呀，林耀华呀，读完之后，后面就读的那个周雪光，周雪光有一本书叫《组织社会学十讲》，那就把周雪光《组织社会学十讲》做一个文献目录，把他提到的就滚雪球

黄秋莉： 可见您是一个非常主动的学生。因为现在好多博士可能更多的是看文献

刘书博： 他们现在博士，从博一开始，那像我们院长就问，你们文章发没？就这种

受访者：像我的导师这边来讲，到博士期间，像我当时北京那个导师虽然说不是一个做学问的一个教授，但是他是读书特别好，当时我们上了一个课叫《组织理论与一般管理》就是经典组织理论，那理论文献的话都是要超过50年的，就是经典的东西，他列了个书单，最核心的是八本，大家一看很有意思，在来之前、在我上博士之前，这里面有六本我是已经读过了，基本上就是提前读完了。读完了之后，因为以前是读书，那这时候来讲你就是自己生产知识，所以我呢就学这个组织社会学，看相关文献，我就照着做这个访谈，开始做质化研究

黄秋莉： 您之前讲到是因为您跟着一个小导师一起做这方面的

受访者：做研究这块儿，其实我刚开始是MBA期间那个寒假就写了一篇文章发《科研管理》，做完之后当时因为我是同时在那个杂志做副主编、做记者嘛，当时就说我也是文科出身，当时那个我硕导张建教授他说长桂你有特点，文字也好，你要不做质化研究，所以我当时的话就是入门的这个质化研究，看书就是陈向明老师的两本书，一本是她的博士论文，《外国人与旅居者》，她哈佛博士论文这是一个范文；另外一个呢是她写的一本书《质化研究方法与社会科学研究》，我进质化研究的入门书就是陈老师，我的硕士论文就是做质化研究

黄秋莉： 其实很奇怪，你像我知道的像那个清华他说他们现在关于质化研究的范式他们都很少接触嘛，就是如果想要学习的话，可能还要去国外去找相关的老师。现在我们学院教的话也还是实证范式偏多嘛。那你们那个时候实证范式或质化研究范式他们有···

受访者： 第一，我们那会儿没有那么细，那会儿就哪怕实证研究方法教的其实也不多，就比较很大陆货那种，其二这个研究方法是完全自学，没人教，就是自学，自学就是边看边琢磨，照猫画虎。所以我硕士论文没发表，但是我的论文其实都还可以，我以访谈的关键时间法做这个东西，做了一个基础

吕梦娜： 那您当时说寒假写的那篇文章也是质化研究嘛

受访者： 我寒假那是个文献综述，所以我当时把我那个文章发完之后，把那个北科大当时一个副教授吓一跳，他说我们这儿这么厉害呀，MBA学生写的文献综述论文竟然能发《科研管理》，当时把他们吓一跳，这是我硕士期间干的一件好玩儿的事儿，其实我就一个月就写完了，我博士期间反而没发出来，因为博士论文你改行了，不做这个；另外因为组织理论本来就比较晦涩，同时呢也不是很热门儿，所以我想做的真正的学术的、有思想的东西就是组织理论这一块儿，也就是用社会语言叫组织社会学，我的博士论文就转这个，我做完博士论文很快，我大概就是博二的时候我博士论文初稿写完了，那会儿发表的不是很顺利，但是我大文初稿已经写完了，写完之后我去了香港中文大学做访问，我用语言学做管理研究是从香港中文大学访问开始，是来探索的，因为这个以前国内用语言学技术做管理研究的，在我们之前有一点儿，但做得一般、没做好，就是算我跟刘老师算开创的，就是这么一个过程

黄秋莉： 所以你读博士阶段第一个就是觉得有很多出错的地方嘛，第二个觉得您这个博士学习的阶段还有哪些不好的感觉或者是好的感觉

受访者：博士期间我一个是把组织理论的东西读的比较深入，另外我干过一件事儿应该是你们现在不会干的，我有一年我翻译这个组织社会学那个经典著作理论的文献，40万字，一字不落翻译，翻译40万字

吕梦娜： 为什么？

黄秋莉：那时候您周围的同学？

受访者： 他们不做，他们不做

吕梦娜： 对呀，那你为什么要做这个工作呢？

受访者： 为什么做这个工作？因为没人训练你，我自己训练自己

吕梦娜： 你是为了训练自己的中英翻译还是···

受访者： 训练自己对组织理论的感觉

黄秋莉：您周围的同学他们是怎么样一个状态，

受访者： 他们就是巴巴看哪个好发，然后赶紧弄一个毕业

黄秋莉： 你们那时候毕业也要发论文吗？

受访者： 很多年就是这个，因为我就是太关注学这个真东西，所以我当时发表不是···

黄秋莉： 就感觉你是一个不太一样的感觉

吕梦娜：很特立独行的一个人

受访者：我估计像你们没人干这个苦事儿，对呀，翻译四十万字我真的对组织理论掌握得相当合适了。像我现在训练我的研究生我怎么训练的？找A1/A2合适的好文章把它给我翻译，每个副词我都要求翻译准确，因为你翻译准确的话你就知道学术语言怎么表达的，怎么逻辑链条，因为这个过程呢，我们入行很困难，只有通过强制性翻译逼着他进入这个，因为要翻译就得把这句话弄明白，他得权利以赴，你必须用中文话说出来，包括我不允许他ppt上出现英文，你就是要中文讲出来，你必须翻译讲明白，你自己讲明白，能翻译明白了，你就真懂了

黄秋莉： 所以这是一个非常漫长的过程。

吕梦娜： 对，这是一个很扎实的过程，

受访者：对，做的很扎实、很训练，但是这个活很苦，但是做完之后受益无穷，像我后面做的很多研究包括我博士毕业论文以及之后发《管理世界》那几篇文章，都是这个基本功、童子功，训练下来之后是终身受益的

刘书博： 那个吕老师也说了嘛，就现在有一些博士他只读paper，他不读这个原著、不读书，所以他就是一种非常反式脂肪，它不是天然，它是一种人工的，但味道很甜很腻

受访者：速成品

刘书博：但是呢它其实缺乏一种···它不是自然的，它就是一个工业化的东西。

受访者： 用我北京导师的话讲就是博士研究这个东西，知识学术要长成大树不要长成旗杆，旗杆下面是没有根的，就是一个干巴巴的就这么上去了，但是大树是有很多根系，很磅礴

黄秋莉： 我怎么觉得我们现在的博士就是没有这种基础性的训练，我自己就没有这种感觉

刘书博：，等一下我们讲自己中财都很熟悉嘛，其实它就是一个流水线工厂，就对标，就是把你们弄成旗杆，所以你们很痛苦，因为你们是人，你们是有生命的，但是结果出来他是按照旗杆来···

受访者： 因为你们看一下我的这个学习、博士研究经历就是完全就是真正、纯正的学历。

吕梦娜： 对，我向往的就是这样

受访者： 我上北京科技大学的时候就没几个人做研究，因为我是追求太高，但是北科大人都跟不上，所以我北京那个导师把我送到吕源教授那儿，吕老师是我北京那个导师的老朋友，因为我老拿我的论文去找他谈，谈的我导师很痛苦，因为他有东西还不懂，他说好长桂，这样吧，你既然这么好学、愿意做这个，我把你送到一个可以谈学问的地方去，我是2008年认识之后2010年去访问，那到现在这11年多，我们俩可以说是无话不谈，就是虽然说吕老师他有另外一个学生是李伟文在中山大学那个管理学教授也发过发过两篇文章，但我想吕老师对我的认可度、信任度比他自个儿学生的要更多，因为我这东西真的是有思想、有自个儿动力的一个做法

刘书博：所以就彭老师是野生的

受访者：生命力激情非常旺盛，我是平均两三年会转一个小方向，但是我不是说别人那样这一步直接踩空了，不是的，我是半步，每次落半步这样你永远就是实的，同时永远有激情和创新

刘书博： 对，像刚才说的吕老师，他是港中文的，那他带的一个博士生，就刚才说的李伟文，他就是那种单发那个smj这种达拉斯，第一作者发两篇，他就很能发很能发，但是其实香港培养的这种都是这个特征，他非常的聚焦

受访者： 模式化、标准化产品

刘书博： 标准化，所以其实咱们学院那个于广涛老师他就说，诶香港的其实他们水平高，其实背后就是他们发文章很好，很快，很好用。但是我又觉得像于老师他是缺乏一种反思的视角，因为对于组织来说可能达标是非常好的

受访者：像香港中文大学那些教授，我当时去访问的时候我是博士生，那所有教授都对我很客气很尊重，他说不是按他们来操作，就是因为那标准化产品他们很熟，像我是野生那种、很有力量那种、谈事儿很有创造性，因为我知识面经常就说···

我和吕老师两个在香港干个好玩儿的事情，我在那儿半年，他说你在这儿半年是我很开心的半年。我们俩每天呢，他一早去工作，去上课；我的话，每天大概十点到他办公室，我在那儿我们看看新闻，中午一块儿吃饭，我跟学生吃饭，他下午教学，一般下午他最晚4:30回来，4:30回来。因为中国香港大学在山上嘛，海边儿嘛，我们俩就去溜达一个半小时，我们就慢谈，从管理学，管理学界，企业界，中国政治、哲学、语言学、社会学、政治学、历史学、文学，啥都谈，他说他发现“小彭，我谈任何问题你都接得住，我说的任何书你都读过，只是说你可能读的中文版我读的是英文版，但是没有这个理解差异”，就是说他比我大20岁，吕老师55年的，我75年的，他说按你这个年龄段有这种知识结构让我是很诧异，所以我在香港中文大学访问的半年那边教授对我的评价很高，他说这个虽说是北京科技学科，但一般北京的孩子未来肯定很厉害

黄秋莉： 就可以看见在香港有这种学术氛围，那您在国内会不会有觉得自己和这个环境有一种格格不入的感觉？没有，你你你你是超越

受访者： 没有，你寻找自己的通道就好了，你不用说跟他们去玩，他们那块儿你可以说按照专业训练，他们就说同行的可以去参与，但是我有个群是全球一百五十多个学者都是跨学科的，历史学、语言学、人类学、经济学、公共管理的，全都有，我属于他们所有人谈任何问题我都可以对话的

黄秋莉： 那您能不能给我们介绍一下您身边人的一些状态

受访者： 我身边人就是说可以这么讲吧，管理学其实有关注，我们可以先谈管理学，管理学基本上大部分老一批的（学者），除了我导师吕源教授比较尊敬之外，老一批管理学者基本上算不上有什么真正的学术创造性。这是第一批，第二批来讲的话，像类似于像朱昌辉、魏江、毛新宇这一批，他们其实刚刚在国外有点儿东西，但是他们最大问题是跟着跑，跑着跑着自己现在也没有，也很痛苦，因为出不来，因为学术话语权在人家那边。那年轻一批，像刘老师一个年龄，较好的这一批，也是跟着国外跑，但是呢现在也投靠一些体制意识形态跟着走。

刘书博：杜运周这些

受访者：杜运周还有杨俊，他们这一批就是八零后这一批，李伟文也算，李伟文82年的，就这一批的80后学者，他们大部分来讲的话可以说是，他们就是属于徐淑英在中国的产品，就这么一个性

刘书博： 几乎不是学向他学习，而是被他给管理住，没有自主，不是我可以向你学还可以不学，他们是完全的追随者

受访者： 他们是追随者，用一个政治学语言讲的话，他们是西方管理学者的群众，群众都是跟随的，你知道那个群众的概念，群众那样就知道了，因为像那个社会学者鲍曼他说过一个话语，就知识分子的定位有两类，一类是立法者，一类是阐释者，他们都还没到阐释者，他们阐释完全是鹦鹉学舌，那么就是最差的阐释，只是给西方那些理论、已有关系做个脚注、做个应用。

但是我并不是反对西方，我的一个话就是“对话但不对抗”，都可以对话，但不一定是敌人，那么西方知识，用毛主席的话说就是“洋为中用”，你怎么用能够创造新知识的呀，我们用语言学的话将，就是重复讲，你把那些话讲了一遍之后反过来再讲，再讲一遍，重复讲；另外一种呢所谓就是想自个儿弄一套，对着讲，就是说对抗，就比如像他认识那个赵向阳老师和吕丽他们搞那个中国本土学派，这个他们是对着讲，就是想反着讲；还有一套呢是叫接着讲，就是把你的观念和我的观点融到一块儿，往下发展，那这是一种接着想。那最好的、最高的第四种呢是讲出你自己的东西来。

那么中国的管理学研究讲最多就是他们就是杜运周、李伟文那一批的，好听来讲叫接着讲，不好听的就是鹦鹉学舌，就这么一个状态。我跟吕老师我们想自己讲，像包括话语分析用语言学技术做管理研究，我跟吕老师俩算是国内最早开创者，包括今天发的文章，虽然只发一个ipjm那个文章，但是真正用语言学技术做管理研究，我们是全球第一篇，那其实我们想有点儿新东西

黄秋莉： 创立者？

受访者： 就是说有点儿新的东西吧，是不一样的东西出来，所以来讲的话就说我们文章那个“叽唧堂”公众号，我们那文章其实是一个B刊，“叽唧堂”公众号一般都关注A刊的发表嘛，能够把我们谷歌和苹果那篇文章推广一下其实不容易。

另外我之前做内蒙草原治理的课题，因为现在民族拆迁的地方政策问题，政治上对边疆这些方面比较敏感，草原治理项目相关文章投出去之后一篇也没发出来，但是我项目评的是优。

吕梦娜： 您之前读书经历大多是出于管理实践需要，后来自己写的论文都是希望能够解决实践问题。那您有没有接触过好像跟实践比较脱离的那些研究？	受访者： 我曾经做过，但是我认为我不是那个路数，所以我放弃了。我感觉我现在挺如鱼得水的呀，就是做自己喜欢研究，看自己喜欢的书。

黄秋莉：您刚刚提到原来也经历过挫折，您能不能展开讲讲？

受访者： 之前有人会鄙视做质化研究，包括各种会议上，多了去了。长期以来一直有这种经历，那会儿更厉害。

刘书博： 这是一个学术范式的认可度的问题，我非常能够体会刚刚说的这种中国学术对研究范式的压迫。但是你们得明白，不要别人说什么你们就信什么，尤其是学院的一些年轻的领导或者所谓的学术精英，看起来他们发的很好，但是当我们需要拉长时间轴来看这件事。

我在你们这个岁数读博士的时候，后来找工作以及投稿的时候，以至到现在我回过去看，我觉得我还是应该坚持自己。如果我完全被他们说服了或者恐吓住了，那这个事情可能就做错了。

所以有一些老师说博士做案很难，你们做不好，我认为这是不负责任的说法。

受访者： 用一句话解释：我是一个不能驯化的品种。我不接受学术内“圈”的驯化，我们不应该被那些所左右。

刘书博： 这种驯化你们在很多时候都可以感受到，在上课的时候、面对评审的时候、收到答辩意见的时候、甚至跟同事闲聊的时候、吃饭的时候，他们不经意间就要驯化你，就得让你符合所谓要求和规矩。

吕梦娜： 您有没有受到同辈的压力，比如说身边的人都是走那种（实证/灌水/追热点）的路数？

受访者： 当然有。我就说两件事儿：第一件我博士毕业，当初那会儿就算比较有点儿思想了，我在读MBA期间就已经发了一篇A，博士毕业也就要求一篇。但是因为我没有特别跟体制走，我文章已经发了，但是因为文章检索要晚一个礼拜才能检索到，那导致我2012年6月份毕业，但是我拿不到博士学位，直到2013年1月份才拿到。那这对我来说是一个很大的打击，整个学院里面学问最好的人毕业拿不到学位，那对他来说绝对是一个羞辱。

另外一件事是我刚工作的时候，我毕业的时候已经37岁，年纪本来已经很大了，当时很多学位比我明显差很多的人，但人家就是三年就评上副教授了。那我由于一些原因评副教授一直都不太顺利。

黄秋莉： 现在晋升都是指标导向嘛，您对这种评价体系怎么看？

受访者： 指标评价体系会存在很多问题，比如会先看数量再看质量，我发了管理世界，质量很好，但是数量达不到。

刘书博：会计学院有一位老师在评副高的时候有15篇成果。

受访者： 因为人事处首先一查，就是你数量不够，这样你连从人事处往上报的机会都没有。

黄秋莉： 那关注的就不是学术本身。

受访者：我再讲另外一件事：我们系里边儿年底的时候都一块聚餐嘛，当时有一个副教授发了一篇中国工业经济，也很不错，他们那时候号称他是学院里的“学术男神”。然后人家一说这位老师就很不好意思，他就说人家彭老师发管理世界了。这就是说，我去之前人家活得好好的，是男神，但是我这几篇一发表出来，人家就觉得毫无尊严、成就感可言了。

另外，我发管理世界一共发了三篇文章，第一篇是跟吕老师一起写的，我是第二作者，当时他们的反应是，彭老师发了一篇管理世界，彭老师很厉害。第二第二篇发出来之后，大家觉得我运气不错。第三篇发出来后，首先在管理世界的微信公众号上发出来了嘛，另外一位同事就把微信推文转发到微信群里面了，但两个小时过去了都没任何人反应，也不是我跟人家结梁子，而是人家就不知道说什么了。

黄秋莉： 就被震撼到了吗？

受访者： 他们肯定觉得震撼，但当时也有种不屑，或者认为我发的文章可能不正当的那种酸葡萄心理。人家从合法性的否定你这是保护自己尊严最好的一个方法。

黄秋莉： 刚才你提到个体在组织里会遇到一些不顺利，那这个可能来自于组织政治、组织惯例，另外也可能来自于身边的个体。

其实我还很奇怪或者震惊，因为我觉得这是你们学院的荣誉。

受访者： 我跟你试图解释一下，你到一个新的学校，如果你跟原有老师的研究范式什么都是一致的，同辈挤压会非常厉害的。同时如果你跟他们研究的范式完全不一样，大家也不一定更愿意接纳你。

同时如果你做的非常凸出，以至于打破了原有的学术生态，论资排辈情况下那种高资历的人就会在内心上非常抵触你。

刘书博：所以这个事儿反映出来，学术共同体在他们那个组织环境下并不明显。

受访者：根本就没有学术共同体。

刘书博：这只是一个案例，但我们可以看到很多类似的事情。学术共同体可能低于政治、低于人的自我排位。说白了就是我看到一个你做出来很有趣的东西，我肯定特别的想要了解或者是发自内心的想要跟你交流。但是这是一个政治性的对抗，而不是说共同交流。

受访者：很多时候学的理论是人要适应环境，但因为我作为原有制度下的一个异类，现在做了很多别人以前做不了的事儿。我那时候不仅仅说我自己发了三篇管理世界，我还带领别人发了很多顶级期刊文章。

 就是说学院层面的共同体是很稀薄的，甚至于在学院里还不存在关于学术的争论，首先是说都不做学问。

我离开以前工作岗位的时候总结了两句话， 第一行政欺负老师，第二不做学问的欺负做学问的，那还不涉及到第三部分，即学术内部之间关于什么是真知识真思想的争论，还不存在学术观点的对抗。因为可能很多人没观点还只有自己的利益。

黄秋莉：请您展开讲讲关于行政欺负老师的事情。

受访者：当初我在内大拿面上国家自然科学基金项目，项目评价评很高，得了三个A一个B，那时候可能是历史上第一个这么高，同时当年又发管理世界，所以学院里副院长就说觉得不错，今年肯定要推荐你做优秀员工，那学院领导是这么说的，但说完之后到年底又没这事儿了，优秀员工给了另外一个副系主任，所谓的当地实力派。就说哪怕我学问做的再好，最后拿优秀员工的还是搞行政的。	黄秋莉：学院并不是以知识和学术对判断。

受访者： 嘴上说是学术为先，但实际上就不是那么回事儿了。

黄秋莉： 他之所以被评为优秀员工，是因为他做了很多的行政工作吗？

受访者： 这个就是政治性因素、地方利益的关系。关系搞不清楚就很多了，因为他们那个圈儿我也不进去，也不知道。我自己学术做得好，发了很好的期刊，而且还带别人发了好期刊，并且基金项目做的也非常好，可以说学术性贡献非常大照样欺负你。

受访者： 这个这是一方面，另外给钱很不公平。像我发三篇管理世界，实际奖励总共不到10万块钱了。像当地一个老师会搞各种课题，他们那头制度的人，年底考核拿一百多万。

吕梦娜：这种情况下下您是怎么回应的？你是做学问还是怎么来钱怎么干？有种劣币驱逐良币的感觉。

受访者：这得看你守不守得住自己。可以说在那种恶劣生态下我能杀出来的话，其实真的很不容易。这种生态特别打击人的积极性，很多人直接就废了。只有像我这种是环境驯化不了的。但是在那儿发展空间受限，所以我最终还是走了。

刘书博： 现在在北大清华，行政可能会屈尊于专业老师。但是在中财明显就是行政主导。行政老师会觉得自己是管理者，你要听我的管理，而不是说像国外或者像港中（深）高校内是服务于老师的这种心态。

受访者： 中国管理学，第一是有没有学术观念的问题，其次才是所谓内部观念争论的问题。

刘书博： 中国大学虽然名字和西方一样也叫大学，包括学术研究，但其实内涵跟西方是不一样的。我们没有独立性，我们是依附于从上到下的权力，更像是一个政府事业单位组织。

很明显就是书记一直不断的在调配各个学院的资源和精力，比如说现在要搞扶贫，然后纪律卫生检查之类的事情，学院领导就非常关注这个事情，他们一个命令下来我们就颤颤悠悠的那种。

受访者： 中国社会嵌入性很强，大部分中国大学主导力量，一个是政治力量，一个是行政力量。这两个主导力量下，基本上就是围着转才有机会。建议读读韦伯的著名演讲“以学术为志业”。你知道这么黑暗之后你还依然热爱那说明是真爱。

刘书博： 尼采的“超人”概念。

黄秋莉： 在行政力量和政治力量主导下，学校如何提倡学术？

受访者： 你不能说他们不提学术，学校是个混合性的状态，有时候先满足各种利益情况下再看，但不是说完全不看。最低程度他也要用学术来装饰自己的。

刘书博： 就像朝鲜的空姐最漂亮，就是在window dressing这一块儿做的工作是挺足的。但是真实内容有待考证，双一流是否真的是世界一流，还有什么学科一流是否真的达到实质性效果？我们需要去把这些内容给透析出来。

包括学术共同体，虽然我们也经常说，但似乎跟老外理解的不一样，姜峰在跟那边面试，然后老外听到他护照还被学校收走了，老外就觉得非常的诧异。

受访者： 在国外他们想象这种事儿，包括体制内的一些东西，以前我们大学生结婚还要导师签字。

刘书博： 之前哪个新闻还提到有学校规定不让有婚前性行为。所以我们要做的研究也是一种文化研究，老外他们不知道这些。

受访者： 在行政、政治或商业三大力量合力围剿下，管理学就像一个大浪中的小帆船，我们要看它是怎么从此岸到彼岸的。如果从这个视角看就比较有趣，一定是研究多方风浪之下如何发展的问题。

刘书博：我们经过前面的几轮访谈，有一些theme已经浮现出来了，包括从上到下的、学习外部的、我们没有自主、没有从下到上的学科发展，完全是听别人，完全是依附的。

受访者： 我在内蒙做草原的研究，总结出内蒙一个特点，这也是中国特点：最大的优点是好学，缺点就是太好学，好学到迷失自己。不考虑自己是不是能用，反正把什么搬过来。

刘书博： 有一个社会心理学理论叫后皈依者狂热，像一些中国华人移民到美国，他们的二代比当地美国人还要歧视华人。因为他们只有极度摆脱自己原有的身份成为新教徒才能获得这种身份感应。像博雅光华里体现出来的，对美国就是那种朝圣之旅，字里行间感觉去西天取经一样。

有一个人统计过研究范式，管理学研究中美国80%实证，中国90%，欧洲可能是颠倒的，所以你看其实在数据上也证明了，我们虽然是后来者，但比美国更加的实证。

受访者： 这也是我离开管理学的原因之一。因为你想要在国际上冒头，你必须走国家道路，那走国家道路你就免不了要跟徐淑英这帮人一块儿混。但是我本身看不上他们，说到底就那点东西。

吕梦娜： 所以我感觉您属于那种我命由我不由天，是不会被异化的那种。您从管理学出走到经济学，经济学会好一点儿吗？

受访者： 我是做经济思想史，它有经济学相关的东西，但是更多是想做思想史。我讲的是中国改革开放这个这一段的思想史，这个国家政策的经济认知，这个别人讲不了，这只有中国人能把这个故事讲好，别人讲不好，现在讲了很多外部的因素，我直接从认知的角度讲——国家集体认知转型。

刘书博： 我们做研究一定要考虑一下做哪些能比老外做的好一些的研究方向，就比如说我们研究自己熟悉的商学院，那老外他进不来的，他理解不了很多文化现象，比如说到了一定的行政职位，那护照就被收走，包括教务处随便要求某副院长，虽然是中秋节，但要求明天把这东西就要做好给他，完全不是那种服务的心态，而是一种从上到下的命令。老外他们就会觉得你又没给我钱。

而且他们也不会觉得在节假日工作就显得你很努力，我们就觉得这是一件高高在上的、具有道德意义的这种感觉。

那这个就是我们需要表达出来的，在paper里面我们对于知识的贡献就是类似于这些的东西，当然这是比较偏向于现实的，我们还是要把背后的一套理由，即为什么会这样给构建起来，所以这个研究我们有条件做好。

吕梦娜： 所以彭老师算不算我们研究的一种例外？他是没有被异化的个体。刘书博：对，我也是。

受访者： 我觉得管理学届还是有一些未被驯化的吧，只不过很少。

刘书博： 尤其是要警惕那些咱们现在眼中的那种学术明星，就是年纪轻轻的已经发了很多文章那种，像林嵩等，他们的意识形态是包裹的比较紧。我们在听他讲话的时候，其实他也是在散发一种不自觉的规训，他要影响你，让你变成他那个标准。

但是我们需要知道还有其他的存在方式。尤其是管理学者他那么年轻，那么早就成教授，那意味着他其实没有太多的时间了解真实的世界，他的视角是比较窄的，非常专业主义的。所以就他未来怎么办，他似乎也很迷茫。

受访者： 别人看到我也是觉得我很谦虚，同时也会觉得我很骄傲。我谦虚在于对知识的谦虚，像我以前做语言学这一块儿，现在我转到经济学，因为我没学过嘛，所以我去学的话所有人都是我老师，我很谦虚地学习。

但是对于管理学里的有些人我就看不上，有些人发了很多文章又如何？可以讲他的管理学贡献没有我的大。我的三篇发在管理世界上的文章，现在商务印书馆准备出书，商务书馆出管理学书很少，一般看不上管理学的这些东西。

刘书博： 越跟学术明星明星接触越多对刚刚这番话越有同感，因为跟他们聊人生，你会觉得他好幼稚呀，他走向社会这么多年，却依然像一个老好学生。

他们的精神状态跟我高中时可能差不多。就比较封闭，像澳洲生态一样就没进化，他只是在某一点上不断的做大量的工作，但是作为一个完整的人，你不觉得他是成熟的人，他就没成长。为什么说管理学生很痛苦呀，我们大部分学生，你跟他们聊，感受不到思想，感受不到智慧，只能感受到焦虑。

受访者： 那你可能看我的研究，就会发现我对整个世界有一种关怀，同时我有自己的判断，我是有智慧的。我知道这个社会走到哪儿了，我们能做什么。别人说我去经济学是不是要成为一个经济学家，我说不是，我既不想成为一个经济学家，我也不是一个语言学家，我也不是社会学家，也不是管理学家，我是想成为学者！Thinker！思想家！这是我想干的事儿，至于那些所谓的专业，对我来讲没有意义。

受访者：我前几天在内蒙见一位老作家，我说再过五年我会编一本书找你们一起来写稿，这本书名字叫《当国家年轻的时候》，副标题叫80年代私人回忆录，这个事还是有点儿感觉的。

刘书博： 有些人虽然发了很多文章，但精神生活可能比较贫瘠，比如生活中最大的爱好就是收集小虎队、四大天王等唱片。这样来看，我觉得管理学教育其实挺失败的，因为他也是“三清”嘛，从本科到博士一直是那个环境下，他们没有接触真正的社会。这种教授说的东西要国外别的教授一看，就觉得是个笑话。

但现实中我们培养了大量这样的好学生。

受访者： 管理学博士学位是哲学博士，得有点儿哲学素养，有点智慧的。不能一直是这种非常狭窄的精神空间。

受访者：个人觉得你们这一批年轻博士学生，没有好好的去引导培训，要像我给你们当老师，你们会玩儿的很嗨，做东西也不会痛苦焦虑。

吕梦娜： 我前两年最怕碰到我们院长，他每次给我们上课一大半的时间全都是灌输焦虑，说你这个时间要怎么卡，包括前两天我们新生博士，那个副院长，说话，当时我就一直在冷笑，因为我们是线上的嘛，然后一开始音频我就一直冷笑。就什么时候都在灌输焦虑，感觉没有别的新词儿，全都是灌注焦虑，你这个时间必须要倒着推，你要不倒你就会怎么样，就是这种恐吓的方法。

我觉得幼儿园小学生入学，老师还会给你一种很好积极的引导，为什么博士全都是这种负面的打压，没有看到希望的感觉。

刘书博： 这就是教育的教化，而不是发育，只是前面的那种规训。可以看一个美国拍的电影叫小王子。

受访者： 对大多数博士生，我都持一种同情的态度。就是说其实大部分学生，只要知道什么是好的都能做出东西。像你们没有那么浪费的话，合适的时机文章不都出来了嘛。很多人跟我聊完都是觉得生活充满了希望。

刘书博： 跟师兄聊，就发现他的专业意义构建不一样，你要再找像窦超，林嵩这种，他们是完全不一样的。他们是那种标准化流水线的方式，他只有那种冷冰冰的东西。那师兄是一种热情洋溢和充满无限希望与可能性的这种意义构建。这是我们目前见到的professional的两种类型。

吕梦娜： 所以也是打铁还需自身硬，自己真的有实力之后才不会被这个东西异化，不像我们现在一片空白的时候。

受访者：你们可怜也正是在于你们还是一片空白。因为像我的话在企业工作过十年，我自己能有自己的主意，就说你现在打击我没有用。

黄秋莉： 就跟您之前招的那个学生一样，他本身是做语言学的，但是可能大家都告诉他说你要做实证，我们学院也会说做案例的话就出的慢嘛。

受访者： 你就要想办法必须干出来一个。我当时上博士的时候，我们的院长是管理科学与工程的张群，当时他们就是科学主义式思维，他跟我谈，问我这么大岁数读博士是怎么考虑的？我说想做点学问，另外我说发个管理世界吧。在他们看来，他们觉得是个笑话，科大的学生怎么可能呢？

刘书博： 所以你们一定要做自己，就不要被那些老师说的话吓住，尤其现在讨厌的是他经常在任何场合，包括开题答辩等说学生不行，不要做这个质性，你们做不来，这个很难的，你们还是做我熟悉的那一套。英国一个大学教授听了之后很生气，他说这样的老师其实是没有职业道德的，就immoral，他当时就这么说的。

受访者： 我给《哲学之道》那本书写了一个书评，那个车明珠老师讲的很好，跟你说大学里面最好的一个隐喻是“击鼓与撞钟”，学生你不撞老师他不会多想，那老师要刺激你们的兴趣出来，学生的知识和能力不是老师能培养的，是通过激发出来然后你们自己去成长去学习的。

书评里面第一个提到“读”，你要通过读书学会读书，每一个人读出来的东西不一样，你要自己去读，不懂再读，反复读。第二个就是击鼓与撞钟，老师最好给学生一个良性反馈，若老师不断打击羞辱学生，那学生就会受到很大打击。第三个就说爱才，要人尽其才，并不是说我是做学问的，会写paper的就所有人才就只能是写paper的人，那人家有的是有商业才能的，有的教书很好，你有哪方面能力你发展哪方面就好了，这才是世道。

黄秋莉： 回到行政化，您刚刚讲到行政和政治是指挥棒，使得大家都需要围绕这些力量行事；另外一个就是涉及利益的分配。除此之外您还有感受到行政力量压迫的其他形式？

受访者： 最核心的还是考核，用技术管理来讲，考什么我就背什么，激励什么我就做什么，反复讲创新才会创新。现在行政最大的问题就是讲的东西跟学术没关系。因为中国的大学在政治化之后就变成一个驯化的场所。北大的林教授讲的一句话，大学不是养鸡场，实际上大学就是把你们当鸡养鸡，养殖肉鸡后赶紧去宰杀，关于大学所有的描述中形容最真切的就是这个，这是就是最大的一个行政化的问题。

刘书博： 我觉得中国的大学比西方大学更像养鸡场，他们还好一些。

黄秋莉： 是不是会说跟咱们商学院要急着赶超也有关系？

受访者： 行政化、政治化是大环境，商学院尤其突出是为什么呢？道理很简单，因为人家学科都是有传承有基础的，比如说像哲学，你跟我谈这个新的东西，我可以依据海德格尔这一套东西反驳你。

讲社会学，从韦伯、马克思到图尔干，他们有一套传统过来，管理学是没有传统的，管理学诞生之初就不是有思想性的学科，而是应用学科。管理学是个没根儿的学科。一篇文章叫大学科布局，里面讲管理学属于职业教育，它不属于基础学科。英语niche，就是说有个小的环境，就是人家社会学，历史学，文学一直有一个自个儿空间，有个小生命在，商学院容易受到商业诱导，他自己没有一个专业主义，风太大了，它又没有主心骨，没有锚，这是商学院最大的问题，

黄秋莉： 全世界都是这个样子吗？

受访者： 应该说都是这样的，中国尤其如此。

刘书博： 像哈佛商学院，他们其实还是研究生阶段有商学院教育，然后博士其实还是有很强的学术大学环境，而且在本科阶段比较强调的还是个性的培养，虽然也有一定的工业化、人造化。但中国似乎又加上传统的一些因素，然后再加上管理学本身又是一个很人造的学科，他没有学术的根儿，然后又加上我们特别的比学赶超，特别想快速的发展，改革开放后国际化，然后充分的学习外国，现在又有点像学术大跃进，各种因素弄到一块儿，导致你们感受到这个异化，或者就把你们看作是养鸡场的鸡。

受访者： 最焦虑的一个是博士生，一个是青椒。青椒焦虑的是内卷，非升即走等。

我为什么以前离开企业进入大学来，我受康德的一个影响就是人不是工具。我们大学充斥着工具主义、公司化帝国主义。现在全国更不要说企业了，就是所有大学、行政部门、政府部门，都公司化，然后又加上意识形态，所以你们现在应该比我们大人更焦虑。

黄秋莉： 我现在就感觉我是学院的一个工具，文章达到要求我就能毕业，达不到就是一个垃圾。

受访者： 这就是为什么你们院长老问文章发几篇了，到哪一阶段了？就是因为你们每一个都算工分嘛，算下来对他就是KPI，就换成绩效了，就换成钱了。	吕梦娜：把人当工具，而不是说发展人。

彭老师您带过多少学生？

受访者： 我带的大部分都是本科生，我的内大就带一个研究生毕业，因为我不符合他们条件嘛，所以很多年的话都不让我指导学生。我后面去山东机会会多一些。

吕梦娜： 您带研究生有什么感觉吗？有没有感觉出焦虑等等。

受访者： 当然焦虑呀，但是我的学生的话比较好，因为对我来讲的话，第一，那学生念上研究生就发过一篇c刊，我做指导，他做完之后是跟其他老师去发，我觉得文章太差了，挂我名字是对我的侮辱。

黄秋莉： 非常爱护自己的羽毛。

受访者： 对，是这样，因为我挣那个钱，但因为你发完那垃圾文章，文章是会挂你名上的。所以一般文章挂其他老师就可以，人家还能够拿个职称。

我到山大去也是一样的，做文章要做好的，我可以指导你做，但是你文章差了，你就别挂我名字。

刘书博： 之前我们和受访者老师也聊了很多，他说的比较谨慎，中规中矩，四平八稳的，他其实有很多话他知道，但他没说。所以我们需要通过二手资料、三角调查，再把他说的背景弄出来。

受访者：其实我觉得很绝大多数老师还是有一些学术理想或者报复的。但是在这个制度下他的的确确面临着一个要被考核、被评价的竞争的压力。如果说我们想做一些比较长期的一些研究，特别是做比较偏质性的、人文的一些研究，可能更需要时间比较久。而且现在你看光看论文，他其实著作就是看你发的什么级别的期刊的论文。这个评价体系说是代表作啊，就我们学校目前的这个评价来说的话，有代表作评价，但实际上还是有很多这种数数的呀。

我之前看应星有一篇文章，应星就讲中国的“学父”，就是说这些老师把学术作为自己利益的一个工具。

我不知道在管理学领域怎么样，但是我觉得在教育学里面那种山头鼎立的局面还是很严重

刘书博老师：我们也是。

受访者：我看到有一些年轻老师要想申课题，就必须混到某个圈子里去，或者依附于某一个老师。那这样的话学术自主性就很明显的（削弱）。很多老师其实就是做只做十年学术，然后很多资历深的老师年轻的时候有一些代表性的东西，慢慢好像就成了一个权威，但是其学术产出有的时候反而成了这个学科发展的一些阻碍，后面的人就很难有发展空间。

刘书博老师： 对，是的，我们管理学也是这样子，虽然发展的历史不是很长，但是就是很多山头林立、学阀横行。我前几天还跟一个比较岁数比较大的、60后的一一个老师聊，然后他可能见的比较多，他个人总结了一下。他说相比于他见到的美国的那些学者，中国的学者呈现出的一个特色就是好像身体精神状况不是很够。大概就是到了一定年纪就干不动学术了，他就躺平了。然后我跟他说不是身体问题，可能是前面他是一种为稻粱谋的态度，然后那他已经拿到了这些资源，那干嘛还要继续的努力呢？那美国可能还是会兴趣为导向的以学术为业，所以他会持续很长时间嘛。所以我们经常看到一些到了50岁、甚至不到50岁的老师后面不再有真正的价值产出了。

我觉得你书上写的传统文化对于读书人的影响其实也挺重要。那您觉得除了这些文化，有没有比如说大学治理方式、管理模式会导致目前的一些问题？

受访者：我感觉我们从小到大社会化的过程中，沾染了很多不求真的东西吧。就是我觉得跟我们这个文化、政治等制度性的东西可能有关系。我们做学术，“吾爱吾师，吾更爱真理”，但我们那种学术热情，我觉得还是会很容易被浇灭。就比如说对大学来说的话，有些制度我觉得是非常不利于去培养学术人才的。他培养的只是服从于规则的人，就是在这个规则下最大程度的去取得利益的人，比如说这个保研，就特别的积分制嘛。最后大家刚上大一的时候好多同学就特别关心这个。

刘书博老师：我看我们中财各种评比，就要参加好多学生活动呀什么的，然后就这个分数体系就建立起来了。就现在有人会认为这是一种制度的进步，对于人的评价更加的细致。

那可能我们刚才讨论的似乎并不是一种进步，我们总是在强调制度建设，但制度建设的理念有没有副作用，好像很少有人去关注。尤其现在每年都要有培养方案的修正，教育部要求每年都要修正，那为什么每年都要修正？然后修正之后效果怎么样？也没有人过问，只不过是要求你每年修正，最后就变成了一种形式主义的工作。

受访者：我觉得我们在制度设计的时候，很多时候考虑的一个因素是积分嘛，按照这个工分制好像是很公平公正的。但是其实公平公正有很多种办法，而且这种公平公正呢很容易导向极端的精致利己主义、而且是精致的计算。很多同学其实他内心也是有点厌恶的。

比如说我们这边有些规定简直就是特别的不合理啊，比如说你只能加入学校的八大社团，这八大社团是加分的，但是其他小社团或者是一些兴趣类的社团就不行，那就把学生的兴趣都要限制啊。那一个学生以后做学术，他从里面脱颖而出保研了，然后后面做学术了。那整个人的这个天性啊、创造力都被压抑了。这个东西就让我们每个人都会束缚住的。

刘书博老师： 甚至我会觉得在分数机制筛选出来的特别会算的、特别乖巧的那种学生。他可能会放大他的那种理念，然后把他的这种理念给再传递给、强化式传递给自己的学生。

然后像我们现在的大学老师，他会进入到大学的管理体系，那我们选拔管理者的时候也是只看这种标准嘛。所以这个体系、这个制度建设就朝着单一的维度去发展。所以我比较担心的是这样的问题。就是我们会认为学而优则仕，那“优”的判断可能就是比较单维度、有问题的判断，进而他会复制这样的制度，甚至强化这样的制度。

受访者： 对，其实人被压抑了之后，比如说一个学生吧，如果在老师这儿他感受到的是老师是非常的压榨或者是剥削的，或者是对学术非常功利的态度的话。学生其实有的时候一方面可能自己对学术就没有什么憧憬了，然后另一方面如果他还想自己做学术的话，有可能以后对学生会办变本加厉，就是媳妇熬成婆了这种感觉

刘书博老师： 是的，那现在我感觉好像这个趋势越来越明显，像我们学院，就是有很多老师做学问等同于发论文，然后每年能发十几篇，然后他就搭建一个学术流水线，我们访谈的时候他也会坦然的说，生产就是垃圾，就是灌水。甚至我导师都会说这些都是没价值的。但是他要上岸啊，他可能就是通过这样的方式把工作做到极致，进而能够在评价标准当中突出自己。

像人文学科的价值不能够被明显的彰显出来，那似乎就被边缘化。那对于这种边缘化的趋势，我们是有办法去进行所谓的抵抗嘛。

受访者： 我觉得这个可能一方面是有个人的坚持吧，然后更广泛的来说，其实个人在这个方面力量是非常有限的。因为这个制度设计规则在这儿，你不按这个规则玩很容易就把自己玩死了。

我觉得国家在科研体系评价方面，内在是有张力和矛盾的。就是一方面是要破五维啊，是要防止评价成了一种数字游戏，其实没有真正有价值的学术成果产出。但是呢，另一方面在偏人文的学科里面，经常是被警惕的。我之前看到网上有一个规定上写的就是期刊发文都要优先省部级或者是国家级的课题。就是说人文学科方向有非常强烈的国家取向。因为课题都是提前拟好的，就跟选择题似的，列出了好多选题。

但是其实人文学科跟理工科不太一样，对于理工科来说，国家确实可以做顶层设计，就说哪些是我们国家目前比较需要前沿发展的领域，我们需要重点来投入。但是人文学科的想象力或者内在的创造力应该是来自于没有约束、就是思想自由嘛，本来就是一个关于思想的学问，还是保持一种批判的态度。但是这些东西，不一定是当政者喜欢的嘛。因为我本科学社会工作，然后这个社会学就是个讨人嫌的学科嘛哈哈，它不是说要去唱赞歌啊。所以做比较偏人文性或者有批判性的一些研究的话，很多时候国家还是希望把它统合到一个大的体系里面。但其实有些东西不用统合，不统合反而会让这个学科发展的非常好，让古今中外或者一切可以用的资源都可以生成真正的思想。要不然我们人文学科的发展还是会受到很多的这个限制。

就包括我之前也是有一个乌龙啊，就是我这本书本来印的就非常少，就印了几百本吧，然后后来就卖光了。我就看到有一个微信群里一个同事发给我的，就说有人在传这本书被封杀了，就哪都买不到了。当然这是一个乌龙啊，是因为那个出版社脱销了。但是就这么一个事情，大家真的有人信啊，你想就这样一本书如果说它都被封杀的话，那就很难想象什么样的东西才能够出版。

刘书博老师： 那就成样板戏了，那就样板的东西才能够出版呗。

受访者： 对呀，那我觉得我们思想就不可能有活力了，那我们生活在一个什么样的时代呢？这个时代就对做人文性或者批判性的学者就太不友好了。那只能是大家天天窝在家里水一水无关痛痒的东西就算了。

刘书博老师：对对，是的，是的。尤其是人文社科，要有一种桀骜不驯的精神气质，或者说所谓的自主性吧。那现在我们是感觉很多知识生产是需要自我审查的、批判性不足。尤其管理学，我们几乎都是站在效率至上这边、管理者资本家这一边。像前一段热议的系统对于人的压榨啊，我们管理学的视角就是说算法特别好，就有一种颂扬的，怎么去让算法发挥最大的对于人的管理。但是几乎没有中国的管理学者看被管理者、看劳动人民。那在欧洲这一块儿是有很多研究者都关注的，他们会有所谓的批判管理研究，但是中国管理学这一块儿是零。然后我前面也是参加一个管理学会议，我去提说能不能讲批判管理主义，那他们就说批判这个事情其实是敏感的，最好不要讲。我就感受到我们不能批判。虽然马克思很批判，然后我们又是把马克思看做是信仰，但是我们不能批判，这一点就很糟糕嘛。

受访者： 所以这个真的是有一点让人灰心的事情啊。但是像您这边其实也还好，就是有些东西也可以拿到国际上发表。像我们国内教育学也还好，但确实也有这方面的限制，比如说有些词啊，反正有一段时间就是“公民”都不太让提。那你说中华人民共和国公民，这宪法都要这么写吧，然后公民教育却不能这么提，那就有很多意识形态的考虑。其实我觉得是一种很不自信的表现吧，我们天天讲这几个自信，至少思想上要自信嘛，对吧？对自己的发展道路应该是保持一个开放性的态度，要不然最后就只能是自己说自己好，那就没什么意思了。

刘书博老师： 我就想到那个詹姆斯·斯科特，也是人类学家吧。他写的《国家的视角》讲的就是国家有一个宏大的、向好的、理性的计划。但是有时候挺自负的，自己想的跟现实还是有距离的。反倒是说我们去让一片森林繁荣，那你不要太改造它，你要关注它的生态系统发展，那生态系统土壤比较肥沃的时候，它自然就会繁荣。那现在我们似乎总是想这边切一块儿，那边嫁接一块儿，最后生长出来很多橘生淮北的感觉。

受访者：有时候我觉得自己写的东西比较空泛，或者是比较虚妄，或者是比较矫情，经常是对文字有这种感觉。那个博士论文还是经过很长一段时间的凝练修改，然后后面包括我导师也帮我整体上做了很多细致的修改。所以我感觉这个写作确实是一个需要长期去做的事情。

而且写作我觉得很重要一个是要言之有物吧，就是我感觉现在我读到很多东西都很学术文化。但是读了之后感觉他对我没有什么触动或者启发，就我没觉得他说出了什么东西，这个东西就比较不好。

如果是想做社会学、人类学的话，社会学、人类学这些经典的作品可能需要看一看。像我自己学这个，像费孝通的《乡土中国》现在已经进入到初高中的教材里了，就是他对于中国社会的洞察，还有对一些理论性东西的使用，我觉得是特别值得我们学习的，但这个东西又不是轻易能学到的。

还有一个我想最引导性的问题，就是我们自己的问题呀。还是要有自己真实的困惑、真实的问题、就和自己有关的东西。所以如果写东西的话，特别是毕业论文那种缘起的地方，其实很多同学有的时候会觉得不太重要。就觉得哎呀，那我就提出问题，我好好的论述文献，然后方法，然后结论发现。但其实对我来说的话，我会特别看重一个同学写的研究缘起，我觉得这个东西写好了后面才能成立。如果这个东西写不好，你不能让读者进入到你自己见过的问题域的话，其实后面很多东西有的时候就总感觉不太对。所以研究缘起是带着读者进入到你的那个独特的看问题的视角里去，如果读不进去，那后面就越读越读不进去。这是我的一个感受啊。

刘书博老师：您说这个就是缘起啊，其实我经常也跟同学们讲，就研究问题的引出也是你的研究动力和激情的一个来源吧。我觉得管理学最要命的问题除了范式的单一化和美式的霸权后皈依者狂热。还有就是我们的研究问题距离自己的生活太过遥远。学生在本科时候就研究董事会的决策、公司的治理模式、跨国企业在海外的投资决策等等。他们完全是从文献到文献，对于现实没有任何的感知。在这种情况下就比葫芦画瓢，然后做出来的东西呢，我觉得是两看相厌啊，老师看起来很头疼，他自己做起来很痛苦。最后就产出了非常多的精致的废话。现在我们本科写毕业论文百分之九十就是实证研究了，很恐怖的一个事情。但是现在我们这个范式导向让学生在本科阶段就只是完成一个比葫芦画瓢的工作。导致他对于研究的看法也非常的不正确，更不要谈研究的激情和兴趣了。所以我非常赞同程老师说的，我们在做研究肯定还是要带着一个激情、好奇心，然后才能够持续的把一些东西给挖掘出来。

吕梦娜： 程老师，刚刚我们说到这个研究缘起，我感觉特别有体会。因为我这个专业是企业管理，我一个没有去过企业的人去研究企业管理，就是这种论文，尤其是同学们写的论文，明显能感觉到隔了一层。就是这个问题，你再怎么引人入胜，感觉有点像假案例的感觉。但是做我们这个企业管理的，尤其是没有接触过实践的、没有进入过田野的人，就会觉得好像犹犹豫豫的不太能够接受你的这个观点。然后说到这个田野，我就很好奇，您之前去高中、初中做过这个田野嘛，去了很长一段时间，我就想知道在这个过程中你有没有遇到过到现在感触还比较深的事情？

受访者： 像你做的是这个企业方面的研究的话，要想做比较偏社会学、人类学，不做那么多这个假的、或者空的、远的这种研究的话，其实到企业里去就是一个比较好的办法。你就到一个企业里去嘛，比如说你研究某个类型的企业，那你就真的像人类学那种在里面以某种身份进去。我以前读过那个潘毅的，因为她是做劳工社会学，当然她去的那个企业可能都是制造业，都是流水线。但她主要是从人的角度去关心。这也是一种很好的方式吧，不一定都是研究董事会，都是研究那个很大的东西，可以从小的地方入手，大的东西一起考虑。

然后我自己做田野研究，比较深刻的事情，其实还是有挺多的。比如说我开始做探索的时候，先去这个农村的学校，所谓的这个成绩好的学生，大多数还是跟我这样一个外来的保持距离的。但是反而是那些不怎么学习的，平时就喜欢各种惹是生非，各种在学校里打闹、玩耍打游戏的、谈恋爱的这些同学，他反而会更主动的接近你，然后他们的也会做更多让你觉得有意思的事情。但是那些好学生是比较神圣的，他即使对你有好奇，他也不会有那种很热情的感觉。像我后来做访谈的话，我会感觉到人讲自己经历、诉说自己的这种愿望还是比我们想象的要大。就是我们有时候会觉得，哎呀，别人可能不好意思说，或者都不会愿意跟我说，然后怎么怎么样。但是其实每个人在合宜的情况下会愿意去讲自己的事情，也会有这种讲述的意愿。这些东西他不讲，它也会存在，但是它不是以有形的方式存在，他以一种不可见的方式存在，而我们很多时候还是希望能把这些不可见的变得可见。

就我们做社会学、教育学、管理学，很多时候你看做企业研究也是这个企业的经验嘛，企业的经验其实也是很多企业中的人的经验、人的生活、人的故事。所以最终还是要做偏质性的、偏人文的研究，还是要把这个故事讲清楚，把那个故事的复杂性体现出来。

吕梦娜： 对，我觉得其实大家能够有这种写作欲望，一个是本身有意愿，另一个我觉得您给出的这个激励也是蛮棒的。就是像我们做这个企业方面的研究，可能会做问卷吧，也会有一些“小恩小惠”吧。但是我觉得那个东西对他好像没有特别大的吸引力，但是您当时提到说，包括自己的自传可能会分享给大家，包括给导师的书。如果说我是这个被访的，我也会很愿意去分享。我觉得这是一个互惠的一件事情，我也能从这个过程当中学到很多。

那您当时提到了在田野的过程中，其实“家伙们”会跟您有更多的接触嘛。在这样的过程，你内心有没有什么变动？比如说因为您读过那个《学做工》那本书，可能大概能够预测说，这些同学未来会子承父业嘛。所以说您当时跟他们交流的过程中有没有一些内心的波动？

受访者： 刚才你说的意思是说我看到这些田野里的这些所谓的“家伙们”和这个威利斯笔下的“家伙们”有什么区别，是吗？

吕梦娜： 就是您观察到这些人的时候，包括您之前读的那本书嘛，然后您内心有什么感触？就是我自己代入的话，那看到他们虽然现在还很天真浪漫，然后生活很丰富多彩，但是可能会为他们后面的这个生活有一点触动，我是这么觉得哈，不知道您当时是怎么想的？

受访者： 因为当时其实去到那之后，中国的情景和英国的情景还不太一样，像有一些在中学的同学，他有时候还是很想要努力，但是又处于那种比如说某个学科特别差，比如说英语，我当时甚至还买过这个英语卷子去给他们，想要给他们补补课之类的。

但是像有些同学他就是完全放弃的，但这些同学确实也是没办法在学校里获得认可、获得意义，他确实是在那混日子，他也不一定就是为了抵制某些更宏大的所谓的阶级之类的。其实这些同学他们的未来也不一定就不好，因为他们有的家里做生意，有的以后也许会去学一个技术。像现在整个城镇化的过程之中，他们很多人还是可以在县城买房子，然后过上（好一点的生活），有人去工厂打工，但是也不一定就是这样。就是他们的未来也是比较多样的，但是它主要还是依赖于整个国家经济形势比较好。特别是近一两年吧，因为疫情加各种原因，反正我感觉各行各业的人，特别是在比较做不太稳定工作的，需要特别依赖这个国家经济的人，还过得挺艰难的。

吕梦娜： 了解。那“家伙们”比较能够映入你的眼帘嘛，那可能像“读书的料”会稍微默默无闻一些，但是您其实研究的是后者，您当时这个角度是怎么有意识的去聚焦过去的？

受访者：我开始就带着这样一个问题去的，所以我就是想要去关注这些孩子。但是其实这个田野做的也很失败嘛，后来就变成了这个成长叙事了。

吕梦娜： 了解，好的陈老师，因为时间的原因，咱们争取线下能够有更多交流。

黄秋莉：陈老师您好，就是我看到《读书的料》嘛，然后我就想起来我们以前同学的一个经历，就是我们也是从村里出来的，然后他高中非常的努力，可能就有这种您说的文化再生产的过程，他有自己一个先赋性的努力呀等等。但是他到大学之后，他就可能觉得自己的高中太苦了，然后就自己给自己放松了很长的一段时间。

然后就让我联想到刚刚您和刘老师提到的第一个问题，就是我们现在的这个学术场域中有很多“只做十年学术”的这样的一类学者吧。就是刚刚您可能和刘老师提到了很多这种社会层面、体系层面的原因，包括他从小接受的教育制度呀，或者是我们这种评价考核，还有这个“占据山头”。那就是想说据您的观察，还有没有别的原因，就导致了很多学者“只做十年学术”？

受访者：我觉得不光是做学术，整个社会都有一种这个功利化的一种文化的蔓延。其实我们传统文化里面，读书人或者说知识分子还是有一种就是道统的这个传统的。但是我觉得像在我们这样一个国家，经过那么多次的政治运动……

其实我觉得很多时候还是这个制度本身不够。就是大家不是每个人都想说什么、随便说什么都可以，这个制度本身就是这种在某些时期比较好，在某些时期就不好，他就有比较大的这个不确定性。然后每个人还是有一点“自我审查”，就自己给自己约束了。就慢慢的我觉得这个自我审查的东西、敏感的东西太多了，我们的语言就会越来越贫瘠，我们的这个想法也都会自我设限吧。而且这个东西有时候也不是说是从上面来的，就是慢慢的，人们自己就已经在心里结成了这种自我审查的机制。

然后学者的功利化，其实也跟各种各样的评价、绩效主义的这种管理有关。很多评价的情况下，他没有说去培养一个人的长久的热情。刚才刘老师说的特别好，就是说这个土壤建好了就行了，不是说还是要从下面拖着、从上面压着。有很多时候还是从上面来压着，总是来评价你，或者是催促你，来考核你、监督你。他不是说给你提供一个支持性的土壤，然后尽量少的去干扰你，甚至说这个土都空了，他还是要抓着你往上走，那这个后来长出来的就不可能。好。

黄秋莉：刚刚您提到可能以前的学者会好一点，因为我自己看的话，我就会觉得像钱理群老师呀，或者是像费孝通老师他们这种，都是有一种终身学习的志向在的。那您在现实生活中有没有看到一直做学术、一直对学术保持激情的这样的一些老师啊？

受访者： 那肯定是有的呀，还是有不少老师是我觉得挺有激情或者还是比较有学术坚守的吧。就还是想做真正的学术研究，不是说只是为了某种外在的东西去做，我觉得还是有不少的。但是的的确确就是这个分化也很严重。

刘书博老师： 就这个分化，我觉得其实我们应该把它写成英文的论文，我觉得西方学者似乎没有看到这个。但我不知道教育学领域怎么样？像我们有一个组织行为的这个organization study，还有human relations这两本，是我们管理学的顶刊。像human relations他有很多对于商学院老师的这个研究，但是他这个背景都是在欧洲，我看美国的也很少，主要是欧洲的学者在做，那似乎我们也可以去看一看中国的学者，然后描述一下，让世界认识认识。

黄秋莉： 对。其实我们自己在访谈的过程中，我就会发现好像跟那个布迪厄提到的一样，如果你有更多的资本的话，可能就会有利于你去抵抗住这种评价、考核或者是土壤带给你的影响。比如家里有钱呀，当然这可能只是一个方面。

受访者：当你就是不太需要通过学术来获得物质上的回馈，或者也不太需要通过学术一定要得到某些人的承认，就像某个阶段，或者有的大学是非升即走嘛，那如果我不需要这些，我就自己想做什么就做什么。我如果是一个有学术热情的人，即使我的学术研究的主题或者是我的研究范式不太得到承认，那我还是能在这里生活下去，就我还可以有这样一个学术职位，这是一个最基本的东西。如果这个东西能保障的话，就是说其实很重要的还是学术共同体内部的一个声誉的评价。我想国外很多学者他其实很在意自己的这个学术声誉。

当然这个外在的评价，外在的什么奖和荣誉啊也是一个方面，但是同行评价（对他而言更重要），别人觉得你的研究怎么样，或者别人觉得，哎呀，你这些年就没什么研究，或者说你的研究整个就变味了，或者是就那这个东西大家也都会在意你在意这个学术声誉，那这个就不是三年聘期或者是发某个期刊有多少钱。自己还想做出真正能被学界所承认的东西，这个东西我觉得是维护和真正建设学术共同体，而不是把学术共同体搞得乌烟瘴气的。特别是我们的很多期刊，如果它变成某个人的一个私人物品，或者是一个小圈子的一个自留地了，那对整个学术圈就是一个很大的破坏。因为你就迫使这个学者他必须要去讨好、巴结，或者是怎么样才能发表某个类型的文章。那这个学术共同体还有什么声誉可言呢？如果是这样一个状态的话，大家就会变得山头主义嘛，变得圈地文化，然后就完全没有把学术作为一个公共的事情，它的公共性就被忽略了。

黄秋莉： 就我自己的观察的话，我会觉得像欧洲回来的一些学者哈，或者是以前在企业工作过的很多学者，他们对学术的这种热爱好像就是会比较持久一点。

受访者： 有可能，但这个我不知道是不是有大量的访谈或者大量的数据的证实。但是怎么说呢？可能在不同的学术文化下成长，自己对学术的这个热情还是会有差别，他对学术的理解本身就会有差别，所以这个你说的这个有可能是有区别的。

黄秋莉： 就是影响他们对学术的一个理解，可能造成了他们对学术热情的不一样。

受访者： 我感觉不管在哪种学术文化背景下成长，可能很重要的还是你能感受到它的魅力，就是你能感受到你做的事情是一个有意义的事情，你能感受到它跟社会公益，跟整个社会甚至说跟整个国家的某些公共性的事业是相关的，就是这种感召吧。所以像韦伯说的这个学术作为志业，不管你在哪个地方，你能有这种感召，那可能你的学术热情就会比较久。

而且你在现实生活中你能感受到你的这些热情还是有同道中人，就不是说别人都是很功利化的，让你自己一个人这样，自己一个人真的还是挺难去长期的坚持的吧。最后就自己，只能变得非常压抑，因为你得不到任何人的认可，那这个过程就是憋着憋着。所以这样一个属于公共性的东西还是需要所有人一起来维护吧。

刘书博老师： 有一个非常大的意义体系，就是你做的事儿不是单独为自己，而且你可以链接到人类共同体的更宏大的意义。

黄秋莉： 然后第二个问题，就是我看到您提到“懂事”，我自己的感觉是我经常听到这个话，如果家长夸一个小孩儿比较好的话，就是说这个小孩儿好懂事儿。那除了“读书的料”在他获得学术成就的过程当中有这样一个懂事儿的因素，在我们现在大学当中有没有看到这种比较懂事的现象啊？或者“懂事”的内涵，我看您写的就是说能看到不合理的当中的一个合理性，那除了这一点之外，这个懂事的内涵有没有一个扩充呀或者是什么的？

受访者： 你说师生关系吗？

黄秋莉： 就懂事儿这个内涵来说吧。

受访者： 这个内涵我觉得它在不同的情境下是不一样的。比如老师有的也会说，哎呀，这个学生比较懂事。我说那个懂事还是在这个家庭生活中的。你刚才说的那个能看到不合理当中的合理，它主要讲的还是一种理解，接近同情式的理解，他能够去看到观念背后更多的东西。比如说我们观念不一样，然后我能够理解你观念形成的原因，就是你为什么会有这样的观念，而不是只是觉得我们不是同道中人，甚至就是敌人那种，或者就是完全不理解它的一种认知。

黄秋莉： 对。就像我们村里夸一个人懂事的话，有一个场景就是说如果你想买一个比较贵的东西，然后家长说哎呀没有那么多钱，然后你就说好吧。

受访者： 那这也是“懂事”，就是说更进一步的场景就是他根本就不会提。

黄秋莉： 当一个学生进入到大学以后，他这种懂事儿会不会就会体现出一种精致的利己导向？

受访者： 这两个还是完全在不同的情境下来说的，因为在家庭里面这种懂事还是一种利他导向的，但是从家庭的私领域进入到公共领域，那这种懂事他会有什么样的一个演变？一般来说的话，他在家庭中关心他人或者体谅他人，是在公领域道德的一个基础。我们中国人讲推己及人嘛，就是如果你对自己的家人不好，也很难说你就能对别人好。但是你确实对家人好，这是不是就意味着你能能把这种私领域的这个道德或者对大人的体谅和关心能够推广？那也不一定。因为有的人他是非常在意家人的利益，但是他到公共领域，他就是非常的就是家庭主义，他就对我好，我就什么都能干。

刘书博老师： 差序格局嘛，就中国人首先得满足身边亲人的，然后再扩展。所以可能亲人都满足不了他，更不要说扩展。

黄秋莉： 我感觉公共话语的懂事儿就是和私人当中完全是不太一样的，就是在私人领域能接受不合理（比如接受不去买一个自己非常喜欢但是买不起的东西），在工作领域它会不会变成一种对标准的非常清晰的认知，然后去服从这种标准？

受访者： 在公领域的话，很多时候你比如说有的在单位里面，有的时候大家也会说，哎呀，谁谁谁他就不懂事儿，或者他可能乱说话，对吧？那个懂事儿和家庭中的懂事儿指向是不同的。

黄秋莉： 好的，那我没别的问题了，谢谢程老师。

刘书博老师： 那今天大家线上交流了好多维度问题，我受益匪浅，也很开心。我认识的一个人，他其实不是典型的读书的料，他只是在高中好好读书，前面非常调皮，后来考上的是西安财经，但是他后来在工作就还是比较顺利的，也不是说顺利吧，就比较幸运吧，也比较能吃苦，现在就是在做一个企业的联合创始人和管理者。那他的这种心路似乎跟在学校当老师的这个寒门子弟的心路又有一些不同，包括像刘强东这样的。如果能够了解他们的这种内心世界，我觉得还是挺有意思的一件事情。所以我们看这个未来如果有机会的话，还是能够在线下大家一起交流。

受访者： 那太好了。

# 受访者26

刘书博：上次聊就是到这个1998年，然后后面我们也看了一些您推荐的材料，包括《20世纪管理科学》，其中有一部分专门讲中国的管理学的发展，所以还结合这个钱颖一，他作为清华经管院长那么多年讲话的合集，那我这边读了之后我有一个可能是一个错误偏见印象，就是我觉得中国的管理学科好像是完全的自上而下的，好像就看不到太多的这种学术共同体的、这种自下而上的感觉。

我这边看好像就管理工程类，这个似乎是从建国到现在它的发展还是比较一致的，就是他还是理工科的这种发展，但是从新中国到计划经济一直到改革开放之前，因为管理学它主要是涉及意识形态，包括经济学，包括社会学，其实那段时间都是空白，所以真正发展还是在改革开放之后。

然后改革开放之后呢，我们可能就是首先还是倡导科学性，管理科学，然后因为也是钱学森、华罗庚他们这些人一直在推动，其实他们是自然科学家嘛，所以我看到就是管理学从在中国诞生就带有很强的这种自然科学的影响。后来我们在朱镕基时期又强调国际化，那这个那国际化其实就主要还是美国化，然后美国的管理学也是非常这种自然科学导向，就是实证主义研究，所以其实我看到的是似乎我们的管理学研究要么是从上到下的这种要求，要么就是对于外部的学习，似乎没有自己太多的东西，我不知道我说的对不对。

受访者：一定程度上有道理。大概的情况我觉得跟你的那个判断或者是归纳应该差不多，几个方面的原因，就是说一个呢就是说中国长期的那个从封建社会然后几乎没有比较正常的这样的一个所谓的资本主义的社会阶段，民国的那个时间很短嘛。而且呢这个1949年我们有一小段儿新民主主义时期，但是很快呢就做了社会主义的过渡，那么就一直按照这样一种模式来做。所以那个时候呢就是学苏联、学计划经济的那套东西，那么使得政府，特别是中央政府，对这种资源呀、调配呀、计划呀、控制等等方面，几乎全部是这样的一种力量或者是这样的一种资源。

所以很自然的就是说文革结束以后，我们大学重新恢复我们的教学体系也好，然后我们去做的一些叫政策咨询呀、企业服务呀等等这些东西呢也都没有逃离出社会性的、政治性的或者是政治经济紧密相连的这样的一个大的体制性的框架，但这个事儿呢实际上我认为就是很快呢我们自上到下大家在改革的过程当中呢都发现了它是一个问题，就是说我们后来的这个改革把它导向了市场化，那么实际上就是想去弥补原来政府这一只手来掌控所有问题、掌控所有资源，特别是其中低效的、无效的、甚至有的一些方面我们讲可能是失败的。

一方面，你刚才讲到这个自上而下和自下而上。那么最早这个经济体制改革从农村开始我们叫包产到户也好，那么他实际上就是把政府的权利，哪怕包括那个时候在基层的乡镇、村一级的、这样的一个集体经济的集中的权利还给家庭，就还到最小的生产单元。那么在工商领域，我们一般都是说企业，因为我们过去是叫全民所有制经济，这是占主体的，所以呢就是说国营企业（现在当然我们叫国有企业）是占绝大多数的地位。那么这样的话就是说如果要改变这样的一种所有制，或者是所有制掌控的这种资源，他这种经营方式就等于是我们的整个的经济体制就要做变化，那这个方面实际上是有一定的争议的，不管我们现在有的时候笼统说什么左呀、右什么之类的，或者什么这个市场派呀、计划派呀等等，那等于是一个很现实的东西。这里面呢我举一偏小的文章，你回头查一下，好像是蒋一苇写过一篇叫《企业本位论》，那么他实际上核心的意思呢就是说我们要放权给企业，让企业成为我们现在讲的独立经营、自负盈亏的这种市场的主体，政府呢来做政府应该做的这个事情，不要越俎代庖去代替企业做很多经营、开发、运作等等这方面的工作。那这个呢就拉开了经济体制改革，特别是企业体制改革（的序幕），体制要改革了以后呢，就是计划体制要改革配套，对宏观的、中观的，那么这个问题呢实际上跟管理学或者是我们讲的商学有一定的联系，但是它还有很大的一块儿呢是跨在这个经济学范畴里，所以你像那个厉以宁也好、吴敬琏也好等等，他们我们都还会统称为是经济学家。

但是这个比方说股份制改革也好、股票市场也好、价格机制也好、定价机制的改革也好，这么这些东西最后要去落地、要去操作的时候一定会碰到企业，一到企业的这个层面，那上去就很自然就出现了，包括后来我们讲这个叫《企业法》或者叫《公司法》，我们很早的时候定《公司法》还是分类定的，如《外商投资企业法》或者是《民营企业法》等，当然现在我们后来把它统一称之为《企业法》，现在还直接把这个《企业法》放到《民法》里面，那么这样的话就是说，我们更多的是从企业形态上而不是从所有制上去划分，那么使得对他们的研究，对他们的一些改革发展的这种实践呢就更具有一般性，就跟甚至国际上的一般的这种企业的认识就越来越接近，那么这里边呢我们还回到这个我们讲的这个商学院也好，或者是管理学院也好等等这样的一个学科的发展。

我前两天又把我们上次聊到的有几个东西呢我稍微理了一下，那么跟上次呢可能稍微有一点点小的交叉，那交叉在什么地方呢？上次我们差不多聊到这个1998年、2000年前后那个时段，是吧，那么我记得就是说98年的时候教育部呢颁布了一个新的本科专业目录，独立设置这个管理学这个门类，实际上呢比他早两年，是1996年的时候呢，国务院学院办颁布了一个研究生的专业目录，就是说我们现在的本科的专业目录和研究生的专业目录是分开的，不是统一的。所以这样的话就1996年的研究生的那个专业目录，那里边呢已经把管理学单独列出来了，所以1996年研究生的专业目录实际上是给1998年本科的专业目录独立设置管理学门类呢等于打了基础，这是一个我认为是蛮重要的一个工作。

那当然还有一个呢就是上次我们也聊到了，就是1996年的时候呢国家自然科学基金委独立设置管理科学部，然后讲到“管理科学、兴国之道”的一个讲话，那么刚好呢在这个1997年、1998年的时候呢，我在西交大的时候呢，我导师席酉民老师主持承担了一个基金委管理科学部的应急项目。

这个背景是什么呢？就是说1996年基金委独立设置管理科学部，然后呢朱镕基出面请成思危担任管理科学部的首任主任。那么那个时候成思危在化工部担任总工（也可能是副部长），那么他同时呢还是西安的农民民建的主席，就是这个民主党派的民建的这个主席，但是那个时候呢他还没有进人大，那么我们国家的几个主要的民主党派的主席或者是常务副主席都是在人大或者是政协要安排副委员长或者是政协副主席的这样的一个副国级的位置，对吧，但是那个时候成思危他还没有到正常的人大换届，所以他还没有担任副委员长，但是他去了基金委以后呢做了几个蛮重要的工作，一个呢就是说设立了应急项目，因为他在化工部，然后呢跟国务院的高层有比较密切的一些联系，然后呢他在80年代早期也是我们国内派到美国去比较早的算公派去到那边去读了学位，而且他读的就是MBA回来的，那么他自己本身又有一个家族的一个渊源，就他们家以前是资本家，后来他回到大陆了以后呢，等于是他就跟家庭、跟过去的历史等有一个切割的过程。所以后来文革结束了以后，由于成思危化工方面的专业还是很不错的，所以就把他带到美国去学管理，所以他等于是技术和管理两个方面都兼顾、都擅长的一个学者吧，然后他也写了很多东西，所以他很知道，就是说高层、政府在做很多决策的时候，他们会对什么东西觉得很困难，或者是觉得把握不准，或者是需要做一些比较系统的分析和思考，这就是他设立这个应急研究项目的一个基本的目的。

那么应急项目的实现一般不超过一年，就是说这个基金委科学部公布出来了以后在不长的时间里边，接受申请了以后他们会做一个评议，然后很快就确定有哪家或者是哪几家合作一起来承担，然后要求在半年到一年左右的时间呢就要拿出相应的研究成果，因为时间很短，所以他不要求你的理论上或者是论证或者什么之类的，是多么多么的严谨，但是呢就是大的框架、大的思路方向要能够给出一些重点的把握，所以这些应急项目呢比较多的是给政府决策来提供服务的。

那么刚好呢在1997年的时候呢，那我在西交大的时候呢，我们承担了当时的一个应急研究的项目，就研究企业和政府的关系。那么当时呢他们根据各个学校报了的这个情况呢选了四家学校，清华、西交大，另外有南昌大学，还有一个是哪个大学我忘记了，分别开展研究。清华大学主要研究美国，然后呢南昌大学甘筱青老师他是留法的，所以呢他带了一帮人主要研究法国，然后呢我们研究新加坡，还有个谁家大概有那么五六家要分国别去研究，研究完了以后呢在这个1997年的8月份的时候呢，那个基金委在北航组织了一次阶段性的报告，还没有完全结束，但是说你们阶段性的一些研究成果，那么大家去做一个汇报，那次呢因为我导师席酉民老师刚好在国外，所以呢我就代表我们学校去做一个汇报，而且那个汇报呢把我排的比较靠后，然后前面的几个老师呢都讲的内容特别丰富，当然时间也就比较长了，那么到最后呢给我留的时间就很少，所以呢我把我们那个报告里面最核心的东西讲给成先生听了，就是说因为各个国家里面政府和这个市场企业的这样的一个关系都不完全一样。然后呢我是把新加坡里面最核心的那个特点归纳了几条，那么同时呢也点出来了，就是说这些体制的形成，他的这个历史的渊源，这种国情，这个国家经济包括企业发展阶段的这种适应性等等这些方面，会后相应的反馈还不错。

所以呢那次汇报完了以后呢，这个基金委就把这些分散的国别的研究合在一起了，然后呢要求整合出来一个“中国政府应该跟企业之间建立一种什么样的关系？过去我们是什么样的？现在是什么样的？未来我们应该往什么样的方向转换？”所以呢等于是那个应急项目的后半段就是我们几家有相应的一些协作，然后去找一些具有共性的、特别是其中有哪些是适合中国的一些特点。那么类似这样的工作呢，这个基金委的管理科研支部呢，一直到现在为止也还有，但是我后来没有再去持续的去跟踪，就是这些应急项目的提出，然后呢他的申请或者是评审，那最后的这个验收等等，跟我们讲的这个面上项目或者是青年基金项目还是有一些不一样，那么它的一个主要的方向是为政府服务，那么这样回到前面，我们讲那个自上而下的这样的一个话题，就是说政府需求。说的不好听一点，就是有一定的刚性，那么政府掌握着权利，政府呢又给你研究的资金，甚至呢还会给你一些研究的渠道，因为那个时候出国或者是跟国外的这种专业联系已经有一些了，但是也不是说随便你想出国，然后想去跟国外去联络或者什么之类的都就能够很顺利。这个等会儿后面我会讲到2001年我们在西安开一个国际管理会议也有一定关系，这是成思危在这个基金委主任上做的其中的一个我认为是蛮重要的一个工作。

第二个工作呢就他总结或者是归纳形成了一个三学科的体系框架，就是我们现在讲的管理科学工程、工商管理、公共管理，就是说基金委管理科学部现在底下有三个处，管理科学工程叫一处，工商管理是二处，公共管理是三处。早期的时候呢没有那么多人，二处和三处呢是合的，现在好像是分开了。那么这样的话就对应的是我们现在讲的那个学科目录里边的管理科学工程一级学科、工商管理一级学科、公共管理一级学科，那大概是这样的一个架构。那当然以前我们讲这个学科目录的设置的时候，里面还有什么农民管理，图书情报管理等等，但是那些呢在基金委学科的这个框架里面，它都把它分散在不同的三学科的框架里面，这个三学科的框架呢，对于后来我们在比方说一些研究方向、研究团队、研究资源的这种整合呀、设计呀、聚焦呀等等这些方面呢，还是有比较大的影响的，为什么呢？因为基金委的指挥棒重点呢就在往这个方向去做了，为了配合他这样的一个工作，那当然基金委老早也在做这个工作，就是说每过个四五年的这个时间呢他就会做一次那个叫学科规划，这个90年代早期就曾经做过，但是那个时候呢因为管理学科的规模和体量都还很小，那个时候只是一个组，所以呢没有产生太大的影响，后来成立单独的一个学部，然后那个经费呀、项目呀、甚至包括杰青也好，或者优秀群体也好，而且优秀群体本身资助力度很大，他还是可以申请持续资助或者延续资助等等，那么它里边所配置的这个项目的研究经费力度就很大。所以呢他做的这种规划、他做的这样的学科布局，对我们的学校、对我们的学科规划，对我们的学科布局就有非常大的导向作用。这是我认为成思危做的第二件蛮重要的事情。

第三件蛮重要的一件事情呢就是办国际会议，有一个叫international conference on management，这个会议呢我有点忘记第一届是在哪儿开的了，因为成思危在国外经常跑，所以呢他一开始的设计呢是这样，这个会呢每两年开一次，一次国内开、下一次在国外开。那个时候我也比较年轻，直接参与这方面的工作也比较少，但是到了1999年底、2000年初的时候，我们临时接到了一个应急的任务，就是说本来应该在2000年由新加坡承担的那次会议，因为其他的一些原因没办法承担了，但是呢这个成思危呢还想把这个会议继续做下去，不要就因为这个事儿呢就给断掉，然后呢后来基金委管理科学部就把这个会转到西交大管理学院，说你们能不能很快组织力量来承担，把这个会组织起来、做起来，哪怕时间稍微往后推一推。所以后来我们就临时抽调力量来准备，但当年就没办法开了，因为还牵扯到会晤、征文、出文集呀，那个时候的条件，不像现在这样可以找一家会议公司外包一下，完了以后呢要出版一个论文集，那个会议论文集是一定要正式出版的，那个时候呢这个很多出版社还有书号等等这样的一些比较具体的一些限制，所以呢用了大半年的时间做了一些准备。所以到了2001年的5月份在西安把这个会开下去，那么后边呢就又继续的这个会。因为这个会议呢是以中方为主导的，而且呢基金委是作为主办，那么学校或者其他机构呢作为承办，也邀请一些国外的大学或者是一些其他的基金会呀或者什么之类的可以来参与协办，所以呢他的这个影响呢还比较大。至少在这个国内的这个管理学界它有点儿像奥运会员，因为它不是一个专项、单独某一个领域的，它是一个比较综合的，所以成先生还在世的时候，他基本上每届那都会去参加，这是一个。

另外一个呢就是刚才我讲到就是说每过个三、五年基金委呢他要做这个学科规划，在这个规划里面呢比方说学科发展方向也好，关键的一些选题也好，或者应该有一些突破性的技术、工具等等也好，所以每过个三、五年的时间，大家都会去做一些梳理。

我是直接参与过其中的几个工作吧，一个2001的那个国际会议，成先生是大会主席，那么我是会议的秘书长，然后2003、2004年的时候呢，我们承担了那一轮的学科规划的编制，然后呢专门在中期的时候呢在北京九华山庄开过一次应该是相对来说是比较高层的专家研讨会来征求大家的意见。那基金委呢他有一个相当于学部委员一样的、大牌的一些教授，就是像杰青呀，或者是重点项目等等，最后评审的核心的就是这样的一帮人，那么像这些，成思危他都是亲自要去参加的。

而且呢，大概是在1999年、2000年左右，那么人大换届后成先生已经做人大副委员长了，所以2001年他到西安去参加国际会议的时候呢，是以人大副委员长的身份去的。那当然就是我们接待上是有点儿麻烦的，但是呢就是他去做演讲，然后呢他讲问题的这个视角，或者高度，跟就我们普通的学者就有很大的不同，所以这是他很重要的一个我认为我们叫引领也好，或者是带动也好，这是我想到的。

所以刚才你讲到的就是自上而下和自下而上，你想吧，就是说如果真的有一个普通的学者也好，或者是某一家企业也好，这个方面做的还不错，但是他的这个影响仍然我认为是有限的，这个是有限的一个可能是范围有限，再一个呢它的这种可持续的时间往往也是有限的。所以这块儿呢就是说关于自下而上的这个问题，后边呢等会儿我再讲几个其他的例子，可能稍微会有一些弥补。那我先聊着，然后那个你中间比方说觉得我可能差的比较远了，你可以把你想到的问题提出来。

刘书博：好的梁老师，那成思危他是经济学家，我看他这个专业背景，但是呢他父辈应该是做企业的，好像资本家这样，所以他对于这个工商管理应该是比较有这种概念，就是他知道应该怎么样去发展。那像成思危先生之外，还有其他的这种关键性的对于学科发展起到推动作用的人吗？像朱镕基他算吗？

受访者：成思危呢？因为他文章比较多，就具他的名的文章，那当然有些文章呢是跟别人一起合作的，但是有些呢可能是他出思想、别人成稿，那最后是以他具名来写的文章是比较多的，所以就光他在基金委管理科学部主任的这个职位上所做的一些工作都是非常有历史价值、历史意义的。

另外呢，他在几所大学里边设立了几个研究的基地，中科院、管理科学学院，然后北大有一个复杂性研究中心，中科院的那个叫什么中心等等吧，然后要研究几个问题，那几个问题呢实际上有一部分是经济的问题，有一部分是管理的问题，所以呢严格来说他你说他是一个纯粹的经济学家，肯定不是的；你说他是一个技术专家，因为他化工方面还是有很多技术的，也不完全；那么他讲的一些管理上的一些东西，你把它笼统的都归到那个经济学里面，肯定也是不合适的。所以这块儿呢他的这个评价应该是一个比较综合的。

我们先翻过这个成先生，我想到的就是具体的人物方面的一些东西呢，我还没有再去做其他的一些梳理，反正那个上次讲到的几个管理学家的那个传、就那两本书里面，其中有一些吧，那个回头你们再看看。

另外一个呢就是我想到的差不多2000年前后，对我们现在的管理学或者商学院的发展比较有影响的，大概有这么几件事情。就大学合并和扩招。那个时候李岚清不是在主管教育的时候，很多学校合校嘛，比方说四所学校合成浙大，然后我原来在西交大，我们是西交大财经学院和西安医科大学。

然后后来很快就搞了这个扩招，这个呢对我们现在讲的这个管理学科或者商学的这个发展呢是有很大的一个作用的。就是说那个时候资源还是比较紧缺的，什么叫比较紧缺的呢？就是说缺钱、缺地儿、缺设备、缺人员，那么这个时候怎么办呢？比方说我要扩招的时候招什么专业好呢？那些需要比方说实验室、实验工厂、实验设备呀这样的一些条件的专业，比如说计算机呀、炼钢、炼铁等等一些东西，那就需要比较多的增加一些装备、钱等。那相对比较少的，我们有时候讲叫轻资产的这种专业，比方说文科呀、商科呀，相对来说你原来三五十个人一个小班，你现在稍微弄个九十人、百八十人稍微稍微再多一点是比较容易扩招的，所以很多学校的招生规模的扩大，比较多的不在一些硬的学科专业里边，比如说医学完了以后还要解剖，那么他是不太容易扩招起来的，包括一些个专科。

所以我们现在形成比较大的一个商科的这种体量，那这样的一个基础实际上是有一定作用，当然不排除这个空间里边水平高低不一样、良莠不齐，但有一句话我觉得还是有一定道理，就是清华经管院原来那个老院长赵纯均，2000年前后我们不是开始搞那个MBA的合格评估嘛，那么他到各个学校去就给各个学校的校长讲他说要“有数量才有质量”，意思就是你们不要限制这个MBA的发展，MBA的这个发展过程当中一定会有问题的，那他的这个质量的提高不是说做精才能做好，做精肯定是能做好的，不排除，肯定是能做好的，但是呢你的贡献呢实际上有限，你发展到一定规模了以后、发展到一定量了以后，你的短板到底在哪里？你可能看的更清楚，那么这样的话你有针对性去补那个短板，对吧，那么的这个质量、水平才能够跟上来。所以呢大学的合并和扩招，对商科专业的这个发展整体上我们应该可能还是要从正面来去给他评价，那这是我自己的一个基本的观点。

刘书博：那这个时候扩招的师资能跟上吗？就像比如说理工科他们需要一个实验室呀，那同样这个文科虽然说不需要实验室，但是老师这方面似乎也要数量有所提升，才能跟得上扩招的这个学生增长的要求。所以师资我们是当时也是跟着扩大了嘛，就比如说博士的培养规模，应该也是从那时候开始的吧？

受访者：是，因为本科扩招了以后呢，很快研究生也就相应的包括硕士、包括博士也在扩招，这样一扩招了以后就带来了一个问题——老师课上不过来，学生带不过来等等这样的问题。那么所有的这样的一个改革、推进一开始呢都不是齐步走，或者大家按比例来去做，所以有些地方呢就开始出现了师生比比较高的问题——一个老师要带几十个学生，二三十个、三五十个等等这样的一些情况。那大家就发现老师少、课上不过来、学生带不过来，所以就适当增加教师嘛，那么后来教育部也开始有一些相应的政策来降低这个师生比，所以后来搞这个评估或者什么之类的认证之类的这些东西的时候呢，像这种师资配置，比如说量、结构上会有一定的要求，这是我认为跨世纪的时候一件比较重要的事情。

还有一个更大的一个背景呢就是911和中国加入世界贸易组织，这两个都是在2001年的事情，那么911对国际的政治经济等等影响很大，那么中国入世了以后，有一种说法就是中国刚好借了这个美国顾不过来的这样的一个机会，然后呢谋求了一个比较好的一种发展格局。那么很自然的，这样的一种机会、这样的一种发展也带进了管理学或者商科。中国整个的发展了以后，比方说我们给教育的这个投资然后呢有相应的这样一个增加，还有企业呀、经济呀、市场等等的需求，有很大的一个释放。举个例子，最早以前我们MBA的学费几千块钱、万把块钱、2万块钱大家都觉得挺贵的，对吧，那你现在花8万、10万、20万、三四十万，光MBA就已经到这个水平，对吧？那么这个呢实际上我们有时候叫文化环境也好，这个大的土壤也好，这个大的机会或者是影响。

还有一个呢就是说入世了以后中国开放，开放了以后不光是经济、贸易、市场的开放，文化的、教育的、科技的、国际的效率，包括人员的也带了一个机会。所以呢2002年，IACMR，中国管理学者的那个学会，对吧，2001年筹备，2002年创立，差不多也是在这个时期，那么所有的这些事情呢我自己理解呢，它实际上都是有一个前史的，就跟共产党一大，他不是随随便便就21年7月份就在上海开了，对吧？前面做了很多很多的基础呀、铺垫呀、准备呀等等一些工作。那个IACMR在成立之前，因为他最初的发起是徐淑英他们这些就是海外比较有成就的一批华裔的角色，或者我们叫管理学家吧或者管理学者，对不对，那么原来他们为什么在美国不搞，对吧？在英国不搞，在中国香港不搞，为什么要到中国大陆来搞？而且是2001年的，差不多那个时候2002年创立，差不多呢在这个香港回归之前，那香港新设的科技大学新成立了，新成立科技大学了以后呢面临着一个问题，就是说是招老师，面向国际招老师，而且那个时候呢香港当时还没回归嘛，所以呢香港科技大学呢给的待遇条件比那个时候香港的其他的所有的大学给的待遇的条件都要好，甚至高过了欧美的一些学校，那么这样的话就有一批海外的学者，特别是其中有一批华裔的学者回到了香港到了香港科技大学，像徐淑英，像那个现在的那个长江商学院的那个夏冰都是从美国加拿大回到了香港科技大学。

刘书博：还有那个中欧的昕荣，好像也是港科大的

受访者：昕荣前面还在新加坡待过一段时间，我印象是这样，完了以后再到香港，然后再过来。等于是这中间呢给我们培养了一批人一样、搭那个桥一样的，然后呢把这些人呢聚到了我们的周围，因为它离我们近了，我们的可获得性就更强了，所以后来呢包括上海交大也好，北京大学也好，清华大学也好，包括原来咱们西交大也好等等，然后呢就开始比较广泛的、比较深入的跟这些学者们开始有一些接触、一些交流，然后请他们来讲学也好呀，给他们做这个兼职教授也好，甚至给他们做名誉院长呀等等这些东西。那在这个过程当中，除了当时兼过这个教授、给你讲两门课、带两个学生之外，他们总觉得那个时候中国的管理研究的规范性或者整体的水平或者运用到的工具方法，可能还比较low，所以呢很自然的就把他们在欧美的一些套路开始想往国内来做介绍。所以在IACMR成立之前，徐淑英他们有一帮这个圈圈里边的一些学者呢已经开始在国内的一些相对比较好的一些学校、有一些渠道的一些学校里边呢，开始在做一些比如研究方法论等这方面的一些讲座呀、培训呀等等，所以像这些给IACMR的2002年的创立都做了很好的一个铺垫。

那与其零零散散地去做那样的一些工作，我们干脆统一搞一个机构，对吧？然后完了以后呢大家协调一些工作，不要去做一些简单的、重复性的事情，所以这个机构成立呢，对我们整个国内商学的研究、管理的研究呢还是有很大的影响，包括我们这些研究成果出来了以后国际性的发表等等还是有很大的促进作用。

再一个呢他也是搞双年会，那而且呢通过他们在海外的这样的一个学术的网络搞的有声有色，因为每次都能请到一些国外的这个领域里面的大牌或者是比较知名的一些学者，后来还增加了博士论坛，workshop之类的一些东西，那等于是我们手把手的来教你怎么选题、怎么做研究、怎么设计方案、最后怎么成文、怎么改稿子等。所以这个呢比我们过去要靠自己去摸索会有很大的一个提升作用，这是一个我觉得需要去关注的东西。

还有一个呢就是上次我已经提到过了，就是MBA的合格认证，第一批应该是这个2000年做的，然后我前两天又查了一下他的资料，2012年，这个国务院学位委员会和MBA的教育指导委员会又搞了一个高水平MBA的认证，这中间跨大概有12年，那么这个中间呢又穿插了一些国内比较好的一些院校去做国际的认证

刘书博：那梁老师这个认证这个事情是只有商学院才做吗？我看这个其他就经济学院、心理学院他们似乎没有这么重视认证这个事情

受访者：对，因为本身这个认证呢在国际上呢也是商学院的玩的比较多、玩的比较热闹。

刘书博：那为什么是这样呢？为什么我们商学院好像这个特别的突出。

受访者：这个我没有专门去研究过，但是呢实际上目前现在国内的其他学科特别是工科也玩的挺厉害，为什么呢？就是说因为我们那个WTO加入了以后呢，很快开放了以后呢，就国际的要进来、我们要走出去，很自然就开始有一个互相的学历认可的问题，那么这个认可的话，那你们的水平我得评估一下，对不对？那你评估按什么标准呢

刘书博：那其实也是国际交流在推国际化。

受访者：对，对，我们可以提到前面我们讲到的211、985。211的这个提法呢，就是21世纪我们要建100所国际一流的学科，那么985工程呢，就是建设世界先进水平的一流大学。就是说你要形成国际一流，那你就得和人家可比，而且呢不是我们关起门来说一流，别人也能够认可，所以后来呢教育部成立了一个高等教育教学评估中心。

我那天还查了一下他的底子，大概2004年成立评估中心，现在它是一个独立的下属机构，应该算事业单位吧。吴岩，就现在高教司的那个司长吴岩都是做过他（高等教育教学评估中心）原来的主任，然后现在的主任叫范唯，原来是吴启迪的秘书。他们最早做的教学评估实际上比较多的集中在工科的认证或者叫工程任证。对于工科专业，他们按照国际上通行的一套认证体系，做了一定的变化后，然后用在中国，或者是在我们原来的那个工科认证基础上呢做了一个变化来去适应国际上的一套，因为国际上的工科认证呢有一个叫《华盛顿协议》，国际上大家公认的。

大概在2017年有一次刚好那个范唯到浙大，他们在这边来做几个专业认证的时候呢，我专门去找过一下他，为啥呢？教育部呢后来要搞除了工科以外其他学科的认证，就是我们现在讲的叫医科、文科、商科、师范等等，那么在这些认证里面呢，国际上原来医科、文科等这些是没有统一的，然后呢我们学院呢MBA我们是做过一个认证的，然后呢这个合格认证就是我们国内的这个合格认证我们也是做过的，但是国际的那个商学院的这个认证里面呢它有两个范围大小的差别，像我们刚讲的这个合格认证的是只局限于MBA的，就本科呀、博士或者学硕什么的，这些他是不涉及的。但是像EQUIS或AACSB呀，这都是整个学院的，那么这样的话就带来了一个问题，就是说我们后面呢想做那个AACSB的认证。那么后来我就去请教范唯，我说我们把AACSB的认证做完了以后，是不是如果将来你们要推这个国内的商科认证，我们是不是就可以不用做了？为啥呢？因为他们工科的认证，比方说我们学校有个机械工程、有个化学工程、计算机工程这几个都是通过了这个我们国内的工科认证的，这个工程认证完了他们就跟国际认证是等效的。就你不用去做这个国际认证了，等同于你已经达到国际水平了，大概是这么个意思。那么后来我就去请教他，我说那我们如果比方说后边我们很周密的组织一下，我们通过了，因为国内AACSB已经有一批学校了，但因为他做的工作量还是挺大的、工作难度也还是有的，然后我说我不可能就是这边准备的AACSB，然后过两天呢你们那边通知说我们国内商科也要搞认证，然后我们再同时再组织一帮人再来应对你这个，当时范唯给我讲的意思呢，他们现在光做工科的认证都已经做不过来了，所以不可能顾及理科呀、文科呀、商科呀、师范呀等等这些其他的专业，这是当时他的话。

那么给我的一个反馈的意思就是说短期来看商科的这个认证呢是不可能一下子全面铺开来做的，但是呢现实的情况呢是这样，就是说有两个方面的这个影响，影响在什么地方呢？一个呢就很多学校在招生的时候他会打这个牌子，我们学校通过了什么什么认证？但认证的范围大小，外行人也不是太懂的。比方说央财，然后你说我们通过了什么什么认证，但这个认证是你们金融类专业的认证，还是公管专业的认证，还是其他专业的，有时候不是太管。然后是他是面向这个研究生层次的呢还是本科生层次也覆盖，这个时候不太有人管，但是他能起这个宣传作用，所以很多学校呢现在还去做认证，甚至有些学校呢包括我们学校自己主动做认证，我们学校做了五年规划，五年之内所有的工科专业你们都必须去把那个工科认证做了，而且都必须拿下。那这就是行政命令，对吧？那你其他专业没有认证怎么办？我们学校自己搞，然后校内请专家、校外请专家，所以呢我们是四年搞一轮，我们学院呢刚好是四个专业，我们管理学院刚好是四个专业，然后就排好了队了，然后今年做哪个、明年做哪个，像过筛子一样就要过一遍。

那当然你说这个认证好不好，你如果真是不用花太大的力气、不用投太多的资源，我们讲低投入、高产出，我们今天讲以评促建呀或者什么之类的东西，是吧？但是呢现在比较多的呢做成了一种形式了，就是说反正我最后给你包装出来的这样一个材料、这样的一个样子，最后一看合格的、有特色的、还不错的，当然也有些问题那你们再去整改吧，类似于这样。所以呢早些年我们国内搞的教学评估、合格评估、审核评估，包括MBA搞的这个或者商学院里面搞的这个相应的评估，或者是现在搞的什么专业的认证等等这些东西。整体上呢就是对各个学校，比方说管理的系统性、规范性、标准化呀等等这方面的，多少还是有一些促进作用的，就不是说我反正我关起门来我自己想怎么样我就怎么样，为什么呢？因为大家相互一交叉了以后，你发现诶谁谁谁家做的还是很有特色，相互之间呢也有一些交流、一些学习，然后呢这个我们的“以评促建”还是有一定的带动作用，总是能够发现一些短板嘛，然后呢慢慢的去做一些弥补，所以这是一部分我觉得比较有意义的一些工作。

还有一个呢就是更早一点，这个是非常官方的啦，就是说我们经常讲的学科评估。这是面向研究生的，就是我们现在讲的这个学科评估早期一点的呢我们叫重点学科，我查了一下，国家曾经公布过三次重点学科，比较早的公布过三次，1986、1987年全国曾经公布过416个重点学科，所有的专业都算，然后2001、2002年中国做第二次学科评估的时候呢，全国有964个，然后2006年的时候呢是第三次，这次做的就比较复杂了，然后有286个一级学科是重点学科，然后677个二级学科是重点学科，而且还增加了217个培育学科。这是关于这个重点学科的这个评法，然后今年我们不是又在做第五轮的那个学科评估吧，央财是不是应该也参加了？

刘书博：对

受访者：然后这个学科评估呢比较多的是从研究生教育的这个角度上去做的。然后我看了看他的这个相应的资料呢，大概是这样，就是说2002年做了一轮，算是第一轮，就是他差不多四年做一轮，那他的那个结果呢是大概在2004年公布出来的，所以呢有时候我们也算2002年，有时候也算2004年。然后第二轮呢是大概2006年开始做，然后2008年结束，然后2009年公布出来的，这是第二轮。第三轮呢是2012年，第四轮是2016年，我印象第五轮好像是去年年底，所以他有可能会算2020年进入这个（第五轮）学科评估，那么这些学科评估呢就跟有几个东西呢是有一定的这个绑定关系了。

第一个呢就是双一流，对吧？这个我们讲的叫一流大学、一流学科，对吧？这2017年提出来的搞双一流建设，那当然我们现在有时候说双一流，是不是就是原来的211、985的延续或者这样一个方法，因为现在大家已经慢慢淡化了211和985，211不评了，985也不延续了，对吧，那么现在大家强调的就是这个双一流，那么一流大学是整体的评价，然后一流学科是单学科的评价。那么2017年的首批评完，然后我看了一下那个数据里面是有137所高校和465个学科，而且里边儿还分什么a类、b类的，划分的就很细了，这是一个情况。

那么很快呢教育部又推出来了另外两个“一”，就是一流专业和一流课程。那么这个我自己的理解呢就是，大学学科里面的比较多考虑的还是研究，那就是至少在研究生或者是学术这样一个层面，对教学人才培养的成绩呢考虑的相对是比较少的，但是这次就是今年搞的第五轮学科评估，它整个体系里面增加了人才培养、教学课程等方面的一部分内容，那这样的话呢也是在突出这个大学里面人才培养的一个重点、这样的一个导向，那么很自然就带出来了前两天我们做的这个一流专业一流课程这方面的工作，那么我自己理解呢，他应该纳入到跟一流大学、一流学科整体再去规划、建设、培育等。因为本身我在管理学院嘛，我又在这个工商学科里，所以呢相关的情况呢前两年稍微关注过一下，它里边呢做这个一流专业和一流课程在这个未来的4~5年的时间里边要搞1万个一流专业、1万个一流课程，所以前面是双一流建设，后面是双万计划

刘书博：那评这些新的这种项目呀或者是title也是以评促建，是这个意图吧？

受访者：对，因为它是你申报，申报完了以后呢，评审了以后呢，是给你一个见识的资格，当然也给你一定投入，完了以后呢要验收的，就是说你要拿成果最后来交账的，大概是这么个意思，那么第一轮的一流专业和一流课程呢，前年已经做过一次了，2019年已经做过一轮了，全国已经有几千个专业、几千门课程。今年呢在做第二轮，结果还没出来，那么这两轮做完了以后呢可能大几千就有了，那剩下的名额可能就不是太多了，对于未来的这个方面的竞争就会比较强烈。

但是呢这个一流专业和一流课程呢有一个计算的基数，就是我们国家的大学里边什么什么学科的专业，这个布点数有多少，就是有多少需要开设这个专业，那么这样的话我们在一流专业的配比上你不能这个厚此薄彼。那么这样的话就在一流专业的配比上，相对来说呢基数大的专业，那可能给他的按差不多相同比例的这个数字给他的可以进到一流专业建设的队列里面的数量呢就多一点。我看了看教育部的这个规划里边，比方说工商管理，这就是算全国一流专业规划布点最多的专业之一。

刘书博：所以以评促建这个应该就是中国特色吧，国外他们是没有的。

受访者：是，最后我会讲到一点我们这个教育或者是大学体制上的这样一个问题，就是我们我上次也聊到这样一个话题，就最早的时候，我们比方说这个学科呀、专业呀、设立也好、审批也好等等，政府或者是教育主管部门在这里边所发挥的一个作用，因为我们现在还是公立大学为主体的这样的一个教育体制嘛，就是国家的这样一种体制，所以你不按照这个游戏规则去跟别人玩，你可能连露脸的机会都没有。我导师席酉民老师现在去做了西交利物浦大学的执行校长，那么国内这种中外合作大学，之前讲过宁波诺丁汉，包括这个西交利物浦，然后北师大在珠海港跟香港浸会大学合作办的一个学院，后来上海大学跟纽约大学合办等等，那么这里边呢他的基本的这个体制是校董会领导下的校长负责制，这个有点像我们现在讲的股份公司制度，或者叫现代企业制度里面董事会领导或者股东大会领导下的总经理负责制。但是呢中国的《高等教育法》都是这样，党委领导下的，对吧？然后呢特别是本科招生是国家考试，现在研究生的招生特别是初试，那也算是国家考试，复试也算是国家考试，就是说你要取得大学研究生的入学资格，得通过国家考试才能进，所以很早的时候大概2006、2007年，因为在席酉民老师之前，西交利物浦大学呢，还有几位前任的执行校长，那都是英方派来的，派来了以后呢就有一个很大的问题，就是说跟江苏省教育厅之间这个关系，有的时候就搞的比较紧，比方说每年招生的指标名额要到教育厅去拿，那给你几个专业、每个专业给你多少人，那说的不好听点，那就是别人高兴了，多给你点儿；不高兴了，少给你点儿。你是一点儿话都没有的，对不对。因为你过去没有基础，你也不是过去的比方说我们老牌儿的、重点大学，对吧？然后你也不是这个行业的省部共建的，或者什么样的一个大学，你的历史又这么短，对吧，完了以后呢，你说你的培养质量高，但质量高的很多学生后来都跑到国外去了，是吧？然后呢未来对中国做的这个贡献可能会怎么样我们也不知道。那么回过头来说，那就是说我多给你的名额，那等于是其他的学校可能得到的名额就会少一些，如果在总量一定的这个条件底下，对吧？所以呢席酉民老师去了苏州了以后呢，做了大部分的工作就是跟政府之间的关系，他的一种这个梳理、打通，包括让他们来接受中外合作大学这样的一种新的大学形态，新的一种校董会领导下的执行校长负责制，还有这个校长是名誉性的，就跟这个国家主席有点像，开学典礼来一下、毕业典礼来一下，一般是差不多是这样的一个形式。他们也有党委，但是他的这个党委呢跟我们现在公办大学的这个党委的地位或者是作用、权利，那就有很大的不同。所以就刚才你讲到的就是说，我们的政府，我们政府的教育主管部门，他所规划、他所提倡、他所倡导的要做的这样的一些工作，那么说的不好听一点，就是底下还是要乖乖的听话的

刘书博：所以这个自上而下的很清晰

受访者：对，你要不跟着指挥棒走，要么你可能就得不到应有的资源，你的发展等就会受到很大的限制。我举一个去年我们学校碰到的一个具体的例子，那当然其他学校跟我们一比的话就有反差的，就能知道大学校长的底气或者叫骨气是什么样的。这件事就是，我们每年的研究生毕业了以后，答辩之前我们学校里边有内审，然后他们从内容格式方面会做一些把关，然后还要送外审，我们硕士至少要送两个校外的评审，那博士至少要送五个匿名评审。学生的论文两份都合格，或者五份都合格，你才能够去答辩，答辩完了之后学术委员会答辩通过了，学术委员会大部分当然都会通过，都做完了学位就授予了。但是到了2021年，教委要从把2020年毕业的学生论文抽10%或者抽5%，各个学科里边随机抽，然后你把名单拿去，然后他随机的一画圈儿，谁被抽到了，抽到了以后了他们就拿出去了，拿出去干什么？又是匿名评审，学生也不署名，老师也不著名，致谢里反正有可能露马脚的地方通通都去掉，然后去找一些专家来评，评完了以后觉得还行，通过了就罢了，这是一份合格的论文，给他博士学位，没错。

多多少少都能查出几篇不合格的，我就觉得这篇论文，你说问题吧好像也不大，一般都不会说打个5分、8分、10分、20分的这种，但是了你说没问题吧，他也还是有些问题。像我们评的比较多的就是学硕的和专硕MBA的论文。我们送出去的将近有30篇论文，有5篇论文最后评定为不应该通过，换句话就是说论文质量问题，你们把关不够严，当然你不可能让他再重新去写。当然了，导师肯定也不会接受这种事情。

为什么我们国家的学位条例规定答辩委员会和学位委员会有最终决定权？你现在等于又增加了一个秋后算账的环节，除非是查到说有学术造假呀，或者是抄袭之类的，比如说工科做实验样本是假的，数据是假的，曲线是假的或者什么之类的，那么一般的都不会涉及到这样的一个颠覆性的问题。就算论文抽查评审时不合格，学校也没办法说那把学位撤回。

但是学校又咽不下这口气，假如哪篇论文被省教育厅、市教委批评了，那我们学校的校长就会被教育厅拉去约谈一次，就会说李院长你来一下，你们去年论文评审过程当中问题比较多，相当于就是要你抓工作。那你要是校长，你到那儿去你还能直着脖子，然后跟人家拍桌子？现在我们没有任何一个校长会当着这些官员的面理直气壮地跟他们去讲事实、摆道理，没办法阐明我们认为哪个专家提的哪些意见是对的，哪些地方专家的尺度把握是不准的，对吧？我们没有机会去争论这些。

那校长在外边儿受气了，回来院长、研究生院的院长、管理学院的院长过来一下，那你说我这一肚子气往哪儿撒，不是单独跟你们俩谈完了就完了，学校各种大大小小的会议上，反反复复的他就会提。当然去年不光是我们学院，还有其他几个学院里面还有一些问题。校长会不断提哪个学院有什么问题，哪个学院有几篇论文有问题等等。

因为我曾经有过一个学生，外审的专家给了个58分，就是不到60分，60分就过了，他就给到了58分。核心的问题在什么呢？就说这是一篇MBA的论文，但是他的选题不是工商企业。我学生在温州公安局工作，我怎么让他去写企业的问题呀？对吧？然后我就跟他聊，我说你们有什么工作可以跟工商管理密切联系的了？学生在110报警台，他们就要接警，有些问题报案，或者是不明白的，可能要给人家做一些回复、跟进等。我就跟他说，那你可以把理解成一种公共服务，但这种公共服务具有一定的产品性，在服务的过程当中，比如说有质量呀，有民众的满意度等这样一种评价，然后你能不能够借用《服务营销》里边的一些东西来去想怎么样去改善民众的满意度，从角度上来改善我们110报警服务、全流程、标准规范制度等。

实际上从一开始定下题目的时候，我们学院开题答辩，有内审，一直到正式答辩的时候，也还有老师提出来说这不是一篇工商管理的个题目。那我认为算，因为他用的这方法是从工商课程里借鉴去的。现在浙大MBA里面专门搞了个医疗管理，因为很早的时候美国有个学生写了一个叫以企业精神改造政府，我们不是说把政府变成企业，或者是我们讲的教育产业化，我们不是说把教育变成一个产业，但是教育也好、政府的运作也好，它里边有很多产业的特性或者跟企业特性相似的一些东西，那么很自然的它里边的一些工具或者方法，我们都可以运用。所以我们就觉得他论文工作量也还可以，写作也还是蛮规范的，但人家觉得选题就是不行，就给了58分。我说你们爱咋咋，反正只要你们不要把我学生的学位证儿要回来就行。但是他们也不可能要回来。然后你说我导师不合格，然后你取消我的什么资格都可以，但他们也不敢取消，反正这事儿就这么过去了。

刘书博：所以从这事儿也看出来，我们对于学术评价似乎不是很信任的这种感觉。比如说我熟悉英国，他们还是比较相信一个人的专业判断，不会像那些老师是秋后算账，或者再给你抽查一遍。

受访者：现在我估计向阳在北师大肯定也是经常受气，因为我们一批老师可能都比较抗拒。

赵向阳：我在北师大不受气，因为我压根儿就是游离在体制之外的，就是我又不参加MBA的面试，又不参加答辩，然后我高兴了带学生，我不高兴就不带。不过你刚才提出的问题，是挺有争议的问题，我觉得这是一个灰色地带。就是你哪怕用了工商管理的方法和理论来研究110报警平台或者说之类的，是有争议的。

我觉得包括浙大MBA做的医疗管理，我前不久在微信朋友圈里面讲了，因为我之前要去上海医药集团给他们做一个讲座嘛，然后他们就寄了一本《大生物医药大时代》书，我很感兴趣，我就把书就看完了，事实上验证了我的一个想法，我认为医疗管理真的应该从商学院那边儿搬出去，主要是因为现在的商学院太过于功利，如果把医药产业放在这里边儿，它会更加的商业化。

刚才讲的以企业精神改造政府，都是属于七八十年代兴起的新自由主义的意识形态下，用私营的东西来经营监狱等公共管理场所，引起的麻烦很大。所以我觉得医疗管理最好是放在公共管理，可能更合适。所以我那天发的朋友圈，立刻有一个国企的，一个属于国资委以前退下来的领导就特别特别支持，然后就给我发了一些东西。我觉得东西是可争论的，但是靠这种行政的检查来保证论文的质量，在中国大的背景，事实上就是因为我们弄虚作假太普遍了，单靠学术自律已经是做不了了，但是有的地方你本来可以依靠内部的力量，现在都不行，只能是靠外界的强制性的手段，所以我觉得这个事情很吊诡，是很难的一个事情。

刘书博：那能不能够这么说，就是我们其实也没有一个学术共同体的这种自我约束。

赵向阳：我们压根儿没有，我们商学院大家都是关起门来，然后都是一些学术个体户，然后其实像你说这种community所形成那种value，就是共同坚守的一些标准和底线。就是有一个词叫integrity，就是一个正直，我们国内比较缺乏。

像我举一个例子，我在德国读博士，你答辩完了以后是分等级的，等级是用拉丁文写的，我拿到的是最高等级的，然后完了以后还有一个宣誓仪式，是用德语写的，你要跟着朗读，就跟入党一样，大概意思就是说我现在是博士了，从此以后我只是为了人类的真理而服务，大概的意思就是这样。反正我是信以为真，我后来在50人论坛的群里面，我就说我信以为真，后来发现中国的学术界根本没把这当回事儿，你知道吗？就涉及到文化和制度方面，没有integrity的共同体的文化，这也是后来才有政府在不断的检查，或者说就是因为政府一直把它看作一个小孩儿一样，都不相信，也导致它自己成长不起来。

受访者：确实是就是这样。向阳老师，我记得你有一次在你朋友圈里边儿抱怨你们北师大的报销、审查等。

刘书博：对，这些东西实际上都是有一点我们叫行政权也好，官本位也好，他对我们正常的业务性活动都是一种干扰或者扭曲，因为它本身内在自由的规律或者机制被人为的外力作用在中间有很大的一个打破或者是干扰，就是说我已经不用对最后的东西来负责了，为什么？你各方面的指挥棒已经给我指挥的我都不知道北在哪儿，我能那耐下性子来帮你做已经是很好的一件事情了，就是那好像大概是到了这样的一种程度。

那这是我们刚才讲到的关于学科评估也好，或者是专业评估，专业认证等所引出来的一些问题。

受访者：另外我在准备材料时还考虑到了这么一个东西，我们先回到前面讲2000年前后。2000年，中国工程院单独设立了工程管理学部，最早他有几个工科的学部但没有管理学部的。那么后来了专门设立了工程管理学部。因为原来没有管理学院士，所以首届工程管理学部的那些院士们都是从其他工科的学部里边跨过来兼的，包括机械的呀，能源的呀，医药的呀或者是信息的呀，自动控制的等，后来慢慢开始有一批、两批、三批、四批，一些相对比较纯粹的属于管理学的院士以后，其中一些工科院士开始退出。

那么因为工程院、科学院里面形成了管理学部，工程院和科学院是中国最高的两个学术团体了吧，然后他们起到很重要的标志性作用，再一个那些院士作为学科权威、学科带头人等，在评审、立项，或者一些重大的咨询、参议性的一些工作上发挥了的一些作用。再一个大家去参评院士也具有很重要的一个标志性的导向性的意义，因为我们前面去评一流学科呀，一流大学呀或者什么专业，学院只要有院士在那儿坐着，那这个学科就不会弱。有院士没院士，有长江没长江，有诺奖没诺奖，那我们叫打分也好，或者是评级也好，就有很大的不同。

原来我们讲合肥工业大学的管理学科怎么着都排不到比较靠前的位置，后来杨善林以及他的团队带来他整个的学科，起到很重要的作用。

工程院里的工程管理学部底下还成立过一个教育委员会，还是教科方面的委员会？我没有去跟踪，有机会你可以跟清华陈劲聊聊，因为他原来跟徐经瑞院士他们做的关于技术创新的方向，实验室增选院士了以后，后来承担了中科院的一些工作，其中有一部分是跟教科结合的方向。

在2000年以后，教育部除了高教司、科技司等机构，它底下还有一些委员会，教学方面讲的比较多的就是教学指导委员会，科技司有一套体系叫科技技术委员会，科技委里边也分了很多的学部，其中也包括管理学部。当然因为科技师对口的还是研究，所以跟自然基金委有一定联系，但是因为不牵扯到拨款、立项等东西，所以科技委更多的还是一种咨询的作用。主要就是给教育部或者是国务院的科教领导小组，即分管科教的那些副总理、国务委员提一些政策性的建议。所以教育部科技司在科技委的建设过程当中，后来联系了几所有一定基础的学校设立了几个战略研究基地，清华薛兰老师他们那边有一个基地就是比较早的，然后上海交大高教所刘剑才老师他们那儿也有一个基地，现在软科的排名的，就是他们那边在做的。

然后我当时在西交大的时候，我们有一个中国管理问题研究中心，原来有一个老书记，他当时是在科技委的管理科学部里边的，所以他那边有一个科教结合的研究中心，它不归属于高教所，也不归属于公共管理学院，也不归属于管理学院，它是一个相对独立的，因为他有书记在那儿，后来陈劲接了做主任。后来当选院士了以后参与了一些工程院，它里边的一些关于科教结合方面的一些研究工作，陈劲他们直接间接参加的、承担的一些任务还是比较多的。所以你要有兴趣或者有机会的时候了，可以跟陈劲直接了解了解。因为我后面就没怎么关注了，他后面出了一些什么样的一些成果、文件、重大的决策，有没有什么样的影响，这块儿我都没有特别的关注。我觉得还是有一定的比较高层次、重要标志性的作用，因为他们能评上院士，对吧？

另外，上次向阳老师曾经讲过，我们国内有两所比较特别的商学院，一个是长江，一个是中欧。我前两天了又把这两个学校的东西稍微翻了翻，长江是2002年设立的，东欧比较早，1984就设立了，当时的基地在北京，1994年迁到上海，然后跟上海交大有合办的渊源。

从商学从商教育的这角度上来说，因为他们不做本科，从MBA起步，那么从MBA起步之后他们实际上招生了都招了漫长一段时间，他们的证书最一开始教育部是不认可的，中欧的MBA到了2002年才得到认可，可以算中国的硕士证书。

长江最开始也是这样，我印象中到了2005年还是2006年，也才开始认可他。因为这两所学校跟其他的所有的商学院的都不太一样，他没有本科，然后采取的是这种应用型的、相对高端一点的培养职业经理人才的方式，所以是比较特殊类型的商学院，国家开了这样的一个口子。

而且这两所学校的建校基础还不太一样，中欧的有点官方合作，而长江还是民营的，李嘉诚长江集团去做。你们可以去看它里边产生了哪些效应。可能对于我们商学的发展它是有正面的积极的作用的，还有哪些方面甚至可能会产生一些负面的消极的影响，都可以去讨论。

刘书博：好的，中欧这边我比较熟，我2008年～2014年都在那边，他们是比较商业化、市场化的那种，我觉得像培训机构。

受访者：还有一块儿我没有特别去跟踪过，但是应该在我身边还是有一些关系，就是关于高职高专的商科类，因为高职高专他偏重应用型的。那么讲到商科的教育，其中我们讲精英人才，这实际上也只是一方面，还是有很多的学生出去了以后去做一些事务性的、程序性的、基础性的工作。向阳老师也提出来说现在学生都不愿意进工厂了，不愿意把手弄脏了，不想做一些要出力、流汗的工作。很多专业细分之后，好像觉得是不是工科专业的学生也在工厂都是正常的？他们要去实习。我们商科见习应该是去坐办公室，商务街之类的，打个领带，穿上西装之类的。

在大学扩招过程中，高职高专也是其中很大的一块儿。大学扩招的时候，那时候建了一批国立学院，北京很多学校也有下属叫独立学院，这两年独立学院又要改制嘛，今年我们浙江有几个独立学院，包括我们学校的独立学院都遇到了这样的一个问题。有的学院想变成公办，跟原来主办的院校脱钩，或者逐步脱钩，还有一部分学院可能会变成民办，有的已经明确了。然后有些学院在民办的过程当中就遇到阻力了，学生不愿意啦，老师不愿意啦，然后弄的家长也不愿意啦，然后就成了社会的不安定因素。

我们学校这事儿刚好碰到七一建党一百周年，那肯定是你总不能冲百年大庆的喜事儿，所以就先搁下了。但是这事儿你回避不了，他每年还招那么多人，还有很多毕业生，还有专升本甚至还有升学读研等这样的一些情况。现在从大的定位上来说，其中有条件的以后真的可能就会转成本科吧。

还有一些就是应用型大学或者我们要培养职业性人才，那么这一类我之前没有特别的去做过一些思考或者是研究，所以说你可能也要考虑要不要把它们纳入到整体的一个跟踪中。

刘书博：我看群里赵向阳老师发了两本书，一个是《西交利物浦》，一个是《中欧神话》，第二本我看过，然后他主要讲了中欧做市场项目公司特训是怎么样搞起来的，然后第一本我回头再找来看一下，是邢国明老师写的。

受访者：最后有一个话题，我前面也提到过，就是说我们是就商学院讲管理学科的发展还是也包括其他类型的管理学科？就管理学科来讲可能会有一些困难或者不足，因为它是包含在大学里边的，那它不可能脱离大学的环境去谈。我们国内的大学除了体制上的问题，还有类型上的划分，比方说师范类的学校、财经类的学校、理工类的学校、综合类的学校等，那么它里边的条件也不太一样。

我不知道向阳老师去北师大经济管理学院的时候，这几年有没有碰到其他院系学科的瞧不起或者排挤等这样子，因为我一直是在理工科的学校就会存在这样的情况。

赵向阳：我自己对东西完全无感，你知道吗？你让我就体质讲，我三句话就能讲完，我压根就没感觉，我完全就是躲进小楼成一统那种。

受访者：所以这边还牵扯出来一个大学管理的体制、法制的问题。然后到了院系这一层，到了学科这一层我们还是用院系这样一种体制来做的。那么到了院系的这一层又是什么样一种体制，甚至包括一些关键的院长，校长。西交大从来没有一任校长是非工科出身的，对吧？我来的浙江工业大学其中有一任校长，那有一任校长叫吴添祖，他原来是搞技术经济出身的，是从我们学院出去了。也不是说他会有本位主义的思想，而是说是他本身的一种价值观也好，或者是一种知识体系也好，他看问题的视角或者是方法也好，有的时候可能跟工科的那种思维还是会有一些不同吧。所以像这些东西可能都会对某学科的发展产生影响。

我们现在是拿商科为例，来去做相应的研究。所以刚才我前面讲到的教育部科技委，我们当初搞了几个战略研究基地，那会有一些硬性工作任务的。就是他每年会给我们一点儿钱，10万块钱左右，然后每年给我们报几个题目，然后每半年左右是不是能出一个专家证书，或者是一个专家咨询的一个报告，然后他们把它整理以后作为一个内部的文件上报给教育部的国务院的科技领导小组，这里边主要的成员包括我刚才讲的副总理，国务委员级别的这些人，他们讨论过很多问题，比方说大学治理的问题，大学排名的问题。那上海交大他们本身就搞排名嘛，然后包括科教结合的问题、学科规划的问题，那当然还有些是偏宏观一点，有些可能偏排名这类技术性的操作问题。像这些东西实际上对学科影响还是挺大的。

因为现在的校长也好、院长也好，他都是有任期的，那任期最后可能要看能不能升一升，这些东西都要看你任期里边能拿出什么东西，所以刚才我们讲到的就是自上而下的指挥棒，为什么有这么大的作用，那就是说我们吃这一套。比方说我们执行的是董事会领导下的校长责任制，然后如果我们不靠财政，而是像国外大学自筹经费，靠捐助等这样的一种方式，那么这样的话你自主的能力就会强很多。反过来那在相应位置上的人，他要承担的责任或者压力也会大很多。所以说是对上负责还是对下负责，方向不同结果可能也有很大的不同。短期之内我们按照依法治国的这样的一个理念继续办我们的教育、办我们商学教育的话，大的格局其实也是不会变的，因为高层架构，比方说党委领导下的校长负责制是没有变的。

那么这样的话，我们做的很多工作比较多的就是零敲碎打性质的，我个人认为不会在短期之内有一个特别大的突破。

刘书博：我这边还有个问题，您在这个领域已经工作很长时间，那您经历过的有没有一些学校或学院转变的很快呀，就急转急停的。因为我也是听说是不是西安交大经管学院之前也是在做一些非常激进的改革，比如像咱们现在看到有些“非升即走”的负面新闻一样，它完全是量化考核，完全是这种丛林法则这样的一些改革，您有没有经历过类似这种的商学院的变化。

受访者：那段大家反映比较强烈的时候，我刚好在西交大。在我离开之前，我们有三个院长，最早的就是前面我们介绍过的汪应洛院士，后来因为在北京做学术报告的时候突发脑溢血，然后我的导师席酉民接院长，到了1997年、1998年做副校长了，那么学校就说你要把工作的重心要放到学校，然后就让当时的副院长李垣做院长，从2000年一直到2010年。大概就是这十年左右的时间，比较关键的是中间2004～2005年前后那一段时间，我是2007年离开的，那时实际上很多学校里边尝试着做一些内部管理方面的改革。我印象最深的实际上是上海交大，上海交大在80年代、90年代初的时候，他们的书记邓旭初，他当时主持上海交大做了很多内部管理体制方面的改革，当然大的框架可能还是原来老的国立大学的体制，但是它里边搞了一些具体的做法，搞活了一些东西，包括后来像华中科大、武汉大学等他们都做过一些。

那么西交大是怎么做的呢？一个就是我们管理学院原来算是恢复设立比较早的，1984年我们就算恢复设立了，然后我们的几个学科发展都比较快，特别是每个学科里边都找到了几个比较有成就的学者，然后我们配置的方向梯队做的也还不错，包括像管理科学工程我们讲的关于企业理论方向做的都还不错。反正那段时期，然后同行大家一起交流的时候都提到了领域提到方向，好像都会介绍到西交大的谁谁谁，那么这样的话在外部环境里边我们还是比较有利的，比方说原来王路老师是国务院举办的学科评议组的召集人，比方说国家科基金委成立管理科学组或者是管理科学部的时候，那么他也是其中的成员。这些指导委员会，不管是国内科学工程的或者是工商管理的，我们有几个老师在里面要么是主任委员，要么是负责的组员，所以就是说国内管理学科几个标志性的人物，标志性的岗位，都在我们学院，这比较多的是对外了。那么对内呢，西交大原来有些工科专业是比较强的，那也有几个比较老的院士也好，或者是最初的有几个长江也好，像最早的管理学部一开始设优秀青年基金，然后后来设协助青年基金，这样我们有一定的条件能够争取更好的发展，但是因为我们跟工科还是不太一样，所以学校里边所有的政策一定都要一碗水端平，要把它弄齐，我们觉得我们跟工科，包括其他的文科，特别是2000年以后，我们不是合并了财经学院和西安医科大学嘛，那么这样的话就有很多东西就没办法大家完全一致，大领导们也觉得就是说是不是我们应该分类施策来做这样的一个工作。

所以大概在2004年的时候，我们学院里面就提出说我们是不是能够做个校内体制改革的试点，然后把其中的一部分权力下放给我们，比如说人员的聘任、岗位的设置等。那时候除了国家政府、学校给的相对比较固定的资金以外，我们那时候也已经有一定的财力了，我们就觉得可以给老师一些更好的激励。激励的话不用学校掏钱，但是我们需要有一套具体的办法，办法需要得到学校的认可。后来学校说光管理学院试点好像也不行，就又选了一个能动学院，反正就是选了三个学院来做一个试点。那么其他学院的力度没有我们学院力度那么大，所以他们的反应也不是那么强烈。因为我们那时候岗位聘任基本上就是业绩说话，或者我们今天讲的话就是要数豆子、数工分的，比如你有什么项目？你有什么文章？你有什么获奖？基本上主要是这几项。那时候还不牵扯到发高索引之类的，但是就已经有了很大的触动。

因为其中我们很大一批老师是教学型老师，特别是我们合并了陕西财经学院里边的原来商科的一些老师，比如说财务管理、市场营销方向的老师。曾经有一段时间单设了一个会计学院，就是说那会儿我们除了经济学院、管理学院单独还有一个会计学院，学校给会计学院的一个条件，就是当时国务院准备在全国找几所叫国家会计学院嘛，就是上海有一所，厦门有几所。因为上海会计学院我是去过的，然后它老早是人民银行总行的下属学校，所以我们就想通过这样一个关系跟人民银行跟财政部搭上关系，然后做一些工作，看看能不能在西安建一所在会计学院，最后就是说他们自己独立去做，但是学科划分上来说就不顺了，因为会计学、财务管理这都是属于管理学门类的，所以说按学科门类就把他们划归我们院里边。但是他们很多老师都是教学型老师，然后我们开始数基金项目啦，要数文章了，要数获奖什么之类的，但是获奖比较多的是科研的选项，那大家就有不同的意见。反正我们做方案的时候尽量兼顾，然后让不同的老师有一定的过渡期，所以把这事儿还是推下去了，学校也批了我们的方案。

但是推归推，老师们还是不满，大家还会借各种各样的机会或者是用不同的方式表达，有的可能就是发发牢骚，那时候网络还不如现在这么发达嘛，有时候可能有些工作的安排大家也不太配合，比较突出的就是干脆走人，我觉得这儿不舒服了，我走可以吧？所以很多老师去上海了、去北京去其他的一些兄弟院校了，但数量整体还是比较有限。但是由于关注这事儿了的人还是挺多的，因为总是有一些会议之类的，然后听到一些风声，大家议论很多，所以外边了还是有一些负面的些声音。

这事儿到什么时候产生的影响就比较大了呢？因为有几个标志性的人离开了。第一个就是秦老师去了福州，他应该是在2008年完全不做副校长，任期到了，做了两届了，他去了西交利物浦大学做校长。再一个就是王路老师，年龄相对比较大了，所以他出去活动也比较少，我在的时候他每年还会出差，后来我离开了以后他露面就越来越少。再一个我们有个副院长叫王康良的老师去了人民大学商学院，还有一个当时副院长叫孙林老师，他是得癌症后来去世了。我是2007年离开，所以一下子走了好几个中坚力量。

学院曾经做过两个方面的工作，一个是委派学院党委书记，我在的时候就有党委书记了，后来由于个人的一些事情，后来换了，换了以后临时派来一个部长做副书记。那么过渡了一下以后又派了一个我们原来的一个教授做了书记，那么后来也是有一些个人和家庭的问题，但后来没有到任期，又把他换掉，又重新派了一个书记，这中间的档口，行政都有比较大的变动，所以班子就不是很稳定。

曾经中间还考虑过一次说面向海外招聘海外的院长，有两个人选，又一个接受了西交大的聘任以后又接受了上海另外一所大学院长的邀请，所以这样的话他同时接了两所国内大学的管理学院的院长。那后来西交大管理学院觉得不合适就算了。还有一个人选从西交大出去到国外去读学位，然后后来他从新加坡去了澳大利亚，从澳大利亚去了美国，所以我们把他从美国又重新请回来做院长，他是做大数据方面的一些研究，所以回来做了一段时间，相对还算比较长一点，那当然我们也给了他一个比较好的条件，然后他爱人回来，一起给他安排的待遇，后来还是因为一些原因没有续聘。人事不稳定带来了一系列的问题，包括学科建设的规划连续性、学校配套的一些支持，中间了就会有一些波动。

其中一个就是外部有比较多的诱惑，包括比方说我是因为老家在这边，我就离开了，那么有的是因为上海交大请别人去，给他安排院长的这样的一个职位，那西交大和上海交大给的条件那肯定是不能同日而语的。我自己认为过去几年时间内，西交大发展当中小的波折也好，或者是下滑也好，但是他们反正还是有一些条件的吧，他原来的学科基础还是有的。另外，综合的环境条件可能还是不如沿海的，像北京，上海，广州，深圳，所以西交大能保持到现在的这样的一个水平，实际上还是挺不容易的。

我去年回去了以后也能够感受到它们的难处。因为西交大又搞了一个新校区叫创新港，那搞一些研究院等就有了一些基础的空间。但是人文类、经管类这样一些学科根本没办法去跟那些硬学科pk，硬学科添了很多设备，所以我觉得他们也是挺难的。

我们不叫受排挤吧，就是说艰苦还是艰苦的，艰苦是相对的，条件比原来老校区更好啦，但是跟其他的兄弟院校比还是存在劣势。

刘书博：梁老师，您觉得现在年轻一代博士以及青年研究人员，他们的生存环境是不是压力要更大一点？

受访者：相比于之前那些年，肯定是现在压力大。这些年不是都在讨论内卷之类的，因为比较多的东西要去评、要去比，比方说入门的时候大家起步都差不多，最多个别人是某位学者的小孩儿，但这都是极个别，大部分可能就是什么都没有的，所以他们几乎要从零起步。

因为现在大部分的学校里边了开始在减少近亲繁殖，那么这样的话原有的学术团队、合作可能就要重新建立，原来可能真的是导师可以带着，现在你可能要自己去投靠其他的一些团队，慢慢找到自己的，中间肯定有一段时间是比较艰苦的。

另外年轻的博士，不管是男的、女的，也会碰到成家的问题呀、孩子的问题呀，或者是孩子入学、入托、择校之类的问题，各个学校的做法还是有一些不同，但是都差不多。那么有些学院这一级的有资源的、有条件话，就是有关系门路或者是有一点钱的，那么他们可能会采取一些辅助性的支持，这些东西比早些年了肯定是只好不差。那什么叫只好不差呢？比方说有启动资金呀，我们现在很多学校里面他们的一些研究团队有一定的开放性，他们也欢迎年轻人加入，这样的话如果你自己不排斥，而且能够比较好的接受或者是融入的话，还是能够在比较短的时间里面去成就自己的事业，或者是起步的。

关键是什么呢？就是说可能还是要耐得下性子，耐得住气，就是你不能去跟别人比，不管是师门的或者是其他的学校，因为你越比越没信心。

刘书博：好，梁老师，我看今天咱们谈了挺长时间，特别感谢您提供这么多宝贵的信息，我们后续再去找一些相关的材料学习阅读。后续可能还有一些补充问题，那我们能不能把问题文字发给您，您看用语音回答一下也可以。

受访者：你梳理梳理，然后看看哪个地方有遗漏，我知道了我可以告诉你们，然后我知道到哪个方向，比如去找什么人就跟其他的人引荐引荐。

# 受访者27

刘书博：管老师这边我们大概是有一些主题，那个我们这个研究其实是想看一下，因为之前也在网上看到您写的这个中国、美国、英国的这种商学院的研究模式，这个教育模式，所以其实我们这个研究现阶段也是一个探索阶段，就看一下中国现阶段商学院，包括管理学，我们是有什么样的特点，尤其是前一段时间，有一些新闻在热议，就比如说飞船疾走呀，或者是这个长聘制呀，它产生一些副作用，那我就想要了解一下，在目前对于这个青年研究者来说，他们在这个身份构建呀在学术工作的意义上，他们有一些什么样的特点和认识，所以现阶段还是比较这个初步的摸索的阶段，那我们同时也在了解就是历史性的一些素材，包括前一段时间跟受访者老师聊中国的管理学教育从改革开放之前一直到现在经历了什么样的宏观环境变化，然后现在我们的这个商学院组织又有什么样制度性的特点？那现在呢就是想要了解一下，尤其是作为您这个在这个中国英国目前又回到咱们中国，我听说是在深圳那边，然后经历了不同的这个商学院组织，所以想要了解一下，您的视角来看，这种显著的差异是什么，因为我个人也是在英国那边读的书，我是在爱丁堡大学那边念的博士，所以我我觉得咱们还是有一些共同的这个认识和话题，所以我就想要请教一下您，就是首先一个问题就是您现在为什么从英国那边又回到了中国，这种选择背后的动机，或者是您的想法是什么。

受访者：其实你刚才讲的这个大的背景，就是包括我们现在看到的，我觉得我们现在看到的这个现象层面会有一些会有一些极端化，就是一方面，我们会看到中国的大学的排名其实升的是很快的，如果你去看，不管是qs还是什么泰晤士，还是其他的，甚至我们经常讲就是北大清华都进全球化前20了，就说在宏观层面，学校层面就是一种高歌猛进的这种态势。如果具体到教师这个层面，我会发现教师这个层面就会有一些个体呢，他在这个过程当中呢也是有他的价值的呈现，同时呢也得到了相应的回报，但是会有一批教师，尤其是年轻教师，我们会感觉就是比较内卷的这样一个状态，所以我特别同意你说的，就是这个问题的分析呢可能是需要几个层面的研究，几个层面的这样的一个视角整合起来，因为呢你的不同的视角他的诉求是不一样的，就是从大学的角度来讲，那可能是出于一个提高排名的诉求，国际影响力的诉求，但是从老师的角度来讲，可能更多的是职业生涯发展、工作生活平衡，这里面有可能会在转型期产生一定的冲突。

当然就我个人而言的话呢，我觉得回国这样的一个决定，首先我一直是有一个很强的动力去教到中国的学生的，当然每个人在这个方面的看法不一样，有的人觉得在国外教到优秀的学生也是很有价值感的，我还是会觉得希望能够教到更多中国的有潜力的学生，因为另外我也有看到我们现在确实不管是在教育还是在研究上确实有可以发挥价值的空间，这是一个我觉得可以被认为是职业生涯当中的一个很重要的目标吧，就是说这个即使在英国工作，我也愿意跟国内的很多同行、学生交流。

当然现在呢有另外一个因素，就是一个职业生涯发展阶段，就说我觉得我现在比较有优势的一点就是，我回国的话我是可以在比如说评职称或者谈待遇这些方面是有一定的选择的空间的，而一般的青年教师回国可能还要面临职称的压力呀、或者跟学校去谈什么样的合同，而我因为已经有所积累了嘛。

然后我还是挺在意有没有一个比较合理的回报和比较合适的工作环境，所以当时的一个基本的情况就是我会去想，那我回国我基本上达到什么样的一个基本要求或者条件我才愿意回去，而且回去了能够比较好地去工作。而不是说，我回了国了还得想办法从额外的渠道去赚钱，甚至说去捞一些钱，我觉得这个就不是我回国的一个初衷了。

所以我会觉得就是我现在入职的学校确实能在很多基本的方面上满足我这样的一些基本的诉求，这里面比较有意思的一点呢就是说，我会感觉现在国内如果我笼统的区分的话，我们会讲有体制内的985高校和体制外的高校，其实像港中深，它属于体制外高校，它的类型跟莫丁汉、西交利物浦等是一个类型叫中外合办大学，相对来讲他们其实从国家层面拿到资源会少一点，所以说主要是通过市场，就是说他学费很高，一年本科生学费就九万多呢，研究生也是有学费的，当然你也可以申请奖学金，但奖学金也不是说有那么大比例，所以说我们是学费很高，同时对老师的要求、对老师的待遇也挺高的，是这样的一个地方。这样的一个好处就是，老师来了之后他按照一个市场行情market value直接给你一个相应的薪酬和待遇。

但是体制内的高校，就是说如果我回人大或者类似的高校，他可能取决于你有没有拿到一些国家的人才计划，这个就很重要。因为我确实都是了解的很深入、很细致的，也确实不止一个学校会愿意希望我能够回去，所以我会发现有一个很鲜明的对比，就是相对来讲，体制内的高校，他会希望你能够拿到人才计划，然后给学校先带来资源，因为人才计划本身就是特别重要的资源，不管是在双一流的排名，还是很多其他的这种评比当中，就是你帮助学校处于一个比较有优势的位置上，如果你拿到了就是很多待遇，就一步到位了，就是会有非常好的待遇。但是如果你拿不到的话呢，这也是让我感到别扭的一个地方，就是如果你拿不到，他并没有一个特别好的一个标准去评判应该给你什么待遇，所以经常就是给低了。可能好一点的就说那我给你解决职称，但是待遇上就没有什么了，或者更严格一点的高校，他甚至就说只是说给你解决了长聘职位的问题，甚至说是不是正教授可能都要再重新去讨论，这个会让我觉得是有待提升的一种做法吧。因为我们高校招人的话，你应该是有自己的一套独立的学术标准，那这个人能在你这个大学里当正教授副教授或者什么样的职位，应该是有这个判断，你应该是按照这个判断给他相应的职位和待遇，那至于拿不拿得到国家的人才计划，那是另外一个独立的事件，拿到了更好，拿不到也不应该因此而在某种程度上降低待遇，在我看来。

我其实是有申请过像这样的一些人才计划，但真的很难，我起码到目前为止我还没有拿到，后来我就反过来问自己，我说如果是最差的场景，如果我去一个地方，这些都拿不到，但是它还能让我平心静气的工作，有一个不错的平台，我后来发现可能还真的就是这种体制外的大学反而做到了这一点。就是他就直接就是按照我们国际上通用的请外省专家去评定你的学术履历，如果能够对得上他学校的正教授，并且拿到终身教职tenure，他就直接给你了。另外现在南方很多城市他其实也有地方上的一些支持嘛，所以我觉得加起来就已经是挺好的一个工作环境了。所以我后来选择了来深圳，主要就是这个原因，就香港中文大学深圳的这个校区。

其实每年的学校我是有很强的认同感的，就不管是说我以前学习过的学校还是工作过的学校，我的关系一直保持的非常好，他们也很有动力说，那你回来应该就是可以很好的融入，我确实有考虑过一些可能性。但是后来我经常听到的一点就是现在我们很多国内的高校确实是进步的很快，但同时呢它对老师的要求也会非常高，而且这种要求是带有一点外部性和动态性的，所谓的外部性就是说它会取决于你拿不拿得到外部的资源，然后再给你这个待遇；另外所谓的动态性就是我经常讲“坐地起价”，就说你来的时候可能是跟你说了一个标准，你在这里面开始工作了，这个标准就开始突然提升了，或者最后让老师相互之间pk，虽然大家都达到要求了，但是我只能留一个，我觉得这就会使一个还不错的老师可不会得到太合适的激励，甚至使得这个职业的生态就会产生一些负面的一些变化。

当然你如果看这个整体上，如果你上升一个层面去看的话，那大学的排名那确实是在提升，因为他是在用这种淘汰制不断增强他的实力嘛，但是如果我们是想建构一个可持续发展的学术生态，我会觉得可能将来这些方面也会有所调整吧，包括你刚才提到的这个“非升即走”它造成的负面的后果。我会觉得“非升即走”本身并没有错，是你怎么去用它的问题，因为“非升即走”，它其实就是在一个规定的时间之内看看你的研究成果嘛，我觉得整体上他还是有助于说去留下一些真正有竞争力的这样的一些师资力量的，并且呢有利于接触到比较高的国际水准上，我觉得在国内“非升即走”最大的问题就是刚才说的那一点，它并不是一个客观的标准，说你达到了你就可以留下，他很多时候是说这个标准在这里，然后大部分人达到了再从这些人里面再挑几个留下来，就采取一种限制名额，最终把它变成了一种零和游戏这样的一种做法，那大家肯定是工作起来没有心理安全感。

另外在这个过程当中如果前后不一致可能会导致严重的不公平感，所以这可能就会导致一些这种比较负面后果，尤其是在个体层面的这种负面的后果。所以我会觉得这个其实也是需要我们去重视的。

刘书博：好，谢谢管老师，那就还是回到这个比如说咱们做学者，学者现在中国这一块儿其实大学层面发展的比较快，那也提到了就是对于学者个人来说压力会比较大。那您会认为学术贡献主要是以什么样的一个方式来呈现？就是我们在评判学者的一个水平能力的时候，是不是还是按照论文为主，或者您刚才也提到说这个可以培养最好的学生，培养这个中国最好的学生，所以您对于这个职业的这个追求、它的意义感，这个能不能再更多的跟我们分享一下。

受访者：我觉得学术贡献呀，那首先这确实是一个见仁见智的事情，我们经常说“文无第一武无第二”嘛，就说如果你是跟人打架，那肯定能打出个输赢来，但是你要说像我们社会科学、人文科学这个领域的贡献，它确实带有一定的多元性，评定的时候也会带有一定的主观性。

我会觉得，如果是从管理和评定的角度，那不外乎就是，如果是特别北美这种风格的这种商学院，那基本上就是最主要的其实还是看顶级期刊论文发表嘛，这是他们评判的一个标准，这也是为什么你如果去看北美风格的这种商学院的老师，你看他们的简历，那基本上就是清一色的就是在国际最高端的一些期刊上有他的代表作，而且他们的风格一般就是发文章也没有那么多，就是有些人你看正教授也就十几篇文章，但是每篇拿出来可能都让你觉得至少这个期刊是有分量的，我觉得这就是美式的一种导向，但是它影响了很多地方，包括新加坡、中国香港，现在国内很多大学也在追捧这样的体系，我觉得它最大的一个优势呢就是，美式的这样的一些顶级期刊，它确实强调方法的严谨性，就是说包括他对中国的整个的管理学研究的影响，其实在方法上的影响我觉得是非常非常大的，就大家普遍现在开始去考虑说这个所谓的同源偏差呀，比如说这个什么跨层的问题，其实这个确实是给予了中国的管理学研究很大的一个严谨性的层面的一个提升。当然在所谓的创新性层面呢，这个就有点见仁见智了，因为所谓的创新性所谓的theoretical contribution，虽然所有的顶级期刊都在讲，但是我一直觉得这个所谓的理论贡献是一个被夸大的词汇，其实我们有时候做的研究，当然我自己顶级期刊论文发的少，可能我讲起来有我的个人偏差，但是我会觉得很多时候我们所做的贡献仅仅是一个在知识上的贡献，就说现有的文献研究到这个程度增加了一点新的知识，我有的时候很难去讲说这真的算是一个理论上的贡献，甚至你如果去说管理学当中真正的理论上的这种我们说特别有贡献的人，可能也没有那么多，在我们看来，但是我会觉得现在的就说美式的顶级期刊他会逼着你去写这个东西，因为他们的定位就是要有这种（理论贡献），所以我会觉得会造成一些这种现在我们觉得不是特别好的风气，就是带有一定功利性倾向，就是你去设计一个研究，可能大家会有一个共识，就是你这个数据越难，可能就发表的概率会高一点。我我觉得这个可能是他们也需要去考虑的一个地方。就是说将来你作为一个世界领先的这种期刊，你将来把大家导向到哪里去，我觉得他们应该也是意识到这样的一个问题，所以说他们现在不仅强调要有趣，要严谨，也会强调有他的这种价值嘛，就说它的重要性也是需要提升的。

所以说我会觉得如果我们是从一个评价体系上去讲的话，那肯定是说中国现在是在吸取很多其他国家的经验嘛，可能每个国家都有他自己的一些比较有优势的经验，我觉得美式的经验就是大家都追求卓越，并且去追求这种方法上的，或者说在实证层面的一些进步，这个其实是我们应该学习和保留的。但是呢它这个里面如果是过于极端化，可能是他的问题。

那相对来讲呢，包括像你也在英国待过，其实英国的评价体系它就相对更宽容一点，也更多元一点，英国的评价体系它是属于那种尤其对于年轻老师来讲，英国的评价体系它是基本的策略就是说，你要保住你的工作是容易的，就说他有probation嘛，就是试用期你过了，基本上就是permanent contract，那两三年基本上你正常是可以拿到一个永久的合同的，但是后面你升职的话，他会考察你的东西比较多，它包括研究，也包括你的教学带学生，包括研究基金申请，包括甚至说你不仅是要论文达到要求，你还要有影响力的证明，包括说社会服务的证明、你的领导力等等，他会考察的东西比较多元。

而且我觉得英国的评价体系跟美国的不太一样的地方就是，英国的评价体系是说你不能出现短板，就是他考察的方面很多：研究教学服务领导力。英国有个词叫well-rounded，所谓的well-rounded就是你很全面,就是有点像木桶理论，就说你这些都得达到了，这样才能给你评。而美国呢他就是说突出优势，尤其是研究上的优势，你甚至说可能教课平平无奇，社会服务你也不太感兴趣，但是你就是能发顶级期刊文章，在美国是很容易的，就说就够了，够了就直接给你评，甚至你在这个market上会很受追捧。英国可能就不太一样，就是英国的导向，传统的导向就是你的各个方面都表现出来，这个他才会给你。

但是这几年呢，因为我觉得全世界受美国的影响都会大一点，其实英国现在特别领先的这种大学，你像牛津、剑桥这种，他其实也在追逐美式的体系，就说老师凭这个tenure其实越来越像美国人这种评法，所以他们也会感受到这种压力。因为英国的这种体系，他是希望说大家环境都有一个宽松的环境，不着急马上发文章，你可以慢慢的去想一些大问题，所以说一个宽松的环境嘛，一方面是给有一些天才的人物、一些精英式的人物给了他们这种成长空间嘛，包括英国历史上也有很多大科学家，就是说包括说像牛顿等等很多瓦特也有一些大的社会科学领域的这种研究者，包括说亚当斯密，很多时候你去看他们成长的一个环境和他们当时做研究的，他们很多时候真的是有很强的内在动机，然后家境一般也都很不错，就是说他们也不追求说我要马上发论文、凭tenure，他们就是说我就是有兴趣，也有这个环境，我就慢慢的去做、去写，我觉得英国是想保留这样的一种传统，所以他创造一个宽松环境，让老师慢慢去做，但是这个环境也会养一些比较偷懒的人，这个也是一个事实，就例如说我本来水平也不咋地，正好反正学校要求就这么也不是那么难嘛，那我在英国立足也是ok的，所以说整体上呢有可能对他的排名就不太有利嘛，因为他不是说所有老师都有那么强的一个产出的。至少因为我们现在这个大学排名也是在数很多指标嘛，可能在指标上就不太有利。所以说有一些这种比较最后排名的英国第一梯队的大学，他们可能也会适当的说，希望老师还是按照美式的标准去发表他的研究成果，但是我觉得英国传统的他这种评价体系还是有它的一些好的地方的，尤其是你如果对接中国的这样一个环境，其实我觉得中国真正开始国际化也没有很多年的时间，我记得我当时十几年之前找工作的时候，甚至说你有篇的SSCI的文章发表，可能都是比较有竞争力的，都不看你的什么级别abc，但是现在大家都开始追逐什么达拉斯之类的，所以中国变化很快，但在这个很快的变化当中可能要保持某种均衡性，我觉得中国的这个商学院改革的一个最大的问题就是有时候矫枉过正，就是现在我们导向是追逐这个那大家就全力追逐这个，甚至把一些有价值的已有的东西给抛弃了，我觉得十几年之前大家就是在全力追逐西式的SSCI，但是最近几年呢又开始回调，因为大家会发现首先CCSI也有369等，就是有些甚至就是很垃圾的期刊，我们毫不客气的可以这样讲，另外即使顶级期刊也不是说他总是有那么强的一个思想性，有的时候这个方法性反而超过了思想性。另外一个呢他很多时候不太关心中国发生的问题，所以说现在的导向就是说中文的发表应该要受到重视，我觉得这种适当的回调是有帮助的，但是我特别反对就是你在回调的过程当中又把已积累好的一些东西又给扔掉了，因为我会听到一些比较极端的声音，就是我们可以将来就完全不发表它了，就是只看中国的，但是我觉得中国的期刊根本没有那么大的一个力量可以承载这么多的人想去发表研究成果的这种需求，另外水平也没有真正达到别人那么高的一个水平，我觉得是不太好的，又回到另外一个极端的，其实就是在发展的过程当中，我觉得是可以有些改良的，但是千万不要有点像是说把以前的就全部抛弃掉，抛弃掉可能也不是一个最佳的模式。那我觉得目前来讲那不外乎就是说将来有可能我们这边也会变得更多元化一点吧，就是说一方面如果你是有能力，就是说发表国际最顶级期刊的，那很好，但是如果你是例如说你偏实践一点，就是说你有这种政策上的贡献，或者说其他方面的企业层面的贡献，那应该也是会受到一些认可。另外呢就是中文的期刊和英文的期刊，可能也会有一个更均衡的这种评价体系，我觉得这个应该是大家会比较有共识的，就说这个未来应该是朝这个方向去发展，商学院它会更有活力一点。因为现在的一个问题就是有的时候过于极端的强调某一个方面，就会使得在那个方面有特长的可能少数人吧，就更容易去有一个过多的奖赏，但是其他的大家也是在认真工作的，可能就这方面的就得到的就比较少，所以我觉得还是处在一个探索的阶段。

当然对我个人来言的话，我会觉得，我本身是学心理学的，后来进了商学院，所以有这样的一个跨界的这样的一个履历，当然我觉得这种跨界呢它也是有好有坏，因为如果说你看我个人的profile，我发表的文章其实还是比较偏心理学一点，那这个其实就使得我的这个背景在有些商学院里面其实也不会得到特别高的一个认可，因为他们会觉得领域的差异嘛，他会把你定位为是应该去心理系的一个人，但是因为我当时去的是英国嘛，所以他们一个多元化的评价体系，他还是会认可到我的研究成果，所以我会觉得这个对我来讲也是一个挺适合的一个工作环境。

当然我我会觉得可能在不同的阶段，大家获取意义感的这个来源是不一样的，就是我完全理解说在比较年轻的这样的一个职业生涯阶段上，就是说你要从讲师要变成副教授，或者你要拿tenure这个阶段，我觉得大家的这个意义感很多时候是来源于一个security，来源于一种安全感，就说我安全了，我才感觉他有时间和精力去兼顾别的，所以我完全能理解在这个比较早的职业生涯阶段，大家可能会发文章就会比较的务实一点，就说这个对我下一阶段有什么帮助，但是我普遍上会感觉就是一般你拿了tenure或者说你的职位比较稳定了，但英国变成这个permanent contract，大家可能都会想一想就是说更高层一点的心理上的意义感。所以就我目前而言的话，我会觉得研究肯定我还是想往前推进的，当然推进的模式可能更多的是希望会有一点理论上的进步吧，就比如说这几年我会写的理论性的文章会多一点，另外呢可能发表的话我也会尽力去发这种世界上最顶级的期刊，虽然世界上最顶级的期刊也不是完美的，但它至少是一个很好的检验，就是说看你的这个水准有没有得到同行的认可，而一般你能发到上面的基本上还算是有点分量的东西，所以这个我也是在努力能够达到这个水准，但是我不会说过于强求，说我为了达到这个我一定要去做一些我可能不太认同的事情，这个我可能会有这样一个自由度吧，至少在这个阶段上，所以说研究基本上有这样的一个定位。

其他的那基本上就是带学生嘛，我觉得带学生我个人的基本的原则就是用我们中国人的话来讲，就只要有缘分的，那基本上都可以带一下，所以有时候跟我一起开会合作的学生也不是我真正带的博士生，可能就是在网上认识的，或者说他主动跟我联系的其他学校的，我觉得如果大家有共同的这个方向，而且他有能力往前推，那基本上就可以合作。

另外呢就是还有一些偏公益性的，就比如说像什么b站的讲座、公众号，这个如果有时间的话我也会尽量弄一下，但是现在面临主要的问题就是有时候确实难以兼顾，所以有时候有很多东西也很难做到很及时的更新，我觉得等我在这个深圳这边可能稍微更安稳一点，有可能时间上会调理地会更好一点，可能会带领一些不仅是我自己的学生，可能外部的学生，包括说大家同行之间的交流可能会更多一点，这是在这个教育这个层面。

当然在社会服务这个层面，我觉得目前来讲的话，可能主要还是跟公司的合作会多一点，就是说我们可以去开发一些这种测评工具，或者说这种测评的系统，当然如果你要说大一点，我觉得将来不外乎就是说能够跟现在整个社会大家比较关心的一些这种现象层面的问题，如果能够结合起来会更好，就例如说像职业生涯教育这个事情，我们现在大家都会看到，就是家长也很焦虑，因为国家政策也是拼命的把大家的时间和精力引导到说不要过度去上那种课后班儿这种，不要走这个道路。但是我会觉得如果把这条路堵上，那家长的选择在哪里？这可能就构成一个问题，因为你把这条路堵上，相当于是说你不能用这种商业的模式去解决小孩儿的课外活动、兴趣班的问题，那这个家长应该怎么去安排这个时间？我觉得这可能作为研究者来讲，有可能是可以在这个过程当中去起到一些作用，就比如说像职业生涯教育这个方面的有一些这种比较成型的理论，你如何在这个场景之下落地，或者说去解决一些实际的社会的政策层面的问题，这个有可能是也会是一个社会服务的一个很重要的方向。另外呢还有一个就是一些特殊的群体吧，就比如说包括留守儿童，或者说包括说有一些特定的职业，就说像我们说这种零工经济，那在这个过程当中他们的职业生涯发展的一些基本的诉求，如何上升的一个法律的政策的角度，就是给他们去争取到一些合适的这种权益或者保护，我我觉得这个应该都是可以去做的一些事情，所以说我会觉得就我目前这个阶段的话，我会觉得在做研究这个层面还是有比较大的自由度可以去选择，就是基本上是兴趣驱动了，另外一个呢还是希望说应更严格的要求，看看自己能不能达到那个水准上，然后教育和社会服务，我觉得就看你自己有多大的能力能够拓展到更多的人身上吧。

但我觉得这个时间确实是很有限的，再加上我现在实际上在英国杜伦那边还是有一个兼职的，所以有时候还要面临两边跑的问题，另外还有家庭的问题，所以有的时候我会觉得其实可能自己做不了那么多事情，那在意识到这一点之后，我会觉得有些事情是必须不能耽误的，我可能会划定一个边界，就是说如果是学校里面的这些事情，我肯定是不想耽误的，就是上课或者说带学生或者什么的；然后其他的延伸性的活动，那基本上就是要看时机和资源是否合适吧，如果合适的话就可以做一点，但是如果实在是顾不过来，那也没办法，那可能就只能是做一点减法，大体上是这样。

所以说我觉得你刚才提的这个问题基本上可以从管理的角度，就是包括商学院变革的角度去考虑这个问题。我会觉得中国的商学院肯定是变得更好了，总体上是变得更好了，起码也跟国际接轨了，水平更高了，这个确实是一个事实。但是在这个更好的过程当中有哪些可以改良的方面？这个其实就是面向未来的一个更重要的问题吧。而且这里面可能比较忌讳的一点就是矫枉过正式的这种改革，那可能会丢掉一些已经有的好的东西，然后新的东西也没有完全学会，这个可能就会比较尴尬。

当然个人层面的，你刚才问的这个个人层面的这种意义感的来源，我觉得这个确实个体差异比较大，所以我也仅仅能说一下我个人感受而已，大概是这样。

刘书博：那还有一个问题就是您现在杜伦大学有个职位，包括之前也是全职在那边做全职教授，那在英国那边您观察到的这种大学或者是商学院的这种管理模式，跟中国比如说人民大学这种体制内的，还有现在港中（深），那这种大学和学院的管理模式，您觉得是不是有非常明显的差异？

还有就是相关的学术共同体，那在中国的这个学术共同体的概念是否跟英国、美国他们这个学术共同体的概念是不同的？

受访者：我觉得从管理上来讲的话，这个差别确实挺大的。因为英国相对来讲，我觉得英国首先是有它的传统的，尤其是有点历史，大家都保留了很多历史传统，像它的三学期制，包括说这种书院制等等，有他的这种很传统的东西。这种传统有时候体现在它有一个背后的体系，再去指导这个大学的一些日常的基本活动。就是说你如果去查杜伦大学的校历，三年之后的校历都给你出来了，就说我们就是按照这个不断的去运行它，它是有一套背后的体制的。但是另外一个方面了，因为英国也是一个比较崇尚民主自由的这样的一个国家，所以他同时又特别注重学生的体验，同时老师也可以加入工会去保护自己的一些基本权益。在这个里面你会发现英国的大学也具有它的某种两面性，就是他比较传统的这一面了就会导致有些事情进行的不太有效率，就例如说如果我在英国我要去开一门新课，那就要经过好几个委员会审批，而且那个委员要开会，但是它有时候一年就开两次会，那你如果要开一门新课，你今年申请你明年才能开。但如果我记得我以前在国内就说如果你要开课的话，那基本上12个人选课成功了，你跟学校简单申请报备一下就ok了。所以说我会觉得我在英国的一个感受就是他确实很尊重老师，你如果去跟学校打交道，一般他都会有回应，他会尊重你的基本的权益，但另外一方面了，有时候他们问题解决的能力可能受制于他这种传统性，这样的一个庞大体系的这种限制，有时候降低了效率，就是说一个事情，他其实也是要经过层层审批的，有的时候了你可能等待的过程确实也会比较长一点，这是我的一个基本感受。所以说我在工作上遇到了一件事情，就是你需要打电话去催行政人员，因为你发邮件的话，他有时候就处理的没那么快嘛，而你打电话去催，他们永远都是极其有礼貌，然后特别郑重地给你道歉，但是有时候他这个问题解决能力真的就不是那么强，或者说不是他一个人能解决的问题，他也没办法，所以就不停给你道歉，不停给你道歉，但是到最后说那我应该怎么办？我还要等几天吗？他说你再等等吧，有时候会出现这种情况。

然后以前国内工作的一个经验，就是说中国国内的体制内大学，一般来讲还是秉承着一个行政管理的思路去管理老师，这里面也会有这种两面性。如果你刚进学校，作为一个新老师，办事情就会很程序化，但现在会改进很多，中国大学一直在改，在不断的提升嘛。我记得以前的话就真的有点像进入一个事业单位，这种感觉是你跟项目机关打交道时这种感觉最强烈，就是你有的时候去办事儿，有可能就不是那么有效率，就有时候会跑好几趟，或者说有时候就是盖章，或者有些你稍微有个细节没弄好，你可能就要再去找别的人，所以有的时候尤其是年轻老师，你刚进入这个可能就会有点不太习惯，尤其是海归的那种。但是我后来又发现有另外一个极端出现，就是如果你在这个大学待了几年，就大家都混熟了，关系也都搞得不错，就进入另外一个阶段，就怎么着都好说话了，就说甚至有时候就会帮你走一个绿色通道，可以，可以，这样就可以了这种。但如果我不认识他或者我还是个年轻老师，这个绝对搞不成的。所以就会让你觉得这个平衡好像就不太好去界定，或者说你作为一个新人来讲，有的时候你摸不透的话可能就会有比较多的试错的代价。

另外一个了就是因为他是一个行政督导的这样的一个体系，他有的时候了还是从一个管理的角度去想问题。比如说老师的基本的诉求，有的时候他没有那么敏感，那基本上就是说你有些基本的生活或者说工作上的诉求的话，他可能会告诉你说可能学校就是这个样子，那你可能要自己克服一下或者怎样的，这个细节就很多了，比如说你报销这个方面有时候就其实规定的东西太多嘛，然后你要自己去把这些都弄好，其实是挺麻烦的，但是有的时候一时半会儿改变，改变不了这个，所以有时候老师只能是再找一个学生助理全权去处理这样的事情。再比如说你日常办公室的基本的配备，或者说你有一些跟人事处要打交道的这样一些流程，其实有的时候就会让你感觉这个在管理上一刀切嘛就比较容易，但是具体到每个人身上，他有的时候不会让大家的生活变得更简单，反而增加了一定的复杂性，这是我的一个基本的感受。但是我会觉得像这种情况，你一旦混熟了，你知道一些在关键岗位上的关键的人之后，反而有的时候是可以有一些便利。但我不觉得这是一个特别健康的运行办法，因为它带有一定的不公平性。

然后我在港中（深）的一个入职的体验还是不错的，但是我说这个话还是有一些个人偏向性，因为我们组织管理学当中有一个很有趣的效应，叫做honeymoon效应，就是你到一个单位还没超过三个月的时候，你总是会说他的好话的，所以有可能我还现在还处在那个阶段上，所以容易看到他所有的优点。但是我想我是6月份入职的，现在已经9月份了，基本上过了三个月了，有些事也是基本的事实。我觉得港中（深）他有一点做的很好，就是它的行政人员配备的特别的足，这个就能解决很多的问题。因为我以前在体制内的高校工作过，我会觉得其实很多行政人员都是非常有能力的，但是因为配置的行政人员太少，他一个人就会要忙很多的事情，所以在这个过程当中，我有时候会觉得不是怨某个人，而是说因为他配置的资源少嘛，他一个人忙各个方面的事情就使得他疲于奔命，有时候那有些细节照顾不到可能就容易出问题。

但是我觉得是港中（深）这边其实配置的人首先是足够的，至少从我面试到入职到入职后的一些基本的行政管理，首先我会觉得我接触的人很多，就是说他每个人分管一摊儿嘛，面试的时候院系层面的秘书我会有接触，后来再上升到学校讨论那个阶段，那我会跟学校人事的人对接，然后当时他给我发了offer之后又有一个入职的同事跟我对接，而且入职之后像说给你找房子什么的，这些都是有专人跟我对接的。而且专人对接一方面是人比较多，另外他们之间的对接我觉得还是很有效率的，没有说你找了这头儿，那头儿就找不到人的这种情况，这种确实比较少。所以说在某种程度上，我会觉得他是兼顾了一种效率和关注老师个性化的体验，这个是很难的，因为我能想象到这背后其实要投入很多资源，因为相当于行政人员的数量，可能是有些学校行政人员的好几倍，这是一个基本的情况。那你这个基本的这个运营的成本肯定会增加很多嘛，同时他还是需要有一种人力资源管理的理念去贯彻在这个流程当中，就不仅仅是一个最基本的对老师的一个行政管理，所以说至少从这个角度来讲，我觉得这个体验是不错的。就是说实话实说，因为我待过的学校比较多，就大部分学校给我感觉就是前后不太一样，跟自己想的不一样，而且大部分就是比自己想的差，其实港中（深）的活会让我觉得可能比自己想的还要稍微好一点，我会有这种感觉。因为一开始我之所以愿意考虑这个学校的职位，不外乎就是说我首先是香港中文大学毕业的博士，就说算是回母校做贡献，虽然是不一样的学校，就是其实这两个学校是独立运行的，但是挂着同一个牌子嘛，会有这种基本面的认同感。

另外一个就是我觉得薪酬层面，他们的薪酬确实是挺好的。但是你说的这种管理的一些机制，这个其实真的是要通过你个人体验，你才能做一个判断的。我会觉得确实做的是不错的，就说很少有事情说系主任不给你办，也很少有说不太考虑你的个人诉求，就直接给你比较简单粗暴的处理了，不太容易出现这种情况。

所以我会觉得就说刚才讲的这三大类，就说英国的话，我会觉得他其实对老师的这种基本的态度，对待你的方式其实还是充满了尊敬的，充满了对你的个人的一个基本的尊重，但是我会觉得他的这个背后的体系是一个注重质量控制、防止出错的体系，就是一个比较传统的一个体系，所以说他有的时候会拖慢它的效率，这是英国给我的一种感受。

那国内的比较典型的体制内高校，我会觉得他更多的就是采用一种国企式的，就是我们比较传统的，当然我就可能对国企有偏见，我可能不应该用这个词，就是他们更多的是用一种行政管理的方式去管理老师，我觉得可能是这样的一种风格。另外因为配备的人手不太足够，所以说有些事情可能处理起来他的效率，包括说他的这个效果可能就不是那么理想，所以说刚入职的老师可能会导致遇到的问题比较多。当然我刚才也有讲就是国内的这种这样的一个大的环境，就是如果你在学校里待的久，你摸透了这个环境，就会知道怎样做都有效率，或者说你可能认识某个人可能也会容易给你一些便利，这可能就是这样一个特点。

然后港中（深）的话，目前来讲我会觉得他是在努力把效率和人性化看能不能做一个结合，因为港中（深）面临的问题就是在学校层面他拿到的资源没有那么多，因为它是靠一个市场化的机制在保持这个学校的运转，当然政府是有投资的，只不过投资力度确实没有本地的其他的高校那么多，就是说在深圳本地，像深圳大学或者南方科大拿到的政府的直接资源肯定是远远高于我们的。但是港中（深）这几年我觉得在老师的招聘方面，他的口碑实际上是不错的，现在港中（深）是非常难进的一个学校。另外在这个招生方面，现在每年口碑也在增长，再加上最近办了商学，办了这个医学院，学校的规模也在不断扩大，就说如果是一个市场化的学校，这个学校能在规模上不断扩大，说明他这个教育这一端其实做的应该也还是不错。所以有可能这也算是一个还不错的新的模式吧，在我看来我大体上是这样的一个感受。

刘书博：好的，谢谢管老师。那还有一个相关的问题，就是那同事之间的这种相处，就这种非正式组织的那种文化氛围，因为我在英国爱大，包括在华威商学院，我在那边读书，我觉得可能跟中国的这种同事的氛围还是有不同的，不知道您有没有同样的感受？

受访者：就学者与学者之间的这种学术共同体的这种的感觉，我确实觉得非常不一样。我觉得相对来讲，英国，包括说有一些这种偏个人主义的国家，大家首先工作和生活的边界会划定的更加清楚一点。就是说至少在英国是这样的，就是如果你请一个同事吃饭，像喝酒是ok的，就有时候好几个同事一起下了班喝喝酒小聚一下，这个是大家觉得是正常的社交活动，但是如果你单独请某个人吃饭，或者说请人去家里吃饭，这是很大的事儿了，就证明你们的关系真的是很亲近了，但是像这种就发生的概率就很少。我在杜伦我个人的一个体验，包括以前在萨里我个人的一个体验，就是大家就是各自独立的，然后你甚至可以说在这个学校里我就是把自己该做的事情做好，然后比较极端的情况，我跟别人打交道比较少，这个也都是ok的。

那他会不会影响你评审呢？这个影响我觉得取决于你在这个过程当中，如果是你在英国，他有时候考察你的领导力，你行政上的贡献嘛，那在这个方面了除非你有可以表现的场景，能够表现出这种，但是如果你没有的话，那确实会有一点影响，但是其他的影响就不太大。这是我的英国的一个感受。

那这种情况下这个学术共同体验这种community，这种感觉如何建立呢？我觉得学术共同体其实也是有不同的这种identity，你的不同的身份的。一个就是你基于大学这个架构的一种identity，就是说我是这个大学的员工，我是这个院系的员工，那我们应该团结一致，我们应该怎么样。这个大学里也会组织一些活动，就是说每年开会呀，包括圣诞节，大家都会在院系层面组织一些活动，只不过了我会觉得反而这个层面的这个链接其实并没有那么那么强，我会有这种感觉。

那什么样层面的链接强呢？它反而是你出去开国际会议，你找到一个共同研究领域的这样的一个可以跟你去聊这个共同兴趣的这样的一群人，大家反而关系会更紧密一点，甚至能成为很亲密亲密的合作者。所以说我刚才说的这个边界感可能指的就是这些层面，就说他如果要有这种community，他很多时候是体现在可能是跨了学校和院系的一个在专业层面有共同兴趣的这样的一个community，他们其实还是很活跃的，包括说开国际会议的时候。但我们会发现其实就是英美国家的这种同行，他们很爱提问，很爱去跟别人去做这种社交活动。

当然在这个还有另外一个层面的community，我觉得就是一种业余的爱好或者兴趣这方面，其实英国做的还是挺好的，就是说你可能在工作上没什么交集，但是我发现大家都是去跳某种舞的，就是课后大家参加了一个兴趣小组，跳舞的或者某种运动，他会通过这些形成各自不同的这样的一些community，形成这样的一些小的社交的这种群体，所以基本上就是基于你个人，一个是专业上的有哪些人你愿意去跟他们产生联系，还有就是业余爱好产生联系。当然院系大学这个层面是因为大家都在一个共同的环境当中工作，所以说也必然会产生联系，但他并不强求说，我们作为这个同一个院系的员工，大家要团结一致，或者他有时候不是特别说强调一定要干这个。

所以说这个可能跟国内就会不太一样，我觉得国内这种特点是什么呢？国内的特点就是首先这种导向，大家一般会有这种集体主义导向，说你都是这个大学或者这个学院的老师或者员工了，那你应该怎么样怎么样会有这种基本导向。另外一个就是这里面其实有些利益关联的。其实国内的这个很多评职称，这方面的一些操作还是要通过学术委员会或者教授来投票的，它会形成一种导向，说你总得搞好关系嘛，就是说你让大家认识你也好，或者说给大家留一个好的印象也好，你可能会有这样的一个动力，这个跟国外不是完全一样的。在我看来当国外你平时成绩要经过这样一些资深的学校里一个小的委员会。

但是国外他不太容易产生那么大的一个长期的这样一个经营的这种感觉，就是因为我觉得中国人在社会关系上边界感是很弱的，有时候工作生活这个边界甚至都分不清的，包括老师跟学生，我觉得我在国内教学很重要的一个个人体验，就是有时候我跟我学生因为关系太近了，他有的时候会有一种分寸感，我觉得还是要维持，这个会造成这方面的困难，当然我一般愿意长期交往的学生，他们基本上还是有分寸感的，他知道关系好，好是好，但是在专业上的工作上还是要认真要求自己的。但是我也会遇到一种学生，说难听一点就是有点恃宠而骄，就说我跟老师关系好，我就可以耍个赖皮，或者做的不是那么好，我也可以去跟他交差，当然像这样的，我后面可能会刻意的跟他们保持距离。

只不过说可能对于我们很多人来讲，这个其实是挺难的，就是你跟学生毕竟不是一个天天接触的群体，你跟同事的话这个可能就会有点难。我记得我记得我以前在的时候一个老师讲过一句话，说这年头你离个同事比离婚还难，就是有时候大家就是要一起工作好几十年的，除非大家跳槽或者说有别的事情发生，要不然你就得年复一年的去面对同样的一群人，所以在这个过程当中就容易因为边界模糊产生了这种关系上的这样的一种复杂性，这个确实是一个对很多人来讲是一个挺难处理的难题。尤其是在比较敏感的阶段上，如评职称或者说有什么利益分配的这样一个场景当中，就会导致圈子的出现，就说在关键的场景谁支持我，谁跟我产生了竞争，然后这种将来就会产生某种圈子的格局。然后甚至会影响到新来的年轻人所谓的站队的问题，这个确实就会造成一些特别不好的一些后续的影响。

只不过说根据我个人的观察，我会觉得这里面会有代际差异，我会觉得更年轻一点的老师，大家的交往还是相对简单一点的，当然我对老一辈人并没有那么多的成见，我会觉得可能因为之前的制度的设计会使得大家会有某种倾向性，现在的话我觉得在制度上虽然有可以更继续提升的地方，但是整个的大家的这个眼界，包括说年轻一代的老师的价值观可能也在发生改变，就会觉得比以前会更简单一点，这是我个人的感觉，只不过说这种系统性的差异他可能还是会存在一段时间，大家还是这样的一个感受。

刘书博：那管老师在英国那边对于申请基金项目，跟国内这种感觉是不是也不一样？因为国内好像把这个事情变成一种每年都要去做的锦标赛，那这个英国那边是同样这样吗？

受访者：这个其实还是挺重要的，在英国申请方面，就是说在英国评职称的时候还是会看你的funding的。虽然商学院有的时候很难拿到大的funding，但是他还是会看到你，你至少得努力去申请过，甚至拿一些小的funding，像1万的小基金吧，但你拿到了也是一件大事儿，就相当于我们国内上拿了一个国家级的基金嘛。

其实在英国最好申请的基金是那种特别偏重一些社会上的热点问题，就是说在西方国家社会上的热点问题永远都是心理健康、少数人群的保护，LGBT，包括说对某些第三世界国家的关注，就说非洲某个国家我去研究一下，他们确实是有这样的一种导向性，就是你研究这个就是天生的占便宜，就会导致商学院的项目很难申。比如我去研究领导力，那很多评论就说地球都这么水深火热，研究什么领导力，研究领导力不还是为企业服务，然后不跟你批，所以拿到的钱很少，而且拿到钱的机会很少。但是他还是希望你能拿到的，如果拿到了会有很大的优势，基本上这样。

国内是不一样的，我觉得国内其实我们获得研究基金的机会比英国要多太多了，就是说每年的虽然国内有一些不太公平的现象，但至少像自科基金现在做的是不错的。所以了在这个过程当中，年轻老师你申请还是有百分之二三十的命中率嘛，而且拿到了也是不小的一笔钱，大约20万左右。这个真的是很不一样，就是我觉得实事求是的讲，我们国内的年轻老师在研究基金方面的这个资源其实是比英国的要好的，起码商学院的情况是这样的。

# 受访者28

受访者：估计我们说不了太多，因为我回来得比较晚

刘书博：回溯性呢我同时也在做访谈，就是比如说我找到一个西安交大的受访者老师，他是82年就毕业，在工商管理这个口里，我明天会再跟他做第二次访谈，上一次跟他聊，就是从改革开放到98年，然后再一次聊呢就聊98年之后

受访者：哇，那他真是历史的见证人

刘书博：他给我很多有帮助的二手资料，然后我就最近也在读嘛，包括那个钱颖一他是清华经管的院长，他说了好多这种话语，我读了之后，我就觉得其实这个意识形态还有就是中国的这种大转向呈现的非常明显。

受访者：我觉得这个人呢，其实因为我不知道你聚焦的是工商管理还是大管理还是经管，因为它的口方向不一样

刘书博：工商管理

受访者：那工商管理你像钱颖一我猜他还是偏经济口，其实他这个意识形态这一块儿我觉得更多的还是从经济这个（口儿）。因为我之前跟学这个专业的人交流，他们是在90年代或2000年之前经济口都是叫政治经济学，就他没有这个市场经济，即便市场经济已经20年了，对吧，但是感觉还没有，到现在后来又有一波，那现在感觉又有点稍微往回去追溯的感觉。

那你像工商口的历史上咱不知道，因为你做的工商口BA，business administration这个口。

但如果你做的是大管理，大管理必然包括管工这个口，那管工这个口其实就蛮有意思，就是它其实还蛮延续性的，因为这口儿最早就是华罗庚，他们搞的数学就是运筹学或统筹，然后后来西交、清华延续下来这个口的。我感觉啊，如果你不是大牛，基本你参与经济活动的都比较少，就纯粹就是（模型），当然也是现在批判所谓的偏模型化，就是现在在批判这个事儿，但是这个口的特点就是偏模型化，但它的好处就是它一直是中立的嘛，它很难发生左右偏颇的变化。

刘书博：对，所以就是像你们比较偏工程、理工类

受访者：对偏理工，当然也是被《管理世界》批评的，就是所谓你做的东西太过模型化，而且这个模型化还跟你实证化还不一样，因为实证是拿数据去跑模型，那我们那个模型呢就是有点儿工程的模型，那这个东西呢就是虽然被批判，但是他确实一直在走这条路

刘书博：嗯嗯，所以我理解，我在看那个就比如说《20世纪管理科学》那本书，他编的有中国部分，其实就是我们最早的管理学教育就是工程管理，就是新中国包括新中国之前的民国时期，他们其实是工厂管理。

受访者：就是泰勒的，我们现在还是在做泰勒的，没错，就没有任何区别，你像我们自己这门课虽然有很多新的方法，其实那天之前跟林琳交流的时候也聊到这个问题，就我们这个专业(就管科这个专业)他是没有理论的，就纯数学你就搞来搞去，你纯粹都是技术性的东西

刘书博：但是你们理论就是物理学理论

受访者：但这个理论就是没有思想，就是跟管理学的这个theory是没有对应的一个东西的。

刘书博：就比如说它没有假设，比如说人性的假设

受访者：他没有人性假设，他更多的是一些客观的假设，比如说我不知道你人是好是坏，但我知道你的产出是十分钟一个，我不管你好坏，反正十分钟一个，然后我就开始做后面的，但是如果你按实证的套路来说，那可能你这个人之前的好坏，就是价值判断，会影响你这十分钟一个，然后进而影响你的产品效果。但是我们在那一块儿一般都会认为是给定，就是泰勒的做法

刘书博：没错，所以我就看书，他说那个民国到新中国到文革到现在，其实你们没有断

受访者：对，其实是没有断

刘书博：因为你们的研究内容里理论也没有涉及到意识形态

受访者：对，而且还甚至都没有涉及到理论，就是说的东西都没涉及到理论，就纯粹就是天天算1+1=2，只是在不同场景、不同的环境下的1+1=2，那这个理论其实就没有说···

刘书博：所以你刚才说华罗庚还有钱学森他们都是在推动着你们的管理工程领域在往前走。

受访者：对

刘书博：但是呢，比如说经济、工商管理甚至社会学在文革期间期间就停了

受访者：就停滞了，因为你不可能再有一些理论去···，因为他需要理论来推动，因为你无论是实证还是案例法，你肯定前面有一个理论，就是叫假设或者假设发展或者理论拓展的东西，就这一块儿就是很依赖于思想的，我感觉依赖于思想深度和···

刘书博：而且思想和意识形态，比如说像经济学，那可能就是市场经济；那工商管理，它就是市场经济和企业管理吧

受访者：或者举个例子，比如说现在咱们趋势是国内发文章了，有这种类似这种趋势，不一定是真的趋势，反正就假设有这种趋势的情况下，你像对我们的冲击就纯粹仅仅是，比如说国内期刊每月发表十篇，那这个量的问题盘子做大的问题，但是可能对某些学科而言，它的影响不仅仅是盘子做大，而且还有这个风向的问题。就这个很明显的，他有风险，而我们其实就没有风险

刘书博：你们是没有考虑这个刚才说的政治导向、价值观、意识形态···

受访者：对，我们只有盘子做大，比如说一共十个期刊100篇文章，那我们就抢这一篇篇文章的坑呗，对吧，但这个坑的抢夺跟之前的意识形态大部分不太关联

刘书博：ok，所以咱们刚才谈的就是你在这个细分的学科，它应该属于一级学科，我不知道你看没看这本《20世纪的管理科学》书，他其实说的就是中国的管理学怎么样从没有到二级，然后二级到一级，然后自科基金也是专门有一个部出来给工商管理。

受访者：对，因为你看一处是说管工嘛，一处是管工，二处才是工商，你从这个顺序来说，那可能是如果有顺序那应该是先有管工

刘书博：对对，所以你们（学科）在中国它是早于工商管理的

受访者：它（管工）甚至可能还早于经济学，因为我记得80年代中后期就有这个专业的博士跟硕博了，经济学可能还得往后。

刘书博：对对对对，那我们下面就进入第二个大的内容，就是你作为在商学院，尤其是在海外有过这种博士教育经历，然后回到国内，那其实咱俩都一样都有一个对比的视角，那我们就可以聊一下，就比如说到中国之后，回来之后，有没有一些觉得就比较意外的呀，或者是奇怪的呀，或者是不同的呀

受访者：虽然咱们从书面上，国内国外肯定都说这个teaching/research/service大约应该442，对吧？国外肯定也说442，国内也是442，国外也肯定不能严格说你40%、40%、20%，但相对而言感觉国外对这个442这个把控上还是比较符合贴近这个442，比如说service你可能多到25%，但是第二年可能也就···就roughly嘛。国内这个呢，就得看你这个单位是怎么安排你的时间了，而且尤其是像service这一块儿，我估计不同的单位、不同的学校可能要求也不一样，实话实说，如果你这个单位对于0.4的要求比较高，比如说当前你像某些学校他对0.4的要求特别高的情况下，它可能service甚至不给你0.2，他给你0.001，就一点service都不让做，甚至另外的0.4都不给你安排，他给你变成0.9或0.8，0.1，0.1，对吧？那这就相当于组织有他自己的目标要求，但是也有一些学校相对来说就是对0.2的要求会特别高，他要求0.2特别多，但他不care你之前0.4跟0.4做的怎么样，这个就很依托于你就职的学校，因为我之前跟其他的同学交流的时候，包括回来的人也是，就差别非常大。

刘书博：所以你说的就是国内的情况

受访者：对，这是国内的情况，国外呢我感觉跟我朋友交流的情况基本上，除非他是真正的就是专门做postdoc的，就至少做lecturer的，大概率我问0.4、0.4、0.2这个事儿，他们说差不多一学期一门课或一学年两门课，然后再加上research的要求，再加上他service的要求，可能参加一些比如说junior faculty committee，就说年轻老师的一个committee，对吧，那他这算是service的一部分，然后加上他们有很多专业的行政人员来做，其实他0.2也做不了多少，就这个比例上你感觉跟一学期一门课和他一学期干了点事儿比例差不多，2:1的比例。

research而言，就是因为你教课少，你自然有research时间嘛，对吧，差不多你像一周五天，两天教学差不多了，刚开始你还得备课嘛，对吧，上一天的课，当然不是完整一天，一天来教学，然后两天的时间让你去做research，一天的时间让你万一有点儿活儿来单位或者有committee的会呀或者有些活动，那这个我觉得基本上就是我能了解基本上这个442的比例在40个工作小时的时间下基本能保证，那其实变相的就相当于增加你的research时间，因为剩下的两天学校不要求你做嘛，那就是你自己安排了

刘书博：我感受到英国也是这样，那可能根据大学不一样，像教学为主···

受访者：对有教学为主，相对而言嘛

刘书博：然后我感觉对比起来，他们（国外）似乎就是更加悠哉悠哉的，他不是有点像就很急的

受访者：因为它的442的比例是用40个小时来分的，是你工作每周工作40个小时，但是你实际上你每周肯定不止40个小时，你周末至少还有16个小时嘛，对吧，那为什么他悠哉悠哉，因为我既可以用周末的16个小时是来完成我442里面那个research的4，再多做一个、多做一点儿，或者我就不想多做，我就纯工作40个小时，那周末我就肯定有一天要去做礼拜，大部分国外的这个老外基本有这个···

刘书博：半天的礼拜周的faculty，就是大学的这个学者，我不知道你对于美国他们了不了解

受访者：美国少一点，确实可能英联邦更多，因为英联邦有个特色，就是它的promotion的压力可能没那么大，但是美国可能会更多一点，但是我了解在美国的老师，首先那个教学的事也不多呀，我没听说过他们抱怨过教学跟service，就我从来没听说过他们抱怨过，那意味着其实他research压力大是因为他的竞争的压力大，但是学校给他分配的这个其中的一个四和其中一个二应该是不太变化的

刘书博：所以这个442其实在澳洲，我觉得在英国也是，就是大家都是有一个这种比较稳固的这样的一个工作状态

受访者：而且我觉得最重要的一点就是它这个442的这个分母，它是来自于40个工作小时的，而不是七天的，这个不太一样，因为你搞成007的442跟996的442跟855的442那完全不是一个概念

刘书博：那边是不是老师也不是特别的workholic，就是感觉天天都是在就有点锦标赛的那种

受访者：我觉得还好，因为首先招人的时候他就不是这种集权，集权是一下子招很多人，对吧，他一般是填坑式的；然后另一个就是说其实他对promotion的要求也没有到高到说你一定要完成什么，虽然英国有pbrf，现在也学这个所谓的叫performance based research f···，反正类似于就是关于大学的科研评价，也有量化指标，但是呢他这个量化指标其实我理解的就是也没到那种程度，比如说我今年从第10名掉到第12名，我感觉，至少校领导没有到说，哎呦我完蛋了，我这是从第十，是吧，一下子掉了两个名次，我这官儿都当不了的感觉,我觉得是没有那种感觉

刘书博：而且就英国比如说院长呀或者是系主任呀，他们都是好像就是一个服务性的，他是轮着做这个

受访者：对对，这个dean和HOD呢，至少首先从phd修炼的这个角度来说，这个dean和HOD是没有任何官职的，他只是我在某一个报告可能签字，但这个签字其实也是我导师转过去的，其实跟学生没什么关系

刘书博：所以他还是很强的专家治理，对吧？他不是官员

受访者：对，他没有行政，因为他的dean跟HOD肯定都是professor，都是教授级别的人，而且往往是轮流，而且很多时候很多人不愿意去干，因为他确实要花时间

刘书博：对，对对，就像我们那个商学院的dean可能会还有一点权威，但是系主任什么或者是研究中心主任就是轮流做

受访者：它就是个service，你就干个三年、干个四年，有的是很多老师是不愿意干的，因为他会真正影响你的research的，所以你刚才你提到说院长一看掉了，他会很焦虑，好像这个事儿就咱们来说就似乎是因为政绩

对，它跟你的政绩没什么关系，再说就算你把我撸下去，我还巴不得你把我撸下来，我还想搞搞自己的research呢，对吧

刘书博：对对对对，所以这点我觉得咱俩就共鸣，咱们这边就是学而优则仕嘛

受访者：对，他是有这个，但是这个东西呢因为你国家给他定行政级别了，那就不一样了，在国外他是没有国家级别，甚至很多当dean的并没有多工资，他工资也没涨，对吧，那活儿其实也没少干，而且工作的时候他可能他一点儿没减活

刘书博：而且大家对他也不是说有个一官半职就特别的尊重高看，是吧，并没有“官文化”，咱们这边是有的

受访者：反正我觉得这一点儿其实还是蛮明显的，其实我觉得这真的就是442乘以那个分母是什么，你如果乘以···

刘书博：我们刚才说的就是这个中国就刚才有点学而优则仕的这种行政官僚体系，甚至可以影响这个专业，就是大学的这种专业的

受访者：我觉得很明显影响这个专业，就是你像回到刚才我们那个话题，就是我们专业，当然你如果跟着主流走，就咱们说思想政治主流走，肯定不容易学而优则仕，这是肯定的。但是呢如果你就是比较中性的话，也不影响你正常生活，它是这样的一个过程。但是如果你像有些学科经济类的学科，对吧，咱就不避讳说原先说经济类很多学科一定要骂中国才能在国外发表，对吧，假设20年前这样子，那现在可能又不能骂中国了。但在我们这个管理科学这个专业呢，就说你没有那么多的影响，对吧，但是如果你能结合，那肯定越来越好，对吧，就是你又红又专那肯定是更好，但你不结合或者说你结合少一点也不影响你正常的去发表文章。

刘书博：对对，而且我们刚才也讨论了，像工商管理、经济学，包括社会学，都是带有意识形态的

受访者：它是就类似那句话，科学无国界，但科学家有国界。它是在一个特定的环境下的一个···，因为他社会科学就是跟人相关的

刘书博：它有学派

受访者：对，他有学派，而且他有这个诉求，他不是说解决实际问题，他不说解决实用，他是更多解决这个人和人相处的关系

刘书博：对，对对。所以其实像咱俩都是从这个海外这边也熟悉他们的那种氛围文化管理方式，那刚才咱们又聊了，就是他们像澳洲像英国都是442，这个比较···

受访者：我觉得442是比较···，因为我感觉跟我认识的人聊，或者换句话说就是换一个表述说，我回来的时候至少我想的是442，就是我回来那个瞬间我第一反应就是442，当然至于现实是什么样咱不知道，但是你能让我在回来的时候有442这个印象，说明我在国外肯定是潜移默化得到的这个信息嘛

刘书博：对，因为我是研究生和博士都是在那边，然后对于老师的那种工作状态，我觉得他们不是那么的匆忙，或者是大家对于一个事儿会非常的看的那么的···有点像这个排名，比如说这个像咱们这个经常每年都会有这个申请到自科社科（基金），然后会在群里这种好像张灯结彩、敲锣打鼓的，然后大家排队点赞的，那就英国那边这个···

受访者：我觉得在国外可能这种情况更多的是一个broadcast，就是我告诉你们说今年他中了自科、他今年他拿了100万，对吧，那就知道就行了嘛，对吧，我觉得也可能是因为技术的发展或者技术的应用，因为在国外全是用邮件，你邮件你不可能回复说，祝你点个赞，我所有人群发点个赞，那完蛋了，但是这个回来这个微信这个应用，我也不知道这是技术的进步还是技术退步，但是确实是就是你一方面你可能不一定愿意点赞，但是如果在这种即时通讯的情况下，很可能会有一种咱叫什么社会压力，叫social norm，或者说就传统的social norm不一定是对是错，但至少是一个习惯，就逼迫你还是要去点一下，对吧

刘书博：除了点赞之外，还有就是每年都得要申，就有一种四面八方推着你说“诶，你怎么不申”、“你怎么没中”类似这种，那我觉得那边就是大家平和一些，当然也有这种想要申想要中，但是似乎我们更多是说还是研究探索为导向的

受访者：我觉得还有一个原因，就是其实咱们这个专业是不需要钱的，你像你说我们专业做研究需要啥？这个东西（录音笔）可能最贵了，几千块钱，几百块钱，可能是除了电脑以外最贵的一个设备了，但除此之外就没有了，你说我跑个路费，那你出去跑个路费本来就应该是你自己掏钱的一个事儿，对吧，但是你在国内他可能这个东西不仅仅说因为我要去买这个设备、我要去申这个基金，而是因为我必须去申请基金，从而有这个钱去买路费。那你说我国外老师他也没有基金，那他不也正常去做研究、去做采访，那他开车花这个油费难道你还得找人报销吗？那人觉着我自己正常的一个···咱们就今天开会说的那个，就是正常的package里要求你干的活儿，我已经给你发工资了，你凭什么还要找钱去报油费呢，对吧，但是其实是习惯不一样了，因为你要是咱们是理工类的，确实我要养一个大实验室，我一年有1000万的进账，不然我实验室就解散了。但对于文科而言，我觉得大部分文科一年给你1万块钱，几乎都足够你用的了，而且更重要的是，你像我在国外的时候，所谓的开会的这个funding其实是学院的钱，就是说，这个business school有一个collective的一个funding，这个funding实际上是每年他们自己做的，就是说哪个老师说有叫这种的funding，就是说给你人均分了1万块钱，你要开会，那你就正常去开，反正你学院花钱嘛，当然他也不是说你一年只能开一次会，也说开两次，他不会明确跟你说，他就说你看你需求，但每个老师也不可能说我一年开12次会吧，那你这就属于···，我不知道那个专业词叫啥，我觉得肯定有一个词去描述这种social norm带来的压力

刘书博：对，所以我就觉得这个social norm，就是文化，明显的是我们是特别紧、特别一致性的

受访者：对，我觉得这个不一样，完全不一样

刘书博：就完全他们还就是种庄稼，然后想要去收获或者是种树、摘果子，我们现在就聚焦到了申请化肥上，因为你必须得申请化肥，我就不想申请化肥，我自己土肥，我自己种庄稼是主要的事儿

受访者：对呀

刘书博：但是现在就感觉···就像今天开会那个王校长说，十四五要每年每人20万

受访者：有这个吗？

刘书博：你没听到？

受访者：啥时候说的？

刘书博：他说了，他说这个每人争取···

受访者：王校长哪时候开会，哪有王校长开会

刘书博：不是那个林嵩说他去王校长开会，王瑶琪说就是争取十四五，咱们学校每人20万

受访者：我是不是在跟前校长在聊天呢？

刘书博：对

受访者：我就说我没听过每人20万。这个东西其实我听说过很多工科院校有这个，因为工科院校需要有这个funding去保持你实验室运行，但是我觉得对我个人理解，我觉得文科是不需要这么多钱

刘书博：对，我也觉得不用啊，我天天搞哲学研究干嘛还要经费呀

受访者：对呀买什么呀，那我20万我买啥呢，我觉得如果真的是说咱们说一定要搞这个自然基金或社会科学基金，我宁可说，你比如说那年不是从18万涨到24万吗，我宁可你不从18万涨到24万，我宁可从18万降到10万，但是你增加你的比例，他们年轻人其实就说能缺多少钱，不就是1万到十万，几万块钱嘛，你多资助几个年轻人，对吧，就跟咱们说实话，就跟创新管理一样，创新管理很重要一个特点，就是说一定要把变异系数或者方差变大才能共同产生好的点子嘛，对吧，那你把全国博士一人发1万块钱，这样更容易出现人才，因为你所有人都成活了才能真正长出来大树苗嘛，你重点施肥几个，万一你试错了呢，就跟你赌博似的就赌那几个号

刘书博：这就是理性的自负嘛，就是我觉得你现在这些标准都达标了，那你就是人才，但其实在现在标准看起来跟未来标准似乎是有不一样的，所以他理性他就觉得我是能够掌握你未来的，这就是理性的自负

受访者：那可能他就需要这种感觉吧，因为他如果没这感觉那他在这个岗位干啥呢？

刘书博：所以其实我就觉得刚才那个就是大家很一致，这个文化很挤，然后第二个就是我们在种庄稼，但是现在就变成申请化肥更重要

受访者：对，确实申请化肥就变成是就你说的，我觉得这个比喻挺有意思，我觉得你把这个翻译成英文，我觉得还蛮有意思的，这个比喻就从其实生产论文就是一个种种子成果实的，现在基金确实是很重要，没有人说它不重要，有化肥肯定很重要，但是他目的不是化肥，它目的是后面种种子那个结果

刘书博：尤其是咱们是这种不是像工科的那种实验室的学科，我们还是这种，你像我，我更加是手工操作

受访者：不用买数据，就完全的···

刘书博：对呀

受访者：还非得让我申，而且申的还是自然科学

吕梦娜：所以说这种基金会让老师们把这个注意力从培育种子，然后变到买化肥？

刘书博：特别会

受访者：我说你真的把这个这句话放成你的论文题目，就是从注重培养种子变成了注重购买好的化肥，这还真是一个很形象，你这么一说，还真是很形象的一个比喻

刘书博：就是异化，就我来之后我很懵懂，因为那些个老师也不教，他也写（基金申请）但是我就觉得他写的也不是那么难

受访者：真的就···

刘书博：投了肯定有钱，那多多少少，但是咱们这边就变···我来说这么难，然后我写了我收到了回复就不知道他在说什么，就感觉怎么这个意思就是说，你的导师好像不行什么的

受访者：还有这么说的吗？

刘书博：就我当年第一年就收到这个他们委员还是评委的回馈，给我的意见就对我没有丝毫的帮助，就是这样的。然后后来我其实我那个proposal还是基于我参加了一个英国大牛在做一个项目，然后我非常多内容都是在学习他的内容，但是他就申到英国那边，但我就投上去就不行

受访者：我觉得包括之前跟别人交流，也有说其实这个反馈意见纯粹就是为了反馈而反馈的，当然也肯定有为了你的本子提升、为了你研究的提升，也有这样的，但我相信那是少数，对吧，因为他没必要帮你提升，你从这个人自私的角度，他凭什么帮你提升，对他有啥好处呀

吕梦娜：所以学术共同体对咱们来说还是很弱的，没有这样一个···

受访者：学术共同体，这个本来就是一个很松散的一个结构，既然松散他就更多的靠着个人的一个···就今天上午我在上面讲课，就讲integrity，就你要跟老外任何面试工作，他肯定最常见的一个词就是integrity

刘书博：正直

受访者：但是我还真那天讲了半天，那integrity怎么翻译呢？它应该是什么意思呢？它跟truth、跟honesty是一个意思吗？好像不太一样，我后来想了意思，这个integrity是什么呢？是慎独，就是没人管着你但你还是做的很正直，还是你的principle嘛，对吧

受访者：这就跟你学术共同体似的、其实就跟审稿似的，理论上如果是为了学术共同体，我保持我的正直，那我的审稿意见应该是既有constructive去帮助这个作者去提升自己，同时也能反馈我的真实的一个审稿意见，对吧？但其实很多时候现在审稿意见变成一个暴力的发泄，因为他匿名了嘛，对吧，而且是一个泄愤的感觉，就是我也见不得你好，然后我中不了我也见不得你好，或者我中了我就不让你中，这种就是···我也不知道这是人性的好还是人性的坏或者人性的本质。这就为什么有一段时间他们一直强调就是你到底应不应该匿名评审，是不是就是应该真实评审，今年我看院士的推荐就要求公布推荐人的名字，之前所有你想想，从今年之前你就可以想象到从2021年之前这30年的院士评审都是匿名提名的，那这是什么概念？那为什么从2021年才开始（公开）？你推荐人为什么不公开？你为什么不能光明正大地推荐？

刘书博：对

受访者：我既然推荐你当领导，我就是认为你真是当领导的，对吧？那不然我为什么要推荐你？那现在变成了我推荐你偷偷摸摸的，我说我给你投票，我说我推荐这一个人去，那凭什么？那为什么你不好意思站出来呢？对吧？“战队”是个贬义词，但是在有些正直的情况下，你应该去明显的表现出你的态度，说我同意他参加院士评审，因为我相信或者我知道他在某些方面做得好，你得敢说出这话呀，你要不敢说出去，那你这纯粹没啥意思，对吧？以后我觉得基金委改革其实有个方向，就是你就告诉（评审人是谁），凭什么就不能说出来这个评审意见的作者是谁呀？有什么怕的？我说你这个本子写的不好，我有理有节有据的，我怕你找我啊？你就写的不好啊，对吧？而且我给你指出来帮你了，你还来找我，那不纯粹有病吗？

刘书博：因为现在这个评审会有一些···，比如说一个人会评二十多本，那这个时候比如说咱们这种制度设计是否有不合理的地方？

受访者：这个就牵扯到其实就说你为什么回来以后写本子不得要门，然后再举个例子，可能说不得诀窍，其实很重要的一个原因是什么呢，就是因为···其实我也是，因为我首先换了个专业，并且我在国外读的博，就你没有机会接触本子，说不定她俩比你接触的本子还多，你们现在才博一，但是我就举个例子，很有可能就是你们俩将来接触的本子比他到现在为止接触的本子还多，就再过一年或两年，因为为什么？因为一个老师给你20个本子审，那一个本子才十块钱。举个例子就十块钱，那我凭什么花那么长时间给你，我可能就分给学生，你给我写几个意见就得了，那从老师角度省了工作量，但从学生角度实际上他学习了怎么写本子，当然有可能负责、也有可能不负责去评审，那就为什么很多时候说国内毕业的博士生或者你们其实在写本的时候是有优势的，因为你至少见过呀，你看我来的时候我就没见过本子，对吧？或者我见过的本子都是做工程学的或者信息口的，那你说怎么能一样，你看他可能都没见过本子，他写的时候第一年写可能都没见过，到现在他都没见过几个本子，到现在为止

刘书博：就是同事的本

受访者：就同事的，你能见过十个？

刘书博：到不了

受访者：你看他都来了这几年了，差不多7、8年了，5、6年了，总共才见了十个本子不到，说不定再过两年你们一人都能看十个本子

刘书博：所以也是这个本子它有一个标准化的，而且是比较清晰的这种···

受访者：它其实是有套路的，只是你作为一直在国外读硕士、读博士的老师，你不知道这个套路

刘书博：所以这个我就想他就有点像八股，那这个套路···

受访者：很明显它是有八股，它是有套路的，它就是虽然不能用八股来定义，但是确实是有套路的

刘书博：就是有时候这个本子呀，它是对于未来的一种计划，然后现在呢我们感觉就是它甚至比一个paper还要重要

受访者：它就是很重要，因为你有化肥呀，你没有化肥种不出种子呀

刘书博：但是其实我们应该是把成果看得更加重要，然后就是套路反而没有这个实质重要，但是现在似乎就是本子比成果重要

受访者：因为本子重要，所以套路就很重要

刘书博：对对

吕梦娜：所以说其实可以理解为这个过程的管理，让人把对最终结果的注意力转到过程上。其实我个人的话，就是我知道后面三年我将要面临什么，就是可能每三个月大概一次考核之类的，这就会让我觉得···其实我现在就是如果从功利角度来说，我可能最重要的是达到毕业要求、然后是博士毕业论文，但是这些考核会让我觉得很筋疲力尽，就是现在我刚刚入学第一天，我就有种感觉就是，哇塞，我后面每三个月我就要绞尽脑汁应付一下检查

刘书博：因为过称的环节太多了

吕梦娜：对，我就很疲惫

受访者：你是在国内读过硕士吗

吕梦娜：我在咱们学校读硕博连读，就读了两年

受访者：你已经读了两年了？那你这个应该很熟悉了，我以为你是刚开始。你是呢？

黄秋莉：我也是硕博

受访者：你也是在这里读两年了？

黄秋莉：三年了

受访者：已经读三年转了博，是吧？

黄秋莉：对

受访者：那你对这个套路就很熟悉了，因为你有过这个硕士经历

吕梦娜：但是套路归套路，就跟基金似的，基金就放在那儿，你不可能不去申请，就是这个化肥你不要的话就好像···

受访者：其实你是可以不用申请，你看他刚才说的那个意思，那个书博说的，其实你是可以不申请，因为对我而言其实基金没有任何用，就如果咱就是纯科学的角度，你说基金对我的帮助是有，因为没有基金，我没有化肥，我一个化肥都没有，对吧？但是我真的有那么迫切需要那24万的基金吗？或者说24万基金真的能产生一个正向的这一个线性关系还是一个指数性关系？因为我比较做数学嘛，就是你这个曲线是一个指数型的，还是是一个log型的，还是一个线性的？那其实是要打问号的。当然我相信基金委的同事或者国家是希望我给你基金，我给你化肥了，你应该成一个指数型的增长，你的成果，对吧，但是可能有些时候会变成线性，甚至很多时候会变成log型的，对吧？那就你要真正去考虑的问题了

刘书博：对，而且学科差异性很大，你像爱因斯坦研究的理论物理，他就是不做实验的，他要那么多钱干嘛？

受访者：对，但是你说他缺钱也不行，因为他也得有工作也得···，但是就是说我们不说这个基金一点儿用没有或者说用处很少，而是说就是说你需要有基金，但这个基金不一定非得说搞得是我没有基金不行，对吧？他不是个矛盾的问题

刘书博：现在有一个政策，当时提出来一个让大家议的一个稿子，就是学校科研处还是别的处，他说凭正高必须得有国家级项目，他是必须，所以这个就有很多反弹意见，但是他们为什么会有这个东西出来？其实我就说这个异化，就这化肥必须得有，不管你需不需要，不管你是做理论哲学还是做实践实验，他不管了，我觉得这就是中国现在科研管理的一个特点

受访者：这个其实我觉得怎么说呢？就是看你个人的这个具体的思想或者想法，但是就说，就是化肥真的是很重要，真的是很重要，确实是很重要，但是还是种子加成果更重要。对吧？

刘书博：对对，因为我觉得如果是申请化肥让这个农民精疲力尽的，没有本来应该种田的这种精力，反而去搞化肥上，这个制度就有问题了，其实我在回国之后，我就觉得我们充斥着这种制度，就大家就忘记了初衷是什么？就谈到这个标准

受访者：所以我跟你说你真的题目可以写。那天我还给别人发邮件，我说这个不忘初心，这个我还真去找了翻译，对吧，就是你怎么翻译这个你的初心？这个我觉得放在题目上其实还蛮有意思。

刘书博：这个就是韦伯批判的那个官僚体制的一个弊病，因为大家就变成了一个不问目的的，只知道执行的一个机器人

受访者：这个制度或者组织可能慢慢的都会变成这样，尤其组织变大的时候，就组织变大的情况下，对于每个个人而言最保险的方法就是不求其他的东西、只是执行而已，因为这是最保险的，因为你不会出错，对吧？你risk最小，对吧？咱们说你虽然当不了大官，但你至少不会被人打下去

刘书博：但是你看这个是适合那种就政府官僚和制造业，但是我们是学术的···

受访者：但是你的钱是来自政府，咱是纵向基金，是政府支持你做科研

刘书博：而且好像我们更加重视的是纵向的，而不是横向

受访者：我们就是重视纵向的，咱们这个国内就是特别特别注重纵向，但是你想说那个横向其实跟在国外的横向呢也不是一样的，它两个横向不是一个概念的，比如说在国外的横向当然也不影响教授在外赚钱，这教授肯定在外面赚钱，这是很正常的，但是它毕竟是一个社会服务的东西，但很多时候国内的横向会变成什么呢？会变成一个利益交换，或者说学校要收管理费的这个过程，我不知道咱们学校的横向管理费多少，20%？反正少不了这个数，对吧？

刘书博：30%

受访者：30%，好，咱就不说哪个学校了，你就以20%为例子，你也知道做实业，咱们国家鼓励做实业，你的实业的利润是多少？零售利润2%，然后这个普通超市就3%的净利润，对吧？那你现在还要扣20%的管理费，对吧？那你就跟你假设你说我做一个行业项目为了解决社会问题，我一分钱不赚，那一分钱不赚的前提也要保证你先有20%利润才行，你没有20%利润，你交上20%管理费，那不就是倒贴20%吗？对吧？

刘书博：所以我就不知道横向定这么高是谁拍脑袋弄出来

受访者：不知道，所以说我也没做过横项嘛，那你没必要立项嘛，你干嘛立项，你为了解决社会问题嘛

刘书博：所以我就又看到，我们一方面是提倡，一方面在制度上又是打击

受访者：但是他知道你有诉求，因为他从立项的、从基金的kpi的角度上，他又要求你有基金，他管你横纵呢？这意思说你要不从国家拿20%管理费，要不你自己交20%，对吧？

刘书博：那这个就涉及kpi，就是这个学校它越来越像公司的那种

受访者：那人力最流行的概念不就是kpi嘛，对吧？OKR嘛，你ok一下嘛，对吧？

刘书博：OKR是从下至上，好像就像咱们这个签合同，跟你商量着来

吕梦娜：这个O是objective，K是key, R是results，就是目标关键成果法，我觉得他这个意思应该就是刚刚刘老师说的这种，就是跟你商量来

受访者：目标关键成果法，那意思是不需要化肥呗，那不需要化肥吗？我只要目标跟关键成果吗？

刘书博：他说你们这个量化的指标不是别人直接扔给你的，而是说你自己觉得应该是什么，然后再给上头沟通，商议出来

受访者：那就充分发挥下属的主观能动性

刘书博：对，但其实也是量化考核

受访者：也是量化考核，但只是说由下属来提

吕梦娜：我感觉跟MBO有点像，MBO是从上往下往下定，然后OKR可能从下往上，然后稍微人性化一点

受访者：对，就略微人性化一点，那其实也很好了，总比你kpi定指标说···我今天就遇到一个事儿，今天我去麦当劳点餐，他非让我扫外卖码，我一看我说我在堂食我点外卖干嘛？他就说你这个没事儿，你想要啥，我给你弄，外卖费你交上去，反正肯定比你正常点便宜。后来我猜肯定是领导跟他下达了每月外卖量的指标，那他没办法，他不可能真送那么多外卖，那就逼着所有的堂食变外卖呗，对吧？那我觉得这就是典型的kpi嘛，对吧？从上往下分解的嘛

刘书博：所以有这个反思就说，只要是量化考核肯定是有围绕这个量进行,这个作假呀等等。还有就是量化考核最大的问题就是，有很多本质性的东西很难去量化，重要的东西也很难去量化。如果一量化人们就冲着这些量去了，他会忽视掉一些本质重要，就好像咱们做科研，那本来就应该探索一些自己感兴趣的、社会重要相关的，但是一量化我们可能就像他这样子就被牵着鼻子走好发表、快发表的

受访者：所以说你像我原先教那个产品设计或者叫创新管理的时候，其实我不知道就是沃顿那个老师，其实强调的很重要一点，就为什么现在咱老是在搞创新大赛？大学生为什么要搞创新？就是想用非常小的成本获得更多的idea，你看我才给你多少钱，你一堆学生，100万的学生给我出idea多好，对吧？我总共才花了100万，我获得了100万idea，对吧？其实就跟研究似的，确实肯定很多研究者是平庸的，这是肯定的，正态分布嘛，对吧？肯定很多是平庸的，但是你即便给了平庸的人1万块钱你也没损失啥，你就损失1万块钱，但是你可能给一些真正有idea、有想法、有长远计划的人可能让他度过了最开始的难关，那这样的话很可能得到收益远远超过你给出的1万块钱，但是这样的话其实你要从管理者角度来说，他可能背负的最大的罪名是啥呢？就是国有资产流失，对吧？你给了一个平庸，你凭什么把钱给一个平庸的人

吕梦娜：他就不愿意冒这个风险

受访者：没有人会冒险，因为在制度体系下没有人会冒风险，只要你拍板儿你就得罪人，你就有事儿嘛，对吧？你愿意给全国人民发1万块钱吗？没有人愿意。

刘书博：所以其实这个做领导人他必须得得罪人

受访者：领导肯定是的，领导就是得罪人，哪有领导不得罪人的

刘书博：那也有嘛

受访者：没有，你谁都不得罪，就是谁都得罪

刘书博：也是哦

受访者：对，这肯定是这样的，领导肯定favor somebody，也肯定是unfavour somebody，这是英文里都有这个词

刘书博：而且领导有时候必须得对抗制度，对抗一些固化的标准，要不然他事情完全就是复制一些已有的标准

受访者：我知道研究leadership，那至少从这个平民百姓的视角，我也不看你们的论文，但我是从平民百姓的视角来看，那是不是有一个地方就是有担当，我猜有担当就可能有一部分意思就是说你得对抗某些带来的后果或什么东西，对吧？那我不知道你担当你们专业里都研究啥，什么叫有担当？我也不知道你们leadership里担当用英文怎么说

吕梦娜：我们不说担当，我们就说各种领导类型

受访者：那哪种领导类型有担当，我就想问有担当是啥意思？

刘书博：有胆识

受访者：不是

刘书博：integrity

受访者：integrity也不是、也不对，就是测量了嘛

吕梦娜：对，我第一反应就是找量表，但这可能就是还没有开发出来

受访者：那就看出来问题了，那就是说其实研究跟管理不落地。这有担当是中国人说了几千年的话，我们发了1万篇文章，也没有一个人和一个概念来说到底中国特色的有担当到底是什么？

吕梦娜：对。

刘书博：好，那我们再回到刚才这个主题，就是你来到这个组织之后，或者来到中国的这个学术界工作之后，还有什么？

受访者：我想想，一个是442，然后像刚回来的时候一个442，另一个就是communication，刚才说的用微信而不用邮件这种形式，这其实冲击非常大，因为用我记得我印象刚开始用微信，哇，那就是我到现在为止我微信很少删除，就你比如说长按一个人可以把聊天记录删除，就是我从来没敢删过任何一个件事儿，对吧？

刘书博：邮件你不怕？

受访者：邮件不怕，而且说我也不在乎邮件，因为邮件都存在网上去的，对吧？就算我不删除我也不影响，因为我手机是有内存的，我手机是流量的，你知道我换手机想第一件事是什么？

吕梦娜：复制微信

受访者：对，一定要复制，我恨不得我守着那两个小时看两个手机之间复制，因为我这万一出来任何问题，你就丧失了之前所有的记录，那这个其实就是一个很大的冲击，那你在国外我就登录过邮箱嘛，对吧？我而且我也不用着急reply你的东西，对吧？那这个冲击其实是除了工作量以外的，这其实有点儿像候鸟回回家以后的那种叫什么，就这文化冲击了，其实按理说这不应该叫文化冲击，我中国人我有什么冲击，但其实还真有这个冲击，很多人都是这样子，而且现在有了微信以后，你会发现你时间的关系就会越来越淡，比如说我晚上发个邮件，我我即便看到了，我也可以选择不看到，对吧？但微信不行，我看到了你大概率当时或者当天你也不可能不回复，谁不知道你晚上睡觉前肯定玩手机，没有人晚上睡觉的时候会打开电脑刷一下邮件，即便你刷了，人家不可能需要假定你去刷，但如果你当天不回微信，那人就知道你肯定有问题，哪有人当天不回微信

刘书博：看到了故意不回

受访者：那就肯定是看到了不回，对吧？从大概率大数据角度你就知道没有人四小时不刷一次手机的，对吧？如果我发个消息四小时不回，那我觉得大概率你肯定是故意的

刘书博：那这个其实跟中国的公司是一样的

受访者：对，那就是一个问题，

刘书博：国外的公司、国外的学校他们都没有这么好的微信这样的技术，钉钉这样的技术，咱们是有的

受访者：我觉得国外是有的，但是国外没有这么去做，对吧？这就是work-life balance嘛，我下班了就是我的时间，我可以选择回复，我可以选择当时回我可以选择明天回复，那只要是我没有影响你第二天8点上班儿，那理论上我就不算晚的，对吧？即便影响你8点上班儿了，那我第二天我有我的事情，我不可能你发邮件，我立刻就回复，对吧？

刘书博：所以这个就反映出来就是，西方他们，还是不说美国吧，美国可能也比较疯狂，但是澳洲和欧洲他们大家还是会有这种警惕感，就不要说工作和生活就···

受访者：就很不同，而且你想想就不用说别的，就咱不说微信，就说邮件，我觉得我记得我在国外的时候，因为你写邮件其实有一个选项的，是可以要求对方写回执的，就说已阅回执，我在国外都很少用这个，因为我觉得用的这个就是一个很rude的一个行为

刘书博：就强制别人要按照你的这个···

受访者：想法来做，除非你这个事很紧急，那确实是，但是正常情况下你任何工作邮件，没有任何理由要求别人看到邮件告诉你他看到了。对吧？那你想想现在钉钉做的是啥事儿，是吧？已阅，对吧？那就看到了，对吧？你这就是一个问题

刘书博：咱们现在群里也有，就是微信群也是一个通知说“已阅”

受访者：对，

刘书博：这个我觉得就有点多余，有一些老师会觉得很···

受访者：这个东西就是其实我觉得包括我上课也跟学生说，其实做人做成年人最重要的一个特点，也不是说你做多大多好多坏，重要的事就是你做的任何决定，你得承担责任嘛，对吧？你可以说我发了通知，你看到或没看到，whatever，你出事儿你负责就行了，对吧？比如说我让你汇报这个出京的事情，你看到了，但你没有点已阅，或whatever，但是你知道，你又出了问题，你带了病毒回来了或者什么情况，那你负责，这很简单，你负责嘛，把你关着、抓监狱，然后开除公职都可以嘛，对吧？你只要你能负责，你别给我哇哇叫，说当时发通知也没说让我写“已阅”，对吧？

刘书博：所以咱们现在其实从上到下学生也是这样，那学生到国外就独立的，你几点晚上回来不管的

受访者：不管

刘书博：咱们这儿给你安排地跟爸爸妈妈似的，然后查寝、卫生什么的，我觉得这个就是我们的文化，他有那个关怀伦理，他有点越界。

受访者： 昨天新闻说是大学生婚前性行为要受处分，我觉得这就有点儿离奇。你一个学生过了18岁，他有能力承担责任，而且没有出现任何伦理和任何法律问题的情况下，作为一个学校凭什么禁止？

刘书博： 从微信到这样的政策，其实我们社会不太尊重个人的这种独立性和自主性，他就把你裹挟进来，你必须按照规则来的这种感觉。

我们这个地方的聚焦点在于我们抓的很紧，然后信任感比较缺失，感觉更像一个行政的、流程化的、工厂似得。就是泰勒的模式、去人化、标准化。

受访者： 另外我觉得微信对我的冲击非常大，我觉得教学：科研：社会服务为4：4：2的时间安排可能还好一点，即便原先出国之前用微信用了很多，但是现在工作之后又是一种另外的感觉。

刘书博： 关于微信我再补充一下，我觉得特别不爽的一点在于，这个基金升到之后，微信群里排队点赞，从头天晚上到第二天下午一直都是这种点赞，像一大波僵尸似的。

受访者：如果要去对比微信的影响，我觉得还真要去仔细对比即时通信跟延迟通信之间到底有什么本质的区别，尤其在workplace，在工作环境中影响非常非常大。

刘书博： 就是一个组织氛围产生的一个平台嘛，我就觉得我的同事们似乎都在做一些机械性的工作，你看咱们学院没有任何关于学术观点的讨论，没有任何发表论文，甚至是没有针对社会现象用各自专业知识进行评价，都没有，完全是一种行政单位的那种冷冰冰的，然后虚假假的和冷冰冰，然后就是那种标准的上传下达，下面就是鼓掌，感觉大家都戴着面具，所以我觉得这种学术他就缺乏一种真实性。

受访者： 好像之前有篇文章讲这个事，就是说中国很多学术是缺乏争论，要不就真，要不是伪，不存在模糊地带。像实证经常会出现相反的结果，比如说他写了一个结果，你反对他，然后人家回你，你再回一个，有这么个往返的过程，即便发四五篇文章只是为了更坚持你的观点。我们现在做的研究更多的是一种整合，就是你说的对，我说的好，大家都对，然后整合到一起去了，少有人站出来说我更倾向于别的看法。那这其实就是一个问题，因为理论上肯定不可能的，因为你想R^2一般不会接近1，那意味着还有一些变异无法由回归关系解释，那意味着理论上还有足够的空间给你去争论的。但是很多时候我们不去做这个争论，而是做填坑式的，你那有1.5%，那我这里加了个因素变成2.5%，那这样就发展不出来theory，因为那属于补补丁的形式，但真正的theory实际上应该是对抗性的，它肯定是一个互相取代，即便是螺旋增加，它也肯定有个取代过程，而不仅仅是一个互相补充。

刘书博：除了微信，还有没有别的差异？我们可以谈到具体人的这种想法呀等。

受访者：还有一个你说的基金，这种对于过程的控制。

受访者： 另一个我觉得是对休息日的这个定义，当然这可能也是微信的一个衍生，大家都说羡慕大学老师有寒暑假，其实大学老师还真的不是从7月～9月、12月~1月的寒暑假。我不是说做科研，科研是你自己的选择。

其实你会发现有很多事务性的活儿来让你觉得寒暑假也没有明显的那么长。但是理论上比如说国外我们的老师，那学校结束了，除非他愿意去开会，否则他应该跟学校是脱节的，就是他差不多有一两个月是跟学校完全脱节，没有任何邮件通知，甚至都收不到学校邮件，那学校也没人值班，这是这个大方面，再往小一点就是周末很少会收到邮件。连我跟我导师交流的时候，如果他周末看了我的文件他可能都特意不在周末发给我，他要等到周一上班的时间延迟发给我，因为他知道他发给我意味着我就得改，因为学生都有个特色，都是看到就要改嘛，你也不好意思，但其实他会特意延伸到工作日去。那这样的话给你的印象就说我可能工作日要干活，但给你足够的时间休息。那反过来我给他发邮件，我也尽量不在周六、周日给他发邮件，除非特别着急。这意味着你给别人空间让人休息，去体谅别人，你会考虑到别人是不是在休息，因为本来老外就有礼拜的这个事情，那咱们国内这边的或者我了解别的学校，基本上对这个休息的概念其实没有明显的界定，就是工作和生活的边界很模糊。

刘书博：我听说像美国一些华人夫妻学者，他们几乎吃早饭的时候就开始讨论学术问题。

受访者： 那是个人选择，但是制度给人休息的空间，不会要求他们这样。我从不在周六周日给我导师发邮件，因为我特意考虑到他周六周日即便有时间理论上也不应该思考我发的内容。

刘书博： 制度分成三个方面来看，第一个是文件，即法律规章，咱们文件规定有周末，但其实到行规层面咱们就不一样了，价值观、文化意识形态等会特别强调奋进或者刻苦读书。

 如果某人在周末一直工作，他会有一种很骄傲的姿态告诉别人或者告诉学生：看我很辛苦勤奋，你们要按照我这样去做。

受访者： 这个在国外华人圈很明显。我接触的有些华人老师，就恨不得你晚上10点多给他发邮件，老师晚上1:00～2:00给你回邮件。

乘风破浪的姐姐里有个叫张萌发的微博，她就喜欢晚上2点多给员工回邮件，对吧？从一个角度说，她作为老板要宣扬这种刻苦努力的创业精神，但是从另一个角度来说，在学术圈里你不应该这样子，因为你学习刻苦，那是你个人的选择，不过你不能要求别人，你的下属或你的学生都要睡觉，这很重要的一点。而且更重要的是并不是说你强制要求别人晚上1:00睡觉，而是你给别人造成了一种无形的社交压力，如果老师晚上1:00都没睡，你还凭什么12点就睡了？我们说软刀子比硬刀子还狠，这种老师给学生的压力氛围比硬着说你必须11点才能打卡睡觉还要狠，对吧？这种压力太大了。

刘书博： 造成了一种恐怖的氛围。

受访者： 这种本身是自我要求，变相会变成对你下属的要求。现实生活中就这样，好多领导说我2:00回邮件，我都没睡你凭什么睡？

刘书博： 其实好多中国人对于这种事儿他不敏感。

受访者： 就你说那个早上吃完饭跟老公两个人一起讨论论文，那是个人偏好，但是不应该把这个去“强加”给他的学生或者他的合作者。这种“强加”也不是明面上的硬性规定，就跟PUA似得，也不是天天骂你让你觉得自卑，而是通过一些隐蔽的软性的方式让你觉得自卑。

刘书博： 我觉得中国好多那种工作狂，但是他会觉得自己是站在道德制高点上，他会鄙视你，他会觉得你没有我这样就好像你没有我优秀，他觉得自己很卓越。

受访者： 你个人选择那是你愿意，但你不能以此来要求别人。

刘书博： 对，不能以此为傲，然后到处宣扬。其实我们国家一些英雄人物的宣传，包括好多医生一下连续工作四十多个小时，然后累倒了，我们会宣扬，我们会觉得这个事儿，会觉得这个医生好了不起。但是我们看不到这背后为什么没有制度来保护他们，你难道不觉得四十多小时的工作时间对于他自己和对病人来说都是不负责吗？我们不会这样去想。

受访者：现在新冠大家都知道自我隔离，但其实在新冠发生之前，大部分中国人在国外会觉着我感冒去lab是一件很骄傲的事情：我感冒了，我还勤奋做事，但其实这也会给别人带来危险。

反而很多外国学生会觉得我感冒，我就应该在家休息，我没有觉得休息有什么不好的地方，我不去学校，我没去做科研，他反而觉得是为别人的安全负责。

受访者：文科不需要坐实验室，在工科坐实验室，尤其是打卡那种，如果说我感冒不来学校，你首先得请假；其次大家会觉得你不就感冒了吗？你感冒就不能来学校呢？不能写论文呢？不能看文章呢？

刘书博： 从深层次来说，我觉得还是西方他们更人本、要更关注人的感受和人的苦难。

受访者： 其实还是要考虑一下别人，就是你可以做任何选择，但前提是你不能去影响别人过自己想要的生活。

还有一点，我觉得是学术工作也是需要休息和休闲的。就像你说的那对夫妻也仅仅是这么多里边的一个，无论你是男性、女性，正常的人都需要生活，你需要有家庭生活、有自己的社交圈。学习和科研对大部分人来说，我觉得不是生命的全部，对吧？而且即便它是生命的全部，你应该把它拉长到100岁这个年龄来看。你首先得保障身体健康，才能更好的工作。即便你想把生命奉献给科研，那也希望你奉献的时间更长一点，我希望你奉献60年，而不是奉献十年乘以24小时，对吧？ 这是两个概念。

刘书博： 我觉得我在休闲的时候会有灵感，我会想要创作，但是一忙我就没写作的欲望了，就对比上课一多我就完全不想写了，静下来后更想要创作。

受访者：这就是为什么有人说，读博其实是最轻松的。当然你们现在刚开始读博，我在读博的时候也觉得读博是最累的，这么多年的时间其实就干这么一点儿事儿。但当你毕业工作以后就会觉得读博真的好轻松。

我现在特别怀念，我读博的时候只考虑写论文就行了，延期一年就延期嘛，其实也没什么，现在工作后教学、科研、社会服务都要做，教学和社会服务工作又比较多，然后我还要保证我的科研，还得加上事务性的事、基金的事情，还有好多事，就是你会发现你工作后还不如读博的时候轻松，那时候顶多家庭跟学习，现在是家庭、学习、工作，而且边界还不一定那么清楚。

刘书博： 那你说的是在澳洲读博，在中财读博就另外一个景象。他们也是有各种不同的考核。

受访者： 那也轻松，你想你需要教课吗？你会去干别的活儿吗？换句话说，你的博士津贴是基于你的performance吗？它不是kpi驱动的，比如说考核没过，你可能会延期，但是你的博士津贴没有跟工作挂钩。挂钩跟不挂钩是完全不是概念。你现在三个月考核只是影响你毕业时间，并不影响你每个月拿到的收入。

刘书博： 那咱们是和kpi挂钩的。

受访者： 咱们是明显的挂钩，基金等都是跟你的salary直接相关的，这其实是把物质奖励过于给放大了，

刘书博： 其实就是公司管理的模式，就是这种市场机制在管理着人们的学术动机。所以我们不再是想要研究一个好奇心的问题，去学术工作，更多是想要生活，想要赚钱。

受访者： 有一篇文章说有趣的的不那么重要，重要的是有意义的事情。其实我个人觉得，如果研究者是integrity的，我觉得他判断是有趣的问题肯定也是有意义的。就是说只要研究者本心是integrity，他有正直的想法的话，他如果判断这个问题值得研究，有意思研究，那这个问题肯定是重要的。“有趣的的不那么重要”这话的前提是指有的研究者纯粹为了有意思而不是为了研究，这个内心没有任何principle的情况下，他就说这个东西好像没人做过，所以我去做个简单的填空。

刘书博： 他们总是会强调两个词，一个是integrity，一个是dignity。

受访者： 对，integrity就是我要有自己的principle，我对学术有一个严谨判断的情况下，那我认为它有意思，值得研究，那肯定是有意义的，因为我用我的理念进行过了一次判断。dignity就是尊严、体面，这个跟钱没关系，我穿普通的衣服，但我只要干干净净的，我内心就强大，我认为就是可以的。

刘书博： 有点像电影“无问西东”里面说的一个词：笃定，就是说他看到当时那些学者眼中很笃定，就是因为他们过得很体面。他们不会说，今年考核我好像没达到标准，有点唯唯诺诺的，或者是那种焦虑的，他就很笃定。因为他们知道自己该做什么，然后他们可以去表达出来自己的学术志向，但现在我觉得这种笃定就越来越少。

受访者：电影“无问西东”背景其实是发生在这抗日战争时期的西南联大，那其实那时候，他们可能水都不见得能喝得上，但是人就是有自己的dignity，有这个独立人格。

Integrity、dignity绝对是西方学者使用的高频词。这两个词你可以套到任何场合上，就跟扣帽子似的，在任何场合你都可以说：你这个人要有integrity，你这个人要有dignity。

我觉得这两个词就跟中国人“慎独”的理念是一致的，不要求你成为完人，但是我希望你有自己的原则,只要这个原则是合理合法合情的。比如说我愿意在宿舍里边，你只要不违法，不违反社会原则就行。那别人就想周末休息，人家就是这样的生活节奏，人就觉得这是我的principle，我需要七天里面分一天给家庭，那你你就不应该去破坏别人的生活习惯，而且正常的在社会环境下，周六日本来就是休息时间，人拿出一天休息，有什么问题呢？

刘书博： 我们就缺乏这种尊敬，缺乏这种体面的生活。

受访者： 我老板每他每周工作五天半，就是前五天加周日下午，他周六是要休息，就是啥都不干，在家躺着，跟孩子在一起，他什么也不管，然后周日上午做礼拜，他肯定不在电脑旁边，人家就是这种生活节奏。

也没有人周六给他发邮件说你今天能不能给我回复一下，或者说你明天能不能回复，人家都不理你。人家就觉得我凭什么要回复你，我已经下班儿了，你只发40小时的工资，凭什么让我干80个小时的活儿？

刘书博： 国外学校会不会像咱们学校建设“双一流”等，类似我们的学科建设等？

受访者： 我们学校学英国，英国有科研绩效评价体系叫卓越框架（REF），REF也是一个科研导向，但是更多的是说鼓励你去多做点研究，而不是说你不做我会对你进行惩罚。这是两个概念，他还是希望起到引导作用，让你去做的越来越好，而不是说你不做到那么好，我就换一个新人。

刘书博：所以我觉得，欧洲、澳洲跟美国还不一样，美国就有点太福特主义。然后中国好像比美国还要更加明显。

受访者： 可能咱们现在要超越嘛，想弯道超车，或者换道超车。只能说这种事情是现实存在的，我觉得对于社会或者组织而言，合理的方法也不需要避讳，就不是说中国一定好，外国一定差，没有说谁好谁不好，而是说既然有这么个现实情况，那我们就应该考虑在现实情况下怎么去把它做的更好。比如说我们考虑到我们都习惯微信这种即时通信方式，那能不能不太紧急的事件还是采用发邮件的方式？就是要区分场合、充分考虑到现实情况以及每个人的利益。不过话说回来这个对管理的压力就很大，所以要充分考量各种情况。

刘书博： 除了刚才讨论的这些，还有其他方面吗？

受访者： 还有一个很浅显的东西就是工资，你之前发的“非升即走”那篇文章提到一个transparent（透明度）的问题。像国外，人家告诉你多少多少工资，文件都公开的，税率都是定死的。

学校招人的时候就写的清清楚楚，而且人家工会跟学校谈判，人有这个专门的collective，通过共同谈判产生共同同意书，就说工资应该怎么涨都说的很清楚。除非你不认可我的学术成就，多多少少扣一点，那这是没办法。反正大家都按标准来，劳工组织跟资方学校谈好一个文件，五年一谈或三年一谈标准就定死了。除非你说我不加入工会，你想单独跟学校谈，那也没问题，但是只要你加工会这标准就是一致的。

刘书博：所以这个也是中国特色，中国没有真正的工会。

受访者：国内很多时候，学校就是不够transparent的，现实得到的和预期得到的不一样。

刘书博： 我就想到像中大、武大他们对于年轻人的那种“非升即走”就非常……

受访者： 完全就是权力的对人的碾压，或者那纯粹就是一个生产线，人变成生产工具，关键问题是这个事情竟然没有任何反响，这是个有意思的事情。说明我们这个年轻博士就是不值钱，除了复旦杀人以外有啥反响？中山大学还继续这么招人，没造成什么区别，你听说他改变了吗？就被人骂成狗，然后呢？so what？对吧？你骂人家，人家也还这样子/

刘书博： 对，其实比较讽刺的就是，咱们是社会主义国家，但似乎资本主义国家的工会更强，我们没有这个。

受访者：人力资源管理课程，估计这一章都不讲。

确实就是这个问题，中山大学跟武汉大学到目前为止没有什么改变，你会发现他还是这样。这个东西很好验证，你去搜一下中山大学招聘通告，就看他每年进人计划就行了，无论实际进了多少人，他竟然写这个招聘计划，说明内部已经同意今年应该进那么多人，那这已经反映情况了，你不用管他留下多少，因为留下多少肯定是一个基本的比例。比如说厦门大学的管理学院，他比我们大一点儿，但是你知道厦门大学去年和前年每年招差不多六七个助理教授，四五个副教授，然后两三个正教授，每年都这样子，那肯定不正常，怎么可能？这就相当于80个人的一个院，你每年就进20个人，财务里面有周转率嘛，那四年就有1/4的人被辞退。

刘书博： 他们就非常公司化。

受访者： 甚至是餐饮行业的，人员1/4的周转率就已经很高了，虽然物流专业或运营专业是追求高周转率的，因为我们不希望货物在仓库里存时间太长，但其实你希望员工在你的组织里待的时间长一些，货物跟人是不一样的，对吧？

现在你每年进20个，每年进1/4的人员，也就是四年就翻新一波，除非你的管理学院扩张一倍，但是在整个体制不变的情况下，这就是unsustainable，是不可持续性的。

厦门大学在国外的名声很大，因为他是华侨建的学校，他与国外联系很多，中央财经大学可能在国内知名度可以，因为咱喜欢中央这两个字。外经贸可能在国外更有名，因为对外交流多嘛，然后中山大学即孙中山大学。

那你就看他们的进人计划，然后结束结合人力资源管理的概念进行理解，那其实就能发现问题，你都不用谈是研究导向还是什么导向，纯粹从人力资源管理来说，他就是不合理的，你去任何世界五百强公司，人员流转率都没有高达20%的。中财还没有出现这么明显的人员流动。

吕梦娜：那中财对新老师是那种达标赛还是锦标赛？

受访者：是锦标赛还是达标赛就取决于人员周转率，无论达标还是锦标，只要周转率不那么恐怖的，其实都是可容忍范围内的，不能简单判断说达标赛就轻松或者锦标赛就不轻松。

主要跟现实情况做对比，比如说博士将来要去找教职，只要说我们只有三四个人竞争一个岗位就还可以接受，组织可能也不希望太少的人竞争，但也不能1万个人过来竞争，中间肯定有一个比较合适的竞争值。

那同样在学校里也一样，你不能希望说以后没有任何压力，必须有岗位需求的，但是你也不能希望这个变成一个残忍的岗位。

刘书博：国内好像更加认可丛林法则。

受访者： 这个只能说肯定有人得利了嘛，我们是丛林法则之下的，我年薪制，（但有些人没有处在丛林法则之下），只要在这个赛道你就面临这个问题。但是我想强调的说，丛林法则不一定有问题，而是说不能过度，有一定竞争压力是好事儿。但是这个绝对是个倒u型曲线或者正u型曲线，你关键是要保持在合理范围之内，那中大肯定就属于把这个倒u型曲线用到一个最右边或者最左边去了，那这就是不合理的，那你从人力资源管理角度就得批判他，hr都忙死了，要面试那么多人。

刘书博： 面试我昨天也聊到过，就是他们尽量不怎么面，就大概看一下，然后你来就行了。

受访者： 对，他都不需要面试嘛，那这其实就弱化了人力资源管理的一个理念，你入口就有问题，你怎么保证你招的人是合理的？

刘书博： 就看你最后达标没。

吕梦娜： 我室友做招人什么的，在咱们学校就数人头，就是拉微信群要求数量指标达成，然后下一个指标可能是转化率多少，然后再进一步。

刘书博：所以还是回到kpi，他肯定会围绕这个量化数据。

受访者：我今天不就被人拉人头了嘛？去麦当劳买个东西，非得让我点外卖，明明我是坐在那儿吃的。

刘书博： 这个也是中国特色，就是我们并没有形成一个同等权利状态下的对话。

受访者： 因为它我们还是传统下的上下级关系，然后加上供需匹配已经扭曲了。

刘书博： 对，我们的博士太多了。

受访者：本来供需匹配是存在竞争，这没问题，博士多就应该竞争，但是现在这个竞争已经变得变态了。就是说我们不应该否认有竞争，不是说博士一定要有岗位，而是说竞争一定要适度。

刘书博： 我们博士扩招，也没有考虑到需求量。

受访者： 那就是更强烈的扭曲了嘛。

刘书博： 博士数量大量的增长，我们来之前讨论过，就是学校也的确在赶超，然后他怎么评价学校发展，就只看量。

受访者： 这就是问题，那博士其实跟青年研究员一样嘛，招青年研究员是为了提高文章产量，然后博士发文章不也一样吗？

黄秋莉： 从我们这一届开始，我们学院好像开始加大对博士生的培养，以前好像说没怎么重视博士这一块，招的好多都是在职的博士，我想着这是不是也是一种什么方面的倾向？

刘书博： 就结果导向，反正让你们尽量多发文章，这个也是学院发展是否进步的一个评量标准。

受访者： 有压力是挺正常的，但是不能把这个事儿过度化，就说你明年发不了十篇文章不让毕业，那我觉得就没有必要了嘛。像我们在国外，博士毕业明确规定是只要你导师觉得你能毕业了，你就能毕业了。那你会觉得这是不是太主观化了啊？导师的权力是不是过大了啊？但反而其实并没有，因为他相信他招来的老师是有他的自尊心、有他的政治性，他知道这个老师不会变态的要求这个学生发100篇文章才能毕业。那如果导师个人比较坏，永远不松口，你永远毕业不了，但其实这种事情发生的很少。

刘书博： 所以他们是有一个学术共同体的。

受访者： 就是导师对学生水平会有一个基本的判断，认为学生的水平跟整个community是一致的，那我觉得他就能毕业，我没必要特殊的给他加要求。这就是社会需要相信老师是正直的，他是有他的principle的，他是明白当前博士的要求是什么的，不需要你明确写出类似于abcd的那个list。现在不是提倡要给员工赋能吗？那这就是给员工赋能，凭什么不能把决定权交给老师？有博士学位，干了好几十年的人，对吧？你应该把权力交给他们。

刘书博： 那这个就还是回到关键词integrity。我觉得我们这边好像就没有，我们走答辩什么的好像很过场化的。

受访者： 比如说你跟着我读博，然后要求同学发不了十篇文章，千万不让他毕业，我觉得没必要。当然这要求老师的整体水平方差不能太大，要让人相信整体的教师水平，这是有一个大概的标准的，而不是说有人是禽兽，有人是根本连禽兽都不如，对吧？那这就导致方差太大。

如果我要说你一定要有三篇文章才能毕业，那就这有点本末倒置，本来员工能解决的事情，你非得从管理层从上往下来定这个规矩。所以说我一直觉得也存在另外一个问题，国外很多学生回来找工作就没有优势，因为老师觉得你能毕业就毕业，结果发现你就写了个专著。当然这是另一回事，但是实际上是说你要对你的员工有基本的信任，你要主动的赋能你的员工，对吧？你要相信你员工在现场解决问题的能力，而不是需要你从高层123456去安排这些环节。这种考核不仅是对老师的不信任，也是对学生的不信任。所以说就因为没有这个信任，才变成了一定要用kpi。当然肯定要强调对员工进行赋能。

刘书博： 所以不信任的结果就是不体面，大家相互都提防着，那这个社会也就缺乏一种体面，自己也不尊重自己。

受访者： 所以说有时候我挺困惑，因为我本来学工科，然后转到商科以后，我感受最大的一个变化就是我觉得人是有主观能动性的，因为我原先硕士做的都是管机器，因为这个设备是没有思想的，我记得当时为什么出去读博，我就跟老师说，我说我不想管设备，我觉得设备好无聊，都冷冰冰的，我就直接用cold的这个词儿，因为我英文也不好，我说人至少是warm的，人跟机器有很大的区别，然后我就发现这个人是真的很fancy的，就是人是非常多样性的。我记得我当时在国外读博，我当时看到一个人力资源管理概念，对我冲击很大，你觉得肯定很好笑，因为这太基本了，就讲工作的有五个维度，包括工作自由度等，这就是说这个人是有自主性的。你现在想会觉得很傻，这不废话吗？你人肯定有自主性啊！但是你要想想，我当时是面对的机器，就是我让他干嘛，他就得干嘛，然后我发现人有自性，反正特别有意思。

刘书博： 所以他看《失控玩家》很有感觉。

吕梦娜：我本科的时候学人力，就是觉得人很有趣，可能跟老师刚刚您提到的想法一样，但是到研究生之后做那个研究时候，你会发现都把人当做理性的人来看待。

受访者：我现在做人的研究我都是分情况来看待，因为人都是不一样的。我现在有时候看人力资源管理的文章，我觉得他们也意识到这个问题，你看大牛孙老师写的是柔性人力资源管理综述，那柔性不是你们最基本的吗？这柔性不就是flexible，这都是几百年前的事情，但现在又提出来了，所以说人的自主性，这是很基本的概念，可是随着科学主义把人都看成一样的，就很冷冰冰的，所以现在开始有点儿反思。我现在就有点儿反古， 谁说training的目的一定是提升效率的？其实人是完全不一样的，每个人的容忍度等都不一样。

刘书博：我们学校编的教材，清华大学出版的那个，然后我还参编了，那个管理学对于管理的定义，也是那种福特主义的定义，偏效率主义。

受访者： 对，那你说效率主义你学啥？你还不是使用运营管理，运营管理全是管机器的，我们纯粹就是效率主义，运营管理就是效率，当然我们现在也强调别的维度，但是其本质上就是效率管理。

刘书博： 咱们其实透析出来就是咱们高校其实像工厂一样在被管理，人本来做学术的这种知识密集创作，是创造性的和探索导向、未来导向，现在反倒是被过去的标准给束缚起来。

受访者： 对，反正我现在觉得运营管理或者工业管理，我个人觉得没啥思想，我自己的想法就是越来复古，我从我开始读博，我就觉得人就是一个完全不一样的，我现在做的论文就是管人，我觉得人就很有意思，人太不一样了，任何一个不一样，拿到我论文里其实我个人觉得都是一个新点，可能别人觉得没啥意义，但是从我的principle来看，我觉得这是有意义的，并且能解决实际问题。

# 受访者29

刘书博：那么咱们今天的访谈就通过李老师，他一直是在这个商学院、清华经管学院，我们就想了解一下，李老师是从研究生阶段吧然后到读博士，然后现阶段呢是在中央财经大学在做这个科研教学工作，也是专职的老师，所以我们就通过你的视角去看一下商学院的环境、管理学的特色在中国是什么样子？

那我们就是以个人叙事的方式谈一下，从研究生开始，为什么选择读研究生？可能是个人志向还是什么？为什么选这个专业？

受访者：这个研究生指的是硕士还是博士？

刘书博：硕士吧，从硕士开始。

受访者：好，我高考的时候是在山东那边，高考之前呢家里边儿遇到点儿事情就耽误了比较宝贵的一段儿时间，所以高考的时候呢这个成绩发挥也不是太好，刚过一本线也没有多少，就选了一个985靠后一点的高校，但刚进学校的时候，其实我也给自己有一个定位，就是我不属于这个学校，我早晚要去一个更好一点的（学校），把原来的这个遗憾要弥补一下。那么在山东的学生也是比较能学、比较能考，所以本科的时候成绩还可以，一直保持是我们专业的第一名。然后后面到大三的时候，那个时候就有了高校开始这种自主招生，清华呢是第一批，恰好呢清华又创建了一个专业叫管理学硕士，他是把所有管理学专业，如市场营销、人力资源、战略管理等全部整合到一个专业里边，叫管理学硕士。

刚才还跟刘老师在说，今年清华的管理学硕士全球排名是第20，亚洲第一。当时我是第二届，正好是自主招生这种方式；当时我也是试着去投了个简历，那投这个简历呢主要还想是在学历上有一个提升，然后呢再看一看外面的世界，就多接触一点商学院的知识，这是最初的一个初衷。

很巧呢我也通过夏令营的方式也录取了，其实这个录取的机会还是挺难得的一个机会，那（我本科）学校那边呢当时都觉得这不太可能，因为我是我们（本科）学校第一个保送到清华经管的学生。我也就通过这样的一个方式到了清华经管这边读了硕士。

我硕士的专业是夏令营的时候决定的，我本科的专业就是工商管理，当然还有其他专业录取了，但是也都是大管理类的方向，因为我的方向还不想有一个太大的一个变动。

那读了这个硕士呢读了三年，那在这个读硕士的过程中，因为硕士的时候是自选导师嘛，选了一个导师，是年龄比较大的一个老先生，这也是在课上选的一个导师。后来涉及到一些个人的事情、还有一些课程的事情，在跟他讨论的时候，他会经常追问，就是以一种很严谨的（方式），因为他不知道这个事儿，但是他能从这个逻辑里面去推断，他能往下去很深入的去挖一些事情，他每次能把我问出来一身汗，我想这个还是有一些训练，或者是思维上的训练、需要提升的地方。

后来我跟老师聊一聊，他说你要不要再读个博士吧，我说要读就读吧，后面呢就硕士毕业了之后呢就跟着他读博士，博士是16年入学。16年入学的时候，本来我们那个系在12年的时候是叫“技术经济系”，技术经济系跟“战略管理系”是分开的，但是后来呢国家在12、13年的时候有这个双创。那双创这个事情呢就创新创业推到了业界的前沿，当时理论上呢也缺乏，也是需要做一些研究的。那跟这个最近的一个专业就是原来的技术经济，因为它是看一些大工程、大项目的，涉及到效益、涉及到优化的一些问题，跟创新创业关系比较大，所以清华就把技术经济系跟战略系合并了，合并成“创新创业与战略系”。那我一直呢就在这个专业里边儿就读，这是整个的一个研究生学习的一个情况。

刘书博：所以你刚才说你受到了当时研究生导师的一个这种所谓的冲击吧？

受访者：对。

刘书博：你觉得自己的思维是可以通过学习然后进行提升，是有这样的想法吗？

受访者：我当时肯定是有其他备选的方案，但是当时我因为这个榜样的力量是很大的，他就是我的一个榜样，那我问他，我说你这个事情你也没接触过，比如说一些业界的事情，那为什么我这么说你就能推断出来说这个企业下一步会是什么样方向，或者现在他的这个方向是不是合乎逻辑的？

他说呢，就他告诉我的说这是一个逻辑的训练，你要严谨，就是这种因果关系的推断的时候一定要一步一步地往前推，中间不要有跳跃、不要有断层才能得出了这样的一个关系，所以呢就这样的一个榜样的影响，我就觉得这是读博士是一种比较好的去训练你逻辑的一个方式，但因为也没有接触过其他备选的这个方案嘛，所以就把这个唯一的一个方案当成了一个选择。

刘书博：所以当时也没有想去就比如说先工作，到公司到社会上先实践

受访者：这个也考虑过，因为硕士的时候最后一年多基本上也都是在实习，也拿到了一些offer，但是呢当时是想如果说先工作的话可能要曲曲折折的，比如说假设能力、逻辑能力还真是一个在工作过程中必备的很重要的一个能力，如果现在不补齐这个短板，可能这几年的这个工作也不会有一个很好的表现，或者说会走比较多的弯路，那就一口气把这个短板先补齐之后再去工作吧，当时是这样的一个想法。

刘书博：所以当时你对于读博士来说，有没有更加长远的一个所谓志向性。

受访者：说实话当时并没有说我一定未来要做学术研究，或者是说在理论上在哪一个领域做出了多大的贡献，没有这么个清晰的想法。

刘书博：那当时对于未来的这种博士训练和学习有没有一些想法？你会不会想象他应该是什么样子。

受访者：因为我硕士跟博士在同一个院校，然后也是同一帮老师，但是他们之间授课的风格、讲述的内容、训练的目标是完全完全不一样的。

刘书博：那这个能详细讲一讲吗？

受访者：硕士的时候，因为我们那个管理硕士它是一个偏实践方向，就是偏应用方向的一个硕士项目，他很多的课程设置就完全是按照企业实践的那一套。

刘书博：所以你说的这个专业就是刚才说排名全球第20的那一个专业。

受访者：对，那这个专业呢它就是完全实践导向的一个硕士项目，他刚开始呢他招不到很好的学生，所以给他一个学术硕士的这样一个title，

刘书博：就是现在比较专业（硕士）···

受访者：对，那现在其实已经改成专业硕士了，管理硕士master in management，MIM。

刘书博：所以这个感觉好像是清华经管独有的，咱们有没有呢？

受访者：咱们没有。咱们没有这么大的改革的力度。那他这个改革当时就是你刚才还在聊钱颖一，这是钱颖一做了一个大的改革，他呢做了我们院长之后就跟业界走的比较近一些，他发现说，管人事的如果不懂财务，管营销的如果不了解研发，管战略的如果不懂运营，那这可能都是要出问题的，就太片面。那他改革的一个思路就是说你既然要做这个未来这个管理工作，就一定要有一个全面的一个了解，那所以才搞这样的一个硕士项目。

刘书博：钱颖一是你当年就跟他重合了时间，对吧？他做院长就是你正好也在

受访者：对，我去入学的时候包括夏令营，包括什么讲话都是他在。然后我快毕业的时候，应该是18年左右吧，他退休，我是2020年毕业。

刘书博：所以他是退休了，不是被换掉，就是年龄到了？

受访者：对他年龄也到了，然后呢他在外面也做了一堆的兼职，像什么西湖大学这一类的。

刘书博：你们对于这个院长有什么感觉

受访者：这个院长，嗯，怎么说呢？我先说说我刚进学院的时候的一些这种大的冲击吧，因为我们东北大学你也了解，就是一个很传统的工科院校，搞冶金，搞什么地址这一块儿的，但是到了清华经管之后，那立马感觉不一样了，其实当时我对我冲击还是挺大的。

第一就是这种有一词叫“高端大气上档次”，他特别国际化，然后呢什么事情都喜欢搞个大排场，然后就业导向这一块儿呢，我刚一入学的时候，感觉到一种无形的压力，大家都在投行、都在咨询公司实习，这就是传下来的一种···

刘书博：是指你们研究生梯队，还是也包括本科？

受访者：包括本科，包括研究生甚至部分的博士。就是我刚入学的时候，那我当时还想着说我以后要到实业界，无论是央企还是民企也好，要真正的做点事，但到那儿之后突然发现说大家都说这个金融好、咨询好，甚至有些人都逃了课，去金融、去咨询、去投行、去实习，这种事儿很常见，我们有些同学就是去了我们国家很高级别的top1的投行，当时为了在那儿实习，毕业论文都没有过，有些课都没有修完，但是（他们）不在乎。对他们来说就是能有这样的一个实习就有了一切。

刘书博：那他们是怎么解读这样的一个行为？

受访者：那他们其实有一些呢是本科就在那边（清华）就读，但是本科的时候呢没抓住这个机会，因为他是要一层一层才能到那个最top的那个机构去工作的，实习的时候比如说先从个小同行开始，然后再往上走，那他们也是受到上面一届一届的这种影响，告诉你说来这儿好、工资也多、然后工作环境也是那种高大上的，你这个圈子呢也都是这种高学历的圈子，其实这个我个人推测是间接地受到了院长非常大（加重语气）的影响。

刘书博：为什么？

受访者：因为他在美国他读的就是经济，然后呢他来到了国内之后，当时给清华经管的这个定位···当然这再往前推的话，就可能是原来朱镕基总理的一套想法。那当时呢，朱镕基总理当我们院长的时候，他是首任院长，那个时候呢他就说我们国家没有国际化的管理人才，包括视野也好，包括思路也好，在跟国际化在打交道的时候还是很吃亏的，特别是在加入世贸组织这个艰难的谈判的这个过程中，他也体会到了，说我们的法律也好，经济也好，管理也好，跟人家相差很远，因为他还是一个挺朴实的一个院长，他的目标呢是说我们要补这个短板，所以呢当时就这个挑人，他还是亲自把这个钱院长从美国挖回来的，那就挖了这么样的一个院长，想补齐这样的一个短板，但是这个院长来了之后呢，可能也是有一些他个人的想法在里面，那就把国际化这一块儿做的是，我个人觉得是有一些过了。

刘书博：那其实北大也这样？

受访者：北大比我们更早实施了这样的国际化战略或者是导向。那光华在更早的时候，原来张维为做院长的时候就是这样，他也是经济学家，所以呢北大跟清华的风格在这一方面还是很像。

刘书博：为什么一个经济学家到商学院做院长，就比如光华，这个你知道为什么吗？

受访者：这个我不太清楚具体的原因，因为我没了解过他的历史。那清华经管这边的原因就是刚才我说的，为了把经管学院的国际化这一块儿补齐，所以选了一个有国际视野的经济学家来做这样的一个院长。

刘书博：好，那我们再回到刚才你说的就是你入学之后你会觉得很受冲击，对吧？那当时你学的是一个非常实践导向，那些老师是比较偏实践的。然后呢你说有另外的一些老师风格，那我们能比如说这个冲击当然是高大上、有些国际化的，这是一个让你感觉到冲击的一个因素，还有别的吗？

受访者：除了这个之外呢就是这些老师讲的这些内容，因为本科大多是普及性的这个管理学的一些知识，但是在硕士上课的时候，老师研究的领域就非常的细致了，比如说我们有一个创新创业课，那当时一个老师就给我们讲手机的一个行业发展历史，他能细致到对于手机的每一个小的发展的这个方向，然后每一个阶段的变化的趋势以及未来的判断，就是从来不看教材，什么都没有，就一个人从前讲到后讲了两个小时、三个小时都没有问题，那他不仅仅是对这一个领域熟悉，对其他的任何一个行业，就他所熟悉的这些行业讲起来都是能让企业的这些负责人听起来都觉得说我需要补这一部分东西的，所以就是在业界这一块儿，那些老师做的是非常非常的专业。

刘书博：ok，所以还是实践导向的老师，你会觉得他的专业能力是非常令人印象深刻的？

受访者：对

刘书博：ok，那还有其他的让你印象深刻的···

受访者：冲击这一块儿是吧？

刘书博：嗯嗯

受访者：还有一个呢就是整个的这种氛围，因为当时这些同学来自天南海北的都有。我们班五十多个人，全世界应该有十几个国家的人都有了，那在这样一个氛围之下，并且他们之前也都相当的聪明、成绩很好，竞争非常的大，那这种竞争呢他不仅仅是说我考试成绩的竞争，或者是一些这个实习谁的好谁的不好的一些竞争，就整体的这种氛围就导致大家说我们相互之间要瞄着一个最好的方向，比如说金融方向、咨询方向，都一块儿往那去。那在这样的一个氛围之下，你本来就受到了冲击了，并且呢冲击之下还有这么大的一个压力，就很快的沿着这个道路哗哗的往前走，就基本上不会有那么多的想法说，我再看看别的路。很少。

刘书博：所以就是你进入这个环境很快就被这个环境所同化了，

受访者：对同化了

刘书博：然后上了一个快车道。

受访者：对

刘书博：ok，那你刚才说这个老师是不一样的，刚才说这个实践性导师他对于行业如数家珍，那有没有不同的一些老师？

受访者：也有，但是这个课程呢他在选老师的时候，他跟其他的项目还不太一样，他就是一定选那种最实践化的老师，他不管你这个学术做的一定要多好，或者说一定要是正教授或者一定要杰青、长江之类的，所以他在选老师的时候就已经把这种特色化的东西体现出来了。

刘书博：ok，所以在研究生阶段你没有看到其他类型的太多的老师，比如学术型的？

受访者：对，基本上没太接触过，也有过一个，当时是一个从多伦多大学刚来的一个年轻的老师给我们讲OB，那这是一个很国际范儿、很学术化的一个老师，来了之后给我们上课的过程中就是各种这个OB的理论，然后最前沿的知识，其实讲这个课的时候他非常有激情，但学生反映的是：就感觉，第一，你不够那么实用，再一个呢就离我们比较远。后来这个老师又回去了

刘书博：所以你说这个前沿理论和国际范儿是在一起的，那前面那些实践导向的老师，他们有国际范儿吗？

受访者：实践导向的这些老师国际范儿稍微要弱一些

刘书博：你怎么定义国际范儿？

受访者：比如说他上课时候用的案例是国内的案例，还是国外的案例。再一个呢这些老师他所这个推荐的一些比如说就业的机会、实习的机会，是外资的还是中资的。再一个呢就是他们平常的这个生活作风，因为国际范儿的是属于那种11点之后到酒吧的，然后其他的老师呢可能还是比如说跟同学聚餐的呢，还是那种很中国风格的。

刘书博：所以国际范儿的老师就几乎都是从北美那边引入的？

受访者：多一些，就是至少是在海外读了博士的这一种，本土的它有一个词叫“三清”——就是清本清硕清博的，这种要明显的弱一些。我老板就是给国际范儿跟他自己之间划定了一个界定，因为我老板姓杨嘛，他每次就是遇到这样的场合介绍的时候说我姓杨，但我是个土鳖。

刘书博：那在当时你上研究生的时候有没有你感觉到一种氛围，就是好像海龟会更高级一点？因为院长，你知道，他是在倡导···

受访者：有这种感觉，这个不仅仅是隐形的感觉，而是会有明面上的表达

刘书博：他怎么表达？

受访者：我老板就跟我说，他是从2002、2003年入职那会儿，他说刚入职没多久，2004年、2005年的时候他就感觉到学院有个变化，变化是什么呢？就是新院长来了之后，钱院长来了之后，花了很大的这个力气从海外挖来了很多的人，有年轻的学者，还有这种就是在职的一些教授之类的，那来的这些人呢会给他们更高的工资、更好的待遇，那title也高，那当时呢我老板就说感觉到了一种危机，因为他说他自己是个土鳖嘛，他是中科院毕业的，所以他如果补不上这一块短板的话，可能在这边儿就很快会被···，至少没有优势，甚至会被淘汰。所以他自己也花了很大的功夫去斯坦福那边做那个福布莱特的访问学者，做了两届吧应该，然后跟那边呢就有一些学术上的一些合作，最后呢也搞出来几篇top A的文章。所以在那个阶段他是比较敏感、比较早的发现了这样的一个变化。

他这是更早的，那我们入学之后呢也能感觉到，比如说这些更国际化的老师有一些大的会议、大的活动的时候发言的，或者是说有这种主题讲话的，基本上都是这一类老师，相反的一些国内的、就是我们说国际化不是那么强的这些老师更多的是参加一些这种业界或者是本土的一些活动。

刘书博：ok，那这种会议能具体举例吗？

受访者：你比如说我们学院有那个学院顾问委员会，我们那个学院顾问委员会的级别相当高，每次每年开会的时候基本上都是国务院一个副总理来接待，然后习大大还有一次接待过，那在这个会议上呢会有这种海外的顾问过来，那接待的这些人，或者是他们一个小的圆桌的这样···

刘书博：我记得《新闻联播》上还播过，上一次就是习大大就参加了，好像是网络的线上会议，我还说这个学院顾问委员会规格真高。

受访者：我们相当高的学院顾问委员会的规格，这学院顾问委员会里边就是副国级的领导都有好几个的

刘书博：对对对对

受访者：所以你看就是在这样的一些场合的时候，那哪些老师去负责接待哪些人或者是参加哪一些的圆桌，就基本上能够把本土的跟海外的能够有一个区分。

刘书博：所以就感受到，其实土鳖明显在这个时候会低人一等，就是没有太多的话语权？

受访者：是，就这样一个高大上的事情，你参加和不参加就能够体现出来你这个身份上的一个细微的差别

刘书博：ok，ok，好，还有别的差别吗

受访者：别的方面的话就是发表论文这一方面，我觉得。因为那个时候我们上学的时候就已经开始实行这种“非升即走”的一个机制了，那当时呢更多的是看这种英文的top A的这种发表。

曾经有一段时间，我刚读博士的时候吧，那时候老师对中文是不太care的，所以我们学院那时候很尴尬的一个事情就是英文短期发不出来，中文呢又没人愿意发，那学科排名有一段时间是很快速的下降的，因为学科排名的时候他是看一部分中文的文章的，那中文呢当时在学院里也不会那么重视。

刘书博：那是怎么一个不被重视？

受访者：他有一个清单，就是评选的时候，比如说你要几篇什么几篇什么的这样一个清单，那中文在里边排名可能更靠后一点、权重更低一点，但是这个具体的数值我不知道。

刘书博：ok，明白了

受访者：所以你看后面这些老师，我跟我导师合作的时候，比如说一些中文的文章，他们基本上都不会帮你改或者不看的。

刘书博：明白了，ok，那我们再进一步说就是你后面研究生毕业，然后为那么选择继续读博士？

受访者：研究生毕业读博士，刚才我也有聊到一点原因，就是我跟我导师在聊一些事情的时候，就在这个逻辑上还是总能被他去挑战，我想这是一个我的短板。再一个呢就是想这种学历教育嘛，就是能在尽快的时间之内把所有的都完成，那是最好的一个选择。

黄秋莉：是不是您的导师也是跟企业界联系非常的紧密呀？

受访者：相当的紧密，他···当然后面这个录音这一部分就不要了，我就给你们都介绍一下背景。他到海外去，在斯坦福那边接触了一个合作者，这个合作者呢是张朝阳的导师，搜狐那个创始人，然后呢后来让他这个导师呢让张朝阳出了笔钱，让我们在国内做一个调查，拿了一个数据，当时给了100万美金，那时候还是很多的钱。我老板就说你怎么能轻轻松松上一个学生给你拿100万美金，这就不得了，那人民币当时是一千多万呢，然后他那个导师说这对我来说是个小数字，然后他那个导师在美国他带这个MBA的学生、带本科生什么的，因为他是做创新创业的，然后这些学生呢有创新创业的项目，他免费给他们做咨询，然后呢他会持有5%的股份或者百分之几的股份，那后面的这些公司上市了之后呢，他把这股份再变现，这个搜狐其实是他持股的公司之一，那可能为他带来了几千万美金的一个回报，但这只是在他所有的投资项目里边中等的一个回报。后来呢我老板他来到国内之后就把这一套学会了，他也这么搞。

刘书博：就是孵化，有点那个天使投资的那种。

受访者：对他呢更专注，说我在这边帮你提一些意见之类的，然后通过这样的一个方式，再一个比如说必须要用到清华的这个资源，因为太特殊了嘛，那他就在中间帮个忙，所以后面至于他搞了多少项目、多少收益，这个我是不知道。

黄秋莉：其实我看我们张老师（张云起）他也跟实践比较多嘛，但是他就完全是偏向企业那种类型，那我感觉在张老师那边儿，他的学术和他的这种实践导向是比较割裂的，我觉得是这种样子。所以您的导师是怎么去平衡这种，他要去服务实践哈，然后要去做理论上的一种···

受访者：我老师他是这种原则性比较强、纪律性比较强的，就是我跟企业里边儿我只是做一个顾问，我绝对不说我把顾问做成股东，就是很多的去参与你日常经营，这些我不做，我只做我最擅长的，比如说在战略方面我给你做一个策划，你未来方向应该往哪儿走，产品也好还是服务也好，我们怎么调整。那做这种大的变革他帮你来做，至于说日常运营管理什么的，他绝对不参与，这样的话就能说用我最有优势的地方去服务这个企业，那你认可就认可，不认可再换别的。

黄秋莉：所以他最强调的是自己的一种专业性。

受访者：对，专业性，因为他自己能到那儿去教书，在学术上还是不会那么差的。

黄秋莉：我想说这种专业性到底是怎么体现的呢？体现在他觉得我对你这个行业非常的了解，还是说我有些什么别的？他这种专业性是怎么体现的？

刘书博：你的问题是不是说，像张老师他可以帮企业，但是学术呢他就这个专业跟这个专业就不是一个意思，是吧？

黄秋莉：对

刘书博：就咱们知道要发top A···。

黄秋莉：对对对对，他可能是偏理论上的一些东西，就是在您导师看来他的一种身份象征是什么？他的一种优势体现在什么地方？

受访者：我觉得，因为我还跟他去做过一个项目，就他一个学生的。那我老板在这里面体现出来他的优势就是我觉得他的这个逻辑上的一些东西，比如说，当时那个公司做了一个就这种灯，但是那种圆的那个白炽灯，你见过吗？

黄秋莉：见过

受访者：他们那个公司呢就把这个灯壳儿做了一个很特殊材料的，灯掉地上摔不坏，然后这个还不算什么，然后它里边呢因为这个灯它亮的时候灯芯就会发热，发热的过程中呢很多灯这个坏掉，就是开灯或者关灯的一瞬间，因为它那个时候温差高，有一个热胀冷缩就容易坏，他们呢弄了个液体把整个灯芯儿泡在里面，就是一种导热的液体，那这个液体跟那个灯芯儿是一体的温度，那它一瞬间的时候就不会有那么大的一个温差，那灯卖地特别贵当时，在国内他们卖到上百块钱一个，我在校庆的时候我一个人卖出去一万多块钱的然后我老板呢当时就给他们做这么一个顾问，他的这个思路呢就说其实这算不上一个创新，灯逻辑上是要解决照明的，那更多的可能是不同的氛围、不同的色调、冷光暖光，这样就够了，那你把这些元素，比如说不怕摔呀，有这个导热液体，就是···

刘书博：它本身也是一个耐用品

受访者：对，你把这个因素加进去之后，这种创新有一点冗余的这种创新，谁家没事儿那个灯去摔着玩儿，或者谁家就天天把灯弄坏···

刘书博：它不会因为灯熔断会造成很大的···

受访者：对，反而呢是一些特种场合需要，最后我把这个灯给卖到哪儿了呢？山东有一个招远做金矿的，它金矿呢它外面会有那个大的那个机器会转，然后呢那个石子可能会崩，它原来呢那个露天的那个灯只要有一个石子撞下它就碎了，然后把这个弄到那边去呢，反而会好一些，那这是一种。还有一个呢就是矿山上那种高危的化工的行业里面，因为他这个他也不会找，也不会坏的，那就避免了很多安全事故，那这样的话我老板就认为说这一定不是一个面向大众的一个创新，那这种创新最后他的这个价格可能不是那么大。

刘书博：所以你老板提供的很像咨询公司···

受访者：对，就是专业的逻辑上的一些东西，他会帮你梳理，就是按他的这套理论呀、加逻辑呀来去做他的判断。

刘书博：就是实践的智慧。

受访者：对，他不是咱们现在说学术上的这些。他不是说按什么资源基础理论，按照什么交易成本理论，你这个这么做不合适，

刘书博：所以其实他也是实践做的很好的同时，他能捕捉到未来这个比如说国际化对于他发表的要求，他还是到斯坦福去及时地补齐了他的短板。所以他是在这个环境下，我觉得像张老师他已经是教授了，他不在乎也没有必要。

受访者：因为我老板当时去美国的时候，那时候还是副教授为了评教授，就是尽快的要过这一关，后来时间证明他这是正确的，跟他同一批的，后来因就是因为大量的海归回来，他们被拖了十几年，到现在···

黄秋莉：上次那个老师是不是就这样

受访者：咱们开会遇见的张老师，他正好赶上了，因为他比我老板晚几年，他年轻一点，他后边就top A的文章哗哗的，他后面也费了劲，给咱讲了那一篇应该是他最好的一篇了，后面讲AMJ之类的文章就很难，基本上上不去。

刘书博：对，是这样的。没错

黄秋莉：那您的导师在跟您指导的时候，他怎么说是您的文章要怎么去写，还是像我老师说你一定要做一些就是对实践有意义的东西，不要写那些···

刘书博：刚才杨老师，是研究生阶段？

受访者：硕士、博士都是

刘书博：ok，那咱们就进到博士阶段，那这个时候你又有什么感觉？冲击？或者是有一些失落，或者是···

受访者：有啊，这个就跟硕士就完全不一个路子了，因为博士这边一开始你就要开始搞这个研究，就是我导师刚才秋莉问说他怎么指导我的文章，说实话他基本上指导不了。

黄秋莉：对，我也觉得是

受访者：每次我把文章发给他，希望他能提点儿意见的时候，基本上都是边边角角，就是我们很客观的评价，他是已经在他的那个训练的那个水平之上，到了极限了，他就基本上他也不太说这些事情，但他有办法呀。他拉了几个年轻的助理教授构建了一个团队，然后每个周开组会，用他的个人的影响力呢把那几个老师强制的每次要求他们来，然后让那几个老师来帮他去做这个点评，他来做开场跟总结，这就是一个套路。

我们再接着说刚才刘老师问的这个冲击的事情，我刚读博士，我老板就给我找了一个小老板，从台湾回来没多久的一个···

刘书博：哪个国家？

受访者：美国，美国南加州的，他本科是北大的，然后让他来带我的这个论文，那我当时觉得写论文不就写论文吗，哐哐哐写了好多，然后拿过去让他改，基本上没有一个能入法眼的，那当时手把手的一点点从实证方法教起，一点一点的改，我们一篇文章改了30遍，就后来发了一篇SSCI，那篇文章改了20多遍，那是改了两三年，那后边手里边儿还有几篇到现在还在改着.

刘书博：你之前就是刚拿给他的论文是因为研究生是比较专业性的

受访者：对我写的他说这不叫论文，这叫实践报告或者是叫商业分析，不能当做论文的。因为商业分析里面多数是你的个人的判断，甚至有一些是很主观的观点，比如说这个市场很大呀、或者是未来的前景很好。他呢当时就特别反对这个观点，说你以后不要有你的主观判断在里面，你每一句话在写的时候要跟前面要联系的起来，就是谁能推导出来这一句话。那除此之外你那种主观判断的就不要有，因为我之前我们写报告的时候会特别喜欢说很大的很好的，或者说很有前景的，他说你没法去在这个论文里边儿去做这个判断，那你除非文献里边儿别人有什么观点你可以这么用，但是一定要有数据支撑的，后边儿的这个风格就其实那个时候是特别难受的，每一次次都被否定，每一次都会被否定，一周我们是固定的一个talk，两个小时，他在办公室准点儿等着，然后我抱着一堆的东西又准备了一周过来了，然后垂头丧气的走，下周再来，我能这样呢坚持了四年，所以我其实后来我还是怎么说呢，还挺感激这个老师的，那如果没有他就只是靠上课或者我老板，我是毕业论文都过不了。

刘书博：所以这个标准也是你的二导师手把手指导，这一套是怎么写···

受访者：对，其实说实话导师当时就跟我们现在看学生是很看不上的，然后我老板也是靠着个人的影响力硬把我，给揪过去了，到那儿之后呢，我一讲东西他是看不上的，所以那时候是压力非常非常他大，但是你也没办法，你还得硬着头皮往前推这个事情。

刘书博：所以这个就是有一个不同标准的，中间是有距离的，这是一个冲击

受访者：对，这是最大的一个冲击。第二个冲击呢就是这个强度上，硕士的时候就一年多的、一年半的课，上完了之后后面就你想选修点儿别的课也可以或者实习去交换也都行，当时硕士的时间还是比较自由的，我觉得我硕士什么事儿都干了，有各种各样的实习，然后还跑到台湾跑了好几趟，就那边合作的项目，然后呢我还自己联系企业干了一堆事儿，到现在跟这些上市公司都保持很好的一个关系，那读了博士之后，我想还是不是能继续这个节奏，发现不行了，他会给你特别大的工作量，比如说第一年我们上课的时候，我不知道你们现在上课，比如说像管理理论、管理方法的这个课是什么个上法，

黄秋莉：跟硕士跟本科一样的

受访者：我们的一周比如说管理研究方法这个课，下一周我们要讲这个调节作用跟中介作用，这一周我会给你五篇六篇文章，全英文。然后你去读，读完之后写这个critic，写完之后你还要交上去，然后上课堂上你要去讲，就这篇文章他用的什么方法，你觉得里面哪些好，哪些不好，你要讲，讲完之后大家还要一块儿评价，所以他是一个很小班儿的一个教学嘛，你没法偷懒，对，这种seminar你没法偷懒的，那上课的时候哐哐哐的就是读论文，睡觉的时间就基本上没有了。

刘书博：那你们阅读量压力很大了，然后呢这个就是必须得发言，然后其实就是他考核的压力的，是吧？你这个学习内容量很密，考核又很勤，又很到位，还有···

受访者：对，就这一块儿，因为大家也都朝着这个方向去做。那还有一个呢就是我们有一个出口观，并不是说我论文读完了及格了我就能毕业了，我们有一个博士生资格考试。

刘书博：对，这个也是美国的

受访者：哇，这个这就是美国完全那一套。资格考试，就是第一年上完课了之后到第二年的中秋、10月份的时候也就下个月的时候会对你进行一个考核，那这个考核可不是说你交一篇文章就行了，那它是有分为理论的考试跟方法的考试，那现场不知在哪儿找一篇小破文章，然后让你去写评论，然后还有各种奇奇怪怪的问题，有英文的文章，也有中文的文章，然后涉及到实证部分的、涉及到理论部分的，那方法部分呢还会让你写proposal，一个大的一个小的，就跟你写论文那种差不多，这个你觉得你答的挺好，但那个成绩每次都很惨，就是几乎每次都会挂一半儿的人，挂第一次你没问题，你下一次再考，挂第二次你走人。

刘书博：ok，那最终的通过率有多少？百分之多少？

受访者：不同的专业不太一样，你看经济系有些时候好像最终的通过率也就百分之六七十，我们的高的，我们最后好像都通过了，第一次我们挂了三两个人，后面他就第二次考的时候就过了。

刘书博：那你们学习的内容方面就主要还是美国的···

受访者：就完全是美国这一套，就是这一块儿呢，其实刘老师你不问我，我也想说的是，那你比如说我们第一看文献这一方面，说实话从头到尾没有任何一个老师给你布置过一篇中文的文献，全是英文的，这是第一个。

第二个呢就是这个范式，因为我们当时没有做case、做这个定性的这一块儿的，全是定量全是实证的，所以后面呢我们还专门开了这个计量经济学、统计学的这些课程，就是做这些补充嘛。

再一个呢就是定期请国外的一些或者是做比较好的一些人来做讲座，基本上也都是实证这个领域。所以你看这个内容上、方式上很多的时候都是采用了国外这一套。所以因为我老板后来跟我说过一句话，说你虽然没去美国读博士，但是美国博士所用的那一套你基本上也差不了哪儿去，说资源也好还是方法也好，他们有的我们都有，因为清华经管确实花了大钱从海外找了这些老师回来，那他们在方法上、在理论还是相当广泛的。

黄秋莉：其实我想问一下，咱们这个国际化是咱们经管的一个特色，还是说整个清华都倾向于这种国际化

受访者：都这样

黄秋莉：连文科都是这个样子的吗？

受访者：文科除了马哲，马哲就这么说吧，我去专门去选修过马院的课程，马院讲党史的一个老师，他讲的课非常非常的好，能好到什么程度？他是我大学以来，我觉得讲课最有魅力的一个，青教赛北京市的特等奖，然后教育部的特等奖，然后他跟中组部去讲课。那他是教党史，然后他们每一年暑假的时候要跑到斯坦福，跑到剑桥去跟那边去合作做一些交流，一个是那边存了很多我们国内找不到的这个党建的资料，再一个呢海外有一些研究学者一些新方法、新思路，他们也要过去借鉴，所以他们有一个夏令营，专门是每年去，我记得还跟我说过，问我要不要去当时，因为我不准备在那个方向去做一些更深入的嘛，就只是上那个课，所以马院国际化多多少少也有一些，其他的学院就更不用说了

黄秋莉：他们考核的方式也都是这种比较资格考试这种吗？

受访者：对，全校基本上都是这样，包括老师，包括学生。后面老师的这种长聘机制，那是就是全校长聘，上次张老师来，他不是说他长聘了吗，那是校长发的聘书，就是全校一次选多少个长聘副教授，那这样的话就是他不是院内竞争，而是全校竞争。

刘书博：那还是回到刚才你说全是美国的那一套范式在训练，为什么全是美国？就没有欧洲的吗？因为我觉得欧洲也是有一些····你们当时就没有看欧洲的一些期刊呀？

受访者：欧洲的期刊不是主流，因为欧洲呢更多的是这种偏定性一点的一个范式。再一个呢就是欧洲这边这种理论，就有点儿跟你们现在做的批判性的理论多一点，但是实用性、工具性的理论没那么多。你像我们说战略管理里面，美国的范式呢就是有资源的理论，有什么制度的理论。那欧洲的呢更多的是一些，比如说在文化上、在社会上、在伦理上，对这一类的批判性的理论。

刘书博：甚至欧洲会哲学层面，所以你们当时有没有学诠释主义、构建主义、社会建构主义呀？

受访者：有非常非常的少。

刘书博：但实证之外还很多别的研究范式，比如后现代主义、解构主义等。

受访者：这种风格有点儿偏我刚才说的报告，会更主观一些，因为这没法说拿一个数据去做支撑。

黄秋莉：是不是学院里欧洲来的老师比较少啊？

受访者： 对，欧洲来的比较少，基本上都是美国那边过来的。然后论文方面，欧洲的期刊比较少，基本上不在考核的期刊指定目录上。

欧洲的范式，其实还是来中财之后刘老师才给我灌输的，像这种后现代主义、资本主义等，这一套我们上课的时候比较少，基本上没有接触。

黄秋莉： 上次来我们学校的老师您的同门，跟整个学校的主流就不太一样，她自己就是做的案例研究嘛。

受访者： 因为当时我们学校没开案例研究相关的课，她自己比较感兴趣然后自学的相关内容，然后自学过程中发现各种流派、各种方法杂七杂八的，最后她也没搞明白，后来他去了美国的一个学校待了接近一年，那个学校做定性这一块儿做的非常好，然后她到那儿之后真正的把这个方法梳理清楚了，你看她那天来我们学校那个论坛讲的时候，其实她心里是有底的。她在这一块儿做的也相当的不错了，未来方向也基本上就定这个方向。现在她已经回老家了， 今年入职西北大学，西北大学也是一个老牌儿的大学。

黄秋莉： 是的，是的。那您读博之后有没有想着自己以后就是要去当老师了？还是说坚定了自己原来的想法，想着还是要去企业？

受访者： 其实没有那么坚定，其实我读博士的时候硕士同学每次聚一聚都要笑话我一顿，就说我为什么读硕士的时候那么好的机会，你非得去读个博士去受虐。我的学校里边儿的故事他们也都知道，其实读博士的时候非常辛苦，最后你那个文化与产出其实完全不成正比。所以每一次吃饭他们都要笑话我一顿。

黄秋莉：那您的同门师兄师姐他们读博是为了当老师，还是说是其他的想法呢？

受访者： 很奇怪，我们这一届毕业有一多半儿做了教师，这个比例是清华经管历史最高的。我们下几届全都不做老师了，要么去业届赚钱要么就直接去当选调生了，因为他们去直接就给副县长。

刘书博： 所以你在读博士这个过程中会不会有一种怀疑、动荡、摇摆的念头，就是说自己这么辛苦去做这些研究？另外，自己会不会想着做这些研究到底有没有价值？

受访者：对，我就是在这种不断否定、怀疑的过程中往前走。这事儿我也跟好多的老师讨论过。

刘书博：能不能再详细说一下，比如你觉得这个研究是不是太偏离实践呢？

受访者：因为我们做论文的时候是越来越窄了，是一定要跟漏斗一样窄到一个能跟哪个文献对话，或者是能接续到前面的一篇文章。比如说我就看a跟b之间的一个很小很小的一个关系，那我们最后看R2方解释了百分之多少的变异。我们会关注这个研究结论拿到企业里去到底有多大的一个价值？比如说企业按照这个结论去做就能赚钱吗？或者说就能有竞争优势吗？

后来跟企业打交道，他们说他们根本不信这一套，至少这种单篇文章的观点不足以让他们实际去采用这样的办法，可能更多的是这种理论上一个大的思路上对企业有一些帮助。所以这个时候我们也是很怀疑自己做这套东西到底有什么意思，其实到现在也没有说完全肯定这个东西很有价值。

刘书博：据你了解，在清华经管的有些老师们是怎么做到始终如一的对这个事情充满了热情，尤其像美国那些老师那样非常投入呢？

受访者：因为我们跟有些年轻的老师关系都挺好的，有时候是教我们的老师，也有可能不是教我们的老师聊到这个事情。刚开始来的时候他们也非常有激情，我觉得从国外接受这一套研究训练，然后回到国内之后，有了这样的一个施展空间，并且国内这么多的企业能够做一个研究的对象，所以他们就会很卖力的去写东西。

但是去做这个工作特别累，尤其是女老师，你不知道有些女老师生了宝宝之后，那还要上课、带孩子、做科研，其中的辛苦是超乎想象的。后来不能说全部的人，是有一部分的人，就说工作这么累到底为了啥？慢慢的就被现实给打败了之后，就说这只是我的一份工作，就是一个可供谋生的饭碗、工具。包括我们之前质化研究会上请的清华那位老师，开完会之后我们一块儿吃饭聊了聊，他就说“我也看开了，我那么累干嘛呀”。那会儿这位老师有一篇ASQ在审，后来修改了两三次被拒了，这对他来说是个很大的打击，因为他也面临着对他的考核嘛。他们比中财还好点儿，就是他们周期长，至少六年，不过周期虽然长，但是英文的文章你一年两年才能搞出来，好文章周期同时也是五六年的周期。所以后面他慢慢也就躺平了，开心就好。

刘书博：那他对于未来要面临的考核，他会怎么安慰自己或者是有什么自己的办法吗？

受访者：原来他们是非常坚持自己做学术的taste，就是说要做就要做很好的研究。那现在慢慢的，他们就想着说要不要再弄点儿中文的凑一凑，有总比没有好嘛，就慢慢的有了这种心理。

其实我刚来中财的时候我也有这种想法，就一开始还是有点学术志向的，就是说我要做一个好的东西，但是因为未来的考核标准会让你不断放低自己内心的标准。

你看原来国内的一些杂七杂八的会，他们都不太想参加，你发邮件发通知请他们去，他们都不太想去。现在没事儿也得去混个脸熟，现在发中文文章的话就是这么个学术生态。

我跟我小导师一块儿做的那篇文章，英文的还没发出来，中文方面就发了一篇综述。之前他也看不上中文的文章，包括我们发的那个期刊，但后来发了之后就觉得：有总比没有好吧，那篇文章被我们学院弄去做在首页上还宣传了好几个月。

其实哪有那么快能做出来东西的？全国甚至全球几千上万个人都在这一小块领域。所以后面我的那位小导师慢慢的也转了，也开始做一些中文的。其实不只是一个老师是这样，好多都是这样的。

黄秋莉： 有没有要转行的老师啊？

受访者： 有，今年清华经管学院有两个老师来我们学校了嘛，因为他考核期的deadline到了，但是成果还没有出来，不过当时他们也都是坚持着要做好一点儿的东西。

但实际上他们手里边儿是存了一些东西，那这个东西可能只是半年一年出不来，但长期来看，后面可能会有一些石破天惊的结果出来。

刘书博：其实我们学校在招人的时候，也会有一种所谓的傲娇的心态，就是说你一开始不投我的，你现在又想着再来我们学校，那我们学校也不要你。

另外，学校在招聘的时候，招聘的刚毕业的人员会比较多，其实这个背后的原因就是他会假定年轻人好管理、听话、好用。那如果是岁数大一点，学校层面会有一些假设，第一个是学校的傲娇心态，第二个就是觉得岁数大的人不好管理。这两点是我自己的一个总结：管理主义在高校越来越凸显，他在挑人的时候就会想着去挑一些好的“员工”。

受访者： 我跟企业接触的其实也不少，企业的高管或者创始人有一个很重要的特质，即包容性。有些人就不敢招那种很有才华，但是不好管的人。因为他想着我要把这个人弄过来了之后，他可能就敢挑战我。所以他就想着去招那些听话的，哪怕你技术烂一些什么也没太大的影响，首先是你一定要听话。这种情况导致的一个问题就在于，企业在成长的过程中难以形成一个好的团队，他会慢慢的把企业中那些带刺儿的成员都赶走。所以你看很多公司，比如说在一些传统制造业，他可能也不需要员工有太多的想法，就螺丝钉化，就你按照我的标准做到就行了。

其实现在学校不也就是按这种想法来做的嘛。

受访者： 我刚说的这个企业是我非常熟悉的一个企业，就是一个做制造业的，研发、市场得找专业的人，因为研发、市场的活儿可不是说随便招一个人培训两三天就能干的。所以在这两个阶段他会多花点钱把人弄来，但是用完了之后就想着你快点儿走才好。这其实是一种非常短视的行为，当你后面再需要人才的时候，比如说在遇到发展瓶颈，再想用那些被赶走的人去帮他过关的时候就很难了。这类企业的发展天花板就很低。学校现在其实是一样的，如果不改变这种情况其实就很难长远发展。

刘书博：公司化、市场化导向就是追求短平快、短期效用最大化。清华经管的钱院长在任的时候，他一定要有显性的国际化的效果，那他就直接大量的招外部人快速的复制，那结果肯定就很快出来了，但是这个东西对于中国来说是否合适呢？因为它是在美国土壤上成长起来的制度，咱们在中国现在其实也感受到了这个“非升即走”的副作用。

而且美国的“非升即走”，有外部的一个健康的流动的市场，从哈佛离开之后，也可以到南加州，他是有地方去的。但中国现在其实就有点尴尬，你从北大清华走了，可能中财都不想要。那你从中财走了，你想到政大去，但是人家政大内部可能是一个萝卜一个坑，人家的岗位在内部市场就被消化完了。所以我们国家“非升即走”的外部市场还不是很完善。

受访者：刘老师说的这点跟我老板做的一个研究比较像。他们是做的一个制度与创业的一个研究，中国大学前几年搞了“985”工程，“985”工程的目标是在多少年之内培养多少所具有创新精神、国际化视野等特质的高校，然后为了配合这样一个政策，国家在拨款、在其他的方面给了很多的优惠待遇，学校确实拿着这些钱去做了一些创新的事情，在专利等项目上做的都很好，要比之前没有“985”政策的时候有明显的区别。

他们研究问题是：这些在学校里创新项目搞得很好，即有专利、有技术的人，到了社会上是不是也同样搞得很好。

研究结果发现：这些人到社会上比别人更惨。原因就在于跟你说的一样，没有一个统一的市场。第一个方面，学校是保护着你，实验室随便用、技术随便搞，然后也包容你失败，包容你去各种尝试。但是到社会上之后即使做出来好东西了，明天可能就被抄袭了，外部市场没有专利保护。第二个方面，比如说资金支持靠招商引资，明后年没做出东西来我就不给你支持了。这就是包容性不过，同时对知识的保护不够，导致了在学校里做的很好，到了社会碰的满头包。

研究结论就是说这种内外不一致，形不成一个统一的市场，导致创新没法进行规模化扩散的。

受访者： 回到高校“非升即走”的制度，在美国外部和内部大家都统一意见，社会层面存在统一的共识。那在中国就不是这样，你走出来之后就死了。

我个人觉得我们国内在搞制度变革的时候，我们喜欢“摸着石头过河”，就是先找一两个典型试试，试完了之后我再去慢慢推广。但是试的过程中没有太长远的眼光在这里边，这里面可怕的一个点在于试完之后你觉得这个制度好，那再往别的地方去推广的过程中，如果没有一个统一的保障体系，就会导致推广过程中一方只学到表面，把里边儿的一些好的东西全都弄掉来。

“非升即走”本来就是想引进一种竞争机制，我觉得这种竞争机制很好，但问题在于你引入这个竞争机制之后，你只是把竞争从所有人层面的竞争变成了一个小圈子层面的竞争。“老人老办法、新人新办法”，那你怎么搞，“老人”又不是说三五天就退休。那三年都不退，二十年也不退，那这怎么弄？

刘书博：这个就是中国特色的制度过程。像哈耶克、波普尔他们就认为，如果国家层面在进行计划，就像中国现在高校的管理模式，“985”就是多少年你一定要世界一流，就觉得这个事儿就跟发射火箭到月球是一样的。但是社会层面没有办法达到工程精确制定的结果，这就导致像哈耶克在《通往奴役之路》说的，这个计划是善意的，但往往会造成灾难，本质是一样的。

大学这个事情不是简单造的火箭，这个教育包括改造社会以及思想层面的推进，它是一个生态性的建设。不是说你规划好了，然后就建起来，而是说人们慢慢的在草地上会走出一条小道，就像市场调节机制一样，现在就相当于没有配套，我先把一个最核心的东西建起来了，然后至于你后面怎么走就不管了。计划生育是一个道理，我就管理人口，反正目标在那，然后中间过程会出现各种各样的对于人的压迫。我们就会说为什么当年就没有想到现在，其实这就是一个思维倒推的过程，就是说明天我们要到哪儿去？然后我们就必须几点到哪儿。

受访者： “非升即走”在国内现在被搞成这个样子，我个人感觉另一个原因是做这个事情的人跟考核这个事情的人不是一批人，考核的那帮人可能还是原来那种事业编的或者是不会受到 “非升即走”影响的。那他来考核你，并且标准由他来定，这个时候我们先不说外行考核的管内行，至少他们不是在一个机制之下，那设置考核规则的人才不会管这个过程机制，这是第一个方面。

第二个方面，在国内多数情况下中文文献还是有很大的作用的，那他们掌握着话语权，最后会把这帮被考核的人全部拉到他那边儿去，你必须得找依靠，为什么呢？原来没有压力的时候，你还可以说我慢慢悠悠，我去做一个真正的好的一个研究，要做一点深入的发现。那现在有了考核压力，别的都没变，还是这帮人的标准，并且你的这个压力更大了、时间更短、节奏更紧了，那这个时候就把这些人完全的推向了原来那帮人。因为短平快最好的方式就是在出口那个地方去找办法。

刘书博： 对，不考虑土壤环境直接嫁接过来，结果就会产生很多负面的意想不到的结果。那现在就对于你个人来说，你会觉得应该是压力更大，你学术志向这方面可以先放一放的情况吗？

受访者：对，像我去年入职的时候，我还是坚持着要做几篇英文的文章，我们当时改这个文章的节奏也是比较慢，后来给我考核的时间是两年到三年的时间，这个两年如果到了的话，工资会给我降很多，我那合同里边儿是有这个标准的，那这样的话你比如说我要买个房子，我是不敢买的，那个时候万一有点儿什么问题，你这房贷还不上。

再一个就是比如说你押注的那个英文文章没有发出来怎么办？所以有一段时间就非常的焦虑，那就赶紧想着说还要到处东抓西抓、拼拼凑凑，先找一些顶上。那后来慢慢的打开自己东拼西凑的东西，你自己看着都恶心。有些也不是你自己想干的事儿，自己还得整天面对这样的事情的时候就很不爽。

后来想了想，算了，我去哪儿不能过得比现在好一点（哈哈）。我同班同学，就是那位第一次资格考试被挂掉的那个同学，我们都是战略系的，我四年毕业，他五年毕业，前两天一块儿吃饭送他们毕业来着。他现在是去了美团做这个美团的战略部，起薪60万。

刘书博：其实这么看起来，在中国管理学商学院市场主义、管理主义的情景下，那其实作为老师还是有退路的，那也可以到行业里面去。

受访者：但是这也分专业。管理专业，咱们跟商业结合的还算是比较紧。但是有一些比如说搞理论的那些，他们的路就会越走越窄，他到了企业也做不了什么，业界也不会有很好的待遇给他，那这时候他怎么办？

黄秋莉： 我之前问过我们学院的另外一个老师，我问他对中山大学“非升即走”看法，他说难道他们不觉得中山大学是给了一个非常好的平台吗？他即使在那个地方没有留下来，他去广州其他学校照样可以谋得一个很好的职业，为什么大家都要批判这个事情呢？他是这么一个想法。

受访者： 我觉得对中山的批判是什么呢？他创造了一种非常恶的方法，让别人都不得不去这么学，别的学校不这么搞就会觉得吃亏。因为中山大学先筛选了一次，然后中山大学花了大量的钱，做出来的东西是它的，做不出来你走。个人到哪儿能拿点儿钱，然后能拿个比较好的履历。

刘书博：就是中山大学之外的一些学校，他们会觉得咱们不学中山咱们就会落后，所以都这么学，并且这个坏是正大光明的，是天经地义的，是合法的。

受访者： 对，导致大家都不得不这么做。

黄秋莉： 对，但是他对“非升即走”这件事儿持好的态度呢？

受访者： 我觉得引入竞争机制是一个好事儿，那错的地方是这个竞争是恶性竞争，他不是说竞争的同时是要发展人、促进人、激励人，而是说把这些年轻人的价值压榨完。因为我们看现实就是他每年招几十个，然后只留下很少一部分。美国的“非升即走”，他可不是说尽量的换血，他是说能够让你尽量的保留下来，然后杜绝滥竽充数的机率。

黄秋莉： 哦，就是对人的一种压榨。

受访者： 另外一个问题就是大学太不自信了，他不相信自己选人的眼光，不相信他自己用人的环境，我只相信市场第三方的评判。

刘书博：我补充一下，我当时找工作的时候遇到一些学校，他招人就是有点像一些互联网企业他大量的招，然后面试非常流程化，他不会问想要真正了解你，他就是先把人聚拢过来，然后你自己去平台上竞争吧，我组织就把自己该做的工作给排清了，然后也不用担责，让市场评价就是了。

反而现在给了其他第三方，像基金委员特别大的权利，我们其实缺乏一个健康的学术共同体。

首先，学院和学校应该去保护自己人嘛，但是他明显给你隔离开，我不要对你负责，你自己在市场上试着生存吧，我撇清我自己的责任，这就是没有共同体了。比如说企业里边该冲锋陷阵的一定是带头人，是老大，这才对。但是现在的问题是他们都在后方运筹帷幄，让新招的老师到前边儿去冲锋陷阵去了，被砍死了，那是你的问题，说明你实力不强，然后十个还剩了一个，那这一个就代表我的战斗力，就代表我的事情。所以其实我们说中国跟美国的土壤环境不同，就是他们有一个共同体系，大家是互助的状态。

刘书博：就说管理学学科，那哈佛招的人他们肯定是充分了解的，而且据我了解，像中欧招人也是好多老师在深入交谈。

受访者：然后我经历过就华南一个学校的招聘，就是一堆人排着队，像公司招聘一样，然后他们围一个大圆桌，一个个进来，每个人可能就十分钟时间，随便介绍下自己。这能了解你什么，比如说我们现在招人了，我觉得时间长了对我们班同学谁能做出来东西还是能有一个基本的判断。那有些人是那种慢工出细活，非常严谨，非常有品味的，但是这种可能3、4年都出不来，那他现在手里边儿是什么都没有，那这种人可能连第一关都过不了。

刘书博：但这种判断就是需要很深入的了解，所以我觉得华南或华东那种市场化就太着急了，他就看简历，数你发几篇什么级别的期刊，他甚至也不会说想着读一下你写的文章怎么样，那我觉得这就是学术商业化、市场化的一个表现

黄秋莉：在现在的中国，老师之间的竞争非常的激烈，会不会导致老师之间的交流会进一步的被阻碍？就比如说我要好好搞我自己的事情，不能让你知道我的情况？

刘书博： 你说到的是进来之后的一个状况，我们说的还是在招人的时候。

受访者：人进来之后可能也不会管，相互之间也是对缺乏一种合作。

我举个例子，关于慢工出细活这个事儿。我我还是读硕士那会儿，有一次在清华门口剪头发和旁边一个老爷子聊天。他原来是清华精仪系车间的一个老技工，他讲到原来他们做的是要怎么样把中国的这个精密制造水平提上去，那这个机床的精度、加工的精度、金属的硬度都要提高，它是一整套的东西。但是现在多少年了，精密制造还是我们国家的一个短板。他当时就举个例子说，原来在国企的时候，这些老车工不用担心饭碗，你下了班儿，你可以拿着那个那个设备慢慢的磨，就一点一点的去做这些东西。现在没有一个企业会这样，都是赶紧计件，那去做这个研发的基本没有。

然后他说，九几年的时候有一个国企改制，现在很多上市公司做机床的技术还是来源于改制之前那个老国企的技术，也就是说后边这些年机床水平基本上没有太大的提升。

黄秋莉： 企业比较更关注自己在市场上的相对的表现。刚刚李老师也提到研究跟实践相关性的问题，其实大家现在好像有一种批判，就是说大家做的研究就是一种自说自话嘛，好像就写了一堆文字，可能自己要写文章的时候才要去翻一下，那这种情况的出现也是跟竞争有关吗？还是说是什么其他的原因导致的？

受访者： 我个人就感觉到这种变化，比如说我硕士的时候是写报告这种，那你是还是要挺全面的去了解一个东西，然后你需要提出一个方案。那现在就属于论文的考核体系，他只是要就着一个小问题往前推一点点，你要是推大了，他说你这个文章不聚焦。现在基本上提不出来一个大的理论的一个重要原因就是每一个问题小接口都是设计好的了，那你现在突然提出一个新理论怎么怎么样，那前边儿没有跟任何一个人对话，这样是不行的。

黄秋莉： 整个论文评价体系的问题哈。

刘书博： 学术追求的是严谨性。不能够随心所欲的想或者进行一个主观的判断。整个研究问题要是做大了，也很难在一个文章的篇幅里呈现出来严谨性，所以就尽量研究小的问题，这样才能够达到一个严谨的标准。

黄秋莉：是这种研究问题要小的严谨性导致理论跟实践之间的差距吗？

刘书博：对，其实这是科学性和实践性之间的矛盾。其实真正的管理实践有科学的一面，但还有经验和艺术、手艺的一面。那我们学术只强调科学，所以他把其他很大一片全部都给忽视掉了，因此导致两者之间出现很大的一个鸿沟。

受访者： 另外一方面，没有人愿意去把小零碎的发现拼接成一个大的整体，比如说我们现在的教材也好还是什么别的也好，很少是把某一个领域最新的发现按照条例一个一个的串起来，然后展现出一个新面貌。

暑假的时候参与了一个梳理创研领域前沿文献的项目，我负责弄三篇文献，我感觉这一个活儿都够我干一个月的。那如果不是我导师来组织这件事，这个领域基本上没有人做，那我们看的文献或者了解的东西也都是多少年前教材上的东西。

黄秋莉： 梦娜比较关注您当老师之后的感受，失落呀或者是意外的惊喜等等，她比较关注老师身份意义上面的一些问题。

受访者： 老师这是一个靠良心吃饭的活儿。现在讲课讲好与讲不好基本上没有太大的区别，这也不会影响你的工资，因为教学是一个底线考核，你只要没有教学事故就行，但是论文是一个顶线考核。

我觉得做老师比较快乐的地方就是看着大家能有点儿收获还是挺高兴的。我之前给夜校上课讲了一个内容，有一位同学听完了跟我说，老师你能再讲一遍吗？他当着那么多人面儿在那个大群里让我再讲一遍，我当时挺吃惊的，我说我是没讲清楚吗？他说不是，是讲的太好，对他企业价值特别大，他觉得还没听够，还想再再听听。后来他写邮件找我好几次，我把资料和视频都给他了，他后来还想再来找我聊一聊，不过我一直还没找到时间。

再一个就是在沙河那边上课，其实有些人他很想了解企业的东西，我第一节课是七八十个人，讲到第二节课的时候还剩24个人，我当时特别失落，想着自己没讲好吗？后来他们说要求太严了，那严的话也不是我的问题，我就给剩下这些人好好上课。后来我讲课的过程中有不少人又回来旁听。后边这些学生会问很多课堂之外的东西，因为我之前做的事情也不少，我跟他们聊这些事情，他们感觉还是特别有收获的。我上课的时候我从来也没强调过纪律，我也没强调过说你们要发言，谁发言了我就去记一次发言，然后谁没来我也没让他们签过名什么的，但是从来没有人上课玩手机，也从来没有人不来，我觉得我还是挺欣慰的。

因为老师是一个靠成就别人来成就自己的一个活儿，跟别的工作完全不一样。比如说我们要跑销售或者做市场什么的，那就是你自己做多少就是多少。但老师这个事儿你是要让别人做好了你才能有收获的一个事情。

黄秋莉： 像李老师您那么优秀，我也想问一下老师的同门问您的那个问题，就是说您明明有很多更好的选择，为什么最终还是选择做老师了呢？

受访者： 可能也是在等个更好的机会吧。

黄秋莉：就是也是想着再积淀一下，是吗？

受访者： 说不定过两年我就去企业了，到时候你们别笑话我（哈哈哈）。

黄秋莉： 不会呀，我们都会羡慕说您去赚大钱去了。

受访者： 科研真实越来越难做，并且你很少有成就感。比如说我们发完的论文，我到现在我自己都不愿意看，就觉得是为了凑数去搞的这么一件事情。

网上有张图，就是马头到尾巴，前面画的很好，后面就很潦草，这两天做课件，我还把那个图放在上面。因为创新是一个观点，是受到多方因素的影响，最后被建构出来后，那跟你最初的设计可能就不一样了。这个时候还是有点儿虐心，那你能受得了这种虐，你就可以去做这个研究，受不了的话挺折磨人的一件事情。

刘书博：我发的论文有时候也不愿意再看一遍，之前我翻译了一篇文章叫“废话的胜利”，那一篇其实说的就是就改来改去，最后你想写的东西已经面目全非。

受访者：最后真正迎合的就是审稿人的意见。

刘书博：这个是全球学术界共同面对的一个问题，就是因为审稿的这样的一个机制，而且标准越来越高，大家越来越内卷，最后就导致精致的平庸，虽然写的很精致，挑不出来什么毛病，但是这个研究问题缺乏重大发现。

# 受访者30

刘书博： 特别感谢赵老师，尤其前前后后这么长时间一直关注我关照我，而且赵老师也是我学习的榜样。这一次赵老师听说我做这样的一个研究，特别的上心，帮忙联系到梁老师您，所以也特别感谢您。那我们这个研究主题呢，现在也没有特别细，就是想看一看中国的管理学和商学院，这两大块儿，我希望对接到的理论对话点是工作社会学，也就是作为学者，尤其是管理学的学者，在中国的历史环境背景中是怎么样发展的？那这样的一个学科，包括做这方面研究的学者，那现在有什么样的工作特点、身份构建，包括如何进行工作意义的构建、以及会遇到什么样的问题；还有学科标准，这些比较宏观隐晦的东西，它的发展脉络是什么；还有商学院的管理特色是什么，它可能存在什么有待改进的方面等。

总之，目前还是比较粗线条，我希望能够研究一下这些方面，因为我之前看到周雪光老师写了讲斯坦佛大学的论文，并且他们在创新创业方面做的很强，所以我其实也是想关注一下中国的商学院是否有一些类似的特色？我们的土壤环境又是怎样的？这种土壤环境是怎样逐步发展起来的？大概是这个方向，那现在参加这个研究项目的还有两位博士生，她们在读博的时候也会反映出来一些苦恼，或者有一些不理解的地方，所以她们也想真正去探索一下，为什么现在我们的管理学教育是这样的一个现状，那到底它的历史沿革是什么、怎么样发展出来的，我们大概是想要去探索这方面的内容，所以就想请教梁老师，我们可以一块儿来探讨一下。

受访者：我跟赵向阳老师相识是在16年，很偶然，那年陈明哲老师有一个叫中国管理学者交流营，然后他每年有一个年会，那年的会议呢刚好是在杭州，然后我们和浙大一起来承办的，那么那年的会议呢刚好跟浙大承办的那个IACMR的千人大会，在时间上是有重叠交叉的，那么浙大管理学院就把主要的精力都投到那个会上，所以呢就把主场放在我们这个工业大学，然后向阳老师呢本身是陈明哲老师很欣赏的一个年轻才俊，那一年陈明哲老师专门要求我把赵向阳老师请到杭州来参加我们这个会，我们那年才认识的，这是16年的事儿。

17年呢，那个年会是在这个吉林大学开，然后我们都到了长春，要等深圳大学的韩巍老师一起。韩巍老师以前跟我在西安交通大学，我们俩是同事，然后他后来先去了深圳大学，然后我后来来到了浙江工业大学，当然中间我们也有过一些见面。但是在长春我们那次刚好碰到一起开会，也是很偶然。然后赵向阳老师跟韩巍老师还有非常非常密切的关系。那天我和向阳老师先到，然后那个韩巍老师航班比较晚所以我们吃完晚饭就在向阳老师的房间聊天儿，然后一边聊天儿，一边在等韩巍老师。然后赵老师就想了解了解管理学的圈圈——过去发展当中的八卦也好，故事也好，脉络也好，反正就是茶余饭后，我给他讲了一些故事，然后后来等到韩巍老师到。

然后这个事儿呢，反正我理解就是赵向阳老师应该是没有什么目的的，不像书博老师现在是还是想要做一些研究、积累一些素材、或者为后边的研究梳理一些脉络或打下一下基础。所以那次我们聊完就完了，因为讲的一些东西都不是要打算写到书里边或文章里边的、也不是在公开的音频视频里可以听到的，所以我估计赵向阳老师可能觉得也蛮有趣的，可能有点印象。所以前天他跟我说这个事儿的时候，我觉得可能也是类似这样的一个偶然的背景。夸一句向阳老师，然后就回到我们的正题里。

因为向阳老师科班儿本身不是学管理的，但是又在北师大的经济管理学院，又要给学生教管理学方面的课程，所以管理学的这个“50人论坛”他也是其中之一，所以他现在应该是我们圈儿内人士。但是呢实际上这个大家学管理的这个过程当中都会有这样的一个体会，就是管理学本身它是一个交叉学科、边缘学科，那么多学科的融合是很自然的一件事情，所以等会儿我讲到我自己的一些理解的时候，也会穿插到这样的一个话题，因为在这个里边呢肯定能够发展出来或者是演化出来一些新的或者是跟过去的、传统的学科发展脉络、模式、思路有一些不同的地方，那肯定就是有一些创新性的成果，所以呢···

赵向阳： 受访者老师，我先插一句，我们当时聊了半天，基本可以提炼出一个很重要的信息，就是我是学理论物理的，后来又是学心理学，后来又在德国的心理学做博士，但是我研究的是创业，所以事实上从这个时候才开始慢慢的转向创业。然后书博呢，他本科是学社会学的，然后慢慢转向管理学。那韩巍呢，韩巍实际上最早学的是化学，然后他硕士和博士就是在西安交大，跟席酉民老师学习。韩巍硕士读的是营销对吗？

受访者：对，他现在仍然是在教marketing。

赵向阳： 然后你的本科是什么呢？就是我们那一代有很多的人呢，他实际上是五花八门，从那个不同的（专业跨过来的）。就比如说席酉民老师，他一开始是物理，然后转到管理学的，然后呢像刚才这其中一位博士生是从哲学转到了管理学，另一位没说我也不知道，ok。但是现在他们基本上都是硕博连读，就是说他们现在的这个商学院里边儿很多人都是从本科就读商学院的，然后就一直在这样的框架里面，而不像我们以前的五花八门的不同的专业。因为30年前中国是没有管理学这个体系的。对，我插这一句后，咱们不妨就开始从这个地方慢慢往深讲。

受访者：向阳老师刚刚揪了一下我刚才的偏差。好了，我们现在开始逼近核心的题目。OK，我先介绍一下我自己的背景，我本科、硕士、博士都是在西交大的，所以跟赵向阳老师不一样，他们前面儿是土鳖出身，然后后边儿呢又变成了海龟，我就是一个很地道的土鳖，但是我的特点可能跟他们不一样，就是我从头到尾基本上就都在管理学的那个圈圈里面，为啥呢？这个我是81年入西交大，然后本科读的工业管理工程，就是我拿的是工学的学位，因为我们那个专业的名称后边儿有工程两字，所以带工程的都是拿工学的这个学位，一直到我硕士的时候，后面我本科毕业，然后就留校先做八年辅导员，然后呢一边做辅导员，一边在职读的硕士，然后跟的是李怀祖老师。我的硕士三年拿学位也还是工学学位，这里面有一个具体的年份，就是1998年教育部把我们国家的学科专业学科目录做了一个调整，那我们现在本科和研究生是划开的，那么98年的时候呢，教育部把研究生的这个学科目录呢做了一个调整以后呢，其中专门画出来一个类，就是管理学，在这个之前，就是98年之前，凡是在中华人民共和国拿到学位的，不管他是本科的、硕士的、博士的，只要是他拿到学位的，没有管理学的。所以呢刚才这个向阳老师介绍到的我的博士导师席酉民老师，有的时候你们去看那个报道或者媒体里边儿讲，中国大陆的第一个管理学博士之类的实际上是不准确的，因为他拿博士学位那个时候还没有管理学，他拿的仍然是工学，只是他因为专业的方向是偏管理的系统工程，导师是汪应洛老师，那么汪老师呢算是国内第一批的管理学这个领域这个方向的博导，所以呢我们一般有时候也就这么说，说这个是我们在大陆的首个管理学博士。

如果再往外去延伸出来，前两年呢我们在杭州，在浙大有一个会，当时席酉民老师也来了，恰巧呢那个会在浙大的公共管理学院，让他们从美国请了一个华裔的人口学方面的一个学生，名字叫Li Nan，他也是从西交大出来的，他的导师是蒋正华，也是前两届的中国人大的副委员长，因为他是农工民主党的。然后这个Li Nan呢和这个席酉民老师呢两个人实际上是同时拿的学位，他拿的是人口系统工程的这个专业的博士学位，席酉民老师拿的是管理系统工程的。那么人口研究，有的时候我们讲他是经济学的，因为西交大那个时候的博士点只有一个叫管理科学与工程，所以呢蒋正华老师也好，汪应洛老师也好，都是这个管理科学与工程博士点的导师，所以他们带出来的学生实际上都可以归在管理科学工程的一级学科。但是那个时候就是因为没有管理学，所以他们也拿的同样是工学的学位。就我们讲这个概念准确的去讲这个事儿的时候，可能是这样的一个情况，所以真正的管理学独立出来成为一门学科门类，就是跟哲学也好，经济学也好，理学、工学、文学、法学这些平起平坐的，1998年是标志性。

赵向阳： 我觉得你这个讲的非常好，那个就是你能不能那个先给大家一个整体的印象，就说我怀疑，尤其是两个博士生，他们完全没有这个概念，你能不能讲一下这个西交大在整个中国管理学界的江湖地位？就是你可以大致讲讲中国管理学界几大重镇，比如说西交大的江湖地位。

受访者：向阳老师说的是一种思路啊，就是说我从一个点上，把西交大的情况介绍出来。但是我今天早上我把书博的提纲打出来了以后呢，实际上我就在整理我自己的思路看，就是应该从哪儿开始来讲。因为这里边呢我觉得可能涉及到两个问题，一个是商学院，一个是管理学。就商学和管理学呢，我觉得这两个之间呢可能还是有一点区别，为什么？我们国家叫商学院的有叫管理学院的，也有叫经济管理学院或者经济与管理学院的，也有这三个是比较常见的名称，我们学校原来叫经贸管理学院，我觉得还挺奇葩的，因为我们国际贸易还不错，所以想突出这个贸易，把那个“贸”放到这个名称里面，但是我们在一九年头上已经把经济和管理分开了所以呢经济学院、管理学院、商学院、经济管理学院这几个在我们国家都有。

但是不像书博的提纲里面比较一致的统一的用商学院的这个词，所以呢我自己理解呢，就是说这个商学院应该涵盖管理学院，甚至涵盖一部分经济与管理学院，可能经济学院可能跟我们商学院还是有一定的距离，但有些学校里边的经济学院里面还有管理学方面的专业。那么在我们国家我自己这两年也在做一些叫企业史也好、管理思想史也好、管理理论发展史也好等这些方面的一些思考，但是我没有写文章。因为原来我是想开一门新的课程，给本科生给研究生，不过没时间来准备，但是呢收集了一些资料，然后买了一些书，然后现在也在想慢慢做点儿时间，然后把这个整体的这个框架或者思路搭起来。因为在中国的历史上，商学本身一直是排在比较低的这个位置的，市农工商。所以呢商学本身进入到大学或者是要去登大雅之堂，都是比较晚的事情。那么讲管理学呢，号称是1911年泰勒的《科学管理原理》开始，那么换句话说就差不多是90年代末20世纪初，大概这个时候，而那个时间段呢刚好也差不多是中国的现代意义上的大学开始初步建立，而我们讲的管理学，这样一个学科或者是承担了管理学或者是商学教育、研究等主要功能的组织载体——商学院，差不多应该也是在那样的一个时期开始逐步建立。但是呢因为我没有特别的去研究过，就是中国大学商科的历史发展，所谓早期嘛，我理解的就应该是一八九几年，因为号称嘛，天津的北洋大学1895年，南阳工学就是西交大上交大的前身，这个1896年，号称这两所一南一北，是我们国内这个现代意义上大学最早两家嘛，但我没有去了解北洋大学商科的发展情况，因为我在西交大，它是跟南阳工学有一个非常明确的历史渊源，那么南洋工学最早发展的时候也是没有商科的，到了后来逐渐开始建立，大概到了一九一几年，那么我原来在西交大管理学院，我们后来去写院士的时候，我们现在大家都是想把这个历史稍微往前翻的越早越好嘛，所以呢他们也去找到过一些相关的一些记录，说号称在老的交通大学，一九二几年就有这个管理，那个时候叫铁路管理科，这样的一个专业科系的设置，所以呢，我自己想一定不会只有交通大学有管理的学科，一定有其他的一些学校里面也有管理呀、经济呀、商科呀等等一些专业，那这个呢肯定要从不同的方向去找寻。

赵向阳：我插一句，我觉得你刚才讲的这个历史很有意思，就说你去看那个清教徒的文物，这是一本美国的管理文化史，你看美国的管理，你会发现现代意义上的美国管理，事实上就是从铁路管理发展起来的，这是第一个需要大规模的协调运作的，所以我觉得你说的铁路管理真的是很大的一个亮点，你接着讲。

受访者： 我稍微拿向阳老师的话再稍微说两句，就是说因为这个工业革命之后，这个工厂化的生产使得我们的生产组织规模扩大了，各方面都需要协调工作，那么管理当中的一个很重要的职能就是协调嘛，那么无线电铁路发展起来了以后，那么更大的、跨时空的一个协调，就变成了一个我们必须面对的问题，所以在生产力发展比较领先的这个国家，英国也好，美国也好，率先孕育出来这样一个学科、这样一个领域是有历史必然性的。那么在中国的历史上，同样也是这样，比方说清末我们被动挨打啦，然后要搞洋务运动、搞大学，去培养高精尖的人才等等，我们开始也去引进一些培养人才的模式方案，就是我觉得如果我们去找相对比较严格意义上商科的历史渊源的话，可能应该是能够找到19世纪末20世纪初大概的那个时期。

但是那个时期跟我们现在所讲的这个管理学呢，可能内涵和外延上可能又有点不太一样，那么我早上还在查这么几个东西，因为今年是建党一百周年嘛，这个去年开始我们就开始学习党史、新中国史、改革开放史、还有社会主义发展史，然后我就在算那些年份，那党史呢很简单，1921年。当然还有一个前史嘛， 就是说在这个筹备建党的这个过程当中的一些早期的工作，包括新文化运动等。然后新中国史，标志性的事件呢是1949年往后来说，那么我们现在来去讲中国的管理学发展史的话，那肯定很自然的也会讲到一些前面的，但是比较多的可能还是考虑到建国后的。

但是建国前和建国后呢又有一个很大的制度上的变化，就是因为建国后我们是学苏联的。而民国那个时候大学里面的商科，他们学的或者是主流的东西和建国后我们很长一段时间在计划经济条件下执行的系科设置、教学体系、教材、师资等苏联那一套有很大不同。因为我是81年入学，那个时候呢实际上我们是没有什么书的，没有什么教材的，我们那个时候有几门课程，比方说我们现在给商科的学生或者是管理专业、工商管理专业的学生会上一门叫生产运作管理，或者叫生产计划管理，但那个时候我们叫工业生产计划、或者叫计划组织学，这些东西呢都要做成一个既定的、模式化的，这就是从苏联来的。

到了后期84、85年，我们才开始接触到一些相对来说就是欧美的一些课程，比方说我们现在讲的OB、组织行为学，那时候我们用的名称还叫管理心理学；比方说我们现在讲到marketing市场，那个时候我们名称不叫营销学，而叫市场学，类似这样。那么再往后，学欧美的东西可能就更多一点。

赵向阳：你们在80年代初的时候有没有学鞍钢宪法？

受访者： 那个时候已经不是特别突出了，所以呢中间我再插一小段，补一下刚好向阳老师讲的这句话。就是说49年到77年恢复高考，刚恢复高考的时候呢，大多数学校里面的所谓的商科好像是没怎么听到过的，比较多的是什么？比方说人大，他有国民经济计划，就是偏宏观的。像金融、财政等现在我们会笼统地把它们放在经济学学科里边的这些专业呢，实际上在文革中间，尽管没有完全停办，但是呢他培养这个方向跟我们现在讲的商科的还是不太一样，为什么呢？因为他比较多的还是为计划经济的那一套东西来服务的。我自己理解我们讲的这个商科呢，应该是偏工商管理，而工商管理的主要研究对象是企业，它的边界就是企业这个整体，再大一点呢可能就到行业，不能再大了，因为再大了就到了宏观的国民经济的层次了，这可能就应该回到经济学的这样一个学科里了。

赵向阳：你这个说的很对，就是国民经济计划是从苏联学的，而苏联呢实际上在很大程度上从德国借鉴的，借鉴的德国的经济系，就是偏宏观的，另外一个就是企业层面的。ok，你接着讲。

受访者：我再补一点恢复高考前的一些情况，到文革之前我们国家的大学还相对比较正常，都是培养人什么的，还比较正常，那个时候呢，还是有一批人学俄语、或送到苏联去培养的。所以呢在那个时候呢，这个偏经济的、偏经济管理的、偏经济计划等方面的专业学科还是正常的，但是呢，关于微观管理的、企业组织运作的不是很多。回到刚才前面赵老师提到的我在西交大的时候汪应洛老师，他是工程院的院士了，他当时呢，五十年年代，从上海的老的交通大学毕业的时候没有直接到西安，他是从上海去了哈尔滨工业大学，那里搞了一个研究生办，是苏联到那边去办了一个研究生办，跟他们苏联合作。学什么呢？学生产组织。这个生产组织呢就是我们现在讲到的生产运作管理、生产运营管理或企业运营管理这些方面。苏联讲微观的东西，比如说布局、流程、节拍，相当于我们现在比较狭义的工业工程。

赵向阳：我再插一句，就是你看汪应洛老师那个时候，他们在学的那个重点实际上是在生产运营组织管理、工厂管理计划，而这些是现在商学院根本就不教的，也就是凡是那些能把手弄脏的、下基层、下工厂的就完全不教。ok，你接着来。

受访者：很快呢就到文革了，那么文革的话，全国的经济、企业什么的都不是特别正常了。再一个呢就是大学的教学，已经不在原来这个套路上。就我自己理解，因为十年也是挺长的一段时间了，里边儿应该还是能够挖掘出来一些故事，人物、文物之类的东西的。然后继续说改革开放以后，77年开始恢复高考以后，那当然肯定是原来有基础的学校、有基础的专业肯定是率先恢复，当然那个时候呢国家百废待兴嘛，那么所以呢比较急用的一些学科，学完了以后马上能够去帮助解决生产技术等方面的一些问题。

关键是什么呢？我们讲的管理学在当时的国内是没有这样的门类的，所以我上大学的时候我学的那个叫工业管理工程，同时还有比方说国民经济计划、比方说劳动人事管理，诶他们都叫管理，但是又没有管理 这个学科，那它归在哪儿？所有的跟管理有关的专业都分散在各种不同的学科门类，比如我学的是工业管理工程，那就是在工科，然后国民经济计划，那就是在经济学，还有这个农林经济管理，或者是农民管理等等，那他有可能在这个农学里面。所以呢所有的跟管理有关的专业都是分散在不同的学科门类里面的。所以刚才我讲了九八年我们独立设立出来管理学这个门类也是有了这么大概20年左右的时间了。那大家都知道这个管理学（在国外他们叫商学也好），它仍然是有一个相对跟其他学科不同的一些特点，所以呢各方面争取了以后，也当然也做了很多很多的基础性的工作，然后呢把所有分散在其他不同学科领域里面的专业给汇集起来，然后做了管理学的门类。

赵向阳：我再插一句，从78年到98年，这20年间，可以称作中国管理学或商学院的前史，你觉得这段前史里有没有明确的阶段性，就是你能看得出来他有阶段性的一些转向，不管是师资、教材、还是评价方式等，那时候大家怎么做研究、怎么升职称？对，就是这个东西你觉得还能分吗？

受访者：20年肯定是能分的，但至于怎么个分法，肯定不同的人有不同的观点，我还没有特别地去研究过。就是从我自己的实际感受来说，可能是这样一个情况：刚恢复高考招生培养人，国家特别需要人，那个时候尽管规模不大，但师资也好、校舍也好、工科还有实验室，这些资源条件实际上是比较差的。所以我在不断地学这个专业，我就可以看到我们这个学院里边不断的进一些老师。现在博士毕业去到一个学校去应聘还不一定能聘得上，它要看你跟导师做过一些什么项目、发过什么样的文章、影响因子有多多少、然后还可能要跟别人pk一下，然后还不一定能够当得上。但我们那个时候隔壁旁边的有一所学校，西安理工大学，以前教陕西财经学院，他的本科管理学专业实际上比西交大要早一点，所以呢他们毕业的比我早两届的本科生到我们学校（西交大）这儿来做老师，那就是本科毕业生给本科在读生上课，在那个时候都是很常见的。所以那个时候比较多的学校里边，我们叫商学院也好，叫管理学院也好，它最主要的职能就是教学，所以不太牵扯刚才向阳老师讲到管理学的研究，为啥呢？因为党国缺人呐，你能把学生给我培养出来，对吧？每年给我弄几千号管理专业的学生，那这就是你最大的贡献，但是同时呢，还是有很多研究的工作是需要去做的。我自己理解呢，因为管理学还是一个实用学科，那就是我们讲的学以致用，它还是有服务对象的，那商科嘛应该是为企业服务比较多一些，那么其中呢，在我们国家的一个很重要的服务方向就是政府。在那个时候呢有两类比较具体的跟经济和管理有关的部门，一类是综合类的经济管理部门，主要有两个，一个是叫纪委，一个是叫经委——国家纪委、国家经委，各省市呢还有省市一级的纪委、经委，这是一类综合性。纪委就是我们现在讲的发改委，经委现在相当于差不多经信委。那么另外一类呢就是专业型的，就是各个部委机械部、水利部等等，那么它是有专业领域的，那么这些部委综合性的也好，还是这个专业性的也好，他也有很多很多的问题需要去研究，比方说我要做规划、比方说我有一些大的项目要去做一些论证或者什么之类的，因为在80年代初我在的西交大会参与一些很重要的工作，比如长江三峡工程论证，那专业性的比较多的应该是水利呀、发电呀、防控、地址、建筑，那些技术性的学科，但是它里面也牵扯到了一些经济和社会的问题，其中涉及到比方说爬高——大坝的设计会影响到他库容的大小，影响到它的发电量，但是同时它会影响到他淹没区域的大小，影响到移民的规模多少，然后配套的我们要去做这个移民搬迁的财政的预算，以及防洪防汛防地质变化，比方说它有可能会引起地震，那就需要相应的财政方面的补贴、预防配置。

所以呢国内有一批学校的管理学院也好、经济学院也好，不同程度的参与过一些国家部委牵头做的一些工作，这是一个方面，就是说政府机构，因为在我们国家特别是早期的计划经济时期，当然现在仍然是这样的，就是政府的力量仍然是非常非常强的，那么他的这种需求也好，或者是他可以配置的资源所产生的影响力也好，都是一般的企业没办法跟他去比的。所以包括我们现在去申请基金项目，都是国家财政的钱嘛，如果他不拿这个钱的话，我们才可能去考虑申请其他企业，或者我们自己去立项什么之类，这是一个很重要的方面。那么到后期，因为八四年我印象很深刻，那时候我还没有毕业，国家有个叫《关于经济体制改革的决定》说，我们要从原来的计划经济走向这个有计划的商品经济，然后才可以创立一些企业，特别是民营企业、乡镇企业，国家才开始给这些企业一些空间。那再往后呢，就是有计划的市场经济，这就到了80年代的后期、90年代的初期才开始。所以呢企业的需求实际上也是有的。

赵向阳：我就插一句，就是中国不是有那个九三派的、九二派的，还有那个八七派的嘛，就是企业界、民营企业嘛，所以像那个张瑞敏都是八七年折腾的，所以这个84年、85年提出了有计划的商品经济，然后呢私营企业才开始搞起来，所以就是可以这么讲吧，八七年以前所有的管理学研究都是服务于国民经济建设的，而且是大的工程，比如像席酉民老师参与的三峡，但现在管理学院根本就不参与这方面的事情了吧？就是如果说国家同样还有比如高铁之类的工程，我们现在的管理学院、商学院没什么关系了吧？西交大有人参与吗？

受访者：肯定有。但是呢因为高铁本身不是西交大的特长，给两位小同学普及一下知识，这个我们国家的交通大学里边呢，唯独西安交通大学跟交通的关系是最弱的，即便他挂了交通。上海交通大学跟海运联系是很密切，它里面有船舶等专业，西南交大和北方交大跟铁路运输关系是比较密切的，然后唯独呢西安交通大学，因为他从上海搬到西安去的时候呢，航运的东西怎么搬到西安去，对吧？然后呢本身铁路那块儿呢有很多搭建的试验条件、设备呀之类的，还没来得及搬，那后来就说那就算了，因为那个时候也不是特别稳定，所以就留在上海。

刚才向阳老师插话所讲到的是它的一个现实，这个情况呢有一个很大的变化——就是说我们大学孵化出来了一些人才，现在开始呢有很多单位机构、一些研究院呀、设计院呀、或者一些部委企业它底下下设的一些机构，它的翅膀就比较硬了，什么硬了呢？就是说，黄秋莉你虽然是学哲学的，但对不起我有一个哲学的问题，我不一定要请教你了，为什么呢？因为我们自己招了有哲学专业的博士，我们还成立了一个研究所，我们那里有几个经过实践锻炼过的、知道哲学在这个领域里面可以怎么去用的人才，那我干嘛一定要去找大学里边找不太懂实践的人呢？那这里我举一个席酉民老师曾带过一个在职博士的例子，他已经拿过工科的博士，是当时高工部在西安有个研究所的所长，已经是教授级的高工了，但是呢他还是想学习管理。然后呢就又找席酉民老师作博导，他后来去了北京的一个研究院，然后他自己在做很多工作的时候呢，他自己有自己一整套的思路，比如说席酉民老师你很厉害，但是你可能掌握的是一般管理的东西，我现在不管那些东西，我现在就要研究航空航天它的具体应用，所以他的这个研究院里面呢有一帮人是研究那个硬技术，有一帮的人呢是研究经济呀、技术呀、评价呀，分析呀等等这方面的东西。所以呢现在这样的一些机构或者是分散在不同的专业领域里边的、产业里边的、甚至企业里边的管理专业的人才，现在应该也是比较多了。我想中财应该也有很多学科有博士后的流动站，那么我们国家不是很早就开始在很多企业里面设立博士后的工作站嘛···

赵向阳：你这讲的有点儿时间穿越了，你刚才那个举的这个人的这个例子是哪一年的呀？

受访者：2000年前后。

赵向阳：ok，就是那个时候呢基本上大家还是很深入地跟行业问题紧密结合起来的，然后我想你接着从八七年开始讲。因为98年呢基本上一设立管理学以后呢徐淑英老师他们就开始先在香港办培训班儿，因为98年我上了研究生嘛，所以我印象特深刻的就是我快毕业的时候嘛，2001年第一次在光华我上徐淑英老师的这个课，就是我就想知道87年~98年之间发生了什么事情？

受访者：好，回到前面，刚才我讲到早期的国内商学院也好、管理学院也好，比较多的还是以教学为主，那个时候你不发表文章也好，你不写书也好，不承担省部级的国家级的这些项目也好，同样也是可以评职称的，那个时候反正工资也不高。那么到了80年代末特别是90年代初开始，为什么那个时候开始有了一些变化的呢？一个是因为中国本身在经济体制改革方面又有了很大的一步的迈进，那么原来的“吃不饱”问题已经基本解决了，所以当时面临的问题是企业怎么才能做大一点、大家怎么生活地更好一点，那么国家呢在政策上也适当地放宽了一点，这个呢我自己理解呢可能差不多跟92年邓小平南巡讲话有一定关系，92年算是第二次开放，有了很多政策的空间。这个政策的空间一个是带来企业本身的活力重新释放，还有一个呢就是国内的商学院，特别是有MBA的学院。早起的MBA学院只有8所，但是很快几年的时间，十几所、二十几所、三十几所就开始。那么MBA的这个培养模式呢就是···

赵向阳：是什么意思？你说中国的MBA是哪一年开始办的？

受访者：90年

赵向阳：90年开始办MBA？那时候就叫MBA吗？90年最早是在哪儿办的？大连理工？

受访者：大连理工呢是跟美国合作办，但是那个时候他是不发学位的，他只是做一些课程的师资培训，他主要是给老师做培训的。

赵向阳：对，这个我知道，90年就有一个呀？

刘书博：对，我记得那个中欧好像是这八几年就有了吧，好像在北京，中欧国际工商学院的前身。

受访者：对，那么因为我们国家的教育这块儿呢是国家一直控制的，到现在为止就是反正你不参加国家考试，你是不可能在中中华人民共和国拿学位的嘛，所以呢长江商学院的那个MBA都是北京都办了好多年以后，后来国务院学位办才认可，把他纳入到MBA这条线，那时候他发的文凭在学信网里面才能够查到。这个是后话。80年代中间有一件蛮有意思的事情，那个时候呢因为我们国内的管理学科不是商科，整体都还比较low，西交大的汪应洛老师是比较敏锐的一个人，他抓住了几个比较重要的机会，一个就是把系统工程钱学森洞见，他们所做的一些工作，那么从军事或者是国家的宏观的规划方面开始放往区域发展、行业发展或者是项目评价，这个就发展出一个学科的方向，就是我们现在讲的管理科学与工程，就管理学里边第一个的一级学科。但是刚才我们讲到的偏工商的领域里面呢，我们仍然是相对比较散的、比较弱的，所以在80年代早期的时候，刚才向阳老师提到的大连理工大学，邓小平跟卡特说了说我们能不能送人来培养或者是你帮我们来培养，在大连理工大学呢搞了一个中美联合培养的基地。所以当时国内有一批大学送了一些青年老师到那儿去学了一些课程，但是这些课呢当时是比较分散的，比较零碎的，我的大学本科的那个班主任当时去大连学了一门质量管理，那现在讲的叫全面质量管理也好，或者六西格玛早期的雏形也好。等他回来之后就开设这样一门课，所以不是所有的学校所有的课程所有的老师都全面地去接受了美国的培训。所以呢就碰到了这样的一个机会，就是加拿大跟中国政府有一个教育合作，这个合作呢是以加拿大的国际开发署出资资助加拿大的一批比较好的学校和中国的一批比较好的学校，这里边就不是一个班了，不是一个学校了，是一批加拿大的学校和一批中国的学校，而且都是相对都是top的几个学校，对口一对一，有一点像现在我们讲的帮扶的这种性质。然后呢对方的老师到我们这儿来帮我们上课，我们这边呢派老师到那边去学习，有的呢是学课程，有的呢去读学位，有的呢去进修做好研究。这个项目在不同学校持续的是时间不一样的，有的时间长一点，像我们西交大跟他们的合作后来不断的续签、一直延续到了05年还是06年。

赵向阳：我还真不知道加拿大跟中国有这样一个（合作），那是不是韩巍第一次出国去加拿大就这个项目吧

受访者：对对是这个

赵向阳：那都哪些学校？有没有the western，阿尔伯特？

受访者：我们学校对口的是阿尔伯特大学。

赵向阳：对，我想起来了。韩巍应该就是在阿尔伯特那个大学，对，可能待了一年左右，你接着讲。

受访者：他那次去可能是半年吧，我去的比他更晚一点，然后费用就不多了，所以我只去了三个月。那么这批学校的这个合作实际上就奠定了国内的比较多的学校里边上课的一个很重要的基础。但是这个呢我后来我自己来评价这个事情，实际上呢有几个重要的作用：一个是开放的环境，国外上课人家是这么玩的，这个我们知道的，他们来给我们上课，然后他们会给我们带一些资料，教科书当然是全英文版的，有的时候呢他们会给我们赠送一些资料，我记得我们学院里边缩微胶片，那大家现在基本上都是用网上的这个数据库了，是吧？完了以后直接pdf文件格式，然后完了自己电子浏览或者是打印出来。但那个时候没有，而是用像情报人员那样很小的胶片一卷一卷弄过来，那比方说赵向阳《大变局下的中国管理》这本书，我们把它拍成照片，然后还有一个胶片阅读机要放大，把它放在一个投影屏上面，就像幻灯机一样的，然后我们才能够读，类似于这样的一些设备。那么这个时候呢就是我们国内开始比较全面系统地接触到一些西方在商科教育实际操作的一些东西，当然也有一些学校后来包括像清华跟美国也好，像同济跟德国是有渊源的，等等吧。一些学校他们也有了其他的一些渠道，我们就了解到国外商科跟我们原来不是一回事儿，不是这么玩的，就开始考虑我们怎么来改造中国的商学院···

赵向阳：我就插一句，现在已经9:40了，我是建议咱们讲到10:00，咱们今天晚上呢只讲98年之前的，就是你争取在20分钟内把98年之前的这个线索给我们，比如说中国第一个改名字叫商学院的，你印象里面是哪个学校？然后你就讲一下98年之前的这些事儿，我估计你这个至少得两次访谈才能梳理个大概。好，我我就先讲到这儿，你接着讲。

受访者：为什么可能我对这方面的情况稍微比向阳老师了解的多一点呢？就是说因为刚才我在这西交大的管理学院的创始人叫汪应洛老师，我们是84年创立学院的，他在北京中央的这些部委里边比较有影响力，比如说管理方面的一些东西，他们经常会找他去，包括国家经委呀、国家纪委呀、国家科委呀、教育部呀、国务院学院办呀、国家级经委呀之类的，所以呢他有很多兼职的头衔。其中一个兼职的头衔就是国务院学位办，他不是有这个学科评议组吗，他是这个管理科学与工程学科的召集人，所以呢早期呢他跟一批老教授们一起商量，就讨论说中国的商科肯定要重新来去规划、构造，然后呢他们就组织了对国外的一些考察和研究，然后提出了要办中国本土的MBA，不是全盘去办美国的，但是美国可能会作为我们的模板，因为英式的MBA和美式的也还不太一样嘛。所以呢在80年代末呢，就开始在酝酿这个事情，然后给教育部建议。

在以前是没有专业学位的，所以也没有专业学位和学术学位之分，MBA是第一个专业学位。而对这个专业学位这个东西来说，当时在教育部在国务院办里面实际上有很多人是不认同的，所以他们做了一些工作以后叫“试办”，所以1990年的时候试办MBA，第一批我印象里是七八所学校，第一批很多学校当年都没有来得及去做宣传，然后就去招生，西交大第一批招了10个人还是11个人，第一个班。去年2020年9月份我回西交大，然后我们办了一个小的庆典，就是西交大MBA办学三十周年。

第一批招生情况比较好的学校到了第二年1991年才算正式开启了第一个班，但那个时候大家都还不知道MBA是什么，也不好招生，我们那些老教授们就到省里边儿那些部委，或者是大的国企里边苦口婆心的去给人家去做工作，说你们这儿有大学生，然后那个时候我们叫第三梯队培养人才，外语还要好一点的，因为其中我们有一部分课程是要请外教来上的，然后你们送到我们这里，我们还不要学费，也不需要脱产，他们可以一边工作，用周末或者其他的时间来上课，拖着拽着把人招起来。

MBA真正成气候，差不多到了1994、1995年。在不同的地区情况可能不太一样，南方经济开放一点的地方可能会好一点。 我自己理解，92年邓小平南巡讲话释放了企业活力。

伴随着中国商学教育自身的发展，原来我们注重本科，规模也有一点，但是研究生那个时候规模是不大的，博士生就更少，但是专业学位这个类型、这个渠道开了以后，情况就不一样了。具体来说有什么不一样呢？我不知道现在中财每年能办到什么样的一个规模，但各个学校一般都会给这些MBA有比较多的留存，早期是不收学费，后来开始多多少少收一点学费，几千块钱万把块钱，然后因为规模小嘛一般都会去跟学校争取比较好的条件，说校长，你看我们这MBA现在刚开始办，这个一穷二白的，我们能不能给学校交个 50%，我们留50%，然后很多学校是收50%上下，有的学校收的少一点，因为学费少，收上来可能总的也没有多少，有的学校可能条件差一点，稍微多收一点，反正那留钱保本儿肯定是够的，用来买教材呀什么的，给老师付一点讲课之类的，那个时候讲课费成本也不是很高嘛。那个时候入学肯定也是要考试的，MBA那时候的考试是各个学校自己考，自己出题，自己组织自己划分。

但是后来这方面的现实需求变大了，考的人多了。过了十年以后MBA已经开始有一些规模了，有一些学校招的人数已经比较多了，2000那个时候，我估计清华可能两三百规模了，当然一定是有好的一方面，也有各方面的问题，包括有人会说了MBA发钱给老师，每年年终可以发几万，哪来的钱啊？这么多钱，里边儿有什么猫腻呀？然后学生他们的论文怎么样？所以那个时候MBA已经有一个教学指导委员会了，头两届就是王路老师，包括我硕士导师李怀祖老师，他们做这个组织者的秘书长。后来说那么我们走一个正式的程序吧，就是把这些所有的学校做一次合格评估，合格评估过就说明这个学校的MBA培养是符合质量要求的，他发这个学位就应该被认可，大家不要再有非议的。因此MBA的评估实际上主要是要避免这样一种负面影响，当然也是为了促进一下学校，因为后面已经有了更多批学生，那么这样的话也是给后边的一些学校树立一个样板。所以2000年，教育部学位办委托北京指导委员会组织专家把国内的头几批的学校，大概有那么二三十所，做了合格评估，然后就公布说这批学校是合格的。

然后各个学校里边的商科借这个机会再次得到发展。因为2000年前后李兰清那个时候在负责教育，又搞了一次大学扩招，本科扩招以后同时会带来研究生培养规模的扩大，那么也就产生压力，因为每年学生多了，那么各个学校里面的师资规模或者是高职称的可以带研究生的老师的规模，也就比较多了。所以研究生扩招里边比较多的是不需要重资产的，这些学科投入少，然后我只要招几个人，课能够开的起来，我有教室学生有地方住就可以了。不管是教育部的学校或者公立的学校，民办的学校更是这样。

所以我自己理解，就是90年代初这个商科教育类里面比较重要的一个发展，可以说是这个MBA带动的，那么2000年以后商科的发展，很重要的是大学扩招带来的。

那当然从学生和人才培养的这个角度上来说，中间可能还要穿插一个刚才向阳老师讲到的问题，就是我们讲的管理学研究的问题。以前我们讲管理学院或者商学院里面，研究比较多的还是服务于国家、企业这样的一些比较客观的需求，真正去申请基金的那个人就自己想的一个题目，然后去申请，给我点儿钱，那个时候钱很少，几万块钱，就管理类的这些项目，能够拿到个6万、8万块钱，这已经算是比较多的了，不像现在几十万甚至大几十万，是吧？那么所以那个时候大家都不会特别去挖空心思去想一个什么样有理论意义的、有理论价值的、有理论创新的题目去申请，大家比较多重心还是放在人才培养上。

赵向阳： 我插一句，就是这个企业就是民企它对于管理的需求最早是什么时候？就是说有企业来找你们，而且当时主要是营销方面的呢？还是生产方面的呢？还是内部的这个组织管理呢？这个问题主要是什么时候开始有这种需求的呢？

受访者：应用性研究是比较分散的。比方说国企，大概可能是这么几个领域，一个是技术改造，要做项目论证，这个我们现在通常称之为技术经济评价也好，可行性研究也好，这个在八九十年代的时候曾经有过一段时间蛮热闹的，就是说这个企业我怎么够申请到钱，或者企业准备去立项的时候，他们一般都会请学校帮他们来做一些论证一类的。

还有一些企业就是准备练内功的，你比方说这个质量管理。那么有一些企业质量管理，因为大家一般都不太对外，就是说这个数据我还是有一些保留要求的。

我昨天晚上碰了个事儿，华为不是出了这个鸿蒙的系统嘛，昨天白天它就提示我，他说你这个手机现在可以升鸿蒙了，我前一段时间实际上曾经关注过能不能升，但是它提示我升的时候呢我又犹豫了，为什么犹豫呢？我说我这款手机升了鸿蒙以后有没有谁碰到问题碰到麻烦了，居然真的有人说升完了以后变板砖了，我就不敢升了。那你比方说华为请人帮他们把这个质量体系做了一遍以后呢，比方说给个合格率、一等品率。但都不会对外说，因为有人觉得说好，你这个合格率都提高到99%了，但是另外有人看的就是那个剩的1%，所以有很多企业在那个时候已经开始注意这方面了，就是说关起门来，我们把它做了就完了，新产品开发、设计之类的这样一些东西。

赵向阳： 韩巍90年代的中期，就开始出去给一些公司做一些策划，那时候点子公司之类的，还是挺热闹的。

受访者：还有一个就是，后来民营企业开始发展起来了，乡镇企业有一些做的好一点的也开始有些规模了，开始需要引进一些新的技术、上一些生产线，甚至是买国外的一些东西，但这个相对来说就比较分散。

所以我们现在讲的所谓的纵向项目或者是横向项目，一般那个时候大家还没有一个氛围，说这个横向到款多少，你做的什么领域的，你做的是一个很高大上的一个题目呢，还是做一个很low的、很基础的这样一个东西，那个时候大家都不会这样子去比。

但是到了90年代末，我们国家开始要建研究型大学的时候，那你们回头可以查看，就是什么时候开始提我们国家要建研究性大学，开始把研究科研放到比较重要的位置上。

研究性大学嘛，那你不能光教学，总要做些项目，总要出些成果，论文也好，专利也好，专著也好等等。那么出成果对于不同的学科就有不同的要求。那么工科这种比如说专利也好，知识产权也好可能会相对多一点，那么商科就不太好说了，说怎么创新呢？国家基金委在1996年有一个标志性的事件，就是国家自然科学基金委把管理学独立设计成一个部门，在这个之前只是一个组。80年代管理学基金的项目就开始有，但是规模很小，跟其他工科的那些学科是没法比的。然后1996年正式成立学部，那就跟工学部、化学部、信息学部并列。然后当时朱镕基做了一个讲话，很标志性的讲话叫“管理科学，兴国之道”，《人民日报》还是这个《光明日报》上的头版发的。

事实上设不设立这样的一个门类，然后是不是一个学部牵扯到很多方面，比如财政拨款问题、合法性问题，还有一个很重要的一点就是管理学的这个研究问题。我们过去的研究比较多的是一种经验，或者是套国外比较成熟的模式，比方说这个质量体系认证，就是从国际上拿来的认证，那么这些东西大家都一般认为是一个简单的、一个重复性的工作，不具有创新性。但是当我们讲到研究性大学，去检验成果的时候，那么这个中间就是说我们怎么能够让我们的这个东西能够显示出来呢？

那么2000年后，又有一个很重要的标志性的事件，就是中国经济要开放了，那么除了经济、金融的开放，实际上教育科技的开放也包含在里面，这个就是要跟国际去进行对接。

那时候中国整体经济情况更好一些了，学生数量就上来了，比如清华都在那盘算说，一个学生的学费乘上招生人数，经管学院每年能拿多少钱。这时候每个学校的这些商学院或是经管学院都不缺钱，一般的话学校不给学院钱，这些学校的院长一般也不会说去找校长要钱，反过来经常有一些学校的校长会去找院长说，你们院里的账户里边儿钱好像也没用，我们学校最近要做个什么事情，是不是能够用你们这个钱把我们学校事情做了。

刘书博： 我们中财现在就是像您说的这个状况。

受访者：南开商学院院长李维安就跟我说起，他们校长已经找了他好几次了，他反正就搪塞说这个事情不是我一个院长就能定的，我们要开业务会之类的，要集体决定什么的。

所以相当于学院都有自己的“小金库”了，这个也不是说是一个负面的“小金库”，就是自己腰包鼓一点了，然后就开始考虑把这个钱用在值得去做的一些工作上面，其中包括比方说请一些更高端一点的、大牌一点的、有国际影响力一点的知名管理学学者来讲学、兼职，或者是到我们这儿来挂职做院长等

另外还有一个蛮好的渠道，就是说引回一些中国人才。前面二十多年我们不断往外送一些人出国去学习、读学位，然后留在欧美的一些学者现在已经取得一些比较好的成就了，比方说获得全职身份了，甚至有一些已经做到学科领域里边比较靠前的位置或者说担任一定管理职务了。

赵向阳： 比如像王崇明的学生陈晓萍，陈晓萍差不多就是1994、1995年左右出去的。

今天咱们这个时间点就卡在1978年~1998、2000年前后，中国改革开放以后20年属于中国商学院的前史，最开始管理学还都是嵌入在各个学院的学科门类里，学科门类他们怎么样服务于国家，服务于国企，然后慢慢的随着民企的发展也提出了这方面的需求，然后随着MBA的发展，学院有钱开始反哺学校之类的，这个思路挺清楚的。

然后明天从2000前后讲到现在，就是这20年整个的演化。

受访者： 我推荐几本书：《20世纪中国知名科学家学术成就概览 管理学卷 第1分册》与《20世纪中国知名科学家学术成就概览 管理学卷 第2分册》。它是分不同学科门类的，经济学已经出了三卷，哲学也有，法学也有，理工的都有。

我们一般理解科学家们都是称呼理科类的、工科类的研究学者，但是它反正就是人文社科类的这些也包括在这里边。这两本里面讲了78个管理学者，基本上都是50年代以前出生的这一批。

赵向阳： 有修明老师吗？就是那个王芳华那一代有吗？

受访者： 都还没进去。他们选的时候肯定也是有一些自己的考虑，这本书前面有一篇标题就叫“20世纪的中国管理学”，这样的一个属于综述性回顾性的稿子，然后在他最后七页有大事记，可以根据这里面的内容理出一些线索、一些标志性的事件、人物。这本书从1904年左右开始写，写到了2000年。

2001年有一个标志性事件，中国工程院工程管理学部设立，然后首批聘用的院士32个，这20年前后也是一个比较重要的时期。

另外一本是陈晓田他们写的《国家自然科学基金我国管理科学（1986-2008）》，他原来是基金委管理科学部的副主任，基金委主任一般都是大牌教授兼的职，之前陈思维也做过一段时间，后来吴启迪也做了一段时间。等于陈晓田相当于常务副主任一样的，反正日常的工作也不用他管，他做的时间还蛮长的，从80年代就一直做，做到了2000年零几年吧到他退休。

刚才我讲，就是说国家基金开始设立管理科学组，然后到独立建立管理科学部，然后陈思维去主持这方面工作的时候对管理科学这方面有很大的推动作用。这本书就从基金的这个角度，有很多相关的介绍。

今天我自己也在想，就是说商学院的发展，首先要理清时间轴，那么这个发展我们把它放在多大的时间跨度上呢？

那当然有条件的话，我们要从它的这个起源开始。没条件，比方说我们可以先讲这个改革开放以后，或者说管理学学位设立之后也可以，把事件大的脉络先理出来，然后各个阶段里边有不同的特点。

再一个，除了时间轴之外，我们还要讲一讲事情的宽度。就是说这个学科本身的发展，或者我们叫商学的发展，如果仅仅从学校、商学院角度去讲商学，我自己理解宽度可能窄了一点，因为管理学和商学院可能不是完全能够对应的。比如说中国管理学的这个发展，大学在里边儿起到很大的作用，但是不是全部，一定受到很多其他方面的影响。

比方说这个工程院里边的工程管理学部，现在评院士的时候，我们好多大学的教授、即使是非常大牌儿的教授你都是评不上的，因为你没有工程项目的背景，你没做过大工程。所以中国管理学的这个发展来说，商学院可能只是其中的一部分。反正每年评这个国家奖项的时候，教育部都说大学里面选60%～70%，还有百分之三四十可能包括一些中央部的研究院所，企业可能不会包含在里面，所以说除了大学还有一些别的方面对管理学的发展有很重要的影响。

刘元璋老院士，他的生日是1月1号元旦，2005年的时候我们把他请到西安交大去过，因为刚好第二年是他八十大寿，我们代表我们学院去给他祝寿。那他是一直在中科院，对中国质量管理的发展具有重大影响。那你说中国的质量管理发展，如果只讲这个商学院、大学里边儿的一些东西，那这块儿就是一个很大的缺失。

所以后面再考虑整体的工作方案的时候，一个就是说刚才我们讲的时间轴，可能需要确定放到多大的跨度上去讲。还有一个，就是我们是否要考虑别的一些领域，比方说除了大学之外， 还有学刊学报、社科基金，还有政府机构，工程院等等一些机构，还有现在企业、产业里的协会、学会、商联合会等等这样的一些半官的、半商的、半民间的这样的一些机构，还有一个很重要的力量就是海外……海外的影响我们刚刚已经稍微提到了一点点，就是一些大学也好、基金会之类的，比如我原来在西交大的时候，我们有一个人口研究所，然后长期在福特基金会下来做，所以我们在研究的时候要不要扩展到这么大的宽度上？

再一个就是，商学院要不要定义成比较清楚的对象？就笼统认为凡是管理的、经济的这样的学科都放在里面还是其他方式去认定？因为目前来说，我们国内现在对管理这块儿，定的这个框还是比较大的，比方说从基金管理来看，一共三个处，管理科学工程一个处，然后工商是一个处，公共管理是一个处，这个基本上在陈思维做这个主任的时候搭起来的一个架子，到目前为止也是这样。你看那个复旦管理学奖，他的这个评奖也基本上是这么一个框架，今年我评这个管理科学工程，明年我就评工商管理，后年我就评公共管理，因为都是一个一级学科下面，所以商科可能不能跟国外的纯粹的商科或者说我们商学院简单的去对照。

我按照这个你白天发给我的这样的一个提纲来看的话，我自己理解，就说相应的发展里边有什么样的一些规律，从我们过去的历史上来看，我们能够总结一些东西，然后有一些借鉴的作用。那么有些可能就属于就事论事的问题，即“我们怎么去做”的这个问题。

另外，其中还涉及到了一个问题“谁来做”？主体依托的对象应该是教师。那这个事情就又复杂了一点了，为啥呢？就刚才我们讲那些故事都是讲事儿。那么谁来做这个事儿？谁有资格做这个事情？谁有条件能够把这个事情做好？谁能把这个事情做的影响更大一点？比方说刚才我讲到，丁宇他原来在华东师范大学做过党委书记，后来他去做这个管理科学部的主任，他能不能做好？因为原来你不是在这个管理科学的领域里边的。中间还有一段时间是个同济大学的郭重庆在陈思维之后挂管理科学部主任，然后后来才交给吴启杰。那么郭重庆他原来他是工科院士，吴启军是因为在教育部做了部长，然后这个中国科协都任过职，然后就邀请他来做。

这些比较重要位置的、这个关键的人是什么样的情况？比方说北大光华管理学院，光华管理学院的历任院长基本上都是经济学出身的，比方说经济学家来做管理学院的院长或者管理学家来做这个管理学院的院长，有没有什么不同的地方？

另外再说一本书是钱颖一写的《大学的改革》，钱颖一不做院长以后出了三本书，他分了三篇叫学校篇、学院篇和学府篇，它是属于文集性的，就是把他当政时候的一些重要的文件整理整理，学校篇是偏大一点的、宏观一点的线索，学院篇的有一些可能属于专业领域专业方向的一些线索。那么像这些我们都可以去往一个微观的方面做一些挖掘，比方说商学院的这个负责人、学科领域方向的带头人、骨干教师也好，或者是中青年教师在实际工作里面他也是有实实在在影响的。

所以这么宏大的一个题目，我们要做的工作可能后面就会做的比较辛苦。比方说从里边我们把它再做一些细分的设计，做一些小题目，然后在某一个有限的领域或者方向里边把它一部分深化下去，这是我今天白天考虑到的一些情况。

刘书博：说的很对，应该是这样。因为我这个也是一个学习的过程，还有很多事情我今天听了我才知道，所以是边学习边去设计子项目，把这个题目再清晰化，就像您刚才说的，它包括事也包括人，那这个人可能他又包括做了不同事情的人，所以可以切分出来很多的子项目，所以到时候我再思考一下，修改一下。

赵向阳：研究商学院，这个韩巍实际上一直希望有人去研究，比如说我们现在老研究别人，那你自己是怎么管理的，对吧？我们做这个是最有条件的。做这个我第一个就想起了你，你这个打了一个很好的一个基础，按照时间脉络把大的这个架构基本上说清楚了，然后我觉得再来两个小时，我们就比较有方向感了。

受访者：我补充一下，我在这个事情过程当中算亲历者之一，但是这个过程当中还不是核心人物，那比如我们我的几位老师，他们不仅是亲历者，而且在这个中间他们做过一些很重要的工作，其中有一些人已经过世了，那么还有一些人健在，所以今天我曾经考虑过一件事，就是说你要去做一些抢救性发掘性工作，去找一些已经七老八十的那些人，让他去做一些这个回忆、访谈。比方说赵春君老师，再比方说MBA的教育指导委员会头两届冯唐老师等。像这些代表性的这些人物我们要不要去做一下？

赵向阳：我怀疑他感兴趣的事实上是中国商学院怎么走上一条异化之路。

受访者：还有一个，就是目前是院长的这些人，其中有一些稍微年纪大一点的，现在还有一批商学院的院长很年轻，40刚出头，甚至三十几岁都有可能的。那么这些人的特点对商学院的发展可能又是另外的一种思路，一种想法。

赵向阳： 最早的提纲，我看了以后我觉得这个问题太多，所以后来我就建议他分为三类，一个是50岁以上的，一个35岁～50岁的，一个35岁以下的，我提供的是50岁以上的，但是你刚才提的是说70岁以上的，像徐少林老师、马老师这样的，都是七十多岁以上了，如果他继续需要这方面的话，再找你帮忙。

受访者：有几个我觉得还是蛮有意思的问题，一个是我们从过去教学型商学院变成研究型商学院，那么这个中间又发展出来其他的一些力量加入到这个里面，就是不光就是商学院单独在里边儿唱独角戏，中间出现了很多的促进的、干扰的因素，我们现在的商业学院大概走到了这样的一步，比如说赵向阳老师讲是一个畸形的、不正常的，还是一个有褒有贬的这样的一个情况，但是反正这是个现实，我们也没办法去绕开它或者是回避它。

刘书博：而且从最开始就是比较国家导向，国家的力量非常的重要，几乎在决定性的影响管理学商学院的这个走向，所以现在我看国家政府又在倡导说把研究做在中国大地上，好像这个又是一个非常重要的转向，也不知道未来是到底会怎么样。

受访者： 但是，我自己的理解，有很多提法一开始是比较模糊的，就是说领导脑子里边儿想的和他说出来的这样的一个概括性的东西，他会说一个稍微更宏观的或者层次更高一点话。那别人在读这个话的时候，可能又加上其他的理解，那么到了基层去做的时候，那可能也就会有很多很多的变化。

# 受访者31

HQL：学长先介绍一下自己的学术经历吧，就是学长也是硕博连读项目上来的。那本科的时候也是做管理学相关的吗？

YWH：对，本科的时候可能偏管理工程一点，因为我在北邮嘛。然后我大四就跟着于老师开组会什么的，其实已经开始接触这个东西了。

HQL：那是什么契机？你是提早就联系上这边了是嘛？

YWH：对，因为离得比较近嘛，我夏令营完了就联系联系老师了，老师说，“啊，你就在那儿，离得也比较近，然后就有个空就过来什么的”

LMN：那个时候你为什么要转，就是从管理工程转变到OB

YWH：跟这没关系，我就是想换个学校了，我就想换个学习环境。

HQL：那选择老师这个方向的时候有什么考虑？

YWH：没有什么考虑。这个怎么说，这个跟学术无关吧。因为当时夏令营的时候，我们也没有你们比较完善的夏令营制度，我们是第二届，然后就去了仨老师，一个林院长、一个我老师，还有一个周老师。所以见过了的就这3个老师，最后就选了于老师。但是有名额的不止这三位哈，但是当时好多老师都没见到。

LMN：其实我们也一样。

HQL：我们老师很多，但是让你面试的时间非常的仓促，基本上只能面一个人。

LMN：当时为什么要读硕博连读，就是已经把学术道路给选好了？

YWH：没有，我说实话，我一点都没有想要做学术什么什么的。一点都没有想，就是来了之后既来之则安之嘛，就跟着做呗、跟着走呗，就也没想那么多。

HQL：你有选别的学校吗？

YWH：也有选别的学校，但是别的学校都没有成功，人大什么的最后没有录。然后我在北邮其实也找好导师了，也留条后路。但是当时大三吧说实话，对这个做研究什么的还没有概念，包括研究方向，什么经济方向、管理方向的。我在北邮找的是偏经济方向，也没有什么完整的概念，就到时候再说吧

HQL：男生嘛其实还是有这种概念，就是想着说读个硕士然后出去找工作，这种思想的人还是比较多的

YWH：是，当时也有人跟我说过，读个博士以后不好找工作，以后这选择就窄了什么的。然后后来我算了一下，其实咱们硕博连读读完年纪也并不是很大，这个确实还是比较年轻的，我说其实也没有太多的限制。当时其实夏令营的时候也跟于老师聊过，他说，到时候你选择还是由自己的，也不是说它是一种限制

HQL：那你有没有想过，你要付出很多的努力

LMN：你刚刚说毕业时你的年龄比较小，是建立在如期毕业甚至提前毕业的基础上，就是你对这个还是很有信心的样子

YWH：当时我感觉我是有点那种出生牛犊不怕虎，没有设想过有多么难，就没有设想过有这么难的，就当时就进来了嘛，就不会想它有多么多么难的。

HQL：那时候对学术界有什么想法吗？像在大三的时候你会觉得对做研究有什么初印象吗？

YWH：完全没有感觉，因为我说实话，我在北邮那个学校嘛，北邮的其他学院可能还好，经管学院是一个特别偏实践的学院，那边学院的老师吧在外面搞事业的特别多，因为跟技术界走的也比较近，然后搞技术搞实践的比较多，而且那边老师上课的时候也会催促大家，大三没事的话就多出去实习，就这种氛围，然后什么创新创业的氛围比较强。学术氛围淡一些，可能最近他们学术氛围好起来了吧，反正当时我觉得没什么学术氛围，然后我参加夏令营的时候来咱们这边，当时是林院长嘛，感觉这边和那边完全不一样，这边大家都在发文章，就感觉大家都在做这些学术的东西，做那些实践的就比较少。

LMN：那时候有什么感觉？

YWH：其实参加夏令营的时候听着他们说我还挺压抑的，就感觉氛围比较压抑吧

LMN：那为什么还要留下来。

YWH：但是确实于老师比较好，那会是于老师做讲座嘛，还是感觉这个老师就是想法都很透彻的，当时就感觉应该是对学生比较好的那种，我觉得这个老师还挺不错的

LMN：所以之前其实之前对于老师这块还是有想法的，就是知道导师如果对学生好的话……

YWH：是，当时觉得这个老师还是可以的

HQL：那我们来了第一第二年就开始上课嘛，就感觉我们的那个课程安排也不是特别的……

YWH：我们比你们惨多了。我们当时是在沙河住，好多课在这边上，我们还要天天早上坐校车过来上课，然后再坐地铁回去，跑了一年。第一年特别辛苦。每天早上6:20的校车，5:30就得起床，而且那边还有课。不过我觉得那边环境好一点，那边适合做学术。那边图书馆那么好。那边确实比这边适合做学术，就这边太浮躁了，周围这四道口什么的，那边就周围什么都没有，比较清净。

HQL：那第一年上课的时候我印象比较深的就是刘XX老师的文献综述，其实当时给我留下了很深的阴影，就是因为她一上来就要我们写文献综述，那你肯定就要确定一个研究领域，这对我来说是很痛苦的，那对学长怎么说？

YWH：唉呀，确实我觉得她那个课可以放到下学期上可能好一点，那课一开始上我确实有点懵，尤其是我们刚来，不过我幸运的是什么呢，我大四跟着老师这边还有点基础，就基本上那会老师已经给我说先去做职业生涯吧，就基本有一个自己心里面的方向了。所以我感觉我上那课的时候还是比较符合的，但是你们刚来的话，我觉得那个课确实调整到下学期会好一点，就是先有个探索，然后再去上那个课，而且她那个课讲的吧，没有铺垫，直接上来就内容分析了。

HQL：所以我一上来就特别懵，想着一上来就要选个方向，然后还要几周一汇报，就快崩溃了。

YWH：对，你想他那个内容分析，首先你基本上得读到一定量的文献，你才有感觉。

HQL：那就类似相关的课程，学者有比较印象深刻的嘛？就可能不是给你带来一个不好的感觉，也可能是带来比较好的感觉的。

YWH：现在回想起来……。首先说对我比较有增益的课吧。对我比较有增益的课就是那个姜老师的课，他那个课还是可以的，最起码你每周能跟着他读好多文献，因为他也是在人力领域，他介绍都是心理学的理论嘛。然后其实我觉得战略课，就林院长和葛书记一起上的硕士的课，其实那个课也挺好的，可能上的有点压抑，那个课可能上的有点压抑，但是其实那些经典的文献我觉得现在回想起来，人家那些文献确实挺好的，因为都是老文献嘛，现在的这些很套路化的文献，你读完一篇也没什么感觉，没什么增益，但是人家那些老文献还是思考的蛮深的，然后那些文献设计的问题、讨论的东西虽然可能讲的比较啰嗦，可能有的观点也过时了，但是确实发现人家那会儿的思考蛮深的。你看都是七几年，五几年，还有二几年的。

LMN：这个都能记住呀，我感觉我都还给老师了哈哈。

YWH：我只是感觉我只是记住当时的那个感觉，每次的文献都是相矛盾的观点，这个说这个好，那个说那个号，这个是渐进的，那个要激进的，而且他们是没有实证的那种，那种是我觉得最能体现我们作为社会科学、作为文科的闪光点，就不是说被一些数据所套住，你必须按这个数据说话，怎么怎么样啊，就是通过我的思考。虽然可能思考出来不一样嘛，但是那种碰撞还是挺好的。

HQL：那感觉不太好的地方呢？

YWH：不太好的地方就是上课的方式嘛，怎么说呢，葛老师还会讲一讲，那会儿林老师就完全成了抽查，让人倍感压力，而且可能会让你抵触那个课。其实我觉得那俩老师不用就这样穿插着来，其实应该每人上半学期，然后能把他教的那部分串一下，把这些东西提纲理出来可能会记得更深一点，能学的更多一点。这样穿插着其实这个老师不知道那个老师是怎么讲的，反而有点散，感觉每堂课和每堂课都联系不起来这种感觉。而且我们那个课当时还考试了，英文试题。当时还要复习还要考试

HQL：我记得印象比较深刻，因为我们是跟硕士同学一起上的，然后林老师就特别喜欢说两个字“垃圾”，然后我们室友做了个比较好笑的事情，就是把垃圾袋放在我们的门上，就这种话语就非常深刻地印到了我们的头脑里，现在就是一提起林老师，就感觉自带语音，“你们都是垃圾”。

YWH：而且还有就是咱们学校的很多课其实……我出去参加过几次别的学校的培训课，我参加过人大的一个老师的讲座，当然她也来咱们学校讲过，讲Mplus的，还有刘东讲HLM。我发现可能咱们学校就是缺这种手把手教你的这种课

LMN：可这种不是方法类的课嘛

YWH：对，其实方法类的课也还蛮重要的，现在实证研究还是一个……，这些东西还是要会的，不会的话也不行。而且就是咱们学校老师上的课，可能就是更理论一点。然后更讲ppt一点，就缺少这种手把手教你方法。包括《定性研究》就上那么几次课，只能讲一讲前面的那些理论的东西。我还听过贾旭东的课，那个老师不是也做质性嘛，就是手把手教你一步一步怎么编码，可能咱们学校偏少这种比较干货的课。

LMN：我很好奇你对那些seminar的课是怎么看待的。

YWH：这种课其实怎么说？如果你们平时开组会开的比较少的话，这种课还是很有必要的。你每学期参加这种课，讲讲文献，就你读完这篇文献和你把这篇文献讲出来的感觉是不一样，而且这篇文献对你的印象也是更深的，只要你准备了、讲过这篇文献了，这篇文献真的两三年都不会忘了。你讲出来就完全不一样，但是对我来说，我们就每周开组会嘛，也都会时不时地讲文献，我记得我上上学期可能一学期就讲了7、8篇文献，每次组会讲一篇，这一学期下来积累下7、8篇了。那这样的话就是，讲完之后同师门会一起讨论、轮流发言。轮流发言就是这篇文献怎么样、你从这篇文章中学到了什么，然后老师也会总结告诉我们这篇文献哪里是比较好的地方大家学习，然后也会就这些文献里面的一些理论问题、概念啊进行一些讨论吧，拓展性的讨论，这种其实还是很好的，但是其实这个任务我觉得不是在课堂上的任务，它应该是放到组会里面的任务，组会是一个博士生培养的一个很重要的东西

LMN：但是其实所谓的seminar就是大家汇报、老师一两句话点评

YWH：对，因为课堂上就是大家都会汇报。像我们组会的话，基本上就是每周就汇报一篇，然后大家深入讨论这篇，然后讨论完了，再说每个人的进度，我觉得这就这就比较好。

LMN：导致我们现在上课好像还是流于形式那种

YWH：对，形式就是你讲一篇，老师看看你讲明白了就算了

HQL：我其实觉得像清华北大来的老师，他们的课程和我们的课程还是存在很大的不一样，不知道学长怎么看？

YWH：我觉得他们的培养和我们都不一样，其实清华北大的老师更不管。他们老师更散养，他们是让自己的学生找好方向之后去找老师，因为清华的资源比咱们丰富一点，咱们只有老师嘛，人家还有博后呀或者新来的老师啊，他们就让学生自己去找那些人结对子，他们可能比较自由一点；而且合作这种理念也比较形容共识了。其实他们的大导师管的更少，大导师更忙，因为他们外面的社会事务也更多。我觉得唉怎么说呢，这样的话可能也好，就是自己学的东西会很多。像清华北大是这种感觉，然后中科院就不一样了，我老师就是中科院的，中科院就管的比较严，每周都要开组会，就跟我们一样。而且还有大组会，小组会。

HQL：我听这种方式比较是在物理学科呀这样自然一点的学科，但是……

YWH：对呀，因为中科院心理研究所嘛，他们就标榜自己是自然科学，他们所有的那些东西都是往自然科学上靠，毕竟人家是中科院的嘛哈哈

HQL：那OB的话，现在也是这种嘛？是整体的气象都比较往心理科学、自然科学上靠嘛

YWH：我感觉这种事就为什么他会往心理学边上靠？因为好写文章、好发文章、好做研究，收一批数据，就用心理学那种，做实验啊，问卷调查这种，各种测量啊，前测后测，方法也比较多，但是这样是不好的。因为管理嘛，你除了往那方面靠，还有组织管理嘛，那个只是一条路径，就自下而上先从研究个体的行为开始，然后能产生出管理意义；其实组织管理还有从上往下看，就真正的从组织架构、组织管理的角度，但这种研究就是不好写文章，你用什么方法呢，就案例研究法，你只能写案例的，或者是你用实证的文章去做的话，测度又不太好，很多东西你没法测。像我现在做权力的研究，我也是尽量地还是从组织行为的角度走。我也想从组织架构的角度走啊，但是组织架构测度的时候，权力这个东西都测度不出来

LMN：所以我们现在做研究就是会被这个方法给限制住

YWH：对，是是是会受这些方法的限制。而且你那样做的话，可能你写出来的文章被期刊的接收度也比较低，因为人家都发的是那种，你突然来一个这种的，就感觉哈哈。

LMN：那你用实验也好、问卷调查法也好，就是一般常用的这些研究方法，有没有觉得有一些可能研究并不是那么合适或者说有些烦了？我说说我自己的感受吧，比如做实验，然后可能设计完这个实验之后也不觉得它有多么的妙，就是觉得可能是为了获得这样的一个数据，我会觉得心里很难受。

YWH：确实，怎么说呢，社会心理学的实验没有不成功的对吧？

LMN：对，特别是王老师跟咱们说过一句话，几乎每次答辩他在的时候都会说，组织行为学相关的概念很少有不相关的。

YWH：对，你做实验你就是那么控制的，那么操作了肯定会出来那样的结果。这实验是很少会不成功的，哎怎么说……

LMN：所以就相当于是一种，我们假设它会成功，我们操作完它确实是成功了，然后用这个成功结果验证我们想的是成功的。

YWH：对，是，你说的非常对。其实我们做实验、问卷调查出来的这些东西啊，它真的就是现实吗？它不是，它不是现实，它只是对现实的一个很片面的观察。它这个很片面的观察就已经是脱离了现实，它就不是实证啊。本质上，我们现在所用的这些方法都不是实证，都是伪实证。

所以为什么说我们现在要用建构主义的，诠释主义的这些，因为我们社会科学的这些东西是很难触及到现实的，我们根本观测不到什么是现实，现实也是一个被建构出来的，很多现实也都是非现实的，是我们人眼中的现实。你的那个概念已经主观化了，然后你非得说拿个东西把它客观测量，那个概念就是主观的了，为什么还要把它客观地测量出来呢？而且你会发现很多文献里面的概念和概念是不一样的，他们都叫那个，但是他们的定义其实是不一样的

LMN：那你是怎么处理或者看待这种事情呢？

YWH：用自己的概念，用自己的理解，你的研究最起码要精确地限定住你对这个概念的理解，然后你后面讲的那些东西都得围绕着这个来说。所以你刚才那个问题说，我们用这些方法感觉很难受，因为它本质上就是难受的，它本身就是一个别扭的东西

HQL：似乎咱们就从这个方法的角度对这个学术的意义，我觉得是有一种否定的，那我们做出来的东西好像从根本上就是错的。那我们……

YWH：所以说我们要更加注重……，就不能像心理学那样去注重从数据来推论结果，还是要更注重前面的思辨过程，我觉得这个过程是比较好的。

HQL：那你自己在研究当中是会比较注意这方面的吗。

YWH：是，我自己就觉得一篇文章就前面的理论部分肯定是最重要的，然后结果是什么、用的什么方法，这个就是次要的。然后，反而后面的讨论部分要比中间的那个要重要，就即使你的结果不显著，也不能推导你的假设是错误的，你还是要去解释你这个结果为什么不显著。

LMN：你刚提到这个理论推导的这个部分比较重要、比较有思辨性哈，我好像感觉就是看逻辑，好像逻辑顺了就可以了。

YWH：确实，因为现在很多都套路化了嘛，就是大前提、小前提、结论三段论，或者它能导出它，它又能导出它，怎么说都能推导出来。就是话是好说，关键是前面的东西你有没有一个洞见，这种东西也很难描述吧，就是说你能不能把概念和概念的关系说清楚，就是说你这个概念和概念的关系是不是一种洞见，它是一种语言还是一种洞见。这个东西还是有区别的，你发现我们写出的文章大部分都是语言，就是好像说清楚了，但是就感觉没滋没味的，它不是你读了之后能给你当头一棒的或者是让你感觉特别闪光的一种东西。

LMN：感觉我听到这儿已经觉得很惊讶了

YWH：没有没有，这还是要修炼，因为我也写不出来。

LMN：但你知道什么样是好的

YWH：对，因为你去读一下就知道，你看那些大的学者的东西，可能他的语言说的很散漫，但是你就是觉得特别有光，他说的东西特别有光。什么福科啊这些，虽然很多都是他的访谈，但是你读完之后就很……。

HQL：我们现在期刊上都是这种实证的感觉，读起来也味同嚼蜡。会让你觉得做学术是一个没有价值的东西嘛？

YWH：我会呀，很长时间我都会觉得。

LMN：这个时间有多长？大概是一个什么样的阶段？

YWH：大概从博二的时候我就有这种感觉，哎呀这都没有什么意义，这都干嘛呀，我就想借用鲁迅的一句话，“学术界我最佩服诸公的一点就是，这东西都敢拿来发表”。

一众人：哈哈哈哈哈哈

YWH：但是你不要把它归因于自己，这跟自己无关呀。这是一个环境的东西，评价体系包括我们这个学术的评价体系，或者是大家的那种内卷，导致的这些东西，也不是说由于我们的原因，既然游戏是这样，你已经深处其中了嘛，那你即便厌恶它，但是该完成还得完成。

LMN：有没有想过改变游戏规则？

YWH：其实现在上面已经有这个方向了，要改变，已经提出一些……这不是一朝一夕的事情，就是你尽可能地在这么一个规则里发挥出自己想表达的东西。也不是说这个规则完全不好，也不是说完完全就是反人性，其实不好的里面也有好处。你会发现那些文献里面也有好的，也有挺好的东西

HQL：对，似乎就是学长很长一段时间内会有一种愤青的小感觉。那什么时候你会觉得自己能够消化这种心情？

YWH：我觉得你不要把自己和它融的太融在一起

HQL：你之前有很融入的那种感觉嘛

YWH：对啊，融入就很痛苦啊，你要脱离出来。我现在心里面的概念是，做研究和发文章是两件事情，做研究是做研究，做研究是在思考，就像项飚说的，把自己作为方法，用你自己的生命体验，用你的思考，可能你的思考是很笨拙的，可能你思考出来的东西是不对的，但这就是做研究的过程，你在其中体验。

发文章是另外一件事情，包括我们现在，老师们讲的最多的就是“你如何发文章”，其实大家现在研究的最多的不是他那个研究话题、而是如何发文章这个话题。所以你要把自己脱离开，你要给自己一个不断的正反馈。然后发文章是另外一件事情嘛，发文章你就要	按他们的游戏规则，看看能不能把你做出的这些东西套进去，弄出一篇东西来发表一下。

HQL：那似乎写论文已经不是一个做研究的过程了，就是不是你思考的主要路径了

YWH：写论文可以是，因为这里你可以写出很多东西来，但是你发文章要面对评审、你要符合他的要求，其实很多意见反馈回来你就会发现，很多都是文字游戏了，他说你那样没说清楚，然后你要解释清楚；还有那个概念和那个概念的关系怎么怎么样，哎呀，就感觉就是一种文字游戏。

LMN：答复评审人的过程煎熬不

YWH：煎熬啊，肯定是，你还得卑躬屈膝的。

HQL：你觉得写到发这个过程，如果是写的过程，我觉得一般的概念嘛就是我写完了这篇文章好像就已经完成了，但是现在在发表体系下好像不是。

YWH：不是，你写完了还得按他的要求、按他的风格改，改完了投了还得按人家的意见改。就是这个过程还是挺漫长的。

HQL：那写到发的过程，你觉得这个比例大概是什么样子的。我自己的一个感觉就是写完了叫做万里长征的第一步，我会觉得好像写到发之间……，我本来觉得我写完了，我自己想表达的东西已经表达完了，我原先觉得起码占了70%吧，那我现在可能会觉得，好像就完成了10%，

YWH：对对对，你发出来的和你之前写的就完全不一样了。对呀，没办法。现在就都这样吧，论文的发表流程都是这样的，感觉很难，但是确实因为我们现在是博士生嘛，其实我们写出来的东西都是很肤浅的，我们写出来的东西也没那么高大上，评审这个过程其实确实也能给你提出很多针对性的建议，但是有的评审就不是了，有的评审他带着报仇的心态，可能有人否定过他，他就完全来否定你，说你这不对那不对的，他也不给你提建设性的意见，但是有的评审能给你提出很多有建设性的意见，你会觉得，确实有收获。

你说到这个发表，我就想到之前听项飚的一个讲座，项飚就说不允许他的学生发文章。他的学生都偷偷背着他发，为什么不允许呢，他说你们现在这个思想都是处于初级阶段的，你现在写出来的东西、发表出来的东西很可能成为你学术生涯的污点。

LMN：我很好奇你大四那一年是怎么过来的，就是包括这边开组会，包括你自己去做研究，做些探索

YWH：那一年我大四还是一边实习，因为我们北邮那学校吧会催着你去实习，还会让你多实践这种。所以大四还在做一个实习，然后当时的组会是晚上开。

LMN：那你有时间看文献之类的嘛。

YWH：一周也就看一篇文献，当时我还小，然后就刚来嘛，也不会让我讲太多东西，就常听吧。我第一次看文献就看AMJ的，老长了，那一篇文献我看了一周，排版之后30页吧，不算参考文献有25页吧，大概差不多。那时候看文献真的是得一周，那会儿也看的认真，就一句一句的看了，很多概念也不知道。

LMN：那不知道的地方你会查一下相关的文献去了解一下吗。

YWH：会。那当时不知道就查一些中文的，就先了解一下吧。

LMN：那这个amj是你找的，还是老师给你指定的。

YWH：学长学姐发的

HQL：你平时组会都看什么文献？英文的还是中文的？

YWH：英文的，我们组会分享的文献，基本上不下A开头的期刊，就是AMJ/AMR/AMA这些基本上了，然后次一点第二阶梯的就是JAP，再低的文献就不让讲了，就只学习比较好的嘛。

LMN：那欧洲的期刊有嘛

YWH：ASQ也可以讲，欧洲的期刊的确实比较少

HQL：咱们中文期刊OB领域的比较顶级的是什么

YWH：《中国人力资源开发》。

HQL：这个属于你们的学习范围吗？

YWH：可以，这个还是可以的，这个期刊虽然在我们学校不是A类

HQL：为什么你们OB领域最好的期刊不是我们学校的A

YWH：对，不是我们学校的A，但是它在有些学校是和《管理世界》同个级别的。我们学校这个期刊评级也是挺挺乱的其实。

LMN：于老师没讲过去规整规整嘛

YWH：这不是于老师能管的事情，这是学校定的。但是咱们学校的期刊列表不也是学院提的意见嘛。

YWH：按各学院的话语权，像我们学院这个组织人力，毕竟在财经类院校他还是导向让你发更偏经济财经的期刊吧，毕竟是财经类学校。

LMN：能问一下你现在已经发表、在投多少篇文章了吗？

YWH：没有多少篇，我发出来一篇肯定是够毕业了，前两天发的一篇又被拒了，那个是真的是特别……就到最后于老师都觉得有点玩文字游戏了，就已经审了一年多了最后被拒了呵呵

LMN：是啥期刊呀？《心理学报》之类的嘛？

YWH：《心理科学进展》。它和《心理学报》是同一个编委嘛

HQL：最后那个拒绝你的理由是什么？

YWH：他就是不喜欢你嘛，最后就是他的那个编委，觉得这个内容可能还不是不是很干、有点软

LMN：那你已经发表出来的那篇文章是中文的还是英文的？

YWH：中文的，《经济管理》啊。

LMN：除此之外嘞？

YWH：除此之外还有一篇英文的在投，被拒了一次然后又投了，正在初审。然后应该还会再投几篇，但是毕业前应该会出不来。就是把我的博士论文拆开来投一下，我的博士论文还没投呢。

LMN：那你发表的那篇，跟你的博士论文相关度不是很高？

YWH：不是很高，但是硬扯的话也有一点相关。

LMN：你是啥时候开题的？

YWH：我是疫情的时候，2020年七月，博一下学期

HQL：学长我记得有一次我们不是一起开座谈会嘛，然后林老师也是问起你发了几篇了，我觉得当时还挺有那种紧迫感的，好像你说发了两篇吧。然后林老师摇了摇头，他就觉得好像找工作不是很好

YWH：那找工作就是拼文章嘛

HQL：对呀，那他对你这种说法给你带来什么样的感受吗？我作为旁观者，我觉得完蛋了，我这辈子可能找不到工作了。

YWH：管他那么干嘛呢，人要保持自己的这个稳定性

就稳定性，

不要随便被外在的一些因素干扰。而且他就是那么个人，可能你发三篇他也还是觉得很少。

H：那你平时会有这种发文章的紧迫感吗？就是周围人没有这种催促啊，你们老师也好，同学也好。

Y：没有，我老师这边基本上你达到毕业要求就行，不会催你。当然也会说，你这个先投了吧，就先投着吧。不会说非得催你，还必须发什么。

H：那你周围的同学呢？同学会对你心情或者会让你觉得自己要发文章，有这样的情况吗？

Y：不大会有吧？不大会有。

H：我觉得你算是比较快的。

Y：应该也不会。就是我认为发文章这个事情就是一个水到渠成的东西，这个东西不是能够证明你什么的东西，他就是能给你获益的一个东西，不是说你发了文章你就厉害。

LMN：那当时有想去工作吗？

YWH：我是比较喜欢自由的，就不太喜欢受管束的。

HQL：在大学里面可能会更自由一点，但是现在像考核和非升即走，弄的压力好像也挺大的。

YWH：反正你就完成他那个任务吧，只要完成了任务，他就不会开除你。

HQL：昨天王老师发了一个北京化工的招聘，现在211学校都是师资博士后，就3+3，如果能评上你就评，评不上就再延三年，延三年评不上就走了，相当于六年。你不害怕吗？

YWH：害怕啥呀？你能完成就完成呗，完不成你就走呗。我爸妈是做生意的，我爸16岁就孤身一人就去外边闯荡，然后我爷我奶没钱，当时在那做生意也没钱，就借高利贷，借黑社会的，然后慢慢才打拼出来的。所以性格里也不怕这个东西，是觉得随时可以重新再来。这些东西就他不给你，自己再来呗，在哪不能搞这些东西了。

HQL：而且我发现，其实你看书看的挺多的，我感觉就是相比一般的博士生来说看得比较多。

YWH：没有吧，大家看书应该都看了很多吧，可能很多博士要看文献吧，不过还是要多看书的。书给你的，尤其是经典，他给你的那些思想要比文献多。文献的话就那么一个东西嘛，而且很散，我还是比较爱读书的啊。

哎呀，女孩子追求稳定也是对的，不稳定也是对的，毕竟还是有一些生理差异的。女孩子出去打拼也是不太容易的，而且好像面临着更多的任务。最近上映的《爱情神话》这个电影特别好，你看人家人到中年也有爱情。你看人家里面那几个女性，人家活的多精致。谁说女人非得生孩子？其中有个人就说你没生过的女人是不完整的，然后马伊琍扮演者就说那没睡够100个男人的女人是不完整，那都是人说的。

HQL：似乎你是对外在的标准不是很care。

YWH：对，因为我有个创业的想法。就是我想着我30多岁的时候想出去开健身房，所以可能到时候我会全心扑到那个上面。

HQL：我们本科的时候肯定是对学术界不是很了解嘛，我可能就是觉得好像老师很厉害很神圣，我会觉得学术界是个很神圣的地方。那可能博士之后就跟老师会有比较近距离的接触啊，了解教师这个行业大概是什么样子的。

YWH：对，这不就是心理距离的拉近。拿你会觉得学术圈有一点神圣，不过我从一开始就没觉得它有多么神圣。因为科学这个东西吧，它和宗教还不一样。宗教可能会让人感觉神圣，但是科学这个东西就是一个途径、一个方法，就是了解这个世界的途径，了解这个世界的一种方式，而且只是一种方式，还有很多方式可能现在不被大家所重视，比如周易、八卦、风水，这也是了解世界的方式。我是觉得如果你觉得科学神圣，那就是最没有科学精神的。科学精神就是说要反思、要批判、要不断进步、要不断质疑，之前的都叫假设，

不可能有真理，我们的理论是在不断的进步，这才叫科学精神，科学不是用来崇拜的。所以科学这个东西，我没觉得学术圈是个多神圣的地方，我觉得学术圈就是一个充满争论的地方。

HQL：可是似乎现在学术界不是那么的充满争议，给我的一个感觉就是老师之间的交流也是彬彬有礼。

YWH：现在已经就是也没有尊重科学，也没有敬畏科学，反正现在就是很混沌的一种状态。其实我是觉得其实搞学术，尤其是搞理论学术啊，术其实不需要这么多人，哪需要这么多博士生？就是我们这些博士生，我觉得应该放开，让大家多出去。就尤其是一些工科的，我不知道我们啊，就商科我还没想到办法，就那些学工科的博士生，天天读论文，应该把他们放开，产学研结合去，是吧？去公司里面去，或者去国企里面去，就去做一些实践的项目会好一点，就不需要做很多理论的文献的探讨。

像我们商学院这个东西吧，就比较尴尬。怎么说每个商学院成立一个咨询公司，附属的咨询公司？

LMN：这个好像就有点偏离学术最本质的那种追求的，因为这个跟商业捆绑在一块去了可能也会异化一些东西？

YWH：就可以模仿医院，你看医学院不是也有附属医院？我们商学院可以附属咨询公司。这个可能说，就是咱们没有一个很好的合法性。我们学的东西就感觉没用的，这是因为我们没有把就这个学院、就没有把这个东西运用起来，要是开个咨询公司的话，你看好多企业的问题找你来，正好成立一个小组来解决这个企业的问题，大家用学到的知识去解决真实的问题。

LMN：收费吗？

YWH：收费肯定收费。

LMN：我们是一个事业单位，我们还收费？

YWH：能收费啊，我们事业单位也要创收的，我们收费，但是肯定会比那些咨询公司要低啊，我们是抢这些公司的项目。

LMN：那咨询公司也会抢学院的人啊。

YWH：那不是正好给我们提供工作机会了。

HQL：我觉得管理学其实现在挺尴尬的。

YWH：对，确实，管理学，毕竟它的定位还是一个应用学科，我们现在就感觉不伦不类了。

HQL：我还是想回到那个问题，就是老师之间似乎也没啥争论。

YWH：哎呀，大家还争论啥！我们于老师常说的一句话，发表的目的是什么？发表的目的是学术探讨，是去争论一个问题，这是发表的最初的目的，但是现在发表的目的成了评职称、晋升这些东西。就是以前的话，论文发出来是放在那儿给大家去看，你要等着大家的回应，以前这个人发了一个东西，另外一个人又来反对你，甚至为了反对你创造出一些新的东西，以前是这样一个发表的机制。现在这个机制已经异化了，就是成了发表完了我就没事了，这个文章也与我无关了，他的版权给了杂志社了。不要再问我这个问题，我要继续下一篇文章，就大家都开始重发表本身。而且学术界开始慢慢崇拜那些发文章多的人啊，也不去真正去探讨他的文章里面内容是什么，反而问他这篇文章是怎么发出来的，问你的发文经验是什么，你看咱们学院就是那个什么论文大赛的系列讲座，也都是讲讲怎么写论文的，什么精诚合作、精准定位这些。

LMN：我看着很好笑，然后再挂一个名字，把论文写在祖国大地上。

YWH：就是把论文写在祖国大地上，这句话是很好的，是让你洞察我们国家的这些特殊的管理现象，就特殊的这些东西来发表你的一些观点，人家的重点是“在祖国大地上”，大家理解的重点可能是论文，是“把论文写”。所以说就导致了现在大家不去争论了，因为发文章的这个目的已经异化，就大家就不再去争论那些东西了，大家的重点就改变了。

LMN：关于争论，就是老师们会教你写论文的时候，就是你不要跟以往的观点产生正面的冲突，是一定要委婉再委婉，这是一方面。另外一个就是我目前为止看了这么多，也不能说这么多，就我目前为止看的论文来说，我只看到一两篇文章是和以往的论文直接对话的，他就是说，比如读什么什么的文章的一些反思。其他的很少有这种直接对话。所以当时我们到那个时候反而觉得很稀奇，但这其实才是学术或者是发表的本质，对吧？

LMN：嗯，现在这种很少了。

YWH：为什么我说战略的那个课好，其实人家那些文章都是在争论，人家那个确实都是在争论。所以说你看那个年代还是特别那个啥，就是大家都在围绕特定的问题在进行争论。包括什么管理学的流派什么的，我们现在好像就是一言堂一个流派，咱们没有流派了，现在只有话题。咱们研究哪个话题，只有他是那个话题的引领者，那个人是那个话题的指导者，然后大家就疯狂引用他。

LMN：那你现在会不会经常性参加讲座呀之类的？

YWH：我参加讲座还是比较多的，你是说咱们学院内的还是外边的？

LMN：所有，都可以啊。

YWH：我参加的还是比较多的，我发现很多人讲的还是……就有些东西吧，他讲出来我觉得很可笑很肤浅，但是还敢上去讲，而且还是人大的教授，我当时就想他怎么这么肤浅？

HQL：他讲的啥？他

YWH：讲的是什么我忘了，是去年我看的一个他在清华讲，我在线上看的啊，就是他也是研究权力的，我看了之后就感觉他就是自己想了一个关系，在那不断的讲东西去充实这个东西，就感觉还是比较肤浅的，最终的模型也是跟咱们差不多的。

LMN：有没有让你觉得看过后印象比较深刻的那种讲座呀或者是研究呀？

YWH：哎呀，最近啊，我感觉项飙可能是，但是项飙是很早了。最近的话确实很少，最近的话我想不到什么印象特别深刻的。

HQL：有的时候就会想起西南联大那个时候，虽然那个时候没有管理学嘛，但是文学界的“大家”就很多嘛，现在我们也会回忆那是个黄金时代，但反观现在的话，起码从管理学来看，没有这样的，可能它发展时间短是一个原因吧，但好像似乎还有别的原因呢？

YWH：德鲁克算“大家”，但是德鲁克没发过什么好期刊。

HQL：哈哈哈，对。

YWH：其实我们评价可能不是以他文章引用高或者数量多。你发现了没？那个年代其实我们管理学的大家都挺牛的，但是都停留在了那个年代。你看那个年代那些人啊，德鲁克出来标榜自己是管理学之父，然后科特勒营销管理，这些大家一出来都是拿自己的一个研究领域来标榜自己。然后现在我们所谓的大牛出来发过几篇论文以外还有发过了什么？你是搞啥的呀，研究啥的呀，具体内容不重要。

HQL：哎呀，其实听起来挺让人心痛的。

YWH：当然能出思想的那些东西也不是一时半会就会有的，我觉得中国肯定会出。因为中国的管理你会发现跟西方的很不一样，但是这个还是需要时间的，需要时间的。

确实你像那些老的，那个以前年代的人，他们写篇文章就随便找个期刊，人家重要的是让我的内容让大家看到，人家不管是什么期刊，让你看到你支持我也好，不支持我也好，你跟我争论也好，首先是发出来让你看到，人家不追求说发一个什么样的期刊。而且那会儿期刊也没那么多话语权，也不像现在的期刊牛逼哄哄的。以前都是期刊“恳求赐稿”，现在的期刊真的是……哎呀，现在学术界都已经被期刊执掌。现在搞个学术讲座还要让编辑上去讲，哎呦，我真的是觉得……你就觉得这个特别奇怪。你想想那些写小说的，什么莫言】余华搞个文学讲座，还要请一群文学编辑上去讲一下你们该怎么写小说。

LMN：看来是整个学术界的，不仅仅局限在管理学。那你对讲座一般会有什么诉求吗？还是说也不是有什么希望，就是去看，去听听就好？

YWH：一般没什么诉求，就是以听为主，以听为主。因为学术交流还是不能封闭自己，还是要跟人交流的，即使他的想法你觉得不太好，但是可以多听听，多听一下你就知道，唉，他们是我怎么思考这些问题的。

LMN：我近半年吧有一个变化，就之前讲座我也是只要差不多相关的我就去听听，后来就发现这个讲座吧，你看到这个提供的资料就知道他要写什么东西，就变得索然无味，就不想去听了，知道吗？

YWH：这跟现在的研究也一样啊。

LMN：现在研究或者是有的时候会弄一个非常宏大的一个帽子，让我觉得哇，太相关太有用，再看就会觉得你讲的都是什么啊。

HQL：然后我还想一起探讨下这个“卷”，就是到处都在卷嘛，但是卷可能有一个明确定义，就是说低效的竞争、无谓的损耗嘛，就是内耗嘛，无谓的投入，你对这点有争议吗？

YWH：没有争议。

HQL：那我们学术界的这种体现是什么样子？

YWH：我觉得大家都在说“好卷啊”，具体来说大家发文章的竞争越来越大，但是发的这些文章对学术一点贡献都没有，这就是一种“卷”。发出来的东西没有争议，对这个学术思想，哪怕咱就不说对管理学思想的发展，你哪怕对企业有一定指导意义也行啊？

LMN：我的一位博士生同学，他就觉得是有那么一点点贡献的，哪怕是就是一亿分之一的贡献也行。

YWH：你那一点点的贡献，在真正做企业的人来说就是常识，对吧？就是常识！然后你说你用这些所谓的数据来验证了我这个常识是正确的，我用你验证？我用你验证吗？而且你收的这些数据是真数据还在假数据？所以其实是真的，以我们目前水平来看做研究，其实贡献就可以说是约等于零。但是有些人会说有什么什么样的贡献，可以说他是有贡献，但是那些都是编出来的啊，都是建构出来的，说的好是建构意义，是建筑出来的，说的不好点那就是瞎编的。

HQL：嗯嗯。之前我们不是一起去参加那个座谈会嘛？就是那次座谈会对我伤害还挺大的，因为他这个意思是说为我们博士排忧解难，对不对，但最后就变成了为学院谏言献策，然后那个葛老师还是那种居高临下的那种感觉，。我就觉得我再也不想参加学院组织的活动这种感觉。

YWH：那个确实定位没有定好，而且那几个老师没商量好，之前也没通好气。东西应该是于老师办的，后来就因为俩领导都在那嘛，最后就成了那两个老师在主导，就是林老师和葛老师两个人主导，其实那个东西是于老师在弄吧，后来于老师还走了，就导致确实到最后变味了。于老师的最初的初心还是说给大家提供一些心理上的支持，但是确实他们三个老师也没商量。你看那个座谈会咱们就应该讨论出一个共识，就是说大家不发文章也能毕业，那不就挺好的。

HQL：于老师有没有跟你们讲过，就是我们学院博士的毕业情况非常的堪忧？我听他们讲说于老师非常的操心。

YWH：对，于老师很操心。于老师基本上那些人都认识，就是没毕业了的那些人也都认识嘛，确实比较操心。去年不就四个，今年也有。确实我们学院发两篇的话也挺尴尬，就是你要找教职，这两篇又不够，对那些不想找教学的学生来说比较高，到时候看变不变吧，反正清华北大也是临时变的，就是说要求不发的那种也毕业了。这个你还得看教育部他们上面的态度，就他们对这个发文章是怎么看的。其实我是觉得博士生就不需要发文章，你就还没有基础，博士生发那个干嘛？你发出来到底是什么样你自己心里都知道，其实学院这么做可能也就是要做出绩效。那学校要发展、学院要发展，不都得看整个论文数量嘛。上面虽然说不“唯论文”，但具体到实操层面，那他这个导向还是以发文为导向的嘛。

HQL：那假如说就是不发文章的话，你理想状态是什么样的？

YWH：过程管理吧。就是你把这些环节都走过了，而且这个过程管理要加严，不仅仅是这个博士论坛这么一个纯水的形式，是吧？你这个过程一定要严，每年的学年论文一定要评审通过，这个评审要加严，不是发论文，你的学年论文就可以各种形式来写，不一定是发表的，就是你可以讲你的研究可以干嘛，而且这个过程就是要严格到完全需要校外的老师来评，就你可能是校内两个老师然后再找校外三个老师。就要有这个自信，我们培养出来的博士生是合格的，或者说是用专业性的标准让专业的人来评专业的事情。就是我们的那个学术委员会也一直没有发挥作用。

HQL：学术委员会去哪里了？我没感觉到它的存在。

LMN：我们都没感觉学术委员会。

YWH：其实我们学术委员会应该对博士生的培养起到一个很关键的作用，就是说学术委员会要确定怎么去评价每个人的研究，每个人都干嘛。然后每年的学年论文或者每半年都要有一个汇报，把这些过程走完其实是不需要发论文的。你把这个培养过程走完，都在做事情，都在慢慢做事，最后慢慢的把你的博士论文做出来。而且也是为以后打基础啊，就是为以后漫长的教职生涯奠定基础。

其实葛老师说的那点还挺好的，就是应该把博士生当成员工一样，就是你可以在学院的网站上把博士生的名字挂出来，每个人的研究方向都挂出来，你把这些东西让大家有更多的交流，然后放上研究者每个人的联系方式。那我们其实还是被当做学生对待，而且跟老师之间的权力距离很不对等呀。

LMN：刚刚像你说过程管理里面可能有一些人短期之内可能没有什么成果……

YWH：但是你做了什么你要讲。你这个阶段虽然没有成果，但是你做了什么你总有的，你不能什么都不做吧？就是类似于说我们那个考核东西，不是说局限于论文那种形式，哪怕你这半年你就只读文献了，你说你读了50篇文献，那你把你读的文献做个汇报，整理一下做个汇报，是吧？哪怕你这种跟现在相比都更有实质性的帮助。你就完全按照这种培养下来，最后你的学位的定夺权就是完全学院说了算吧，就不是说你非得发文章，又期刊说了算。

HQL：嗯嗯。我想问一下就是以前有一个这个土博士和洋博士的区别，那对学长来说这会是个困扰吗。

YWH：国外的不也很水的吗？是但是怎么说呢？就是像北大清华，他们那个底色就是要出国，然后这种风气慢慢扩散到这个社会。其实我觉得怎么说呢？外来的和尚就会念经吗？那不对，要看长期发展吧。

HQL：但好像学校又偏爱这种。

YWH：因为他们要国际化，非得要在国际上把中国的这个学术实力搞起来，也有这方面的需求。但是你想能真正能关注到本土问题的，其实还是本土培养的这些博士吧，他们能更关切这个社会。

HQL那我们要去国外发期刊论文呢？

YWH：这一方面语言是一关，我们写出来的东西可能确实不太符合他们英文的那种，而且可能我们的研究问题也不是他们国外想关心的。你像他们更关心什么哈，在权力这个研究领域，他们更关心性别歧视、种族歧视啊这些，在国内这些问题都没有。所以怎么说呢？这个东西还是一个发展进程吧，你看中国要是真的能强大起来的话，那说不定外国还能跑着发中国期刊。这其实也就是个话语权的问题，你厉害，你这个国家真的能指导一二的话，你还发什么英文期刊呢？大家都跑来发中文期刊。

HQL：那我们OB现在会跟随西方的话题吗？感觉就是中国本土的问题，现在上层有这个号召，那我们再在这个体制内的话，我们现在的研究导向是什么样子的呢？

YWH：就从中西方这个角度来看的话，那可能还是西方的多。那我们OB领域比较热门的话题就领导力嘛。那你觉得这是一个西方情景下的问题还是中国本土的一个问题呢？这个东西啊，领导力这个东西，你说中国没有也不可能。其实我们当领导的也有领导力，但是可能我们的领导力和他们的那个领导力还是不一样的。尤其是他们弄了好多好多概念啊，什么变革型领导啊，什么威权型领导啊等等，但是在中国来说，可能有些领导有那种特质，有威权型，有变革型的，有服务型的，但是每个领导都是一个很综合的。尤其是中国人见人说人话，见鬼说鬼话，对上一套对下一套，是吧？所以说就有没有领导力这个概念在中国还是值得讨论的。

LMN：也特别想问一下你，你知道关于研究领导担当精神的吗？就之前有一次访谈，因为老师就提到他不是人力这个领域的，他只是对人比较感兴趣，然后就提到这个，当时我确实我恍惚了一会儿，我没想起来担当到底有什么，然后他就说研究跟实践的脱节嘛，他说咱们中国说了几千年的担当了，但是好像没有一个真正对应的概念。当时我就在脑袋里想量表吧，好像也没有这个量表似的。

YWH：担当和责任还是不一样的，担当是一种主动的责任，责任是很多东西被压上来了，你看西安现在都成啥样了，谁都不敢担责，责任是一方面，我们师门在研究这个。另一方面就叫问责，这好像是被迫担责，还有一种责任就responsible，好像有点主动的那种意思。那个担当就更愿意主动去承担这个责任，甚至是冒着一定风险。

LMN：它对应的英文是啥？

YWH：哎呀，我也不知道。可能xxx现在用的概念叫内感责任，这个观点可能跟担当还有点类似。

LMN：这种就是有细微的差别，好像又不是非常清晰。

YWH：这个东西都是随人说的嘛，这现实是复杂的，你只能通过一个概念来片面的把握显示，只能撇现实一眼，我们的理论就是这样吧，你就只能从一个侧面从一个视角去窥探一下。

LMN：真正的这个世界还是复杂的，就我们研究还是把这世界给看得很简单了。

YWH：研究就是要把世界看简单，就是要从去繁就简，从这个复杂的世界里面抽离出简单的东西，抽离出关键的东西，从一个视角出发抽离出这个关键。然后你懂了这个关键之后，然后你再去实践的时候，你就打蛇打七寸吧，就只用控制这个关键的因素就行了，就不用管其他了，你就不迷茫了，我们研究的初衷就这样。但是会发现，我们有很多理论，越研究越多，我们的理论，我都笑死了，什么组织管理必备的40个理论，然后又来60个理论，前两天你们见了吗？又出了一本管理研究必备的45个理论，这就说明这个学科非常不成熟，成熟的学科只有两个理论。量子力学和相对论就两个，而且人家还正逐步的走向一个大一统。

LMN：那什么内感责任、问责制、变革性领导，这都是从西方过来的概念，我们现在就是在这套话体系下挣扎。而且我们我们挣扎的时候，我们非要把我们的一些东西和人家相结合。你看北大研究那个王辉研究辩证领导力的，生搬硬套的，我都感觉他那个很尴尬。而且你还不能讲，人家是北大的教授，大家都是夸啊。

YWH：他那个辩证是想要结合中国的特色，他是想要结合中国的阴阳，包括马克思的一些辩证思维，但还是要跟从西方的那套，要跟他们对话。我觉得可能那个台湾的做的比较好，就是因为主要是我们现在看不到人家的期刊，因为我们有政治的这些隔阂。就他们写文献就直接会把台湾当成一个国家，很多这些政治的因素，所以我们看不到。但是人家台湾做的很好，比如台湾的黄光国那些学者，人家就是有一本本土心理学，人家就是完全做的本土的东西，可能人家方法上会使用方西方的，但是人家不是为了与西方对话而进行写作，也不是在和西方的概念对话，人家就是在做自己的，我们确实有很大的问题，反正人家台湾那边做的比较好，但是我们基本不和台湾的对话。

HQL：现在之前你转的那篇项彪写的那篇文章，那天我想搜一下那个期刊才发现根本就搜不出来。唉，明明我们可以有很多特色的表达和独特的现象，我们还要用人家的解释，就很奇怪。

YWH：台湾比我们有个好处是，他们保留了民国的一些东西。他们的那些东西可以从民国的一些学术概念上往外生发，就有我们中国的一些文化基因。就你说的那个黄金年代，他们其实是留下了一些就属于我们中国的一些概念，后来我们经历了文革这些，而且我们是信仰马克思的，我们还是马克思的这种导向，好像我们管理学里面中国特色的东西在讲，但一直都没讲出来。

LMN：确实一直没讲出来，导致我现在都不太知道什么是中国的特色。

YWH：我现在觉得管理学领导力里面，可能最有中国特色的就是家长型领导，这个应该是从中国生发出来的，但是说实话这个有点过时。现在咱们国家本土的，哪有家长，咱们也是完全西方那一套，咱们很多企业里面也没有什么家长。我们可能更多的是一种人情式往来。。

LMN：最近就老是发现这种人情啊、关系啊、圈子啊这东西好像是无处不在。

YWH：中国根大树大呀，生长存在了2000多年，从孔子那会儿都已经把这个东西讲的很透彻了，这些是不可能消灭的，但是这一块社会学的研究还有社会心理学研究可能多一点。还有我们管理学，可能我们追求的结果变量就是绩效，就是你的研究的导向、问题的导向就是怎么提高绩效，根本的问题就是怎么让管理更高效嘛。包括你们营销就是怎么卖东西卖的更多一点。所以我们的研究可能更多的还是手段性的东西多一点，所以会有什么变革式领导的心理怎么怎么样，用这些手段你可以很好的让这个绩效提高。所以我们就关注那些弥漫在我们周围的这些什么人情啊、关系啊、圈子啊这些东西比较少。

LMN：那我们会不会就是过于关注这个绩效之后其实忽略一些很重要的变量？就咱们学院要求发论文，他就是为了提高自己的指标，但其实对我们学生的损害也是显而易见的，就大家就是短期主义。

YWH：其实我觉得这个东西，他们只是没有去测量，或者说故意没有看到而已，但其实影响是非常大的。那如果到企业里面，我们只是一门心思想要提高他绩效如何如何，可能对于员工的一些隐性的伤害都没有考虑到。现在我们很多的管理都是叫燃烧式管理，就燃烧你的激情，然后让你去干活，但是没关注到那个燃烧后，你是有灰烬的，是有损失的。

HQL：好像我们也有一种科学主义的导向嘛，仿佛不可测量的东西也就不值得研究了。

YWH：对，所以我一直认为社会科学就不叫科学，就很多很多东西都不能称之为科学，只有自然科学才能叫科学。但我从来认为社会学是有很强大的力量的。所以就是说科学不是研究社会学的唯一途径，有很多途径可以研究的，包括人家那些算命的那些，比如相面什么的，你别小看人家，人家有时候理解东西也蛮深的，很有道理我觉得。

HQL：我应该暂时没问题了，太谢谢学长了。

# 受访者32

LMN：两位学姐都是经历过硕士之后然后考上来的哈，为什么呢。

LYN：那我先说吧，我上本科的时候就没想过读研究生，因为我入学那一天开始，我就觉得我适合工作，因为我那个年龄嘛就比较简单，然后也没接触过什么东西，就想什么事都很简单，我就觉得我的性格就适合工作，而且我一直觉得我要工作肯定是个工作狂。我从入学那一天就没有关注过什么研究生、硕士、博士这些信息，因为当你不关注的时候，就算你接受到这些信息，你也就自动不听了，所以我关注全是跟工作有关的。

因为我是一直觉得自己应该去工作嘛，然后我想着那我要去工作，怎么也上四年大学呢，也得一次奖学金吧。一是写简历上好看，二也是证明我上大学了，然后我就好好学。大二得了一次奖学金，我说还挺好的，还能拿钱，我说大三我再得一次吧。后来是大三下学期就知道保研名额了，我连保研名额都是我同学帮我去看的，因为我当时本来想放弃保研的，然后我同学去帮我看了，就我排第一名，然后我就纠结，我就找各种老师，我说“老师，我想工作，那我要不要读研呀”，人家老师都说，“人家拿到这个名额机会的时候，第一反应是我要去哪个学校，你怎么第一反应是我要不要接受呢”。就是老师们一般都是希望学生读研读博的。然后我就给家里打电话，我家里也没什么想法，就说你要读就读，你不读就不读，怎么着家里都支持你。

然后后来，我之所以要去读研是因为我觉得我排第一了，然后我放弃这个名额有点不甘心，如果排第二我可能就放弃了。然后另外一个就是我其实那个时候有慢慢意识到……就是那些信息你不去接触的话就有可能是遗漏的，然后我就想为什么大家那么多人就抢这个名额，肯定有他的道理所在。所以我说，那我就去看看吧，我看看硕士到底是一个什么东西，大家都抢那名额，然后我就保研了

LMN：哈？你就这么去看的呀，不是去打听打听嘛

LYN：没，我就想我自己去看一看了。但是我也没怎么努力，因为要参加夏令营什么的，然后我也没怎么报，所以我就保到我们自己学校了，然后我们班第二第三都去南京大学了，我就留在山大了。

我这个三年下来读完了，我觉得我很庆幸当时我是那样做选择的，没有直接就放弃。我觉得硕士阶段的收获还挺大的，因为我大学就相当于睡了四年，也不出门哪也不去；然后硕士的时候，我就觉得接触挺多的，老师就很好，我选老师也是看照片选的，就很随意，我老师很负责任也很好，并且我们跟业界接触又很紧密，然后也搞学术，我们老师当时给我们建议就是无论你要不要读博，你在什么阶段就做什么事，所以我在硕士阶段也不去实习。

我刚入学的时候，老师就问我要不要读博，我说老师我先不说的，因为我原先还说不读硕士呢，我怕我打脸，所以我一直说我暂时先不读，因为我从来没想过要读博的事，是我男朋友从我研一开始劝我读博吧读博吧。但是因为我不在意，所以我就没把这些信息当回事，我就一直没在意，然后研三的时候他就开始发力了，我那个时候已经找好工作了，他把这个事情提上日程了，就跟我生气。

LMN：他为啥这么强烈地让你读博？

LYN：后来我才知道的，就是他就跟我生气，然后说你必须读博，不读博那咱分手吧。就是有一种逼的程度了，然后我就开始在意了，当你把这个当回事儿了，你就会收集那方面的信息嘛。但是我跟他说，我还是……因为一是我工作offer已经拿到手了，另外一个就是我不想一下就转变自己的人生，因为读博我虽然了解地不多，但是注定是另外一条路了，跟我硕士毕业以后就工作是不一样的路了。我就不想一下子改变人生轨迹，我就沉淀了十个月，从研三就开始考虑，我也不刻意地去想，我就偶尔跟我宿舍两个硕士结束准备读博的室友聊。

2019年我就毕业了嘛。然后我那个时候offer也拿到了，就去工作了五个月。这十个月的过程，我觉得读博挺好的。一是因为我上五个月的班，我每天都要挤地铁，我觉得好像我一上班就想请假，我觉得不自由。虽然我们总监很好，你跟他说一声就行，但你也不能每天都请假啊，所以第一个原因就是我不想挤地铁了。另外一个原因就是我男朋友老让我读博，我说，都有人愿意养我了，那我就读呗。

LMN：你男朋友是已经工作了嘛？

LYN：对对对他已经工作了。最后我11月份才决定离职。虽然我决定读博了，但是我还是总拖啊拖啊，就拖到11月份了，11月份我才提离职，我12月5号才彻底离完。那个时候其实很多学校结束了（博士报名），像北航报名已经招完了，11月27号就招完了；然后人大是12月底面试，但是人家很早就报完名了，我都没赶上。到最后就是哪个学校还有，我就报哪个，我就联系，后来反正就是阴差阳错来读了吧。

然后我男朋友之所以想让我读博士，他觉得一是我那个工作没什么出息，就是一眼望到头了，要是去一个很有挑战性的工作也还行，至少在成长对吧？另外一个就是他觉得那个工资对不起我们的付出，因为他工作过，所以他觉得我们上班那个钱是不能来弥补我们付出的价值的，是浪费人生的。他让我来读博不是希望我在博士里有什么造诣，他是希望我有四年的时间来缓冲，让我自己来好好想一想这一辈子到底要干点啥。他说，“如果你博二就想好了这辈子就干这件事，你可以辍学”，就是让我来缓一下。

所以我一方面就是他督促他、催促我去收集这方面信息。然后我就觉得，读博是挺好的，我记得我沉淀了十个月，我还是觉得读博挺好的，然后我就裸辞了，就开始准备考博嘛。然后正好12月份五号那个时候，我已经看到腾讯新闻上说华南海鲜市场有病情，但是那个时候不叫新冠，就是不知道会爆发这么大，所以我在备考期间就是疫情也爆发了，然后我也没有工作，然后我还裸辞了，那个时候也有点压力吧。但是我是那种很喜欢去给自己心理建设的，我考不上正好明年还可以报人大，因为我目标是去人大，今年没赶上那正好，考不上就拉倒，明年就报人大了，后来就没什么压力了，就我是属于那种给自己洗脑，慢慢让自己去相信那些。后来就很幸运地就考上这了嘛，然后就也很顺利来读。

我来这边读了半年，就是我越来越喜欢那种状态。这里我要穿插一点——为什么我要来读博。就是我沉淀的那十个月我还经历了写硕士毕业论文。我每天睡到自然醒，基本上我彻底清醒了，就是下午一两点了。然后醒了以后，我就穿着睡衣去隔壁写论文。就那个过程，我很享受。因为我研三的时候也实习过，我就会发现实习的时候很难专心干一件事，会一直有人找你，一直有人找你，但是写论文的时候，你就可以专心干一件事，没人找你就写论文就行了。所以包括我来这读了半年到一年的博士之后，我就越来越享受这种状态，我觉得真挺好的，就啥事也不用想，也没人打扰你。然后现在也没有什么毕业发表的压力，就想干啥就干啥，踏踏实实干，我还挺享受这个状态的。

LMN：什么叫没有毕业发表的压力？

LYN：就是我现在还没到那个节点，可能当我马上面临开题了，我会有压力，但我现在没有到那个节点呢。比如说我早就给自己心理建设，我要五年毕业呢哈哈

HQL：林老师不会有要求嘛

LYN：没有没有没有没有，我们师门很随意，老师对我们很好，老师现在就怕我们有心理压力，老师说“不用让自己太累啊，差不多就行”就那样。但是我很享受现在的状态，我觉得挺好的，就是每天该干啥干啥，然后我感觉我没什么压力。但是你说是没有压力，身体怎么可能没有压力呢，就是身体可能比你意识要更敏感一些。有时候就比如说休息不好呀，其实是有压力的，但是你意识上可能感受不到，但你身体比你意识先感受到了。所以我还是挺享受现在的状态的

LMN：像你研三的时候就是醒来之后就去隔壁学习嘛，一个人的那个过程，不会觉得孤独或者是觉得很枯燥嘛

LYN：不会呀，我虽然是属于那种很贪伴儿的，你看我平常大大咧咧嘻嘻哈哈的，我很爱玩，就跟大家聊大天，但是我要干啥的时候就很投入。我那个毕业论文因为我数据之前就收好了，模型早就跑好了，所以我十几天就把毕业论文写完了。然后我不是说一坐就坐一天，我就下午两三点开始坐那儿，然后到晚上吃饭了，我就约我同门师姐一起吃饭，我们每天都在一起。然后有时候还会去老师的办公室，在那里上自习。就是我是那种很需要陪伴的。比如说我第一年来这的时候，上半年我就很不开心，虽然都有同学嘛，但是不是每天要在一起，同学还是各自搞各自的，但是我贾师姐回来以后我就变开心很多，因为每天都去她宿舍聊聊天，每天都去她宿舍聊聊天。

我觉得在我是那种……你们听过mbti吗？就是一个人格测试，我是属于另外一类，就是那种通过跟别人交流汲取能量的人。你比如说，就是很多人有同辈压力，不爱看朋友圈，有些人就把朋友圈屏蔽，我听我师弟他们就聊过，他们会把朋友圈关闭，因为他看到别人的朋友圈会有压力，但是我不是，我就是喜欢看别人的朋友圈，我在这个八卦的过程中会汲取很多能量，我说，哎呀，人家又变优秀了，我是外部驱动的。那别人变优秀了，我就希望我也变那么优秀，然后我不会因为朋友圈产生压力。

好啦，该你讲啦，为什么读博？

GX：主要是工作导向。就是我从小的梦想就是当一名人民教师，但是之前就是想当小学老师，然后初中老师、高中老师。但是后来发现小学老师、初中老师、高中老师吧就太累了，然后你还要教他们，你想把他们的成绩提上去吧，就是不是说你想提上去他们就可以提上去，在提的过程当中，你可能会用到其他的一些手段，不管是正向激励还是不好的激励，然后这样可能对他们的成长过程可能是不好的。所以我不希望那样面对孩子，然后就想，那我还是当大学老师吧。当大学老师，而且还有暑假，寒假，这两个假期也是特别吸引我的地方。

然后读完硕士以后，我就没有立马找工作，也是就找找看看，也没有特别合适的。而且硕士毕业之后，现在高校吧大部分也都是那种行政岗位，然后还是要朝九晚五的做一些就是特别繁琐的事，我也不太喜欢。所以后来我就自己开了一个小的辅导班，然后主要就针对是小学生。但是我又特别怕他们下课出点什么事儿什么的，因为孩子就确实也不大，然后又是那种喜欢打打闹闹的男孩子。然后每天都紧张、每天都紧张，就只要他们一来我就开始紧张，一来就紧张。但是我确实挺喜欢他们的，他们一来我都特别开心、特别高兴，然后也想要逗他什么什么的，对他们也特别好。然后就是长期神经就绷着，孩子们他们在学校就是待到下午三点多，因为下午就两节课，然后三点多就去我们那儿，一直待到五六七八点。所以跟我们待的时间也比较久，家长有什么事就全都是问我们，包括他作业的事各种事，然后每天就是到了晚上十点经常还会回家长信息、会接家长电话，会问你不光是作业的事，学习的事，还有心理的问题。尤其是心理这块问题，就各种什么早恋的问题啊还有各种被同学欺负啊、什么校园霸凌都有。然后你除了要梳理家长的情绪，你还要梳理孩子们情绪，因为在学校发生的事，家长很多都不知道。但是他们（的情绪）可能会被带到辅导班这边了，因为他们刚离开学校，然后到这可能会延续那种情绪什么的。总的来说，我主要是还是怕他们安全上出什么问题，后来我就想那再继续考博吧。

MN：你这个工作做了多久？

GX：两年多到三年吧。有的孩子我真的是从一年级然后带到了快四年级了，小孩现在上一年级比较早，就是六岁。我记得特别早的时候，我带的第一批五岁半开始上一年级的，然后鞋带都不会系。那时候我还教他们系鞋带啊，虽然我一看他们的鞋带就不是跟我学的，但是他们会记得这个老师教过我。实际上他们就是跟别人学的，但是他们脑子里边就有一个瞬间我教过他们，我觉得还挺有成就感。我说那如果换工作还是当老师的话，那还是当大学老师吧。后来就是想考研，然后就跟我家人说，然后他们也特别支持我。

HQL：然后为什么一定要当老师？是因为家里有人做这方面的工作？

GX：没有，我就是从小就想当老师。就从小大家都是当老师那种啊，就是可能想管着别，哈哈哈哈，因为从小被管。然后就一直到现在也是喜欢当老师。因为还是说，那时候跟学生在一起，虽然是跟小孩在一起，每天都很开心。

然后就是比如说有的小孩就会跟你说，就一年级小孩放学后他会就很开心跟你说：“葛老师，你知道吗？我有男朋友了”。我心想你知道什么是男朋友吗？我就问他，我说你男朋友是男生还是女生？那当然是男生啦！他们在哪啊？在那个那个幼儿园了。我说你还姐弟恋了。我就觉得就特别好玩。他们就是经常会有那种很多小想法呀什么的，都特别有意思。

HQL：这个小学老师和大学老师还是不太一样呀。

GX：对，但是有一方面就是比较通的地方就是比较想跟小孩在一起，然后比如说那时候跟小学生在一起就很开心，但是你当大学老师也会跟学生在一起，你还是会把很多年轻的元素融合到你的生活当中去，就很开心。

HQL：那对研究有没有什么想法呀？或者是觉得要探索这个世界啊？有没有这种动机？

LYN：我觉得我觉得我在这个方面读博，特别是现在读上以后我越来越享受，然后我觉得这个职业路径其实挺好的，我，我可能跟欣欣不太一样，就是。我是后来导向的，然后往这条路上走的，但是我觉得明明之中可能就注定，比如说，我现在特别感谢我硕士老师，他会告诉我在什么阶段就做什么事。所以如果没有硕士阶段积累，我可能读博的时候就很吃力，因为我硕士确实没干用不着的，就一直在学校里，然后就该写就写，跟老师该干啥干啥，然后心心的是觉得当老师好所以也是往这条路上走的。

虽然是性格推着我，但是我真正选择读博是我自己做决定，就是我觉得挺好的，GX是说觉得老师好，所以想来走这条路的，我那个时候没有从什么教书育人呀这个角度来考虑，没有想过这些事儿，我是觉得对自己的这个整个人生是挺好的。

首先就是时间上来说，特别是我入学以后发现老师这个职业虽然真的很累也很卷，但是他时间是自由的，比如今天我想去干什么，我不用跟校长请假，跟院长请假，我想干什么就干什么，可能这两天我在外头忙，但是过两天我可能就把时间集中起来，我就把这时间补回来了，我是从自己的这个时间上自由这个角度来考虑的我的职业发展。我倒没想着说将来我当老师以后会带学生，没想那么远。然后虽然现在也没有确定将来要不要搞学术，但是我觉得这条路是挺好的，我也没想过说带学生我会变年轻或者怎么样，但是校园的这个环境确实会单纯很多。搞学术的话我觉得挺好的，就是可以一直干一件事，研究生的时候也做研究了嘛，我当时第一年的时候最大的感触就是我从被动的状态变成一个主动的状态，研一上课，研二开始搞一年多的学术，但是那个时候是没有读博的打算，我是研三才有读博打算的。所以研二的时候我是那种听话的孩子，就是没有没有所谓的喜欢或不喜欢，就是老师让干什么就干什么，这是我的任务，该干就得干，我也踏踏实实干。但是谈不上我给自己干的，我愿意干了或怎么样，因为那个时候你是一个被动状态。

但是我到博一的时候我就主动去学东西，因为我是觉得那是给我自己干的，我自己的人生要自己负责，是我选的这条路我就得好好走。因为我从入学第一天，就没把博士当做上学，我觉得这是一种职业。

LMN：硕士阶段是学生，到博士其实你对自己身份是有一个转变，不是把自己定为一个学生而是一研究者。

LYN：我把自己坚定为研究者什么也好像也没有那么深，我是觉得那是要为我自己的人生负责了，反正就是不是一个上学的状态了。你上学是差不多就行，但是你现在就得给自己搞，就包括欣歌让我读博的另外一个原因就还是说你现在读书是给自己读的。就是你要是工作，那是去给资本家给别人打工，你怎么着都那么回事，但你读书是永远为你自己读的。

我觉得当老师虽然你是在这个体系制度下，但是你终究是给自己奋斗的，就是因为自己做事，你发100篇论文那你就在这个行业有名了，但是你在企业，你给企业赚100个亿，你只能说是一个明星员工，但是当有一天这公司裁员的时候，可能说把你裁了就把你裁了，对吧？但是你，你在这个学术圈里，你为自己搞100篇，别人拿不走你这些东西。

LMN：GX你呢？当时对研究有什么想法吗？

GX：我觉得博士生活吧，他真的是一种生活，但是我没有把他当成是一种职业，我始终还是一种学生的心态。然后体会最多就是还在学校有导师的时候，然后这个时候你就会觉得就还是挺珍惜的，你觉得现在你写东西有人给你辅导，然后你科研有不会的问题都可以跟老师探讨。但是如果说你将来毕业了，然后你的科研能走多远，就取决于你博士期间你自己的积累。然后将来可能就没有人在管你了，就没有人像现在导师一样这么管你了，所以我觉得现在我还是不愿意把导师当成老板，或者是把现在当成一种职业，我就还觉得自己是学生。

然后科研方面也是没有特别地说我非要研究什么，就是我之前对这个玩意儿反正也没有那么多的热情，然后我只是把它当成是一种生活。

然后我的专业一直是变化的，就是本科的时候是会计，然后研究生的时候我调到旅游管理，然后就只要还在管理里边，然后对于我来说，因为应该也有一些基础的素养，所以都还能接受。就是我老师也比较比较强势就是他让我研究什么东西，然后我也没有特别多的想法。因为我不像有的同学，他们都有自己想法，因为他们当老师也一直在从事这方面研究，但是我工作了三年，相当于是没有什么科研基础，所以来到这也就是老师基本上让我做什么就做什么。但是时间长就是觉得你积累的稍微多一点，然后还挺感兴趣的，最起码能就是一段时间你只能做一件事情，然后专用在一件事情上。那除了这件事情，你可以安排其他的业余时间，可以自己随意调配自己的时间。我还是更想把现在的生活当成一种学生生活吧。

LMN：我不知道是不是因为你们都有这个工作经验哈，然后对比后觉得科研挺好，对比之后会享受学术这种自主，这种长时间可以干一件事。就我本科的时候也是能够静下，但是当大四的时候开始写论文的时候，我其实挺痛苦的。可能是因为我老师对我比较严格，他当时知道我要硕博连读嘛，所以他对我要求一直比同组成要高一些。当然可能跟他要求不高，也可能跟我天资比较平庸有关，就是那个时候就感觉其实挺痛苦的，就是可能在图书馆里面一上午可能只能看完一篇文章，甚至看的还是模棱两可，不是特别理解那种。然后可能马上过几天就要开组会，就是那种非常高强度，所以那时候真的是动不动就自己开始哭了，你知道吗？就是没有体验过工作是个什么的感觉，那个时候就只觉得科研好像还挺痛苦的，就跟你说那种可以去教室自己去学习还不太一样。

LYN：嗯，怎么说呢？就是刚才GX点到我了，就是确实我很喜欢那种专注的感觉。就是我是第一次启蒙是在我写硕士毕业论文的时候，然后有这个感觉，就是在博一下学期课就比较少了嘛。然后特别是博二上学期就开始去年502了，去那感觉更好了，因为大家有伴，然后就大家都在那学，我觉得挺好的。

虽然我只工作过五个月，但是那五个月其实对我没什么挑战性。就包括欣哥也说大家夸你的话你就不用太在意。但是这么多年走下来，我在每一个岗位上都得到了肯定的，我觉得大家虽然会有一些人就是习惯性的鼓励你，但是我觉得我确实做的应该是不差的，所以我工作五个月，其实我觉得没有什么挑战性，因为那个时候我就知道我会辞职啊，然后我只用了50%的力量就得到总监那么强的肯定，所以我就觉得其实工作没什么挑战性。

我为什么出来实习呢？是我保完研了，因为我在地方上的大学，在山东上的大学。我就觉得那个时候我想象的北京是很厉害的，我觉得大家已经比我厉害很多，然后我又躺了四年多，然后我就觉得我应该出来，我当时的想法这样特简单，我就想来北京碰碰壁，我想看看我跟别人的差距到底在哪，然后那个工作就奠定了我的职业价值观。那个我来实习是在那个海中关村软件园，那边一个企业就是欣歌他们公司，就是那个过程中我认识欣歌的，然后我是做一个hr实习生，然后从2015年的12月份开始实习的。然后那个工作给了我很大的自信，就是我发现我跟别人没有区别，因为我们部门有很多人大的实习生，就是那个劳资学院的。就是我从地方来了，可能更有那种敬畏之心，就很珍惜这种这种机会，然后我就踏踏实实干，那个主管的又对我很好，然后他就很喜欢放手，就是让我单独的对接一个项目给那个项目招聘，然后我就在这个过程中，一是我确定了就是世界是主观的，就是不要听别人说什么社会尔虞我诈的，这跟你没有关系，就是你看到什么样就是什么样，然后我就发现我掏心对他好，他是感知道的，他也对我好，所以就奠定了我未来步入社会的价值观，就是你不管怎么样就是你，你对别人好，虽然可能你会遇到骗子或者怎么样，但是那也都是少数，大部分人都是愿意掏心掏肺的。另外就是职业价值观，那个阶段我真的我太开心，我太快乐了，虽然我接受保研，保研成功了，我差一点都不想回去了，我想说跟学校申请，就把我档案给我弄回来，我不去了，是那个主管姐姐跟我说你赶紧去上学。工作以后还一辈子的时间了，你不差这两年，然后在她劝说下我就回去了。

就是像你说的就是确实是我，我之前跟你们班同学聊过，你们很多人读博士不知道自己的真正想法，有些人是不想工作，是还觉得还小不想工作，还有一些人是读到头以后再说工作的事。反正大部分就是年龄小的是不知道自己为什么这样活的，就是不是说将来要当老师的。但是我是觉得我硕士阶段除了接触的多，另外就是年龄大了，你懂的可能会多一些，然后你就会慢慢的清楚自己到底想干什么。

LMN：我真的觉得我们硕博连读的这几个就是没有想清楚，没有像你经历过硕士之后会有对学习的这种渴望。

对，我当时就是感觉还很小，就是视野也很窄。但是我觉得其实一下读完也挺好的，但是那种过程可能会痛苦一下，因为你没有未来目标，跟我没有未来目标还不一样。我工作过，我知道这个读博这个感觉更好，所以我来读，不管我未来要干什么，但是我现在一条路，此时此刻的路是我愿意去做的。但是你们是此时此刻的路又过得很痛苦，然后又不知道未来干什么，假如你们未来去工作了，可能有一天你们会后悔我为什么要来读博士呢？我都去工作了。确实是那个样子，但是我觉得确实是需要年龄的增长才知道什么。其实现在我们不断压缩这个学制啊，包括这个直博，有的可能本科就直博那个，其实我觉得不是很好。就他们还小，那个时候人的判断更多的是受氛围的影响。我是觉得你像我们，我上大学的时候，我们学校是可以去别学校交换的。然后你像我们上一级了，就所有人都出去交换，一个班有出一半人去交换，但是到我们这一级，一个人没出去，大家都干什么？去修双学位去了啊。对，这就是一个氛围的影响。像我们保研，我们2016年保研其实没有那么夸张的，我很多同学本科毕业就工作了，不像这几年。就是大家大学毕业自然第一反应是去考研、保研，而不是去工作，我们那个时候第一反应都是去工作的，所以保研就是没有那么夸张的，但是这几年大部分都在抢。

所以我是觉得怎么说呢？就是这个阅历还还挺关键的，然后很多人都是被……也不叫被忽悠，他是被氛围影响的。就像林老师其实他不爱表达，他总是直接说他要劝退你们或者怎么样，但是其实他是知道的，他不反对你搞学术，但是真需要你真的喜欢、我适合这个东西我去搞他，他非常欢迎的。他其实很喜欢学术。

HQL：你觉得林老师喜欢学术？

LYN：我跟你说，虽然他经常跟大家说，他很后悔他当时读完博士为什么不去工作，如果去的话可能都坐上大中华区什么副总裁了之类的，但是其实跟他接触的多，你就知道他其实很喜欢学术吧，他自己有那个执着劲。虽然他现在这样说，到时候你再让他去选，他可能还选这条路。就是我这种感觉我说不出来，但是我就感觉他其实喜欢学术的，那不然他那么拼干嘛？

LMN：拼可能是种表现啊，那有没有通过他的一些话语啊，或者是给你们说的一些话能够感觉说他喜欢学术？

LYN：我佩服我老师的地方就是他搞学术是从现象出发的，他搞很多有意思研究，比如说他面试我的时候我背后全是变形金刚，他会想到变形金刚可以研究呀。就是很多人喜欢这种东西，你可以研究。

就跟踪他平台卖变形金刚这个产品或者怎么样，就跟踪这个过程。他会从实际出发去考虑。但是说实话，就是你也上过李家涛老师的课，对吧？说实话就是接触香港，然后国外那些学者，可能是幸存者偏见或者怎么样，我会觉得从他们身上，就你能感受到他们对学术是真的热爱，也不叫真的热爱，可能首先是生存啊，他就是一份工作，对吧？但是你能感受到他们是真的喜欢。

但是说实话我可能也没有刻意去感受或者是我大脑中潜意识的刻板印象，我没有在中国的学者上看到大学里的学者说我就喜欢这个，然后我不计生存、不计未来，什么都不计较，我就要去研究它，管理学科其实应该很少吧。

HQL：那你觉得为什么？为什么中国的管理学者好像不是特别的热爱？

LYN：我说不出来，特别像那种现在速成的博士，我觉得在这方面有理想的更少。

LMN：是不是现在想起肉食鸡了？肉食鸡就是40天就能长成的那种。

LYN：就是速成的话，他就有一个弊端，就是没有那种阅历感。我当然我不排除肯定有喜欢的，你不喜欢的也不可能搞那么多，对吧？可能很多人会把他当作一种职业和工作，但是怎么说呢？有一些学者大部分都是清高的，包括我们其实会会比同龄工作的人会清高很多，可能你不愿意承认，但是你就是清高，就是你接触世界浅显，然后你接手的事情很简单。

比如说有很多老师，他在学校里会有自己的理想报负，然后他想出社会，然后等他工作差不多他想辞职去社会，他发现他根本适应不了，他适应不了那个社会。你还记得有一次人大的刘军老师给咱们分享，他就是搞学术搞得挺好了，然后他想去社会就赚点钱啊，或者没说这么直白了就是想去社会，那他最后发现适应不了，他又回来了。

LMN：嗯，那你觉得这是个好的现象？

LYN：可能是一个脱节吧，我觉得管理学科跟实践脱节挺大的。

我硕士阶段，我收获还挺大的，因为我硕士阶段是这样的，就是因为我老师是搞旅游创业、搞酒店那些东西，所以我们接触业界比较多。比如说我们在学校学两个月，就是搞研究，搞两个月我们就会出去，一是放松，二是接触业界。所以，我们会去乡村旅游调研，然后去酒店调研，然后我们去酒店调研就不像咱们学校那种大规模的，去什么中关村软件有人参观什么的，我们去酒店调研是真的。因为我老师在酒店届的名声可能比较厉害了，然后我们座谈的话就真的是董事长总经理，然后各部门总监跟我们一起座谈，就真的是只跟这种人聊，就真的去聊，还去一线调研什么的，所以就接触比较深。这样的话，我觉得其实对于我们有很多的好处，包括我师姐啊，当时为了写论文，我老师让他去希尔顿实习了一个月。那她接触后感觉肯定是跟完全造是不一样的。

说实话我会觉得像管理学科，很多东西叫咨询吧，咨询其实就是挖坑，然后你再给埋上。有时候写论文就是无病呻吟，没有病，你就可以把它写有病了，然后你再给他解决方案。

LMN：对，那这样你有感触，那你为什么觉得做研究很有意思？

LYN：我倒觉得。我现在还没有产生出说做研究很有意思，那有一些是有意思的研究话题，但是我没有做出来的，另外一个就是我喜欢这种状态，我不是说我喜欢搞研究。就这种状态就是你可以专注干一件事，然后没人打扰你。我不是说我写出一篇论文，我是有多有成就感，不是那种感觉。就是不是说喜欢搞研究本身，而是喜欢工作模式。我刚才想强调一点就是别误会，就是我说我把博士当做一份工作，但是我从来没把我老师当做老板，我从来没叫老板。就是我进一个师门就容易跟人建立感情，我就真的走心的那种，我会把我师姐当家人，把老师当家人，就是站在他们对方角度想问题那种。就是我没有觉得研究本身多有趣儿，没有上升到这个高度。

LMN：因为像我吧，我就是因为也是看到了这个，比如说研究与实践脱节，比如说挖坑再埋坑，比如说自说自话这种不太好地方的时候，我可能就很难去维持我这个研究动力。就是可能我从早上八点做到晚上十点，这都没问题，但是这种可能就是不是心甘情愿的。

LYN：我知道你那种状态。我觉得我之所以没有那种那种困扰，是唯有一个好处就是我刚才跟你说了，我把我现在的这个研究当做一份工作，没有把他当做自己的兴趣，所以说就是做完这件事情本身已经就可以了。

LMN：那林老师是哪种爱？

LYN：我说不出来，我老师不怎么爱跟我们表达这些，但是你说不喜欢他怎么能没事就能冒出一个新点子呢？我老师经常会琢磨，不然这些点怎么出来的？就我经常跟欣欣说我的数据，她就觉得很有意思呀。研究小说、研究音乐、研究英雄联盟研究……就我老师身上有一处特点，他从实践出发，他那种实践不是说工作实践，因为你们是搞人力的，可能必须要从工作场所来说。那我们搞战略的就可能从战略本身或者产品来说，你比如说一个创业项目，他本身里面就会有很多战略，他可能从这个角度来讲就很落地，很接地气。

LMN：所以你通过老师这种，就比如不断有新的点子出来，然后也愿意去投入到这个科研中，所以你会觉得他其实是很感兴趣的，对吗？

LYN：感兴趣我倒说不上，他爱不爱我也不知道啊，这个可能只有他自己知道了。但是他应该在这方面投入时间应该不少，不然他成果也出不来呀，对吧？反正我就觉得我老师很聪明，就是我能感受感受到他很聪明，不然的话，那文章哗哗写，然后说干啥就干啥，他做事很快。

他不像那种还需要职称什么的，他职称都评完了，他现在白天主要是没有时间，他白天行政那么多，他从早上八点到晚上六点基本上不能搞学术，每天他办公室门只要开着，一直有人去找他。他一般都晚上回家会干，我之前还听说过十点把他女儿哄睡了，之后看到十二点多还是看到一点多，晚上12:30到一点他才睡觉。

其实你想啊，都不用说他了，就我搞两天论坛把我给累了，每天早上去盯着，其实他累。我觉得如果是我的话，可能八点到六点这个行政工作都已经把我透支。跟你说实话，就是咱们学院这些院长副院长，他们的行政事务真挺多的。你想我老师，除了有行政，他也要去上课，他们都有课程安排的，要是我真用不过来他脑子。你比较我们，做一件事真的要做很久才能做完，他如果不够速度的话他可能做都做不完，所以我觉得他们还是聪明，脑子很不一样。

LMN：感觉这种安排会不会有点问题？就是院长这种本身做学术特别厉害的人们，给他们这么繁重的行政事务蛮可惜的。

LYN：来咱们学校以后，其实我对咱们学校、学院的印象特别好，我觉得风气很正。你不觉得咱们学院出名的老师都是基本功，就是功力很强的老师，就是在学术上很出色的老师。你像王老师很有名，他真的做得好所以他有名。包括就是第一天我来这跟老师接触，我就觉得我老师肯定是靠实力当成院长。我在山东待久了，不是那种……我老师不是那种说话特好听或说好听的话那种人，很实在，所以我觉得他肯定是靠实力。你看王震老师呀，那些有名的老师都很有实力，才在院里被大家崇拜。

那我在山东的话就是可能因为我们专业比较小，我们系比较小，就没有那么强的内卷，我们那一点不内卷，我一直都不内卷，那过去的七年我都不卷啊。

LMN：就学生不内卷还是学生和老师都不卷？

LYN：你像旅游管理系在任何一个学校，好像基本上都是除了南开大学，南开大学专门有旅游学院，你像好多在管理学院下边的，基本上那个班级的本科生都是调剂过去的，大家都不会选旅游管理的，都是被调剂过去的。比如说我有一个师姐，他毕业以后找到郑州大学的老师，他拿到那个offer，就是她的成果相对来说要差一点，所以人家学院就问你要不要来工程专业或者是旅游管理专业，这两个专业就是对学术要求没有像企业管理那么高，所以成果差一点可能去这两个专业要求没那么高。

HQL：你刚才说实力，我还想问一点哈，就是刚也说就是主要是看他们这个发表的这个成果比较多嘛，有没有就是那些可能发表不多，但你觉得还是很有实力的这种人？

LYN：肯定有呀，咱们学院也有啊，我觉得LSB老师就是。我觉得我虽然对他不熟，但是江湖中有他的传闻，我就觉得他应该是真的热爱这个质性研究吧，因为质性研究发文章是很慢的，虽然这两年要把什么研究扎在祖国大地上，要搞案例研究什么的……

我关注他的公众号，我觉得他一直在坚持在发文。比如说很多人都公众号，ZF老师也有公众号，ZF老师以前也是自己写，他们从今年开始，然后学生也开始写了，但是我觉得LSB老师更新的最久，就一直在更新，然后做那些很有意思。你会发现他的思想，他看的书一定很多，然后他又很深奥，所以我觉得他会热爱吧，反正应该是挺有挺感性的一个人。

LMN：你刚你有提到把研究做在祖国大地上吗？这林老师有跟你们提倡过吗？

LYN：我老师不怎么跟我们说这些，我老师务实不提这些虚的，哈哈哈。

LMN：但是在咱们学院比如说经常出个什么文件，出个什么讲座，就是把研究作为祖国大地上去作为第一句话开头。

HQL：你也是在做质性研究吗？

LYN：我没做质性研究，但是我喜欢案例研究。我硕士毕业以后才发出来的就是案例研究的论文。我那个时候也不怎么会做案例，反正就把它写完了。我们是做乡村旅游的，以前那个山东淄博的一个乡村旅游村做的很出色的一个村作为案例来研究的。那个时候硕士还谈不上什么，搞学术都不懂这些东西，反正就写完了发出来了。

但是这个过程我觉得很有价值，我为什么觉得做案例很有价值，是因为咱不是强调什么外部效度和内部信度吗？我是觉得内部信度是很关键，但是你如果连外部效果都不能保证的话，你保证内部信度有什么用啊？

比如说案例研究跟实证研究中问卷，这两个方法我觉得就是一个内部效度和外部效度和外部效果，实证量大，是，那部信度很好了，但是怎么说呢？就是你说这个数据都支持你了，但是你从实际出发就没什么价值，你有哪个公司会拿我们这个模型，来管理公司都倒闭了。但是我会觉得这个案例研究，你哪怕你比如说，我搞这个乡村旅游这个乡村做的很好，我把这文章写出，我发表了，哪怕有一个企业家真的看到这个了，我就把这个模式给推广了，我觉得太有价值了。我会觉得案例会很有价值，因为他是真的深度剖析，你不管你得没得出结论，最起码你把这个案例传播出去，我觉得就是一种价值。

LMN：你以后会一直做这个方面吗？

LYN：我现在都不做了，因为我老师没有，就其实就跟着老师做嘛。我博导不太管我们说你必须做什么，你有好点子，你有好想法可以跟他讨论的。但是你得成熟了，你去跟他讨论，得实现呀。不然你就对老师说，我想做香港旅游，你也不知道怎么做，你让老师给你解决。

我为什么想写一个案例？其实包括李家涛老师也会跟我说案例研究特别不好发，但是我是觉得，我现在还没有定，如果我以后搞学术的话，我会觉得是将来可以不研究案例，但是你得会这种方法，对吧？这就是我就想从头到尾的做一篇，然后我真的去实操。因为我跟硕士阶段不一样，硕士我确实去调研、去访谈或者怎么样，但那个时候我是没想过读博的，我是为了完成工作。但是我现在不一样了，我现在主动想去学这个方，然后我从头到尾做一遍，然后我看看这案例研究到底怎么写，我知道套路，虽然不研究，但是我要知道。

LMN：那你去访谈了吗？因为我当时好像记得你提到了这个数据啥的。

LYN：我们之前去那个，我做那个文章的时候其实去那个村庄也访谈很多次了。然后套路什么都知道，那现在还没往下做，因为我一直没有机会出京。我手头这个还没开始动，我手头就做老师的，跟着林老师做做实证。

LMN：他是分配一个项目然后大家一起做吗？

LYN：不是，我们都是自己做自己的。我们基本上没有什么合作，就是我老师会有很多点子，我听说我老师有很多数据库，不知道是真的假，因为他也不会告诉我有什么什么什么。就是你恰好赶上这个项目了，那就给你了啊。

我老师会跟我师姐跟我师弟说。人不可能一直做自己喜欢的，因为他们俩有一次遇到困难了，想跟老师讨论嘛，然后老师说人不可能一直做自己喜欢的事的，你现在遇到这个数据难你就不能说我不做了，你也做下去，你该怎么做呀？是难就解决，有什么问题就解决什么问题。然后我们基本上就是正好他想到这个点子想要做的时候，恰好这个你现在手头没事那就给你。

LMN：这么听感觉你们效率都好高呀。

LYN：不是，我刚开学的时候那个数据现在我还没整理明白了啊，也不是特别的快那种哈，因为我们的数据都是二手数据，都是爬的那种，你们做案例你们就知道它很慢的，你要把文字整理出来，然后你们是编码，我们是把文字整理成用数据可以表示的，你有很多要整理的，还挺麻烦的。

LMN：那过程枯燥吗？

LYN：怎么说呢？我觉得还行，我没感觉到枯燥。我一直跟我老师说，为什么我还挺喜欢这状态，没有压力呢？是不是因为我现在还没有毕业的事儿，然后也没有发表的压力？我说实话，就是我一直没有觉得两篇很难，可能是我还没有真正的去实践，自己去干一件事，但是我觉得四年写两篇文章其实不难，所以我没把这个发表当作一个压力，我从来没想过说，我毕业要发两篇文章，我好压力，我从来没想过这个事，就我没把他当做一个事。我觉得正常人大部分都能发，但是大概就是因为我太乐观，我过于乐观，哈哈哈哈哈。

我就很感谢我老师给我这机会，让我跟林老师合作，所以我把我博一写的东西都当做一种练手，就是我老师说你得练，你得会写作套路了才能做的快，所以我把所有的合作、所有的写作都当做一个练习的机会。

其实我你比如说我老师特别好就是，你比如说那个文章我说老师把三作给我吧，你做二作，我是真心的，因为我觉得老师给我这机会就已经很不错了，但是他不会，他会让我们在前边，因为他可能也不怎么需要这些。

GX：我是一入师门，基本上确定大概研究什么东西，就是跨公司知识转移，就是正向的或者是逆向的知识转移。然后导师就一直让我做这个，然后我之前写的综述，然后他是一个非常非常严谨的人。我记得我记当时。

LMN：我上老师第一堂课的时候，然后直接给我劝退了，不是老师的原因，是老师给的这个工作量我受不了。

LYN：我会感觉他是非常严谨，当时的话就是也听过这个传闻，所以就想选那块的课，到后来发现我感觉我压力好大呀，因为老师布置了许多任务，你一个星期就汇报一样，然后中间还有一个什么类似于期中考试啊的，然后最后期末还有一个论文，就很麻烦。我们那一届就两个人上课，就是基本上每周他俩都得讲一遍。

GX：像我们那一届，因为我来这复试的时候我跟金山他们就挺熟的了，然后后来一复试完回家之后就一直都有联系加微信了。所以我当天就说，我说我老师课你们都必须得来，然后他们就都去了，然后上完了，大家说你们老师这两学分太难拿了，比人家那个四学分都拿的难。

LMN：那个你现在跟老师写的综述写出来了吗？

GX：综述就是一直在写，但是他总是不满意。他就是明明就知道我会做错，但是他不会告诉，想让你自己去走一遍，他就是你错了之后，然后他就会问你，你每次一做完东西就会问你，你觉得你写的怎么样？我说，我觉得我写的特别好。那我觉得不好我怎么会给他，或者是我都看不下肯定不回给他，我肯定会做完善的。然后给他之后，他基本上就是会跟我聊很多吧，打电话都能打两个小时。就是他一直必须得他引导，就是不会说你哪错了，他就举例子，然后让我举例举一个例子，不行的话他会慢慢就接近那个结果，然后去引导我。什么时候引导我说我知道怎么回事了，然后他才放心。他就是怕他说出来之后你不明白啊，我就这样反复。所以在一个地方，有的时候就总是卡老久老久的时间，所以我内心也比较着急。

但是他就是那种可能也比较强势吧，他这么多年，他觉得他培养出来的学生很多都挺优秀的，所以他很信任他这种方法，所以他觉得在我们身上也适用的，他就一直沿用他这个方法。

LMN：那你压力会大吗？比如接两小时电话。

GX：现在还不大，但是我师姐他们都还挺有压力啊，基本上就是100连问，就是各种这问题，他会引导你，但是他不会直接告诉你，一个问题你回答完之后，他就会问你相关的下一个问题，然后你就沿着他那个思路去想。然后你就一直不停的在回答问题，回答问题再想再回答。就是也挺那个啥的，就思考也挺多的。但是就是比如说他就会经常，比如说他给你说了三个问题，然后让你改半个月。但是了，他会怕你半个月没有改，或者是你玩了，比如说你五天就能改完，但是你非要改半个月，然后那十天你就玩去了。所以他就说，他说你不要都改完再给我，然后比如说你第一部分已经改完了，然后你觉得可以了，你做第二个时候，你就把第一个给我发过去。但是结果就是，你第一天把第一个发过去的，他第二天就会找你说两个小时的第一个问题，然后可能第二问题你还没做去，接着你就还再改第一个问题，就一直在第一个问题来回这样来回来回转。所以我就觉得很慢，然后我也很着急。

HQL：咱的这个自主性好像不是很……

GX：他就说你有什么想法赶紧告诉我，但是你告诉他完了之后了，就是各种回答问题，然后你们俩都商量完了之后了，其实他是比较强势的，比如说你说一个，他说啊，你这样做也行，但是你做不出来，你怎么怎么着，你这个大部分时候是做不出来的，如果做不出来的话，你就浪费很多时间。他会暗示你，你知道吗？你这么一看，还是希望你听他的。其实他明里暗里就是已经摆明了就是这意思。那他那么说完，你就肯定会选择他的那个办法。

我记着我刚进师门的时候，他就让我们每个人在群里写，就是不管他发什么文章，他原话就是如果你想法多你就多说。想法少你就少说，但是你不能不发言，就是在群里之后，比如说他发了一个帖子，或者他发了一件事情或者他讲什么东西，然后就每一个人必须得回，然后如果24小时不回他就会@你。然后嗯他就让我们就是回答各种问题，比如说那个你们认为独立的科研能力是什么，就是做到什么程度了才算是具有独立科研能力。

因为他始终都觉得，不管你快还是慢，什么时候你有独立科研能力，我就让你毕业了。他说你们不要想着为了毕业，为了拿着这个学位去找工作，你们要为这个学位的话，你们可以去人大门口去买，然后就什么学位都能买着啊。然后他说，你们根本就是不需要来经历这些事情，然后他说你们要想挣钱，你们就快去挣，你们不要在这做学术，在这做学术不会挣大钱的，我告诉你们。

LMN：但是我觉得有点矛盾的，就是老师一方面希望你们有独立的科研能力，可是一方面如果你们表达起话，他也会及时去否定你们那这种。你觉得不冲突吗？

GX：他觉得能发出来一篇咱们学校规定那种a类文章，不说英文的，如果发中文的话，他觉得至少得三年半四年的时间。因为他这种培养方式就是这样，就比较慢。我老师就会觉得需要那么久的时间去沉淀，他一直说的就是，你有做冷板凳的潜质吗？你自己好好想一想。

HQL：就感觉老师的想法和咱们学生的需求……

GX：他们那一代的人，可能就是这样，他们那代做学生的时候跟咱们状态也不太一样，就每天都是学习啊。因为那一代人就是学习机会得之不易吧，而且他们可能很多老师就赶上恢复高考那个时候，所以他们就觉得上学这个事情对于他们来说机会难得。

LYN：我就觉得一代不如一代。就是我们幸福生活，我们太幸福了，不需要说我只有拼命才能……那时候每个老师培养模式可能都不一样。

我们现在可能是结果导向比较多，就是最后给老师展示个结果就行，他不管你过程怎么，你也可以跟他讨论，你也可以就是求助或者怎么样，但是可能因为我老师现在太忙了，就是讨论的过程就没法持续的几个小时，就很短，所以我们每个组会基本上就是以汇报为主，就是你现这周进展是什么，你跟我说。但是我听我师姐说，以前我老师就是没这么忙的时候，他们经常跟我老师一讨论就讨论两个小时，因为老师那个时候没那么忙。好像那一年是师母出国访问，然后带着女儿出去，他自己没什么事，他每天都十一点多才走。但是我们现在的话我估计就是精力真的没那么多，没办法一抽出两个小时跟你一个人讨论，但是你随时可以去找他那种。大部分我们是直接看结果的，但是比如说我们的论文，他真的会也会给我们一句一句改。

但是据我了解就是模式有点不一样。你像王震老师就会直接把病句都会改，就是这么细，就是你这句话不能这样说，我们也是一句一句改，但是不会细到到病句那样。毕竟我觉得是因为人力做的本来就更细，你没有说新提出什么新理论或者怎么样。但是战略不一样，因为战略可能本来就是大一点，面要大一点，所以他不是那么追求，说你有病句我就不给你发了。但是我会觉得人力卷到那个地步就可以，那病句都不行。

GX：我觉得其实是跟老师有关系。

HQL：我想说那个林老师现在是一个什么样的想法。他是想说把这个院长做到很好吗？还是要在学术界有很高的影响力？

LYN：我老师没跟我们说过这些东西，但是我是觉得他应该不会放弃学术，因为他现在年龄太小，他40出头的，四十二岁。他不可能放弃学术，而且现在随着职业越来越年轻化，现在很多领导都搞学术的。包括副校长人家都在搞学术，对吧？

那个葛书记不是马上退了，但是人家今年评上博导了，她就搞学术了。今年才评完，我看那名单上有她，她开始搞学术了。人家现在都这样，就是行政也做，同时人家不会放弃学术。你包括那个山大的管理学院院长，现在是是北大的副院长，人家搞学术搞得可好可热闹了。然后因为他不现在是院长了嘛，然后本来就是人家搞学术搞得很好，经常组织学术会议，就是组织那种全国的，邀请各个学校的去，做的还挺好。我觉得那这是真爱吧。因为不是为了生存。

GX：但是你还要考虑另外一个事，就是在学术圈，你虽然当院长了，但是你还是得有实力的，所以你的学术不能拉垮呀，对吧，你还是得往前冲，这是一个职业，这是一种很专业性的体系。

LMN：你帮忙学院举办博士论坛，你对这有啥看法？

LYN：我觉得大家展示汇报能力真的需要提升，包括自己。

我觉得读博真的是一件，就是你就啥也不干了啥也不想了，就是老师们的想法就是你别再心猿意马，就单单搞学术就行，我明显觉得我生活变得越来越单调了。给你举个例子就是说我经常就是忙完了，然后突然有一天我很累，我想休息一下，不知道干什么，我觉得除了学习我没有什么可干的，我想逛街吗？不想逛。想躺着吗？也睡不着。我在家一躺能躺一天，但是学校我真的躺不着，就是那样你不知道干啥了。然后我那天的感受就是来读博士以后，大家都专心跟电脑打交道，我觉得我的表达能力都下降了，还有很多理礼节性的东西都下降的也挺快的。

因为我本科学旅游管理的，我们上大学的时候都要在酒店学会实习的，所以很多礼节，我们上课都要学的，我们也去酒店实习，这些端茶倒水都会的，那现在因为到不需要这些，就慢慢就忘了就不知道给老师接点水。

然后我今天听博士开题答辩的时候，我就是这种感觉。因为我觉得管理的东西，终究是讲故事。今天我还跟欣欣讲呢，我觉得这个论文只要老师看见了，就是你展示的过程，绝对占到给你的评价50%以上，就你可以写的很不好，但你讲故事要讲的很好的话，你最后可能就过了。最后一个虽然不是以故事形式说，但是最起码讲的就是很口语化的，就是你能看到她的自信，第二个那讲的是啥呢？念稿子啊！因为你将来也要当老师，你就不能这样了。

我们管理战略就是讲故事，你比如说讲个乡村里的故事，那肯定有意思，大家都愿意听。我其实我觉得每一个文章，每一个博士论文，都应该是一个特别好的故事，就是积累了你这三四年的研究吧。所以我就希望将来我也那样，但是我知道很难，其实挺难的，你写出来，就只能说我在我展示之前能梳理一下，用学术语言把它讲出来，我不会讲，我也不会讲故事。你用案例很容易讲故事，但如果不用案例的话，就其实挺难讲的。

确实有一些，包括老师在内，有一些展示能力也挺需要提升的，会写但是不会讲。听听就能让人睡着了，不知道他想讲什么。

LMN：然后那一天论坛还有什么感受？

LYN：嗯没啥感觉，大家做的挺有意思。我想不出来，我就觉得要是让我做，我可能会做的很散。还没开学我就开组会嘛，然后就问老师老师你对我将来有什么期待期望嘛，老师就说希望你能有独立的学术能力。

HQL：那你现在对自己有什么样的期望吗？

LYN：唉，说实话，我现在就是没什么期望，就很容易沉浸到一件事，我就完全不想未来的事了。

其实说实话，我自己心里也认可，就是偶尔需要给自己简短的憧憬展望一下，可能对你来说更有动力。但是我现在就已经懒到不想憧憬未来了，所以先走一步看一步，就是不想憧憬。

LMN：那你平时的正反馈都有哪些呢？或者是你怎么去调节？因为像我这种吧就长期没有什么那个比较正常的一个反馈嘛？现在遇到刘老师还好，在之前的更是那种就有时候会怀疑自己这样做对不对呀这种。

LYN：我感觉我博士这一年我都不需要什么肯定，我都没想过这件事儿。就自己已经肯定自己。我就觉得我变笨了，就是我脑子反应慢了，因为你每天看文献，你脑子看文献其实很简单，所以平常的事你就不想想了。就这个动作很简单，你的肢体很简单。就是看文献这件事吧，你的思绪跟你要做一件那个事物性工作是不一样的。

做事物性工作，你脑子可能要一直动，一直反应着，然后你才能跟上下一步要干什么。但是你的学术的东西，你需要一直安静的坐在那，你脑子虽然在反应，但是跟事物性的这个灵活还不一样，你就需要把你知道写什么，记下来就行了。

齐老师给我打电话让我做那个学术部，我之所以接受是我不太会拒绝别人，因为是怕忙，我都博二了，我还干这种事，是吧？你一般的这种不都是博一做的。然后齐老师跟我说，没什么事。我是为了帮个忙，然后后来这个论坛就交给学术部了嘛。但是我做了这两天以后，我发现其实挺好的，我周末不过是花两天的时间，但是我在做事务性的工作过程其实会找到很多自信。就是搞学术可能是延迟满足感，就是你要延迟幸福，延迟成就，但是这个事务性工作会立刻给你反馈，比如W老师就跟我说你做的真好，下次你自己就可以一个人做了。这个过程是找回自信的，包括我大学大四那年出去实习，在奠定我未来的走向社会的价值观的那个过程也是给我很多自信的，因为那是事务性工作，我今天干了，明天就可以得到肯定，但是学术不是这样子的，你你搞研究，没有人肯定你，除非有一天你功成名就，你发了很多篇amg，可能有人羡慕你崇拜你。

LMN：就是你刚才说去做事务能够快速给你正反馈，那做学术可以满足你自主性的这个需求。

LYN：我就觉得也不叫自主性的需求吧，就是简单生活、环境简单，你不需要操心，没有杂事，就很纯粹。所以变傻、变笨了。那你工作会有很多杂事，你比如说，你跟这部门沟通，跟那个部门沟通，领导交代你什么你要下达，你每天要接触很多信息，就狂呼乱炸，那微信要加很多人。我现在不行，我以前喜欢，但我觉得现在我做不了这种事了，就是我会觉得很烦，我想安静下来，脑袋嗡嗡的就是一会要跟这个人说，一会要跟那个人说，然后一会大家还要反馈给我，我再反馈给领导就那样。

哎，我不知道为什么，也可能是实习那种比较简单哈，我反而挺喜欢这种，就像你说的事物性及时反馈嘛，阶段不一样。我本科的时候也还行，我本科就干这个的呀。我要上传下达，然后招聘发简历然后约面试，那这很有意思。我不跟你说我都不想回去上学了嘛。但是我硕士阶段，我研三再去实习，我就不喜欢这些东西了，我觉得很烦，就是跟年龄有关系。你随着年龄增长你见识的多了，这件事已经对你来说没有敬畏感了。另外一个就是你变懒了。你像我本科同学基本上都是去民营企业，虽然跟学历本身有关系，但是民营企业其实活力更强，一个喜欢年轻人。但是我硕士的同学，一大部分都是去了国企啊，然后事业单位啊或者是考公务员，虽然是跟学历本身有关，大家会想把这个学历发挥到最大值，但另外一方面跟思绪还有关系。因为大家年龄大了，见得多了就觉得还是公务员好呀，或者是就也不想再奋斗了，不想再拼了，不想去“四大”累死了，然后就其实都有关系。

LMN：那办这个学术论坛的时候感觉到这个氛围怎么样？

LYN：就感觉人数也不是很多，于老师一直问博一的学生怎么都没来。

GX：那博一没来是因为人家考试。你博一没有做多少东西啊，你论坛总得说点什么，那东西就没办法参加。

LMN：那你俩都是啥感受？

GX：就是觉得简单的交流一下吧，然后也没有特别的，其实解决不了你什么问题，解决问题还是得靠自己，因为首先这个论坛就不存在挂和不挂的问题，所以我们准备的可能就是挺水的，或者一个什么东西随便就拿去了，然后就是为了完成任务，然后也没有什么实质性的，也得不到建议。因为你不挂人了或者不怎么着了，然后老师一般也就不怎么提问，或者是他简单说一说，可能结束了就可以了。然后卫老师还要求一个人一个小时，到最后了老师都开始硬聊了。

LYN：我是觉得没什么互动性，就是包括现场同学，虽然你老师说大家有什么想说说的。大家提前拿不到东西，然后也看不了，也没有什么可交流。再说了那个交的都是一个摘要，很短的，就一页多，再加上大家讲东西根本就没有吸引力了，这讲的我根本就听不下去。就是xx学姐那个，我虽然对她题目很感兴趣，但是她讲了两次我都不知道她在研究什么，因为我真的听不下去。但是我觉得是跟培养模式有关系，你像国外的话，博士你就必须独当一面，你从头到尾你就得自己去研究。自己钻研模式。为什么国内的高校那么喜欢国外的留学生，他培养模式就不一样，人家确实博士毕业以后就是能独当一面。但是我们大部分都是抱着老师大腿，你说你能独立研究吗？可以是可以，但是发文章可能太难了，发不出来高质量的文章。或者跟我们的评价制度有关系啊，你不抱大腿、不挂人家的名字期刊也不发你的文章。那你发国外文章你不找国外的人那你不也没发出来嘛，自己你发不出。

我觉得可能就是培养模式是一个原因。另外一个肯定有我们看不到的东西，比如说国外，它的学术水平上就是要比我们高。你没有想过这个问题？我们就是以美国为老大。而且了，人家发的是英文的期刊，像我们也发英文期刊，但我们母语不是英语啊。

GX：但是有几个老师他在分享的时候都已经说了，现在语言真的不是我们被拒搞的唯一原因，就是占大部分的原因不是因为语言，因为现在有很多润色的公司的，但是可能做润色好像也不是那种学术性的语言。但是国内有很多老师跟国外老师合作了，他们母语是英语，也能帮你改了。

LYN：我自己的感受是……我不知道对不对，我觉得国外的这个研究是积跬步行万里路，他们是真的做的很小很小的一点。但是我们的管理学了就是画大饼，一下子就挖一个这么大的坑，然后一下跳这么大步伐，会说我这个创新点有多大多大，然后研究很大的其实特别虚。反正我不知道是不是我错觉，反正战略这边真的是研究这一小点点，然后100个人堆上一大步，我们（国内）是一个人就迈这么大步，给人一种不实在的感觉。

可能我觉得教育体制可能就不太一样，国外不是像咱们中国博导老师给一个名额，国外是如果你没有那个项目或者没有课题什么的，你是不能招博士的。其实人家的招博士是有门槛的，你不像咱们。那我是觉得按照他这种形式进展之后大家也会真的就干活，不仅是说为了培养出来一个博士，而是真的需要他。

但是我觉得我挺有信心的，因为我总觉得各方面都是因为国家发展阶段的问题。但是年轻一代学术人员出来你会发现这些新博士们搞得也很小，就是也慢慢也会变小变细，特别从国外回来的那些，慢慢就好了。

HQL：我觉得崔老师是特别有对知识的那种敬畏感。

GX：太敬畏了，让人可怕，哈哈哈哈。他对我们毕业论文要求特别高，就是你发的论文只是学校的要求，毕业论文学校尽管没有要求，然后他也特别希望你能有一个特别好的作品。

LMN：挺好呀，然后你们学生怎么想？

GX：我感觉我们觉得压力挺大的，因为慢，主要是时间。就是我们所需要的跟他所需要的就不太一样，我们可能就是毕业导向，然后他可能就觉得这种能力很重要。他会觉得因为你既然选择了来这读书，就说明你一辈子要做科研的，那要做科研的你肯定得有科研能力，你有了你才出校门啊，你能力都没有，那你别出去了之后有什么用啊？

LMN：你们有没有跟老师沟通过你们的想法？

GX：他知道，他知道他也会这么说，他还是始终相信他自己教育方法是没有问题的。然后每次他都会说，我是因才施教的，然后都是适合你们的，让你没有办法反驳。

LMN：但其实是一样的，对吧？

GX：我觉得应该也不一样，因为每周你如果交东西的话他都会沟通1-2h，然后他会问你下一个解决问题需要多久，然后你说完之后，然后基本上你也会按照这个时间，或者是即便晚也不会晚很久，然后再给他发过去，然后再改下一个。你知道我那个彭旭师兄吗？他写了三篇毕业论文，你们知道吗？就是30万字！就是他写了一篇，然后后来我老师不知道去哪好像开了一个什么企业社会责任的会议，然后他需要写那个，就是那种世界的那种企业社会责任报告，然后英文可能就我师兄好一点，但是博二了，我师兄原来是做知识转移的。然后老师说，你别弄知识转移了，你弄这个吧。然后因为需要读很多之前的那个报告，但是我们其他人很多功底都不太好，因为他好像是澳大利亚的嘛，然后英语还算那个可以，所以就带着他弄这个了，然后就发论文，写论文全都是那个方向了，他就彻底改了，然后那个论文他就写了一个，还有还写一个什么，然后最后就是说改就改。就是感觉我们师兄对哪个东西一看，那个东西好像在闪光，他就必须得变过去，你知道吗？他就特别有热情的一个人。然后他也特别辛苦，他基本上除了睡觉和吃饭不干别的，他觉也少，他每天就3、4个小时的觉。我师兄简直就是特别热情，他身体特别特别不好，你们可别学。我师兄那时候因为他是华裔嘛，所以他没有咱们这种医保。然后当时他腰疼这疼那疼，还让我给他去医务室拿药，我说我怎么拿，他又教我让我去描述我有那个症状，然后去帮他拿，学校医务室便宜嘛。就是他其实就付出很多，他年龄很大，他现在已经40多岁了，所以他付出也挺多的。之前疫情的时候，那时候还没有毕业，他就回去了，然后回去以后那边比较严重，不让他回来了，他又在那边做毕业论文，反正也是在线上答辩的。但那边现在对咱们学校认可度不是特别高，所以他在那边找工作也不好找，然后所以他在那边想再读一个博士学位，他现在正在读。他现在发文章特别厉害，反正现在发2A就是就随便发。他特别辛苦，到现在还没有找到工作，就是找国内的一些学校，然后他就是想发文章，挂点名什么的，但是都没有办法入编制。然后这个问题就是华裔这块，有很多学校都不愿意担责任，还有这种知识的这种或者是专利的那个啥就很防着。他现在就是发文章，他去年发了五篇。

我们老师的那个意思就是厚积薄发，好像每一个人都是这样的。我们老师很注重学习态度，你先别说做东西怎么样，一看你态度不行，比如说最近如果最近你汇报的东西少了，或者学的东西时间短了，他就觉得你态度不行啊，有个博士样吗？

他导师不是那个王巾英老师，然后就挺厉害的，当时好像文化大革命的时候，咱们这边不是停招了嘛，但是东北那边一直还在培养，没有停，所以我们当时在建校的时候，让那边过来一批老师，王巾英老师过来以后反正就没有回去。然后老师是跟着他，现在王巾英老师好像是80多岁了，还是挺大的吧，每天看六个小时的书。

我是到现在都没怎么看过，做到哪的时候才会看文献，不会主动去看文献。我现在就感觉自己的积累不够，我感觉一天我感觉要榨干了。我师兄现在说他终于觉得有一点像能驾驭理论的那种感觉了，他毕业的时候发文章cssci就已经很顺畅了，但是他那时也没有那种驾驭理论的感觉，然后后来这是第七年了嘛，就有这种感觉了。

HQL：我觉得还是需要长期导向。

LYN：所以窦老师说了，社会科学不像自然科学一样需要智力，咱们这就需要勤劳。但似乎我们现在的博士和以前像王巾英老师比就够勤了。就不一样，就没有那种发自内心的爱。

GX：我倒觉得那个时候也不是爱，我觉得很多就是把它作为一个工作，那时候压力没有咱们那么大，那时候也不能换工作呀，咱们这时候一生能换多少个工作呀？可能五年之内你就会换，或者是十年之内你就会换两个工作，现在真的是对于年轻大家讲压力确实很大。一个就觉得有那个铁饭碗了，就感觉做什么事都挺踏实的。我真的觉得这个稳定性啊，对于学者来说很重要，之前咱们有那个终身教职的保障嘛，但是就现在还需要什么“非升即走”或者如何如何。我觉得没有这个保障之后很多人就很浮，就是我真的我需要先保证我这个饭碗之后我才能去稳下来，但问题就是这六年非常急躁，可能已经把我的精力值给耗尽了，到后面就也没办法改变了。

LYN：怎么说呢?我觉得这个跟很多有关，就是你不能说一方面原因。还有一个时代的原因就是卷这是一个阶段的问题，而且除了那个他们工作稳定，他们可以专心干一件事以外，另外一个那个年代的人都很淳朴的，他们有种敬畏心，老公他妈是那个做文字工作者，可发愁写东西啊，但是人家可以不管自己怎么发愁，但是也可以把这份工作做的很优秀，他妈是给那个领导性写稿的这种。那时候就是他们不是爱一个干一个，而是干一个爱一个，但是现在大部分都随时代发展，我们更有自己的思想了，我们是先爱再去干。上上周我还跟新哥讨论了，就是那个国和日本也有我们这个阶段，日本的卷已经过去了，他们在90年代的时候也卷的不行，是因为人口达到那个阶段，然后国家发展达到那个卷的阶段。像旅游业也是，我们现在是现在是世界的主力，对吧？全世界各地都是我们的游客，但是其实90年代的时候大家都去日本和韩国的，特别日本，你像他的那些科技企业在全球都是数一数二的，这些东西，所以他们的游客真的遍布全球，原因他们已经到那个阶段，但是现在已经过那个阶段，现在一方面人口本来就少，另外一方面反正就是中国的发展，就是日本90年代的那个路线的就是我们现在发展的那个阶段，就该卷了。

但是慢慢的你会发现人口越来越少，他没法卷。就是我们这一代就是在物质保障比较好的基础上，然后到这个阶段就真的要拼了、就是比呀，因为我们物质上，父母都给积累了一定的物质经济基础，这个时候就得比上大学了呀，那我也上大学你也上大学，我们就得竞争工作了。但是老老师那个阶段不是所有人都能上大学，上大学人还是少，是特别特别少，所以才能分配呀。你可能通过访谈会总结出一些原因，但是我觉得这些原因他不是说有先后，或者他们是叠加起来造成了现在这种局面。

我的感觉是时间变短了，那种开心的感觉也短了，然后也没有什么荣誉感了，就那时候在一个厂子里边当会计，然后姥姥都给自己买个金的小算盘在那摆着，就很开心。但是现在呢？咱们就想着那还不如买个包呢，买lv或者买什么，因为我们太幸福了，期望值已经拉高了。他们那个时候苦，有一点甜就很甜了，但是我们是一直都很甜，所以我们需要很甜很甜，才会感觉到甜多了。

我一直有个问题，我觉得商学院在中国就是一个帽子。因为很多学校可能就是有一些学院，我知道管理学院或者经济学院，只是我们学校叫商学院，虽然我们的使命是为了培养商科人才怎么样，但是经济学院和管理学院就是对应的学科是没有什么区别的。我不知道国外商学院是不是真的就是为了培养商科的人才，我要学什么市场分析，商业分析什么的，而不是我们学校的市场营销什么的。说实话我觉得虽然我不了解国外的上课到底怎么培养的，但是我觉得我们中国的商科没有说培养一个商业人才。那你简单来说商学院是不是培养你将来要自己做生意或者怎么样，那不是那，我们大部分人出去不还是打工，根本就没有从小树立那种我要创业的意识。商学院教过你商业模式创新吗？教过你商业画布吗？你都不知道，更不用说搞创业了，如果不读这些书，我都不知道商业画布是什么东西。

你像那个我师弟，他们就是在英国读书上课，我觉得可能会跟我们的培养模式稍微有点不一样。我们这里是盖一个帽子，比如中央财经大学没有管理学院，就叫商学院，或者上海财经大学也叫商学院，但是有一些学院只是把经济和管理分开了，叫经济学院和管理学院。最起码在山东大学跟中央财业大学在管理学方面的培养是没什么区别的，山东大学那叫管理学院，中央财业大学这里叫商学院，但是我觉得说模式上没有什么区别。这也算是一个比较有意思的点，所以我一直好奇的点就是你们一直在说我的题目是研究商学院怎么怎么样，那这个就出来一个问题，那中国的商学院跟管理学院有没有什么区别？

LMN：我们可能到时候会说明一下，就是关于商科教育相关的都算，就是其实我们是以商学院为案例研究的对象，那我们研究的问题可能更多的会涉及这种专业性、专业人士甚至可能会推广到高校，只是说商学院的表现更突出。我们为什么会关注这个研究呢？因为西方做的比我们更前面一点，在西方这个新自由主义的这种意识形态之下，市场逻辑已经侵入到高校。高校本身应该是学术逻辑或者是专业逻辑为主导的这样一个场域之内嘛，就导致出现了很多腐化变质的现象。

那在中国肯定有很多不一样的地方。首先我们很明确我们是国家管理一切，我们的行政逻辑非常的明显，非常的鲜明，所以说我们会有西方不一样东西。你像很多公司的话语体系都进来了，比如叫我自己的导师为老板，

GX：这个工科比较多，因为工科很多老师本来在外面就有开公司对。

LMN：另外绩效管理主义，尤其特别是非升即走，这就完全是市场化导向呀，绩效考核呀。你说现在对于学生的培养、对老师的培养，他们是以学术探究为主的吗？如果真是这样的话，那学术讲座不至于搞那个样子，说实话，我就是很愤慨。

LYN：可能阶段不一样，我很少有愤慨的时候。就是我知道年龄不一样，你更血气方刚，可能你就是更理想一些，有自己的理想和志向，你会希望学术是怎么怎么样的。可能随年龄增长就可能已经习惯了，习惯了之后你接受度可能就高了。我觉得这是一种赤子之心对挺好的，就需要这种敬畏之情。但是怎么说呢？就是可能有多方面的原因，另外一个就是还有我学院培养跟业界还挺脱节的，但是你看国外的学者就包括我师姐那天听讲座的那个人，他是自己开医院的，他是创业者，但是他现在又发文章，搞创业者健康的，因为他的医院平常会有很多数据。就是国外有很多这种人，我是感觉他们相对来说比我们脱离的要少一点，就是他们有很多人搞着学术的同时人家真的创业成功了，就是我确实一开始搞学术的，我搞搞我发现这个我要去干，然后他就搞创业，他就成功了，很多这样人。但是中国很少，中国真是搞学术就一直搞学术，然后他出去以后发现他根本就不适应。所以我觉得也有点可悲吧，就感觉做的研究好像有点自说自画，派不上什么用场。但是我只能说跟学科有关系吧，毕竟我们是社会科学，但是自然科学就不一样，那工科真研究出来的东西是真……你比如说山东大学机械臂估值好几个亿了，每次创业大赛都是金奖，机械臂在全国推广。有很多那个物理学院，什么生物学院，他们真的搞出来的东西有专利，然后真的可以卖很多很多钱，这跟学科有关系。我们这个社会科学就注定了我们不能立马兑现。

HQL：我有点好奇，我们一直都说我们商科属于社会科学，很多同学都这样告诉我，但是我们现在似乎有种倾向，会更热衷于各种数据。

LYN：我觉得就很简单的来说，我感觉质化研究在商学院属于一个边缘方法。我觉得是这个样子，但是可能还是学术发展阶段吧，不过我自己就觉得可能质性研究的意义对我们来说更大一些。然后还有很多学科，比如战略越来越卷，就追求什么用机器学习呀，然后大家都用同样的方法发出来论文了，你只有用更新的方法你才能发出来。那这个时候就只能跨专业了，用什么脑科学、神经学之类的。

HQL：你觉得真的有意义吗？跟师兄聊起来，他就说上战略管理课时，他觉得那些很老的文章，70年代时候的文章之类的，但是他觉得，哇，好有洞见呀，但是人家也没有用什么很高深的方法。

LYN：但是那种人很少呀。似乎我们现在也不培养人的“洞见”，而且我们会鄙视你满篇都是文字。所以现在国家也慢慢在调整啊，就是破除“五唯”。但是其实也没有别的好办法，如果你只破不立，大家还是更关注你发文章。怎么说呢？这种东西是没有办法解决的，是发展阶段的问题，我觉得将来某一天我们会更追求它的实际意义。但是现在这个阶段就是因为卷各方面，你比如说举个简单例子，就是很多公司都是都要985高校毕业的嘛。你说国家是规定了说不能学历歧视，不能以这个为标准，是，现在公司不提了，但事实上。你比如说我就招十个人，我有1万份简历，那我怎么筛？我不就是先把非985的排除出去，然后再在985什么华东五校什么清北的，我再留一下。然后我再在这里再看，就不看条件了，我就看简历了，谁真的符合我们这个岗。没办法，你就只能先刷下你一批，然后你再去挖掘真正的人才。但是刷下去那批确实有可能有人才，那没办法呀。

LMN：但是你像你刚刚说的，就是虽然是我们现在发展阶段的问题，然后你说以后可能会好起来，但是现在如果说少了一些，就是学者主动自发去改变这种事情，他怎么能好起来呢？靠国家的倡导吗？

LYN：确实是像你们说的，现在很多事情就是良性循环就会一直良性，恶性循环就会越来越坏，就是也没办法。

LMN：你有没有感觉到就是这种实证主义的泛滥呢？

LYN：我觉得就是这种倾向！怎么说呢，这就是各方面的一个循环。比如很多老师都说，哎呀，质性不好发，如果做这个的话我什么也评不上或者怎么样，对吧？就没办法。如果他实证做出来了，他就得到了正的激励，比如说他发出来一篇，他想我再发两篇的话，我就能得到副教授了，然后他肯定就特别有动力的去做另外两个了。发出来之后他可能有了经验，就会觉得自己希望更大了，他绝对不会去看质性研究的，他肯定还会那个啥。

不过说实话，如果真的不是唯论文论，我相信肯定更多人愿意去搞这个质性研究的。其实怎么说呢？这些学者骨子里是有那种高傲和清高的，我们愿意做出点啥。但是有时候就是这个职称逼的我们不得不去搞一些研究。但是如果没有这个要求的时候，我相信有很多老师愿意去搞。对，其实很有意思，要不然我也不会做，比搞那些数据什么的有意思多了，虽然后续编码的时候可能会有点难或者怎么样，但是你在这个过程接触到很多东西，你会觉得做的是有意义的那一类。

LMN：就是整天整理那些表格啊，跑数据啊，你觉得这是研究吗？

LYN：就是刚访谈的时候很新奇嘛。在不断看的过程中，每看一次就会有新的发现。其实那个时候还是挺自豪的，就是比当时整理excel数据要自豪的多，这是真的。嗯，挺好的，我觉得做这些，那你们要一直做下去吗？

LMN：毕业论文……你看你俩都给否了呀，很多老师也会说这不是企业管理，这是个很大的问题是，我们也考虑过了。

LYN：那你们还是又倒回了，你们确实也面临着将来要毕业嘛，你们不可能只做这一个项目。我就是觉得你们也应该为自己考虑。其实说实话，这研究要付出的精力还要挺多的，因为你们访谈每个人五十分钟还挺大的。

LMN：其实我之前就像跟你们是一样，就是不会想去毕业，我真的我不去想同辈压力，我就是想能学点东西，就是实证这一套我大概知道该怎么搞了，那我就想学。尽管我老师看不起质性研究，我导师之前都不承认质性研究是个研究，你知道吗？我只是觉得就是做一个博士生吧，我觉得应该有一点自主性，而且我觉得我要对得起那文凭。我发了十篇论文又如何？我不羡慕这样的人，说实话啊。

LYN：所以说实话，你这种人才适合搞学术，哈哈哈哈。

对了，有一位同学跟我们说，他们学校那个马克思学院的老师是被重点关注的对象。就马克思学院的老师会有很多独立的思想，然后他们更容易冒头。我现在觉得有思想的人比较少，大家好像在说一样的话，是吧？对，这个没办法，就是各方面压力，有些东西就是不能说，不过我们还好，校园环境比较简单。

LMN：你知道姜老师咋回事，为啥走了吗？

LYN：他老婆跟孩子一直在国外，他都两年没见他孩子了。他孩子正处于成长期，朋友都该不认识他了。我觉得他牺牲挺大的，就不管他去英国发展好还是不好，其实他放弃这边的一切他付出的还挺多的，为了家庭。但是我觉得挺好了，因为工作什么都可以再建立，但家庭没了就难搞了。但是确实放弃挺大的，这边毕竟到副院长了，其实他马上就可以评博导了，到那边的大学好像排名全球750，而且好像是当副教授。他当时还投了一些更好的学校，但是也没成功进去。这边博导应该也马上就评上了，但是为了家里没办法，那孩子正处于成长期，他老婆不可能回来，人家从整体发展来看对于他们来说，国外对孩子的培养可能会更好一些，而且他拿的好像是终身教职，我觉得对于学者来说，英国的环境还蛮好的。我室友在英国嘛，他就一直跟我说那边环境很好。我没出去过，但是我确实听说过说国外这个制度。让学者们更幸福，可以专心的钻研，而且他们的工资应该相对来说不低，对于他们来说。没有经济压力，他们可以安心搞科研。

你像那个周卫中老师不是在日本呆了很多年嘛，他回来就说，自从回了国以后都没有看过纸质的书，书太贵了，而且学校好像不怎么报销，就是不给买啊，说咱们学校每年才才买200块钱还是买500块钱的书，他说就不够看。他说回国后就没怎么看过纸质书，整天看着这个电脑啊，他觉得不开心。

就包括那个清华那个叫严宁的老师，就是很优秀的一个工科的老师，一个最年轻的女教授，然后也出走去美国了。然后很多网友也就瞎说嘛，但是其实我觉得她之所以离开，可能是她真的想搞出什么东西，但是可能有一些环境不太适应。

LMN：就之前有人说嘛，就咱们现在这个非升即走的制度，爱因斯坦大概第一个考核期勉强过，第二个肯定得走。

LYN：所以就没办法，可能就各方面原因吧，慢慢来吧。但是现在国外的形势不好，很多人愿意回来。

LMN：你还能回忆起你们上课的时候，就是有没有印象比较深刻的课堂或者是老师。

GX：哈哈哈哈，我又没起我导师上课呵，然后本来上到9:40都要聊到10:30了，有时候他还坐到桌子上，他一个屁股坐上去，然后让一个腿就这样搭着，然后就在那里讲很多东西。他经常有那种比较开放的话题，就比如说石油了什么的，就课堂上每个人都得发言。那比如说十个人，他就从这开始，问大家对一些问题有什么样的想法，问你们觉得这个是什么原因就造成了，然后大家要讨论，有没有什么想法。我觉得这种开放的环境很好，大家脑子去动，你像我们从高中开始就封闭式管理，没有什么创造力的。

LYN：我老师就很少去否定别人，老师说咱们是可以讨论的，他希望我跟他去讨论，不是说老师说对就对。当然好几次跟我强调说我们俩是合作的关系。你不要把我当老师，他鼓励我说，你看你有时候经常跟我说些话，你会给我启发，然后你可以去否定我，或者怎么怎么着的，需要battle。

GX：咱们就battle不起来，就潜意识里就同意了他的话，可能你有自己的想法都没有去说。

LYN：我在咱们学院，我现在想不起来我听过什么有意思的东西。

就是很有意思吸引我的东西我想不起，可能有，但是我想不起来了，可能一下子想不起来。但是我在山大的时候可能就会有，因为我们会听一些历史学院或者是社会学院的课，他们讲的东西我觉得很有意思，那边的老师大胡子一留，他们讲的东西很有意思，你愿意去听，因为人都愿意听故事嘛。他们讲的确实好，而且他们的思想确实会比我们要深一点，其实就还是说一个问题，就是说研究应该是一个很好的故事，但感觉我们的教育呀这一块还是比较缺乏，我自己是这么觉得。你像咱们战略呀，不论是我们去表达呀去做研究呀都是要去讲一个比较好的故事嘛，因为我们的模型比较简单。

HQL：就是刘老师经常会说这个管理学不仅仅要强调科学性，还要强调艺术性。那仿佛我们的教育体系当中，或者是老师教育过程中，人文的东西比较缺乏。

LYN：怎么说呢？我觉得老师们都没有，他们怎么传导给我们呢？咱们学校老师讲文章也不是讲故事呀，对吧？那怎么传导呀？所以还是研究目的不一样，你像国外他们可能就是有那种项目，然后就会以实际出发，但是中国这些研究者可能还是看看怎么写。你们可以访谈一下芳芳，她的感受会跟我们不一样走了吗？现在你跟人家打电话，他在家，他在广西了，你跟人家打电话。因为芳芳是在在公务员体系工作过将近十年了，他偶尔就会表达说，现在搞的是什么东西？她就突然找不到自我了，就是觉得没意义啊，就因为她工作过，她知道那些不能解决实际问题的。所以我们现在研究的东西，她会觉得咱们现在的研究很没有意义，她可能会比我们更有感触。

LMN：我觉得好的肯定是有姜老师的。然后说一个不是特别好的一个例子，就是咱们上的那个领导组织、领导力那个课，记得好像快要末尾的时候张宏宇老师给咱们上过，当时上课的时候她就讲投稿的经历，投稿的经历那么曲折，特别是听到说是她当时要生孩子，然后那个她的母亲好像生病了，在医院照顾。我当时说实话，我听着挺压抑的，我不知道你当时什么想法。

LYN：我没想过，就觉得张老师好厉害呀。然后特别是最近，我对她印象很深，因为她评博导了嘛，我在想这个学院就是这个社会吧，可能女性真的很难冒头，你要付出比男生要多十倍的努力。其实就是你想，咱们私下里说就是王振老师很厉害，确实发文章很厉害，在学院也很有名。张老师的名声就没有那么大，但是其实她也很厉害呀，她今年评估的他多大了，她没多大吧，然后刚生完孩子，也就是说她生孩子同时她一直在发，保持着发文章，然后评上博导。就张老师还是很厉害，但是她在学院的名气就没那么大，很多人也不认识她，对吧？也有可能性别只是一个原因，就是跟人有关系，有一些人就愿意讲座呀，交际呀，但有一些人他都不愿意弄这些，但是我还是觉得，女性还是要付出很多很多的努力才能平衡或者是跟上男性。

HQL：我觉得我们学院也有女性领导，比如葛老师，李季老师。

LYN：李季本身很厉害，他跟王老师差不多同时毕业，然后是三北的，我老师是三清的，我听我们班同学刘洋讲过，就那个时候他们俩是学院的风云人物，因为那个时候才开始，我听我师兄隐约说过，2003年我师兄来这个学校上大学的时候，咱们商学院还很差，整体还挺差，那个时候好像刚组建不久，也没有很厉害的老师。好像就是从王老师，李季老师他们来以后才会慢慢卷起来，2006—2007年的时候，慢慢的开始有清北的老师来了，所以他们俩是一个三清一个三北，所以在学院就很有名，就是风云人物。然后这个学院才基本上有清北的。以前的时候都是老老师嘛，事业也也很厉害，学术上搞得应该也很好，但是那个时候学院刚组建，还没有很那什么。

LMN：不过我当时我听完之后，我的感觉就是有两点吧。第一点就是我会觉得，为了发这个文章牺牲很多，就是我难以想象她的合作者在她母亲的病床旁然后敲字。就是我有点于心不忍吧，就是这种感觉。

LYN：那你要想到一个问题，如果她不是当老师的，她已经很幸福了，她可以有时间去病床上照顾他们。如果是企业上班，你觉得你领导会给你批一星期假，让你去病床照顾你妈吗？一次可以，但是你总这样肯定不愿意，但是你当老师你就会很自由，你可以去病床上照顾她。但是你其实有很多不知道的，就是有很多那种人，父母生病他们都无法在床前照顾，只能雇保姆的。其实我听到这个我还有种想法，她其实好像不努力发论文，她也没有什么威胁，她也不会被辞退，这是她的一个个人选择，大家都想出头嘛，想上进。谁不想有名？

一方面，我不太了解，我是自己臆想的，就是当你在学术上真的有造诣的时候，有成就的时候，你出去可以赚钱的。你想呀，一方面平台差不多，你想张宏宇老师的合作者肯定平台都已经很不错了。然后你出去，你真的有名了，那你都评了教授了，然后有很多企业愿意邀请学者们去讲。我自己理解，这不是说学者真能解决他们公司的问题，一方面是可以帮他们解决，另外一方面是帮我们打广告，他们说哪教授来我们公司给我们讲东西，会给我们分析问题，这说出来是很厉害的。

所以我上研究生的时候，我就感觉就是企业不一定是真心真的崇拜这个学者，然后让他来给我讲，而是说为了跟你建立合作关系，我为了让你给我指导意见，所以我邀请你来，他不是真的尊敬你，就是互相吸取利益吧。所以你像欣哥和他弟他们俩，一个在北京一个在上海，他爸爸生病都没办法，都只能雇佣保姆的，他们俩都不能回去，那公司不可能让你一直请假的。那你想他们要是真的请假回去了，那要有工作不也得在床前敲字吗？你把老师这个职业也当个一个工作，只是这个是在事业单位下，你就能理解了。

LMN：我觉得这是一个感受啊，另外一感受就是有没有必要在我们还在刚刚上博士的那个阶段，就给我们灌输博士这么辛苦？因为她投稿的过程经历很多挫折嘛，就有没有必要在一开始的时候，而不是她说她多么的热爱科研，或者是给我们一种正向的一种激励。

LYN：我觉得这就希望让你们早点认清现实，因为大家都是成年人，你现在选择读博这不是一年能做完的事情，所以需要跟你说清。

LMN：但是问题是说清楚没问题，但是我觉得也要得全面一点，至少让我知道科研有什么好的地方。

LYN：其实我老师不是旨在分享负能量，而是想让你们真的认清你们到底是不是喜欢学术，他也会有正能量，但是他会觉得正能量你自己都已经想的差不多了，所以你要认清现实，他是希望你可以认清你到底适不适合读博士，然后你如果合适的话，你可以走这条路的，不适合你的话就赶紧去工作呗。因为咱们学校说实话，硕士的那个找工作还挺好找的。

HQL：然后我想了解一下咱战略现在比较前沿的研究是啥啊？咱们现在是比较倡导把研究做在祖国大地上，因为我听他们OB的讲，就是会比较跟随西方的话题进行研究，就是他们会引入很多的概念，比如说这个变革型领导等等，然后有一个现象就是说，他们会通过这些概念来解释我们中国的现象，那个同学他也说了，就是我们中国的领导其实可能会有这些变革性的，会有威权力型的，但是我们中国人往往都是见人说人话，见鬼说鬼话，可能是多种因素的叠加，那西方的这种概念可能并不完全能够解释我们的这种现象。就是我们研究当中就很缺乏从中国本土的这种特色去做相关研究。那他还指出，像在台湾的话。那他们就有个本土心理学，那就是只就是根据自己的实际情况去做研究就不会说要去跟随西方的研究。

LYN：那我们战略的话，我觉得我看的文献可能比较少的原因，我倒没有涉及到就是他们写文章会以什么中国的什么什么案例或者以美为例，但是其实很少个体现像，因为人力呀，还有心理呀，这确实跟文化有关系，但是战略的话，你说这个市场战略，因为你的目标人群会涉到文化，但是战略本身很少有这种什么在中国适应然后美国不适应，然后还是比较具有普遍性的，对，我是觉得战略是这样。因为它没有那么细，我是觉得OB呀，那个心理是底层建筑，我觉得那个战略会拔高了一些，它更多的是一个战略，然后人力就是一个战术的问题，战略就是不需要那么细。所以不是说可能会有一些概念是只适合中国不适合其他国家的。

根据我看到的文章，我觉得在这方面就是你比如说那个变革型领导，中国会提出什么家长式领导。但是战略上提出这种概念的还挺少的，我觉得挺少。也很少出现说这个理论在美国比较适应，但是因为什么什么然后在中国不适应了。很少有这个作为切入点，在语言中就是写出这个，然后来研究一个新的东西，我感觉是很少。有人提出，比如说创业拼凑这些概念，拼凑者的意识呀经验、文化不一样可能会有不同的结果，但是真正去研究这个很少。因为我们很少涉及到心理的东西，战略就很少用到心理学，所以它不会抠那么细，所以那个文化对它的影响就不是很大。但是当研究创业者的时候，可能就会涉及到国别差异，对这个创业的影响就会有了。但是这个不涉及到水土不服呀，因为人家国外也认为这国籍差异是对创业者本身是有影响的。

那我换个角度来说的话，我们学术人员的研究主题会跟随着西方的热点进行研究，我觉得会。不过这就是我自己的想法，看的文献也比较少，不具有代表性，有欠缺。那我没有看到过说哪个学者根据中国的情景下，然后提出一个新的理论，肯定是有，但是我没有看到过，好多东西都是跟随国外的，包括很多人写那个本子，写什么自科什么的，都是看那个top期刊，然后可能里面会有一两个词人家提到但是没有深入研究的，然后国内有些学者写本子的时候就会按照这个做下去，那就很新了，很新的时候可能就会中。感觉西方的、美国的，创业者健康的就是前沿，很多人都追溯，老师们都让你引用，毕竟是好的嘛。

HQL：那你觉得这是合理的吗？

LYN：那我自己研究不出来，从现象出发是需要阅历的，我们这个阶段很难看出来，那就只能去模仿别人，对吧？但是前段时间不还有人说，就是发top期刊的中国学者越来越多了，而且国外很多学者就是愿意跟我们国内的学者合作，因为他们想要中国数据。

HQL：你觉得这是一种进步吗？

LYN：我觉得不是什么进步，是中国作为第二大经济体，然后可能是因为这个中国新兴经济体发展特别快，所以国外这方面的文章可能比较好投，所以他们国外的学者就愿意跟国内学生合作，很多老师都是的，你包括很多人跟林老师合作，跟我导合作都是想拿到中国数据，然后一起合作写文章的。

LMN：他写出来的这个东西和外国的东西有什么不一样吗？

LYN：嗯，不一样。

LMN：我就想不知道他会不会突出显示出在中国的一些管理经验呀？

LYN：有一些会有呀，你听过那个阴阳吗？那个说阴阳、八卦、量子理论跟这个战略的结合。我觉得说实话，他们会把那个量子理论拿来放在管理里。那领导者可能都不知道量子理论是什么，这怎么用啊？但是那个什么法家文化呀、儒家文化呀是有的，咱们战略的于晓东老师，他不是研究这个姓名中出现了这个儒家符号对捐赠的影响什么的，是有这些的，国外很喜欢，国外还挺喜欢的。

就是之前窦老师就讲了，有一些国内好发，有些国外好发，但是你要判定国外好发，但是那个该不该发。比如说有些人研究中国企业的腐败，中国政府的腐败对于企业什么的影响，那国外是好发，你敢发吗？政治导向不对呀！你得考虑一下你要不要发就这样。但是你突然提醒我了，就是你像那个阴阳就是中国传统文化，现在研究还挺多的，跟管理的结合。什么管仲啊，什么法家啊，阴阳什么的。

LMN：之前有个学生开题，就研究了“闯”，然后把它作为调节变量去看他对领导者的影响。

LYN：其实这只是一个用了一个新词而已，国外的那个创业研究是有这个变量的。就是有一些人是那个创新型的或激进型的，他只是用了一个很有意思的中国字，但是国外也有闯这个字眼，人家只是学术上没叫闯而已，他好像只是在响应国家的号召，但实际上好像也没变化，不就是那个向上进取劲儿嘛。

HQL：那我们跨国呢？跨国这方面的研究的话会不会更西方化？

GX：没有。跨国这两年我觉得研究那个知识转移都是那个什么会关注新兴国家，比如说像印度的这个。以前都觉得外国的东西好，然后就比较先进，所以他们很看不起中国，然后很多人了解到中国组装那仪器，就大部分都不用看说明书就可以组装起来，当时他们就惊呆了，就觉得中国的已经不是20年前。因为当时他们来旅游什么的去过深圳，他们觉得深圳好破呀，结果后来就是对中国刮目相看。那我们就想看，就中国进步太快了，所以中国进步是怎么快的？就说外国的国际化就是为了获取市场，获取更多的利润，占据更多的市场，然后像中国的国际化就很多都是被动的、被迫的，就比如说中国的运动服装，他卖到国外去并不是想占国外市场，他就想跟国外竞争，然后收购一个国外企业，学来技术，然后在中国内部跟国外竞争，因为国外的市场把咱们都侵占的不行了，所以中国的国际化其实是被迫的，他只是想在国内生存下去，所以他迫不得已国际化，这样也是一种发展的方式。那我想说做这种研究的，就是关于咱们这种新兴国家的，很多都是国外的，加拿大的比较多。

我又突然想到前段时间看那个什么儒家文化、四书五经、心经什么的，然后你会发现啊，就是有很多这种管理学的理论。你会发现国外的人对我们这个孙子兵法什么的理解比我们要深得多。

LMN：我觉得你对很多事情，看到都很积极正向，就你在学校里面、学院里面有没有一些你觉得不太好的一些事情？

LYN：怎么说呢？我是讨厌一个人啊或者讨厌一个事很快就能忘记，就不太深想，但是当时我会吐槽。我从小就不怎么喜欢那些特别会说话、圆滑虚伪的人。我可能跟老师接触也不多，我对学院评价特别高，然后我就觉得挺好的，可能毕竟是北京吧，比较务实。我有吐槽过学院有什么不好事吗？好像没有吧。

GX：我也没有什么，就感觉都挺好的。有一点就是我觉得学院什么事情都通知特别晚，有的我就不能接受。就比如说明天开题了，前天才通知。然后你就觉得这么大个事……

LYN：老师们的时间都说不准，每次都得预约这个，因为卫老师要一个老师一个老师敲定时间，然后还能还得保证这几个老师在这同一时间的都有空。

GX：我是觉得有个什么通知都前一天晚上才通知，然后过两天就必须得交，不交后果自负了，好像是有好几次都是这样的。

LYN：就是那个咱们学院现在，尤其是这两年还是很重视这个大家学术发展的，因为毕不了业嘛压力大，然后就很重视嘛，又开始给我们弄各种讲座。但是有一个问题我自己是觉得有点太在意，因为太在意学生、收集学生的那种看法，然后去给他解决了，就是咱们之前不是开过一次座谈会，就是让博三博四然后就是年级比较高的去那坐谈吧，有什么困难你就说什么，学院都记录下来，然后去给你解决，比如说之前就有人反馈说那个分组。就是安排的老师对不上，就是不够有指导性建议，所以这次论坛就是让每一组自己去找学生，但是我发现一个问题就是，我是觉得现在学院过于关注学生们的这种反馈了，就是过于关注了你，如果大部分人都认为这是一个问题，我们可以努力去给解决。但是其实你会发现冒头的就总是那一两个人，他总是提出五花八门的问题，你这次让大家自己去找老师，然后这次不是还有一个反馈意见吗？他又提出另外一个问题了。

GX：这是学生的问题。

LYN：不是，就是我是觉得学院没必要那么在意每个人的那种反馈，就是你可以解决大面问题，不用每一个都给他去解决了，因为你把这个定下来了，那个又有问题了。

HQL：这次同学提了啥？

LYN：这次又觉得那个自己找老师没有什么合法性，就还应该让学院安排。就你没法说，因为就是众口难调，所以是可以接受大家的反馈，但是现在我觉得不要每一个都去给解决。其实有时候你不觉得就是很多人毕不了业或者是怎么样跟自己有关系的，他不能把所有的问题都赖在外在。我有这样的问题，我有那样的问题，那你为什么非得要通过论坛来获取那个反馈，你可以去找他呀。我刚才想吐槽就是学院不要那么惯着，大家就是有一些可以解决，不要所有的都去给解决。

我师兄也说，现在学院对大家的培养确实挺费心的，就是各方面都给大家弄的挺好，以前没有这么夸张，现在还挺在意大家心理问题，没事让你们吐吐槽。我导师还让我去组织让大家吐吐槽，我老师虽然他不管研究生，但是他会经常问大家怎么怎么样。

LMN：谢谢，我们暂时没啥问题了。

# 受访者33

吕梦娜：我先给你简要介绍一下我们这个研究哈，主要关心的是商学院以及管理学在中国的发展情况，然后呢，在这个大的课题之下，我们就是可能会研究一些细分的东西，像我主要是研究身份这一块的

B05同学：嗯嗯

吕梦娜：就大概聊一下，比较随意。

B05同学：没事你就问我吧，有啥问我就好了

吕梦娜：行行。你是之前在那个英国读了读了硕士？

B05同学：对，嗯

吕梦娜：是几年的

B05同学：一年的

吕梦娜：哦，就一年的项目，那后来为什么想要回来读博士。

B05同学：嗯，我是之前从来没有想过我要读博士做研究

吕梦娜：嗯，

B05同学：本科的时候一直到研究生的前半年吧都没有想过这件事情。然后是后半年才去想说要不要读博，因为那个时候我一直想的是毕业之后就去工作的

吕梦娜：嗯

B05同学：而且我一直在读大学的时候，包括研究生的时候吧，都是比较怎么说，就是比较功利的那种心态吧，就是我做什么事情一定要准备的很好啊，然后跟这个最后的结果有关系的我就去做，然后我觉得没有关系，我就不去做。但是后面我就觉得这样我的心就是......反正到了硕士的时候，我心态就变了，就变得很佛系了。

吕梦娜：哈哈

B05同学：然后就可能是国外这个文化的原因吧，又是自己的年龄心态有点让我觉得很多时候如果把宝都压在一个事情身上，你没有实现这个东西是特别难接受的，我就想要么就佛系一点，而且到大四的时候身体也总是会出毛病啊什么的，觉得没有必要把自己搞得那么焦虑那么紧张，然后就突然变得佛系了起来

吕梦娜：嗯嗯

B05同学：然后等到硕士的时候就突然要想自己要做什么，就是真的你要开始找工作的时候，我去搜寻一下我相对应的专业就人力资源管理，在国内我们能找到的工作就想了一下。然后企业无非就是去里面做HR，然后去做咨询，然后还有什么，反正就是这几种嘛，我都觉得好像并不是十分的喜欢，而且我可能也用不上我所学的东西。

吕梦娜：嗯

B05同学：我就想那要不要再继续再在这个里面深耕一下，然后能发挥自己所学的东西，而且我可能从本科到研究生都一直是会读文献的，就是我们写东西也是用英文在写，我就觉得起码在这个方面，我不是非常的弱，那我可能能够做下来

吕梦娜：嗯

B05同学：对，然后就决定可能回国之后要读博士，然后真正让我决定的是我回国之后做了一段时间的科研助理。

吕梦娜：啊

B05同学：就是我是九月底回国嘛，然后就开始准备，当时就是想考博。

吕梦娜：是19年吗？

B05同学：对，19年，19年的九月份我就想考博，然后那个时候就是统招也不用准备什么别的，就是只准备笔试那个阶段

吕梦娜：嗯嗯

B05同学：我就想我就对于国内的这个研究一点都不明白是怎么回事，然后也不知道他们愿意要什么样的学生，就是对于这点，这个圈子相当于有一个墙一样，根本就没挨上这个边。我就想，那我先靠拢这方面吧。就我也不着急必须这一年都得考上，我考个几年也行。反正你最终都是要作为一个学者，都是要学会一定的技能啊，对吧

吕梦娜：嗯嗯

B05同学：然后你有这个专业的态度啊，那这样的话我可能需要好几年的时间去探索、去构建自己，那我也不着急，非得就在那个时候要考上博士。然后我就去做科研助理了，然后我也是想从这段时间看一下自己到底喜不喜欢这个东西，能不能做

吕梦娜：嗯嗯

B05同学：然后做的过程当中，就是我就冥冥之中的......那个时候那个项目特别着急的要人啊，然后我肯定是俩眼一抹黑啥都不会，但她又特别着急就把我抛过去了，就是我投完简历第二天好像就面试，然后晚上九十点钟电话面试面完之后，她就说你第二天能来吗？我说能，我去

吕梦娜：这个科研助理就是现在心理所这个吗？

B05同学：对，就是这个老师，然后现在跟我合作的师姐也是当时带我的师姐

吕梦娜：哦（比较惊讶）

B05同学：然后她也很神奇，她是一个读博特......她40多岁了，然后她之前呢是在企业里面做HR，做高管的，然后后来呢，读了一个心理所在职研，然后读完在职研她就要思考去干什么的时候也是非常巧合的，就读了心理学的博士。然后因为她做项目这方面比较能挑起大梁来，所以很多这边的项目都是她来做，然后她一个人肯定做不了啊，然后还有其她同事都在忙自己的事情，所以她当时就招了一个科研助理

吕梦娜：嗯

B05同学：其实她在科研上面也是两眼一抹黑刚开始，所以我们两个就一起探索这件事情，可能有的地方我比她还强点，有地方她比我强点，反正就是我们俩差不多。但是她在做项目方面，就比如说有点类似于咨询项目吧，她比我要强很多，所以我们两个就逐渐的......，我相当于在项目初期就进入这个项目，然后我们两个逐渐的把这个项目做完，做下来。然后到后来，数据收完了都19年年底了，我要考博了，我要准备考试了。你想想，当时应该是三月份考试，我一月十几号才她才放我假，我开始复习，时间是非常非常紧张的

吕梦娜：嗯

B05同学：然后，当然那个项目我是反正是送佛送到西把它做完了嘛，我才开始准备考试。

吕梦娜：嗯

B05同学：然后那个时候我就笃定说，第一我觉得我是能做这个事的，第二是我真的觉得就做这件事情很有意义，就是因为这个项目也正好做的是安全方面的事情了

吕梦娜：哦（感觉比较新鲜）

B05同学：真的就是你在那几个月当中，我们正在做研究的时候，正在做干预项目的时候就有的装置——就是有的生产线上就有人就这么倒下了，就脑出血啊

吕梦娜：哦

B05同学：我就会觉得做这件事情真的很有意义，而且你每次去收数据或者是跟他们直接去这样交流的时候，他们都会特别感兴趣，特别好奇、特别期待你做的这件事情能给他们带来什么实质性的改变。

吕梦娜：嗯嗯嗯

B05同学：然后我就觉得做这个真的是很有意义的一件事情。比起其它的工作，你比如说你在一个企业，你就在一个岗位上，这个螺丝钉上，你干这件事情，你不一定能够产生这么好的效果，能够获得这么大的成就感。

吕梦娜：嗯嗯对对对

B05同学：所以我就觉得还是挺有意义的

吕梦娜：那你能说一下这个项目大概具体是什么吗。

B05同学：这个项目就是当时给一个石油石化行业做的一个项目，

吕梦娜：啊（get）

B05同学：然后是做的干预实验，当时做了两个干预实验。然后还做了一个很长时间的类似于日记法的一个数据的收集。

吕梦娜：啊、啊，就是主要目的是为了提高这个工人的安全是吗？

B05同学：对对，这个项目其实它本身就是上手的也特别着急。就是这个项目是我们老师接的，然后主要是师姐来做的

吕梦娜：嗯

B05同学：然后从这个项目对接可能是19年年中了，然后年底就要结项，所以说时间非常紧张，当然前期调研、读文献我都没有参加，我来的时候就是马上就要开始做了，马上就开始做干预了，但是我们是类似于干预和读文献和不停的修正是同时在进行的，就根本来不及。你想干预要做八周，然后做完八周之后又要赶在年底之前想收数据。然后你往前倒吧，从12月往前倒就倒到十月了，我来的时候是十月中旬。

吕梦娜：嗯

B05同学：对，时间已经非常紧了

吕梦娜：所以这个研究就是基本上完全是实践导向，问题导向的对吗？

B05同学：对，但是你写文章呢，你要把它拧成理论导向的嘛，对不对，你要有点理论贡献。我们现在就在写这个文章，我写的已经头都秃了哈哈哈哈

我也很幸运，就我跟师姐我们两个人特别合拍。就刚开始认识的时候，她也是个比较大大咧咧的人，然后我们两个就聊了好多事情，发现好多事好多有共同点，我们俩都成了忘年交了现在

吕梦娜：哈哈哈哈

B05同学：然后她最打动我的一点就是她对于研究特别的热忱，这种热爱是真的非常纯粹的热爱。我说实话，可能在其他同学当中，我在任何一个人身上都没有见到过这种热爱，就是每个人或多或少过来读博，都是有一点现实的考量的

但她不是，她真的是百分之百的热爱，就有的时候我也感觉有点惭愧，但是我觉得她也是我的一个榜样，然后她带领我走上这个道路，包括她也是非常好的一个人，她帮我引荐到心理所这个老师，然后后来又推动我......就是因为我跟心理所的老师不熟嘛，之前做项目的时候，有一次那个老师就问我说你以后要干嘛，就真的不怎么认识，我都不敢说话，也更不好意思说让人家帮我怎么怎么样，然后我师姐当时就说她想读博。

吕梦娜：哈哈哈哈

B05同学：然后那个老师就说那我帮你联系联系，我有一个特别好的师弟在央财，就是于老师

吕梦娜：啊对对对

B05同学：那我给你打电话，问问他有没有名额，要有的话你就可以去试一试。就是她真的是带领我走上这条道路，然后让我坚定的继续在这条道路上。包括我们现在也是，从我上完博一的课回来，我们两个一直都在合作这篇文章，然后现在马上要写完了。她的处女作也是我的处女作，然后我们就可以从头到尾的做下来，然后把它投出去，像一个孩子给她送出去一样

吕梦娜：对对对哈哈，那你们说去年年底那个结项，这个项目和研究还是不太一样，对吗？

B05同学：项目是给客户交付呀，对吧，客户说你这个东西做完了，你给我交了一个什么什么报告，要一个调研报告，结束就给他交过去就可以了。但是你做研究，肯定是要写成一篇文章，然后就是有什么理论贡献呀，这个贡献那个贡献才行嘛。

吕梦娜：嗯嗯对，所以现在在写这个文章还是跟这个项目相关的研究是吧？之前上课的时候听你汇报，比如说正念，跟这个研究有关系吗？

B05同学：正念跟这个研究没关系，但是正念是另外一个心理所老师在做的一个东西，是她申的一个课题，是她申的一个基金

吕梦娜：嗯嗯

B05同学：然后我当时就想着我为什么选这个话题呢？第一就是我刚看到这个基金的时候，反正我脑子里面是不知道自己要做什么的，但是我只知道一定要做老师相关的。

吕梦娜：为什么要做老师相关的呢

B05同学：你做老师不相关的，他都没有办法给你提意见。他没读过相关的文献，你读的文献远远超越他的话，你们两个永远在没有办法在文献上面产生共鸣。而且一般老师不会因为你对这个感兴趣就会转变他的研究方向跟着你来做，除了于老师，他没有自己的研究，就是学生做什么他做什么，他这样的实在是太太少了，万里挑一。

吕梦娜：对对

B05同学：但是一般的老师都不会这个样子的

吕梦娜：对对

B05同学：所以我当时就觉得一定要做老师正在做的东西。还有一个就是，基金是要结项的，基金要结项一定要出东西，然后这个话题肯定是要找人来做。

吕梦娜：嗯

B05同学：那目前没有人占上它，那我就先占上，然后以后也对我发文章，肯定是有帮助的嘛，就是我想发，然后导师也想让我发出来，因为她要结项嘛。我肯定是以后起码会比较顺利的，而且我现在越来越发现，其实我关起门来跟你讲，我觉得在同门的同学之间对导师的精力是有个竞争关系的，争夺关系的，对吧？

吕梦娜：对对

B05同学：她有的时候总会在某些人身上花费的心思比较多

吕梦娜：对对是

B05同学：那当然这个原因可能有很多，就为什么她会在你身上花很多的精力，当然如果她觉得你做这个东西对她来讲更重要的话，那她当然更愿意推进你的研究嘛

吕梦娜：对对对，我好像有一次记得你是不是把正念和安全联合到一块去了？

B05同学：哦哦，有点印象

吕梦娜：对对对，因为你一开始做安全的嘛？

B05同学：对

吕梦娜：那现在这个模型或者这个研究有继续推进下去吗。

B05同学：嗯，正在做啊，包括我们那个综合考试我也在做这个。我毕业论文也是想做这个，因为我觉得是可以做下去的。而且我觉得，在我心目当中没有很好做的话题，没有很不好做的话题。当然了那些很明显的，比如说很落伍的，不是落伍的，就是很多年没有人提起的那些topic，然后你去做的话可能会有些挑战，对不对

吕梦娜：嗯

B05同学：现在大家都广泛讨论的话题，没有好做与不好做之分，只有深耕不深耕。你锚定了一个，然后你去深耕，肯定有东西可以做出来的

吕梦娜：嗯嗯

B05同学：所以我觉得还是不要轻易的换题。然后我也确实觉得这个题挺好的，就是我能在这里面有自己的思考，我觉得我能挖掘出来点什么东西，也不知道是基于什么，就是一种无可根据的自信

吕梦娜：了解，那现在你的研究大概就是两块，一个是安全，一个是正念，可以这么说吗？

B05同学：嗯可以，但是还是以安全为主吧。因为正念是在安全环境下的正念啊

吕梦娜：嗯了解，那除此之外还有其她的不？

B05同学：嗯没有了，当然写的这个合作的文章是安全氛围的，还是安全相关的

吕梦娜：嗯了解，然后也没有再自己去挖掘其她的topic，就像刚刚说的要在一个领域扎下来然后去深耕是吗？

B05同学：对，因为我觉得换topic是一件很有挑战性的事情，然后我觉得也可能你换半天，如果没有人帮助你的话，没有导师跟你讨论的话，这个是对自己是很不利的，因为首先你很难在短时间内抓取到这个topic，目前大家都在做什么？可做的还有什么？甚至比如说你想到了一个点子，都没有人能够跟你讨论这个是不是可以做下去的。

吕梦娜：嗯，不是就是你想做什么，于老师她会跟着去做吗？

B05同学：嗯，是会这个样子，但是我目前还是想跟心理所这边更多合作一些

吕梦娜：了解了解。但是这边如果开组会啥的你也会参加对吧

B05同学：对，两边组合我都参加，我其实对李老师就是心理所的老师，还包括我师姐有一种回报、感恩在心里。

吕梦娜：嗯嗯

B05同学：所以我当时刚考上博士的时候，是可以做选择的，就是我可以继续跟着所里去做，然后我也可以完全脱离她们，跟于老师去做，然后于老师当时也跟我提过说如果对那边研究不感兴趣了，随时欢迎你回来。

吕梦娜：嗯嗯

B05同学：然后我当时没有做决定，因为我觉得我就是一片白纸，然后让我做什么都行，我做什么都感兴趣，然后我现在反正也没有想脱离的想法，因为我觉得首先李老师是一个对学生非常非常好的人，她一直都拿我当个亲学生。

吕梦娜：嗯嗯嗯

B05同学：而且我觉得我也有义务去回报她对我的好，那我能通过的方式就只有是发文章带她的名，然后在这边给她干点活啊，然后做点什么东西。

吕梦娜：对，就是感觉你那个项目其实给了你很大的动力去做研究也好，去读博也好，是不是？

B05同学：对，因为以前你想象读博只是一个想象，你包括跟别人聊天也是别人的故事，你永远不知道你身处其中是什么样子的。然后那个就给我一个机会，就是先做一个练习生，然后去看一下你把自己想象成一个博士生，你在这种生活里面是不是适用。当时真的特别苦，就我师姐她其实是一个精力特别充沛的人，她可以早上8点一直工作到晚上十二点，然后她的工作和生活可以完全替换。比如说，我们平时吃饭的时候，我们在外面做项目出差嘛，我们两个就24小时在一起恨不得。然后吃饭的时候比如说有时候聊到这个生活上的话题吧，然后你这句话还没说完呢，她就说，哎，你说我们这个实验是不是怎么怎么着。就是她永远都是没有任何心理脱离你知道嘛

吕梦娜：哈哈哈

B05同学：然后我就觉得哎呀，好累呀，就是超级累。我甚至曾经有一段时间就很抵触，我就觉得还是太累了，但是后来又一想，觉得做什么事情都挺有挑战的。但是这件事情还是更有成就感

吕梦娜：嗯嗯嗯，那现在会有这种感觉吗？就是说苦啊累啊，有点抵触这种

B05同学：有苦有累，但不是抵触了，有的时候这个苦和累就是不知不觉，不是说在这件事情之前，而是完成这件事情之后

吕梦娜：嗯嗯

B05同学：你就比如说我有的时候写文章，或者是改什么东西就改到晚上十一点钟才回去，然后躺在床上就想，啊我也有这一天呀，我怎么也变成一个加班狂了。这是以前我非常不耻的一件事，或者说我没有完全没有想到过的一件事。我上大学的时候，人家给我起个外号叫油子、混子你知道吗。

吕梦娜：哈哈哈哈

B05同学：差不多就得了，差不多60分就好了，然后现在变了。你沉浸在这件事情就想把它变得越来越好了，

吕梦娜：就是在这过程当中，是不觉得苦和累。只是说躺床上那会是会这么感觉是吧

B05同学：对对

吕梦娜：那就当时对这个科研也好，或者读博的一些想法也好，和目前的状态有什么出入没？

B05同学：嗯有啊，第一个就是我当时读之前我知道它很累、很难，都说发文很难、毕业很难，毕业了找工作也很难。但是我不知道会这么难。我读博之前我跟好多人都聊，我就正在读的，她们都劝退我。所有的口径都是你一定要想好了，你真的要想好了，

吕梦娜：嗯嗯

B05同学：我当时就想这有什么可想的，这再难，我自认为我在大学过得也非常的艰难，（读博）还能有这个难？是我太天真了，就是你要做一个真的非常好的研究，不是说外界对你的要求有多难，而是说你要做研究，你要探究事情的真相、探究世界的真相这件事情就很难。

吕梦娜：嗯嗯

B05同学：就是你需要付出很多很多的思维，然后你需要提升各种各样的能力

吕梦娜：嗯

B05同学：这个不是外界对你的要求，这是你想办成这件事情对你的要求。我以前没想象过，因为你没真正做过这件事情嘛。第二个有什么差异，就是没想到这么卷。

吕梦娜：哈哈哈

B05同学：就是我在外国读了一年硕士吧，我改变了一种观念，就是我并不觉得一件事情非得一条道走到黑，你可以中途变换你的目标，就比如说，之前我一直想工作，后来我想读博了，我觉得我开始的并不晚，我甚至觉得我在大学没有接受过科研训练，然后硕士就读了一个那种授课性的硕士，也不是研究性的硕士，我都不觉得是走弯路，但是其实你在国内这种竞争的环境里面，你就觉得自己起步晚了。

吕梦娜：嗯

B05同学：当然了，还有比你更晚的，比如说我师姐，对不对？她毕业都已经40多岁，她肯定不能上高校工作了，但是我是觉得，就是因为国内大家开始的越来越早，很多人像你们这种硕博连读的，对吧？大三就开始跟着做研究。虽然我们都是同一个年级，肯定大家出成果的速度是不一样的。

吕梦娜：嗯，我没有参考性哈哈哈哈

B05同学：就是明显就能感觉到啊，硕博要比博士要手更快，对吧

吕梦娜：嗯，是有这样一个优势，但是你觉得这样就好嘛？其实我比较羡慕你这种，就是知道研究的乐趣或者是兴趣所在之后再决定走这条路会更坚定一些。像我们大三能知道点啥，当时就是也问了几个同学的想法，我们硕博连读的坑，但是就是......

B05同学：确实会有这样的一个内在驱动力的问题，但是这个东西是不能及时表现在成果上的，我只是说功利来讲成果啊，就是比如说我可能大概率会延毕，因为现在还没有文章，对吧

吕梦娜：没事，我跟你们一块哈哈哈

B05同学：然后很多人呢，大家就知道做研究是怎么回事了。包括你往上看那些清华北大的，或者就是那些更top的学生，她们很早就踏上了这个科研的道路，然后她们就是也更优秀，她们掌握的资源也更好。假如我在刚入大学的时候就知道我以后要做科研，我也许就留在国外读博士了呀，对吧，这个就是我现在在考虑的。当然我觉得这个东西也不是非常困扰我，因为我觉得，你找到了自己喜欢的东西，多晚都不算晚

吕梦娜：对对是的，是这样的，就是像刚刚你说这个问题哈，就是很多学生有的可能发的比较多的（就不是咱学校，比如说别的学校那种），她也不觉得有什么乐趣，我觉得这样反而很可惜

B05同学：但是我最近就在想一个问题，当然也是有可能是我自我安慰。就是老师们她们真的喜欢做研究吗？发的很好的人

吕梦娜：不是。

B05同学：对呀对呀，你如果外表上看，我不具体说谁，就是比如说发的比较快的，觉得就是那种年轻有为的，她们开心吗？她们喜欢自己的成果吗？

吕梦娜：并不

B05同学：那么不喜欢，但是也不影响她们工作呀，也不影响她们生活呀。

吕梦娜：嗯

B05同学：所以现在这个驱动力我可能觉得对于我自己来讲是很重要的，因为我如果不喜欢做一件事情的话，我真的会做的很糟糕或者根本不会去做。可能对于某些人来讲就不是很重要

吕梦娜：是，嗯，我可能跟你这边会比较像。我也就是可能对之前的那种研究，比如说实证范式，就是那种大批量那种哈，我可能不太喜欢，所以就很难说服自己去跟着去做这样的研究，就包括有的人，还劝我说你赶紧投，就不要管它写的怎么样，就赶在路上等等，就是可能我自己还说服不了我自己，目前是这样的一个状况。

B05同学：嗯是，对呀，你心里也过不去

吕梦娜：对对

B05同学：然后还没说完，想象的差异就是我以前可能会觉得生活变得比较单调啊，变得比较简单呀，因为你就是做研究，直线思维、逻辑思维嘛。变得比较单纯啊，不是一件非常大的事情。然后后来呢，包括我现在也觉得跟工作的那些人相比呢，我确实比如说人际交往啊、或者是一些人情事故上面，我还停留在学生的那个时候，或者说是硕士的那个时候。硕士毕业的那个时候她们已经在社会里面滚了好几圈，然后都知道这件事情就是这个样子的啊，人跟人之间啊就是这样，就突然多了好多感慨，我就没有。然后呢，我还就是比较单纯。

吕梦娜：嗯嗯

B05同学：这个是我以前没有想象过会差异这么大的，因为我没有想象到过说我停滞不前，或者走的很慢，这件事情我觉得还ok，但是我没有想到过人家已经走的很快了，跟我同龄的人。

吕梦娜：嗯嗯，你是会觉得大家有那种感悟，是因为她们成长的快，所以有那种感悟吧？

B05同学：这就是你接触的环境更复杂了嘛，你遇到的事情更不可预料，更不能掌控，更让你变得更无力感，就是你知道生活上有很多现实的事情，但是我们还在当学生嘛，我们可能体会不到那么多现实的东西

吕梦娜：是，你刚刚提到了学生嘛？你觉得就是你现在被周围的人，比如说这边的老师、或者同学，就是类似于同行吧，更多的是（被）看作学生，还是看做一个研究者researcher？

B05同学：我觉得老师肯定是研究者呀

吕梦娜：啊我是说就是你被别人怎么看待？

B05同学：我被别人，emmm，分情况，我觉得在讨论学术的时候，我是非常被导师期待或者是被抬到一个研究者的位置上的。就可能我跟于老师讨论的比较少，我跟李老师讨论比较多。李老师是希望学生跟她吵架，就是比如说你有什么想法，然后我们就开始吵嘛，她就是这样，她也很ok，就是在这个时候，她说在学术面前没有老师和学生，大家都是一样的。

吕梦娜：嗯嗯

B05同学：因为你读的文献我也读了，有可能学生比她读的还多，对吧？然后她就很尊重你，你有什么想法，然后大家的battle呢也不是说我觉得你就错了，这是对事的一个battle。但是脱离了学术，比如说我们平时一起吃饭的时候啊，生活上啊，又变成学生了。也不是学生吧，就是晚辈吧。她称我们都是这个娃那个娃，是这样，她可能确实也跟我们有年龄差，她应该是75年的。

吕梦娜：嗯啊

B05同学：然后她就是也没孩子，然后她就拿我们，我觉得真的是当自己的孩子，比如说谁有个头疼脑热呀，然后谁最近没什么消息了呀，她就打听她最近怎么样啊。她最近又生病了，哎呀，那她可太累了.......就是这种。她还是很关心我们的，这个时候也不是学生嘛，就是长辈对晚辈的一种......

吕梦娜：嗯嗯，那如果说到这个学术交流的环境当中哈，你觉得除了你跟导师是平等，就类似于是平等的嘛，学术上是平等这种感觉之外，还有什么其它的感觉没有？比如说我是一个很专业的一个人，就是在跟老师探讨一些专业的问题，有这种身份感没有？

B05同学：嗯这种身份感我觉得...也会有这种感觉吧，但是我觉得这种身份感其实主要是在不懂的人里面，你会有这种身份感

吕梦娜：哈哈哈哈

B05同学：就是你知道多深，或者是说你真正觉得你做的这件事情是别人做不到的，是只有一个学者才能做的，我觉得这种感觉是会特别的凸显。

吕梦娜：嗯嗯嗯，了解

B05同学：其实有的时候还有一种会...有一种感觉就是你平时跟别人聊天的时候，你提出了一个对一件事情的看法，然后试图去了解真相，可能这个话题不是你现在的研究领域，但是你就想搞明白，就是一种好奇心

吕梦娜：好奇心，对对

B05同学：然后你带着好奇心去找资料，找证据，然后你就觉得我是一个非常具有思考能力的人

吕梦娜：是的，是的，也就是说，其实你不会局限于你的研究话题进行探索。可能就平常的一句话，你想知道这个事儿到底是怎么回事，你也是会去寻找一个资料。

B05同学：对，我觉得这个我导师给我做了很好的一个榜样，就是之前我就听过这样的一个理论，也不是个理论的，看过一个讲座，就是一个做研究很厉害的人，她说她的主要驱动力就是好奇心，后来我们有一次开组会的时候，然后有一个人汇报了交易成本理论。然后这个理论是一个经济学的理论，大家在场的人都不是很熟悉，然后李老师就说起这个，就是点评comments的时候，她说我为什么知道这个呢？是因为之前看李诞的一本书就是脱口秀——手把手教你说脱口秀，提到了这个理论，就是怎么怎么回事。我觉得很惊讶她怎么还喜欢脱口秀？后来我一想，确实是她有一段时间对脱口秀特别感兴趣，所有脱口秀都看了。

吕梦娜：嗯嗯

B05同学：然后我就觉得，这是一个学者应该有的态度，就是你对一切事情...也不是一切事情，你对有些事情保持好奇心，然后呢你不去容忍自己有这种模糊的认识，然后你抱着一种好奇心去了解它，去探索它，然后你在这个里面获得了很大的满足

吕梦娜：嗯，是

B05同学：然后我就觉得这个是我应该学习的。其实我本身我从小也是这样的一个人，但是只不过小的时候我妈总说我“你老想那些没用的干嘛啊”。包括我平时跟有些朋友聊天，她说你怎么那么多为什么？没有为什么，就是这个样子的，你老问为什么。我说那我脑袋就是不停的在想这些东西啊。然后我可能慢慢的没有人跟我共鸣，我就不去想了，但是这个给我一个启发就是实际上你就是这样的人，你身边就存在这样一个非常好奇的人，而且她就是一个很好的科研的榜样，那你就可以继续向她学习

吕梦娜：对，哎你说这个我很有感触，小时候也是，可能不仅仅是问为什么，可能对一些不合理的现象我也会去质疑说这样不好怎么怎么着，然后我妈就是说你又改变不了环境，你要改变自己的心态等等

B05同学：对对，就是大家都这样。我说为什么大家都这样，你们为什么要挑战大家的社会规范嘛，对吧？你为什么非要说所有这个社会规范，我就不想这样。

吕梦娜：对，然后就导致后面我真的慢慢被打压下去，就没什么好奇心。然后直到跟刘老师做这个研究之后，特别是进行了访谈之后，让我慢慢发现其实探索一些事情——就是为什么会这样，是很有趣的一件事

B05同学：本身就会给你带来很大的满足

吕梦娜：对，所以每次我访谈都特别快乐，就感觉学到了很多新的东西

B05同学：对对对

吕梦娜：嗯，那现在咱们课也上完了，你觉得你对咱们课——之前上一年上的课有啥评价不？

B05同学：觉得太多了，那太多了哈哈哈

吕梦娜：哈哈太多了，那除了数量太多了之外嘞？

B05同学：其它都还挺好的，其实我很支持老师们在一周给你甩好几篇论文，然后你一下都给她读完，然后汇报的时候做一个PPT。就是你在这一周特别难受，哎呀读不完了，然后你把它都串起来，做一个汇报，我觉得我很感谢这样的课。

吕梦娜：嗯嗯

B05同学：虽然大家都嘴上说哎呀这个事太多了，做不完，但是其实我觉得这样才是你系统学习一个东西的方法，我到现在还能够...比如说跟别人讨论，我说哎我知道这样的一个研究，虽然不是这个领域的，但是用了这个方法，或者是她是这么做的，或者她是这么验证的，我给你找出来，我立马就能找出来。

吕梦娜：你这读的还是很认真，我有点惭愧哈哈哈

B05同学：你要汇报你就很有心理压力呀，你就想给她读的很...你就要预备万一老师问我这个问题，不会答不上来怎么办？是吧，所以记得就比较清楚。

吕梦娜：不过我很惭愧的是，当时就是读文献的时候，基本上汇报哪篇就读哪篇，其它就略过去

B05同学：我也是啊。所以我不是说自己多么主动，所以我很感谢老师非常强迫你读所有的文献。你看王老师，还有朱老师吗？我忘了，反正有那么几个老师。还有林老师，她不就要求我们都读了然后都做笔记嘛，对吧？

吕梦娜：对对对

B05同学：所以我印象是比较深的。

吕梦娜：嗯，那大家基本上都在读英文文章嘛，你对这个有啥看法？

B05同学：唉，我觉得这是迫不得已吧

吕梦娜：怎么说是迫不得已？

B05同学：英文或者是说西方研究占据了研究的主流，包括你看很多大家用的比较广泛的理论也好概念也好，都是西方人提出来的，你要强大，你要增加你的话语权，你就要跟他们对话，你不能故步自封啊，对吧？

吕梦娜：嗯嗯

B05同学：你就要跟他们对话，然后呢再去输出，你就是先得了解人家，你才能输出你的观点。

吕梦娜：嗯，问题是这些理论的概念都是从西方的土壤中长出来的，那我们以西方的土壤长出来的东西，跟他们对话会不会不太公平，或者说这样对我们来说是没有什么增益的，因为我们的国情跟人家不一样嘛。

B05同学：嗯，我倒没想过这个问题，但是我虽然很多时候大家都在讨论中西方差异，但是我觉得东西方是没有很大的差异的。因为我在英国也待过，我觉得可能表现形式上有很多差异，但是本质上大家都是差不多的。

吕梦娜：嗯嗯

B05同学：就比如说，人家有宗教，我们没有，对吧。但是宗教是什么？其实宗教我理解就是一个约束人的道德观念嘛，我们也有道德观念呀，所以说你要用这个去说外国人道德高尚我们都不行，那肯定是不对。就是说表现形式不一样。

吕梦娜：嗯嗯，你是这么理解的

B05同学：对，我是觉得人跟人都差不多。包括你说外国人，比如说我们都整关系是吧？外国人他们就不那么在乎，其实也在乎

吕梦娜：对对

B05同学：也在乎，逃不了这个，就只不过可能说是程度的差异，或者是说在中国的某些地方。你看中国的很多地方，比如说去私企，去阿里巴巴，去那里肯定这个搞关系没有那么重要，大家还是看实力。就是那种在厮杀竞争很激烈的环境里面，对不对？可能比如说你在小县城，或者是在事业单位或者是公务员系统里面可能关系就比较重要。因为什么？因为考核不完善呀。

吕梦娜：嗯

B05同学：一旦抓着你干活不行，我天天就抓着你，绩效好不好，然后你上头也是这么考核的，然后你跟我关系再好我也不敢把你安排到特别重要的位置上啊

吕梦娜：嗯嗯

B05同学：就是还是国内的差异也比较大，而且中国的很多意识形态都非常西方化了我觉得，尤其是在很大城市。

吕梦娜：是的，嗯

B05同学：而且我并不主张非得搞对立的样子，就是中国怎么着外国怎么怎么着。外国不是统一的外国，外国也有英美法，也有中东，也有这也有那对吧。

吕梦娜：对

B05同学：就是都不一样

吕梦娜：嗯，现在不是倡导把研究做在祖国大地上，还有讲中国故事之类的，你对这个有啥想法不？

B05同学：那你要真的讲中国故事，你就要发外国文章讲中国故事，你得让外边的人知道呀，你光发在自己的土地上，自己自娱自乐，外国人还是不知道。

吕梦娜：嗯，其实我会觉得她这个号召，可能也是想真正促进中国的...比如说管理的一个繁荣吧，就是她可能也并不是想着要跟西方真正的去对话呀，或者是怎么，当然有这方面的原因啊，可能更主要的是用中国的理论解决中国的问题，会不会这样？

B05同学：嗯，可能有学科差异。比如说做宏观经济啊，做金融啊那种确实国内国外有很大差异，他这个不是文化来的，她可能是国家的体制，还有政府的角色呀什么的，就是系统性的那些差异，你懂我意思吗？

吕梦娜：嗯嗯嗯

B05同学：就是比如说人家国外做这样一个policy，一个政策就是有这样的效果，我们做这样的就有那样的效果。所以，我们要做自己的研究。但是管理人文社科这个东西你除非专门做文化的研究，其它的其实国内外的结论都是很实用的，我认为。

吕梦娜：嗯嗯了解，你刚刚还提到了绩效嘛，就刚刚讲中西方的时候提到这个绩效，其实我们现在是不是也有这种绩效管理的倾向？

B05同学：我不知道，我没有办法站到很高的角度上去评论我们学院现在对于我们博士生的绩效考核要求，可能人家领导有人家自己的考虑，但是作为一个博士生，对于我来讲我可能更得去平衡吃饭和初心的问题。

吕梦娜：嗯嗯

B05同学：就是我要毕业，我要短平快发文章，对吧，我很难避免去做一些功利的事情

吕梦娜：嗯嗯

B05同学：比如说什么好发我做什么，哪个期刊好投我做什么。然后我去研究我们的期刊目录，不在目录里的我不投。

吕梦娜：嗯嗯

B05同学：对吧，包括我现在正在做的这篇文章，我们要投的那篇期刊其实是很有挑战性的。它其实在这个细分领域里面已经是很好的期刊了，而且我又是个二作，就是我说半天都没什么用，就对我毕业来讲。说实话我从开学到现在，我没有做自己的文章，我对自己的毕业一点帮助都没有

吕梦娜：我也是，我就是最近录了一篇文章，也是（和毕业）一点关系都没有

B05同学：但是这个东西是你想做的，是想善始善终把它做完的

吕梦娜：对对对对

B05同学：你有了初心不能吃饭的问题

吕梦娜：对，那你现在是怎么平衡的，balance哈哈？

B05同学：我现在就是首先，我还是保持我佛系的态度

吕梦娜：我觉得不能是佛系，我觉得就是保持初心吧，我不觉得这是佛系

B05同学：嗯，就是在功利角度上讲佛系，就是对于毕满足毕业要求上这个角度来讲比较佛系。我不非得逼迫着自己提前毕业呀，我要按时毕业呀什么的。第二个就是你只要做好你手头上的事情，不要浪费时间就好了，总会有回报的。你总盯着那点发表，说实话把一篇干预的研究从头到尾做好做完的机会真的是可遇不可求。

吕梦娜：嗯嗯对，理解理解

B05同学：第一点就是你很难再复制，再走过一遍，你错过了就错过了。

吕梦娜：是的是的

B05同学：对于发小文章，那些搞吃饭的问题，什么时候都可以做，不是说再不济再不济，不是还有ssci那些末流吗，对吧

吕梦娜：对

B05同学：那就是你真的要只考虑吃饭的问题。

吕梦娜：嗯，嗯，了解。现在其实你也不是特别担心正常毕业这些问题？

B05同学：我也担心，但是我总觉得我磨刀不误砍柴工。就是你又不是忙别的去了，你不是还在写文章吗？对吧。你自己吭哧吭哧半天写一篇自己的，处女作完了投了就，然后你为了吃饭你投一个特别好中的，可能也没有太大的心理成就感，你也得不到什么太多的收获。就还不如踏踏实实的吧，机会来了就把手头的做好。因为我当时刚开始可以做这篇文章的时候，我手里确实也没有什么数据，我也遇见不到收数据的机会，我只能做这个。

其实我对以后找不找的到教职这件事情也放的比较宽心，我不想现在就定死了，你作为一个博士生来讲，衡量你是不是一个合格的博士生就是你的发表吗？发表肯定还是要做好的，至于你以后要不要做教职的话，我真的觉得不是很确定，就是不一定非得做这个东西。我真的觉得这段时间就是对我自己的一个修炼，就对我自己的内心的收获，我自己的成长是更重要的。包括那天于老师在组会上也跟我们讲说，你不一定做高校老师才能做科研，你抱着一种探索的态度去对待人生的所有事情就可以了，这就是你不白读一个博士，你不白接受这些训练。

吕梦娜：是的，嗯，于老师的这个想法也特别好

B05同学：她是安慰我师姐，因为我师姐要毕业了吗？

吕梦娜：对对对，她现在工作找的咋样你知道不？

B05同学：她可能不是太顺利，因为确实今年找工作比较难。

吕梦娜：是，她是准备找教职吗？

B05同学：什么都找

吕梦娜：哦，了解。我其实大概问的就是差不多这些，我感觉还是很开心，就是跟你聊到了不一样的地方。就是因为之前也访谈过几个博士生同学嘛，然后就是你在这让我特别的开心兴奋，就听到了不一样的声音。

B05同学：她们都大概都说啥？

吕梦娜：大概就会比较功利导向的那种论文的那种态度，就是想先达标嘛，然后达标之后可能也没有再想其她的东西，就这样。你是亲口说出了好奇心、探索这些其实是跟科研本质相关的一些东西的。但是好像之前同学没有，都没有提到过，包括跟老师的访谈也有。有些老师可能也就是把它当成一个升级打怪的过程，一年发好几篇文章，但是他确实不见得开心。他甚至自己都亲口会说，我可能做就是垃圾

B05同学：所以我觉得这个样子的话，这个行业我甚至都可以说越来越没有希望

吕梦娜：是的呀，所以我很有激励一点是什么呢？这可能是我对科研的一个最本质的、最初的看法。我觉得就是要敢于去发声，哪怕你这个声音很微弱，但是如果说有更多的人去支持你，你的声音越来越大的话是可以改变的。小的时候，我妈经常会说，哎，你改变不了环境就改变心态。但是我觉得作为学者不应该有这种心态

B05同学：就是因为你在这个位置上，你不做这件事情，就更没有人去做，

吕梦娜：对呀，对呀

B05同学：只有你有这个能力可以去做

吕梦娜：所以觉得这种也是一种...往大了说是学者的使命感、责任感，差不多是这意思

B05同学：嗯嗯，其实是这样子，比如说你作为高校老师，或者你以后一直从事科研工作，你发的文章可以被更多的人看到，那影响力就更大，你的思想的影响力就会更大。然后如果你不做高校老师呢，你带着这种思想只影响周围的人也是可以的呀，我现在就在影响我的家人，比如说影响我的爸爸妈妈

吕梦娜：我也是

B05同学：对，有时候跟他们讨论问题，然后我说这件事情我不想这么做，因为我觉得123或者我觉得这个样子。他们就会觉得要重新思考这件事情，就是觉得这样也是在影响。

吕梦娜：对对，就是一朵云带动另一朵云，一棵树带动另一棵树，大概这种感觉。

B05同学：这个就是你的意义所在吧

吕梦娜：对对对对是的

B05同学：然后还有一点其实我特别想补充的，就是你最后一个问题：想象我毕业了我希望是什么样子。其实我还有一点特别特别的在意就是身体健康。一方面呢我们组是做这个的，做职业健康的。还有一方面呢，可能我确实是最近，我可能对这些信息比较敏感吧，就我朋友啊同龄人啊什么的，年纪轻轻身体就出问题了，可能是体质原因，可能是什么原因，我就觉得没必要，就是你真的太拼了，然后好像生病了就是一场空。就比如说我有一个高中同学，然后她就得癌症了，就刚刚硕士毕业，名校毕业找着工作了，有户口了，癌症的有啥用？

吕梦娜：是，这个病越来越低龄化了

B05同学：然后还有身边一个朋友，也就比我们大不了几岁，也是癌症，就这种事情吧，而且我现在越来越觉得，比如说熬夜，也不是熬夜吧，就睡晚一点，睡少一点，第二天早上起来心脏就是突突突跳特别快。你跟原来上大学的时候不一样了，身体状况不一样了。我眼睁睁看着我师姐在开题准备中期这段时间长了好多白头发，就是我就觉得我希望我在读完博士之后，人家不都说写一个博士论文扒一层皮嘛，我就希望我在扒过皮之后我还能有一个比较好的身体。

吕梦娜：对对对，这个真的很重要很重要。那除了身体健康，你后面还有想期望自己会是什么样子吗？

B05同学：就是能够独立做研究，然后各个能力都有提升嘛。比如说我现在就觉得我到了写文章我语言表达不大行，很痛苦，表达不出自己想写的东西啊。我希望我毕业的时候这些能力比如说逻辑思维这种能力能够提升一些吧，然后坚守自己的初心和好奇心。

吕梦娜：那会有功利性的这个考量吗？比如说有多少成果之类的会有这种想法嘛

B05同学：嗯大概想了想，没想太仔细，我觉得这事就随缘。这是尽人事听天命的，你肯定是有计划写几个是吧，几个一作、几个几作，发呢就看命吧。况且那个人家外国老师说，她说每一篇文章都有适合自己的期刊。然后我再加后半句，只不过这个期刊在我们的期刊目录里排名高不高而已。

吕梦娜：是是是是

B05同学：咱只要写出来了，它是很好的一个研究，你不愁没地方发。

吕梦娜：对对对对，嗯嗯挺好挺好的，谢谢你谢谢你。这就是我访谈的全部拉，跟你聊的特别开心。

# 受访者34

吕梦娜：我们先按照时间线来说吧，就是一九年之前和一九年之后，你当时为什么想要读博士？

B03同学：其实当时没有一个特别严谨的思考，就是觉得硕士一定要读的嘛。

吕梦娜：为啥说是一定要读硕士？

B03同学：首先就从最功利的角度来看，找工作，本科特别是我们专业女生竞争力并不是很大。然后因为我实习过，所以对工作就是进职场这件事情本身也并不是特别感兴趣，或者手到擒来那种感觉，而且本科也没学到什么东西，说实话。

吕梦娜：当时就是人力专业嘛？

B03同学：嗯嗯，后来就想着读硕士嘛，就参加夏令营那些，最后只拿到了两个offer，一个是硕士项目，一个是硕博连读项目。当时想着综合来看，既然都读了（硕士了），肯定就是到底越精越好嘛，不然搞了不上不下的就也挺难受。就想就选了这个硕博连读项目的，就来了。

吕梦娜：那当时会有考虑后面就业或者啥的吗？

B03同学：你是说就是读完博毕业之后怎么就业是吗？

吕梦娜：对，就是当时站在你那个时点上，有考虑以后要做什么工作嘛

B03同学：我那时候首先不喜欢职场，然后呢刚开始对科研也没有太了解，也没有什么太大的兴趣，就是你让我做也行，但是我不是很热衷的那种感觉，所以呢当时是不考虑当老师，就想着进那些科研院所，就比如说研究院这些或者哪个大厂的研究院这种，当时特别流行对吧？就想着这个是比较倾向的一个路径。

吕梦娜：所以那个时候其实对研究或者科研称不上一个特别大的兴趣？

B03同学：不能说兴不兴趣，因为那时候不懂。

吕梦娜：排除法可以这么说嘛。

B03同学：对，可以。

吕梦娜：大四你是不是去实习过？

B03同学：嗯对啊，寒假之前那段时间在实习

吕梦娜：嗯嗯，因为你知道后面可能要五年的时间都在读博，对学校里你有过一些憧憬或者想象？比如说科研什么样子、学习什么样子、生活什么样等等各方面

B03同学：想法、憧憬肯定有，但是那时候因为大四前半年实习，后半年写毕业论文，就也没有特别有空去想这件事儿，其实当时对未来没有规划，就觉得先把毕业论文写完，然后当时也没有想到写完毕业论文以后会怎么样，就是没有一个长远的计划，就是立足当下那种感觉。然后关于憧憬那些，也是到暑假之后，就毕完业之后才开始慢慢的有一些期待，然后当时导师也给了一些工作嘛。然后就想着从那些给的文献或者是那些书入手开始，对科研有一个初步的认识，一个好的论文是什么样子的，或者做文献的这种。当时做一些梳理工作，开始慢慢的有一点感觉了。

吕梦娜：那你刚刚提到暑假的时候，导师当时给了你一些文章什么的，大概是什么呢还记得吗？

B03同学：主题我都忘了，但是给了我Excel，然后里面就是文章、标题、作者什么都有

吕梦娜：书和文献都有？

B03同学：就只有文献。

吕梦娜：中英文都有？

B03同学：中文比较多。那时候因为刚开始嘛，就让我帮忙整一些东西啊。

吕梦娜：那个时候因为你大四其实写过论文，你也算知道一点科研或论文吧，有没有遇到过什么困难，就跟老师沟通或者怎么的。

B03同学：当时最大的困难是英文的文献阅读啊，因为本科时候全是中文，就觉得有点费劲。可能一两天才能看完一篇。

吕梦娜：那个有没有想过不读博了？

B03同学：没有。

吕梦娜：就还是很坚定？

B03同学：对，来都来了啊。而且主要是没有什么退路。

吕梦娜：为啥没有退路？因为当时不是刚毕业嘛，也不能叫没有退路

B03同学：你那意思是去工作嘛。

吕梦娜：啊对呀

B03同学：工作是不会工作的

吕梦娜：哦哦哦了解。那个时候有没有想过，就是后面比如说要做科研要做到什么样的程度、什么成果？

B03同学：那时候完全没有概念啊

吕梦娜：就是一步步走、边走边看那种感觉哈。

B03同学：嗯嗯对

吕梦娜：那现在咱们已经过了两年半了吧。你感觉怎么样？

B03同学：挺好的。就是个“混子”的感觉，怎么说呢，没有什么大的成就，但是至少在这个道上我在慢慢的在学了

吕梦娜：感觉很有收获是吧？

B03同学：嗯，至少不像按刚来说一问三不知。

吕梦娜：对对对，肯定会有进步的嘛。那你这个学期选择去实习了，之前不是说不想去工作，为什么会在这个这个时候实习去？

B03同学：因为机会好呀。党和国家机关的那个实习嘛？

吕梦娜：哦是那样，不是中国人民银行嘛

B03同学：嗯，他是国务院下属的，和发改委应该是平行的

吕梦娜：哦哦，所以它其实还是比较事业单位性质的那种

B03同学：它都是编制的。

吕梦娜：哦，那这种工作你喜欢做吗？如果以后作为工作的话

B03同学：就内容的话可以，因为这个实习它不是那种特别杂活的，是很系统的，比如说让我负责一整个招投标或者一整个什么管理办法怎么撰写，就这种大的工作，就让我负责的或者是让我整个跟进的那种工作，就会很有成就感，然后呢不会有被小事折磨的、那种内耗的感觉。

但是啊，就这个工作这个事情来说真的非常累，就算啥也不干，就算工作内容很轻松，我也会觉得很累。因为时间不是自己的，不像搞学术，就是我可以完全安排我一天的计划，或者这个打时间段内我要干什么

吕梦娜：所以你还是很看重学术的这个自主性的特点的，对吧

B03同学：是的

吕梦娜：那刨除目前这段实习的话，像之前，你一般一天大概会比较有规律的吗？

B03同学：时间方面比较有规律，内容方面就比较机动。

吕梦娜：嗯嗯这个可以理解。那一般情况会怎么过这一天

B03同学：早上八点起床，我会前一天晚上大致规划一下第二天要完成的大概内容。然后就按那个规划走嘛。然后这个东西是该休息休息，该干嘛干嘛，就还是比较有逻辑、有规划的。

吕梦娜：嗯嗯，那你学的内容主要就是跟写作、论文相关的，还是？

B03同学：如果手头有活儿的话，就是以写为主，如果没活儿的话，就是轻轻松松看一些文献，就这种感觉。

吕梦娜：你导师会注重你看书嘛，不是论文。

B03同学：会

吕梦娜：会给你推荐一些书目吗

B03同学：会

吕梦娜：哦~那还挺好的。

B03同学：但是直到这学期才开始硬性让我们看，之前只是纯推荐。

吕梦娜：是要求报告之类的吗？

B03同学：说是这么说，但还没开始。

吕梦娜：哦~现在你对这个转变有没有什么想法？

B03同学：就是看书确实能增加那种底层逻辑、那个地基的。比如说你看一篇论文，你可以把它归到比如说一个流派，或者是怎么说呢，就是这个研究对整个大的人力资源或者OB，就是能给它一个一个的扎进那个缝隙里，而不是说就是散的铺在平面上。但目前也没空看哈哈，因为这学期实习，所以···

吕梦娜：对对对

B03同学：一直想看。

吕梦娜：嗯嗯，那你现在的话会对后面几年有什么想法吗？如果说大四那会儿还没有对研究太多的想法哈，那现在呢？

B03同学：想当老师

吕梦娜：想当老师，很坚定了现在，是吧？

B03同学：嗯

吕梦娜：包括你明年不是也要出国去交流，主要的原因或者是都有哪些考虑？

B03同学：第一个是想跟国外老师，因为我找交换机会的话不是看学校，而是看领域的老师，我看哪个老师发表多，研究的和我的兴趣点比较契合，我就去邮件联系那个老师。所以是想学到和我感兴趣的领域更多、更前沿的一些想法。因为毕竟中西方观念差距很大

吕梦娜：所以还是想在学术上有更进一步的这个发展。

B03同学：然后再表面一点的，就是一年的那个访学经验对以后找工作有帮助，所以这是两个主要方面。

吕梦娜：那你现在就是想要当老师，有没有听说过比如说青椒可能后面会面临一些比较悲惨的一段时间。

B03同学：怎么说呢，我就比较属于那种不管别人怎么样，不管大环境怎么样，我就干呗，这样我想做我就去做，可能有点理想主义。

吕梦娜：我觉得理想主义是好的，作为研究科学者，我觉得还是需要一点···

B03同学：但是会遇到很多问题嘛，确实后面说青椒的一些问题就会遇到

吕梦娜：所以现在还是属于那种边走边看，不会去想夸大这种困难是吧？

B03同学：对。不太喜欢想太多。

吕梦娜：你有目标的、或者意愿的高校嘛

B03同学：没有。论文还啥东西呢

吕梦娜：你大概有设想就是毕业之后能够取得什么样的科研的成果也好，或者是在学术上有什么作为之类的。

B03同学：想发个三、四篇不差的论文。

吕梦娜：至少是a以上的？

B03同学：嗯那。

吕梦娜：你之前提到你的研究嘛，就是暑假的时候老师给你的研究是你目前在做的这个方向吗？

B03同学：不是

吕梦娜：那个大概是什么样的一个研究？

B03同学：我忘了

吕梦娜：那就你现在这个研究方向是不是基本上是从一开始到现在一直没太有变化呢，还是中间有变化？

B03同学：因为我导做雇主品牌的嘛，然后刚开始接触雇主品牌的还比较多

吕梦娜：是你自己选择还是老师给你的？

B03同学：之前是刚好师兄在做那个综述，然后我来的时候就是第一个活吧差不多，就是跟师兄一起做雇主品牌的综述，就有个先行的那种认知，就是从这个扎根下来，然后往旁边去找他的结果变量，这些前因后果等等。

吕梦娜：嗯嗯了解

B03同学：然后再后来，就是我导会有自己的数据，然后我是从里面扒，然后扒出来一些有意思的结果，就从那个再出发，就把那个有意思的点作为我的新的···就比如说职业发展这块，就是新的、感兴趣的点。

吕梦娜：所以我大概理解就是，以雇主品牌为中心，然后去发散，但是不会离开雇主品牌对嘛

B03同学：嗯嗯，我以后也想继续这样做。

吕梦娜：你目前是对它是真的有兴趣吗？

B03同学：嗯挺有意思的，第一个认为他有意思，第二个，它现在发展也刚好就在势头上

吕梦娜：算是属于前沿的一个研究，可以这么说嘛

B03同学： 也不算前沿，但是还有很大空间。

吕梦娜：你觉得有意思的点体现在哪？

B03同学：我觉得它是连接宏观与微观的一个点，你说它微观吧，它其实是组织层面的一种偏战略的东西；然后你说它很宏观吧，它又像是一种组织氛围，它能给员工一些很直接的影响。就感觉看你怎么去argue，还蛮好玩的。

吕梦娜：那你有想到要拓展一些其他的研究方向嘛？就比如说脱离雇主品牌，去研究一些其他的一些项目，就是全新的那种。

B03同学：其实看很多研究，别人做的研究也会有很有意思的点，但我不会想着做那个。

吕梦娜：为啥呢？

B03同学：还是想着立足于自己比较熟悉的那种舒适圈吧。

吕梦娜：理解理解。

B03同学：但是这样不好。

吕梦娜：为什么不好？

B03同学：就是没有打破舒适圈了。

吕梦娜：但是如果说这个像你说的，如果很值得去进一步挖掘的话，好像也不叫舒适圈，因为你也不是说就躺平了，而是在继续的去挖掘

B03同学：但是也不能局限于这个领域。

吕梦娜：那你希望是怎么样？就是不能说你做不做得到，就是你觉得比较合适的应该是怎么样？是多个领域一起去挖掘，还是···？

B03同学：立足一个领域，然后想办法把感兴趣的别的领域再融进来、或者建立一个联系，我觉得这样会比较好。

吕梦娜：那你说的“这样比较好”，主要也是因为···

B03同学：有意义。

吕梦娜：那是哪方面的意义呢？

B03同学：就是又没有忘本，但是又在创新，有一点这种感觉。

吕梦娜：有点理论上的意义算是，能这么讲吗？

B03同学：实践意义还是不够

吕梦娜：那你去做研究的时候，会去追求这个实践意义吗？

B03同学：我觉得目前还没有做到那么深。

吕梦娜：你觉得对你目前来说，实践意义其实要比理论意义要更深一些，是吧？

B03同学：嗯对。

吕梦娜：如果以后随着水平啊、能力的提高，也会追求这一方面嘛

B03同学：肯定呀，还想做那种不只是研究者能看懂的。就比如说我们有一个公众号，现在我们团队一起在写。就虽然现在我才负责一两期这样，但看下面真的会有实践者有一些探讨，就觉得这样还蛮好的，就是你可以给他们一些启发。而不是只是在研究上、理论上有。

吕梦娜：那你觉得就是我们做管理学研究的这个叫合法性也好或者正当性也好，体现在哪里啊？

B03同学：没太没太明白。

吕梦娜：就是我们之所以能够存在，是因为什么？比如说有些人可能会觉得因为实践有需要，所以我们才存在。如果说实践管理都没有需要的话，那可能我们这个学科或者是这个领域都没有必要去存在。你会怎么想？会有考虑过这方面吗？

B03同学：我觉得对我来说有点深。

吕梦娜：可能是这个概念你可能之前没太有接触过哈，那我们换下一个话题也可以。就是你在跟导师合作方面、交流方面还是蛮多的，导师影响也蛮大的，是吧？那你觉得像这种就是基本上是把你一点点给带起来的方式，会对你的自主性有什么影响嘛？因为你比较看重这块嘛

B03同学：其实我们导师特别尊重我们的主观意志，就是你想做什么就做什么，就像我师姐她们就有做薪酬、做柔性，可能并不是我导师之前的研究重点，但是她们感兴趣的，我导就帮她们或者跟着她们一块儿去探索。所以并没有说抑制自主性啊或者是什么问题

吕梦娜：就是也不会说，因为可能有时候帮老师做些工作，然后耽误自己是吧？

B03同学：嗯嗯

吕梦娜：以你目前和导师的相处经历，还有你见到其他同学与导师的相处经历，你会给你和导师的关系打多少分，假如区间是零到十分

B03同学：十分

吕梦娜：满分。

B03同学：嗯等会儿，九分。

吕梦娜：是怕骄傲，还是说确实有什么值得改进的地方？

B03同学：嗯~这一分扣在我，而不是扣在就是和导师交流的这个过程，可能还有一些东西我没有悟到的，或者他提到但我没有理解到

吕梦娜：但你对老师是百分之百的满意的，对吧？

B03同学：嗯。然后前段时间我们还在宿舍讨论学生和导师之间真的好像有缘分在。学生的性格和老师的性格是会融合在一块儿。如果我是跟了王老师那样特别push的老师，我可能会受不了，因为我本人是一个特别自由的那种，就刚好跟我导师契合上了。

然后XX（王老师的学生）呢，现在和王老师就融合地非常好

吕梦娜：所以这其实是一种像是天生不需要磨合，就非常契合那种的

B03同学：这是一种情况了，就是刚好的那种情况。还有一些可能需要双方比较大的磨合。就还比较幸运。

吕梦娜：那像咱们上课，就是所谓的博士课程，你觉得和本科有啥区别吗？你整体怎么评价？

B03同学：首先我看过很多人就是说硕士课程、博士课程没有意义，就是我花钱来，不是让老师听我做汇报的，但我特别特别反对这种观点，因为至少我们上的这种博士课程啊，就是非常有意识地在培养我们的一种批判性

吕梦娜：批判性是怎么体现出来的？

B03同学：比如说一篇文章让我们去做comments，就不是接受到一篇文章，我就认为它是最好的，我认为它完美的。这一点是我本科完全没有的那种品质。

吕梦娜：你觉得现在的comments已经达到了批判的这个点了嘛？

B03同学：可能说不上批判那么大，至少我知道它有哪里做得不好，不是我照单全收，而是辩证地去看它，有这种感觉。

吕梦娜：咱们上学期的时候上了很多seminar的课，我特别记得一个周可能要做三个ppt。我们读的好像又主要是英文的，我记得除了我老师当时布置的全都是中文之外，其他老师全都是布置的英文的论文，有没有觉得会不太好？为什么我们都要读英文的，而不是结合着看呢

B03同学：你说中英文这方面是嘛。

吕梦娜：嗯嗯嗯

B03同学：我的理解啊，可能是因为中文看起来真的太快了

吕梦娜：但是快不是什么问题吧。

B03同学：我的意思是你可以自主的把它作为课外的、补充性的中文阅读材料，而不是说···我知道你的意思，就是虽然中文它是很容易读，但它也会有一些新的思想在里面。

吕梦娜：我先说下我的观点，我是觉得一篇文章的好坏不在于他用什么语言来写，重点是他的思想性如何，甚至不在于它的方法如何，不在于它的文笔如何，我还是比较看重他的思想性。但是我们目前看的这些文章，我会觉得老师们会比较注重方法上面会学到什么东西、有什么值得借鉴的地方。反而呢对这篇文章的思想好像会关注的比较少，这是我个人的一个感觉啊，我不知道你是什么想法

B03同学：我觉得那门课上有很多新的知识点嘛，还没有到说我已经了解的那么深，可以去评判他在思想上的什么

吕梦娜：就只要是能我们能从中能获得一些知识就可以对嘛

B03同学：对，我还没有到评价他思想的这个地步。

吕梦娜：哦哦

B03同学：以我们雇主品牌这个角度来看的话，刚开始的时候，是中文开始，然后再往后全看英文，当英文文章已经不能在方法上或者是基础的这些方面给我更新的那种感受了，我就又回到中文，就看最前沿的，甚至是一些会议论文，或者一些发言稿，看最前沿的那些大牛有什么新的感想、新的观点。

吕梦娜：那除了你自己导师之外，咱们上课的时候也会遇到过很多老师嘛，有没有印象比较深刻的？

B03同学：姜老师，我会特别喜欢姜老师那种态度，就是悠然自得去做学术，可以说是不急、不慢、不愠、不火的那种态度。还有另一些让我印象深刻的老师，就是于老师，他做学术的那种动机是为全人类那种大爱。

吕梦娜：这个怎么体现的？

B03同学：这个最初的印象是夏令营的时候，不是有个一对一的交流环节嘛，那时候我先找完我导师，然后去找于老师，他就慢悠悠在那儿跟我们聊了好多，说做学术应该做那种能造福人类的学术，不是说去发一篇文章啊什么的，虽然我现在达不到这个境界啊，但我觉得以后如果真的走学术这条路的话，最后肯定还要以这个为最终的目标。然后听完他当时说的那些之后，特别感动，我也不知道感动啥，因为那时候对学术就毫无认知，以为就是发论文。我当时听的热泪盈眶。然后夏令营回到酒店之后，我就给他发了个邮件，说不管最后结果怎么样，我真的特别感谢您。我也不知道他记不记得我。就是觉得不再那么狭隘了。

吕梦娜：这是你个人的视角来看待的嘛，就是我可能还会看到，因为现在于老师也是副院长，就有时候很多发言，针对我们博士生也好、硕博连读生也好的发言当中，就可能感受到比较着急，会催着让我们去发一些论文。还有林院长经常push我们去发论文。

B03同学：我觉得这是一个短期的目标，但长期来看不应该把这个做成我奋斗的···就是我的价值不应该只是体现在这几篇论文上。

吕梦娜：那你觉得如果站在顺利毕业那个节点上，你会对你这五年的学习教育比较满意嘛，你觉得你到时候会是一个称职的研究者，或者是管理学者，或者是怎么着，就按照目前的这个趋势来看的话

B03同学：至少是一步一步认真做好我该做的事儿，倒不能说是什么好的研究者、管理者什么的，因为我觉得现在都没有到研究的那个层面上。

吕梦娜：为什么还没到研究的层面上

B03同学：因为感觉还不够格

吕梦娜：那你期望的够格是什么样子？

B03同学：扎在一个点上，扎根完了之后再去要创新，再去开枝散叶那种，而不是像我们现在做的变一个变量、两个变量，改一些方法。虽然这样发文章真的很快，也很好写，因为八股文范式嘛，但是真的就没有创新

吕梦娜：你有没有考虑过就是这个范式会觉得不太舒服，就是我们可能整个套路都是死的，包括王老师经常给我们开讲座，比如说论文怎么写，就很范式化，确实就是听完他讲座之后，会觉得好像研究也就那么一回事儿——就把变量找好，好像就可以了

B03同学：但是那个确实是最符合我们现在的短期目标。

吕梦娜：但是有没有想过这样其实会损害一定的长远目标。当大家只知道做这些事情的时候，可能就是局限到这个地方的时候···

B03同学：会扼杀最初的创新

吕梦娜：对对对，然后我对这些文章哈经常有一种食之无味、弃之可惜的感觉。说它食之无味是因为缺少新的知识上的一个冲击，就是这种东西make sense，就你不做研究，我也知道；说它弃之可惜，是因为每一句写的都很好，至少达到论文的那种标准，很符合学术的语言，很严谨。总结下可能就是精致的平庸吧，甚至经常会怀疑为什么这就可以发表出来。

如果说“道”和“术”吧，感觉我们的很多讲座好像都是关于“术”的一个讲座，教你怎么写论文、怎么发论文，但是对于更深度的这些没有，然后我们现在似乎也没有太多这种讲座的交流，在我看来很多讲座、论坛都是流于形式。给你规定二十分钟答辩、十分钟提问，一结束答辩秘书就告诉你，不能再继续了，然后我们就结束了。就是没有真正的去讨论开，大家就是还是在走形式，我会有这种感觉

B03同学：但我觉得答辩这种事就是在最短期的时间内，因为大家时间都很宝贵，那在短时间内让在场的老师还有同学了解到你到底在做什么。然后如果从你的这个这么简短的介绍中能感受到一些创新点或者感兴趣的点，那就是后面再交流的事情，而不是局限在这个论坛上。这只是一个引子，而不是全部。

我是这样觉得，如果你觉得这个老师提的意见特别好的，就可以私下去跟他再讨论一下这个点呀，没必要说全放在论坛上，而且论坛上那么短的时间，说实话也不能很好的交流。就是很多点在短时间内是想不清楚的。

吕梦娜：可能我的想法有点浪漫主义了，就我会觉得做学术可能大概有一半、百分之四十的时间，是大家坐在一块儿畅所欲言，就是发表任何的观点，然后剩下的时间可能就是各自去补充自己的知识、独立思考，我会这样想。但是现在在我看来，好像是可能百分之九十以上都是自己在搞了。

B03同学：你说这个，让我想到我特别特别反感的一点就是，好多包括论坛啊，还有平时同学或者老师之间交流的时候，就会说“有没有考虑把这个变量换成什么、那在职场上会不会怎么怎么样？”我就特别想说，您去过多少职场、经历过多少这种事情，就很随意的就去探讨它的前因或者后果，可能都没有那么简单，你就简简单单的说这个变量可能更合适。我就觉得，说不好听点，有点纸上谈兵，没有任何价值，可能我们现在模型里说的东西拿到实践中去，人一看就会说怎么可能是这样呢？很明显的是人各有志，每个人之间差异那么大，你就这样一概而论，完全不对呀。

吕梦娜：对对对。

B03同学：哎呦，我经常听到他们在讨论这个的时候就特别难受，这种我听都不想听，可能你（评委老师）自己什么经验都没有，你（评委老师）就在这儿···然后其实我觉得我们现在的研究还是，站在员工的角度考虑的比较少。就是你研究完那么多东西，你会发现到职场上去整个颠覆了。就拿最简单的例子，可能之前我们常说到的观点是，线上沟通很有效率。就举个例子，前段时间我们公司要统计一下选哪个体检机构，因为有很多选择嘛，然后让员工选。其实每个体检机构它提供的内容、服务都有一个Excel的电子表，然后呢这电子表也都发给了员工了，当时我们要去统计每个员工的意向机构嘛，带我的姐姐就说你挨个去问这些员工，看他们意向是哪个。我当时特别不解，我说既然大家都有电子版，为什么不线上统计？

带我的姐姐就跟我，在我们公司不是这样，因为大家手头都有很多很多工作，而且你这个对他们来说其实是个并不重要或者并不紧急的一个工作，你发给他们很快就会忘了呀。你不如一个一个拿过去，虽然耗点人力，但是效率上其实会高很多，你就拿着表压着他们，马上就会给你反馈了。如果线上的话，可能今天、明天他都不一定回复。这跟我之前的认知确实不大一样。

吕梦娜：从我的角度来看，不是考虑效率的问题，我是觉得这种东西其实是尊重大家意愿，往大了说可能算是一种福利。就这种情况下，我们不应该把它当成一种工作，如果是线上发给他的话，大家只会觉得这是一种工作，但是如果说你当面跟他说，他可能会觉得，诶，你好像还蛮尊重我的这项权利的，它是体现这种人性化的管理。所以就是我们会发现，我们现在一直是一种效率至上的管理方式，对于博士生也好，对于老师也好，大家只关心他的kpi，效率崇拜，但这种我就会觉得很多人性的关怀都没有了。

比如咱们林院长给咱们上课也好、讲座也好，一来先劝退，劝退完之后开始打击，打击完了再制造焦虑。

B03同学：可是我还挺喜欢他的这种风格，这个很真实。

吕梦娜：但是你觉得这是一个真实的研究生活吗？就是他说的都是真实的吗？

B03同学：他只是想用这种方式去劝退一些不坚定的、并不适合、抱着功利的目的来做研究的人

吕梦娜：那你从他的话语当中，你觉得他是真的喜爱科研吗？

B03同学：我读不出来他对科研的态度，就只是能读出来，他不希望不合适的学生进入这个领域，我只能读出这种态度。

吕梦娜：我可能会觉得是他本身可能对科研也没有太感兴趣，因为我之前有学妹问过我，她想来中材读博，然后后来我就说，你要来读博这个想法我很开心也很鼓励嘛。但是就是有些事情我会跟她讲清楚，就是尤其是我们熟悉的这个环境，我会跟她一五一十地讲，我说我把事情摆给你，但是很多东西可能你需要自己去判断。就是我觉得稍微热爱一点科研的人不是上来就跟别人说你不要读博、千万不要读博、太无聊了、浪费时间的。我觉得不应该是这样的。

B03同学：我觉得这跟个人有关，我跟你的态度就不一样，如果有学妹、学弟过来问我要不要读博，之前我师弟就遇到这种情况，我当时我不像你一样，怎么说呢？你的观点就是他读博这件事已经确定了，他想读博已经确定了，你只是在说学校的优势和劣势，但是如果是我，我就会在这个点上开始跟他battle，我就会问他，你是真的喜欢科研，还是为了拿个学历、拿个证才读？我会纠结，我会不相信这个点，而不是说你后面会遇到什么。

吕梦娜：你为啥不相信这个点？你觉得大家普遍情况下都不是发自内心的喜欢嘛

B03同学：我不是说对大家有什么观点，我就是希望···其实跟林老师有一点相似，就是及时止损

吕梦娜：那如果你觉得科研真的有趣的话，为什么不说呢？

B03同学：我觉得有趣、别人不一定觉得有趣啊，我很坚定我要这样走，但不代表别人的想法就是成熟的，我会先排除一波，不然会浪费他的时间，也浪费这个资源。

吕梦娜：那你会不会跟他讲一下，就是感兴趣的或者是真正有意义的科研是什么样，让他再去做一些判断。好像如果说只跟他说消极的一面，或者说问他为什么要读博，因为就像我们在过去也都不太了解嘛，会不会给他一个更客观、更全方位的视角，让他自己去选择呢

B03同学：我说你多看几篇文献，看完你再说，你到底对这个文献里说的东西感不感兴趣

吕梦娜：但是文献也不是研究的全部嘛

B03同学：但是你刚开始接触，你没办法接触到更深层次的东西了。你刚说道和术，我觉得我们现在都不算，就是大多数的学校开展什么博士能力提升这种都不叫“术”，那就算是个“器”，就是最硬的东西

吕梦娜：干货嘛

B03同学：干货都是个褒义词了哈哈哈

我觉得比如iacmr的那些讲座算是一个更好的方式吧，相对来说比咱们学校的这种要好。我其实是觉得很有意思啊、好玩儿，并不是我要做这个研究，并不是就为了发论文什么的。

···

B03同学：我觉得我们师门还有一点好处是，就是我们并不会对方法看得那么重。其实我导师给我们修改论文的时候，他那个method那部分基本上不看，或者让别的老师帮忙看，可能自己也不是很擅长，他关注的是理论部分。

吕梦娜：那你来学校这么两年半的时间，有没有和你之前想象的地方不太一样，或者觉得不太容易的地方

B03同学：我觉得比我想象的要好一点。大家都说读博压力很大，但我觉得压力就还还好，就可能个别的时候有一些焦虑的时候，但是总的来说还是比我想象的舒服、比较舒服的状态，当然如果没有这段实习就更好了。

吕梦娜：有没有想过为什么会这样？就是相比一般人会比较焦虑，但自己会过的比较舒服

B03同学：我导师影响比较大。他是最近这几年很佛系，就不会push我，但是我找他，他就知无不言、言无不尽这种状态。但是我不找他，他很少来push我，除非是什么急活儿他过来催一下。就这种让我整个的状态，包括自己研究的这个状态，都很慢悠悠的，虽然可能不能那么高产，还是挺舒服的。

吕梦娜：然后除了这一点呢还有什么？

B03同学：然后在研究方面确实有慢慢的在上道了。

吕梦娜：你这个上道主要是指哪个方面的？

B03同学：比如说一篇文章，我之前看看就是哎一些句子抄下来，现在看这些可能能做一些整理，就是把别的人发的那些empirical的文章和原来那种理论的文章可以一个对比，看他到底是从理论哪方面出发，是哪个视角出发的。就这种感觉

吕梦娜：就相当于前后都互通起来了，是吧？

B03同学：就是会有一个小小的网络，而不是单独的一篇文章。大概就像有一个小的综述的在脑子里，至少在雇主品牌这方面。我觉得文献综述是一个特别好的熟悉的领域的方式。

吕梦娜：那除此之外你还有吗？觉得跟你想要有出入的，无论好的还是不好都行。你刚说两方面都是好的嘛，导师啊、研究呀。

B03同学：其实我对自己要求不高。

吕梦娜：其实也不能说不高吧，因为你前面说希望四五篇论文。你觉得这个也不算高，是吗？

B03同学：这个就是如果一步一步、认认真真走的话，应该不是很高的要求。

吕梦娜：那你后面这些文章有没有初步打算？比如说都是做实证类的

B03同学：实证或者综述，然后像案例的话，其实对案例并没有太感兴趣，之前和师姐一起写过一个案例，然后觉得那个打磨的过程啊、那个语言组织的过程啊对我来说比较难，我可能不擅长这方面，做起来挺痛苦，说实话。而且还觉得可能案例太主观了，我会这样觉得，虽然案例本来就是一个由研究者的意志来决定的一个东西，可以这么说。

吕梦娜：你喜欢客观一些的东西嘛

B03同学：嗯对。

吕梦娜：那你对咱们学院的这个支持，就学术上的支持有啥看法？

B03同学：支持挺多的。但太麻烦了，可能我这个人比较不喜欢折腾，我一想到拿个一万块钱（博士重点选题项目的资助）就得先参加这个东西，然后再那个什么来报销，然后前百分之多少、后百分之多少，就觉得太麻烦了，就不是按实际进度来，就说不清楚那种感觉。

吕梦娜：除了资金方面的支持之外，你觉得还有什么？

B03同学：还有就是科研提升这种讲座还是太浅了，或者其实可以搞成一个就类似于慕课那个。比如说像王老师那种论文怎么写的讲座已经讲了很多遍了，完全可以录一套下来，然后后面你再深入，这种一步一步更深，然后放到什么公开课的课堂上，这样也方便。

吕梦娜：对。其实我觉得现在讲座最大的优势没有发挥出来。就是讨论可能还可以更深入一点，但是现在讲的可能还比较浅。

B03同学：对，现在不是有个office hour那个东西，那东西其实还挺好的，但是我没去过。就是觉得虽然挺好，但是没有把这个开展得很深入。我觉得其实可以每个老师一个办公室，然后就不叫什么office hour，就是在上班、坐班嘛，有问题你就来，不局限哪天office hour，每天都是，你啥时候有问题你就来，不然，比如说这个月集中搞一个下午，然后我可能要整理很多问题我才能过去，不然就老觉得这个效率不高或者浪费了。而不是那种我有一个小小的问题我也敢去问，就老是担心、害怕耽误老师的时间，因为你把他搞成了一个很形式化的东西，就没那么自由。
