# Supplementary material for: Professional, scholar, or knowledge worker? Identity construction of Chinese management researchers amid the research–practice gap
Source: PLoS One. 2024 Aug 29;19(8):e0306833. doi: 10.1371/journal.pone.0306833 (PMC11361602; doi:10.1371/journal.pone.0306833)
Supplement: S5 File — (PDF) [file pone.0306833.s005.pdf]

Approval Number:AC-SB-CUFE-2021-0013

# Business School of Central University of Finance and Economics (BS-CUFE)

# Application Form for Research Ethical Approval

| **Date:** 2021-08-20 | **Version:** 1.0 |
|---|---|

**SECTION 1. APPLICANT DETAILS**

**1.1 APPLICANT**

| | |
|---|---|
| **Applicant's Title (optional):** | Associate Professor |
| **Applicant's Forename:** | Shubo |
| **Applicant's Surname:** | Liu |
| **School or Department:** | Business School, Central University of Finance and Economics |
| **E-mail address:** | liushubo@cufe.edu.cn |
| **Contact telephone number:** | +86 17313092964 |

**Applicant's Status:**

| **STUDENT:** | | **STAFF:** | |
|---|---|---|---|
| **Undergraduate Student** | ☐ | **Professor** | ☐ |
| **Taught Postgraduate Student** | ☐ | **Associate Professor** | ☒ |
| **Postgraduate Research Student** | ☐ | **Assistant Professor** | ☐ |
| | | **Research Fellow** | ☐ |
| **Name of course/qualification:** | | **Teaching Fellow** | ☐ |
| | | **Other** | ☐ |
| | | **Please specify:** | |

**1.2 SUPERVISOR (COMPLETE FOR ALL STUDENT PROJECTS)**

**Supervisor's Title:**
**Supervisor's Forename:**
**Supervisor's Surname:**
**Supervisor's Post:**
**Supervisor's Faculty/School and Department:**
**Supervisor's e-mail address:**
**Supervisor's contact telephone number:**

**1.3 OTHER INVESTIGATORS/COLLABORATORS (INTERNAL & EXTERNAL)**

**Please list all other known collaborators, internal and external to the School of Business, Central University of Finance and Economics, including the name of the company/organisation or Investigator's department/school and their role in the project:**

N/A

**1.4 REFERRALS**



CS 扫描全能王
3亿人都在用的扫描App

| |
|---|
| **Has the Project been referred to the relevant Ethics Review Committee from another REC or delegated process within BS-CUFE?**<br>**Yes** ☐ **No** ☒<br>*If yes, please provide the reason:*<br>**Referred by department as not within the remit for delegated approval** ☐<br>**Other** ☐<br>Please provide details: |

| SECTION 2. PROJECT DETAILS | |
|---|---|
| **2.1 Project Title:** | Institutional Transformations and the Survival Dynamics of Management Scholars in Chinese Business Schools |
| **2.2 Estimated start date:**<br>*Please indicate when you intend to commence research activities that involve human participants / data.* | September 1, 2021 |
| **2.3 Estimated completion date of project:** | December 30, 2023 |
| **2.4 Does the project involve public health or social services:** | **Yes** ☐ **No** ☒ |
| **2.5 Type of Project:**<br>Research ☒<br>Hospital Service Evaluation or Development ☐<br>Clinical Audit ☐<br>Other - please specify: | |
| **2.6 Research Sponsor:**<br>If **not** research in the NHS, please state N/A | N/A |
| **2.7 Funder:**<br>If unfunded, please state N/A | N/A |
| **2.8 IDEATE/Funder reference (if applicable)**<br>If your study is funded, please provide a reference | N/A |
| **2.9 Links with other BS-CUFE applications**<br>Is the project linked to any other BS-CUFE application? **Yes** ☐ **No** ☒<br>If yes, detail:<br>Project title:<br>Chief Investigator:<br>BS-CUFE Reference (if known):<br>Nature of linkage: | |

| SECTION 3: BACKGROUND/LAY SUMMARY |
|---|
| **Please provide a lay summary of the project:**<br>The summary should be brief and easily understood by someone who is not an expert in the area. Definitions and explanation of terms should be provided (avoid technical language).<br>*To include:*<br>• *a description of the proposed study and population to be studied building on review of previous studies/evidence*<br>• *the scientific benefit of the proposed study*<br>**The implementation of managerialism in Chinese higher education has fundamentally reshaped its landscape through specific manifestations across external and internal dimensions. Externally, a strategic pivot from centralized educational planning to market-driven mechanisms has led to** |

reduced public funding, aimed at intensifying inter-university competition, and institutionalized academic autonomy, enhancing decision-making capacities. This shift also introduced stringent accountability measures to elevate efficiency in academic endeavors [1]. Internally, managerialism has spurred a transformation towards corporatization, where universities, once bastions of shared values, now prioritize profit, reflected in the adoption of performance metrics for course, research, and faculty evaluations [2-3]. This new paradigm fosters a culture of audits and accountability, compelling institutions to engage in branding and marketing to improve their image and attract resources, including students [4]. Furthermore, it has led to the capitalization of academic work, turning educational and research outputs into revenue-generating assets [5], and ushered in a shift towards technocratic governance, characterized by the rise of professional managerial roles and a departure from collegial decision-making towards a more centralized bureaucratic model [1,6]. Collectively, these developments highlight managerialism's profound impact on the operations and ethos of Chinese universities, driving them to adapt to societal needs while also stirring debate and dissent.

Academic identity emerges through an intricate negotiation of meanings within the social milieu, drawing on influences from pivotal communities to establish a space for self-definition and elucidation. This process encompasses both the external expectations and regulatory frameworks imposed on educators, alongside their personal commitment to self-identification and introspection. Questions such as "Who am I?" and "How should I act?" are central to this dialogue, reflecting a deep engagement with one's role and purpose within the academic sphere [7]. The introduction of managerialism has significantly altered this landscape, presenting a dual-pronged challenge. On one hand, traditional academic norms face erosion under managerialist policies, leading to a fragmented evolution of academic identities amidst the clash of established and emerging paradigms. On the other hand, managerialism introduces new environments that pose fresh challenges to the notion of academic identity itself. Specifically, reforms have shifted scholars' positioning, moving away from tenure systems towards employment models that commodify academic labor. This shift redefines academics from "unit personnel" to "societal individuals" and from "academic elites" to "knowledge workers" necessitated to validate their worth through measurable performance [8].

These insecurities highlight the profound impact of managerialism on the personal and professional lives of academic professionals, underscoring the need for a critical examination of how academic identities are constructed and challenged in contemporary higher education. The scientific benefit of this research lies in its potential to provide a detailed understanding of the relationship between institutional changes and faculty well-being. These insights could be used to enhance the effectiveness of policy implementations and improve the working conditions for academics in business schools, ultimately fostering a more supportive and productive educational environment.


[1] *Sun, G. (2003). A review of managerialism in Western higher education management. Comparative Education Review, (10), 67-71.*

[2] *Lin, X. and Xue, Y. (2020). The macro logic of university personnel system reform and the micro-action of teachers' academic work: The game between audit culture and academic culture. Journal of East China Normal University (Educational Sciences), 38(4), 40-61.*

[3] *Clark, B.R. (1998). Creating Entrepreneurial University: organizational pathway of transformation. New York, Elsevier.*

[4] *Frandsen, S., Gotsi, M., Johnston, A., et al. (2018). Faculty responses to business school branding: a discursive approach. European Journal of Marketing, 52(1).*

[5] *Robinson, G. (2009). The 41st chair: Defining Careers in the Current Biomedical Research Environment. The Journal of Research Administration, XL (1), 101-130.*

[6] *Zhang, Y. (2012). The plight of Western academic profession under the background of new managerialism. Higher Education Research, 33(4), 105-109.*

[7] *Henkel, M. (2005). Academic identity and autonomy in a changing policy environment. Higher Education, 49, 155-176.*

[8] *Zhang, Y. (2012). The plight of Western academic profession under the background of new managerialism. Higher Education Research, 33(4), 105-109.*


## SECTION 4 RISK AND ETHICAL CONSIDERATIONS CHECKLIST



CS 扫描全能王
3亿人都在用的扫描App

| Complete the checklist ticking **'Yes'** or **'No'** to **all** questions.<br><br>Where you have ticked **'Yes'** to a question below, you will need to specifically address the ethical issues raised by that point and detail what safeguards will be put in place to minimise the potential risks/harm in the relevant section of the application form or in the space provided. | | | |
|---|---|---|---|
| | | **Yes** | **No** |
| **A** | Does the study involve **participants who are particularly vulnerable** or **unable to give informed consent** or **in a dependent position** (e.g. children, your own students, over-researched groups, people with learning difficulties, people with mental health problems, young offenders, people in care facilities, prisoners)?<br>*If yes, please provide details:* | ☐ | ☒ |
| **B** | Will **pregnant individuals** be participants in the study?<br>• *Please note, while you may not purposefully be recruiting pregnant individuals to the study, consider if any special measures would need to be put into place or if it is appropriate for these individuals to take part, e.g. safety risks*<br>• *If you are not excluding pregnant individuals but not asking for this information (e.g. it is not relevant for the study) please tick 'Yes' but state that there are no foreseeable risks for this group, if applicable.*<br>*If yes, please provide details:* | ☐ | ☒ |
| **C** | Will the study involve children **under 5 years** old?<br>*If yes, please provide details:* | ☐ | ☒ |
| **D** | Will participants be taking part in the study without their consent or knowledge at the time, or will **deception** of any sort be involved (e.g. covert observation of people in non-public places)?<br>*If yes, please provide details:* | ☐ | ☒ |
| **E** | Is there a risk that the **highly sensitive nature** of the subject might lead to **disclosures** from the participant concerning their involvement in illegal activities or other activities that represent a threat to themselves or others (e.g. sexual activity, drug use, or professional misconduct)?<br>*If yes, please provide details:* | ☐ | ☒ |
| **F** | Could the study induce **psychological distress or anxiety**, or produce **humiliation**, or **cause harm**, or lead to **negative consequences** beyond the risks encountered in normal life?<br>• *Applicable to studies involving sensitive topics, vulnerable participants as well as studies involving driving experiments, simulators, computational or physiological experiments. For the latter, please detail potential risks associated with any equipment and how these will be monitored and addressed in the space below.*<br>• *Please also consider the risk to individuals if any personally identifiable data collected as part of the study is accidently disclosed. Please see guidance note for more information.*<br>*If yes, please provide details:* | ☐ | ☒ |
| **G** | Does the study involve **substantial physical exertion**?<br>*If yes, please provide details:* | ☐ | ☒ |
| **H** | Does the research involve an **intervention?**<br>• *An "intervention" here is understood as a systematic controlled change of participant conditions, which could be psychological or physical. It can include but is not limited to changes in diet, activity, access to information, or use of certain products.*<br>*If yes, please provide details:* | ☐ | ☒ |
| **I** | Does the study involve the **administration** of any substance?<br>*If yes, please provide details:* | ☐ | ☒ |
| **J** | Does the study involve **physically intrusive procedures**, use of **bodily materials** or **human tissue**, or **DNA/RNA analysis**? | ☐ | ☒ |

扫描全能王
3亿人都在用的扫描App

| | | | |
|---|---|---|---|
| | • *Approval from the University's GMBSC (Genetic Modification and Biosafety Committee) is required before collection or use of any of these materials within the United Kingdom.*<br>• *For studies overseas, please consult the GMBSC to confirm that the required risk assessments are completed.*<br>*If yes, please provide details:* | | |
| **K** | Is any **reward**, including travelling and other expenses, to be given to participants?<br>If yes, please provide details and justification for this, to ensure this is appropriate, and **not** seen as a bribe or to coerce participants into taking part.<br>*If yes, please provide details:* | ☐ | ☒ |
| **L** | Could the proposal give rise to researchers having any **conflicts of interest**?<br>• *Consider relationships/previous personal interactions with participating organisations, participants etc.*<br>*If yes, please provide details including how this will be managed:* | ☐ | ☒ |
| **M** | Will any part of the project be undertaken overseas?<br>*If yes, please state which Country/Countries, the locations at which the project will be undertaken, e.g. public place, school, company, hospital, University, researcher's office, including the services of an overseas cloud hosting provider for storage or a market research company etc. and the local permissions in place for this (where required):* | ☐ | ☒ |
| **N** | Will the researchers go to any areas where their **safety may be compromised**?<br>*If yes, please provide details, including what measures will be put in place to minimise risks and ensure the researcher's safety. A risk assessment should be submitted with the application:* | ☐ | ☒ |
| **O** | Is the research commissioned by the military?*<br>*If yes, please provide details:* | ☐ | ☒ |
| **P** | Does the research concern **terrorist or extreme groups?***<br>*If yes, please provide details:* Click here to enter text. | ☐ | ☒ |
| **Q** | Is your research funded by or are you collaborating with a military organisation?<br>*Military Organisations means organizations, departments, or individuals authorized by a Governmental Entity to defend or engage in combat for a country or who otherwise engage in activities of a military nature or function.*<br>*If yes, please provide details:* | ☐ | ☒ |
| **R** | Are you transferring (physically, electronically or verbally) any technologies, material, equipment or know-how listed in the categories below, to any non-BS-CUFE organisation?<br>Categories:<br>0- Nuclear materials, facilities and equipment<br>1- Special materials and related equipment<br>2- Materials processing<br>3- Electronics<br>4- Computers<br>5- Telecommunications and "information security"<br>6- Sensors and lasers<br>7- Navigation and avionics<br>8- Marine<br>9- Aerospace and Propulsion<br>*If yes, please provide details:* | ☐ | ☒ |
| **S** | Does the technology, material, equipment or know-how have the potential to support the design, development, production, stockpiling or use of nuclear, chemical or biological weapons? | ☐ | ☒ |



扫描全能王
3亿人都在用的扫描App

| | | | |
|---|---|---|---|
| | *If yes, please provide details:* | | |
| **T** | Do you have any concerns that the end user of this research could use the technology, material, equipment or know-how to support the design, development, production, stockpiling or use of nuclear, chemical or biological weapons?<br>*The end user can be one or all of the following: The funder of research; Partners and/or collaborators in the research project and organisations that these partners/collaborators engage with, whether or not these are directly involved in the project; Organisations that you are sharing data/materials/know-how with, whether or not these organisations are directly involved in the production of the research.*<br>*If yes, please provide details:* | ☐ | ☒ |
| **U** | Does the study involve any additional ethical considerations or risks to participants or the researcher that are not listed above?<br>*If yes, please provide details:* | ☐ | ☒ |

## SECTION 5: STUDY DESIGN, METHODOLOGY & ANALYSIS

**5.1 Clearly state the research aim(s) and objectives of the project:**

*To include:*

- *a clear explanation and justification for the research question(s)/aim(s)*
- *Objectives are intermediate steps that will help you to meet your research aim(s)*

Research Aim:

**The primary aim of this research is to analyze how institutional changes within Chinese business schools have impacted the career trajectories and work environments of management scholars. This study seeks to uncover the broader effects of policy and administrative shifts on academic staff, providing a comprehensive overview of their current professional challenges and opportunities.**

Research Objectives:

1. **To document and analyze the specific institutional changes that have occurred in Chinese business schools over the last two decades, focusing on changes in governance, policy, and academic culture.**
2. **To evaluate the impact of these changes on management scholars, particularly in terms of career progression, job security, and work-life balance. This will involve gathering quantitative and qualitative data through surveys and interviews.**
3. **To compare the experiences of management scholars across different types of business schools (e.g., public vs. private, large vs. small) to identify any variations in impact related to institutional context.**
4. **To develop recommendations for policy and practice that could mitigate negative impacts on management scholars and enhance their professional growth and satisfaction.**

**5.2 Study design and data collection methods:**

*To include:*

- *a clear description of the study design and data collection methods*
- *a suitable design should reflect the aim(s) of the study*

Study Design:

**The research is structured as a qualitative semi-structured interview study, aiming to deeply explore the career identity construction of Chinese management researchers. This design is specifically chosen to accommodate detailed, nuanced insights into personal experiences and perceptions, which are critical for understanding how academic identities are shaped in the context of evolving institutional norms and the research-practice gap prevalent in Chinese management studies.**

Data Collection Methods:

CS 扫描全能王
3亿人都在用的扫描App

Data will be collected through semi-structured interviews, which allows for both the exploration of predefined themes and the flexibility for respondents to introduce and elaborate on topics they deem significant. The interview protocol will begin with questions about the respondents' academic backgrounds and their general impressions of management studies, progressing to more specific discussions about the inspirations and challenges associated with their research careers.

Interviewees will be selected using purposive sampling to ensure a diverse representation across various career stages—ranging from full, assistant, and associate professors to PhD candidates—and multiple management disciplines, such as strategy, marketing, OBHRM, operations, finance, and accounting. This method will ensure that the sample adequately reflects the broad spectrum of experiences and perspectives within the academic community of interest.

Additionally, interviewees will be recruited through established social networks and snowball sampling techniques, enhancing the richness of the data by leveraging existing academic connections. Data collection will continue until saturation is reached, meaning no new relevant information is observed in the responses, ensuring comprehensive coverage of the topic.

### 5.3 Data Analysis

*To include:*

- *Detail the analysis methods that will be undertaken e.g. content analysis, framework analysis, interpretative phenomenological analysis etc. and any statistical analyses.*
- *Describe how any data will be transcribed, coded, de-identified, stored, transferred, accessed, archived*

Analysis Methods:

The data collected from semi-structured interviews will be analyzed using the grounded theory approach. This qualitative method involves several detailed steps to develop a robust theoretical framework grounded in the empirical data collected.

1. Open Coding: Initially, the raw interview text will be examined line by line. This stage involves generating initial codes that are descriptive and adhere closely to the language used by the interviewees. This process aims to uncover underlying meanings and patterns without imposing preconceived notions or theories.
2. Axial Coding: The next step involves making connections between the codes identified during the open coding phase. This stage includes several iterations between analyzing the data and comparing it to emerging theories. The axial coding will help to identify the main categories and subcategories, establishing a more structured analysis that aligns the data with potential theoretical frameworks.
3. Selective Coding: The final step involves integrating and refining the codes into coherent themes. During selective coding, the analysis will focus on consolidating the data around core concepts that emerge as central to the developed framework, ensuring that these themes effectively represent the collective data and address the research aims.

Data Handling Protocols:

1. Transcription: All interviews will be transcribed verbatim to ensure accuracy in the representation of participants' responses. Special attention will be paid to maintaining the integrity and nuances of the verbal data.
2. Coding: The transcribed data will be coded using qualitative data analysis software Nvivo 12, which facilitates the organization and retrieval of coded data for further analysis.
3. De-identification: All data will be de-identified before analysis to protect the confidentiality of the participants. Identifiable information will be replaced with pseudonyms or numerical identifiers.
4. Storage and Transfer: De-identified data will be stored on secure, encrypted servers accessible only to the research team.



扫描全能王
3亿人都在用的扫描App

5. **Access and Archiving: Access to the data will be restricted to authorized members of the research team.**

## SECTION 6: RECRUITMENT

**6.1 State the total number of planned participants and the sampling strategy; provide justification for this:**

*To include:*

- *The rationale behind the proposed size of the sample*
- *Will the sample size provide enough data to answer the research question?*
- *If sampling will be continued until saturation is reached, this should be stated and linked to the research question*
- *Sampling strategy- is this random, snowball, purposive, convenience etc.*
- *What is the rationale for this- it should reflect the methodological framework for the study*

**The study will include 30-40 management scholars from research-oriented business schools in China, determined to be sufficient for reaching data saturation and capturing a broad range of experiences across different career stages and disciplines.**

**We will employ purposive and snowball sampling to select participants who can provide relevant insights aligned with the study's aims. Purposive sampling targets scholars at various career stages, ensuring the relevance and depth of data. Snowball sampling will be used to extend our reach within this specific academic community.**

**The chosen sample size is ideal for qualitative analysis, allowing for comprehensive coverage while maintaining manageability for in-depth analysis. Purposive and snowball sampling are particularly suited to the study's qualitative, exploratory nature and align with our grounded theory approach. Sampling will continue until theoretical saturation is achieved, ensuring the data collection is sufficiently comprehensive to explore the research questions effectively.**

**6.2 Where applicable, state the breakdown of participants by type and number of each type of participant, e.g. children (include age), parents, teachers, health care professionals etc.:**

| Type of Participant: | Number: |
|---|---|
| Full Professor | 10 |
| Associate Professors | 10 |
| Assistant Professors | 10 |
| PhD Candidates | 10 |

**6.3 Please provide clear inclusion criteria:**

1. **Participants must be currently affiliated with a research-oriented business school in China.**
2. **Participants must hold one of the following positions: Full Professor, Associate Professor, Assistant Professor, or be enrolled as a PhD candidate.**
3. **Participants must have at least one year of experience in their current role at the time of the study.**

**6.4 Please provide clear exclusion criteria:**

**Scholars not affiliated with research-oriented business schools (or management schools).**

**6.5 Please detail how participants will be recruited to the study:**

*To include:*

- *How participants will be identified/screened and approached; by whom?*
- *Where participants will be recruited from and when?*
- *Detail the source of any personal information that may be used to identify participants. If this information will be accessed by someone outside the team who would have access to this information as part of their day to day role, the reason for this should be explained, and permissions detailed e.g. healthcare, student records etc.*
- *Will any vulnerable groups be recruited?*
- *What materials will be used to recruit participants- please provide copies of posters, leaflets, invitation emails, etc.*
- *Where will the above materials be advertised: list and provide details of locations, websites, social media etc.*
- *Will any recruitment tools be used e.g. SONA- please specify and provide details.*

扫描全能王
3亿人都在用的扫描App

**Identification and Approach:**
**Participants will be identified and screened based on their role and affiliation with research-oriented business schools in China. The recruitment will be conducted by the research team members who have existing connections within these institutions. Potential participants will be approached via email, which will include an invitation to participate and an information sheet about the study. Initial contact will be made by a lead researcher or designated team member familiar with the academic community.**

**Recruitment Sources and Timing:**
**Participants will be recruited from top research-oriented business schools in major Chinese cities. Recruitment will begin shortly after receiving ethical approval and is expected to last for approximately 18 months.**

**Source of Personal Information:**
**Personal information necessary for recruitment, such as contact details and professional affiliations, will be accessed through public academic profiles and professional networks. Access to this information will be managed strictly by the research team to ensure confidentiality and compliance with data protection regulations.**

**Vulnerable Groups:**
**The study does not target vulnerable groups. All participants are professionals in the field of management education. Recruitment Materials: The recruitment will utilize personalized invitation emails, tailored to each potential participant. These emails will outline the study's purpose, what participation involves, and the confidentiality measures in place.**

**Advertisement of Recruitment Materials:**
**Recruitment materials will be distributed mainly via email and academic networks. Additionally, calls for participation may be posted on relevant academic social media groups and online forums associated with the business schools from which we are recruiting.**

**Recruitment Tools:**
**No specialized recruitment tools like SONA systems will be used. Recruitment will rely primarily on direct contact methods and professional networks.**

## SECTION 7: INFORMED CONSENT

**7.1 Please detail the process for obtaining informed consent.**

*Informed consent **must** be obtained prior to the participant undergoing any research activities that are specifically for the purposes of the study. This should involve discussion with potential participants or their legally acceptable representative; the presentation of written materials e.g. participant information leaflet(s) –PIL(s) and consent form, and the opportunity to ask questions.*

*To include:*

- *How and when informed consent will be obtained- written, verbal etc. provide details and justification. Justification must also be provided if informed consent will **not** be sought or if consent will be assumed (please note this needs to be appropriate to the study type).*
- *Who will be taking consent? What training has been undertaken for this?*
- *When and how potential participants will be issued with the information leaflet, in what format and how long they will be given to consider taking part?*
- *Does the study involve children- if so, will consent be obtained from parents, if not provide clear justification why not.*
- *Are the informed consent materials appropriate for the target audience- consider age / language / literacy levels / cultures etc.*

**Method and Timing of Consent:**

**Informed consent will be obtained in writing to ensure that participants fully understand their involvement and the scope of the study. Participants will be provided with a Participant Information Leaflet (PIL) and a consent form during the initial meeting or via email prior to any research**



扫描全能王
3亿人都在用的扫描App

activities. Written consent is chosen due to its ability to provide a clear, documented agreement, which is crucial for the ethical standards of this research.

Consent Personnel and Training:
The research team members, trained in ethical research practices, will be responsible for taking consent. Training includes workshops on research ethics, informed consent procedures, and data protection laws to ensure compliance and understanding of participants' rights.

Distribution and Consideration Time for PIL:
The Participant Information Leaflet will be issued to potential participants electronically along with the invitation email. A hard copy will also be made available during the first contact if in-person meetings occur. Participants will be given a minimum of one week to consider the information and decide on their participation. This will ensure they have ample time to review the details, discuss with peers or advisors, and pose any questions they may have.

Children as Participants:
The study does not involve children; all participants are adult management scholars. Hence, consent from parents or guardians is not applicable.

Appropriateness of Consent Materials:
The informed consent materials, including the PIL and consent form, are designed to be clear and understandable, avoiding technical jargon. They will be provided in both English and Mandarin to accommodate the primary languages of the target audience. The materials consider the academic background of participants, ensuring that they are appropriate for individuals with a high level of education and familiarity with research processes.

**7.2 Please detail how participants withdraw from the study if they have requested to do so.**

*To include:*

- *The process by which an individual can withdraw their participation from the study without giving a reason or experiencing any detrimental effects e.g. should they not wish to continue with their participation in an interview or focus group.*
- *Consideration should be given for any data already collected up until this point- whether it is possible for this to be removed. E.g. it may not be possible to identify data once submitted for an anonymous survey.*

Procedure for Withdrawing:
Participants can withdraw their participation from the study at any time, without needing to provide a reason and without experiencing any negative effects. To withdraw, participants can simply inform the research team via email or telephone, using the contact details provided in the Participant Information Leaflet (PIL). If a participant decides to withdraw during an interview, they may do so by verbally expressing their desire to stop, and the session will be ended immediately by the researcher.
Handling of Collected Data:
In cases where a participant withdraws from the study, consideration will be given to any data already collected:
If the withdrawal occurs before data analysis, the participant's data will be completely removed from the dataset upon their request.
For data that has already been included in the analysis, we would be unable to retract your data because it has already been anonymized. This limitation will be clearly stated in the Participant Information Leaflet to ensure participants are aware before consenting to the study.

**7.3 Please also describe how participants can withdraw their data from the study after participation (if possible).**

*To include:*

- *Consideration should be given to when data will be anonymised, analysed, published etc. make sure it is possible/feasible for data to be withdrawn if this is being offered to participants. It may be appropriate to provide a time frame for withdrawal.*

Participants are entitled to withdraw their data from the study at any time. Those wishing to withdraw their data should contact the study coordinator using the provided contact details on the participant information sheet, and submit their request along with their participant ID. But it is important to note

扫描全能王
3亿人都在用的扫描App

**that participants can not withdraw from the interview after data analysis, because at that time, their data has already been anonymized. This limitation will be clearly stated in the Participant Information Leaflet to ensure participants are aware before consenting to the study.**

## SECTION 8: DATA COLLECTION, USE & STORAGE

**8.1 Does the project involve the collection, analysis, or storage of personally identifiable data and/or personally identifiable special category data or criminal offence data?**

**Yes ☐ No ☒**

*'Personal data' is any information relating to an **identified** or identifiable natural person- a 'data subject'.*

*An identifiable natural person is one who can be identified, **directly or indirectly**, in particular by reference to an identifier (such as a name, an identification number, location data, financial data, opinion, an online identifier), or to one or more factors specific to the **physical, physiological, genetic, mental, socio- economic, cultural, race, religion, trade union membership, political beliefs, medical, gender or social identity** of that natural person.*

*Special category data includes personal data which is by its nature, particularly sensitive in relation to fundamental rights and freedoms of individuals such as: racial or ethnic origin, political opinions, religious or philosophical beliefs, trade union membership, genetic data, biometric data (for the purpose of identifying a natural person), data concerning health or data concerning a natural person's sex life or sexual orientation. This type of data merits specific protection as the context of its processing. Failure to handle this data correctly could result in significant risks to the fundamental rights and freedoms of the individuals.*

**If yes, please provide details of what will be collected and for what purpose:**

**8.2 Does the project involve the collection or analysis of personal data relating to children under 13 or vulnerable groups?**

**Yes ☐ No ☒**

**If yes, please provide details of what will be collected and for what purpose:**

**8.3 Who will have access to the study data?**

*Include individuals internal and external to the University and what level of access they have to the data e.g. anonymised, pseudonymised, identifiable etc.*

*Please note you will need to hold a University approved data sharing/processing agreement with each third party (external to the University) with whom data is to be shared.*

**Access to the study data will be restricted solely to the primary investigator(s) involved in the research. The data may include identifiable information, which will be accessed only by the authors for analysis and preparation of the manuscript. For purposes such as peer review upon manuscript submission, only pseudonymized data will be provided, ensuring that reviewers cannot trace data back to individual participants. This approach adheres to strict data privacy standards and limits data exposure to essential academic processes.**

**8.4 During the project, will data be hosted on any external platforms or use new technology other than University approved software?**

**Yes ☐ No ☒**

**If yes, please provide details of the system(s) and how they operate:**

**8.5 Will any research activities be audio or video recorded?**

**Yes ☒ No ☐**



CS 扫描全能王
3亿人都在用的扫描App

| |
|---|
| *This needs to be clear in the participant information leaflet and consent form.*<br>**If yes, please provide details of what will be recorded, how long it will be kept, how it will be stored securely and how it will be deleted:**<br>**Entire Interviews will be recorded by using professional recording pen. The recorded interview will be automatically transcribed into text. Subsequently, we will meticulously review and check the text, then send it to you for verification. Recording texts will be securely stored on encrypted university servers with restricted access to designated research team members. Audio files will be retained until the accuracy of transcriptions is verified and will be held for an additional six months thereafter to address any queries. Following this period, the recordings will be permanently deleted from all storage mediums to ensure the protection of participant privacy and data security. Regular backups will be conducted during the retention period to prevent data loss.** |
| **8.6 Please detail how, where, in what format and for how long the research data will be stored securely, including on back up storage. Please include how and when will the data be deleted and who by?**<br>*e.g. hard/electronic copies, locked filing cabinets in researcher's office, encrypted files, password protected devices. Please also consider consent forms here. These should be stored separately to research data. Where possible, it is often preferable to digitise hard copies of data (e.g. consent forms) as quickly as possible and store securely on the University servers rather than in locked offices.*<br>*The University's data retention policy for staff is that anonymised research data should be reviewed after 10 years to see if it should then be retained or deleted. Identifiable data such as audio/video recordings should be deleted as soon as they are no longer needed (e.g. after transcripts have been made).*<br>**Research data in this study will be stored in both electronic and hard copy formats. Electronic data, including audio recordings and digitized documents, will be stored on encrypted, password-protected university servers. Access to these servers is strictly controlled, ensuring that only authorized members of the research team can retrieve or alter the data. Backups will be performed regularly to ensure data integrity and to prevent loss due to system failures.**<br><br>**Hard copies, such as consent forms and interview notes, will be stored in locked filing cabinets located in the principal researcher's office. These physical documents will be digitized as soon as possible to reduce reliance on physical storage and to enhance data security. Once digitized, hard copies will be securely shredded.**<br><br>**The retention period for all data will adhere to university policies and specific research guidelines. Identifiable data will be kept only until it has been transcribed and verified, after which it will be pseudonymized or securely deleted. pseudonymized data will be reviewed after ten years to decide whether it should be retained further or deleted, as per the university's data retention policy.**<br><br>**Data deletion will be conducted by designated members of the research team. Electronic data will be permanently erased using secure deletion methods that ensure the data cannot be recovered. This task will be logged and monitored by the university's IT department to ensure compliance with data protection regulations. Hard copies will be destroyed using cross-cut shredders to prevent reconstruction and ensure complete data destruction.** |
| **8.7 Will data be shared with any organisation external to the University for processing?**<br>**Yes ☐ No ☒**<br>*e.g. external transcription services, external statistics support, archiving etc.*<br>**If yes, please provide details of the sharing arrangements: clarify whether the data shared will be identifiable, the external organisation to which it will be sent and what contracts/arrangements are in place to safeguard the data and ensure the data processors/controllers will comply with data protection requirements:** |
| **8.8 For this project, will data be processed, (to include the collation, collecting, distributing, sharing, accessing, reviewing, amending, deletion) transferred or stored in any Countries outside China?**<br>**Yes ☐ No ☒**<br>*e.g. the use of transcribing service outside the China , market research company, cloud hosting provider*<br>**If yes, please provide details of the country/countries and the collection/transfer/storage arrangements:** |

扫描全能王
3亿人都在用的扫描App

**8.9 Is it anticipated that there will be any future use of the data?**

**Yes ☐ No ☒**

*Future use of the data here refers to separate projects which may wish to make use of the research data collected for this project. E.g. you may later realise that research data for this project are useful for another project, in which case separate ethical approval will be required. Gaining consent from participants to allow for this future use will strengthen your case for ethical approval to use this data in future projects.*

*Please note, 'future use' is separate to 'dissemination' (below) which refers to the dissemination of research findings from this project.*

**If yes, please provide details (if known at this stage). This should be clear in the Participant Information Leaflet and on the consent form if there is potential for future use of this data:**

## SECTION 9: DISSEMINATION

**Please describe the dissemination arrangements for the study:**

*To include:*

- *What will happen to the results at the end of the study*
- *Will this study have any opportunities for impact or are any impact-related activities planned?*
- *How and where will the results be reported/published?*
- *Are there any plans to notify/debrief the participants of the outcome of the study, either by provision of the publication, or via a specifically designed newsletter, presentation etc.?*
- *If it is possible for the participant to specifically request results from the researcher when would this information be provided e.g. after the Final Study Report had been compiled or after the results had been published?*

**At the conclusion of this study, the results will be systematically compiled and analyzed, with the aim of achieving significant impact. We plan to disseminate the findings widely, both through academic channels and direct engagement with the public and stakeholders. The results will be published in both international and domestic peer-reviewed journals, ensuring global and local academic outreach. Additionally, presentations at international and national conferences will further the reach and impact of our research. Participants in the study will be debriefed on the outcomes via specifically designed newsletters or direct communications providing access to the published findings. Participants desiring more detailed information can request the results after the final study report has been compiled or after publication. This multi-faceted approach ensures that the study's findings are accessible to both the scientific community and the general public, maximizing the potential for real-world impact.**

## SECTION 10: FURTHER INFORMATION (OPTIONAL)

**Please provide any further details/information relevant to this application that may aid the ethical review process.**

*To include:*

- *For complex studies with multiple work packages, collaborators or steering groups, applicants may wish to submit a protocol or supplementary documents in addition to this application form detailing the roles and responsibilities of each party.*
- *Peer review*
- *Patient and public involvement*
- *Flow diagram*
- *Data management plan*

## SECTION 11: SUPPORTING DOCUMENTS



CS 扫描全能王
3亿人都在用的扫描App

BSREC/HSSREC will need to review **all** participant facing documents associated with this application.

There may be more than one type of each document for each study, i.e. multiple participant information leaflets if there are different participant groups, or work packages.

Please specify below, which documents have been submitted with this application (where applicable):

☒ Participant information leaflet(s)
☒ Consent form(s)
☐ Draft recruitment materials
☐ Questionnaire(s)/Survey question(s)
☒ Interview schedule(s)/topic guide(s)
☐ Epigeum Research Integrity Training Certificate
☐ Risk assessment
☐ Local ethics approvals
☐ Other, please specify:

## SECTION 12. SIGNATURES AND DECLARATIONS

扫描全能王
3亿人都在用的扫描App

***The information in this form together with any accompanying information is complete and correct to the best of my knowledge and belief, and I take full responsibility for it.***

***I undertake to abide by the Business School of Central University of Finance and Economics' Research Code of Practice in undertaking this study.***

***I understand that BS-CUFE grants ethical approval for projects, and that the seeking and obtaining of all other necessary approvals and permissions prior to starting the project is my responsibility.***

***I understand that I must not begin research and related projects with human participants, their data, or tissue until I have received full approval from the relevant Research Ethics Committee of the Business School of Central University of Finance and Economics.***

***I understand that any changes that I would like to make to this study after receiving approval from BS-CUFE, require further review. As such, they must be submitted to cufebs_yjs@126.com before such changes are implemented.***

**Signature of Applicant:** Shubo Liu **Date: 2021-08-20**

**Signature of Supervisor (If applicable): N/A** **Date:**

**Signature of Head of Department:** Xiaoyuan Liu **Date: 2021-08-20**

**Signature of Head of Research Governance:** Xiaoyuan Liu **Date: 2021-08-20**

**Official Stamp/Seal:** **Date: 2021-08-20**

**Approval Number:AC-SB-CUFE-2021-0013**

*Please note, student applications do not require a Head of Department signature.*

**Note. Your electronic submission should contain signatures (electronic signatures will be accepted) of all relevant parties. Applications without the necessary signatures will be returned.**



扫描全能王
3亿人都在用的扫描App

中央财经大学 | 商学院
Central University of Finance and Economics | Business School

Dear Dr. Liu,

I write to inform that we have approved in principle for you to conduct the surveys or observation of human behaviour by non-clinical means as stated below at the Central University of Finance and Economics:

Project Title: Institutional Transformations and the Survival Dynamics of Management Scholars in Chinese Business Schools.
Principal investigator: Shubo Liu

**Approval Number:*AC-SB-CUFE-2021-0013***

Thank you for your attention.

Sincerely,

Signature of Head of Research Governance: 刘小元 Xiaoyuan Liu Date: 2021-08-20

Prof. Xiaoyuan Liu
Business School
Central University of Finance and Economics

中央财经大学 商学院 1100000851263
