# Supplementary material for: Professional, scholar, or knowledge worker? Identity construction of Chinese management researchers amid the research–practice gap
Source: PLoS One. 2024 Aug 29;19(8):e0306833. doi: 10.1371/journal.pone.0306833 (PMC11361602; doi:10.1371/journal.pone.0306833)
Supplement: S6 File — (PDF) [file pone.0306833.s006.pdf]

Consolidated criteria for reporting qualitative studies (COREQ): 32-item checklist

Developed from:
Tong A, Sainsbury P, Craig J. Consolidated criteria for reporting qualitative research (COREQ): a 32-item checklist for interviews and focus groups. Int. J. Qual. Health Care 2007;19(6): 349–57. doi: 10.1093/intqhc/mzm042

DOMAIN 1: Research team and reflexivity

**Items**	**Guide question/description**	**Answers**
Personal characteristics		
1.Interviewer/facilitator	Which author/s conducted the interview or focus group?	Shubo Liu, Mengna Lv, and Qiuli Huang<br>Also see Lines 251-253: "The first three authors of this paper, comprising one male associate professor and two female doctoral students, all had experience in interview methods, and conducted the interviews with each participant."
2.Credentials	What were the researcher's credentials? *E.g. PhD, MD*	Shubo Liu: PhD<br>Mengna Lv: PhD candidate<br>Qiuli Huang: PhD candidate
3.Occupation	What was their occupation at the time of the study?	Shubo Liu: Associate Professor of the School of Business at Central University of Finance and Economics<br>Mengna Lv: PhD candidate of the School of Business at Central University of Finance and Economics<br>Qiuli Huang: PhD candidate of the School of Business at Central University of Finance and Economics
4.Gender	Was the researcher male or female?	Shubo Liu: male<br>Mengna Lv: female<br>Qiuli Huang: female
5.Experience and training	What experience or training did the researcher have?	The researchers all had experience in interview methods.
Relationship with participants		

6.Relationship established	Was a relationship established prior to study commencement?	The team members had varying degrees of familiarity with the interviewees, ranging from professional colleagues to new acquaintances recruited via snowball sampling (also see lines 262-264).<br><br>Specifically, the rapport between interviewers and interviewees was diverse, encompassing academic connections such as encounters at conferences, shared institutional affiliations like colleagues, peers, and teacher-student relationships, as well as personal ties such as friendships and shared regional origins.<br><br>Additionally, recruiting some participants through snowball sampling brought in individuals who had no prior relationship with the researchers, thus expanding the network beyond immediate academic and personal circles. This diversity in relationships enriched the study with a multitude of perspectives and insights.
7. Participant knowledge of the interviewer	What did the participants know about the researcher? *e.g. personal goals, reasons for doing the research*	Prior to the commencement of the study, participants were provided with a Participant Information Leaflet (see S2 Appendix). This document served to inform them about the researchers' identities, the nature of the research being conducted, and the objectives of the study. The leaflet ensured that the participants had a clear understanding of the research goals and the reasons behind the study, facilitating informed consent and setting the stage for a transparent research process.
8. Interviewer characteristics	What characteristics were reported about the interviewer/facilitator? *e.g. Bias, assumptions, reasons and interests in the research topic*	Prior to the commencement of the study, participants were provided with a Participant Information Leaflet (see S2 Appendix) which outlined the researchers' qualifications, the reasons for conducting the research, and their interests in the research topic. The leaflet also described the researchers' roles and their commitment to maintaining objectivity throughout the research process. By being open about

		these aspects, we aimed to mitigate any potential impact on data collection and analysis, thereby enhancing the trustworthiness and credibility of the study.

DOMAIN 2: Study design

**Items**	**Guide question/description**	**Answers**
Theoretical framework<br>9.Methodological orientation and Theory	What methodological orientation was stated to underpin the study? *e.g. grounded theory, discourse analysis, ethnography, phenomenology, content analysis*	Grounded theory.<br>Also see lines 315-316: "We conducted an inductive data analysis, employing the grounded theory approach [39]."
Participant selection		
10.Sampling	How were participants selected? *e.g. purposive, convenience, consecutive, snowball*	Purposive and snowball.<br>Also see lines 246-251: "To ensure a representative sample, we purposively selected interviewees at various career stages (i.e., complete, assistant, and associate professors and PhD candidates), who represented a range of management research fields (i.e., strategy, marketing, organizational behavior and human resource management (OBHRM), operations, as well as finance and accounting). We approached the interviewees through established social networks and snowball methods [37]."
11.Method of approach	How were participants approached? *e.g. face-to-face, telephone, mail, email*	Face-to-face and WeChat video<br>Also see lines 264-265: "Over half of the interviews were face-to-face, while the remaining interviews were via WeChat video, a necessary adaptation due to COVID-19 restrictions."
12.Sample size	How many participants were in the study?	34 participants.<br>Also see lines 264-266: "[…] we conducted interviews with 34 Chinese management researchers […]"

13.Non-participation	How many people refused to participate or dropped out? Reasons?	None.
Setting		
14.Setting of data collection	Where was the data collected? *e.g. home, clinic, workplace*	The face-to-face interviews were conducted at various locations based on the participants' preferences, including their offices or homes. The WeChat video interviews were conducted online.<br>Also see lines 266-272: "The face-to-face interviews were conducted at various locations based on the participants' preferences, including their offices or homes, providing a comfortable and convenient environment for open dialogue. The WeChat video interviews were conducted online, allowing for remote participation and accommodating a flexible approach to data collection that included participants who might not be able to attend in-person meetings. This hybrid approach to the setting of data collection ensured accessibility and participant comfort, facilitating a rich and diverse dataset."
15. Presence of non-participants	Was anyone else present besides the participants and researchers?	No.
16.Description of sample	What are the important characteristics of the sample? *e.g. demographic data, date*	Table 1 described the interviewees' profiles (line 290).
Data collection		
17.Interview guide	Were questions, prompts, guides provided by the authors? Was it pilot tested?	See lines 253-258: "The interviewers usually began the interview by asking the respondents to describe their academic background and impressions of management studies before discussing the factors that inspire and guide their research. These questions often led to conversations regarding their experiences in research, including selecting research questions,

		conducting empirical studies, and publishing practices."<br><br>Also see S1 Appendix "Interview Schedules."<br><br>Yes, the study was pilot tested. We conducted six preliminary interviews, which included two professors, two associate professors, one assistant professor, and one doctoral student. These pilot interviews helped us refine the interview guide and ensure the clarity and relevance of the questions, thereby providing a solid foundation for the subsequent formal interviews.
18.Repeat interviews	Were repeat interviews carried out? If yes, how many?	No.
19.Audio/visual recording	Did the research use audio or visual recording to collect the data?	Yes, we used audio recording to collect data. Entire interviews were recorded by using professional recording pen. The recorded interview was automatically transcribed into text. Subsequently, we meticulously reviewed and checked the text, then sent it to interviewees for verification.<br><br>Also see lines 279-285: "Each interview, lasting between 45 to 150 minutes, was audio-recorded with a professional voice recorder capable of automatic verbatim transcription. Since the interviews were conducted in Chinese, the transcripts are also in Chinese characters. Within 72 hours of each interview, the second and third authors independently reviewed and cross-checked the transcriptions to ensure accuracy and maintain the integrity of the original meaning. These transcriptions were then sent back to the interviewees for verification. The process resulted in over 650,000 Chinese characters of transcribed text."
20.Field notes	Were field notes made during and/or after the interview or focus group?	Yes, each interviewer maintained a reflexive journal after every interview. This practice involved continuously questioning and reflecting on their assumptions, emotions, and values to

		minimize the influence of personal biases, preconceptions, and assumptions on the data collection and interpretation.<br><br>Also see lines 377-381.
21.Duration	What was the duration of the inter views or focus group?	See Line 279: "Each interview, lasting between 45 to 150 minutes […]"
22.Data saturation	Was data saturation discussed?	Data collection continued until saturation was reached, meaning no new relevant information was observed in the responses, ensuring comprehensive coverage of the topic.<br><br>Also see lines 258-260 "To adhere to theoretical sampling principles, we terminated the interview when no new concepts, categories, or relationships emerged from further interview analyses, that is, when data reached saturation [38]."
23.Transcripts returned	Were transcripts returned to participants for comment and/or correction?	Yes, see lines 283-284 "These transcriptions were then sent back to the interviewees for verification."

DOMAIN 3: Analysis and findings

**Items**	**Guide question/description**	**Answers**
Data analysis		
24.Number of data coders	How many data coders coded the data?	The first three authors coded the data.<br><br>Also see lines 320-359, 381-385.
25.Description of the coding tree	Did authors provide a description of the coding tree?	Yes, please see figure 1.
26.Derivation of themes	Were themes identified in advance or derived from the data?	Our themes were derived from iterative cycles of data examination and theory development.<br><br>Also see lines 316, 334-335, 344-345.
27.Software	What software, if applicable, was used to manage the data?	We used Nvivo12 software to code data.

		Also see lines 316-319: "[…] facilitated by Nvivo12 software. This software facilitated the creation of a coding tree, allowing us to visualize the hierarchical structure of our coding scheme and track the relationships between different codes."
28.Participant checking	Did participants provide feedback on the findings?	Yes. We randomly selected and invited eight participants to review our findings. These participants generally provided affirmative responses, expressing agreement and validation of the research findings.<br>Also see lines 385-392: "Third, after data analysis, we randomly fed back the analysis results to eight interviewees to solicit their opinions and feedback. The chosen participants generally provided affirmative responses, expressing agreement and validation of the research findings. This step is crucial as it aligns with the qualitative research practice of member checking, ensuring that the research outcomes are both researcher-conceived and participant-endorsed [41]."
Reporting		
29.Quotations presented	Were participant quotations presented to illustrate the themes/findings? Was each quotation identified? *e.g. participant number*	In the section "Findings," we have presented quotations for each theme and identified each quotation with a participant number.
30.Data and findings consistent	Was there consistency between the data presented and the findings?	Yes.
31.Clarity of major themes	Were major themes clearly presented in the findings?	We detailed the specific connotations of three major themes in Table 2 (also see line 371).<br>The section "Findings" illustrates each major theme and sub-theme (minor theme). Furthermore, we described each theme within the text in the Findings in more detail.
32.Clarity of minor themes	Is there a description of diverse cases or discussion of minor themes?	
